# All roads lead to Rome: QTL analysis for vernalization requirement and dissection of allelic variation uncovered unexpected diversity of *FLC* loci in *Camelina sativa*

**DOI:** 10.3389/fpls.2025.1639872

**Published:** 2025-07-25

**Authors:** Vicky Roslinsky, Raju Chaudhary, Annette Zatylny, Isobel A. P. Parkin, Christina Eynck

**Affiliations:** ^1^ Agriculture and Agri-Food Canada – Saskatoon Research and Development Center, Saskatoon, SK, Canada; ^2^ Global Institute for Food Security, Saskatoon, SK, Canada

**Keywords:** *Camelina sativa*, flowering time, vernalization, phenology, Flowering Locus C

## Abstract

Winter camelina (*Camelina sativa*) is a climate-resilient oilseed crop that has received attention as a feedstock crop for advanced, low-carbon-intensity biofuels. Breeding programs working on winter camelina improvement have to contend with heterogeneous germplasm, oftentimes erroneously identified as winter biotypes, and a gene pool that is much smaller than that of spring-type camelina, the latter having motivated crosses between winter and spring biotypes. For the unequivocal differentiation of winter from spring types at an early stage, breeders require a tool to track the vernalization requirement trait in segregating breeding populations as well as in putative winter cultivars, breeding lines, and accessions to be used as parental lines. Linkage mapping in a winter (‘Joelle’) × spring (‘SES0787LS’) *C. sativa* biparental F_2_ population identified two major quantitative trait loci (QTLs) for vernalization requirement on chromosomes 8 and 13. Both regions contained orthologs of *Flowering Locus C* (*FLC*), a gene known to have a significant effect on flowering time and vernalization requirement in plants. Based on the *FLC* gene sequences, allele-specific PCR-based markers were developed, suitable for the routine screening of *C. sativa* germplasm for the presence of the winter and spring alleles of all three *C. sativa FLC* orthologs, including a chromosome 20 locus. The analysis of the winter cultivar ‘Joelle’ and a diverse *C. sativa* germplasm panel uncovered greater than expected variability for the *FLC* alleles, with most lines possessing several different allele combinations and still undergoing genetic segregation. Contrary to previous reports, spring camelina lines can carry the spring and/or winter alleles of *Csa.FLC.C20*, indicating that this gene by itself only plays a subordinate role in the regulation of flowering and vernalization requirement. In winter *C. sativa* germplasm, combinations of *Csa.FLC.C08* winter alleles with the winter alleles of one or both of *Csa.FLC.C13* and *Csa.FLC.C20* result in vernalization requirement, while winter *Csa.FLC.C08* by itself leads to a semi-winter type. The results of this study and the tools developed herein are a first step to orchestrating the genes underlying vernalization requirement in *C. sativa* and developing winter camelina cultivars optimized for different winter environments.

## 1 Introduction

Camelina (*Camelina sativa* [L.] Crantz) is a short-season crucifer oilseed adapted to the temperate and continental climates of the mid-latitudes (Weiss et al., 2024). It has been shown to have potential as a low maintenance crop in North America (Blackshaw et al., 2011; Enjalbert et al., 2013; Gugel and Falk, 2006; Plessers et al., 1962; Robinson, 1987), Europe (Vollmann et al., 2007; Zanetti et al., 2017, 2021, 2024), South America (Berti et al., 2011; Solis et al., 2013), China (Gao et al., 2022; Zhang et al., 2021), and Russia (Kon’kova et al., 2021). Camelina has a number of favorable agronomic characteristics, including frost and drought tolerance (Angelini et al., 1997; Bonjean and Goffic, 1999; French et al., 2009; Hunsaker et al., 2011; Putnam et al., 1993), resistance to insect pests (Soroka et al., 2015) and diseases (reviewed in Séguin-Swartz et al., 2009), and good performance on economically marginal lands (Johnson et al., 2019; Putnam et al., 1993; Robinson, 1987). It also possesses a seed oil that has the unusual property of being both rich in unsaturated fatty acids—primarily linolenic acid (C18:3) (Budin et al., 1995; Vollmann et al., 2007; Zubr and Matthäus, 2002)—and high in antioxidants (Abramovič and Abram, 2005). Because of these characteristics, camelina has received attention for diametrically opposed applications: as a feedstock crop for advanced biofuels (Fröhlich and Rice, 2005; Shonnard et al., 2010; Wu and Leung, 2011) and as a source of healthy oil for food, feed, and nutraceutical applications (Hixson et al., 2014; Kirkhus et al., 2013; Ngo et al., 2023; Ratusz et al., 2018).

There are both spring and winter annual biotypes (Mirek, 1980; Plessers et al., 1962; Putnam et al., 1993; Zubr, 1997). Winter cultivars, which are usually seeded in the fall, possess exceptional cold hardiness and typically survive the winters in the northern USA and Canada (Gesch and Cermak, 2011; Gesch et al., 2018; Horvath et al., 2019). In the USA, winter camelina has been extensively studied for use in double- and relay-cropping systems in combination with short-season summer crops as a means to produce a biofuel feedstock crop without devoting land traditionally used for food production (Berti et al., 2015, 2017; Gesch et al., 2014; Gesch and Archer, 2013; Gesch and Johnson, 2015; Johnson et al., 2017; Sindelar et al., 2017). Winter camelina is also the only winter oilseed that can be grown on the Canadian Prairies and, together with winter cereals, allows for the establishment of true winter crop rotations. Growing winter camelina, like other crops that cover the soil over the winter months, provides environmental benefits, including the prevention of soil erosion (Lal et al., 1991) and the uptake of excess nitrogen (Staver and Brinsfield, 1998). As one of the first plants to flower in the spring, winter camelina also offers early-season feed to pollinators (Eberle et al., 2015).

To avoid flowering before the onset of winter, winter annuals have evolved to require exposure to low temperatures for several weeks, known as vernalization, to transition from vegetative to reproductive growth (Sheldon et al., 2000; Kiefer et al., 2017). In Brassicaceae, a major regulatory gene in the vernalization pathway is *FLOWERING LOCUS C* (*FLC*) (Michaels and Amasino, 1999; Swiezewski et al., 2009), a MADS-box transcription factor that under ambient temperatures suppresses the expression of the floral integrators *FLOWERING LOCUS T* (*FT*) and *SUPPRESSOR OF OVEREXPRESSION OF CONSTANS 1* (*SOC1*) (Deng et al., 2011; Helliwell et al., 2006; Michaels and Amasino, 1999; Samach et al., 2000; Searle et al., 2006), which prevents the progression of the apical meristem from vegetative to floral development (Henderson and Dean, 2004). Vernalization brings about epigenetic silencing of *FLC*; in consequence, repression of *FT* and *SOC1* is reduced, and the apical meristem transitions to produce reproductive structures (Anderson et al., 2018; Bastow et al., 2004; Sheldon et al., 2000; Schiessl et al., 2019; Takada et al., 2019). As the role of functional *FLC* alleles is to prevent flowering, non-functional alleles, or *flc* null mutants, are unresponsive to cold and result in early and vernalization-independent flowering (Michaels and Amasino, 1999). In the hexaploid genome of *C. sativa*, there are three orthologous copies of the *FLC* gene on chromosomes 8, 13, and 20 (Kagale et al., 2014). The importance of *FLC* for flowering time in general and vernalization requirement in camelina species has been demonstrated by quantitative trait locus (QTL) analyses, which showed that QTLs co-localized with *FLC* in both spring (Li et al., 2021; Lily et al., 2021) and spring × winter inter- and intra-specific mapping populations (Chaudhary et al., 2023; Kandel et al., 2024).

With a winter camelina germplasm pool that is much smaller than that of spring camelina, some breeders are resorting to winter × spring crosses to increase the genetic diversity of the winter material and to transfer traits from spring cultivars to winter breeding lines. Others have proposed winter × spring crosses to develop winter cultivars that mature earlier than current commercial varieties, which is desirable for double and relay cropping (Kandel et al., 2024). However, phenotypic differentiation between winter- and spring-type progeny is difficult, particularly when populations are grown with a vernalization period. Thus, time to flowering after vernalization is not a reliable indicator for whether a plant should be categorized as a spring, semi-winter, or winter type. Additionally, winter-type accessions deposited at gene banks are often highly variable in their expression of the winter phenotype, with many being admixtures of winter-, semi-winter-, and even spring-type plants (Chao et al., 2019). For the unequivocal differentiation of winter from spring types, breeders require a tool that affords the ability to track the vernalization requirement trait in segregating breeding populations as well as in putative winter cultivars, breeding lines, and accessions to be used as parental lines.

In this study, we report on the identification of QTLs and the development of widely applicable PCR-based markers linked to the candidate genes controlling flowering time and vernalization requirement in a winter × spring biparental population. Furthermore, we uncover an unexpected degree of genetic variation at the *FLC* loci in both spring- and winter-type *C. sativa* germplasm and propose that segregation still exists within many of the publicly available winter camelina accessions and cultivars.

## 2 Materials and methods

### 2.1 Plant materials and phenotyping for flowering behavior

#### 2.1.1 F_2_ population development

Two F_2_ populations were developed by manual reciprocal crossing of the winter camelina cultivar ‘Joelle’ with the spring camelina cultivar ‘SES0787LS’ in 2018 and subsequent selfing of F_1_ plants. Seed of ‘Joelle’, a publicly available cultivar developed at Limagrain (Saint-Beauzire, France), was obtained from Dr. Russ Gesch, USDA-ARS (Morris, MN, USA). ‘SES0787LS’ was provided by Smart Earth Camelina Corporation (Saskatoon, Canada). The cross where ‘Joelle’ was used as the female and ‘SES0787LS’ was the pollen donor gave rise to F_2_ population 19CS1178-F2, while the F_2_ population 19CS1179-F2 was derived from the reciprocal cross. For crossing, closed mature buds of the female parent were manually opened, the anthers removed, and the stigma pollinated using pollen from the male parent. ‘Joelle’ plants and semi-winter-type F_1_ plants were placed in vernalization at 5°C for 35 days after 2 weeks in the greenhouse before being moved back to the greenhouse to induce flowering. The F_2_ populations were originally developed as breeding populations and are therefore composed of bulked seed derived from several F_1_ plants that were generated by crossing several plants of the parental genotypes.

For phenotyping, in the winter of 2020/2021, a total of 427 F_2_ plants (216 from 19C1178-F2 and 211 from 19CS1179-F2) plus 10 plants each of the parent lines ‘Joelle’ and ‘SES0787LS’ as well as 10 plants each of the reciprocal F_1_ hybrids (19CS1178 and 19CS1179) were grown in the greenhouse at 20°C/17°C day/night with a light/dark cycle of 16/8 h. Three seeds were sown in individual pots containing soilless potting mix (Stringam, 1971) amended with the controlled release fertilizer 15-9–12 Osmocote PLUS (Scotts Miracle-Gro Company, Marysville, Ohio). One week after germination, seedlings were thinned to one per pot. Plants remained in the greenhouse until the opening of the first flower, at which point days to flowering (DTF) was recorded. After 170 days, plants that had not flowered yet were assigned a DTF value of 170.

#### 2.1.2 Diverse winter- and spring-type germplasm

The diverse camelina germplasm panel consisted of 13 winter *C. sativa* cultivars and accessions; 53 spring *C. sativa* cultivars, breeding lines, and accessions; and three *Camelina microcarpa* genotypes (**Supplementary Table 1**). The latter included one tetraploid *C. microcarpa* (syn. *Camelina intermedia*) line and one each of Type 1 and Type 2 hexaploid *C. microcarpa* genotypes. All lines were grown in the greenhouse without vernalization, and flowering behavior was observed. For marker analysis, DNA of leaf tissue from four plants was pooled at the six-to-eight– leaf stage.

### 2.2 Quantitative trait locus mapping

#### 2.2.1 Genotyping by sequencing

Genomic DNA of F_2_ plants was extracted from young leaf tissue utilizing a modified sodium dodecyl sulfate method (Somers et al., 1998). A genotyping-by-sequencing (GBS) library was constructed for both the F_2_ mapping populations and the diverse germplasm panel following the protocol described by Fu et al. (2021), with modifications. In brief, 200 ng of DNA from each line was digested with the restriction enzymes *Pst*I and *Msp*I. Samples were then ligated to common adapters and size-selected via an AMPure XP (Beckman Coulter, Indianapolis, Indiana) bead cleanup. Subsequently, for each sample, 20 μL of the resulting ligation mix was added to 1× KAPA Fidelity buffer (Roche, Indianapolis, Indiana), 250 μM of KAPA dNTPs, 0.5 U KAPA HiFi HotStart DNA polymerase, and 0.5 μM of a unique NEBNext UDI (Unique Dual Index) Primer (NEB, Ipswich, Massachusetts) in a total reaction volume of 50 μL. The ensuing PCR program consisted of an initial denaturation at 98°C for 30 s, 14 cycles of denaturation at 98°C for 10 s and annealing/extension at 65°C for 75 s, and a final extension at 65°C for 5 min. All PCR samples were quantified using a Quant-iT assay (Thermo Fisher Scientific, Waltham, Massachusetts), and four samples of equal quantity were combined. The combined samples were concentrated using a Zymo DNA Clean and Concentrator-5 kit (CedarLane Labs, Burlington, Ontario) following the provided protocol. Samples were again combined, and a second AMPure XP size selection bead cleanup was completed. The final size-selected samples were again quantified with a Quant-iT PicoGreen assay, and equal amounts of each were used to create one library pool. The library was sequenced using one lane of the Illumina NovaSeq 6000 with the SE-150 sequencing protocol at the Genome Quebec Innovation Centre, Montreal, Quebec.

#### 2.2.2 Genetic analysis of segregating populations

Sequences were de-multiplexed and trimmed of low-quality bases and adapters using Trimmomatic version 0.32 (Bolger et al., 2014) with a minimum read length of 75 bp for retention. All high-quality reads were mapped to the *C. sativa* reference genome (Kagale et al., 2014) using bowtie2 version 2.4.1 (Langmead and Salzberg, 2012) and SAMtools version 1.15.1 (Danecek et al., 2021), with default parameters. Subsequently, the aligned binary alignment map files were used to call single-nucleotide polymorphisms (SNPs) using the BCFtools mpileup tool (Danecek et al., 2021). The same process was followed to analyze individual ‘Joelle’ lines utilizing three separate ‘Joelle’ reference genomes [NCBI: GCA_036769185.1; DOE-JGI Phytozome Joelle v1.1 (https://phytozome.jgi.doe.gov/info/CsativaJoelle_v1_1) and the AAFC Joelle reference (unpublished)].

The genetic maps for the F_2_ populations were constructed using JoinMap 4.0 (Van Ooijen, 2006). Linkage groups were generated with a minimum logarithm of the odds (LOD) threshold of 5.0. The regression mapping algorithm and Kosambi mapping function were utilized to develop mapped linkage groups. QTL analysis for DTF was performed using the MQM mapping method of MapQTL 6.0 (Van Ooijen, 2009). A permutation test (10,000 permutations, 95% confidence level, mapping step size 1.0) was performed to determine the significant LOD threshold for DTF.

### 2.3 Marker development

Utilizing whole-genome sequences of two hexaploid and one tetraploid *C. microcarpa* lines and 15 spring *C. sativa* lines (Parkin et al., unpublished) (**Supplementary Table 1**), the coding region for all three *FLC* genes and surrounding up- and downstream sequences were isolated (**Supplementary Tables 4**-**6**). Public reference genome sequences from spring-type *C. sativa* lines DH55 (Kagale et al., 2014) and ‘CO46’ (GCA_036971115.1), as well as from the winter-type *C. sativa* cultivar ‘Joelle’ [GCA_036769185.1; DOE-JGI Phytozome Joelle v1.1 (https://phytozome.jgi.doe.gov/info/CsativaJoelle_v1_1) and AAFC Joelle reference (unpublished)], were also included in the analysis. Sequences were aligned using the EMBL-EBI online tool MUSCLE (Madeira et al., 2022). A selective Kompetitive Allele-Specific PCR (KASP) primer for the *Csa.FLC.C08* allele was developed based on a SNP located 700 bp upstream of the start codon. The KASP primer that was first developed for *Csa.FLC.C13* was found to be segregating in individual ‘Joelle’ plants. Because of this, several *Csa.FLC.C13*-specific primers were developed, and Sanger sequencing was carried out to determine the ‘Joelle’ *Csa.FLC.C13* allele sequence. Subsequently, a selective KASP primer for *Csa.FLC.C13* was developed based on a SNP located in the intron 637 bp before the start of exon 2. In addition, utilizing the above-mentioned whole-genome sequences and the INDEL present in exon 5 identified by Anderson et al. (2018), KASP primers were developed for *Csa.FLC.C20*. All primers are listed in **Supplementary Table 2**. Each KASP reaction contained 50 ng of genomic DNA, 4.0 μL of KASP 2× Master Mix (LGC Genomics, St. Alexandria, Minnesota), and 0.11 μL of primer assay mix in a total volume of 8.0 μL. All amplifications were performed in a CFX96 Real-Time Thermal Cycler (Bio-Rad Laboratories, Hercules, California) using the touchdown PCR protocol recommended by the manufacturer. To analyze potential heterogeneity within ‘Joelle’, 310 individual plants were tested using the *FLC* markers described above. Only ‘Joelle’ plants that were homozygous at all loci for the respective spring or winter alleles were kept, and self-pollination was performed using selfing bags. All diverse lines listed in **Supplementary Table 1** were also examined using the *FLC* KASP markers.

### 2.4 Field experiments

In the spring of 2021, seeds of 19CS1178-F2 and 19CS1179-F2 were planted in a field trial at the AAFC Saskatoon Research Farm. Each reciprocal population was seeded in two replicates of four 20-ft. rows at a low seeding rate. Additionally, one 20-ft. row each of parent lines ‘SES0787LS’ and ‘Joelle’ was seeded in each replicate. At the rosette stage, plants of each F_2_ population were thinned to 125 lines per replicate. Both ‘SES0787LS’ and ‘Joelle’ were thinned to 10 plants per row. Numbered marker flags were placed next to every fifth plant to keep track of plant numbers. Leaf tissue samples were taken at the rosette stage. For each plant, the date when the first flower opened was recorded, and days to flowering calculated. After flowering, plants were cut at ground level.

## 3 Results

### 3.1 Population development and phenotyping for flowering behavior

All 427 individual F_2_ plants from the ‘Joelle’ × ‘SES0787LS’ (and reciprocal) cross, as well as parental lines and F_1_ hybrids, were evaluated for DTF under greenhouse conditions without vernalization. Plants were evaluated daily and marked as having flowered when the first open flower was detected. Plants that had not flowered after 170 days were classified as true winter types. The reciprocal crosses produced semi-winter F_1_ hybrids with average DTF of 59 (19CS1178) and 55 (19CS1179) days, compared to an average of 35 days for the spring-type parent, ‘SES0787LS’. There was no statistically significant difference between the DTF for the reciprocal F_1_ hybrids, indicating that flowering time and vernalization requirement were not influenced by maternal genetic effects.

**Figure 1:**
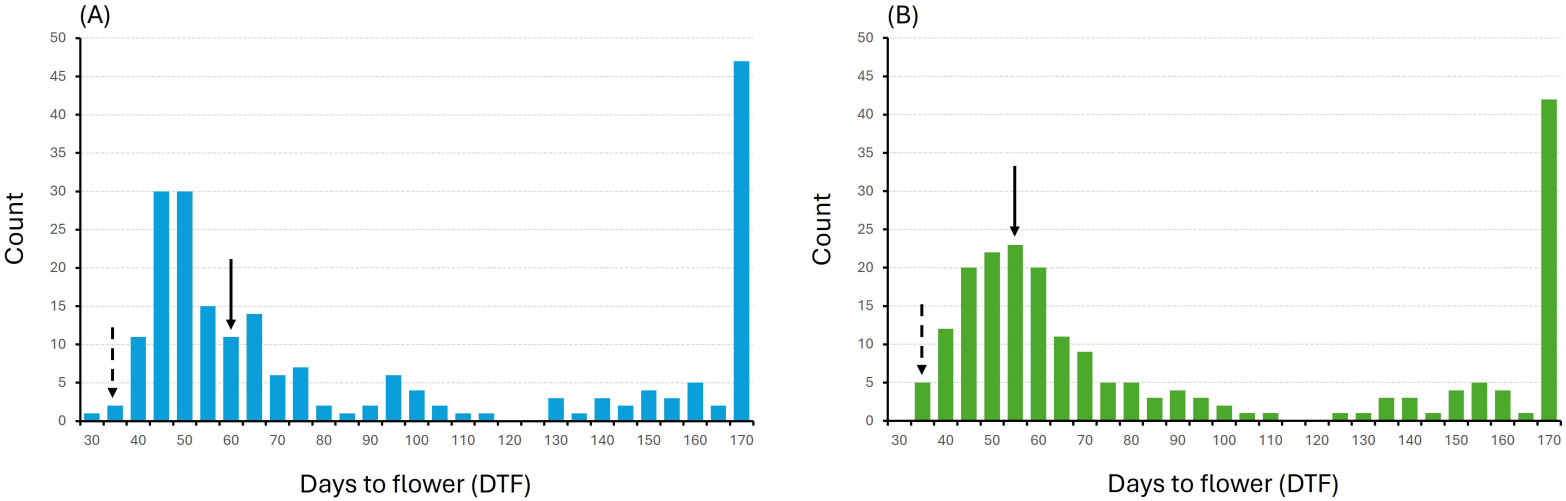
Frequency distribution of days to flowering (DTF) for F_2_ populations **(A)** 19CS1178-F2 (derived from ‘Joelle’ × ‘SES0787LS’) and **(B)** 19CS1179-F2 (derived from ‘SES0787LS’ × ‘Joelle’). Lines that did not flower after 170 days were assigned a DTF value of 170. Solid arrows point to the average DTF of F_1_ hybrids; dotted arrows point to average DTF of spring type parent, ‘SES0787LS’.

The reciprocal populations showed a similar frequency distribution for DTF, with most of the plants exhibiting delayed flowering compared to the spring-type parent, ranging from 40 to 80 days after seeding. Both F_2_ populations had a mean DTF of 71 days (**Figures 1A, B**). Furthermore, 22% and 20% of the plants of 19CS1178-F2 and 19CS1179-F2, respectively, did not flower after 170 days and were classified as true winter types. Only two plants flowered slightly earlier than 35 days after seeding, the average DTF for the spring-type parent.

**Figure 2:**
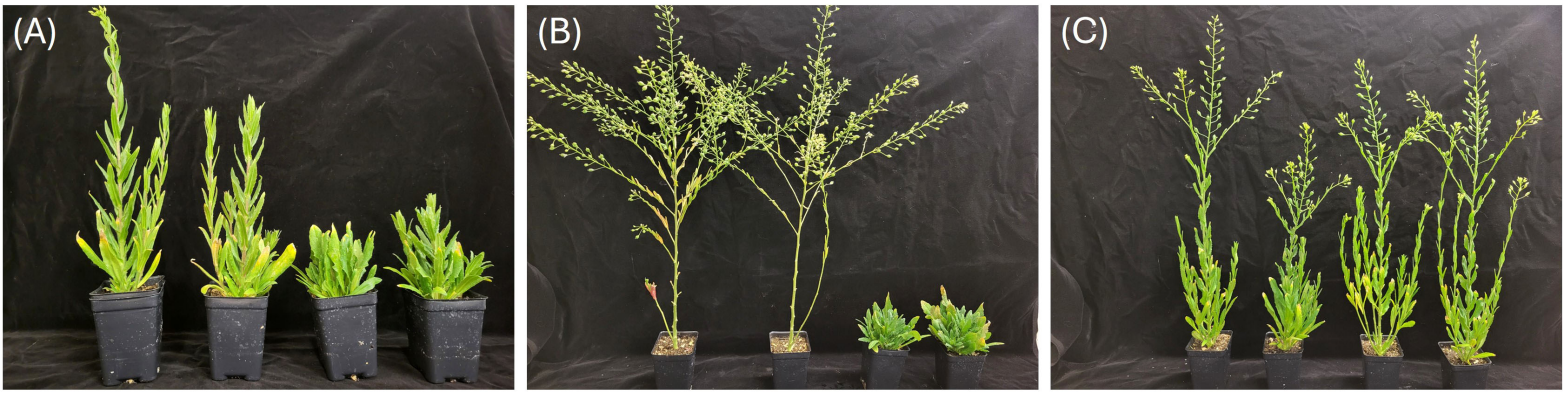
*Camelina sativa* accessions, 44 days after seeding. **(A)** CN113691, a mix of true winter types and plants with delayed flowering without vernalization. **(B)** CN113660, a mix of true winter types and spring types. **(C)** CN113668, a semi-winter type, with delayed flowering compared to typical spring-type plants, as shown on the left of **(B)**.

Segregation was detected within many of the winter lines (**Figure 2**) of the diverse germplasm collection; plants ranged from true-breeding winter types, where none of the plants flowered without vernalization, to spring types and various stages in between, with plants displaying different degrees of delayed flowering.

### 3.2 QTL mapping

**Figure 3:**
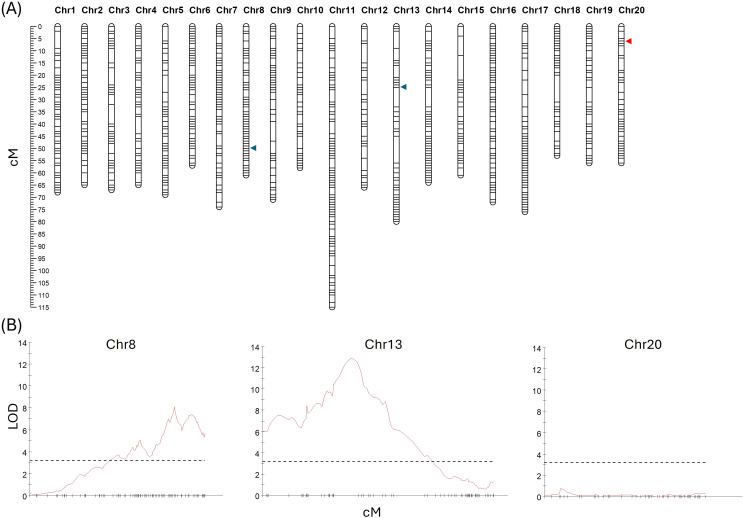
**(A)** Genetic linkage map derived from populations 19CS1178-F2 and 19CS1179-F2, with the position of quantitative trait loci (QTLs) associated with vernalization requirement (blue arrowheads) on chromosomes 8 and 13. The position of Csa.FLC.20 is marked with a red arrowhead. The distance in cM is shown on the left side. **(B)** QTL traces for chromosomes 8, 13, and 20. The dashed line is at 3.2 and represents the significant LOD threshold.

A genetic map was developed utilizing GBS data from a total of 240 randomly selected F_2_ plants (118 from 19CS1178-F2 and 122 from 19CS1179-F2). A total of 54,788 SNPs were detected. To ensure the accurate calling of heterozygous SNPs, extra caution was taken during SNP selection; those with more than 5% missing data or distorted segregation were removed. After filtering, a total of 1,252 SNPs were used to develop a linkage map with a total length of 1,354.4 cM (**Figure 3**). The average mapping interval was 1.10 cM, and the number of markers per linkage group ranged from 40 on chromosome 12 to 107 on chromosome 11 (**Figure 3**, **Supplementary Table 3**). A QTL analysis of DTF in the absence of vernalization identified two significant QTLs: the first, located on Chr13 (LOD = 12.57), explained 21.4% of the variation and peaked between the Chr13–1720684 and Chr13–4293244 SNP markers. The second QTL was located on Chr8 (LOD = 7.99), explained 14.2% of the phenotypic variance, and peaked between the Chr8–23073155 and Chr8–23387904 SNP markers. *FLC* gene-specific markers (described in detail below) were also mapped and used to further hone the QTL analyses. An apparent double recombination event that was ~2.0 Mb in size and contained *Csa.FLC.C13* was detected. The ‘Joelle’ parental line was determined to be heterozygous in this region, and SNPs with distorted segregation were added back to the map to allow for the complete coverage of the region. The spring parental line ‘SES0787LS’ contained the winter *Csa.FLC.C20* allele, and the ‘Joelle’ parental line used in the development of the F_2_ population was also identified to be heterozygous for the *Csa.FLC.C20* marker; thus, markers in this region were treated as dominant, and those with distorted segregation were added back to the map to allow for the complete coverage of this region. A second QTL analysis with the updated map identified the same two significant QTLs, and each was associated with one of the *FLC* gene-specific markers. The first, located on Chr13 (LOD = 12.87), explained 21.9% of the variation and peaked between the *FLC-13*_KASP and Chr13–4293244 SNP markers. The second QTL was located on Chr8 (LOD = 8.07), explained 14.3% of the phenotypic variance, and peaked directly with the *FLC-8*_KASP marker. Two minor QTLs were also detected in both analyses: on Chr7 (LOD = 4.29), which explained 7.9% of the variation and peaked between the Chr7–20823349 and Chr7–20905187 markers, and on Chr16 (LOD = 3.75), which explained 6.9% and with the maximum peak between the Chr16–21827277 and Chr16–21959032 markers.

### 3.3 Marker development

**Figure 4:**
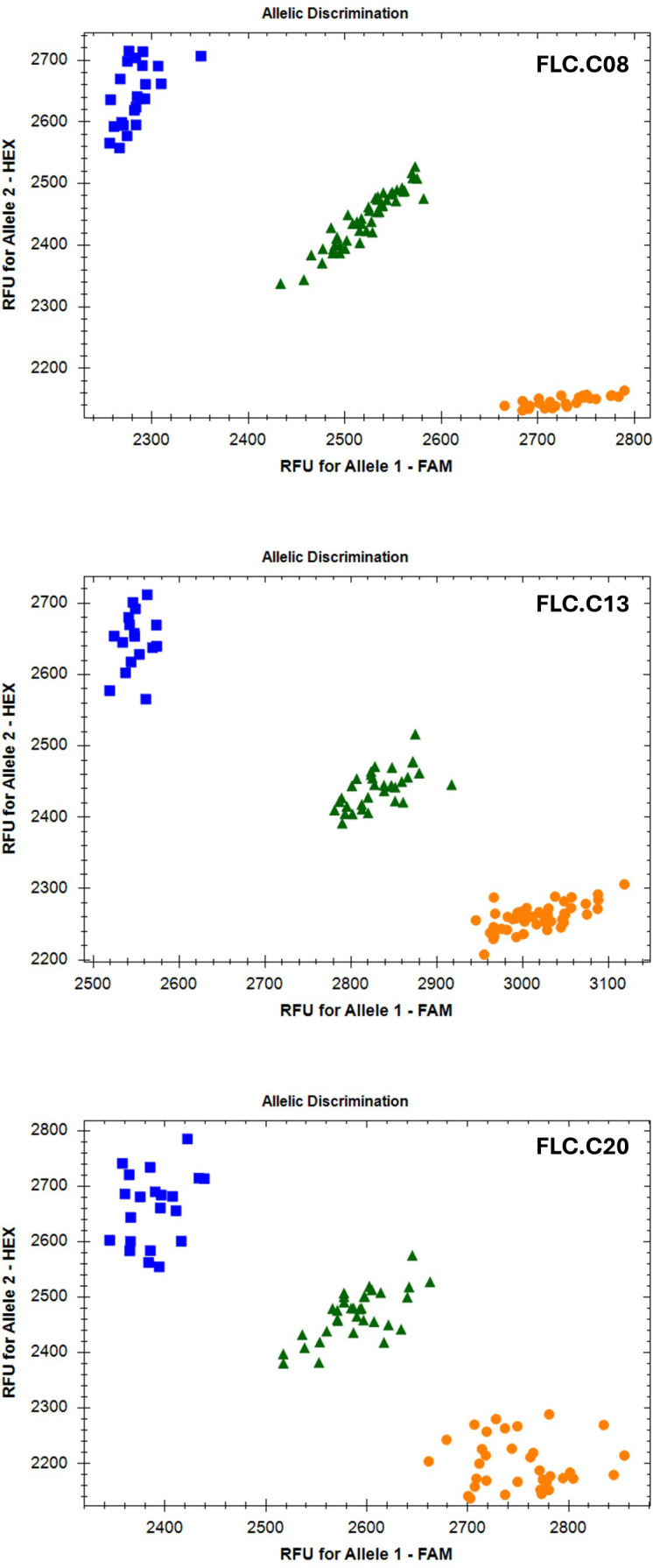
Bio-Rad CFX Maestro images of the Kompetitive Allele-Specific PCR (KASP) markers for the three *FLC* orthologs. Orange circles represent samples homozygous for the A1 allele, blue squares represent samples homozygous for the A2 allele, and green triangles represent heterozygous samples.

**Figure 5:**
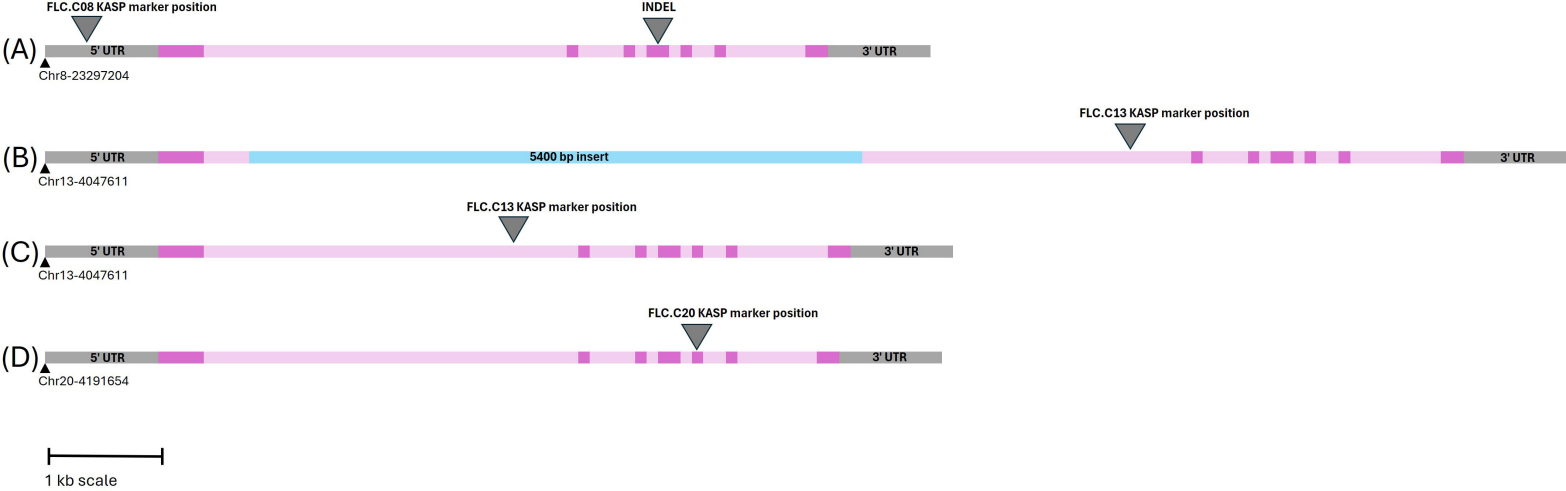
Schematic representation of the three *Csa.FLC* orthologs. Exons and introns are shown in dark and light pink, respectively. Base pair positions are based on the DH55 reference genome. **(A)** *Csa.FLC.C08*. The arrowhead on the left side points to the Kompetitive Allele-Specific PCR (KASP) marker position; the arrowhead on the right side points to the 3-bp INDEL that distinguishes the spring allele from the winter allele. **(B)** Spring allele of *Csa.FLC.C13*. The insert in intron 1 is shown in blue. **(C)** Winter allele of *Csa.FLC.C13*. The arrowhead in both panels B and C points to the KASP marker position. **(D)** *Csa.FLC.C20*. The arrowhead points to 1-bp INDEL that distinguishes the spring allele from the winter allele and serves as the KASP marker position.

*FLC* gene sequences were isolated from the whole-genome sequence of three *C. microcarpa* (winter type) and 15 spring *C. sativa* lines (Parkin, unpublished), as well as the DH55 reference genome (Kagale et al., 2014) and three winter-type cultivar ‘Joelle’ reference genomes [NCBI: GCA_036769185.1; DOE-JGI Phytozome Joelle v1.1; (https://phytozome.jgi.doe.gov/info/CsativaJoelle_v1_1) and AAFC ‘Joelle’ reference (unpublished)], to develop markers suitable for routine screening. The *FLC.C08* sequence alignment (**Supplementary Table 4**) revealed an A/G SNP 700 bp upstream of the start codon. This SNP was converted to a KASP marker (**Figure 4**; **Supplementary Table 4**). The alignment of the *FLC.C13* genes identified a large insert in intron 1 in all spring-type alleles (**Figure 5**; **Supplementary Table 5**). The initial KASP primer pair differentiated spring and winter genotypes, but in a sample of 10 individual ‘Joelle’ plants, segregation for winter and spring *Csa.FLC.C13* alleles was detected. Two representative ‘Joelle’ plants, one with the winter and one with the spring *Csa.FLC.C13* allele, were Sanger sequenced with several pairs of *FLC.C13*-specific primers to analyze the region from 600 bp upstream of the start codon to the end of exon 6. The results confirmed that both the spring and winter *Csa.FLC.C13* alleles were present in ‘Joelle’. Aligning these sequences with those of the *C. microcarpa* lines resulted in the identification of 26 SNPs and 13 INDELs between the spring and winter alleles of *FLC.C13*. Interestingly, the hexaploid *C. microcarpa* line CN119205, which is a winter type, had all the SNPs and INDELs common to the spring-type lines but did not have the above-mentioned large insert in intron 1. A G/T SNP was identified 637 bp before the start of exon 2 in *FLC.C13*, and KASP primers were developed (**Figures 4**, **5**; **Supplementary Table 5**). For *FLC.C20*, KASP primers were developed based on the previously identified single-base-pair INDEL in exon 5 (Anderson et al., 2018), which was confirmed with the extended sequence alignment (**Figures 4**, **5**; **Supplementary Table 6**).

Because of the noted variation at the *Csa.FLC.C13* locus, the variation of each of the *FLC* alleles present in ‘Joelle’ was examined by genotyping 310 individual plants (**Table 1**). Ten different genotypes were isolated; 130 plants had winter *Csa.FLC.C08* and *Csa.FLC.C20* alleles and a spring *Csa.FLC.C13* allele, while 101 plants had winter *Csa.FLC.C08* and *Csa.FLC.C13* alleles and a spring *Csa.FLC.C20* allele. Interestingly, only 30 plants, or just under 10%, were homozygous for all three winter *Csa.FLC* alleles. Plants that were homozygous for the *Csa.FLC.C08* winter allele and the spring *Csa.FLC.C13* and *Csa.FLC.C20* alleles (29) were kept in the greenhouse without vernalization, and while all flowered, they were significantly delayed (>60 DTF). At least one heterozygous allele was identified in 5.8% of the samples, suggesting that some level of segregation is still occurring within this seed source.

**Table 1:** Summary of KASP marker results for Flowering Locus C genes *Csa.FLC.C08*, *Csa.FLC.C13*, and *Csa.FLC.C20* for 310 individual plants of winter camelina variety ‘Joelle’.

FLC.C08	FLC.C13	FLC.C20	Count	%
W	S	W	130	41.9
W	W	S	101	32.6
W	W	W	30	9.7
W	S	S	29	9.4
S	S	W	2	0.65
W	H	H	9	2.9
W	H	S	5	1.6
W	S	H	2	0.65
S	S	H	1	0.3
H	S	W	1	0.3
			310	100

W, winter allele; S, spring allele; H, heterozygous; KASP, Kompetitive Allele-Specific PCR.

A GBS analysis was carried out on the 10 ‘Joelle’ lines, which were used for KASP marker optimization to determine the overall extent of variability within ‘Joelle’. All sequences were aligned to the DH55 reference genome. All SNPs with a quality score below 30 and more than 10% missing data were removed, as well as SNPs that were polymorphic only between DH55 and ‘Joelle’. After filtering, 79,740 SNPs, including 3,265 INDELs, were detected solely among the ‘Joelle’ lines (**Supplementary Table 7**). Two of these lines (Joelle.1 and Joelle.7) were heterozygous for both the *Csa.FLC.C13* and *Csa.FLC.C20* alleles. The overall percentage of heterozygous SNPs was also higher in these two lines compared to the remaining ‘Joelle’ plants, suggesting that segregation still exists within the ‘Joelle’ genome. The GBS data were also aligned to the three ‘Joelle’ reference genomes (NCBI, DOE-JGI, and AAFC). Special interest was paid to the regions of Chr13 and Chr20, which contained *FLC* alleles. The Chr13 double recombination event and the associated distorted segregation present in the genetic map were confirmed through the identification of a region of ~750,000 bp that was found to be segregating between individual ‘Joelle’ lines (**Supplementary Table 8**). The ‘Joelle’ individuals with the spring *Csa.FLC.C13* allele matched the NCBI and DOE-JGI reference genomes across the entire region, while the ‘Joelle’ lines with the winter *Csa.FLC.C13* allele matched the AAFC reference genome. The two ‘Joelle’ lines, which had both *Csa.FLC.C13* alleles, were heterozygous for this entire region when compared to all three reference genomes (**Supplementary Table 8**). A similar pattern was detected in the *Csa.FLC.C20* region; a region of ~1.1 Mb was identified on Chr20, which corresponds to a similar double recombination event in the genetic map (**Supplementary Table 9**). For *Csa.FLC.C20*, both the NCBI and DOE-JGI reference genomes had the winter allele, while the AAFC reference genome had the spring allele. These results agree with the *Csa.FLC.C20* KASP scores, indicating that three of the ‘Joelle’ lines were heterozygous for this allele.

For markers to be useful in a breeding program, they must be polymorphic in a diverse range of breeding lines and gene bank accessions. To this end, 13 winter *C. sativa*, three winter-type *C. microcarpa*, and 53 spring *C. sativa* lines were assessed using the *FLC*-specific markers (**Table 2**; **Supplementary Table 10**). For four of the winter *C. sativa* accessions, two different seed batches were investigated. To ensure a representative sample, four individual plants were bulked for this analysis, except for the parental lines of the population, ‘Joelle’ and ‘SES0787LS’, for which 10 individual plants were tested. The reference genome marker scores were determined based on the SNPs in the actual sequence. All spring lines contained the spring alleles for *Csa.FLC.C08* and *Csa.FLC.C13*, but variation was detected for the *Csa.FLC.C20* INDEL, first identified by Anderson et al. (2018). Thus, 13 lines carried the winter allele, and eight were heterozygous at this locus. For the winter types, segregation was detected in many lines; only ‘Bison’, BSX, BSX-WG1, and the Type 1 hexaploid *C. microcarpa* accession were homozygous for either spring or winter alleles at all three *FLC* loci. The remainder of the lines was segregated for at least one *FLC* ortholog. All winter lines carried the *FLC.C08* winter allele, and the *FLC.C20* winter allele was present in most, except in CN113692 and CN113668. CN113692 had the *FLC.C13* winter allele, as did the tetraploid and Type 1 hexaploid *C. microcarpa* lines, as was documented above for some of the ‘Joelle’ plants. CN113668 only carried the winter *Csa.FLC.C08* gene and showed delayed flowering without vernalization.

**Table 2:** *FLC* gene combinations in spring and winter *Camelina sativa* and *Camelina microcarpa* lines.

Species and biotype	*FLC.C08*	*FLC.C13*	*FLC.C20*
*C. sativa*, winter	W	S	W
	W	S	H
	H	S	W
	H	S	H
	W	S	S
	H	W	S
	**W**	**W**	**S**
	**W**	**W**	**H**
	**W**	**H**	**H**
	**W**	**W**	**W**
	**S**	**S**	**W**
	**W**	**H**	**S**
*C. microcarpa* (4x), winter	W	W	/
*C. microcarpa* (6x), Type 1, winter	W	W	W
*C. microcarpa* (6x), Type 2, winter	W	S	W
*C. sativa*, spring	S	S	S
	S	S	H
	S	S	W

Winter genotypes in bold were detected in ‘Joelle’. A detailed overview of genotypes for all diverse lines tested in this study can be found in **Supplementary Table 10**.

### 3.4 Marker validation under field conditions

To assess the utility of the *FLC* markers under field conditions, 233 F_2_ individuals from the ‘Joelle’ × ‘SES0787LS’ cross were planted in Saskatoon, Canada, in the spring. Tissue was collected, and the lines were tested with the *FLC* allele-specific markers (**Table 3**). Days to flowering was recorded until Sept. 1; plants that had not flowered by this date were labelled as true winter types. From the population, 27 plants were classified as true winter types, and 15 lines died for unknown reasons before the flowering date could be assessed. The average DTF for the population was 51 days. Based on segregation at the three *FLC* loci, all 27 genotypes previously observed in the greenhouse experiment were detected, and as expected, the average DTF increased in lines with a greater number of winter *FLC* alleles. Plants with all three winter *FLC* alleles took twice as long to flower on average (mean DTF = 72) than plants with three spring *FLC* alleles (mean DTF = 36). Half of the plants with all three winter alleles did not flower by the end of the season and were considered true winter types.

**Table 3:** Summary of KASP marker results for the Flowering Locus C genes *Csa.FLC.C08*, *Csa.FLC.C13*, and *Csa.FLC.C20* for 218 individual F_2_ plants from a cross between winter camelina cultivar ‘Joelle’ with spring camelina cultivar ‘SES0787LS’.

*FLC.C08*	*FLC.C13*	*FLC.C20*	Total	DTF	% winter	Gene combinations	Avg. DTF
S	S	S	4	36	0	3 S	36
S	S	H	9	34	0	2 S, 1 H	38
H	S	S	5	38	0		
S	H	S	7	43	0		
H	S	H	14	42	0	1 S, 2 H	46
S	H	H	13	43	0		
H	H	S	13	53	0		
S	S	W	11	44	0	2 S, 1 W	46
W	S	S	4	49	0		
S	W	S	1	57	0		
S	H	W	1	42	0	1 S, 1 W, 1 H	52
W	S	H	9	45	11.1		
H	W	S	5	51	20		
H	S	W	21	52	9.5		
W	H	S	8	56	12.5		
S	W	H	6	56	16.7		
H	H	H	18	52	11.1	3 H	52
H	H	W	12	51	16.7	1 W, 2 H	55
W	H	H	7	57	0		
H	W	H	8	62	38		
S	W	W	6	52	33.3	2 W, 1S	56
W	S	W	11	57	18.2		
W	W	S	4	68	50		
W	H	W	4	47	25	2 W, 1 H	60
W	W	H	8	55	62.5		
H	W	W	7	72	14.3		
W	W	W	2	72	50	3 W	72
Total			218	51	12.4		

F_2_ lines were assessed for days to flowering (DTF) in the field in Saskatoon, Canada. Plants were classified as true winter types if no flowering was detected before Sept. 1. DTF represents the average DTF for all lines with the same genotype, and Avg. DTF represents the average DTF for all lines within the same genotype group.

W, winter allele; S, spring allele; H, heterozygous; KASP, Kompetitive Allele-Specific PCR.

## 4 Discussion

For a species’ survival, it is crucial to temporally synchronize development with the occurrence of favorable environmental conditions; accordingly, plants exhibit different life history strategies. Annuality, the completion of a plant’s life cycle in one growing season, is an adaptive evolutionary trait that emerged during the domestication of crop species from perennial ancestors, which can persist for several years (Ågren et al., 2017). Winter annuals, which require vernalization to initiate the development of reproductive tissues, have evolved in temperate climates to avoid flowering shortly before winter. Approximately a decade ago, winter annual biotypes of camelina drew the interest of plant breeders and agronomists due to their exceptional level of winter hardiness and compatibility with innovative cropping systems (Berti et al., 2015; Gesch et al., 2014). Most research on the winter camelina crop has been conducted using ‘Joelle’, a publicly available winter cultivar developed at Limagrain (France).

QTL mapping was employed to identify genomic regions underlying flowering time and gain insights into the genetic architecture of vernalization requirement in *C. sativa*, utilizing ‘Joelle’ as the source of winter hardiness. Two major QTLs were identified on chromosomes 8 (subgenome 1) and 13 (subgenome 2), which explained 14.3% and 21.9% of the phenotypic variation, respectively, and which both co-localized with orthologs of *FLC*, a well-characterized gene, which has been shown to be involved in the control of winter annual behavior in *Arabidopsis thaliana* (Michaels and Amasino, 1999; Swiezewski et al., 2009) and other Brassicaceae species (Schiessl et al., 2019; Takada et al., 2019), including camelina (Anderson et al., 2018; Chao et al., 2019; Chaudhary et al., 2023; Kandel et al., 2024).

Previously, one of the three *C. sativa FLC* orthologs, located on chromosome 20 (subgenome 3), was shown to be differentially expressed in the winter-type cultivar ‘Joelle’ when compared to the spring-type cultivar ‘CO46’, prior to and in response to vernalization. It was therefore proposed to be the main determinant for vernalization requirement in *C. sativa* (Anderson et al., 2018). Subsequent QTL analysis for flowering time in a population derived from a ‘Joelle’ × ‘CO46’ cross, however, did not identify a QTL on chromosome 20, but rather two major QTLs on chromosomes 8 and 13, both co-localizing with *FLC* genes (Kandel et al., 2024), which corroborates our own observations. Chaudhary et al. (2023) identified three major QTLs for flowering time and vernalization requirement: two on chromosomes 13 and 20 in a population derived from an interspecific cross between spring-type *C. sativa* and winter-type *Camelina alyssum* and one on chromosome 8 in a population derived from the intraspecific cross between spring-type *C. sativa* and semi-winter-type *C. sativa* ssp. *pilosa*. Again, all three QTL regions contained orthologs of *FLC*. Similarly, the mapping of QTLs for flowering time in a spring × spring population identified, among others, a QTL on chromosome 8 encompassing a *FLC* gene (Li et al., 2021), and a genome-wide association study (GWAS) of a spring camelina diversity panel resulted in the identification of significant SNPs located in the upstream and downstream regions of the *FLC* copy on chromosome 8 (Lily et al., 2021).

*FLC* has been proposed as candidate gene for the regulation of flowering time in previous QTL studies and GWAS in species that are members of the same botanical family as camelina, such as *A. thaliana* (Salomé et al., 2011; Brachi et al., 2010), *Brassica napus* (rapeseed and canola) (Tadege et al., 2001; Hou et al., 2012; Schiessl et al., 2019), and the latter’s progenitor species *Brassica oleracea* (Okazaki et al., 2007; Irwin et al., 2016) and *Brassica rapa* (Wu et al., 2012; Yuan et al., 2009) (reviewed in Leijten et al., 2018). A recent study on freezing tolerance in *C. sativa* found that QTLs for this trait also co-locate with the *FLC* orthologs on chromosomes 8 and 13 (Shaikh et al., 2023). This suggests that *FLC* may be a key regulator for multiple physiological processes in camelina, as was previously proposed for *A. thaliana* (Deng et al., 2011).

The co-localization of *FLC* with QTLs for vernalization requirement and its established role in regulating flowering time make the *Csa.FLC* genes ideal targets for the development of molecular markers to assist in the identification of spring- and winter-type camelina plants. For each *FLC* ortholog, spring and winter alleles showed distinct differences in their gene sequence, with the nucleotide sequence of spring alleles very likely rendering them non-functional or not expressed. As noted previously (Chaudhary et al., 2023), the *Csa.FLC.C08* spring allele differed from the winter allele through a 3-bp deletion. This deletion causes a loss of glutamine in proximity to a binding pocket of the corresponding enzyme (unpublished data), which may impact its functionality. This hypothesis is supported by the fact that in spring types, *Csa.FLC.C08* is expressed at relatively high levels independent of temperature (Chaudhary et al., 2023). The alignment of the *Csa.FLC.C13* alleles identified a large insert close to the 5′ end of intron 1, which is present in all spring-type *C. sativa* alleles. Noteworthily, this insert is lacking from the *Cmi.FLC.C13* allele of winter-type *C. microcarpa* CN119205, which otherwise has all of the SNPs and INDELs common to the spring-type *C. sativa* lines under study. This provides grounds for the hypothesis that the intron 1 insert is causal for non-functionality and/or the lack of expression of the spring allele of *Csa.FLC.C13* documented by Chaudhary et al. (2023). Intron 1 of *Csa.FLC.C13* may be a *cis*-acting gene region that, through the process of alternative splicing, contributes to the regulation of flowering time in *Camelina* spp., similar to instances where sequence differences in non-coding regions of *FLC* have been associated with differences in flowering time in *Arabidopsis* (Shindo et al., 2005; Li et al., 2014, 2015; Schiessl et al., 2019) and *B. rapa* (Yuan et al., 2009; Kitamoto et al., 2014; Wu et al., 2012) and even correlated with the divergence of annuality and perenniality in *Brassica* species (Kiefer et al., 2017). Although we did not identify a QTL for vernalization requirement on chromosome 20 and in association with *Csa.FLC.C20*, the results of previous studies (Anderson et al., 2018; Chaudhary et al., 2023) and a desire for a comprehensive analysis of *FLC*’s role in the genetic architecture of vernalization requirement in *C. sativa* led us to include *Csa.FLC.C20* in subsequent investigations. Sequence analysis confirmed previous results (Anderson et al., 2018; Chao et al., 2019) that found a 1-bp frameshift mutation in exon 5 of the spring allele of *Csa.FLC.C20*, which results in a disrupted reading frame and consequently a non-functional enzyme. In addition to non-functionality, at ambient temperatures, *Csa.FLC.C20* is expressed at a much lower level in spring types than in winter types (Anderson et al., 2018; Chaudhary et al., 2023). We used the previously described INDEL in exon 5 to develop KASP primers for *Csa.FLC.C20*; we chose an A/G SNP 700 bp before the start codon and a G/T SNP 673 bp before exon 2 for *Csa.FLC.C08* and *Csa.FLC.C13*, respectively. As we were able to demonstrate, the developed co-dominant KASP markers allow for the unambiguous identification of homozygous and heterozygous *Csa.FLC* alleles in a high-throughput manner.

For field-grown F_2_ material, the marker results aligned well with observed DTF, with a greater number of winter alleles resulting in later flowering and eventually vernalization requirement. This confirms the usefulness of the markers developed herein for tracking flowering time in camelina. Overall, the results of the field trial indicated that *Csa.FLC.C13* had a stronger effect on delaying flowering time than *Csa.FLC.C08*, which is in agreement with the results of the QTL analysis.

The F_2_ plants comprising the population were derived from more than one F_1_ plant. This certainly is unusual and generally undesired for conducting mapping studies. In addition, we inferred that the inadvertent use of genotypically different F_1_ plants—both homozygous for the spring *Csa.FLC.C13* alleles (SS) and heterozygous (H) ones—were the cause for the distorted segregation that was observed in the Chr13 region containing *Csa.FLC.C13*. However, this proved to be serendipitous because it led us to deduce that the winter type parent, ‘Joelle’, was heterozygous in the region surrounding *Csa.FLC.C13*, which ultimately allowed us to uncover an unexpected degree of genotypic variation at all *FLC* loci in *C. sativa*. Thus, if a single F_1_ plant had been used to form each population, this plant would have been either homozygous for the spring *Csa.FLC.C13* allele or heterozygous, with one spring and one winter allele. In the former case, no segregation would have occurred in the F_2_, and the QTLs on Chr13 would have been missed. In the latter case, segregation would have occurred as expected (1SS:2H:1WW). While this would have resulted in the identification of the QTLs on Chr13, it was the issue of distorted segregation that motivated us to investigate more closely the genotypic variation for *FLC* in ‘Joelle’ and other *C. sativa* germplasm.

The degree of variation we observed for the *FLC* alleles in ‘Joelle’, a commercial cultivar with a strong vernalization requirement and thus expected to be true breeding (homozygous) for winter alleles at all three loci, was extraordinary. Ten different allele combinations were identified, and surprisingly, only approximately 10% of the analyzed plants were indeed homozygous for the winter *FLC* alleles at all loci. Most plants were homozygous for a combination of the winter alleles of *Csa.FLC.C08* and *CsaFLC.C20* (42%) or *Csa.FLC.C08* and *Csa.FLC.C13* (33%), with the consistent winter locus being *Csa.FLC.C08*. Similarly, the three ‘Joelle’ reference genomes were homozygous for either the *Csa.FLC.C13* spring and *Csa.FLC.C20* winter alleles (NCBI and DOE-JGI) or the *Csa.FLC.C13* winter and *Csa.FLC.C20* spring alleles (AAFC reference genome), but all three had the *Csa.FLC.C08* winter locus in the homozygous state. Plants with these combinations of loci needed to undergo vernalization in order to flower. Within the selected seed source, 9% of the ‘Joelle’ plants were homozygous only for the winter alleles of *Csa.FLC.C08*. These plants flowered without vernalization, albeit significantly delayed, indicating that one *FLC* winter locus leads to a semi-winter phenotype, which is in agreement with the results of Chaudhary et al. (2023). Interestingly, *Csa.FLC.C13* winter alleles could only be found in combination with *Csa.FLC.C08* winter alleles; 6% of ‘Joelle’ plants were heterozygous at one *Csa.FLC* locus at least, which corroborates our earlier findings in the ‘Joelle’ × ‘SES0787LS’ mapping population and, as does the existence of different allele combinations, also indicates that the cultivar ‘Joelle’ is still segregating. In hindsight, previous work had hinted at this phenomenon specifically for *Csa.FLC.C20*. Anderson et al. (2018) reported that the 1-bp deletion that is characteristic of the spring allele of this gene was present in both ‘CO46’ (spring) and ‘Joelle’ (winter), with greater frequency in the spring cultivar. This means that also in their study, ‘Joelle’ (and ‘CO46’) possessed both spring and winter *Csa.FLC.C20* alleles. However, the present study is the first to draw the conclusion that the cultivar ‘Joelle’ constitutes a collection of genotypes and is still undergoing genetic segregation.

In order to validate their general utility and to determine the degree of variation at *FLC* in other germplasm, we tested the molecular markers developed in this study for all three *FLC* loci on a number of different winter *C. sativa* (13), winter-type *C. microcarpa* (3), and spring *C. sativa* (53) cultivars and accessions. All spring *C. sativa* lines were found to be homozygous for the *Csa.FLC.C08* and *Csa.FLC.C13* spring alleles; however, out of 53 lines, 13 were homozygous for the *Csa.FLC.C20* winter allele, and eight were heterozygous at this locus. The presence of both spring and winter alleles of *Csa.FLC.C20* in spring-type *C. sativa* germplasm clearly shows that this gene by itself only plays a subordinate role in the regulation of flowering and vernalization requirement, contrary to the proposition made by Anderson et al. (2018). Our hypothesis is corroborated by the fact that neither Kandel et al. (2024) nor the present study identified a QTL on chromosome 20. Although Chaudhary et al. (2023) did identify a chromosome 20 QTL that contained *Csa.FLC.C20*, it was in a population derived from an interspecific cross with *C. alyssum*.

The situation for winter germplasm appeared to be more complex than for spring types. Out of 13 C*. sativa* lines, only three were homozygous (for either spring or winter alleles) at all three *FLC* loci—’Bison’, BSX, and BSX-WG1—which can be traced back to one US breeding program (High Plains Crop Development). The remainder of the lines was segregated for at least one *FLC* ortholog. Given the strong self-fertilizing nature of *C. sativa* (Walsh et al., 2012), this degree of heterozygosity at the *FLC* loci in winter biotypes is surprising.

Only CN120025, a Type 1 hexaploid *C. microcarpa* genotype, had winter alleles in the homozygous state at all three loci; as shown for ‘Joelle’, this combination was remarkably rare. All other winter germplasm carried winter *FLC.C08* and *FLC.C20* alleles, with the exception of, not surprisingly, the tetraploid *C. microcarpa* accession CN119243 and *C. sativa* CN113692, which had *FLC.C08* and *FLC.C13* winter loci, as previously described for some of the ‘Joelle’ plants. Taken together, the comprehensive marker data set for ‘Joelle’ and the results for other winter *C. sativa* germplasm indicate that the combination of at least two winter *FLC* loci from different subgenomes leads to plants requiring vernalization. This may also be the case for *C. microcarpa*; however, additional accessions would need to be analyzed to verify this hypothesis for the wild relative. Chaudhary et al. (2023) drew similar conclusions based on their study of progeny from intra- and interspecific crosses. In the present study, combinations involving *Csa.FLC.C08*—*Csa.FLC.C08* and *Csa.FLC.C13* or *CsaFLC.C08* and *Csa.FLC.C20*—produced a winter phenotype. This observation is consistent with work in *B. napus*, where different *FLC* composition strategies resulted in the same crop type within the Renewable Industrial Products from Rapeseed (RIPR) accession panel (Calderwood et al., 2021).

The only instance where one winter locus was found by itself was in CN113668; this accession had only the winter *Csa.FLC.C08* gene. Like ‘Joelle’ plants with the same genotype, this accession flowered without vernalization, but significantly delayed, and therefore represents a semi-winter type.

Our results strongly suggest that *Csa.FLC.C08* may be the most decisive ortholog for the regulation of flowering time and vernalization requirement in *C. sativa*. Central to this proposition is that the winter allele of this gene by itself causes a semi-winter type, and its combination with the winter alleles of one or both of the other two orthologs results in vernalization requirement. The importance of the other *Csa.FLC* copies is less clear, and it remains to be elucidated how the different orthologs interact with each other to bring about vernalization requirement. Both *Csa.FLC.C13* and *Csa.FLC.C20* act synergistically with *Csa.FLC.C08* to cause vernalization requirement; however, while the results of the QTL analysis and the marker results of field-grown material suggest that *Csa.FLC.C13* has the strongest effect on flowering time and vernalization requirement, the winter alleles of this gene only occur in combination with winter *Csa.FLC.C08* alleles, and *Csa.FLC.C20* by itself did not affect flowering time. Nevertheless, what the present study clearly shows is that all three orthologs are involved in regulating the transition to reproductive growth in *C. sativa*, with no indication for sub-functionalization or pseudogenization.

## 5 Conclusion

This study is the first to uncover an unexpected degree of variability at the *FLC* loci in spring- and winter-type *C. sativa* germplasm and to describe the development of universally applicable molecular markers that distinguish spring from winter alleles for all three orthologs. It is our hope that going forward, the developed KASP markers may serve as tools for winter camelina breeders to identify suitable parent plants to be used in crosses and to enrich segregating breeding populations for winter alleles.

An ideal winter cultivar combines a robust vernalization requirement to prevent flowering before winter with a quick resumption of growth and flowering in the spring, which is essential for early maturity. Depending on the environment—mainly defined by the length of winter—different combinations of winter *FLC* alleles may be required to facilitate both traits; the results of this study and the tools developed herein are a first step to designing winter camelina cultivars that are optimized for different growing regions. Near-isogenic ‘Joelle’ lines with different combinations of homozygous *FLC* alleles are currently being developed; future greenhouse and field experiments using these lines should yield important insights about the independent and combined effects of the different *FLC* copies on flowering time and vernalization requirement in winter camelina.

## Data Availability

The datasets presented in this study can be found in online repositories. The names of the repository/repositories and accession number(s) can be found in the article/**Supplementary Material**.

## Supplementary material

Supplementary Table 1 Camelina germplasm used in the study.

Supplementary Table 2 Selective KASP primer sets for each of the *FLC* alleles. The selective primer associated with the winter phenotype is in bold.

Supplementary Table 3 Mapping data for the F_2_ population derived from the ‘Joelle’ x ‘SES0787LS’ cross. Marker names are based on the SNP position in relation to the DH55 reference genome. The reference and alternative base are listed at the end with the Joelle base bolded. The regions with distorted segregation on Chr13 and Chr20 are highlighted in blue.

Supplementary Table 4 Sequence alignment of the *FLC.C08* gene. Three *C. microcarpa* (CN 120025, CN 119205 and CN 119243); ‘CO46’ (GCA_036971115.1) and ‘Joelle’ (GCA_036769185.1) reference sequences from NCBI; ‘Joelle’ sequences from the AAFC and the DOE-JGI phytozome reference genomes; the DH55 reference genome sequence and 15 spring type *C. sativa* lines were aligned using the EMBL-EBI online tool MUSCLE. All winter Camelina lines are in blue font. Exons are shown in purple font and the SNP utilized for marker development is highlighted in blue. The three-base pair INDEL that distinguishes spring- and winter alleles is highlighted in green.

Supplementary Table 5 Sequence alignment of the *FLC.C13* gene. Three *C. microcarpa* (CN 120025, CN 119205 and CN 119243); ‘CO46’ (GCA_036971115.1) and ‘Joelle’ (GCA_036769185.1) reference sequences from NCBI; ‘Joelle’ sequences from the AAFC and the DOE-JGI phytozome reference genomes; the DH55 reference genome sequence and 15 spring type *C. sativa* lines were aligned using the EMBL-EBI online tool MUSCLE. All winter Camelina lines are in blue font. Exons are shown in purple font and the SNP utilized for marker development is highlighted in blue. SNPs which distinguish the winter and spring alleles are highlighted in green.

Supplementary Table 6 Sequence alignment of the *FLC.C20* gene. Two *C. microcarpa* (CN 120025 and CN 119205; CN 119243 is a tetraploid *C. microcarpa* line that does not have a third subgenome and thus is not included); ‘CO46’ (GCA_036971115.1) and ‘Joelle’ (GCA_036769185.1) reference sequences from NCBI; ‘Joelle’ sequences from the AAFC and the DOE-JGI phytozome reference genomes; the DH55 reference genome sequence and 15 spring type *C. sativa* lines were aligned using the EMBL-EBI online tool MUSCLE. All winter Camelina lines are in blue font. Exons are shown in purple font and the SNP utilized for marker development is highlighted in blue (Anderson et al., 2018). A large insert only present within the ‘CO46’ reference sequence is represented by an N base highlighted in red. The complete sequence for the insert is presented at the end of the alignment and is in red font.

Supplementary Table 7 Summary of the alignment of GBS data from 10 individual ‘Joelle’ plants with the DH55 reference genome. A total of 79740 SNPs were detected, which have a quality score over 30 and less than 20% missing data. As per standard nomenclature ‘0/0’ calls represent the reference genome SNP, ‘0/1’ heterozygous calls, ‘1/1’ represent the alternative SNPs and ‘./.’ represent a no call due to insufficient data.

Supplementary Table 8 Comparison of GBS data from 10 individual ‘Joelle’ plants to three separate ‘Joelle’ reference genomes for the regions surrounding the *Csa.FLC.C13* gene. For reference, the spring-type *Csa.FLC.C13*_KASP marker is present in Joelle.2, Joelle.9 & Joelle.10, the winter-type *Csa.FLC.C13*_KASP marker is present in Joelle.3, Joelle.4, Joelle.5, Joelle.6 & Joelle.8 and a heterozygous *Csa.FLC.C13*_KASP marker is present in Joelle.1 & Joelle.7. As per standard nomenclature ‘0/0’ calls represent the reference genome SNP, ‘0/1’ heterozygous calls, ‘1/1’ represent the alternative SNPs and ‘./.’ represent a no call due to insufficient data.

Supplementary Table 9 Comparison of GBS data from 10 individual ‘Joelle’ plants to three separate ‘Joelle’ reference genomes for the regions surrounding the *Csa.FLC.C20* allele. For reference, the spring-type *Csa.FLC.C20*_KASP marker is present in Joelle.3, Joelle.4, Joelle.6, Joelle.8, Joelle.9 & Joelle.10, the winter-type *Csa.FLC.C20*_KASP marker is present in Joelle.2 and a heterozygous *Csa.FLC.C20*_KASP marker is present in Joelle.1, Joelle.5 & Joelle.7. As per standard nomenclature ‘0/0’ calls represent the reference genome SNP, ‘0/1’ heterozygous calls, ‘1/1’ represent the alternative SNPs and ‘./.’ represent a no call due to insufficient data.

Supplementary Table 10 KASP allele scores for a diverse panel of Camelina lines. Lines in blue font have the winter phenotype. Each line is a bulk of four plants, except for the ‘Joelle’ and ‘SES0787LS’ lines and those marked as REF (reference genomes). The REF samples are predictions based on the *Csa.FLC* gene sequences. Two separate bulks of several CN lines were analyzed and are labelled PRGC #1 and PGRC #2 (Plant Gene Resources of Canada). A score of “W” represents the winter *FLC* allele, a “S” the spring *FLC* allele and “H” the presence of both alleles.
