# Supplementary material for: All roads lead to Rome: QTL analysis for vernalization requirement and dissection of allelic variation uncovered unexpected diversity of FLC loci in Camelina sativa
Source: Front Plant Sci. 2025 Jul 25;16:1639872. doi: 10.3389/fpls.2025.1639872 (PMC12331742; doi:10.3389/fpls.2025.1639872)
Supplement: Supplementary Table 1 — Camelina germplasm used in the study. [file Table1.docx]

**Supplementary File 1.** Camelina germplasm used in the study.

| Cultivar / Accession | Species | Source / Breeding Institution |
| --- | --- | --- |
| Winter types | | |
| ‘Joelle’ | *C. sativa* | Limagrain, via USDA-ARS |
| BSX | *C. sativa* | High Plains Crop Development, United States |
| BSX-WG1 | *C. sativa* | High Plains Crop Development, United States |
| WG-4 | *C. sativa* | High Plains Crop Development, United States |
| ‘Bison’ | *C. sativa* | High Plains Crop Development, United States |
| CN 119243* | *C. microcarpa* (2n = 26) | PGRC |
| CN 120025* | *C. microcarpa* (2n = 38) | PGRC |
| CN 119205* | *C. microcarpa* (2n = 40) | PGRC |
| CN 113660 | *C. sativa* | PGRC |
| CN 113668 | *C. sativa* | PGRC |
| CN 113691 | *C. sativa* | PGRC |
| CN 113692 | *C. sativa* | PGRC |
| PI 650158 | *C. sativa* | USDA-ARS GRIN |
| PI 650168 | *C. sativa* | USDA-ARS GRIN |
| ‘Maczuga’ | *C. sativa* | Department of [Plant Genetics](https://www.sciencedirect.com/topics/agricultural-and-biological-sciences/plant-genetics) and Breeding, Poznań University of Life Sciences, Poland |
| ‘Luna’ | *C. sativa* | Department of [Plant Genetics](https://www.sciencedirect.com/topics/agricultural-and-biological-sciences/plant-genetics) and Breeding, Poznań University of Life Sciences, Poland |
| ‘Lenka’ | *C. sativa* | Department of [Plant Genetics](https://www.sciencedirect.com/topics/agricultural-and-biological-sciences/plant-genetics) and Breeding, Poznań University of Life Sciences, Poland |
| Spring types | | |
| ‘SES0787LS’ | *C. sativa* | Smart Earth Camelina Corp., Canada |
| ‘SES0887IOR’ | *C. sativa* | Smart Earth Camelina Corp., Canada |
| ‘Yellowstone’* | *C. sativa* | Montana State University, United States |
| ‘Glacier’ | *C. sativa* | Montana State University, United States |
| ‘Suneson’ | *C. sativa* | Montana State University, United States |
| ‘Orovata’ | *C. sativa* | Montana State University, United States |
| ‘Jasper’* | *C. sativa* | Montana State University, United States |
| ‘Blaine Creek’* | *C. sativa* | Montana State University, United States |
| ‘Zlatka’ | *C. sativa* | Department of [Plant Genetics](https://www.sciencedirect.com/topics/agricultural-and-biological-sciences/plant-genetics) and Breeding, Poznań University of Life Sciences, Poland |
| ‘Slatka’ | *C. sativa* | Department of [Plant Genetics](https://www.sciencedirect.com/topics/agricultural-and-biological-sciences/plant-genetics) and Breeding, Poznań University of Life Sciences, Poland |
| ‘Omega’ | *C. sativa* | Department of [Plant Genetics](https://www.sciencedirect.com/topics/agricultural-and-biological-sciences/plant-genetics) and Breeding, Poznań University of Life Sciences, Poland |
| ‘Olivia’ | *C. sativa* | Department of [Plant Genetics](https://www.sciencedirect.com/topics/agricultural-and-biological-sciences/plant-genetics) and Breeding, Poznań University of Life Sciences, Poland |
| ‘Hoga’* | *C. sativa* | Department of Agricultural Sciences, The Royal Veterinary and Agricultural University, Copenhagen, Denmark |
| ‘Vega’ | *C. sativa* | Department of Agricultural Sciences, The Royal Veterinary and Agricultural University, Copenhagen, Denmark |
| ‘Eica’ | *C. sativa* | Landwirtschaftszentrum Eichhof, Landesbetrieb Landwirtschaft Hessen, Germany |
| ‘Dolly | *C. sativa* | KWS Saat SE & Co. KGaA, Germany |
| ‘Sonny’ | *C. sativa* | KWS Saat SE & Co. KGaA, Germany |
| ‘AAC 10CS0048’ | *C. sativa* | Agriculture and Agri-Food Canada (AAFC), Canada |
| ‘Jubiljar’ | *C. sativa* | Siberian Research Institute of Feed, Russia |
| ’Chulymskij’ | *C. sativa* | Siberian Research Institute of Feed, Russia |
| ‘Ekaterininskij’ | *C. sativa* | Siberian Research Institute of Feed, Russia |
| ‘Uzhurskij’ | *C. sativa* | Siberian Research Institute of Feed, Russia |
| ‘Cheyenne’ | *C. sativa* | Blue Sun Biodiesel, United States |
| ‘Columbia’ | *C. sativa* | GP Oil, United States |
| ‘Ligena’ | *C. sativa* | Deutsche Saatveredelung (DSV, Germany |
| ‘Calena’ | *C. sativa* | University of Natural Resources and Life Sciences, Vienna, Austria |
| 09CS0040* | *C. sativa* | Agriculture and Agri-Food Canada (AAFC), Canada |
| CAM 236* | *C. sativa* | IPK |
| CAM 241* | *C. sativa* | IPK |
| 17CS1133 (RIL 516)* | *C. sativa* | Washington State University, United States |
| CN 113754* | *C. sativa* | PGRC |
| CN 119300* | *C. sativa* | PGRC |
| CN 120017* | *C. sativa* | PGRC |
| CN 120027* | *C. sativa* | PGRC |
| CN 120030* | *C. sativa* | PGRC |
| CN 119294* | *C. sativa* | PGRC |
| CN 120013* | *C. sativa* | PGRC |
| CN 120019* | *C. sativa* | PGRC |
| PI 304270 | *C. sativa* | USDA-ARS GRIN |
| PI 597833 | *C. sativa* | USDA-ARS GRIN |
| PI 633192 | *C. sativa* | USDA-ARS GRIN |
| PI 633193 | *C. sativa* | USDA-ARS GRIN |
| PI 650140 | *C. sativa* | USDA-ARS GRIN |
| PI 650145 | *C. sativa* | USDA-ARS GRIN |
| PI 650146 | *C. sativa* | USDA-ARS GRIN |
| PI 650147 | *C. sativa* | USDA-ARS GRIN |
| PI 650151 | *C. sativa* | USDA-ARS GRIN |
| PI 650154 | *C. sativa* | USDA-ARS GRIN |
| PI 650160 | *C. sativa* | USDA-ARS GRIN |
| PI 652885 | *C. sativa* | USDA-ARS GRIN |
| PI 652886 | *C. sativa* | USDA-ARS GRIN |
| UGI108 | *C. sativa* | Department of Plant Breeding, Justus-Liebig-University Giessen, Germany |
| UGI212 | *C. sativa* | Department of Plant Breeding, Justus-Liebig-University Giessen, Germany |
| UGI217 | *C. sativa* | Department of Plant Breeding, Justus-Liebig-University Giessen, Germany |

*Lines for which whole-genome sequences were generated (Parkin et al., unpublished).

IPK: Leibniz Institute of Plant Genetics and Crop Plant Research (Germany); PGRC: Plant Gene Resources of Canada; USDA-ARS GRIN: United States Department of Agriculture - Agricultural Research Service Germplasm and Resources Network.
