# Supplementary material for: All roads lead to Rome: QTL analysis for vernalization requirement and dissection of allelic variation uncovered unexpected diversity of FLC loci in Camelina sativa
Source: Front Plant Sci. 2025 Jul 25;16:1639872. doi: 10.3389/fpls.2025.1639872 (PMC12331742; doi:10.3389/fpls.2025.1639872)
Supplement: Supplementary Table 2 — Selective KASP primer sets for each of the FLC alleles. The selective primer associated with the winter phenotype is in bold. [file Table2.docx]

**Supplementary File 2.** Selective KASP primer sets for each of the FLC alleles. The selective primer associated with the winter phenotype is in bold.

| Primer Name | Sequence |
| --- | --- |
|  | **GAAGGTGACCAAGTTCATGCTGAAGGATCTCTCCTCAGAAGGTTTA** |
| FLC-8 | GAAGGTCGGAGTCAACGGATTAAGGATCTCTCCTCAGAAGGTTTG |
|  | AGAACAATCACTATTGAAATATATTTTCCC |
|  | GAAGGTGACCAAGTTCATGCTATTATAAATCAAAATTACTTTCTGTAAGACTCG |
| FLC-13 | **GAAGGTCGGAGTCAACGGATTATAAATCAAAATTACTTTCTGTAAGACTCT** |
|  | CAGACGTTCAATACGTATGTTTAAGCCAA |
|  | **GAAGGTGACCAAGTTCATGCTTCTTTGAGGTTCTCAACAAGCTTCAA** |
| FLC-20 | GAAGGTCGGAGTCAACGGATTCTTTGAGGTTCTCAACAAGCTTCAC |
|  | TTCCGAGAATAACCTTATTGCTTTTATTTG |
