# Supplementary material for: All roads lead to Rome: QTL analysis for vernalization requirement and dissection of allelic variation uncovered unexpected diversity of FLC loci in Camelina sativa
Source: Front Plant Sci. 2025 Jul 25;16:1639872. doi: 10.3389/fpls.2025.1639872 (PMC12331742; doi:10.3389/fpls.2025.1639872)
Supplement: Supplementary Table 4 — Sequence alignment of the FLC.C08 gene. Three C. microcarpa (CN 120025, CN 119205 and CN 119243); ‘CO46’ (GCA_036971115.1) and ‘Joelle’ (GCA_036769185.1) reference sequences from NCBI; ‘Joelle’ sequences from the AAFC and the DOE-JGI phytozome reference genomes; the DH55 reference genome sequence and 15 spring type C. sativa lines were aligned using the EMBL-EBI online tool MUSCLE. All winter Camelina lines are in blue font. Exons are shown in purple font and the SNP utilized for marker development is highlighted in blue. The three-base pair INDEL that distinguishes spring- and winter alleles is highlighted in green. [file Table4.docx]

**Supplementary File 4.** Sequence alignment of the *FLC.C08* gene. Three *C. microcarpa* (CN 120025, CN 119205 and CN 119243); ‘CO46’ (GCA_036971115.1) and ‘Joelle’ (GCA_036769185.1) reference sequences from NCBI; ‘Joelle’ sequences from the AAFC and the DOE-JGI phytozome reference genomes; the DH55 reference genome sequence and 15 spring type *C. sativa* lines were aligned using the EMBL-EBI online tool MUSCLE. All winter Camelina lines are in blue font. Exons are shown in purple font and the SNP utilized for marker development is highlighted in blue. The three-base pair INDEL that distinguishes spring- and winter alleles is highlighted in green.

CN 120025 GAGATGCCTTAAATAAAAAAGATAAAATGACATATCCAGAATAGAAAAAGGAGGTGGGAT

CN 119205 GAGATGCCTTAAATAAAAAAGATAAAATGACATATCCAGAATAGAAAAAGGAGGTGGGGT

CN 119243 GAGATGCCTTAAATAAAAAAGATAAAATGACATATCCAGAATAGAAAAAGGAGGTGGGGT

Joelle NCBI GAGATGCCTTAAATAAAAAAGATAAAATGACATATCCAGAATAGAAAAAGGAGGTGGGGT

Joelle AAFC GAGATGCCTTAAATAAAAAAGATAAAATGACATATCCAGAATAGAAAAAGGAGGTGGGGT

Joelle phyto GAGATGCCTTAAATAAAAAAGATAAAATGACATATCCAGAATAGAAAAAGGAGGTGGGGT

DH55 ref genome GAGATGCCTTAAATAAAAAAGATAAAATGACATATCCAGAATAGAAAAAGGAGGTGGGGT

CN 119300 GAGATGCCTTAAATAAAAAAGATAAAATGACATATCCAGAATAGAAAAAGGAGGTGGGGT

CAM 241 GAGATGCCTTAAATAAAAAAGATAAAATGACATATCCAGAATAGAAAAAGGAGGTGGGGT

CN 113754 GAGATGCCTTAAATAAAAAAGATAAAATGACATATCCAGAATAGAAAAAGGAGGTGGGGT

CAM 236 GAGATGCCTTAAATAAAAAAGATAAAATGACATATCCAGAATAGAAAAAGGAGGTGGGGT

CO46 NCBI GAGATGCCTTAAATAAAAAAGATAAAATGACATATCCAGAATAGAAAAAGGAGGTGGGGT

CN 120013 GAGATGCCTTAAATAAAAAAGATAAAATGACATATCCAGAATAGAAAAAGGAGGTGGGGT

Blaine Creek GAGATGCCTTAAATAAAAAAGATAAAATGACATATCCAGAATAGAAAAAGGAGGTGGGGT

09-CS0040 GAGATGCCTTAAATAAAAAAGATAAAATGACATATCCAGAATAGAAAAAGGAGGTGGGGT

17CS1133 GAGATGCCTTAAATAAAAAAGATAAAATGACATATCCAGAATAGAAAAAGGAGGTGGGGT

Jasper GAGATGCCTTAAATAAAAAAGATAAAATGACATATCCAGAATAGAAAAAGGAGGTGGGGT

CN 120017 GAGATGCCTTAAATAAAAAAGATAAAATGACATATCCAGAATAGAAAAAGGAGGTGGGGT

CN 120027 GAGATGCCTTAAATAAAAAAGATAAAATGACATATCCAGAATAGAAAAAGGAGGTGGGGT

CN 120030 GAGATGCCTTAAATAAAAAAGATAAAATGACATATCCAGAATAGAAAAAGGAGGTGGGGT

Hoga GAGATGCCTTAAATAAAAAAGATAAAATGACATATCCAGAATAGAAAAAGGAGGTGGGGT

Yellowstone GAGATGCCTTAAATAAAAAAGATAAAATGACATATCCAGAATAGAAAAAGGAGGTGGGGT

CN 119294 GAGATGCCTTAAATAAAAAAGATAAAATGACATATCCAGAATAGAAAAAGGAGGTGGGGT

********************************************************** *

CN 120025 GATTGGATATAAACGATGCCGTACATTCACCCATCCATTCGTAGAAGTGCTTTTCGGATT

CN 119205 GATTGGATATAAACGATGCCGTACATTCACCCATCCATTCGTAGAAGTGCTTTTCGGATT

CN 119243 GATTGGATATAAACGATGCCGTACATTCACCCATCCATTCGTAGAAGTGCTTTTCGGATT

Joelle NCBI GATTGGATATAAACGATGCCGTACATTCACCCATCCATTCGTAGAAGTGCTTTTCGGATT

Joelle AAFC GATTGGATATAAACGATGCCGTACATTCACCCATCCATTCGTAGAAGTGCTTTTCGGATT

Joelle phyto GATTGGATATAAACGATGCCGTACATTCACCCATCCATTCGTAGAAGTGCTTTTCGGATT

DH55 ref genome GATTGGATATAAACGATGCCGTACATTCACCCATCCATTCGTAGAAGTGCTTTTCGGATT

CN 119300 GATTGGATATAAACGATGCCGTACATTCACCCATCCATTCGTAGAAGTGCTTTTCGGATT

CAM 241 GATTGGATATAAACGATGCCGTACATTCACCCATCCATTCGTAGAAGTGCTTTTCGGATT

CN 113754 GATTGGATATAAACGATGCCGTACATTCACCCATCCATTCGTAGAAGTGCTTTTCGGATT

CAM 236 GATTGGATATAAACGATGCCGTACATTCACCCATCCATTCGTAGAAGTGCTTTTCGGATT

CO46 NCBI GATTGGATATAAACGATGCCGTACATTCACCCATCCATTCGTAGAAGTGCTTTTCGGATT

CN 120013 GATTGGATATAAACGATGCCGTACATTCACCCATCCATTCGTAGAAGTGCTTTTCGGATT

Blaine Creek GATTGGATATAAACGATGCCGTACATTCACCCATCCATTCGTAGAAGTGCTTTTCGGATT

09-CS0040 GATTGGATATAAACGATGCCGTACATTCACCCATCCATTCGTAGAAGTGCTTTTCGGATT

17CS1133 GATTGGATATAAACGATGCCGTACATTCACCCATCCATTCGTAGAAGTGCTTTTCGGATT

Jasper GATTGGATATAAACGATGCCGTACATTCACCCATCCATTCGTAGAAGTGCTTTTCGGATT

CN 120017 GATTGGATATAAACGATGCCGTACATTCACCCATCCATTCGTAGAAGTGCTTTTCGGATT

CN 120027 GATTGGATATAAACGATGCCGTACATTCACCCATCCATTCGTAGAAGTGCTTTTCGGATT

CN 120030 GATTGGATATAAACGATGCCGTACATTCACCCATCCATTCGTAGAAGTGCTTTTCGGATT

Hoga GATTGGATATAAACGATGCCGTACATTCACCCATCCATTCGTAGAAGTGCTTTTCGGATT

Yellowstone GATTGGATATAAACGATGCCGTACATTCACCCATCCATTCGTAGAAGTGCTTTTCGGATT

CN 119294 GATTGGATATAAACGATGCCGTACATTCACCCATCCATTCGTAGAAGTGCTTTTCGGATT

************************************************************

CN 120025 AAGAGAGATATCGTATTTTCTCTGCCACAGTTTTGCTAGTAGTGAGAGGTTTTTATATTG

CN 119205 AAGAGAGATATCGTATTTTCTCTGCCACAATTTTGCTAGTAGTGAGAGGTTTTTATATTG

CN 119243 AAGAGAGATATCGTATTTTCTCTGCCACAATTTTGCTAGTAGTGAGAGGTTTTTATATTG

Joelle NCBI AAGAGAGATATCGTATTTTCTCTGCCACAATTTTGCTAGTAGTGAGAGGTTTTTATATTG

Joelle AAFC AAGAGAGATATCGTATTTTCTCTGCCACAATTTTGCTAGTAGTGAGAGGTTTTTATATTG

Joelle phyto AAGAGAGATATCGTATTTTCTCTGCCACAATTTTGCTAGTAGTGAGAGGTTTTTATATTG

DH55 ref genome AAGAGAGATATCGTATTTTCTCTGCCACAATTTTGCTAGTAGTGAGAGGTTTTTATATTG

CN 119300 AAGAGAGATATCGTATTTTCTCTGCCACAATTTTGCTAGTAGTGAGAGGTTTTTATATTG

CAM 241 AAGAGAGATATCGTATTTTCTCTGCCACAATTTTGCTAGTAGTGAGAGGTTTTTATATTG

CN 113754 AAGAGAGATATCGTATTTTCTCTGCCACAATTTTGCTAGTAGTGAGAGGTTTTTATATTG

CAM 236 AAGAGAGATATCGTATTTTCTCTGCCACAATTTTGCTAGTAGTGAGAGGTTTTTATATTG

CO46 NCBI AAGAGAGATATCGTATTTTCTCTGCCACAATTTTGCTAGTAGTGAGAGGTTTTTATATTG

CN 120013 AAGAGAGATATCGTATTTTCTCTGCCACAATTTTGCTAGTAGTGAGAGGTTTTTATATTG

Blaine Creek AAGAGAGATATCGTATTTTCTCTGCCACAATTTTGCTAGTAGTGAGAGGTTTTTATATTG

09-CS0040 AAGAGAGATATCGTATTTTCTCTGCCACAATTTTGCTAGTAGTGAGAGGTTTTTATATTG

17CS1133 AAGAGAGATATCGTATTTTCTCTGCCACAATTTTGCTAGTAGTGAGAGGTTTTTATATTG

Jasper AAGAGAGATATCGTATTTTCTCTGCCACAATTTTGCTAGTAGTGAGAGGTTTTTATATTG

CN 120017 AAGAGAGATATCGTATTTTCTCTGCCACAATTTTGCTAGTAGTGAGAGGTTTTTATATTG

CN 120027 AAGAGAGATATCGTATTTTCTCTGCCACAATTTTGCTAGTAGTGAGAGGTTTTTATATTG

CN 120030 AAGAGAGATATCGTATTTTCTCTGCCACAATTTTGCTAGTAGTGAGAGGTTTTTATATTG

Hoga AAGAGAGATATCGTATTTTCTCTGCCACAATTTTGCTAGTAGTGAGAGGTTTTTATATTG

Yellowstone AAGAGAGATATCGTATTTTCTCTGCCACAATTTTGCTAGTAGTGAGAGGTTTTTATATTG

CN 119294 AAGAGAGATATCGTATTTTCTCTGCCACAATTTTGCTAGTAGTGAGAGGTTTTTATATTG

***************************** ******************************

CN 120025 ACAAATCATAGGCTAAGGAACGTTCCATAGGGTTACCAAACCTTAGGCTTTTTTTTTTTT

CN 119205 ACAAATCATAGGCTAAGAAACGTTCCATAGGGTTACCAAACCTTTCGC--ATTTTTTTTT

CN 119243 ACAAATCATAGGCTAAGAAACGTTCCATAGGGTTACCAAACCTTTCGC--ATTTTTTTTT

Joelle NCBI ACAAATCATAGGCTAAGAAACGTTCCATAGGGTTACCAAACCTTTCGC--ATTTTTTTTT

Joelle AAFC ACAAATCATAGGCTAAGAAACGTTCCATAGGGTTACCAAACCTTTCGC--ATTTTTTTTT

Joelle phyto ACAAATCATAGGCTAAGAAACGTTCCATAGGGTTACCAAACCTTTCGC--ATTTTTTTTT

DH55 ref genome ACAAATCATAGGCTAAGAAACGTTCCATAGGGTTACCAAACCTTTCGC--ATTTTTTTTT

CN 119300 ACAAATCATAGGCTAAGAAACGTTCCATAGGGTTACCAAACCTTTCGC--ATTTTTTTTT

CAM 241 ACAAATCATAGGCTAAGAAACGTTCCATAGGGTTACCAAACCTTTCGC--ATTTTTTTTT

CN 113754 ACAAATCATAGGCTAAGAAACGTTCCATAGGGTTACCAAACCTTTCGC--ATTTTTTTTT

CAM 236 ACAAATCATAGGCTAAGAAACGTTCCATAGGGTTACCAAACCTTTCGC--ATTTTTTTTT

CO46 NCBI ACAAATCATAGGCTAAGAAACGTTCCATAGGGTTACCAAACCTTTCGC--ATTTTTTTTT

CN 120013 ACAAATCATAGGCTAAGAAACGTTCCATAGGGTTACCAAACCTTTCGC--ATTTTTTTTT

Blaine Creek ACAAATCATAGGCTAAGAAACGTTCCATAGGGTTACCAAACCTTTCGC--ATTTTTTTTT

09-CS0040 ACAAATCATAGGCTAAGAAACGTTCCATAGGGTTACCAAACCTTTCGC--ATTTTTTTTT

17CS1133 ACAAATCATAGGCTAAGAAACGTTCCATAGGGTTACCAAACCTTTCGC--ATTTTTTTTT

Jasper ACAAATCATAGGCTAAGAAACGTTCCATAGGGTTACCAAACCTTTCGC--ATTTTTTTTT

CN 120017 ACAAATCATAGGCTAAGAAACGTTCCATAGGGTTACCAAACCTTTCGC--ATTTTTTTTT

CN 120027 ACAAATCATAGGCTAAGAAACGTTCCATAGGGTTACCAAACCTTTCGC--ATTTTTTTTT

CN 120030 ACAAATCATAGGCTAAGAAACGTTCCATAGGGTTACCAAACCTTTCGC--ATTTTTTTTT

Hoga ACAAATCATAGGCTAAGAAACGTTCCATAGGGTTACCAAACCTTTCGC--ATTTTTTTTT

Yellowstone ACAAATCATAGGCTAAGAAACGTTCCATAGGGTTACCAAACCTTTCGC--ATTTTTTTTT

CN 119294 ACAAATCATAGGCTAAGAAACGTTCCATAGGGTTACCAAACCTTTCGC--ATTTTTTTTT

***************** ************************** ** **********

CN 120025 TCTACCAACATTTTTCCATTATATATCTATAGTTAAGTCTCAGCTTATTCTTATTGATTT

CN 119205 TCTACCAACATTTTTCCATTATATATCTATAGTTAAGTCTCAGCTTATTCTTATTGATTT

CN 119243 TCTACCAACATTTTTCCATTATATATCTATAGTTAAGTCTCAGCTTATTCTTATTGATTT

Joelle NCBI TCTACCAACATTTTTCCATTATATATCTATAGTTAAGTCTCAGCTTATTCTTATTGATTT

Joelle AAFC TCTACCAACATTTTTCCATTATATATCTATAGTTAAGTCTCAGCTTATTCTTATTGATTT

Joelle phyto TCTACCAACATTTTTCCATTATATATCTATAGTTAAGTCTCAGCTTATTCTTATTGATTT

DH55 ref genome TCTACCAACATTTTTCCATTATATATCTATAGTTAAGTCTCAGCTTATTCTTATTGATTT

CN 119300 TCTACCAACATTTTTCCATTATATATCTATAGTTAAGTCTCAGCTTATTCTTATTGATTT

CAM 241 TCTACCAACATTTTTCCATTATATATCTATAGTTAAGTCTCAGCTTATTCTTATTGATTT

CN 113754 TCTACCAACATTTTTCCATTATATATCTATAGTTAAGTCTCAGCTTATTCTTATTGATTT

CAM 236 TCTACCAACATTTTTCCATTATATATCTATAGTTAAGTCTCAGCTTATTCTTATTGATTT

CO46 NCBI TCTACCAACATTTTTCCATTATATATCTATAGTTAAGTCTCAGCTTATTCTTATTGATTT

CN 120013 TCTACCAACATTTTTCCATTATATATCTATAGTTAAGTCTCAGCTTATTCTTATTGATTT

Blaine Creek TCTACCAACATTTTTCCATTATATATCTATAGTTAAGTCTCAGCTTATTCTTATTGATTT

09-CS0040 TCTACCAACATTTTTCCATTATATATCTATAGTTAAGTCTCAGCTTATTCTTATTGATTT

17CS1133 TCTACCAACATTTTTCCATTATATATCTATAGTTAAGTCTCAGCTTATTCTTATTGATTT

Jasper TCTACCAACATTTTTCCATTATATATCTATAGTTAAGTCTCAGCTTATTCTTATTGATTT

CN 120017 TCTACCAACATTTTTCCATTATATATCTATAGTTAAGTCTCAGCTTATTCTTATTGATTT

CN 120027 TCTACCAACATTTTTCCATTATATATCTATAGTTAAGTCTCAGCTTATTCTTATTGATTT

CN 120030 TCTACCAACATTTTTCCATTATATATCTATAGTTAAGTCTCAGCTTATTCTTATTGATTT

Hoga TCTACCAACATTTTTCCATTATATATCTATAGTTAAGTCTCAGCTTATTCTTATTGATTT

Yellowstone TCTACCAACATTTTTCCATTATATATCTATAGTTAAGTCTCAGCTTATTCTTATTGATTT

CN 119294 TCTACCAACATTTTTCCATTATATATCTATAGTTAAGTCTCAGCTTATTCTTATTGATTT

************************************************************

CN 120025 GACTTAAAAATAATATCAGTAAAACATCTTTCAACAAGTCAAACATACCTAGTTATATCC

CN 119205 GACTTAAAAATAATATCAGTAAAACATCTCTCAACAAGTCAAACATACCTTGTTATATCC

CN 119243 GACTTAAAAATAATATCAGTAAAACATCTCTCAACAAGTCAAACATACCTTGTTATATCC

Joelle NCBI GACTTAAAAATAATATCAGTAAAACATCTCTCAACAAGTCAAACATACCTTGTTATATCC

Joelle AAFC GACTTAAAAATAATATCAGTAAAACATCTCTCAACAAGTCAAACATACCTTGTTATATCC

Joelle phyto GACTTAAAAATAATATCAGTAAAACATCTCTCAACAAGTCAAACATACCTTGTTATATCC

DH55 ref genome GACTTAAAAATAATATCAGTAAAACATCTCTCAACAAGTCAAACATACCTTGTTATATCC

CN 119300 GACTTAAAAATAATATCAGTAAAACATCTCTCAACAAGTCAAACATACCTTGTTATATCC

CAM 241 GACTTAAAAATAATATCAGTAAAACATCTCTCAACAAGTCAAACATACCTTGTTATATCC

CN 113754 GACTTAAAAATAATATCAGTAAAACATCTCTCAACAAGTCAAACATACCTTGTTATATCC

CAM 236 GACTTAAAAATAATATCAGTAAAACATCTCTCAACAAGTCAAACATACCTTGTTATATCC

CO46 NCBI GACTTAAAAATAATATCAGTAAAACATCTCTCAACAAGTCAAACATACCTTGTTATATCC

CN 120013 GACTTAAAAATAATATCAGTAAAACATCTCTCAACAAGTCAAACATACCTTGTTATATCC

Blaine Creek GACTTAAAAATAATATCAGTAAAACATCTCTCAACAAGTCAAACATACCTTGTTATATCC

09-CS0040 GACTTAAAAATAATATCAGTAAAACATCTCTCAACAAGTCAAACATACCTTGTTATATCC

17CS1133 GACTTAAAAATAATATCAGTAAAACATCTCTCAACAAGTCAAACATACCTTGTTATATCC

Jasper GACTTAAAAATAATATCAGTAAAACATCTCTCAACAAGTCAAACATACCTTGTTATATCC

CN 120017 GACTTAAAAATAATATCAGTAAAACATCTCTCAACAAGTCAAACATACCTTGTTATATCC

CN 120027 GACTTAAAAATAATATCAGTAAAACATCTCTCAACAAGTCAAACATACCTTGTTATATCC

CN 120030 GACTTAAAAATAATATCAGTAAAACATCTCTCAACAAGTCAAACATACCTTGTTATATCC

Hoga GACTTAAAAATAATATCAGTAAAACATCTCTCAACAAGTCAAACATACCTTGTTATATCC

Yellowstone GACTTAAAAATAATATCAGTAAAACATCTCTCAACAAGTCAAACATACCTTGTTATATCC

CN 119294 GACTTAAAAATAATATCAGTAAAACATCTCTCAACAAGTCAAACATACCTTGTTATATCC

***************************** ******************** *********

CN 120025 AAAAATAATATTAAACAACCGTTTATGAATTATGGGTGCTTAATTTCTAGGATTCCATCT

CN 119205 AAAAATAATATTAAACAACCGTTTATGAATTATGGGTGCTTAATTTCTAGGATTCCATCT

CN 119243 AAAAATAATATTAAACAACCGTTTATGAATTATGGGTGCTTAATTTCTAGGATTCCATCT

Joelle NCBI AAAAATAATATTAAACAACCGTTTATGAATTATGGGTGCTTAATTTCTAGGATTCCATCT

Joelle AAFC AAAAATAATATTAAACAACCGTTTATGAATTATGGGTGCTTAATTTCTAGGATTCCATCT

Joelle phyto AAAAATAATATTAAACAACCGTTTATGAATTATGGGTGCTTAATTTCTAGGATTCCATCT

DH55 ref genome AAAAATAATATTAAACAACCGTTTATGAATTATGGGTGCTTAATTTCTAGGATTCCATCT

CN 119300 AAAAATAATATTAAACAACCGTTTATGAATTATGGGTGCTTAATTTCTAGGATTCCATCT

CAM 241 AAAAATAATATTAAACAACCGTTTATGAATTATGGGTGCTTAATTTCTAGGATTCCATCT

CN 113754 AAAAATAATATTAAACAACCGTTTATGAATTATGGGTGCTTAATTTCTAGGATTCCATCT

CAM 236 AAAAATAATATTAAACAACCGTTTATGAATTATGGGTGCTTAATTTCTAGGATTCCATCT

CO46 NCBI AAAAATAATATTAAACAACCGTTTATGAATTATGGGTGCTTAATTTCTAGGATTCCATCT

CN 120013 AAAAATAATATTAAACAACCGTTTATGAATTATGGGTGCTTAATTTCTAGGATTCCATCT

Blaine Creek AAAAATAATATTAAACAACCGTTTATGAATTATGGGTGCTTAATTTCTAGGATTCCATCT

09-CS0040 AAAAATAATATTAAACAACCGTTTATGAATTATGGGTGCTTAATTTCTAGGATTCCATCT

17CS1133 AAAAATAATATTAAACAACCGTTTATGAATTATGGGTGCTTAATTTCTAGGATTCCATCT

Jasper AAAAATAATATTAAACAACCGTTTATGAATTATGGGTGCTTAATTTCTAGGATTCCATCT

CN 120017 AAAAATAATATTAAACAACCGTTTATGAATTATGGGTGCTTAATTTCTAGGATTCCATCT

CN 120027 AAAAATAATATTAAACAACCGTTTATGAATTATGGGTGCTTAATTTCTAGGATTCCATCT

CN 120030 AAAAATAATATTAAACAACCGTTTATGAATTATGGGTGCTTAATTTCTAGGATTCCATCT

Hoga AAAAATAATATTAAACAACCGTTTATGAATTATGGGTGCTTAATTTCTAGGATTCCATCT

Yellowstone AAAAATAATATTAAACAACCGTTTATGAATTATGGGTGCTTAATTTCTAGGATTCCATCT

CN 119294 AAAAATAATATTAAACAACCGTTTATGAATTATGGGTGCTTAATTTCTAGGATTCCATCT

************************************************************

CN 120025 GTAATTACCAAGTCCATGAATATCATTTGGTCAGTACTTCGTTTATATGGTAATATGGAA

CN 119205 GTAATTACCAAGTCCATGAATATCATTTGGTCAGTACTTCGTTTATATGGTAATATGGAA

CN 119243 GTAATTACCAAGTCCATGAATATCATTTGGTCAGTACTTCGTTTATATGGTAATATGGAA

Joelle NCBI GTAATTACCAAGTCCATGAATATCATTTGGTCAGTACTTCGTTTATATGGTAATATGGAA

Joelle AAFC GTAATTACCAAGTCCATGAATATCATTTGGTCAGTACTTCGTTTATATGGTAATATGGAA

Joelle phyto GTAATTACCAAGTCCATGAATATCATTTGGTCAGTACTTCGTTTATATGGTAATATGGAA

DH55 ref genome GTAATTACCAAGTCCATGAATATCATTTGGTCAGTACTTCGTTTATATGGTAATATGGAA

CN 119300 GTAATTACCAAGTCCATGAATATCATTTGGTCAGTACTTCGTTTATATGGTAATATGGAA

CAM 241 GTAATTACCAAGTCCATGAATATCATTTGGTCAGTACTTCGTTTATATGGTAATATGGAA

CN 113754 GTAATTACCAAGTCCATGAATATCATTTGGTCAGTACTTCGTTTATATGGTAATATGGAA

CAM 236 GTAATTACCAAGTCCATGAATATCATTTGGTCAGTACTTCGTTTATATGGTAATATGGAA

CO46 NCBI GTAATTACCAAGTCCATGAATATCATTTGGTCAGTACTTCGTTTATATGGTAATATGGAA

CN 120013 GTAATTACCAAGTCCATGAATATCATTTGGTCAGTACTTCGTTTATATGGTAATATGGAA

Blaine Creek GTAATTACCAAGTCCATGAATATCATTTGGTCAGTACTTCGTTTATATGGTAATATGGAA

09-CS0040 GTAATTACCAAGTCCATGAATATCATTTGGTCAGTACTTCGTTTATATGGTAATATGGAA

17CS1133 GTAATTACCAAGTCCATGAATATCATTTGGTCAGTACTTCGTTTATATGGTAATATGGAA

Jasper GTAATTACCAAGTCCATGAATATCATTTGGTCAGTACTTCGTTTATATGGTAATATGGAA

CN 120017 GTAATTACCAAGTCCATGAATATCATTTGGTCAGTACTTCGTTTATATGGTAATATGGAA

CN 120027 GTAATTACCAAGTCCATGAATATCATTTGGTCAGTACTTCGTTTATATGGTAATATGGAA

CN 120030 GTAATTACCAAGTCCATGAATATCATTTGGTCAGTACTTCGTTTATATGGTAATATGGAA

Hoga GTAATTACCAAGTCCATGAATATCATTTGGTCAGTACTTCGTTTATATGGTAATATGGAA

Yellowstone GTAATTACCAAGTCCATGAATATCATTTGGTCAGTACTTCGTTTATATGGTAATATGGAA

CN 119294 GTAATTACCAAGTCCATGAATATCATTTGGTCAGTACTTCGTTTATATGGTAATATGGAA

************************************************************

CN 120025 TGTTTTTTTACCTTTATCTCAGGAATCCTAATTATTGGTCTGAACTATAGGGTCTTACCA

CN 119205 TGTTTTTTTACCTTTATTTCAGGAATCCTAATTATTGGTCTGAACTATAGGGTCTTACCA

CN 119243 TGTTTTTTTACCTTTATTTCAGGAATCCTAATTATTGGTCTGAACTATAGGGTCTTACCA

Joelle NCBI TGTTTTTTTACCTTTATTTCAGGAATCCTAATTATTGGTCTGAACTATAGGGTCTTACCA

Joelle AAFC TGTTTTTTTACCTTTATTTCAGGAATCCTAATTATTGGTCTGAACTATAGGGTCTTACCA

Joelle phyto TGTTTTTTTACCTTTATTTCAGGAATCCTAATTATTGGTCTGAACTATAGGGTCTTACCA

DH55 ref genome TGTTTTTTTACCTTTATTTCAGGAATCCTAATTATTGGTCTGAACTATAGGGTCTTACCA

CN 119300 TGTTTTTTTACCTTTATTTCAGGAATCCTAATTATTGGTCTGAACTATAGGGTCTTACCA

CAM 241 TGTTTTTTTACCTTTATTTCAGGAATCCTAATTATTGGTCTGAACTATAGGGTCTTACCA

CN 113754 TGTTTTTTTACCTTTATTTCAGGAATCCTAATTATTGGTCTGAACTATAGGGTCTTACCA

CAM 236 TGTTTTTTTACCTTTATTTCAGGAATCCTAATTATTGGTCTGAACTATAGGGTCTTACCA

CO46 NCBI TGTTTTTTTACCTTTATTTCAGGAATCCTAATTATTGGTCTGAACTATAGGGTCTTACCA

CN 120013 TGTTTTTTTACCTTTATTTCAGGAATCCTAATTATTGGTCTGAACTATAGGGTCTTACCA

Blaine Creek TGTTTTTTTACCTTTATTTCAGGAATCCTAATTATTGGTCTGAACTATAGGGTCTTACCA

09-CS0040 TGTTTTTTTACCTTTATTTCAGGAATCCTAATTATTGGTCTGAACTATAGGGTCTTACCA

17CS1133 TGTTTTTTTACCTTTATTTCAGGAATCCTAATTATTGGTCTGAACTATAGGGTCTTACCA

Jasper TGTTTTTTTACCTTTATTTCAGGAATCCTAATTATTGGTCTGAACTATAGGGTCTTACCA

CN 120017 TGTTTTTTTACCTTTATTTCAGGAATCCTAATTATTGGTCTGAACTATAGGGTCTTACCA

CN 120027 TGTTTTTTTACCTTTATTTCAGGAATCCTAATTATTGGTCTGAACTATAGGGTCTTACCA

CN 120030 TGTTTTTTTACCTTTATTTCAGGAATCCTAATTATTGGTCTGAACTATAGGGTCTTACCA

Hoga TGTTTTTTTACCTTTATTTCAGGAATCCTAATTATTGGTCTGAACTATAGGGTCTTACCA

Yellowstone TGTTTTTTTACCTTTATTTCAGGAATCCTAATTATTGGTCTGAACTATAGGGTCTTACCA

CN 119294 TGTTTTTTTACCTTTATTTCAGGAATCCTAATTATTGGTCTGAACTATAGGGTCTTACCA

***************** ******************************************

CN 120025 AAAAAGCGTAATATATTGAACTAGAGCTCTTAGCATTCTTATCATGAATAATGTTATACC

CN 119205 AAAAAGCGTAATATATTGAACTAGAGCTCTTAGCATTCTTATCATGAATAATGTTATACC

CN 119243 AAAAAGCGTAATATATTGAACTAGAGCTCTTAGCATTCTTATCATGAATAATGTTATACC

Joelle NCBI AAAAAGCGTAATATATTGAACTAGAGCTCTTAGCATTCTTATCATGAATAATGTTATACC

Joelle AAFC AAAAAGCGTAATATATTGAACTAGAGCTCTTAGCATTCTTATCATGAATAATGTTATACC

Joelle phyto AAAAAGCGTAATATATTGAACTAGAGCTCTTAGCATTCTTATCATGAATAATGTTATACC

DH55 ref genome AAAAAGCGTAATATATTGAACTAGAGCTCTTAGCATTCTTATCATGAATAATGTTATACC

CN 119300 AAAAAGCGTAATATATTGAACTAGAGCTCTTAGCATTCTTATCATGAATAATGTTATACC

CAM 241 AAAAAGCGTAATATATTGAACTAGAGCTCTTAGCATTCTTATCATGAATAATGTTATACC

CN 113754 AAAAAGCGTAATATATTGAACTAGAGCTCTTAGCATTCTTATCATGAATAATGTTATACC

CAM 236 AAAAAGCGTAATATATTGAACTAGAGCTCTTAGCATTCTTATCATGAATAATGTTATACC

CO46 NCBI AAAAAGCGTAATATATTGAACTAGAGCTCTTAGCATTCTTATCATGAATAATGTTATACC

CN 120013 AAAAAGCGTAATATATTGAACTAGAGCTCTTAGCATTCTTATCATGAATAATGTTATACC

Blaine Creek AAAAAGCGTAATATATTGAACTAGAGCTCTTAGCATTCTTATCATGAATAATGTTATACC

09-CS0040 AAAAAGCGTAATATATTGAACTAGAGCTCTTAGCATTCTTATCATGAATAATGTTATACC

17CS1133 AAAAAGCGTAATATATTGAACTAGAGCTCTTAGCATTCTTATCATGAATAATGTTATACC

Jasper AAAAAGCGTAATATATTGAACTAGAGCTCTTAGCATTCTTATCATGAATAATGTTATACC

CN 120017 AAAAAGCGTAATATATTGAACTAGAGCTCTTAGCATTCTTATCATGAATAATGTTATACC

CN 120027 AAAAAGCGTAATATATTGAACTAGAGCTCTTAGCATTCTTATCATGAATAATGTTATACC

CN 120030 AAAAAGCGTAATATATTGAACTAGAGCTCTTAGCATTCTTATCATGAATAATGTTATACC

Hoga AAAAAGCGTAATATATTGAACTAGAGCTCTTAGCATTCTTATCATGAATAATGTTATACC

Yellowstone AAAAAGCGTAATATATTGAACTAGAGCTCTTAGCATTCTTATCATGAATAATGTTATACC

CN 119294 AAAAAGCGTAATATATTGAACTAGAGCTCTTAGCATTCTTATCATGAATAATGTTATACC

************************************************************

CN 120025 GGATCCACACTAGCTATATTAGTGAAGCTATATCGAAGAACAAGAAAAATGGCTAAAAAA

CN 119205 GGATCCACACTAGCTATATAAGTGAAGCTATATCGAAGAACAAGAAAAATGGCTAAAAAA

CN 119243 GGATCCACACTAGCTATATAAGTGAAGCTATATCGAAGAACAAGAAAAATGGCTAAAAAA

Joelle NCBI GGATCCACACTAGCTATATAAGTGAAGCTATATCGAAGAACAAGAAAAATGGCTAAAAAA

Joelle AAFC GGATCCACACTAGCTATATAAGTGAAGCTATATCGAAGAACAAGAAAAATGGCTAAAAAA

Joelle phyto GGATCCACACTAGCTATATAAGTGAAGCTATATCGAAGAACAAGAAAAATGGCTAAAAAA

DH55 ref genome GGATCCACACTAGCTATATAAGTGAAGCTATATCGAAGAACAAGAAAAATGGCTAAAAAA

CN 119300 GGATCCACACTAGCTATATAAGTGAAGCTATATCGAAGAACAAGAAAAATGGCTAAAAAA

CAM 241 GGATCCACACTAGCTATATAAGTGAAGCTATATCGAAGAACAAGAAAAATGGCTAAAAAA

CN 113754 GGATCCACACTAGCTATATAAGTGAAGCTATATCGAAGAACAAGAAAAATGGCTAAAAAA

CAM 236 GGATCCACACTAGCTATATAAGTGAAGCTATATCGAAGAACAAGAAAAATGGCTAAAAAA

CO46 NCBI GGATCCACACTAGCTATATAAGTGAAGCTATATCGAAGAACAAGAAAAATGGCTAAAAAA

CN 120013 GGATCCACACTAGCTATATAAGTGAAGCTATATCGAAGAACAAGAAAAATGGCTAAAAAA

Blaine Creek GGATCCACACTAGCTATATAAGTGAAGCTATATCGAAGAACAAGAAAAATGGCTAAAAAA

09-CS0040 GGATCCACACTAGCTATATAAGTGAAGCTATATCGAAGAACAAGAAAAATGGCTAAAAAA

17CS1133 GGATCCACACTAGCTATATAAGTGAAGCTATATCGAAGAACAAGAAAAATGGCTAAAAAA

Jasper GGATCCACACTAGCTATATAAGTGAAGCTATATCGAAGAACAAGAAAAATGGCTAAAAAA

CN 120017 GGATCCACACTAGCTATATAAGTGAAGCTATATCGAAGAACAAGAAAAATGGCTAAAAAA

CN 120027 GGATCCACACTAGCTATATAAGTGAAGCTATATCGAAGAACAAGAAAAATGGCTAAAAAA

CN 120030 GGATCCACACTAGCTATATAAGTGAAGCTATATCGAAGAACAAGAAAAATGGCTAAAAAA

Hoga GGATCCACACTAGCTATATAAGTGAAGCTATATCGAAGAACAAGAAAAATGGCTAAAAAA

Yellowstone GGATCCACACTAGCTATATAAGTGAAGCTATATCGAAGAACAAGAAAAATGGCTAAAAAA

CN 119294 GGATCCACACTAGCTATATAAGTGAAGCTATATCGAAGAACAAGAAAAATGGCTAAAAAA

******************* ****************************************

CN 120025 CATGTATCAGCCATGAAACTGATTAGATATTTAGTAACTATATTGGTGGTAGTAAGAATT

CN 119205 CATGTATCAGCCATGAAACTGATTAGATATTTAGTAACTATATTGGTGGTAGTAAGAATT

CN 119243 CATGTATCAGCCATGAAACTGATTAGATATTTAGTAACTATATTGGTGGTAGTAAGAATT

Joelle NCBI CATGTATCAGCCATGAAACTGATTAGATATTTAGTAACTATATTGGTGGTAGTAAGAATT

Joelle AAFC CATGTATCAGCCATGAAACTGATTAGATATTTAGTAACTATATTGGTGGTAGTAAGAATT

Joelle phyto CATGTATCAGCCATGAAACTGATTAGATATTTAGTAACTATATTGGTGGTAGTAAGAATT

DH55 ref genome CATGTATCAGCCATGAAACTGATTAGATATTTAGTAACTATATTGGTGGTAGTAAGAATT

CN 119300 CATGTATCAGCCATGAAACTGATTAGATATTTAGTAACTATATTGGTGGTAGTAAGAATT

CAM 241 CATGTATCAGCCATGAAACTGATTAGATATTTAGTAACTATATTGGTGGTAGTAAGAATT

CN 113754 CATGTATCAGCCATGAAACTGATTAGATATTTAGTAACTATATTGGTGGTAGTAAGAATT

CAM 236 CATGTATCAGCCATGAAACTGATTAGATATTTAGTAACTATATTGGTGGTAGTAAGAATT

CO46 NCBI CATGTATCAGCCATGAAACTGATTAGATATTTAGTAACTATATTGGTGGTAGTAAGAATT

CN 120013 CATGTATCAGCCATGAAACTGATTAGATATTTAGTAACTATATTGGTGGTAGTAAGAATT

Blaine Creek CATGTATCAGCCATGAAACTGATTAGATATTTAGTAACTATATTGGTGGTAGTAAGAATT

09-CS0040 CATGTATCAGCCATGAAACTGATTAGATATTTAGTAACTATATTGGTGGTAGTAAGAATT

17CS1133 CATGTATCAGCCATGAAACTGATTAGATATTTAGTAACTATATTGGTGGTAGTAAGAATT

Jasper CATGTATCAGCCATGAAACTGATTAGATATTTAGTAACTATATTGGTGGTAGTAAGAATT

CN 120017 CATGTATCAGCCATGAAACTGATTAGATATTTAGTAACTATATTGGTGGTAGTAAGAATT

CN 120027 CATGTATCAGCCATGAAACTGATTAGATATTTAGTAACTATATTGGTGGTAGTAAGAATT

CN 120030 CATGTATCAGCCATGAAACTGATTAGATATTTAGTAACTATATTGGTGGTAGTAAGAATT

Hoga CATGTATCAGCCATGAAACTGATTAGATATTTAGTAACTATATTGGTGGTAGTAAGAATT

Yellowstone CATGTATCAGCCATGAAACTGATTAGATATTTAGTAACTATATTGGTGGTAGTAAGAATT

CN 119294 CATGTATCAGCCATGAAACTGATTAGATATTTAGTAACTATATTGGTGGTAGTAAGAATT

************************************************************

CN 120025 AATGAAGTTTGGCAAAAATATTTCTTTTGTGATAGCTAATGGCCTAATGTAACTATGTTA

CN 119205 AATGAAGTTTGGCAAAAATATTTCTTTTGTGATAGCTAATGGCCTAATGTAACTATGTTA

CN 119243 AATGAAGTTTGGCAAAAATATTTCTTTTGTGATAGCTAATGGCCTAATGTAACTATGTTA

Joelle NCBI AATGAAGTTTGGCAAAAATATTTCTTTTGTGATAGCTAATGGCCTAATGTAACTATGTTA

Joelle AAFC AATGAAGTTTGGCAAAAATATTTCTTTTGTGATAGCTAATGGCCTAATGTAACTATGTTA

Joelle phyto AATGAAGTTTGGCAAAAATATTTCTTTTGTGATAGCTAATGGCCTAATGTAACTATGTTA

DH55 ref genome AATGAAGTTTGGCAAAAATATTTCTTTTGTGATAGCTAATGGCCTAATGTAACTATGTTA

CN 119300 AATGAAGTTTGGCAAAAATATTTCTTTTGTGATAGCTAATGGCCTAATGTAACTATGTTA

CAM 241 AATGAAGTTTGGCAAAAATATTTCTTTTGTGATAGCTAATGGCCTAATGTAACTATGTTA

CN 113754 AATGAAGTTTGGCAAAAATATTTCTTTTGTGATAGCTAATGGCCTAATGTAACTATGTTA

CAM 236 AATGAAGTTTGGCAAAAATATTTCTTTTGTGATAGCTAATGGCCTAATGTAACTATGTTA

CO46 NCBI AATGAAGTTTGGCAAAAATATTTCTTTTGTGATAGCTAATGGCCTAATGTAACTATGTTA

CN 120013 AATGAAGTTTGGCAAAAATATTTCTTTTGTGATAGCTAATGGCCTAATGTAACTATGTTA

Blaine Creek AATGAAGTTTGGCAAAAATATTTCTTTTGTGATAGCTAATGGCCTAATGTAACTATGTTA

09-CS0040 AATGAAGTTTGGCAAAAATATTTCTTTTGTGATAGCTAATGGCCTAATGTAACTATGTTA

17CS1133 AATGAAGTTTGGCAAAAATATTTCTTTTGTGATAGCTAATGGCCTAATGTAACTATGTTA

Jasper AATGAAGTTTGGCAAAAATATTTCTTTTGTGATAGCTAATGGCCTAATGTAACTATGTTA

CN 120017 AATGAAGTTTGGCAAAAATATTTCTTTTGTGATAGCTAATGGCCTAATGTAACTATGTTA

CN 120027 AATGAAGTTTGGCAAAAATATTTCTTTTGTGATAGCTAATGGCCTAATGTAACTATGTTA

CN 120030 AATGAAGTTTGGCAAAAATATTTCTTTTGTGATAGCTAATGGCCTAATGTAACTATGTTA

Hoga AATGAAGTTTGGCAAAAATATTTCTTTTGTGATAGCTAATGGCCTAATGTAACTATGTTA

Yellowstone AATGAAGTTTGGCAAAAATATTTCTTTTGTGATAGCTAATGGCCTAATGTAACTATGTTA

CN 119294 AATGAAGTTTGGCAAAAATATTTCTTTTGTGATAGCTAATGGCCTAATGTAACTATGTTA

************************************************************

CN 120025 TGTGGTAATAATGCACTTAAAAAGAAAATATGCTAGTTAGCTTCATGAGAAGATAAAATT

CN 119205 TGTGGTAATAATACACTTAAAAAGAAAATATGCTAGTTAGCTTCATGAGAAGATAAAATG

CN 119243 TGTGGTAATAATACACTTAAAAAGAAAATATGCTAGTTAGCTTCATGAGAAGATAAAATG

Joelle NCBI TGTGGTAATAATACACTTAAAAAGAAAATATGCTAGTTAGCTTCATGAGAAGATAAAATG

Joelle AAFC TGTGGTAATAATACACTTAAAAAGAAAATATGCTAGTTAGCTTCATGAGAAGATAAAATG

Joelle phyto TGTGGTAATAATACACTTAAAAAGAAAATATGCTAGTTAGCTTCATGAGAAGATAAAATG

DH55 ref genome TGTGGTAATAATACACTTAAAAAGAAAATATGCTAGTTAGCTTCATGAGAAGATAAAATG

CN 119300 TGTGGTAATAATACACTTAAAAAGAAAATATGCTAGTTAGCTTCATGAGAAGATAAAATG

CAM 241 TGTGGTAATAATACACTTAAAAAGAAAATATGCTAGTTAGCTTCATGAGAAGATAAAATG

CN 113754 TGTGGTAATAATACACTTAAAAAGAAAATATGCTAGTTAGCTTCATGAGAAGATAAAATG

CAM 236 TGTGGTAATAATACACTTAAAAAGAAAATATGCTAGTTAGCTTCATGAGAAGATAAAATG

CO46 NCBI TGTGGTAATAATACACTTAAAAAGAAAATATGCTAGTTAGCTTCATGAGAAGATAAAATG

CN 120013 TGTGGTAATAATACACTTAAAAAGAAAATATGCTAGTTAGCTTCATGAGAAGATAAAATG

Blaine Creek TGTGGTAATAATACACTTAAAAAGAAAATATGCTAGTTAGCTTCATGAGAAGATAAAATG

09-CS0040 TGTGGTAATAATACACTTAAAAAGAAAATATGCTAGTTAGCTTCATGAGAAGATAAAATG

17CS1133 TGTGGTAATAATACACTTAAAAAGAAAATATGCTAGTTAGCTTCATGAGAAGATAAAATG

Jasper TGTGGTAATAATACACTTAAAAAGAAAATATGCTAGTTAGCTTCATGAGAAGATAAAATG

CN 120017 TGTGGTAATAATACACTTAAAAAGAAAATATGCTAGTTAGCTTCATGAGAAGATAAAATG

CN 120027 TGTGGTAATAATACACTTAAAAAGAAAATATGCTAGTTAGCTTCATGAGAAGATAAAATG

CN 120030 TGTGGTAATAATACACTTAAAAAGAAAATATGCTAGTTAGCTTCATGAGAAGATAAAATG

Hoga TGTGGTAATAATACACTTAAAAAGAAAATATGCTAGTTAGCTTCATGAGAAGATAAAATG

Yellowstone TGTGGTAATAATACACTTAAAAAGAAAATATGCTAGTTAGCTTCATGAGAAGATAAAATG

CN 119294 TGTGGTAATAATACACTTAAAAAGAAAATATGCTAGTTAGCTTCATGAGAAGATAAAATG

************ **********************************************

CN 120025 TTATAAATTCTAAGCTCCTAATAAGAAAAAATTACCTTAACACACATCAAAGAGAAGAAC

CN 119205 TTATAAATACTAAGCTCCTAATAAGAAAAAATTACCTTAACACACATCAAAGAGAAGAAC

CN 119243 TTATAAATACTAAGCTCCTAATAAGAAAAAATTACCTTAACACACATCAAAGAGAAGAAC

Joelle NCBI TTATAAATACTAAGCTCCTAATAAGAAAAAATTACCTTAACACACATCAAAGAGAAGAAC

Joelle AAFC TTATAAATACTAAGCTCCTAATAAGAAAAAATTACCTTAACACACATCAAAGAGAAGAAC

Joelle phyto TTATAAATACTAAGCTCCTAATAAGAAAAAATTACCTTAACACACATCAAAGAGAAGAAC

DH55 ref genome TTATAAATACTAAGCTCCTAATAAGAAAAAATTACCTTAACACACATCAAAGAGAAGAAC

CN 119300 TTATAAATACTAAGCTCCTAATAAGAAAAAATTACCTTAACACACATCAAAGAGAAGAAC

CAM 241 TTATAAATACTAAGCTCCTAATAAGAAAAAATTACCTTAACACACATCAAAGAGAAGAAC

CN 113754 TTATAAATACTAAGCTCCTAATAAGAAAAAATTACCTTAACACACATCAAAGAGAAGAAC

CAM 236 TTATAAATACTAAGCTCCTAATAAGAAAAAATTACCTTAACACACATCAAAGAGAAGAAC

CO46 NCBI TTATAAATACTAAGCTCCTAATAAGAAAAAATTACCTTAACACACATCAAAGAGAAGAAC

CN 120013 TTATAAATACTAAGCTCCTAATAAGAAAAAATTACCTTAACACACATCAAAGAGAAGAAC

Blaine Creek TTATAAATACTAAGCTCCTAATAAGAAAAAATTACCTTAACACACATCAAAGAGAAGAAC

09-CS0040 TTATAAATACTAAGCTCCTAATAAGAAAAAATTACCTTAACACACATCAAAGAGAAGAAC

17CS1133 TTATAAATACTAAGCTCCTAATAAGAAAAAATTACCTTAACACACATCAAAGAGAAGAAC

Jasper TTATAAATACTAAGCTCCTAATAAGAAAAAATTACCTTAACACACATCAAAGAGAAGAAC

CN 120017 TTATAAATACTAAGCTCCTAATAAGAAAAAATTACCTTAACACACATCAAAGAGAAGAAC

CN 120027 TTATAAATACTAAGCTCCTAATAAGAAAAAATTACCTTAACACACATCAAAGAGAAGAAC

CN 120030 TTATAAATACTAAGCTCCTAATAAGAAAAAATTACCTTAACACACATCAAAGAGAAGAAC

Hoga TTATAAATACTAAGCTCCTAATAAGAAAAAATTACCTTAACACACATCAAAGAGAAGAAC

Yellowstone TTATAAATACTAAGCTCCTAATAAGAAAAAATTACCTTAACACACATCAAAGAGAAGAAC

CN 119294 TTATAAATACTAAGCTCCTAATAAGAAAAAATTACCTTAACACACATCAAAGAGAAGAAC

******** ***************************************************

CN 120025 CAACTGACTTTAAGCATATTAAGATATCAAACCTTTCGGGTTAATTGATACTAACTGCCA

CN 119205 CAACTGACTTTAAGCATATTAAGATATCAAACCTTTCGGGTTAATTGATACTAACTGCCA

CN 119243 CAACTGACTTTAAGCATATTAAGATATCAAACCTTTCGGGTTAATTGATACTAACTGCCA

Joelle NCBI CAACTGACTTTAAGCATATTAAGATATCAAACCTTTCGGGTTAATTGATACTAACTGCCA

Joelle AAFC CAACTGACTTTAAGCATATTAAGATATCAAACCTTTCGGGTTAATTGATACTAACTGCCA

Joelle phyto CAACTGACTTTAAGCATATTAAGATATCAAACCTTTCGGGTTAATTGATACTAACTGCCA

DH55 ref genome CAACTGACTTTAAGCATATTAAGATATCAAACCTTTCGGGTTAATTGATACTAACTGCCA

CN 119300 CAACTGACTTTAAGCATATTAAGATATCAAACCTTTCGGGTTAATTGATACTAACTGCCA

CAM 241 CAACTGACTTTAAGCATATTAAGATATCAAACCTTTCGGGTTAATTGATACTAACTGCCA

CN 113754 CAACTGACTTTAAGCATATTAAGATATCAAACCTTTCGGGTTAATTGATACTAACTGCCA

CAM 236 CAACTGACTTTAAGCATATTAAGATATCAAACCTTTCGGGTTAATTGATACTAACTGCCA

CO46 NCBI CAACTGACTTTAAGCATATTAAGATATCAAACCTTTCGGGTTAATTGATACTAACTGCCA

CN 120013 CAACTGACTTTAAGCATATTAAGATATCAAACCTTTCGGGTTAATTGATACTAACTGCCA

Blaine Creek CAACTGACTTTAAGCATATTAAGATATCAAACCTTTCGGGTTAATTGATACTAACTGCCA

09-CS0040 CAACTGACTTTAAGCATATTAAGATATCAAACCTTTCGGGTTAATTGATACTAACTGCCA

17CS1133 CAACTGACTTTAAGCATATTAAGATATCAAACCTTTCGGGTTAATTGATACTAACTGCCA

Jasper CAACTGACTTTAAGCATATTAAGATATCAAACCTTTCGGGTTAATTGATACTAACTGCCA

CN 120017 CAACTGACTTTAAGCATATTAAGATATCAAACCTTTCGGGTTAATTGATACTAACTGCCA

CN 120027 CAACTGACTTTAAGCATATTAAGATATCAAACCTTTCGGGTTAATTGATACTAACTGCCA

CN 120030 CAACTGACTTTAAGCATATTAAGATATCAAACCTTTCGGGTTAATTGATACTAACTGCCA

Hoga CAACTGACTTTAAGCATATTAAGATATCAAACCTTTCGGGTTAATTGATACTAACTGCCA

Yellowstone CAACTGACTTTAAGCATATTAAGATATCAAACCTTTCGGGTTAATTGATACTAACTGCCA

CN 119294 CAACTGACTTTAAGCATATTAAGATATCAAACCTTTCGGGTTAATTGATACTAACTGCCA

************************************************************

CN 120025 AATTTTAGTTTTGGAGAAGTGGAAGAGTTCAATAGCGATAAAGAGAACAAAAAATTAAGA

CN 119205 AATTTTAGTTTTGGAGAAGTGGAAGAGTTCAATAGCGATAAAGAGAACAAAAAATTAAGA

CN 119243 AATTTTAGTTTTGGAGAAGTGGAAGAGTTCAATAGCGATAAAGAGAACAAAAAATTAAGA

Joelle NCBI AATTTTAGTTTTGGAGAAGTGGAAGAGTTCAATAGCGATAAAGAGAACAAAAAATTAAGA

Joelle AAFC AATTTTAGTTTTGGAGAAGTGGAAGAGTTCAATAGCGATAAAGAGAACAAAAAATTAAGA

Joelle phyto AATTTTAGTTTTGGAGAAGTGGAAGAGTTCAATAGCGATAAAGAGAACAAAAAATTAAGA

DH55 ref genome AATTTTAGTTTTGGAGAAGTGGAAGAGTTCAATAGCGATAAAGAGAACAAAAAATTAAGA

CN 119300 AATTTTAGTTTTGGAGAAGTGGAAGAGTTCAATAGCGATAAAGAGAACAAAAAATTAAGA

CAM 241 AATTTTAGTTTTGGAGAAGTGGAAGAGTTCAATAGCGATAAAGAGAACAAAAAATTAAGA

CN 113754 AATTTTAGTTTTGGAGAAGTGGAAGAGTTCAATAGCGATAAAGAGAACAAAAAATTAAGA

CAM 236 AATTTTAGTTTTGGAGAAGTGGAAGAGTTCAATAGCGATAAAGAGAACAAAAAATTAAGA

CO46 NCBI AATTTTAGTTTTGGAGAAGTGGAAGAGTTCAATAGCGATAAAGAGAACAAAAAATTAAGA

CN 120013 AATTTTAGTTTTGGAGAAGTGGAAGAGTTCAATAGCGATAAAGAGAACAAAAAATTAAGA

Blaine Creek AATTTTAGTTTTGGAGAAGTGGAAGAGTTCAATAGCGATAAAGAGAACAAAAAATTAAGA

09-CS0040 AATTTTAGTTTTGGAGAAGTGGAAGAGTTCAATAGCGATAAAGAGAACAAAAAATTAAGA

17CS1133 AATTTTAGTTTTGGAGAAGTGGAAGAGTTCAATAGCGATAAAGAGAACAAAAAATTAAGA

Jasper AATTTTAGTTTTGGAGAAGTGGAAGAGTTCAATAGCGATAAAGAGAACAAAAAATTAAGA

CN 120017 AATTTTAGTTTTGGAGAAGTGGAAGAGTTCAATAGCGATAAAGAGAACAAAAAATTAAGA

CN 120027 AATTTTAGTTTTGGAGAAGTGGAAGAGTTCAATAGCGATAAAGAGAACAAAAAATTAAGA

CN 120030 AATTTTAGTTTTGGAGAAGTGGAAGAGTTCAATAGCGATAAAGAGAACAAAAAATTAAGA

Hoga AATTTTAGTTTTGGAGAAGTGGAAGAGTTCAATAGCGATAAAGAGAACAAAAAATTAAGA

Yellowstone AATTTTAGTTTTGGAGAAGTGGAAGAGTTCAATAGCGATAAAGAGAACAAAAAATTAAGA

CN 119294 AATTTTAGTTTTGGAGAAGTGGAAGAGTTCAATAGCGATAAAGAGAACAAAAAATTAAGA

************************************************************

CN 120025 TTTCCATTTGAAAGAGTTCAAAGGTGATGGAGAGAAGGATCTCTCCTCAGAAGGTTT**A**GA

CN 119205 TTTCCATTTGAAAGAGTTCAAAGGTGATGGAGAGAAGGATCTCTCCTCAGAAGGTTT**A**GA

CN 119243 TTTCCATTTGAAAGAGTTCAAAGGTGATGGAGAGAAGGATCTCTCCTCAGAAGGTTT**A**GA

Joelle NCBI TTTCCATTTGAAAGAGTTCAAAGGTGATGGAGAGAAGGATCTCTCCTCAGAAGGTTT**A**GA

Joelle AAFC TTTCCATTTGAAAGAGTTCAAAGGTGATGGAGAGAAGGATCTCTCCTCAGAAGGTTT**A**GA

Joelle phyto TTTCCATTTGAAAGAGTTCAAAGGTGATGGAGAGAAGGATCTCTCCTCAGAAGGTTT**A**GA

DH55 ref genome TTTCCATTTGAAAGAGTTCAAAGGTGATGGAGAGAAGGATCTCTCCTCAGAAGGTTT**G**GA

CN 119300 TTTCCATTTGAAAGAGTTCAAAGGTGATGGAGAGAAGGATCTCTCCTCAGAAGGTTT**G**GA

CAM 241 TTTCCATTTGAAAGAGTTCAAAGGTGATGGAGAGAAGGATCTCTCCTCAGAAGGTTT**G**GA

CN 113754 TTTCCATTTGAAAGAGTTCAAAGGTGATGGAGAGAAGGATCTCTCCTCAGAAGGTTT**G**GA

CAM 236 TTTCCATTTGAAAGAGTTCAAAGGTGATGGAGAGAAGGATCTCTCCTCAGAAGGTTT**G**GA

CO46 NCBI TTTCCATTTGAAAGAGTTCAAAGGTGATGGAGAGAAGGATCTCTCCTCAGAAGGTTT**G**GA

CN 120013 TTTCCATTTGAAAGAGTTCAAAGGTGATGGAGAGAAGGATCTCTCCTCAGAAGGTTT**G**GA

Blaine Creek TTTCCATTTGAAAGAGTTCAAAGGTGATGGAGAGAAGGATCTCTCCTCAGAAGGTTT**G**GA

09-CS0040 TTTCCATTTGAAAGAGTTCAAAGGTGATGGAGAGAAGGATCTCTCCTCAGAAGGTTT**G**GA

17CS1133 TTTCCATTTGAAAGAGTTCAAAGGTGATGGAGAGAAGGATCTCTCCTCAGAAGGTTT**G**GA

Jasper TTTCCATTTGAAAGAGTTCAAAGGTGATGGAGAGAAGGATCTCTCCTCAGAAGGTTT**G**GA

CN 120017 TTTCCATTTGAAAGAGTTCAAAGGTGATGGAGAGAAGGATCTCTCCTCAGAAGGTTT**G**GA

CN 120027 TTTCCATTTGAAAGAGTTCAAAGGTGATGGAGAGAAGGATCTCTCCTCAGAAGGTTT**G**GA

CN 120030 TTTCCATTTGAAAGAGTTCAAAGGTGATGGAGAGAAGGATCTCTCCTCAGAAGGTTT**G**GA

Hoga TTTCCATTTGAAAGAGTTCAAAGGTGATGGAGAGAAGGATCTCTCCTCAGAAGGTTT**G**GA

Yellowstone TTTCCATTTGAAAGAGTTCAAAGGTGATGGAGAGAAGGATCTCTCCTCAGAAGGTTT**G**GA

CN 119294 TTTCCATTTGAAAGAGTTCAAAGGTGATGGAGAGAAGGATCTCTCCTCAGAAGGTTT**G**GA

********************************************************* **

CN 120025 TCTCTCTCTACAATGAGGGAAAATATATTTCAATAGTGATTGTTCTTTAAC-TTTGAAGA

CN 119205 TCTCTCTCTACAATGAGGGAAAATATATTTCAATAGTGATTGTTCTTTAACTTTTGAAGA

CN 119243 TCTCTCTCTACAATGAGGGAAAATATATTTCAATAGTGATTGTTCTTTAACTTTTGAAGA

Joelle NCBI TCTCTCTCTACAATGAGGGAAAATATATTTCAATAGTGATTGTTCTTTAACTTTTGAAGA

Joelle AAFC TCTCTCTCTACAATGAGGGAAAATATATTTCAATAGTGATTGTTCTTTAACTTTTGAAGA

Joelle phyto TCTCTCTCTACAATGAGGGAAAATATATTTCAATAGTGATTGTTCTTTAACTTTTGAAGA

DH55 ref genome TCTCTCTCTACAATGAGGGAAAATATATTTCAATAGTGATTGTTCTTTAACTTTTGAAGA

CN 119300 TCTCTCTCTACAATGAGGGAAAATATATTTCAATAGTGATTGTTCTTTAACTTTTGAAGA

CAM 241 TCTCTCTCTACAATGAGGGAAAATATATTTCAATAGTGATTGTTCTTTAACTTTTGAAGA

CN 113754 TCTCTCTCTACAATGAGGGAAAATATATTTCAATAGTGATTGTTCTTTAACTTTTGAAGA

CAM 236 TCTCTCTCTACAATGAGGGAAAATATATTTCAATAGTGATTGTTCTTTAACTTTTGAAGA

CO46 NCBI TCTCTCTCTACAATGAGGGAAAATATATTTCAATAGTGATTGTTCTTTAACTTTTGAAGA

CN 120013 TCTCTCTCTACAATGAGGGAAAATATATTTCAATAGTGATTGTTCTTTAACTTTTGAAGA

Blaine Creek TCTCTCTCTACAATGAGGGAAAATATATTTCAATAGTGATTGTTCTTTAACTTTTGAAGA

09-CS0040 TCTCTCTCTACAATGAGGGAAAATATATTTCAATAGTGATTGTTCTTTAACTTTTGAAGA

17CS1133 TCTCTCTCTACAATGAGGGAAAATATATTTCAATAGTGATTGTTCTTTAACTTTTGAAGA

Jasper TCTCTCTCTACAATGAGGGAAAATATATTTCAATAGTGATTGTTCTTTAACTTTTGAAGA

CN 120017 TCTCTCTCTACAATGAGGGAAAATATATTTCAATAGTGATTGTTCTTTAACTTTTGAAGA

CN 120027 TCTCTCTCTACAATGAGGGAAAATATATTTCAATAGTGATTGTTCTTTAACTTTTGAAGA

CN 120030 TCTCTCTCTACAATGAGGGAAAATATATTTCAATAGTGATTGTTCTTTAACTTTTGAAGA

Hoga TCTCTCTCTACAATGAGGGAAAATATATTTCAATAGTGATTGTTCTTTAACTTTTGAAGA

Yellowstone TCTCTCTCTACAATGAGGGAAAATATATTTCAATAGTGATTGTTCTTTAACTTTTGAAGA

CN 119294 TCTCTCTCTACAATGAGGGAAAATATATTTCAATAGTGATTGTTCTTTAACTTTTGAAGA

*************************************************** ********

CN 120025 GGCGACGGATCAATAGTACCAAAAACAGAATTATAATTTAAATACATATCAATGCATAAT

CN 119205 GGCGACGGATCAATAGTACCAAAAACAGAATTATAATTTAAATACATATCAATGCATAAT

CN 119243 GGCGACGGATCAATAGTACCAAAAACAGAATTATAATTTAAATACATATCAATGCATAAT

Joelle NCBI GGCGACGGATCAATAGTACCAAAAACAGAATTATAATTTAAATACATATCAATGCATAAT

Joelle AAFC GGCGACGGATCAATAGTACCAAAAACAGAATTATAATTTAAATACATATCAATGCATAAT

Joelle phyto GGCGACGGATCAATAGTACCAAAAACAGAATTATAATTTAAATACATATCAATGCATAAT

DH55 ref genome GGCGACGGATCAATAGTACCAAAAACAGAATTATAATTTAAATACATATCAATGCATAAT

CN 119300 GGCGACGGATCAATAGTACCAAAAACAGAATTATAATTTAAATACATATCAATGCATAAT

CAM 241 GGCGACGGATCAATAGTACCAAAAACAGAATTATAATTTAAATACATATCAATGCATAAT

CN 113754 GGCGACGGATCAATAGTACCAAAAACAGAATTATAATTTAAATACATATCAATGCATAAT

CAM 236 GGCGACGGATCAATAGTACCAAAAACAGAATTATAATTTAAATACATATCAATGCATAAT

CO46 NCBI GGCGACGGATCAATAGTACCAAAAACAGAATTATAATTTAAATACATATCAATGCATAAT

CN 120013 GGCGACGGATCAATAGTACCAAAAACAGAATTATAATTTAAATACATATCAATGCATAAT

Blaine Creek GGCGACGGATCAATAGTACCAAAAACAGAATTATAATTTAAATACATATCAATGCATAAT

09-CS0040 GGCGACGGATCAATAGTACCAAAAACAGAATTATAATTTAAATACATATCAATGCATAAT

17CS1133 GGCGACGGATCAATAGTACCAAAAACAGAATTATAATTTAAATACATATCAATGCATAAT

Jasper GGCGACGGATCAATAGTACCAAAAACAGAATTATAATTTAAATACATATCAATGCATAAT

CN 120017 GGCGACGGATCAATAGTACCAAAAACAGAATTATAATTTAAATACATATCAATGCATAAT

CN 120027 GGCGACGGATCAATAGTACCAAAAACAGAATTATAATTTAAATACATATCAATGCATAAT

CN 120030 GGCGACGGATCAATAGTACCAAAAACAGAATTATAATTTAAATACATATCAATGCATAAT

Hoga GGCGACGGATCAATAGTACCAAAAACAGAATTATAATTTAAATACATATCAATGCATAAT

Yellowstone GGCGACGGATCAATAGTACCAAAAACAGAATTATAATTTAAATACATATCAATGCATAAT

CN 119294 GGCGACGGATCAATAGTACCAAAAACAGAATTATAATTTAAATACATATCAATGCATAAT

************************************************************

CN 120025 TTATTATACTATAAATTACTTATGTGGAGAAAACTAGAACGCATATTCATAGTAGTATAA

CN 119205 TTATTATACTATAAATTACTTATGTGGAGAAAACTAGAACGCATATTCATAGTAGTATAA

CN 119243 TTATTATACTATAAATTACTTATGTGGAGAAAACTAGAACGCATATTCATAGTAGTATAA

Joelle NCBI TTATTATACTATAAATTACTTATGTGGAGAAAACTAGAACGCATATTCATAGTAGTATAA

Joelle AAFC TTATTATACTATAAATTACTTATGTGGAGAAAACTAGAACGCATATTCATAGTAGTATAA

Joelle phyto TTATTATACTATAAATTACTTATGTGGAGAAAACTAGAACGCATATTCATAGTAGTATAA

DH55 ref genome TTATTATACTATAAATTACTTATGTGGAGAAAACTAGAACGCATATTCATAGTAGTATAA

CN 119300 TTATTATACTATAAATTACTTATGTGGAGAAAACTAGAACGCATATTCATAGTAGTATAA

CAM 241 TTATTATACTATAAATTACTTATGTGGAGAAAACTAGAACGCATATTCATAGTAGTATAA

CN 113754 TTATTATACTATAAATTACTTATGTGGAGAAAACTAGAACGCATATTCATAGTAGTATAA

CAM 236 TTATTATACTATAAATTACTTATGTGGAGAAAACTAGAACGCATATTCATAGTAGTATAA

CO46 NCBI TTATTATACTATAAATTACTTATGTGGAGAAAACTAGAACGCATATTCATAGTAGTATAA

CN 120013 TTATTATACTATAAATTACTTATGTGGAGAAAACTAGAACGCATATTCATAGTAGTATAA

Blaine Creek TTATTATACTATAAATTACTTATGTGGAGAAAACTAGAACGCATATTCATAGTAGTATAA

09-CS0040 TTATTATACTATAAATTACTTATGTGGAGAAAACTAGAACGCATATTCATAGTAGTATAA

17CS1133 TTATTATACTATAAATTACTTATGTGGAGAAAACTAGAACGCATATTCATAGTAGTATAA

Jasper TTATTATACTATAAATTACTTATGTGGAGAAAACTAGAACGCATATTCATAGTAGTATAA

CN 120017 TTATTATACTATAAATTACTTATGTGGAGAAAACTAGAACGCATATTCATAGTAGTATAA

CN 120027 TTATTATACTATAAATTACTTATGTGGAGAAAACTAGAACGCATATTCATAGTAGTATAA

CN 120030 TTATTATACTATAAATTACTTATGTGGAGAAAACTAGAACGCATATTCATAGTAGTATAA

Hoga TTATTATACTATAAATTACTTATGTGGAGAAAACTAGAACGCATATTCATAGTAGTATAA

Yellowstone TTATTATACTATAAATTACTTATGTGGAGAAAACTAGAACGCATATTCATAGTAGTATAA

CN 119294 TTATTATACTATAAATTACTTATGTGGAGAAAACTAGAACGCATATTCATAGTAGTATAA

************************************************************

CN 120025 GAATTACTTATGTGAAGAAAACTTGTGGTCATACATCTTGCTTCAA-TTTTTTATATACC

CN 119205 GAATTACTTATGTGAAGAAAACTTGTGGTCATACATCTTGCTTCAATTTTTTTTTATACC

CN 119243 GAATTACTTATGTGAAGAAAACTTGTGGTCATACATCTTGCTTCAATTTTTTTTTATACC

Joelle NCBI GAATTACTTATGTGAAGAAAACTTGTGGTCATACATCTTGCTTCAATTTTTTTTTATACC

Joelle AAFC GAATTACTTATGTGAAGAAAACTTGTGGTCATACATCTTGCTTCAATTTTTTTTTATACC

Joelle phyto GAATTACTTATGTGAAGAAAACTTGTGGTCATACATCTTGCTTCAATTTTTTTTTATACC

DH55 ref genome GAATTACTTATGTGAAGAAAACTTGTGGTCATACATCTTGCTTCAATTTTTTTTTATACC

CN 119300 GAATTACTTATGTGAAGAAAACTTGTGGTCATACATCTTGCTTCAATTTTTTTTTATACC

CAM 241 GAATTACTTATGTGAAGAAAACTTGTGGTCATACATCTTGCTTCAATTTTTTTTTATACC

CN 113754 GAATTACTTATGTGAAGAAAACTTGTGGTCATACATCTTGCTTCAATTTTTTTTTATACC

CAM 236 GAATTACTTATGTGAAGAAAACTTGTGGTCATACATCTTGCTTCAATTTTTTTTTATACC

CO46 NCBI GAATTACTTATGTGAAGAAAACTTGTGGTCATACATCTTGCTTCAATTTTTTTTTATACC

CN 120013 GAATTACTTATGTGAAGAAAACTTGTGGTCATACATCTTGCTTCAATTTTTTTTTATACC

Blaine Creek GAATTACTTATGTGAAGAAAACTTGTGGTCATACATCTTGCTTCAATTTTTTTTTATACC

09-CS0040 GAATTACTTATGTGAAGAAAACTTGTGGTCATACATCTTGCTTCAATTTTTTTTTATACC

17CS1133 GAATTACTTATGTGAAGAAAACTTGTGGTCATACATCTTGCTTCAATTTTTTTTTATACC

Jasper GAATTACTTATGTGAAGAAAACTTGTGGTCATACATCTTGCTTCAATTTTTTTTTATACC

CN 120017 GAATTACTTATGTGAAGAAAACTTGTGGTCATACATCTTGCTTCAATTTTTTTTTATACC

CN 120027 GAATTACTTATGTGAAGAAAACTTGTGGTCATACATCTTGCTTCAATTTTTTTTTATACC

CN 120030 GAATTACTTATGTGAAGAAAACTTGTGGTCATACATCTTGCTTCAATTTTTTTTTATACC

Hoga GAATTACTTATGTGAAGAAAACTTGTGGTCATACATCTTGCTTCAATTTTTTTTTATACC

Yellowstone GAATTACTTATGTGAAGAAAACTTGTGGTCATACATCTTGCTTCAA-TTTTTTTTATACC

CN 119294 GAATTACTTATGTGAAGAAAACTTGTGGTCATACATCTTGCTTCAATTTTTTTTTATACC

********************************************** ****** ******

CN 120025 TAAGTATCTGATCTTAGTATTGAACAAATCTTTGATTAATTTCATCGGATGTTTGTTTAT

CN 119205 TAAGTATCTGATCTTAGTATTGAACAAATCTTTGATTAATTTCATCGGATGTTTGTTTAT

CN 119243 TAAGTATCTGATCTTAGTATTGAACAAATCTTTGATTAATTTCATCGGATGTTTGTTTAT

Joelle NCBI TAAGTATCTGATCTTAGTATTGAACAAATCTTTGATTAATTTCATCGGATGTTTGTTTAT

Joelle AAFC TAAGTATCTGATCTTAGTATTGAACAAATCTTTGATTAATTTCATCGGATGTTTGTTTAT

Joelle phyto TAAGTATCTGATCTTAGTATTGAACAAATCTTTGATTAATTTCATCGGATGTTTGTTTAT

DH55 ref genome TAAGTATCTGATCTTAGTATTGAACAAATCTTTGATTAATTTCATCGGATGTTTGTTTAT

CN 119300 TAAGTATCTGATCTTAGTATTGAACAAATCTTTGATTAATTTCATCGGATGTTTGTTTAT

CAM 241 TAAGTATCTGATCTTAGTATTGAACAAATCTTTGATTAATTTCATCGGATGTTTGTTTAT

CN 113754 TAAGTATCTGATCTTAGTATTGAACAAATCTTTGATTAATTTCATCGGATGTTTGTTTAT

CAM 236 TAAGTATCTGATCTTAGTATTGAACAAATCTTTGATTAATTTCATCGGATGTTTGTTTAT

CO46 NCBI TAAGTATCTGATCTTAGTATTGAACAAATCTTTGATTAATTTCATCGGATGTTTGTTTAT

CN 120013 TAAGTATCTGATCTTAGTATTGAACAAATCTTTGATTAATTTCATCGGATGTTTGTTTAT

Blaine Creek TAAGTATCTGATCTTAGTATTGAACAAATCTTTGATTAATTTCATCGGATGTTTGTTTAT

09-CS0040 TAAGTATCTGATCTTAGTATTGAACAAATCTTTGATTAATTTCATCGGATGTTTGTTTAT

17CS1133 TAAGTATCTGATCTTAGTATTGAACAAATCTTTGATTAATTTCATCGGATGTTTGTTTAT

Jasper TAAGTATCTGATCTTAGTATTGAACAAATCTTTGATTAATTTCATCGGATGTTTGTTTAT

CN 120017 TAAGTATCTGATCTTAGTATTGAACAAATCTTTGATTAATTTCATCGGATGTTTGTTTAT

CN 120027 TAAGTATCTGATCTTAGTATTGAACAAATCTTTGATTAATTTCATCGGATGTTTGTTTAT

CN 120030 TAAGTATCTGATCTTAGTATTGAACAAATCTTTGATTAATTTCATCGGATGTTTGTTTAT

Hoga TAAGTATCTGATCTTAGTATTGAACAAATCTTTGATTAATTTCATCGGATGTTTGTTTAT

Yellowstone TAAGTATCTGATCTTAGTATTGAACAAATCTTTGATTAATTTCATCGGATGTTTGTTTAT

CN 119294 TAAGTATCTGATCTTAGTATTGAACAAATCTTTGATTAATTTCATCGGATGTTTGTTTAT

************************************************************

CN 120025 AAATGAAGAATTAAAAATTGATGAAGAGGTATTTTTTTTTTTTAATAAAAAATTAAAGAG

CN 119205 AAATGAAGAATTAAAAATTGATGAAGAGGTAATTTTTTTTTTTAATAAAAAATTAAAGAG

CN 119243 AAATGAAGAATTAAAAATTGATGAAGAGGTAATTTTTTTTTTTAATAAAAAATTAAAGAG

Joelle NCBI AAATGAAGAATTAAAAATTGATGAAGAGGTAATTTTTTTTTTTAATAAAAAATTAAAGAG

Joelle AAFC AAATGAAGAATTAAAAATTGATGAAGAGGTAATTTTTTTTTTTAATAAAAAATTAAAGAG

Joelle phyto AAATGAAGAATTAAAAATTGATGAAGAGGTAATTTTTTTTTTTAATAAAAAATTAAAGAG

DH55 ref genome AAATGAAGAATTAAAAATTGATGAAGAGGTAATTTTTTTTTTTAATAAAAAATTAAAGAG

CN 119300 AAATGAAGAATTAAAAATTGATGAAGAGGTAATTTTTTTTTTTAATAAAAAATTAAAGAG

CAM 241 AAATGAAGAATTAAAAATTGATGAAGAGGTAATTTTTTTTTTTAATAAAAAATTAAAGAG

CN 113754 AAATGAAGAATTAAAAATTGATGAAGAGGTAATTTTTTTTTTTAATAAAAAATTAAAGAG

CAM 236 AAATGAAGAATTAAAAATTGATGAAGAGGTAATTTTTTTTTTTAATAAAAAATTAAAGAG

CO46 NCBI AAATGAAGAATTAAAAATTGATGAAGAGGTAATTTTTTTTTTTAATAAAAAATTAAAGAG

CN 120013 AAATGAAGAATTAAAAATTGATGAAGAGGTAATTTTTTTTTTTAATAAAAAATTAAAGAG

Blaine Creek AAATGAAGAATTAAAAATTGATGAAGAGGTAATTTTTTTTTTTAATAAAAAATTAAAGAG

09-CS0040 AAATGAAGAATTAAAAATTGATGAAGAGGTAATTTTTTTTTTTAATAAAAAATTAAAGAG

17CS1133 AAATGAAGAATTAAAAATTGATGAAGAGGTAATTTTTTTTTTTAATAAAAAATTAAAGAG

Jasper AAATGAAGAATTAAAAATTGATGAAGAGGTAATTTTTTTTTTTAATAAAAAATTAAAGAG

CN 120017 AAATGAAGAATTAAAAATTGATGAAGAGGTAATTTTTTTTTTTAATAAAAAATTAAAGAG

CN 120027 AAATGAAGAATTAAAAATTGATGAAGAGGTAATTTTTTTTTTTAATAAAAAATTAAAGAG

CN 120030 AAATGAAGAATTAAAAATTGATGAAGAGGTAATTTTTTTTTTTAATAAAAAATTAAAGAG

Hoga AAATGAAGAATTAAAAATTGATGAAGAGGTAATTTTTTTTTTTAATAAAAAATTAAAGAG

Yellowstone AAATGAAGAATTAAAAATTGATGAAGAGGTAA-TTTTTTTTTTAATAAAAAATTAAAGAG

CN 119294 AAATGAAGAATTAAAAATTGATGAAGAGGTAATTTTTTTTTTTAATAAAAAATTAAAGAG

******************************* ***************************

CN 120025 CCATTAGAGAAAGCCAACGGAAGAAGAGCAAACGCGGTCGCATGATATTCGTCATGCGGT

CN 119205 CCATTAGAGAAAGCCAATGGAAGAAGAGCAAACGCGGTCGCATGATATTCGTCATGCGGT

CN 119243 CCATTAGAGAAAGCCAATGGAAGAAGAGCAAACGCGGTCGCATGATATTCGTCATGCGGT

Joelle NCBI CCATTAGAGAAAGCCAATGGAAGAAGAGCAAACGCGGTCGCATGATATTCGTCATGCGGT

Joelle AAFC CCATTAGAGAAAGCCAATGGAAGAAGAGCAAACGCGGTCGCATGATATTCGTCATGCGGT

Joelle phyto CCATTAGAGAAAGCCAATGGAAGAAGAGCAAACGCGGTCGCATGATATTCGTCATGCGGT

DH55 ref genome CCATTAGAGAAAGCCAATGGAAGAAGAGCAAACGCGGTCGCATGATATTCGTCATGCGGT

CN 119300 CCATTAGAGAAAGCCAATGGAAGAAGAGCAAACGCGGTCGCATGATATTCGTCATGCGGT

CAM 241 CCATTAGAGAAAGCCAATGGAAGAAGAGCAAACGCGGTCGCATGATATTCGTCATGCGGT

CN 113754 CCATTAGAGAAAGCCAATGGAAGAAGAGCAAACGCGGTCGCATGATATTCGTCATGCGGT

CAM 236 CCATTAGAGAAAGCCAATGGAAGAAGAGCAAACGCGGTCGCATGATATTCGTCATGCGGT

CO46 NCBI CCATTAGAGAAAGCCAATGGAAGAAGAGCAAACGCGGTCGCATGATATTCGTCATGCGGT

CN 120013 CCATTAGAGAAAGCCAATGGAAGAAGAGCAAACGCGGTCGCATGATATTCGTCATGCGGT

Blaine Creek CCATTAGAGAAAGCCAATGGAAGAAGAGCAAACGCGGTCGCATGATATTCGTCATGCGGT

09-CS0040 CCATTAGAGAAAGCCAATGGAAGAAGAGCAAACGCGGTCGCATGATATTCGTCATGCGGT

17CS1133 CCATTAGAGAAAGCCAATGGAAGAAGAGCAAACGCGGTCGCATGATATTCGTCATGCGGT

Jasper CCATTAGAGAAAGCCAATGGAAGAAGAGCAAACGCGGTCGCATGATATTCGTCATGCGGT

CN 120017 CCATTAGAGAAAGCCAATGGAAGAAGAGCAAACGCGGTCGCATGATATTCGTCATGCGGT

CN 120027 CCATTAGAGAAAGCCAATGGAAGAAGAGCAAACGCGGTCGCATGATATTCGTCATGCGGT

CN 120030 CCATTAGAGAAAGCCAATGGAAGAAGAGCAAACGCGGTCGCATGATATTCGTCATGCGGT

Hoga CCATTAGAGAAAGCCAATGGAAGAAGAGCAAACGCGGTCGCATGATATTCGTCATGCGGT

Yellowstone CCATTAGAGAAAGCCAATGGAAGAAGAGCAAACGCGGTCGCATGATATTCGTCATGCGGT

CN 119294 CCATTAGAGAAAGCCAATGGAAGAAGAGCAAACGCGGTCGCATGATATTCGTCATGCGGT

***************** ******************************************

CN 120025 ACACGTGGCAATGTTGCGTTCAAAACGCAACGTTTTTATTCATAAATTTTATTTTCATCA

CN 119205 ACACGTGGCAATGTTGCGTTCAAAACGCAACGTTTTTATTCATAAATTTTATTTTCATCA

CN 119243 ACACGTGGCAATGTTGCGTTCAAAACGCAACGTTTTTATTCATAAATTTTATTTTCATCA

Joelle NCBI ACACGTGGCAATGTTGCGTTCAAAACGCAACGTTTTTATTCATAAATTTTATTTTCATCA

Joelle AAFC ACACGTGGCAATGTTGCGTTCAAAACGCAACGTTTTTATTCATAAATTTTATTTTCATCA

Joelle phyto ACACGTGGCAATGTTGCGTTCAAAACGCAACGTTTTTATTCATAAATTTTATTTTCATCA

DH55 ref genome ACACGTGGCAATGTTGCGTTCAAAACGCAACGTTTTTATTCATAAATTTTATTTTCATCA

CN 119300 ACACGTGGCAATGTTGCGTTCAAAACGCAACGTTTTTATTCATAAATTTTATTTTCATCA

CAM 241 ACACGTGGCAATGTTGCGTTCAAAACGCAACGTTTTTATTCATAAATTTTATTTTCATCA

CN 113754 ACACGTGGCAATGTTGCGTTCAAAACGCAACGTTTTTATTCATAAATTTTATTTTCATCA

CAM 236 ACACGTGGCAATGTTGCGTTCAAAACGCAACGTTTTTATTCATAAATTTTATTTTCATCA

CO46 NCBI ACACGTGGCAATGTTGCGTTCAAAACGCAACGTTTTTATTCATAAATTTTATTTTCATCA

CN 120013 ACACGTGGCAATGTTGCGTTCAAAACGCAACGTTTTTATTCATAAATTTTATTTTCATCA

Blaine Creek ACACGTGGCAATGTTGCGTTCAAAACGCAACGTTTTTATTCATAAATTTTATTTTCATCA

09-CS0040 ACACGTGGCAATGTTGCGTTCAAAACGCAACGTTTTTATTCATAAATTTTATTTTCATCA

17CS1133 ACACGTGGCAATGTTGCGTTCAAAACGCAACGTTTTTATTCATAAATTTTATTTTCATCA

Jasper ACACGTGGCAATGTTGCGTTCAAAACGCAACGTTTTTATTCATAAATTTTATTTTCATCA

CN 120017 ACACGTGGCAATGTTGCGTTCAAAACGCAACGTTTTTATTCATAAATTTTATTTTCATCA

CN 120027 ACACGTGGCAATGTTGCGTTCAAAACGCAACGTTTTTATTCATAAATTTTATTTTCATCA

CN 120030 ACACGTGGCAATGTTGCGTTCAAAACGCAACGTTTTTATTCATAAATTTTATTTTCATCA

Hoga ACACGTGGCAATGTTGCGTTCAAAACGCAACGTTTTTATTCATAAATTTTATTTTCATCA

Yellowstone ACACGTGGCAATGTTGCGTTCAAAACGCAACGTTTTTATTCATAAATTTTATTTTCATCA

CN 119294 ACACGTGGCAATGTTGCGTTCAAAACGCAACGTTTTTATTCATAAATTTTATTTTCATCA

************************************************************

CN 120025 CTCTCGTTTACCCCAAAC-AAAAAAAAAAATCTAGCCCGAGGAAGG--AAAAAAAAAATT

CN 119205 CTCTCGTTTACCCCAAAC-AAAAAAAAAAATCTAGCCCGAGGAAGG-AAAAAAAAAAATT

CN 119243 CTCTCGTTTACCCCAAACAAAAAAAAAAAATCTAGCCCGAGGAAGG-AAAAAAAAAAATT

Joelle NCBI CTCTCGTTTACCCCAAACAAAAAAAAAAAATCTAGCCCGAGGAAGG-AAAAAAAAAAATT

Joelle AAFC CTCTCGTTTACCCCAAACAAAAAAAAAAAATCTAGCCCGAGGAAGG-AAAAAAAAAAATT

Joelle phyto CTCTCGTTTACCCCAAACAAAAAAAAAAAATCTAGCCCGAGGAAGG-AAAAAAAAAAATT

DH55 ref genome CTCTCGTTTACCCCAAAC-AAAAAAAAAAATCTAGCCCGAGGAAGG-AAAAAAAAAAATT

CN 119300 CTCTCGTTTACCCCAAAC--AAAAAAAAAATCTAGCCCGAGGAAGGAAAAAAAAAAAATT

CAM 241 CTCTCGTTTACCCCAAAC--AAAAAAAAAATCTAGCCCGAGGAAGGAAAAAAAAAAAATT

CN 113754 CTCTCGTTTACCCCAAAC-AAAAAAAAAAATCTAGCCCGAGGAAGG-AAAAAAAAAAATT

CAM 236 CTCTCGTTTACCCCAAAC-AAAAAAAAAAATCTAGCCCGAGGAAGG-AAAAAAAAAAATT

CO46 NCBI CTCTCGTTTACCCCAAAC-AAAAAAAAAAATCTAGCCCGAGGAAGG-AAAAAAAAAAATT

CN 120013 CTCTCGTTTACCCCAAAC-AAAAAAAAAAATCTAGCCCGAGGAAGG-AAAAAAAAAAATT

Blaine Creek CTCTCGTTTACCCCAAAC-AAAAAAAAAAATCTAGCCCGAGGAAGG-AAAAAAAAAAATT

09-CS0040 CTCTCGTTTACCCCAAAC-AAAAAAAAAAATCTAGCCCGAGGAAGG-AAAAAAAAAAATT

17CS1133 CTCTCGTTTACCCCAAAC-AAAAAAAAAAATCTAGCCCGAGGAAGG-AAAAAAAAAAATT

Jasper CTCTCGTTTACCCCAAAC-AAAAAAAAAAATCTAGCCCGAGGAAGG-AAAAAAAAAAATT

CN 120017 CTCTCGTTTACCCCAAAC-AAAAAAAAAAATCTAGCCCGAGGAAGG-AAAAAAAAAAATT

CN 120027 CTCTCGTTTACCCCAAAC-AAAAAAAAAAATCTAGCCCGAGGAAGG-AAAAAAAAAAATT

CN 120030 CTCTCGTTTACCCCAAAC-AAAAAAAAAAATCTAGCCCGAGGAAGG-AAAAAAAAAAATT

Hoga CTCTCGTTTACCCCAAAC-AAAAAAAAAAATCTAGCCCGAGGAAGGAAAAAAAAAAAATT

Yellowstone CTCTCGTTTACCCCAAAC--AAAAAAAAAATCTAGCCCGAGGAAGG-AAAAAAAAAAATT

CN 119294 CTCTCGTTTACCCCAAAC---AAAAAAAAATCTAGCCCGAGGAAGG---AAAAAAAAATT

****************** ************************* ***********

CN 120025 AGATTAGACAAAAAAATAGAAAGAAATAAAGGGAAAAAAGGAAAAAAAAAAATAGGAAAG

CN 119205 AGATTAGACAAAAAAATAGAAAGAAATAAAAGGAAAAAAGGAAATAAAAAAATAGGAAAG

CN 119243 AGATTAGACAAAAAAATAGAAAGAAATAAAAGGAAAAAAGGAAATAAAAAAATAGGAAAG

Joelle NCBI AGATTAGACAAAAAAATAGAAAGAAATAAAAGGAAAAAAGGAAATAAAAAAATAGGAAAG

Joelle AAFC AGATTAGACAAAAAAATAGAAAGAAATAAAAGGAAAAAAGGAAATAAAAAAATAGGAAAG

Joelle phyto AGATTAGACAAAAAAATAGAAAGAAATAAAAGGAAAAAAGGAAATAAAAAAATAGGAAAG

DH55 ref genome AGATTAGACAAAAAAATAGAAAGAAATAAAAGGAAAAAAGGAAATAAAAAAATAGGAAAG

CN 119300 AGATTAGACAAAAAAATAGAAAGAAATAAAAGGAAAAAAGGAAATAAAAAAATAGGAAAG

CAM 241 AGATTAGACAAAAAAATAGAAAGAAATAAAAGGAAAAAAGGAAATAAAAAAATAGGAAAG

CN 113754 AGATTAGACAAAAAAATAGAAAGAAATAAAAGGAAAAAAGGAAATAAAAAAATAGGAAAG

CAM 236 AGATTAGACAAAAAAATAGAAAGAAATAAAAGGAAAAAAGGAAATAAAAAAATAGGAAAG

CO46 NCBI AGATTAGACAAAAAAATAGAAAGAAATAAAAGGAAAAAAGGAAATAAAAAAATAGGAAAG

CN 120013 AGATTAGACAAAAAAATAGAAAGAAATAAAAGGAAAAAAGGAAATAAAAAAATAGGAAAG

Blaine Creek AGATTAGACAAAAAAATAGAAAGAAATAAAAGGAAAAAAGGAAATAAAAAAATAGGAAAG

09-CS0040 AGATTAGACAAAAAAATAGAAAGAAATAAAAGGAAAAAAGGAAATAAAAAAATAGGAAAG

17CS1133 AGATTAGACAAAAAAATAGAAAGAAATAAAAGGAAAAAAGGAAATAAAAAAATAGGAAAG

Jasper AGATTAGACAAAAAAATAGAAAGAAATAAAAGGAAAAAAGGAAATAAAAAAATAGGAAAG

CN 120017 AGATTAGACAAAAAAATAGAAAGAAATAAAAGGAAAAAAGGAAATAAAAAAATAGGAAAG

CN 120027 AGATTAGACAAAAAAATAGAAAGAAATAAAAGGAAAAAAGGAAATAAAAAAATAGGAAAG

CN 120030 AGATTAGACAAAAAAATAGAAAGAAATAAAAGGAAAAAAGGAAATAAAAAAATAGGAAAG

Hoga AGATTAGACAAAAAAATAGAAAGAAATAAAAGGAAAAAAGGAAATAAAAAAATAGGAAAG

Yellowstone AGATTAGACAAAAAAATAGAAAGAAATAAAAGGAAAAAAGGAAATAAAAAAATAGGAAAG

CN 119294 AGATTAGACAAAAAAATAGAAAGAAATAAAAGGAAAAAAGGAAATAAAAAAATAGGAAAG

****************************** ************* ***************

CN 120025 GCAAAAAAAAAAAAAGAGAAACGCTTAGTATCTCTCCGGCGACTTGAACCCAAACCTGAG

CN 119205 GC-AAAAAAAAAAAAGAGAAACGCTTAGTATCTCTCCGGCGACTTGAACCCAAACCTGAG

CN 119243 GC-AAAAAAAAAAAAGAGAAACGCTTAGTATCTCTCCGGCGACTTGAACCCAAACCTGAG

Joelle NCBI GC-AAAAAAAAAAAAGAGAAACGCTTAGTATCTCTCCGGCGACTTGAACCCAAACCTGAG

Joelle AAFC GC-AAAAAAAAAAAAGAGAAACGCTTAGTATCTCTCCGGCGACTTGAACCCAAACCTGAG

Joelle phyto GC-AAAAAAAAAAAAGAGAAACGCTTAGTATCTCTCCGGCGACTTGAACCCAAACCTGAG

DH55 ref genome GC-AAAAAAAAAAAAGAGAAACGCTTAGTATCTCTCCGGCGACTTGAACCCAAACCTGAG

CN 119300 GC-AAAAAAAAAAAAGAGAAACGCTTAGTATCTCTCCGGCGACTTGAACCCAAACCTGAG

CAM 241 GC-AAAAAAAAAAAAGAGAAACGCTTAGTATCTCTCCGGCGACTTGAACCCAAACCTGAG

CN 113754 GC-AAAAAAAAAAAAGAGAAACGCTTAGTATCTCTCCGGCGACTTGAACCCAAACCTGAG

CAM 236 GC-AAAAAAAAAAAAGAGAAACGCTTAGTATCTCTCCGGCGACTTGAACCCAAACCTGAG

CO46 NCBI GC-AAAAAAAAAAAAGAGAAACGCTTAGTATCTCTCCGGCGACTTGAACCCAAACCTGAG

CN 120013 GC-AAAAAAAAAAAAGAGAAACGCTTAGTATCTCTCCGGCGACTTGAACCCAAACCTGAG

Blaine Creek GC-AAAAAAAAAAAAGAGAAACGCTTAGTATCTCTCCGGCGACTTGAACCCAAACCTGAG

09-CS0040 GC-AAAAAAAAAAAAGAGAAACGCTTAGTATCTCTCCGGCGACTTGAACCCAAACCTGAG

17CS1133 GC-AAAAAAAAAAAAGAGAAACGCTTAGTATCTCTCCGGCGACTTGAACCCAAACCTGAG

Jasper GC-AAAAAAAAAAAAGAGAAACGCTTAGTATCTCTCCGGCGACTTGAACCCAAACCTGAG

CN 120017 GC-AAAAAAAAAAAAGAGAAACGCTTAGTATCTCTCCGGCGACTTGAACCCAAACCTGAG

CN 120027 GC-AAAAAAAAAAAAGAGAAACGCTTAGTATCTCTCCGGCGACTTGAACCCAAACCTGAG

CN 120030 GC-AAAAAAAAAAAAGAGAAACGCTTAGTATCTCTCCGGCGACTTGAACCCAAACCTGAG

Hoga GC-AAAAAAAAAAAAGAGAAACGCTTAGTATCTCTCCGGCGACTTGAACCCAAACCTGAG

Yellowstone GC-AAAAAAAAAAAAGAGAAACGCTTAGTATCTCTCCGGCGACTTGAACCCAAACCTGAG

CN 119294 GC-AAAAAAAAAAAAGAGAAACGCTTAGTATCTCTCCGGCGACTTGAACCCAAACCTGAG

** *********************************************************

CN 120025 GATCAAATTAGGGCACAAGGGCCTCTCGGAGACTGAAGCTATGGGAAGGAAAAAACTAGA

CN 119205 GATCAAATTAGGGCACAAGGGCCTCTCGGAGACTAAAGCTATGGGAAGGAAAAAACTAGA

CN 119243 GATCAAATTAGGGCACAAGGGCCTCTCGGAGACTAAAGCTATGGGAAGGAAAAAACTAGA

Joelle NCBI GATCAAATTAGGGCACAAGGGCCTCTCGGAGACTAAAGCTATGGGAAGGAAAAAACTAGA

Joelle AAFC GATCAAATTAGGGCACAAGGGCCTCTCGGAGACTAAAGCTATGGGAAGGAAAAAACTAGA

Joelle phyto GATCAAATTAGGGCACAAGGGCCTCTCGGAGACTAAAGCTATGGGAAGGAAAAAACTAGA

DH55 ref genome GATCAAATTAGGGCACAAGGGCCTCTCGGAGACTAAAGCTATGGGAAGGAAAAAACTAGA

CN 119300 GATCAAATTAGGGCACAAGGGCCTCTCGGAGACTAAAGCTATGGGAAGGAAAAAACTAGA

CAM 241 GATCAAATTAGGGCACAAGGGCCTCTCGGAGACTAAAGCTATGGGAAGGAAAAAACTAGA

CN 113754 GATCAAATTAGGGCACAAGGGCCTCTCGGAGACTAAAGCTATGGGAAGGAAAAAACTAGA

CAM 236 GATCAAATTAGGGCACAAGGGCCTCTCGGAGACTAAAGCTATGGGAAGGAAAAAACTAGA

CO46 NCBI GATCAAATTAGGGCACAAGGGCCTCTCGGAGACTAAAGCTATGGGAAGGAAAAAACTAGA

CN 120013 GATCAAATTAGGGCACAAGGGCCTCTCGGAGACTAAAGCTATGGGAAGGAAAAAACTAGA

Blaine Creek GATCAAATTAGGGCACAAGGGCCTCTCGGAGACTAAAGCTATGGGAAGGAAAAAACTAGA

09-CS0040 GATCAAATTAGGGCACAAGGGCCTCTCGGAGACTAAAGCTATGGGAAGGAAAAAACTAGA

17CS1133 GATCAAATTAGGGCACAAGGGCCTCTCGGAGACTAAAGCTATGGGAAGGAAAAAACTAGA

Jasper GATCAAATTAGGGCACAAGGGCCTCTCGGAGACTAAAGCTATGGGAAGGAAAAAACTAGA

CN 120017 GATCAAATTAGGGCACAAGGGCCTCTCGGAGACTAAAGCTATGGGAAGGAAAAAACTAGA

CN 120027 GATCAAATTAGGGCACAAGGGCCTCTCGGAGACTAAAGCTATGGGAAGGAAAAAACTAGA

CN 120030 GATCAAATTAGGGCACAAGGGCCTCTCGGAGACTAAAGCTATGGGAAGGAAAAAACTAGA

Hoga GATCAAATTAGGGCACAAGGGCCTCTCGGAGACTAAAGCTATGGGAAGGAAAAAACTAGA

Yellowstone GATCAAATTAGGGCACAAGGGCCTCTCGGAGACTAAAGCTATGGGAAGGAAAAAACTAGA

CN 119294 GATCAAATTAGGGCACAAGGGCCTCTCGGAGACTAAAGCTATGGGAAGGAAAAAACTAGA

********************************** *************************

CN 120025 AATCAAGCGAATTGAGAACAAAAGTAGCCGACAAGTCACCTTCTCCAAACGTCGCAATGG

CN 119205 AATCAAGCGAATTGAGAACAAAAGTAGCCGACAAGTCACCTTCTCCAAACGTCGCAATGG

CN 119243 AATCAAGCGAATTGAGAACAAAAGTAGCCGACAAGTCACCTTCTCCAAACGTCGCAATGG

Joelle NCBI AATCAAGCGAATTGAGAACAAAAGTAGCCGACAAGTCACCTTCTCCAAACGTCGCAATGG

Joelle AAFC AATCAAGCGAATTGAGAACAAAAGTAGCCGACAAGTCACCTTCTCCAAACGTCGCAATGG

Joelle phyto AATCAAGCGAATTGAGAACAAAAGTAGCCGACAAGTCACCTTCTCCAAACGTCGCAATGG

DH55 ref genome AATCAAGCGAATTGAGAACAAAAGTAGCCGACAAGTCACCTTCTCCAAACGTCGCAATGG

CN 119300 AATCAAGCGAATTGAGAACAAAAGTAGCCGACAAGTCACCTTCTCCAAACGTCGCAATGG

CAM 241 AATCAAGCGAATTGAGAACAAAAGTAGCCGACAAGTCACCTTCTCCAAACGTCGCAATGG

CN 113754 AATCAAGCGAATTGAGAACAAAAGTAGCCGACAAGTCACCTTCTCCAAACGTCGCAATGG

CAM 236 AATCAAGCGAATTGAGAACAAAAGTAGCCGACAAGTCACCTTCTCCAAACGTCGCAATGG

CO46 NCBI AATCAAGCGAATTGAGAACAAAAGTAGCCGACAAGTCACCTTCTCCAAACGTCGCAATGG

CN 120013 AATCAAGCGAATTGAGAACAAAAGTAGCCGACAAGTCACCTTCTCCAAACGTCGCAATGG

Blaine Creek AATCAAGCGAATTGAGAACAAAAGTAGCCGACAAGTCACCTTCTCCAAACGTCGCAATGG

09-CS0040 AATCAAGCGAATTGAGAACAAAAGTAGCCGACAAGTCACCTTCTCCAAACGTCGCAATGG

17CS1133 AATCAAGCGAATTGAGAACAAAAGTAGCCGACAAGTCACCTTCTCCAAACGTCGCAATGG

Jasper AATCAAGCGAATTGAGAACAAAAGTAGCCGACAAGTCACCTTCTCCAAACGTCGCAATGG

CN 120017 AATCAAGCGAATTGAGAACAAAAGTAGCCGACAAGTCACCTTCTCCAAACGTCGCAATGG

CN 120027 AATCAAGCGAATTGAGAACAAAAGTAGCCGACAAGTCACCTTCTCCAAACGTCGCAATGG

CN 120030 AATCAAGCGAATTGAGAACAAAAGTAGCCGACAAGTCACCTTCTCCAAACGTCGCAATGG

Hoga AATCAAGCGAATTGAGAACAAAAGTAGCCGACAAGTCACCTTCTCCAAACGTCGCAATGG

Yellowstone AATCAAGCGAATTGAGAACAAAAGTAGCCGACAAGTCACCTTCTCCAAACGTCGCAATGG

CN 119294 AATCAAGCGAATTGAGAACAAAAGTAGCCGACAAGTCACCTTCTCCAAACGTCGCAATGG

************************************************************

CN 120025 TCTCATCGAGAAAGCTCGTCAGCTTTCTGTTCTCTGTGACGCATCCGTCGCTCTTCTCGT

CN 119205 TCTCATCGAGAAAGCTCGTCAGCTTTCTGTTCTCTGTGACGCATCCGTCGCTCTTCTCGT

CN 119243 TCTCATCGAGAAAGCTCGTCAGCTTTCTGTTCTCTGTGACGCATCCGTCGCTCTTCTCGT

Joelle NCBI TCTCATCGAGAAAGCTCGTCAGCTTTCTGTTCTCTGTGACGCATCCGTCGCTCTTCTCGT

Joelle AAFC TCTCATCGAGAAAGCTCGTCAGCTTTCTGTTCTCTGTGACGCATCCGTCGCTCTTCTCGT

Joelle phyto TCTCATCGAGAAAGCTCGTCAGCTTTCTGTTCTCTGTGACGCATCCGTCGCTCTTCTCGT

DH55 ref genome TCTCATCGAGAAAGCTCGTCAGCTTTCTGTTCTCTGTGACGCATCCGTCGCTCTTCTCGT

CN 119300 TCTCATCGAGAAAGCTCGTCAGCTTTCTGTTCTCTGTGACGCATCCGTCGCTCTTCTCGT

CAM 241 TCTCATCGAGAAAGCTCGTCAGCTTTCTGTTCTCTGTGACGCATCCGTCGCTCTTCTCGT

CN 113754 TCTCATCGAGAAAGCTCGTCAGCTTTCTGTTCTCTGTGACGCATCCGTCGCTCTTCTCGT

CAM 236 TCTCATCGAGAAAGCTCGTCAGCTTTCTGTTCTCTGTGACGCATCCGTCGCTCTTCTCGT

CO46 NCBI TCTCATCGAGAAAGCTCGTCAGCTTTCTGTTCTCTGTGACGCATCCGTCGCTCTTCTCGT

CN 120013 TCTCATCGAGAAAGCTCGTCAGCTTTCTGTTCTCTGTGACGCATCCGTCGCTCTTCTCGT

Blaine Creek TCTCATCGAGAAAGCTCGTCAGCTTTCTGTTCTCTGTGACGCATCCGTCGCTCTTCTCGT

09-CS0040 TCTCATCGAGAAAGCTCGTCAGCTTTCTGTTCTCTGTGACGCATCCGTCGCTCTTCTCGT

17CS1133 TCTCATCGAGAAAGCTCGTCAGCTTTCTGTTCTCTGTGACGCATCCGTCGCTCTTCTCGT

Jasper TCTCATCGAGAAAGCTCGTCAGCTTTCTGTTCTCTGTGACGCATCCGTCGCTCTTCTCGT

CN 120017 TCTCATCGAGAAAGCTCGTCAGCTTTCTGTTCTCTGTGACGCATCCGTCGCTCTTCTCGT

CN 120027 TCTCATCGAGAAAGCTCGTCAGCTTTCTGTTCTCTGTGACGCATCCGTCGCTCTTCTCGT

CN 120030 TCTCATCGAGAAAGCTCGTCAGCTTTCTGTTCTCTGTGACGCATCCGTCGCTCTTCTCGT

Hoga TCTCATCGAGAAAGCTCGTCAGCTTTCTGTTCTCTGTGACGCATCCGTCGCTCTTCTCGT

Yellowstone TCTCATCGAGAAAGCTCGTCAGCTTTCTGTTCTCTGTGACGCATCCGTCGCTCTTCTCGT

CN 119294 TCTCATCGAGAAAGCTCGTCAGCTTTCTGTTCTCTGTGACGCATCCGTCGCTCTTCTCGT

************************************************************

CN 120025 CGTCTCCGCCTCCGGCAAGCTCTACAGCTTCTCCTCCGGTGATAAGTACGTCTTTTCCTT

CN 119205 CGTCTCCGCCTCCGGCAAGCTCTACAGCTTCTCCTCCGGTGATAAGTACGTCTTTTCCTT

CN 119243 CGTCTCCGCCTCCGGCAAGCTCTACAGCTTCTCCTCCGGTGATAAGTACGTCTTTTCCTT

Joelle NCBI CGTCTCCGCCTCCGGCAAGCTCTACAGCTTCTCCTCCGGTGATAAGTACGTCTTTTCCTT

Joelle AAFC CGTCTCCGCCTCCGGCAAGCTCTACAGCTTCTCCTCCGGTGATAAGTACGTCTTTTCCTT

Joelle phyto CGTCTCCGCCTCCGGCAAGCTCTACAGCTTCTCCTCCGGTGATAAGTACGTCTTTTCCTT

DH55 ref genome CGTCTCCGCCTCCGGCAAGCTCTACAGCTTCTCCTCCGGTGATAAGTACGTCTTTTCCTT

CN 119300 CGTCTCCGCCTCCGGCAAGCTCTACAGCTTCTCCTCCGGTGATAAGTACGTCTTTTCCTT

CAM 241 CGTCTCCGCCTCCGGCAAGCTCTACAGCTTCTCCTCCGGTGATAAGTACGTCTTTTCCTT

CN 113754 CGTCTCCGCCTCCGGCAAGCTCTACAGCTTCTCCTCCGGTGATAAGTACGTCTTTTCCTT

CAM 236 CGTCTCCGCCTCCGGCAAGCTCTACAGCTTCTCCTCCGGTGATAAGTACGTCTTTTCCTT

CO46 NCBI CGTCTCCGCCTCCGGCAAGCTCTACAGCTTCTCCTCCGGTGATAAGTACGTCTTTTCCTT

CN 120013 CGTCTCCGCCTCCGGCAAGCTCTACAGCTTCTCCTCCGGTGATAAGTACGTCTTTTCCTT

Blaine Creek CGTCTCCGCCTCCGGCAAGCTCTACAGCTTCTCCTCCGGTGATAAGTACGTCTTTTCCTT

09-CS0040 CGTCTCCGCCTCCGGCAAGCTCTACAGCTTCTCCTCCGGTGATAAGTACGTCTTTTCCTT

17CS1133 CGTCTCCGCCTCCGGCAAGCTCTACAGCTTCTCCTCCGGTGATAAGTACGTCTTTTCCTT

Jasper CGTCTCCGCCTCCGGCAAGCTCTACAGCTTCTCCTCCGGTGATAAGTACGTCTTTTCCTT

CN 120017 CGTCTCCGCCTCCGGCAAGCTCTACAGCTTCTCCTCCGGTGATAAGTACGTCTTTTCCTT

CN 120027 CGTCTCCGCCTCCGGCAAGCTCTACAGCTTCTCCTCCGGTGATAAGTACGTCTTTTCCTT

CN 120030 CGTCTCCGCCTCCGGCAAGCTCTACAGCTTCTCCTCCGGTGATAAGTACGTCTTTTCCTT

Hoga CGTCTCCGCCTCCGGCAAGCTCTACAGCTTCTCCTCCGGTGATAAGTACGTCTTTTCCTT

Yellowstone CGTCTCCGCCTCCGGCAAGCTCTACAGCTTCTCCTCCGGTGATAAGTACGTCTTTTCCTT

CN 119294 CGTCTCCGCCTCCGGCAAGCTCTACAGCTTCTCCTCCGGTGATAAGTACGTCTTTTCCTT

************************************************************

CN 120025 ATCTGGGTTCTCGTTTTTTCCCCCCTTTAAGCTTCGGTTTTGTGCTTTCTCTTTACTTTT

CN 119205 ATCTGGGTTCTCGTTTTTTCCCCCCTTTAAGCTTCGGTTTTGTGCTTTCTCTTTACTTTT

CN 119243 ATCTGGGTTCTCGTTTTTTCCCCCCTTTAAGCTTCGGTTTTGTGCTTTCTCTTTACTTTT

Joelle NCBI ATCTGGGTTCTCGTTTTTTCCCCCCTTTAAGCTTCGGTTTTGTGCTTTCTCTTTACTTTT

Joelle AAFC ATCTGGGTTCTCGTTTTTTCCCCCCTTTAAGCTTCGGTTTTGTGCTTTCTCTTTACTTTT

Joelle phyto ATCTGGGTTCTCGTTTTTTCCCCCCTTTAAGCTTCGGTTTTGTGCTTTCTCTTTACTTTT

DH55 ref genome ATCTGGGTTCTCGTTTTTTCCCCCCTTTAAGCTTCGGTTTTGTGCTTTCTCTTTACTTTT

CN 119300 ATCTGGGTTCTCGTTTTTTCCCCCCTTTAAGCTTCGGTTTTGTGCTTTCTCTTTACTTTT

CAM 241 ATCTGGGTTCTCGTTTTTTCCCCCCTTTAAGCTTCGGTTTTGTGCTTTCTCTTTACTTTT

CN 113754 ATCTGGGTTCTCGTTTTTTCCCCCCTTTAAGCTTCGGTTTTGTGCTTTCTCTTTACTTTT

CAM 236 ATCTGGGTTCTCGTTTTTTCCCCCCTTTAAGCTTCGGTTTTGTGCTTTCTCTTTACTTTT

CO46 NCBI ATCTGGGTTCTCGTTTTTTCCCCCCTTTAAGCTTCGGTTTTGTGCTTTCTCTTTACTTTT

CN 120013 ATCTGGGTTCTCGTTTTTTCCCCCCTTTAAGCTTCGGTTTTGTGCTTTCTCTTTACTTTT

Blaine Creek ATCTGGGTTCTCGTTTTTTCCCCCCTTTAAGCTTCGGTTTTGTGCTTTCTCTTTACTTTT

09-CS0040 ATCTGGGTTCTCGTTTTTTCCCCCCTTTAAGCTTCGGTTTTGTGCTTTCTCTTTACTTTT

17CS1133 ATCTGGGTTCTCGTTTTTTCCCCCCTTTAAGCTTCGGTTTTGTGCTTTCTCTTTACTTTT

Jasper ATCTGGGTTCTCGTTTTTTCCCCCCTTTAAGCTTCGGTTTTGTGCTTTCTCTTTACTTTT

CN 120017 ATCTGGGTTCTCGTTTTTTCCCCCCTTTAAGCTTCGGTTTTGTGCTTTCTCTTTACTTTT

CN 120027 ATCTGGGTTCTCGTTTTTTCCCCCCTTTAAGCTTCGGTTTTGTGCTTTCTCTTTACTTTT

CN 120030 ATCTGGGTTCTCGTTTTTTCCCCCCTTTAAGCTTCGGTTTTGTGCTTTCTCTTTACTTTT

Hoga ATCTGGGTTCTCGTTTTTTCCCCCCTTTAAGCTTCGGTTTTGTGCTTTCTCTTTACTTTT

Yellowstone ATCTGGGTTCTCGTTTTTTCCCCCCTTTAAGCTTCGGTTTTGTGCTTTCTCTTTACTTTT

CN 119294 ATCTGGGTTCTCGTTTTTTCCCCCCTTTAAGCTTCGGTTTTGTGCTTTCTCTTTACTTTT

************************************************************

CN 120025 TCTCTGAAGAAAATAAATATATAAAAAGACACAAATAAAATAAAAACAATTAATGTATAG

CN 119205 TCTCCGAAGAAAATAAATATATAAAAAGACACAAATAAAATAAAAACAATTAATGTATAG

CN 119243 TCTCCGAAGAAAATAAATATATAAAAAGACACAAATAAAATAAAAACAATTAATGTATAG

Joelle NCBI TCTCCGAAGAAAATAAATATATAAAAAGACACAAATAAAATAAAAACAATTAATGTATAG

Joelle AAFC TCTCCGAAGAAAATAAATATATAAAAAGACACAAATAAAATAAAAACAATTAATGTATAG

Joelle phyto TCTCCGAAGAAAATAAATATATAAAAAGACACAAATAAAATAAAAACAATTAATGTATAG

DH55 ref genome TCTCCGAAGAAAATAAATATATAAAAAGACACAAATAAAATAAAAACAATTAATGTATAG

CN 119300 TCTCCGAAGAAAATAAATATATAAAAAGACACAAATAAAATAAAAACAATTAATGTATAG

CAM 241 TCTCCGAAGAAAATAAATATATAAAAAGACACAAATAAAATAAAAACAATTAATGTATAG

CN 113754 TCTCCGAAGAAAATAAATATATAAAAAGACACAAATAAAATAAAAACAATTAATGTATAG

CAM 236 TCTCCGAAGAAAATAAATATATAAAAAGACACAAATAAAATAAAAACAATTAATGTATAG

CO46 NCBI TCTCCGAAGAAAATAAATATATAAAAAGACACAAATAAAATAAAAACAATTAATGTATAG

CN 120013 TCTCCGAAGAAAATAAATATATAAAAAGACACAAATAAAATAAAAACAATTAATGTATAG

Blaine Creek TCTCCGAAGAAAATAAATATATAAAAAGACACAAATAAAATAAAAACAATTAATGTATAG

09-CS0040 TCTCCGAAGAAAATAAATATATAAAAAGACACAAATAAAATAAAAACAATTAATGTATAG

17CS1133 TCTCCGAAGAAAATAAATATATAAAAAGACACAAATAAAATAAAAACAATTAATGTATAG

Jasper TCTCCGAAGAAAATAAATATATAAAAAGACACAAATAAAATAAAAACAATTAATGTATAG

CN 120017 TCTCCGAAGAAAATAAATATATAAAAAGACACAAATAAAATAAAAACAATTAATGTATAG

CN 120027 TCTCCGAAGAAAATAAATATATAAAAAGACACAAATAAAATAAAAACAATTAATGTATAG

CN 120030 TCTCCGAAGAAAATAAATATATAAAAAGACACAAATAAAATAAAAACAATTAATGTATAG

Hoga TCTCCGAAGAAAATAAATATATAAAAAGACACAAATAAAATAAAAACAATTAATGTATAG

Yellowstone TCTCCGAAGAAAATAAATATATAAAAAGACACAAATAAAATAAAAACAATTAATGTATAG

CN 119294 TCTCCGAAGAAAATAAATATATAAAAAGACACAAATAAAATAAAAACAATTAATGTATAG

**** *******************************************************

CN 120025 TTTGATTTTTCCGGCGAATCTCTTGTTGTTTTACTCGGTTCGGTCTTTGTTAGTGTTTTT

CN 119205 TTTGATTTTTCCGGCGAATCTCTTGTTGTTTTACTCGGTTCGGTCTTTGTTAGTGTTTTT

CN 119243 TTTGATTTTTCCGGCGAATCTCTTGTTGTTTTACTCGGTTCGGTCTTTGTTAGTGTTTTT

Joelle NCBI TTTGATTTTTCCGGCGAATCTCTTGTTGTTTTACTCGGTTCGGTCTTTGTTAGTGTTTTT

Joelle AAFC TTTGATTTTTCCGGCGAATCTCTTGTTGTTTTACTCGGTTCGGTCTTTGTTAGTGTTTTT

Joelle phyto TTTGATTTTTCCGGCGAATCTCTTGTTGTTTTACTCGGTTCGGTCTTTGTTAGTGTTTTT

DH55 ref genome TTTGATTTTTCCGGCGAATCTCTTGTTGTTTTACTCGGTTCGGTCTTTGTTAGTGTTTTT

CN 119300 TTTGATTTTTCCGGCGAATCTCTTGTTGTTTTACTCGGTTCGGTCTTTGTTAGTGTTTTT

CAM 241 TTTGATTTTTCCGGCGAATCTCTTGTTGTTTTACTCGGTTCGGTCTTTGTTAGTGTTTTT

CN 113754 TTTGATTTTTCCGGCGAATCTCTTGTTGTTTTACTCGGTTCGGTCTTTGTTAGTGTTTTT

CAM 236 TTTGATTTTTCCGGCGAATCTCTTGTTGTTTTACTCGGTTCGGTCTTTGTTAGTGTTTTT

CO46 NCBI TTTGATTTTTCCGGCGAATCTCTTGTTGTTTTACTCGGTTCGGTCTTTGTTAGTGTTTTT

CN 120013 TTTGATTTTTCCGGCGAATCTCTTGTTGTTTTACTCGGTTCGGTCTTTGTTAGTGTTTTT

Blaine Creek TTTGATTTTTCCGGCGAATCTCTTGTTGTTTTACTCGGTTCGGTCTTTGTTAGTGTTTTT

09-CS0040 TTTGATTTTTCCGGCGAATCTCTTGTTGTTTTACTCGGTTCGGTCTTTGTTAGTGTTTTT

17CS1133 TTTGATTTTTCCGGCGAATCTCTTGTTGTTTTACTCGGTTCGGTCTTTGTTAGTGTTTTT

Jasper TTTGATTTTTCCGGCGAATCTCTTGTTGTTTTACTCGGTTCGGTCTTTGTTAGTGTTTTT

CN 120017 TTTGATTTTTCCGGCGAATCTCTTGTTGTTTTACTCGGTTCGGTCTTTGTTAGTGTTTTT

CN 120027 TTTGATTTTTCCGGCGAATCTCTTGTTGTTTTACTCGGTTCGGTCTTTGTTAGTGTTTTT

CN 120030 TTTGATTTTTCCGGCGAATCTCTTGTTGTTTTACTCGGTTCGGTCTTTGTTAGTGTTTTT

Hoga TTTGATTTTTCCGGCGAATCTCTTGTTGTTTTACTCGGTTCGGTCTTTGTTAGTGTTTTT

Yellowstone TTTGATTTTTCCGGCGAATCTCTTGTTGTTTTACTCGGTTCGGTCTTTGTTAGTGTTTTT

CN 119294 TTTGATTTTTCCGGCGAATCTCTTGTTGTTTTACTCGGTTCGGTCTTTGTTAGTGTTTTT

************************************************************

CN 120025 TCTATGACCATGTGAGATACATGAGATAACCAAATCTATGGAAGAACAATGTCGTGTTGA

CN 119205 TCTATGACCATGTGAGATACATGAGATAACCAAATCTATGGAAGAACAATGTCGTGTTGA

CN 119243 TCTATGACCATGTGAGATACATGAGATAACCAAATCTATGGAAGAACAATGTCGTGTTGA

Joelle NCBI TCTATGACCATGTGAGATACATGAGATAACCAAATCTATGGAAGAACAATGTCGTGTTGA

Joelle AAFC TCTATGACCATGTGAGATACATGAGATAACCAAATCTATGGAAGAACAATGTCGTGTTGA

Joelle phyto TCTATGACCATGTGAGATACATGAGATAACCAAATCTATGGAAGAACAATGTCGTGTTGA

DH55 ref genome TCTATGACCATGTGAGATACATGAGATAACCAAATCTATGGAAGAACAATGTCGTGTTGA

CN 119300 TCTATGACCATGTGAGATACATGAGATAACCAAATCTATGGAAGAACAATGTCGTGTTGA

CAM 241 TCTATGACCATGTGAGATACATGAGATAACCAAATCTATGGAAGAACAATGTCGTGTTGA

CN 113754 TCTATGACCATGTGAGATACATGAGATAACCAAATCTATGGAAGAACAATGTCGTGTTGA

CAM 236 TCTATGACCATGTGAGATACATGAGATAACCAAATCTATGGAAGAACAATGTCGTGTTGA

CO46 NCBI TCTATGACCATGTGAGATACATGAGATAACCAAATCTATGGAAGAACAATGTCGTGTTGA

CN 120013 TCTATGACCATGTGAGATACATGAGATAACCAAATCTATGGAAGAACAATGTCGTGTTGA

Blaine Creek TCTATGACCATGTGAGATACATGAGATAACCAAATCTATGGAAGAACAATGTCGTGTTGA

09-CS0040 TCTATGACCATGTGAGATACATGAGATAACCAAATCTATGGAAGAACAATGTCGTGTTGA

17CS1133 TCTATGACCATGTGAGATACATGAGATAACCAAATCTATGGAAGAACAATGTCGTGTTGA

Jasper TCTATGACCATGTGAGATACATGAGATAACCAAATCTATGGAAGAACAATGTCGTGTTGA

CN 120017 TCTATGACCATGTGAGATACATGAGATAACCAAATCTATGGAAGAACAATGTCGTGTTGA

CN 120027 TCTATGACCATGTGAGATACATGAGATAACCAAATCTATGGAAGAACAATGTCGTGTTGA

CN 120030 TCTATGACCATGTGAGATACATGAGATAACCAAATCTATGGAAGAACAATGTCGTGTTGA

Hoga TCTATGACCATGTGAGATACATGAGATAACCAAATCTATGGAAGAACAATGTCGTGTTGA

Yellowstone TCTATGACCATGTGAGATACATGAGATAACCAAATCTATGGAAGAACAATGTCGTGTTGA

CN 119294 TCTATGACCATGTGAGATACATGAGATAACCAAATCTATGGAAGAACAATGTCGTGTTGA

************************************************************

CN 120025 GCTTAAGCTTCTTACTTTTTTTCTTCTTTTCTCTCTCTATCTCTCTCTCTCTATTTCCTT

CN 119205 GCTTAAGCTTCTTACTTTTTTTCTTCTTTTCTCTCTCTATCGCTCTCTCTCTATTTCCTT

CN 119243 GCTTAAGCTTCTTACTTTTTTTCTTCTTTTCTCTCTCTATCGCTCTCTCTCTATTTCCTT

Joelle NCBI GCTTAAGCTTCTTACTTTTTTTCTTCTTTTCTCTCTCTATCGCTCTCTCTCTATTTCCTT

Joelle AAFC GCTTAAGCTTCTTACTTTTTTTCTTCTTTTCTCTCTCTATCGCTCTCTCTCTATTTCCTT

Joelle phyto GCTTAAGCTTCTTACTTTTTTTCTTCTTTTCTCTCTCTATCGCTCTCTCTCTATTTCCTT

DH55 ref genome GCTTAAGCTTCTTACTTTTTTTCTTCTTTTCTCTCTCTATCGCTCTCTCTCTATTTCCTT

CN 119300 GCTTAAGCTTCTTACTTTTTTTCTTCTTTTCTCTCTCTATCGCTCTCTCTCTATTTCCTT

CAM 241 GCTTAAGCTTCTTACTTTTTTTCTTCTTTTCTCTCTCTATCGCTCTCTCTCTATTTCCTT

CN 113754 GCTTAAGCTTCTTACTTTTTTTCTTCTTTTCTCTCTCTATCGCTCTCTCTCTATTTCCTT

CAM 236 GCTTAAGCTTCTTACTTTTTTTCTTCTTTTCTCTCTCTATCGCTCTCTCTCTATTTCCTT

CO46 NCBI GCTTAAGCTTCTTACTTTTTTTCTTCTTTTCTCTCTCTATCGCTCTCTCTCTATTTCCTT

CN 120013 GCTTAAGCTTCTTACTTTTTTTCTTCTTTTCTCTCTCTATCGCTCTCTCTCTATTTCCTT

Blaine Creek GCTTAAGCTTCTTACTTTTTTTCTTCTTTTCTCTCTCTATCGCTCTCTCTCTATTTCCTT

09-CS0040 GCTTAAGCTTCTTACTTTTTTTCTTCTTTTCTCTCTCTATCGCTCTCTCTCTATTTCCTT

17CS1133 GCTTAAGCTTCTTACTTTTTTTCTTCTTTTCTCTCTCTATCGCTCTCTCTCTATTTCCTT

Jasper GCTTAAGCTTCTTACTTTTTTTCTTCTTTTCTCTCTCTATCGCTCTCTCTCTATTTCCTT

CN 120017 GCTTAAGCTTCTTACTTTTTTTCTTCTTTTCTCTCTCTATCGCTCTCTCTCTATTTCCTT

CN 120027 GCTTAAGCTTCTTACTTTTTTTCTTCTTTTCTCTCTCTATCGCTCTCTCTCTATTTCCTT

CN 120030 GCTTAAGCTTCTTACTTTTTTTCTTCTTTTCTCTCTCTATCGCTCTCTCTCTATTTCCTT

Hoga GCTTAAGCTTCTTACTTTTTTTCTTCTTTTCTCTCTCTATCGCTCTCTCTCTATTTCCTT

Yellowstone GCTTAAGCTTCTTACTTTTTTTCTTCTTTTCTCTCTCTATCGCTCTCTCTCTATTTCCTT

CN 119294 GCTTAAGCTTCTTACTTTTTTTCTTCTTTTCTCTCTCTATCGCTCTCTCTCTATTTCCTT

***************************************** ******************

CN 120025 AAAAAAATTTCTGCATGGATTTTTATTTTATTTGGAAATTTTTTGCATGTCCTTCGAGAT

CN 119205 AAAAAAATTTCTGCATGGATTTTTATTTTATTTGGAAATTTTTTGCATGTCCTTCGAGAT

CN 119243 AAAAAAATTTCTGCATGGATTTTTATTTTATTTGGAAATTTTTTGCATGTCCTTCGAGAT

Joelle NCBI AAAAAAATTTCTGCATGGATTTTTATTTTATTTGGAAATTTTTTGCATGTCCTTCGAGAT

Joelle AAFC AAAAAAATTTCTGCATGGATTTTTATTTTATTTGGAAATTTTTTGCATGTCCTTCGAGAT

Joelle phyto AAAAAAATTTCTGCATGGATTTTTATTTTATTTGGAAATTTTTTGCATGTCCTTCGAGAT

DH55 ref genome AAAAAAATTTCTGCATGGATTTTTATTTTATTTGGAAATTTTTTGCATGTCCTTCGAGAT

CN 119300 AAAAAAATTTCTGCATGGATTTTTATTTTATTTGGAAATTTTTTGCATGTCCTTCGAGAT

CAM 241 AAAAAAATTTCTGCATGGATTTTTATTTTATTTGGAAATTTTTTGCATGTCCTTCGAGAT

CN 113754 AAAAAAATTTCTGCATGGATTTTTATTTTATTTGGAAATTTTTTGCATGTCCTTCGAGAT

CAM 236 AAAAAAATTTCTGCATGGATTTTTATTTTATTTGGAAATTTTTTGCATGTCCTTCGAGAT

CO46 NCBI AAAAAAATTTCTGCATGGATTTTTATTTTATTTGGAAATTTTTTGCATGTCCTTCGAGAT

CN 120013 AAAAAAATTTCTGCATGGATTTTTATTTTATTTGGAAATTTTTTGCATGTCCTTCGAGAT

Blaine Creek AAAAAAATTTCTGCATGGATTTTTATTTTATTTGGAAATTTTTTGCATGTCCTTCGAGAT

09-CS0040 AAAAAAATTTCTGCATGGATTTTTATTTTATTTGGAAATTTTTTGCATGTCCTTCGAGAT

17CS1133 AAAAAAATTTCTGCATGGATTTTTATTTTATTTGGAAATTTTTTGCATGTCCTTCGAGAT

Jasper AAAAAAATTTCTGCATGGATTTTTATTTTATTTGGAAATTTTTTGCATGTCCTTCGAGAT

CN 120017 AAAAAAATTTCTGCATGGATTTTTATTTTATTTGGAAATTTTTTGCATGTCCTTCGAGAT

CN 120027 AAAAAAATTTCTGCATGGATTTTTATTTTATTTGGAAATTTTTTGCATGTCCTTCGAGAT

CN 120030 AAAAAAATTTCTGCATGGATTTTTATTTTATTTGGAAATTTTTTGCATGTCCTTCGAGAT

Hoga AAAAAAATTTCTGCATGGATTTTTATTTTATTTGGAAATTTTTTGCATGTCCTTCGAGAT

Yellowstone AAAAAAATTTCTGCATGGATTTTTATTTTATTTGGAAATTTTTTGCATGTCCTTCGAGAT

CN 119294 AAAAAAATTTCTGCATGGATTTTTATTTTATTTGGAAATTTTTTGCATGTCCTTCGAGAT

************************************************************

CN 120025 TTGCTTGACACGTTCTGCTGCGTACTCGATGTTGTCCAGTGAAGTTTCAAATCCGTCTTT

CN 119205 TTGCTTGACACGTTCTGCTGCGTACTCGATGTTGACCAGTGAAGTTTCAAAGCCGTCTTT

CN 119243 TTGCTTGACACGTTCTGCTGCGTACTCGATGTTGACCAGTGAAGTTTCAAAGCCGTCTTT

Joelle NCBI TTGCTTGACACGTTCTGCTGCGTACTCGATGTTGACCAGTGAAGTTTCAAAGCCGTCTTT

Joelle AAFC TTGCTTGACACGTTCTGCTGCGTACTCGATGTTGACCAGTGAAGTTTCAAAGCCGTCTTT

Joelle phyto TTGCTTGACACGTTCTGCTGCGTACTCGATGTTGACCAGTGAAGTTTCAAAGCCGTCTTT

DH55 ref genome TTGCTTGACACGTTCTGCTGCGTACTCGATGTTGACCAGTGAAGTTTCAAAGCCGTCTTT

CN 119300 TTGCTTGACACGTTCTGCTGCGTACTCGATGTTGACCAGTGAAGTTTCAAAGCCGTCTTT

CAM 241 TTGCTTGACACGTTCTGCTGCGTACTCGATGTTGACCAGTGAAGTTTCAAAGCCGTCTTT

CN 113754 TTGCTTGACACGTTCTGCTGCGTACTCGATGTTGACCAGTGAAGTTTCAAAGCCGTCTTT

CAM 236 TTGCTTGACACGTTCTGCTGCGTACTCGATGTTGACCAGTGAAGTTTCAAAGCCGTCTTT

CO46 NCBI TTGCTTGACACGTTCTGCTGCGTACTCGATGTTGACCAGTGAAGTTTCAAAGCCGTCTTT

CN 120013 TTGCTTGACACGTTCTGCTGCGTACTCGATGTTGACCAGTGAAGTTTCAAAGCCGTCTTT

Blaine Creek TTGCTTGACACGTTCTGCTGCGTACTCGATGTTGACCAGTGAAGTTTCAAAGCCGTCTTT

09-CS0040 TTGCTTGACACGTTCTGCTGCGTACTCGATGTTGACCAGTGAAGTTTCAAAGCCGTCTTT

17CS1133 TTGCTTGACACGTTCTGCTGCGTACTCGATGTTGACCAGTGAAGTTTCAAAGCCGTCTTT

Jasper TTGCTTGACACGTTCTGCTGCGTACTCGATGTTGACCAGTGAAGTTTCAAAGCCGTCTTT

CN 120017 TTGCTTGACACGTTCTGCTGCGTACTCGATGTTGACCAGTGAAGTTTCAAAGCCGTCTTT

CN 120027 TTGCTTGACACGTTCTGCTGCGTACTCGATGTTGACCAGTGAAGTTTCAAAGCCGTCTTT

CN 120030 TTGCTTGACACGTTCTGCTGCGTACTCGATGTTGACCAGTGAAGTTTCAAAGCCGTCTTT

Hoga TTGCTTGACACGTTCTGCTGCGTACTCGATGTTGACCAGTGAAGTTTCAAAGCCGTCTTT

Yellowstone TTGCTTGACACGTTCTGCTGCGTACTCGATGTTGACCAGTGAAGTTTCAAAGCCGTCTTT

CN 119294 TTGCTTGACACGTTCTGCTGCGTACTCGATGTTGACCAGTGAAGTTTCAAAGCCGTCTTT

********************************** **************** ********

CN 120025 GATTGCTACATAGCTTTAGGGATTAATTCCCTATGTTTCTGATTAGTTTTTATATTAGAA

CN 119205 GATTGCTACATAGCTTTAGGGATTAATTCCCTATGTTTCTGATTAGTTTTTATATTAGAA

CN 119243 GATTGCTACATAGCTTTAGGGATTAATTCCCTATGTTTCTGATTAGTTTTTATATTAGAA

Joelle NCBI GATTGCTACATAGCTTTAGGGATTAATTCCCTATGTTTCTGATTAGTTTTTATATTAGAA

Joelle AAFC GATTGCTACATAGCTTTAGGGATTAATTCCCTATGTTTCTGATTAGTTTTTATATTAGAA

Joelle phyto GATTGCTACATAGCTTTAGGGATTAATTCCCTATGTTTCTGATTAGTTTTTATATTAGAA

DH55 ref genome GATTGCTACATAGCTTTAGGGATTAATTCCCTATGTTTCTGATTAGTTTTTATATTAGAA

CN 119300 GATTGCTACATAGCTTTAGGGATTAATTCCCTATGTTTCTGATTAGTTTTTATATTAGAA

CAM 241 GATTGCTACATAGCTTTAGGGATTAATTCCCTATGTTTCTGATTAGTTTTTATATTAGAA

CN 113754 GATTGCTACATAGCTTTAGGGATTAATTCCCTATGTTTCTGATTAGTTTTTATATTAGAA

CAM 236 GATTGCTACATAGCTTTAGGGATTAATTCCCTATGTTTCTGATTAGTTTTTATATTAGAA

CO46 NCBI GATTGCTACATAGCTTTAGGGATTAATTCCCTATGTTTCTGATTAGTTTTTATATTAGAA

CN 120013 GATTGCTACATAGCTTTAGGGATTAATTCCCTATGTTTCTGATTAGTTTTTATATTAGAA

Blaine Creek GATTGCTACATAGCTTTAGGGATTAATTCCCTATGTTTCTGATTAGTTTTTATATTAGAA

09-CS0040 GATTGCTACATAGCTTTAGGGATTAATTCCCTATGTTTCTGATTAGTTTTTATATTAGAA

17CS1133 GATTGCTACATAGCTTTAGGGATTAATTCCCTATGTTTCTGATTAGTTTTTATATTAGAA

Jasper GATTGCTACATAGCTTTAGGGATTAATTCCCTATGTTTCTGATTAGTTTTTATATTAGAA

CN 120017 GATTGCTACATAGCTTTAGGGATTAATTCCCTATGTTTCTGATTAGTTTTTATATTAGAA

CN 120027 GATTGCTACATAGCTTTAGGGATTAATTCCCTATGTTTCTGATTAGTTTTTATATTAGAA

CN 120030 GATTGCTACATAGCTTTAGGGATTAATTCCCTATGTTTCTGATTAGTTTTTATATTAGAA

Hoga GATTGCTACATAGCTTTAGGGATTAATTCCCTATGTTTCTGATTAGTTTTTATATTAGAA

Yellowstone GATTGCTACATAGCTTTAGGGATTAATTCCCTATGTTTCTGATTAGTTTTTATATTAGAA

CN 119294 GATTGCTACATAGCTTTAGGGATTAATTCCCTATGTTTCTGATTAGTTTTTATATTAGAA

************************************************************

CN 120025 TTGCTAAATCATTCACTGGATCTCTCTTTTTTAAAAGTAATTTAAAACTCATTCGATCTC

CN 119205 TTGCTAAATCATTCACTGGATCTCTCTTTTTTAAAAGTAATTTAAAACTCATTCGATCTC

CN 119243 TTGCTAAATCATTCACTGGATCTCTCTTTTTTAAAAGTAATTTAAAACTCATTCGATCTC

Joelle NCBI TTGCTAAATCATTCACTGGATCTCTCTTTTTTAAAAGTAATTTAAAACTCATTCGATCTC

Joelle AAFC TTGCTAAATCATTCACTGGATCTCTCTTTTTTAAAAGTAATTTAAAACTCATTCGATCTC

Joelle phyto TTGCTAAATCATTCACTGGATCTCTCTTTTTTAAAAGTAATTTAAAACTCATTCGATCTC

DH55 ref genome TTGCTAAATCATTCACTGGATCTCTCTTTTTTAAAAGTAATTTAAAACTCATTCGATCTC

CN 119300 TTGCTAAATCATTCACTGGATCTCTCTTTTTTAAAAGTAATTTAAAACTCATTCGATCTC

CAM 241 TTGCTAAATCATTCACTGGATCTCTCTTTTTTAAAAGTAATTTAAAACTCATTCGATCTC

CN 113754 TTGCTAAATCATTCACTGGATCTCTCTTTTTTAAAAGTAATTTAAAACTCATTCGATCTC

CAM 236 TTGCTAAATCATTCACTGGATCTCTCTTTTTTAAAAGTAATTTAAAACTCATTCGATCTC

CO46 NCBI TTGCTAAATCATTCACTGGATCTCTCTTTTTTAAAAGTAATTTAAAACTCATTCGATCTC

CN 120013 TTGCTAAATCATTCACTGGATCTCTCTTTTTTAAAAGTAATTTAAAACTCATTCGATCTC

Blaine Creek TTGCTAAATCATTCACTGGATCTCTCTTTTTTAAAAGTAATTTAAAACTCATTCGATCTC

09-CS0040 TTGCTAAATCATTCACTGGATCTCTCTTTTTTAAAAGTAATTTAAAACTCATTCGATCTC

17CS1133 TTGCTAAATCATTCACTGGATCTCTCTTTTTTAAAAGTAATTTAAAACTCATTCGATCTC

Jasper TTGCTAAATCATTCACTGGATCTCTCTTTTTTAAAAGTAATTTAAAACTCATTCGATCTC

CN 120017 TTGCTAAATCATTCACTGGATCTCTCTTTTTTAAAAGTAATTTAAAACTCATTCGATCTC

CN 120027 TTGCTAAATCATTCACTGGATCTCTCTTTTTTAAAAGTAATTTAAAACTCATTCGATCTC

CN 120030 TTGCTAAATCATTCACTGGATCTCTCTTTTTTAAAAGTAATTTAAAACTCATTCGATCTC

Hoga TTGCTAAATCATTCACTGGATCTCTCTTTTTTAAAAGTAATTTAAAACTCATTCGATCTC

Yellowstone TTGCTAAATCATTCACTGGATCTCTCTTTTTTAAAAGTAATTTAAAACTCATTCGATCTC

CN 119294 TTGCTAAATCATTCACTGGATCTCTCTTTTTTAAAAGTAATTTAAAACTCATTCGATCTC

************************************************************

CN 120025 TTTGGATTTGTATCCAGTGCAATGTACCTTCGGGAGATCTATGCAAATCCGAGAGATCCA

CN 119205 TTTGGATTTGTATCCAGTGCAATGTACCTTCGGGAGATCTATGCAAATCCGAGAGATCCA

CN 119243 TTTGGATTTGTATCCAGTGCAATGTACCTTCGGGAGATCTATGCAAATCCGAGAGATCCA

Joelle NCBI TTTGGATTTGTATCCAGTGCAATGTACCTTCGGGAGATCTATGCAAATCCGAGAGATCCA

Joelle AAFC TTTGGATTTGTATCCAGTGCAATGTACCTTCGGGAGATCTATGCAAATCCGAGAGATCCA

Joelle phyto TTTGGATTTGTATCCAGTGCAATGTACCTTCGGGAGATCTATGCAAATCCGAGAGATCCA

DH55 ref genome TTTGGATTTGTATCCAGTGCAATGTACCTTCGGGAGATCTATGCAAATCCGAGAGATCCA

CN 119300 TTTGGATTTGTATCCAGTGCAATGTACCTTCGGGAGATCTATGCAAATCCGAGAGATCCA

CAM 241 TTTGGATTTGTATCCAGTGCAATGTACCTTCGGGAGATCTATGCAAATCCGAGAGATCCA

CN 113754 TTTGGATTTGTATCCAGTGCAATGTACCTTCGGGAGATCTATGCAAATCCGAGAGATCCA

CAM 236 TTTGGATTTGTATCCAGTGCAATGTACCTTCGGGAGATCTATGCAAATCCGAGAGATCCA

CO46 NCBI TTTGGATTTGTATCCAGTGCAATGTACCTTCGGGAGATCTATGCAAATCCGAGAGATCCA

CN 120013 TTTGGATTTGTATCCAGTGCAATGTACCTTCGGGAGATCTATGCAAATCCGAGAGATCCA

Blaine Creek TTTGGATTTGTATCCAGTGCAATGTACCTTCGGGAGATCTATGCAAATCCGAGAGATCCA

09-CS0040 TTTGGATTTGTATCCAGTGCAATGTACCTTCGGGAGATCTATGCAAATCCGAGAGATCCA

17CS1133 TTTGGATTTGTATCCAGTGCAATGTACCTTCGGGAGATCTATGCAAATCCGAGAGATCCA

Jasper TTTGGATTTGTATCCAGTGCAATGTACCTTCGGGAGATCTATGCAAATCCGAGAGATCCA

CN 120017 TTTGGATTTGTATCCAGTGCAATGTACCTTCGGGAGATCTATGCAAATCCGAGAGATCCA

CN 120027 TTTGGATTTGTATCCAGTGCAATGTACCTTCGGGAGATCTATGCAAATCCGAGAGATCCA

CN 120030 TTTGGATTTGTATCCAGTGCAATGTACCTTCGGGAGATCTATGCAAATCCGAGAGATCCA

Hoga TTTGGATTTGTATCCAGTGCAATGTACCTTCGGGAGATCTATGCAAATCCGAGAGATCCA

Yellowstone TTTGGATTTGTATCCAGTGCAATGTACCTTCGGGAGATCTATGCAAATCCGAGAGATCCA

CN 119294 TTTGGATTTGTATCCAGTGCAATGTACCTTCGGGAGATCTATGCAAATCCGAGAGATCCA

************************************************************

CN 120025 TAGATTTTCAATGGTGTTAATGCTGAATAATGTATACCACATTGTCATGGACCTATATTA

CN 119205 TAGATTTTCAATGGTGTTAATGCTGAATAATGTATACCACATTGTCATGGACCTATATTA

CN 119243 TAGATTTTCAATGGTGTTAATGCTGAATAATGTATACCACATTGTCATGGACCTATATTA

Joelle NCBI TAGATTTTCAATGGTGTTAATGCTGAATAATGTATACCACATTGTCATGGACCTATATTA

Joelle AAFC TAGATTTTCAATGGTGTTAATGCTGAATAATGTATACCACATTGTCATGGACCTATATTA

Joelle phyto TAGATTTTCAATGGTGTTAATGCTGAATAATGTATACCACATTGTCATGGACCTATATTA

DH55 ref genome TAGATTTTCAATGGTGTTAATGCTGAATAATGTATACCACATTGTCATGGACCTATATTA

CN 119300 TAGATTTTCAATGGTGTTAATGCTGAATAATGTATACCACATTGTCATGGACCTATATTA

CAM 241 TAGATTTTCAATGGTGTTAATGCTGAATAATGTATACCACATTGTCATGGACCTATATTA

CN 113754 TAGATTTTCAATGGTGTTAATGCTGAATAATGTATACCACATTGTCATGGACCTATATTA

CAM 236 TAGATTTTCAATGGTGTTAATGCTGAATAATGTATACCACATTGTCATGGACCTATATTA

CO46 NCBI TAGATTTTCAATGGTGTTAATGCTGAATAATGTATACCACATTGTCATGGACCTATATTA

CN 120013 TAGATTTTCAATGGTGTTAATGCTGAATAATGTATACCACATTGTCATGGACCTATATTA

Blaine Creek TAGATTTTCAATGGTGTTAATGCTGAATAATGTATACCACATTGTCATGGACCTATATTA

09-CS0040 TAGATTTTCAATGGTGTTAATGCTGAATAATGTATACCACATTGTCATGGACCTATATTA

17CS1133 TAGATTTTCAATGGTGTTAATGCTGAATAATGTATACCACATTGTCATGGACCTATATTA

Jasper TAGATTTTCAATGGTGTTAATGCTGAATAATGTATACCACATTGTCATGGACCTATATTA

CN 120017 TAGATTTTCAATGGTGTTAATGCTGAATAATGTATACCACATTGTCATGGACCTATATTA

CN 120027 TAGATTTTCAATGGTGTTAATGCTGAATAATGTATACCACATTGTCATGGACCTATATTA

CN 120030 TAGATTTTCAATGGTGTTAATGCTGAATAATGTATACCACATTGTCATGGACCTATATTA

Hoga TAGATTTTCAATGGTGTTAATGCTGAATAATGTATACCACATTGTCATGGACCTATATTA

Yellowstone TAGATTTTCAATGGTGTTAATGCTGAATAATGTATACCACATTGTCATGGACCTATATTA

CN 119294 TAGATTTTCAATGGTGTTAATGCTGAATAATGTATACCACATTGTCATGGACCTATATTA

************************************************************

CN 120025 CTTGATGATTATCCAAATTAGTGTTTCTAATTGATTATTAACTTTTTTTATATATATAGT

CN 119205 CTTGATGATTATCCAAATTAGTGTTTCTAATTGATTATTAACTTTTTTTATATATATAGT

CN 119243 CTTGATGATTATCCAAATTAGTGTTTCTAATTGATTATTAACTTTTTTTATATATATAGT

Joelle NCBI CTTGATGATTATCCAAATTAGTGTTTCTAATTGATTATTAACTTTTTTTATATATATAGT

Joelle AAFC CTTGATGATTATCCAAATTAGTGTTTCTAATTGATTATTAACTTTTTTTATATATATAGT

Joelle phyto CTTGATGATTATCCAAATTAGTGTTTCTAATTGATTATTAACTTTTTTTATATATATAGT

DH55 ref genome CTTGATGATTATCCAAATTAGTGTTTCTAATTGATTATTAACTTTTTTTATATATATAGT

CN 119300 CTTGATGATTATCCAAATTAGTGTTTCTAATTGATTATTAACTTTTTTTATATATATAGT

CAM 241 CTTGATGATTATCCAAATTAGTGTTTCTAATTGATTATTAACTTTTTTTATATATATAGT

CN 113754 CTTGATGATTATCCAAATTAGTGTTTCTAATTGATTATTAACTTTTTTTATATATATAGT

CAM 236 CTTGATGATTATCCAAATTAGTGTTTCTAATTGATTATTAACTTTTTTTATATATATAGT

CO46 NCBI CTTGATGATTATCCAAATTAGTGTTTCTAATTGATTATTAACTTTTTTTATATATATAGT

CN 120013 CTTGATGATTATCCAAATTAGTGTTTCTAATTGATTATTAACTTTTTTTATATATATAGT

Blaine Creek CTTGATGATTATCCAAATTAGTGTTTCTAATTGATTATTAACTTTTTTTATATATATAGT

09-CS0040 CTTGATGATTATCCAAATTAGTGTTTCTAATTGATTATTAACTTTTTTTATATATATAGT

17CS1133 CTTGATGATTATCCAAATTAGTGTTTCTAATTGATTATTAACTTTTTTTATATATATAGT

Jasper CTTGATGATTATCCAAATTAGTGTTTCTAATTGATTATTAACTTTTTTTATATATATAGT

CN 120017 CTTGATGATTATCCAAATTAGTGTTTCTAATTGATTATTAACTTTTTTTATATATATAGT

CN 120027 CTTGATGATTATCCAAATTAGTGTTTCTAATTGATTATTAACTTTTTTTATATATATAGT

CN 120030 CTTGATGATTATCCAAATTAGTGTTTCTAATTGATTATTAACTTTTTTTATATATATAGT

Hoga CTTGATGATTATCCAAATTAGTGTTTCTAATTGATTATTAACTTTTTTTATATATATAGT

Yellowstone CTTGATGATTATCCAAATTAGTGTTTCTAATTGATTATTAACTTTTTTTATATATATAGT

CN 119294 CTTGATGATTATCCAAATTAGTGTTTCTAATTGATTATTAACTTTTTTTATATATATAGT

************************************************************

CN 120025 ACTTGTGAATATTGGTAGCTTTTAAAAAACTCAGCCTCACAATTCGTCCTTACCGCACAT

CN 119205 ACTTGTGAATATTGGTAGCTTTAAAAAAACTCAGCCTCACAATTAGTCCTTACCGCACAT

CN 119243 ACTTGTGAATATTGGTAGCTTTAAAAAAACTCAGCCTCACAATTAGTCCTTACCGCACAT

Joelle NCBI ACTTGTGAATATTGGTAGCTTTAAAAAAACTCAGCCTCACAATTAGTCCTTACCGCACAT

Joelle AAFC ACTTGTGAATATTGGTAGCTTTAAAAAAACTCAGCCTCACAATTAGTCCTTACCGCACAT

Joelle phyto ACTTGTGAATATTGGTAGCTTTAAAAAAACTCAGCCTCACAATTAGTCCTTACCGCACAT

DH55 ref genome ACTTGTGAATATTGGTAGCTTTAAAAAAACTCAGCCTCACAATTAGTCCTTACCGCACAT

CN 119300 ACTTGTGAATATTGGTAGCTTTAAAAAAACTCAGCCTCACAATTAGTCCTTACCGCACAT

CAM 241 ACTTGTGAATATTGGTAGCTTTAAAAAAACTCAGCCTCACAATTAGTCCTTACCGCACAT

CN 113754 ACTTGTGAATATTGGTAGCTTTAAAAAAACTCAGCCTCACAATTAGTCCTTACCGCACAT

CAM 236 ACTTGTGAATATTGGTAGCTTTAAAAAAACTCAGCCTCACAATTAGTCCTTACCGCACAT

CO46 NCBI ACTTGTGAATATTGGTAGCTTTAAAAAAACTCAGCCTCACAATTAGTCCTTACCGCACAT

CN 120013 ACTTGTGAATATTGGTAGCTTTAAAAAAACTCAGCCTCACAATTAGTCCTTACCGCACAT

Blaine Creek ACTTGTGAATATTGGTAGCTTTAAAAAAACTCAGCCTCACAATTAGTCCTTACCGCACAT

09-CS0040 ACTTGTGAATATTGGTAGCTTTAAAAAAACTCAGCCTCACAATTAGTCCTTACCGCACAT

17CS1133 ACTTGTGAATATTGGTAGCTTTAAAAAAACTCAGCCTCACAATTAGTCCTTACCGCACAT

Jasper ACTTGTGAATATTGGTAGCTTTAAAAAAACTCAGCCTCACAATTAGTCCTTACCGCACAT

CN 120017 ACTTGTGAATATTGGTAGCTTTAAAAAAACTCAGCCTCACAATTAGTCCTTACCGCACAT

CN 120027 ACTTGTGAATATTGGTAGCTTTAAAAAAACTCAGCCTCACAATTAGTCCTTACCGCACAT

CN 120030 ACTTGTGAATATTGGTAGCTTTAAAAAAACTCAGCCTCACAATTAGTCCTTACCGCACAT

Hoga ACTTGTGAATATTGGTAGCTTTAAAAAAACTCAGCCTCACAATTAGTCCTTACCGCACAT

Yellowstone ACTTGTGAATATTGGTAGCTTT-AAAAAACTCAGCCTCACAATTAGTCCTTACCGCACAT

CN 119294 ACTTGTGAATATTGGTAGCTTTAAAAAAACTCAGCCTCACAATTAGTCCTTACCGCACAT

********************** ********************* ***************

CN 120025 ATGCTACTTAAGCTATGTGTTCTGGTTTGGATTGCGATTAATTGCAATTGTTGTATGCAT

CN 119205 ATGCTACTTAAGCTATGTGTTCTGGTTTGGATTGCGATTAATTGCAATTGTTGTATGCAT

CN 119243 ATGCTACTTAAGCTATGTGTTCTGGTTTGGATTGCGATTAATTGCAATTGTTGTATGCAT

Joelle NCBI ATGCTACTTAAGCTATGTGTTCTGGTTTGGATTGCGATTAATTGCAATTGTTGTATGCAT

Joelle AAFC ATGCTACTTAAGCTATGTGTTCTGGTTTGGATTGCGATTAATTGCAATTGTTGTATGCAT

Joelle phyto ATGCTACTTAAGCTATGTGTTCTGGTTTGGATTGCGATTAATTGCAATTGTTGTATGCAT

DH55 ref genome ATGCTACTTAAGCTATGTGTTCTGGTTTGGATTGCGATTAATTGCAATTGTTGTATGCAT

CN 119300 ATGCTACTTAAGCTATGTGTTCTGGTTTGGATTGCGATTAATTGCAATTGTTGTATGCAT

CAM 241 ATGCTACTTAAGCTATGTGTTCTGGTTTGGATTGCGATTAATTGCAATTGTTGTATGCAT

CN 113754 ATGCTACTTAAGCTATGTGTTCTGGTTTGGATTGCGATTAATTGCAATTGTTGTATGCAT

CAM 236 ATGCTACTTAAGCTATGTGTTCTGGTTTGGATTGCGATTAATTGCAATTGTTGTATGCAT

CO46 NCBI ATGCTACTTAAGCTATGTGTTCTGGTTTGGATTGCGATTAATTGCAATTGTTGTATGCAT

CN 120013 ATGCTACTTAAGCTATGTGTTCTGGTTTGGATTGCGATTAATTGCAATTGTTGTATGCAT

Blaine Creek ATGCTACTTAAGCTATGTGTTCTGGTTTGGATTGCGATTAATTGCAATTGTTGTATGCAT

09-CS0040 ATGCTACTTAAGCTATGTGTTCTGGTTTGGATTGCGATTAATTGCAATTGTTGTATGCAT

17CS1133 ATGCTACTTAAGCTATGTGTTCTGGTTTGGATTGCGATTAATTGCAATTGTTGTATGCAT

Jasper ATGCTACTTAAGCTATGTGTTCTGGTTTGGATTGCGATTAATTGCAATTGTTGTATGCAT

CN 120017 ATGCTACTTAAGCTATGTGTTCTGGTTTGGATTGCGATTAATTGCAATTGTTGTATGCAT

CN 120027 ATGCTACTTAAGCTATGTGTTCTGGTTTGGATTGCGATTAATTGCAATTGTTGTATGCAT

CN 120030 ATGCTACTTAAGCTATGTGTTCTGGTTTGGATTGCGATTAATTGCAATTGTTGTATGCAT

Hoga ATGCTACTTAAGCTATGTGTTCTGGTTTGGATTGCGATTAATTGCAATTGTTGTATGCAT

Yellowstone ATGCTACTTAAGCTATGTGTTCTGGTTTGGATTGCGATTAATTGCAATTGTTGTATGCAT

CN 119294 ATGCTACTTAAGCTATGTGTTCTGGTTTGGATTGCGATTAATTGCAATTGTTGTATGCAT

************************************************************

CN 120025 CTTTAACACTTTGTGCCGCACATAATGTACATTAACTGGACTATTTTTAGACTAAAATTC

CN 119205 CTTTAACACTTTGTGCCGCACATAATGTACATTAACTGGACTA-TTTTAGACTAAAATTC

CN 119243 CTTTAACACTTTGTGCCGCACATAATGTACATTAACTGGACTA-TTTTAGACTAAAATTC

Joelle NCBI CTTTAACACTTTGTGCCGCACATAATGTACATTAACTGGACTA-TTTTAGACTAAAATTC

Joelle AAFC CTTTAACACTTTGTGCCGCACATAATGTACATTAACTGGACTA-TTTTAGACTAAAATTC

Joelle phyto CTTTAACACTTTGTGCCGCACATAATGTACATTAACTGGACTA-TTTTAGACTAAAATTC

DH55 ref genome CTTTAACACTTTGTGCCGCACATAATGTACATTAACTGGACTA-TTTTAGACTAAAATTC

CN 119300 CTTTAACACTTTGTGCCGCACATAATGTACATTAACTGGACTA-TTTTAGACTAAAATTC

CAM 241 CTTTAACACTTTGTGCCGCACATAATGTACATTAACTGGACTA-TTTTAGACTAAAATTC

CN 113754 CTTTAACACTTTGTGCCGCACATAATGTACATTAACTGGACTA-TTTTAGACTAAAATTC

CAM 236 CTTTAACACTTTGTGCCGCACATAATGTACATTAACTGGACTA-TTTTAGACTAAAATTC

CO46 NCBI CTTTAACACTTTGTGCCGCACATAATGTACATTAACTGGACTA-TTTTAGACTAAAATTC

CN 120013 CTTTAACACTTTGTGCCGCACATAATGTACATTAACTGGACTA-TTTTAGACTAAAATTC

Blaine Creek CTTTAACACTTTGTGCCGCACATAATGTACATTAACTGGACTA-TTTTAGACTAAAATTC

09-CS0040 CTTTAACACTTTGTGCCGCACATAATGTACATTAACTGGACTA-TTTTAGACTAAAATTC

17CS1133 CTTTAACACTTTGTGCCGCACATAATGTACATTAACTGGACTA-TTTTAGACTAAAATTC

Jasper CTTTAACACTTTGTGCCGCACATAATGTACATTAACTGGACTA-TTTTAGACTAAAATTC

CN 120017 CTTTAACACTTTGTGCCGCACATAATGTACATTAACTGGACTA-TTTTAGACTAAAATTC

CN 120027 CTTTAACACTTTGTGCCGCACATAATGTACATTAACTGGACTA-TTTTAGACTAAAATTC

CN 120030 CTTTAACACTTTGTGCCGCACATAATGTACATTAACTGGACTA-TTTTAGACTAAAATTC

Hoga CTTTAACACTTTGTGCCGCACATAATGTACATTAACTGGACTA-TTTTAGACTAAAATTC

Yellowstone CTTTAACACTTTGTGCCGCACATAATGTACATTAACTGGACTA-TTTTAGACTAAAATTC

CN 119294 CTTTAACACTTTGTGCCGCACATAATGTACATTAACTGGACTA-TTTTAGACTAAAATTC

******************************************* ****************

CN 120025 ATTGCTCTCTTGGATTTGCATACAAATACACTCCGGGGAGATTTGTAAATATAATTAGTA

CN 119205 ATTGCTCTCTTGGATTTGCATACAAATACACTCCGGGGAGATTTGTAAATATAATTAGTA

CN 119243 ATTGCTCTCTTGGATTTGCATACAAATACACTCCGGGGAGATTTGTAAATATAATTAGTA

Joelle NCBI ATTGCTCTCTTGGATTTGCATACAAATACACTCCGGGGAGATTTGTAAATATAATTAGTA

Joelle AAFC ATTGCTCTCTTGGATTTGCATACAAATACACTCCGGGGAGATTTGTAAATATAATTAGTA

Joelle phyto ATTGCTCTCTTGGATTTGCATACAAATACACTCCGGGGAGATTTGTAAATATAATTAGTA

DH55 ref genome ATTGCTCTCTTGGATTTGCATACAAATACACTCCGGGGAGATTTGTAAATATAATTAGTA

CN 119300 ATTGCTCTCTTGGATTTGCATACAAATACACTCCGGGGAGATTTGTAAATATAATTAGTA

CAM 241 ATTGCTCTCTTGGATTTGCATACAAATACACTCCGGGGAGATTTGTAAATATAATTAGTA

CN 113754 ATTGCTCTCTTGGATTTGCATACAAATACACTCCGGGGAGATTTGTAAATATAATTAGTA

CAM 236 ATTGCTCTCTTGGATTTGCATACAAATACACTCCGGGGAGATTTGTAAATATAATTAGTA

CO46 NCBI ATTGCTCTCTTGGATTTGCATACAAATACACTCCGGGGAGATTTGTAAATATAATTAGTA

CN 120013 ATTGCTCTCTTGGATTTGCATACAAATACACTCCGGGGAGATTTGTAAATATAATTAGTA

Blaine Creek ATTGCTCTCTTGGATTTGCATACAAATACACTCCGGGGAGATTTGTAAATATAATTAGTA

09-CS0040 ATTGCTCTCTTGGATTTGCATACAAATACACTCCGGGGAGATTTGTAAATATAATTAGTA

17CS1133 ATTGCTCTCTTGGATTTGCATACAAATACACTCCGGGGAGATTTGTAAATATAATTAGTA

Jasper ATTGCTCTCTTGGATTTGCATACAAATACACTCCGGGGAGATTTGTAAATATAATTAGTA

CN 120017 ATTGCTCTCTTGGATTTGCATACAAATACACTCCGGGGAGATTTGTAAATATAATTAGTA

CN 120027 ATTGCTCTCTTGGATTTGCATACAAATACACTCCGGGGAGATTTGTAAATATAATTAGTA

CN 120030 ATTGCTCTCTTGGATTTGCATACAAATACACTCCGGGGAGATTTGTAAATATAATTAGTA

Hoga ATTGCTCTCTTGGATTTGCATACAAATACACTCCGGGGAGATTTGTAAATATAATTAGTA

Yellowstone ATTGCTCTCTTGGATTTGCATACAAATACACTCCGGGGAGATTTGTAAATATAATTAGTA

CN 119294 ATTGCTCTCTTGGATTTGCATACAAATACACTCCGGGGAGATTTGTAAATATAATTAGTA

************************************************************

CN 120025 CCATAGATCAATAATACTTTTGGTTCAAATGTATGCCACATTACTTGTAAAACTATTGAC

CN 119205 CCATAGATCAATAATACTTTTGGTTCAAATGTATGCCACATTACTTGTAAAACTATTGAC

CN 119243 CCATAGATCAATAATACTTTTGGTTCAAATGTATGCCACATTACTTGTAAAACTATTGAC

Joelle NCBI CCATAGATCAATAATACTTTTGGTTCAAATGTATGCCACATTACTTGTAAAACTATTGAC

Joelle AAFC CCATAGATCAATAATACTTTTGGTTCAAATGTATGCCACATTACTTGTAAAACTATTGAC

Joelle phyto CCATAGATCAATAATACTTTTGGTTCAAATGTATGCCACATTACTTGTAAAACTATTGAC

DH55 ref genome CCATAGATCAATAATACTTTTGGTTCAAATGTATGCCACATTACTTGTAAAACTATTGAC

CN 119300 CCATAGATCAATAATACTTTTGGTTCAAATGTATGCCACATTACTTGTAAAACTATTGAC

CAM 241 CCATAGATCAATAATACTTTTGGTTCAAATGTATGCCACATTACTTGTAAAACTATTGAC

CN 113754 CCATAGATCAATAATACTTTTGGTTCAAATGTATGCCACATTACTTGTAAAACTATTGAC

CAM 236 CCATAGATCAATAATACTTTTGGTTCAAATGTATGCCACATTACTTGTAAAACTATTGAC

CO46 NCBI CCATAGATCAATAATACTTTTGGTTCAAATGTATGCCACATTACTTGTAAAACTATTGAC

CN 120013 CCATAGATCAATAATACTTTTGGTTCAAATGTATGCCACATTACTTGTAAAACTATTGAC

Blaine Creek CCATAGATCAATAATACTTTTGGTTCAAATGTATGCCACATTACTTGTAAAACTATTGAC

09-CS0040 CCATAGATCAATAATACTTTTGGTTCAAATGTATGCCACATTACTTGTAAAACTATTGAC

17CS1133 CCATAGATCAATAATACTTTTGGTTCAAATGTATGCCACATTACTTGTAAAACTATTGAC

Jasper CCATAGATCAATAATACTTTTGGTTCAAATGTATGCCACATTACTTGTAAAACTATTGAC

CN 120017 CCATAGATCAATAATACTTTTGGTTCAAATGTATGCCACATTACTTGTAAAACTATTGAC

CN 120027 CCATAGATCAATAATACTTTTGGTTCAAATGTATGCCACATTACTTGTAAAACTATTGAC

CN 120030 CCATAGATCAATAATACTTTTGGTTCAAATGTATGCCACATTACTTGTAAAACTATTGAC

Hoga CCATAGATCAATAATACTTTTGGTTCAAATGTATGCCACATTACTTGTAAAACTATTGAC

Yellowstone CCATAGATCAATAATACTTTTGGTTCAAATGTATGCCACATTACTTGTAAAACTATTGAC

CN 119294 CCATAGATCAATAATACTTTTGGTTCAAATGTATGCCACATTACTTGTAAAACTATTGAC

************************************************************

CN 120025 TAAAAGATTAATTGGGATATACATGTTTTTTATAATGATTTCTCTCCTTTTTATGGATTT

CN 119205 TAAAAGATTAATTGGGATATACATGTTTTTTATAATGATTTCTCTCCTTTTTATGGATTT

CN 119243 TAAAAGATTAATTGGGATATACATGTTTTTTATAATGATTTCTCTCCTTTTTATGGATTT

Joelle NCBI TAAAAGATTAATTGGGATATACATGTTTTTTATAATGATTTCTCTCCTTTTTATGGATTT

Joelle AAFC TAAAAGATTAATTGGGATATACATGTTTTTTATAATGATTTCTCTCCTTTTTATGGATTT

Joelle phyto TAAAAGATTAATTGGGATATACATGTTTTTTATAATGATTTCTCTCCTTTTTATGGATTT

DH55 ref genome TAAAAGATTAATTGGGATATACATGTTTTTTATAATGATTTCTCTCCTTTTTATGGATTT

CN 119300 TAAAAGATTAATTGGGATATACATGTTTTTTATAATGATTTCTCTCCTTTTTATGGATTT

CAM 241 TAAAAGATTAATTGGGATATACATGTTTTTTATAATGATTTCTCTCCTTTTTATGGATTT

CN 113754 TAAAAGATTAATTGGGATATACATGTTTTTTATAATGATTTCTCTCCTTTTTATGGATTT

CAM 236 TAAAAGATTAATTGGGATATACATGTTTTTTATAATGATTTCTCTCCTTTTTATGGATTT

CO46 NCBI TAAAAGATTAATTGGGATATACATGTTTTTTATAATGATTTCTCTCCTTTTTATGGATTT

CN 120013 TAAAAGATTAATTGGGATATACATGTTTTTTATAATGATTTCTCTCCTTTTTATGGATTT

Blaine Creek TAAAAGATTAATTGGGATATACATGTTTTTTATAATGATTTCTCTCCTTTTTATGGATTT

09-CS0040 TAAAAGATTAATTGGGATATACATGTTTTTTATAATGATTTCTCTCCTTTTTATGGATTT

17CS1133 TAAAAGATTAATTGGGATATACATGTTTTTTATAATGATTTCTCTCCTTTTTATGGATTT

Jasper TAAAAGATTAATTGGGATATACATGTTTTTTATAATGATTTCTCTCCTTTTTATGGATTT

CN 120017 TAAAAGATTAATTGGGATATACATGTTTTTTATAATGATTTCTCTCCTTTTTATGGATTT

CN 120027 TAAAAGATTAATTGGGATATACATGTTTTTTATAATGATTTCTCTCCTTTTTATGGATTT

CN 120030 TAAAAGATTAATTGGGATATACATGTTTTTTATAATGATTTCTCTCCTTTTTATGGATTT

Hoga TAAAAGATTAATTGGGATATACATGTTTTTTATAATGATTTCTCTCCTTTTTATGGATTT

Yellowstone TAAAAGATTAATTGGGATATACATGTTTTTTATAATGATTTCTCTCCTTTTTATGGATTT

CN 119294 TAAAAGATTAATTGGGATATACATGTTTTTTATAATGATTTCTCTCCTTTTTATGGATTT

************************************************************

CN 120025 GCTTACTTGAAGATTAATTATCCAAAGGTTAATAGTTTCCTACCCTAGTTACCTCACATA

CN 119205 GCTTACTTGAAGATTAATTATCCAAAGGTTAATAGTTTCCTACCCTAGTTACCTCACATA

CN 119243 GCTTACTTGAAGATTAATTATCCAAAGGTTAATAGTTTCCTACCCTAGTTACCTCACATA

Joelle NCBI GCTTACTTGAAGATTAATTATCCAAAGGTTAATAGTTTCCTACCCTAGTTACCTCACATA

Joelle AAFC GCTTACTTGAAGATTAATTATCCAAAGGTTAATAGTTTCCTACCCTAGTTACCTCACATA

Joelle phyto GCTTACTTGAAGATTAATTATCCAAAGGTTAATAGTTTCCTACCCTAGTTACCTCACATA

DH55 ref genome GCTTACTTGAAGATTAATTATCCAAAGGTTAATAGTTTCCTACCCTAGTTACCTCACATA

CN 119300 GCTTACTTGAAGATTAATTATCCAAAGGTTAATAGTTTCCTACCCTAGTTACCTCACATA

CAM 241 GCTTACTTGAAGATTAATTATCCAAAGGTTAATAGTTTCCTACCCTAGTTACCTCACATA

CN 113754 GCTTACTTGAAGATTAATTATCCAAAGGTTAATAGTTTCCTACCCTAGTTACCTCACATA

CAM 236 GCTTACTTGAAGATTAATTATCCAAAGGTTAATAGTTTCCTACCCTAGTTACCTCACATA

CO46 NCBI GCTTACTTGAAGATTAATTATCCAAAGGTTAATAGTTTCCTACCCTAGTTACCTCACATA

CN 120013 GCTTACTTGAAGATTAATTATCCAAAGGTTAATAGTTTCCTACCCTAGTTACCTCACATA

Blaine Creek GCTTACTTGAAGATTAATTATCCAAAGGTTAATAGTTTCCTACCCTAGTTACCTCACATA

09-CS0040 GCTTACTTGAAGATTAATTATCCAAAGGTTAATAGTTTCCTACCCTAGTTACCTCACATA

17CS1133 GCTTACTTGAAGATTAATTATCCAAAGGTTAATAGTTTCCTACCCTAGTTACCTCACATA

Jasper GCTTACTTGAAGATTAATTATCCAAAGGTTAATAGTTTCCTACCCTAGTTACCTCACATA

CN 120017 GCTTACTTGAAGATTAATTATCCAAAGGTTAATAGTTTCCTACCCTAGTTACCTCACATA

CN 120027 GCTTACTTGAAGATTAATTATCCAAAGGTTAATAGTTTCCTACCCTAGTTACCTCACATA

CN 120030 GCTTACTTGAAGATTAATTATCCAAAGGTTAATAGTTTCCTACCCTAGTTACCTCACATA

Hoga GCTTACTTGAAGATTAATTATCCAAAGGTTAATAGTTTCCTACCCTAGTTACCTCACATA

Yellowstone GCTTACTTGAAGATTAATTATCCAAAGGTTAATAGTTTCCTACCCTAGTTACCTCACATA

CN 119294 GCTTACTTGAAGATTAATTATCCAAAGGTTAATAGTTTCCTACCCTAGTTACCTCACATA

************************************************************

CN 120025 GTTATGCTGCATATTTATGTTATTTGTTTATCAATTATCGCTCTTAATCTTTTAATGGAT

CN 119205 GTTATGCTGCATATTTATGTTATTTGTTTATCAATTATCGCTCTTAATCTGTTAATGGAT

CN 119243 GTTATGCTGCATATTTATGTTATTTGTTTATCAATTATCGCTCTTAATCTGTTAATGGAT

Joelle NCBI GTTATGCTGCATATTTATGTTATTTGTTTATCAATTATCGCTCTTAATCTGTTAATGGAT

Joelle AAFC GTTATGCTGCATATTTATGTTATTTGTTTATCAATTATCGCTCTTAATCTGTTAATGGAT

Joelle phyto GTTATGCTGCATATTTATGTTATTTGTTTATCAATTATCGCTCTTAATCTGTTAATGGAT

DH55 ref genome GTTATGCTGCATATTTATGTTATTTGTTTATCAATTATCGCTCTTAATCTGTTAATGGAT

CN 119300 GTTATGCTGCATATTTATGTTATTTGTTTATCAATTATCGCTCTTAATCTGTTAATGGAT

CAM 241 GTTATGCTGCATATTTATGTTATTTGTTTATCAATTATCGCTCTTAATCTGTTAATGGAT

CN 113754 GTTATGCTGCATATTTATGTTATTTGTTTATCAATTATCGCTCTTAATCTGTTAATGGAT

CAM 236 GTTATGCTGCATATTTATGTTATTTGTTTATCAATTATCGCTCTTAATCTGTTAATGGAT

CO46 NCBI GTTATGCTGCATATTTATGTTATTTGTTTATCAATTATCGCTCTTAATCTGTTAATGGAT

CN 120013 GTTATGCTGCATATTTATGTTATTTGTTTATCAATTATCGCTCTTAATCTGTTAATGGAT

Blaine Creek GTTATGCTGCATATTTATGTTATTTGTTTATCAATTATCGCTCTTAATCTGTTAATGGAT

09-CS0040 GTTATGCTGCATATTTATGTTATTTGTTTATCAATTATCGCTCTTAATCTGTTAATGGAT

17CS1133 GTTATGCTGCATATTTATGTTATTTGTTTATCAATTATCGCTCTTAATCTGTTAATGGAT

Jasper GTTATGCTGCATATTTATGTTATTTGTTTATCAATTATCGCTCTTAATCTGTTAATGGAT

CN 120017 GTTATGCTGCATATTTATGTTATTTGTTTATCAATTATCGCTCTTAATCTGTTAATGGAT

CN 120027 GTTATGCTGCATATTTATGTTATTTGTTTATCAATTATCGCTCTTAATCTGTTAATGGAT

CN 120030 GTTATGCTGCATATTTATGTTATTTGTTTATCAATTATCGCTCTTAATCTGTTAATGGAT

Hoga GTTATGCTGCATATTTATGTTATTTGTTTATCAATTATCGCTCTTAATCTGTTAATGGAT

Yellowstone GTTATGCTGCATATTTATGTTATTTGTTTATCAATTATCGCTCTTAATCTGTTAATGGAT

CN 119294 GTTATGCTGCATATTTATGTTATTTGTTTATCAATTATCGCTCTTAATCTGTTAATGGAT

************************************************** *********

CN 120025 GTGTGCATAGATATAAGATTGTGTCCTCAATATGAATCCACAACCTTGTAACTTTTGTTG

CN 119205 GTGTGCATAGATATAAGACTGTGTCCTCAATATGAATCCACAACCTTGTAACTTTTGTTG

CN 119243 GTGTGCATAGATATAAGACTGTGTCCTCAATATGAATCCACAACCTTGTAACTTTTGTTG

Joelle NCBI GTGTGCATAGATATAAGACTGTGTCCTCAATATGAATCCACAACCTTGTAACTTTTGTTG

Joelle AAFC GTGTGCATAGATATAAGACTGTGTCCTCAATATGAATCCACAACCTTGTAACTTTTGTTG

Joelle phyto GTGTGCATAGATATAAGACTGTGTCCTCAATATGAATCCACAACCTTGTAACTTTTGTTG

DH55 ref genome GTGTGCATAGATATAAGACTGTGTCCTCAATATGAATCCACAACCTTGTAACTTTTGTTG

CN 119300 GTGTGCATAGATATAAGACTGTGTCCTCAATATGAATCCACAACCTTGTAACTTTTGTTG

CAM 241 GTGTGCATAGATATAAGACTGTGTCCTCAATATGAATCCACAACCTTGTAACTTTTGTTG

CN 113754 GTGTGCATAGATATAAGACTGTGTCCTCAATATGAATCCACAACCTTGTAACTTTTGTTG

CAM 236 GTGTGCATAGATATAAGACTGTGTCCTCAATATGAATCCACAACCTTGTAACTTTTGTTG

CO46 NCBI GTGTGCATAGATATAAGACTGTGTCCTCAATATGAATCCACAACCTTGTAACTTTTGTTG

CN 120013 GTGTGCATAGATATAAGACTGTGTCCTCAATATGAATCCACAACCTTGTAACTTTTGTTG

Blaine Creek GTGTGCATAGATATAAGACTGTGTCCTCAATATGAATCCACAACCTTGTAACTTTTGTTG

09-CS0040 GTGTGCATAGATATAAGACTGTGTCCTCAATATGAATCCACAACCTTGTAACTTTTGTTG

17CS1133 GTGTGCATAGATATAAGACTGTGTCCTCAATATGAATCCACAACCTTGTAACTTTTGTTG

Jasper GTGTGCATAGATATAAGACTGTGTCCTCAATATGAATCCACAACCTTGTAACTTTTGTTG

CN 120017 GTGTGCATAGATATAAGACTGTGTCCTCAATATGAATCCACAACCTTGTAACTTTTGTTG

CN 120027 GTGTGCATAGATATAAGACTGTGTCCTCAATATGAATCCACAACCTTGTAACTTTTGTTG

CN 120030 GTGTGCATAGATATAAGACTGTGTCCTCAATATGAATCCACAACCTTGTAACTTTTGTTG

Hoga GTGTGCATAGATATAAGACTGTGTCCTCAATATGAATCCACAACCTTGTAACTTTTGTTG

Yellowstone GTGTGCATAGATATAAGACTGTGTCCTCAATATGAATCCACAACCTTGTAACTTTTGTTG

CN 119294 GTGTGCATAGATATAAGACTGTGTCCTCAATATGAATCCACAACCTTGTAACTTTTGTTG

****************** *****************************************

CN 120025 TGCAAATTGACAAATCACACAACCTTTGTATCTTGGGTCTTTTGTCATGAATATTGTCAA

CN 119205 TGCAAATTGACAAATCACACAACCTTTGTATCTTGGGTCTTTTGTCATGAATATTGTCAA

CN 119243 TGCAAATTGACAAATCACACAACCTTTGTATCTTGGGTCTTTTGTCATGAATATTGTCAA

Joelle NCBI TGCAAATTGACAAATCACACAACCTTTGTATCTTGGGTCTTTTGTCATGAATATTGTCAA

Joelle AAFC TGCAAATTGACAAATCACACAACCTTTGTATCTTGGGTCTTTTGTCATGAATATTGTCAA

Joelle phyto TGCAAATTGACAAATCACACAACCTTTGTATCTTGGGTCTTTTGTCATGAATATTGTCAA

DH55 ref genome TGCAAATTGACAAATCACACAACCTTTGTATCTTGGGTCTTTTGTCATGAATATTGTCAA

CN 119300 TGCAAATTGACAAATCACACAACCTTTGTATCTTGGGTCTTTTGTCATGAATATTGTCAA

CAM 241 TGCAAATTGACAAATCACACAACCTTTGTATCTTGGGTCTTTTGTCATGAATATTGTCAA

CN 113754 TGCAAATTGACAAATCACACAACCTTTGTATCTTGGGTCTTTTGTCATGAATATTGTCAA

CAM 236 TGCAAATTGACAAATCACACAACCTTTGTATCTTGGGTCTTTTGTCATGAATATTGTCAA

CO46 NCBI TGCAAATTGACAAATCACACAACCTTTGTATCTTGGGTCTTTTGTCATGAATATTGTCAA

CN 120013 TGCAAATTGACAAATCACACAACCTTTGTATCTTGGGTCTTTTGTCATGAATATTGTCAA

Blaine Creek TGCAAATTGACAAATCACACAACCTTTGTATCTTGGGTCTTTTGTCATGAATATTGTCAA

09-CS0040 TGCAAATTGACAAATCACACAACCTTTGTATCTTGGGTCTTTTGTCATGAATATTGTCAA

17CS1133 TGCAAATTGACAAATCACACAACCTTTGTATCTTGGGTCTTTTGTCATGAATATTGTCAA

Jasper TGCAAATTGACAAATCACACAACCTTTGTATCTTGGGTCTTTTGTCATGAATATTGTCAA

CN 120017 TGCAAATTGACAAATCACACAACCTTTGTATCTTGGGTCTTTTGTCATGAATATTGTCAA

CN 120027 TGCAAATTGACAAATCACACAACCTTTGTATCTTGGGTCTTTTGTCATGAATATTGTCAA

CN 120030 TGCAAATTGACAAATCACACAACCTTTGTATCTTGGGTCTTTTGTCATGAATATTGTCAA

Hoga TGCAAATTGACAAATCACACAACCTTTGTATCTTGGGTCTTTTGTCATGAATATTGTCAA

Yellowstone TGCAAATTGACAAATCACACAACCTTTGTATCTTGGGTCTTTTGTCATGAATATTGTCAA

CN 119294 TGCAAATTGACAAATCACACAACCTTTGTATCTTGGGTCTTTTGTCATGAATATTGTCAA

************************************************************

CN 120025 TAACACAACCTTGTTTCTTTGTTGCCTCTAGGAAATGTAAAACTCCAGAAAACTTGTCTT

CN 119205 TAACACAACCTTGTTTCTTTGTTGCCTCTAGGAAATGTAAAACTCCAGAAAACTTGTCTT

CN 119243 TAACACAACCTTGTTTCTTTGTTGCCTCTAGGAAATGTAAAACTCCAGAAAACTTGTCTT

Joelle NCBI TAACACAACCTTGTTTCTTTGTTGCCTCTAGGAAATGTAAAACTCCAGAAAACTTGTCTT

Joelle AAFC TAACACAACCTTGTTTCTTTGTTGCCTCTAGGAAATGTAAAACTCCAGAAAACTTGTCTT

Joelle phyto TAACACAACCTTGTTTCTTTGTTGCCTCTAGGAAATGTAAAACTCCAGAAAACTTGTCTT

DH55 ref genome TAACACAACCTTGTTTCTTTGTTGCCTCTAGGAAATGTAAAACTCCAGAAAACTTGTCTT

CN 119300 TAACACAACCTTGTTTCTTTGTTGCCTCTAGGAAATGTAAAACTCCAGAAAACTTGTCTT

CAM 241 TAACACAACCTTGTTTCTTTGTTGCCTCTAGGAAATGTAAAACTCCAGAAAACTTGTCTT

CN 113754 TAACACAACCTTGTTTCTTTGTTGCCTCTAGGAAATGTAAAACTCCAGAAAACTTGTCTT

CAM 236 TAACACAACCTTGTTTCTTTGTTGCCTCTAGGAAATGTAAAACTCCAGAAAACTTGTCTT

CO46 NCBI TAACACAACCTTGTTTCTTTGTTGCCTCTAGGAAATGTAAAACTCCAGAAAACTTGTCTT

CN 120013 TAACACAACCTTGTTTCTTTGTTGCCTCTAGGAAATGTAAAACTCCAGAAAACTTGTCTT

Blaine Creek TAACACAACCTTGTTTCTTTGTTGCCTCTAGGAAATGTAAAACTCCAGAAAACTTGTCTT

09-CS0040 TAACACAACCTTGTTTCTTTGTTGCCTCTAGGAAATGTAAAACTCCAGAAAACTTGTCTT

17CS1133 TAACACAACCTTGTTTCTTTGTTGCCTCTAGGAAATGTAAAACTCCAGAAAACTTGTCTT

Jasper TAACACAACCTTGTTTCTTTGTTGCCTCTAGGAAATGTAAAACTCCAGAAAACTTGTCTT

CN 120017 TAACACAACCTTGTTTCTTTGTTGCCTCTAGGAAATGTAAAACTCCAGAAAACTTGTCTT

CN 120027 TAACACAACCTTGTTTCTTTGTTGCCTCTAGGAAATGTAAAACTCCAGAAAACTTGTCTT

CN 120030 TAACACAACCTTGTTTCTTTGTTGCCTCTAGGAAATGTAAAACTCCAGAAAACTTGTCTT

Hoga TAACACAACCTTGTTTCTTTGTTGCCTCTAGGAAATGTAAAACTCCAGAAAACTTGTCTT

Yellowstone TAACACAACCTTGTTTCTTTGTTGCCTCTAGGAAATGTAAAACTCCAGAAAACTTGTCTT

CN 119294 TAACACAACCTTGTTTCTTTGTTGCCTCTAGGAAATGTAAAACTCCAGAAAACTTGTCTT

************************************************************

CN 120025 CATATAAGAAATATTAATATGATTTGTACATTGTTAAAACTAAACCCGGTGTAGTTTCAC

CN 119205 CATATAAGAAATATTAATATGATTTGTACATTGTTAAAACTAAACCCGGTGTAGTTTCAC

CN 119243 CATATAAGAAATATTAATATGATTTGTACATTGTTAAAACTAAACCCGGTGTAGTTTCAC

Joelle NCBI CATATAAGAAATATTAATATGATTTGTACATTGTTAAAACTAAACCCGGTGTAGTTTCAC

Joelle AAFC CATATAAGAAATATTAATATGATTTGTACATTGTTAAAACTAAACCCGGTGTAGTTTCAC

Joelle phyto CATATAAGAAATATTAATATGATTTGTACATTGTTAAAACTAAACCCGGTGTAGTTTCAC

DH55 ref genome CATATAAGAAATATTAATATGATTTGTACATTGTTAAAACTAAACCCGGTGTAGTTTCAC

CN 119300 CATATAAGAAATATTAATATGATTTGTACATTGTTAAAACTAAACCCGGTGTAGTTTCAC

CAM 241 CATATAAGAAATATTAATATGATTTGTACATTGTTAAAACTAAACCCGGTGTAGTTTCAC

CN 113754 CATATAAGAAATATTAATATGATTTGTACATTGTTAAAACTAAACCCGGTGTAGTTTCAC

CAM 236 CATATAAGAAATATTAATATGATTTGTACATTGTTAAAACTAAACCCGGTGTAGTTTCAC

CO46 NCBI CATATAAGAAATATTAATATGATTTGTACATTGTTAAAACTAAACCCGGTGTAGTTTCAC

CN 120013 CATATAAGAAATATTAATATGATTTGTACATTGTTAAAACTAAACCCGGTGTAGTTTCAC

Blaine Creek CATATAAGAAATATTAATATGATTTGTACATTGTTAAAACTAAACCCGGTGTAGTTTCAC

09-CS0040 CATATAAGAAATATTAATATGATTTGTACATTGTTAAAACTAAACCCGGTGTAGTTTCAC

17CS1133 CATATAAGAAATATTAATATGATTTGTACATTGTTAAAACTAAACCCGGTGTAGTTTCAC

Jasper CATATAAGAAATATTAATATGATTTGTACATTGTTAAAACTAAACCCGGTGTAGTTTCAC

CN 120017 CATATAAGAAATATTAATATGATTTGTACATTGTTAAAACTAAACCCGGTGTAGTTTCAC

CN 120027 CATATAAGAAATATTAATATGATTTGTACATTGTTAAAACTAAACCCGGTGTAGTTTCAC

CN 120030 CATATAAGAAATATTAATATGATTTGTACATTGTTAAAACTAAACCCGGTGTAGTTTCAC

Hoga CATATAAGAAATATTAATATGATTTGTACATTGTTAAAACTAAACCCGGTGTAGTTTCAC

Yellowstone CATATAAGAAATATTAATATGATTTGTACATTGTTAAAACTAAACCCGGTGTAGTTTCAC

CN 119294 CATATAAGAAATATTAATATGATTTGTACATTGTTAAAACTAAACCCGGTGTAGTTTCAC

************************************************************

CN 120025 TACAACCCTCCAATATATTAACTTAGTGGTTGTAGTGGTTTGGCCATGTTGGTCAAGATC

CN 119205 TACAACCCTCCAATATATTAACTTAGTGGTTGTAGTGGTTTGGCCATGTTGGTCAAGATC

CN 119243 TACAACCCTCCAATATATTAACTTAGTGGTTGTAGTGGTTTGGCCATGTTGGTCAAGATC

Joelle NCBI TACAACCCTCCAATATATTAACTTAGTGGTTGTAGTGGTTTGGCCATGTTGGTCAAGATC

Joelle AAFC TACAACCCTCCAATATATTAACTTAGTGGTTGTAGTGGTTTGGCCATGTTGGTCAAGATC

Joelle phyto TACAACCCTCCAATATATTAACTTAGTGGTTGTAGTGGTTTGGCCATGTTGGTCAAGATC

DH55 ref genome TACAACCCTCCAATATATTAACTTAGTGGTTGTAGTGGTTTGGCCATGTTGGTCAAGATC

CN 119300 TACAACCCTCCAATATATTAACTTAGTGGTTGTAGTGGTTTGGCCATGTTGGTCAAGATC

CAM 241 TACAACCCTCCAATATATTAACTTAGTGGTTGTAGTGGTTTGGCCATGTTGGTCAAGATC

CN 113754 TACAACCCTCCAATATATTAACTTAGTGGTTGTAGTGGTTTGGCCATGTTGGTCAAGATC

CAM 236 TACAACCCTCCAATATATTAACTTAGTGGTTGTAGTGGTTTGGCCATGTTGGTCAAGATC

CO46 NCBI TACAACCCTCCAATATATTAACTTAGTGGTTGTAGTGGTTTGGCCATGTTGGTCAAGATC

CN 120013 TACAACCCTCCAATATATTAACTTAGTGGTTGTAGTGGTTTGGCCATGTTGGTCAAGATC

Blaine Creek TACAACCCTCCAATATATTAACTTAGTGGTTGTAGTGGTTTGGCCATGTTGGTCAAGATC

09-CS0040 TACAACCCTCCAATATATTAACTTAGTGGTTGTAGTGGTTTGGCCATGTTGGTCAAGATC

17CS1133 TACAACCCTCCAATATATTAACTTAGTGGTTGTAGTGGTTTGGCCATGTTGGTCAAGATC

Jasper TACAACCCTCCAATATATTAACTTAGTGGTTGTAGTGGTTTGGCCATGTTGGTCAAGATC

CN 120017 TACAACCCTCCAATATATTAACTTAGTGGTTGTAGTGGTTTGGCCATGTTGGTCAAGATC

CN 120027 TACAACCCTCCAATATATTAACTTAGTGGTTGTAGTGGTTTGGCCATGTTGGTCAAGATC

CN 120030 TACAACCCTCCAATATATTAACTTAGTGGTTGTAGTGGTTTGGCCATGTTGGTCAAGATC

Hoga TACAACCCTCCAATATATTAACTTAGTGGTTGTAGTGGTTTGGCCATGTTGGTCAAGATC

Yellowstone TACAACCCTCCAATATATTAACTTAGTGGTTGTAGTGGTTTGGCCATGTTGGTCAAGATC

CN 119294 TACAACCCTCCAATATATTAACTTAGTGGTTGTAGTGGTTTGGCCATGTTGGTCAAGATC

************************************************************

CN 120025 GTAGGCCGATTCTCTCACTTGCTGCATACTTTGTTAGGGTTTGTTCACGCCTTATACTGA

CN 119205 GTAGGCCGATTCTCTCACTTGCTGCATACTTTGTTAGGGTTTGTTCACGCCTTATACTGA

CN 119243 GTAGGCCGATTCTCTCACTTGCTGCATACTTTGTTAGGGTTTGTTCACGCCTTATACTGA

Joelle NCBI GTAGGCCGATTCTCTCACTTGCTGCATACTTTGTTAGGGTTTGTTCACGCCTTATACTGA

Joelle AAFC GTAGGCCGATTCTCTCACTTGCTGCATACTTTGTTAGGGTTTGTTCACGCCTTATACTGA

Joelle phyto GTAGGCCGATTCTCTCACTTGCTGCATACTTTGTTAGGGTTTGTTCACGCCTTATACTGA

DH55 ref genome GTAGGCCGATTCTCTCACTTGCTGCATACTTTGTTAGGGTTTGTTCACGCCTTATACTGA

CN 119300 GTAGGCCGATTCTCTCACTTGCTGCATACTTTGTTAGGGTTTGTTCACGCCTTATACTGA

CAM 241 GTAGGCCGATTCTCTCACTTGCTGCATACTTTGTTAGGGTTTGTTCACGCCTTATACTGA

CN 113754 GTAGGCCGATTCTCTCACTTGCTGCATACTTTGTTAGGGTTTGTTCACGCCTTATACTGA

CAM 236 GTAGGCCGATTCTCTCACTTGCTGCATACTTTGTTAGGGTTTGTTCACGCCTTATACTGA

CO46 NCBI GTAGGCCGATTCTCTCACTTGCTGCATACTTTGTTAGGGTTTGTTCACGCCTTATACTGA

CN 120013 GTAGGCCGATTCTCTCACTTGCTGCATACTTTGTTAGGGTTTGTTCACGCCTTATACTGA

Blaine Creek GTAGGCCGATTCTCTCACTTGCTGCATACTTTGTTAGGGTTTGTTCACGCCTTATACTGA

09-CS0040 GTAGGCCGATTCTCTCACTTGCTGCATACTTTGTTAGGGTTTGTTCACGCCTTATACTGA

17CS1133 GTAGGCCGATTCTCTCACTTGCTGCATACTTTGTTAGGGTTTGTTCACGCCTTATACTGA

Jasper GTAGGCCGATTCTCTCACTTGCTGCATACTTTGTTAGGGTTTGTTCACGCCTTATACTGA

CN 120017 GTAGGCCGATTCTCTCACTTGCTGCATACTTTGTTAGGGTTTGTTCACGCCTTATACTGA

CN 120027 GTAGGCCGATTCTCTCACTTGCTGCATACTTTGTTAGGGTTTGTTCACGCCTTATACTGA

CN 120030 GTAGGCCGATTCTCTCACTTGCTGCATACTTTGTTAGGGTTTGTTCACGCCTTATACTGA

Hoga GTAGGCCGATTCTCTCACTTGCTGCATACTTTGTTAGGGTTTGTTCACGCCTTATACTGA

Yellowstone GTAGGCCGATTCTCTCACTTGCTGCATACTTTGTTAGGGTTTGTTCACGCCTTATACTGA

CN 119294 GTAGGCCGATTCTCTCACTTGCTGCATACTTTGTTAGGGTTTGTTCACGCCTTATACTGA

************************************************************

CN 120025 ATGTTAGGTCCAGCCTTGGAATAGCCGAGACACCTGACTCAGTAATTATGAGTTAAAAAG

CN 119205 ATGTTAGGTCCAGCCTTGGAATAGCCGAGACACCTGAATCAGTAATTATGAGTTAAAAAG

CN 119243 ATGTTAGGTCCAGCCTTGGAATAGCCGAGACACCTGAATCAGTAATTATGAGTTAAAAAG

Joelle NCBI ATGTTAGGTCCAGCCTTGGAATAGCCGAGACACCTGAATCAGTAATTATGAGTTAAAAAG

Joelle AAFC ATGTTAGGTCCAGCCTTGGAATAGCCGAGACACCTGAATCAGTAATTATGAGTTAAAAAG

Joelle phyto ATGTTAGGTCCAGCCTTGGAATAGCCGAGACACCTGAATCAGTAATTATGAGTTAAAAAG

DH55 ref genome ATGTTAGGTCCAGCCTTGGAATAGCCGAGACACCTGAATCAGTAATTATGAGTTAAAAAG

CN 119300 ATGTTAGGTCCAGCCTTGGAATAGCCGAGACACCTGAATCAGTAATTATGAGTTAAAAAG

CAM 241 ATGTTAGGTCCAGCCTTGGAATAGCCGAGACACCTGAATCAGTAATTATGAGTTAAAAAG

CN 113754 ATGTTAGGTCCAGCCTTGGAATAGCCGAGACACCTGAATCAGTAATTATGAGTTAAAAAG

CAM 236 ATGTTAGGTCCAGCCTTGGAATAGCCGAGACACCTGAATCAGTAATTATGAGTTAAAAAG

CO46 NCBI ATGTTAGGTCCAGCCTTGGAATAGCCGAGACACCTGAATCAGTAATTATGAGTTAAAAAG

CN 120013 ATGTTAGGTCCAGCCTTGGAATAGCCGAGACACCTGAATCAGTAATTATGAGTTAAAAAG

Blaine Creek ATGTTAGGTCCAGCCTTGGAATAGCCGAGACACCTGAATCAGTAATTATGAGTTAAAAAG

09-CS0040 ATGTTAGGTCCAGCCTTGGAATAGCCGAGACACCTGAATCAGTAATTATGAGTTAAAAAG

17CS1133 ATGTTAGGTCCAGCCTTGGAATAGCCGAGACACCTGAATCAGTAATTATGAGTTAAAAAG

Jasper ATGTTAGGTCCAGCCTTGGAATAGCCGAGACACCTGAATCAGTAATTATGAGTTAAAAAG

CN 120017 ATGTTAGGTCCAGCCTTGGAATAGCCGAGACACCTGAATCAGTAATTATGAGTTAAAAAG

CN 120027 ATGTTAGGTCCAGCCTTGGAATAGCCGAGACACCTGAATCAGTAATTATGAGTTAAAAAG

CN 120030 ATGTTAGGTCCAGCCTTGGAATAGCCGAGACACCTGAATCAGTAATTATGAGTTAAAAAG

Hoga ATGTTAGGTCCAGCCTTGGAATAGCCGAGACACCTGAATCAGTAATTATGAGTTAAAAAG

Yellowstone ATGTTAGGTCCAGCCTTGGAATAGCCGAGACACCTGAATCAGTAATTATGAGTTAAAAAG

CN 119294 ATGTTAGGTCCAGCCTTGGAATAGCCGAGACACCTGAATCAGTAATTATGAGTTAAAAAG

************************************* **********************

CN 120025 AAACGTTATTCACTCAATAACTCATTTTTGCATCCTTAATTTTGGTGCAAAGAGCTTAAC

CN 119205 AAACGTTATTCACTCAATAACTCATTTTTGCATCCTTAATTTGGGTGCAAAGAGCTTAAC

CN 119243 AAACGTTATTCACTCAATAACTCATTTTTGCATCCTTAATTTGGGTGCAAAGAGCTTAAC

Joelle NCBI AAACGTTATTCACTCAATAACTCATTTTTGCATCCTTAATTTGGGTGCAAAGAGCTTAAC

Joelle AAFC AAACGTTATTCACTCAATAACTCATTTTTGCATCCTTAATTTGGGTGCAAAGAGCTTAAC

Joelle phyto AAACGTTATTCACTCAATAACTCATTTTTGCATCCTTAATTTGGGTGCAAAGAGCTTAAC

DH55 ref genome AAACGTTATTCACTCAATAACTCATTTTTGCATCCTTAATTTGGGTGCAAAGAGCTTAAC

CN 119300 AAACGTTATTCACTCAATAACTCATTTTTGCATCCTTAATTTGGGTGCAAAGAGCTTAAC

CAM 241 AAACGTTATTCACTCAATAACTCATTTTTGCATCCTTAATTTGGGTGCAAAGAGCTTAAC

CN 113754 AAACGTTATTCACTCAATAACTCATTTTTGCATCCTTAATTTGGGTGCAAAGAGCTTAAC

CAM 236 AAACGTTATTCACTCAATAACTCATTTTTGCATCCTTAATTTGGGTGCAAAGAGCTTAAC

CO46 NCBI AAACGTTATTCACTCAATAACTCATTTTTGCATCCTTAATTTGGGTGCAAAGAGCTTAAC

CN 120013 AAACGTTATTCACTCAATAACTCATTTTTGCATCCTTAATTTGGGTGCAAAGAGCTTAAC

Blaine Creek AAACGTTATTCACTCAATAACTCATTTTTGCATCCTTAATTTGGGTGCAAAGAGCTTAAC

09-CS0040 AAACGTTATTCACTCAATAACTCATTTTTGCATCCTTAATTTGGGTGCAAAGAGCTTAAC

17CS1133 AAACGTTATTCACTCAATAACTCATTTTTGCATCCTTAATTTGGGTGCAAAGAGCTTAAC

Jasper AAACGTTATTCACTCAATAACTCATTTTTGCATCCTTAATTTGGGTGCAAAGAGCTTAAC

CN 120017 AAACGTTATTCACTCAATAACTCATTTTTGCATCCTTAATTTGGGTGCAAAGAGCTTAAC

CN 120027 AAACGTTATTCACTCAATAACTCATTTTTGCATCCTTAATTTGGGTGCAAAGAGCTTAAC

CN 120030 AAACGTTATTCACTCAATAACTCATTTTTGCATCCTTAATTTGGGTGCAAAGAGCTTAAC

Hoga AAACGTTATTCACTCAATAACTCATTTTTGCATCCTTAATTTGGGTGCAAAGAGCTTAAC

Yellowstone AAACGTTATTCACTCAATAACTCATTTTTGCATCCTTAATTTGGGTGCAAAGAGCTTAAC

CN 119294 AAACGTTATTCACTCAATAACTCATTTTTGCATCCTTAATTTGGGTGCAAAGAGCTTAAC

****************************************** *****************

CN 120025 TTCACAATGGAACTGAAACCTATTCGCACAAATTATTAAGTGACTTAGCGGTAGTTTTGT

CN 119205 TTCACAATGGAACTGAAACCTATTCGCACAAATTATTAAGTGACTTAGCGGTAGTTTTGT

CN 119243 TTCACAATGGAACTGAAACCTATTCGCACAAATTATTAAGTGACTTAGCGGTAGTTTTGT

Joelle NCBI TTCACAATGGAACTGAAACCTATTCGCACAAATTATTAAGTGACTTAGCGGTAGTTTTGT

Joelle AAFC TTCACAATGGAACTGAAACCTATTCGCACAAATTATTAAGTGACTTAGCGGTAGTTTTGT

Joelle phyto TTCACAATGGAACTGAAACCTATTCGCACAAATTATTAAGTGACTTAGCGGTAGTTTTGT

DH55 ref genome TTCACAATGGAACTGAAACCTATTCGCACAAATTATTAAGTGACTTAGCGGTAGTTTTGT

CN 119300 TTCACAATGGAACTGAAACCTATTCGCACAAATTATTAAGTGACTTAGCGGTAGTTTTGT

CAM 241 TTCACAATGGAACTGAAACCTATTCGCACAAATTATTAAGTGACTTAGCGGTAGTTTTGT

CN 113754 TTCACAATGGAACTGAAACCTATTCGCACAAATTATTAAGTGACTTAGCGGTAGTTTTGT

CAM 236 TTCACAATGGAACTGAAACCTATTCGCACAAATTATTAAGTGACTTAGCGGTAGTTTTGT

CO46 NCBI TTCACAATGGAACTGAAACCTATTCGCACAAATTATTAAGTGACTTAGCGGTAGTTTTGT

CN 120013 TTCACAATGGAACTGAAACCTATTCGCACAAATTATTAAGTGACTTAGCGGTAGTTTTGT

Blaine Creek TTCACAATGGAACTGAAACCTATTCGCACAAATTATTAAGTGACTTAGCGGTAGTTTTGT

09-CS0040 TTCACAATGGAACTGAAACCTATTCGCACAAATTATTAAGTGACTTAGCGGTAGTTTTGT

17CS1133 TTCACAATGGAACTGAAACCTATTCGCACAAATTATTAAGTGACTTAGCGGTAGTTTTGT

Jasper TTCACAATGGAACTGAAACCTATTCGCACAAATTATTAAGTGACTTAGCGGTAGTTTTGT

CN 120017 TTCACAATGGAACTGAAACCTATTCGCACAAATTATTAAGTGACTTAGCGGTAGTTTTGT

CN 120027 TTCACAATGGAACTGAAACCTATTCGCACAAATTATTAAGTGACTTAGCGGTAGTTTTGT

CN 120030 TTCACAATGGAACTGAAACCTATTCGCACAAATTATTAAGTGACTTAGCGGTAGTTTTGT

Hoga TTCACAATGGAACTGAAACCTATTCGCACAAATTATTAAGTGACTTAGCGGTAGTTTTGT

Yellowstone TTCACAATGGAACTGAAACCTATTCGCACAAATTATTAAGTGACTTAGCGGTAGTTTTGT

CN 119294 TTCACAATGGAACTGAAACCTATTCGCACAAATTATTAAGTGACTTAGCGGTAGTTTTGT

************************************************************

CN 120025 CAAATTTGCTTTGACCTCTATTAGGTAATTATGTAGTTTTAGGTTTTTCCTTCTTAATTT

CN 119205 CAAATTTGCTTTGACCTCTATTAGGTAATTATGTAGTTTTAGGTTTTTCCTTCTTAATTT

CN 119243 CAAATTTGCTTTGACCTCTATTAGGTAATTATGTAGTTTTAGGTTTTTCCTTCTTAATTT

Joelle NCBI CAAATTTGCTTTGACCTCTATTAGGTAATTATGTAGTTTTAGGTTTTTCCTTCTTAATTT

Joelle AAFC CAAATTTGCTTTGACCTCTATTAGGTAATTATGTAGTTTTAGGTTTTTCCTTCTTAATTT

Joelle phyto CAAATTTGCTTTGACCTCTATTAGGTAATTATGTAGTTTTAGGTTTTTCCTTCTTAATTT

DH55 ref genome CAAATTTGCTTTGACCTCTATTAGGTAATTATGTAGTTTTAGGTTTTTCCTTCTTAATTT

CN 119300 CAAATTTGCTTTGACCTCTATTAGGTAATTATGTAGTTTTAGGTTTTTCCTTCTTAATTT

CAM 241 CAAATTTGCTTTGACCTCTATTAGGTAATTATGTAGTTTTAGGTTTTTCCTTCTTAATTT

CN 113754 CAAATTTGCTTTGACCTCTATTAGGTAATTATGTAGTTTTAGGTTTTTCCTTCTTAATTT

CAM 236 CAAATTTGCTTTGACCTCTATTAGGTAATTATGTAGTTTTAGGTTTTTCCTTCTTAATTT

CO46 NCBI CAAATTTGCTTTGACCTCTATTAGGTAATTATGTAGTTTTAGGTTTTTCCTTCTTAATTT

CN 120013 CAAATTTGCTTTGACCTCTATTAGGTAATTATGTAGTTTTAGGTTTTTCCTTCTTAATTT

Blaine Creek CAAATTTGCTTTGACCTCTATTAGGTAATTATGTAGTTTTAGGTTTTTCCTTCTTAATTT

09-CS0040 CAAATTTGCTTTGACCTCTATTAGGTAATTATGTAGTTTTAGGTTTTTCCTTCTTAATTT

17CS1133 CAAATTTGCTTTGACCTCTATTAGGTAATTATGTAGTTTTAGGTTTTTCCTTCTTAATTT

Jasper CAAATTTGCTTTGACCTCTATTAGGTAATTATGTAGTTTTAGGTTTTTCCTTCTTAATTT

CN 120017 CAAATTTGCTTTGACCTCTATTAGGTAATTATGTAGTTTTAGGTTTTTCCTTCTTAATTT

CN 120027 CAAATTTGCTTTGACCTCTATTAGGTAATTATGTAGTTTTAGGTTTTTCCTTCTTAATTT

CN 120030 CAAATTTGCTTTGACCTCTATTAGGTAATTATGTAGTTTTAGGTTTTTCCTTCTTAATTT

Hoga CAAATTTGCTTTGACCTCTATTAGGTAATTATGTAGTTTTAGGTTTTTCCTTCTTAATTT

Yellowstone CAAATTTGCTTTGACCTCTATTAGGTAATTATGTAGTTTTAGGTTTTTCCTTCTTAATTT

CN 119294 CAAATTTGCTTTGACCTCTATTAGGTAATTATGTAGTTTTAGGTTTTTCCTTCTTAATTT

************************************************************

CN 120025 GGAACAATTTATCTAGATTGGTAATACTTGATAGAGGCCTCAGTAAGTTGATGTAATTTC

CN 119205 GGAACAATTTATCTAGATTGGTAATACTTGATAGAGGCCTCAGTAAGTTGATGTAATTTC

CN 119243 GGAACAATTTATCTAGATTGGTAATACTTGATAGAGGCCTCAGTAAGTTGATGTAATTTC

Joelle NCBI GGAACAATTTATCTAGATTGGTAATACTTGATAGAGGCCTCAGTAAGTTGATGTAATTTC

Joelle AAFC GGAACAATTTATCTAGATTGGTAATACTTGATAGAGGCCTCAGTAAGTTGATGTAATTTC

Joelle phyto GGAACAATTTATCTAGATTGGTAATACTTGATAGAGGCCTCAGTAAGTTGATGTAATTTC

DH55 ref genome GGAACAATTTATCTAGATTGGTAATACTTGATAGAGGCCTCAGTAAGTTGATGTAATTTC

CN 119300 GGAACAATTTATCTAGATTGGTAATACTTGATAGAGGCCTCAGTAAGTTGATGTAATTTC

CAM 241 GGAACAATTTATCTAGATTGGTAATACTTGATAGAGGCCTCAGTAAGTTGATGTAATTTC

CN 113754 GGAACAATTTATCTAGATTGGTAATACTTGATAGAGGCCTCAGTAAGTTGATGTAATTTC

CAM 236 GGAACAATTTATCTAGATTGGTAATACTTGATAGAGGCCTCAGTAAGTTGATGTAATTTC

CO46 NCBI GGAACAATTTATCTAGATTGGTAATACTTGATAGAGGCCTCAGTAAGTTGATGTAATTTC

CN 120013 GGAACAATTTATCTAGATTGGTAATACTTGATAGAGGCCTCAGTAAGTTGATGTAATTTC

Blaine Creek GGAACAATTTATCTAGATTGGTAATACTTGATAGAGGCCTCAGTAAGTTGATGTAATTTC

09-CS0040 GGAACAATTTATCTAGATTGGTAATACTTGATAGAGGCCTCAGTAAGTTGATGTAATTTC

17CS1133 GGAACAATTTATCTAGATTGGTAATACTTGATAGAGGCCTCAGTAAGTTGATGTAATTTC

Jasper GGAACAATTTATCTAGATTGGTAATACTTGATAGAGGCCTCAGTAAGTTGATGTAATTTC

CN 120017 GGAACAATTTATCTAGATTGGTAATACTTGATAGAGGCCTCAGTAAGTTGATGTAATTTC

CN 120027 GGAACAATTTATCTAGATTGGTAATACTTGATAGAGGCCTCAGTAAGTTGATGTAATTTC

CN 120030 GGAACAATTTATCTAGATTGGTAATACTTGATAGAGGCCTCAGTAAGTTGATGTAATTTC

Hoga GGAACAATTTATCTAGATTGGTAATACTTGATAGAGGCCTCAGTAAGTTGATGTAATTTC

Yellowstone GGAACAATTTATCTAGATTGGTAATACTTGATAGAGGCCTCAGTAAGTTGATGTAATTTC

CN 119294 GGAACAATTTATCTAGATTGGTAATACTTGATAGAGGCCTCAGTAAGTTGATGTAATTTC

************************************************************

CN 120025 ATTGGCAGAAAACTCTTGACCTTTACTGCTTGATTTTTGGCTTTCAATATAGTTAATTGG

CN 119205 ATTGGCAGAAAACTCTTGACCTTTACTGCTTGATTTTTGGCTTTCAATATAGTTAATTGG

CN 119243 ATTGGCAGAAAACTCTTGACCTTTACTGCTTGATTTTTGGCTTTCAATATAGTTAATTGG

Joelle NCBI ATTGGCAGAAAACTCTTGACCTTTACTGCTTGATTTTTGGCTTTCAATATAGTTAATTGG

Joelle AAFC ATTGGCAGAAAACTCTTGACCTTTACTGCTTGATTTTTGGCTTTCAATATAGTTAATTGG

Joelle phyto ATTGGCAGAAAACTCTTGACCTTTACTGCTTGATTTTTGGCTTTCAATATAGTTAATTGG

DH55 ref genome ATTGGCAGAAAACTCTTGACCTTTACTGCTTGATTTTTGGCTTTCAATATAGTTAATTGG

CN 119300 ATTGGCAGAAAACTCTTGACCTTTACTGCTTGATTTTTGGCTTTCAATATAGTTAATTGG

CAM 241 ATTGGCAGAAAACTCTTGACCTTTACTGCTTGATTTTTGGCTTTCAATATAGTTAATTGG

CN 113754 ATTGGCAGAAAACTCTTGACCTTTACTGCTTGATTTTTGGCTTTCAATATAGTTAATTGG

CAM 236 ATTGGCAGAAAACTCTTGACCTTTACTGCTTGATTTTTGGCTTTCAATATAGTTAATTGG

CO46 NCBI ATTGGCAGAAAACTCTTGACCTTTACTGCTTGATTTTTGGCTTTCAATATAGTTAATTGG

CN 120013 ATTGGCAGAAAACTCTTGACCTTTACTGCTTGATTTTTGGCTTTCAATATAGTTAATTGG

Blaine Creek ATTGGCAGAAAACTCTTGACCTTTACTGCTTGATTTTTGGCTTTCAATATAGTTAATTGG

09-CS0040 ATTGGCAGAAAACTCTTGACCTTTACTGCTTGATTTTTGGCTTTCAATATAGTTAATTGG

17CS1133 ATTGGCAGAAAACTCTTGACCTTTACTGCTTGATTTTTGGCTTTCAATATAGTTAATTGG

Jasper ATTGGCAGAAAACTCTTGACCTTTACTGCTTGATTTTTGGCTTTCAATATAGTTAATTGG

CN 120017 ATTGGCAGAAAACTCTTGACCTTTACTGCTTGATTTTTGGCTTTCAATATAGTTAATTGG

CN 120027 ATTGGCAGAAAACTCTTGACCTTTACTGCTTGATTTTTGGCTTTCAATATAGTTAATTGG

CN 120030 ATTGGCAGAAAACTCTTGACCTTTACTGCTTGATTTTTGGCTTTCAATATAGTTAATTGG

Hoga ATTGGCAGAAAACTCTTGACCTTTACTGCTTGATTTTTGGCTTTCAATATAGTTAATTGG

Yellowstone ATTGGCAGAAAACTCTTGACCTTTACTGCTTGATTTTTGGCTTTCAATATAGTTAATTGG

CN 119294 ATTGGCAGAAAACTCTTGACCTTTACTGCTTGATTTTTGGCTTTCAATATAGTTAATTGG

************************************************************

CN 120025 AACCTTACAGTTTCTATACAAACTAGGAAGAAAATGGAAGACCGGTTTCCTATTCTTAGG

CN 119205 AACCTTACAGTTTCTATACAAACGAGGAAGAAAATGGAAGACCGGTTTCCTATTCTTAGG

CN 119243 AACCTTACAGTTTCTATACAAAAGAGGAAGAAAATGGAAGACCGGTTTCCTATTCTTAGG

Joelle NCBI AACCTTACAGTTTCTATACAAAAGAGGAAGAAAATGGAAGACCGGTTTCCTATTCTTAGG

Joelle AAFC AACCTTACAGTTTCTATACAAAAGAGGAAGAAAATGGAAGACCGGTTTCCTATTCTTAGG

Joelle phyto AACCTTACAGTTTCTATACAAAAGAGGAAGAAAATGGAAGACCGGTTTCCTATTCTTAGG

DH55 ref genome AACCTTACAGTTTCTATACAAACGAGGAAGAAAATGGAAGACCGGTTTCCTATTCTTAGG

CN 119300 AACCTTACAGTTTCTATACAAACGAGGAAGAAAATGGAAGACCGGTTTCCTATTCTTAGG

CAM 241 AACCTTACAGTTTCTATACAAACGAGGAAGAAAATGGAAGACCGGTTTCCTATTCTTAGG

CN 113754 AACCTTACAGTTTCTATACAAACGAGGAAGAAAATGGAAGACCGGTTTCCTATTCTTAGG

CAM 236 AACCTTACAGTTTCTATACAAACGAGGAAGAAAATGGAAGACCGGTTTCCTATTCTTAGG

CO46 NCBI AACCTTACAGTTTCTATACAAACGAGGAAGAAAATGGAAGACCGGTTTCCTATTCTTAGG

CN 120013 AACCTTACAGTTTCTATACAAACGAGGAAGAAAATGGAAGACCGGTTTCCTATTCTTAGG

Blaine Creek AACCTTACAGTTTCTATACAAACGAGGAAGAAAATGGAAGACCGGTTTCCTATTCTTAGG

09-CS0040 AACCTTACAGTTTCTATACAAACGAGGAAGAAAATGGAAGACCGGTTTCCTATTCTTAGG

17CS1133 AACCTTACAGTTTCTATACAAACGAGGAAGAAAATGGAAGACCGGTTTCCTATTCTTAGG

Jasper AACCTTACAGTTTCTATACAAACGAGGAAGAAAATGGAAGACCGGTTTCCTATTCTTAGG

CN 120017 AACCTTACAGTTTCTATACAAACGAGGAAGAAAATGGAAGACCGGTTTCCTATTCTTAGG

CN 120027 AACCTTACAGTTTCTATACAAACGAGGAAGAAAATGGAAGACCGGTTTCCTATTCTTAGG

CN 120030 AACCTTACAGTTTCTATACAAACGAGGAAGAAAATGGAAGACCGGTTTCCTATTCTTAGG

Hoga AACCTTACAGTTTCTATACAAACGAGGAAGAAAATGGAAGACCGGTTTCCTATTCTTAGG

Yellowstone AACCTTACAGTTTCTATACAAACGAGGAAGAAAATGGAAGACCGGTTTCCTATTCTTAGG

CN 119294 AACCTTACAGTTTCTATACAAACGAGGAAGAAAATGGAAGACCGGTTTCCTATTCTTAGG

********************** ************************************

CN 120025 AAAGTGTTTAGATTTTCATTTCAAAGAAACATAAAATAAAATTATGAGATTGTTGTCTAA

CN 119205 AAAGTGTTTAGATTTTCATTTCAAAGAAACATAAAATAAAATTATGAGATTGTTGTCTAA

CN 119243 AAAGTGTTTAGATTTTCATTTCAAAGAAACATAAAATAAAATTATGAGATTGTTGTCTAA

Joelle NCBI AAAGTGTTTAGATTTTCATTTCAAAGAAACATAAAATAAAATTATGAGATTGTTGTCTAA

Joelle AAFC AAAGTGTTTAGATTTTCATTTCAAAGAAACATAAAATAAAATTATGAGATTGTTGTCTAA

Joelle phyto AAAGTGTTTAGATTTTCATTTCAAAGAAACATAAAATAAAATTATGAGATTGTTGTCTAA

DH55 ref genome AAAGTGTTTAGATTTTCATTTCAAAGAAACATAAAATAAAATTATGAGATTGTTGTCTAA

CN 119300 AAAGTGTTTAGATTTTCATTTCAAAGAAACATAAAATAAAATTATGAGATTGTTGTCTAA

CAM 241 AAAGTGTTTAGATTTTCATTTCAAAGAAACATAAAATAAAATTATGAGATTGTTGTCTAA

CN 113754 AAAGTGTTTAGATTTTCATTTCAAAGAAACATAAAATAAAATTATGAGATTGTTGTCTAA

CAM 236 AAAGTGTTTAGATTTTCATTTCAAAGAAACATAAAATAAAATTATGAGATTGTTGTCTAA

CO46 NCBI AAAGTGTTTAGATTTTCATTTCAAAGAAACATAAAATAAAATTATGAGATTGTTGTCTAA

CN 120013 AAAGTGTTTAGATTTTCATTTCAAAGAAACATAAAATAAAATTATGAGATTGTTGTCTAA

Blaine Creek AAAGTGTTTAGATTTTCATTTCAAAGAAACATAAAATAAAATTATGAGATTGTTGTCTAA

09-CS0040 AAAGTGTTTAGATTTTCATTTCAAAGAAACATAAAATAAAATTATGAGATTGTTGTCTAA

17CS1133 AAAGTGTTTAGATTTTCATTTCAAAGAAACATAAAATAAAATTATGAGATTGTTGTCTAA

Jasper AAAGTGTTTAGATTTTCATTTCAAAGAAACATAAAATAAAATTATGAGATTGTTGTCTAA

CN 120017 AAAGTGTTTAGATTTTCATTTCAAAGAAACATAAAATAAAATTATGAGATTGTTGTCTAA

CN 120027 AAAGTGTTTAGATTTTCATTTCAAAGAAACATAAAATAAAATTATGAGATTGTTGTCTAA

CN 120030 AAAGTGTTTAGATTTTCATTTCAAAGAAACATAAAATAAAATTATGAGATTGTTGTCTAA

Hoga AAAGTGTTTAGATTTTCATTTCAAAGAAACATAAAATAAAATTATGAGATTGTTGTCTAA

Yellowstone AAAGTGTTTAGATTTTCATTTCAAAGAAACATAAAATAAAATTATGAGATTGTTGTCTAA

CN 119294 AAAGTGTTTAGATTTTCATTTCAAAGAAACATAAAATAAAATTATGAGATTGTTGTCTAA

************************************************************

CN 120025 AGACTCGGTCAATGTATTTGGAGGTTGGATTTACGTATTGGTCATCTGCTTACCGGCCAC

CN 119205 AGACTCGGTCAATGTATTTGGAGTTTGGATTTACGTATTGGTCATCTGCTTACCGGCCAC

CN 119243 AGACTCGGTCAATGTATTTGGAGTTTGGATTTACGTATTGGTCATCTGCTTACCGGCCAC

Joelle NCBI AGACTCGGTCAATGTATTTGGAGTTTGGATTTACGTATTGGTCATCTGCTTACCGGCCAC

Joelle AAFC AGACTCGGTCAATGTATTTGGAGTTTGGATTTACGTATTGGTCATCTGCTTACCGGCCAC

Joelle phyto AGACTCGGTCAATGTATTTGGAGTTTGGATTTACGTATTGGTCATCTGCTTACCGGCCAC

DH55 ref genome AGACTCGGTCAATGTATTTGGAGTTTGGATTTACGTATTGGTCATCTGCTTACCGGCCAC

CN 119300 AGACTCGGTCAATGTATTTGGAGTTTGGATTTACGTATTGGTCATCTGCTTACCGGCCAC

CAM 241 AGACTCGGTCAATGTATTTGGAGTTTGGATTTACGTATTGGTCATCTGCTTACCGGCCAC

CN 113754 AGACTCGGTCAATGTATTTGGAGTTTGGATTTACGTATTGGTCATCTGCTTACCGGCCAC

CAM 236 AGACTCGGTCAATGTATTTGGAGTTTGGATTTACGTATTGGTCATCTGCTTACCGGCCAC

CO46 NCBI AGACTCGGTCAATGTATTTGGAGTTTGGATTTACGTATTGGTCATCTGCTTACCGGCCAC

CN 120013 AGACTCGGTCAATGTATTTGGAGTTTGGATTTACGTATTGGTCATCTGCTTACCGGCCAC

Blaine Creek AGACTCGGTCAATGTATTTGGAGTTTGGATTTACGTATTGGTCATCTGCTTACCGGCCAC

09-CS0040 AGACTCGGTCAATGTATTTGGAGTTTGGATTTACGTATTGGTCATCTGCTTACCGGCCAC

17CS1133 AGACTCGGTCAATGTATTTGGAGTTTGGATTTACGTATTGGTCATCTGCTTACCGGCCAC

Jasper AGACTCGGTCAATGTATTTGGAGTTTGGATTTACGTATTGGTCATCTGCTTACCGGCCAC

CN 120017 AGACTCGGTCAATGTATTTGGAGTTTGGATTTACGTATTGGTCATCTGCTTACCGGCCAC

CN 120027 AGACTCGGTCAATGTATTTGGAGTTTGGATTTACGTATTGGTCATCTGCTTACCGGCCAC

CN 120030 AGACTCGGTCAATGTATTTGGAGTTTGGATTTACGTATTGGTCATCTGCTTACCGGCCAC

Hoga AGACTCGGTCAATGTATTTGGAGTTTGGATTTACGTATTGGTCATCTGCTTACCGGCCAC

Yellowstone AGACTCGGTCAATGTATTTGGAGTTTGGATTTACGTATTGGTCATCTGCTTACCGGCCAC

CN 119294 AGACTCGGTCAATGTATTTGGAGTTTGGATTTACGTATTGGTCATCTGCTTACCGGCCAC

*********************** ************************************

CN 120025 ATCATCATCATCATGTTATGGCTTATCAATACTCCATTACGAGAAAGAACCTTGAGGTCA

CN 119205 ATCATCATCATCATGTTATGGCTTATCAATACTCCATTACCAGAAAGAACCTTGAGGTCA

CN 119243 ATCATCATCATCATGTTATGGCTTATCAATACTCCATTACCAGAAAGAACCTTGAGGTCA

Joelle NCBI ATCATCATCATCATGTTATGGCTTATCAATACTCCATTACCAGAAAGAACCTTGAGGTCA

Joelle AAFC ATCATCATCATCATGTTATGGCTTATCAATACTCCATTACCAGAAAGAACCTTGAGGTCA

Joelle phyto ATCATCATCATCATGTTATGGCTTATCAATACTCCATTACCAGAAAGAACCTTGAGGTCA

DH55 ref genome ATCATCATCATCATGTTATGGCTTATCAATACTCCATTACCAGAAAGAACCTTGAGGTCA

CN 119300 ATCATCATCATCATGTTATGGCTTATCAATACTCCATTACCAGAAAGAACCTTGAGGTCA

CAM 241 ATCATCATCATCATGTTATGGCTTATCAATACTCCATTACCAGAAAGAACCTTGAGGTCA

CN 113754 ATCATCATCATCATGTTATGGCTTATCAATACTCCATTACCAGAAAGAACCTTGAGGTCA

CAM 236 ATCATCATCATCATGTTATGGCTTATCAATACTCCATTACCAGAAAGAACCTTGAGGTCA

CO46 NCBI ATCATCATCATCATGTTATGGCTTATCAATACTCCATTACCAGAAAGAACCTTGAGGTCA

CN 120013 ATCATCATCATCATGTTATGGCTTATCAATACTCCATTACCAGAAAGAACCTTGAGGTCA

Blaine Creek ATCATCATCATCATGTTATGGCTTATCAATACTCCATTACCAGAAAGAACCTTGAGGTCA

09-CS0040 ATCATCATCATCATGTTATGGCTTATCAATACTCCATTACCAGAAAGAACCTTGAGGTCA

17CS1133 ATCATCATCATCATGTTATGGCTTATCAATACTCCATTACCAGAAAGAACCTTGAGGTCA

Jasper ATCATCATCATCATGTTATGGCTTATCAATACTCCATTACCAGAAAGAACCTTGAGGTCA

CN 120017 ATCATCATCATCATGTTATGGCTTATCAATACTCCATTACCAGAAAGAACCTTGAGGTCA

CN 120027 ATCATCATCATCATGTTATGGCTTATCAATACTCCATTACCAGAAAGAACCTTGAGGTCA

CN 120030 ATCATCATCATCATGTTATGGCTTATCAATACTCCATTACCAGAAAGAACCTTGAGGTCA

Hoga ATCATCATCATCATGTTATGGCTTATCAATACTCCATTACCAGAAAGAACCTTGAGGTCA

Yellowstone ATCATCATCATCATGTTATGGCTTATCAATACTCCATTACCAGAAAGAACCTTGAGGTCA

CN 119294 ATCATCATCATCATGTTATGGCTTATCAATACTCCATTACCAGAAAGAACCTTGAGGTCA

**************************************** *******************

CN 120025 AGGTTGATATATATGAAAGCTCAGAAAAGTTGTCTTGTATATATGTTCAGATGGTGTTAC

CN 119205 AGGTTGATATATATGAAAGCTCAAAAAAGTTGTCTTGTATATATGTTCAAATGGTGTTAC

CN 119243 AGGTTGATATATATGAAAGCTCAAAAAAGTTGTCTTGTATATATGTTCAGATGGTGTTAC

Joelle NCBI AGGTTGATATATATGAAAGCTCAAAAAAGTTGTCTTGTATATATGTTCAGATGGTGTTAC

Joelle AAFC AGGTTGATATATATGAAAGCTCAAAAAAGTTGTCTTGTATATATGTTCAGATGGTGTTAC

Joelle phyto AGGTTGATATATATGAAAGCTCAAAAAAGTTGTCTTGTATATATGTTCAGATGGTGTTAC

DH55 ref genome AGGTTGATATATATGAAAGCTCAAAAAAGTTGTCTTGTATATATGTTCAAATGGTGTTAC

CN 119300 AGGTTGATATATATGAAAGCTCAAAAAAGTTGTCTTGTATATATGTTCAAATGGTGTTAC

CAM 241 AGGTTGATATATATGAAAGCTCAAAAAAGTTGTCTTGTATATATGTTCAAATGGTGTTAC

CN 113754 AGGTTGATATATATGAAAGCTCAAAAAAGTTGTCTTGTATATATGTTCAAATGGTGTTAC

CAM 236 AGGTTGATATATATGAAAGCTCAAAAAAGTTGTCTTGTATATATGTTCAAATGGTGTTAC

CO46 NCBI AGGTTGATATATATGAAAGCTCAAAAAAGTTGTCTTGTATATATGTTCAAATGGTGTTAC

CN 120013 AGGTTGATATATATGAAAGCTCAAAAAAGTTGTCTTGTATATATGTTCAAATGGTGTTAC

Blaine Creek AGGTTGATATATATGAAAGCTCAAAAAAGTTGTCTTGTATATATGTTCAAATGGTGTTAC

09-CS0040 AGGTTGATATATATGAAAGCTCAAAAAAGTTGTCTTGTATATATGTTCAAATGGTGTTAC

17CS1133 AGGTTGATATATATGAAAGCTCAAAAAAGTTGTCTTGTATATATGTTCAAATGGTGTTAC

Jasper AGGTTGATATATATGAAAGCTCAAAAAAGTTGTCTTGTATATATGTTCAAATGGTGTTAC

CN 120017 AGGTTGATATATATGAAAGCTCAAAAAAGTTGTCTTGTATATATGTTCAAATGGTGTTAC

CN 120027 AGGTTGATATATATGAAAGCTCAAAAAAGTTGTCTTGTATATATGTTCAAATGGTGTTAC

CN 120030 AGGTTGATATATATGAAAGCTCAAAAAAGTTGTCTTGTATATATGTTCAAATGGTGTTAC

Hoga AGGTTGATATATATGAAAGCTCAAAAAAGTTGTCTTGTATATATGTTCAAATGGTGTTAC

Yellowstone AGGTTGATATATATGAAAGCTCAAAAAAGTTGTCTTGTATATATGTTCAAATGGTGTTAC

CN 119294 AGGTTGATATATATGAAAGCTCAAAAAAGTTGTCTTGTATATATGTTCAAATGGTGTTAC

*********************** ************************* **********

CN 120025 TTAGAAAACTAATAGTTGGTGTCACCCTTTTTGACACACGATTAGGCGTTTTCTTTTTAT

CN 119205 TTAGAAAACTAATAGTTGGTGTCACCCTTTTTGACACACGATTAGGCGTTTTCTTTTTAT

CN 119243 TTAGAAAACTAATAGTTGGTGTCACCCTTTTTGACACACGATTAGGCGTTTTCTTTTTAT

Joelle NCBI TTAGAAAACTAATAGTTGGTGTCACCCTTTTTGACACACGATTAGGCGTTTTCTTTTTAT

Joelle AAFC TTAGAAAACTAATAGTTGGTGTCACCCTTTTTGACACACGATTAGGCGTTTTCTTTTTAT

Joelle phyto TTAGAAAACTAATAGTTGGTGTCACCCTTTTTGACACACGATTAGGCGTTTTCTTTTTAT

DH55 ref genome TTAGAAAACTAATAGTTGGTGTCACCCTTTTTGACACACGATTAGGCGTTTTCTTTTTAT

CN 119300 TTAGAAAACTAATAGTTGGTGTCACCCTTTTTGACACACGATTAGGCGTTTTCTTTTTAT

CAM 241 TTAGAAAACTAATAGTTGGTGTCACCCTTTTTGACACACGATTAGGCGTTTTCTTTTTAT

CN 113754 TTAGAAAACTAATAGTTGGTGTCACCCTTTTTGACACACGATTAGGCGTTTTCTTTTTAT

CAM 236 TTAGAAAACTAATAGTTGGTGTCACCCTTTTTGACACACGATTAGGCGTTTTCTTTTTAT

CO46 NCBI TTAGAAAACTAATAGTTGGTGTCACCCTTTTTGACACACGATTAGGCGTTTTCTTTTTAT

CN 120013 TTAGAAAACTAATAGTTGGTGTCACCCTTTTTGACACACGATTAGGCGTTTTCTTTTTAT

Blaine Creek TTAGAAAACTAATAGTTGGTGTCACCCTTTTTGACACACGATTAGGCGTTTTCTTTTTAT

09-CS0040 TTAGAAAACTAATAGTTGGTGTCACCCTTTTTGACACACGATTAGGCGTTTTCTTTTTAT

17CS1133 TTAGAAAACTAATAGTTGGTGTCACCCTTTTTGACACACGATTAGGCGTTTTCTTTTTAT

Jasper TTAGAAAACTAATAGTTGGTGTCACCCTTTTTGACACACGATTAGGCGTTTTCTTTTTAT

CN 120017 TTAGAAAACTAATAGTTGGTGTCACCCTTTTTGACACACGATTAGGCGTTTTCTTTTTAT

CN 120027 TTAGAAAACTAATAGTTGGTGTCACCCTTTTTGACACACGATTAGGCGTTTTCTTTTTAT

CN 120030 TTAGAAAACTAATAGTTGGTGTCACCCTTTTTGACACACGATTAGGCGTTTTCTTTTTAT

Hoga TTAGAAAACTAATAGTTGGTGTCACCCTTTTTGACACACGATTAGGCGTTTTCTTTTTAT

Yellowstone TTAGAAAACTAATAGTTGGTGTCACCCTTTTTGACACACGATTAGGCGTTTTCTTTTTAT

CN 119294 TTAGAAAACTAATAGTTGGTGTCACCCTTTTTGACACACGATTAGGCGTTTTCTTTTTAT

************************************************************

CN 120025 AATGGTATTGATGCCACCATTTGTGGTTCAAATCACAGCCGAGACAAAAGAAAGAAGATA

CN 119205 AATGGTATTGATGCCACCATTTGTGGTTCAAATCACAGCCGAGACAAAAGAAAGAAGATA

CN 119243 AATGGTATTGATGCCACCATTTGTGGTTCAAATCACAGCCGAGACAAAAGAAAGAAGATA

Joelle NCBI AATGGTATTGATGCCACCATTTGTGGTTCAAATCACAGCCGAGACAAAAGAAAGAAGATA

Joelle AAFC AATGGTATTGATGCCACCATTTGTGGTTCAAATCACAGCCGAGACAAAAGAAAGAAGATA

Joelle phyto AATGGTATTGATGCCACCATTTGTGGTTCAAATCACAGCCGAGACAAAAGAAAGAAGATA

DH55 ref genome AATGGTATTGATGCCACCATTTGTGGTTCAAATCACAGCCGAGACAAAAGAAAGAAGATA

CN 119300 AATGGTATTGATGCCACCATTTGTGGTTCAAATCACAGCCGAGACAAAAGAAAGAAGATA

CAM 241 AATGGTATTGATGCCACCATTTGTGGTTCAAATCACAGCCGAGACAAAAGAAAGAAGATA

CN 113754 AATGGTATTGATGCCACCATTTGTGGTTCAAATCACAGCCGAGACAAAAGAAAGAAGATA

CAM 236 AATGGTATTGATGCCACCATTTGTGGTTCAAATCACAGCCGAGACAAAAGAAAGAAGATA

CO46 NCBI AATGGTATTGATGCCACCATTTGTGGTTCAAATCACAGCCGAGACAAAAGAAAGAAGATA

CN 120013 AATGGTATTGATGCCACCATTTGTGGTTCAAATCACAGCCGAGACAAAAGAAAGAAGATA

Blaine Creek AATGGTATTGATGCCACCATTTGTGGTTCAAATCACAGCCGAGACAAAAGAAAGAAGATA

09-CS0040 AATGGTATTGATGCCACCATTTGTGGTTCAAATCACAGCCGAGACAAAAGAAAGAAGATA

17CS1133 AATGGTATTGATGCCACCATTTGTGGTTCAAATCACAGCCGAGACAAAAGAAAGAAGATA

Jasper AATGGTATTGATGCCACCATTTGTGGTTCAAATCACAGCCGAGACAAAAGAAAGAAGATA

CN 120017 AATGGTATTGATGCCACCATTTGTGGTTCAAATCACAGCCGAGACAAAAGAAAGAAGATA

CN 120027 AATGGTATTGATGCCACCATTTGTGGTTCAAATCACAGCCGAGACAAAAGAAAGAAGATA

CN 120030 AATGGTATTGATGCCACCATTTGTGGTTCAAATCACAGCCGAGACAAAAGAAAGAAGATA

Hoga AATGGTATTGATGCCACCATTTGTGGTTCAAATCACAGCCGAGACAAAAGAAAGAAGATA

Yellowstone AATGGTATTGATGCCACCATTTGTGGTTCAAATCACAGCCGAGACAAAAGAAAGAAGATA

CN 119294 AATGGTATTGATGCCACCATTTGTGGTTCAAATCACAGCCGAGACAAAAGAAAGAAGATA

************************************************************

CN 120025 TATAGGGTGTTGTTTTGACTTTTGATGCTAATTGCGGTATGGATCGAATCCAAAAATGGA

CN 119205 TATAGGGTGTTGTTTTGACTTTTGATGCTAATTGCGGTATGGATCGAATCCAAAAATGGA

CN 119243 TATAGGGTGTTGTTTTGACTTTTGATGCTAATTGCGGTATGGATCGAATCCAAAAATGGA

Joelle NCBI TATAGGGTGTTGTTTTGACTTTTGATGCTAATTGCGGTATGGATCGAATCCAAAAATGGA

Joelle AAFC TATAGGGTGTTGTTTTGACTTTTGATGCTAATTGCGGTATGGATCGAATCCAAAAATGGA

Joelle phyto TATAGGGTGTTGTTTTGACTTTTGATGCTAATTGCGGTATGGATCGAATCCAAAAATGGA

DH55 ref genome TATAGGGTGTTGTTTTGACTTTTGATGCTAATTGCGGTATGGATCGAATCCAAAAATGGA

CN 119300 TATAGGGTGTTGTTTTGACTTTTGATGCTAATTGCGGTATGGATCGAATCCAAAAATGGA

CAM 241 TATAGGGTGTTGTTTTGACTTTTGATGCTAATTGCGGTATGGATCGAATCCAAAAATGGA

CN 113754 TATAGGGTGTTGTTTTGACTTTTGATGCTAATTGCGGTATGGATCGAATCCAAAAATGGA

CAM 236 TATAGGGTGTTGTTTTGACTTTTGATGCTAATTGCGGTATGGATCGAATCCAAAAATGGA

CO46 NCBI TATAGGGTGTTGTTTTGACTTTTGATGCTAATTGCGGTATGGATCGAATCCAAAAATGGA

CN 120013 TATAGGGTGTTGTTTTGACTTTTGATGCTAATTGCGGTATGGATCGAATCCAAAAATGGA

Blaine Creek TATAGGGTGTTGTTTTGACTTTTGATGCTAATTGCGGTATGGATCGAATCCAAAAATGGA

09-CS0040 TATAGGGTGTTGTTTTGACTTTTGATGCTAATTGCGGTATGGATCGAATCCAAAAATGGA

17CS1133 TATAGGGTGTTGTTTTGACTTTTGATGCTAATTGCGGTATGGATCGAATCCAAAAATGGA

Jasper TATAGGGTGTTGTTTTGACTTTTGATGCTAATTGCGGTATGGATCGAATCCAAAAATGGA

CN 120017 TATAGGGTGTTGTTTTGACTTTTGATGCTAATTGCGGTATGGATCGAATCCAAAAATGGA

CN 120027 TATAGGGTGTTGTTTTGACTTTTGATGCTAATTGCGGTATGGATCGAATCCAAAAATGGA

CN 120030 TATAGGGTGTTGTTTTGACTTTTGATGCTAATTGCGGTATGGATCGAATCCAAAAATGGA

Hoga TATAGGGTGTTGTTTTGACTTTTGATGCTAATTGCGGTATGGATCGAATCCAAAAATGGA

Yellowstone TATAGGGTGTTGTTTTGACTTTTGATGCTAATTGCGGTATGGATCGAATCCAAAAATGGA

CN 119294 TATAGGGTGTTGTTTTGACTTTTGATGCTAATTGCGGTATGGATCGAATCCAAAAATGGA

************************************************************

CN 120025 AGATCAGATAGAGGTTACATACAATCAGAATAATGTAAATGAATGAATTGGAAGCAGTCT

CN 119205 AGATCAGATAGAGGTTACATACAATCAGAATAATGTAAATGAATGAATTGGAAGCAGTCT

CN 119243 AGATCAGATAGAGGTTACATACAATCAGAATAATGTAAATGAATGAATTGGAAGCAGTCT

Joelle NCBI AGATCAGATAGAGGTTACATACAATCAGAATAATGTAAATGAATGAATTGGAAGCAGTCT

Joelle AAFC AGATCAGATAGAGGTTACATACAATCAGAATAATGTAAATGAATGAATTGGAAGCAGTCT

Joelle phyto AGATCAGATAGAGGTTACATACAATCAGAATAATGTAAATGAATGAATTGGAAGCAGTCT

DH55 ref genome AGATCAGATAGAGGTTACATACAATCAGAATAATGTAAATGAATGAATTGGAAGCAGTCT

CN 119300 AGATCAGATAGAGGTTACATACAATCAGAATAATGTAAATGAATGAATTGGAAGCAGTCT

CAM 241 AGATCAGATAGAGGTTACATACAATCAGAATAATGTAAATGAATGAATTGGAAGCAGTCT

CN 113754 AGATCAGATAGAGGTTACATACAATCAGAATAATGTAAATGAATGAATTGGAAGCAGTCT

CAM 236 AGATCAGATAGAGGTTACATACAATCAGAATAATGTAAATGAATGAATTGGAAGCAGTCT

CO46 NCBI AGATCAGATAGAGGTTACATACAATCAGAATAATGTAAATGAATGAATTGGAAGCAGTCT

CN 120013 AGATCAGATAGAGGTTACATACAATCAGAATAATGTAAATGAATGAATTGGAAGCAGTCT

Blaine Creek AGATCAGATAGAGGTTACATACAATCAGAATAATGTAAATGAATGAATTGGAAGCAGTCT

09-CS0040 AGATCAGATAGAGGTTACATACAATCAGAATAATGTAAATGAATGAATTGGAAGCAGTCT

17CS1133 AGATCAGATAGAGGTTACATACAATCAGAATAATGTAAATGAATGAATTGGAAGCAGTCT

Jasper AGATCAGATAGAGGTTACATACAATCAGAATAATGTAAATGAATGAATTGGAAGCAGTCT

CN 120017 AGATCAGATAGAGGTTACATACAATCAGAATAATGTAAATGAATGAATTGGAAGCAGTCT

CN 120027 AGATCAGATAGAGGTTACATACAATCAGAATAATGTAAATGAATGAATTGGAAGCAGTCT

CN 120030 AGATCAGATAGAGGTTACATACAATCAGAATAATGTAAATGAATGAATTGGAAGCAGTCT

Hoga AGATCAGATAGAGGTTACATACAATCAGAATAATGTAAATGAATGAATTGGAAGCAGTCT

Yellowstone AGATCAGATAGAGGTTACATACAATCAGAATAATGTAAATGAATGAATTGGAAGCAGTCT

CN 119294 AGATCAGATAGAGGTTACATACAATCAGAATAATGTAAATGAATGAATTGGAAGCAGTCT

************************************************************

CN 120025 TCCACTGTTTCCTATGTTTAGGGTTGTCTTTTAACTATGTGCCGAAATTATAAATAAAAA

CN 119205 TCCACTGTTTCCTATGTTTAGGGTTGTCTTTTAACTATGTGCCGAAATTATAAATAAAAA

CN 119243 TCCACTGTTTCCTATGTTTAGGGTTGTCTTTTAACTATGTGCCGAAATTATAAATAAAAA

Joelle NCBI TCCACTGTTTCCTATGTTTAGGGTTGTCTTTTAACTATGTGCCGAAATTATAAATAAAAA

Joelle AAFC TCCACTGTTTCCTATGTTTAGGGTTGTCTTTTAACTATGTGCCGAAATTATAAATAAAAA

Joelle phyto TCCACTGTTTCCTATGTTTAGGGTTGTCTTTTAACTATGTGCCGAAATTATAAATAAAAA

DH55 ref genome TCCACTGTTTCCTATGTTTAGGGTTGTCTTTTAACTATGTGCCGAAATTATAAATAAAAA

CN 119300 TCCACTGTTTCCTATGTTTAGGGTTGTCTTTTAACTATGTGCCGAAATTATAAATAAAAA

CAM 241 TCCACTGTTTCCTATGTTTAGGGTTGTCTTTTAACTATGTGCCGAAATTATAAATAAAAA

CN 113754 TCCACTGTTTCCTATGTTTAGGGTTGTCTTTTAACTATGTGCCGAAATTATAAATAAAAA

CAM 236 TCCACTGTTTCCTATGTTTAGGGTTGTCTTTTAACTATGTGCCGAAATTATAAATAAAAA

CO46 NCBI TCCACTGTTTCCTATGTTTAGGGTTGTCTTTTAACTATGTGCCGAAATTATAAATAAAAA

CN 120013 TCCACTGTTTCCTATGTTTAGGGTTGTCTTTTAACTATGTGCCGAAATTATAAATAAAAA

Blaine Creek TCCACTGTTTCCTATGTTTAGGGTTGTCTTTTAACTATGTGCCGAAATTATAAATAAAAA

09-CS0040 TCCACTGTTTCCTATGTTTAGGGTTGTCTTTTAACTATGTGCCGAAATTATAAATAAAAA

17CS1133 TCCACTGTTTCCTATGTTTAGGGTTGTCTTTTAACTATGTGCCGAAATTATAAATAAAAA

Jasper TCCACTGTTTCCTATGTTTAGGGTTGTCTTTTAACTATGTGCCGAAATTATAAATAAAAA

CN 120017 TCCACTGTTTCCTATGTTTAGGGTTGTCTTTTAACTATGTGCCGAAATTATAAATAAAAA

CN 120027 TCCACTGTTTCCTATGTTTAGGGTTGTCTTTTAACTATGTGCCGAAATTATAAATAAAAA

CN 120030 TCCACTGTTTCCTATGTTTAGGGTTGTCTTTTAACTATGTGCCGAAATTATAAATAAAAA

Hoga TCCACTGTTTCCTATGTTTAGGGTTGTCTTTTAACTATGTGCCGAAATTATAAATAAAAA

Yellowstone TCCACTGTTTCCTATGTTTAGGGTTGTCTTTTAACTATGTGCCGAAATTATAAATAAAAA

CN 119294 TCCACTGTTTCCTATGTTTAGGGTTGTCTTTTAACTATGTGCCGAAATTATAAATAAAAA

************************************************************

CN 120025 TGGCTTTCTGTTAGACTCAGTCCATGTCCTTGGAATTTGGCTTAAACATAGGTATATTGG

CN 119205 TGGCTTTCTGTAAGACTCAGTCCATGTCCTTGGAATTTGGCTTAAACATAGGTATATTGG

CN 119243 TGGCTTTCTGTAAGACTCAGTCCATGTCCTTGGAATTTGGCTTAAACATAGGTATATTGG

Joelle NCBI TGGCTTTCTGTAAGACTCAGTCCATGTCCTTGGAATTTGGCTTAAACATAGGTATATTGG

Joelle AAFC TGGCTTTCTGTAAGACTCAGTCCATGTCCTTGGAATTTGGCTTAAACATAGGTATATTGG

Joelle phyto TGGCTTTCTGTAAGACTCAGTCCATGTCCTTGGAATTTGGCTTAAACATAGGTATATTGG

DH55 ref genome TGGCTTTCTGTAAGACTCAGTCCATGTCCTTGGAATTTGGCTTAAACATAGGTATATTGG

CN 119300 TGGCTTTCTGTAAGACTCAGTCCATGTCCTTGGAATTTGGCTTAAACATAGGTATATTGG

CAM 241 TGGCTTTCTGTAAGACTCAGTCCATGTCCTTGGAATTTGGCTTAAACATAGGTATATTGG

CN 113754 TGGCTTTCTGTAAGACTCAGTCCATGTCCTTGGAATTTGGCTTAAACATAGGTATATTGG

CAM 236 TGGCTTTCTGTAAGACTCAGTCCATGTCCTTGGAATTTGGCTTAAACATAGGTATATTGG

CO46 NCBI TGGCTTTCTGTAAGACTCAGTCCATGTCCTTGGAATTTGGCTTAAACATAGGTATATTGG

CN 120013 TGGCTTTCTGTAAGACTCAGTCCATGTCCTTGGAATTTGGCTTAAACATAGGTATATTGG

Blaine Creek TGGCTTTCTGTAAGACTCAGTCCATGTCCTTGGAATTTGGCTTAAACATAGGTATATTGG

09-CS0040 TGGCTTTCTGTAAGACTCAGTCCATGTCCTTGGAATTTGGCTTAAACATAGGTATATTGG

17CS1133 TGGCTTTCTGTAAGACTCAGTCCATGTCCTTGGAATTTGGCTTAAACATAGGTATATTGG

Jasper TGGCTTTCTGTAAGACTCAGTCCATGTCCTTGGAATTTGGCTTAAACATAGGTATATTGG

CN 120017 TGGCTTTCTGTAAGACTCAGTCCATGTCCTTGGAATTTGGCTTAAACATAGGTATATTGG

CN 120027 TGGCTTTCTGTAAGACTCAGTCCATGTCCTTGGAATTTGGCTTAAACATAGGTATATTGG

CN 120030 TGGCTTTCTGTAAGACTCAGTCCATGTCCTTGGAATTTGGCTTAAACATAGGTATATTGG

Hoga TGGCTTTCTGTAAGACTCAGTCCATGTCCTTGGAATTTGGCTTAAACATAGGTATATTGG

Yellowstone TGGCTTTCTGTAAGACTCAGTCCATGTCCTTGGAATTTGGCTTAAACATAGGTATATTGG

CN 119294 TGGCTTTCTGTAAGACTCAGTCCATGTCCTTGGAATTTGGCTTAAACATAGGTATATTGG

*********** ************************************************

CN 120025 ACGTCTGCTTACTGCCATGTTATCACGTTGTGGTTCATCAATATATGTGTCTACGTTTTC

CN 119205 ACGTCTGCTTACTGCCATGTTATCACGTTGTGGTTCATCAATATATGTGTCTACGTTTTC

CN 119243 ACGTCTGCTTACTGCCATGTTATCACGTTGTGGTTCATCAATATATGTGTCTACGTTTTC

Joelle NCBI ACGTCTGCTTACTGCCATGTTATCACGTTGTGGTTCATCAATATATGTGTCTACGTTTTC

Joelle AAFC ACGTCTGCTTACTGCCATGTTATCACGTTGTGGTTCATCAATATATGTGTCTACGTTTTC

Joelle phyto ACGTCTGCTTACTGCCATGTTATCACGTTGTGGTTCATCAATATATGTGTCTACGTTTTC

DH55 ref genome ACGTCTGCTTACTGCCATGTTATCACGTTGTGGTTCATCAATATATGTGTCTACGTTTTC

CN 119300 ACGTCTGCTTACTGCCATGTTATCACGTTGTGGTTCATCAATATATGTGTCTACGTTTTC

CAM 241 ACGTCTGCTTACTGCCATGTTATCACGTTGTGGTTCATCAATATATGTGTCTACGTTTTC

CN 113754 ACGTCTGCTTACTGCCATGTTATCACGTTGTGGTTCATCAATATATGTGTCTACGTTTTC

CAM 236 ACGTCTGCTTACTGCCATGTTATCACGTTGTGGTTCATCAATATATGTGTCTACGTTTTC

CO46 NCBI ACGTCTGCTTACTGCCATGTTATCACGTTGTGGTTCATCAATATATGTGTCTACGTTTTC

CN 120013 ACGTCTGCTTACTGCCATGTTATCACGTTGTGGTTCATCAATATATGTGTCTACGTTTTC

Blaine Creek ACGTCTGCTTACTGCCATGTTATCACGTTGTGGTTCATCAATATATGTGTCTACGTTTTC

09-CS0040 ACGTCTGCTTACTGCCATGTTATCACGTTGTGGTTCATCAATATATGTGTCTACGTTTTC

17CS1133 ACGTCTGCTTACTGCCATGTTATCACGTTGTGGTTCATCAATATATGTGTCTACGTTTTC

Jasper ACGTCTGCTTACTGCCATGTTATCACGTTGTGGTTCATCAATATATGTGTCTACGTTTTC

CN 120017 ACGTCTGCTTACTGCCATGTTATCACGTTGTGGTTCATCAATATATGTGTCTACGTTTTC

CN 120027 ACGTCTGCTTACTGCCATGTTATCACGTTGTGGTTCATCAATATATGTGTCTACGTTTTC

CN 120030 ACGTCTGCTTACTGCCATGTTATCACGTTGTGGTTCATCAATATATGTGTCTACGTTTTC

Hoga ACGTCTGCTTACTGCCATGTTATCACGTTGTGGTTCATCAATATATGTGTCTACGTTTTC

Yellowstone ACGTCTGCTTACTGCCATGTTATCACGTTGTGGTTCATCAATATATGTGTCTACGTTTTC

CN 119294 ACGTCTGCTTACTGCCATGTTATCACGTTGTGGTTCATCAATATATGTGTCTACGTTTTC

************************************************************

CN 120025 GTGAGTATATTTTTTCTTTTAACAGT-AAAAGTCTGTGTGTATTGTACACTCTCTTGAGC

CN 119205 GTGAGTATATTTTTTCTTTTAACAGTAAAAAGTCTGTGTGTATTGTACACTCTCTTGAGC

CN 119243 GTGAGTATATTTTTTCTTTTAACAGTAAAAAGTCTGTGTGTATTGTACACTCTCTTGAGC

Joelle NCBI GTGAGTATATTTTTTCTTTTAACAGTAAAAAGTCTGTGTGTATTGTACACTCTCTTGAGC

Joelle AAFC GTGAGTATATTTTTTCTTTTAACAGTAAAAAGTCTGTGTGTATTGTACACTCTCTTGAGC

Joelle phyto GTGAGTATATTTTTTCTTTTAACAGTAAAAAGTCTGTGTGTATTGTACACTCTCTTGAGC

DH55 ref genome GTGAGTATATTTTTTCTTTTAACAGTAAAAAGTCTGTGTGTATTGTACACTCTCTTGAGC

CN 119300 GTGAGTATATTTTTTCTTTTAACAGTAAAAAGTCTGTGTGTATTGTACACTCTCTTGAGC

CAM 241 GTGAGTATATTTTTTCTTTTAACAGTAAAAAGTCTGTGTGTATTGTACACTCTCTTGAGC

CN 113754 GTGAGTATATTTTTTCTTTTAACAGTAAAAAGTCTGTGTGTATTGTACACTCTCTTGAGC

CAM 236 GTGAGTATATTTTTTCTTTTAACAGTAAAAAGTCTGTGTGTATTGTACACTCTCTTGAGC

CO46 NCBI GTGAGTATATTTTTTCTTTTAACAGTAAAAAGTCTGTGTGTATTGTACACTCTCTTGAGC

CN 120013 GTGAGTATATTTTTTCTTTTAACAGTAAAAAGTCTGTGTGTATTGTACACTCTCTTGAGC

Blaine Creek GTGAGTATATTTTTTCTTTTAACAGTAAAAAGTCTGTGTGTATTGTACACTCTCTTGAGC

09-CS0040 GTGAGTATATTTTTTCTTTTAACAGTAAAAAGTCTGTGTGTATTGTACACTCTCTTGAGC

17CS1133 GTGAGTATATTTTTTCTTTTAACAGTAAAAAGTCTGTGTGTATTGTACACTCTCTTGAGC

Jasper GTGAGTATATTTTTTCTTTTAACAGTAAAAAGTCTGTGTGTATTGTACACTCTCTTGAGC

CN 120017 GTGAGTATATTTTTTCTTTTAACAGTAAAAAGTCTGTGTGTATTGTACACTCTCTTGAGC

CN 120027 GTGAGTATATTTTTTCTTTTAACAGTAAAAAGTCTGTGTGTATTGTACACTCTCTTGAGC

CN 120030 GTGAGTATATTTTTTCTTTTAACAGTAAAAAGTCTGTGTGTATTGTACACTCTCTTGAGC

Hoga GTGAGTATATTTTTTCTTTTAACAGTAAAAAGTCTGTGTGTATTGTACACTCTCTTGAGC

Yellowstone GTGAGTATATTTTTTCTTTTAACAGTAAAAAGTCTGTGTGTATTGTACACTCTCTTGAGC

CN 119294 GTGAGTATATTTTTTCTTTTAACAGTAAAAAGTCTGTGTGTATTGTACACTCTCTTGAGC

************************** *********************************

CN 120025 CTCAATTGCTTGTTTGCATTTAAGTTTTCTGCTGTGTTTCCATATTATATTTTATCAATC

CN 119205 CTCAATTGCTTGTTTGCATTTAAGTTTTCTGCTGTGTTTCCATATTATATTTTATCAATC

CN 119243 CTCAATTGCTTGTTTGCATTTAAGTTTTCTGCTGTGTTTCCATATTATATTTTATCAATC

Joelle NCBI CTCAATTGCTTGTTTGCATTTAAGTTTTCTGCTGTGTTTCCATATTATATTTTATCAATC

Joelle AAFC CTCAATTGCTTGTTTGCATTTAAGTTTTCTGCTGTGTTTCCATATTATATTTTATCAATC

Joelle phyto CTCAATTGCTTGTTTGCATTTAAGTTTTCTGCTGTGTTTCCATATTATATTTTATCAATC

DH55 ref genome CTCAATTGCTTGTTTGCATTTAAGTTTTCTGCTGTGTTTCCATATTATATTTTATCAATC

CN 119300 CTCAATTGCTTGTTTGCATTTAAGTTTTCTGCTGTGTTTCCATATTATATTTTATCAATC

CAM 241 CTCAATTGCTTGTTTGCATTTAAGTTTTCTGCTGTGTTTCCATATTATATTTTATCAATC

CN 113754 CTCAATTGCTTGTTTGCATTTAAGTTTTCTGCTGTGTTTCCATATTATATTTTATCAATC

CAM 236 CTCAATTGCTTGTTTGCATTTAAGTTTTCTGCTGTGTTTCCATATTATATTTTATCAATC

CO46 NCBI CTCAATTGCTTGTTTGCATTTAAGTTTTCTGCTGTGTTTCCATATTATATTTTATCAATC

CN 120013 CTCAATTGCTTGTTTGCATTTAAGTTTTCTGCTGTGTTTCCATATTATATTTTATCAATC

Blaine Creek CTCAATTGCTTGTTTGCATTTAAGTTTTCTGCTGTGTTTCCATATTATATTTTATCAATC

09-CS0040 CTCAATTGCTTGTTTGCATTTAAGTTTTCTGCTGTGTTTCCATATTATATTTTATCAATC

17CS1133 CTCAATTGCTTGTTTGCATTTAAGTTTTCTGCTGTGTTTCCATATTATATTTTATCAATC

Jasper CTCAATTGCTTGTTTGCATTTAAGTTTTCTGCTGTGTTTCCATATTATATTTTATCAATC

CN 120017 CTCAATTGCTTGTTTGCATTTAAGTTTTCTGCTGTGTTTCCATATTATATTTTATCAATC

CN 120027 CTCAATTGCTTGTTTGCATTTAAGTTTTCTGCTGTGTTTCCATATTATATTTTATCAATC

CN 120030 CTCAATTGCTTGTTTGCATTTAAGTTTTCTGCTGTGTTTCCATATTATATTTTATCAATC

Hoga CTCAATTGCTTGTTTGCATTTAAGTTTTCTGCTGTGTTTCCATATTATATTTTATCAATC

Yellowstone CTCAATTGCTTGTTTGCATTTAAGTTTTCTGCTGTGTTTCCATATTATATTTTATCAATC

CN 119294 CTCAATTGCTTGTTTGCATTTAAGTTTTCTGCTGTGTTTCCATATTATATTTTATCAATC

************************************************************

CN 120025 AGTGTACCATATAGAGAAGCACAAACAAATTGCATAGAAACAATCTGGACAGTGGACGCT

CN 119205 AGTGTACCATATAGAGAAGCACAAACAAATTGCATAGAAACAATCTGGACAGTGGACGCT

CN 119243 AGTGTACCATATAGAGAAGCACAAACAAATTGCATAGAAACAATCTGGACAGTGGACGCT

Joelle NCBI AGTGTACCATATAGAGAAGCACAAACAAATTGCATAGAAACAATCTGGACAGTGGACGCT

Joelle AAFC AGTGTACCATATAGAGAAGCACAAACAAATTGCATAGAAACAATCTGGACAGTGGACGCT

Joelle phyto AGTGTACCATATAGAGAAGCACAAACAAATTGCATAGAAACAATCTGGACAGTGGACGCT

DH55 ref genome AGTGTACCATATAGAGAAGCACAAACAAATTGCATAGAAACAATCTGGACAGTGGACGCT

CN 119300 AGTGTACCATATAGAGAAGCACAAACAAATTGCATAGAAACAATCTGGACAGTGGACGCT

CAM 241 AGTGTACCATATAGAGAAGCACAAACAAATTGCATAGAAACAATCTGGACAGTGGACGCT

CN 113754 AGTGTACCATATAGAGAAGCACAAACAAATTGCATAGAAACAATCTGGACAGTGGACGCT

CAM 236 AGTGTACCATATAGAGAAGCACAAACAAATTGCATAGAAACAATCTGGACAGTGGACGCT

CO46 NCBI AGTGTACCATATAGAGAAGCACAAACAAATTGCATAGAAACAATCTGGACAGTGGACGCT

CN 120013 AGTGTACCATATAGAGAAGCACAAACAAATTGCATAGAAACAATCTGGACAGTGGACGCT

Blaine Creek AGTGTACCATATAGAGAAGCACAAACAAATTGCATAGAAACAATCTGGACAGTGGACGCT

09-CS0040 AGTGTACCATATAGAGAAGCACAAACAAATTGCATAGAAACAATCTGGACAGTGGACGCT

17CS1133 AGTGTACCATATAGAGAAGCACAAACAAATTGCATAGAAACAATCTGGACAGTGGACGCT

Jasper AGTGTACCATATAGAGAAGCACAAACAAATTGCATAGAAACAATCTGGACAGTGGACGCT

CN 120017 AGTGTACCATATAGAGAAGCACAAACAAATTGCATAGAAACAATCTGGACAGTGGACGCT

CN 120027 AGTGTACCATATAGAGAAGCACAAACAAATTGCATAGAAACAATCTGGACAGTGGACGCT

CN 120030 AGTGTACCATATAGAGAAGCACAAACAAATTGCATAGAAACAATCTGGACAGTGGACGCT

Hoga AGTGTACCATATAGAGAAGCACAAACAAATTGCATAGAAACAATCTGGACAGTGGACGCT

Yellowstone AGTGTACCATATAGAGAAGCACAAACAAATTGCATAGAAACAATCTGGACAGTGGACGCT

CN 119294 AGTGTACCATATAGAGAAGCACAAACAAATTGCATAGAAACAATCTGGACAGTGGACGCT

************************************************************

CN 120025 TGAGATTAGGGTTTTCTGTAAACGAATTGTTAGATCACAGGGATAATCTATATACATGCC

CN 119205 TGAGATTAGGGTTTTCTGTAAACGAATTGTTAGATCACAGGGATAATCTATATACATGCC

CN 119243 TGAGATTAGGGTTTTCTGTAAACGAATTGTTAGATCACAGGGATAATCTATATACATGCC

Joelle NCBI TGAGATTAGGGTTTTCTGTAAACGAATTGTTAGATCACAGGGATAATCTATATACATGCC

Joelle AAFC TGAGATTAGGGTTTTCTGTAAACGAATTGTTAGATCACAGGGATAATCTATATACATGCC

Joelle phyto TGAGATTAGGGTTTTCTGTAAACGAATTGTTAGATCACAGGGATAATCTATATACATGCC

DH55 ref genome TGAGATTAGGGTTTTCTGTAAACGAATTGTTAGATCACAGGGATAATCTATATACATGCC

CN 119300 TGAGATTAGGGTTTTCTGTAAACGAATTGTTAGATCACAGGGATAATCTATATACATGCC

CAM 241 TGAGATTAGGGTTTTCTGTAAACGAATTGTTAGATCACAGGGATAATCTATATACATGCC

CN 113754 TGAGATTAGGGTTTTCTGTAAACGAATTGTTAGATCACAGGGATAATCTATATACATGCC

CAM 236 TGAGATTAGGGTTTTCTGTAAACGAATTGTTAGATCACAGGGATAATCTATATACATGCC

CO46 NCBI TGAGATTAGGGTTTTCTGTAAACGAATTGTTAGATCACAGGGATAATCTATATACATGCC

CN 120013 TGAGATTAGGGTTTTCTGTAAACGAATTGTTAGATCACAGGGATAATCTATATACATGCC

Blaine Creek TGAGATTAGGGTTTTCTGTAAACGAATTGTTAGATCACAGGGATAATCTATATACATGCC

09-CS0040 TGAGATTAGGGTTTTCTGTAAACGAATTGTTAGATCACAGGGATAATCTATATACATGCC

17CS1133 TGAGATTAGGGTTTTCTGTAAACGAATTGTTAGATCACAGGGATAATCTATATACATGCC

Jasper TGAGATTAGGGTTTTCTGTAAACGAATTGTTAGATCACAGGGATAATCTATATACATGCC

CN 120017 TGAGATTAGGGTTTTCTGTAAACGAATTGTTAGATCACAGGGATAATCTATATACATGCC

CN 120027 TGAGATTAGGGTTTTCTGTAAACGAATTGTTAGATCACAGGGATAATCTATATACATGCC

CN 120030 TGAGATTAGGGTTTTCTGTAAACGAATTGTTAGATCACAGGGATAATCTATATACATGCC

Hoga TGAGATTAGGGTTTTCTGTAAACGAATTGTTAGATCACAGGGATAATCTATATACATGCC

Yellowstone TGAGATTAGGGTTTTCTGTAAACGAATTGTTAGATCACAGGGATAATCTATATACATGCC

CN 119294 TGAGATTAGGGTTTTCTGTAAACGAATTGTTAGATCACAGGGATAATCTATATACATGCC

************************************************************

CN 120025 TTAATTATAGTAAGCCTTTTCTTTGTGGAAAAATGTTCAATTTCAACCTACCGAAAAATA

CN 119205 TTAATTATAGTAAGCCTTTTCTTTGTGGAAAAATGTTCAATTTCAACCTACCGAAAAATA

CN 119243 TTAATTATAGTAAGCCTTTTCTTTGTGGAAAAATGTTCAATTTCAACCTACCGAAAAATA

Joelle NCBI TTAATTATAGTAAGCCTTTTCTTTGTGGAAAAATGTTCAATTTCAACCTACCGAAAAATA

Joelle AAFC TTAATTATAGTAAGCCTTTTCTTTGTGGAAAAATGTTCAATTTCAACCTACCGAAAAATA

Joelle phyto TTAATTATAGTAAGCCTTTTCTTTGTGGAAAAATGTTCAATTTCAACCTACCGAAAAATA

DH55 ref genome TTAATTATAGTAAGCCTTTTCTTTGTGGAAAAATGTTCAATTTCAACCTACCGAAAAATA

CN 119300 TTAATTATAGTAAGCCTTTTCTTTGTGGAAAAATGTTCAATTTCAACCTACCGAAAAATA

CAM 241 TTAATTATAGTAAGCCTTTTCTTTGTGGAAAAATGTTCAATTTCAACCTACCGAAAAATA

CN 113754 TTAATTATAGTAAGCCTTTTCTTTGTGGAAAAATGTTCAATTTCAACCTACCGAAAAATA

CAM 236 TTAATTATAGTAAGCCTTTTCTTTGTGGAAAAATGTTCAATTTCAACCTACCGAAAAATA

CO46 NCBI TTAATTATAGTAAGCCTTTTCTTTGTGGAAAAATGTTCAATTTCAACCTACCGAAAAATA

CN 120013 TTAATTATAGTAAGCCTTTTCTTTGTGGAAAAATGTTCAATTTCAACCTACCGAAAAATA

Blaine Creek TTAATTATAGTAAGCCTTTTCTTTGTGGAAAAATGTTCAATTTCAACCTACCGAAAAATA

09-CS0040 TTAATTATAGTAAGCCTTTTCTTTGTGGAAAAATGTTCAATTTCAACCTACCGAAAAATA

17CS1133 TTAATTATAGTAAGCCTTTTCTTTGTGGAAAAATGTTCAATTTCAACCTACCGAAAAATA

Jasper TTAATTATAGTAAGCCTTTTCTTTGTGGAAAAATGTTCAATTTCAACCTACCGAAAAATA

CN 120017 TTAATTATAGTAAGCCTTTTCTTTGTGGAAAAATGTTCAATTTCAACCTACCGAAAAATA

CN 120027 TTAATTATAGTAAGCCTTTTCTTTGTGGAAAAATGTTCAATTTCAACCTACCGAAAAATA

CN 120030 TTAATTATAGTAAGCCTTTTCTTTGTGGAAAAATGTTCAATTTCAACCTACCGAAAAATA

Hoga TTAATTATAGTAAGCCTTTTCTTTGTGGAAAAATGTTCAATTTCAACCTACCGAAAAATA

Yellowstone TTAATTATAGTAAGCCTTTTCTTTGTGGAAAAATGTTCAATTTCAACCTACCGAAAAATA

CN 119294 TTAATTATAGTAAGCCTTTTCTTTGTGGAAAAATGTTCAATTTCAACCTACCGAAAAATA

************************************************************

CN 120025 TATAATAATAATTCATTGGATAATCAAACTTTGAACATTTCTTGGAAAATTTTTAGAGTT

CN 119205 TATAATAATAATTCATTGGATAATCAAACTTTGAACATTTCTTGGAAAATTTTTAGAGTT

CN 119243 TATAATAATAATTCATTGGATAATCAAACTTTGAACATTTCTTGGAAAATTTTTAGAGTT

Joelle NCBI TATAATAATAATTCATTGGATAATCAAACTTTGAACATTTCTTGGAAAATTTTTAGAGTT

Joelle AAFC TATAATAATAATTCATTGGATAATCAAACTTTGAACATTTCTTGGAAAATTTTTAGAGTT

Joelle phyto TATAATAATAATTCATTGGATAATCAAACTTTGAACATTTCTTGGAAAATTTTTAGAGTT

DH55 ref genome TATAATAATAATTCATTGGATAATCAAACTTTGAACATTTCTTGGAAAATTTTTAGAGTT

CN 119300 TATAATAATAATTCATTGGATAATCAAACTTTGAACATTTCTTGGAAAATTTTTAGAGTT

CAM 241 TATAATAATAATTCATTGGATAATCAAACTTTGAACATTTCTTGGAAAATTTTTAGAGTT

CN 113754 TATAATAATAATTCATTGGATAATCAAACTTTGAACATTTCTTGGAAAATTTTTAGAGTT

CAM 236 TATAATAATAATTCATTGGATAATCAAACTTTGAACATTTCTTGGAAAATTTTTAGAGTT

CO46 NCBI TATAATAATAATTCATTGGATAATCAAACTTTGAACATTTCTTGGAAAATTTTTAGAGTT

CN 120013 TATAATAATAATTCATTGGATAATCAAACTTTGAACATTTCTTGGAAAATTTTTAGAGTT

Blaine Creek TATAATAATAATTCATTGGATAATCAAACTTTGAACATTTCTTGGAAAATTTTTAGAGTT

09-CS0040 TATAATAATAATTCATTGGATAATCAAACTTTGAACATTTCTTGGAAAATTTTTAGAGTT

17CS1133 TATAATAATAATTCATTGGATAATCAAACTTTGAACATTTCTTGGAAAATTTTTAGAGTT

Jasper TATAATAATAATTCATTGGATAATCAAACTTTGAACATTTCTTGGAAAATTTTTAGAGTT

CN 120017 TATAATAATAATTCATTGGATAATCAAACTTTGAACATTTCTTGGAAAATTTTTAGAGTT

CN 120027 TATAATAATAATTCATTGGATAATCAAACTTTGAACATTTCTTGGAAAATTTTTAGAGTT

CN 120030 TATAATAATAATTCATTGGATAATCAAACTTTGAACATTTCTTGGAAAATTTTTAGAGTT

Hoga TATAATAATAATTCATTGGATAATCAAACTTTGAACATTTCTTGGAAAATTTTTAGAGTT

Yellowstone TATAATAATAATTCATTGGATAATCAAACTTTGAACATTTCTTGGAAAATTTTTAGAGTT

CN 119294 TATAATAATAATTCATTGGATAATCAAACTTTGAACATTTCTTGGAAAATTTTTAGAGTT

************************************************************

CN 120025 CTTTTTTTTAGAGATGAAAAGATCTATATATGTGTAATTATTAAGGTTTTGTCTCTAAAT

CN 119205 CTTTTTTTTAGAGATGAAAAGATCTATATATGTGTAATTATTAAGGTTTTGTCTCTAAAT

CN 119243 CTTTTTTTTAGAGATGAAAAGATCTATATATGTGTAATTATTAAGGTTTTGTCTCTAAAT

Joelle NCBI CTTTTTTTTAGAGATGAAAAGATCTATATATGTGTAATTATTAAGGTTTTGTCTCTAAAT

Joelle AAFC CTTTTTTTTAGAGATGAAAAGATCTATATATGTGTAATTATTAAGGTTTTGTCTCTAAAT

Joelle phyto CTTTTTTTTAGAGATGAAAAGATCTATATATGTGTAATTATTAAGGTTTTGTCTCTAAAT

DH55 ref genome CTTTTTTTTAGAGATGAAAAGATCTATATATGTGTAATTATTAAGGTTTTGTCTCTAAAT

CN 119300 CTTTTTTTTAGAGATGAAAAGATCTATATATGTGTAATTATTAAGGTTTTGTCTCTAAAT

CAM 241 CTTTTTTTTAGAGATGAAAAGATCTATATATGTGTAATTATTAAGGTTTTGTCTCTAAAT

CN 113754 CTTTTTTTTAGAGATGAAAAGATCTATATATGTGTAATTATTAAGGTTTTGTCTCTAAAT

CAM 236 CTTTTTTTTAGAGATGAAAAGATCTATATATGTGTAATTATTAAGGTTTTGTCTCTAAAT

CO46 NCBI CTTTTTTTTAGAGATGAAAAGATCTATATATGTGTAATTATTAAGGTTTTGTCTCTAAAT

CN 120013 CTTTTTTTTAGAGATGAAAAGATCTATATATGTGTAATTATTAAGGTTTTGTCTCTAAAT

Blaine Creek CTTTTTTTTAGAGATGAAAAGATCTATATATGTGTAATTATTAAGGTTTTGTCTCTAAAT

09-CS0040 CTTTTTTTTAGAGATGAAAAGATCTATATATGTGTAATTATTAAGGTTTTGTCTCTAAAT

17CS1133 CTTTTTTTTAGAGATGAAAAGATCTATATATGTGTAATTATTAAGGTTTTGTCTCTAAAT

Jasper CTTTTTTTTAGAGATGAAAAGATCTATATATGTGTAATTATTAAGGTTTTGTCTCTAAAT

CN 120017 CTTTTTTTTAGAGATGAAAAGATCTATATATGTGTAATTATTAAGGTTTTGTCTCTAAAT

CN 120027 CTTTTTTTTAGAGATGAAAAGATCTATATATGTGTAATTATTAAGGTTTTGTCTCTAAAT

CN 120030 CTTTTTTTTAGAGATGAAAAGATCTATATATGTGTAATTATTAAGGTTTTGTCTCTAAAT

Hoga CTTTTTTTTAGAGATGAAAAGATCTATATATGTGTAATTATTAAGGTTTTGTCTCTAAAT

Yellowstone CTTTTTTTTAGAGATGAAAAGATCTATATATGTGTAATTATTAAGGTTTTGTCTCTAAAT

CN 119294 CTTTTTTTTAGAGATGAAAAGATCTATATATGTGTAATTATTAAGGTTTTGTCTCTAAAT

************************************************************

CN 120025 GTCTCTTCTGATGCCACCATATACATGTCTCATAGTTTCCAGTGGTCTTTTGAAGGGTTA

CN 119205 GTCTCTTCTGATGCCACCATATACATGTCTCATAGTTTCCAGTGGTCTTTTGAAGGGTTA

CN 119243 GTCTCTTCTGATGCCACCATATACATGTCTCATAGTTTCCAGTGGTCTTTTGAAGGGTTA

Joelle NCBI GTCTCTTCTGATGCCACCATATACATGTCTCATAGTTTCCAGTGGTCTTTTGAAGGGTTA

Joelle AAFC GTCTCTTCTGATGCCACCATATACATGTCTCATAGTTTCCAGTGGTCTTTTGAAGGGTTA

Joelle phyto GTCTCTTCTGATGCCACCATATACATGTCTCATAGTTTCCAGTGGTCTTTTGAAGGGTTA

DH55 ref genome GTCTCTTCTGATGCCACCATATACATGTCTCATAGTTTCCAGTGGTCTTTTGAAGGGTTA

CN 119300 GTCTCTTCTGATGCCACCATATACATGTCTCATAGTTTCCAGTGGTCTTTTGAAGGGTTA

CAM 241 GTCTCTTCTGATGCCACCATATACATGTCTCATAGTTTCCAGTGGTCTTTTGAAGGGTTA

CN 113754 GTCTCTTCTGATGCCACCATATACATGTCTCATAGTTTCCAGTGGTCTTTTGAAGGGTTA

CAM 236 GTCTCTTCTGATGCCACCATATACATGTCTCATAGTTTCCAGTGGTCTTTTGAAGGGTTA

CO46 NCBI GTCTCTTCTGATGCCACCATATACATGTCTCATAGTTTCCAGTGGTCTTTTGAAGGGTTA

CN 120013 GTCTCTTCTGATGCCACCATATACATGTCTCATAGTTTCCAGTGGTCTTTTGAAGGGTTA

Blaine Creek GTCTCTTCTGATGCCACCATATACATGTCTCATAGTTTCCAGTGGTCTTTTGAAGGGTTA

09-CS0040 GTCTCTTCTGATGCCACCATATACATGTCTCATAGTTTCCAGTGGTCTTTTGAAGGGTTA

17CS1133 GTCTCTTCTGATGCCACCATATACATGTCTCATAGTTTCCAGTGGTCTTTTGAAGGGTTA

Jasper GTCTCTTCTGATGCCACCATATACATGTCTCATAGTTTCCAGTGGTCTTTTGAAGGGTTA

CN 120017 GTCTCTTCTGATGCCACCATATACATGTCTCATAGTTTCCAGTGGTCTTTTGAAGGGTTA

CN 120027 GTCTCTTCTGATGCCACCATATACATGTCTCATAGTTTCCAGTGGTCTTTTGAAGGGTTA

CN 120030 GTCTCTTCTGATGCCACCATATACATGTCTCATAGTTTCCAGTGGTCTTTTGAAGGGTTA

Hoga GTCTCTTCTGATGCCACCATATACATGTCTCATAGTTTCCAGTGGTCTTTTGAAGGGTTA

Yellowstone GTCTCTTCTGATGCCACCATATACATGTCTCATAGTTTCCAGTGGTCTTTTGAAGGGTTA

CN 119294 GTCTCTTCTGATGCCACCATATACATGTCTCATAGTTTCCAGTGGTCTTTTGAAGGGTTA

************************************************************

CN 120025 GCTTGTATTTTTATTATCCTAACATACTTTTCTATTTTGTCATCTCTCCAGCCTGGTCAA

CN 119205 GCTTGTATTTTTATTATCCTGACATACTTTTCTATTTTGTCATCTCTCCAGCCTGGTCAA

CN 119243 GCTTGTATTTTTATTATCCTAACATACTTTTCTATTTTGTCATCTCTCCAGCCTGGTCAA

Joelle NCBI GCTTGTATTTTTATTATCCTAACATACTTTTCTATTTTGTCATCTCTCCAGCCTGGTCAA

Joelle AAFC GCTTGTATTTTTATTATCCTAACATACTTTTCTATTTTGTCATCTCTCCAGCCTGGTCAA

Joelle phyto GCTTGTATTTTTATTATCCTAACATACTTTTCTATTTTGTCATCTCTCCAGCCTGGTCAA

DH55 ref genome GCTTGTATTTTTATTATCCTAACATACTTTTCTATTTTGTCATCTCTCCAGCCTGGTCAA

CN 119300 GCTTGTATTTTTATTATCCTAACATACTTTTCTATTTTGTCATCTCTCCAGCCTGGTCAA

CAM 241 GCTTGTATTTTTATTATCCTAACATACTTTTCTATTTTGTCATCTCTCCAGCCTGGTCAA

CN 113754 GCTTGTATTTTTATTATCCTAACATACTTTTCTATTTTGTCATCTCTCCAGCCTGGTCAA

CAM 236 GCTTGTATTTTTATTATCCTAACATACTTTTCTATTTTGTCATCTCTCCAGCCTGGTCAA

CO46 NCBI GCTTGTATTTTTATTATCCTAACATACTTTTCTATTTTGTCATCTCTCCAGCCTGGTCAA

CN 120013 GCTTGTATTTTTATTATCCTAACATACTTTTCTATTTTGTCATCTCTCCAGCCTGGTCAA

Blaine Creek GCTTGTATTTTTATTATCCTAACATACTTTTCTATTTTGTCATCTCTCCAGCCTGGTCAA

09-CS0040 GCTTGTATTTTTATTATCCTAACATACTTTTCTATTTTGTCATCTCTCCAGCCTGGTCAA

17CS1133 GCTTGTATTTTTATTATCCTAACATACTTTTCTATTTTGTCATCTCTCCAGCCTGGTCAA

Jasper GCTTGTATTTTTATTATCCTAACATACTTTTCTATTTTGTCATCTCTCCAGCCTGGTCAA

CN 120017 GCTTGTATTTTTATTATCCTAACATACTTTTCTATTTTGTCATCTCTCCAGCCTGGTCAA

CN 120027 GCTTGTATTTTTATTATCCTAACATACTTTTCTATTTTGTCATCTCTCCAGCCTGGTCAA

CN 120030 GCTTGTATTTTTATTATCCTAACATACTTTTCTATTTTGTCATCTCTCCAGCCTGGTCAA

Hoga GCTTGTATTTTTATTATCCTAACATACTTTTCTATTTTGTCATCTCTCCAGCCTGGTCAA

Yellowstone GCTTGTATTTTTATTATCCTAACATACTTTTCTATTTTGTCATCTCTCCAGCCTGGTCAA

CN 119294 GCTTGTATTTTTATTATCCTAACATACTTTTCTATTTTGTCATCTCTCCAGCCTGGTCAA

******************** ***************************************

CN 120025 GATCCTTGATCGATATGGGAAACAACATGCTGATGATCTCAAAGCCTTGGTAATACAAAT

CN 119205 GATCCTTGATCGATATGGGAAACAACATGCTGATGATCTCAAAGCCTTGGTAATACAAAT

CN 119243 GATCCTTGATCGATATGGGAAACAACATGCTGATGATCTCAAAGCCTTGGTAATACAAAT

Joelle NCBI GATCCTTGATCGATATGGGAAACAACATGCTGATGATCTCAAAGCCTTGGTAATACAAAT

Joelle AAFC GATCCTTGATCGATATGGGAAACAACATGCTGATGATCTCAAAGCCTTGGTAATACAAAT

Joelle phyto GATCCTTGATCGATATGGGAAACAACATGCTGATGATCTCAAAGCCTTGGTAATACAAAT

DH55 ref genome GATCCTTGATCGATATGGGAAACAACATGCTGATGATCTCAAAGCCTTGGTAATACAAAT

CN 119300 GATCCTTGATCGATATGGGAAACAACATGCTGATGATCTCAAAGCCTTGGTAATACAAAT

CAM 241 GATCCTTGATCGATATGGGAAACAACATGCTGATGATCTCAAAGCCTTGGTAATACAAAT

CN 113754 GATCCTTGATCGATATGGGAAACAACATGCTGATGATCTCAAAGCCTTGGTAATACAAAT

CAM 236 GATCCTTGATCGATATGGGAAACAACATGCTGATGATCTCAAAGCCTTGGTAATACAAAT

CO46 NCBI GATCCTTGATCGATATGGGAAACAACATGCTGATGATCTCAAAGCCTTGGTAATACAAAT

CN 120013 GATCCTTGATCGATATGGGAAACAACATGCTGATGATCTCAAAGCCTTGGTAATACAAAT

Blaine Creek GATCCTTGATCGATATGGGAAACAACATGCTGATGATCTCAAAGCCTTGGTAATACAAAT

09-CS0040 GATCCTTGATCGATATGGGAAACAACATGCTGATGATCTCAAAGCCTTGGTAATACAAAT

17CS1133 GATCCTTGATCGATATGGGAAACAACATGCTGATGATCTCAAAGCCTTGGTAATACAAAT

Jasper GATCCTTGATCGATATGGGAAACAACATGCTGATGATCTCAAAGCCTTGGTAATACAAAT

CN 120017 GATCCTTGATCGATATGGGAAACAACATGCTGATGATCTCAAAGCCTTGGTAATACAAAT

CN 120027 GATCCTTGATCGATATGGGAAACAACATGCTGATGATCTCAAAGCCTTGGTAATACAAAT

CN 120030 GATCCTTGATCGATATGGGAAACAACATGCTGATGATCTCAAAGCCTTGGTAATACAAAT

Hoga GATCCTTGATCGATATGGGAAACAACATGCTGATGATCTCAAAGCCTTGGTAATACAAAT

Yellowstone GATCCTTGATCGATATGGGAAACAACATGCTGATGATCTCAAAGCCTTGGTAATACAAAT

CN 119294 GATCCTTGATCGATATGGGAAACAACATGCTGATGATCTCAAAGCCTTGGTAATACAAAT

************************************************************

CN 120025 ATTTCGGATATTTTCCCAGATGGATTTTTATAAGGCGTAGATTTACTAAAGACGTAGAGA

CN 119205 ATTTCGGATATTTTCCCAGATGGATTTTTATAAGGCGTAGATTTACTAAAGACGTAGAGA

CN 119243 ATTTCGGATATTTTCCCAGATGGATTTTTATAAGGCGTAGATTTACTAAAGACGTAGAGA

Joelle NCBI ATTTCGGATATTTTCCCAGATGGATTTTTATAAGGCGTAGATTTACTAAAGACGTAGAGA

Joelle AAFC ATTTCGGATATTTTCCCAGATGGATTTTTATAAGGCGTAGATTTACTAAAGACGTAGAGA

Joelle phyto ATTTCGGATATTTTCCCAGATGGATTTTTATAAGGCGTAGATTTACTAAAGACGTAGAGA

DH55 ref genome ATTTCGGATATTTTCCCAGATGGATTTTTATAAGGCGTAGATTTACTAAAGACGTAGAGA

CN 119300 ATTTCGGATATTTTCCCAGATGGATTTTTATAAGGCGTAGATTTACTAAAGACGTAGAGA

CAM 241 ATTTCGGATATTTTCCCAGATGGATTTTTATAAGGCGTAGATTTACTAAAGACGTAGAGA

CN 113754 ATTTCGGATATTTTCCCAGATGGATTTTTATAAGGCGTAGATTTACTAAAGACGTAGAGA

CAM 236 ATTTCGGATATTTTCCCAGATGGATTTTTATAAGGCGTAGATTTACTAAAGACGTAGAGA

CO46 NCBI ATTTCGGATATTTTCCCAGATGGATTTTTATAAGGCGTAGATTTACTAAAGACGTAGAGA

CN 120013 ATTTCGGATATTTTCCCAGATGGATTTTTATAAGGCGTAGATTTACTAAAGACGTAGAGA

Blaine Creek ATTTCGGATATTTTCCCAGATGGATTTTTATAAGGCGTAGATTTACTAAAGACGTAGAGA

09-CS0040 ATTTCGGATATTTTCCCAGATGGATTTTTATAAGGCGTAGATTTACTAAAGACGTAGAGA

17CS1133 ATTTCGGATATTTTCCCAGATGGATTTTTATAAGGCGTAGATTTACTAAAGACGTAGAGA

Jasper ATTTCGGATATTTTCCCAGATGGATTTTTATAAGGCGTAGATTTACTAAAGACGTAGAGA

CN 120017 ATTTCGGATATTTTCCCAGATGGATTTTTATAAGGCGTAGATTTACTAAAGACGTAGAGA

CN 120027 ATTTCGGATATTTTCCCAGATGGATTTTTATAAGGCGTAGATTTACTAAAGACGTAGAGA

CN 120030 ATTTCGGATATTTTCCCAGATGGATTTTTATAAGGCGTAGATTTACTAAAGACGTAGAGA

Hoga ATTTCGGATATTTTCCCAGATGGATTTTTATAAGGCGTAGATTTACTAAAGACGTAGAGA

Yellowstone ATTTCGGATATTTTCCCAGATGGATTTTTATAAGGCGTAGATTTACTAAAGACGTAGAGA

CN 119294 ATTTCGGATATTTTCCCAGATGGATTTTTATAAGGCGTAGATTTACTAAAGACGTAGAGA

************************************************************

CN 120025 GTTCAGTAATCAATACTGTCAGAGCCTATTCATAGCCTCCTGTCCAGCTTGCA-TTTTAT

CN 119205 GTTCAGTAATCAATACTGTCAGAGCCTATTCATAGCCTCCTGTCCAGCTTGCA-TTTTAT

CN 119243 GTTCAGTAATCAATACTGTCAGAGCCTATTCATAGCCTCCTGTCCAGCTTGCA-TTTTAT

Joelle NCBI GTTCAGTAATCAATACTGTCAGAGCCTATTCATAGCCTCCTGTCCAGCTTGCA-TTTTAT

Joelle AAFC GTTCAGTAATCAATACTGTCAGAGCCTATTCATAGCCTCCTGTCCAGCTTGCA-TTTTAT

Joelle phyto GTTCAGTAATCAATACTGTCAGAGCCTATTCATAGCCTCCTGTCCAGCTTGCA-TTTTAT

DH55 ref genome GTTCAGTAATCAATACTGTCAGAGCCTATTCATAGCCTCCTGTCTAGCTTGCATTTTTAT

CN 119300 GTTCAGTAATCAATACTGTCAGAGCCTATTCATAGCCTCCTGTCCAGCTTGCA-TTTTAT

CAM 241 GTTCAGTAATCAATACTGTCAGAGCCTATTCATAGCCTCCTGTCCAGCTTGCA-TTTTAT

CN 113754 GTTCAGTAATCAATACTGTCAGAGCCTATTCATAGCCTCCTGTCCAGCTTGCA-TTTTAT

CAM 236 GTTCAGTAATCAATACTGTCAGAGCCTATTCATAGCCTCCTGTCCAGCTTGCA-TTTTAT

CO46 NCBI GTTCAGTAATCAATACTGTCAGAGCCTATTCATAGCCTCCTGTCCAGCTTGCA-TTTTAT

CN 120013 GTTCAGTAATCAATACTGTCAGAGCCTATTCATAGCCTCCTGTCCAGCTTGCA-TTTTAT

Blaine Creek GTTCAGTAATCAATACTGTCAGAGCCTATTCATAGCCTCCTGTCCAGCTTGCA-TTTTAT

09-CS0040 GTTCAGTAATCAATACTGTCAGAGCCTATTCATAGCCTCCTGTCCAGCTTGCA-TTTTAT

17CS1133 GTTCAGTAATCAATACTGTCAGAGCCTATTCATAGCCTCCTGTCCAGCTTGCA-TTTTAT

Jasper GTTCAGTAATCAATACTGTCAGAGCCTATTCATAGCCTCCTGTCCAGCTTGCA-TTTTAT

CN 120017 GTTCAGTAATCAATACTGTCAGAGCCTATTCATAGCCTCCTGTCCAGCTTGCA-TTTTAT

CN 120027 GTTCAGTAATCAATACTGTCAGAGCCTATTCATAGCCTCCTGTCCAGCTTGCA-TTTTAT

CN 120030 GTTCAGTAATCAATACTGTCAGAGCCTATTCATAGCCTCCTGTCCAGCTTGCA-TTTTAT

Hoga GTTCAGTAATCAATACTGTCAGAGCCTATTCATAGCCTCCTGTCCAGCTTGCA-TTTTAT

Yellowstone GTTCAGTAATCAATACTGTCAGAGCCTATTCATAGCCTCCTGTCCAGCTTGCA-TTTTAT

CN 119294 GTTCAGTAATCAATACTGTCAGAGCCTATTCATAGCCTCCTGTCCAGCTTGCA-TTTTAT

******************************************** ******** ******

CN 120025 AGGCCTGGGCTAAGAGGCATATAACGTTTTCACATATAGTCACTATTTGGAGTTGTGTAG

CN 119205 AGGCCTGGGCTAAGAGGCATATAACGTTTTCACATATAGTCACTATTTGGAGTTGTGTAG

CN 119243 AGGCCTGGGCTAAGAGGCATATAACGTTTTCACATATAGTCACTATTTGGAGTTGTGTAG

Joelle NCBI AGGCCTGGGCTAAGAGGCATATAACGTTTTCACATATAGTCACTATTTGGAGTTGTGTAG

Joelle AAFC AGGCCTGGGCTAAGAGGCATATAACGTTTTCACATATAGTCACTATTTGGAGTTGTGTAG

Joelle phyto AGGCCTGGGCTAAGAGGCATATAACGTTTTCACATATAGTCACTATTTGGAGTTGTGTAG

DH55 ref genome AGGCCTGGGCTAAGAGGCATATAACGTTTTCACATATAGTCACTATTTGGAGTTGTGTAG

CN 119300 AGGCCTGGGCTAAGAGGCATATAACGTTTTCACATATAGTCACTATTTGGAGTTGTGTAG

CAM 241 AGGCCTGGGCTAAGAGGCATATAACGTTTTCACATATAGTCACTATTTGGAGTTGTGTAG

CN 113754 AGGCCTGGGCTAAGAGGCATATAACGTTTTCACATATAGTCACTATTTGGAGTTGTGTAG

CAM 236 AGGCCTGGGCTAAGAGGCATATAACGTTTTCACATATAGTCACTATTTGGAGTTGTGTAG

CO46 NCBI AGGCCTGGGCTAAGAGGCATATAACGTTTTCACATATAGTCACTATTTGGAGTTGTGTAG

CN 120013 AGGCCTGGGCTAAGAGGCATATAACGTTTTCACATATAGTCACTATTTGGAGTTGTGTAG

Blaine Creek AGGCCTGGGCTAAGAGGCATATAACGTTTTCACATATAGTCACTATTTGGAGTTGTGTAG

09-CS0040 AGGCCTGGGCTAAGAGGCATATAACGTTTTCACATATAGTCACTATTTGGAGTTGTGTAG

17CS1133 AGGCCTGGGCTAAGAGGCATATAACGTTTTCACATATAGTCACTATTTGGAGTTGTGTAG

Jasper AGGCCTGGGCTAAGAGGCATATAACGTTTTCACATATAGTCACTATTTGGAGTTGTGTAG

CN 120017 AGGCCTGGGCTAAGAGGCATATAACGTTTTCACATATAGTCACTATTTGGAGTTGTGTAG

CN 120027 AGGCCTGGGCTAAGAGGCATATAACGTTTTCACATATAGTCACTATTTGGAGTTGTGTAG

CN 120030 AGGCCTGGGCTAAGAGGCATATAACGTTTTCACATATAGTCACTATTTGGAGTTGTGTAG

Hoga AGGCCTGGGCTAAGAGGCATATAACGTTTTCACATATAGTCACTATTTGGAGTTGTGTAG

Yellowstone AGGCCTGGGCTAAGAGGCATATAACGTTTTCACATATAGTCACTATTTGGAGTTGTGTAG

CN 119294 AGGCCTGGGCTAAGAGGCATATAACGTTTTCACATATAGTCACTATTTGGAGTTGTGTAG

************************************************************

CN 120025 TTGTAATTGTTTAATAGATATGAGTAAACGAATGTTTAGCACCATAACATTGTGGAGATA

CN 119205 TTGTAATTGTTTAATAGATATGAGTAAACGAATGTTTAGCACCATAACATTGTGGAGATA

CN 119243 TTGTAATTGTTTAATAGATATGAGTAAACGAATGTTTAGCACCATAACATTGTGGAGATA

Joelle NCBI TTGTAATTGTTTAATAGATATGAGTAAACGAATGTTTAGCACCATAACATTGTGGAGATA

Joelle AAFC TTGTAATTGTTTAATAGATATGAGTAAACGAATGTTTAGCACCATAACATTGTGGAGATA

Joelle phyto TTGTAATTGTTTAATAGATATGAGTAAACGAATGTTTAGCACCATAACATTGTGGAGATA

DH55 ref genome TTGTAATTGTTTAATAGATATGAGTAAACGAATGTTTAGCACCATAACATTGTGGAGATA

CN 119300 TTGTAATTGTTTAATAGATATGAGTAAACGAATGTTTAGCACCATAACATTGTGGAGATA

CAM 241 TTGTAATTGTTTAATAGATATGAGTAAACGAATGTTTAGCACCATAACATTGTGGAGATA

CN 113754 TTGTAATTGTTTAATAGATATGAGTAAACGAATGTTTAGCACCATAACATTGTGGAGATA

CAM 236 TTGTAATTGTTTAATAGATATGAGTAAACGAATGTTTAGCACCATAACATTGTGGAGATA

CO46 NCBI TTGTAATTGTTTAATAGATATGAGTAAACGAATGTTTAGCACCATAACATTGTGGAGATA

CN 120013 TTGTAATTGTTTAATAGATATGAGTAAACGAATGTTTAGCACCATAACATTGTGGAGATA

Blaine Creek TTGTAATTGTTTAATAGATATGAGTAAACGAATGTTTAGCACCATAACATTGTGGAGATA

09-CS0040 TTGTAATTGTTTAATAGATATGAGTAAACGAATGTTTAGCACCATAACATTGTGGAGATA

17CS1133 TTGTAATTGTTTAATAGATATGAGTAAACGAATGTTTAGCACCATAACATTGTGGAGATA

Jasper TTGTAATTGTTTAATAGATATGAGTAAACGAATGTTTAGCACCATAACATTGTGGAGATA

CN 120017 TTGTAATTGTTTAATAGATATGAGTAAACGAATGTTTAGCACCATAACATTGTGGAGATA

CN 120027 TTGTAATTGTTTAATAGATATGAGTAAACGAATGTTTAGCACCATAACATTGTGGAGATA

CN 120030 TTGTAATTGTTTAATAGATATGAGTAAACGAATGTTTAGCACCATAACATTGTGGAGATA

Hoga TTGTAATTGTTTAATAGATATGAGTAAACGAATGTTTAGCACCATAACATTGTGGAGATA

Yellowstone TTGTAATTGTTTAATAGATATGAGTAAACGAATGTTTAGCACCATAACATTGTGGAGATA

CN 119294 TTGTAATTGTTTAATAGATATGAGTAAACGAATGTTTAGCACCATAACATTGTGGAGATA

************************************************************

CN 120025 TATCTAGATAAAGAATTGCATCATTGATCCACCATGCGCTATTAGCGTGCCAAGTGATCC

CN 119205 TATCTAGATAAAGAATTGCATCATTGATCCACCATGCGCTATTAGCTTGCCAAGTGATCC

CN 119243 TATCTAGATAAAGAATTGCATCATTGATCCACCATGCGCTATTAGCTTGCCAAGTGATCC

Joelle NCBI TATCTAGATAAAGAATTGCATCATTGATCCACCATGCGCTATTAGCTTGCCAAGTGATCC

Joelle AAFC TATCTAGATAAAGAATTGCATCATTGATCCACCATGCGCTATTAGCTTGCCAAGTGATCC

Joelle phyto TATCTAGATAAAGAATTGCATCATTGATCCACCATGCGCTATTAGCTTGCCAAGTGATCC

DH55 ref genome TATCTAGATAAAGAATTGCATCATTGATCCACCATGCGCTATTAGCTTGCCAAGTGATCC

CN 119300 TATCTAGATAAAGAATTGCATCATTGATCCACCATGCGCTATTAGCTTGCCAAGTGATCC

CAM 241 TATCTAGATAAAGAATTGCATCATTGATCCACCATGCGCTATTAGCTTGCCAAGTGATCC

CN 113754 TATCTAGATAAAGAATTGCATCATTGATCCACCATGCGCTATTAGCTTGCCAAGTGATCC

CAM 236 TATCTAGATAAAGAATTGCATCATTGATCCACCATGCGCTATTAGCTTGCCAAGTGATCC

CO46 NCBI TATCTAGATAAAGAATTGCATCATTGATCCACCATGCGCTATTAGCTTGCCAAGTGATCC

CN 120013 TATCTAGATAAAGAATTGCATCATTGATCCACCATGCGCTATTAGCTTGCCAAGTGATCC

Blaine Creek TATCTAGATAAAGAATTGCATCATTGATCCACCATGCGCTATTAGCTTGCCAAGTGATCC

09-CS0040 TATCTAGATAAAGAATTGCATCATTGATCCACCATGCGCTATTAGCTTGCCAAGTGATCC

17CS1133 TATCTAGATAAAGAATTGCATCATTGATCCACCATGCGCTATTAGCTTGCCAAGTGATCC

Jasper TATCTAGATAAAGAATTGCATCATTGATCCACCATGCGCTATTAGCTTGCCAAGTGATCC

CN 120017 TATCTAGATAAAGAATTGCATCATTGATCCACCATGCGCTATTAGCTTGCCAAGTGATCC

CN 120027 TATCTAGATAAAGAATTGCATCATTGATCCACCATGCGCTATTAGCTTGCCAAGTGATCC

CN 120030 TATCTAGATAAAGAATTGCATCATTGATCCACCATGCGCTATTAGCTTGCCAAGTGATCC

Hoga TATCTAGATAAAGAATTGCATCATTGATCCACCATGCGCTATTAGCTTGCCAAGTGATCC

Yellowstone TATCTAGATAAAGAATTGCATCATTGATCCACCATGCGCTATTAGCTTGCCAAGTGATCC

CN 119294 TATCTAGATAAAGAATTGCATCATTGATCCACCATGCGCTATTAGCTTGCCAAGTGATCC

********************************************** *************

CN 120025 TAAATATATAATGAGGTGGTGTACCAACATGCATACTGAAATCCTCGTAGCTTTTTCTTT

CN 119205 TAAATATATAATGAGGTGGTGTACCAACATGCATACTGAAATCCTCGTAGCTTTTTCTTT

CN 119243 TAAATATATAATGAGGTGGTGTACCAACATGCATACTGAAATCCTCGTAGCTTTTTCTTT

Joelle NCBI TAAATATATAATGAGGTGGTGTACCAACATGCATACTGAAATCCTCGTAGCTTTTTCTTT

Joelle AAFC TAAATATATAATGAGGTGGTGTACCAACATGCATACTGAAATCCTCGTAGCTTTTTCTTT

Joelle phyto TAAATATATAATGAGGTGGTGTACCAACATGCATACTGAAATCCTCGTAGCTTTTTCTTT

DH55 ref genome TAAATATATAATGAGGTGGTGTACCAACATGCATACTGAAATCCTCGTAGCTTTTTCTTT

CN 119300 TAAATATATAATGAGGTGGTGTACCAACATGCATACTGAAATCCTCGTAGCTTTTTCTTT

CAM 241 TAAATATATAATGAGGTGGTGTACCAACATGCATACTGAAATCCTCGTAGCTTTTTCTTT

CN 113754 TAAATATATAATGAGGTGGTGTACCAACATGCATACTGAAATCCTCGTAGCTTTTTCTTT

CAM 236 TAAATATATAATGAGGTGGTGTACCAACATGCATACTGAAATCCTCGTAGCTTTTTCTTT

CO46 NCBI TAAATATATAATGAGGTGGTGTACCAACATGCATACTGAAATCCTCGTAGCTTTTTCTTT

CN 120013 TAAATATATAATGAGGTGGTGTACCAACATGCATACTGAAATCCTCGTAGCTTTTTCTTT

Blaine Creek TAAATATATAATGAGGTGGTGTACCAACATGCATACTGAAATCCTCGTAGCTTTTTCTTT

09-CS0040 TAAATATATAATGAGGTGGTGTACCAACATGCATACTGAAATCCTCGTAGCTTTTTCTTT

17CS1133 TAAATATATAATGAGGTGGTGTACCAACATGCATACTGAAATCCTCGTAGCTTTTTCTTT

Jasper TAAATATATAATGAGGTGGTGTACCAACATGCATACTGAAATCCTCGTAGCTTTTTCTTT

CN 120017 TAAATATATAATGAGGTGGTGTACCAACATGCATACTGAAATCCTCGTAGCTTTTTCTTT

CN 120027 TAAATATATAATGAGGTGGTGTACCAACATGCATACTGAAATCCTCGTAGCTTTTTCTTT

CN 120030 TAAATATATAATGAGGTGGTGTACCAACATGCATACTGAAATCCTCGTAGCTTTTTCTTT

Hoga TAAATATATAATGAGGTGGTGTACCAACATGCATACTGAAATCCTCGTAGCTTTTTCTTT

Yellowstone TAAATATATAATGAGGTGGTGTACCAACATGCATACTGAAATCCTCGTAGCTTTTTCTTT

CN 119294 TAAATATATAATGAGGTGGTGTACCAACATGCATACTGAAATCCTCGTAGCTTTTTCTTT

************************************************************

CN 120025 TACCGCATCTTATATGTCCCTTTTCTTTGGCCAGGATCTTCAGTCAAAAGCTCTGAACTA

CN 119205 TACCGCATCTTATATGTCCCTTTTCTTTGGCCAGGATCTTCAGTCAAAAGCTCTGAACTA

CN 119243 TACCGCATCTTATATGTCCCTTTTCTTTGGCCAGGATCTTCAGTCAAAAGCTCTGAACTA

Joelle NCBI TACCGCATCTTATATGTCCCTTTTCTTTGGCCAGGATCTTCAGTCAAAAGCTCTGAACTA

Joelle AAFC TACCGCATCTTATATGTCCCTTTTCTTTGGCCAGGATCTTCAGTCAAAAGCTCTGAACTA

Joelle phyto TACCGCATCTTATATGTCCCTTTTCTTTGGCCAGGATCTTCAGTCAAAAGCTCTGAACTA

DH55 ref genome TACCGCATCTTATATGTCCCTTTTCTTTGGCCAGGATCTTCAGTCAAAAGCTCTGAACTA

CN 119300 TACCGCATCTTATATGTCCCTTTTCTTTGGCCAGGATCTTCAGTCAAAAGCTCTGAACTA

CAM 241 TACCGCATCTTATATGTCCCTTTTCTTTGGCCAGGATCTTCAGTCAAAAGCTCTGAACTA

CN 113754 TACCGCATCTTATATGTCCCTTTTCTTTGGCCAGGATCTTCAGTCAAAAGCTCTGAACTA

CAM 236 TACCGCATCTTATATGTCCCTTTTCTTTGGCCAGGATCTTCAGTCAAAAGCTCTGAACTA

CO46 NCBI TACCGCATCTTATATGTCCCTTTTCTTTGGCCAGGATCTTCAGTCAAAAGCTCTGAACTA

CN 120013 TACCGCATCTTATATGTCCCTTTTCTTTGGCCAGGATCTTCAGTCAAAAGCTCTGAACTA

Blaine Creek TACCGCATCTTATATGTCCCTTTTCTTTGGCCAGGATCTTCAGTCAAAAGCTCTGAACTA

09-CS0040 TACCGCATCTTATATGTCCCTTTTCTTTGGCCAGGATCTTCAGTCAAAAGCTCTGAACTA

17CS1133 TACCGCATCTTATATGTCCCTTTTCTTTGGCCAGGATCTTCAGTCAAAAGCTCTGAACTA

Jasper TACCGCATCTTATATGTCCCTTTTCTTTGGCCAGGATCTTCAGTCAAAAGCTCTGAACTA

CN 120017 TACCGCATCTTATATGTCCCTTTTCTTTGGCCAGGATCTTCAGTCAAAAGCTCTGAACTA

CN 120027 TACCGCATCTTATATGTCCCTTTTCTTTGGCCAGGATCTTCAGTCAAAAGCTCTGAACTA

CN 120030 TACCGCATCTTATATGTCCCTTTTCTTTGGCCAGGATCTTCAGTCAAAAGCTCTGAACTA

Hoga TACCGCATCTTATATGTCCCTTTTCTTTGGCCAGGATCTTCAGTCAAAAGCTCTGAACTA

Yellowstone TACCGCATCTTATATGTCCCTTTTCTTTGGCCAGGATCTTCAGTCAAAAGCTCTGAACTA

CN 119294 TACCGCATCTTATATGTCCCTTTTCTTTGGCCAGGATCTTCAGTCAAAAGCTCTGAACTA

************************************************************

CN 120025 TGGTTCGCACCATGAGCTACTAGAACTCGTGGAAAGGTTAGTACTAACTAAGACTATATT

CN 119205 TGGTTCGCACCATGAGCTACTAGAACTCGTGGAAAGGTTAGTACTAACTAAGACTATATT

CN 119243 TGGTTCGCACCATGAGCTACTAGAACTCGTGGAAAGGTTAGTACTAACTAAGACTATATT

Joelle NCBI TGGTTCGCACCATGAGCTACTAGAACTCGTGGAAAGGTTAGTACTAACTAAGACTATATT

Joelle AAFC TGGTTCGCACCATGAGCTACTAGAACTCGTGGAAAGGTTAGTACTAACTAAGACTATATT

Joelle phyto TGGTTCGCACCATGAGCTACTAGAACTCGTGGAAAGGTTAGTACTAACTAAGACTATATT

DH55 ref genome TGGTTCGCACCATGAGCTACTAGAACTCGTGGAAAGGTTAGTACTAACTAAAACTATATT

CN 119300 TGGTTCGCACCATGAGCTACTAGAACTCGTGGAAAGGTTAGTACTAACTAAGACTATATT

CAM 241 TGGTTCGCACCATGAGCTACTAGAACTCGTGGAAAGGTTAGTACTAACTAAGACTATATT

CN 113754 TGGTTCGCACCATGAGCTACTAGAACTCGTGGAAAGGTTAGTACTAACTAAGACTATATT

CAM 236 TGGTTCGCACCATGAGCTACTAGAACTCGTGGAAAGGTTAGTACTAACTAAGACTATATT

CO46 NCBI TGGTTCGCACCATGAGCTACTAGAACTCGTGGAAAGGTTAGTACTAACTAAGACTATATT

CN 120013 TGGTTCGCACCATGAGCTACTAGAACTCGTGGAAAGGTTAGTACTAACTAAGACTATATT

Blaine Creek TGGTTCGCACCATGAGCTACTAGAACTCGTGGAAAGGTTAGTACTAACTAAGACTATATT

09-CS0040 TGGTTCGCACCATGAGCTACTAGAACTCGTGGAAAGGTTAGTACTAACTAAGACTATATT

17CS1133 TGGTTCGCACCATGAGCTACTAGAACTCGTGGAAAGGTTAGTACTAACTAAGACTATATT

Jasper TGGTTCGCACCATGAGCTACTAGAACTCGTGGAAAGGTTAGTACTAACTAAGACTATATT

CN 120017 TGGTTCGCACCATGAGCTACTAGAACTCGTGGAAAGGTTAGTACTAACTAAGACTATATT

CN 120027 TGGTTCGCACCATGAGCTACTAGAACTCGTGGAAAGGTTAGTACTAACTAAGACTATATT

CN 120030 TGGTTCGCACCATGAGCTACTAGAACTCGTGGAAAGGTTAGTACTAACTAAGACTATATT

Hoga TGGTTCGCACCATGAGCTACTAGAACTCGTGGAAAGGTTAGTACTAACTAAGACTATATT

Yellowstone TGGTTCGCACCATGAGCTACTAGAACTCGTGGAAAGGTTAGTACTAACTAAGACTATATT

CN 119294 TGGTTCGCACCATGAGCTACTAGAACTCGTGGAAAGGTTAGTACTAACTAAGACTATATT

*************************************************** ********

CN 120025 TGCTCTCCTGCTTTGAGTATAAAGGAATTAGGGCTTTCTTGTCAAACTATGAATATATGC

CN 119205 TGCTCTCCTGCTTTGAGTATAAAGGAATTAGGGCTTTCTTGTCAAACTATGAATATATGC

CN 119243 TGCTCTCCTGCTTTGAGTATAAAGGAATTAGGGCTTTCTTGTCAAACTATGAATATATGC

Joelle NCBI TGCTCTCCTGCTTTGAGTATAAAGGAATTAGGGCTTTCTTGTCAAACTATGAATATATGC

Joelle AAFC TGCTCTCCTGCTTTGAGTATAAAGGAATTAGGGCTTTCTTGTCAAACTATGAATATATGC

Joelle phyto TGCTCTCCTGCTTTGAGTATAAAGGAATTAGGGCTTTCTTGTCAAACTATGAATATATGC

DH55 ref genome TGCTCTCCTGCTTTGAGTATAAAGGAATTAGGGCTTTCTTGTCAAACTATGAATATATGC

CN 119300 TGCTCTCCTGCTTTGAGTATAAAGGAATTAGGGCTTTCTTGTCAAACTATGAATATATGC

CAM 241 TGCTCTCCTGCTTTGAGTATAAAGGAATTAGGGCTTTCTTGTCAAACTATGAATATATGC

CN 113754 TGCTCTCCTGCTTTGAGTATAAAGGAATTAGGGCTTTCTTGTCAAACTATGAATATATGC

CAM 236 TGCTCTCCTGCTTTGAGTATAAAGGAATTAGGGCTTTCTTGTCAAACTATGAATATATGC

CO46 NCBI TGCTCTCCTGCTTTGAGTATAAAGGAATTAGGGCTTTCTTGTCAAACTATGAATATATGC

CN 120013 TGCTCTCCTGCTTTGAGTATAAAGGAATTAGGGCTTTCTTGTCAAACTATGAATATATGC

Blaine Creek TGCTCTCCTGCTTTGAGTATAAAGGAATTAGGGCTTTCTTGTCAAACTATGAATATATGC

09-CS0040 TGCTCTCCTGCTTTGAGTATAAAGGAATTAGGGCTTTCTTGTCAAACTATGAATATATGC

17CS1133 TGCTCTCCTGCTTTGAGTATAAAGGAATTAGGGCTTTCTTGTCAAACTATGAATATATGC

Jasper TGCTCTCCTGCTTTGAGTATAAAGGAATTAGGGCTTTCTTGTCAAACTATGAATATATGC

CN 120017 TGCTCTCCTGCTTTGAGTATAAAGGAATTAGGGCTTTCTTGTCAAACTATGAATATATGC

CN 120027 TGCTCTCCTGCTTTGAGTATAAAGGAATTAGGGCTTTCTTGTCAAACTATGAATATATGC

CN 120030 TGCTCTCCTGCTTTGAGTATAAAGGAATTAGGGCTTTCTTGTCAAACTATGAATATATGC

Hoga TGCTCTCCTGCTTTGAGTATAAAGGAATTAGGGCTTTCTTGTCAAACTATGAATATATGC

Yellowstone TGCTCTCCTGCTTTGAGTATAAAGGAATTAGGGCTTTCTTGTCAAACTATGAATATATGC

CN 119294 TGCTCTCCTGCTTTGAGTATAAAGGAATTAGGGCTTTCTTGTCAAACTATGAATATATGC

************************************************************

CN 120025 AGCAATCTTGTGGAATCAAATGTCAATAATGTAAGTGTCGATGCCCTCGTTCAACTGGAG

CN 119205 AGCAATCTTGTGGAATCAAATGTCAATAATGTAAGTGTCGATGCCCTCGTTCAACTGGAG

CN 119243 AGCAATCTTGTGGAATCAAATGTCAATAATGTAAGTGTCGATGCCCTCGTTCAACTGGAG

Joelle NCBI AGCAATCTTGTGGAATCAAATGTCAATAATGTAAGTGTCGATGCCCTCGTTCAACTGGAG

Joelle AAFC AGCAATCTTGTGGAATCAAATGTCAATAATGTAAGTGTCGATGCCCTCGTTCAACTGGAG

Joelle phyto AGCAATCTTGTGGAATCAAATGTCAATAATGTAAGTGTCGATGCCCTCGTTCAACTGGAG

DH55 ref genome AGCAATCTTGTGGAATCAAATGTCAATAATGTAAGTGTCGATGCCCTCGTT---CTGGAG

CN 119300 AGCAATCTTGTGGAATCAAATGTCAATAATGTAAGTGTCGATGCCCTCGTT---CTGGAG

CAM 241 AGCAATCTTGTGGAATCAAATGTCAATAATGTAAGTGTCGATGCCCTCGTT---CTGGAG

CN 113754 AGCAATCTTGTGGAATCAAATGTCAATAATGTAAGTGTCGATGCCCTCGTT---CTGGAG

CAM 236 AGCAATCTTGTGGAATCAAATGTCAATAATGTAAGTGTCGATGCCCTCGTT---CTGGAG

CO46 NCBI AGCAATCTTGTGGAATCAAATGTCAATAATGTAAGTGTCGATGCCCTCGTT---CTGGAG

CN 120013 AGCAATCTTGTGGAATCAAATGTCAATAATGTAAGTGTCGATGCCCTCGTT---CTGGAG

Blaine Creek AGCAATCTTGTGGAATCAAATGTCAATAATGTAAGTGTCGATGCCCTCGTT---CTGGAG

09-CS0040 AGCAATCTTGTGGAATCAAATGTCAATAATGTAAGTGTCGATGCCCTCGTT---CTGGAG

17CS1133 AGCAATCTTGTGGAATCAAATGTCAATAATGTAAGTGTCGATGCCCTCGTT---CTGGAG

Jasper AGCAATCTTGTGGAATCAAATGTCAATAATGTAAGTGTCGATGCCCTCGTT---CTGGAG

CN 120017 AGCAATCTTGTGGAATCAAATGTCAATAATGTAAGTGTCGATGCCCTCGTT---CTGGAG

CN 120027 AGCAATCTTGTGGAATCAAATGTCAATAATGTAAGTGTCGATGCCCTCGTT---CTGGAG

CN 120030 AGCAATCTTGTGGAATCAAATGTCAATAATGTAAGTGTCGATGCCCTCGTT---CTGGAG

Hoga AGCAATCTTGTGGAATCAAATGTCAATAATGTAAGTGTCGATGCCCTCGTT---CTGGAG

Yellowstone AGCAATCTTGTGGAATCAAATGTCAATAATGTAAGTGTCGATGCCCTCGTT---CTGGAG

CN 119294 AGCAATCTTGTGGAATCAAATGTCAATAATGTAAGTGTCGATGCCCTCGTT---CTGGAG

*************************************************** ******

CN 120025 GAACACCTTGAGACCGCCCTCTCCGTAACGAGAGCCAAGAAGGTTAGTGGATTCTCTAAT

CN 119205 GAACACCTTGAGACCGCCCTCTCCGTAACTAGAGCCAAGAAGGTTAGTGGATTCTCTAAT

CN 119243 GAACACCTTGAGACCGCCCTCTCCGTAACTAGAGCCAAGAAGGTTAGTGGATTCTCTAAT

Joelle NCBI GAACACCTTGAGACCGCCCTCTCCGTAACTAGAGCCAAGAAGGTTAGTGGATTCTCTAAT

Joelle AAFC GAACACCTTGAGACCGCCCTCTCCGTAACTAGAGCCAAGAAGGTTAGTGGATTCTCTAAT

Joelle phyto GAACACCTTGAGACCGCCCTCTCCGTAACTAGAGCCAAGAAGGTTAGTGGATTCTCTAAT

DH55 ref genome GAACACCTTGAGACCGCCCTCTCCGTAACTAGTGCCAAGAAGGTTAGTGGATTCTCTAAT

CN 119300 GAACACCTTGAGACCGCCCTCTCCGTAACTAGAGCCAAGAAGGTTAGTGGATTCTCTAAT

CAM 241 GAACACCTTGAGACCGCCCTCTCCGTAACTAGAGCCAAGAAGGTTAGTGGATTCTCTAAT

CN 113754 GAACACCTTGAGACCGCCCTCTCCGTAACTAGTGCCAAGAAGGTTAGTGGATTCTCTAAT

CAM 236 GAACACCTTGAGACCGCCCTCTCCGTAACTAGTGCCAAGAAGGTTAGTGGATTCTCTAAT

CO46 NCBI GAACACCTTGAGACCGCCCTCTCCGTAACTAGTGCCAAGAAGGTTAGTGGATTCTCTAAT

CN 120013 GAACACCTTGAGACCGCCCTCTCCGTAACTAGAGCCAAGAAGGTTAGTGGATTCTCTAAT

Blaine Creek GAACACCTTGAGACCGCCCTCTCCGTAACTAGAGCCAAGAAGGTTAGTGGATTCTCTAAT

09-CS0040 GAACACCTTGAGACCGCCCTCTCCGTAACTAGAGCCAAGAAGGTTAGTGGATTCTCTAAT

17CS1133 GAACACCTTGAGACCGCCCTCTCCGTAACTAGAGCCAAGAAGGTTAGTGGATTCTCTAAT

Jasper GAACACCTTGAGACCGCCCTCTCCGTAACTAGAGCCAAGAAGGTTAGTGGATTCTCTAAT

CN 120017 GAACACCTTGAGACCGCCCTCTCCGTAACTAGAGCCAAGAAGGTTAGTGGATTCTCTAAT

CN 120027 GAACACCTTGAGACCGCCCTCTCCGTAACTAGAGCCAAGAAGGTTAGTGGATTCTCTAAT

CN 120030 GAACACCTTGAGACCGCCCTCTCCGTAACTAGAGCCAAGAAGGTTAGTGGATTCTCTAAT

Hoga GAACACCTTGAGACCGCCCTCTCCGTAACTAGAGCCAAGAAGGTTAGTGGATTCTCTAAT

Yellowstone GAACACCTTGAGACCGCCCTCTCCGTAACTAGAGCCAAGAAGGTTAGTGGATTCTCTAAT

CN 119294 GAACACCTTGAGACCGCCCTCTCCGTAACTAGAGCCAAGAAGGTTAGTGGATTCTCTAAT

***************************** ** ***************************

CN 120025 GACTACTCTTTTCAGAATTTGTTTGCAGAGAATAACCTTATTGCTTTTGTTTGTTACAGA

CN 119205 GTCTACTCTTTTCAGAATTTGTTTGCAGAGAATAACCTTATTGCTTTTGTTTGTTACAGA

CN 119243 GTCTACTCTTTTCAGAATTTGTTTGCAGAGAATAACCTTATTGCTTTTGTTTGTTACAGA

Joelle NCBI GTCTACTCTTTTCAGAATTTGTTTGCAGAGAATAACCTTATTGCTTTTGTTTGTTACAGA

Joelle AAFC GTCTACTCTTTTCAGAATTTGTTTGCAGAGAATAACCTTATTGCTTTTGTTTGTTACAGA

Joelle phyto GTCTACTCTTTTCAGAATTTGTTTGCAGAGAATAACCTTATTGCTTTTGTTTGTTACAGA

DH55 ref genome GTCTACTCTTTTCAGAATTTGTTTGCAGAGAATAACCTTATTGCTTTTGTTTGTTACAGA

CN 119300 GTCTACTCTTTTCAGAATTTGTTTGCAGAGAATAACCTTATTGCTTTTGTTTGTTACAGA

CAM 241 GTCTACTCTTTTCAGAATTTGTTTGCAGAGAATAACCTTATTGCTTTTGTTTGTTACAGA

CN 113754 GTCTACTCTTTTCAGAATTTGTTTGCAGAGAATAACCTTATTGCTTTTGTTTGTTACAGA

CAM 236 GTCTACTCTTTTCAGAATTTGTTTGCAGAGAATAACCTTATTGCTTTTGTTTGTTACAGA

CO46 NCBI GTCTACTCTTTTCAGAATTTGTTTGCAGAGAATAACCTTATTGCTTTTGTTTGTTACAGA

CN 120013 GTCTACTCTTTTCAGAATTTGTTTGCAGAGAATAACCTTATTGCTTTTGTTTGTTACAGA

Blaine Creek GTCTACTCTTTTCAGAATTTGTTTGCAGAGAATAACCTTATTGCTTTTGTTTGTTACAGA

09-CS0040 GTCTACTCTTTTCAGAATTTGTTTGCAGAGAATAACCTTATTGCTTTTGTTTGTTACAGA

17CS1133 GTCTACTCTTTTCAGAATTTGTTTGCAGAGAATAACCTTATTGCTTTTGTTTGTTACAGA

Jasper GTCTACTCTTTTCAGAATTTGTTTGCAGAGAATAACCTTATTGCTTTTGTTTGTTACAGA

CN 120017 GTCTACTCTTTTCAGAATTTGTTTGCAGAGAATAACCTTATTGCTTTTGTTTGTTACAGA

CN 120027 GTCTACTCTTTTCAGAATTTGTTTGCAGAGAATAACCTTATTGCTTTTGTTTGTTACAGA

CN 120030 GTCTACTCTTTTCAGAATTTGTTTGCAGAGAATAACCTTATTGCTTTTGTTTGTTACAGA

Hoga GTCTACTCTTTTCAGAATTTGTTTGCAGAGAATAACCTTATTGCTTTTGTTTGTTACAGA

Yellowstone GTCTACTCTTTTCAGAATTTGTTTGCAGAGAATAACCTTATTGCTTTTGTTTGTTACAGA

CN 119294 GTCTACTCTTTTCAGAATTTGTTTGCAGAGAATAACCTTATTGCTTTTGTTTGTTACAGA

* **********************************************************

CN 120025 CAGAACTAATGTTGAAGCTTGTTGAGAACCTCAAAGAAAAGGTTAGATATCTGATTCCAA

CN 119205 CAGAACTAATGTTGAAGCTTGTTGAGAACCTCAAAGAAAAGGTTAGATATCTGATTCCAA

CN 119243 CAGAACTAATGTTGAAGCTTGTTGAGAACCTCAAAGAAAAGGTTAGATATCTGATTCCAA

Joelle NCBI CAGAACTAATGTTGAAGCTTGTTGAGAACCTCAAAGAAAAGGTTAGATATCTGATTCCAA

Joelle AAFC CAGAACTAATGTTGAAGCTTGTTGAGAACCTCAAAGAAAAGGTTAGATATCTGATTCCAA

Joelle phyto CAGAACTAATGTTGAAGCTTGTTGAGAACCTCAAAGAAAAGGTTAGATATCTGATTCCAA

DH55 ref genome CAGAACTAATGTTGAAGCTTGTTGAGAACCTCAAAGAAAAGGTTAGATATCTGATTCCAA

CN 119300 CAGAACTAATGTTGAAGCTTGTTGAGAACCTCAAAGAAAAGGTTAGATATCTGATTCCAA

CAM 241 CAGAACTAATGTTGAAGCTTGTTGAGAACCTCAAAGAAAAGGTTAGATATCTGATTCCAA

CN 113754 CAGAACTAATGTTGAAGCTTGTTGAGAACCTCAAAGAAAAGGTTAGATATCTGATTCCAA

CAM 236 CAGAACTAATGTTGAAGCTTGTTGAGAACCTCAAAGAAAAGGTTAGATATCTGATTCCAA

CO46 NCBI CAGAACTAATGTTGAAGCTTGTTGAGAACCTCAAAGAAAAGGTTAGATATCTGATTCCAA

CN 120013 CAGAACTAATGTTGAAGCTTGTTGAGAACCTCAAAGAAAAGGTTAGATATCTGATTCCAA

Blaine Creek CAGAACTAATGTTGAAGCTTGTTGAGAACCTCAAAGAAAAGGTTAGATATCTGATTCCAA

09-CS0040 CAGAACTAATGTTGAAGCTTGTTGAGAACCTCAAAGAAAAGGTTAGATATCTGATTCCAA

17CS1133 CAGAACTAATGTTGAAGCTTGTTGAGAACCTCAAAGAAAAGGTTAGATATCTGATTCCAA

Jasper CAGAACTAATGTTGAAGCTTGTTGAGAACCTCAAAGAAAAGGTTAGATATCTGATTCCAA

CN 120017 CAGAACTAATGTTGAAGCTTGTTGAGAACCTCAAAGAAAAGGTTAGATATCTGATTCCAA

CN 120027 CAGAACTAATGTTGAAGCTTGTTGAGAACCTCAAAGAAAAGGTTAGATATCTGATTCCAA

CN 120030 CAGAACTAATGTTGAAGCTTGTTGAGAACCTCAAAGAAAAGGTTAGATATCTGATTCCAA

Hoga CAGAACTAATGTTGAAGCTTGTTGAGAACCTCAAAGAAAAGGTTAGATATCTGATTCCAA

Yellowstone CAGAACTAATGTTGAAGCTTGTTGAGAACCTCAAAGAAAAGGTTAGATATCTGATTCCAA

CN 119294 CAGAACTAATGTTGAAGCTTGTTGAGAACCTCAAAGAAAAGGTTAGATATCTGATTCCAA

************************************************************

CN 120025 GTTTAGAACATTTATCAGATATATACTCTAGGGTCTTTTCATTGTTTCTGTAAGTATGTT

CN 119205 GTTTAGAACAATTATCAGATATATACTCTAGGGTGTTTTCATTGTTTCTGTAAGTATGTT

CN 119243 GTTTAGAACAATTATCAGATATATACTCTAGGGTGTTTTCATTGTTTCTGTAAGTATGTT

Joelle NCBI GTTTAGAACAATTATCAGATATATACTCTAGGGTGTTTTCATTGTTTCTGTAAGTATGTT

Joelle AAFC GTTTAGAACAATTATCAGATATATACTCTAGGGTGTTTTCATTGTTTCTGTAAGTATGTT

Joelle phyto GTTTAGAACAATTATCAGATATATACTCTAGGGTGTTTTCATTGTTTCTGTAAGTATGTT

DH55 ref genome GTTTAGAACAATTATCAGATATATACTCTAGGGTGTTTTCATTGTTTCTGTAAGTATGTT

CN 119300 GTTTAGAACAATTATCAGATATATACTCTAGGGTGTTTTCATTGTTTCTGTAAGTATGTT

CAM 241 GTTTAGAACAATTATCAGATATATACTCTAGGGTGTTTTCATTGTTTCTGTAAGTATGTT

CN 113754 GTTTAGAACAATTATCAGATATATACTCTAGGGTGTTTTCATTGTTTCTGTAAGTATGTT

CAM 236 GTTTAGAACAATTATCAGATATATACTCTAGGGTGTTTTCATTGTTTCTGTAAGTATGTT

CO46 NCBI GTTTAGAACAATTATCAGATATATACTCTAGGGTGTTTTCATTGTTTCTGTAAGTATGTT

CN 120013 GTTTAGAACAATTATCAGATATATACTCTAGGGTGTTTTCATTGTTTCTGTAAGTATGTT

Blaine Creek GTTTAGAACAATTATCAGATATATACTCTAGGGTGTTTTCATTGTTTCTGTAAGTATGTT

09-CS0040 GTTTAGAACAATTATCAGATATATACTCTAGGGTGTTTTCATTGTTTCTGTAAGTATGTT

17CS1133 GTTTAGAACAATTATCAGATATATACTCTAGGGTGTTTTCATTGTTTCTGTAAGTATGTT

Jasper GTTTAGAACAATTATCAGATATATACTCTAGGGTGTTTTCATTGTTTCTGTAAGTATGTT

CN 120017 GTTTAGAACAATTATCAGATATATACTCTAGGGTGTTTTCATTGTTTCTGTAAGTATGTT

CN 120027 GTTTAGAACAATTATCAGATATATACTCTAGGGTGTTTTCATTGTTTCTGTAAGTATGTT

CN 120030 GTTTAGAACAATTATCAGATATATACTCTAGGGTGTTTTCATTGTTTCTGTAAGTATGTT

Hoga GTTTAGAACAATTATCAGATATATACTCTAGGGTGTTTTCATTGTTTCTGTAAGTATGTT

Yellowstone GTTTAGAACAATTATCAGATATATACTCTAGGGTGTTTTCATTGTTTCTGTAAGTATGTT

CN 119294 GTTTAGAACAATTATCAGATATATACTCTAGGGTGTTTTCATTGTTTCTGTAAGTATGTT

********** *********************** *************************

CN 120025 TAATGAGTTTTGCACACGTCTTTGCAACTTCTTCCCAATGCATATGTTGTGGATTTCAAA

CN 119205 TAATGAGTTTTGCACACGTCTTTGCAACTTCTTCCCAATGCATATGTTGTGGATTTCAAA

CN 119243 TAATGAGTTTTGCACACGTCTTTGCAACTTCTTCCCAATGCATATGTTGTGGATTTCAAA

Joelle NCBI TAATGAGTTTTGCACACGTCTTTGCAACTTCTTCCCAATGCATATGTTGTGGATTTCAAA

Joelle AAFC TAATGAGTTTTGCACACGTCTTTGCAACTTCTTCCCAATGCATATGTTGTGGATTTCAAA

Joelle phyto TAATGAGTTTTGCACACGTCTTTGCAACTTCTTCCCAATGCATATGTTGTGGATTTCAAA

DH55 ref genome TAATGAGTTTTGCACACGTCTTTGCAACTTCTTCCCAATGCATATGTTGTGGATTTCAAA

CN 119300 TAATGAGTTTTGCACACGTCTTTGCAACTTCTTCCCAATGCATATGTTGTGGATTTCAAA

CAM 241 TAATGAGTTTTGCACACGTCTTTGCAACTTCTTCCCAATGCATATGTTGTGGATTTCAAA

CN 113754 TAATGAGTTTTGCACACGTCTTTGCAACTTCTTCCCAATGCATATGTTGTGGATTTCAAA

CAM 236 TAATGAGTTTTGCACACGTCTTTGCAACTTCTTCCCAATGCATATGTTGTGGATTTCAAA

CO46 NCBI TAATGAGTTTTGCACACGTCTTTGCAACTTCTTCCCAATGCATATGTTGTGGATTTCAAA

CN 120013 TAATGAGTTTTGCACACGTCTTTGCAACTTCTTCCCAATGCATATGTTGTGGATTTCAAA

Blaine Creek TAATGAGTTTTGCACACGTCTTTGCAACTTCTTCCCAATGCATATGTTGTGGATTTCAAA

09-CS0040 TAATGAGTTTTGCACACGTCTTTGCAACTTCTTCCCAATGCATATGTTGTGGATTTCAAA

17CS1133 TAATGAGTTTTGCACACGTCTTTGCAACTTCTTCCCAATGCATATGTTGTGGATTTCAAA

Jasper TAATGAGTTTTGCACACGTCTTTGCAACTTCTTCCCAATGCATATGTTGTGGATTTCAAA

CN 120017 TAATGAGTTTTGCACACGTCTTTGCAACTTCTTCCCAATGCATATGTTGTGGATTTCAAA

CN 120027 TAATGAGTTTTGCACACGTCTTTGCAACTTCTTCCCAATGCATATGTTGTGGATTTCAAA

CN 120030 TAATGAGTTTTGCACACGTCTTTGCAACTTCTTCCCAATGCATATGTTGTGGATTTCAAA

Hoga TAATGAGTTTTGCACACGTCTTTGCAACTTCTTCCCAATGCATATGTTGTGGATTTCAAA

Yellowstone TAATGAGTTTTGCACACGTCTTTGCAACTTCTTCCCAATGCATATGTTGTGGATTTCAAA

CN 119294 TAATGAGTTTTGCACACGTCTTTGCAACTTCTTCCCAATGCATATGTTGTGGATTTCAAA

************************************************************

CN 120025 ATCTGAATTTGTTAAGTTGTGATTTGTGATTGCTGAACATGATGATCTTTAAAACAGGAA

CN 119205 ATCTGAATTTGTTAAATTGTGATTTGTGATTGCTGAACATGATGATCTTTAAAACAGGAA

CN 119243 ATCTGAATTTGTTAAATTGTGATTTGTGATTGCTGAACATGATGATCTTTAAAACAGGAA

Joelle NCBI ATCTGAATTTGTTAAATTGTGATTTGTGATTGCTGAACATGATGATCTTTAAAACAGGAA

Joelle AAFC ATCTGAATTTGTTAAATTGTGATTTGTGATTGCTGAACATGATGATCTTTAAAACAGGAA

Joelle phyto ATCTGAATTTGTTAAATTGTGATTTGTGATTGCTGAACATGATGATCTTTAAAACAGGAA

DH55 ref genome ATCTGAATTTGTTAAATTGTGATTTGTGATTGCTGAACATGATGATCTTTAAAACAGGAA

CN 119300 ATCTGAATTTGTTAAATTGTGATTTGTGATTGCTGAACATGATGATCTTTAAAACAGGAA

CAM 241 ATCTGAATTTGTTAAATTGTGATTTGTGATTGCTGAACATGATGATCTTTAAAACAGGAA

CN 113754 ATCTGAATTTGTTAAATTGTGATTTGTGATTGCTGAACATGATGATCTTTAAAACAGGAA

CAM 236 ATCTGAATTTGTTAAATTGTGATTTGTGATTGCTGAACATGATGATCTTTAAAACAGGAA

CO46 NCBI ATCTGAATTTGTTAAATTGTGATTTGTGATTGCTGAACATGATGATCTTTAAAACAGGAA

CN 120013 ATCTGAATTTGTTAAATTGTGATTTGTGATTGCTGAACATGATGATCTTTAAAACAGGAA

Blaine Creek ATCTGAATTTGTTAAATTGTGATTTGTGATTGCTGAACATGATGATCTTTAAAACAGGAA

09-CS0040 ATCTGAATTTGTTAAATTGTGATTTGTGATTGCTGAACATGATGATCTTTAAAACAGGAA

17CS1133 ATCTGAATTTGTTAAATTGTGATTTGTGATTGCTGAACATGATGATCTTTAAAACAGGAA

Jasper ATCTGAATTTGTTAAATTGTGATTTGTGATTGCTGAACATGATGATCTTTAAAACAGGAA

CN 120017 ATCTGAATTTGTTAAATTGTGATTTGTGATTGCTGAACATGATGATCTTTAAAACAGGAA

CN 120027 ATCTGAATTTGTTAAATTGTGATTTGTGATTGCTGAACATGATGATCTTTAAAACAGGAA

CN 120030 ATCTGAATTTGTTAAATTGTGATTTGTGATTGCTGAACATGATGATCTTTAAAACAGGAA

Hoga ATCTGAATTTGTTAAATTGTGATTTGTGATTGCTGAACATGATGATCTTTAAAACAGGAA

Yellowstone ATCTGAATTTGTTAAATTGTGATTTGTGATTGCTGAACATGATGATCTTTAAAACAGGAA

CN 119294 ATCTGAATTTGTTAAATTGTGATTTGTGATTGCTGAACATGATGATCTTTAAAACAGGAA

*************** ********************************************

CN 120025 AAATTGCTGAAAGAAGAGAACCAGGTTTTGGCTAGCCAGGTAACGAAAGCTACTTTTTCT

CN 119205 AAATTGCTGAAAGAAGAGAACCAGGTTTTGGCTAGCCAGGTAACGAAAGCTACTTTTTCT

CN 119243 AAATTGCTGAAAGAAGAGAACCAGGTTTTGGCTAGCCAGGTAACGAAAGCTACTTTTTCT

Joelle NCBI AAATTGCTGAAAGAAGAGAACCAGGTTTTGGCTAGCCAGGTAACGAAAGCTACTTTTTCT

Joelle AAFC AAATTGCTGAAAGAAGAGAACCAGGTTTTGGCTAGCCAGGTAACGAAAGCTACTTTTTCT

Joelle phyto AAATTGCTGAAAGAAGAGAACCAGGTTTTGGCTAGCCAGGTAACGAAAGCTACTTTTTCT

DH55 ref genome AAATTGCTGAAAGAAGAGAACCAGGTTTTGGCTAGCCAGGTAACGAAAGCTACTTTTTCT

CN 119300 AAATTGCTGAAAGAAGAGAACCAGGTTTTGGCTAGCCAGGTAACGAAAGCTACTTTTTCT

CAM 241 AAATTGCTGAAAGAAGAGAACCAGGTTTTGGCTAGCCAGGTAACGAAAGCTACTTTTTCT

CN 113754 AAATTGCTGAAAGAAGAGAACCAGGTTTTGGCTAGCCAGGTAACGAAAGCTACTTTTTCT

CAM 236 AAATTGCTGAAAGAAGAGAACCAGGTTTTGGCTAGCCAGGTAACGAAAGCTACTTTTTCT

CO46 NCBI AAATTGCTGAAAGAAGAGAACCAGGTTTTGGCTAGCCAGGTAACGAAAGCTACTTTTTCT

CN 120013 AAATTGCTGAAAGAAGAGAACCAGGTTTTGGCTAGCCAGGTAACGAAAGCTACTTTTTCT

Blaine Creek AAATTGCTGAAAGAAGAGAACCAGGTTTTGGCTAGCCAGGTAACGAAAGCTACTTTTTCT

09-CS0040 AAATTGCTGAAAGAAGAGAACCAGGTTTTGGCTAGCCAGGTAACGAAAGCTACTTTTTCT

17CS1133 AAATTGCTGAAAGAAGAGAACCAGGTTTTGGCTAGCCAGGTAACGAAAGCTACTTTTTCT

Jasper AAATTGCTGAAAGAAGAGAACCAGGTTTTGGCTAGCCAGGTAACGAAAGCTACTTTTTCT

CN 120017 AAATTGCTGAAAGAAGAGAACCAGGTTTTGGCTAGCCAGGTAACGAAAGCTACTTTTTCT

CN 120027 AAATTGCTGAAAGAAGAGAACCAGGTTTTGGCTAGCCAGGTAACGAAAGCTACTTTTTCT

CN 120030 AAATTGCTGAAAGAAGAGAACCAGGTTTTGGCTAGCCAGGTAACGAAAGCTACTTTTTCT

Hoga AAATTGCTGAAAGAAGAGAACCAGGTTTTGGCTAGCCAGGTAACGAAAGCTACTTTTTCT

Yellowstone AAATTGCTGAAAGAAGAGAACCAGGTTTTGGCTAGCCAGGTAACGAAAGCTACTTTTTCT

CN 119294 AAATTGCTGAAAGAAGAGAACCAGGTTTTGGCTAGCCAGGTAACGAAAGCTACTTTTTCT

************************************************************

CN 120025 ATATATACGCATACCTAATTAAGCCATTCTTTCCACTTTAAGCATTTACTAGTTAAAACT

CN 119205 ATATATACGCATACCTAATTAAGCCATTCTTTCCACTTTAAGCATTTACTAGTTAAAACT

CN 119243 ATATATACGCATACCTAATTAAGCCATTCTTTCCACTTTAAGCATTTACTAGTTAAAACT

Joelle NCBI ATATATACGCATACCTAATTAAGCCATTCTTTCCACTTTAAGCATTTACTAGTTAAAACT

Joelle AAFC ATATATACGCATACCTAATTAAGCCATTCTTTCCACTTTAAGCATTTACTAGTTAAAACT

Joelle phyto ATATATACGCATACCTAATTAAGCCATTCTTTCCACTTTAAGCATTTACTAGTTAAAACT

DH55 ref genome ATATATACGCATACCTAATTAAGCCATTCTTTCCACTTTAAGCATTTACTAGTTAAAACT

CN 119300 ATATATACGCATACCTAATTAAGCCATTCTTTCCACTTTAAGCATTTACTAGTTAAAACT

CAM 241 ATATATACGCATACCTAATTAAGCCATTCTTTCCACTTTAAGCATTTACTAGTTAAAACT

CN 113754 ATATATACGCATACCTAATTAAGCCATTCTTTCCACTTTAAGCATTTACTAGTTAAAACT

CAM 236 ATATATACGCATACCTAATTAAGCCATTCTTTCCACTTTAAGCATTTACTAGTTAAAACT

CO46 NCBI ATATATACGCATACCTAATTAAGCCATTCTTTCCACTTTAAGCATTTACTAGTTAAAACT

CN 120013 ATATATACGCATACCTAATTAAGCCATTCTTTCCACTTTAAGCATTTACTAGTTAAAACT

Blaine Creek ATATATACGCATACCTAATTAAGCCATTCTTTCCACTTTAAGCATTTACTAGTTAAAACT

09-CS0040 ATATATACGCATACCTAATTAAGCCATTCTTTCCACTTTAAGCATTTACTAGTTAAAACT

17CS1133 ATATATACGCATACCTAATTAAGCCATTCTTTCCACTTTAAGCATTTACTAGTTAAAACT

Jasper ATATATACGCATACCTAATTAAGCCATTCTTTCCACTTTAAGCATTTACTAGTTAAAACT

CN 120017 ATATATACGCATACCTAATTAAGCCATTCTTTCCACTTTAAGCATTTACTAGTTAAAACT

CN 120027 ATATATACGCATACCTAATTAAGCCATTCTTTCCACTTTAAGCATTTACTAGTTAAAACT

CN 120030 ATATATACGCATACCTAATTAAGCCATTCTTTCCACTTTAAGCATTTACTAGTTAAAACT

Hoga ATATATACGCATACCTAATTAAGCCATTCTTTCCACTTTAAGCATTTACTAGTTAAAACT

Yellowstone ATATATACGCATACCTAATTAAGCCATTCTTTCCACTTTAAGCATTTACTAGTTAAAACT

CN 119294 ATATATACGCATACCTAATTAAGCCATTCTTTCCACTTTAAGCATTTACTAGTTAAAACT

************************************************************

CN 120025 TCTCTGTCTTGTGTTCTAGAAAAATTATTTCTCAAGATTAGGGTTGTTGGTAGATTAGGT

CN 119205 TCTCTGTCTTGTGTTCTAGAAAAATTATTTCTCAAGATTAGGGTTGTTGGTAGATTAGGT

CN 119243 TCTCTGTCTTGTGTTCTAGAAAAATTATTTCTCAAGATTAGGGTTGTTGGTAGATTAGGT

Joelle NCBI TCTCTGTCTTGTGTTCTAGAAAAATTATTTCTCAAGATTAGGGTTGTTGGTAGATTAGGT

Joelle AAFC TCTCTGTCTTGTGTTCTAGAAAAATTATTTCTCAAGATTAGGGTTGTTGGTAGATTAGGT

Joelle phyto TCTCTGTCTTGTGTTCTAGAAAAATTATTTCTCAAGATTAGGGTTGTTGGTAGATTAGGT

DH55 ref genome TCTCTGTCTTGTGTTCTAGAAAAATTATTTCTCAAGATTAGGGTTGTTGGTAGATTAGGT

CN 119300 TCTCTGTCTTGTGTTCTAGAAAAATTATTTCTCAAGATTAGGGTTGTTGGTAGATTAGGT

CAM 241 TCTCTGTCTTGTGTTCTAGAAAAATTATTTCTCAAGATTAGGGTTGTTGGTAGATTAGGT

CN 113754 TCTCTGTCTTGTGTTCTAGAAAAATTATTTCTCAAGATTAGGGTTGTTGGTAGATTAGGT

CAM 236 TCTCTGTCTTGTGTTCTAGAAAAATTATTTCTCAAGATTAGGGTTGTTGGTAGATTAGGT

CO46 NCBI TCTCTGTCTTGTGTTCTAGAAAAATTATTTCTCAAGATTAGGGTTGTTGGTAGATTAGGT

CN 120013 TCTCTGTCTTGTGTTCTAGAAAAATTATTTCTCAAGATTAGGGTTGTTGGTAGATTAGGT

Blaine Creek TCTCTGTCTTGTGTTCTAGAAAAATTATTTCTCAAGATTAGGGTTGTTGGTAGATTAGGT

09-CS0040 TCTCTGTCTTGTGTTCTAGAAAAATTATTTCTCAAGATTAGGGTTGTTGGTAGATTAGGT

17CS1133 TCTCTGTCTTGTGTTCTAGAAAAATTATTTCTCAAGATTAGGGTTGTTGGTAGATTAGGT

Jasper TCTCTGTCTTGTGTTCTAGAAAAATTATTTCTCAAGATTAGGGTTGTTGGTAGATTAGGT

CN 120017 TCTCTGTCTTGTGTTCTAGAAAAATTATTTCTCAAGATTAGGGTTGTTGGTAGATTAGGT

CN 120027 TCTCTGTCTTGTGTTCTAGAAAAATTATTTCTCAAGATTAGGGTTGTTGGTAGATTAGGT

CN 120030 TCTCTGTCTTGTGTTCTAGAAAAATTATTTCTCAAGATTAGGGTTGTTGGTAGATTAGGT

Hoga TCTCTGTCTTGTGTTCTAGAAAAATTATTTCTCAAGATTAGGGTTGTTGGTAGATTAGGT

Yellowstone TCTCTGTCTTGTGTTCTAGAAAAATTATTTCTCAAGATTAGGGTTGTTGGTAGATTAGGT

CN 119294 TCTCTGTCTTGTGTTCTAGAAAAATTATTTCTCAAGATTAGGGTTGTTGGTAGATTAGGT

************************************************************

CN 120025 ATTAGGGTTTGTGAGATTATTACTGCTGAATAAGGACAGAAATTTGATTCGGTCT-GAAA

CN 119205 ATTAGGGTTTGTGAGATTATTACTGCTGAATAAGGACAGAAATTTGATTCGGTCTGGAAA

CN 119243 ATTAGAGTTTGTGAGATTATTACTGCTGAATAAGGACAGAAATTTGATTCGGTCTGGAAA

Joelle NCBI ATTAGAGTTTGTGAGATTATTACTGCTGAATAAGGACAGAAATTTGATTCGGTCTGGAAA

Joelle AAFC ATTAGAGTTTGTGAGATTATTACTGCTGAATAAGGACAGAAATTTGATTCGGTCTGGAAA

Joelle phyto ATTAGAGTTTGTGAGATTATTACTGCTGAATAAGGACAGAAATTTGATTCGGTCTGGAAA

DH55 ref genome ATTAGGGTTTGTGAGATTATTACTGCTGAATAAGGACAGAAATTTGATTCGGTCTGGAAA

CN 119300 ATTAGGGTTTGTGAGATTATTACTGCTGAATAAGGACAGAAATTTGATTCGGTCTGGAAA

CAM 241 ATTAGGGTTTGTGAGATTATTACTGCTGAATAAGGACAGAAATTTGATTCGGTCTGGAAA

CN 113754 ATTAGGGTTTGTGAGATTATTACTGCTGAATAAGGACAGAAATTTGATTCGGTCTGGAAA

CAM 236 ATTAGGGTTTGTGAGATTATTACTGCTGAATAAGGACAGAAATTTGATTCGGTCTGGAAA

CO46 NCBI ATTAGGGTTTGTGAGATTATTACTGCTGAATAAGGACAGAAATTTGATTCGGTCTGGAAA

CN 120013 ATTAGGGTTTGTGAGATTATTACTGCTGAATAAGGACAGAAATTTGATTCGGTCTGGAAA

Blaine Creek ATTAGGGTTTGTGAGATTATTACTGCTGAATAAGGACAGAAATTTGATTCGGTCTGGAAA

09-CS0040 ATTAGGGTTTGTGAGATTATTACTGCTGAATAAGGACAGAAATTTGATTCGGTCTGGAAA

17CS1133 ATTAGGGTTTGTGAGATTATTACTGCTGAATAAGGACAGAAATTTGATTCGGTCTGGAAA

Jasper ATTAGGGTTTGTGAGATTATTACTGCTGAATAAGGACAGAAATTTGATTCGGTCTGGAAA

CN 120017 ATTAGGGTTTGTGAGATTATTACTGCTGAATAAGGACAGAAATTTGATTCGGTCTGGAAA

CN 120027 ATTAGGGTTTGTGAGATTATTACTGCTGAATAAGGACAGAAATTTGATTCGGTCTGGAAA

CN 120030 ATTAGGGTTTGTGAGATTATTACTGCTGAATAAGGACAGAAATTTGATTCGGTCTGGAAA

Hoga ATTAGGGTTTGTGAGATTATTACTGCTGAATAAGGACAGAAATTTGATTCGGTCTGGAAA

Yellowstone ATTAGGGTTTGTGAGATTATTACTGCTGAATAAGGACAGAAATTTGATTCGGTCTGGAAA

CN 119294 ATTAGGGTTTGTGAGATTATTACTGCTGAATAAGGACAGAAATTTGATTCGGTCTGGAAA

***** ************************************************* ****

CN 120025 ATTTTGATTTGGATTTTAGAATGACAAATGAAAAGTTTAATATAGTTTTAAGATAGTAAT

CN 119205 ATTTTGATTTGGATTTTAGAATGACAAATGAAAAGTTTAATATAGTTTTAAGATAGTAAT

CN 119243 ATTTTGATTTGGATTTTAGAATGACAAATGAAAAGTTTAATATAGTTTTAAGATAGTAAT

Joelle NCBI ATTTTGATTTGGATTTTAGAATGACAAATGAAAAGTTTAATATAGTTTTAAGATAGTAAT

Joelle AAFC ATTTTGATTTGGATTTTAGAATGACAAATGAAAAGTTTAATATAGTTTTAAGATAGTAAT

Joelle phyto ATTTTGATTTGGATTTTAGAATGACAAATGAAAAGTTTAATATAGTTTTAAGATAGTAAT

DH55 ref genome ATTTTGATTTGGATTTTAGAATGACAAATGAAAAGTTTAATATAGTTTTAAGATAGTAAT

CN 119300 ATTTTGATTTGGATTTTAGAATGACAAATGAAAAGTTTAATATAGTTTTAAGATAGTAAT

CAM 241 ATTTTGATTTGGATTTTAGAATGACAAATGAAAAGTTTAATATAGTTTTAAGATAGTAAT

CN 113754 ATTTTGATTTGGATTTTAGAATGACAAATGAAAAGTTTAATATAGTTTTAAGATAGTAAT

CAM 236 ATTTTGATTTGGATTTTAGAATGACAAATGAAAAGTTTAATATAGTTTTAAGATAGTAAT

CO46 NCBI ATTTTGATTTGGATTTTAGAATGACAAATGAAAAGTTTAATATAGTTTTAAGATAGTAAT

CN 120013 ATTTTGATTTGGATTTTAGAATGACAAATGAAAAGTTTAATATAGTTTTAAGATAGTAAT

Blaine Creek ATTTTGATTTGGATTTTAGAATGACAAATGAAAAGTTTAATATAGTTTTAAGATAGTAAT

09-CS0040 ATTTTGATTTGGATTTTAGAATGACAAATGAAAAGTTTAATATAGTTTTAAGATAGTAAT

17CS1133 ATTTTGATTTGGATTTTAGAATGACAAATGAAAAGTTTAATATAGTTTTAAGATAGTAAT

Jasper ATTTTGATTTGGATTTTAGAATGACAAATGAAAAGTTTAATATAGTTTTAAGATAGTAAT

CN 120017 ATTTTGATTTGGATTTTAGAATGACAAATGAAAAGTTTAATATAGTTTTAAGATAGTAAT

CN 120027 ATTTTGATTTGGATTTTAGAATGACAAATGAAAAGTTTAATATAGTTTTAAGATAGTAAT

CN 120030 ATTTTGATTTGGATTTTAGAATGACAAATGAAAAGTTTAATATAGTTTTAAGATAGTAAT

Hoga ATTTTGATTTGGATTTTAGAATGACAAATGAAAAGTTTAATATAGTTTTAAGATAGTAAT

Yellowstone ATTTTGATTTGGATTTTAGAATGACAAATGAAAAGTTTAATATAGTTTTAAGATAGTAAT

CN 119294 ATTTTGATTTGGATTTTAGAATGACAAATGAAAAGTTTAATATAGTTTTAAGATAGTAAT

************************************************************

CN 120025 TTATTTATAAAAGACACACAAACAAACAAAATTCATGAACAGTATAAATTCATTATTACA

CN 119205 TTATTTATAAAAGACACACAAACAAACAAAATTCATGAACAGTATAAATTCATTATTACA

CN 119243 TTATTTATAAAAGACACACAAACAAACAAAATTCATGAACAGTATAAATTCATTATTACA

Joelle NCBI TTATTTATAAAAGACACACAAACAAACAAAATTCATGAACAGTATAAATTCATTATTACA

Joelle AAFC TTATTTATAAAAGACACACAAACAAACAAAATTCATGAACAGTATAAATTCATTATTACA

Joelle phyto TTATTTATAAAAGACACACAAACAAACAAAATTCATGAACAGTATAAATTCATTATTACA

DH55 ref genome TTATTTATAAAAGACACACAAACAAACAAAATTCATGAACAGTATAAATTCATTATTACA

CN 119300 TTATTTATAAAAGACACACAAACAAACAAAATTCATGAACAGTATAAATTCATTATTACA

CAM 241 TTATTTATAAAAGACACACAAACAAACAAAATTCATGAACAGTATAAATTCATTATTACA

CN 113754 TTATTTATAAAAGACACACAAACAAACAAAATTCATGAACAGTATAAATTCATTATTACA

CAM 236 TTATTTATAAAAGACACACAAACAAACAAAATTCATGAACAGTATAAATTCATTATTACA

CO46 NCBI TTATTTATAAAAGACACACAAACAAACAAAATTCATGAACAGTATAAATTCATTATTACA

CN 120013 TTATTTATAAAAGACACACAAACAAACAAAATTCATGAACAGTATAAATTCATTATTACA

Blaine Creek TTATTTATAAAAGACACACAAACAAACAAAATTCATGAACAGTATAAATTCATTATTACA

09-CS0040 TTATTTATAAAAGACACACAAACAAACAAAATTCATGAACAGTATAAATTCATTATTACA

17CS1133 TTATTTATAAAAGACACACAAACAAACAAAATTCATGAACAGTATAAATTCATTATTACA

Jasper TTATTTATAAAAGACACACAAACAAACAAAATTCATGAACAGTATAAATTCATTATTACA

CN 120017 TTATTTATAAAAGACACACAAACAAACAAAATTCATGAACAGTATAAATTCATTATTACA

CN 120027 TTATTTATAAAAGACACACAAACAAACAAAATTCATGAACAGTATAAATTCATTATTACA

CN 120030 TTATTTATAAAAGACACACAAACAAACAAAATTCATGAACAGTATAAATTCATTATTACA

Hoga TTATTTATAAAAGACACACAAACAAACAAAATTCATGAACAGTATAAATTCATTATTACA

Yellowstone TTATTTATAAAAGACACACAAACAAACAAAATTCATGAACAGTATAAATTCATTATTACA

CN 119294 TTATTTATAAAAGACACACAAACAAACAAAATTCATGAACAGTATAAATTCATTATTACA

************************************************************

CN 120025 TAATAATTTGTTCAGTAAAATATAGTTCTTTGTAACGATTATTGCAATATCATTGTACTA

CN 119205 TAATAATTTGTTCAGTAAAATATAGTTCTTTGTAACGATTATTGCAATATCATTGTACTA

CN 119243 TAATAATTTGTTCAGTAAAATATAGTTCTTTGTAACGATTATTGCAATATCATTGTACTA

Joelle NCBI TAATAATTTGTTCAGTAAAATATAGTTCTTTGTAACGATTATTGCAATATCATTGTACTA

Joelle AAFC TAATAATTTGTTCAGTAAAATATAGTTCTTTGTAACGATTATTGCAATATCATTGTACTA

Joelle phyto TAATAATTTGTTCAGTAAAATATAGTTCTTTGTAACGATTATTGCAATATCATTGTACTA

DH55 ref genome TAATAATTTGTTCAGTAAAATATAGTTCTTTGTAACGATTATTGCAATATCATTGTACTA

CN 119300 TAATAATTTGTTCAGTAAAATATAGTTCTTTGTAACGATTATTGCAATATCATTGTACTA

CAM 241 TAATAATTTGTTCAGTAAAATATAGTTCTTTGTAACGATTATTGCAATATCATTGTACTA

CN 113754 TAATAATTTGTTCAGTAAAATATAGTTCTTTGTAACGATTATTGCAATATCATTGTACTA

CAM 236 TAATAATTTGTTCAGTAAAATATAGTTCTTTGTAACGATTATTGCAATATCATTGTACTA

CO46 NCBI TAATAATTTGTTCAGTAAAATATAGTTCTTTGTAACGATTATTGCAATATCATTGTACTA

CN 120013 TAATAATTTGTTCAGTAAAATATAGTTCTTTGTAACGATTATTGCAATATCATTGTACTA

Blaine Creek TAATAATTTGTTCAGTAAAATATAGTTCTTTGTAACGATTATTGCAATATCATTGTACTA

09-CS0040 TAATAATTTGTTCAGTAAAATATAGTTCTTTGTAACGATTATTGCAATATCATTGTACTA

17CS1133 TAATAATTTGTTCAGTAAAATATAGTTCTTTGTAACGATTATTGCAATATCATTGTACTA

Jasper TAATAATTTGTTCAGTAAAATATAGTTCTTTGTAACGATTATTGCAATATCATTGTACTA

CN 120017 TAATAATTTGTTCAGTAAAATATAGTTCTTTGTAACGATTATTGCAATATCATTGTACTA

CN 120027 TAATAATTTGTTCAGTAAAATATAGTTCTTTGTAACGATTATTGCAATATCATTGTACTA

CN 120030 TAATAATTTGTTCAGTAAAATATAGTTCTTTGTAACGATTATTGCAATATCATTGTACTA

Hoga TAATAATTTGTTCAGTAAAATATAGTTCTTTGTAACGATTATTGCAATATCATTGTACTA

Yellowstone TAATAATTTGTTCAGTAAAATATAGTTCTTTGTAACGATTATTGCAATATCATTGTACTA

CN 119294 TAATAATTTGTTCAGTAAAATATAGTTCTTTGTAACGATTATTGCAATATCATTGTACTA

************************************************************

CN 120025 AAATAATATATACTTTTCTGTTGGTGGAAATAATATTAGGTTTTGGTTGTTCTTTTTCTG

CN 119205 AAATAATATATACTTTTCTGTTGGTGGAAATAATATTAGGTTTTGGTTGTTCTTTTTCTG

CN 119243 AAATAATATATACTTTTCTGTTGGTGGAAATAATATTAGGTTTTGGTTGTTCTTTTTCTG

Joelle NCBI AAATAATATATACTTTTCTGTTGGTGGAAATAATATTAGGTTTTGGTTGTTCTTTTTCTG

Joelle AAFC AAATAATATATACTTTTCTGTTGGTGGAAATAATATTAGGTTTTGGTTGTTCTTTTTCTG

Joelle phyto AAATAATATATACTTTTCTGTTGGTGGAAATAATATTAGGTTTTGGTTGTTCTTTTTCTG

DH55 ref genome AAATAATATATACTTTTCTGTTGGTGGAAATAATATTAGGTTTTGGTTGTTCTTTTTCTG

CN 119300 AAATAATATATACTTTTCTGTTGGTGGAAATAATATTAGGTTTTGGTTGTTCTTTTTCTG

CAM 241 AAATAATATATACTTTTCTGTTGGTGGAAATAATATTAGGTTTTGGTTGTTCTTTTTCTG

CN 113754 AAATAATATATACTTTTCTGTTGGTGGAAATAATATTAGGTTTTGGTTGTTCTTTTTCTG

CAM 236 AAATAATATATACTTTTCTGTTGGTGGAAATAATATTAGGTTTTGGTTGTTCTTTTTCTG

CO46 NCBI AAATAATATATACTTTTCTGTTGGTGGAAATAATATTAGGTTTTGGTTGTTCTTTTTCTG

CN 120013 AAATAATATATACTTTTCTGTTGGTGGAAATAATATTAGGTTTTGGTTGTTCTTTTTCTG

Blaine Creek AAATAATATATACTTTTCTGTTGGTGGAAATAATATTAGGTTTTGGTTGTTCTTTTTCTG

09-CS0040 AAATAATATATACTTTTCTGTTGGTGGAAATAATATTAGGTTTTGGTTGTTCTTTTTCTG

17CS1133 AAATAATATATACTTTTCTGTTGGTGGAAATAATATTAGGTTTTGGTTGTTCTTTTTCTG

Jasper AAATAATATATACTTTTCTGTTGGTGGAAATAATATTAGGTTTTGGTTGTTCTTTTTCTG

CN 120017 AAATAATATATACTTTTCTGTTGGTGGAAATAATATTAGGTTTTGGTTGTTCTTTTTCTG

CN 120027 AAATAATATATACTTTTCTGTTGGTGGAAATAATATTAGGTTTTGGTTGTTCTTTTTCTG

CN 120030 AAATAATATATACTTTTCTGTTGGTGGAAATAATATTAGGTTTTGGTTGTTCTTTTTCTG

Hoga AAATAATATATACTTTTCTGTTGGTGGAAATAATATTAGGTTTTGGTTGTTCTTTTTCTG

Yellowstone AAATAATATATACTTTTCTGTTGGTGGAAATAATATTAGGTTTTGGTTGTTCTTTTTCTG

CN 119294 AAATAATATATACTTTTCTGTTGGTGGAAATAATATTAGGTTTTGGTTGTTCTTTTTCTG

************************************************************

CN 120025 GATTTGGGATAAAGGTTTTAGTTAGGTTTTGGTTCAGTTTGAGATTTACGAACGGATTAA

CN 119205 GATTTGGGATAAAGGTTTTAGTTAGGTTTTGGTTCAGTTTGAGATTTACGAACGGATTAA

CN 119243 GATTTGGGATAAAGGTTTTAGTTAGGTTTTGGTTCAGTTTGAGATTTACGAACGGATTAA

Joelle NCBI GATTTGGGATAAAGGTTTTAGTTAGGTTTTGGTTCAGTTTGAGATTTACGAACGGATTAA

Joelle AAFC GATTTGGGATAAAGGTTTTAGTTAGGTTTTGGTTCAGTTTGAGATTTACGAACGGATTAA

Joelle phyto GATTTGGGATAAAGGTTTTAGTTAGGTTTTGGTTCAGTTTGAGATTTACGAACGGATTAA

DH55 ref genome GATTTGGGATAAAGGTTTTAGTTAGGTTTTGGTTCAGTTTGAGATTTACGAACGGATTAA

CN 119300 GATTTGGGATAAAGGTTTTAGTTAGGTTTTGGTTCAGTTTGAGATTTACGAACGGATTAA

CAM 241 GATTTGGGATAAAGGTTTTAGTTAGGTTTTGGTTCAGTTTGAGATTTACGAACGGATTAA

CN 113754 GATTTGGGATAAAGGTTTTAGTTAGGTTTTGGTTCAGTTTGAGATTTACGAACGGATTAA

CAM 236 GATTTGGGATAAAGGTTTTAGTTAGGTTTTGGTTCAGTTTGAGATTTACGAACGGATTAA

CO46 NCBI GATTTGGGATAAAGGTTTTAGTTAGGTTTTGGTTCAGTTTGAGATTTACGAACGGATTAA

CN 120013 GATTTGGGATAAAGGTTTTAGTTAGGTTTTGGTTCAGTTTGAGATTTACGAACGGATTAA

Blaine Creek GATTTGGGATAAAGGTTTTAGTTAGGTTTTGGTTCAGTTTGAGATTTACGAACGGATTAA

09-CS0040 GATTTGGGATAAAGGTTTTAGTTAGGTTTTGGTTCAGTTTGAGATTTACGAACGGATTAA

17CS1133 GATTTGGGATAAAGGTTTTAGTTAGGTTTTGGTTCAGTTTGAGATTTACGAACGGATTAA

Jasper GATTTGGGATAAAGGTTTTAGTTAGGTTTTGGTTCAGTTTGAGATTTACGAACGGATTAA

CN 120017 GATTTGGGATAAAGGTTTTAGTTAGGTTTTGGTTCAGTTTGAGATTTACGAACGGATTAA

CN 120027 GATTTGGGATAAAGGTTTTAGTTAGGTTTTGGTTCAGTTTGAGATTTACGAACGGATTAA

CN 120030 GATTTGGGATAAAGGTTTTAGTTAGGTTTTGGTTCAGTTTGAGATTTACGAACGGATTAA

Hoga GATTTGGGATAAAGGTTTTAGTTAGGTTTTGGTTCAGTTTGAGATTTACGAACGGATTAA

Yellowstone GATTTGGGATAAAGGTTTTAGTTAGGTTTTGGTTCAGTTTGAGATTTACGAACGGATTAA

CN 119294 GATTTGGGATAAAGGTTTTAGTTAGGTTTTGGTTCAGTTTGAGATTTACGAACGGATTAA

************************************************************

CN 120025 TTTTTTGTGCGTCATGGTTAAGGTTTGGATCCAAGTGTCAAGTAGTCTAGCTACTTTTAC

CN 119205 TTTTTTGTGCGTCATGGTTAAGGTTTGGATCCAAGTGTCAAGTAGTCTAGCTACTTTTAC

CN 119243 TTTTTTGTGCGTCATGGTTAAGGTTTGGATCCAAGTGTCAAGTAGTCTAGCTACTTTTAC

Joelle NCBI TTTTTTGTGCGTCATGGTTAAGGTTTGGATCCAAGTGTCAAGTAGTCTAGCTACTTTTAC

Joelle AAFC TTTTTTGTGCGTCATGGTTAAGGTTTGGATCCAAGTGTCAAGTAGTCTAGCTACTTTTAC

Joelle phyto TTTTTTGTGCGTCATGGTTAAGGTTTGGATCCAAGTGTCAAGTAGTCTAGCTACTTTTAC

DH55 ref genome TTTTTTGTGCGTCATGGTTAAGGTTTGGATCCAAGTGTCAAGTAGTCTAGCTACTTTTAC

CN 119300 TTTTTTGTGCGTCATGGTTAAGGTTTGGATCCAAGTGTCAAGTAGTCTAGCTACTTTTAC

CAM 241 TTTTTTGTGCGTCATGGTTAAGGTTTGGATCCAAGTGTCAAGTAGTCTAGCTACTTTTAC

CN 113754 TTTTTTGTGCGTCATGGTTAAGGTTTGGATCCAAGTGTCAAGTAGTCTAGCTACTTTTAC

CAM 236 TTTTTTGTGCGTCATGGTTAAGGTTTGGATCCAAGTGTCAAGTAGTCTAGCTACTTTTAC

CO46 NCBI TTTTTTGTGCGTCATGGTTAAGGTTTGGATCCAAGTGTCAAGTAGTCTAGCTACTTTTAC

CN 120013 TTTTTTGTGCGTCATGGTTAAGGTTTGGATCCAAGTGTCAAGTAGTCTAGCTACTTTTAC

Blaine Creek TTTTTTGTGCGTCATGGTTAAGGTTTGGATCCAAGTGTCAAGTAGTCTAGCTACTTTTAC

09-CS0040 TTTTTTGTGCGTCATGGTTAAGGTTTGGATCCAAGTGTCAAGTAGTCTAGCTACTTTTAC

17CS1133 TTTTTTGTGCGTCATGGTTAAGGTTTGGATCCAAGTGTCAAGTAGTCTAGCTACTTTTAC

Jasper TTTTTTGTGCGTCATGGTTAAGGTTTGGATCCAAGTGTCAAGTAGTCTAGCTACTTTTAC

CN 120017 TTTTTTGTGCGTCATGGTTAAGGTTTGGATCCAAGTGTCAAGTAGTCTAGCTACTTTTAC

CN 120027 TTTTTTGTGCGTCATGGTTAAGGTTTGGATCCAAGTGTCAAGTAGTCTAGCTACTTTTAC

CN 120030 TTTTTTGTGCGTCATGGTTAAGGTTTGGATCCAAGTGTCAAGTAGTCTAGCTACTTTTAC

Hoga TTTTTTGTGCGTCATGGTTAAGGTTTGGATCCAAGTGTCAAGTAGTCTAGCTACTTTTAC

Yellowstone TTTTTTGTGCGTCATGGTTAAGGTTTGGATCCAAGTGTCAAGTAGTCTAGCTACTTTTAC

CN 119294 TTTTTTGTGCGTCATGGTTAAGGTTTGGATCCAAGTGTCAAGTAGTCTAGCTACTTTTAC

************************************************************

CN 120025 ATCCTCAAGGTTAAATTATAAACAGGGAAGTAGTCTAAAACAGACAAATTATGGAAGCAA

CN 119205 ATCCTCAAGGTTAAATTATAAACAGGGAAGTAGTCTAAAACAGACAAATTATGGAAGCAA

CN 119243 ATCCTCAAGGTTAAATTATAAACAGGGAAGTAGTCTAAAACAGACAAATTATGGAAGCAA

Joelle NCBI ATCCTCAAGGTTAAATTATAAACAGGGAAGTAGTCTAAAACAGACAAATTATGGAAGCAA

Joelle AAFC ATCCTCAAGGTTAAATTATAAACAGGGAAGTAGTCTAAAACAGACAAATTATGGAAGCAA

Joelle phyto ATCCTCAAGGTTAAATTATAAACAGGGAAGTAGTCTAAAACAGACAAATTATGGAAGCAA

DH55 ref genome ATCCTCAAGGTTAAATTATAAACAGGGAAGTAGTCTAAAACAGACAAATTATGGAAGCAA

CN 119300 ATCCTCAAGGTTAAATTATAAACAGGGAAGTAGTCTAAAACAGACAAATTATGGAAGCAA

CAM 241 ATCCTCAAGGTTAAATTATAAACAGGGAAGTAGTCTAAAACAGACAAATTATGGAAGCAA

CN 113754 ATCCTCAAGGTTAAATTATAAACAGGGAAGTAGTCTAAAACAGACAAATTATGGAAGCAA

CAM 236 ATCCTCAAGGTTAAATTATAAACAGGGAAGTAGTCTAAAACAGACAAATTATGGAAGCAA

CO46 NCBI ATCCTCAAGGTTAAATTATAAACAGGGAAGTAGTCTAAAACAGACAAATTATGGAAGCAA

CN 120013 ATCCTCAAGGTTAAATTATAAACAGGGAAGTAGTCTAAAACAGACAAATTATGGAAGCAA

Blaine Creek ATCCTCAAGGTTAAATTATAAACAGGGAAGTAGTCTAAAACAGACAAATTATGGAAGCAA

09-CS0040 ATCCTCAAGGTTAAATTATAAACAGGGAAGTAGTCTAAAACAGACAAATTATGGAAGCAA

17CS1133 ATCCTCAAGGTTAAATTATAAACAGGGAAGTAGTCTAAAACAGACAAATTATGGAAGCAA

Jasper ATCCTCAAGGTTAAATTATAAACAGGGAAGTAGTCTAAAACAGACAAATTATGGAAGCAA

CN 120017 ATCCTCAAGGTTAAATTATAAACAGGGAAGTAGTCTAAAACAGACAAATTATGGAAGCAA

CN 120027 ATCCTCAAGGTTAAATTATAAACAGGGAAGTAGTCTAAAACAGACAAATTATGGAAGCAA

CN 120030 ATCCTCAAGGTTAAATTATAAACAGGGAAGTAGTCTAAAACAGACAAATTATGGAAGCAA

Hoga ATCCTCAAGGTTAAATTATAAACAGGGAAGTAGTCTAAAACAGACAAATTATGGAAGCAA

Yellowstone ATCCTCAAGGTTAAATTATAAACAGGGAAGTAGTCTAAAACAGACAAATTATGGAAGCAA

CN 119294 ATCCTCAAGGTTAAATTATAAACAGGGAAGTAGTCTAAAACAGACAAATTATGGAAGCAA

************************************************************

CN 120025 TATGGTGGACCGTGGACGAGTGTTAGTCTTAAATCGGTGGTGGAATGAAAAGAATTTTCA

CN 119205 TATGGTGGACCGTGGACGAGTGTTAGTCTTAAATCGGTGGTGGAATGAAAAGAATTTTCA

CN 119243 TATGGTGGACCGTGGACGAGTGTTAGTCTTAAATCGGTGGTGGAATGAAAAGAATTTTCA

Joelle NCBI TATGGTGGACCGTGGACGAGTGTTAGTCTTAAATCGGTGGTGGAATGAAAAGAATTTTCA

Joelle AAFC TATGGTGGACCGTGGACGAGTGTTAGTCTTAAATCGGTGGTGGAATGAAAAGAATTTTCA

Joelle phyto TATGGTGGACCGTGGACGAGTGTTAGTCTTAAATCGGTGGTGGAATGAAAAGAATTTTCA

DH55 ref genome TATGGTGGACCGTGGACGAGTGTTAGTCTTAAATCGGTGGTGGAATGAAAAGAATTTTCA

CN 119300 TATGGTGGACCGTGGACGAGTGTTAGTCTTAAATCGGTGGTGGAATGAAAAGAATTTTCA

CAM 241 TATGGTGGACCGTGGACGAGTGTTAGTCTTAAATCGGTGGTGGAATGAAAAGAATTTTCA

CN 113754 TATGGTGGACCGTGGACGAGTGTTAGTCTTAAATCGGTGGTGGAATGAAAAGAATTTTCA

CAM 236 TATGGTGGACCGTGGACGAGTGTTAGTCTTAAATCGGTGGTGGAATGAAAAGAATTTTCA

CO46 NCBI TATGGTGGACCGTGGACGAGTGTTAGTCTTAAATCGGTGGTGGAATGAAAAGAATTTTCA

CN 120013 TATGGTGGACCGTGGACGAGTGTTAGTCTTAAATCGGTGGTGGAATGAAAAGAATTTTCA

Blaine Creek TATGGTGGACCGTGGACGAGTGTTAGTCTTAAATCGGTGGTGGAATGAAAAGAATTTTCA

09-CS0040 TATGGTGGACCGTGGACGAGTGTTAGTCTTAAATCGGTGGTGGAATGAAAAGAATTTTCA

17CS1133 TATGGTGGACCGTGGACGAGTGTTAGTCTTAAATCGGTGGTGGAATGAAAAGAATTTTCA

Jasper TATGGTGGACCGTGGACGAGTGTTAGTCTTAAATCGGTGGTGGAATGAAAAGAATTTTCA

CN 120017 TATGGTGGACCGTGGACGAGTGTTAGTCTTAAATCGGTGGTGGAATGAAAAGAATTTTCA

CN 120027 TATGGTGGACCGTGGACGAGTGTTAGTCTTAAATCGGTGGTGGAATGAAAAGAATTTTCA

CN 120030 TATGGTGGACCGTGGACGAGTGTTAGTCTTAAATCGGTGGTGGAATGAAAAGAATTTTCA

Hoga TATGGTGGACCGTGGACGAGTGTTAGTCTTAAATCGGTGGTGGAATGAAAAGAATTTTCA

Yellowstone TATGGTGGACCGTGGACGAGTGTTAGTCTTAAATCGGTGGTGGAATGAAAAGAATTTTCA

CN 119294 TATGGTGGACCGTGGACGAGTGTTAGTCTTAAATCGGTGGTGGAATGAAAAGAATTTTCA

************************************************************

CN 120025 TATATGTGAGTAGAAGACAAAAAGAAAAAAGTGAATAGTGATTTTGACCTATGA-TATCG

CN 119205 TATATTTGAGTAGAAGACAAAAAGAAAAAAGTGAATAGTGATTTTGACCTATGATTATCG

CN 119243 TATATTTGAGTAGAAGACAAAAAGAAAAAAGTGAATAGTGATTTTGACCTATGATTATCG

Joelle NCBI TATATTTGAGTAGAAGACAAAAAGAAAAAAGTGAATAGTGATTTTGACCTATGATTATCG

Joelle AAFC TATATTTGAGTAGAAGACAAAAAGAAAAAAGTGAATAGTGATTTTGACCTATGATTATCG

Joelle phyto TATATTTGAGTAGAAGACAAAAAGAAAAAAGTGAATAGTGATTTTGACCTATGATTATCG

DH55 ref genome TATATTTGAGTAGAAGACAAAAAGAAAAAAGTGAATAGTGATTTTGACCTATGATTATCG

CN 119300 TATATTTGAGTAGAAGACAAAAAGAAAAAAGTGAATAGTGATTTTGACCTATGATTATCG

CAM 241 TATATTTGAGTAGAAGACAAAAAGAAAAAAGTGAATAGTGATTTTGACCTATGATTATCG

CN 113754 TATATTTGAGTAGAAGACAAAAAGAAAAAAGTGAATAGTGATTTTGACCTATGATTATCG

CAM 236 TATATTTGAGTAGAAGACAAAAAGAAAAAAGTGAATAGTGATTTTGACCTATGATTATCG

CO46 NCBI TATATTTGAGTAGAAGACAAAAAGAAAAAAGTGAATAGTGATTTTGACCTATGATTATCG

CN 120013 TATATTTGAGTAGAAGACAAAAAGAAAAAAGTGAATAGTGATTTTGACCTATGATTATCG

Blaine Creek TATATTTGAGTAGAAGACAAAAAGAAAAAAGTGAATAGTGATTTTGACCTATGATTATCG

09-CS0040 TATATTTGAGTAGAAGACAAAAAGAAAAAAGTGAATAGTGATTTTGACCTATGATTATCG

17CS1133 TATATTTGAGTAGAAGACAAAAAGAAAAAAGTGAATAGTGATTTTGACCTATGATTATCG

Jasper TATATTTGAGTAGAAGACAAAAAGAAAAAAGTGAATAGTGATTTTGACCTATGATTATCG

CN 120017 TATATTTGAGTAGAAGACAAAAAGAAAAAAGTGAATAGTGATTTTGACCTATGATTATCG

CN 120027 TATATTTGAGTAGAAGACAAAAAGAAAAAAGTGAATAGTGATTTTGACCTATGATTATCG

CN 120030 TATATTTGAGTAGAAGACAAAAAGAAAAAAGTGAATAGTGATTTTGACCTATGATTATCG

Hoga TATATTTGAGTAGAAGACAAAAAGAAAAAAGTGAATAGTGATTTTGACCTATGATTATCG

Yellowstone TATATTTGAGTAGAAGACAAAAAGAAAAAAGTGAATAGTGATTTTGACCTATGATTATCG

CN 119294 TATATTTGAGTAGAAGACAAAAAGAAAAAAGTGAATAGTGATTTTGACCTATGATTATCG

***** ************************************************ *****

CN 120025 TACAGATGGAGACGAATCATGTTGTTGGAGCAGAAGCTGATATGGAGATGGAGATGTCAC

CN 119205 TACAGATGGAGACGAATCATGTTGTTGGAGCAGAAGCTGATATGGAGATGGAGATGTCAC

CN 119243 TACAGATGGAGACGAATCATGTTGTTGGAGCAGAAGCTGATATGGAGATGGAGATGTCAC

Joelle NCBI TACAGATGGAGACGAATCATGTTGTTGGAGCAGAAGCTGATATGGAGATGGAGATGTCAC

Joelle AAFC TACAGATGGAGACGAATCATGTTGTTGGAGCAGAAGCTGATATGGAGATGGAGATGTCAC

Joelle phyto TACAGATGGAGACGAATCATGTTGTTGGAGCAGAAGCTGATATGGAGATGGAGATGTCAC

DH55 ref genome TACAGATGGAGACGAATCATGTTGTTGGAGCAGAAGCTGATATGGAGATGGAGATGTCAC

CN 119300 TACAGATGGAGACGAATCATGTTGTTGGAGCAGAAGCTGATATGGAGATGGAGATGTCAC

CAM 241 TACAGATGGAGACGAATCATGTTGTTGGAGCAGAAGCTGATATGGAGATGGAGATGTCAC

CN 113754 TACAGATGGAGACGAATCATGTTGTTGGAGCAGAAGCTGATATGGAGATGGAGATGTCAC

CAM 236 TACAGATGGAGACGAATCATGTTGTTGGAGCAGAAGCTGATATGGAGATGGAGATGTCAC

CO46 NCBI TACAGATGGAGACGAATCATGTTGTTGGAGCAGAAGCTGATATGGAGATGGAGATGTCAC

CN 120013 TACAGATGGAGACGAATCATGTTGTTGGAGCAGAAGCTGATATGGAGATGGAGATGTCAC

Blaine Creek TACAGATGGAGACGAATCATGTTGTTGGAGCAGAAGCTGATATGGAGATGGAGATGTCAC

09-CS0040 TACAGATGGAGACGAATCATGTTGTTGGAGCAGAAGCTGATATGGAGATGGAGATGTCAC

17CS1133 TACAGATGGAGACGAATCATGTTGTTGGAGCAGAAGCTGATATGGAGATGGAGATGTCAC

Jasper TACAGATGGAGACGAATCATGTTGTTGGAGCAGAAGCTGATATGGAGATGGAGATGTCAC

CN 120017 TACAGATGGAGACGAATCATGTTGTTGGAGCAGAAGCTGATATGGAGATGGAGATGTCAC

CN 120027 TACAGATGGAGACGAATCATGTTGTTGGAGCAGAAGCTGATATGGAGATGGAGATGTCAC

CN 120030 TACAGATGGAGACGAATCATGTTGTTGGAGCAGAAGCTGATATGGAGATGGAGATGTCAC

Hoga TACAGATGGAGACGAATCATGTTGTTGGAGCAGAAGCTGATATGGAGATGGAGATGTCAC

Yellowstone TACAGATGGAGACGAATCATGTTGTTGGAGCAGAAGCTGATATGGAGATGGAGATGTCAC

CN 119294 TACAGATGGAGACGAATCATGTTGTTGGAGCAGAAGCTGATATGGAGATGGAGATGTCAC

************************************************************

CN 120025 CTGTTGGACAAATCTCCGACAATCTTCCGGTGACTCTCCCGCTGCTCAATTAGGGCGGTT

CN 119205 CTGTTGGACAAATCTCCGACAATCTTCCGGTGACTCTCCCGCTGCTCAATTAGGGCGGTT

CN 119243 CTGTTGGACAAATCTCCGACAATCTTCCGGTGACTCTCCCGCTGCTCAATTAGGGCGGTT

Joelle NCBI CTGTTGGACAAATCTCCGACAATCTTCCGGTGACTCTCCCGCTGCTCAATTAGGGCGGTT

Joelle AAFC CTGTTGGACAAATCTCCGACAATCTTCCGGTGACTCTCCCGCTGCTCAATTAGGGCGGTT

Joelle phyto CTGTTGGACAAATCTCCGACAATCTTCCGGTGACTCTCCCGCTGCTCAATTAGGGCGGTT

DH55 ref genome CTGTTGGACAAATCTCCGACAATCTTCCGGTGACTCTCCCGCTGCTCAATTAGGGCGGTT

CN 119300 CTGTTGGACAAATCTCCGACAATCTTCCGGTGACTCTCCCGCTGCTCAATTAGGGCGGTT

CAM 241 CTGTTGGACAAATCTCCGACAATCTTCCGGTGACTCTCCCGCTGCTCAATTAGGGCGGTT

CN 113754 CTGTTGGACAAATCTCCGACAATCTTCCGGTGACTCTCCCGCTGCTCAATTAGGGCGGTT

CAM 236 CTGTTGGACAAATCTCCGACAATCTTCCGGTGACTCTCCCGCTGCTCAATTAGGGCGGTT

CO46 NCBI CTGTTGGACAAATCTCCGACAATCTTCCGGTGACTCTCCCGCTGCTCAATTAGGGCGGTT

CN 120013 CTGTTGGACAAATCTCCGACAATCTTCCGGTGACTCTCCCGCTGCTCAATTAGGGCGGTT

Blaine Creek CTGTTGGACAAATCTCCGACAATCTTCCGGTGACTCTCCCGCTGCTCAATTAGGGCGGTT

09-CS0040 CTGTTGGACAAATCTCCGACAATCTTCCGGTGACTCTCCCGCTGCTCAATTAGGGCGGTT

17CS1133 CTGTTGGACAAATCTCCGACAATCTTCCGGTGACTCTCCCGCTGCTCAATTAGGGCGGTT

Jasper CTGTTGGACAAATCTCCGACAATCTTCCGGTGACTCTCCCGCTGCTCAATTAGGGCGGTT

CN 120017 CTGTTGGACAAATCTCCGACAATCTTCCGGTGACTCTCCCGCTGCTCAATTAGGGCGGTT

CN 120027 CTGTTGGACAAATCTCCGACAATCTTCCGGTGACTCTCCCGCTGCTCAATTAGGGCGGTT

CN 120030 CTGTTGGACAAATCTCCGACAATCTTCCGGTGACTCTCCCGCTGCTCAATTAGGGCGGTT

Hoga CTGTTGGACAAATCTCCGACAATCTTCCGGTGACTCTCCCGCTGCTCAATTAGGGCGGTT

Yellowstone CTGTTGGACAAATCTCCGACAATCTTCCGGTGACTCTCCCGCTGCTCAATTAGGGCGGTT

CN 119294 CTGTTGGACAAATCTCCGACAATCTTCCGGTGACTCTCCCGCTGCTCAATTAGGGCGGTT

************************************************************

CN 120025 GATATCAAAAATCCAAAACATATATATAATTAAGGGGAAAAAAAAAAAGATATGTAATTA

CN 119205 GATATCAAAAATCCAAAACATATATATAATTAAGGGG--AAAAAAAAAGATATGTAATTA

CN 119243 GATATCAAAAATCCAAAACATATATATAATTAAGGGG-AAAAAAAAAAGATATGTAATTA

Joelle NCBI GATATCAAAAATCCAAAACATATATATAATTAAGGGG-AAAAAAAAAAGATATGTAATTA

Joelle AAFC GATATCAAAAATCCAAAACATATATATAATTAAGGGG-AAAAAAAAAAGATATGTAATTA

Joelle phyto GATATCAAAAATCCAAAACATATATATAATTAAGGGG-AAAAAAAAAAGATATGTAATTA

DH55 ref genome GATATCAAAAATCCAAAACATATATATAATTAAGGGG-AAAAAAAAAAGATATGTAATTA

CN 119300 GATATCAAAAATCCAAAACATATATATAATTAAGGGG-AAAAAAAAAAGATATGTAATTA

CAM 241 GATATCAAAAATCCAAAACATATATATAATTAAGGGG-AAAAAAAAAAGATATGTAATTA

CN 113754 GATATCAAAAATCCAAAACATATATATAATTAAGGGG-AAAAAAAAAAGATATGTAATTA

CAM 236 GATATCAAAAATCCAAAACATATATATAATTAAGGGG-AAAAAAAAAAGATATGTAATTA

CO46 NCBI GATATCAAAAATCCAAAACATATATATAATTAAGGGG-AAAAAAAAAAGATATGTAATTA

CN 120013 GATATCAAAAATCCAAAACATATATATAATTAAGGGG-AAAAAAAAAAGATATGTAATTA

Blaine Creek GATATCAAAAATCCAAAACATATATATAATTAAGGGG-AAAAAAAAAAGATATGTAATTA

09-CS0040 GATATCAAAAATCCAAAACATATATATAATTAAGGGG-AAAAAAAAAAGATATGTAATTA

17CS1133 GATATCAAAAATCCAAAACATATATATAATTAAGGGG-AAAAAAAAAAGATATGTAATTA

Jasper GATATCAAAAATCCAAAACATATATATAATTAAGGGG-AAAAAAAAAAGATATGTAATTA

CN 120017 GATATCAAAAATCCAAAACATATATATAATTAAGGGG-AAAAAAAAAAGATATGTAATTA

CN 120027 GATATCAAAAATCCAAAACATATATATAATTAAGGGG-AAAAAAAAAAGATATGTAATTA

CN 120030 GATATCAAAAATCCAAAACATATATATAATTAAGGGG-AAAAAAAAAAGATATGTAATTA

Hoga GATATCAAAAATCCAAAACATATATATAATTAAGGGG-AAAAAAAAAAGATATGTAATTA

Yellowstone GATATCAAAAATCCAAAACATATATATAATTAAGGGG--AAAAAAAAAGATATGTAATTA

CN 119294 GATATCAAAAATCCAAAACATATATATAATTAAGGGG-AAAAAAAAAAGATATGTAATTA

************************************* *********************

CN 120025 TTCCGCTGATAAGGGCGCGTCGTATGTATGTATATCTTAATACTCCCTCCTCTCTCTCTC

CN 119205 TTCCGCTGATAAGGGCGCGTCGTATGTATGTATATCTTAATACTCCCTC--CTCTCTCTC

CN 119243 TTCCGCTGATAAGGGCGCGTCGTATGTATGTATATCTTAATACTCCCTC----CTCTCTC

Joelle NCBI TTCCGCTGATAAGGGCGCGTCGTATGTATGTATATCTTAATACTCCCTC----CTCTCTC

Joelle AAFC TTCCGCTGATAAGGGCGCGTCGTATGTATGTATATCTTAATACTCCCTC----CTCTCTC

Joelle phyto TTCCGCTGATAAGGGCGCGTCGTATGTATGTATATCTTAATACTCCCTC----CTCTCTC

DH55 ref genome TTCCGCTGATAAGGGCGCGTCGTATGTATGTATATCTTAATACTCCCTC----CTCTCTC

CN 119300 TTCCGCTGATAAGGGCGCGTCGTATGTATGTATATCTTAATACTCCCTC----CTCTCTC

CAM 241 TTCCGCTGATAAGGGCGCGTCGTATGTATGTATATCTTAATACTCCCTC----CTCTCTC

CN 113754 TTCCGCTGATAAGGGCGCGTCGTATGTATGTATATCTTAATACTCCCTC----CTCTCTC

CAM 236 TTCCGCTGATAAGGGCGCGTCGTATGTATGTATATCTTAATACTCCCTC----CTCTCTC

CO46 NCBI TTCCGCTGATAAGGGCGCGTCGTATGTATGTATATCTTAATACTCCCTC----CTCTCTC

CN 120013 TTCCGCTGATAAGGGCGCGTCGTATGTATGTATATCTTAATACTCCCTC----CTCTCTC

Blaine Creek TTCCGCTGATAAGGGCGCGTCGTATGTATGTATATCTTAATACTCCCTC----CTCTCTC

09-CS0040 TTCCGCTGATAAGGGCGCGTCGTATGTATGTATATCTTAATACTCCCTC----CTCTCTC

17CS1133 TTCCGCTGATAAGGGCGCGTCGTATGTATGTATATCTTAATACTCCCTC----CTCTCTC

Jasper TTCCGCTGATAAGGGCGCGTCGTATGTATGTATATCTTAATACTCCCTC----CTCTCTC

CN 120017 TTCCGCTGATAAGGGCGCGTCGTATGTATGTATATCTTAATACTCCCTC----CTCTCTC

CN 120027 TTCCGCTGATAAGGGCGCGTCGTATGTATGTATATCTTAATACTCCCTC----CTCTCTC

CN 120030 TTCCGCTGATAAGGGCGCGTCGTATGTATGTATATCTTAATACTCCCTC----CTCTCTC

Hoga TTCCGCTGATAAGGGCGCGTCGTATGTATGTATATCTTAATACTCCCTC----CTCTCTC

Yellowstone TTCCGCTGATAAGGGCGCGTCGTATGTATGTATATCTTAATACTCCCTC----CTCTCTC

CN 119294 TTCCGCTGATAAGGGCGCGTCGTATGTATGTATATCTTAATACTCCCTC----CTCTCTC

************************************************* *******

CN 120025 TCTCTCTCTTTGGCTTGTGTGTGATACTTCAAAGTAGATTGAAAGTCAAATATTATCTGT

CN 119205 TCTCTCTCTTTGGCTTGTGTGTGATACTTAAAAGTAGATTGAAAGTCAAATATTATCTGT

CN 119243 TCTCTCTCTTTGGCTTGTGTGTGATACTTAAAAGTAGATTGAAAGTCAAATATTATCTGT

Joelle NCBI TCTCTCTCTTTGGCTTGTGTGTGATACTTAAAAGTAGATTGAAAGTCAAATATTATCTGT

Joelle AAFC TCTCTCTCTTTGGCTTGTGTGTGATACTTAAAAGTAGATTGAAAGTCAAATATTATCTGT

Joelle phyto TCTCTCTCTTTGGCTTGTGTGTGATACTTAAAAGTAGATTGAAAGTCAAATATTATCTGT

DH55 ref genome TCTCTCTCTTTGGCTTGTGTGTGATACTTAAAAGTAGATTGAAAGTCAAATATTATCTGT

CN 119300 TCTCTCTCTTTGGCTTGTGTGTGATACTTAAAAGTAGATTGAAAGTCAAATATTATCTGT

CAM 241 TCTCTCTCTTTGGCTTGTGTGTGATACTTAAAAGTAGATTGAAAGTCAAATATTATCTGT

CN 113754 TCTCTCTCTTTGGCTTGTGTGTGATACTTAAAAGTAGATTGAAAGTCAAATATTATCTGT

CAM 236 TCTCTCTCTTTGGCTTGTGTGTGATACTTAAAAGTAGATTGAAAGTCAAATATTATCTGT

CO46 NCBI TCTCTCTCTTTGGCTTGTGTGTGATACTTAAAAGTAGATTGAAAGTCAAATATTATCTGT

CN 120013 TCTCTCTCTTTGGCTTGTGTGTGATACTTAAAAGTAGATTGAAAGTCAAATATTATCTGT

Blaine Creek TCTCTCTCTTTGGCTTGTGTGTGATACTTAAAAGTAGATTGAAAGTCAAATATTATCTGT

09-CS0040 TCTCTCTCTTTGGCTTGTGTGTGATACTTAAAAGTAGATTGAAAGTCAAATATTATCTGT

17CS1133 TCTCTCTCTTTGGCTTGTGTGTGATACTTAAAAGTAGATTGAAAGTCAAATATTATCTGT

Jasper TCTCTCTCTTTGGCTTGTGTGTGATACTTAAAAGTAGATTGAAAGTCAAATATTATCTGT

CN 120017 TCTCTCTCTTTGGCTTGTGTGTGATACTTAAAAGTAGATTGAAAGTCAAATATTATCTGT

CN 120027 TCTCTCTCTTTGGCTTGTGTGTGATACTTAAAAGTAGATTGAAAGTCAAATATTATCTGT

CN 120030 TCTCTCTCTTTGGCTTGTGTGTGATACTTAAAAGTAGATTGAAAGTCAAATATTATCTGT

Hoga TCTCTCTCTTTGGCTTGTGTGTGATACTTAAAAGTAGATTGAAAGTCAAATATTATCTGT

Yellowstone TCTCTCTCTTTGGCTTGTGTGTGATACTTAAAAGTAGATTGAAAGTCAAATATTATCTGT

CN 119294 TCTCTCTCTTTGGCTTGTGTGTGATACTTAAAAGTAGATTGAAAGTCAAATATTATCTGT

***************************** ******************************

CN 120025 TTAAGACAAAGCTTATGATGAAACTTTGTACCTTATTCGAGAGAGCTTTGCATCGAGATG

CN 119205 TTAAGACAAAGCTTATGATGAAACTTTGTACCTTATTCGAGAGAGCTTTGCATCGAGATG

CN 119243 TTAAGACAAAGCTTATGATGAAACTTTGTACCTTATTCGAGAGAGCTTTGCATCGAGATG

Joelle NCBI TTAAGACAAAGCTTATGATGAAACTTTGTACCTTATTCGAGAGAGCTTTGCATCGAGATG

Joelle AAFC TTAAGACAAAGCTTATGATGAAACTTTGTACCTTATTCGAGAGAGCTTTGCATCGAGATG

Joelle phyto TTAAGACAAAGCTTATGATGAAACTTTGTACCTTATTCGAGAGAGCTTTGCATCGAGATG

DH55 ref genome TTAAGACAAAGCTTATGATGAAACTTTGTACCTTATTCGAGAGAGCTTTGCATCGAGATG

CN 119300 TTAAGACAAAGCTTATGATGAAACTTTGTACCTTATTCGAGAGAGCTTTGCATCGAGATG

CAM 241 TTAAGACAAAGCTTATGATGAAACTTTGTACCTTATTCGAGAGAGCTTTGCATCGAGATG

CN 113754 TTAAGACAAAGCTTATGATGAAACTTTGTACCTTATTCGAGAGAGCTTTGCATCGAGATG

CAM 236 TTAAGACAAAGCTTATGATGAAACTTTGTACCTTATTCGAGAGAGCTTTGCATCGAGATG

CO46 NCBI TTAAGACAAAGCTTATGATGAAACTTTGTACCTTATTCGAGAGAGCTTTGCATCGAGATG

CN 120013 TTAAGACAAAGCTTATGATGAAACTTTGTACCTTATTCGAGAGAGCTTTGCATCGAGATG

Blaine Creek TTAAGACAAAGCTTATGATGAAACTTTGTACCTTATTCGAGAGAGCTTTGCATCGAGATG

09-CS0040 TTAAGACAAAGCTTATGATGAAACTTTGTACCTTATTCGAGAGAGCTTTGCATCGAGATG

17CS1133 TTAAGACAAAGCTTATGATGAAACTTTGTACCTTATTCGAGAGAGCTTTGCATCGAGATG

Jasper TTAAGACAAAGCTTATGATGAAACTTTGTACCTTATTCGAGAGAGCTTTGCATCGAGATG

CN 120017 TTAAGACAAAGCTTATGATGAAACTTTGTACCTTATTCGAGAGAGCTTTGCATCGAGATG

CN 120027 TTAAGACAAAGCTTATGATGAAACTTTGTACCTTATTCGAGAGAGCTTTGCATCGAGATG

CN 120030 TTAAGACAAAGCTTATGATGAAACTTTGTACCTTATTCGAGAGAGCTTTGCATCGAGATG

Hoga TTAAGACAAAGCTTATGATGAAACTTTGTACCTTATTCGAGAGAGCTTTGCATCGAGATG

Yellowstone TTAAGACAAAGCTTATGATGAAACTTTGTACCTTATTCGAGAGAGCTTTGCATCGAGATG

CN 119294 TTAAGACAAAGCTTATGATGAAACTTTGTACCTTATTCGAGAGAGCTTTGCATCGAGATG

************************************************************

CN 120025 TTGTTGTTGTGTGTTTTCTCCTCTTCTGTCGAAAGCTTGTGTTTGCTTCACAGTGAAGAA

CN 119205 TTGTTGTTGTGTGTTTTCTCCTCTTCTGTCGAAAGCTTGTGTTTGCTTCACAGTGAAGAA

CN 119243 TTGTTGTTGTGTGTTTTCTCCTCTTCTGTCGAAAGCTTGTGTTTGCTTCACAGTGAAGAA

Joelle NCBI TTGTTGTTGTGTGTTTTCTCCTCTTCTGTCGAAAGCTTGTGTTTGCTTCACAGTGAAGAA

Joelle AAFC TTGTTGTTGTGTGTTTTCTCCTCTTCTGTCGAAAGCTTGTGTTTGCTTCACAGTGAAGAA

Joelle phyto TTGTTGTTGTGTGTTTTCTCCTCTTCTGTCGAAAGCTTGTGTTTGCTTCACAGTGAAGAA

DH55 ref genome TTGTTGTTGTGTGTTTTCTCCTCTTCTGTCGAAAGCTTGTGTTTGCTTCACAGTGAAGAA

CN 119300 TTGTTGTTGTGTGTTTTCTCCTCTTCTGTCGAAAGCTTGTGTTTGCTTCACAGTGAAGAA

CAM 241 TTGTTGTTGTGTGTTTTCTCCTCTTCTGTCGAAAGCTTGTGTTTGCTTCACAGTGAAGAA

CN 113754 TTGTTGTTGTGTGTTTTCTCCTCTTCTGTCGAAAGCTTGTGTTTGCTTCACAGTGAAGAA

CAM 236 TTGTTGTTGTGTGTTTTCTCCTCTTCTGTCGAAAGCTTGTGTTTGCTTCACAGTGAAGAA

CO46 NCBI TTGTTGTTGTGTGTTTTCTCCTCTTCTGTCGAAAGCTTGTGTTTGCTTCACAGTGAAGAA

CN 120013 TTGTTGTTGTGTGTTTTCTCCTCTTCTGTCGAAAGCTTGTGTTTGCTTCACAGTGAAGAA

Blaine Creek TTGTTGTTGTGTGTTTTCTCCTCTTCTGTCGAAAGCTTGTGTTTGCTTCACAGTGAAGAA

09-CS0040 TTGTTGTTGTGTGTTTTCTCCTCTTCTGTCGAAAGCTTGTGTTTGCTTCACAGTGAAGAA

17CS1133 TTGTTGTTGTGTGTTTTCTCCTCTTCTGTCGAAAGCTTGTGTTTGCTTCACAGTGAAGAA

Jasper TTGTTGTTGTGTGTTTTCTCCTCTTCTGTCGAAAGCTTGTGTTTGCTTCACAGTGAAGAA

CN 120017 TTGTTGTTGTGTGTTTTCTCCTCTTCTGTCGAAAGCTTGTGTTTGCTTCACAGTGAAGAA

CN 120027 TTGTTGTTGTGTGTTTTCTCCTCTTCTGTCGAAAGCTTGTGTTTGCTTCACAGTGAAGAA

CN 120030 TTGTTGTTGTGTGTTTTCTCCTCTTCTGTCGAAAGCTTGTGTTTGCTTCACAGTGAAGAA

Hoga TTGTTGTTGTGTGTTTTCTCCTCTTCTGTCGAAAGCTTGTGTTTGCTTCACAGTGAAGAA

Yellowstone TTGTTGTTGTGTGTTTTCTCCTCTTCTGTCGAAAGCTTGTGTTTGCTTCACAGTGAAGAA

CN 119294 TTGTTGTTGTGTGTTTTCTCCTCTTCTGTCGAAAGCTTGTGTTTGCTTCACAGTGAAGAA

************************************************************

CN 120025 GTCTTCTGCTTATTTTGCATTAGAGGCGTGGCACAACTCTGCTCTATTTGTCCTCGTAAT

CN 119205 GTCTTCTGCTTATTTTGCATTAGAGGCGTGGCACAACTCTGCTCTATTTGTCCTCGTAAT

CN 119243 GTCTTCTGCTTATTTTGCATTAGAGGCGTGGCACAACTCTGCTCTATTTGTCCTCGTAAT

Joelle NCBI GTCTTCTGCTTATTTTGCATTAGAGGCGTGGCACAACTCTGCTCTATTTGTCCTCGTAAT

Joelle AAFC GTCTTCTGCTTATTTTGCATTAGAGGCGTGGCACAACTCTGCTCTATTTGTCCTCGTAAT

Joelle phyto GTCTTCTGCTTATTTTGCATTAGAGGCGTGGCACAACTCTGCTCTATTTGTCCTCGTAAT

DH55 ref genome GTCTTCTGCTTATTTTGCATTAGAGGCGTGGCACAACTCTGCTCTATTTGTCCTCGTAAT

CN 119300 GTCTTCTGCTTATTTTGCATTAGAGGCGTGGCACAACTCTGCTCTATTTGTCCTCGTAAT

CAM 241 GTCTTCTGCTTATTTTGCATTAGAGGCGTGGCACAACTCTGCTCTATTTGTCCTCGTAAT

CN 113754 GTCTTCTGCTTATTTTGCATTAGAGGCGTGGCACAACTCTGCTCTATTTGTCCTCGTAAT

CAM 236 GTCTTCTGCTTATTTTGCATTAGAGGCGTGGCACAACTCTGCTCTATTTGTCCTCGTAAT

CO46 NCBI GTCTTCTGCTTATTTTGCATTAGAGGCGTGGCACAACTCTGCTCTATTTGTCCTCGTAAT

CN 120013 GTCTTCTGCTTATTTTGCATTAGAGGCGTGGCACAACTCTGCTCTATTTGTCCTCGTAAT

Blaine Creek GTCTTCTGCTTATTTTGCATTAGAGGCGTGGCACAACTCTGCTCTATTTGTCCTCGTAAT

09-CS0040 GTCTTCTGCTTATTTTGCATTAGAGGCGTGGCACAACTCTGCTCTATTTGTCCTCGTAAT

17CS1133 GTCTTCTGCTTATTTTGCATTAGAGGCGTGGCACAACTCTGCTCTATTTGTCCTCGTAAT

Jasper GTCTTCTGCTTATTTTGCATTAGAGGCGTGGCACAACTCTGCTCTATTTGTCCTCGTAAT

CN 120017 GTCTTCTGCTTATTTTGCATTAGAGGCGTGGCACAACTCTGCTCTATTTGTCCTCGTAAT

CN 120027 GTCTTCTGCTTATTTTGCATTAGAGGCGTGGCACAACTCTGCTCTATTTGTCCTCGTAAT

CN 120030 GTCTTCTGCTTATTTTGCATTAGAGGCGTGGCACAACTCTGCTCTATTTGTCCTCGTAAT

Hoga GTCTTCTGCTTATTTTGCATTAGAGGCGTGGCACAACTCTGCTCTATTTGTCCTCGTAAT

Yellowstone GTCTTCTGCTTATTTTGCATTAGAGGCGTGGCACAACTCTGCTCTATTTGTCCTCGTAAT

CN 119294 GTCTTCTGCTTATTTTGCATTAGAGGCGTGGCACAACTCTGCTCTATTTGTCCTCGTAAT

************************************************************

CN 120025 TTAATTTG-TTTTATCTAAAACGCGTTGGGGTTGTCATGGGTTTCTATTGGGCCCATGTC

CN 119205 TTAATTTGTTTTTATCTAAAACGCGTTGGGGTTGTCATGGGTTTCTATTGGGCCCATGTC

CN 119243 TTAATTTGTTTTTATCTAAAACGCGTTGGGGTTGTCATGGGTTTCTATTGGGCCCATGTC

Joelle NCBI TTAATTTGTTTTTATCTAAAACGCGTTGGGGTTGTCATGGGTTTCTATTGGGCCCATGTC

Joelle AAFC TTAATTTGTTTTTATCTAAAACGCGTTGGGGTTGTCATGGGTTTCTATTGGGCCCATGTC

Joelle phyto TTAATTTGTTTTTATCTAAAACGCGTTGGGGTTGTCATGGGTTTCTATTGGGCCCATGTC

DH55 ref genome TTAATTTGTTTTTATCTAAAACGCGTTGGGGTTGTCATGGGTTTCTATTGGGCCCATGTC

CN 119300 TTAATTTGTTTTTATCTAAAACGCGTTGGGGTTGTCATGGGTTTCTATTGGGCCCATGTC

CAM 241 TTAATTTGTTTTTATCTAAAACGCGTTGGGGTTGTCATGGGTTTCTATTGGGCCCATGTC

CN 113754 TTAATTTGTTTTTATCTAAAACGCGTTGGGGTTGTCATGGGTTTCTATTGGGCCCATGTC

CAM 236 TTAATTTGTTTTTATCTAAAACGCGTTGGGGTTGTCATGGGTTTCTATTGGGCCCATGTC

CO46 NCBI TTAATTTGTTTTTATCTAAAACGCGTTGGGGTTGTCATGGGTTTCTATTGGGCCCATGTC

CN 120013 TTAATTTGTTTTTATCTAAAACGCGTTGGGGTTGTCATGGGTTTCTATTGGGCCCATGTC

Blaine Creek TTAATTTGTTTTTATCTAAAACGCGTTGGGGTTGTCATGGGTTTCTATTGGGCCCATGTC

09-CS0040 TTAATTTGTTTTTATCTAAAACGCGTTGGGGTTGTCATGGGTTTCTATTGGGCCCATGTC

17CS1133 TTAATTTGTTTTTATCTAAAACGCGTTGGGGTTGTCATGGGTTTCTATTGGGCCCATGTC

Jasper TTAATTTGTTTTTATCTAAAACGCGTTGGGGTTGTCATGGGTTTCTATTGGGCCCATGTC

CN 120017 TTAATTTGTTTTTATCTAAAACGCGTTGGGGTTGTCATGGGTTTCTATTGGGCCCATGTC

CN 120027 TTAATTTGTTTTTATCTAAAACGCGTTGGGGTTGTCATGGGTTTCTATTGGGCCCATGTC

CN 120030 TTAATTTGTTTTTATCTAAAACGCGTTGGGGTTGTCATGGGTTTCTATTGGGCCCATGTC

Hoga TTAATTTGTTTTTATCTAAAACGCGTTGGGGTTGTCATGGGTTTCTATTGGGCCCATGTC

Yellowstone TTAATTTGTTTTTATCTAAAACGCGTTGGGGTTGTCATGGGTTTCTATTGGGCCCATGTC

CN 119294 TTAATTTGTTTTTATCTAAAACGCGTTGGGGTTGTCATGGGTTTCTATTGGGCCCATGTC

******** ***************************************************

CN 120025 GGTCATTTTTTTGGCCCAATAGGCTTAGTTCCTGAAATTCATTGCTCTTAAGTGTTGTTA

CN 119205 GGTCATTTTTTTGGCCCAATAGGCTTAGTTCCTGAAATTCATTGCTCTTAAGTGTTGTTA

CN 119243 GGTCATTTTTTTGGCCCAATAGGCTTAGTTCCTGAAATTCATTGCTCTTAAGTGTTGTTA

Joelle NCBI GGTCATTTTTTTGGCCCAATAGGCTTAGTTCCTGAAATTCATTGCTCTTAAGTGTTGTTA

Joelle AAFC GGTCATTTTTTTGGCCCAATAGGCTTAGTTCCTGAAATTCATTGCTCTTAAGTGTTGTTA

Joelle phyto GGTCATTTTTTTGGCCCAATAGGCTTAGTTCCTGAAATTCATTGCTCTTAAGTGTTGTTA

DH55 ref genome GGTCATTTTTTTGGCCCAATAGGCTTAGTTCCTGAAATTCATTGCTCTTAAGTGTTGTTA

CN 119300 GGTCATTTTTTTGGCCCAATAGGCTTAGTTCCTGAAATTCATTGCTCTTAAGTGTTGTTA

CAM 241 GGTCATTTTTTTGGCCCAATAGGCTTAGTTCCTGAAATTCATTGCTCTTAAGTGTTGTTA

CN 113754 GGTCATTTTTTTGGCCCAATAGGCTTAGTTCCTGAAATTCATTGCTCTTAAGTGTTGTTA

CAM 236 GGTCATTTTTTTGGCCCAATAGGCTTAGTTCCTGAAATTCATTGCTCTTAAGTGTTGTTA

CO46 NCBI GGTCATTTTTTTGGCCCAATAGGCTTAGTTCCTGAAATTCATTGCTCTTAAGTGTTGTTA

CN 120013 GGTCATTTTTTTGGCCCAATAGGCTTAGTTCCTGAAATTCATTGCTCTTAAGTGTTGTTA

Blaine Creek GGTCATTTTTTTGGCCCAATAGGCTTAGTTCCTGAAATTCATTGCTCTTAAGTGTTGTTA

09-CS0040 GGTCATTTTTTTGGCCCAATAGGCTTAGTTCCTGAAATTCATTGCTCTTAAGTGTTGTTA

17CS1133 GGTCATTTTTTTGGCCCAATAGGCTTAGTTCCTGAAATTCATTGCTCTTAAGTGTTGTTA

Jasper GGTCATTTTTTTGGCCCAATAGGCTTAGTTCCTGAAATTCATTGCTCTTAAGTGTTGTTA

CN 120017 GGTCATTTTTTTGGCCCAATAGGCTTAGTTCCTGAAATTCATTGCTCTTAAGTGTTGTTA

CN 120027 GGTCATTTTTTTGGCCCAATAGGCTTAGTTCCTGAAATTCATTGCTCTTAAGTGTTGTTA

CN 120030 GGTCATTTTTTTGGCCCAATAGGCTTAGTTCCTGAAATTCATTGCTCTTAAGTGTTGTTA

Hoga GGTCATTTTTTTGGCCCAATAGGCTTAGTTCCTGAAATTCATTGCTCTTAAGTGTTGTTA

Yellowstone GGTCATTTTTTTGGCCCAATAGGCTTAGTTCCTGAAATTCATTGCTCTTAAGTGTTGTTA

CN 119294 GGTCATTTTTTTGGCCCAATAGGCTTAGTTCCTGAAATTCATTGCTCTTAAGTGTTGTTA

************************************************************

CN 120025 ACGTGACCGACCTCTTTCCAAAATTATGCTTGATATCTAAACCGGCTTAACTAGCCGTAT

CN 119205 ACGTGACCGACCTCTTTCCAAAATTATGCTTGATATCTAAACCGGCTTAACTAGCCGTAT

CN 119243 ACGTGACCGACCTCTTTCCAAAATTATGCTTGATATCTAAACCGGCTTAACTAGCCGTAT

Joelle NCBI ACGTGACCGACCTCTTTCCAAAATTATGCTTGATATCTAAACCGGCTTAACTAGCCGTAT

Joelle AAFC ACGTGACCGACCTCTTTCCAAAATTATGCTTGATATCTAAACCGGCTTAACTAGCCGTAT

Joelle phyto ACGTGACCGACCTCTTTCCAAAATTATGCTTGATATCTAAACCGGCTTAACTAGCCGTAT

DH55 ref genome ACGTGACCGACCTCTTTCCAAAATTATGCTTGATATCTAAACCGGCTTAACTAGCCGTAT

CN 119300 ACGTGACCGACCTCTTTCCAAAATTATGCTTGATATCTAAACCGGCTTAACTAGCCGTAT

CAM 241 ACGTGACCGACCTCTTTCCAAAATTATGCTTGATATCTAAACCGGCTTAACTAGCCGTAT

CN 113754 ACGTGACCGACCTCTTTCCAAAATTATGCTTGATATCTAAACCGGCTTAACTAGCCGTAT

CAM 236 ACGTGACCGACCTCTTTCCAAAATTATGCTTGATATCTAAACCGGCTTAACTAGCCGTAT

CO46 NCBI ACGTGACCGACCTCTTTCCAAAATTATGCTTGATATCTAAACCGGCTTAACTAGCCGTAT

CN 120013 ACGTGACCGACCTCTTTCCAAAATTATGCTTGATATCTAAACCGGCTTAACTAGCCGTAT

Blaine Creek ACGTGACCGACCTCTTTCCAAAATTATGCTTGATATCTAAACCGGCTTAACTAGCCGTAT

09-CS0040 ACGTGACCGACCTCTTTCCAAAATTATGCTTGATATCTAAACCGGCTTAACTAGCCGTAT

17CS1133 ACGTGACCGACCTCTTTCCAAAATTATGCTTGATATCTAAACCGGCTTAACTAGCCGTAT

Jasper ACGTGACCGACCTCTTTCCAAAATTATGCTTGATATCTAAACCGGCTTAACTAGCCGTAT

CN 120017 ACGTGACCGACCTCTTTCCAAAATTATGCTTGATATCTAAACCGGCTTAACTAGCCGTAT

CN 120027 ACGTGACCGACCTCTTTCCAAAATTATGCTTGATATCTAAACCGGCTTAACTAGCCGTAT

CN 120030 ACGTGACCGACCTCTTTCCAAAATTATGCTTGATATCTAAACCGGCTTAACTAGCCGTAT

Hoga ACGTGACCGACCTCTTTCCAAAATTATGCTTGATATCTAAACCGGCTTAACTAGCCGTAT

Yellowstone ACGTGACCGACCTCTTTCCAAAATTATGCTTGATATCTAAACCGGCTTAACTAGCCGTAT

CN 119294 ACGTGACCGACCTCTTTCCAAAATTATGCTTGATATCTAAACCGGCTTAACTAGCCGTAT

************************************************************

CN 120025 AAAACTTCATTTGAACTAATCATAGGACTTGACTTCGAACTTAGGAAAATAAAATACACA

CN 119205 AAAACTTCATTTGAACTAATCATAGGACTTGACTTCGAACTTAGGAAAACAAAATACACA

CN 119243 AAAACTTCATTTGAACTAATCATAGGACTTGACTTCGAACTTAGGAAAATAAAATACACA

Joelle NCBI AAAACTTCATTTGAACTAATCATAGGACTTGACTTCGAACTTAGGAAAATAAAATACACA

Joelle AAFC AAAACTTCATTTGAACTAATCATAGGACTTGACTTCGAACTTAGGAAAATAAAATACACA

Joelle phyto AAAACTTCATTTGAACTAATCATAGGACTTGACTTCGAACTTAGGAAAATAAAATACACA

DH55 ref genome AAAACTTCATTTGAACTAATCATAGGACTTGACTTCGAACTTAGGAAAATAAAATACACA

CN 119300 AAAACTTCATTTGAACTAATCATAGGACTTGACTTCGAACTTAGGAAAATAAAATACACA

CAM 241 AAAACTTCATTTGAACTAATCATAGGACTTGACTTCGAACTTAGGAAAATAAAATACACA

CN 113754 AAAACTTCATTTGAACTAATCATAGGACTTGACTTCGAACTTAGGAAAATAAAATACACA

CAM 236 AAAACTTCATTTGAACTAATCATAGGACTTGACTTCGAACTTAGGAAAATAAAATACACA

CO46 NCBI AAAACTTCATTTGAACTAATCATAGGACTTGACTTCGAACTTAGGAAAATAAAATACACA

CN 120013 AAAACTTCATTTGAACTAATCATAGGACTTGACTTCGAACTTAGGAAAATAAAATACACA

Blaine Creek AAAACTTCATTTGAACTAATCATAGGACTTGACTTCGAACTTAGGAAAATAAAATACACA

09-CS0040 AAAACTTCATTTGAACTAATCATAGGACTTGACTTCGAACTTAGGAAAATAAAATACACA

17CS1133 AAAACTTCATTTGAACTAATCATAGGACTTGACTTCGAACTTAGGAAAATAAAATACACA

Jasper AAAACTTCATTTGAACTAATCATAGGACTTGACTTCGAACTTAGGAAAATAAAATACACA

CN 120017 AAAACTTCATTTGAACTAATCATAGGACTTGACTTCGAACTTAGGAAAATAAAATACACA

CN 120027 AAAACTTCATTTGAACTAATCATAGGACTTGACTTCGAACTTAGGAAAATAAAATACACA

CN 120030 AAAACTTCATTTGAACTAATCATAGGACTTGACTTCGAACTTAGGAAAATAAAATACACA

Hoga AAAACTTCATTTGAACTAATCATAGGACTTGACTTCGAACTTAGGAAAATAAAATACACA

Yellowstone AAAACTTCATTTGAACTAATCATAGGACTTGACTTCGAACTTAGGAAAATAAAATACACA

CN 119294 AAAACTTCATTTGAACTAATCATAGGACTTGACTTCGAACTTAGGAAAATAAAATACACA

************************************************* **********

CN 120025 TCTATCTGTTATCGTCGCCAGTCACTTTCAACTTCTGATCCTCTAACTATGAGCGTTGGA

CN 119205 TCTATCTGTTATCGTCGCCAGTCACTTTCAACTTCTGATCCTCTAACTATGAGCGTTGGA

CN 119243 TCTATCTGTTATCGTCGCCAGTCACTTTCAACTTCTGATCCTCTAACTATGAGCGTTGGA

Joelle NCBI TCTATCTGTTATCGTCGCCAGTCACTTTCAACTTCTGATCCTCTAACTATGAGCGTTGGA

Joelle AAFC TCTATCTGTTATCGTCGCCAGTCACTTTCAACTTCTGATCCTCTAACTATGAGCGTTGGA

Joelle phyto TCTATCTGTTATCGTCGCCAGTCACTTTCAACTTCTGATCCTCTAACTATGAGCGTTGGA

DH55 ref genome TCTATCTGTTATCGTCGCCAGTCACTTTCAACTTCTGATCCTCTAACTATGAGCGTTGGA

CN 119300 TCTATCTGTTATCGTCGCCAGTCACTTTCAACTTCTGATCCTCTAACTATGAGCGTTGGA

CAM 241 TCTATCTGTTATCGTCGCCAGTCACTTTCAACTTCTGATCCTCTAACTATGAGCGTTGGA

CN 113754 TCTATCTGTTATCGTCGCCAGTCACTTTCAACTTCTGATCCTCTAACTATGAGCGTTGGA

CAM 236 TCTATCTGTTATCGTCGCCAGTCACTTTCAACTTCTGATCCTCTAACTATGAGCGTTGGA

CO46 NCBI TCTATCTGTTATCGTCGCCAGTCACTTTCAACTTCTGATCCTCTAACTATGAGCGTTGGA

CN 120013 TCTATCTGTTATCGTCGCCAGTCACTTTCAACTTCTGATCCTCTAACTATGAGCGTTGGA

Blaine Creek TCTATCTGTTATCGTCGCCAGTCACTTTCAACTTCTGATCCTCTAACTATGAGCGTTGGA

09-CS0040 TCTATCTGTTATCGTCGCCAGTCACTTTCAACTTCTGATCCTCTAACTATGAGCGTTGGA

17CS1133 TCTATCTGTTATCGTCGCCAGTCACTTTCAACTTCTGATCCTCTAACTATGAGCGTTGGA

Jasper TCTATCTGTTATCGTCGCCAGTCACTTTCAACTTCTGATCCTCTAACTATGAGCGTTGGA

CN 120017 TCTATCTGTTATCGTCGCCAGTCACTTTCAACTTCTGATCCTCTAACTATGAGCGTTGGA

CN 120027 TCTATCTGTTATCGTCGCCAGTCACTTTCAACTTCTGATCCTCTAACTATGAGCGTTGGA

CN 120030 TCTATCTGTTATCGTCGCCAGTCACTTTCAACTTCTGATCCTCTAACTATGAGCGTTGGA

Hoga TCTATCTGTTATCGTCGCCAGTCACTTTCAACTTCTGATCCTCTAACTATGAGCGTTGGA

Yellowstone TCTATCTGTTATCGTCGCCAGTCACTTTCAACTTCTGATCCTCTAACTATGAGCGTTGGA

CN 119294 TCTATCTGTTATCGTCGCCAGTCACTTTCAACTTCTGATCCTCTAACTATGAGCGTTGGA

************************************************************

CN 120025 TAAAATCGTAAAAGCCATAACTACTACTAATTTAATTTAGATCGCTACGCCACTGTGAAC

CN 119205 TAAAATCGTAAAAGCCATAACTACTACTAATTTAATTTAGATCGCTACGCCACTGTGAAC

CN 119243 TAAAATCGTAAAAGCCATAACTACTACTAATTTAATTTAGATCGCTACGCCACTGTGAAC

Joelle NCBI TAAAATCGTAAAAGCCATAACTACTACTAATTTAATTTAGATCGCTACGCCACTGTGAAC

Joelle AAFC TAAAATCGTAAAAGCCATAACTACTACTAATTTAATTTAGATCGCTACGCCACTGTGAAC

Joelle phyto TAAAATCGTAAAAGCCATAACTACTACTAATTTAATTTAGATCGCTACGCCACTGTGAAC

DH55 ref genome TAAAATCGTAAAAGCCATAACTACTACTAATTTAATTTAGATCGCTACGCCACTGTGAAC

CN 119300 TAAAATCGTAAAAGCCATAACTACTACTAATTTAATTTAGATCGCTACGCCACTGTGAAC

CAM 241 TAAAATCGTAAAAGCCATAACTACTACTAATTTAATTTAGATCGCTACGCCACTGTGAAC

CN 113754 TAAAATCGTAAAAGCCATAACTACTACTAATTTAATTTAGATCGCTACGCCACTGTGAAC

CAM 236 TAAAATCGTAAAAGCCATAACTACTACTAATTTAATTTAGATCGCTACGCCACTGTGAAC

CO46 NCBI TAAAATCGTAAAAGCCATAACTACTACTAATTTAATTTAGATCGCTACGCCACTGTGAAC

CN 120013 TAAAATCGTAAAAGCCATAACTACTACTAATTTAATTTAGATCGCTACGCCACTGTGAAC

Blaine Creek TAAAATCGTAAAAGCCATAACTACTACTAATTTAATTTAGATCGCTACGCCACTGTGAAC

09-CS0040 TAAAATCGTAAAAGCCATAACTACTACTAATTTAATTTAGATCGCTACGCCACTGTGAAC

17CS1133 TAAAATCGTAAAAGCCATAACTACTACTAATTTAATTTAGATCGCTACGCCACTGTGAAC

Jasper TAAAATCGTAAAAGCCATAACTACTACTAATTTAATTTAGATCGCTACGCCACTGTGAAC

CN 120017 TAAAATCGTAAAAGCCATAACTACTACTAATTTAATTTAGATCGCTACGCCACTGTGAAC

CN 120027 TAAAATCGTAAAAGCCATAACTACTACTAATTTAATTTAGATCGCTACGCCACTGTGAAC

CN 120030 TAAAATCGTAAAAGCCATAACTACTACTAATTTAATTTAGATCGCTACGCCACTGTGAAC

Hoga TAAAATCGTAAAAGCCATAACTACTACTAATTTAATTTAGATCGCTACGCCACTGTGAAC

Yellowstone TAAAATCGTAAAAGCCATAACTACTACTAATTTAATTTAGATCGCTACGCCACTGTGAAC

CN 119294 TAAAATCGTAAAAGCCATAACTACTACTAATTTAATTTAGATCGCTACGCCACTGTGAAC

************************************************************

CN 120025 AGTTAACCTCCTTCAACTATTCTGATATAAATTAAATTATAATAACCTCCTTCAACTATT

CN 119205 AGTTAACCTCCTTCAACTATTCTGATATAAATTAAATTATAATAACCTCCTTCAACTATT

CN 119243 AGTTAACCTCCTTCAACTATTCTGATATAAATTAAATTATAATAACCTCCTTCAACTATT

Joelle NCBI AGTTAACCTCCTTCAACTATTCTGATATAAATTAAATTATAATAACCTCCTTCAACTATT

Joelle AAFC AGTTAACCTCCTTCAACTATTCTGATATAAATTAAATTATAATAACCTCCTTCAACTATT

Joelle phyto AGTTAACCTCCTTCAACTATTCTGATATAAATTAAATTATAATAACCTCCTTCAACTATT

DH55 ref genome AGTTAACCTCCTTCAACTATTCTGATATAAATTAAATTATAATAACCTCCTTCAACTATT

CN 119300 AGTTAACCTCCTTCAACTATTCTGATATAAATTAAATTATAATAACCTCCTTCAACTATT

CAM 241 AGTTAACCTCCTTCAACTATTCTGATATAAATTAAATTATAATAACCTCCTTCAACTATT

CN 113754 AGTTAACCTCCTTCAACTATTCTGATATAAATTAAATTATAATAACCTCCTTCAACTATT

CAM 236 AGTTAACCTCCTTCAACTATTCTGATATAAATTAAATTATAATAACCTCCTTCAACTATT

CO46 NCBI AGTTAACCTCCTTCAACTATTCTGATATAAATTAAATTATAATAACCTCCTTCAACTATT

CN 120013 AGTTAACCTCCTTCAACTATTCTGATATAAATTAAATTATAATAACCTCCTTCAACTATT

Blaine Creek AGTTAACCTCCTTCAACTATTCTGATATAAATTAAATTATAATAACCTCCTTCAACTATT

09-CS0040 AGTTAACCTCCTTCAACTATTCTGATATAAATTAAATTATAATAACCTCCTTCAACTATT

17CS1133 AGTTAACCTCCTTCAACTATTCTGATATAAATTAAATTATAATAACCTCCTTCAACTATT

Jasper AGTTAACCTCCTTCAACTATTCTGATATAAATTAAATTATAATAACCTCCTTCAACTATT

CN 120017 AGTTAACCTCCTTCAACTATTCTGATATAAATTAAATTATAATAACCTCCTTCAACTATT

CN 120027 AGTTAACCTCCTTCAACTATTCTGATATAAATTAAATTATAATAACCTCCTTCAACTATT

CN 120030 AGTTAACCTCCTTCAACTATTCTGATATAAATTAAATTATAATAACCTCCTTCAACTATT

Hoga AGTTAACCTCCTTCAACTATTCTGATATAAATTAAATTATAATAACCTCCTTCAACTATT

Yellowstone AGTTAACCTCCTTCAACTATTCTGATATAAATTAAATTATAATAACCTCCTTCAACTATT

CN 119294 AGTTAACCTCCTTCAACTATTCTGATATAAATTAAATTATAATAACCTCCTTCAACTATT

************************************************************

CN 120025 CTGATCTAAATTAAATTATAATCTATACAACCAGCCTACAATATACGGAATCGTTAAGAG

CN 119205 CTGATCTAAATTAAATTATAATCTATACAACCAGCCTACAATATACGGAATCGTTAAGAG

CN 119243 CTGATCTAAATTAAATTATAATCTATACAACCAGCCTACAATATACGGAATCGTTAAGAG

Joelle NCBI CTGATCTAAATTAAATTATAATCTATACAACCAGCCTACAATATACGGAATCGTTAAGAG

Joelle AAFC CTGATCTAAATTAAATTATAATCTATACAACCAGCCTACAATATACGGAATCGTTAAGAG

Joelle phyto CTGATCTAAATTAAATTATAATCTATACAACCAGCCTACAATATACGGAATCGTTAAGAG

DH55 ref genome CTGATCTAAATTAAATTATAATCTATACAACCAGCCTACAATATACGGAATCGTTAAGAG

CN 119300 CTGATCTAAATTAAATTATAATCTATACAACCAGCCTACAATATACGGAATCGTTAAGAG

CAM 241 CTGATCTAAATTAAATTATAATCTATACAACCAGCCTACAATATACGGAATCGTTAAGAG

CN 113754 CTGATCTAAATTAAATTATAATCTATACAACCAGCCTACAATATACGGAATCGTTAAGAG

CAM 236 CTGATCTAAATTAAATTATAATCTATACAACCAGCCTACAATATACGGAATCGTTAAGAG

CO46 NCBI CTGATCTAAATTAAATTATAATCTATACAACCAGCCTACAATATACGGAATCGTTAAGAG

CN 120013 CTGATCTAAATTAAATTATAATCTATACAACCAGCCTACAATATACGGAATCGTTAAGAG

Blaine Creek CTGATCTAAATTAAATTATAATCTATACAACCAGCCTACAATATACGGAATCGTTAAGAG

09-CS0040 CTGATCTAAATTAAATTATAATCTATACAACCAGCCTACAATATACGGAATCGTTAAGAG

17CS1133 CTGATCTAAATTAAATTATAATCTATACAACCAGCCTACAATATACGGAATCGTTAAGAG

Jasper CTGATCTAAATTAAATTATAATCTATACAACCAGCCTACAATATACGGAATCGTTAAGAG

CN 120017 CTGATCTAAATTAAATTATAATCTATACAACCAGCCTACAATATACGGAATCGTTAAGAG

CN 120027 CTGATCTAAATTAAATTATAATCTATACAACCAGCCTACAATATACGGAATCGTTAAGAG

CN 120030 CTGATCTAAATTAAATTATAATCTATACAACCAGCCTACAATATACGGAATCGTTAAGAG

Hoga CTGATCTAAATTAAATTATAATCTATACAACCAGCCTACAATATACGGAATCGTTAAGAG

Yellowstone CTGATCTAAATTAAATTATAATCTATACAACCAGCCTACAATATACGGAATCGTTAAGAG

CN 119294 CTGATCTAAATTAAATTATAATCTATACAACCAGCCTACAATATACGGAATCGTTAAGAG

************************************************************

CN 120025 TTAAAATGCAAACTACGTAAAGAGGAAACATAAGCACGTTCTGTCGGACAGAAGCTAAGC

CN 119205 TTAAAATGCAAACTACGTAAAGAGGAAACATAAGCACGTTCTGTCGGACAGAAGCTAAGC

CN 119243 TTAAAATGCAAACTACGTAAAGAGGAAACATAAGCACGTTCTGTCGGACAGAAGCTAAGC

Joelle NCBI TTAAAATGCAAACTACGTAAAGAGGAAACATAAGCACGTTCTGTCGGACAGAAGCTAAGC

Joelle AAFC TTAAAATGCAAACTACGTAAAGAGGAAACATAAGCACGTTCTGTCGGACAGAAGCTAAGC

Joelle phyto TTAAAATGCAAACTACGTAAAGAGGAAACATAAGCACGTTCTGTCGGACAGAAGCTAAGC

DH55 ref genome TTAAAATGCAAACTACGTAAAGAGGAAACATAAGCACGTTCTGTCGGACAGAAGCTAAGC

CN 119300 TTAAAATGCAAACTACGTAAAGAGGAAACATAAGCACGTTCTGTCGGACAGAAGCTAAGC

CAM 241 TTAAAATGCAAACTACGTAAAGAGGAAACATAAGCACGTTCTGTCGGACAGAAGCTAAGC

CN 113754 TTAAAATGCAAACTACGTAAAGAGGAAACATAAGCACGTTCTGTCGGACAGAAGCTAAGC

CAM 236 TTAAAATGCAAACTACGTAAAGAGGAAACATAAGCACGTTCTGTCGGACAGAAGCTAAGC

CO46 NCBI TTAAAATGCAAACTACGTAAAGAGGAAACATAAGCACGTTCTGTCGGACAGAAGCTAAGC

CN 120013 TTAAAATGCAAACTACGTAAAGAGGAAACATAAGCACGTTCTGTCGGACAGAAGCTAAGC

Blaine Creek TTAAAATGCAAACTACGTAAAGAGGAAACATAAGCACGTTCTGTCGGACAGAAGCTAAGC

09-CS0040 TTAAAATGCAAACTACGTAAAGAGGAAACATAAGCACGTTCTGTCGGACAGAAGCTAAGC

17CS1133 TTAAAATGCAAACTACGTAAAGAGGAAACATAAGCACGTTCTGTCGGACAGAAGCTAAGC

Jasper TTAAAATGCAAACTACGTAAAGAGGAAACATAAGCACGTTCTGTCGGACAGAAGCTAAGC

CN 120017 TTAAAATGCAAACTACGTAAAGAGGAAACATAAGCACGTTCTGTCGGACAGAAGCTAAGC

CN 120027 TTAAAATGCAAACTACGTAAAGAGGAAACATAAGCACGTTCTGTCGGACAGAAGCTAAGC

CN 120030 TTAAAATGCAAACTACGTAAAGAGGAAACATAAGCACGTTCTGTCGGACAGAAGCTAAGC

Hoga TTAAAATGCAAACTACGTAAAGAGGAAACATAAGCACGTTCTGTCGGACAGAAGCTAAGC

Yellowstone TTAAAATGCAAACTACGTAAAGAGGAAACATAAGCACGTTCTGTCGGACAGAAGCTAAGC

CN 119294 TTAAAATGCAAACTACGTAAAGAGGAAACATAAGCACGTTCTGTCGGACAGAAGCTAAGC

************************************************************

CN 120025 AATTTCATCACTCTATTGTACATAGTAGTTATTCCACAAACACTACAAG-AAAACATAGT

CN 119205 AATTTCATCACTCTATTGTACATAGTAGTTATTCCACAAACACTACAAG-AAAAGATAGT

CN 119243 AATTTCATCACTCTATTGTACATAGTAGTTATTCCACAAACACTACAAG-AAAAGATAGT

Joelle NCBI AATTTCATCACTCTATTGTACATAGTAGTTATTCCACAAACACTACAAG-AAAAGATAGT

Joelle AAFC AATTTCATCACTCTATTGTACATAGTAGTTATTCCACAAACACTACAAG-AAAAGATAGT

Joelle phyto AATTTCATCACTCTATTGTACATAGTAGTTATTCCACAAACACTACAAG-AAAAGATAGT

DH55 ref genome AATTTCATCACTCTATTGTACATAGTAGTTATTCCACAAACACTACAAG-AAAAGATAGT

CN 119300 AATTTCATCACTCTATTGTACATAGTAGTTATTCCACAAACACTACAAG-AAAAGATAGT

CAM 241 AATTTCATCACTCTATTGTACATAGTAGTTATTCCACAAACACTACAAG-AAAAGATAGT

CN 113754 AATTTCATCACTCTATTGTACATAGTAGTTATTCCACAAACACTACAAG-AAAAGATAGT

CAM 236 AATTTCATCACTCTATTGTACATAGTAGTTATTCCACAAACACTACAAG-AAAAGATAGT

CO46 NCBI AATTTCATCACTCTATTGTACATAGTAGTTATTCCACAAACACTACAAG-AAAAGATAGT

CN 120013 AATTTCATCACTCTATTGTACATAGTAGTTATTCCACAAACACTACAAGAAAAAGATAGT

Blaine Creek AATTTCATCACTCTATTGTACATAGTAGTTATTCCACAAACACTACAAG-AAAAGATAGT

09-CS0040 AATTTCATCACTCTATTGTACATAGTAGTTATTCCACAAACACTACAAG-AAAAGATAGT

17CS1133 AATTTCATCACTCTATTGTACATAGTAGTTATTCCACAAACACTACAAG-AAAAGATAGT

Jasper AATTTCATCACTCTATTGTACATAGTAGTTATTCCACAAACACTACAAG-AAAAGATAGT

CN 120017 AATTTCATCACTCTATTGTACATAGTAGTTATTCCACAAACACTACAAG-AAAAGATAGT

CN 120027 AATTTCATCACTCTATTGTACATAGTAGTTATTCCACAAACACTACAAG-AAAAGATAGT

CN 120030 AATTTCATCACTCTATTGTACATAGTAGTTATTCCACAAACACTACAAG-AAAAGATAGT

Hoga AATTTCATCACTCTATTGTACATAGTAGTTATTCCACAAACACTACAAG-AAAAGATAGT

Yellowstone AATTTCATCACTCTATTGTACATAGTAGTTATTCCACAAACACTACAAG-AAAAGATAGT

CN 119294 AATTTCATCACTCTATTGTACATAGTAGTTATTCCACAAACACTACAAG-AAAAGATAGT

************************************************* **** *****

CN 120025 TTTTGCAACGAATTTTAGTGAGGAAAGTAGAACGTTGCAACGTAATGACATTTTTCAAAT

CN 119205 TTTTGCAACGAATTTTAGTGAGGAAAGTAGAACGTTGCAACGTAATGACATTTTTCAAAT

CN 119243 TTTTGCAACGAATTTTAGTGAGGAAAGTAGAACGTTGCAACGTAATGACATTTTTCAAAT

Joelle NCBI TTTTGCAACGAATTTTAGTGAGGAAAGTAGAACGTTGCAACGTAATGACATTTTTCAAAT

Joelle AAFC TTTTGCAACGAATTTTAGTGAGGAAAGTAGAACGTTGCAACGTAATGACATTTTTCAAAT

Joelle phyto TTTTGCAACGAATTTTAGTGAGGAAAGTAGAACGTTGCAACGTAATGACATTTTTCAAAT

DH55 ref genome TTTTGCAACGAATTTTAGTGAGGAAAGTAGAACGTTGCAACGTAATGACATTTTTCAAAT

CN 119300 TTTTGCAACGAATTTTAGTGAGGAAAGTAGAACGTTGCAACGTAATGACATTTTTCAAAT

CAM 241 TTTTGCAACGAATTTTAGTGAGGAAAGTAGAACGTTGCAACGTAATGACATTTTTCAAAT

CN 113754 TTTTGCAACGAATTTTAGTGAGGAAAGTAGAACGTTGCAACGTAATGACATTTTTCAAAT

CAM 236 TTTTGCAACGAATTTTAGTGAGGAAAGTAGAACGTTGCAACGTAATGACATTTTTCAAAT

CO46 NCBI TTTTGCAACGAATTTTAGTGAGGAAAGTAGAACGTTGCAACGTAATGACATTTTTCAAAT

CN 120013 TTTTGCAACGAATTTTAGTGAGGAAAGTAGAACGTTGCAACGTAATGACATTTTTCAAAT

Blaine Creek TTTTGCAACGAATTTTAGTGAGGAAAGTAGAACGTTGCAACGTAATGACATTTTTCAAAT

09-CS0040 TTTTGCAACGAATTTTAGTGAGGAAAGTAGAACGTTGCAACGTAATGACATTTTTCAAAT

17CS1133 TTTTGCAACGAATTTTAGTGAGGAAAGTAGAACGTTGCAACGTAATGACATTTTTCAAAT

Jasper TTTTGCAACGAATTTTAGTGAGGAAAGTAGAACGTTGCAACGTAATGACATTTTTCAAAT

CN 120017 TTTTGCAACGAATTTTAGTGAGGAAAGTAGAACGTTGCAACGTAATGACATTTTTCAAAT

CN 120027 TTTTGCAACGAATTTTAGTGAGGAAAGTAGAACGTTGCAACGTAATGACATTTTTCAAAT

CN 120030 TTTTGCAACGAATTTTAGTGAGGAAAGTAGAACGTTGCAACGTAATGACATTTTTCAAAT

Hoga TTTTGCAACGAATTTTAGTGAGGAAAGTAGAACGTTGCAACGTAATGACATTTTTCAAAT

Yellowstone TTTTGCAACGAATTTTAGTGAGGAAAGTAGAACGTTGCAACGTAATGACATTTTTCAAAT

CN 119294 TTTTGCAACGAATTTTAGTGAGGAAAGTAGAACGTTGCAACGTAATGACATTTTTCAAAT

************************************************************

CN 120025 GATTTAGTGACTTTATAACAAATTGTCGCAAATTCGTCACTATTTTGTGATTATTTTAAT

CN 119205 GATTTAGTGACTTTATAACAAATTGTCGCAAATTCGTCACTATTTTGTGATTATTTTAAT

CN 119243 GATTTAGTGACTTTATAACAAATTGTCGCAAATTCGTCACTATTTTGTGATTATTTTAAT

Joelle NCBI GATTTAGTGACTTTATAACAAATTGTCGCAAATTCGTCACTATTTTGTGATTATTTTAAT

Joelle AAFC GATTTAGTGACTTTATAACAAATTGTCGCAAATTCGTCACTATTTTGTGATTATTTTAAT

Joelle phyto GATTTAGTGACTTTATAACAAATTGTCGCAAATTCGTCACTATTTTGTGATTATTTTAAT

DH55 ref genome GATTTAGTGACTTTATAACAAATTGTCGCAAATTCGTCACTATTTTGTGATTATTTTAAT

CN 119300 GATTTAGTGACTTTATAACAAATTGTCGCAAATTCGTCACTATTTTGTGATTATTTTAAT

CAM 241 GATTTAGTGACTTTATAACAAATTGTCGCAAATTCGTCACTATTTTGTGATTATTTTAAT

CN 113754 GATTTAGTGACTTTATAACAAATTGTCGCAAATTCGTCACTATTTTGTGATTATTTTAAT

CAM 236 GATTTAGTGACTTTATAACAAATTGTCGCAAATTCGTCACTATTTTGTGATTATTTTAAT

CO46 NCBI GATTTAGTGACTTTATAACAAATTGTCGCAAATTCGTCACTATTTTGTGATTATTTTAAT

CN 120013 GATTTAGTGACTTTATAACAAATTGTCGCAAATTCGTCACTATTTTGTGATTATTTTAAT

Blaine Creek GATTTAGTGACTTTATAACAAATTGTCGCAAATTCGTCACTATTTTGTGATTATTTTAAT

09-CS0040 GATTTAGTGACTTTATAACAAATTGTCGCAAATTCGTCACTATTTTGTGATTATTTTAAT

17CS1133 GATTTAGTGACTTTATAACAAATTGTCGCAAATTCGTCACTATTTTGTGATTATTTTAAT

Jasper GATTTAGTGACTTTATAACAAATTGTCGCAAATTCGTCACTATTTTGTGATTATTTTAAT

CN 120017 GATTTAGTGACTTTATAACAAATTGTCGCAAATTCGTCACTATTTTGTGATTATTTTAAT

CN 120027 GATTTAGTGACTTTATAACAAATTGTCGCAAATTCGTCACTATTTTGTGATTATTTTAAT

CN 120030 GATTTAGTGACTTTATAACAAATTGTCGCAAATTCGTCACTATTTTGTGATTATTTTAAT

Hoga GATTTAGTGACTTTATAACAAATTGTCGCAAATTCGTCACTATTTTGTGATTATTTTAAT

Yellowstone GATTTAGTGACTTTATAACAAATTGTCGCAAATTCGTCACTATTTTGTGATTATTTTAAT

CN 119294 GATTTAGTGACTTTATAACAAATTGTCGCAAATTCGTCACTATTTTGTGATTATTTTAAT

************************************************************

CN 120025 CATCACTAAGCTGTGGTGAGTTTGGGTCGATTTAGCAACGCTTTTAAGATGTTTATTTAA

CN 119205 CATCACTAAGCTGTGGTGAGTTTGGGTCGATTTAGCAACGCTTTTAAGATGTTTATTAAA

CN 119243 CATCACTAAGCTGTGGTGAGTTTGGGTCGATTTAGCAACGCTTTTAAGATGTTTATTAAA

Joelle NCBI CATCACTAAGCTGTGGTGAGTTTGGGTCGATTTAGCAACGCTTTTAAGATGTTTATTAAA

Joelle AAFC CATCACTAAGCTGTGGTGAGTTTGGGTCGATTTAGCAACGCTTTTAAGATGTTTATTAAA

Joelle phyto CATCACTAAGCTGTGGTGAGTTTGGGTCGATTTAGCAACGCTTTTAAGATGTTTATTAAA

DH55 ref genome CATCACTAAGCTGTGGTGAGTTTGGGTCGATTTAGCAACGCTTTTAAGATGTTTATTAAA

CN 119300 CATCACTAAGCTGTGGTGAGTTTGGGTCGATTTAGCAACGCTTTTAAGATGTTTATTAAA

CAM 241 CATCACTAAGCTGTGGTGAGTTTGGGTCGATTTAGCAACGCTTTTAAGATGTTTATTAAA

CN 113754 CATCACTAAGCTGTGGTGAGTTTGGGTCGATTTAGCAACGCTTTTAAGATGTTTATTAAA

CAM 236 CATCACTAAGCTGTGGTGAGTTTGGGTCGATTTAGCAACGCTTTTAAGATGTTTATTAAA

CO46 NCBI CATCACTAAGCTGTGGTGAGTTTGGGTCGATTTAGCAACGCTTTTAAGATGTTTATTAAA

CN 120013 CATCACTAAGCTGTGGTGAGTTTGGGTCGATTTAGCAACGCTTTTAAGATGTTTATTAAA

Blaine Creek CATCACTAAGCTGTGGTGAGTTTGGGTCGATTTAGCAACGCTTTTAAGATGTTTATTAAA

09-CS0040 CATCACTAAGCTGTGGTGAGTTTGGGTCGATTTAGCAACGCTTTTAAGATGTTTATTAAA

17CS1133 CATCACTAAGCTGTGGTGAGTTTGGGTCGATTTAGCAACGCTTTTAAGATGTTTATTAAA

Jasper CATCACTAAGCTGTGGTGAGTTTGGGTCGATTTAGCAACGCTTTTAAGATGTTTATTAAA

CN 120017 CATCACTAAGCTGTGGTGAGTTTGGGTCGATTTAGCAACGCTTTTAAGATGTTTATTAAA

CN 120027 CATCACTAAGCTGTGGTGAGTTTGGGTCGATTTAGCAACGCTTTTAAGATGTTTATTAAA

CN 120030 CATCACTAAGCTGTGGTGAGTTTGGGTCGATTTAGCAACGCTTTTAAGATGTTTATTAAA

Hoga CATCACTAAGCTGTGGTGAGTTTGGGTCGATTTAGCAACGCTTTTAAGATGTTTATTAAA

Yellowstone CATCACTAAGCTGTGGTGAGTTTGGGTCGATTTAGCAACGCTTTTAAGATGTTTATTAAA

CN 119294 CATCACTAAGCTGTGGTGAGTTTGGGTCGATTTAGCAACGCTTTTAAGATGTTTATTAAA

********************************************************* **

CN 120025 GGCGTCACTATATTATTGTTAGAGCATGACTATTGTTTTTAAAATAAGGAAGAAAACTAA

CN 119205 GGCGTCACTATACTATTGTTAGAGCATGACTATTGTTTTTAAAATAAGGAAGAAAACTAA

CN 119243 GGCGTCACTATACTATTGTTAGAGCATGACTATTGTTTTTAAAGTAAGGAAGAAAACTAA

Joelle NCBI GGCGTCACTATACTATTGTTAGAGCATGACTATTGTTTTTAAAGTAAGGAAGAAAACTAA

Joelle AAFC GGCGTCACTATACTATTGTTAGAGCATGACTATTGTTTTTAAAGTAAGGAAGAAAACTAA

Joelle phyto GGCGTCACTATACTATTGTTAGAGCATGACTATTGTTTTTAAAGTAAGGAAGAAAACTAA

DH55 ref genome GGCGTCACTATACTATTGTTAGAGCATGACTATTGTTTTTAAAATAAGGAAGAAAACTAA

CN 119300 GGCGTCACTATACTATTGTTAGAGCATGACTATTGTTTTTAAAATAAGGAAGAAAACTAA

CAM 241 GGCGTCACTATACTATTGTTAGAGCATGACTATTGTTTTTAAAATAAGGAAGAAAACTAA

CN 113754 GGCGTCACTATACTATTGTTAGAGCATGACTATTGTTTTTAAAATAAGGAAGAAAACTAA

CAM 236 GGCGTCACTATACTATTGTTAGAGCATGACTATTGTTTTTAAAATAAGGAAGAAAACTAA

CO46 NCBI GGCGTCACTATACTATTGTTAGAGCATGACTATTGTTTTTAAAATAAGGAAGAAAACTAA

CN 120013 GGCGTCACTATACTATTGTTAGAGCATGACTATTGTTTTTAAAATAAGGAAGAAAACTAA

Blaine Creek GGCGTCACTATACTATTGTTAGAGCATGACTATTGTTTTTAAAATAAGGAAGAAAACTAA

09-CS0040 GGCGTCACTATACTATTGTTAGAGCATGACTATTGTTTTTAAAATAAGGAAGAAAACTAA

17CS1133 GGCGTCACTATACTATTGTTAGAGCATGACTATTGTTTTTAAAATAAGGAAGAAAACTAA

Jasper GGCGTCACTATACTATTGTTAGAGCATGACTATTGTTTTTAAAATAAGGAAGAAAACTAA

CN 120017 GGCGTCACTATACTATTGTTAGAGCATGACTATTGTTTTTAAAATAAGGAAGAAAACTAA

CN 120027 GGCGTCACTATACTATTGTTAGAGCATGACTATTGTTTTTAAAATAAGGAAGAAAACTAA

CN 120030 GGCGTCACTATACTATTGTTAGAGCATGACTATTGTTTTTAAAATAAGGAAGAAAACTAA

Hoga GGCGTCACTATACTATTGTTAGAGCATGACTATTGTTTTTAAAATAAGGAAGAAAACTAA

Yellowstone GGCGTCACTATACTATTGTTAGAGCATGACTATTGTTTTTAAAATAAGGAAGAAAACTAA

CN 119294 GGCGTCACTATACTATTGTTAGAGCATGACTATTGTTTTTAAAATAAGGAAGAAAACTAA

************ ****************************** ****************

CN 120025 TATTAGTGTTTATAACTCATCGTGGCAACACTGTCACAAAATTTCCAATTAATTTCTCAC

CN 119205 TATTAGTGTTTATAACTCATCGTGGCAACACTGTCACAAAATTTCCAATTAATTTCTCAC

CN 119243 TATTAGTGTTTATAACTCATCGTGGCAACACTGTCACAAAATTTCCAATTAATTTCTCAC

Joelle NCBI TATTAGTGTTTATAACTCATCGTGGCAACACTGTCACAAAATTTCCAATTAATTTCTCAC

Joelle AAFC TATTAGTGTTTATAACTCATCGTGGCAACACTGTCACAAAATTTCCAATTAATTTCTCAC

Joelle phyto TATTAGTGTTTATAACTCATCGTGGCAACACTGTCACAAAATTTCCAATTAATTTCTCAC

DH55 ref genome TATTAGTGTTTATAACTCATCGTGGCAACACTGTCACAAAATTTCCAATTAATTTCTCAC

CN 119300 TATTAGTGTTTATAACTCATCGTGGCAACACTGTCACAAAATTTCCAATTAATTTCTCAC

CAM 241 TATTAGTGTTTATAACTCATCGTGGCAACACTGTCACAAAATTTCCAATTAATTTCTCAC

CN 113754 TATTAGTGTTTATAACTCATCGTGGCAACACTGTCACAAAATTTCCAATTAATTTCTCAC

CAM 236 TATTAGTGTTTATAACTCATCGTGGCAACACTGTCACAAAATTTCCAATTAATTTCTCAC

CO46 NCBI TATTAGTGTTTATAACTCATCGTGGCAACACTGTCACAAAATTTCCAATTAATTTCTCAC

CN 120013 TATTAGTGTTTATAACTCATCGTGGCAACACTGTCACAAAATTTCCAATTAATTTCTCAC

Blaine Creek TATTAGTGTTTATAACTCATCGTGGCAACACTGTCACAAAATTTCCAATTAATTTCTCAC

09-CS0040 TATTAGTGTTTATAACTCATCGTGGCAACACTGTCACAAAATTTCCAATTAATTTCTCAC

17CS1133 TATTAGTGTTTATAACTCATCGTGGCAACACTGTCACAAAATTTCCAATTAATTTCTCAC

Jasper TATTAGTGTTTATAACTCATCGTGGCAACACTGTCACAAAATTTCCAATTAATTTCTCAC

CN 120017 TATTAGTGTTTATAACTCATCGTGGCAACACTGTCACAAAATTTCCAATTAATTTCTCAC

CN 120027 TATTAGTGTTTATAACTCATCGTGGCAACACTGTCACAAAATTTCCAATTAATTTCTCAC

CN 120030 TATTAGTGTTTATAACTCATCGTGGCAACACTGTCACAAAATTTCCAATTAATTTCTCAC

Hoga TATTAGTGTTTATAACTCATCGTGGCAACACTGTCACAAAATTTCCAATTAATTTCTCAC

Yellowstone TATTAGTGTTTATAACTCATCGTGGCAACACTGTCACAAAATTTCCAATTAATTTCTCAC

CN 119294 TATTAGTGTTTATAACTCATCGTGGCAACACTGTCACAAAATTTCCAATTAATTTCTCAC

************************************************************

CN 120025 AAAGTTAACACATCATAATATTAATTTTAAAATTGTAGGAATACCGTAGCAGCGTTTTTG

CN 119205 AAAGTTAACACATCATAATATTAATTTTAAAATTGTAGGAATACCGTAGCAGCGTTTTTG

CN 119243 AAAGTTAACACATCATAATATTAATTTTAAAATTGTAGGAATACCGTAGCAGCGTTTTTG

Joelle NCBI AAAGTTAACACATCATAATATTAATTTTAAAATTGTAGGAATACCGTAGCAGCGTTTTTG

Joelle AAFC AAAGTTAACACATCATAATATTAATTTTAAAATTGTAGGAATACCGTAGCAGCGTTTTTG

Joelle phyto AAAGTTAACACATCATAATATTAATTTTAAAATTGTAGGAATACCGTAGCAGCGTTTTTG

DH55 ref genome AAAGTTAACACATCATAATATTAATTTTAAAATTGTAGGAATACCGTAGCAGCGTTTTTG

CN 119300 AAAGTTAACACATCATAATATTAATTTTAAAATTGTAGGAATACCGTAGCAGCGTTTTTG

CAM 241 AAAGTTAACACATCATAATATTAATTTTAAAATTGTAGGAATACCGTAGCAGCGTTTTTG

CN 113754 AAAGTTAACACATCATAATATTAATTTTAAAATTGTAGGAATACCGTAGCAGCGTTTTTG

CAM 236 AAAGTTAACACATCATAATATTAATTTTAAAATTGTAGGAATACCGTAGCAGCGTTTTTG

CO46 NCBI AAAGTTAACACATCATAATATTAATTTTAAAATTGTAGGAATACCGTAGCAGCGTTTTTG

CN 120013 AAAGTTAACACATCATAATATTAATTTTAAAATTGTAGGAATACCGTAGCAGCGTTTTTG

Blaine Creek AAAGTTAACACATCATAATATTAATTTTAAAATTGTAGGAATACCGTAGCAGCGTTTTTG

09-CS0040 AAAGTTAACACATCATAATATTAATTTTAAAATTGTAGGAATACCGTAGCAGCGTTTTTG

17CS1133 AAAGTTAACACATCATAATATTAATTTTAAAATTGTAGGAATACCGTAGCAGCGTTTTTG

Jasper AAAGTTAACACATCATAATATTAATTTTAAAATTGTAGGAATACCGTAGCAGCGTTTTTG

CN 120017 AAAGTTAACACATCATAATATTAATTTTAAAATTGTAGGAATACCGTAGCAGCGTTTTTG

CN 120027 AAAGTTAACACATCATAATATTAATTTTAAAATTGTAGGAATACCGTAGCAGCGTTTTTG

CN 120030 AAAGTTAACACATCATAATATTAATTTTAAAATTGTAGGAATACCGTAGCAGCGTTTTTG

Hoga AAAGTTAACACATCATAATATTAATTTTAAAATTGTAGGAATACCGTAGCAGCGTTTTTG

Yellowstone AAAGTTAACACATCATAATATTAATTTTAAAATTGTAGGAATACCGTAGCAGCGTTTTTG

CN 119294 AAAGTTAACACATCATAATATTAATTTTAAAATTGTAGGAATACCGTAGCAGCGTTTTTG

************************************************************

CN 120025 TGATGAGGTGTAAAATGGTCAAAACAGCACAAACATTATTTTCTTTACTAATAAACCCTA

CN 119205 TGATGAGGTGTAAAATGGTCAAAACAGCACAAACATTATTTTCTTTACTAATAAACCCTA

CN 119243 TGATGAGGTGTAAAATGGTCAAAACAGCACAAACATTATTTTCTTTACTAATAAACCCTA

Joelle NCBI TGATGAGGTGTAAAATGGTCAAAACAGCACAAACATTATTTTCTTTACTAATAAACCCTA

Joelle AAFC TGATGAGGTGTAAAATGGTCAAAACAGCACAAACATTATTTTCTTTACTAATAAACCCTA

Joelle phyto TGATGAGGTGTAAAATGGTCAAAACAGCACAAACATTATTTTCTTTACTAATAAACCCTA

DH55 ref genome TGATGAGGTGTAAAATGGTCAAAACAGCACAAACATTATTTTCTTTACTAATAAACCCTA

CN 119300 TGATGAGGTGTAAAATGGTCAAAACAGCACAAACATTATTTTCTTTACTAATAAACCCTA

CAM 241 TGATGAGGTGTAAAATGGTCAAAACAGCACAAACATTATTTTCTTTACTAATAAACCCTA

CN 113754 TGATGAGGTGTAAAATGGTCAAAACAGCACAAACATTATTTTCTTTACTAATAAACCCTA

CAM 236 TGATGAGGTGTAAAATGGTCAAAACAGCACAAACATTATTTTCTTTACTAATAAACCCTA

CO46 NCBI TGATGAGGTGTAAAATGGTCAAAACAGCACAAACATTATTTTCTTTACTAATAAACCCTA

CN 120013 TGATGAGGTGTAAAATGGTCAAAACAGCACAAACATTATTTTCTTTACTAATAAACCCTA

Blaine Creek TGATGAGGTGTAAAATGGTCAAAACAGCACAAACATTATTTTCTTTACTAATAAACCCTA

09-CS0040 TGATGAGGTGTAAAATGGTCAAAACAGCACAAACATTATTTTCTTTACTAATAAACCCTA

17CS1133 TGATGAGGTGTAAAATGGTCAAAACAGCACAAACATTATTTTCTTTACTAATAAACCCTA

Jasper TGATGAGGTGTAAAATGGTCAAAACAGCACAAACATTATTTTCTTTACTAATAAACCCTA

CN 120017 TGATGAGGTGTAAAATGGTCAAAACAGCACAAACATTATTTTCTTTACTAATAAACCCTA

CN 120027 TGATGAGGTGTAAAATGGTCAAAACAGCACAAACATTATTTTCTTTACTAATAAACCCTA

CN 120030 TGATGAGGTGTAAAATGGTCAAAACAGCACAAACATTATTTTCTTTACTAATAAACCCTA

Hoga TGATGAGGTGTAAAATGGTCAAAACAGCACAAACATTATTTTCTTTACTAATAAACCCTA

Yellowstone TGATGAGGTGTAAAATGGTCAAAACAGCACAAACATTATTTTCTTTACTAATAAACCCTA

CN 119294 TGATGAGGTGTAAAATGGTCAAAACAGCACAAACATTATTTTCTTTACTAATAAACCCTA

************************************************************

CN 120025 TTTGAATAGTGATCTTCTAATAGCCGTCGCAGAG-AAAAAAAAAAGAATAGTGTTCTAAC

CN 119205 TTTGAATAGTGATCTTCTAATAGCCGTCGCAGAG-AAAAAAAAAAGAATAGTGTTCTAAC

CN 119243 TTTGAATAGTGATCTTCTAATAGCCGTCGCAGAGAAAAAAAAAAAGAATAGTGTTCTAAC

Joelle NCBI TTTGAATAGTGATCTTCTAATAGCCGTCGCAGAGAAAAAAAAAAAGAATAGTGTTCTAAC

Joelle AAFC TTTGAATAGTGATCTTCTAATAGCCGTCGCAGAGAAAAAAAAAAAGAATAGTGTTCTAAC

Joelle phyto TTTGAATAGTGATCTTCTAATAGCCGTCGCAGAGAAAAAAAAAAAGAATAGTGTTCTAAC

DH55 ref genome TTTGAATAGTGATCTTCTAATAGCCGTCGCAGAG-AAAAAAAAAAGAATAGTGTTCTAAC

CN 119300 TTTGAATAGTGATCTTCTAATAGCCGTCGCAGAG-AAAAAAAAAAGAATAGTGTTCTAAC

CAM 241 TTTGAATAGTGATCTTCTAATAGCCGTCGCAGAG-AAAAAAAAAAGAATAGTGTTCTAAC

CN 113754 TTTGAATAGTGATCTTCTAATAGCCGTCGCAGAG-AAAAAAAAAAGAATAGTGTTCTAAC

CAM 236 TTTGAATAGTGATCTTCTAATAGCCGTCGCAGAGAAAAAAAAAAAGAATAGTGTTCTAAC

CO46 NCBI TTTGAATAGTGATCTTCTAATAGCCGTCGCAGAGAAAAAAAAAAAGAATAGTGTTCTAAC

CN 120013 TTTGAATAGTGATCTTCTAATAGCCGTCGCAGAG-AAAAAAAAAAGAATAGTGTTCTAAC

Blaine Creek TTTGAATAGTGATCTTCTAATAGCCGTCGCAGAG-AAAAAAAAAAGAATAGTGTTCTAAC

09-CS0040 TTTGAATAGTGATCTTCTAATAGCCGTCGCAGAG-AAAAAAAAAAGAATAGTGTTCTAAC

17CS1133 TTTGAATAGTGATCTTCTAATAGCCGTCGCAGAG-AAAAAAAAAAGAATAGTGTTCTAAC

Jasper TTTGAATAGTGATCTTCTAATAGCCGTCGCAGAG-AAAAAAAAAAGAATAGTGTTCTAAC

CN 120017 TTTGAATAGTGATCTTCTAATAGCCGTCGCAGAG-AAAAAAAAAAGAATAGTGTTCTAAC

CN 120027 TTTGAATAGTGATCTTCTAATAGCCGTCGCAGAG-AAAAAAAAAAGAATAGTGTTCTAAC

CN 120030 TTTGAATAGTGATCTTCTAATAGCCGTCGCAGAG-AAAAAAAAAAGAATAGTGTTCTAAC

Hoga TTTGAATAGTGATCTTCTAATAGCCGTCGCAGAG-AAAAAAAAAAGAATAGTGTTCTAAC

Yellowstone TTTGAATAGTGATCTTCTAATAGCCGTCGCAGAGAAAAAAAAAAAGAATAGTGTTCTAAC

CN 119294 TTTGAATAGTGATCTTCTAATAGCCGTCGCAGAG-AAAAAAAAAAGAATAGTGTTCTAAC

********************************** *************************

CN 120025 GTTTGTGACGTTTTAGTGACGACTCGACGCAAAATCGATGCTCGCTAAGGTGTTGCTATT

CN 119205 GTTTGTGACGTTTTAGTGACGACTCGACGCAAAATCGATGCTCGCTAAGGTGTTGCTATT

CN 119243 GTTTGTGACGTTTTAGTGACGACTCGACGCAAAATCGATGCTCGCTAAGGTGTTGCTATT

Joelle NCBI GTTTGTGACGTTTTAGTGACGACTCGACGCAAAATCGATGCTCGCTAAGGTGTTGCTATT

Joelle AAFC GTTTGTGACGTTTTAGTGACGACTCGACGCAAAATCGATGCTCGCTAAGGTGTTGCTATT

Joelle phyto GTTTGTGACGTTTTAGTGACGACTCGACGCAAAATCGATGCTCGCTAAGGTGTTGCTATT

DH55 ref genome GTTTGTGACGTTTTAGTGACGACTCGACGCAAAATCGATGCTCGCTAAGGTGTTGCTATT

CN 119300 GTTTGTGACGTTTTAGTGACGACTCGACGCAAAATCGATGCTCGCTAAGGTGTTGCTATT

CAM 241 GTTTGTGACGTTTTAGTGACGACTCGACGCAAAATCGATGCTCGCTAAGGTGTTGCTATT

CN 113754 GTTTGTGACGTTTTAGTGACGACTCGACGCAAAATCGATGCTCGCTAAGGTGTTGCTATT

CAM 236 GTTTGTGACGTTTTAGTGACGACTCGACGCAAAATCGATGCTCGCTAAGGTGTTGCTATT

CO46 NCBI GTTTGTGACGTTTTAGTGACGACTCGACGCAAAATCGATGCTCGCTAAGGTGTTGCTATT

CN 120013 GTTTGTGACGTTTTAGTGACGACTCGACGCAAAATCGATGCTCGCTAAGGTGTTGCTATT

Blaine Creek GTTTGTGACGTTTTAGTGACGACTCGACGCAAAATCGATGCTCGCTAAGGTGTTGCTATT

09-CS0040 GTTTGTGACGTTTTAGTGACGACTCGACGCAAAATCGATGCTCGCTAAGGTGTTGCTATT

17CS1133 GTTTGTGACGTTTTAGTGACGACTCGACGCAAAATCGATGCTCGCTAAGGTGTTGCTATT

Jasper GTTTGTGACGTTTTAGTGACGACTCGACGCAAAATCGATGCTCGCTAAGGTGTTGCTATT

CN 120017 GTTTGTGACGTTTTAGTGACGACTCGACGCAAAATCGATGCTCGCTAAGGTGTTGCTATT

CN 120027 GTTTGTGACGTTTTAGTGACGACTCGACGCAAAATCGATGCTCGCTAAGGTGTTGCTATT

CN 120030 GTTTGTGACGTTTTAGTGACGACTCGACGCAAAATCGATGCTCGCTAAGGTGTTGCTATT

Hoga GTTTGTGACGTTTTAGTGACGACTCGACGCAAAATCGATGCTCGCTAAGGTGTTGCTATT

Yellowstone GTTTGTGACGTTTTAGTGACGACTCGACGCAAAATCGATGCTCGCTAAGGTGTTGCTATT

CN 119294 GTTTGTGACGTTTTAGTGACGACTCGACGCAAAATCGATGCTCGCTAAGGTGTTGCTATT

************************************************************

CN 120025 GAAAAGTTTATTACTCCAACTTATCACATTTAAATTTAAGGAGTAGAGAATGAATAAATA

CN 119205 GAAAAGTTTATTACTCCAACTTATCACATTTAAATTTAAGGAGTAGAGAATGAATAAATA

CN 119243 GAAAAGTTTATTACTCCAACTTATCACATTTAAATTTAAGGAGTAGAGAATGAATAAATA

Joelle NCBI GAAAAGTTTATTACTCCAACTTATCACATTTAAATTTAAGGAGTAGAGAATGAATAAATA

Joelle AAFC GAAAAGTTTATTACTCCAACTTATCACATTTAAATTTAAGGAGTAGAGAATGAATAAATA

Joelle phyto GAAAAGTTTATTACTCCAACTTATCACATTTAAATTTAAGGAGTAGAGAATGAATAAATA

DH55 ref genome GAAAAGTTTATTACTCCAACTTATCACATTTAAATTTAAGGAGTAGAGAATGAATAAATA

CN 119300 GAAAAGTTTATTACTCCAACTTATCACATTTAAATTTAAGGAGTAGAGAATGAATAAATA

CAM 241 GAAAAGTTTATTACTCCAACTTATCACATTTAAATTTAAGGAGTAGAGAATGAATAAATA

CN 113754 GAAAAGTTTATTACTCCAACTTATCACATTTAAATTTAAGGAGTAGAGAATGAATAAATA

CAM 236 GAAAAGTTTATTACTCCAACTTATCACATTTAAATTTAAGGAGTAGAGAATGAATAAATA

CO46 NCBI GAAAAGTTTATTACTCCAACTTATCACATTTAAATTTAAGGAGTAGAGAATGAATAAATA

CN 120013 GAAAAGTTTATTACTCCAACTTATCACATTTAAATTTAAGGAGTAGAGAATGAATAAATA

Blaine Creek GAAAAGTTTATTACTCCAACTTATCACATTTAAATTTAAGGAGTAGAGAATGAATAAATA

09-CS0040 GAAAAGTTTATTACTCCAACTTATCACATTTAAATTTAAGGAGTAGAGAATGAATAAATA

17CS1133 GAAAAGTTTATTACTCCAACTTATCACATTTAAATTTAAGGAGTAGAGAATGAATAAATA

Jasper GAAAAGTTTATTACTCCAACTTATCACATTTAAATTTAAGGAGTAGAGAATGAATAAATA

CN 120017 GAAAAGTTTATTACTCCAACTTATCACATTTAAATTTAAGGAGTAGAGAATGAATAAATA

CN 120027 GAAAAGTTTATTACTCCAACTTATCACATTTAAATTTAAGGAGTAGAGAATGAATAAATA

CN 120030 GAAAAGTTTATTACTCCAACTTATCACATTTAAATTTAAGGAGTAGAGAATGAATAAATA

Hoga GAAAAGTTTATTACTCCAACTTATCACATTTAAATTTAAGGAGTAGAGAATGAATAAATA

Yellowstone GAAAAGTTTATTACTCCAACTTATCACATTTAAATTTAAGGAGTAGAGAATGAATAAATA

CN 119294 GAAAAGTTTATTACTCCAACTTATCACATTTAAATTTAAGGAGTAGAGAATGAATAAATA

************************************************************

CN 120025 GCTTTGGTATTGACTTATTTTATGTAGGATGATGATATTTATGAATTTGTCTAAGATATT

CN 119205 GCTTTGGTATTGACTTATTTTATGTAGGATGATGATATTTATGAATTTGTCTAAGATATT

CN 119243 GCTTTGGTATTGACTTATTTTATGTAGGATGATGATATTTATGAATTTGTCTAAGATATT

Joelle NCBI GCTTTGGTATTGACTTATTTTATGTAGGATGATGATATTTATGAATTTGTCTAAGATATT

Joelle AAFC GCTTTGGTATTGACTTATTTTATGTAGGATGATGATATTTATGAATTTGTCTAAGATATT

Joelle phyto GCTTTGGTATTGACTTATTTTATGTAGGATGATGATATTTATGAATTTGTCTAAGATATT

DH55 ref genome GCTTTGGTATTGACTTATTTTATGTAGGATGATGATATTTATGAATTTGTCTAAGATATT

CN 119300 GCTTTGGTATTGACTTATTTTATGTAGGATGATGATATTTATGAATTTGTCTAAGATATT

CAM 241 GCTTTGGTATTGACTTATTTTATGTAGGATGATGATATTTATGAATTTGTCTAAGATATT

CN 113754 GCTTTGGTATTGACTTATTTTATGTAGGATGATGATATTTATGAATTTGTCTAAGATATT

CAM 236 GCTTTGGTATTGACTTATTTTATGTAGGATGATGATATTTATGAATTTGTCTAAGATATT

CO46 NCBI GCTTTGGTATTGACTTATTTTATGTAGGATGATGATATTTATGAATTTGTCTAAGATATT

CN 120013 GCTTTGGTATTGACTTATTTTATGTAGGATGATGATATTTATGAATTTGTCTAAGATATT

Blaine Creek GCTTTGGTATTGACTTATTTTATGTAGGATGATGATATTTATGAATTTGTCTAAGATATT

09-CS0040 GCTTTGGTATTGACTTATTTTATGTAGGATGATGATATTTATGAATTTGTCTAAGATATT

17CS1133 GCTTTGGTATTGACTTATTTTATGTAGGATGATGATATTTATGAATTTGTCTAAGATATT

Jasper GCTTTGGTATTGACTTATTTTATGTAGGATGATGATATTTATGAATTTGTCTAAGATATT

CN 120017 GCTTTGGTATTGACTTATTTTATGTAGGATGATGATATTTATGAATTTGTCTAAGATATT

CN 120027 GCTTTGGTATTGACTTATTTTATGTAGGATGATGATATTTATGAATTTGTCTAAGATATT

CN 120030 GCTTTGGTATTGACTTATTTTATGTAGGATGATGATATTTATGAATTTGTCTAAGATATT

Hoga GCTTTGGTATTGACTTATTTTATGTAGGATGATGATATTTATGAATTTGTCTAAGATATT

Yellowstone GCTTTGGTATTGACTTATTTTATGTAGGATGATGATATTTATGAATTTGTCTAAGATATT

CN 119294 GCTTTGGTATTGACTTATTTTATGTAGGATGATGATATTTATGAATTTGTCTAAGATATT

************************************************************

CN 120025 TTTTGGGTTCAC--ATATATATATAACATGTAGATAAATACAAGTACATTTAATATATAT

CN 119205 TTTTGGGTTCACATATATATATATAACATGTAGATAAATACAAGTACATTTAATATATAT

CN 119243 TTTTGGGTTCACATATATATATATAACATGTAGATAAATACAAGTACATTTAATATATAT

Joelle NCBI TTTTGGGTTCACATATATATATATAACATGTAGATAAATACAAGTACATTTAATATATAT

Joelle AAFC TTTTGGGTTCACATATATATATATAACATGTAGATAAATACAAGTACATTTAATATATAT

Joelle phyto TTTTGGGTTCACATATATATATATAACATGTAGATAAATACAAGTACATTTAATATATAT

DH55 ref genome TTTTGGGTTCACATATATATATATAACATGTAGATAAATACAAGTACATTTAATATATAT

CN 119300 TTTTGGGTTCACATATATATATATAACATGTAGATAAATACAAGTACATTTAATATATAT

CAM 241 TTTTGGGTTCACATATATATATATAACATGTAGATAAATACAAGTACATTTAATATATAT

CN 113754 TTTTGGGTTCACATATATATATATAACATGTAGATAAATACAAGTACATTTAATATATAT

CAM 236 TTTTGGGTTCACATATATATATATAACATGTAGATAAATACAAGTACATTTAATATATAT

CO46 NCBI TTTTGGGTTCACATATATATATATAACATGTAGATAAATACAAGTACATTTAATATATAT

CN 120013 TTTTGGGTTCACATATATATATATAACATGTAGATAAATACAAGTACATTTAATATATAT

Blaine Creek TTTTGGGTTCACATATATATATATAACATGTAGATAAATACAAGTACATTTAATATATAT

09-CS0040 TTTTGGGTTCACATATATATATATAACATGTAGATAAATACAAGTACATTTAATATATAT

17CS1133 TTTTGGGTTCACATATATATATATAACATGTAGATAAATACAAGTACATTTAATATATAT

Jasper TTTTGGGTTCACATATATATATATAACATGTAGATAAATACAAGTACATTTAATATATAT

CN 120017 TTTTGGGTTCACATATATATATATAACATGTAGATAAATACAAGTACATTTAATATATAT

CN 120027 TTTTGGGTTCACATATATATATATAACATGTAGATAAATACAAGTACATTTAATATATAT

CN 120030 TTTTGGGTTCACATATATATATATAACATGTAGATAAATACAAGTACATTTAATATATAT

Hoga TTTTGGGTTCACATATATATATATAACATGTAGATAAATACAAGTACATTTAATATATAT

Yellowstone TTTTGGGTTCACATATATATATATAACATGTAGATAAATACAAGTACATTTAATATATAT

CN 119294 TTTTGGGTTCACATATATATATATAACATGTAGATAAATACAAGTACATTTAATATATAT

************ **********************************************

CN 120025 TTCTAATCATGTTTTAACCC-AAAAAAAGAACTATTTCACTTCCTTAATCGATTATATAA

CN 119205 TTCTAATCATGTTTTAACCCAAAAAAAAGAACTATTTCACTTCATTAATCGATTATATAA

CN 119243 TTCTAATCATGTTTTAACCCAAAAAAAAGAACTATTTCACTTCATTAATCGATTATATAA

Joelle NCBI TTCTAATCATGTTTTAACCCAAAAAAAAGAACTATTTCACTTCATTAATCGATTATATAA

Joelle AAFC TTCTAATCATGTTTTAACCCAAAAAAAAGAACTATTTCACTTCATTAATCGATTATATAA

Joelle phyto TTCTAATCATGTTTTAACCCAAAAAAAAGAACTATTTCACTTCATTAATCGATTATATAA

DH55 ref genome TTCTAATCATGTTTTAACCCAAAAAAAAGAACTATTTCACTTCATTAATCGATTATATAA

CN 119300 TTCTAATCATGTTTTAACCCAAAAAAAAGAGCTATTTCACTTCATTAATCGATTATATAA

CAM 241 TTCTAATCATGTTTTAACCCAAAAAAAAGAGCTATTTCACTTCATTAATCGATTATATAA

CN 113754 TTCTAATCATGTTTTAACCCAAAAAAAAGAACTATTTCACTTCATTAATCGATTATATAA

CAM 236 TTCTAATCATGTTTTAACCCAAAAAAAAGAACTATTTCACTTCATTAATCGATTATATAA

CO46 NCBI TTCTAATCATGTTTTAACCCAAAAAAAAGAACTATTTCACTTCATTAATCGATTATATAA

CN 120013 TTCTAATCATGTTTTAACCCAAAAAAAAGAACTATTTCACTTCATTAATCGATTATATAA

Blaine Creek TTCTAATCATGTTTTAACCCAAAAAAAAGAACTATTTCACTTCATTAATCGATTATATAA

09-CS0040 TTCTAATCATGTTTTAACCCAAAAAAAAGAACTATTTCACTTCATTAATCGATTATATAA

17CS1133 TTCTAATCATGTTTTAACCCAAAAAAAAGAACTATTTCACTTCATTAATCGATTATATAA

Jasper TTCTAATCATGTTTTAACCCAAAAAAAAGAACTATTTCACTTCATTAATCGATTATATAA

CN 120017 TTCTAATCATGTTTTAACCCAAAAAAAAGAACTATTTCACTTCATTAATCGATTATATAA

CN 120027 TTCTAATCATGTTTTAACCCAAAAAAAAGAACTATTTCACTTCATTAATCGATTATATAA

CN 120030 TTCTAATCATGTTTTAACCCAAAAAAAAGAACTATTTCACTTCATTAATCGATTATATAA

Hoga TTCTAATCATGTTTTAACCCAAAAAAAAGAACTATTTCACTTCATTAATCGATTATATAA

Yellowstone TTCTAATCATGTTTTAACCCAAAAAAAAGAACTATTTCACTTCATTAATCGATTATATAA

CN 119294 TTCTAATCATGTTTTAACCCAAAAAAAAGAACTATTTCACTTCATTAATCGATTATATAA

******************** ********* ************ ****************

CN 120025 ACAAGTACTTATAAATACTAATACTAGTTTTATTCTACATCTTCATAACAATTACCAATA

CN 119205 ACAAGTACTTATAAATACTAATACTAGTTTTATTCTACATCTTCATAACAATTACCAATA

CN 119243 ACAAGTACTTATAAATACTAATACTAGTTTTATTCTACATCTTCATAACAATTACCAATA

Joelle NCBI ACAAGTACTTATAAATACTAATACTAGTTTTATTCTACATCTTCATAACAATTACCAATA

Joelle AAFC ACAAGTACTTATAAATACTAATACTAGTTTTATTCTACATCTTCATAACAATTACCAATA

Joelle phyto ACAAGTACTTATAAATACTAATACTAGTTTTATTCTACATCTTCATAACAATTACCAATA

DH55 ref genome ACAAGTACTTATAAATACTAATACTAGTTTTATTCTACATCTTCATAACAATTACCAATA

CN 119300 ACAAGTACTTATAAATACTAATACTAGTTTTATTCTACATCTTCATAACAATTACCAATA

CAM 241 ACAAGTACTTATAAATACTAATACTAGTTTTATTCTACATCTTCATAACAATTACCAATA

CN 113754 ACAAGTACTTATAAATACTAATACTAGTTTTATTCTACATCTTCATAACAATTACCAATA

CAM 236 ACAAGTACTTATAAATACTAATACTAGTTTTATTCTACATCTTCATAACAATTACCAATA

CO46 NCBI ACAAGTACTTATAAATACTAATACTAGTTTTATTCTACATCTTCATAACAATTACCAATA

CN 120013 ACAAGTACTTATAAATACTAATACTAGTTTTATTCTACATCTTCATAACAATTACCAATA

Blaine Creek ACAAGTACTTATAAATACTAATACTAGTTTTATTCTACATCTTCATAACAATTACCAATA

09-CS0040 ACAAGTACTTATAAATACTAATACTAGTTTTATTCTACATCTTCATAACAATTACCAATA

17CS1133 ACAAGTACTTATAAATACTAATACTAGTTTTATTCTACATCTTCATAACAATTACCAATA

Jasper ACAAGTACTTATAAATACTAATACTAGTTTTATTCTACATCTTCATAACAATTACCAATA

CN 120017 ACAAGTACTTATAAATACTAATACTAGTTTTATTCTACATCTTCATAACAATTACCAATA

CN 120027 ACAAGTACTTATAAATACTAATACTAGTTTTATTCTACATCTTCATAACAATTACCAATA

CN 120030 ACAAGTACTTATAAATACTAATACTAGTTTTATTCTACATCTTCATAACAATTACCAATA

Hoga ACAAGTACTTATAAATACTAATACTAGTTTTATTCTACATCTTCATAACAATTACCAATA

Yellowstone ACAAGTACTTATAAATACTAATACTAGTTTTATTCTACATCTTCATAACAATTACCAATA

CN 119294 ACAAGTACTTATAAATACTAATACTAGTTTTATTCTACATCTTCATAACAATTACCAATA

************************************************************

CN 120025 TTTATATATAGAATATCAAAAATGATGTATTTGGAATTGTAATTTACGCATTGTAACGCT

CN 119205 TTTATATATAGAATATCAAAAATGATGTATTTGGAATTGTAATTTACGCATTGTAACGCT

CN 119243 TTTATATATAGAATATCAAAAATGATGTATTTGGAATTGTAATTTACGCATTGTAACGCT

Joelle NCBI TTTATATATAGAATATCAAAAATGATGTATTTGGAATTGTAATTTACGCATTGTAACGCT

Joelle AAFC TTTATATATAGAATATCAAAAATGATGTATTTGGAATTGTAATTTACGCATTGTAACGCT

Joelle phyto TTTATATATAGAATATCAAAAATGATGTATTTGGAATTGTAATTTACGCATTGTAACGCT

DH55 ref genome TTTATATATAGAATATCAAAAATGATGTATTTGGAATTGTAATTTACGCATTGTAACGCT

CN 119300 TTTATATATAGAATATCAAAAATGATGTATTTGGAATTGTAATTTACGCATTGTAACGCT

CAM 241 TTTATATATAGAATATCAAAAATGATGTATTTGGAATTGTAATTTACGCATTGTAACGCT

CN 113754 TTTATATATAGAATATCAAAAATGATGTATTTGGAATTGTAATTTACGCATTGTAACGCT

CAM 236 TTTATATATAGAATATCAAAAATGATGTATTTGGAATTGTAATTTACGCATTGTAACGCT

CO46 NCBI TTTATATATAGAATATCAAAAATGATGTATTTGGAATTGTAATTTACGCATTGTAACGCT

CN 120013 TTTATATATAGAATATCAAAAATGATGTATTTGGAATTGTAATTTACGCATTGTAACGCT

Blaine Creek TTTATATATAGAATATCAAAAATGATGTATTTGGAATTGTAATTTACGCATTGTAACGCT

09-CS0040 TTTATATATAGAATATCAAAAATGATGTATTTGGAATTGTAATTTACGCATTGTAACGCT

17CS1133 TTTATATATAGAATATCAAAAATGATGTATTTGGAATTGTAATTTACGCATTGTAACGCT

Jasper TTTATATATAGAATATCAAAAATGATGTATTTGGAATTGTAATTTACGCATTGTAACGCT

CN 120017 TTTATATATAGAATATCAAAAATGATGTATTTGGAATTGTAATTTACGCATTGTAACGCT

CN 120027 TTTATATATAGAATATCAAAAATGATGTATTTGGAATTGTAATTTACGCATTGTAACGCT

CN 120030 TTTATATATAGAATATCAAAAATGATGTATTTGGAATTGTAATTTACGCATTGTAACGCT

Hoga TTTATATATAGAATATCAAAAATGATGTATTTGGAATTGTAATTTACGCATTGTAACGCT

Yellowstone TTTATATATAGAATATCAAAAATGATGTATTTGGAATTGTAATTTACGCATTGTAACGCT

CN 119294 TTTATATATAGAATATCAAAAATGATGTATTTGGAATTGTAATTTACGCATTGTAACGCT

************************************************************
