# Supplementary material for: All roads lead to Rome: QTL analysis for vernalization requirement and dissection of allelic variation uncovered unexpected diversity of FLC loci in Camelina sativa
Source: Front Plant Sci. 2025 Jul 25;16:1639872. doi: 10.3389/fpls.2025.1639872 (PMC12331742; doi:10.3389/fpls.2025.1639872)
Supplement: Supplementary Table 5 — Sequence alignment of the FLC.C13 gene. Three C. microcarpa (CN 120025, CN 119205 and CN 119243); ‘CO46’ (GCA_036971115.1) and ‘Joelle’ (GCA_036769185.1) reference sequences from NCBI; ‘Joelle’ sequences from the AAFC and the DOE-JGI phytozome reference genomes; the DH55 reference genome sequence and 15 spring type C. sativa lines were aligned using the EMBL-EBI online tool MUSCLE. All winter Camelina lines are in blue font. Exons are shown in purple font and the SNP utilized for marker development is highlighted in blue. SNPs which distinguish the winter and spring alleles are highlighted in green. [file Table5.docx]

**Supplementary File 5.** Sequence alignment of the FLC.C13 gene. Three *C. microcarpa* (CN 120025, CN 119205 and CN 119243); ‘CO46’ (GCA_036971115.1) and ‘Joelle’ (GCA_036769185.1) reference sequences from NCBI; ‘Joelle’ sequences from the AAFC and the DOE-JGI phytozome reference genomes; the DH55 reference genome sequence and 15 spring type *C. sativa* lines were aligned using the EMBL-EBI online tool MUSCLE. All winter Camelina lines are in blue font. Exons are shown in purple font and the SNP utilized for marker development is highlighted in blue. SNPs which distinguish the winter and spring alleles are highlighted in green.

CAM 241 TAGAGTGCTGATATTATCTTGTCGTATTCTGAGATTGAAATTAAAACTTTAAAACATAGA

17CS1133 TAGAGTGCTGATATTATCTTGTCGTATTCTGAGATTGAAATTAAAACTTTAAAACATAGA

CAM 236 TAGAGTGCTGATATTATCTTGTCGTATTCTGAGATTGAAATTAAAACTTTAAAACATAGA

Blaine Creek TAGAGTGCTGATATTATCTTGTCGTATTCTGAGATTGAAATTAAAACTTTAAAACATAGA

CN 119300 TAGAGTGCTGATATTATCTTGTCGTATTCTGAGATTGAAATTAAAACTTTAAAACATAGA

Yellowstone TAGAGTGCTGATATTATCTTGTCGTATTCTGAGATTGAAATTAAAACTTTAAAACATAGA

Hoga TAGAGTGCTGATATTATCTTGTCGTATTCTGAGATTGAAATTAAAACTTTAAAACATAGA

CN 120027 TAGAGTGCTGATATTATCTTGTCGTATTCTGAGATTGAAATTAAAACTTTAAAACATAGA

CN 120030 TAGAGTGCTGATATTATCTTGTCGTATTCTGAGATTGAAATTAAAACTTTAAAACATAGA

CN 119294 TAGAGTGCTGATATTATCTTGTCGTATTCTGAGATTGAAATTAAAACTTTAAAACATAGA

CN 120013 TAGAGTGCTGATATTATCTTGTCGTATTCTGAGATTGAAATTAAAACTTTAAAACATAGA

CN 120017 TAGAGTGCTGATATTATCTTGTCGTATTCTGAGATTGAAATTAAAACTTTAAAACATAGA

CN 119205 TAGAGTGCTGATATTATCTTGTCGTATTCTGAGATTGAAATTAAAACTTTAAAACATAGA

DH55 ref genome TAGAGTGCTGATATTATCTTGTCGTATTCTGAGATTGAAATTAAAACTTTAAAACATAGA

09-CS0040 TAGAGTGCTGATATTATCTTGTCGTATTCTGAGATTGAAATTAAAACTTTAAAACATAGA

CN 113754 TAGAGTGCTGATATTATCTTGTCGTATTCTGAGATTGAAATTAAAACTTTAAAACATAGA

CO46 NCBI TAGAGTGCTGATATTATCTTGTCGTATTCTGAGATTGAAATTAAAACTTTAAAACATAGA

Jasper TAGAGTGCTGATATTATCTTGTCGTATTCTGAGATTGAAATTAAAACTTTAAAACATAGA

Joelle phyto TAGAGTGCTGATATTATCTTGTCGTATTCTGAGATTGAAATTAAAACTTTAAAACATAGA

Joelle NCBI TAGAGTGCTGATATTATCTTGTCGTATTCTGAGATTGAAATTAAAACTTTAAAACATAGA

CN 119243 TAGAGTGCTGATATTATCTTGTCGTATTCTGAGATTGAAATTAAAACTTTAAAACATAGA

CN 120025 TAGAGTGCTGATATTATCTTGTCGTATTCTGAGATTGAAATTAAAACTTTAAAACATAGA

Joelle AAFC TAGAGTGCTGATATTATCTTGTCGTATTCTGAGATTGAAATTAAAACTTTAAAACATAGA

************************************************************

CAM 241 GATGCCTTAAATAAAAATATATATATAAAAAAAAGAAAGATAAAATGACATATCCAGAAT

17CS1133 GATGCCTTAAATAAAAATATATATATAAAAAAAAGAAAGATAAAATGACATATCCAGAAT

CAM 236 GATGCCTTAAATAAAAATATATATATAAAAAAAAGAAAGATAAAATGACATATCCAGAAT

Blaine Creek GATGCCTTAAATAAAAATATATATATAAAAAAAAGAAAGATAAAATGACATATCCAGAAT

CN 119300 GATGCCTTAAATAAAAATATATATATAAAAAAAAGAAAGATAAAATGACATATCCAGAAT

Yellowstone GATGCCTTAAATAAAAATATATATATAAAAAAAAGAAAGATAAAATGACATATCCAGAAT

Hoga GATGCCTTAAATAAAAATATATATATAAAAAAAAGAAAGATAAAATGACATATCCAGAAT

CN 120027 GATGCCTTAAATAAAAATATATATATAAAAAAAAGAAAGATAAAATGACATATCCAGAAT

CN 120030 GATGCCTTAAATAAAAATATATATATAAAAAAAAGAAAGATAAAATGACATATCCAGAAT

CN 119294 GATGCCTTAAATAAAAATATATATATAAAAAAAAGAAAGATAAAATGACATATCCAGAAT

CN 120013 GATGCCTTAAATAAAAATATATATATAAAAAAAAGAAAGATAAAATGACATATCCAGAAT

CN 120017 GATGCCTTAAATAAAAATATATATATAAAAAAAAGAAAGATAAAATGACATATCCAGAAT

CN 119205 GATGCCTTAAATAAAAATATATATATAAAAAAAAGAAAGATAAAATGACATATCCAGAAT

DH55 ref genome GATGCCTTAAATAAAAATATATATATAAAAAAAAGAAAGATAAAATGACATATCCAGAAT

09-CS0040 GATGCCTTAAATAAAAATATATATATAAAAAAAAGAAAGATAAAATGACATATCCAGAAT

CN 113754 GATGCCTTAAATAAAAATATATATATAAAAAAAAGAAAGATAAAATGACATATCCAGAAT

CO46 NCBI GATGCCTTAAATAAAAATATATATATAAAAAAAAGAAAGATAAAATGACATATCCAGAAT

Jasper GATGCCTTAAATAAAAATATATATATAAAAAAAAGAAAGATAAAATGACATATCCAGAAT

Joelle phyto GATGCCTTAAATAAAAATATATATATAAAAAAAAGAAAGATAAAATGACATATCCAGAAT

Joelle NCBI GATGCCTTAAATAAAAATATATATATAAAAAAAAGAAAGATAAAATGACATATCCAGAAT

CN 119243 GATGCCTTAAATAAAAATATATATATATAAAAAAGAAAGATAAAATGACATATCCAGAAT

CN 120025 GATGCCTTAAATAAAAATATATATATATAAAAAAGAAAGATAAAATGACATATCCAGAAT

Joelle AAFC GATGCCTTAAATAAAAATATATATATATAAAAAAGAAAGATAAAATGACATATCCAGAAT

*************************** ********************************

CAM 241 AGAAAAAGGAGGTGGGATGATTGGATATAAACGATGCCGT------ACCCATCGATTCGT

17CS1133 AGAAAAAGGAGGTGGGATGATTGGATATAAACGATGCCGT------ACCCATCGATTCGT

CAM 236 AGAAAAAGGAGGTGGGATGATTGGATATAAACGATGCCGT------ACCCATCGATTCGT

Blaine Creek AGAAAAAGGAGGTGGGATGATTGGATATAAACGATGCCGT------ACCCATCGATTCGT

CN 119300 AGAAAAAGGAGGTGGGATGATTGGATATAAACGATGCCGT------ACCCATCGATTCGT

Yellowstone AGAAAAAGGAGGTGGGATGATTGGATATAAACGATGCCGT------ACCCATCGATTCGT

Hoga AGAAAAAGGAGGTGGGATGATTGGATATAAACGATGCCGT------ACCCATCGATTCGT

CN 120027 AGAAAAAGGAGGTGGGATGATTGGATATAAACGATGCCGT------ACCCATCGATTCGT

CN 120030 AGAAAAAGGAGGTGGGATGATTGGATATAAACGATGCCGT------ACCCATCGATTCGT

CN 119294 AGAAAAAGGAGGTGGGATGATTGGATATAAACGATGCCGT------ACCCATCGATTCGT

CN 120013 AGAAAAAGGAGGTGGGATGATTGGATATAAACGATGCCGT------ACCCATCGATTCGT

CN 120017 AGAAAAAGGAGGTGGGATGATTGGATATAAACGATGCCGT------ACCCATCGATTCGT

CN 119205 AGAAAAAGGAGGTGGGATGATTGGATATAAACGATGCCGT------ACCCATCGATTCGT

DH55 ref genome AGAAAAAGGAGGTGGGATGATTGGATATAAACGATGCCGT------ACCCATCGATTCGT

09-CS0040 AGAAAAAGGAGGTGGGATGATTGGATATAAACGATGCCGT------ACCCATCGATTCGT

CN 113754 AGAAAAAGGAGGTGGGATGATTGGATATAAACGATGCCGT------ACCCATCGATTCGT

CO46 NCBI AGAAAAAGGAGGTGGGATGATTGGATATAAACGATGCCGT------ACCCATCGATTCGT

Jasper AGAAAAAGGAGGTGGGATGATTGGATATAAACGATGCCGT------ACCCATCGATTCGT

Joelle phyto AGAAAAAGGAGGTGGGATGATTGGATATAAACGATGCCGT------ACCCATCGATTCGT

Joelle NCBI AGAAAAAGGAGGTGGGATGATTGGATATAAACGATGCCGT------ACCCATCGATTCGT

CN 119243 AGAAAAAGGAGGTGGGATGATTGGATATAAACGATGCCGTACATTCACCCATCGATTCGT

CN 120025 AGAAAAAGGAGGTGGGATGATTGGATATAAACGATGCCGTACATTCACCCATCGATTCGT

Joelle AAFC AGAAAAAGGAGGTGGGATGATTGGATATAAACGATGCCGTACATTCACCCATCGATTCGT

**************************************** **************

CAM 241 AGAAGTGCTTTTTCGCATTAAGAGAGATATTGTATTTTCTCTGCCACAATTTTGCTTGTA

17CS1133 AGAAGTGCTTTTTCGCATTAAGAGAGATATTGTATTTTCTCTGCCACAATTTTGCTTGTA

CAM 236 AGAAGTGCTTTTTCGCATTAAGAGAGATATTGTATTTTCTCTGCCACAATTTTGCTTGTA

Blaine Creek AGAAGTGCTTTTTCGCATTAAGAGAGATATTGTATTTTCTCTGCCACAATTTTGCTTGTA

CN 119300 AGAAGTGCTTTTTCGCATTAAGAGAGATATTGTATTTTCTCTGCCACAATTTTGCTTGTA

Yellowstone AGAAGTGCTTTTTCGCATTAAGAGAGATATTGTATTTTCTCTGCCACAATTTTGCTTGTA

Hoga AGAAGTGCTTTTTCGCATTAAGAGAGATATTGTATTTTCTCTGCCACAATTTTGCTTGTA

CN 120027 AGAAGTGCTTTTTCGCATTAAGAGAGATATTGTATTTTCTCTGCCACAATTTTGCTTGTA

CN 120030 AGAAGTGCTTTTTCGCATTAAGAGAGATATTGTATTTTCTCTGCCACAATTTTGCTTGTA

CN 119294 AGAAGTGCTTTTTCGCATTAAGAGAGATATTGTATTTTCTCTGCCACAATTTTGCTTGTA

CN 120013 AGAAGTGCTTTTTCGCATTAAGAGAGATATTGTATTTTCTCTGCCACAATTTTGCTTGTA

CN 120017 AGAAGTGCTTTTTCGCATTAAGAGAGATATTGTATTTTCTCTGCCACAATTTTGCTTGTA

CN 119205 AGAAGTGCTTTTTCGCATTAAGAGAGATATTGTATTTTCTCTGCCACAATTTTGCTTGTA

DH55 ref genome AGAAGTGCTTTTTCGCATTAAGAGAGATATTGTATTTTCTCTGCCACAATTTTGCTTGTA

09-CS0040 AGAAGTGCTTTTTCGCATTAAGAGAGATATTGTATTTTCTCTGCCACAATTTTGCTTGTA

CN 113754 AGAAGTGCTTTTTCGCATTAAGAGAGATATTGTATTTTCTCTGCCACAATTTTGCTTGTA

CO46 NCBI AGAAGTGCTTTTTCGCATTAAGAGAGATATTGTATTTTCTCTGCCACAATTTTGCTTGTA

Jasper AGAAGTGCTTTTTCGCATTAAGAGAGATATTGTATTTTCTCTGCCACAATTTTGCTTGTA

Joelle phyto AGAAGTGCTTTTTCGCATTAAGAGAGATATTGTATTTTCTCTGCCACAATTTTGCTTGTA

Joelle NCBI AGAAGTGCTTTTTCGCATTAAGAGAGATATTGTATTTTCTCTGCCACAATTTTGCTTGTA

CN 119243 AGAAGTGCTTTTTCGCATTAAGAGAGATATTGTATTTTCTCTGCCACAATTTTGCTTGTA

CN 120025 AGAAGTGCTTTTTCGCATTAAGAGAGATATTGTATTTTCTCTGCCACAATTTTGCTTGTA

Joelle AAFC AGAAGTGCTTTTTCGCATTAAGAGAGATATTGTATTTTCTCTGCCACAATTTTGCTTGTA

************************************************************

CAM 241 GTAAGGTTTTATATTGACAAATCATAGGCTAAGGAAGGTTCCATAGGGTTACCAAACGTT

17CS1133 GTAAGGTTTTATATTGACAAATCATAGGCTAAGGAAGGTTCCATAGGGTTACCAAACGTT

CAM 236 GTAAGGTTTTATATTGACAAATCATAGGCTAAGGAAGGTTCCATAGGGTTACCAAACGTT

Blaine Creek GTAAGGTTTTATATTGACAAATCATAGGCTAAGGAAGGTTCCATAGGGTTACCAAACGTT

CN 119300 GTAAGGTTTTATATTGACAAATCATAGGCTAAGGAAGGTTCCATAGGGTTACCAAACGTT

Yellowstone GTAAGGTTTTATATTGACAAATCATAGGCTAAGGAAGGTTCCATAGGGTTACCAAACGTT

Hoga GTAAGGTTTTATATTGACAAATCATAGGCTAAGGAAGGTTCCATAGGGTTACCAAACGTT

CN 120027 GTAAGGTTTTATATTGACAAATCATAGGCTAAGGAAGGTTCCATAGGGTTACCAAACGTT

CN 120030 GTAAGGTTTTATATTGACAAATCATAGGCTAAGGAAGGTTCCATAGGGTTACCAAACGTT

CN 119294 GTAAGGTTTTATATTGACAAATCATAGGCTAAGGAAGGTTCCATAGGGTTACCAAACGTT

CN 120013 GTAAGGTTTTATATTGACAAATCATAGGCTAAGGAAGGTTCCATAGGGTTACCAAACGTT

CN 120017 GTAAGGTTTTATATTGACAAATCATAGGCTAAGGAAGGTTCCATAGGGTTACCAAACGTT

CN 119205 GTAAGGTTTTATATTGACAAATCATAGGCTAAGGAAGGTTCCATAGGGTTACCAAACGTT

DH55 ref genome GTAAGGTTTTATATTGACAAATCATAGGCTAAGGAAGGTTCCATAGGGTTACCAAACGTT

09-CS0040 GTAAGGTTTTATATTGACAAATCATAGGCTAAGGAAGGTTCCATAGGGTTACCAAACGTT

CN 113754 GTAAGGTTTTATATTGACAAATCATAGGCTAAGGAAGGTTCCATAGGGTTACCAAACGTT

CO46 NCBI GTAAGGTTTTATATTGACAAATCATAGGCTAAGGAAGGTTCCATAGGGTTACCAAACGTT

Jasper GTAAGGTTTTATATTGACAAATCATAGGCTAAGGAAGGTTCCATAGGGTTACCAAACGTT

Joelle phyto GTAAGGTTTTATATTGACAAATCATAGGCTAAGGAAGGTTCCATAGGGTTACCAAACGTT

Joelle NCBI GTAAGGTTTTATATTGACAAATCATAGGCTAAGGAAGGTTCCATAGGGTTACCAAACGTT

CN 119243 GTAAGGTTTTATATTGACAAATCATAGGCTAAGGAAGGTTCCATAGGGTTACCAAACGTT

CN 120025 GTAAGGTTTTATATTGACAAATCATAGGCTAAGGAAGGTTCCATAGGGTTACCAAACGTT

Joelle AAFC GTAAGGTTTTATATTGACAAATCATAGGCTAAGGAAGGTTCCATAGGGTTACCAAACGTT

************************************************************

CAM 241 TCGC-TTTTTTTTCTTTTCTCTTTTCTACCAACATTTTTCGATTATATTTCTATAATTAA

17CS1133 TCGC-TTTTTTTTCTTTTCTCTTTTCTACCAACATTTTTCGATTATATTTCTATAATTAA

CAM 236 TCGC-TTTTTTTTCTTTTCTCTTTTCTACCAACATTTTTCGATTATATTTCTATAATTAA

Blaine Creek TCGC-TTTTTTTTCTTTTCTCTTTTCTACCAACATTTTTCGATTATATTTCTATAATTAA

CN 119300 TCGC-TTTTTTTTCTTTTCTCTTTTCTACCAACATTTTTCGATTATATTTCTATAATTAA

Yellowstone TCGC-TTTTTTTTCTTTTCTCTTTTCTACCAACATTTTTCGATTATATTTCTATAATTAA

Hoga TCGC-TTTTTTTTCTTTTCTCTTTTCTACCAACATTTTTCGATTATATTTCTATAATTAA

CN 120027 TCGC-TTTTTTTTCTTTTCTCTTTTCTACCAACATTTTTCGATTATATTTCTATAATTAA

CN 120030 TCGC-TTTTTTTTCTTTTCTCTTTTCTACCAACATTTTTCGATTATATTTCTATAATTAA

CN 119294 TCGC-TTTTTTTTCTTTTCTCTTTTCTACCAACATTTTTCGATTATATTTCTATAATTAA

CN 120013 TCGC-TTTTTTTTCTTTTCTCTTTTCTACCAACATTTTTCGATTATATTTCTATAATTAA

CN 120017 TCGC-TTTTTTTTCTTTTCTCTTTTCTACCAACATTTTTCGATTATATTTCTATAATTAA

CN 119205 TCGC-TTTTTTTTCTTTTCTCTTTTCTACCAACATTTTTCGATTATATTTCTATAATTAA

DH55 ref genome TCGC-TTTTTTTTCTTTTCTCTTTTCTACCAACATTTTTCGATTATATTTCTATAATTAA

09-CS0040 TCGC-TTTTTTTTCTTTTCTCTTTTCTACCAACATTTTTCGATTATATTTCTATAATTAA

CN 113754 TCGC-TTTTTTTTCTTTTCTCTTTTCTACCAACATTTTTCGATTATATTTCTATAATTAA

CO46 NCBI TCGC-TTTTTTTTCTTTTCTCTTTTCTACCAACATTTTTCGATTATATTTCTATAATTAA

Jasper TCGC-TTTTTTTTCTTTTCTCTTTTCTACCAACATTTTTCGATTATATTTCTATAATTAA

Joelle phyto TCGC-TTTTTTTTCTTTTCTCTTTTCTACCAACATTTTTCGATTATATTTCTATAATTAA

Joelle NCBI TCGC-TTTTTTTTCTTTTCTCTTTTCTACCAACATTTTTCGATTATATTTCTATAATTAA

CN 119243 TCGCTTTTTTTTTCTTTTCTCTTTTCTACCAACATTTTTCGATTAAATTTCTATAATTAA

CN 120025 TCGCTTTTTTTTTCTTTTCTCTTTTCTACCAACATTTTTCGATTAAATTTCTATAATTAA

Joelle AAFC TCGCTTTTTTTTTCTTTTCTCTTTTCTACCAACATTTTTCGATTAAATTTCTATAATTAA

**** **************************************** **************

CAM 241 AGTCTCAGCTTATTTTGATTAATTTGAATTTAATATAATTTCAGTAAAACATCTCAACAA

17CS1133 AGTCTCAGCTTATTTTGATTAATTTGAATTTAATATAATTTCAGTAAAACATCTCAACAA

CAM 236 AGTCTCAGCTTATTTTGATTAATTTGAATTTAATATAATTTCAGTAAAACATCTCAACAA

Blaine Creek AGTCTCAGCTTATTTTGATTAATTTGAATTTAATATAATTTCAGTAAAACATCTCAACAA

CN 119300 AGTCTCAGCTTATTTTGATTAATTTGAATTTAATATAATTTCAGTAAAACATCTCAACAA

Yellowstone AGTCTCAGCTTATTTTGATTAATTTGAATTTAATATAATTTCAGTAAAACATCTCAACAA

Hoga AGTCTCAGCTTATTTTGATTAATTTGAATTTAATATAATTTCAGTAAAACATCTCAACAA

CN 120027 AGTCTCAGCTTATTTTGATTAATTTGAATTTAATATAATTTCAGTAAAACATCTCAACAA

CN 120030 AGTCTCAGCTTATTTTGATTAATTTGAATTTAATATAATTTCAGTAAAACATCTCAACAA

CN 119294 AGTCTCAGCTTATTTTGATTAATTTGAATTTAATATAATTTCAGTAAAACATCTCAACAA

CN 120013 AGTCTCAGCTTATTTTGATTAATTTGAATTTAATATAATTTCAGTAAAACATCTCAACAA

CN 120017 AGTCTCAGCTTATTTTGATTAATTTGAATTTAATATAATTTCAGTAAAACATCTCAACAA

CN 119205 AGTCTCAGCTTATTTTGATTAATTTGAATTTAATATAATTTCAGTAAAACATCTCAACAA

DH55 ref genome AGTCTCAGCTTATTTTGATTAATTTGAATTTAATATAATTTCAGTAAAACATCTCAACAA

09-CS0040 AGTCTCAGCTTATTTTGATTAATTTGAATTTAATATAATTTCAGTAAAACATCTCAACAA

CN 113754 AGTCTCAGCTTATTTTGATTAATTTGAATTTAATATAATTTCAGTAAAACATCTCAACAA

CO46 NCBI AGTCTCAGCTTATTTTGATTAATTTGAATTTAATATAATTTCAGTAAAACATCTCAACAA

Jasper AGTCTCAGCTTATTTTGATTAATTTGAATTTAATATAATTTCAGTAAAACATCTCAACAA

Joelle phyto AGTCTCAGCTTATTTTGATTAATTTGAATTTAATATAATTTCAGTAAAACATCTCAACAA

Joelle NCBI AGTCTCAGCTTATTTTGATTAATTTGAATTTAATATAATTTCAGTAAAACATCTCAACAA

CN 119243 AGTCTCAGCTTATTTTGATTAATTTGAATTTAATATAATTTCAGTAAAACATCTCAACAA

CN 120025 AGTCTCAGCTTATTTTGATTAATTTGAATTTAATATAATTTCAGTAAAACATCTCAACAA

Joelle AAFC AGTCTCAGCTTATTTTGATTAATTTGAATTTAATATAATTTCAGTAAAACATCTCAACAA

************************************************************

CAM 241 GTCAAACATACCTTGTTATTAAACAACCGTTTATGGATGCTTAATTTCTAGGATTCTATA

17CS1133 GTCAAACATACCTTGTTATTAAACAACCGTTTATGGATGCTTAATTTCTAGGATTCTATA

CAM 236 GTCAAACATACCTTGTTATTAAACAACCGTTTATGGATGCTTAATTTCTAGGATTCTATA

Blaine Creek GTCAAACATACCTTGTTATTAAACAACCGTTTATGGATGCTTAATTTCTAGGATTCTATA

CN 119300 GTCAAACATACCTTGTTATTAAACAACCGTTTATGGATGCTTAATTTCTAGGATTCTATA

Yellowstone GTCAAACATACCTTGTTATTAAACAACCGTTTATGGATGCTTAATTTCTAGGATTCTATA

Hoga GTCAAACATACCTTGTTATTAAACAACCGTTTATGGATGCTTAATTTCTAGGATTCTATA

CN 120027 GTCAAACATACCTTGTTATTAAACAACCGTTTATGGATGCTTAATTTCTAGGATTCTATA

CN 120030 GTCAAACATACCTTGTTATTAAACAACCGTTTATGGATGCTTAATTTCTAGGATTCTATA

CN 119294 GTCAAACATACCTTGTTATTAAACAACCGTTTATGGATGCTTAATTTCTAGGATTCTATA

CN 120013 GTCAAACATACCTTGTTATTAAACAACCGTTTATGGATGCTTAATTTCTAGGATTCTATA

CN 120017 GTCAAACATACCTTGTTATTAAACAACCGTTTATGGATGCTTAATTTCTAGGATTCTATA

CN 119205 GTCAAACATACCTTGTTATTAAACAACCGTTTATGGATGCTTAATTTCTAGGATTCTATA

DH55 ref genome GTCAAACATACCTTGTTATTAAACAACCGTTTATGGATGCTTAATTTCTAGGATTCTATA

09-CS0040 GTCAAACATACCTTGTTATTAAACAACCGTTTATGGATGCTTAATTTCTAGGATTCTATA

CN 113754 GTCAAACATACCTTGTTATTAAACAACCGTTTATGGATGCTTAATTTCTAGGATTCTATA

CO46 NCBI GTCAAACATACCTTGTTATTAAACAACCGTTTATGGATGCTTAATTTCTAGGATTCTATA

Jasper GTCAAACATACCTTGTTATTAAACAACCGTTTATGGATGCTTAATTTCTAGGATTCTATA

Joelle phyto GTCAAACATACCTTGTTATTAAACAACCGTTTATGGATGCTTAATTTCTAGGATTCTATA

Joelle NCBI GTCAAACATACCTTGTTATTAAACAACCGTTTATGGATGCTTAATTTCTAGGATTCTATA

CN 119243 GTCAAACATACCTTGTTATTAAACAACCGTTTATGGATGCTTAATTTCTAGGATTCTATA

CN 120025 GTCAAACATACCTTGTTATTAAACAACCGTTTATGGATGCTTAATTTCTAGGATTCTATA

Joelle AAFC GTCAAACATACCTTGTTATTAAACAACCGTTTATGGATGCTTAATTTCTAGGATTCTATA

************************************************************

CAM 241 TGTAATAATTACCAAGTCCATGAATATCATTTGGTCAGTACTTAGTTTATATGGTAATAT

17CS1133 TGTAATAATTACCAAGTCCATGAATATCATTTGGTCAGTACTTAGTTTATATGGTAATAT

CAM 236 TGTAATAATTACCAAGTCCATGAATATCATTTGGTCAGTACTTAGTTTATATGGTAATAT

Blaine Creek TGTAATAATTACCAAGTCCATGAATATCATTTGGTCAGTACTTAGTTTATATGGTAATAT

CN 119300 TGTAATAATTACCAAGTCCATGAATATCATTTGGTCAGTACTTAGTTTATATGGTAATAT

Yellowstone TGTAATAATTACCAAGTCCATGAATATCATTTGGTCAGTACTTAGTTTATATGGTAATAT

Hoga TGTAATAATTACCAAGTCCATGAATATCATTTGGTCAGTACTTAGTTTATATGGTAATAT

CN 120027 TGTAATAATTACCAAGTCCATGAATATCATTTGGTCAGTACTTAGTTTATATGGTAATAT

CN 120030 TGTAATAATTACCAAGTCCATGAATATCATTTGGTCAGTACTTAGTTTATATGGTAATAT

CN 119294 TGTAATAATTACCAAGTCCATGAATATCATTTGGTCAGTACTTAGTTTATATGGTAATAT

CN 120013 TGTAATAATTACCAAGTCCATGAATATCATTTGGTCAGTACTTAGTTTATATGGTAATAT

CN 120017 TGTAATAATTACCAAGTCCATGAATATCATTTGGTCAGTACTTAGTTTATATGGTAATAT

CN 119205 TGTAATAATTACCAAGTCCATGAATATCATTTGGTCAGTACTTAGTTTATATGGTAATAT

DH55 ref genome TGTAATAATTACCAAGTCCATGAATATCATTTGGTCAGTACTTAGTTTATATGGTAATAT

09-CS0040 TGTAATAATTACCAAGTCCATGAATATCATTTGGTCAGTACTTAGTTTATATGGTAATAT

CN 113754 TGTAATAATTACCAAGTCCATGAATATCATTTGGTCAGTACTTAGTTTATATGGTAATAT

CO46 NCBI TGTAATAATTACCAAGTCCATGAATATCATTTGGTCAGTACTTAGTTTATATGGTAATAT

Jasper TGTAATAATTACCAAGTCCATGAATATCATTTGGTCAGTACTTAGTTTATATGGTAATAT

Joelle phyto TGTAATAATTACCAAGTCCATGAATATCATTTGGTCAGTACTTAGTTTATATGGTAATAT

Joelle NCBI TGTAATAATTACCAAGTCCATGAATATCATTTGGTCAGTACTTAGTTTATATGGTAATAT

CN 119243 TGTAATAATTACCAAGTCCATGAATATCATTTGGTCAGTACTTAGTTTATATGGTAATAT

CN 120025 TGTAATAATTACCAAGTCCATGAATATCATTTGGTCAGTACTTAGTTTATATGGTAATAT

Joelle AAFC TGTAATAATTACCAAGTCCATGAATATCATTTGGTCAGTACTTAGTTTATATGGTAATAT

************************************************************

CAM 241 GGAATGTTTTTTTACCTTTGTTTCGTGAATCCTATTTATTGGTCTGAACTAGGGTCTTAC

17CS1133 GGAATGTTTTTTTACCTTTGTTTCGTGAATCCTATTTATTGGTCTGAACTAGGGTCTTAC

CAM 236 GGAATGTTTTTTTACCTTTGTTTCGTGAATCCTATTTATTGGTCTGAACTAGGGTCTTAC

Blaine Creek GGAATGTTTTTTTACCTTTGTTTCGTGAATCCTATTTATTGGTCTGAACTAGGGTCTTAC

CN 119300 GGAATGTTTTTTTACCTTTGTTTCGTGAATCCTATTTATTGGTCTGAACTAGGGTCTTAC

Yellowstone GGAATGTTTTTTTACCTTTGTTTCGTGAATCCTATTTATTGGTCTGAACTAGGGTCTTAC

Hoga GGAATGTTTTTTTACCTTTGTTTCGTGAATCCTATTTATTGGTCTGAACTAGGGTCTTAC

CN 120027 GGAATGTTTTTTTACCTTTGTTTCGTGAATCCTATTTATTGGTCTGAACTAGGGTCTTAC

CN 120030 GGAATGTTTTTTTACCTTTGTTTCGTGAATCCTATTTATTGGTCTGAACTAGGGTCTTAC

CN 119294 GGAATGTTTTTTTACCTTTGTTTCGTGAATCCTATTTATTGGTCTGAACTAGGGTCTTAC

CN 120013 GGAATGTTTTTTTACCTTTGTTTCGTGAATCCTATTTATTGGTCTGAACTAGGGTCTTAC

CN 120017 GGAATGTTTTTTTACCTTTGTTTCGTGAATCCTATTTATTGGTCTGAACTAGGGTCTTAC

CN 119205 GGAATGTTTTTTTACCTTTGTTTCGTGAATCCTATTTATTGGTCTGAACTAGGGTCTTAC

DH55 ref genome GGAATGTTTTTTTACCTTTGTTTCGTGAATCCTATTTATTGGTCTGAACTAGGGTCTTAC

09-CS0040 GGAATGTTTTTTTACCTTTGTTTCGTGAATCCTATTTATTGGTCTGAACTAGGGTCTTAC

CN 113754 GGAATGTTTTTTTACCTTTGTTTCGTGAATCCTATTTATTGGTCTGAACTAGGGTCTTAC

CO46 NCBI GGAATGTTTTTTTACCTTTGTTTCGTGAATCCTATTTATTGGTCTGAACTAGGGTCTTAC

Jasper GGAATGTTTTTTTACCTTTGTTTCGTGAATCCTATTTATTGGTCTGAACTAGGGTCTTAC

Joelle phyto GGAATGTTTTTTTACCTTTGTTTCGTGAATCCTATTTATTGGTCTGAACTAGGGTCTTAC

Joelle NCBI GGAATGTTTTTTTACCTTTGTTTCGTGAATCCTATTTATTGGTCTGAACTAGGGTCTTAC

CN 119243 GGAATGTTTTTTTACC-TTGTTTCGTGAATCCTAATTATTGGTCTGAACTAGGGTCTTAC

CN 120025 GGAATGTTTTTTTACC-TTGTTTCGTGAATCCTAATTATTGGTCTGAACTAGGGTCTTAC

Joelle AAFC GGAATGTTTTTTTACC-TTGTTTCGTGAATCCTAATTATTGGTCTGAACTAGGGTCTTAC

**************** ***************** *************************

CAM 241 CAAAAAAGCGTAATACAGTATATTGAACTAGAGCTCTTAGCATTCTTATCATGAATAATG

17CS1133 CAAAAAAGCGTAATACAGTATATTGAACTAGAGCTCTTAGCATTCTTATCATGAATAATG

CAM 236 CAAAAAAGCGTAATACAGTATATTGAACTAGAGCTCTTAGCATTCTTATCATGAATAATG

Blaine Creek CAAAAAAGCGTAATACAGTATATTGAACTAGAGCTCTTAGCATTCTTATCATGAATAATG

CN 119300 CAAAAAAGCGTAATACAGTATATTGAACTAGAGCTCTTAGCATTCTTATCATGAATAATG

Yellowstone CAAAAAAGCGTAATACAGTATATTGAACTAGAGCTCTTAGCATTCTTATCATGAATAATG

Hoga CAAAAAAGCGTAATACAGTATATTGAACTAGAGCTCTTAGCATTCTTATCATGAATAATG

CN 120027 CAAAAAAGCGTAATACAGTATATTGAACTAGAGCTCTTAGCATTCTTATCATGAATAATG

CN 120030 CAAAAAAGCGTAATACAGTATATTGAACTAGAGCTCTTAGCATTCTTATCATGAATAATG

CN 119294 CAAAAAAGCGTAATACAGTATATTGAACTAGAGCTCTTAGCATTCTTATCATGAATAATG

CN 120013 CAAAAAAGCGTAATACAGTATATTGAACTAGAGCTCTTAGCATTCTTATCATGAATAATG

CN 120017 CAAAAAAGCGTAATACAGTATATTGAACTAGAGCTCTTAGCATTCTTATCATGAATAATG

CN 119205 CAAAAAAGCGTAATACAGTATATTGAACTAGAGCTCTTAGCATTCTTATCATGAATAATG

DH55 ref genome CAAAAAAGCGTAATACAGTATATTGAACTAGAGCTCTTAGCATTCTTATCATGAATAATG

09-CS0040 CAAAAAAGCGTAATACAGTATATTGAACTAGAGCTCTTAGCATTCTTATCATGAATAATG

CN 113754 CAAAAAAGCGTAATACAGTATATTGAACTAGAGCTCTTAGCATTCTTATCATGAATAATG

CO46 NCBI CAAAAAAGCGTAATACAGTATATTGAACTAGAGCTCTTAGCATTCTTATCATGAATAATG

Jasper CAAAAAAGCGTAATACAGTATATTGAACTAGAGCTCTTAGCATTCTTATCATGAATAATG

Joelle phyto CAAAAAAGCGTAATACAGTATATTGAACTAGAGCTCTTAGCATTCTTATCATGAATAATG

Joelle NCBI CAAAAAAGCGTAATACAGTATATTGAACTAGAGCTCTTAGCATTCTTATCATGAATAATG

CN 119243 CAAAAAAGCGTAATACAGTATATTGAACTAGAGCTCTTAGCATTCTTATCATGAATAATG

CN 120025 CAAAAAAGCGTAATACAGTATATTGAACTAGAGCTCTTAGCATTCTTATCATGAATAATG

Joelle AAFC CAAAAAAGCGTAATACAGTATATTGAACTAGAGCTCTTAGCATTCTTATCATGAATAATG

************************************************************

CAM 241 TTATACCGGATCCACACTAGCTATATATAAGTGAAGCTATATCGAAGAACAAGAAAAATG

17CS1133 TTATACCGGATCCACACTAGCTATATATAAGTGAAGCTATATCGAAGAACAAGAAAAATG

CAM 236 TTATACCGGATCCACACTAGCTATATATAAGTGAAGCTATATCGAAGAACAAGAAAAATG

Blaine Creek TTATACCGGATCCACACTAGCTATATATAAGTGAAGCTATATCGAAGAACAAGAAAAATG

CN 119300 TTATACCGGATCCACACTAGCTATATATAAGTGAAGCTATATCGAAGAACAAGAAAAATG

Yellowstone TTATACCGGATCCACACTAGCTATATATAAGTGAAGCTATATCGAAGAACAAGAAAAATG

Hoga TTATACCGGATCCACACTAGCTATATATAAGTGAAGCTATATCGAAGAACAAGAAAAATG

CN 120027 TTATACCGGATCCACACTAGCTATATATAAGTGAAGCTATATCGAAGAACAAGAAAAATG

CN 120030 TTATACCGGATCCACACTAGCTATATATAAGTGAAGCTATATCGAAGAACAAGAAAAATG

CN 119294 TTATACCGGATCCACACTAGCTATATATAAGTGAAGCTATATCGAAGAACAAGAAAAATG

CN 120013 TTATACCGGATCCACACTAGCTATATATAAGTGAAGCTATATCGAAGAACAAGAAAAATG

CN 120017 TTATACCGGATCCACACTAGCTATATATAAGTGAAGCTATATCGAAGAACAAGAAAAATG

CN 119205 TTATACCGGATCCACACTAGCTATATATAAGTGAAGCTATATCGAAGAACAAGAAAAATG

DH55 ref genome TTATACCGGATCCACACTAGCTATATATAAGTGAAGCTATATCGAAGAACAAGAAAAATG

09-CS0040 TTATACCGGATCCACACTAGCTATATATAAGTGAAGCTATATCGAAGAACAAGAAAAATG

CN 113754 TTATACCGGATCCACACTAGCTATATATAAGTGAAGCTATATCGAAGAACAAGAAAAATG

CO46 NCBI TTATACCGGATCCACACTAGCTATATATAAGTGAAGCTATATCGAAGAACAAGAAAAATG

Jasper TTATACCGGATCCACACTAGCTATATATAAGTGAAGCTATATCGAAGAACAAGAAAAATG

Joelle phyto TTATACCGGATCCACACTAGCTATATATAAGTGAAGCTATATCGAAGAACAAGAAAAATG

Joelle NCBI TTATACCGGATCCACACTAGCTATATATAAGTGAAGCTATATCGAAGAACAAGAAAAATG

CN 119243 TTATACCGGATCCACACTAGCTATATATAAGTGAAGCTATATCGAAGAACAAGAAAAATG

CN 120025 TTATACCGGATCCACACTAGCTATATATAAGTGAAGCTATATCGAAGAACAAGAAAAATG

Joelle AAFC TTATACCGGATCCACACTAGCTATATATAAGTGAAGCTATATCGAAGAACAAGAAAAATG

************************************************************

CAM 241 GCTAAAGAACATATATCAGCCATGAAACTGATTAGATATTTAGTAACTATATTGGTGGTA

17CS1133 GCTAAAGAACATATATCAGCCATGAAACTGATTAGATATTTAGTAACTATATTGGTGGTA

CAM 236 GCTAAAGAACATATATCAGCCATGAAACTGATTAGATATTTAGTAACTATATTGGTGGTA

Blaine Creek GCTAAAGAACATATATCAGCCATGAAACTGATTAGATATTTAGTAACTATATTGGTGGTA

CN 119300 GCTAAAGAACATATATCAGCCATGAAACTGATTAGATATTTAGTAACTATATTGGTGGTA

Yellowstone GCTAAAGAACATATATCAGCCATGAAACTGATTAGATATTTAGTAACTATATTGGTGGTA

Hoga GCTAAAGAACATATATCAGCCATGAAACTGATTAGATATTTAGTAACTATATTGGTGGTA

CN 120027 GCTAAAGAACATATATCAGCCATGAAACTGATTAGATATTTAGTAACTATATTGGTGGTA

CN 120030 GCTAAAGAACATATATCAGCCATGAAACTGATTAGATATTTAGTAACTATATTGGTGGTA

CN 119294 GCTAAAGAACATATATCAGCCATGAAACTGATTAGATATTTAGTAACTATATTGGTGGTA

CN 120013 GCTAAAGAACATATATCAGCCATGAAACTGATTAGATATTTAGTAACTATATTGGTGGTA

CN 120017 GCTAAAGAACATATATCAGCCATGAAACTGATTAGATATTTAGTAACTATATTGGTGGTA

CN 119205 GCTAAAGAACATATATCAGCCATGAAACTGATTAGATATTTAGTAACTATATTGGTGGTA

DH55 ref genome GCTAAAGAACATATATCAGCCATGAAACTGATTAGATATTTAGTAACTATATTGGTGGTA

09-CS0040 GCTAAAGAACATATATCAGCCATGAAACTGATTAGATATTTAGTAACTATATTGGTGGTA

CN 113754 GCTAAAGAACATATATCAGCCATGAAACTGATTAGATATTTAGTAACTATATTGGTGGTA

CO46 NCBI GCTAAAGAACATATATCAGCCATGAAACTGATTAGATATTTAGTAACTATATTGGTGGTA

Jasper GCTAAAGAACATATATCAGCCATGAAACTGATTAGATATTTAGTAACTATATTGGTGGTA

Joelle phyto GCTAAAGAACATATATCAGCCATGAAACTGATTAGATATTTAGTAACTATATTGGTGGTA

Joelle NCBI GCTAAAGAACATATATCAGCCATGAAACTGATTAGATATTTAGTAACTATATTGGTGGTA

CN 119243 GCTAAAGAACATATATCAGCCATGAAACTGATTAGATATTTAGTAACTATATTGGTGGTA

CN 120025 GCTAAAGAACATATATCAGCCATGAAACTGATTAGATATTTAGTAACTATATTGGTGGTA

Joelle AAFC GCTAAAGAACATATATCAGCCATGAAACTGATTAGATATTTAGTAACTATATTGGTGGTA

************************************************************

CAM 241 GTAAGAATTAATTAAGTTTGGCAAAAATATTTCTTTTGTGATAGCTAATGGCCTAATGTA

17CS1133 GTAAGAATTAATTAAGTTTGGCAAAAATATTTCTTTTGTGATAGCTAATGGCCTAATGTA

CAM 236 GTAAGAATTAATTAAGTTTGGCAAAAATATTTCTTTTGTGATAGCTAATGGCCTAATGTA

Blaine Creek GTAAGAATTAATTAAGTTTGGCAAAAATATTTCTTTTGTGATAGCTAATGGCCTAATGTA

CN 119300 GTAAGAATTAATTAAGTTTGGCAAAAATATTTCTTTTGTGATAGCTAATGGCCTAATGTA

Yellowstone GTAAGAATTAATTAAGTTTGGCAAAAATATTTCTTTTGTGATAGCTAATGGCCTAATGTA

Hoga GTAAGAATTAATTAAGTTTGGCAAAAATATTTCTTTTGTGATAGCTAATGGCCTAATGTA

CN 120027 GTAAGAATTAATTAAGTTTGGCAAAAATATTTCTTTTGTGATAGCTAATGGCCTAATGTA

CN 120030 GTAAGAATTAATTAAGTTTGGCAAAAATATTTCTTTTGTGATAGCTAATGGCCTAATGTA

CN 119294 GTAAGAATTAATTAAGTTTGGCAAAAATATTTCTTTTGTGATAGCTAATGGCCTAATGTA

CN 120013 GTAAGAATTAATTAAGTTTGGCAAAAATATTTCTTTTGTGATAGCTAATGGCCTAATGTA

CN 120017 GTAAGAATTAATTAAGTTTGGCAAAAATATTTCTTTTGTGATAGCTAATGGCCTAATGTA

CN 119205 GTAAGAATTAATTAAGTTTGGCAAAAATATTTCTTTTGTGATAGCTAATGGCCTAATGTA

DH55 ref genome GTAAGAATTAATTAAGTTTGGCAAAAATATTTCTTTTGTGATAGCTAATGGCCTAATGTA

09-CS0040 GTAAGAATTAATTAAGTTTGGCAAAAATATTTCTTTTGTGATAGCTAATGGCCTAATGTA

CN 113754 GTAAGAATTAATTAAGTTTGGCAAAAATATTTCTTTTGTGATAGCTAATGGCCTAATGTA

CO46 NCBI GTAAGAATTAATTAAGTTTGGCAAAAATATTTCTTTTGTGATAGCTAATGGCCTAATGTA

Jasper GTAAGAATTAATTAAGTTTGGCAAAAATATTTCTTTTGTGATAGCTAATGGCCTAATGTA

Joelle phyto GTAAGAATTAATTAAGTTTGGCAAAAATATTTCTTTTGTGATAGCTAATGGCCTAATGTA

Joelle NCBI GTAAGAATTAATTAAGTTTGGCAAAAATATTTCTTTTGTGATAGCTAATGGCCTAATGTA

CN 119243 GTAAGAATTAATTAAGTTTGGCAAAAATATTTCTTTTGTGATGGCTAATGGCCTAATGTA

CN 120025 GTAAGAATTAATTAAGTTTGGCAAAAATATTTCTTTTGTGATGGCTAATGGCCTAATGTA

Joelle AAFC GTAAGAATTAATTAAGTTTGGCAAAAATATTTCTTTTGTGATGGCTAATGGCCTAATGTA

****************************************** *****************

CAM 241 ACTATGTTTATGTGGTAGTAATGCACTTAAAAAGAAAATATGCTAGTTAACTTCATGAGA

17CS1133 ACTATGTTTATGTGGTAGTAATGCACTTAAAAAGAAAATATGCTAGTTAACTTCATGAGA

CAM 236 ACTATGTTTATGTGGTAGTAATGCACTTAAAAAGAAAATATGCTAGTTAACTTCATGAGA

Blaine Creek ACTATGTTTATGTGGTAGTAATGCACTTAAAAAGAAAATATGCTAGTTAACTTCATGAGA

CN 119300 ACTATGTTTATGTGGTAGTAATGCACTTAAAAAGAAAATATGCTAGTTAACTTCATGAGA

Yellowstone ACTATGTTTATGTGGTAGTAATGCACTTAAAAAGAAAATATGCTAGTTAACTTCATGAGA

Hoga ACTATGTTTATGTGGTAGTAATGCACTTAAAAAGAAAATATGCTAGTTAACTTCATGAGA

CN 120027 ACTATGTTTATGTGGTAGTAATGCACTTAAAAAGAAAATATGCTAGTTAACTTCATGAGA

CN 120030 ACTATGTTTATGTGGTAGTAATGCACTTAAAAAGAAAATATGCTAGTTAACTTCATGAGA

CN 119294 ACTATGTTTATGTGGTAGTAATGCACTTAAAAAGAAAATATGCTAGTTAACTTCATGAGA

CN 120013 ACTATGTTTATGTGGTAGTAATGCACTTAAAAAGAAAATATGCTAGTTAACTTCATGAGA

CN 120017 ACTATGTTTATGTGGTAGTAATGCACTTAAAAAGAAAATATGCTAGTTAACTTCATGAGA

CN 119205 ACTATGTTTATGTGGTAGTAATGCACTTAAAAAGAAAATATGCTAGTTAACTTCATGAGA

DH55 ref genome ACTATGTTTATGTGGTAGTAATGCACTTAAAAAGAAAATATGCTAGTTAACTTCATGAGA

09-CS0040 ACTATGTTTATGTGGTAGTAATGCACTTAAAAAGAAAATATGCTAGTTAACTTCATGAGA

CN 113754 ACTATGTTTATGTGGTAGTAATGCACTTAAAAAGAAAATATGCTAGTTAACTTCATGAGA

CO46 NCBI ACTATGTTTATGTGGTAGTAATGCACTTAAAAAGAAAATATGCTAGTTAACTTCATGAGA

Jasper ACTATGTTTATGTGGTAGTAATGCACTTAAAAAGAAAATATGCTAGTTAACTTCATGAGA

Joelle phyto ACTATGTTTATGTGGTAGTAATGCACTTAAAAAGAAAATATGCTAGTTAACTTCATGAGA

Joelle NCBI ACTATGTTTATGTGGTAGTAATGCACTTAAAAAGAAAATATGCTAGTTAACTTCATGAGA

CN 119243 ACTATGTTTATGTGGTAGTAATGCACTTAAAAAGAAAATATGCTAGTTAACTTCATGAGA

CN 120025 ACTATGTTTATGTGGTAGTAATGCACTTAAAAAGAAAATATGCTAGTTAACTTCATGAGA

Joelle AAFC ACTATGTTTATGTGGTAGTAATGCACTTAAAAAGAAAATATGCTAGTTAACTTCATGAGA

************************************************************

CAM 241 AGATAAAATGTTACAAATACTAATTCTAATAAGAAAAAATTACCTTAACACACATCAAAG

17CS1133 AGATAAAATGTTACAAATACTAATTCTAATAAGAAAAAATTACCTTAACACACATCAAAG

CAM 236 AGATAAAATGTTACAAATACTAATTCTAATAAGAAAAAATTACCTTAACACACATCAAAG

Blaine Creek AGATAAAATGTTACAAATACTAATTCTAATAAGAAAAAATTACCTTAACACACATCAAAG

CN 119300 AGATAAAATGTTACAAATACTAATTCTAATAAGAAAAAATTACCTTAACACACATCAAAG

Yellowstone AGATAAAATGTTACAAATACTAATTCTAATAAGAAAAAATTACCTTAACACACATCAAAG

Hoga AGATAAAATGTTACAAATACTAATTCTAATAAGAAAAAATTACCTTAACACACATCAAAG

CN 120027 AGATAAAATGTTACAAATACTAATTCTAATAAGAAAAAATTACCTTAACACACATCAAAG

CN 120030 AGATAAAATGTTACAAATACTAATTCTAATAAGAAAAAATTACCTTAACACACATCAAAG

CN 119294 AGATAAAATGTTACAAATACTAATTCTAATAAGAAAAAATTACCTTAACACACATCAAAG

CN 120013 AGATAAAATGTTACAAATACTAATTCTAATAAGAAAAAATTACCTTAACACACATCAAAG

CN 120017 AGATAAAATGTTACAAATACTAATTCTAATAAGAAAAAATTACCTTAACACACATCAAAG

CN 119205 AGATAAAATGTTACAAATACTAATTCTAATAAGAAAAAATTACCTTAACACACATCAAAG

DH55 ref genome AGATAAAATGTTACAAATACTAATTCTAATAAGAAAAAATTACCTTAACACACATCAAAG

09-CS0040 AGATAAAATGTTACAAATACTAATTCTAATAAGAAAAAATTACCTTAACACACATCAAAG

CN 113754 AGATAAAATGTTACAAATACTAATTCTAATAAGAAAAAATTACCTTAACACACATCAAAG

CO46 NCBI AGATAAAATGTTACAAATACTAATTCTAATAAGAAAAAATTACCTTAACACACATCAAAG

Jasper AGATAAAATGTTACAAATACTAATTCTAATAAGAAAAAATTACCTTAACACACATCAAAG

Joelle phyto AGATAAAATGTTACAAATACTAATTCTAATAAGAAAAAATTACCTTAACACACATCAAAG

Joelle NCBI AGATAAAATGTTACAAATACTAATTCTAATAAGAAAAAATTACCTTAACACACATCAAAG

CN 119243 AGATAAAATGTTACAAATACTAATTCTAATAAGAAAAAATTACCTTAACACACATCAAAG

CN 120025 AGATAAAATGTTACAAATACTAATTCTAATAAGAAAAAATTACCTTAACACACATCAAAG

Joelle AAFC AGATAAAATGTTACAAATACTAATTCTAATAAGAAAAAATTACCTTAACACACATCAAAG

************************************************************

CAM 241 AGAAGAACCAACTGACTTTAAGCATATTAACGATATCAAACCTTTCGGGTTAATTGATAC

17CS1133 AGAAGAACCAACTGACTTTAAGCATATTAACGATATCAAACCTTTCGGGTTAATTGATAC

CAM 236 AGAAGAACCAACTGACTTTAAGCATATTAACGATATCAAACCTTTCGGGTTAATTGATAC

Blaine Creek AGAAGAACCAACTGACTTTAAGCATATTAACGATATCAAACCTTTCGGGTTAATTGATAC

CN 119300 AGAAGAACCAACTGACTTTAAGCATATTAACGATATCAAACCTTTCGGGTTAATTGATAC

Yellowstone AGAAGAACCAACTGACTTTAAGCATATTAACGATATCAAACCTTTCGGGTTAATTGATAC

Hoga AGAAGAACCAACTGACTTTAAGCATATTAACGATATCAAACCTTTCGGGTTAATTGATAC

CN 120027 AGAAGAACCAACTGACTTTAAGCATATTAACGATATCAAACCTTTCGGGTTAATTGATAC

CN 120030 AGAAGAACCAACTGACTTTAAGCATATTAACGATATCAAACCTTTCGGGTTAATTGATAC

CN 119294 AGAAGAACCAACTGACTTTAAGCATATTAACGATATCAAACCTTTCGGGTTAATTGATAC

CN 120013 AGAAGAACCAACTGACTTTAAGCATATTAACGATATCAAACCTTTCGGGTTAATTGATAC

CN 120017 AGAAGAACCAACTGACTTTAAGCATATTAACGATATCAAACCTTTCGGGTTAATTGATAC

CN 119205 AGAAGAACCAACTGACTTTAAGCATATTAACGATATCAAACCTTTCGGGTTAATTGATAC

DH55 ref genome AGAAGAACCAACTGACTTTAAGCATATTAACGATATCAAACCTTTCGGGTTAATTGATAC

09-CS0040 AGAAGAACCAACTGACTTTAAGCATATTAACGATATCAAACCTTTCGGGTTAATTGATAC

CN 113754 AGAAGAACCAACTGACTTTAAGCATATTAACGATATCAAACCTTTCGGGTTAATTGATAC

CO46 NCBI AGAAGAACCAACTGACTTTAAGCATATTAACGATATCAAACCTTTCGGGTTAATTGATAC

Jasper AGAAGAACCAACTGACTTTAAGCATATTAACGATATCAAACCTTTCGGGTTAATTGATAC

Joelle phyto AGAAGAACCAACTGACTTTAAGCATATTAACGATATCAAACCTTTCGGGTTAATTGATAC

Joelle NCBI AGAAGAACCAACTGACTTTAAGCATATTAACGATATCAAACCTTTCGGGTTAATTGATAC

CN 119243 AGAAGAACCAACTGACTTTAAGCATATTAACGATATCAAACCTTTCGGGTTAATTGATAC

CN 120025 AGAAGAACCAACTGACTTTAAGCATATTAACGATATCAAACCTTTCGGGTTAATTGATAC

Joelle AAFC AGAAGAACCAACTGACTTTAAGCATATTAACGATATCAAACCTTTCGGGTTAATTGATAC

************************************************************

CAM 241 TATACTAACTGCCAAATTTTAGTTTTAGAGATGTGGAAGAGTTCAATTGCAATAAAGAGA

17CS1133 TATACTAACTGCCAAATTTTAGTTTTAGAGATGTGGAAGAGTTCAATTGCAATAAAGAGA

CAM 236 TATACTAACTGCCAAATTTTAGTTTTAGAGATGTGGAAGAGTTCAATTGCAATAAAGAGA

Blaine Creek TATACTAACTGCCAAATTTTAGTTTTAGAGATGTGGAAGAGTTCAATTGCAATAAAGAGA

CN 119300 TATACTAACTGCCAAATTTTAGTTTTAGAGATGTGGAAGAGTTCAATTGCAATAAAGAGA

Yellowstone TATACTAACTGCCAAATTTTAGTTTTAGAGATGTGGAAGAGTTCAATTGCAATAAAGAGA

Hoga TATACTAACTGCCAAATTTTAGTTTTAGAGATGTGGAAGAGTTCAATTGCAATAAAGAGA

CN 120027 TATACTAACTGCCAAATTTTAGTTTTAGAGATGTGGAAGAGTTCAATTGCAATAAAGAGA

CN 120030 TATACTAACTGCCAAATTTTAGTTTTAGAGATGTGGAAGAGTTCAATTGCAATAAAGAGA

CN 119294 TATACTAACTGCCAAATTTTAGTTTTAGAGATGTGGAAGAGTTCAATTGCAATAAAGAGA

CN 120013 TATACTAACTGCCAAATTTTAGTTTTAGAGATGTGGAAGAGTTCAATTGCAATAAAGAGA

CN 120017 TATACTAACTGCCAAATTTTAGTTTTAGAGATGTGGAAGAGTTCAATTGCAATAAAGAGA

CN 119205 TATACTAACTGCCAAATTTTAGTTTTAGAGATGTGGAAGAGTTCAATTGCAATAAAGAGA

DH55 ref genome TATACTAACTGCCAAATTTTAGTTTTAGAGATGTGGAAGAGTTCAATTGCAATAAAGAGA

09-CS0040 TATACTAACTGCCAAATTTTAGTTTTAGAGATGTGGAAGAGTTCAATTGCAATAAAGAGA

CN 113754 TATACTAACTGCCAAATTTTAGTTTTAGAGATGTGGAAGAGTTCAATTGCAATAAAGAGA

CO46 NCBI TATACTAACTGCCAAATTTTAGTTTTAGAGATGTGGAAGAGTTCAATTGCAATAAAGAGA

Jasper TATACTAACTGCCAAATTTTAGTTTTAGAGATGTGGAAGAGTTCAATTGCAATAAAGAGA

Joelle phyto TATACTAACTGCCAAATTTTAGTTTTAGAGATGTGGAAGAGTTCAATTGCAATAAAGAGA

Joelle NCBI TATACTAACTGCCAAATTTTAGTTTTAGAGATGTGGAAGAGTTCAATTGCAATAAAGAGA

CN 119243 TATACTAACTGCCAAATTTTAGTTTTAGAGATGTGGAAGAGTTCAATTGCAATAAAGAGA

CN 120025 TATACTAACTGCCAAATTTTAGTTTTAGAGATGTGGAAGAGTTCAATTGCAATAAAGAGA

Joelle AAFC TATACTAACTGCCAAATTTTAGTTTTAGAGATGTGGAAGAGTTCAATTGCAATAAAGAGA

************************************************************

CAM 241 ACAAAAAAATTAAGAATTCGATTTGAAAGAGTTCAAAGGTGATGGAGAGAAGGATCTCTC

17CS1133 ACAAAAAAATTAAGAATTCGATTTGAAAGAGTTCAAAGGTGATGGAGAGAAGGATCTCTC

CAM 236 ACAAAAAAATTAAGAATTCGATTTGAAAGAGTTCAAAGGTGATGGAGAGAAGGATCTCTC

Blaine Creek ACAAAAAAATTAAGAATTCGATTTGAAAGAGTTCAAAGGTGATGGAGAGAAGGATCTCTC

CN 119300 ACAAAAAAATTAAGAATTCGATTTGAAAGAGTTCAAAGGTGATGGAGAGAAGGATCTCTC

Yellowstone ACAAAAAAATTAAGAATTCGATTTGAAAGAGTTCAAAGGTGATGGAGAGAAGGATCTCTC

Hoga ACAAAAAAATTAAGAATTCGATTTGAAAGAGTTCAAAGGTGATGGAGAGAAGGATCTCTC

CN 120027 ACAAAAAAATTAAGAATTCGATTTGAAAGAGTTCAAAGGTGATGGAGAGAAGGATCTCTC

CN 120030 ACAAAAAAATTAAGAATTCGATTTGAAAGAGTTCAAAGGTGATGGAGAGAAGGATCTCTC

CN 119294 ACAAAAAAATTAAGAATTCGATTTGAAAGAGTTCAAAGGTGATGGAGAGAAGGATCTCTC

CN 120013 ACAAAAAAATTAAGAATTCGATTTGAAAGAGTTCAAAGGTGATGGAGAGAAGGATCTCTC

CN 120017 ACAAAAAAATTAAGAATTCGATTTGAAAGAGTTCAAAGGTGATGGAGAGAAGGATCTCTC

CN 119205 ACAAAAAAATTAAGAATTCGATTTGAAAGAGTTCAAAGGTGATGGAGAGAAGGATCTCTC

DH55 ref genome ACAAAAAAATTAAGAATTCGATTTGAAAGAGTTCAAAGGTGATGGAGAGAAGGATCTCTC

09-CS0040 ACAAAAAAATTAAGAATTCGATTTGAAAGAGTTCAAAGGTGATGGAGAGAAGGATCTCTC

CN 113754 ACAAAAAAATTAAGAATTCGATTTGAAAGAGTTCAAAGGTGATGGAGAGAAGGATCTCTC

CO46 NCBI ACAAAAAAATTAAGAATTCGATTTGAAAGAGTTCAAAGGTGATGGAGAGAAGGATCTCTC

Jasper ACAAAAAAATTAAGAATTCGATTTGAAAGAGTTCAAAGGTGATGGAGAGAAGGATCTCTC

Joelle phyto ACAAAAAAATTAAGAATTCGATTTGAAAGAGTTCAAAGGTGATGGAGAGAAGGATCTCTC

Joelle NCBI ACAAAAAAATTAAGAATTCGATTTGAAAGAGTTCAAAGGTGATGGAGAGAAGGATCTCTC

CN 119243 ACAAAAAAATTAAGAATTCGATTTGAAAGAGTTCAAAGGTGATGGAGAGAAGGATCTCTC

CN 120025 ACAAAAAAATTAAGAATTCGATTTGAAAGAGTTCAAAGGTGATGGAGAGAAGGATCTCTC

Joelle AAFC ACAAAAAAATTAAGAATTCGATTTGAAAGAGTTCAAAGGTGATGGAGAGAAGGATCTCTC

************************************************************

CAM 241 CTCAGAAGGTTTAGATCTCTCTCTACAATGAGGGAAAATATATAGTTTGTCAATAGAGAT

17CS1133 CTCAGAAGGTTTAGATCTCTCTCTACAATGAGGGAAAATATATAGTTTGTCAATAGAGAT

CAM 236 CTCAGAAGGTTTAGATCTCTCTCTACAATGAGGGAAAATATATAGTTTGTCAATAGAGAT

Blaine Creek CTCAGAAGGTTTAGATCTCTCTCTACAATGAGGGAAAATATATAGTTTGTCAATAGAGAT

CN 119300 CTCAGAAGGTTTAGATCTCTCTCTACAATGAGGGAAAATATATAGTTTGTCAATAGAGAT

Yellowstone CTCAGAAGGTTTAGATCTCTCTCTACAATGAGGGAAAATATATAGTTTGTCAATAGAGAT

Hoga CTCAGAAGGTTTAGATCTCTCTCTACAATGAGGGAAAATATATAGTTTGTCAATAGAGAT

CN 120027 CTCAGAAGGTTTAGATCTCTCTCTACAATGAGGGAAAATATATAGTTTGTCAATAGAGAT

CN 120030 CTCAGAAGGTTTAGATCTCTCTCTACAATGAGGGAAAATATATAGTTTGTCAATAGAGAT

CN 119294 CTCAGAAGGTTTAGATCTCTCTCTACAATGAGGGAAAATATATAGTTTGTCAATAGAGAT

CN 120013 CTCAGAAGGTTTAGATCTCTCTCTACAATGAGGGAAAATATATAGTTTGTCAATAGAGAT

CN 120017 CTCAGAAGGTTTAGATCTCTCTCTACAATGAGGGAAAATATATAGTTTGTCAATAGAGAT

CN 119205 CTCAGAAGGTTTAGATCTCTCTCTACAATGAGGGAAAATATATAGTTTGTCAATAGAGAT

DH55 ref genome CTCAGAAGGTTTAGATCTCTCTCTACAATGAGGGAAAATATATAGTTTGTCAATAGAGAT

09-CS0040 CTCAGAAGGTTTAGATCTCTCTCTACAATGAGGGAAAATATATAGTTTGTCAATAGAGAT

CN 113754 CTCAGAAGGTTTAGATCTCTCTCTACAATGAGGGAAAATATATAGTTTGTCAATAGAGAT

CO46 NCBI CTCAGAAGGTTTAGATCTCTCTCTACAATGAGGGAAAATATATAGTTTGTCAATAGAGAT

Jasper CTCAGAAGGTTTAGATCTCTCTCTACAATGAGGGAAAATATATAGTTTGTCAATAGAGAT

Joelle phyto CTCAGAAGGTTTAGATCTCTCTCTACAATGAGGGAAAATATATAGTTTGTCAATAGAGAT

Joelle NCBI CTCAGAAGGTTTAGATCTCTCTCTACAATGAGGGAAAATATATAGTTTGTCAATAGAGAT

CN 119243 CTCAGAAGGTTTAGATCTCTCTCTACAATGAGGGAAAATATATAGTTTGTCAATAGAGAT

CN 120025 CTCAGAAGGTTTAGATCTCTCTCTACAATGAGGGAAAATATATAGTTTGTCAATAGAGAT

Joelle AAFC CTCAGAAGGTTTAGATCTCTCTCTACAATGAGGGAAAATATATAGTTTGTCAATAGAGAT

************************************************************

CAM 241 TGTTCTTTAACTTGAAGAGGCGACGGATCAATAGTACCAAAAACAGAAGTATAATTTAAA

17CS1133 TGTTCTTTAACTTGAAGAGGCGACGGATCAATAGTACCAAAAACAGAAGTATAATTTAAA

CAM 236 TGTTCTTTAACTTGAAGAGGCGACGGATCAATAGTACCAAAAACAGAAGTATAATTTAAA

Blaine Creek TGTTCTTTAACTTGAAGAGGCGACGGATCAATAGTACCAAAAACAGAAGTATAATTTAAA

CN 119300 TGTTCTTTAACTTGAAGAGGCGACGGATCAATAGTACCAAAAACAGAAGTATAATTTAAA

Yellowstone TGTTCTTTAACTTGAAGAGGCGACGGATCAATAGTACCAAAAACAGAAGTATAATTTAAA

Hoga TGTTCTTTAACTTGAAGAGGCGACGGATCAATAGTACCAAAAACAGAAGTATAATTTAAA

CN 120027 TGTTCTTTAACTTGAAGAGGCGACGGATCAATAGTACCAAAAACAGAAGTATAATTTAAA

CN 120030 TGTTCTTTAACTTGAAGAGGCGACGGATCAATAGTACCAAAAACAGAAGTATAATTTAAA

CN 119294 TGTTCTTTAACTTGAAGAGGCGACGGATCAATAGTACCAAAAACAGAAGTATAATTTAAA

CN 120013 TGTTCTTTAACTTGAAGAGGCGACGGATCAATAGTACCAAAAACAGAAGTATAATTTAAA

CN 120017 TGTTCTTTAACTTGAAGAGGCGACGGATCAATAGTACCAAAAACAGAAGTATAATTTAAA

CN 119205 TGTTCTTTAACTTGAAGAGGCGACGGATCAATAGTACCAAAAACAGAAGTATAATTTAAA

DH55 ref genome TGTTCTTTAACTTGAAGAGGCGACGGATCAATAGTACCAAAAACAGAAGTATAATTTAAA

09-CS0040 TGTTCTTTAACTTGAAGAGGCGACGGATCAATAGTACCAAAAACAGAAGTATAATTTAAA

CN 113754 TGTTCTTTAACTTGAAGAGGCGACGGATCAATAGTACCAAAAACAGAAGTATAATTTAAA

CO46 NCBI TGTTCTTTAACTTGAAGAGGCGACGGATCAATAGTACCAAAAACAGAAGTATAATTTAAA

Jasper TGTTCTTTAACTTGAAGAGGCGACGGATCAATAGTACCAAAAACAGAAGTATAATTTAAA

Joelle phyto TGTTCTTTAACTTGAAGAGGCGACGGATCAATAGTACCAAAAACAGAAGTATAATTTAAA

Joelle NCBI TGTTCTTTAACTTGAAGAGGCGACGGATCAATAGTACCAAAAACAGAAGTATAATTTAAA

CN 119243 TGTTCTTTAACTTGAAGAGGCGACGGATCAATAGTACCAAAAACAGAAGTATAATTTAAA

CN 120025 TGTTCTTTAACTTGAAGAGGCGACGGATCAATAGTACCAAAAACAGAAGTATAATTTAAA

Joelle AAFC TGTTCTTTAACTTGAAGAGGCGACGGATCAATAGTACCAAAAACAGAAGTATAATTTAAA

************************************************************

CAM 241 TACATATCAATGCATAATATATACTATAGAGGATAAATTACTTATGTGGAGAAAACTAGA

17CS1133 TACATATCAATGCATAATATATACTATAGAGGATAAATTACTTATGTGGAGAAAACTAGA

CAM 236 TACATATCAATGCATAATATATACTATAGAGGATAAATTACTTATGTGGAGAAAACTAGA

Blaine Creek TACATATCAATGCATAATATATACTATAGAGGATAAATTACTTATGTGGAGAAAACTAGA

CN 119300 TACATATCAATGCATAATATATACTATAGAGGATAAATTACTTATGTGGAGAAAACTAGA

Yellowstone TACATATCAATGCATAATATATACTATAGAGGATAAATTACTTATGTGGAGAAAACTAGA

Hoga TACATATCAATGCATAATATATACTATAGAGGATAAATTACTTATGTGGAGAAAACTAGA

CN 120027 TACATATCAATGCATAATATATACTATAGAGGATAAATTACTTATGTGGAGAAAACTAGA

CN 120030 TACATATCAATGCATAATATATACTATAGAGGATAAATTACTTATGTGGAGAAAACTAGA

CN 119294 TACATATCAATGCATAATATATACTATAGAGGATAAATTACTTATGTGGAGAAAACTAGA

CN 120013 TACATATCAATGCATAATATATACTATAGAGGATAAATTACTTATGTGGAGAAAACTAGA

CN 120017 TACATATCAATGCATAATATATACTATAGAGGATAAATTACTTATGTGGAGAAAACTAGA

CN 119205 TACATATCAATGCATA--ATATACTATAGAGGATAAATTACTTATGTGGAGAAAACTAGA

DH55 ref genome TACATATCAATGCATAATATATACTATAGAGGATAAATTACTTATGTGGAGAAAACTAGA

09-CS0040 TACATATCAATGCATAATATATACTATAGAGGATAAATTACTTATGTGGAGAAAACTAGA

CN 113754 TACATATCAATGCATAATATATACTATAGAGGATAAATTACTTATGTGGAGAAAACTAGA

CO46 NCBI TACATATCAATGCATAATATATACTATAGAGGATAAATTACTTATGTGGAGAAAACTAGA

Jasper TACATATCAATGCATAATATATACTATAGAGGATAAATTACTTATGTGGAGAAAACTAGA

Joelle phyto TACATATCAATGCATAATATATACTATAGAGGATAAATTACTTATGTGGAGAAAACTAGA

Joelle NCBI TACATATCAATGCATAATATATACTATAGAGGATAAATTACTTATGTGGAGAAAACTAGA

CN 119243 TACATATCAATGCATAATATATACTATAGAGGATAAATTACTTATGTGGAGAAAACTAGA

CN 120025 TACATATCAATGCATAATATATACTATAGAGGATAAATTACTTATGTGGAGAAAACTAGA

Joelle AAFC TACATATCAATGCATAATATATACTATAGAGGATAAATTACTTATGTGGAGAAAACTAGA

**************** ******************************************

CAM 241 ACGCATAT---TAGTATAAAAATTACTTATGTGAAGAAAACTAGAATACATGAATCATCC

17CS1133 ACGCATAT---TAGTATAAAAATTACTTATGTGAAGAAAACTAGAATACATGAATCATCC

CAM 236 ACGCATAT---TAGTATAAAAATTACTTATGTGAAGAAAACTAGAATACATGAATCATCC

Blaine Creek ACGCATAT---TAGTATAAAAATTACTTATGTGAAGAAAACTAGAATACATGAATCATCC

CN 119300 ACGCATAT---TAGTATAAAAATTACTTATGTGAAGAAAACTAGAATACATGAATCATCC

Yellowstone ACGCATAT---TAGTATAAAAATTACTTATGTGAAGAAAACTAGAATACATGAATCATCC

Hoga ACGCATAT---TAGTATAAAAATTACTTATGTGAAGAAAACTAGAATACATGAATCATCC

CN 120027 ACGCATAT---TAGTATAAAAATTACTTATGTGAAGAAAACTAGAATACATGAATCATCC

CN 120030 ACGCATAT---TAGTATAAAAATTACTTATGTGAAGAAAACTAGAATACATGAATCATCC

CN 119294 ACGCATAT---TAGTATAAAAATTACTTATGTGAAGAAAACTAGAATACATGAATCATCC

CN 120013 ACGCATAT---TAGTATAAAAATTACTTATGTGAAGAAAACTAGAATACATGAATCATCC

CN 120017 ACGCATAT---TAGTATAAAAATTACTTATGTGAAGAAAACTAGAATACATGAATCATCC

CN 119205 ACGCATAT---TAGTATAAAAATTACTTATGTGAAGAAAACTAGAATACATGAATCATCC

DH55 ref genome ACGCATAT---TAGTATAAAAATTACTTATGTGAAGAAAACTAGAATACATGAATCATCC

09-CS0040 ACGCATAT---TAGTATAAAAATTACTTATGTGAAGAAAACTAGAATACATGAATCATCC

CN 113754 ACGCATAT---TAGTATAAAAATTACTTATGTGAAGAAAACTAGAATACATGAATCATCC

CO46 NCBI ACGCATAT---TAGTATAAAAATTACTTATGTGAAGAAAACTAGAATACATGAATCATCC

Jasper ACGCATAT---TAGTATAAAAATTACTTATGTGAAGAAAACTAGAATACATGAATCATCC

Joelle phyto ACGCATATTCATAGTATAAAAATTACTTATGTGAAGAAAACTAGAATACATGAATCATCC

Joelle NCBI ACGCATATTCATAGTATAAAAATTACTTATGTGAAGAAAACTAGAATACATGAATCATCC

CN 119243 ACGCATATTCATAGTATAAAAATTACTTATGTGAAGAAAACTAGAATACATGAATCATCC

CN 120025 ACGCATATTCATAGTATAAAAATTACTTATGTGAAGAAAACTAGAATACATGAATCATCC

Joelle AAFC ACGCATATTCATAGTATAAAAATTACTTATGTGAAGAAAACTAGAATACATGAATCATCC

******** *************************************************

CAM 241 TTTTTTTTTTCTTCTAAGAATGCAAATAAAAAACACATCTATCTAATTTCTGGAAATATA

17CS1133 TTTTTTTTTTCTTCTAAGAATGCAAATAAAAAACACATCTATCTAATTTCTGGAAATATA

CAM 236 TTTTTTTTTTCTTCTAAGAATGCAAATAAAAAACACATCTATCTAATTTCTGGAAATATA

Blaine Creek TTTTTTTTTTCTTCTAAGAATGCAAATAAAAAACACATCTATCTAATTTCTGGAAATATA

CN 119300 TTTTTTTTTTCTTCTAAGAATGCAAATAAAAAACACATCTATCTAATTTCTGGAAATATA

Yellowstone TTTTTTTTTTCTTCTAAGAATGCAAATAAAAAACACATCTATCTAATTTCTGGAAATATA

Hoga TTTTTTTTTTCTTCTAAGAATGCAAATAAAAAACACATCTATCTAATTTCTGGAAATATA

CN 120027 TTTTTTTTTTCTTCTAAGAATGCAAATAAAAAACACATCTATCTAATTTCTGGAAATATA

CN 120030 TTTTTTTTTTCTTCTAAGAATGCAAATAAAAAACACATCTATCTAATTTCTGGAAATATA

CN 119294 TTTTTTTTTTCTTCTAAGAATGCAAATAAAAAACACATCTATCTAATTTCTGGAAATATA

CN 120013 TTTTTTTTTTCTTCTAAGAATGCAAATAAAAAACACATCTATCTAATTTCTGGAAATATA

CN 120017 TTTTTTTTTTCTTCTAAGAATGCAAATAAAAAACACATCTATCTAATTTCTGGAAATATA

CN 119205 TTTTTTTTTTCTTCTAAGAATGCAAATAAAAAACACATCTATCTAATTTCTGGAAATATA

DH55 ref genome TTTTTTTTTTCTTCTAAGAATGCAAATAAAAAACACATCTATCTAATTTCTGGAAATATA

09-CS0040 TTTTTTTTTTCTTCTAAGAATGCAAATAAAAAACACATCTATCTAATTTCTGGAAATATA

CN 113754 TTTTTTTTTTCTTCTAAGAATGCAAATAAAAAACACATCTATCTAATTTCTGGAAATATA

CO46 NCBI TTTTTTTTTTCTTCTAAGAATGCAAATAAAAAACACATCTATCTAATTTCTGGAAATATA

Jasper TTTTTTTTTTCTTCTAAGAATGCAAATAAAAAACACATCTATCTAATTTCTGGAAATATA

Joelle phyto TTTTTTTTTTCTTCTAAGAATGCAAATAAAAAACACATCTATCTAATTTCTGGAAATATA

Joelle NCBI TTTTTTTTTTCTTCTAAGAATGCAAATAAAAAACACATCTATCTAATTTCTGGAAATATA

CN 119243 TTTTCTTTTTCTTCTAAGAATGCAAATAAAAAACACATCTATCTAATTTCTGGAAATATA

CN 120025 TTTTCTTTTTCTTCTAAGAATGCAAATAAAAAACACATCTATCTAATTTCTGGAAATATA

Joelle AAFC TTTTCTTTTTCTTCTAAGAATGCAAATAAAAAACACATCTATCTAATTTCTGGAAATATA

**** *******************************************************

CAM 241 TAATTAGGATTTCGAATTTGCAAAAACTTGTGGATATAGG--TTTTTTTTTTTTGGTCAT

17CS1133 TAATTAGGATTTCGAATTTGCAAAAACTTGTGGATATAGG--TTTTTTTTTTTTGGTCAT

CAM 236 TAATTAGGATTTCGAATTTGCAAAAACTTGTGGATATAGG--TTTTTTTTTTTTGGTCAT

Blaine Creek TAATTAGGATTTCGAATTTGCAAAAACTTGTGGATATAGG--TTTTTTTTTTTTGGTCAT

CN 119300 TAATTAGGATTTCGAATTTGCAAAAACTTGTGGATATAGG--TTTTTTTTTTTTGGTCAT

Yellowstone TAATTAGGATTTCGAATTTGCAAAAACTTGTGGATATAGG--TTTTTTTTTTTTGGTCAT

Hoga TAATTAGGATTTCGAATTTGCAAAAACTTGTGGATATAGG--TTTTTTTTTTTTGGTCAT

CN 120027 TAATTAGGATTTCGAATTTGCAAAAACTTGTGGATATAGG--TTTTTTTTTTTTGGTCAT

CN 120030 TAATTAGGATTTCGAATTTGCAAAAACTTGTGGATATAGG--TTTTTTTTTTTTGGTCAT

CN 119294 TAATTAGGATTTCGAATTTGCAAAAACTTGTGGATATAGG--TTTTTTTTTTTTGGTCAT

CN 120013 TAATTAGGATTTCGAATTTGCAAAAACTTGTGGATATAGG--TTTTTTTTTTTTGGTCAT

CN 120017 TAATTAGGATTTCGAATTTGCAAAAACTTGTGGATATAGG--TTTTTTTTTTTTGGTCAT

CN 119205 TAATTAGGATTTCGAATTTGCAAAAACTTGTGGATATAGG--TTTTTTTTTTTTGGTCAT

DH55 ref genome TAATTAGGATTTCGAATTTGCAAAAACTTGTGGATATAGG--TTTTTTTTTTTTGGTCAT

09-CS0040 TAATTAGGATTTCGAATTTGCAAAAACTTGTGGATATAGG--TTTTTTTTTTTTGGTCAT

CN 113754 TAATTAGGATTTCGAATTTGCAAAAACTTGTGGATATAGG--TTTTTTTTTTTTGGTCAT

CO46 NCBI TAATTAGGATTTCGAATTTGCAAAAACTTGTGGATATAGG--TTTTTTTTTTTTGGTCAT

Jasper TAATTAGGATTTCGAATTTGCAAAAACTTGTGGATATAGG--TTTTTTTTTTTTGGTCAT

Joelle phyto TAATTAGGATTTCGAATTTGCAGAAACTTGTGGATATAGG--TTTTTTTTTTTTGGTCAT

Joelle NCBI TAATTAGGATTTCGAATTTGCAGAAACTTGTGGATATAGG--TTTTTTTTTTTTGGTCAT

CN 119243 TAATTAGGATTTCGAATTTGCAAAAACTTGTGGATATAGGTTTTTTTTTTTTTTGGTCAT

CN 120025 TAATTAGGATTTCGAATTTGCAAAAACTTGTGGATATAGGTTTTTTTTTTTTTTGGTCAT

Joelle AAFC TAATTAGGATTTCGAATTTGCAAAAACTTGTGGATATAGGTTTTTTTTTTTTTTGGTCAT

********************** ***************** ******************

CAM 241 ACATCTTGCTTCAATTTTTTAATACCTAAGTATCTGACTTAGTTTTGAACAAATCTTTGA

17CS1133 ACATCTTGCTTCAATTTTTTAATACCTAAGTATCTGACTTAGTTTTGAACAAATCTTTGA

CAM 236 ACATCTTGCTTCAATTTTTTAATACCTAAGTATCTGACTTAGTTTTGAACAAATCTTTGA

Blaine Creek ACATCTTGCTTCAATTTTTTAATACCTAAGTATCTGACTTAGTTTTGAACAAATCTTTGA

CN 119300 ACATCTTGCTTCAATTTTTTAATACCTAAGTATCTGACTTAGTTTTGAACAAATCTTTGA

Yellowstone ACATCTTGCTTCAATTTTTTAATACCTAAGTATCTGACTTAGTTTTGAACAAATCTTTGA

Hoga ACATCTTGCTTCAATTTTTTAATACCTAAGTATCTGACTTAGTTTTGAACAAATCTTTGA

CN 120027 ACATCTTGCTTCAATTTTTTAATACCTAAGTATCTGACTTAGTTTTGAACAAATCTTTGA

CN 120030 ACATCTTGCTTCAATTTTTTAATACCTAAGTATCTGACTTAGTTTTGAACAAATCTTTGA

CN 119294 ACATCTTGCTTCAATTTTTTAATACCTAAGTATCTGACTTAGTTTTGAACAAATCTTTGA

CN 120013 ACATCTTGCTTCAATTTTTTAATACCTAAGTATCTGACTTAGTTTTGAACAAATCTTTGA

CN 120017 ACATCTTGCTTCAATTTTTTAATACCTAAGTATCTGACTTAGTTTTGAACAAATCTTTGA

CN 119205 ACATCTTGCTTCAATTTTTTAATACCTAAGTATCTGACTTAGTTTTGAACAAATCTTTGA

DH55 ref genome ACATCTTGCTTCAATTTTTTAATACCTAAGTATCTGACTTAGTTTTGAACAAATCTTTGA

09-CS0040 ACATCTTGCTTCAATTTTTTAATACCTAAGTATCTGACTTAGTTTTGAACAAATCTTTGA

CN 113754 ACATCTTGCTTCAATTTTTTAATACCTAAGTATCTGACTTAGTTTTGAACAAATCTTTGA

CO46 NCBI ACATCTTGCTTCAATTTTTTAATACCTAAGTATCTGACTTAGTTTTGAACAAATCTTTGA

Jasper ACATCTTGCTTCAATTTTTTAATACCTAAGTATCTGACTTAGTTTTGAACAAATCTTTGA

Joelle phyto ACATCTTGCTTCAATTTTTTAATACCTAAGTATCTGACTTAGTTTTGAACAAATCTTTGA

Joelle NCBI ACATCTTGCTTCAATTTTTTAATACCTAAGTATCTGACTTAGTTTTGAACAAATCTTTGA

CN 119243 ACATCTTGCTTCAATTTTTTAATACCTAAGTATCTGACTTAGTTTTGAACAAATCTTTGA

CN 120025 ACATCTTGCTTCAATTTTTTAATACCTAAGTATCTGACTTAGTTTTGAACAAATCTTTGA

Joelle AAFC ACATCTTGCTTCAATTTTTTAATACCTAAGTATCTGACTTAGTTTTGAACAAATCTTTGA

************************************************************

CAM 241 TTAATTTCATCTGATGTTTGTTTATAAATGAAGAATTAAAAATTGATGAAGAGGTATATA

17CS1133 TTAATTTCATCTGATGTTTGTTTATAAATGAAGAATTAAAAATTGATGAAGAGGTATATA

CAM 236 TTAATTTCATCTGATGTTTGTTTATAAATGAAGAATTAAAAATTGATGAAGAGGTATATA

Blaine Creek TTAATTTCATCTGATGTTTGTTTATAAATGAAGAATTAAAAATTGATGAAGAGGTATATA

CN 119300 TTAATTTCATCTGATGTTTGTTTATAAATGAAGAATTAAAAATTGATGAAGAGGTATATA

Yellowstone TTAATTTCATCTGATGTTTGTTTATAAATGAAGAATTAAAAATTGATGAAGAGGTATATA

Hoga TTAATTTCATCTGATGTTTGTTTATAAATGAAGAATTAAAAATTGATGAAGAGGTATATA

CN 120027 TTAATTTCATCTGATGTTTGTTTATAAATGAAGAATTAAAAATTGATGAAGAGGTATATA

CN 120030 TTAATTTCATCTGATGTTTGTTTATAAATGAAGAATTAAAAATTGATGAAGAGGTATATA

CN 119294 TTAATTTCATCTGATGTTTGTTTATAAATGAAGAATTAAAAATTGATGAAGAGGTATATA

CN 120013 TTAATTTCATCTGATGTTTGTTTATAAATGAAGAATTAAAAATTGATGAAGAGGTATATA

CN 120017 TTAATTTCATCTGATGTTTGTTTATAAATGAAGAATTAAAAATTGATGAAGAGGTATATA

CN 119205 TTAATTTCATCTGATGTTTGTTTATAAATGAAGAATTAAAAATTGATGAAGAGGTATATA

DH55 ref genome TTAATTTCATCTGATGTTTGTTTATAAATGAAGAATTAAAAATTGATGAAGAGGTATATA

09-CS0040 TTAATTTCATCTGATGTTTGTTTATAAATGAAGAATTAAAAATTGATGAAGAGGTATATA

CN 113754 TTAATTTCATCTGATGTTTGTTTATAAATGAAGAATTAAAAATTGATGAAGAGGTATATA

CO46 NCBI TTAATTTCATCTGATGTTTGTTTATAAATGAAGAATTAAAAATTGATGAAGAGGTATATA

Jasper TTAATTTCATCTGATGTTTGTTTATAAATGAAGAATTAAAAATTGATGAAGAGGTATATA

Joelle phyto TTAATTTCATCTGATGTTTGTTTATAAATGAAGAATTAAAAATTGATGAAGAGGTATATA

Joelle NCBI TTAATTTCATCTGATGTTTGTTTATAAATGAAGAATTAAAAATTGATGAAGAGGTATATA

CN 119243 TTAATTTCATCTGATGTTTGTTTATAAATGAAGAATTAAAAATTGATGAAGAGGTATATA

CN 120025 TTAATTTCATCTGATGTTTGTTTATAAATGAAGAATTAAAAATTGATGAAGAGGTATATA

Joelle AAFC TTAATTTCATCTGATGTTTGTTTATAAATGAAGAATTAAAAATTGATGAAGAGGTATATA

************************************************************

CAM 241 TATATTTTTTTTTAATAAAAAATCAAAGAGTCATTAGAGCAAACGCGGTCGCATGATATT

17CS1133 TATATTTTTTTTTAATAAAAAATCAAAGAGTCATTAGAGCAAACGCGGTCGCATGATATT

CAM 236 TATATTTTTTTTTAATAAAAAATCAAAGAGTCATTAGAGCAAACGCGGTCGCATGATATT

Blaine Creek TATATTTTTTTTTAATAAAAAATCAAAGAGTCATTAGAGCAAACGCGGTCGCATGATATT

CN 119300 TATATTTTTTTTTAATAAAAAATCAAAGAGTCATTAGAGCAAACGCGGTCGCATGATATT

Yellowstone TATATTTTTTTTTAATAAAAAATCAAAGAGTCATTAGAGCAAACGCGGTCGCATGATATT

Hoga TATATTTTTTTTTAATAAAAAATCAAAGAGTCATTAGAGCAAACGCGGTCGCATGATATT

CN 120027 TATATTTTTTTTTAATAAAAAATCAAAGAGTCATTAGAGCAAACGCGGTCGCATGATATT

CN 120030 TATATTTTTTTTTAATAAAAAATCAAAGAGTCATTAGAGCAAACGCGGTCGCATGATATT

CN 119294 TATATTTTTTTTTAATAAAAAATCAAAGAGTCATTAGAGCAAACGCGGTCGCATGATATT

CN 120013 TATATTTTTTTTTAATAAAAAATCAAAGAGTCATTAGAGCAAACGCGGTCGCATGATATT

CN 120017 TATATTTTTTTTTAATAAAAAATCAAAGAGTCATTAGAGCAAACGCGGTCGCATGATATT

CN 119205 TATATTTTTTTTTAATAAAAAATCAAAGAGTCATTAGAGCAAACGCGGTCGCATGATATT

DH55 ref genome TATATTTTTTTTTAATAAAAAATCAAAGAGTCATTAGAGCAAACGCGGTCGCATGATATT

09-CS0040 TATATTTTTTTTTAATAAAAAATCAAAGAGTCATTAGAGCAAACGCGGTCGCATGATATT

CN 113754 TATATTTTTTTTTAATAAAAAATCAAAGAGTCATTAGAGCAAACGCGGTCGCATGATATT

CO46 NCBI TATATTTTTTTTTAATAAAAAATCAAAGAGTCATTAGAGCAAACGCGGTCGCATGATATT

Jasper TATATTTTTTTTTAATAAAAAATCAAAGAGTCATTAGAGCAAACGCGGTCGCATGATATT

Joelle phyto TATAT-TTTTTTTAATAAAAAATCAAAGAGTCATTAGAGCAAACGCGGTCGCATGATATT

Joelle NCBI TATAT-TTTTTTTAATAAAAAATCAAAGAGTCATTAGAGCAAACGCGGTCGCATGATATT

CN 119243 TATATATTTTTTTAATAAAAAATCAAAGAGTCATTAGAGCAAACGCGGTCGCATGATATT

CN 120025 TATATATTTTTTTAATAAAAAATCAAAGAGTCATTAGAGCAAACGCGGTCGCATGATATT

Joelle AAFC TATATATTTTTTTAATAAAAAATCAAAGAGTCATTAGAGCAAACGCGGTCGCATGATATT

***** ******************************************************

CAM 241 CGTCATGCGGGACACGTGGCAATGTTGCGTTCAAAACGCAACGTTTGTAATCATAAATTT

17CS1133 CGTCATGCGGGACACGTGGCAATGTTGCGTTCAAAACGCAACGTTTGTAATCATAAATTT

CAM 236 CGTCATGCGGGACACGTGGCAATGTTGCGTTCAAAACGCAACGTTTGTAATCATAAATTT

Blaine Creek CGTCATGCGGGACACGTGGCAATGTTGCGTTCAAAACGCAACGTTTGTAATCATAAATTT

CN 119300 CGTCATGCGGGACACGTGGCAATGTTGCGTTCAAAACGCAACGTTTGTAATCATAAATTT

Yellowstone CGTCATGCGGGACACGTGGCAATGTTGCGTTCAAAACGCAACGTTTGTAATCATAAATTT

Hoga CGTCATGCGGGACACGTGGCAATGTTGCGTTCAAAACGCAACGTTTGTAATCATAAATTT

CN 120027 CGTCATGCGGGACACGTGGCAATGTTGCGTTCAAAACGCAACGTTTGTAATCATAAATTT

CN 120030 CGTCATGCGGGACACGTGGCAATGTTGCGTTCAAAACGCAACGTTTGTAATCATAAATTT

CN 119294 CGTCATGCGGGACACGTGGCAATGTTGCGTTCAAAACGCAACGTTTGTAATCATAAATTT

CN 120013 CGTCATGCGGGACACGTGGCAATGTTGCGTTCAAAACGCAACGTTTGTAATCATAAATTT

CN 120017 CGTCATGCGGGACACGTGGCAATGTTGCGTTCAAAACGCAACGTTTGTAATCATAAATTT

CN 119205 CGTCATGCGGGACACGTGGCAATGTTGCGTTCAAAACGCAACGTTTGTAATCATAAATTT

DH55 ref genome CGTCATGCGGGACACGTGGCAATGTTGCGTTCAAAACGCAACGTTTGTAATCATAAATTT

09-CS0040 CGTCATGCGGGACACGTGGCAATGTTGCGTTCAAAACGCAACGTTTGTAATCATAAATTT

CN 113754 CGTCATGCGGGACACGTGGCAATGTTGCGTTCAAAACGCAACGTTTGTAATCATAAATTT

CO46 NCBI CGTCATGCGGGACACGTGGCAATGTTGCGTTCAAAACGCAACGTTTGTAATCATAAATTT

Jasper CGTCATGCGGGACACGTGGCAATGTTGCGTTCAAAACGCAACGTTTGTAATCATAAATTT

Joelle phyto CGTCATGCGGGACACGTGGCAATGTTGCGTTCAAAACGCAACGTTTGTAATCATAAATTT

Joelle NCBI CGTCATGCGGGACACGTGGCAATGTTGCGTTCAAAACGCAACGTTTGTAATCATAAATTT

CN 119243 CGTCATGCGGGACACGTGGCAATGTTGCGTGCAAAACACAACGTTTGTAATCATAAATTT

CN 120025 CGTCATGCGGGACACGTGGCAATGTTGCGTGCAAAACGCAACGTTTGTAATCATAAATTT

Joelle AAFC CGTCATGCGGGACACGTGGCAATGTTGCGTGCAAAACGCAACGTTTGTAATCATAAATTT

****************************** ****** **********************

CAM 241 TATTTTCATCACTCTCGTTTACCCC-AAAAAAAAAAAAATCTAGCCCGAGGAAGAAAAAA

17CS1133 TATTTTCATCACTCTCGTTTACCCC-AAAAAAAAAAAAATCTAGCCCGAGGAAGAAAAAA

CAM 236 TATTTTCATCACTCTCGTTTACCCC-AAAAAAAAAAAAATCTAGCCCGAGGAAGAAAAAA

Blaine Creek TATTTTCATCACTCTCGTTTACCCC-AAAAAAAAAAAAATCTAGCCCGAGGAAGAAAAAA

CN 119300 TATTTTCATCACTCTCGTTTACCCC-AAAAAAAAAAAAATCTAGCCCGAGGAAGAAAAAA

Yellowstone TATTTTCATCACTCTCGTTTACCCC-AAAAAAAAAAAAATCTAGCCCGAGGAAGAAAAAA

Hoga TATTTTCATCACTCTCGTTTACCCC-AAAAAAAAAAAAATCTAGCCCGAGGAAGAAAAAA

CN 120027 TATTTTCATCACTCTCGTTTACCCC-AAAAAAAAAAAAATCTAGCCCGAGGAAGAAAAAA

CN 120030 TATTTTCATCACTCTCGTTTACCCC-AAAAAAAAAAAAATCTAGCCCGAGGAAGAAAAAA

CN 119294 TATTTTCATCACTCTCGTTTACCCC-AAAAAAAAAAAAATCTAGCCCGAGGAAGAAAAAA

CN 120013 TATTTTCATCACTCTCGTTTACCCC-AAAAAAAAAAAAATCTAGCCCGAGGAAGAAAAAA

CN 120017 TATTTTCATCACTCTCGTTTACCCC-AAAAAAAAAAAAATCTAGCCCGAGGAAGAAAAAA

CN 119205 TATTTTCATCACTCTCGTTTACCCC-AAAAAAAAAAAAATCTAGCCCGAGGAAGAAAAAA

DH55 ref genome TATTTTCATCACTCTCGTTTACCCC-AAAAAAAAAAAAATCTAGCCCGAGGAAGAAAAAA

09-CS0040 TATTTTCATCACTCTCGTTTACCCC-AAAAAAAAAAAAATCTAGCCCGAGGAAGAAAAAA

CN 113754 TATTTTCATCACTCTCGTTTACCCC-AAAAAAAAAAAAATCTAGCCCGAGGAAGAAAAAA

CO46 NCBI TATTTTCATCACTCTCGTTTACCCC-AAAAAAAAAAAAATCTAGCCCGAGGAAGAAAAAA

Jasper TATTTTCATCACTCTCGTTTACCCC-AAAAAAAAAAAAATCTAGCCCGAGGAAGAAAAAA

Joelle phyto TATTTTCATCACTCTCGTTTACCCC-AAAAAAAAAAAAATCTAGCCCGAGGAAGAAAAAA

Joelle NCBI TATTTTCATCACTCTCGTTTACCCC-AAAAAAAAAAAAATCTAGCCCGAGGAAGAAAAAA

CN 119243 TATTTTCATCACTCTCGTTTACCCCAAAAAAAAAAAAAATCTAGCCCGAGGAAGAAAAAA

CN 120025 TATTTTCATCACTCTCGTTTACCCC-AAAAAAAAAAAAATCTAGCCCGAGGAAGAAAAAA

Joelle AAFC TATTTTCATCACTCTCGTTTACCCC-AAAAAAAAAAAAATCTAGCCCGAGGAAGAAAAAA

************************* **********************************

CAM 241 AAAATTAGATTAGACAAAAAAATAGAAAGAAATAAAAGGAAAAAAGGAAATAAAAAAATA

17CS1133 AAAATTAGATTAGACAAAAAAATAGAAAGAAATAAAAGGAAAAAAGGAAATAAAAAAATA

CAM 236 AAAATTAGATTAGACAAAAAAATAGAAAGAAATAAAAGGAAAAAAGGAAATAAAAAAATA

Blaine Creek AAAATTAGATTAGACAAAAAAATAGAAAGAAATAAAAGGAAAAAAGGAAATAAAAAAATA

CN 119300 AAAATTAGATTAGACAAAAAAATAGAAAGAAATAAAAGGAAAAAAGGAAATAAAAAAATA

Yellowstone AAAATTAGATTAGACAAAAAAATAGAAAGAAATAAAAGGAAAAAAGGAAATAAAAAAATA

Hoga AAAATTAGATTAGACAAAAAAATAGAAAGAAATAAAAGGAAAAAAGGAAATAAAAAAATA

CN 120027 AAAATTAGATTAGACAAAAAAATAGAAAGAAATAAAAGGAAAAAAGGAAATAAAAAAATA

CN 120030 AAAATTAGATTAGACAAAAAAATAGAAAGAAATAAAAGGAAAAAAGGAAATAAAAAAATA

CN 119294 AAAATTAGATTAGACAAAAAAATAGAAAGAAATAAAAGGAAAAAAGGAAATAAAAAAATA

CN 120013 AAAATTAGATTAGACAAAAAAATAGAAAGAAATAAAAGGAAAAAAGGAAATAAAAAAATA

CN 120017 AAAATTAGATTAGACAAAAAAATAGAAAGAAATAAAAGGAAAAAAGGAAATAAAAAAATA

CN 119205 AAAATTAGATTAGACAAAAAAATAGAAAGAAATAAAAGGAAAAAAGGAAATAAAAAAATA

DH55 ref genome AAAATTAGATTAGACAAAAAAATAGAAAGAAATAAAAGGAAAAAAGGAAATAAAAAAATA

09-CS0040 AAAATTAGATTAGACAAAAAAATAGAAAGAAATAAAAGGAAAAAAGGAAATAAAAAAATA

CN 113754 AAAATTAGATTAGACAAAAAAATAGAAAGAAATAAAAGGAAAAAAGGAAATAAAAAAATA

CO46 NCBI AAAATTAGATTAGACAAAAAAATAGAAAGAAATAAAAGGAAAAAAGGAAATAAAAAAATA

Jasper AAAATTAGATTAGACAAAAAAATAGAAAGAAATAAAAGGAAAAAAGGAAATAAAAAAATA

Joelle phyto AAAATTAGATTAGACAAAAAAATAGAAAGAAATAAAAGGAAAAAAGGAAATAAAAAAATA

Joelle NCBI AAAATTAGATTAGACAAAAAAATAGAAAGAAATAAAAGGAAAAAAGGAAATAAAAAAATA

CN 119243 AAAA-TAGATTAGACAAAAAAATAGAAAGAAATAAAAGGAAAAAAGGAAATAAAAAAATA

CN 120025 AAAA-TAGATTAGACAAAAAAATAGAAAGAAATAAAAGGAAAAAAGGAAATAAAAAAATA

Joelle AAFC AAAA-TAGATTAGACAAAAAAATAGAAAGAAATAAAAGGAAAAAAGGAAATAAAAAAATA

**** *******************************************************

CAM 241 GGAAAGGCAAAAAAAACGCTTAGCATCTCTCCGGCGACTTGAACCCAAACCTGAGGATCA

17CS1133 GGAAAGGCAAAAAAAACGCTTAGCATCTCTCCGGCGACTTGAACCCAAACCTGAGGATCA

CAM 236 GGAAAGGCAAAAAAAACGCTTAGCATCTCTCCGGCGACTTGAACCCAAACCTGAGGATCA

Blaine Creek GGAAAGGCAAAAAAAACGCTTAGCATCTCTCCGGCGACTTGAACCCAAACCTGAGGATCA

CN 119300 GGAAAGGCAAAAAAAACGCTTAGCATCTCTCCGGCGACTTGAACCCAAACCTGAGGATCA

Yellowstone GGAAAGGCAAAAAAAACGCTTAGCATCTCTCCGGCGACTTGAACCCAAACCTGAGGATCA

Hoga GGAAAGGCAAAAAAAACGCTTAGCATCTCTCCGGCGACTTGAACCCAAACCTGAGGATCA

CN 120027 GGAAAGGCAAAAAAAACGCTTAGCATCTCTCCGGCGACTTGAACCCAAACCTGAGGATCA

CN 120030 GGAAAGGCAAAAAAAACGCTTAGCATCTCTCCGGCGACTTGAACCCAAACCTGAGGATCA

CN 119294 GGAAAGGCAAAAAAAACGCTTAGCATCTCTCCGGCGACTTGAACCCAAACCTGAGGATCA

CN 120013 GGAAAGGCAAAAAAAACGCTTAGCATCTCTCCGGCGACTTGAACCCAAACCTGAGGATCA

CN 120017 GGAAAGGCAAAAAAAACGCTTAGCATCTCTCCGGCGACTTGAACCCAAACCTGAGGATCA

CN 119205 GGAAAGGCAAAAAAAACGCTTAGCATCTCTCCGGCGACTTGAACCCAAACCTGAGGATCA

DH55 ref genome GGAAAGGCAAAAAAAACGCTTAGCATCTCTCCGGCGACTTGAACCCAAACCTGAGGATCA

09-CS0040 GGAAAGGCAAAAAAAACGCTTAGCATCTCTCCGGCGACTTGAACCCAAACCTGAGGATCA

CN 113754 GGAAAGGCAAAAAAAACGCTTAGCATCTCTCCGGCGACTTGAACCCAAACCTGAGGATCA

CO46 NCBI GGAAAGGCAAAAAAAACGCTTAGCATCTCTCCGGCGACTTGAACCCAAACCTGAGGATCA

Jasper GGAAAGGCAAAAAAAACGCTTAGCATCTCTCCGGCGACTTGAACCCAAACCTGAGGATCA

Joelle phyto GGAAAGGCAAAAAAAACGCTTAGCATCTCTCCGGCGACTTGAACCCAAACCTGAGGATCA

Joelle NCBI GGAAAGGCAAAAAAAACGCTTAGCATCTCTCCGGCGACTTGAACCCAAACCTGAGGATCA

CN 119243 GGAAAGGCAAAAAAAACGCTTAGCATCTCTCCGGCGACTTGAACCCAAACCTGAGGATCA

CN 120025 GGAAAGGCAAAAAAAACGCTTAGCATCTCTCCGGCGACTTGAACCCAAACCTGAGGATCA

Joelle AAFC GGAAAGGCAAAAAAAACGCTTAGCATCTCTCCGGCGACTTGAACCCAAACCTGAGGATCA

************************************************************

CAM 241 AATTAGGGCACAAGGGACTCTCGGAGACTGAAGCCATGGGAAGGAAAAAACTAGAAATCA

17CS1133 AATTAGGGCACAAGGGACTCTCGGAGACTGAAGCCATGGGAAGGAAAAAACTAGAAATCA

CAM 236 AATTAGGGCACAAGGGACTCTCGGAGACTGAAGCCATGGGAAGGAAAAAACTAGAAATCA

Blaine Creek AATTAGGGCACAAGGGACTCTCGGAGACTGAAGCCATGGGAAGGAAAAAACTAGAAATCA

CN 119300 AATTAGGGCACAAGGGACTCTCGGAGACTGAAGCCATGGGAAGGAAAAAACTAGAAATCA

Yellowstone AATTAGGGCACAAGGGACTCTCGGAGACTGAAGCCATGGGAAGGAAAAAACTAGAAATCA

Hoga AATTAGGGCACAAGGGACTCTCGGAGACTGAAGCCATGGGAAGGAAAAAACTAGAAATCA

CN 120027 AATTAGGGCACAAGGGACTCTCGGAGACTGAAGCCATGGGAAGGAAAAAACTAGAAATCA

CN 120030 AATTAGGGCACAAGGGACTCTCGGAGACTGAAGCCATGGGAAGGAAAAAACTAGAAATCA

CN 119294 AATTAGGGCACAAGGGACTCTCGGAGACTGAAGCCATGGGAAGGAAAAAACTAGAAATCA

CN 120013 AATTAGGGCACAAGGGACTCTCGGAGACTGAAGCCATGGGAAGGAAAAAACTAGAAATCA

CN 120017 AATTAGGGCACAAGGGACTCTCGGAGACTGAAGCCATGGGAAGGAAAAAACTAGAAATCA

CN 119205 AATTAGGGCACAAGGGACTCTCGGAGACTGAAGCCATGGGAAGGAAAAAACTAGAAATCA

DH55 ref genome AATTAGGGCACAAGGGACTCTCGGAGACTGAAGCCATGGGAAGGAAAAAACTAGAAATCA

09-CS0040 AATTAGGGCACAAGGGACTCTCGGAGACTGAAGCCATGGGAAGGAAAAAACTAGAAATCA

CN 113754 AATTAGGGCACAAGGGACTCTCGGAGACTGAAGCCATGGGAAGGAAAAAACTAGAAATCA

CO46 NCBI AATTAGGGCACAAGGGACTCTCGGAGACTGAAGCCATGGGAAGGAAAAAACTAGAAATCA

Jasper AATTAGGGCACAAGGGACTCTCGGAGACTGAAGCCATGGGAAGGAAAAAACTAGAAATCA

Joelle phyto AATTAGGGCACAAGGGACTCTCGGAGACTGAAGCCATGGGAAGGAAAAAACTAGAAATCA

Joelle NCBI AATTAGGGCACAAGGGACTCTCGGAGACTGAAGCCATGGGAAGGAAAAAACTAGAAATCA

CN 119243 AATTAGGGCACAAGGGACTCTCGGAGACTGAAGCCATGGGAAGGAAAAAACTAGAAATCA

CN 120025 AATTAGGGCACAAGGGACTCTCGGAGACTGAAGCCATGGGAAGGAAAAAACTAGAAATCA

Joelle AAFC AATTAGGGCACAAGGGACTCTCGGAGACTGAAGCCATGGGAAGGAAAAAACTAGAAATCA

************************************************************

CAM 241 AGCGAATTGAGAACAAAAGTAGCCGACAAGTCACCTTCTCCAAACGTCGCAATGGTCTCA

17CS1133 AGCGAATTGAGAACAAAAGTAGCCGACAAGTCACCTTCTCCAAACGTCGCAATGGTCTCA

CAM 236 AGCGAATTGAGAACAAAAGTAGCCGACAAGTCACCTTCTCCAAACGTCGCAATGGTCTCA

Blaine Creek AGCGAATTGAGAACAAAAGTAGCCGACAAGTCACCTTCTCCAAACGTCGCAATGGTCTCA

CN 119300 AGCGAATTGAGAACAAAAGTAGCCGACAAGTCACCTTCTCCAAACGTCGCAATGGTCTCA

Yellowstone AGCGAATTGAGAACAAAAGTAGCCGACAAGTCACCTTCTCCAAACGTCGCAATGGTCTCA

Hoga AGCGAATTGAGAACAAAAGTAGCCGACAAGTCACCTTCTCCAAACGTCGCAATGGTCTCA

CN 120027 AGCGAATTGAGAACAAAAGTAGCCGACAAGTCACCTTCTCCAAACGTCGCAATGGTCTCA

CN 120030 AGCGAATTGAGAACAAAAGTAGCCGACAAGTCACCTTCTCCAAACGTCGCAATGGTCTCA

CN 119294 AGCGAATTGAGAACAAAAGTAGCCGACAAGTCACCTTCTCCAAACGTCGCAATGGTCTCA

CN 120013 AGCGAATTGAGAACAAAAGTAGCCGACAAGTCACCTTCTCCAAACGTCGCAATGGTCTCA

CN 120017 AGCGAATTGAGAACAAAAGTAGCCGACAAGTCACCTTCTCCAAACGTCGCAATGGTCTCA

CN 119205 AGCGAATTGAGAACAAAAGTAGCCGACAAGTCACCTTCTCCAAACGTCGCAATGGTCTCA

DH55 ref genome AGCGAATTGAGAACAAAAGTAGCCGACAAGTCACCTTCTCCAAACGTCGCAATGGTCTCA

09-CS0040 AGCGAATTGAGAACAAAAGTAGCCGACAAGTCACCTTCTCCAAACGTCGCAATGGTCTCA

CN 113754 AGCGAATTGAGAACAAAAGTAGCCGACAAGTCACCTTCTCCAAACGTCGCAATGGTCTCA

CO46 NCBI AGCGAATTGAGAACAAAAGTAGCCGACAAGTCACCTTCTCCAAACGTCGCAATGGTCTCA

Jasper AGCGAATTGAGAACAAAAGTAGCCGACAAGTCACCTTCTCCAAACGTCGCAATGGTCTCA

Joelle phyto AGCGAATTGAGAACAAAAGTAGCCGACAAGTCACCTTCTCCAAACGTCGCAATGGTCTCA

Joelle NCBI AGCGAATTGAGAACAAAAGTAGCCGACAAGTCACCTTCTCCAAACGTCGCAATGGTCTCA

CN 119243 AGCGAATTGAGAACAAAAGTAGCCGACAAGTCACCTTCTCCAAACGTCGCAATGGTCTCA

CN 120025 AGCGAATTGAGAACAAAAGTAGCCGACAAGTCACCTTCTCCAAACGTCGCAATGGTCTCA

Joelle AAFC AGCGAATTGAGAACAAAAGTAGCCGACAAGTCACCTTCTCCAAACGTCGCAATGGTCTCA

************************************************************

CAM 241 TCGAGAAAGCTCGTCAGCTTTCTGTTCTCTGTGACGCATCTGTCGCTCTTCTCGTCGTCT

17CS1133 TCGAGAAAGCTCGTCAGCTTTCTGTTCTCTGTGACGCATCTGTCGCTCTTCTCGTCGTCT

CAM 236 TCGAGAAAGCTCGTCAGCTTTCTGTTCTCTGTGACGCATCTGTCGCTCTTCTCGTCGTCT

Blaine Creek TCGAGAAAGCTCGTCAGCTTTCTGTTCTCTGTGACGCATCTGTCGCTCTTCTCGTCGTCT

CN 119300 TCGAGAAAGCTCGTCAGCTTTCTGTTCTCTGTGACGCATCTGTCGCTCTTCTCGTCGTCT

Yellowstone TCGAGAAAGCTCGTCAGCTTTCTGTTCTCTGTGACGCATCTGTCGCTCTTCTCGTCGTCT

Hoga TCGAGAAAGCTCGTCAGCTTTCTGTTCTCTGTGACGCATCTGTCGCTCTTCTCGTCGTCT

CN 120027 TCGAGAAAGCTCGTCAGCTTTCTGTTCTCTGTGACGCATCTGTCGCTCTTCTCGTCGTCT

CN 120030 TCGAGAAAGCTCGTCAGCTTTCTGTTCTCTGTGACGCATCTGTCGCTCTTCTCGTCGTCT

CN 119294 TCGAGAAAGCTCGTCAGCTTTCTGTTCTCTGTGACGCATCTGTCGCTCTTCTCGTCGTCT

CN 120013 TCGAGAAAGCTCGTCAGCTTTCTGTTCTCTGTGACGCATCTGTCGCTCTTCTCGTCGTCT

CN 120017 TCGAGAAAGCTCGTCAGCTTTCTGTTCTCTGTGACGCATCTGTCGCTCTTCTCGTCGTCT

CN 119205 TCGAGAAAGCTCGTCAGCTTTCTGTTCTCTGTGACGCATCTGTCGCTCTTCTCGTCGTCT

DH55 ref genome TCGAGAAAGCTCGTCAGCTTTCTGTTCTCTGTGACGCATCCGTCGCTCTTCTCGTCGTCT

09-CS0040 TCGAGAAAGCTCGTCAGCTTTCTGTTCTCTGTGACGCATCTGTCGCTCTTCTCGTCGTCT

CN 113754 TCGAGAAAGCTCGTCAGCTTTCTGTTCTCTGTGACGCATCTGTCGCTCTTCTCGTCGTCT

CO46 NCBI TCGAGAAAGCTCGTCAGCTTTCTGTTCTCTGTGACGCATCTGTCGCTCTTCTCGTCGTCT

Jasper TCGAGAAAGCTCGTCAGCTTTCTGTTCTCTGTGACGCATCTGTCGCTCTTCTCGTCGTCT

Joelle phyto TCGAGAAAGCTCGTCAGCTTTCTGTTCTCTGTGACGCATCTGTCGCTCTTCTCGTCGTCT

Joelle NCBI TCGAGAAAGCTCGTCAGCTTTCTGTTCTCTGTGACGCATCTGTCGCTCTTCTCGTCGTCT

CN 119243 TCGAGAAAGCTCGTCAGCTTTCTGTTCTCTGTGACGCATCTGTCGCTCTTCTCGTCGTCT

CN 120025 TCGAGAAAGCTCGTCAGCTTTCTGTTCTCTGTGACGCATCTGTCGCTCTTCTCGTCGTCT

Joelle AAFC TCGAGAAAGCTCGTCAGCTTTCTGTTCTCTGTGACGCATCTGTCGCTCTTCTCGTCGTCT

**************************************** *******************

CAM 241 CCGCCTCCGGCAAGCTCTACAGCTTCTCCTCCGGTGATAAGTACGTCTTTTCCTTATCTG

17CS1133 CCGCCTCCGGCAAGCTCTACAGCTTCTCCTCCGGTGATAAGTACGTCTTTTCCTTATCTG

CAM 236 CCGCCTCCGGCAAGCTCTACAGCTTCTCCTCCGGTGATAAGTACGTCTTTTCCTTATCTG

Blaine Creek CCGCCTCCGGCAAGCTCTACAGCTTCTCCTCCGGTGATAAGTACGTCTTTTCCTTATCTG

CN 119300 CCGCCTCCGGCAAGCTCTACAGCTTCTCCTCCGGTGATAAGTACGTCTTTTCCTTATCTG

Yellowstone CCGCCTCCGGCAAGCTCTACAGCTTCTCCTCCGGTGATAAGTACGTCTTTTCCTTATCTG

Hoga CCGCCTCCGGCAAGCTCTACAGCTTCTCCTCCGGTGATAAGTACGTCTTTTCCTTATCTG

CN 120027 CCGCCTCCGGCAAGCTCTACAGCTTCTCCTCCGGTGATAAGTACGTCTTTTCCTTATCTG

CN 120030 CCGCCTCCGGCAAGCTCTACAGCTTCTCCTCCGGTGATAAGTACGTCTTTTCCTTATCTG

CN 119294 CCGCCTCCGGCAAGCTCTACAGCTTCTCCTCCGGTGATAAGTACGTCTTTTCCTTATCTG

CN 120013 CCGCCTCCGGCAAGCTCTACAGCTTCTCCTCCGGTGATAAGTACGTCTTTTCCTTATCTG

CN 120017 CCGCCTCCGGCAAGCTCTACAGCTTCTCCTCCGGTGATAAGTACGTCTTTTCCTTATCTG

CN 119205 CCGCCTCCGGCAAGCTCTACAGCTTCTCCTCCGGTGATAAGTACGTCTTTTCCTTATCTG

DH55 ref genome CCGCCTCCGGCAAGCTCTACAGCTTCTCCTCCGGTGATAAGTACGTCTTTTCCTTATCTG

09-CS0040 CCGCCTCCGGCAAGCTCTACAGCTTCTCCTCCGGTGATAAGTACGTCTTTTCCTTATCTG

CN 113754 CCGCCTCCGGCAAGCTCTACAGCTTCTCCTCCGGTGATAAGTACGTCTTTTCCTTATCTG

CO46 NCBI CCGCCTCCGGCAAGCTCTACAGCTTCTCCTCCGGTGATAAGTACGTCTTTTCCTTATCTG

Jasper CCGCCTCCGGCAAGCTCTACAGCTTCTCCTCCGGTGATAAGTACGTCTTTTCCTTATCTG

Joelle phyto CCGCCTCCGGCAAGCTCTACAGCTTCTCCTCCGGTGATAAGTACGTCTTTTCCTTATCTG

Joelle NCBI CCGCCTCCGGCAAGCTCTACAGCTTCTCCTCCGGTGATAAGTACGTCTTTTCCTTATCTG

CN 119243 CCGCCTCCGGCAAGCTCTACAGCTTCTCCTCCGGTGATAAGTACGTCTTTTCCTTATCTG

CN 120025 CCGCCTCCGGCAAGCTCTACAGCTTCTCCTCCGGTGATAAGTACGTCTTTTCCTTATCTG

Joelle AAFC CCGCCTCCGGCAAGCTCTACAGCTTCTCCTCCGGTGATAAGTACGTCTTTTCCTTATCTG

************************************************************

CAM 241 GGTTCTTCCCCCTTTAAGCTTCGGTTTTGTGCTTTCTCTTTACTTTTTCTCTGAAGAAAA

17CS1133 GGTTCTTCCCCCTTTAAGCTTCGGTTTTGTGCTTTCTCTTTACTTTTTCTCTGAAGAAAA

CAM 236 GGTTCTTCCCCCTTTAAGCTTCGGTTTTGTGCTTTCTCTTTACTTTTTCTCTGAAGAAAA

Blaine Creek GGTTCTTCCCCCTTTAAGCTTCGGTTTTGTGCTTTCTCTTTACTTTTTCTCTGAAGAAAA

CN 119300 GGTTCTTCCCCCTTTAAGCTTCGGTTTTGTGCTTTCTCTTTACTTTTTCTCTGAAGAAAA

Yellowstone GGTTCTTCCCCCTTTAAGCTTCGGTTTTGTGCTTTCTCTTTACTTTTTCTCTGAAGAAAA

Hoga GGTTCTTCCCCCTTTAAGCTTCGGTTTTGTGCTTTCTCTTTACTTTTTCTCTGAAGAAAA

CN 120027 GGTTCTTCCCCCTTTAAGCTTCGGTTTTGTGCTTTCTCTTTACTTTTTCTCTGAAGAAAA

CN 120030 GGTTCTTCCCCCTTTAAGCTTCGGTTTTGTGCTTTCTCTTTACTTTTTCTCTGAAGAAAA

CN 119294 GGTTCTTCCCCCTTTAAGCTTCGGTTTTGTGCTTTCTCTTTACTTTTTCTCTGAAGAAAA

CN 120013 GGTTCTTCCCCCTTTAAGCTTCGGTTTTGTGCTTTCTCTTTACTTTTTCTCTGAAGAAAA

CN 120017 GGTTCTTCCCCCTTTAAGCTTCGGTTTTGTGCTTTCTCTTTACTTTTTCTCTGAAGAAAA

CN 119205 GGTTCTTCCCCCTTTAAGCTTCGGTTTTGTGCTTTCTCTTTACTTTTTCTCTGAAGAAAA

DH55 ref genome GGTTCTTCCCCCTTTAAGCTTCGGTTTTGTGCTTTCTCTTTACTTTTTCTCTGAAGAAAA

09-CS0040 GGTTCTTCCCCCTTTAAGCTTCGGTTTTGTGCTTTCTCTTTACTTTTTCTCTGAAGAAAA

CN 113754 GGTTCTTCCCCCTTTAAGCTTCGGTTTTGTGCTTTCTCTTTACTTTTTCTCTGAAGAAAA

CO46 NCBI GGTTCTTCCCCCTTTAAGCTTCGGTTTTGTGCTTTCTCTTTACTTTTTCTCTGAAGAAAA

Jasper GGTTCTTCCCCCTTTAAGCTTCGGTTTTGTGCTTTCTCTTTACTTTTTCTCTGAAGAAAA

Joelle phyto GGTTCTTCCCCCTTTAAGCTTCGGTTTTGTGCTTTCTCTTTACTTTTTCTCTGAAGAAAA

Joelle NCBI GGTTCTTCCCCCTTTAAGCTTCGGTTTTGTGCTTTCTCTTTACTTTTTCTCTGAAGAAAA

CN 119243 GGTTCTTCCCCCTTTAAGCTTCGGTTTTGTGCTTTCTCTTTACTTTTTCTCTGAAGAAAA

CN 120025 GGTTCTTCCCCCTTTAAGCTTCGGTTTTGTGCTTTCTCTTTACTTTTTCTCTGAAGAAAA

Joelle AAFC GGTTCTTCCCCCTTTAAGCTTCGGTTTTGTGCTTTCTCTTTACTTTTTCTCTGAAGAAAA

************************************************************

CAM 241 TAAATATACAAAAGACACAAATAAAATAAAAAATAAAAAAATAAAAACAATTAATGTATA

17CS1133 TAAATATACAAAAGACACAAATAAAATAAAAAATAAAAAAATAAAAACAATTAATGTATA

CAM 236 TAAATATACAAAAGACACAAATAAAATAAAAAATAAAAAAATAAAAACAATTAATGTATA

Blaine Creek TAAATATACAAAAGACACAAATAAAATAAAAAATAAAAAAATAAAAACAATTAATGTATA

CN 119300 TAAATATACAAAAGACACAAATAAAATAAAAAATAAAAAAATAAAAACAATTAATGTATA

Yellowstone TAAATATACAAAAGACACAAATAAAATAAAAAATAAAAAAATAAAAACAATTAATGTATA

Hoga TAAATATACAAAAGACACAAATAAAATAAAAAATAAAAAAATAAAAACAATTAATGTATA

CN 120027 TAAATATACAAAAGACACAAATAAAATAAAAAATAAAAAAATAAAAACAATTAATGTATA

CN 120030 TAAATATACAAAAGACACAAATAAAATAAAAAATAAAAAAATAAAAACAATTAATGTATA

CN 119294 TAAATATACAAAAGACACAAATAAAATAAAAAATAAAAAAATAAAAACAATTAATGTATA

CN 120013 TAAATATACAAAAGACACAAATAAAATAAAAAATAAAAAAATAAAAACAATTAATGTATA

CN 120017 TAAATATACAAAAGACACAAATAAAATAAAAAATAAAAAAATAAAAACAATTAATGTATA

CN 119205 TAAATATACAAAAGACACAAATAAAATAAAAAATAAAAAAATAAAAACAATTAATGTATA

DH55 ref genome TAAATATACAAAAGACACAAATAAAATAAAAAATAAAAAAATAAAAACAATTAATGTATA

09-CS0040 TAAATATACAAAAGACACAAATAAAATAAAAAATAAAAAAATAAAAACAATTAATGTATA

CN 113754 TAAATATACAAAAGACACAAATAAAATAAAAAATAAAAAAATAAAAACAATTAATGTATA

CO46 NCBI TAAATATACAAAAGACACAAATAAAATAAAAAATAAAAAAATAAAAACAATTAATGTATA

Jasper TAAATATACAAAAGACACAAATAAAATAAAAAATAAAAAAATAAAAACAATTAATGTATA

Joelle phyto TAAATATACAAAAGACACAAATAAAATAAAAAATAAAAAAATAAAAACAATTAATGTATA

Joelle NCBI TAAATATACAAAAGACACAAATAAAATAAAAAATAAAAAAATAAAAACAATTAATGTATA

CN 119243 TAAATATACAAAAGACACAAATAAAATAAAAAATAAAAAAATAAAAACAATTAATGTATA

CN 120025 TAAATATACAAAAGACACAAATAAAATAAAAAAT-AAAAAATAAAAACAATTAATGTATA

Joelle AAFC TAAATATACAAAAGACACAAATAAAATAAAAAATAAAAAAATAAAAACAATTAATGTATA

********************************** *************************

CAM 241 GTTTGATTTTTCCGGCGAATCTCTTGTTGTTTTACTCGGTTCGGTCTTTGTTAGTGTTTT

17CS1133 GTTTGATTTTTCCGGCGAATCTCTTGTTGTTTTACTCGGTTCGGTCTTTGTTAGTGTTTT

CAM 236 GTTTGATTTTTCCGGCGAATCTCTTGTTGTTTTACTCGGTTCGGTCTTTGTTAGTGTTTT

Blaine Creek GTTTGATTTTTCCGGCGAATCTCTTGTTGTTTTACTCGGTTCGGTCTTTGTTAGTGTTTT

CN 119300 GTTTGATTTTTCCGGCGAATCTCTTGTTGTTTTACTCGGTTCGGTCTTTGTTAGTGTTTT

Yellowstone GTTTGATTTTTCCGGCGAATCTCTTGTTGTTTTACTCGGTTCGGTCTTTGTTAGTGTTTT

Hoga GTTTGATTTTTCCGGCGAATCTCTTGTTGTTTTACTCGGTTCGGTCTTTGTTAGTGTTTT

CN 120027 GTTTGATTTTTCCGGCGAATCTCTTGTTGTTTTACTCGGTTCGGTCTTTGTTAGTGTTTT

CN 120030 GTTTGATTTTTCCGGCGAATCTCTTGTTGTTTTACTCGGTTCGGTCTTTGTTAGTGTTTT

CN 119294 GTTTGATTTTTCCGGCGAATCTCTTGTTGTTTTACTCGGTTCGGTCTTTGTTAGTGTTTT

CN 120013 GTTTGATTTTTCCGGCGAATCTCTTGTTGTTTTACTCGGTTCGGTCTTTGTTAGTGTTTT

CN 120017 GTTTGATTTTTCCGGCGAATCTCTTGTTGTTTTACTCGGTTCGGTCTTTGTTAGTGTTTT

CN 119205 GTTTGATTTTTCCGGCGAATCTCTTGTTGTTTTACTCGGTTCGGTCTTTGTTAGTGTTTT

DH55 ref genome GTTTGATTTTTCCGGCGAATCTCTTGTTGTTTTACTCGGTTCGGTCTTTGTTAGTGTTTT

09-CS0040 GTTTGATTTTTCCGGCGAATCTCTTGTTGTTTTACTCGGTTCGGTCTTTGTTAGTGTTTT

CN 113754 GTTTGATTTTTCCGGCGAATCTCTTGTTGTTTTACTCGGTTCGGTCTTTGTTAGTGTTTT

CO46 NCBI GTTTGATTTTTCCGGCGAATCTCTTGTTGTTTTACTCGGTTCGGTCTTTGTTAGTGTTTT

Jasper GTTTGATTTTTCCGGCGAATCTCTTGTTGTTTTACTCGGTTCGGTCTTTGTTAGTGTTTT

Joelle phyto GTTTGATTTTTCCGGCGAATCTCTTGTTGTTTTACTCGGTTCGGTCTTTGTTAGTGTTTT

Joelle NCBI GTTTGATTTTTCCGGCGAATCTCTTGTTGTTTTACTCGGTTCGGTCTTTGTTAGTGTTTT

CN 119243 GTTTGATTTTTCCGGCGAATCTCTTGTTGTTTTACTCGGTTCGGTCTTTGTTAGTGTTTT

CN 120025 GTTTGATTTTTCCGGCGAATCTCTTGTTGTTTTACTCGGTTCGGTCTTTGTTAGTGTTTT

Joelle AAFC GTTTGATTTTTCCGGCGAATCTCTTGTTGTTTTACTCGGTTCGGTCTTTGTTAGTGTTTT

************************************************************

CAM 241 TTCTATGACCATGTGAGATACATGAGATAACCAAATCTATGGAAGAACAATGTCGTGTTG

17CS1133 TTCTATGACCATGTGAGATACATGAGATAACCAAATCTATGGAAGAACAATGTCGTGTTG

CAM 236 TTCTATGACCATGTGAGATACATGAGATAACCAAATCTATGGAAGAACAATGTCGTGTTG

Blaine Creek TTCTATGACCATGTGAGATACATGAGATAACCAAATCTATGGAAGAACAATGTCGTGTTG

CN 119300 TTCTATGACCATGTGAGATACATGAGATAACCAAATCTATGGAAGAACAATGTCGTGTTG

Yellowstone TTCTATGACCATGTGAGATACATGAGATAACCAAATCTATGGAAGAACAATGTCGTGTTG

Hoga TTCTATGACCATGTGAGATACATGAGATAACCAAATCTATGGAAGAACAATGTCGTGTTG

CN 120027 TTCTATGACCATGTGAGATACATGAGATAACCAAATCTATGGAAGAACAATGTCGTGTTG

CN 120030 TTCTATGACCATGTGAGATACATGAGATAACCAAATCTATGGAAGAACAATGTCGTGTTG

CN 119294 TTCTATGACCATGTGAGATACATGAGATAACCAAATCTATGGAAGAACAATGTCGTGTTG

CN 120013 TTCTATGACCATGTGAGATACATGAGATAACCAAATCTATGGAAGAACAATGTCGTGTTG

CN 120017 TTCTATGACCATGTGAGATACATGAGATAACCAAATCTATGGAAGAACAATGTCGTGTTG

CN 119205 TTCTATGACCATGTGAGATACATGAGATAACCAAATCTATGGAAGAACAATGTCGTGTTG

DH55 ref genome TTCTATGACCATGTGAGATACATGAGATAACCAAATCTATGGAAGAACAATGTCGTGTTG

09-CS0040 TTCTATGACCATGTGAGATACATGAGATAACCAAATCTATGGAAGAACAATGTCGTGTTG

CN 113754 TTCTATGACCATGTGAGATACATGAGATAACCAAATCTATGGAAGAACAATGTCGTGTTG

CO46 NCBI TTCTATGACCATGTGAGATACATGAGATAACCAAATCTATGGAAGAACAATGTCGTGTTG

Jasper TTCTATGACCATGTGAGATACATGAGATAACCAAATCTATGGAAGAACAATGTCGTGTTG

Joelle phyto TTCTATGACCATGTGAGATACATGAGATAACCAAATCTATGGAAGAACAATGTCGTGTTG

Joelle NCBI TTCTATGACCATGTGAGATACATGAGATAACCAAATCTATGGAAGAACAATGTCGTGTTG

CN 119243 TTCTATGACCATGTGAGATACATGAGATAACCAAATCTATGGAAGAACAATGTCGTGTTG

CN 120025 TTCTATGACCATGTGAGATACATGAGATAACCAAATCTATGGAAGAACAATGTCGTGTTG

Joelle AAFC TTCTATGACCATGTGAGATACATGAGATAACCAAATCTATGGAAGAACAATGTCGTGTTG

************************************************************

CAM 241 AGCTTAAGCTTCTTACTTTTTTTTCTGCTTTTCTCTCTCTATCTCTCTCTCTATTTCCTT

17CS1133 AGCTTAAGCTTCTTACTTTTTTTTCTGCTTTTCTCTCTCTATCTCTCTCTCTATTTCCTT

CAM 236 AGCTTAAGCTTCTTACTTTTTTTTCTGCTTTTCTCTCTCTATCTCTCTCTCTATTTCCTT

Blaine Creek AGCTTAAGCTTCTTACTTTTTTTTCTGCTTTTCTCTCTCTATCTCTCTCTCTATTTCCTT

CN 119300 AGCTTAAGCTTCTTACTTTTTTTTCTGCTTTTCTCTCTCTATCTCTCTCTCTATTTCCTT

Yellowstone AGCTTAAGCTTCTTACTTTTTTTTCTGCTTTTCTCTCTCTATCTCTCTCTCTATTTCCTT

Hoga AGCTTAAGCTTCTTACTTTTTTTTCTGCTTTTCTCTCTCTATCTCTCTCTCTATTTCCTT

CN 120027 AGCTTAAGCTTCTTACTTTTTTTTCTGCTTTTCTCTCTCTATCTCTCTCTCTATTTCCTT

CN 120030 AGCTTAAGCTTCTTACTTTTTTTTCTGCTTTTCTCTCTCTATCTCTCTCTCTATTTCCTT

CN 119294 AGCTTAAGCTTCTTACTTTTTTTTCTGCTTTTCTCTCTCTATCTCTCTCTCTATTTCCTT

CN 120013 AGCTTAAGCTTCTTACTTTTTTTTCTGCTTTTCTCTCTCTATCTCTCTCTCTATTTCCTT

CN 120017 AGCTTAAGCTTCTTACTTTTTTTTCTGCTTTTCTCTCTCTATCTCTCTCTCTATTTCCTT

CN 119205 AGCTTAAGCTTCTTACTTTTTTTTCTGCTTTTCTCTCTCTATCTCTCTCTCTATTTCCTT

DH55 ref genome AGCTTAAGCTTCTTACTTTTTTTTCTGCTTTTCTCTCTCTATCTCTCTCTCTATTTCCTT

09-CS0040 AGCTTAAGCTTCTTACTTTTTTTTCTGCTTTTCTCTCTCTATCTCTCTCTCTATTTCCTT

CN 113754 AGCTTAAGCTTCTTACTTTTTTTTCTGCTTTTCTCTCTCTATCTCTCTCTCTATTTCCTT

CO46 NCBI AGCTTAAGCTTCTTACTTTTTTTTCTGCTTTTCTCTCTCTATCTCTCTCTCTATTTCCTT

Jasper AGCTTAAGCTTCTTACTTTTTTTTCTGCTTTTCTCTCTCTATCTCTCTCTCTATTTCCTT

Joelle phyto AGCTTAAGCTTCTTACTTTTTTTTCTGCTTTTCTCTCTCTATCTCTCTCTCTATTTCCTT

Joelle NCBI AGCTTAAGCTTCTTACTTTTTTTTCTGCTTTTCTCTCTCTATCTCTCTCTCTATTTCCTT

CN 119243 AGCTTAAGCTTCTTACTTTTTTTTCTGCTTTTCTCTCTCTATCTCTCTCTCTATTTCCTT

CN 120025 AGCTTAAGCTTCTTACTTTTTTTTCTGCTTTTCTCTCTCTATCTCTCTCTCTATTTCCTT

Joelle AAFC AGCTTAAGCTTCTTACTTTTTTTTCTGCTTTTCTCTCTCTATCTCTCTCTCTATTTCCTT

************************************************************

CAM 241 TTTTCTGCATGGATTTTTATTTTATTTGGAAATTTTTTGCATGTCCTTTGAGATTTGCTT

17CS1133 TTTTCTGCATGGATTTTTATTTTATTTGGAAATTTTTTGCATGTCCTTTGAGATTTGCTT

CAM 236 TTTTCTGCATGGATTTTTATTTTATTTGGAAATTTTTTGCATGTCCTTTGAGATTTGCTT

Blaine Creek TTTTCTGCATGGATTTTTATTTTATTTGGAAATTTTTTGCATGTCCTTTGAGATTTGCTT

CN 119300 TTTTCTGCATGGATTTTTATTTTATTTGGAAATTTTTTGCATGTCCTTTGAGATTTGCTT

Yellowstone TTTTCTGCATGGATTTTTATTTTATTTGGAAATTTTTTGCATGTCCTTTGAGATTTGCTT

Hoga TTTTCTGCATGGATTTTTATTTTATTTGGAAATTTTTTGCATGTCCTTTGAGATTTGCTT

CN 120027 TTTTCTGCATGGATTTTTATTTTATTTGGAAATTTTTTGCATGTCCTTTGAGATTTGCTT

CN 120030 TTTTCTGCATGGATTTTTATTTTATTTGGAAATTTTTTGCATGTCCTTTGAGATTTGCTT

CN 119294 TTTTCTGCATGGATTTTTATTTTATTTGGAAATTTTTTGCATGTCCTTTGAGATTTGCTT

CN 120013 TTTTCTGCATGGATTTTTATTTTATTTGGAAATTTTTTGCATGTCCTTTGAGATTTGCTT

CN 120017 TTTTCTGCATGGATTTTTATTTTATTTGGAAATTTTTTGCATGTCCTTTGAGATTTGCTT

CN 119205 TTTTCTGCATGGATTTTTATTTTATTTGGAAATTTTTTGCATGTCCTTTGAGATTTGCTT

DH55 ref genome TTTTCTGCATGGATTTTTATTTTATTTGGAAATTTTTTGCATGTCCTTTGAGATTTGCTT

09-CS0040 TTTTCTGCATGGATTTTTATTTTATTTGGAAATTTTTTGCATGTCCTTTGAGATTTGCTT

CN 113754 TTTTCTGCATGGATTTTTATTTTATTTGGAAATTTTTTGCATGTCCTTTGAGATTTGCTT

CO46 NCBI TTTTCTGCATGGATTTTTATTTTATTTGGAAATTTTTTGCATGTCCTTTGAGATTTGCTT

Jasper TTTTCTGCATGGATTTTTATTTTATTTGGAAATTTTTTGCATGTCCTTTGAGATTTGCTT

Joelle phyto TTTTCTGCATGGATTTTTATTTTATTTGGAAATTTTTTGCATGTCCTTTGAGATTTGCTT

Joelle NCBI TTTTCTGCATGGATTTTTATTTTATTTGGAAATTTTTTGCATGTCCTTTGAGATTTGCTT

CN 119243 TTTTCTGCATGGATTTTTATTTTATTTGGAAATTTTTTGCATGCCCTTTGAGATTTGCTT

CN 120025 TTTTCTGCATGGATTTTTATTTTATTTGGAAATTTTTTGCATGCCCTTTGAGATTTGCTT

Joelle AAFC TTTTCTGCATGGATTTTTATTTTATTTGGAAATTTTTTGCATGCCCTTTGAGATTTGCTT

******************************************* ****************

CAM 241 GACACGTTCTGCTGCGTACTCGATGCTGTTTAGTGAAGTTTCAAGCCGTCTTTGATTGAT

17CS1133 GACACGTTCTGCTGCGTACTCGATGCTGTTTAGTGAAGTTTCAAGCCGTCTTTGATTGAT

CAM 236 GACACGTTCTGCTGCGTACTCGATGCTGTTTAGTGAAGTTTCAAGCCGTCTTTGATTGAT

Blaine Creek GACACGTTCTGCTGCGTACTCGATGCTGTTTAGTGAAGTTTCAAGCCGTCTTTGATTGAT

CN 119300 GACACGTTCTGCTGCGTACTCGATGCTGTTTAGTGAAGTTTCAAGCCGTCTTTGATTGAT

Yellowstone GACACGTTCTGCTGCGTACTCGATGCTGTTTAGTGAAGTTTCAAGCCGTCTTTGATTGAT

Hoga GACACGTTCTGCTGCGTACTCGATGCTGTTTAGTGAAGTTTCAAGCCGTCTTTGATTGAT

CN 120027 GACACGTTCTGCTGCGTACTCGATGCTGTTTAGTGAAGTTTCAAGCCGTCTTTGATTGAT

CN 120030 GACACGTTCTGCTGCGTACTCGATGCTGTTTAGTGAAGTTTCAAGCCGTCTTTGATTGAT

CN 119294 GACACGTTCTGCTGCGTACTCGATGCTGTTTAGTGAAGTTTCAAGCCGTCTTTGATTGAT

CN 120013 GACACGTTCTGCTGCGTACTCGATGCTGTTTAGTGAAGTTTCAAGCCGTCTTTGATTGAT

CN 120017 GACACGTTCTGCTGCGTACTCGATGCTGTTTAGTGAAGTTTCAAGCCGTCTTTGATTGAT

CN 119205 GACACGTTCTGCTGCGTACTCGATGCTGTTTAGTGAAGTTTCAAGCCGTCTTTGATTGAT

DH55 ref genome GACACGTTCTGCTGCGTACTCGATGCTGTTTAGTGAAGTTTCAAGCCGTCTTTGATTGAT

09-CS0040 GACACGTTCTGCTGCGTACTCGATGCTGTTTAGTGAAGTTTCAAGCCGTCTTTGATTGAT

CN 113754 GACACGTTCTGCTGCGTACTCGATGCTGTTTAGTGAAGTTTCAAGCCGTCTTTGATTGAT

CO46 NCBI GACACGTTCTGCTGCGTACTCGATGCTGTTTAGTGAAGTTTCAAGCCGTCTTTGATTGAT

Jasper GACACGTTCTGCTGCGTACTCGATGCTGTTTAGTGAAGTTTCAAGCCGTCTTTGATTGAT

Joelle phyto GACACGTTCTGCTGCGTACTCGATGCTGTTTAGTGAAGTTTCAAGCCGTCTTTGATTGAT

Joelle NCBI GACACGTTCTGCTGCGTACTCGATGCTGTTTAGTGAAGTTTCAAGCCGTCTTTGATTGAT

CN 119243 GACACGTTCTGCTGCGTACTCGATGCTGTTTAGTGAAGTTTCAAGCCGTCTTTGATTGAT

CN 120025 GACACGTTCTGCTGCGTACTCGATGCTGTTTAGTGAAGTTTCAAGCCGTCTTTGATTGAT

Joelle AAFC GACACGTTCTGCTGCGTACTCGATGCTGTTTAGTGAAGTTTCAAGCCGTCTTTGATTGAT

************************************************************

CAM 241 ACTTAGCTTTAGGGATTAATTCCATATGCTTCTGATTTTAGTTTTTATATCAGTATTCAC

17CS1133 ACTTAGCTTTAGGGATTAATTCCATATGCTTCTGATTTTAGTTTTTATATCAGTATTCAC

CAM 236 ACTTAGCTTTAGGGATTAATTCCATATGCTTCTGATTTTAGTTTTTATATCAGTATTCAC

Blaine Creek ACTTAGCTTTAGGGATTAATTCCATATGCTTCTGATTTTAGTTTTTATATCAGTATTCAC

CN 119300 ACTTAGCTTTAGGGATTAATTCCATATGCTTCTGATTTTAGTTTTTATATCAGTATTCAC

Yellowstone ACTTAGCTTTAGGGATTAATTCCATATGCTTCTGATTTTAGTTTTTATATCAGTATTCAC

Hoga ACTTAGCTTTAGGGATTAATTCCATATGCTTCTGATTTTAGTTTTTATATCAGTATTCAC

CN 120027 ACTTAGCTTTAGGGATTAATTCCATATGCTTCTGATTTTAGTTTTTATATCAGTATTCAC

CN 120030 ACTTAGCTTTAGGGATTAATTCCATATGCTTCTGATTTTAGTTTTTATATCAGTATTCAC

CN 119294 ACTTAGCTTTAGGGATTAATTCCATATGCTTCTGATTTTAGTTTTTATATCAGTATTCAC

CN 120013 ACTTAGCTTTAGGGATTAATTCCATATGCTTCTGATTTTAGTTTTTATATCAGTATTCAC

CN 120017 ACTTAGCTTTAGGGATTAATTCCATATGCTTCTGATTTTAGTTTTTATATCAGTATTCAC

CN 119205 ACTTAGCTTTAGGGATTAATTCCATATGCTTCTGATTTTAGTTTTTATATCAGTATT---

DH55 ref genome ACTTAGCTTTAGGGATTAATTCCATATGCTTCTGATTTTAGTTTTTATATCAGTATT---

09-CS0040 ACTTAGCTTTAGGGATTAATTCCATATGCTTCTGATTTTAGTTTTTATATCAGTATT---

CN 113754 ACTTAGCTTTAGGGATTAATTCCATATGCTTCTGATTTTAGTTTTTATATCAGTATT---

CO46 NCBI ACTTAGCTTTAGGGATTAATTCCATATGCTTCTGATTTTAGTTTTTATATCAGTATT---

Jasper ACTTAGCTTTAGGGATTAATTCCATATGCTTCTGATTTTAGTTTTTATATCAGTATT---

Joelle phyto ACTTAGCTTTAGGGATTAATTCCATATGCTTCTGATTTTAGTTTTTATATCAGTATT---

Joelle NCBI ACTTAGCTTTAGGGATTAATTCCATATGCTTCTGATTTTAGTTTTTATATCAGTATT---

CN 119243 ACTTAGCTTTAGGGATTAATTCCATATGCTTCTGATTTTAGTTTTTATATCAGTATT---

CN 120025 ACTTAGCTTTAGGGATTAATTCCATATGCTTCTGATTTTAGTTTTTATATCAGTATT---

Joelle AAFC ACTTAGCTTTAGGGATTAATTCCATATGCTTCTGATTTTAGTTTTTATATCAGTATT---

*********************************************************

CAM 241 TACAACAAATATGTACATTGATAGCAGACGGATATAGCTTCATTTTCCAGTCATCGCTAT

17CS1133 TACAACAAATATGTACATTGATAGCAGACGGATATAGCTCATTTTCC--GTCACTGCTAT

CAM 236 TACAACAAATATGTACATTGATAGCAGACGGATATAGCTCATTTTCC--GTCACTGCTAT

Blaine Creek TACAACAAATATGTACATTGATAGCAGACGGATATAGCTCATTTTCC--GTCACTGCTAT

CN 119300 TACAACAAATATGTACATTGATAGCAGACGGATATAGCTCATTTTCC--GTCACTGCTAT

Yellowstone TACAACAAATATGTACATTGATAGCAGACGGATATAGCTCATTTTCC--GTCACTGCTAT

Hoga TACAACAAATATGTACATTGATAGCAGACGGATATAGCTCATTTTCC--GTCACTGCTAT

CN 120027 TACAACAAATATGTACATTGATAGCAGACGGATATAGCTCATTTTCC--GTCACTGCTAT

CN 120030 TACAACAAATATGTACATTGATAGCAGACGGATATAGCTCATTTTCC--GTCACTGCTAT

CN 119294 TACAACAAATATGTACATTGATAGCAGACGGATATAGCTCATTTTCC--GTCACTGCTAT

CN 120013 TACAACAAATATGTACATTGATAGCAGACGGATATAGCTCATTTTCC--GTCACTGCTAT

CN 120017 TACAACAAATATGTACATTGATAGCAGACGGATATAGCTCATTTTCC--GTCACTGCTAT

CN 119205 ------------------------------------------------------------

DH55 ref genome ---GCTAAATGATTCACTAGATCTC-------------TCCTTTTTT--ATAGTGATTAA

09-CS0040 ---GCTAAATGATTCACTAGATCTC-------------TCCTTTTTT--ATAGTGATTAA

CN 113754 ---GCTAAATGATTCACTAGATCTC-------------TCCTTTTTT--ATAGTGATTAA

CO46 NCBI ---GCTAAATGATTCACTAGATCTC-------------TCCTTTTTT--ATAGTGATTAA

Jasper ---GCTAAATGATTCACTAGATCTC-------------TCCTTTTTT--ATAGTGATTAA

Joelle phyto ---GCTAAATGATTCACTAGATCTC-------------TCCTTTTTT--ATAGTGATTAA

Joelle NCBI ---GCTAAATGATTCACTAGATCTC-------------TCCTTTTTT--ATAGTGATTAA

CN 119243 ------------------------------------------------------------

CN 120025 ------------------------------------------------------------

Joelle AAFC ------------------------------------------------------------

CAM 241 GAAAGATGAGATTTTTTT-CGCTTACTTTT----TAATAAGCATCT--------------

17CS1133 GAAAGATGAGATTTTTTTTCGCTTACTTTT----TAAT-AGCATCT--------------

CAM 236 GAAAGATGAGATTTTTTTTCGCTTACTTTT----TAAT-AGCATCT--------------

Blaine Creek GAAAGATGAGATTTTTTTTCGCTTACTTTT----TAAT-AGCATCT--------------

CN 119300 GAAAGATGAGATTTTTTTTCGCTTACTTTT----TAAT-AGCATCT--------------

Yellowstone GAAAGATGAGATTTTTTTTCGCTTACTTTT----TAAT-AGCATCT--------------

Hoga GAAAGATGAGATTTTTTTTCGCTTACTTTT----TAAT-AGCATCT--------------

CN 120027 GAAAGATGAGATTTTTTTTCGCTTACTTTT----TAAT-AGCATCT--------------

CN 120030 GAAAGATGAGATTTTTTTTCGCTTACTTTT----TAAT-AGCATCT--------------

CN 119294 GAAAGATGAGATTTTTTTTCGCTTACTTTT----TAAT-AGCATCT--------------

CN 120013 GAAAGATGAGATTTTTTTTCGCTTACTTTT----TAAT-AGCATCT--------------

CN 120017 GAAAGATGAGATTTTTTTTCGCTTACTTTT----TAAT-AGCATCT--------------

CN 119205 ------------------------------------------------------------

DH55 ref genome AACTCATTAGATCTCTTTGGATTTGTATTCAGTGCAATGAACCTTC--------------

09-CS0040 AACTCATTAGATCTCTTTGGATTTGTATTCAGTGCAATGAACCTTC--------------

CN 113754 AACTCATTAGATCTCTTTGGATTTGTATTCAGTGCAATGAACCTTC--------------

CO46 NCBI AACTCATTAGATCTCTTTGGATTTGTATTCAGTGCAATGAACCTTC--------------

Jasper AACTCATTAGATCTCTTTGGATTTGTATTCAGTGCAATGAACCTTC--------------

Joelle phyto AACTCATTAGATCTCTTTGGATTTGTATTCAGTGCAATGAACCTTCGGGAGATCCATAGA

Joelle NCBI AACTCATTAGATCTCTTTGGATTTGTATTCAGTGCAATGAACCTTCGGGAGATCCATAGA

CN 119243 ------------------------------------------------------------

CN 120025 ------------------------------------------------------------

Joelle AAFC ------------------------------------------------------------

CAM 241 -----------TATTAACGCTTATGATAGGAAAATTATGTAGCTGGAAACAAAAGTGACT

17CS1133 -----------TATTAACGC-TATGATAGGAAAATTATGTAGCGGGAAACAAAAGTGACT

CAM 236 -----------TATTAACGC-TATGATAGGAAAATTATGTAGCGGGAAACAAAAGTGACT

Blaine Creek -----------TATTAACGC-TATGATAGGAAAATTATGTAAT-GGAAACAAAAGTGACT

CN 119300 -----------TATTAACGCTTATGATAGGAAAATTATGTAGC-GGAAACAAAAGTGACT

Yellowstone -----------TATTAACGC-TATGATAGGAAAATTATGTAGCGGGAAACAAAAGTGACT

Hoga -----------TATTAACGC-TATGATAGGAAAATTATGTAGCGGGAAACAAAAGTGACT

CN 120027 -----------TATTAACGC-TATGATAGGAAAATTATGTAGCGGGAAACAAAAGTGACT

CN 120030 -----------TATTAACGC-TATGATAGGAAAATTATGTAGCGGGAAACAAAAGTGACT

CN 119294 -----------TATTAACGC-TATGATAGGAAAATTATGTAGCGGGAAACAAAAGTGACT

CN 120013 -----------TATTAACGC-TATGATAGGAAAATTATGTAGC-GGAAACAAAAGTGACT

CN 120017 -----------TATTAACGC-TATGATAGGAAAATTATGTAGCGGGAAACAAAAGTGACT

CN 119205 ------------------------------------------------------------

DH55 ref genome -----------GACTACAAC---AAATATGCACATTGT-TAACCAGAGAAAAACGCTATC

09-CS0040 -----------GACTACAAC---AAATATGCACATTGT-TAACCAGAGAAAAACGCTATC

CN 113754 -----------GACTACAAC---AAATATGCACATTGT-TAACCAGAGAAAAACGCTATC

CO46 NCBI -----------GACTACAAC---AAATATGCACATTGT-TAACCAGAGAAAAACGCTATC

Jasper -----------GACTACAAC---AAATATGCACATTGT-TAACCAGAGAAAAACGCTATC

Joelle phyto ATTTCAATGGGCACTACAAC---AAATATGCACATTGT-TAACCAGAGAAAAACGCTATC

Joelle NCBI ATTTCAATGGGCACTACAAC---AAATATGCACATTGT-TAACCAGAGAAAAACGCTATC

CN 119243 ------------------------------------------------------------

CN 120025 ------------------------------------------------------------

Joelle AAFC ------------------------------------------------------------

CAM 241 TTTGTCCGCCAAT-------------------ACTTAGTGAAATTATTCACTTAAT---C

17CS1133 TTTGTCCGCCAAT-------------------ACTTAGTGAAATTATTCACTTAAT---C

CAM 236 TTTGTCCGCCAAT-------------------ACTTAGTGAAATTATTCACTTAAT---C

Blaine Creek TTTGTCTGCCAAT-------------------ACTTAGTGAAATTATTCACTTAAT---C

CN 119300 TTTGTCCGCCAAT-------------------ACTTAGTGAAATTATTCACTTAAT---C

Yellowstone TTTGTCCGCCAAT-------------------ACTTAGTGAAATTATTCACTTAAT---C

Hoga TTTGTCCGCCAAT-------------------ACTTAGTGAAATTATTCACTTAAT---C

CN 120027 TTTGTCCGCCAAT-------------------ACTTAGTGAAATTATTCACTTAAT---C

CN 120030 TTTGTCCGCCAAT-------------------ACTTAGTGAAATTATTCACTTAAT---C

CN 119294 TTTGTCCGCCAAT-------------------ACTTAGTGAAATTATTCACTTAAT---C

CN 120013 TTTGTCCGTCAAT-------------------ACTTAGTGAAATTATTCACTTAAT---C

CN 120017 TTTGTCCGCCAAT-------------------ACTTAGTGAAATTATTCACTTAAT---C

CN 119205 ------------------------------------------------------------

DH55 ref genome ATAGGTCTTTGATAGCGTTTTCATGAGCGCTGTTTTAGGGCACT--GTTATTATATGTAC

09-CS0040 ATAGGTCTTTGATAGCGTTTTCAGTAATATTGTTTTAGAAGCATCTGTTATTATATGTAC

CN 113754 ATAGGTCTTTGATAAGCGTTTTACATAGTATTGTTTTAGAGCACTTGTTATTATATGTAT

CO46 NCBI ATAGGTCTTTGATAGCGTTTTCATGAGCGCTGTTTTAGGGCACT--GTTATTATATGTAC

Jasper ATAGGTCTTTGATAGCGTTTTCATGAATATTGTTTTAG--AGACTCGTTATTATATGTGC

Joelle phyto ATAGGTCTTTGATAGCGTTTTCATGAGCGCTGTTTTAGGGCACT--GTTATTATATGTAC

Joelle NCBI ATAGGTCTTTGATAGCGTTTTCATGAGCGCTGTTTTAGGGCACT--GTTATTATATGTAC

CN 119243 ------------------------------------------------------------

CN 120025 ------------------------------------------------------------

Joelle AAFC ------------------------------------------------------------

CAM 241 GCCAAAT--AAAAAAAACCCAAAAACTCGACACA--CTCTCTCTCTCTCTCGTTAC-AAC

17CS1133 GCCAAAT--AAAAAAAACCCAAAAACTCGACACA--CTCTCTCTCTCTCTCGTTAC-AAC

CAM 236 GCCAAATAAAAAAAAAACCCAAAAACTCGACACA--CTCTCTCTCTCTCTCGTTACTAAC

Blaine Creek GCCAAAT---AAAAAAACCCAAAAACTCGACACA-TCTCTCTCTCTCTCTCGTTAC-AAC

CN 119300 GCCAAAT--AAAAAAAACCCAAAAACTCGACACA--CTCTCTCTCTCTCTCGTTAC-AAC

Yellowstone GCCAAATAAAAAAAAAACCCAAAAACTCGACACA----CTCTCTCTCTCTCGTTAC-AAC

Hoga GCCAAAT--AAAAAAAAACCCAAAACTCGACACA--CTCTCTCTCTCTCTCGTTAC-AAC

CN 120027 GCCAAATAAAAAAAAAACCCAAAAACTCGACACACTCTCTCTCTCTCTCTCGTTAC-AAC

CN 120030 GCCAAATAAAAAAAAAACCCAAAAACTCGACACACTCTCTCTCTCTCTCTCGTTAC-AAC

CN 119294 GCCAAATAAAAAAAAAACCCAAAAACTCGACACA--CTCTCTCTCTCTCTCGTTAC-AAC

CN 120013 GCCAAAT-AAAAAAAAACCCAAAAACTCGACACA--CTCTCTCTCTCTCTCGTTAC-AAC

CN 120017 GCCAAAT-AAAAAAAAACCCAAAAACTCGACACA--CTCTCTCTCTCTCTCGTTAC-AAC

CN 119205 ------------------------------------------------------------

DH55 ref genome CCCATATACAA---------TAGCACTTATTACG------TATGCTATGTTATAAA-AAT

09-CS0040 ATCTCATATATAA-------TAAGACTTATTACG----TATGCTTTATGTTATAAA-AAT

CN 113754 CCTATATACAA--------TAAGCACTTATTACG-----TATCGCTATGTTATAAA-AAT

CO46 NCBI CCCATATACAA---------TAGCACTTATTACG------TATGCTATGTTATAAA-AAT

Jasper ACTCTATATACAA-------TAGCACTTATTACG------TATGCTATGTTATAAA-AAT

Joelle phyto CCCATATACAA---------TAGCACTTATTACG------TATGCTATGTTATAAA-AAT

Joelle NCBI CCCATATACAA---------TAGCACTTATTACG------TATGCTATGTTATAAA-AAT

CN 119243 ------------------------------------------------------------

CN 120025 ------------------------------------------------------------

Joelle AAFC ------------------------------------------------------------

CAM 241 CTAAGCTCAACCTAAGAAGATTTCT-TCTTCTAAATTCTCTC-GTGTGCTCAAATTCTTC

17CS1133 CTAAGCTCAACCTAAGAAGATTTCT-TCTTCTAAATTCTCTGTGTGTGCTCAAATTCTTC

CAM 236 CTAAGCTCAACCTAAGAAGATTTCT-TCTTCTAAATTCTCTGTGTGTGCTCAAATTCTTC

Blaine Creek CTAAGCTCAACCTAAGAAGATTTCT-TCTTCTAAATTCTCTA-GTGTGCTCAAATTCTTC

CN 119300 CTAAGCTCAACCTAAGAAGATTTCT-TCTTCTAAATTCTCTGTGTGTGCTCAAATTCTTC

Yellowstone CTAAGCTCAACCTAAGAAGATTTCT-TCTTCTAAATTCTCTGTGTGTGCTCAAATTCTTC

Hoga CTAAGCTCAACCTAAGAAGATTTCT-TCTTCTAAATTCTCTGTGTGTGCTCAAATTCTTC

CN 120027 CTAAGCTCAACCTAAGAAGATTTCT-TCTTCTAAATTCTCTGTGTGTGCTCAAATTCTTC

CN 120030 CTAAGCTCAACCTAAGAAGATTTCT-TCTTCTAAATTCTCTGTGTGTGCTCAAATTCTTC

CN 119294 CTAAGCTCAACCTAAGAAGATTTCT-TCTTCTAAATTCTCTGTGTGTGCTCAAATTCTTC

CN 120013 CTAAGCTCAACCTAAGAAGATTTCT-TCTTCTAAATTCTCCGTGTGTGCTCAAATTCTTC

CN 120017 CTAAGCTCAACCTAAGAAGATTTCT-TCTTCTAAATTCTCTGTGTGTGCTCAAATTCTTC

CN 119205 ------------------------------------------------------------

DH55 ref genome ATAACAT-AGCTTTAAGAAATTGCTATATTATCAAGATCATAATTTTGAAAAAATTTATA

09-CS0040 ATAACAT-AGCTTTAAGAAATTGCTATATTATCAAGATCATAATTTTGAAAAAATTTATA

CN 113754 ATAACATAGACTTTAAGAAATTGCTATATTATCAAGATCATAATTTTGAAAAAATTTATA

CO46 NCBI ATAACAT-AGCTTTAAGAAATTGCTATATTATCAAGATCATAATTTTGAAAAAATTTATA

Jasper ATAACATAGCCTTTAAGAAATTGCTATATTATCAAGATCATAATTTTGAAAAAATTTATA

Joelle phyto ATAACAT-AGCTTTAAGAAATTGCTATATTATCAAGATCATAATTTTGAAAAAATTTATA

Joelle NCBI ATAACAT-AGCTTTAAGAAATTGCTATATTATCAAGATCATAATTTTGAAAAAATTTATA

CN 119243 ------------------------------------------------------------

CN 120025 ------------------------------------------------------------

Joelle AAFC ------------------------------------------------------------

CAM 241 TAAA--------TTTCTTATCAATTCTTCAG-TTTTAT---CGCCGTTTGTGAAAAGCT-

17CS1133 TAAA--------TTTCTTATCAATTCTTCAG-TTTTAT---CGCCGTTTGTGAAAAGCC-

CAM 236 TAAA--------TTTCTTATCAATTCTTCAGCTTTTAT---CGCCGTTTGTGAAAAGCC-

Blaine Creek TAAA--------TTTCTTATCAATTCTTCAG-TTTTAT---CGCCGCTTGTGAAAAGCC-

CN 119300 TAAA--------TTTCTTATCAATTCTTCAG-TTTTAT---CGCCGTTTGTGAAAAGCC-

Yellowstone TAAA--------TTTCTTATCAATTCTTCAG-TTTTAT---CGCCGTTTGTGAAAAGCC-

Hoga TAAA--------TTTCTTATCAATTCTTCGG-TTTTAT---CGCCGTTTGTGAAAAGCC-

CN 120027 TAAA--------TTTCTTATCAATTCTTCAG-TTTTAT---CGCCGTTTGTGAAAAGCC-

CN 120030 TAAA--------TTTCTTATCAATTCTTCAG-TTTTAT---CGCCGTTTGTGAAAAGCC-

CN 119294 TAAA--------TTTCTTATCAATTCTTCAG-CTTTAT---CGCCGTTTGTGAAAAGCC-

CN 120013 TAAA--------TTTCTTATCAATTCTTCAG-TTTTAT---CGCCGTTTGTGAAAAGCC-

CN 120017 TAAA--------TTTCTTATCAATTCTTCAG-TTTTAT---CGCCGTTTGTGAAAAGCC-

CN 119205 ------------------------------------------------------------

DH55 ref genome CAACATTTGTTTTTTCGT-GCTATGTTATAG-TTTTAT-AGCATAG-CTCTAATAAACT-

09-CS0040 CAACATTTGTTTTTTCGTAGCTATGTTATAG-TTTTATAAGCATAGCTTCTAATAAACT-

CN 113754 CAACATTTGTTTTTTCGT-GCTATGTTATAG-TTTTAT-AGCATAAGCTCTAATAAACTC

CO46 NCBI CAACATTTGTTTTTTCGT-GCTATGTTATAG-TTTTAT-AGCATAG-CTCTAATAAACT-

Jasper CAACATTTGTTTTTTCGT-GCTATGTTATAG-TTTTATAAGCATAG-CTCTAATAAACTC

Joelle phyto CAACATTTGTTTTTTCGT-GCTATGTTATAG-TTTTAT-AGCATAG-CTCTAATAAACT-

Joelle NCBI CAACATTTGTTTTTTCGT-GCTATGTTATAG-TTTTAT-AGCATAG-CTCTAATAAACT-

CN 119243 ------------------------------------------------------------

CN 120025 ------------------------------------------------------------

Joelle AAFC ------------------------------------------------------------

CAM 241 ------------------CCAATTTCGC---CGCTTGTGAAAAGCTCCAATTT-CGCCCT

17CS1133 ------------------CCAATTTCGC---CGTTTGTGAAAAGCCCCAATTT-CGCCCT

CAM 236 ------------------CCAATTTCGC---CGTTTGTGAAAAGCCCCAATTTCGCCTCT

Blaine Creek ------------------CCAATTTCGC---CGTTTGTGAAAAGCCCTAATTT-CGC-CT

CN 119300 ------------------CCAATTTCGC---CGTTTGTGAAAAGCCCCAATTT-CGCCCT

Yellowstone ------------------CCAATTTCGC---CGTTTGTGAAAAGCCCCAATTTCGCCTCT

Hoga ------------------CCAATTTCGC---CGTTTGTGAAAAGCCCCAATTTCGCCTCT

CN 120027 ------------------CCAATTTCGC---CGTTTGTGAAAAGCCCCAATTT-CGCCCT

CN 120030 ------------------CCAATTTCGC---CGTTTGTGAAAAGCCCCAATTTACGCCCT

CN 119294 ------------------CCAATTTCGC---CGTTTGTGAAAAGCCCCAATTT-CGCCCT

CN 120013 ------------------CCAATTTCGC---CGTTTGTGAAAAGCCCCAATTT-CGCTCT

CN 120017 ------------------CCAATTTCGC---CGTTTGTGAAAAGCCCCAATTT-CGCCCT

CN 119205 ------------------------------------------------------------

DH55 ref genome -GCTGTATTATCAAGATTATAATTTTGCAAAAATTTATACAATATTTAATTTTTCGTGCT

09-CS0040 -ACTGTATTATCAAGATTATAATTTTGCAAAAATTTATACAATATTTAATTTTTCGTGCT

CN 113754 ATCTGTATTATCAAGATTATAATTTTGCAAAAATTTATACAATATTTAATTTTTCGTGCT

CO46 NCBI -GCTGTATTATCAAGATTATAATTTTGCAAAAATTTATACAATATTTAATTTTTCGTGCT

Jasper ATCTGTATTATCAAGATTATAATTTTGCAAAAATTTATACAATATTTAATTTTTCGTGCT

Joelle phyto -GCTGTATTATCAAGATTATAATTTTGCAAAAATTTATACAATATTTAATTTTTCGTGCT

Joelle NCBI -GCTGTATTATCAAGATTATAATTTTGCAAAAATTTATACAATATTTAATTTTTCGTGCT

CN 119243 ------------------------------------------------------------

CN 120025 ------------------------------------------------------------

Joelle AAFC ------------------------------------------------------------

CAM 241 AAATC-TCCATATCCATCGCAATTGATAGC-TCGAATCCATTTTTGAGGCATGTCTTTCT

17CS1133 AAATC-TCCATATCCATCGCAATTGATAGC-TCGAATCCATTTTTGAGGCATGTCTTTCT

CAM 236 AAATC-TCCATATCCATCGCAATTGATAAGCTCGAATCCATTTTTGAGGCATGTCTTTCT

Blaine Creek AAATC-TCCATATCCATCGCAATTGATAACTTCGAATCCATTTTTGAGGCATGTCTTTCT

CN 119300 AAATC-TCCATATCCATCGCAATTGATAGC-TCGAATCCATTTTTGAGGCATGTCTTTCT

Yellowstone AAATC-TCCATATCCATCGCAATTGATAGC-TCGAATCCATTTTTGAGGCATGTCTTTCT

Hoga AAATC-TCCATATCCATCGCAATTGATAGC-TCGAATCCATTTTTGAGGCATGTCTTTCT

CN 120027 AAATC-TCCATATCCATCGCAATTGATAGC-TCGAATCCATTTTTGAGGCATGTCTTTCT

CN 120030 AAATC-TCCATATCCATCGCAATTGATAGC-TCGAATCCATTTTTGAGGCATGTCTTTCT

CN 119294 AAATC-TCCATATCCATCGCAATTGATAGC-TCGAATCCATTTTTGAGGCATGTCTTTCT

CN 120013 AAATC-TCCATATCCATCGCAATTGATAGC-TCGAATCCATTTTTGAGGCATGTCTTTCT

CN 120017 AAATC-TCCATATCCATCGCAATTGATAGC-TCGAATCCATTTTTGAGGCATGTCTTTCT

CN 119205 ------------------------------------------------------------

DH55 ref genome ATGTTATACATATCTAATATAGCTGTTG---CGAAATGCTGTTT-------TATCTTGTT

09-CS0040 ATGTTATACATATCTAATATAGTC-ATGTTGCGAAATGCTTGTTT------TATCTTGTT

CN 113754 ATGTTATACATATCTAATATAGCTGTTG---CGAAATGCTGTTT-------TATCTTGTT

CO46 NCBI ATGTTATACATATCTAATATAGCTGTTG---CGAAATGCTGTTT-------TATCTTGTT

Jasper ATGTTATACATATCTAATATAGCTGTTG---CGAAATGCTGTTT-------TATCTTGTT

Joelle phyto ATGTTATACATATCTAATATAGCTGTTG---CGAAATGCTGTTT-------TATCTTGTT

Joelle NCBI ATGTTATACATATCTAATATAGCTGTTG---CGAAATGCTGTTT-------TATCTTGTT

CN 119243 ------------------------------------------------------------

CN 120025 ------------------------------------------------------------

Joelle AAFC ------------------------------------------------------------

CAM 241 TCTTCCTCCTA----ATTTCG-TTATTTGTTTCGATTATTTGTGATTTCA-AA-CAGCTT

17CS1133 TCTTCCTCCTA----ATTTCG-TTATTTGTTTCGATTATTTGTGATTTCA-AA-CAGCTT

CAM 236 TCTTCCTCCTA----ATTTCG-TTATTTGTTTCGATTATTTGTGATTTCA-AA-CAGCTT

Blaine Creek TCTTCCTCCTA----ATTTCG-TTATTTGTTTCGATTATTTGTGATTTCA-AA-CAGCTT

CN 119300 TCTTCCTCCTA----ATTTCG-TTATTTGTTTCGATTATTTGTGATTTCA-AA-CAGCTT

Yellowstone TCTTCCTCCTA----ATTTCG-TTATTTGTTTCGATTATTTGTGATTTCA-AA-CAGCTT

Hoga TCTTCCTCCTA----ATTTCG-TTATTTGTTTCGATTATTTGTGATTTCA-AA-CAGCTT

CN 120027 TCTTCCTCCTA----ATTTCG-TTATTTGTTTCGATTATTTGTGATTTCA-AA-CAGCTT

CN 120030 TCTTCCTCCTA----ATTTCG-TTATTTGTTTCGATTATTTGTGATTTCA-AA-CAGCTT

CN 119294 TCTTCCTCCTA----ATTTCG-TTATTTGTTTCGATTATTTGTGATTTCA-AA-CAGCTT

CN 120013 TCTTCCTCCTA----ATTTCGATTATTTGTTTCGATTATTTGTGATTTCA-AA-CAGCTT

CN 120017 TCTTCCTCCTA----ATTTCG-TTATTTGTTTCGATTATTTGTGATTTCA-AA-CAGCTT

CN 119205 ------------------------------------------------------------

DH55 ref genome TACT-GTGCTATGGTATATTCTTTTATCATAACGTTTACCTGTACTTTTATAG-CATTTT

09-CS0040 CTATGTCGTTATGGTATATTCTTTTATCATAACGTTTACCTGTACTTTTATAG-CATTTT

CN 113754 TACT-GTGCTATGGTATATTCTTTTATCATAACGTTTACTTGTACTTTTATAAGCATTTT

CO46 NCBI TACT-GTGCTATGGTATATTCTTTTATCATAACGTTTACCTGTACTTTTATAG-CATTTT

Jasper TACTCGTGCTATGGTATATTCTTTTATCATAACGTTTACCTGTACTTTTATAG-CATTTT

Joelle phyto TACT-GTGCTATGGTATATTCTTTTATCATAACGTTTACCTGTACTTTTATAG-CATTTT

Joelle NCBI TACT-GTGCTATGGTATATTCTTTTATCATAACGTTTACCTGTACTTTTATAG-CATTTT

CN 119243 ------------------------------------------------------------

CN 120025 ------------------------------------------------------------

Joelle AAFC ------------------------------------------------------------

CAM 241 T-TGAT-TTCGATAAGGGTC-GATTTAGTTATCTCGTTTTGTTAG-GGTTCGATTTATCT

17CS1133 T-TGAT-TTCGATAAGGGTC-GATTTAGTTATCT-GTTTTGTTAG-GGTTCGATTTATCT

CAM 236 T-TGAT-TTCGATAAGGGTC-GATTTAGTTATCT-GTTTTGTTAG-GGTTCGATTTATCT

Blaine Creek T-TGAT-TTCGATAAGGGTC-GATTTAGTTATCT-GTTTTGTTAGTGGTTCGATTTATCT

CN 119300 T-TGAT-TTCGATAAGGGTC-GATTTAGTTATCTCGTTTTGTTAG-GGTTCGATTTATCT

Yellowstone T-TGAT-TTCGATAAGGGTC-GATTTAGTTATCTCGTTTTGTTAG-GGTTCGATTTATCT

Hoga T-TGAT-TTCGATAAGGGTCAGATTTAGTTATCTCGTTTTGTTAG-GGTTCGATTTATCT

CN 120027 T-TGAT-TTCGATAAGGGTC-GATTTAGTTATCTCGTTTTGTTAGTGGTTCGATTTATCT

CN 120030 T-TGAT-TTCGATAAGGGTCAGATTTAGTTATCT-GTTTTGTTAG-GGTTCGATTTATCT

CN 119294 T-TGAT-TTCGATAAGGGTCAGATTTAGTTATCTCGTTTTGTTAG-GGTTCGATTTATCT

CN 120013 T-TGAT-TTCGATAAGGGTC-GATTTAGTTATCT-GTTTTGTTAG-GGTTCGATTTATCT

CN 120017 T-TGAT-TTCGATAAGGGTCAGATTTAGTTATCT-GTTTTGTTAG-GGTTCGATTTATCT

CN 119205 ------------------------------------------------------------

DH55 ref genome C-TGATATGTGATTATAGCA-----CACATAACCTGTTTTATAAA---------------

09-CS0040 CTCGATATGTGATTATAGCA-----CACATAACCTGTTTTATAAA---------------

CN 113754 CTCGATATGTGATTATAAGCACACATA---ACCTTGTTTTATAAA---------------

CO46 NCBI C-TGATATGTGATTATAGCA-----CACATAACCTGTTTTATAAA---------------

Jasper C-CGATATGTGATTATAGCG----ACACATAACCTGTTTTATAAA---------------

Joelle phyto C-TGATATGTGATTATAGCA-----CACATAACCTGTTTTATAAA---------------

Joelle NCBI C-TGATATGTGATTATAGCA-----CACATAACCTGTTTTATAAA---------------

CN 119243 ------------------------------------------------------------

CN 120025 ------------------------------------------------------------

Joelle AAFC ------------------------------------------------------------

CAM 241 AGATTTATTTCGTTATCT-GTTTTTTAG-GGTTCGATTATTTAC-------AGATTTTGA

17CS1133 AGATTTATTTCGTTATCT--GTTTTTAG-GGTTCGATTATTTAC-------AGATTTTGA

CAM 236 AGATTTATTTCGTTATCT-GTTTTTTAGTGGTTCGATTATTTAC-------AGATTTTGA

Blaine Creek AGATTTATTTCGTTATCC-GTTTTTTAGTGGTTCGATTATTTAC-------AGATTTTGA

CN 119300 AGATTTATTTCGTTATCT-GTTTTTAGT-GGTTCGATTATTTAC-------AGATTTTGA

Yellowstone AGATTTATTTCGTTATCT-GTTTTTTAG-GGTTCGATTATTTAC-------AGATTTTGA

Hoga AGATTTATTTCGTTATCT-GTTTTTTAG-GGTTCGATTATTTAC-------AGATTTTGA

CN 120027 AGATTTATTTCGTTATCT-GTTTTTTAG-GGTTCGATTATTTAC-------AGATTTTGA

CN 120030 AGATTTATTTCGTTATCTCGTTTTTTAG-GGTTCGATTATTTAC-------TGATTTTGA

CN 119294 AGATTTATTTCGTTATCT-GTTTTTTAG-GGTTCGATTATTTAC-------AGATTTTGA

CN 120013 AGATTTATTTCGTTATCT-CGTTTTTAG-GGTTCGATTATTTAC-------AGATTTTGA

CN 120017 AGATTTATTTCGTTATCT-GTTTTTTAG-GGTTCGATTATTTAC-------AGATTTTGA

CN 119205 ------------------------------------------------------------

DH55 ref genome -------------------ATCTCATAA-AATCTGATTAATAAACGTCTC-AGATCCAAA

09-CS0040 -------------------ATCTCATAA-AATCTGATTAATAAACGTCTC-AGATCCAAA

CN 113754 -------------------ATCTCATAA-AATCTGATTAATAAACGTCTCGAGATCCAAA

CO46 NCBI -------------------ATCTCATAA-AATCTGATTAATAAACGTCTC-AGATCCAAA

Jasper -------------------ATCTCATAA-AATCTGATTAATAAACGTCTC-AGATCCAAA

Joelle phyto -------------------ATCTCATAA-AATCTGATTAATAAACGTCTC-AGATCCAAA

Joelle NCBI -------------------ATCTCATAA-AATCTGATTAATAAACGTCTC-AGATCCAAA

CN 119243 ------------------------------------------------------------

CN 120025 ------------------------------------------------------------

Joelle AAFC ------------------------------------------------------------

CAM 241 GAATGT----GGAGTTTCGAATCCATCT-GTTT--TGTTAGTGGTTCGATTATTTACAGA

17CS1133 GAATGT----GGAGTTTCGAATCCATCT-GTTT--TGTTAGGGT-TCGATTATTTACAGA

CAM 236 GAATGT----GGAGTTTCGAATCCATCT-GTTT--TGTTAGGGT-TCGATTATTTACAGA

Blaine Creek GAATGT----GGAGTTTCGAATCCATCTCGTTT--TGTTAGGGT-TCGATTATTTACGGA

CN 119300 GAATGT----GGAGTTTCGAATCCATCT-GTTT--TGTTAGGGT-TCGATTATTTACAGA

Yellowstone GAATGT----GGAGTTTCGAATCCATCT-GTTT--TGTTAGGGT-TCGATTATTTACAGA

Hoga GAATGT----GGAGTTTCGAATCCATCT-GTTT--TGTTAGGGT-TCGATTATTTACAGA

CN 120027 GAATGT----GGAGTTTCGAATCCATCT-GTTT--TGTTAGGGT-TCGATTATTTACAGA

CN 120030 GAATGT----GGAGTTTCGAATCCATCT-GTTT--TGTTAGGGT-TCGATTATTTACAGA

CN 119294 GAATGT----GGAGTTTCGAATCCATCT-GTTT--TGTTAGGGT-TCGATTATTTACAGA

CN 120013 GAATGT----GGAGTTTCGAATCCATCT-GTTT--TGTTAGGGT-TCGATTATTTACAGA

CN 120017 GAATGT----GGAGTTTCGAATCCATCT-GTTT--TGTTAGGGT-TCGATTATTTACAGA

CN 119205 ------------------------------------------------------------

DH55 ref genome ATACATAACAAAAGTCTCAAGAACATCA-GTTTACCATCAAAGTCTCAAGAACATCCAAA

09-CS0040 ATACATAACAAAAGTCTCAAGAACATCA-GTTTACCATCAAAGTCTCAAGAACATCCAAA

CN 113754 ATACATAACAAAAGTCTCAAGAACATCA-GTTTACCATCAAAGTCTCAAGAACATCCAAA

CO46 NCBI ATACATAACAAAAGTCTCAAGAACATCA-GTTTACCATCAAAGTCTCAAGAACATCCAAA

Jasper ATACATAACAAAAGTCTCAAGAACATCA-GTTTACCATCAAAGTCTCAAGAACATCCAAA

Joelle phyto ATACATACCAAAAGTCTCAAGAACATCA-GTTTACCATCAAAGTCTCAAGAACATCCAAA

Joelle NCBI ATACATACCAAAAGTCTCAAGAACATCA-GTTTACCATCAAAGTCTCAAGAACATCCAAA

CN 119243 ------------------------------------------------------------

CN 120025 ------------------------------------------------------------

Joelle AAFC ------------------------------------------------------------

CAM 241 TTTGACTCAAAAAT---AGCTTTTGGATTTGAGTATTTGCGCCTTC----ATAAA-CTGA

17CS1133 TTTGACTCAAAAAT---AGCTTTTGGATTTGAGTATTTGCGCCTTC----ATAAA-CTGA

CAM 236 TTTGACTCAAAAAT---AGCTTTTGGATTTGAGTATTTGCGCCTTC----ATAAAGCTGA

Blaine Creek TTTGACTCAAAAAT---AGCTTTTGGATTTGAGTATTTGCGCCTTC----ATAAA-CTGA

CN 119300 TTTGACTCAAAAAT---AGCTTTTGGATTTGAGTATTTGCGCCTTC----ATAAA-CTGA

Yellowstone TTTGACTCAAAAAT---AGCTTTTGGATTTGAGTATTTGCGCCTTC----ATAAA-CTGA

Hoga TTTGACTCAAAAAT---AGCTTTTGGATTTGAGTATTTGCGCCTTC----ATAAA-CTGA

CN 120027 TTTGACTCAAAAAT---AGCTTTTGGATTTGAGTATTTGCGCCTTC----ATAAA-CTGA

CN 120030 TTTGACTCAAAAAT---AGCTTTTGGATTTGAGTATTTGCGCCTTC----ATAAA-CTGA

CN 119294 TTTGACTCAAAAAT---AGCTTTTGGATTTGAGTATTTGCGCCTTC----ATAAA-CTGA

CN 120013 TTTGACTCAAAAAT---AGCTTTTGGATTTGAGTATTTGCGCCTTC----ATAAA-CTGA

CN 120017 TTTGACTCAAAAAT---AGCTTTTGGATTTGAGTATTTGCGCCTTC----ATAAA-CTGA

CN 119205 ------------------------------------------------------------

DH55 ref genome ATAAACAAGCAATCTCAAGTACTTAACATTACACAATAAAACCATC-AGTTTAAGACATA

09-CS0040 ATAAACAAGCAATCTCAAGTACTTAACATTACACAATAAAACCATC-GGTTTAAGACATA

CN 113754 ATAAACAAGCAATCTCAAGTACTTAACATTACACAATAAAACCATCGAGTTTAAGACATA

CO46 NCBI ATAAACAAGCAATCTCAAGTACTTAACATTACACAATAAAACCATC-AGTTTAAGACATA

Jasper ATAAACAAGCAATCTCAAGTACTTAACATTACACAATAAAACCATC-AGTTTAAGACATA

Joelle phyto ATAAACAAGCAATCTCAAGTACTTAACATTACACAATAAAACCATC-AGTTTAAGACATA

Joelle NCBI ATAAACAAGCAATCTCAAGTACTTAACATTACACAATAAAACCATC-AGTTTAAGACATA

CN 119243 ------------------------------------------------------------

CN 120025 ------------------------------------------------------------

Joelle AAFC ------------------------------------------------------------

CAM 241 ACGA---TTTTTTTTTCCCAAGTGTTGGTG---------------------------ATT

17CS1133 ACGA----TTTTTTTTCCCAAGTGTTGGTG---------------------------ATT

CAM 236 ACGA---TTTTTTTTTCCCAAGTGTTGGTG---------------------------ATT

Blaine Creek ACGA---TTTTTTTTTCCCAAGTGTCGGTG---------------------------ATT

CN 119300 ACGA---TTTTTTTTTCCCAAGTGTTGGTG---------------------------ATT

Yellowstone ACGA---TTTTTTTTTCCCAAGTGTTGGTG---------------------------ATT

Hoga ACGA---TTTTTTTTTCCCAAGTGTTGGTG---------------------------ATT

CN 120027 ACGA--TTTTTTTTTTCCCAAGTGTTGGTG---------------------------ATT

CN 120030 ACGA--TTTTTTTTTTCCCAAGTGTTGGTG---------------------------ATT

CN 119294 ACGATTTTTTTTTTTTCCCAAGTGTTGGTG---------------------------ATT

CN 120013 ACGA---TTTTTTTTTCCCAAGTGTTGGTG---------------------------ATT

CN 120017 ACGA---TTTTTTTTTCCCAAGTGTTGGTG---------------------------ATT

CN 119205 ------------------------------------------------------------

DH55 ref genome ATGACCTTGTTCTCTGGCCAAGCAATGCAGGAGCTAAGTGCATCTTCAATAATCTCTATT

09-CS0040 ATGACCTTGTTCTCCGGCCAAGCAATGCAGGAGCTAAGTGCATCTTCAATAATCTCTATT

CN 113754 ATGACCTTGTTCTCT-GCCAAGCAATGCAGGAGCTAAGTGCATCTTCAATAATCTCTATT

CO46 NCBI ATGACCTTGTTCTCTGGCCAAGCAATGCAGGAGCTAAGTGCATCTTCAATAATCTCTATT

Jasper ATGACCTTGTTCTCC-GCCAAGCAATGCAGGAGCTAAGTGCATCTTCAATAATCTCTATT

Joelle phyto ATGACCTTGTTCTCTGGCCAAGCAATGCAGGAGCTAAGTGCATCTTCAATAATCTCTATT

Joelle NCBI ATGACCTTGTTCTCTGGCCAAGCAATGCAGGAGCTAAGTGCATCTTCAATAATCTCTATT

CN 119243 ------------------------------------------------------------

CN 120025 ------------------------------------------------------------

Joelle AAFC ------------------------------------------------------------

CAM 241 GAAATGTTGGGGAACCGAAGCTTCTCATTTCTCTCGTACTCACACAACAGAGTTAGTATC

17CS1133 GAAATGTTGGGGAACTGAAGCTTCTCATTTCTCT-GTACTCACACAACAG-GTTAGTATC

CAM 236 GAAATGTTGGGGAACTGAAGCTTCTCATTTCTCTCGTACTCACACAACAG-GTTAGTATC

Blaine Creek GAAATGTCGGGGAACCGAAGCTTCTCATTTCTCC-GTACTCACACAACAGAGTTAGTATC

CN 119300 GAAATGTTGGGGAACCGAAGCTTCTCATTTCTCC-GTACTCACACAACAG-GTTAGTATC

Yellowstone GAAATGTTGGGGAACCGAAGCTTCTCATTTCTCT-GTACTCACACAACAG-GTTAGTATC

Hoga GAAATGTTGGGGAACTGAAGCTTCTCATTTCTCT-GTACTCACACAACAGAGTTAGTATC

CN 120027 GAAATGTTGGGGAACTGAAGCTTCTCATTTCTCC-GTACTCACACAACAG-GTTAGTATC

CN 120030 GAAATGTTGAGGAACTGAAGCTTCTCATTTCTCT-GTACTCACACAACAG-GTTAGTATC

CN 119294 GAAATGTTGGGGAACTGAAGCTTCTCATTTCTCC-GTACTCACACAACAG-GTTAGTATC

CN 120013 GAAATGTTGGGGAACTGAAGCTTCTCATTTCTCT-GTACTCACACAACAG-GTTAGTATC

CN 120017 GAAATGTTGGGGAACTGAAGCTTCTCATTTCTCT-GTACTCACACAACAG-GTTAGTATC

CN 119205 ------------------------------------------------------------

DH55 ref genome TCATCGGATGGCCTCCAAACTTTTACATTTTTC----ACCTTCACAACA-----------

09-CS0040 TCATCGGATGGCCTCCAAACTTTTACATTTTTC----ACCTTCACAACA-----------

CN 113754 TCATCGGATGGCCTCCAAACTTTTACATTTTTC----ACCTTCACAACA-----------

CO46 NCBI TCATCGGATGGCCTCCAAACTTTTACATTTTTC----ACCTTCACAACA-----------

Jasper TCATCGGATGGCCTCCAAACTTTTACATTTTTC----ACCTTCACAACA-----------

Joelle phyto TCATCGGATGGCCTCCAAACTTTTACATTTTTC----ACCTTCACAACA-----------

Joelle NCBI TCATCGGATGGCCTCCAAACTTTTACATTTTTC----ACCTTCACAACA-----------

CN 119243 ------------------------------------------------------------

CN 120025 ------------------------------------------------------------

Joelle AAFC ------------------------------------------------------------

CAM 241 ACTCAACCAATAGTTAAACATTATTATTACACA--CGAATATTGGTGATTGCTATGTTTT

17CS1133 ACTCAACCAATAGTTAAACATTATTATTACACA--CGAATATTGGTGATTGCTATGTTTT

CAM 236 ACTCAACCAATAGTTAAACATTATTATTACACA--CGAATATTGGTGATTGCTATGTTTT

Blaine Creek ACTCAACCAATAGTTAAACATTATTATTACACA--CGAATATTGGTGATTGCTATGTTTT

CN 119300 ACTCAACCAATAGTTAAACATTATTATTACACA--CGAATATTGGTGATTGCTATGTTTT

Yellowstone ACTCAACCAATAGTTAAACATTATTATTACACA--CGAATATTGGTGATTGCTATGTTTT

Hoga ACTCAACCAATAGTTAAACATTATTATTACACA--CGAATATTGGTGATTGCTATGTTTT

CN 120027 ACTCAACCAATAGTTAAACATTATTATTACACA--CGAATATTGGTGATTGCTATGTTTT

CN 120030 ACTCAACCAATAGTTAAACATTATTATTACACA--CGAATATTGGTGATTGCTATGTTTT

CN 119294 ACTCAACCAATAGTTAAACATTATTATTACACA--CGAATATTGGTGATTGCTATGTTTT

CN 120013 ACTCAACCAATAGTTAAACATTATTATTACACA--CGAATATTGGTGATTGCTATGTTTT

CN 120017 ACTCAACCAATAGTTAAACATTATTATTACACA--CGAATATTGGTGATTGCTATGTTTT

CN 119205 ------------------------------------------------------------

DH55 ref genome --TCTATCCACACTTTAACTGCATTAGAACCCAAGCGAGTA-AAGTGAACCTTATGTTCT

09-CS0040 --TCTATCCACACTTTAACTGCATTAGAACCCAAGCGAGTA-AAGTGAACCTTATGTTCT

CN 113754 --TCTATCCACACTTTAACTGCATTAGAACCCAAGCGAGTA-AAGTGAACCTTATGTTCG

CO46 NCBI --TCTATCCACACTTTAACTGCATTAGAACCCAAGCGAGTA-AAGTGAACCTTATGTTCT

Jasper --TCTATCCACACTTTAACTGCATTAGAACCCA-GCGAGTA-AAGTGAACCTTATGTTCT

Joelle phyto --TCTATCCACACTTTAACTGCATTAGAACCCAAGCGAGTA-AAGTGAACCTTATGTTCT

Joelle NCBI --TCTATCCACACTTTAACTGCATTAGAACCCAAGCGAGTA-AAGTGAACCTTATGTTCT

CN 119243 ------------------------------------------------------------

CN 120025 ------------------------------------------------------------

Joelle AAFC ------------------------------------------------------------

CAM 241 G--TCCTTTGAGTCTGAT--TCGTATAAATAGTGATTAGATTAGTGAGATTTTACACAGT

17CS1133 G--TCCTTTGAGTCTGAT--TCGTATAAATAGTGATTAGATTAGTGAGATTTTACACAGT

CAM 236 G--TCCTTTGAGTCTGAT--TCGTATAAATAGTGATTAGATTAGTGAGATTTTACACAGT

Blaine Creek G--TCCTTTGAGTCCGAT--TCGTATAAATAGTGATTAGATTAGTGAGATTTTACACAGT

CN 119300 G--TCCTTTGAGTCTGAT--TCGTATAAATAGTGATTAGATTAGTGAGATTTTACACAGT

Yellowstone G--TCCTTTGAGTCTGAT--TCGTATAAATAGTGATTAGATTAGTGAGATTTTACACAGT

Hoga G--TCCTTTGAGTCTGAT--TCGTATAAATAGTGATTAGATTAGTGAGATTTTACACAGT

CN 120027 G--TCCTTTGAGTCTGAT--TCGTATAAATAGTGATTAGATTAGTGAGATTTTACACAGT

CN 120030 G--TCCTTTGAGTCTGAT--TCGTATAAATAGTGATTAGATTAGTGAGATTTTACACAGT

CN 119294 G--TCCTTTGAGTCTGAT--TCGTATAAATAGTGATTAGATTAGTGAGATTTTACACAGT

CN 120013 G--TCCTTTGAGTCTGAT--TCGTATAAATAGTGATTAGATTAGTGAGATTTTACACAGT

CN 120017 G--TCCTTTGAGTCTGAT--TCGTATAAATAGTGATTAGATTAGTGAGATTTTACACAGT

CN 119205 ------------------------------------------------------------

DH55 ref genome GGATCATTTGTTCCCCATCGTCCTTCAGCCA----------CAACCCTATTCTTTTCAGT

09-CS0040 GGATCATTTGTTCCCCATCGTCCTTCAGCCA----------CAACCCTATTCTTTTCAGT

CN 113754 G-ATCATTTGTTCCCCATCGTCCTTCAGCCA----------CAACCCTATTCTTTTCAGT

CO46 NCBI GGATCATTTGTTCCCCATCGTCCTTCAGCCA----------CAACCCTATTCTTTTCAGT

Jasper GGATCATTTGTTCCCCATCGTCCTTCAGCCA----------CAACCCTATTCTTTTCAGT

Joelle phyto GGATCATTTGTTCCCCATCGTCCTTCAGCCA----------CAACCCTATTCTTTTCAGT

Joelle NCBI GGATCATTTGTTCCCCATCGTCCTTCAGCCA----------CAACCCTATTCTTTTCAGT

CN 119243 ------------------------------------------------------------

CN 120025 ------------------------------------------------------------

Joelle AAFC ------------------------------------------------------------

CAM 241 TAGAT--ATTGGTTTAAGAGCATCCGTATGTGTCCG-AAGTAGTGCAAGTATTGTCTCGA

17CS1133 TAGAT--ATTGGTTTAAGAGCATCTGTATGTGTATGCAAGTAGTGC-AGTATTGTC-CGA

CAM 236 TAGAT--ATTGGTTTAAGAGCATCCGTATGTGTCTG-AAGTAGTGC-AGTATTGTCTCGA

Blaine Creek TAGAT--ATTGGTTTAAGAGCATCCGTATGTGTCCG-AAGTAGTGCGAGTATTGTC-CGA

CN 119300 TAGAT--ATTGGTTTAAGAGCATCCGTATGTGTCCG-AAGTAGTGC-AGTATTGTCTCGA

Yellowstone TAGAT--ATTGGTTTAAGAGCATCCGTATGTGTCTC-AAGTAGTGC-AGTATTGTC-CGA

Hoga TAGAT--ATTGGTTTAAGAGCATCCGTATGTGTCTCGAAGTAGTGC-AGTATTGTC-CGA

CN 120027 TAGAT--ATTGGTTTAAGAGCATCCGTATGTGTCCG-AAGTAGTGC-AGTATTGTC-CGA

CN 120030 TAGAT--ATTGGTTTAAGAGCATCCGTATGTGTCTGCAAGTAGTGC-AGTATTGTC-TGA

CN 119294 TAGAT--ATTGGTTTAAGAGCATCCGTATGTGTCCG-AAGTAGTGC-AGTATTGTCTCGA

CN 120013 TAGAT--ATTGGTTTAAGAGCATCTGTATGTGTCTG-AAGTAGTGC-GGTATTGTC-CGA

CN 120017 TAGAT--ATTGGTTTAAGAGCATCCGTATGTGTGCG-AAGTAGTGC-AGTATTGTC-CGA

CN 119205 ------------------------------------------------------------

DH55 ref genome TAAATCCATCAACTT--GCACTTCTTATTCTCCTTGGAAATAACTTTATTAATGCGCTGC

09-CS0040 TAAATCCATCAACTT--GCACTTCTTATTCTCCTTGGAAATAACTTTATTAATGCGCTGC

CN 113754 TAAATCCATCAACTT--GCACTTCTTATTCTCCTTGGAAATAACTTTATTAATGCGCTGC

CO46 NCBI TAAATCCATCAACTT--GCACTTCTTATTCTCCTTGGAAATAACTTTATTAATGCGCTGC

Jasper TAAATCCATCAACTT--GCACTTCTTATTCTCCTTGGAAATAACTTTATTAATGCGCTGC

Joelle phyto TAAATCCATCAACTT--GCACTTCTTATTCTCCTTGGAAATAACTTTATTAATGCGCTGC

Joelle NCBI TAAATCCATCAACTT--GCACTTCTTATTCTCCTTGGAAATAACTTTATTAATGCGCTGC

CN 119243 ------------------------------------------------------------

CN 120025 ------------------------------------------------------------

Joelle AAFC ------------------------------------------------------------

CAM 241 ATTGTTGCTTAATGTGAGCTTTGTATAATATGTATCTCTATTTTATCTATAAAGGTTAAA

17CS1133 ATTGTTGCTTAATGTGAGCTTTGTATAATATGTATCTCTATTTTATCTATAAAGGTTAAA

CAM 236 ATTGTTGCTTAATGTGAGCTTTGTATAATATGTATCTCTATTTTATCTATAAAGGTTAAA

Blaine Creek ATTGTTGCTTAATGTGAGCTTTGTATAATATGTATCTCTATTTTATCTATAAAGGTTAAA

CN 119300 ATTGTTGCTTAATGTGAGCTTTGTATAATATGTATCTCTATTTTATCTATAAAGGTTAAA

Yellowstone ATTGTTGCTTAATGTGAGCTTTGTATAATATGTATCTCTATTTTATCTATAAAGGTTAAA

Hoga ATTGTTGCTTAATGTGAGCTTTGTATAATATGTATCTCTATTTTATCTATAAAGGTTAAA

CN 120027 ATTGTTGCTTAATGTGAGCTTTGTATAATATGTATCTCTATTTTATCTATAAAGGTTAAA

CN 120030 ATTGTTGCTTAATGTGAGCTTTGTATAATATGTATCTCTATTTTATCTATAAAGGTTAAA

CN 119294 ATTGTTGCTTAATGTGAGCTTTGTATAATATGTATCTCTATTTTATCTATAAAGGTTAAA

CN 120013 ATTGTTGCTTAATGTGAGCTTTGTATAATATGTATCTCTATTTTATCTATAAAGGTTAAA

CN 120017 ATTGTTGCTTAATGTGAGCTTTGTATAATATGTATCTCTATTTTATCTATAAAGGTTAAA

CN 119205 ------------------------------------------------------------

DH55 ref genome CATGTT-----------------TACAATATAAACCATCAGTCATCATATAATTAACAAA

09-CS0040 CATGTT-----------------TACAATATAAACCATCAGTCATCATATAATTAACAAA

CN 113754 CATGTT-----------------TACAATATAAACCATCAGTCATCATATAATTAACAAA

CO46 NCBI CATGTT-----------------TACAATATAAACCATCAGTCATCATATAATTAACAAA

Jasper CATGTT-----------------TACAATATAAACCATCAGTCATCATATAATTAACAAA

Joelle phyto CATGTT-----------------TACAATATAAACCATCAGTCATCATATAATTAACAAA

Joelle NCBI CATGTT-----------------TACAATATAAACCATCAGTCATCATATAATTAACAAA

CN 119243 ------------------------------------------------------------

CN 120025 ------------------------------------------------------------

Joelle AAFC ------------------------------------------------------------

CAM 241 ATTTATATTATAACATATAA-------TGTTGGACAAGGCATGGGTGCAT-CTATGCAGG

17CS1133 ATTTATATTATAACATATAA-------TGTTGGACAAGGCATGGGTGCAT-CTATGCAGG

CAM 236 ATTTATATTATAACATATAA-------TGTTGGACAAGGCATGGGTGCAT-CTATGCAGG

Blaine Creek ATTTATATTATAACATATAA-------TGTTGGACAAGGCATGGGTGCAT-CTATGCA-G

CN 119300 ATTTATATTATAACATATAA-------TGTTGGACAAGGCATGGGTGCAT-CTATGCAGG

Yellowstone ATTTATATTATAACATATAA-------TGTTGGACAAGGCATGGGTGCAT-CTATGCAGG

Hoga ATTTATATTATAACATATAA-------TGTTGGACAAGGCATGGGTGCAT-CTATGCAGG

CN 120027 ATTTATATTATAACATATAA-------TGTTGGACAAGGCATGGGTGCAT-CTATGCAGG

CN 120030 ATTTATATTATAACATATAA-------TGTTGGACAAGGCATGGGTGCAT-CTATGCAGG

CN 119294 ATTTATATTATAACATATAA-------TGTTGGACAAGGCATGGGTGCAT-CTATGCAGG

CN 120013 ATTTATATTATAACATATAA-------TGTTGGACAAGGCATGGGTGCAT-CTATGCAGG

CN 120017 ATTTATATTATAACATATAA-------TGTTGGACAAGGCATGGGTGCAT-CTATGCGGG

CN 119205 ------------------------------------------------------------

DH55 ref genome AACAACTTCATAACATATGAAAACACTTACTGGAGACTTCCTGAGAGGAGACTGTGATGG

09-CS0040 AACAACTTCATAACATATGAAAACACTTACTGGAGACTTC-CGAGAGGAGACTGTGATGG

CN 113754 AACAACTTCATAACATATGAAAACACTTACTCGGAGACTTCCGAGAGGAGACTGTGATGG

CO46 NCBI AACAACTTCATAACATATGAAAACACTTACTGGAGACTTCCTGAGAGGAGACTGTGATGG

Jasper AACAACTTCATAACATATGAAAACACTTACTGGAGACTTCCTGAGAGGAGACTGTGATGG

Joelle phyto AACAACTTCATAACATATGAAAACACTTACTGGAGACTTCCTGAGAGGAGACTGTGATGG

Joelle NCBI AACAACTTCATAACATATGAAAACACTTACTGGAGACTTCCTGAGAGGAGACTGTGATGG

CN 119243 ------------------------------------------------------------

CN 120025 ------------------------------------------------------------

Joelle AAFC ------------------------------------------------------------

CAM 241 TAAGGAATTATATTTCATAATTAGGTTTTATTTTGGGTTTGGATACTTTAAGATGTTC-T

17CS1133 TAAGGAATTATATTTCATAATTAGGTTTTATTTTGGGTTTGGATACTTTAAGATGTTC-T

CAM 236 TAAGGAATTATATTTCATAATTAGGTTTTATTTTGGGTTTGGATACTTTAAGATGTTC-T

Blaine Creek TAAGGAATTATATTTCATAATTAGGTTTTATTTTGGGTTTGGATACTTTAAGATGTTCTT

CN 119300 TAAGGAATTATATTTCATAATTAGGTTTTATTTTGGGTTTGGATACTTTAAGATGTTC-T

Yellowstone TAAGGAATTATATTTCATAATTAGGTTTTATTTTGGGTTTGGATACTTTAAGATGTTC-T

Hoga TAAGGAATTATATTTCATAATTAGGTTTTATTTTGGGTTTGGATACTTTAAGATGTTC-T

CN 120027 TAAGGAATTATATTTCATAATTAGGTTTTATTTTGGGTTTGGATACTTTAAGATGTTC-T

CN 120030 TAAGGAATTATATTTCATAATTAGGTTTTATTTTGGGTTTGGATACTTTAAGATGTTC-T

CN 119294 TAAGGAATTATATTTCATAATTAGGTTTTATTTTGGGTTTGGATACTTTAAGATGTTC-T

CN 120013 TAAGGAATTATATTTCATAATTAGGTTTTATTTTGGGTTTGGATACTTTAAGATGTTC-T

CN 120017 TAAGGAATTATATTTCATAATTAGGTTTTATTTTGGGTTTGGATACTTTAAGATGTTC-T

CN 119205 ------------------------------------------------------------

DH55 ref genome TAAAGTATT--------------------------------------------------C

09-CS0040 TAAAGTATT--------------------------------------------------C

CN 113754 TAAAGTATT--------------------------------------------------C

CO46 NCBI TAAAGTATT--------------------------------------------------C

Jasper TAAAGTATT--------------------------------------------------C

Joelle phyto TAAAGTATT--------------------------------------------------C

Joelle NCBI TAAAGTATT--------------------------------------------------C

CN 119243 ------------------------------------------------------------

CN 120025 ------------------------------------------------------------

Joelle AAFC ------------------------------------------------------------

CAM 241 TTTAGTTATGATTTTTTTCTTTAATTATCCATTGAAGAGCTGATCCTGGTTATAAAAGTG

17CS1133 TTTAGTTATGATTTTTTTCTTTAATTATCCATTGAAGAGCTGATCCTGGTTATAAAAGTG

CAM 236 TTTAGTTATGATTTTTTTCTTTAATTATCCATTGAAGAGCTGATCCTGGTTATAAAAGTG

Blaine Creek TTTAGTTATGATTTTTTTCTTTAATTATCCATTGAAGAGCTGATCCTGGTTATAAAAGTG

CN 119300 TTTAGTTATGATTTTTTTCTTTAATTATCCATTGAAGAGCTGATCCTGGTTATAAAAGTG

Yellowstone TTTAGTTATGATTTTTTTCTTTAATTATCCATTGAAGAGCTGATCCTGGTTATAAAAGTG

Hoga TTTAGTTATGATTTTTTTCTTTAATTATCCATTGAAGAGCTGATCCTGGTTATAAAAGTG

CN 120027 TTTAGTTATGATTTTTTTCTTTAATTATCCATTGAAGAGCTGATCCTGGTTATAAAAGTG

CN 120030 TTTAGTTATGATTTTTTTCTTTAATTATCCATTGAAGAGCTGATCCTGGTTATAAAAGTG

CN 119294 TTTAGTTATGATTTTTTTCTTTAATTATCCATTGAAGAGCTGATCCTGGTTATAAAAGTG

CN 120013 TTTAGTTATGATTTTTTTCTTTAATTATCCATTGAAGAGCTGATCCTGGTTATAAAAGTG

CN 120017 TTTAGTTATGATTTTTTTCTTTAATTATCCATTGAAGAGCTGATCCTGGTTATAAAAGTG

CN 119205 ------------------------------------------------------------

DH55 ref genome TTTATTGGTGATATT---------GGACCCGTTGCA-------------TCATCAACAGG

09-CS0040 TTTATTGGTGATATT---------GGACCCGTTGCA-------------TCATCAACAGG

CN 113754 TTTATTGGTGATATT---------GGACCCGTTGCA-------------TCATCAACAGG

CO46 NCBI TTTATTGGTGATATT---------GGACCCGTTGCA-------------TCATCAACAGG

Jasper TTTATTGGTGATATT---------GGACCCGTTGCA-------------TCATCAACAGG

Joelle phyto TTTATTGGTGATATT---------GGACCCGTTGCA-------------TCATCAACAGG

Joelle NCBI TTTATTGGTGATATT---------GGACCCGTTGCA-------------TCATCAACAGG

CN 119243 ------------------------------------------------------------

CN 120025 ------------------------------------------------------------

Joelle AAFC ------------------------------------------------------------

CAM 241 GAGCTTGGAAATTTGTAAGGGATGTGTCT-GCG-GCTTTAGGTGACATAGAAATGATAAT

17CS1133 GAGCTTGGAAATTTGTAAGGGATGTGTCTCGCG-GCTTTAGGTGACATAGAAATGATAAT

CAM 236 GAGCTTGGAAATTTGTAAGGGATGTGTCT-GCG-GCTTTAGGTGACATAGAAATGATAAT

Blaine Creek GAGCTTGGAAATTTGTAAGGGATGTGTCT-GCG-GCTTTAGGTGACATAGAAATGATAAT

CN 119300 GAGCTTGGAAATTTGTAAGGGATGTGTCT-GCG-GCTTTAGGTGACATAGAAATGATAAT

Yellowstone GAGCTTGGAAATTTGTAAGGGATGTGTCT-GCG-GCTTTAGGTGACATAGAAATGATAAT

Hoga GAGCTTGGAAATTTGTAAGGGATGTGTCT-GCG-GCTTTAGGTGACATAGAAATGATAAT

CN 120027 GAGCTTGGAAATTTGTAAGGGATGTGTCTCATG-GCTTTAGGTGACATAGAAATGATAAT

CN 120030 GAGCTTGGAAATTTGTAAGGGATGTGTCT-ACG-GCTTTAGGTGACATAGAAATGATAAT

CN 119294 GAGCTTGGAAATTTGTAAGGGATGTGTCT-GCG-GCTTTAGGTGACATAGAAATGATAAT

CN 120013 GAGCTTGGAAATTTGTAAGGGATGTGTCT-GCG-GCTTTAGGTGACATAGAAATGATAAT

CN 120017 GAGCTTGGAAATTTGTAAGGGATGTGTCT-GCG-GCTTTAGGTGACATAGAAATGATAAT

CN 119205 ------------------------------------------------------------

DH55 ref genome AGACTCAGAACCAGAT--------------GCA-GACTTAGAAGACATAG--ATGCAGAT

09-CS0040 AGACTCAGAACCAGAT--------------GCA-GACTTAGAAGACATAG--ATGCAGAT

CN 113754 AGACTCGGAACCGGAT--------------GCGAGACTTAGAAGACATAG--ATGCAGAT

CO46 NCBI AGACTCAGAACCAGAT--------------GCA-GACTTAGAAGACATAG--ATGCAGAT

Jasper AGACTCAGAACCAGAT--------------GCG-GACTTAGAAGACATAG--ATGCAGAT

Joelle phyto AGACTCAGAACCAGAT--------------GCA-GACTTAGAAGACATAG--ATGCAGAT

Joelle NCBI AGACTCAGAACCAGAT--------------GCA-GACTTAGAAGACATAG--ATGCAGAT

CN 119243 ------------------------------------------------------------

CN 120025 ------------------------------------------------------------

Joelle AAFC ------------------------------------------------------------

CAM 241 ATGTCCTT-GTAAAGACTGTCGTAATGT-AGTACTGACGG-TTAAACAG-TGTTGTGGTT

17CS1133 ATGTCCTT-GTAAAGACTGTCGTAATGT-AGTAC-GACAG-TTAAACAG-TGTTGTGGTT

CAM 236 ATGTCCTT-GTAAAGACTGTCGTAATGT-AGTAC-GACAG-TTAAACAGATGTTGTGGTT

Blaine Creek ATGTCCTTAGTAAAGACTGTCGTAATGTAAGTACTGACAGCTTAAACGA-TGTTGTGGTT

CN 119300 ATGTCCTT-GTAAAGACTGTCGTAATGT-AGTAC-GACAG-TTAAACAG-TGTTGTGGTT

Yellowstone ATGTCCTT-GTAAAGACTGTCGTAATGT-AGTAC-GACAG-TTAAACAG-TGTTGTGGTT

Hoga ATGTCCTT-GTAAAGACTGTCGTAATGT-AGTAC-GACAG-TTAAACAG-TGTTGTGGTT

CN 120027 ATGTCCTT-GTAAAGACTGTCGTAATGT-AGTAC-GACAG-TTAAACAG-TGTTGTGGTT

CN 120030 ATGTCCTT-GTAAAGACTGTCGTAATGT-AGTACTGACAG-TTAAACAG-TGTTGTGGTT

CN 119294 ATGTCCTT-GTAAAGACTGTCGTAATGT-AGTAC-GACAG-TTAAACAG-TGTTGTGGTT

CN 120013 ATGTCCTT-GTAAAGACTGTCGTAATGT-AGTAC-GACAG-TTAAACAG-TGTTGTGGTT

CN 120017 ATGTCCTT-GTAAAGACTGTCGTAATGT-AGTACTGACAG-TTAAACAG-TGTTGTGGTT

CN 119205 ------------------------------------------------------------

DH55 ref genome TTGGCCT--------------GCGATGC-AGCCTT------TGAAGCTGTTGCTGAAGTT

09-CS0040 TTGGCCT--------------GCGATGC-AGCCTT------TGAAGCTGTTGCTGAAGTT

CN 113754 TTGGCCT--------------GCGATGC-GGCCTT------TGAAGCTGTTGCTGAAGTT

CO46 NCBI TTGGCCT--------------GCGATGC-AGCCTT------TGAAGCTGTTGCTGAAGTT

Jasper TTGGCCT--------------GCGATGC-AGCCTT------TGAAGCTGTTGCTGAAGTT

Joelle phyto TTGGCCT--------------GCGATGC-AGCCTT------TGAAGCTGTTGCTGAAGTT

Joelle NCBI TTGGCCT--------------GCGATGC-AGCCTT------TGAAGCTGTTGCTGAAGTT

CN 119243 ------------------------------------------------------------

CN 120025 ------------------------------------------------------------

Joelle AAFC ------------------------------------------------------------

CAM 241 GAAGCATCTTGTAATAAGA-----------GGGATGGA-----------TGAGGCATACA

17CS1133 G-AGCATCTTGTAATAAGA-----------GGGATGGA-----------TGAGGCATACA

CAM 236 G-AGCATCTTGTAATAAGA-----------GGGATGGA-----------TGAGGCATACA

Blaine Creek G-AGCATCTTGTAATAAGA-----------GGGATGGA-----------TGAGGCATACA

CN 119300 G-AGCATCTTGTAATAAGA-----------GGGATGGA-----------TGAGGCATACA

Yellowstone G-AGCATCTTGTAATAAGA-----------GGGATGGA-----------TGAGGCATACA

Hoga G-AGCATCTTGTAATAAGA-----------GGGATGGA-----------TGAGGCATACA

CN 120027 G-AGCATCTTGTAATAAGA-----------GGGATGGA-----------TGAGGCATACA

CN 120030 G-AGCATCTTGTAATAAGA-----------GGGATGGA-----------TGAGGCATACA

CN 119294 G-AGCATCTTGTAATAAGA-----------GGGATGGA-----------TGAGGCATACA

CN 120013 G-AGCATCTTGTAATAAGA-----------GGGATGGA-----------TGAGGCATACA

CN 120017 G-AGCATCTTGTAATAAGA-----------GGGATGGA-----------TGAGGCATACA

CN 119205 ------------------------------------------------------------

DH55 ref genome T-TGGACTCTGTAGC-AGACTTAGAAGCTGTGGCTGCAGACTTGGCCTTTGATGCAGACA

09-CS0040 T-TGGACTCTGTAGC-AGACTTAGAAGCTGTGGCTGCAGACTTGGCCTTTGATGCAGACA

CN 113754 T-TGGACTCTGTAGCGAGACTTAGAAGCTGTGGCTGCAGACTTGGCCTTTGATGCAGACA

CO46 NCBI T-TGGACTCTGTAGC-AGACTTAGAAGCTGTGGCTGCAGACTTGGCCTTTGATGCAGACA

Jasper T-TGGACTCTGTAGC-AGACTTAGAAGCTGTGGCTGCAGACTTGGCCTTTGATGCAGACA

Joelle phyto T-TGGACTCTGTAGC-AGACTTAGAAGCTGTGGCTGCAGACTTGGCCTTTGATGCAGACA

Joelle NCBI T-TGGACTCTGTAGC-AGACTTAGAAGCTGTGGCTGCAGACTTGGCCTTTGATGCAGACA

CN 119243 ------------------------------------------------------------

CN 120025 ------------------------------------------------------------

Joelle AAFC ------------------------------------------------------------

CAM 241 AGGTGCATAGTGATTGGTATCATCATGGAGATGTGAAGTCAGTAGATGAATTTCAAAGTA

17CS1133 AGGTGCATAGTGATTGGTATCATCATGGAGATGTGAAGTCAGTAGATGAATTTCAAAGTA

CAM 236 AGGTGCATAGTGATTGGTATCATCATGGAGATGTGAAGTCAGTAGATGAATTTCAAAGTA

Blaine Creek AGGTGCATAGTGATTGGTATCATCATGGAGATGTGAAGTCAGTAGATGAATTTCAAAGTA

CN 119300 AGGTGCATAGTGATTGGTATCATCATGGAGATGTGAAGTCAGTAGATGAATTTCAAAGTA

Yellowstone AGGTGCATAGTGATTGGTATCATCATGGAGATGTGAAGTCAGTAGATGAATTTCAAAGTA

Hoga AGGTGCATAGTGATTGGTATCATCATGGAGATGTGAAGTCAGTAGATGAATTTCAAAGTA

CN 120027 AGGTGCATAGTGATTGGTATCATCATGGAGATGTGAAGTCAGTAGATGAATTTCAAAGTA

CN 120030 AGGTGCATAGTGATTGGTATCATCATGGAGATGTGAAGTCGGTAGATGAATTTCAAAGTA

CN 119294 AGGTGCATAGTGATTGGTATCATCATGGAGATGTGAAGTCAGTAGATGAATTTCAAAGTA

CN 120013 AGGTGCATAGTGATTGGTATCATCATGGAGATGTGAAGTCAGTAGATGAATTTCAAAGTA

CN 120017 AGGTGCATAGTGATTGGTATCATCATGGAGATGTGAAGTCGGTAGATGAATTTCAAAGTA

CN 119205 ------------------------------------------------------------

DH55 ref genome AAGAAGCATGTGAT--GTAGCATTCTGACCAGTTGGAGTTGAGGGAT---TTTCAAAATC

09-CS0040 AAGAAGCATGTGAT--GTAGCATTCTGACCAGTTGGAGTTGAGGGAT---TTTCAAAATC

CN 113754 AAGAAGCATGTGAT--GTAGCATTCTGACCAGTTGGAGTTGAGGGAT---TTTCAAAATC

CO46 NCBI AAGAAGCATGTGAT--GTAGCATTCTGACCAGTTGGAGTTGAGGGAT---TTTCAAAATC

Jasper AAGAAGCATGTGAT--GTAGCATTCTGACCAGTTGGAGTTGAGGGAT---TTTCAAAATC

Joelle phyto AAGAAGCATGTGAT--GTAGCATTCTGACCAGTTGGAGTTGAGGGAT---TTTCAAAATC

Joelle NCBI AAGAAGCATGTGAT--GTAGCATTCTGACCAGTTGGAGTTGAGGGAT---TTTCAAAATC

CN 119243 ------------------------------------------------------------

CN 120025 ------------------------------------------------------------

Joelle AAFC ------------------------------------------------------------

CAM 241 AACC-AACTCAGTGG----------------------------------AATGA------

17CS1133 AACC-AACTCAGTGG----------------------------------AATGA------

CAM 236 AACC-AACTCAGTGG----------------------------------AATGA------

Blaine Creek AACC-AACTCAGTGG----------------------------------AATGA------

CN 119300 AACC-AACTCAGTGG----------------------------------AATGA------

Yellowstone AACC-AACTCAGTGG----------------------------------AATGA------

Hoga AACC-AACTCAGTGG----------------------------------AATGA------

CN 120027 AACC-AACTCAGTGG----------------------------------AATGA------

CN 120030 AACC-AACTCAGTGG----------------------------------AATGA------

CN 119294 AACC-AACTCAGTGG----------------------------------AATGA------

CN 120013 AACC-AACTCAGTGG----------------------------------AATGA------

CN 120017 AACC-AACTCAGTGG----------------------------------AATGA------

CN 119205 ------------------------------------------------------------

DH55 ref genome AACCTTACTCAGGGGCCAAGATACGTAAGCCATCAAACAGTCCTCAAGAAATGACATTTC

09-CS0040 AACCTTACTCAGGGGCCAAGATACGTAAGCCATCAAACAGTCCTCAAGAAATGACATTTC

CN 113754 AACCTTACTCGGGGGCCAAGATACGTAAGCCATCAAACAGTCCTCAAGAAATGACATTTC

CO46 NCBI AACCTTACTCAGGGGCCAAGATACGTAAGCCATCAAACAGTCCTCAAGAAATGACATTTC

Jasper AACCTTACTCAGGGGCCAAGATACGTAAGCCATCAAACAGTCCTCAAGAAATGACATTTC

Joelle phyto AACCTTACTCAGGGGCCAAGATACGTAAGCCATCAAACAGTCCTCAAGAAATGACATTTC

Joelle NCBI AACCTTACTCAGGGGCCAAGATACGTAAGCCATCAAACAGTCCTCAAGAAATGACATTTC

CN 119243 ------------------------------------------------------------

CN 120025 ------------------------------------------------------------

Joelle AAFC ------------------------------------------------------------

CAM 241 ----GGAAGTTT---TTGAGTTATATAAAGCTGCTGAATTTTTTGATCAAGAGTTG----

17CS1133 ----GGAAGTTT---TTGAGTTATATAAAGCTGCTGAATTTTTTGATCAAGAGTTG----

CAM 236 ----GGAAGTTT---TTGAGTTATATAAAGCTGCTGAATTTTTTGATCAAGAGTTG----

Blaine Creek ----GGAAGTTT---TTGAGTTATATAAAGCTGCTGAATTTTTTGATCAAGAGTTG----

CN 119300 ----GGAAGTTT---TTGAGTTATATAAAGCTGCTGAATTTTTTGATCAAGAGTTG----

Yellowstone ----GGAAGTTT---TTGAGTTATATAAAGCTGCTGAATTTTTTGATCAAGAGTTG----

Hoga ----GGAAGTTT---TTGAGTTATATAAAGCTGCTGAATTTTTTGATCAAGAGTTG----

CN 120027 ----GGAAGTTT---TTGAGTTATATAAAGCTGCTGAATTTTTTGATCAAGAGTTG----

CN 120030 ----GGAAGTTT---TTGAGTTATATAAAGCTGCTGAATTTTTTGATCAAGAGTTG----

CN 119294 ----GGAAGTTT---TTGAGTTATATAAAGCTGCTGAATTTTTTGATCAAGAGTTG----

CN 120013 ----GGAAGTTT---TTGAGTTATATAAAGCTGCTGAATTTTTTGATCAAGAGTTG----

CN 120017 ----GGAAGTTT---TTGAGTTATATAAAGCTGCTGAATTTTTTGATCAAGAGTTG----

CN 119205 ------------------------------------------------------------

DH55 ref genome AGCTGTAGGTCTCCATAGTGATGTATCAGGCTGCT---TTACTGAATCCACAAATACTTT

09-CS0040 AGCTGTAGGTCTCCATAGTGATGTATCAGGCTGCT---TTACTGAATCCACAAATACTTT

CN 113754 AGCTGTAGGTCTCCATAGTGATGTATCAGGCTGCT---TTACTGAATCCACAAATACTTT

CO46 NCBI AGCTGTAGGTCTCCATAGTGATGTATCAGGCTGCT---TTACTGAATCCACAAATACTTT

Jasper AGCTGTAGGTCTCCATAGTGATGTATCAGGCTGCT---TTACTGAATCCACAAATACTTT

Joelle phyto AGCTGTAGGTCTCCATAGTGATGTATCAGGCTGCT---TTACTGAATCCACAAATACTTT

Joelle NCBI AGCTGTAGGTCTCCATAGTGATGTATCAGGCTGCT---TTACTGAATCCACAAATACTTT

CN 119243 ------------------------------------------------------------

CN 120025 ------------------------------------------------------------

Joelle AAFC ------------------------------------------------------------

CAM 241 ----GCTTTTAG-----AGGTGACTTAGCTGACCAACCCGTGGGCGACTTAAGTGAGATT

17CS1133 ----GCTTTTAG-----AGGTGACTTAGCTGACCAACCTGTGGGCGACTTAAGTGAGATT

CAM 236 ----GCTTTTAG-----AGGTGACTTAGCTGACCAACCCGTGGGCGACTTAAGTGAGATT

Blaine Creek ----GCTTTTAG-----AGGTGACTTAGCTGACCAACCTGTGGGCGACTTAAGTGAGATT

CN 119300 ----GCTTTTAG-----AGGTGACTTAGCTGACCAACCTGTGGGCGACTTAAGTGAGATT

Yellowstone ----GCTTTTAG-----AGGTGACTTAGCTGACCAACCTGTGGGCGACTTAAGTGAGATT

Hoga ----GCTTTTAG-----AGGTGACTTAGCCGACCAACCTGTGGGCGACTTAAGTGAGATT

CN 120027 ----GCTTTTAG-----AGGTGACTTAGCTGACCAACCTGTGGGCGACTTAAGTGAGATT

CN 120030 ----GCTTTTAG-----AGGTGACTTAGCTGACCAACCTGTGGGCGACTTAAGTGAGATT

CN 119294 ----GCTTTTAG-----AGGTGACTTAGCTGACCAACCTGTGGGCGACTTAAGTGAGATT

CN 120013 ----GCTTTTAG-----AGGTGACTTAGCTGACCAACCTGTGGGCGACTTAAGTGAGATT

CN 120017 ----GCTTTTAG-----AGGTGACTTAGCTGACCAACCTGTGGGCGACTTAAGTGAGATT

CN 119205 ------------------------------------------------------------

DH55 ref genome AACTGCTTTTGGTCCAAGAGGAATTCCGTTAACCAACGCACTTGGTTCTTGTGT----CT

09-CS0040 AACTGCTTTTGGTCCAAGAGGAATTCCGTTAACCAACGCACTTGGTTCTTGTGT----CT

CN 113754 AACTGCTTTTGGTCCAAGAGGAATTCCGTTAACCAACGCACTTGGTTCTTGTGT----CT

CO46 NCBI AACTGCTTTTGGTCCAAGAGGAATTCCGTTAACCAACGCACTTGGTTCTTGTGT----CT

Jasper AACTGCTTTTGGTCCAAGAGGAATTCCGTTAACCAACGCACTTGGTTCTTGTGT----CT

Joelle phyto AACTGCTTTTGGTCCAAGAGGAATTCCGTTAACCAACGCACTTGGTTCTTGTGT----CT

Joelle NCBI AACTGCTTTTGGTCCAAGAGGAATTCCGTTAACCAACGCACTTGGTTCTTGTGT----CT

CN 119243 ------------------------------------------------------------

CN 120025 ------------------------------------------------------------

Joelle AAFC ------------------------------------------------------------

CAM 241 GC-AGAGGGTGAGGACCAACAAGAGGATGAGTTCCTTGCAAAGATCCGGGATGCTGAA--

17CS1133 GC-AGAGGGTGAGGACCAACAAGAGGATGAGTTCCTTGCAAAGATCCGGGATGCTGAA--

CAM 236 GC-AGAGGGTGAGGACCAACAAGAGGATGAGTTCCTTGCAAAGATCC-GGATGCTGAA--

Blaine Creek GC-GAGAGGTGAGGACCAACAAGAGGATGAGTTCCTTGCAAAGATCCGGGATGCTGAA--

CN 119300 GC-AGAGGGTGAGGACCAACAAGAGGATGAGTTCCTTGCAAAGATCCGGGATGCTGAA--

Yellowstone GC-AGAGGGTGAGGACCAACAAGAGGATGAGTTCCTTGCAAAGATCCGGGATGCTGAA--

Hoga GC-AGAGGGTGAGGACCAACAAGAGGATGAGTTCCTTGCAAAGATCCGGGATGCTGAA--

CN 120027 GC-AGAGGGTGAGGACCAACAAGAGGATGAGTTCCTTGCAAAGATCCGGGATGCTGAA--

CN 120030 GC-AGAGGGTGAGGACCAACAAGAGGATGAGTTCCTTGCAAAGATCCGGGATGCTGAA--

CN 119294 GC-AGAGGGTGAGGACCAACAAGAGGATGAGTTCCTTGCAAAGATCCGGGATGCTGAA--

CN 120013 GC-AGAGGGTGAGGACCAACAAGAGGATGAGTTCCTTGCAAAGATCCGGGATGCTGAA--

CN 120017 GCGAGAGGGTGAGGACCAACAAGAGGATGAGTTCCTTGCAAAGATCCGGGATGCTGAA--

CN 119205 ------------------------------------------------------------

DH55 ref genome CC-CAACGCCCCTCAGCAACA--ATGACGTCACCCTTAGACAAATCCAATAGTTTACATT

09-CS0040 CC-CAACGCCCCTCAGCAACA--ATGACGTCACCCTTAGACAAATCCAATAGTTTACATT

CN 113754 CC-CAACGCCCCTCAGCAACA--ATGACGTCACCCTTAGACAAATCCAATAGTTTACATT

CO46 NCBI CC-CAACGCCCCTCAGCAACA--ATGACGTCACCCTTAGACAAATCCAATAGTTTACATT

Jasper CC-CAACGCCCTCAAGCAACA--ATGACGTCACCCTTAGACAAATCCAATAGTTTACATT

Joelle phyto CC-CAACGCCCCTCAGCAACA--ATGACGTCACCCTTAGACAAATCCAATAGTTTACATT

Joelle NCBI CC-CAACGCCCCTCAGCAACA--ATGACGTCACCCTTAGACAAATCCAATAGTTTACATT

CN 119243 ------------------------------------------------------------

CN 120025 ------------------------------------------------------------

Joelle AAFC ------------------------------------------------------------

CAM 241 ---ACCCCACTATACCCTAGCTGTTCAAACCACAGCAAGTTATCGGCTATTGTGACTTTG

17CS1133 ---ACCCCACTATACCCTAGCTGTTCAAACCACAGCAAGTTATCGGCTATTGTGACTTTG

CAM 236 ---ACCCCACTATACCCTAGCTGTTCAAACCACAGCAAGTTATCGGCTATTGTGACTTTG

Blaine Creek ---ACCCCACTATACCCTAGCTGTTCAAACCACAGCAAGTTATCGGCTATTGTGACTTTG

CN 119300 ---ACCCCACTATACCCTAGCTGTTCAAACCACAGCAAGTTATCGGCTATTGTGACTTTG

Yellowstone ---ACCCCACTATACCCTAGCTGTTCAAACCACAGCAAGTTATCGGCTATTGTGACTTTG

Hoga ---ACCCCACTATACCCTAGCTGTTCAAACCACAGCAAGTTATCGGCTATTGTGACTTTG

CN 120027 ---ACCCCACTATACCCTAGCTGTTCAAACCACAGCAAGTTATCGGCTATTGTGACTTTG

CN 120030 ---ACCCCACTATACCCTAGCTGTTCAAACCACAGCAAGTTATCAGCTATTGTGACTTTG

CN 119294 ---ACCCCACTATACCCTAGCTGTTCAAACCACAGCAAGTTATCGGCTATTGTGACTTTG

CN 120013 ---ACCCCACTATACCCTAGCTGTTCAAACCACAGCAAGTTATCGGCTATTGTGACTTTG

CN 120017 ---ACCCCACTATACCCTAGCTGTTCAAACCACAGCAAGTTATCGGCTATTGTGACTTTG

CN 119205 ------------------------------------------------------------

DH55 ref genome TGTTTCCTTGTATACC----CTGTT--AAACACAATCAGTAAACAAATCCAATATAATTA

09-CS0040 TGTTTCCTTGTATACC----CTGTT--AAACACAATCAGTAAACAAATCCAATATAATTA

CN 113754 TGTTTCCTTGTATACC----CTGTT--AAACACAATCAGTAAACAAATCCAATATAATTA

CO46 NCBI TGTTTCCTTGTATACC----CTGTT--AAACACAATCAGTAAACAAATCCAATATAATTA

Jasper TGTTTCCTTGTATACC----CTGTT--AAACACAATCAGTAAACAAATCCAATATAATTA

Joelle phyto TGTTTCCTTGTATACC----CTGTT--AAACACAATCAGTAAACAAATCCAATATAATTA

Joelle NCBI TGTTTCCTTGTATACC----CTGTT--AAACACAATCAGTAAACAAATCCAATATAATTA

CN 119243 ------------------------------------------------------------

CN 120025 ------------------------------------------------------------

Joelle AAFC ------------------------------------------------------------

CAM 241 TTTAGGATTAAGACACATAATGGCTGGTCGGATAAGAGCTT--CAATGAACTGCTTC-AG

17CS1133 TTTAGGATTAAGACACATAATGGCTGGTCGGATAAGAGCTT--CAATGAACTGCTTCGAG

CAM 236 TTTAGGATTAAGACACATAATGGCTGGTCGGATAAGAGCTT--CAATGAACTGCTTC-GG

Blaine Creek TTTAGGATTAAGACACATAATGGCTGGTCGGATAAGAGCTT--CAATGAACTGCTTC-AG

CN 119300 TTTAGGATTAAGACACATAATGGCTGGTCGGATAAGAGCTT--CAATGAACTGCTTC-AG

Yellowstone TTTAGGATTAAGACACATAATGGCTGGTCGGATAAGAGCTT--CAATGAACTGCTTC-GG

Hoga TTTAGGATTAAGACACATAATGGCTGGTCGGATAAGAGCTT--CAATGAACTGCTTCGAG

CN 120027 TTTAGGATTAAGACACATAATGGCTGGTCGGATAAGAGCTT--CAATGAACTGCTTC-AG

CN 120030 TTTAGGATTAAGACACATAATGGCTGGTCGGATAAGAGCTT--CAATGAACTGCTTC-GG

CN 119294 TTTAGGATTAAGACACATAATGGCTGGTCGGATAAGAGCTT--CAATGAACTGCTTC-AG

CN 120013 TTTAGGATTAAGACACATAATGGCTGGTCGGATAAGAGCTT--CAATGAACTGCTGC-AG

CN 120017 TTTAGGATTAAGACACATAATGGCTGGTCGGATAAGAGCTT--CAATGAACTGCTTC-AG

CN 119205 ------------------------------------------------------------

DH55 ref genome ATTAGCATTATAG-ACAG-----------GGGTAATACATTGCTAACACATTGCT---AG

09-CS0040 ATTAGCATTATA-------------GACGGGGTAATACATTGCTAACACATTGCT---AG

CN 113754 ATTAGCATTATAGCATGT-----------GGGTAATACATTGCTAACACATTGCT---AG

CO46 NCBI ATTAGCATTATAG-ACAG-----------GGGTAATACATTGCTAACACATTGCT---AG

Jasper ATTAGCATTATAG-ACAG-----------GGGTAATACATTGCTAACACATTGCT---AG

Joelle phyto ATTAGCATTATAG-ACAG-----------GGGTAATACATTGCTAACACATTGCT---AG

Joelle NCBI ATTAGCATTATAG-ACAG-----------GGGTAATACATTGCTAACACATTGCT---AG

CN 119243 ------------------------------------------------------------

CN 120025 ------------------------------------------------------------

Joelle AAFC ------------------------------------------------------------

CAM 241 ACATTGCCAAGCATGTTGCC-AGATGGTAATGTCTTTCAC----ACATCATTGTATG---

17CS1133 ACATTGCCAAGCATGTTGCCGAGATGGTAATGTCTTTCAC----ACATCATTGTATG---

CAM 236 ACATTGCCAAGCATGTTGCC-AGATGGTAATGTCTTTCAC----ACATCATTGTATG---

Blaine Creek ACATTGCCAAGCATGTTGCC-AGATGGTAATGTCTTTCAC----ACATCATTGTATG---

CN 119300 ACATTGCCAAGCATGTTGCC-GGATGGTAATGTCTTTCAC----ACATCATTGTATG---

Yellowstone ACATTGCCAAGCATGTTGCC-AGATGGTAATGTCTTTCAC----ACATCATTGTATG---

Hoga ACATTGCCAAGCATGTTGCC-AGATGGTAATGTCTTTCAC----ACATCATTGTATG---

CN 120027 ACATTGCCAAGCATGTTGCC-GGATGGTAATGTCTTTCAC----ACATCATTGTATG---

CN 120030 ACATTGCCAAGCATGTTGCC-AGATGGTAATGTCTTTCAC----ACATCATTGTATG---

CN 119294 ACATTGCCAAGCATGTTGCC-AGATGGTAATGTCTTTCAC----ACATCATTGTATG---

CN 120013 ACATTGCCAAGCATGTTGCC-AGATGGTAATGTCTTTCAC----ACATCATTGTATG---

CN 120017 ACATTGCCAAGCATGTTGCC-AGATGGTAATGTCTTTCAC----ACATCATTGTATG---

CN 119205 ------------------------------------------------------------

DH55 ref genome AAATTATAAA------TACCATAGGGGCAATGTCTTCCATTTGGATGTTGTTGTCTACCA

09-CS0040 AAATTATAAA------TACCATAGGGGCAATGTCTTCCATTTGGATGTTGTTGTCTACCA

CN 113754 AAATTATAAA------TACCATAGGGGCAATGTCTTCCATTTGGATGTTGTTGTCTACCA

CO46 NCBI AAATTATAAA------TACCATAGGGGCAATGTCTTCCATTTGGATGTTGTTGTCTACCA

Jasper AAATTATAAA------TACCATAGGGGCAATGTCTTCCATTTGGATGTTGTTGTCTACCA

Joelle phyto AAATTATAAA------TACCATAGGGGCAATGTCTTCCATTTGGATGTTGTTGTCTACCA

Joelle NCBI AAATTATAAA------TACCATAGGGGCAATGTCTTCCATTTGGATGTTGTTGTCTACCA

CN 119243 ------------------------------------------------------------

CN 120025 ------------------------------------------------------------

Joelle AAFC ------------------------------------------------------------

CAM 241 -------------------------------ATGTCAAGAAATTTTT--------AAAGA

17CS1133 -------------------------------ATGTCAAGAAATTTTT--------AAAGA

CAM 236 -------------------------------ATGTCAAGAAATTTTT--------AAAGA

Blaine Creek -------------------------------ATGTCGAGAAATTTTT--------AAAGA

CN 119300 -------------------------------ATGTCAAGAAATTTTT--------AAAGA

Yellowstone -------------------------------ATGTCAAGAAATTTTT--------AAAGA

Hoga -------------------------------ATGTCAAGAAATTTTT--------AAAGA

CN 120027 -------------------------------ATGTCAAGAAATTTTT--------AAAGA

CN 120030 -------------------------------ATGTCAAGAAATTTTT--------AAAGA

CN 119294 -------------------------------ATGTCAAGAAATTTTT--------AAAGA

CN 120013 -------------------------------ATGTCAAGAAATTTTT--------AAAGA

CN 120017 -------------------------------ATGTCAAGAAATTTTT--------AAAGA

CN 119205 ------------------------------------------------------------

DH55 ref genome GTCTACACTTATCAGTTGGCCATGCGATTATATGTCCAACAGCTTCTTTCATATGAAAGA

09-CS0040 GTCTACACTTATCAGTTGGCCATGCGATTATATGTCCAACAGCTTCTTTCATATGAAAGA

CN 113754 GTCTACACTTATCAGTTGGCCATGCGATTATATGTCCAACAGCTTCTTTCATATGAAAGA

CO46 NCBI GTCTACACTTATCAGTTGGCCATGCGATTATATGTCCAACAGCTTCTTTCATATGAAAGA

Jasper GTCTACACTTATCAGTTGGCCATGCGATTATATGTCCAACAGCTTCTTTCATATGAAAGA

Joelle phyto GTCTACACTTATCAGTTGGCCATGCGATTATATGTCCAACAGCTTCTTTCATATGAAAGA

Joelle NCBI GTCTACACTTATCAGTTGGCCATGCGATTATATGTCCAACAGCTTCTTTCATATGAAAGA

CN 119243 ------------------------------------------------------------

CN 120025 ------------------------------------------------------------

Joelle AAFC ------------------------------------------------------------

CAM 241 GCTTTCATATGGGATATGAAAAGATCGACGCATGT-GTTAATGATTGCTGCCTCTTC-GA

17CS1133 GCTTTCATATGGGATATGAAAAGATCGACGCATGT-GTTAATGATTGCTGCCTCTTCGGA

CAM 236 GCTTTCATATGGGATATGAAAAGATCGACGCATGT-GTTAATGATTGCTGCCTCTTCGTA

Blaine Creek GCTTTCATATGGGATATGAAAAGATCGACGCATGT-GTTAATGATTGCTGCCTCTTCGGA

CN 119300 GCTTTCATATGGGATATGAAAAGATCGACGCATGT-GTTAATGATTGCTGCCTCTTCGGA

Yellowstone GCTTTCATATGGGATATGAAAAGATCGACGCATGT-GTTAATGATTGCTGCCTCTTCAGA

Hoga GCTTTCATATGGGATATGAAAAGATCGACGCATGT-GTTAATGATTGCTGCCTCTTCGGA

CN 120027 GCTTTCATATGGGATATGAAAAGATCGACGCATGT-GTTAATGATTGCTGCCTCTTC-GA

CN 120030 GCTTTCATATGGGATATGAAAAGATCGACGCATGT-GTTAATGATTGCTGCCTCTTCGTA

CN 119294 GCTTTCATATGGGATATGAAAAGATCGACGCATGT-GTTAATGATTGCTGCCTCTTC-GA

CN 120013 GCTTTCATATGGGATATGAAAAGATCGACGCATGT-GTTAATGATTGCTGCCTCTTCGGA

CN 120017 GCTTTCATATGGGATATGAAAAGATCGACGCATGT-GTTAATGATTGCTGCCTCTTCGGA

CN 119205 ------------------------------------------------------------

DH55 ref genome TCTTTTGTGCAGGTCTCCATAGAA---ATGCATCTGGTTTATA-----TGCAGCTTCAAC

09-CS0040 TCTTTTGTGCAGGTCTCCATAGAA---ATGCATCTGGTTTATA-----TGCAGCTTCAAC

CN 113754 TCTTTTGTGCAGGTCTCCATAGAA---ATGCATCTGGTTTATA-----TGCAGCTTCAAC

CO46 NCBI TCTTTTGTGCAGGTCTCCATAGAA---ATGCATCTGGTTTATA-----TGCAGCTTCAAC

Jasper TCTTTTGTGCAGGTCTCCATAGAA---ATGCATCTGGTTTATA-----TGCAGCTTCAAC

Joelle phyto TCTTTTGTGCAGGTCTCCATAGAA---ATGCATCTGGTTTATA-----TGCAGCTTCAAC

Joelle NCBI TCTTTTGTGCAGGTCTCCATAGAA---ATGCATCTGGTTTATA-----TGCAGCTTCAAC

CN 119243 ------------------------------------------------------------

CN 120025 ------------------------------------------------------------

Joelle AAFC ------------------------------------------------------------

CAM 241 AAGAAGTTAAAGAAGCTTGATAAATGTCCCAAATGTA-ATGCTTCACGGTGGAAGACTAA

17CS1133 AAGAAGTTAAAGAAGCTTGATAAATGTCCCAAATGTA-ATGCTTCACGGTGGAAGACTAA

CAM 236 AAGAAGTTAAAGAAGCTTGATAAATGTCCCAAATGTA-ATGCTTCACGGTGGAAGACTAA

Blaine Creek AAGAAGTTAAAGAAGCTTGATAAATGTCCCAAATGTA-ATGCTTCACGGTGGAAGACTAA

CN 119300 AAGAAGTTAAAGAAGCTTGATAAATGTCCCAAATGTA-ATGCTTCACGGTGGAAGACTAA

Yellowstone AAGAAGTTAAAGAAGCTTGATAAATGTCCCAAATGTA-ATGCTTCACGGTGGAAGACTAA

Hoga AAGAAGTTAAAGAAGCTTGATAAATGTCCCAAATGTA-ATGCTTCACGGTGGAAGACTAA

CN 120027 AAGAAGTTAAAGAAGCTTGATAAATGTCCCAAATGTA-ATGCTTCACGGTGGAAGACTAA

CN 120030 AAGAAGTTAAAGAAGCTTGATAAATGTCCCAAATGTA-ATGCTTCACGGTGGAAGACTAA

CN 119294 AAGAAGTTAAAGAAGCTTGATAAATGTCCCAAATGTA-ATGCTTCACGGTGGAAGACTAA

CN 120013 AAGAAGTTAAAGAAGCTTGATAAATGTCCCAAATGTA-ATGCTTCACGGTGGAAGACTAA

CN 120017 AAGAAGTTAAAGAAGCTTGATAAATGTCCCAAATGTA-ATGCTTCACGGTGGAAGACTAA

CN 119205 ------------------------------------------------------------

DH55 ref genome TAACACTTTGAGA----TCATAAGGGCCAAGACGACA-GTCATTCACTATGTCATCCGGA

09-CS0040 TAACACATTGAGA----TCATAAGGGCCAAGACGACA-GTCATTCACTATGTCATCCGGA

CN 113754 TAACACATTGAGA----TCATAAGGGCCAAGACGACAGGTCATTCACTATGTCATCCGGA

CO46 NCBI TAACACATTGAGA----TCATAAGGGCCAAGACGACA-GTCATTCACTATGTCATCCGGA

Jasper TAACACATTGAGA----TCATAAGGGCCAAGACGACA-GTCATTCACTATGTCATCCGGA

Joelle phyto TAACACTTTGAGA----TCATAAGGGCCAAGACGACA-GTCATTCACTATGTCATCCGGA

Joelle NCBI TAACACTTTGAGA----TCATAAGGGCCAAGACGACA-GTCATTCACTATGTCATCCGGA

CN 119243 ------------------------------------------------------------

CN 120025 ------------------------------------------------------------

Joelle AAFC ------------------------------------------------------------

CAM 241 TAAGCGGACTAATGAGGTAAAGAAAGGTGTCCCACAGAAAGTATTAAGATATTTTCCAAT

17CS1133 TAAGCGGACTAATGAGGTAAAGAAAGGTGTCCCACAGAAAGTATTAAGATATTTTCCAAT

CAM 236 TAAGCGGACTAATGAGGTAAAGAAAGGTGTCCCACAGAAAGTATTAAGATATTTTCCAAT

Blaine Creek TAAGCGGACTAATGAGGTAAAGAAAGGTGTCCCACAGAAAGTATTAAGATATTTTCCAAT

CN 119300 TAAGCGGACTAATGAGGTAAAGAAAGGTGTCCCACAGAAAGTATTAAGATATTTTCCAAT

Yellowstone TAAGCGGACTAATGAGGTAAAGAAAGGTGTCCCACAGAAAGTATTAAGATATTTTCCAAT

Hoga TAAGCGGACTAATGAGGTAAAGAAAGGTGTCCCACAGAAAGTATTAAGATATTTTCCAAT

CN 120027 TAAGCGGACTAATGAGGTAAAGAAAGGTGTCCCACAGAAAGTATTAAGATATTTTCCAAT

CN 120030 TAAGCGGACTAATGAGGTAAAGAAAGGTGTCCCACAGAAAGTATTAAGATATTTTCCAAT

CN 119294 TAAGCGGACTAATGAGGTAAAGAAAGGTGTCCCACAGAAAGTATTAAGATATTTTCCAAT

CN 120013 TAAGCGGACTAATGAGGTAAAGAAAGGTGTCCCACAGAAAGTATTAAGATATTTTCCAAT

CN 120017 TAAGCGGACTAATGAGGTAAAGAAAGGTGTCCCACAGAAAGTATTAAGATATTTTCCAAT

CN 119205 ------------------------------------------------------------

DH55 ref genome TCAGAAG------------AAAGAATCCGACCCTCACCAA----------------CATT

09-CS0040 TCGAAG-------------AAAGAATCCGACCCTCACCAA----------------CATT

CN 113754 TCAGAAG------------AAAGAATCCGACCCTCACCAA----------------CATT

CO46 NCBI TCAGAAG------------AAAGAATCCGACCCTCACCAA----------------CATT

Jasper TCAGAAG------------AAAGAATCCGACCCTCACCAA----------------CATT

Joelle phyto TCAGAAG------------AAAGAATCCGACCCTCACCAA----------------CATT

Joelle NCBI TCAGAAG------------AAAGAATCCGACCCTCACCAA----------------CATT

CN 119243 ------------------------------------------------------------

CN 120025 ------------------------------------------------------------

Joelle AAFC ------------------------------------------------------------

CAM 241 TATACCAAGGCTGAAGAGAATGTTCAGATCAGAGGACATG-GCTAAGGA------CTTAC

17CS1133 TATACCAAGGCTGAAGAGAATGTTCAGATCAGAGGACATG-GCTAAGGA------CTTAC

CAM 236 TATACCAAGGCTGAAGAGAATGTTCAGATCGGAGGACATG-GCTAAGGA------CTTAC

Blaine Creek TATACCAAGGCTGAAGAGAATGTTCAGATCGGAGGACATG-GCTAAGGA------CTTAC

CN 119300 TATACCAAGGCTGAAGAGAATGTTCAGATCAGAGGACATG-GCTAAGGA------CTTAC

Yellowstone TATACCAAGGCTGAAGAGAATGTTCAGATCAGAGGACATG-GCTAAGGA------CTTAC

Hoga TATACCAAGGCTGAAGAGAATGTTCAGATCGGAGGACATG-GCTAAGGA------CTTAC

CN 120027 TATACCAAGGCTGAAGAGAATGTTCAGATCAGAGGACATG-GCTAAGGA------CTTAC

CN 120030 TATACCAAGGCTGAAGAGAATGTTCAGATCGGAGGACATG-GCTAAGGA------CTTAC

CN 119294 TATACCAAGGCTGAAGAGAATGTTCAGATCAGAGGACATG-GCTAAGGA------CTTAC

CN 120013 TATACCAAGGCTGAAGAGAATGTTCAGATCAGAGGACATG-GCTAAGGA------CTTAC

CN 120017 TATACCAAGGCTGAAGAGAATGTTCAGATCAGAGGACATG-GCTAAGGA------CTTAC

CN 119205 ------------------------------------------------------------

DH55 ref genome TTCATCAATGTCAGA---------CCAATCAACAAACACACACTTAGGATGTGCTCTTTT

09-CS0040 TTCATCAATGTCAGA---------CCAATCAACAAACACACACTTAGGATGTGCTCTTTT

CN 113754 TTCATCAATGTCAGA---------CCAATCAACAAACACACACTTAGGATGTGCTCTTTT

CO46 NCBI TTCATCAATGTCAGA---------CCAATCAACAAACACACACTTAGGATGTGCTCTTTT

Jasper TTCATCAATGTCAGA---------CCAATCAACAAACACACACTTAGGATGTGCTCTTTT

Joelle phyto TTCATCAATGTCAGA---------CCAATCAACAAACACACACTTAGGATGTGCTCTTTT

Joelle NCBI TTCATCAATGTCAGA---------CCAATCAACAAACACACACTTAGGATGTGCTCTTTT

CN 119243 ------------------------------------------------------------

CN 120025 ------------------------------------------------------------

Joelle AAFC ------------------------------------------------------------

CAM 241 GGTGGCATT-----ACACTAACAAGAGCACTCGATGGAAAACTT---CGACATCCAG-TA

17CS1133 GGTGGCATT-----ACACTAACAAGAGCACT-GATGGAAAACTT---CGACATCCAGATA

CAM 236 GGTGGCATT-----ACACTAACAAGAGCACT-GATGGAAAACTT---CGACATCCAG-TA

Blaine Creek GGTGGCATT-----ACACTAACAAGAGCACT-GATGGAAAACTT---CGACATCCAG-TA

CN 119300 GGTGGCATT-----ACACTAACAAGAGCACT-GATGGAAAACTT---CGACATCCAG-TA

Yellowstone GGTGGCATT-----ACACTAACAAGAGCACT-GATGGAAAACTT---CGACATCCAG-TA

Hoga GGTGGCATT-----ACACTAACAAGAGCACT-GATGGAAAACTT---CGACATCCAG-TA

CN 120027 GGTGGCATT-----ACACTAACAAGAGCACT-GATGGAAAACTT---CGACATCCAG-TA

CN 120030 GGTGGCATT-----ACACTAACAAGAGCACT-GATGGAAAACTT---CGACATCCAG-TA

CN 119294 GGTGGCATT-----ACACTAACAAGAGCACT-GATGGAAAACTT---CGACATCCAG-TA

CN 120013 GGTGGCATT-----ACACTAACAAGAGCACC-GATGGAAAACTT---CGACATCCAG-TA

CN 120017 GGTGGCATT-----ACACTAACAAGAGCACT-GATGGAAAACTT---CGACATCCAG-TA

CN 119205 ------------------------------------------------------------

DH55 ref genome GTTTACACTCTGCAACAATTTCATGAGCAAC---TAAGAATCATAAACGACACTAAG-AA

09-CS0040 GTTTACACTCTGCAACAATTTCATGAGCAAC---TAAGAATCATAAACGACACTAAG-AA

CN 113754 GTTTACACTCTGCAACAATTTCATGAGCAAC---TAAGAATCATAAACGACACTAAG-AA

CO46 NCBI GTTTACACTCTGCAACAATTTCATGAGCAAC---TAAGAATCATAAACGACACTAAG-AA

Jasper GTTTACACTCTGCAACAATTTCATGAGCAAC---TAAGAATCATAAACGACACTAAG-AA

Joelle phyto GTTTACACTCTGCAACAATTTCATGAGCAAC---TAAGAATCATAAACGACACTAAG-AA

Joelle NCBI GTTTACACTCTGCAACAATTTCATGAGCAAC---TAAGAATCATAAACGACACTAAG-AA

CN 119243 ------------------------------------------------------------

CN 120025 ------------------------------------------------------------

Joelle AAFC ------------------------------------------------------------

CAM 241 GATTCTGTTACATGGGCTC-AGATGAATGAGAAGTATCCTTCATTTGCAGCTGAAGAAAG

17CS1133 GATTCCGTTACATGGGCTCGAGATGAATGAGAAGTATCCTTCATTTGCAGCTGAAGAAAG

CAM 236 GATTCTGTTACATGGGCTC-AGATGAATGAGAAGTATCCTTCATTTGCAGCTGAAGAAAG

Blaine Creek GATTCTGTTACATGGGCTCGAGATGAATGAGAAGTATCCTTCATTTGCAGCTGAAGAAAG

CN 119300 GATTCTGTTACATGGGCTC-AGATGAATGAGAAGTATCCTTCATTTGCAGCTGAAGAAAG

Yellowstone GATTCTGTTACATGGGCTC-AGATGAATGAGAAGTATCCTTCATTTGCAGCTGAAGAAAG

Hoga GATTCTGTTACATGGGCTC-AGATGAATGAGAAGTATCCTTCATTTGCAGCTGAAGAAAG

CN 120027 GATTCTGTTACATGGGCTC-GGATGAATGAGAAGTATCCTTCATTTGCAGCTGAAGAAAG

CN 120030 GATTCTGTTACATGGGCTC-GGATGAATGAGAAGTATCCTTCATTTGCAGCTGAAGAAAG

CN 119294 GATTCTGTTACATGGGCTC-AGATGAATGAGAAGTATCCTTCATTTGCAGCTGAAGAAAG

CN 120013 GATTCTGTTACATGGGCTC-AGATGAATGAGAAGTATCCTTCATTTGCAGCTGAAGAAAG

CN 120017 GATTCTGTTACATGGGCTC-AGATGAATGAGAAGTATCCTTCATTTGCAGCTGAAGAAAG

CN 119205 ------------------------------------------------------------

DH55 ref genome GAATCACTTAGCTGATATG-AAAAGAAGTAAGAATATGTTACTCTTGCAGCTGA------

09-CS0040 GAATCACTTAGCTGATATG-AAAAGAAGTAAGAATATGTTACTCTTGCAGCTGA------

CN 113754 GAATCACTTAGCTGATATG-AAAAGAAGTAAGAATATGTTACTCTTGCAGCTGA------

CO46 NCBI GAATCACTTAGCTGATATG-AAAAGAAGTAAGAATATGTTACTCTTGCAGCTGA------

Jasper GAATCACTTAGCTGATATG-AAAAGAAGTAAGAATATGTTACTCTTGCAGCTGA------

Joelle phyto GAATCACTTAGCTGATATG-AAAAGAAGTAAGAATATGTTACTCTTGCAGCTGA------

Joelle NCBI GAATCACTTAGCTGATATG-AAAAGAAGTAAGAATATGTTACTCTTGCAGCTGA------

CN 119243 ------------------------------------------------------------

CN 120025 ------------------------------------------------------------

Joelle AAFC ------------------------------------------------------------

CAM 241 GAACATACGGCTTGGGCTGTCCACAGATGGATTTAATCCATTCAACATGAAGAATAGTA-

17CS1133 GAACATACGGCTTGGGCTGTCCACAGATGGATTTAATCCATTCAACATGAAGAATAGTA-

CAM 236 GAACATACGGCTTGGGCTGTCCACAGATGGATTTAATCCATTCAACATGAAGAATAGTA-

Blaine Creek GAACATACGGCTTGGGCTGTCCACAGATGGATTTAATCCATTCAACATGAAGAATAGTA-

CN 119300 GAACATACGGCTTGGGCTGTCCACAGATGGATTTAATCCATTCAACATGAAGAATAGTA-

Yellowstone GAACATACGGCTTGGGCTGTCCACAGATGGATTTAATCCATTCAACATGAAGAATAGTA-

Hoga GAACATACGGCTTGGGCTGTCCACAGATGGATTTAATCCATTCAACATGAAGAATAGTA-

CN 120027 GAACATACGGCTTGGGCTGTCCACAGATGGATTTAATCCATTCAACATGAAGAATAGTA-

CN 120030 GAACATACGGCTTGGGCTGTCCACAGATGGATTTAATCCATTCAACATGAAGAATAGTA-

CN 119294 GAACATACGGCTTGGGCTGTCCACAGATGGATTTAATCCATTCAACATGAAGAATAGTA-

CN 120013 GAACATACGGCTTGGGCTGTCCACAGATGGATTTAATCCATTCAACATGAAGAATAGTA-

CN 120017 GAACATACGGCTTGGGCTGTCCACGGATGGATTTAATCCATTCAACATGAAGAATAGTA-

CN 119205 ------------------------------------------------------------

DH55 ref genome ---------GTTT---TCATCCATTTCAGCTTCTGGTCTCTGTAACATAAACAAAAATAA

09-CS0040 ---------GTTT---TCATCCATTTCAGCTTCCGGTCTCTGTAACATAAACAAAAATAA

CN 113754 ---------GTTT---TCATCCATTTCAGCTTCTGGTCTCTGTAACATAAACAAAAATAA

CO46 NCBI ---------GTTT---TCATCCATTTCAGCTTCTGGTCTCTGTAACATAAACAAAAATAA

Jasper ---------GTTT---TCATCCATTTCAGCTTCTGGTCTCTGTAACATAAACAAAAATAA

Joelle phyto ---------GTTT---TCATCCATTTCAGCTTCTGGTCTCTGTAACATAAACAAAAATAA

Joelle NCBI ---------GTTT---TCATCCATTTCAGCTTCTGGTCTCTGTAACATAAACAAAAATAA

CN 119243 ------------------------------------------------------------

CN 120025 ------------------------------------------------------------

Joelle AAFC ------------------------------------------------------------

CAM 241 -----------ATTATAGTAGCTGGCCTGTGCTATTGGTAAACTACAATTTGCCTCCTCA

17CS1133 -----------ATTATAGTAGCTGGCCTGTGCTATTGGTAAACTACAATTTGCCTCCTCA

CAM 236 -----------ATTATAGTAGCTGGCCTGTGCTATTGGTAAACTACAATTTGCCTCCTCA

Blaine Creek -----------ATTATAGTAGCTGGCCTGTGCTATTGGTAAACTACAATTTGCCTCCTCA

CN 119300 -----------ATTATAGTAGCTGGCCTGTGCTATTGGTAAACTACAATTTGCCTCCTCA

Yellowstone -----------ATTATAGTAGCTGGCCTGTGCTATTGGTAAACTACAATTTGCCTCCTCA

Hoga -----------ATTATAGTAGCTGGCCTGTGCTATTGGTAAACTACAATTTGCCTCCTCA

CN 120027 -----------ATTATAGTAGCTGGCCTGTGCTATTGGTAAACTACAATTTGCCTCCTCA

CN 120030 -----------ATTATAGTAGCTGGCCTGTGCTATTGGTAAACTACAATTTGCCTCCTCA

CN 119294 -----------ATTATAGTAGCTGGCCTGTGCTATTGGTAAACTACAATTTGCCTCCTCA

CN 120013 -----------ATTATAGTAGCTGGCCTGTGCTATTGGTAAACTACAATTTGCCTCCTCA

CN 120017 -----------ATTATAGTAGCTGGCCTGTGCTATTGGTAAACTACAATTTGCCTCCTCA

CN 119205 ------------------------------------------------------------

DH55 ref genome CATCAATTTATATTACTCTAACTGGCTGATG--AATAGTAGAGAACTCTTAAGTATCTAA

09-CS0040 CATCAATTTATATTACTCTAACTGGCTGATG--AATAGTAGAGAACTCTTAAGTATCTAA

CN 113754 CATCAATTTATATTACTCTAACTGGCTGATG--AATAGTAGAGAACTCTTAAGTATCTAA

CO46 NCBI CATCAATTTATATTACTCTAACTGGCTGATG--AATAGTAGAGAACTCTTAAGTATCTAA

Jasper CATCAATTTATATTACTCTAACTGGCTGATG--AATAGTAGAGAACTCTTAAGTATCTAA

Joelle phyto CATCAATTTATATTACTCTAACTGGCTGATG--AATAGTAGAGAACTCTTAAGTATCTAA

Joelle NCBI CATCAATTTATATTACTCTAACTGGCTGATG--AATAGTAGAGAACTCTTAAGTATCTAA

CN 119243 ------------------------------------------------------------

CN 120025 ------------------------------------------------------------

Joelle AAFC ------------------------------------------------------------

CAM 241 CCTTTGTATGAAGAAGGAGAATATAATGTTGACATTATTGATT---CCTGGTCCACAACA

17CS1133 CCTTTGTATGAAGAAGGAGAATATAATGTTGACATTATTGATT---CCTGGTCCACAACA

CAM 236 CCTTTGTATGAAGAAGGAGAATATAATGTTGACATTATTGATT---CCTGGTCCACAACA

Blaine Creek CCTTTGTATGAAGAAGGAGAATATAATGTTGACATTATTGATT---CCTGGTCCACAACA

CN 119300 CCTTTGTATGAAGAAGGAGAATATAATGTTGACATTATTGATT---CCTGGTCCACAACA

Yellowstone CCTTTGTATGAAGAAGGAGAATATAATGTTGACATTATTGATT---CCTGGTCCACAACA

Hoga CCTTTGTATGAAGAAGGAGAATATAATGTTGACATTATTGATT---CCTGGTCCACAACA

CN 120027 CCTTTGTATGAAGAAGGAGAATATAATGTTGACATTATTGATT---CCTGGTCCACAACA

CN 120030 CCTTTGTATGAAGAAGGAGAATATAATGTTGACATTATTGATT---CCTGGTCCACAACA

CN 119294 CCTTTGTATGAAGAAGGAGAATATAATGTTGACATTATTGATT---CCTGGTCCACAACA

CN 120013 CCTTTGTATGAAGAAGGAGAATATAATGTTGACATTATTGATT---CCTGGTCCACAACA

CN 120017 CCTTTGTATGAAGAAGGAGAATATAATGTTGACATTATTGATT---CCTGGTCCACAACA

CN 119205 ------------------------------------------------------------

DH55 ref genome CCTG-----------------------------ATTCTTGATTCTGCCAAGTTCAC-TTT

09-CS0040 CCTG-----------------------------ATTCTTGATTCTGCCAAGTTCAC-TTT

CN 113754 CCTG-----------------------------ATTCTTGATTCTGCCAAGTTCAC-TTT

CO46 NCBI CCTG-----------------------------ATTCTTGATTCTGCCAAGTTCAC-TTT

Jasper CCTG-----------------------------ATTCTTGATTCTGCCAAGTTCAC-TTT

Joelle phyto CCTG-----------------------------ATTCTTGATTCTGCCAAGTTCAC-TTT

Joelle NCBI CCTG-----------------------------ATTCTTGATTCTGCCAAGTTCAC-TTT

CN 119243 ------------------------------------------------------------

CN 120025 ------------------------------------------------------------

Joelle AAFC ------------------------------------------------------------

CAM 241 ACCAGGTAATAA-------TATTGATGTCTACCTAGAACCTCTTATTGAGGATTTGAATC

17CS1133 ACCAGGTAATAA-------TATTGATGTCTACCTAGAACCTCTTATTGAGGATTTGAATC

CAM 236 ACCAGGTAATAA-------TATTGATGTCTACCTAGAACCTCTTATTGAGGATTTGAATC

Blaine Creek ACCAGGTAATAA-------TATTGATGTCTACCTAGAACCTCTTATTGAGGATTTGAATC

CN 119300 ACCAGGTAATAA-------TATTGATGTCTACCTAGAACCTCTTATTGAGGATTTGAATC

Yellowstone ACCAGGTAATAA-------TATTGATGTCTACCTAGAACCTCTTATTGAGGATTTGAATC

Hoga ACCAGGTAATAA-------TATTGATGTCTACCTAGAACCTCTTATTGAGGATTTGAATC

CN 120027 ACCAGGTAATAA-------TATTGATGTCTACCTAGAACCTCTTATTGAGGATTTGAATC

CN 120030 ACCAGGTAATAA-------TATTGATGTCTACCTAGAACCTCTTATTGAGGATTTGAATC

CN 119294 ACCAGGTAATAA-------TATTGATGTCTACCTAGAACCTCTTATTGAGGATTTGAATC

CN 120013 ACCAGGTAATAA-------TATTGATGTCTACCTAGAACCTCTTATTGAGGATTTGAATC

CN 120017 ACCAGGTAATAA-------TATTGATGTCTACCTAGAACCTCTTATTGAGGATTTGAATC

CN 119205 ------------------------------------------------------------

DH55 ref genome CCAAGGCATTGACCTGTTTTACTAAT-TTTACTTGCCGCTCTTCC------ATTTCAGCC

09-CS0040 CCAAGGCATTGACCTGTTTTACTAAT-TTTACTT---GCCGTTTGCAAACGATTTTTTGC

CN 113754 CCAAGGCATTGACCTGTTTTACTAAT-TTTACTT---GCCGTTTGCAAACGATTTTTTGC

CO46 NCBI CCAAGGCATTGACCTGTTTTACTAAT-TTTACTT---GCCGTTTGCAAACGATTTTTTGC

Jasper CCAAGGCATTGACCTGTTTTACTAAT-TTTACTT---GCCGTTTGCAAACGATTTTTTGC

Joelle phyto CCAAGGCATTGACCTGTTTTACTAAT-TTTACTTGCCGCTCTTCC------ATTTCAGCC

Joelle NCBI CCAAGGCATTGACCTGTTTTACTAAT-TTTACTTGCCGCTCTTCC------ATTTCAGCC

CN 119243 ------------------------------------------------------------

CN 120025 ------------------------------------------------------------

Joelle AAFC ------------------------------------------------------------

CAM 241 ATCT-GTGGAA---GAATGGAGAGCTAACGTATGATGCTTTTAGTAAAAGTAC----ATT

17CS1133 ATCT-GTGGAA---GAATGGAGAGCTAACGTATGATGCTTTTAGTAAAAGTAC----ATT

CAM 236 ATCC-GTGGAA---GAATGGAGAGCTAACGTATGATGCTTTTAGTAAAAGTAC----ATT

Blaine Creek ATCTCGTGGAA---GAATGGAGAGCTAACGTATGATGCTTTTAGTAAAAGTAC----ATT

CN 119300 ATCT-GTGGAA---GAATGGAGAGCTAACGTATGATGCTTTTAGTAAAAGTAC----ATT

Yellowstone ATCTCGTGGAA---GAATGGAGAGCTAACGTATGATGCTTTTAGTAAAAGTAC----ATT

Hoga ATCT-GTGGAA---GAATGGAGAGCTAACGTATGATGCTTTTAGTAAAAGTAC----ATT

CN 120027 ATCT-GTGGAA---GAATGGAGAGCTAACGTATGATGCTTTTAGTAAAAGTAC----ATT

CN 120030 ATCT-GTGGAA---GAATGGAGAGCTAACGTATGATGCTTTTAGTAAAAGTAC----ATT

CN 119294 ATCT-GTGGAA---GAATGGAGAGCTAACGTATGATGCTTTTAGTAAAAGTAC----ATT

CN 120013 ATCT-GTGGAA---GAATGGAGAGCTAACGTATGATGCTTTTAGTAAAAGTAC----ATT

CN 120017 ATCT-GTGGAA---GAATGGAGAGCTAACGTATGATGCTTTTAGTAAAAGTAC----ATT

CN 119205 ------------------------------------------------------------

DH55 ref genome ATACACTTGCTCTGCATTTGTAAACAAGCTAATT-TGGTCTTACTCATGCCTCTACCCAT

09-CS0040 ATACC---------TAACTGGAAGAAAGTGAGTCCAGATCTTAAAAAGAGCAC-------

CN 113754 ATACC---------TAACTGGAAGAAAGTGAGTCCAGATCTTAAAAAGAGCAC-------

CO46 NCBI ATACC---------TAACTGGAAGAAAGTGAGTCCAGATCTTAAAAAGAGCAC-------

Jasper ATACC---------TAACTGGAAGAAAGTGAGTCCAGATCTTAAAAAGAGCAC-------

Joelle phyto ATACACTTGCTCTGCATTTGTAAACAAGCTAATT-TGGTCTTACTCATGCCTCTACCCAT

Joelle NCBI ATACACTTGCTCTGCATTTGTAAACAAGCTAATT-TGGTCTTACTCATGCCTCTACCCAT

CN 119243 ------------------------------------------------------------

CN 120025 ------------------------------------------------------------

Joelle AAFC ------------------------------------------------------------

CAM 241 TACTCTAAAGGCAATGCTTCTCTGGACCATTAG----TGATTTTCCTGCGTATGGAAATC

17CS1133 TACTCTAAAGGCAATGCTTCTCTGGACCATTAG----TGATTTTCCTGCGTATGGAAATC

CAM 236 TACTCTAAAGGCAATGCTTCTCTGGACCATTAG----TGATTTTCCTGCGTATGGAAATC

Blaine Creek TACTCTAAAGGCAATGCTTCTCTGGACCATTAG----TGATTTTCCTGCGTATGGAAATC

CN 119300 TACTCTAAAGGCAATGCTTCTCCGGACCATTAG----TGATTTTCCTGCGTATGGAAATC

Yellowstone TACTCTAAAGGCAATGCTTCTCTGGACCATTAG----TGATTTTCCTGCGTATGGAAATC

Hoga TACTCTAAAGGCAATGCTTCTCCGGACCATTAG----TGATTTTCCTGCGTATGGAAATC

CN 120027 TACTCTAAAGGCAATGCTTCTCTGGACCATTAG----TGATTTTCCTGCGTATGGAAATC

CN 120030 TACTCTAAAGGCAATGCTTCTCTGGACCATTAG----TGATTTTCCTGCGTATGGAAATC

CN 119294 TACTCTAAAGGCAATGCTTCTCTGGACCATTAG----TGATTTTCCTGCGTATGGAAATC

CN 120013 TACTCTAAAGGCAATGCTTCTCTGGACCATTAG----TGATTTTCCTGCGTATGGAAATC

CN 120017 TACTCTAAAGGCAATGCTTCTCTGGACCATTAG----TGATTTTCCTGCGTATGGAAATC

CN 119205 ------------------------------------------------------------

DH55 ref genome TGCCCTCA-------GCCTACCAGAATTGTCAGGTCCTAAAAGCTTGGCGAGGTGATCTT

09-CS0040 -ATCCTAA-------GTGTGTGTTAAAGGTTAG----TAAAAGGTTTGCAAA-AAATCTT

CN 113754 -ATCCTAA-------GTGTGTGTTAAAGGTTAG----TAAAAGGTTTGCAAA-AAATCTT

CO46 NCBI -ATCCTAA-------GTGTGTGTTAAAGGTTAG----TAAAAGGTTTGCAAA-AAATCTT

Jasper -ATCCTAA-------GTGTGTGTTAAAGGTTAG----TAAAAGGTTTGCAAA-AAATCTT

Joelle phyto TGCCCTCA-------GCCTACCAGAATTGTCAGGTCCTAAAAGCTTGGCGAGGTGATCTT

Joelle NCBI TGCCCTCA-------GCCTACCAGAATTGTCAGGTCCTAAAAGCTTGGCGAGGTGATCTT

CN 119243 ------------------------------------------------------------

CN 120025 ------------------------------------------------------------

Joelle AAFC ------------------------------------------------------------

CAM 241 TTGCTGGTTGTAAAGTAAAAGGTAAAATGGGATGTCC---------------TATGTGTG

17CS1133 TTGCTGGTTGTAAAGTAAAAGGTAAAATGGGATGTCC---------------TATGTGTG

CAM 236 TTGCTGGTTGTAAAGTAAAAGGTAAAATGGGATGTCC---------------TATGTGTG

Blaine Creek TTGCTGGTTGTAAAGTAAAAGGTAAAATGGGATGTCC---------------TATGTGTG

CN 119300 TTGCTGGTTGTAAAGTAAAAGGTAAAATGGGATGTCC---------------TATGTGTG

Yellowstone TTGCTGGTTGTAAAGTAAAAGGTAAAATGGGATGTCC---------------TATGTGTG

Hoga TTGCTGGTTGTAAAGTAAAAGGTAAAATGGGATGTCC---------------TATGTGTG

CN 120027 TTGCTGGTTGTAAAGTAAAAGGTAAAATGGGATGTCC---------------TATGTGTG

CN 120030 TTGCTGGTTGTAAAGTAAAAGGTAAAATGGGATGTCC---------------TATGTGTG

CN 119294 TTGCTGGTTGTAAAGTAAAAGGTAAAATGGGATGTCC---------------TATGTGTG

CN 120013 TTGCTGGTTGTAAAGTAAAAGGTAAAATGGGATGTCC---------------TATGTGTG

CN 120017 TTGCTGGTTGTAAAGTAAAAGGTAAAATGGGATGTCC---------------TATGTGTG

CN 119205 ------------------------------------------------------------

DH55 ref genome CATCTGGATTTGTTAAAAATGCTGGAGCATCACTTCCACCAATTTCAGCTGCTTTTTGCT

09-CS0040 CTTCTCATTCTTGGACAAAGACCAAGGTG-------------------------CTCGCT

CN 113754 CTTCTCATTCTTGGACAAAGACCAAGGTG--------------------------CTGCT

CO46 NCBI CTTCTCATTCTTGGACAAAGACCAAGGTG--------------------------CTGCT

Jasper CTTCTCATTCTTGGACAAAGACCAAGGTG--------------------------CTGCT

Joelle phyto CATCTGGATTTGTTAAAAATGCTGGAGCATCACTTCCACCAATTTCAGCTGCTTTTTGCT

Joelle NCBI CATCTGGATTTGTTAAAAATGCTGGAGCATCACTTCCACCAATTTCAGCTGCTTTTTGCT

CN 119243 ------------------------------------------------------------

CN 120025 ------------------------------------------------------------

Joelle AAFC ------------------------------------------------------------

CAM 241 GGAAAA-ATACTGAT-AGTATGTGGTTGAAGT-----------------TTAGCAGGAAA

17CS1133 GGAAAA-ATACTGAT-AGTATGTGGTTGAAGT-----------------TTAGCAGGAAA

CAM 236 GGAAAA-ATACTGATAAGTATGTGGTTGAAGT-----------------TTAGCAGGAAA

Blaine Creek GGAAAA-ATACTGAT-AGTATGTGGTTGAAGT-----------------TTAGCAGGAAA

CN 119300 GGAAAA-ATACTGAT-AGTATGTGGTTGAAGT-----------------TTAGCAGGAAA

Yellowstone GGAAAA-ATACTGAT-AGTATGTGGTTGAAGT-----------------TTAGCAGGAAA

Hoga GGAAAA-ATACTGAT-AGTATGTGGTTGAAGT-----------------TTAGCAGGAAA

CN 120027 GGAAAA-ATACTGAT-AGTATGTGGTTGAAGT-----------------TTAGCAGGAAA

CN 120030 GGAAAA-ATACTGAT-AGTATGTGGTTGAAGT-----------------TTAGCAGGAAA

CN 119294 GGAAAA-ATACTGAT-AGTATGTGGTTGAAGT-----------------TTAGCAGGAAA

CN 120013 GGAAAA-ATACTGAT-AGTATGTGGTTGAAGT-----------------TTAGCAGGAAA

CN 120017 GGAAAA-ATACTGAT-AGTATGTGGTTGAAGT-----------------TTAGCAGGAAA

CN 119205 ------------------------------------------------------------

DH55 ref genome GAAAAACATAAAGAC-AGTTTCAGGTTTTACTAGTCGATAACACACTATTTACTGGTGTA

09-CS0040 GGAAGATATGTTCTC---TTTCCTTTTGTGGT----------------------AGGATG

CN 113754 GGAAGATATGTTCTC---TTTCCTTTTGTGGT----------------------AGGATG

CO46 NCBI GGAAGATATGTTCTC---TTTCCTTTTGTGGT----------------------AGGATG

Jasper GGAAGATATGTTCTC---TTTCCTTTTGTGGT----------------------AGGATG

Joelle phyto GAAAAACATAAAGAC-AGTTTCAGGTTTTACTAGTCGATAACACACTATTTACTGGTGTA

Joelle NCBI GAAAAACATAAAGAC-AGTTTCAGGTTTTACTAGTCGATAACACACTATTTACTGGTGTA

CN 119243 ------------------------------------------------------------

CN 120025 ------------------------------------------------------------

Joelle AAFC ------------------------------------------------------------

CAM 241 CATGTCTACATGTGTCATAGAA-AAGGTTTGGCTCCAACACACAGATATAGGGAAAAGA-

17CS1133 CATGTCTACATGTGTCATAGAA-AAGGTTTGGCTCCAACACACAGATATAGGGAAAAGA-

CAM 236 CATGTCTACATGTGTCATAGAA-AAGGTTTGGCTCCAACACACAGATATAGGGAAAAGA-

Blaine Creek CATGTCTACATGTGTCATAGAA-AAGGTTTGGCTCCAACACACAGATATAGGGAAAAGA-

CN 119300 CATGTCTACATGTGTCATAGAA-AAGGTTTGGCTCCAACACACAGATATAGGGAAAAGA-

Yellowstone CATGTCTACATGTGTCATAGAA-AAGGTTTGGCTCCAACACACAGATATAGGGAAAAGA-

Hoga CATGTCTACATGTGTCATAGAA-AAGGTTTGGCTCCAACACACAGATATAGGGAAAAGA-

CN 120027 CATGTCTACATGTGTCATAGAA-AAGGTTTGGCTCCAACACACAGATATAGGGAAAAGA-

CN 120030 CATGTCTACATGTGTCATAGAA-AAGGTTTGGCTCCAACACACAGATATAGGGAAAAGA-

CN 119294 CATGTCTACATGTGTCATAGAA-AAGGTTTGGCTCCAACACACAGATATAGGGAAAAGA-

CN 120013 CATGTCTACATGTGTCATAGAA-AAGGTTTGGCTCCAACACACAGATATAGGGAAAAGA-

CN 120017 CATGTCTACATGTGTCATAGAA-AAGGTTTGGCTCCAACACACAGATATAGGGAAAGA--

CN 119205 ------------------------------------------------------------

DH55 ref genome CAACTCTAT-TCAGACTTACAATCAAATCAGCTGCATTCGTATCTACTGGTGTACCATCC

09-CS0040 CAACTCT-----TTTCTTAGACCAAGATGTTGAAGATCCTTACGAGCATTAAGACCATCC

CN 113754 CAACTCT-----TTTCTTAGACCAAGATGTTGAAGATCCTTACGAGCATTAAGACCATCC

CO46 NCBI CAACTCT-----TTTCTTAGACCAAGATGTTGAAGATCCTTACGAGCATTAAGACCATCC

Jasper CAACTCT-----TTTCTTAGACCAAGATGTTGAAGATCCTTACGAGCATTAAGACCATCC

Joelle phyto CAACTCTAT-TCAGACTTACAATCAAATCAGCTGCATTCGTATCTACTGGTGTACCATCC

Joelle NCBI CAACTCTAT-TCAGACTTACAATCAAATCAGCTGCATTCGTATCTACTGGTGTACCATCC

CN 119243 ------------------------------------------------------------

CN 120025 ------------------------------------------------------------

Joelle AAFC ------------------------------------------------------------

CAM 241 -----------AGACTTGGTTTGATGGAAAAGTTGAG---CATAGGAGAAAGTCAAGAAT

17CS1133 -----------AGACTTGGTTTGATGGAAAAGTTGAG---CATAGGAGAAAGTCAAGAAT

CAM 236 -----------AGACTTGGTTTGATGGAAAAGTTGAG---CATAGGAGAAAGTCAAGAAT

Blaine Creek -----------AGACTTGGTTTGATGGAAAAGTTGAG---CATAGGAGAAAGTCAAGAAT

CN 119300 -----------AGACTTGGTTTGATGGAAAAGTTGAG---CATAGGAGAAAGTCAAGAAT

Yellowstone -----------AGACTTGGTTTGATGGAAAAGTTGAG---CATAGGAGAAAGTCAAGAAT

Hoga -----------AGACTTGGTTTGATGGAAAAGTTGAG---CATAGGAGAAAGTCAAGAAT

CN 120027 -----------AGACTTGGTTTGATGGAAAAGTTGAG---CATAGGAGAAAGTCAAGAAT

CN 120030 -----------AGACTTGGTTTGATGGAAAAGTTGAG---CATAGGAGAAAGTCAAGAAT

CN 119294 -----------AGACTTGGTTTGATGGAAAAGTTGAG---CATAGGAGAAAGTCAAGAAT

CN 120013 -----------AGACTTGGTTTGATGGAAAAGTTGAG---CATAGGAGAAAGTCAAGAAT

CN 120017 -----------AGACTTGGTTTGATGGAAAAGTTGAG---CATAGGAGAAAGTCAAGAAT

CN 119205 ------------------------------------------------------------

DH55 ref genome TTTTTGGTGCGAGACTT-----GATCCAAACATCTAG---TCTTGTCACGCTGGAAGGAT

09-CS0040 TT---------AGATTTTCCACAATGCAACAATGTGGATACAATGCTGTGAGTCACATTC

CN 113754 TT---------AGATTTTCCACAATGCAACAATGTGGATACAATGCTGTGAGTCACATTC

CO46 NCBI TT---------AGATTTTCCACAATGCAACAATGTGGATACAATGCTGTGAGTCACATTC

Jasper TT---------AGATTTTCCACAATGCAACAATGTGGATACAATGCTGTGAGTCACATTC

Joelle phyto TTTTTGGTGCGAGACTT-----GATCCAAACATCTAG---TCTTGTCACGCTGGAAGGAT

Joelle NCBI TTTTTGGTGCGAGACTTGATCCAAACATCTAGTCTTGTCACGCTGG--------AAGGAT

CN 119243 ------------------------------------------------------------

CN 120025 ------------------------------------------------------------

Joelle AAFC ------------------------------------------------------------

CAM 241 TTTAAC-----------TGGTCATGAAGTTCA------TCGGAATCTGAAAAACTTCCAA

17CS1133 TTTAAC-----------TGGTCATGAAGTTCA------TCAGAATCTGAAAAACTTCCAA

CAM 236 TTTAAC-----------TGGTCATGAAGTTCA------TC-GAATCTGAAAAACTTCCAA

Blaine Creek TTTAAC-----------TGGTCATGAAGTTCA------TCAGAATCTGAAAAACTTCCAA

CN 119300 TTTAAC-----------TGGTCATGAAGTTCA------TCAGAATCTGAAAAACTTCCAA

Yellowstone TTTAAC-----------TGGTCATGAAGTTCA------TCAGAATCTGAAAAACTTCCAA

Hoga TTTAAC-----------TGGTCATGAAGTTCA------TCAGAATCTGAAAAACTTCCAA

CN 120027 TTTAAC-----------TGGTCATGAAGTTCA------TCGGAATCTGAAAAACTTCCAA

CN 120030 TTTAAC-----------TGGTCATGAAGTTCA------TCAGAATCTGAAAAACTTCCAA

CN 119294 TTTAAC-----------TGGTCATGAAGTTCA------TCAGAATCTGAAAAACTTCCAA

CN 120013 TTTAAC-----------TGGTCATGAAGTTCA------TCAGAATCTGAAAAACTTCCAA

CN 120017 TTTAAC-----------TGGTCATGAAGTTCA------TCAGAATCTGAAAAACTTCCAA

CN 119205 ------------------------------------------------------------

DH55 ref genome CTTCACTTTCAGCTTTCTGTTCCAGAAAACAAAAT--TTCAGTAACAGTACAA---GCAC

09-CS0040 TTTTCT----------ATGTGCATTACATCCAAATTATGCCGTACTTGGTAGATCCTCCC

CN 113754 TTTTCT----------ATGTGCATTACATCCAAATTATGCCGTA-CTGGTAGATCCTCCC

CO46 NCBI TTTTCT----------ATGTGCATTACATCCAAATTATGCCGTA-CTGGTAGATCCTCCC

Jasper TTTTCT----------ATGTGCATTACATCCAAATTATGCCGTA-CTGGTAGATCCTCCC

Joelle phyto CTTCACTTTCAGCTTTCTGTTCCAGAAAACAAAAT--TTCAGTAACAGTACAA---GCAC

Joelle NCBI CTTCACTTTCAGCTTTCTGTTCCAGAAAACAAAAT--TTCAGTAACAGTACAA---GCAC

CN 119243 ------------------------------------------------------------

CN 120025 ------------------------------------------------------------

Joelle AAFC ------------------------------------------------------------

CAM 241 AATGATTTCGGAAATGTGAAAAAG-----------------------------GCTGGGA

17CS1133 AATGATTTCGGAAATGTGAAAAAG-----------------------------GCTGGGA

CAM 236 AATGATTTCGGAAATGTGAAAAAG-----------------------------GCTGGGA

Blaine Creek AATGATTTCGGAAATGTGAAAAAG-----------------------------GCTGGGA

CN 119300 AATGATTTCGGAAATGTGAAAAAG-----------------------------GCTGGGA

Yellowstone AATGATTTCGGAAATGTGAAAAAG-----------------------------GCTGGGA

Hoga AATGATTTCGGAAATGTGAAAAAG-----------------------------GCTGGGA

CN 120027 AATGATTTCGGAAATGTGAAAAAG-----------------------------GCTGGGA

CN 120030 AATGATTTCGGAAATGTGAAAAAG-----------------------------GCTGGGA

CN 119294 AATGATTTCGGAAATGTGAAAAAG-----------------------------GCTGGGA

CN 120013 AATGATTTCGGAAATGTGAAAAAG-----------------------------GCTGGGA

CN 120017 AATGATTTCGGAAATGTGAAAAAG-----------------------------GCTGGGA

CN 119205 ------------------------------------------------------------

DH55 ref genome TATGTATAATAATAAACAACATAGTTGTAGTATATATGCTTGCCATTTCTTCTGTTAGTC

09-CS0040 AATAAGG-CGAGTAAATAAAATGG--------------------ATCTTTTCTTCCACCT

CN 113754 AATAAGGCAGAGTAAATAAAATGG--------------------ATCTTTTCTTCCACCT

CO46 NCBI AATAAGGCAGAGTAAATAAAATGG--------------------ATCTTTTCTTCCACCT

Jasper AATAAGGCAGAGTAAATAAAATGG--------------------ATCTTTTCTTCCACCT

Joelle phyto TATGTATAATAATAAACAACATAGTTGTAGTATATATGCTTGCCATTTCTTCTGTTAGTC

Joelle NCBI TATGTATAATAATAAACAACATAGTTGTAGTATATATGCTTGCCATTTCTTCTGTTAGTC

CN 119243 ------------------------------------------------------------

CN 120025 ------------------------------------------------------------

Joelle AAFC ------------------------------------------------------------

CAM 241 TGAAGA------------------------------------------------------

17CS1133 TGAAGA------------------------------------------------------

CAM 236 TGAAGA------------------------------------------------------

Blaine Creek TGAAGA------------------------------------------------------

CN 119300 TGAAGA------------------------------------------------------

Yellowstone TGAAGA------------------------------------------------------

Hoga TGAAGA------------------------------------------------------

CN 120027 TGAAGA------------------------------------------------------

CN 120030 TGAAGA------------------------------------------------------

CN 119294 TGAAGA------------------------------------------------------

CN 120013 TGAAGA------------------------------------------------------

CN 120017 TGAAGA------------------------------------------------------

CN 119205 ------------------------------------------------------------

DH55 ref genome TGCTGATCCCTTTACGACTAGTGGTGTGAGGTATCTGATTCTTTCTTTTTGCTTTATATT

09-CS0040 TGATAA-----------------------------------CTCATCTTCATCTACTTCT

CN 113754 TGATAA-----------------------------------CTCATCTTCATCTACTTCT

CO46 NCBI TGATAA-----------------------------------CTCATCTTCATCTACTTCT

Jasper TGATAA-----------------------------------CTCATCTTCATCTACTTCT

Joelle phyto TGCTGATCCCTTTACGACTAGTGGTGTGAGGTATCTGATTCTTTCTTTTTGCTTTATATT

Joelle NCBI TGCTGATCCCTTTACGACTAGTGGTGTGAGGTATCTGATTCTTTCTTTTTGCTTTATATT

CN 119243 ------------------------------------------------------------

CN 120025 ------------------------------------------------------------

Joelle AAFC ------------------------------------------------------------

CAM 241 -------------------------------GAAAGAGAACTGTCTATAAAGAACCAGTG

17CS1133 -------------------------------GAAAGAGAACTGTCTATAAAGAACCAGTG

CAM 236 -------------------------------GAAAGAGAACTGTCTATAAAGAACCAGTG

Blaine Creek -------------------------------GAAAGAGAACTGTCTATAAAGAACCAGTG

CN 119300 -------------------------------GAAAGAGAACTGTCTATAAAGAACCAGTG

Yellowstone -------------------------------GAAAGAGAACTGTCTATAAAGAACCAGTG

Hoga -------------------------------GAAAGAGAACTGTCTATAAAGAACCAGTG

CN 120027 -------------------------------GAAAGAGAACTGTCTATAAAGAACCAGTG

CN 120030 -------------------------------GAAAGAGAACTGTCTATAAAGAACCAGTG

CN 119294 -------------------------------GAAAGAGAACTGTCTATAAAGAACCAGTG

CN 120013 -------------------------------GAAAGAGAACTGTCTATAAAGAACCAGTG

CN 120017 -------------------------------GAAAGAGAACTGTCTATAAAGAACCAGTG

CN 119205 ------------------------------------------------------------

DH55 ref genome TCTCACTCACGGCCTAATAATGAAACATGTTAATTACTAATCACCAATCAACTACTAAAC

09-CS0040 ACTTCCTCATCTTCATCGGATTCACTTTCATCATC----ATCATCTGTCAAACACTGGTT

CN 113754 ACTTCCTCATCTTCATCGGATTCACTTTCATCATCATCA-----CTGTCAAACACTGGTT

CO46 NCBI ACTTCCTCATCTTCATCGGATTCACTTTCATCATCATCA-----CTGTCAAACACTGGTT

Jasper ACTTCCTCATCTTCATCGGATTCACTTTCATCATCATCA-----CTGTCAAACACTGGTT

Joelle phyto TCTCACTCACGGCCTAATAATGAAACATGTTAATTACTAATCACCAATCAACTACTAAAC

Joelle NCBI TCTCACTCACGGCCTAATAATGAAACATGTTAATTACTAATCACCAATCAACTACTAAAC

CN 119243 ------------------------------------------------------------

CN 120025 ------------------------------------------------------------

Joelle AAFC ------------------------------------------------------------

CAM 241 TTTGACAGTG------------------------------------------ATGATGAT

17CS1133 TTTGACAGTG------------------------------------------ATGATGAT

CAM 236 TTTGACAGTG------------------------------------------ATGATGAT

Blaine Creek TTTGACAGTG------------------------------------------ATGATGAT

CN 119300 TTTGACAGTG------------------------------------------ATGATGAT

Yellowstone TTTGACAGTG------------------------------------------ATGATGAT

Hoga TTTGACAGTG------------------------------------------ATGATGAT

CN 120027 TTTGACAGTG------------------------------------------ATGATGAT

CN 120030 TTTGACAGTG------------------------------------------ATGATGAT

CN 119294 TTTGACAGTG------------------------------------------ATGATGAT

CN 120013 TTTGACGGTG------------------------------------------ATGATGAT

CN 120017 TTTGACAGTG------------------------------------------ATGATGAT

CN 119205 ------------------------------------------------------------

DH55 ref genome AATTATAATCACAAGTAATTACCTTAAATGCAGC------------------ACTAGTTT

09-CS0040 CTTTATAGACAGTTCTCTTTCTCTTCATCCCAGCCTTTTTCACATTTCCGAAATCATTTT

CN 113754 CTTTATAGACAGTTCTCTTTCTCTTCATCCCAGCCTTTTTCACATTTCCGAAATCATTTT

CO46 NCBI CTTTATAGACAGTTCTCTTTCTCTTCATCCCAGCCTTTTTCACATTTCCGAAATCATTTT

Jasper CTTTATAGACAGTTCTCTTTCTCTTCATCCCAGCCTTTTTCACATTTCCGAAATCATTTT

Joelle phyto AATTATAATCACAAGTAATTACCTTAAATGCAGC------------------ACTAGTTT

Joelle NCBI AATTATAATCACAAGTAATTACCTTAAATGCAGC------------------ACTAGTTT

CN 119243 ------------------------------------------------------------

CN 120025 ------------------------------------------------------------

Joelle AAFC ------------------------------------------------------------

CAM 241 GAAAGTGAATCCGATGAAGATGA-----GGAAGTAGAAGTAGATGAAGATGAGTT-----

17CS1133 GAAAGTGAATCCGATGAAGATGA-----GGAAGTAGAAGTAGATGAAGATGAGTT-----

CAM 236 GAAAGTGAATCCGATGAAGATGA-----GGAAGTAGAAGTAGATGAAGATGAGTT-----

Blaine Creek GAAAGTGAATCCGATGAAGATGA-----GGAAGTAGAAGTAGATGAAGATGAGTT-----

CN 119300 GAAAGTGAATCCGATGAAGATGA-----GGAAGTAGAAGTAGATGAAGATGAGTT-----

Yellowstone GAAAGTGAATCCGATGAAGATGA-----GGAAGTAGAAGTAGATGAAGATGAGTT-----

Hoga GAAAGTGAATCCGATGAAGATGA-----GGAAGTAGAAGTAGATGAAGATGAGTT-----

CN 120027 GAAAGTGAATCCGATGAAGATGA-----GGAAGTAGAAGTAGATGAAGATGAGTT-----

CN 120030 GAAAGTGAATCCGATGAAGATGA-----GGAAGTAGAAGTAGATGAAGATGAGTT-----

CN 119294 GAAAGTGAATCCGATGAAGATGA-----GGAAGTAGAAGTAGATGAAGATGAGTT-----

CN 120013 GAAAGTGAATCCGATGAAGATGA-----GGAAGTAGAAGTAGATGAAGATGAGTT-----

CN 120017 GAAAGTGAATCCGATGAAGATGA-----GGAAGTAGAAGTAGATGAAGATGAGTT-----

CN 119205 ------------------------------------------------------------

DH55 ref genome TGAGTTTGACAAATTTTTGCCAATCAACAGGACCAATATTTGGTGGTCTCAAATTCATCC

09-CS0040 GGAAGTTTTTCAGATTC-GATGAACTTCATGACCAG---TTAAAATTCTTGACTTTCTCC

CN 113754 GGAAGTTTTTCAGATTCTGATGAACTTCATGACCAG---TTAAAATTCTTGACTTTCTCC

CO46 NCBI GGAAGTTTTTCAGATTCTGATGAACTTCATGACCAG---TTAAAATTCTTGACTTTCTCC

Jasper GGAAGTTTTTCAGATTCTGATGAACTTCATGACCAG---TTAAAATTCTTGACTTTCTCC

Joelle phyto TGAGTTTGACAAATTTTTGCCAATCAACAGGACCAATATTTGGTGGTCTCAAATTCATCC

Joelle NCBI TGAGTTTGACAAATTTTTGCCAATCAACAGGACCAATATTTGGTGGTCTCAAATTCATCC

CN 119243 ------------------------------------------------------------

CN 120025 ------------------------------------------------------------

Joelle AAFC ------------------------------------------------------------

CAM 241 ----ATCAAGGTGGAAGAAAAGATCCATTTTATTTACTCTGCCTTATTGGGAGGATCTAC

17CS1133 ----ATCAAGGTGGAAGAAAAGATCCATTTTATTTACTCTGCCTTATTGGGAGGATCTAC

CAM 236 ----ATCAAGGTGGAAGAAAAGATCCATTTTATTTACTCGCTCTTATTGGGAGGATCTAC

Blaine Creek ----ATCAAGGTGGAAGAAAAGATCCATTTTATTTACTCTGCCTTATTGGGAGGATCTAC

CN 119300 ----ATCAAGGTGGAAGAAAAGATCCATTTTATTTACTCTGCCTTATTGGGAGGATCTAC

Yellowstone ----ATCAAGGTGGAAGAAAAGATCCATTTTATTTACTCTGCCTTATTGGGAGGATCTAC

Hoga ----ATCAAGGTGGAAGAAAAGATCCATTTTATTTACTCTGCCTTATTGGGAGGATCTAC

CN 120027 ----ATCAAGGTGGAAGAAAAGATCCATTTTATTTACTCTGCCTTATTGGGAGGATCTAC

CN 120030 ----ATCAAGGTGGAAGAAAAGATCCATTTTATTTACTCTGCCTTATTGGGAGGATCTAC

CN 119294 ----ATCAAGGTGGAAGAAAAGATCCATTTTATTTACTCTGCCTTATTGGGAGGATCTAC

CN 120013 ----ATCAAGGTGGAAGAAAAGATCCATTTTATTTACTCTGCCTTATTGGGAGGATCTAC

CN 120017 ----ATCAAGGTGGAAGAAAAGATCCATTTTATTTACTC-GCCTTATTGGGAGGATCTAC

CN 119205 ------------------------------------------------------------

DH55 ref genome TCTCTTGGTTATTTGCAGCCAAATTTATAGCCTTCACCGTGACAGACTTGTGTGAT----

09-CS0040 TATGCTCAACTTTTCCATCAAACCAAGTCTTCTTTTCCCTATATCTGTGTGTTG------

CN 113754 TATGCTCAACTTTTCCATCAAACCAAGTCTTCTTTTCCCTATATCTGTGTGTTG------

CO46 NCBI TATGCTCAACTTTTCCATCAAACCAAGTCTTCTTTTCCCTATATCTGTGTGTTG------

Jasper TATGCTCAACTTTTCCATCAAACCAAGTCTTCTTTTCCCTATATCTGTGTGTTG------

Joelle phyto TCTCTTGGTTATTTGCAGCCAAATTTATAGCCTTCACCGTGACAGACTTGTGTGAT----

Joelle NCBI TCTCTTGGTTATTTGCAGCCAAATTTATAGCCTTCACCGTGACAGACTTGTGTGAT----

CN 119243 ------------------------------------------------------------

CN 120025 ------------------------------------------------------------

Joelle AAFC ------------------------------------------------------------

CAM 241 CAG-TACGGCATAATTTGGATGTAATGCACATAGAAAAGAATGTGACTCA----------

17CS1133 CAG-TACGGCATAATTTGGATGTAATGCACATAGAAAAGAATGTGACTCA----------

CAM 236 CAAGTACGGCATAATTTGGATGTAATGCACATAGAAAAGAATGTGACTCA----------

Blaine Creek CAG-TACGGCATAATTTGGATGTAATGCACATAGAAAAGAATGTGACTCA----------

CN 119300 CAG-TACGGCATAATTTGGATGTAATGCACATAGAAAAGAATGTGACTCA----------

Yellowstone CAAGTACGGCATAATTTGGATGTAATGCACATAGAAAAGAATGTGACTCA----------

Hoga CAG-TACGGCATAATTTGGATGTAATGCACATAGAAAAGAATGTGACTCA----------

CN 120027 CAG-TACGGCATAATTTGGATGTAATGCACATAGAAAAGAATGTGACTCA----------

CN 120030 CAG-TACGGCATAATTTGGATGTAATGCACATAGAAAAGAATGTGACTCA----------

CN 119294 CAG-TACGGCATAATTTGGATGTAATGCACATAGAAAAGAATGTGACTCA----------

CN 120013 CAG-TACGGCATAATTTGGATGTAATGCACATAGAAAAGAATGTGACTCA----------

CN 120017 CAGATGCGGCATAATTTGGATGTAATGCACATAGAAAAGAATGTGACTCA----------

CN 119205 ------------------------------------------------------------

DH55 ref genome ----CGCCACAAATGTCCCATCTGGTGAAT-CACAGATATCCTTTGCCAATCTTCATCAA

09-CS0040 -----GAGCCAAACCTTTTCTATGACACATGTAGACATGTTTCCTGCTAAACTTCAACCA

CN 113754 -----GAGCCAAACCTTTTCTATGACACATGTAGACATGTTTCCTGCTAAACTTCAACCA

CO46 NCBI -----GAGCCAAACCTTTTCTATGACACATGTAGACATGTTTCCTGCTAAACTTCAACCA

Jasper -----GAGCCAAACCTTTTCTATGACACATGTAGACATGTTTCCTGCTAAACTTCAACCA

Joelle phyto ----CGCCACAAATGTCCCATCTGGTGAAT-CACAGATATCCTTTGCCAATCTTCATCAA

Joelle NCBI ----CGCCACAAATGTCCCATCTGGTGAAT-CACAGATATCCTTTGCCAATCTTCATCAA

CN 119243 ------------------------------------------------------------

CN 120025 ------------------------------------------------------------

Joelle AAFC ------------------------------------------------------------

CAM 241 -------------CAGCATTGTATCCACATT------------------GT---------

17CS1133 -------------CAGCATTGTATCCACATT------------------GT---------

CAM 236 -------------CAGCATTGTATCCACATT------------------GT---------

Blaine Creek -------------CAGCATTGTATCCACATT------------------GT---------

CN 119300 -------------CAGCATTGTATCCACATT------------------GT---------

Yellowstone -------------CAGCATTGTATCCACATT------------------GT---------

Hoga -------------CAGCATTGTATCCACATT------------------GT---------

CN 120027 -------------CAGCATTGTATCCACATT------------------GT---------

CN 120030 -------------CAGCATTGTATCCACATT------------------GT---------

CN 119294 -------------CAGCATTGTATCCACATT------------------GT---------

CN 120013 -------------CAGCATTGTATCCACATT------------------GT---------

CN 120017 -------------CAGCATTGTATCCACATT------------------GT---------

CN 119205 ------------------------------------------------------------

DH55 ref genome GCTCAAACCTTGCCTGCATT-CACTTACACTCACACCATNNNNNNNNNNNNNNNNNNNNN

09-CS0040 CATACTAT-----CAGTATT-TTCCCACACATAGGACATCCCATTTTACCT---------

CN 113754 CATACTAT-----CAGTATTTTTCCCACACATAGGACATCCCATTTTACCT---------

CO46 NCBI CATACTAT-----CAGTATTTTTCCCACACATAGGACATCCCATTTTACCT---------

Jasper CATACTAT-----CAGTATTTTTCCCACACATAGGACATCCCATTTTACCT---------

Joelle phyto GCTCAAACCTTGCCTGCATT-CACTTACACTCACACCATAACTATAAAAGTAAACATGTT

Joelle NCBI GCTCAAACCTTGCCTGCATT-CACTTACACTCACACCATAACTATAAAAGTAAACATGTT

CN 119243 ------------------------------------------------------------

CN 120025 ------------------------------------------------------------

Joelle AAFC ------------------------------------------------------------

CAM 241 --------TGCATTGTGGAAAATCTAAGGATGGTCTTAATGCTCGTAAGGATCTTCAACA

17CS1133 --------TGCATTGTGGAAAATCTAAGGATGGTCTTAATGCTCGTAAGGATCTTCAACA

CAM 236 --------TGCATTGTGGAAAATCTAAGGATGGTCTTAATGCTCGTAAGGATCTTCAACA

Blaine Creek --------TGCATTGTGGAAAATCTAAGGATGGTCTTAATGCTCGTAAGGATCTTCAACA

CN 119300 --------TGCATTGTGGAAAATCTAAGGATGGTCTTAATGCTCGTAAGGATCTTCAACA

Yellowstone --------TGCATTGTGGAAAATCTAAGGATGGTCTTAATGCTCGTAAGGATCTTCAACA

Hoga --------TGCATTGTGGAAAATCTAAGGATGGTCTTAATGCTCGTAAGGATCTTCAACA

CN 120027 --------TGCATTGTGGAAAATCTAAGGATGGTCTTAATGCTCGTAAGGATCTTCAACA

CN 120030 --------TGCATTGTGGAAAATCTAAGGATGGTCTTAATGCTCGTAAGGATCTTCAACA

CN 119294 --------TGCATTGTGGAAAATCTAAGGATGGTCTTAATGCTCGTAAGGATCTTCAACA

CN 120013 --------TGCATTGTGGAAAATCTAAGGATGGTCTTAATGCTCGTAAGGATCTTCAACA

CN 120017 --------TGCATTGTGGAAAATCTAAGGATGGTCTTAATGCTCGTAAGGATCTTCAACA

CN 119205 ------------------------------------------------------------

DH55 ref genome NNNNNNNNNNNNNNNNNNNNNNNNNNNNNNNNNNNNNNNNNNNNNNNNNNNNNNNNNNNN

09-CS0040 --------TTTACTTTACAA----CCAGCAAGATTTCCATACGCAGGAAAATCACTAA--

CN 113754 --------TTTACTTTACAA----CCAGCAAGATTTCCATACGCAGGAAAATCACTAA--

CO46 NCBI --------TTTACTTTACAA----CCAGCAAGATTTCCATACGCAGGAAAATCACTAA--

Jasper --------TTTACTTTACAA----CCAGCAAGATTTCCATACGCAGGAAAATCACTAA--

Joelle phyto CAATTGAGAGACTTTTTAAAAACAAAAAAAAATTCTTGTTACCTGAACAGATCTCCA---

Joelle NCBI CAATTGAGAGACTTTTTAAAAACAAAAAAAAATTCTTGTTACCTGAACAGATCTCCAGAG

CN 119243 ------------------------------------------------------------

CN 120025 ------------------------------------------------------------

Joelle AAFC ------------------------------------------------------------

CAM 241 TCTTGGTCTAAGAAAAGAGTTGCATCCTACCACAAAAGGAAAGAGAACATATCTTCCAGC

17CS1133 TCTTGGTCTAAGAAAAGAGTTGCATCCTACCACAAAAGGAAAGAGAACATATCTTCCAGC

CAM 236 TCTTGGTCTAAGAAAAGAGTTGCATCCTACCACAAAAGGAAAGAGAACATATCTTCCAGC

Blaine Creek TCTTGGTCTAAGAAAAGAGTTGCATCCTACCACAAAAGGAAAGAGAACATATCTTCCAGC

CN 119300 TCTTGGTCTAAGAAAAGAGTTGCATCCTACCACAAAAGGAAAGAGAACATATCTTCCAGC

Yellowstone TCTTGGTCTAAGAAAAGAGTTGCATCCTACCACAAAAGGAAAGAGAACATATCTTCCAGC

Hoga TCTTGGTCTAAGAAAAGAGTTGCATCCTACCACAAAAGGAAAGAGAACATATCTTCCAGC

CN 120027 TCTTGGTCTAAGAAAAGAGTTGCATCCTACCACAAAAGGAAAGAGAACATATCTTCCAGC

CN 120030 TCTTGGTCTAAGAAAAGAGTTGCATCCTACCACAAAAGGAAAGAGAACATATCTTCCAGC

CN 119294 TCTTGGTCTAAGAAAAGAGTTGCATCCTACCACAAAAGGAAAGAGAACATATCTTCCAGC

CN 120013 TCTTGGTCTAAGAAAAGAGTTGCATCCTACCACAAAAGGAAAGAGAACATATCTTCCAGC

CN 120017 TCTTGGTCTAAGAAAAGAGTTGCATCCTACCACAAAAGGAAAGAGAACATATCTTCCAGC

CN 119205 ------------------------------------------------------------

DH55 ref genome NNNNNNNNNNNNNNNNNNNNNNNNNNNNNNNNNNNNNNNNNNNNNNNNNNNNNNNNNNNN 09-CS0040 ---TGGTCCA-GAGAAGCATTGCCTTT--------------AGAGTAAATGTACTTTTAC

CN 113754 ---TGGTCCA-GAGAAGCATTGCCTTT--------------AGAGTAAATGTACTTTTAC

CO46 NCBI ---TGGTCCA-GAGAAGCATTGCCTTT--------------AGAGTAAATGTACTTTTAC

Jasper ---TGGTCCA-GAGAAGCATTGCCTTT--------------AGAGTAAATGTACTTTTAC

Joelle phyto -----------GAGAAGTGTTTT-----------AAGATCTGGACTCACTTTCTTCCAGT

Joelle NCBI AAGTGTTTTAAGATCTGGACTCACTT-------------------------TCTTCCAGT

CN 119243 ------------------------------------------------------------

CN 120025 ------------------------------------------------------------

Joelle AAFC ------------------------------------------------------------

CAM 241 TG-------CAAGAGTAACATAT-----CTTTGTTTATGTTACAGA-GACCAGAAGCTGA

17CS1133 TG-------CAAGAGTAACATAT-----CTTTGTTTATGTTACAGA-GACCAGAAGCTGA

CAM 236 TG-------CAAGAGTAACATAT-----CTTTGTTTATGTTACAGA-GACCAGAAGCTGA

Blaine Creek TG-------CAAGAGTAACATAT-----CTTTGTTTATGTTACAGA-GACCAGAAGCTGA

CN 119300 TG-------CAAGAGTAACATAT-----CTTTGTTTATGTTACAGA-GACCAGAAGCTGA

Yellowstone TG-------CAAGAGTAACATAT-----CTTTGTTTATGTTACAGA-GACCAGAAGCTGA

Hoga TG-------CAAGAGTAACATAT-----CTTTGTTTATGTTACAGA-GACCAGAAGCTGA

CN 120027 TG-------CAAGAGTAACATAT-----CTTTGTTTATGTTACAGA-GACCAGAAGCTGA

CN 120030 TG-------CAAGAGTAACATAT-----CTTTGTTTATGTTACAGA-GACCAGAAGCTGA

CN 119294 TG-------CAAGAGTAACATAT-----CTTTGTTTATGTTACAGA-GACCAGAAGCTGA

CN 120013 TG-------CAAGAGTAACATAT-----CTTTGTTTATGTTACAGA-GACCAGAAGCTGA

CN 120017 TG-------CAAGAGTAACATAT-----CTTTGTTTATGTTACAGA-GACCAGAAGCTGA

CN 119205 ------------------------------------------------------------

DH55 ref genome NNNNNNNNNNNNNNNNNNNNNNNNNNNNNNNNNNNNNNNNNNNNNNNNNNNNNNNNNNNN

09-CS0040 TA-------AAAGCATCATACGTTAGCTCTCCATTCTT-CCACAGATGATTCAAATCCTC

CN 113754 TA-------AAAGCATCATACGTTAGCTCTCCATTCTT-CCACAGATGATTCAAATCCTC

CO46 NCBI TA-------AAAGCATCATACGTTAGCTCTCCATTCTT-CCACAGATGATTCAAATCCTC

Jasper TA-------AAAGCATCATACGTTAGCTCTCCATTCTT-CCACAGATGATTCAAATCCTC

Joelle phyto TAGGTATGTTGATAGGGATGTGTTCTCGCATCAATGTCCCTACATA-----TGATGCCAA

Joelle NCBI TAGGTATGTTGATAGGGATGTGTTCTCGCATCAATGTCCCTACATA-----TGATGCCAA

CN 119243 ------------------------------------------------------------

CN 120025 ------------------------------------------------------------

Joelle AAFC ------------------------------------------------------------

CAM 241 AATGGATGA---------AAACTCA----------------------------G---CTG

17CS1133 AATGGATGA---------AAACTCA----------------------------G---CTG

CAM 236 AATGGATGA---------AAACTCA----------------------------G---CTG

Blaine Creek AATGGATGA---------AAACTCA----------------------------G---CTG

CN 119300 AATGGATGA---------AAACTCA----------------------------G---CTG

Yellowstone AATGGATGA---------AAACTCA----------------------------G---CTG

Hoga AATGGATGA---------AAACTCA----------------------------G---CTG

CN 120027 AATGGATGA---------AAACTCA----------------------------G---CTG

CN 120030 AATGGATGA---------AAACTCA----------------------------G---CTG

CN 119294 AATGGATGA---------AAACTCA----------------------------G---CTG

CN 120013 AATGGATGA---------AAACTCA----------------------------G---CTG

CN 120017 AATGGATGA---------AAACTCA----------------------------G---CTG

CN 119205 ------------------------------------------------------------

DH55 ref genome NNNNNNNNNNNNNNNNNNNNNNNNNNNNNNNNNNNNNNNNNNNNNNNNNNNNNNNNNNNN

09-CS0040 AATAAGAGGTTCTAGGTAGACATCAATATTATTACCTGGTTGTTGTGGACCAGGAATCAA

CN 113754 AATAAGAGGTTCTAGGTAGACATCAATATTATTACCTGGTTGTTGTGGACCAGGAATCAA

CO46 NCBI AATAAGAGGTTCTAGGTAGACATCAATATTATTACCTGGTTGTTGTGGACCAGGAATCAA

Jasper AATAAGAGGTTCTAGGTAGACATCAATATTATTACCTGGTTGTTGTGGACCAGGAATCAA

Joelle phyto CTTGACTGACCCTGGACCAACCGCT----------------------------A---CTC

Joelle NCBI CTTGACTGACCCTGGACCAACCGCT----------------------------A---CTC

CN 119243 ------------------------------------------------------------

CN 120025 ------------------------------------------------------------

Joelle AAFC ------------------------------------------------------------

CAM 241 CAAGAGTAACATATTCTTACTTCTTTTCA----------TATCAGCTAAGTGATTCTTCT

17CS1133 CAAGAGTAACATATTCTTACTTCTTTTCA----------TATCAGCTAAGTGATTCTTCT

CAM 236 CAAGAGTAACATATTCTTACTTCTTTTCA----------TATCAGCTAAGTGATTCTTCT

Blaine Creek CAAGAGTAACATATTCTTACTTCTTTTCA----------TATCAGCTAAGTGATTCTTCT

CN 119300 CAAGAGTAACATATTCTTACTTCTTTTCA----------TATCAGCTAAGTGATTCTTCT

Yellowstone CAAGAGTAACATATTCTTACTTCTTTTCA----------TATCAGCTAAGTGATTCTTCT

Hoga CAAGAGTAACATATTCTTACTTCTTTTCA----------TATCAGCTAAGTGATTCTTCT

CN 120027 CAAGAGTAACATATTCTTACTTCTTTTCA----------TATCAGCTAAGTGATTCTTCT

CN 120030 CAAGAGTAACATATTCTTACTTCTTTTCA----------TATCAGCTAAGTGATTCTTCT

CN 119294 CAAGAGTAACATATTCTTACTTCTTTTCA----------TATCAGCTAAGTGATTCTTCT

CN 120013 CAAGAGTAACATATTCTTACTTCTTTTCA----------TATCAGCTAAGTGATTCTTCT

CN 120017 CAAGAGTAACATATTCTTACTTCTTTTCA----------TATCAGCTAAGTGATTCTTCT

CN 119205 ------------------------------------------------------------

DH55 ref genome NNNNNNNNNNNNNNNNNNNNNNNNNNNNNNNNNNNNNNNNNNNNNNNNNNNNNNNNNNNN

09-CS0040 TAATGTCAACATTATATTCTCCTTCTTCATACAAAGGTGAGGAGGCAAATTGTAGTTTAC

CN 113754 TAATGTCAACATTATATTCTCCTTCTTCATACAAAGGTGAGGAGGCAAATCGTAGTTTAC

CO46 NCBI TAATGTCAACATTATATTCTCCTTCTTCATACAAAGGTGAGGAGGCAAATTGTAGTTTAC

Jasper TAATGTCAACATTATATTCTCCTTCTTCATACAAAGGTGAGGAGGCAAATTGTAGTTTAC

Joelle phyto CAAGTACATTGTAATCAACTTTCACTCTAACAGATGGATCCTTGGCTAGGTTTTTCATCT

Joelle NCBI CAAGTACATTGTAATCAACTTTCACTCTAACAGATGGATCCTTGGCTAGGTTTTTCATCT

CN 119243 ------------------------------------------------------------

CN 120025 ------------------------------------------------------------

Joelle AAFC ------------------------------------------------------------

CAM 241 TAGTGTCGT----------TTATGATTCTT-------------AGTTGCTCATGAAATTG

17CS1133 TAGTGTCGT----------TTATGATTCTT-------------AGTTGCTCATGAAATTG

CAM 236 TAGTGTCGT----------TTATGATTCTT-------------AGTTGCTCATGAAATTG

Blaine Creek TAGTGTCGT----------TTATGATTCTT-------------AGTTGCTCATGAAATTG

CN 119300 TAGTGTCGT----------TTATGATTCTT-------------AGTTGCTCATGAAATTG

Yellowstone TAGTGTCGT----------TTATGATTCTT-------------AGTTGCTCATGAAATTG

Hoga TAGTGTCGT----------TTATGATTCTT-------------AGTTGCTCATGAAATTG

CN 120027 TAGTGTCGT----------TTATGATTCTT-------------AGTTGCTCATGAAATTG

CN 120030 TAGTGTCGT----------TTATGATTCTT-------------AGTTGCTCATGAAATTG

CN 119294 TAGTGTCGT----------TTATGATTCTT-------------AGTTGCTCATGAAATTG

CN 120013 TAGTGTCGT----------TTATGATTCTT-------------AGTTGCTCATGAAATTG

CN 120017 TAGTGTCGT----------TTATGATTCTT-------------AGTTGCTCATGAAATTG

CN 119205 ------------------------------------------------------------

DH55 ref genome NNNNNNNNNNNNNNNNNNNNNNNNNNNNNNNNNNNNNNNNNNNNNNNNNNNNNNNNNNNN 09-CS0040 CAATAGCACAGGCCAGCTACTATAATTACT--------------ATTCTTCATG------

CN 113754 CAATAGCACAGGCCAGCTACTATAATTACT--------------ATTCTTCATG------

CO46 NCBI CAATAGCACAGGCCAGCTACTATAATTACT--------------ATTCTTCATG------

Jasper CAATAGCACAGGCCAGCTACTATAATTACT--------------ATTCTTCATG------

Joelle phyto TTGTTG-GT----------CCTCGTTTTCTTTTACGCGTAGCAGGCTGCTCATGTTCTTC

Joelle NCBI TTGT-TGGT----------CCTCGTTTTCTTTTACGCGTAGCAGGCTGCTCATGTTCTTC

CN 119243 ------------------------------------------------------------

CN 120025 ------------------------------------------------------------

Joelle AAFC ------------------------------------------------------------

CAM 241 TTGCAGAGTGTAAA--CAAAAGAGCACATCCTAAG-TGTGTGTTTGTTGATTGGTCTGAC

17CS1133 TTGCAGAGTGTAAA--CAAAAGAGCACATCCTAAG-TGTGTGTTTGTTGATTGGTCTGAC

CAM 236 TTGCAGAGTGTAAA--CAAAAGAGCACATCCTAAG-TGTGTGTTTGTTGATTGGTCTGAC

Blaine Creek TTGCAGAGTGTAAA--CAAAAGAGCACATCCTAAG-TGTGTGTTTGTTGATTGGTCTGAC

CN 119300 TTGCAGAGTGTAAA--CAAAAGAGCACATCCTAAG-TGTGTGTTTGTTGATTGGTCTGAC

Yellowstone TTGCAGAGTGTAAA--CAAAAGAGCACATCCTAAG-TGTGTGTTTGTTGATTGGTCTGAC

Hoga TTGCAGAGTGTAAA--CAAAAGAGCACATCCTAAG-TGTGTGTTTGTTGATTGGTCTGAC

CN 120027 TTGCAGAGTGTAAA--CAAAAGAGCACATCCTAAG-TGTGTGTTTGTTGATTGGTCTGAC

CN 120030 TTGCAGAGTGTAAA--CAAAAGAGCACATCCTAAG-TGTGTGTTTGTTGATTGGTCTGAC

CN 119294 TTGCAGAGTGTAAA--CAAAAGAGCACATCCTAAG-TGTGTGTTTGTTGATTGGTCCGAC

CN 120013 TTGCAGAGTGTAAA--CAAAAGAGCACATCCTAAG-TGTGTGTTTGTTGATTGGTCTGAC

CN 120017 TTGCAGAGTGTAAA--CAAAAGAGCACATCCTAAG-TGTGTGTTTGTTGATTGGTCCGAC

CN 119205 ------------------------------------------------------------

DH55 ref genome NNNNNNNNNNNNNNNNNNNNNNNNNNNNNNNNNNNNNNNNNNNNNNNNNNNNNNNNNNNN

09-CS0040 TTGAATGGATTAAATCCATCTGTGGACAGCCCAAGCCGTATGTTCCTTTCTTCAACTGCA

CN 113754 TTGAATGGATTAAATCCATCTGTGGACAGCCCAAGCCGTATGTTCCTTTCTTCAACTGCA

CO46 NCBI TTGAATGGATTAAATCCATCTGTGGACAGCCCAAGCCGTATGTTCCTTTCTTCAACTGCA

Jasper TTGAATGGATTAAATCCATCTGTGGACAGCCCAAGCCGTATGTTCCTTTCTTCAACTGCA

Joelle phyto AGACAA----------CACAAACTCTCCTACTGGTTCTTCTGGTTGCTGTTGAGTTTGTG

Joelle NCBI AGACAA----------CACAAACTCTCCTACTGGTTCTTCTGGTTGCTGTTGAGTTTGTG

CN 119243 ------------------------------------------------------------

CN 120025 ------------------------------------------------------------

Joelle AAFC ------------------------------------------------------------

CAM 241 ATTGATGAAAATG-------------------------------TTGGTGAGGGTCGGAT

17CS1133 ATTGATGAAAATG-------------------------------TTGGTGAGGGTCGGAT

CAM 236 ATTGATGAAAATG-------------------------------TTGGTGAGGGTCGGAT

Blaine Creek ATTGATGAAAATG-------------------------------TTGGTGAGGGTCGGAT

CN 119300 ATTGATGAAAATG-------------------------------TTGGTGAGGGTCGGAT

Yellowstone ATTGATGAAAATG-------------------------------TTGGTGAGGGTCGGAT

Hoga ATTGATGAAAATG-------------------------------TTGGTGAGGGTCGGAT

CN 120027 ATTGATGAAAATG-------------------------------TTGGTGAGGGTCGGAT

CN 120030 ATTGATGAAAATG-------------------------------TTGGTGAGGGTCGGAT

CN 119294 ATTGATGAAAATG-------------------------------TTGGTGAGGGTCGGAT

CN 120013 ATTGATGAAAATG-------------------------------TTGGTGAGGGTCGGAT

CN 120017 ATTGATGAAAATG-------------------------------TTGGTGAGGGTCGGAT

CN 119205 ------------------------------------------------------------

DH55 ref genome NNNNNNNNNNNNNNNNNNNNNNNNNNNNNNNNNNNNNNNNNNNNNNNNNNNNNNNNNNNN 09-CS0040 AATGAAGGATACTTCTCATTCATCTCGAGCCCATGTAACAGAATCTACTGGATGTCGAAG

CN 113754 AATGAAGGATACTTCTCATTCATCTGAGCCCATGTAACGGAAT-CTACTGGATGTCGAAG

CO46 NCBI AATGAAGGATACTTCTCATTCATCTGAGCCCATGTAACAGAAT-CTACTGGATGTCGAAG

Jasper AATGAAGGATACTTCTCATTCATCTGAGCCCATGTAACAGAAT-CTACTGGATGTCGAAG

Joelle phyto GTTCTTCGGAATGATGCTCCTCTTC--CTCCACATGCTGTTCTGTTGCTACAGGTTGGGC

Joelle NCBI GTTCTTCGGAATG--ATGCTCCTCTTCCTCCACATGCTGTTCTGTTGCTACAGGTTGGGC

CN 119243 ------------------------------------------------------------

CN 120025 ------------------------------------------------------------

Joelle AAFC ------------------------------------------------------------

CAM 241 TCTTTCTTCTGATCC----------GGATGA-----CATAGTGAA---------------

17CS1133 TCTTTCTTCTGATCC----------GGATGA-----CATAGTGAA---------------

CAM 236 TCTTTCTTCTGATCC----------GGATGA-----CATAGTGAA---------------

Blaine Creek TCTTTCTTCTGATCC----------GGATGA-----CATAGTGAA---------------

CN 119300 TCTTTCTTCTGATCC----------GGATGA-----CATAGTGAA---------------

Yellowstone TCTTTCTTCTGATCC----------GGATGA-----CATAGTGAA---------------

Hoga TCTTTCTTCTGATCC----------GGATGA-----CATAGTGAA---------------

CN 120027 TCTTTCTTCTGATCC----------GGATGA-----CATAGTGAA---------------

CN 120030 TCTTTCTTCTGATCC----------GGATGA-----CATAGTGAA---------------

CN 119294 TCTTTCTTCTGATCC----------GGATGA-----CATAGTGAA---------------

CN 120013 TCTTTCTTCTGATCC----------GGATGA-----CATAGTGAA---------------

CN 120017 TCTTTCTTCTGATCC----------GGATGA-----CATAGTGAA---------------

CN 119205 ------------------------------------------------------------

DH55 ref genome NNNNNNNNNNNNNNNNNNNNNNNNNNNNNNNNNNNNNNNNNNNNNNNNNNNNNNNNNNNN

09-CS0040 TTTTCCATCAGTGCTCTTGTTAGTGTAATGC-----CACCGTAAGTCCTTAGCCATGTCC

CN 113754 TTTTCCATCAGTGCTCTTGTTAGTGTAATGC-----CACCGTAAGTCCTTAGCCATGTCC

CO46 NCBI TTTTCCATCAGTGCTCTTGTTAGTGTAATGC-----CACCGTAAGTCCTTAGCCATGTCC

Jasper TTTTCCATCAGTGCTCTTGTTAGTGTAATGC-----CACCGTAAGTCCTTAGCCATGTCC

Joelle phyto CTCCACATGCTGTTCTGTTGCTACAGGTTGGGCCTCCACAGTACAAAT----AAAC----

Joelle NCBI CTCCACATGCTGTTCTGTTGCTACAGGTTGGGCCTCCACAGTACAAATAAAC--------

CN 119243 ------------------------------------------------------------

CN 120025 ------------------------------------------------------------

Joelle AAFC ------------------------------------------------------------

CAM 241 --------TGACTGTCGTCTTGGCCCTT---ATGATCTCAAAGTGTTAGTTGAAGCTGCA

17CS1133 --------TGACTGTCGTCTTGGCCCTT---ATGATCTCAAAGTGTTAGTTGAAGCTGCA

CAM 236 --------TGACTGTCGTCTTGGCCCTT---ATGATCTCAAAGTGTTAGTTGAAGCTGCA

Blaine Creek --------TGACTGTCGTCTTGGCCCTT---ATGATCTCAAAGTGTTAGTTGAAGCTGCA

CN 119300 --------TGACTGTCGTCTTGGCCCTT---ATGATCTCAAAGTGTTAGTTGAAGCTGCA

Yellowstone --------TGACTGTCGTCTTGGCCCTT---ATGATCTCAAAGTGTTAGTTGAAGCTGCA

Hoga --------TGACTGTCGTCTTGGCCCTT---ATGATCTCAAAGTGTTAGTTGAAGCTGCA

CN 120027 --------TGACTGTCGTCTTGGCCCTT---ATGATCTCAAAGTGTTAGTTGAAGCTGCA

CN 120030 --------TGACTGTCGTCTTGGCCCTT---ATGATCTCAAAGTGTTAGTTGAAGCTGCA

CN 119294 --------TGACTGTCGTCTTGGCCCTT---ATGATCTCAAAGTGTTAGTTGAAGCTGCA

CN 120013 --------TGACTGTCGTCTTGGCCCTT---ATGATCTCAAAGTGTTAGTTGAAGCTGCA

CN 120017 --------TGACTGTCGTCTTGGCCCTT---ATGATCTCAAAGTGTTAGTTGAAGCTGCA

CN 119205 ------------------------------------------------------------

DH55 ref genome NNNNNNNNNNNNNNNNNNNNNNNNNNNNNNNNNNNNNNNNNNNNNNNNNNNNNNNNNNNN

09-CS0040 TCT-GATCTGAACATTCTCTTCAGCCTTGGTATAATTGGAAAATATC--TTAATACTTTC

CN 113754 TCTCGATCTGAACATTCTCTTCAGCCTTGGTATAATTGGAAAATATC--TTAATACTTTC

CO46 NCBI TCTGATC-TGAACATTCTCTTCAGCCTTGGTATAATTGGAAAATATC--TTAATACTTTC

Jasper TCT-GATCTGAACATTCTCTTCAGCCTTGGTATAATTGGAAAATATC--TTAATACTTTC

Joelle phyto --------TCAGGTTCGTCATCATCCTTAGACTTTCTCTTTCTTTTTCCTCCACCCTTCT

Joelle NCBI --------TCAGGTTCGTCATCATCCTTAGACTTTCTCTTTCTTTTTCCTCCACCCTTCT

CN 119243 ------------------------------------------------------------

CN 120025 ------------------------------------------------------------

Joelle AAFC ------------------------------------------------------------

CAM 241 TATAAACCAGATGCATTTC-----------------TATGGAGACCTGCACAAAAGATCT

17CS1133 TATAAACCAGATGCATTTC-----------------TATGGAGACCTGCACAAAAGATCT

CAM 236 TATAAACCAGATGCATTTC-----------------TATGGAGACCTGCACAAAAGATCT

Blaine Creek TATAAACCAGATGCATTTC-----------------TATGGAGACCTGCACAAAAGATCT

CN 119300 TATAAACCAGATGCATTTC-----------------TATGGAGACCTGCACAAAAGATCT

Yellowstone TATAAACCAGATGCATTTC-----------------TATGGAGACCTGCACAAAAGATCT

Hoga TATAAACCAGATGCATTTC-----------------TATGGAGACCTGCACAAAAGATCT

CN 120027 TATAAACCAGATGCATTTC-----------------TATGGAGACCTGCACAAAAGATCT

CN 120030 TATAAACCAGATGCATTTC-----------------TATGGAGACCTGCACAAAAGATCT

CN 119294 TATAAACCAGATGCATTTC-----------------TATGGAGACCTGCACAAAAGATCT

CN 120013 TATAAACCAGATGCATTTC-----------------TATGGAGACCTGCACAAAAGATCT

CN 120017 TATAAACCAGATGCATTTC-----------------TATGGAGACCTGCACAAAAGATCT

CN 119205 ------------------------------------------------------------

DH55 ref genome NNNNNNNNNNNNNNNNNNNNNNNNNNNNNNNNNNNNNNNNNNNNNNNNNNNNNNNNNNNN 09-CS0040 TGTGGGACACCTTTCTTTA----------------CCTCATTAGTCCGCTTATTAGTCTT

CN 113754 TGTGGGACACCTTTCTTTA----------------CCTCATTAGTCCGCTTATTAGTCTT

CO46 NCBI TGTGGGACACCTTTCTTTA----------------CCTCATTAGTCCGCTTATTAGTCTT

Jasper TGTGGGACACCTTTCTTTA----------------CCTCATTAGTCCGCTTATTAGTCTT

Joelle phyto TCCCTCCAACCTTCTTTTTAGTTCCACCCATTTTAATTCACCTGCGTAAACAGAAAATTA

Joelle NCBI TCCCTCCAACCTTCTTTTTAGTTCCACCCATTTTAATTCACCTGCGTAAACAGAAAATTA

CN 119243 ------------------------------------------------------------

CN 120025 ------------------------------------------------------------

Joelle AAFC ------------------------------------------------------------

CAM 241 TTCATATGAAAGA--AGCTGTTGGACATATAAT-CGCATGGCCAACTGATAAGTGTAGAC

17CS1133 TTCATATGAAAGA--AGCTGTTGGACATATAAT-CGCATGGCCAACTGATAAGTGTAGAC

CAM 236 TTCATATGAAAGA--AGCTGTTGGACATATAAT-CGCATGGCCAACTGATAAGTGTAGAC

Blaine Creek TTCATATGAAAGA--AGCTGTTGGACATATAAT-CGCATGGCCAACTGATAAGTGTAGAC

CN 119300 TTCATATGAAAGA--AGCTGTTGGACATATAAT-CGCATGGCCAACTGATAAGTGTAGAC

Yellowstone TTCATATGAAAGA--AGCTGTTGGACATATAAT-CGCATGGCCAACTGATAAGTGTAGAC

Hoga TTCATATGAAAGA--AGCTGTTGGACATATAAT-CGCATGGCCAACTGATAAGTGTAGAC

CN 120027 TTCATATGAAAGA--AGCTGTTGGACATATAAT-CGCATGGCCAACTGATAAGTGTAGAT

CN 120030 TTCATATGAAAGA--AGCTGTTGGACATATAAT-CGCATGGCCAACTGATAAGTGTAGAC

CN 119294 TTCATATGAAAGA--AGCTGTTGGACATATAAT-CGCATGGCCAACTGATAAGTGTAGAC

CN 120013 TTCATATGAAAGA--AGCTGTTGGACATATAAT-CGCATGGCCAACTGATAAGTGTAGAC

CN 120017 TTCATATGAAAGA--AGCTGTTGGACATATAAT-CGCATGGCCAACTGATAAGTGTAGAC

CN 119205 ------------------------------------------------------------

DH55 ref genome NNNNNNNNNNNNNNNNNNNNNNNNNNNNNNNNNNNNNNNNNNNNNNNNNNNNNNNNNNNN

09-CS0040 CCACCGTGAAGCATTACATTTGGGACATTTATCAAGCTTCTTTAACTTCTTTCTGAAGAG

CN 113754 CCACCGTGAAGCATTACATTTGGGACATTTATCAAGCTTCTTTAACTTCTTT-CGAAGAG

CO46 NCBI CCACCGTGAAGCATTACATTTGGGACATTTATCAAGCTTCTTTAACTTCTTTCTGAAGAG

Jasper CCACCGTGAAGCATTACATTTGGGACATTTATCAAGCTTCTTTAACTTCTTTCCGAAGAG

Joelle phyto ATCACTTAGAACA----------GAAACTTAAT-CACGTAGAACTTATATAATAGAATAC

Joelle NCBI ATCACTTAGAA----------CAGAAACTTAAT-CACGTAGAACTTATATAATAGAATAC

CN 119243 ------------------------------------------------------------

CN 120025 ------------------------------------------------------------

Joelle AAFC ------------------------------------------------------------

CAM 241 -TGGTAGA--CAACAACA---------------------TCCAAATGGAAGACATTGCCC

17CS1133 -TGGTAGA--CAACAACA---------------------TCCAAATGGAAGACATTGCCC

CAM 236 -TGGTAGA--CAACAACA---------------------TCCAAATGGAAGACATTGCCC

Blaine Creek -TGGTAGA--CAACAACA---------------------TCCAAATGGAAGACATTGCCC

CN 119300 -TGGTAGA--CAACAACA---------------------TCCAAATGGAAGACATTGCCC

Yellowstone -TGGTAGA--CAACAACA---------------------TCCAAATGGAAGACATTGCCC

Hoga -TGGTAGA--CAACAACA---------------------TCCAAATGGAAGACATTGCCC

CN 120027 -CGGTAGA--CAACAACA---------------------TCCAAATGGAAGACATTGCCC

CN 120030 -TGGTAGA--CAACAACA---------------------TCCAAATGGAAGACATTGCCC

CN 119294 -TGGTAGA--CAACAACA---------------------TCCAAATGGAAGACATTGCCC

CN 120013 -TGGTAGA--CAACAACA---------------------TCCAAATGGAAGACATTGCCC

CN 120017 -TGGTAGA--CAACAACA---------------------TCCAAATGGAAGACATTGCCC

CN 119205 ------------------------------------------------------------

DH55 ref genome NNNNNNNNNNNNNNNNNNNNNNNNNNNNNNNNNNNNNNNNNNNNNNNNNNNNNNNNNNNN

09-CS0040 GCAGCAAT--CATTAACACATGCGTCGATCTTTTCATATCCCATATGAAAGCTCTT----

CN 113754 GCAGCAAT--CATTAACACATGCGTCGATCTTTTCATATCCCATATGAAAGCTCTT----

CO46 NCBI GCAGCAAT--CATTAACACATGCGTCGATCTTTTCATATCCCATATGAAAGCTCTT----

Jasper GCAGCAAT--CATTAACACATGCGTCGATCTTTTCATATCCCATATGAAAGCTCTT----

Joelle phyto ATCATAAAATCTACAACA---------------------TTCAAC----AAACATCAACC

Joelle NCBI ATCATAAAATCTACAACA---------------------TTCAAC----AAACATCAACC

CN 119243 ------------------------------------------------------------

CN 120025 ------------------------------------------------------------

Joelle AAFC ------------------------------------------------------------

CAM 241 CTATGGTATTTATAATTTCTAGCAATG-TGTTAGCAATGTATTACCC-CTGTCTATAATG

17CS1133 CTATGGTATTTATAATTTCTAGCAATG-TGTTAGCAATGTATTACCC-CTGTCTATAATG

CAM 236 CTATGGTATTTATAATTTCTAGCAATG-TGTTAGCAATGTATTACCC-CTGTCTATAATG

Blaine Creek CTATGGTATTTATAATTTCTAGCAATG-TGTTAGCAATGTATTACCCCCTGTCTATAATG

CN 119300 CTATGGTATTTATAATTTCTAGCAATG-TGTTAGCAATGTATTACCC-CTGTCTATAATG

Yellowstone CTATGGTATTTATAATTTCTAGCAATG-TGTTAGCAATGTATTACCC-CTGTCTATAATG

Hoga CTATGGTATTTATAATTTCTAGCAATG-TGTTAGCAATGTATTACCC-CTGTCTATAATG

CN 120027 CTATGGTATTTATAATTTCTAGCAATG-TGTTAGCAATGTATTACCC-CTGTCTATAATG

CN 120030 CTATGGTATTTATAATTTCTAGCAATG-TGTTAGCAATGTATTACCC-CTGTCTATAATG

CN 119294 CTATGGTATTTATAATTTCTAGCAATG-TGTTAGCAATGTATTACCC-CTGTCTATAATG

CN 120013 CTATGGTATTTATAATTTCTAGCAATG-TGTTAGCAATGTATTACCCACTGTCTATAATG

CN 120017 CTATGGTATTTATAATTTCTAGCAATG-TGTTAGCAATGTATTACCC-CTGTCTATAATG

CN 119205 ------------------------------------------------------------

DH55 ref genome NNNNNNNNNNNNNNNNNNNNNNNNNNNNNNNNNNNNNNNNNNNNNNNNNNNNNNNNNNNN 09-CS0040 -TAAAAATTTCTTGACATCATACAATGATGTGTGAAAGACATTACCATCTGGCAAC-ATG

CN 113754 -TAAAAATTTCTTGACATCATACAATGATGTGTGAAAGACATTACCATCCGGCAAC-ATG

CO46 NCBI -TAAAAATTTCTTGACATCATACAATGATGTGTGAAAGACATTACCATCTGGCAAC-ATG

Jasper -TAAAAATTTCTTGACATCATACAATGATGTGTGAAAGACATTACCATCTGGCAAC-ATG

Joelle phyto ATAAACATTTCTTGACATCATACAATGATGTGTGAAAGACATTACCATCTGGCAAC-ATG

Joelle NCBI ATAAACATTTCTTGACATCATACAATGATGTGTGAAAGACATTACCATCTGGCAAC-ATG

CN 119243 ------------------------------------------------------------

CN 120025 ------------------------------------------------------------

Joelle AAFC ------------------------------------------------------------

CAM 241 CTAATTAAT-----------TATATTGGATTTGTTTACTGATTGTGTTTAACAGGGTATA

17CS1133 CTAATTAAT-----------TATATTGGATTTGTTTACTGATTGTGTTTAACAGGGTATA

CAM 236 CTAATTAAT-----------TATATTGGATTTGTTTACTGATTGTGTTTAACAGGGTATA

Blaine Creek CTAATTAAT-----------TATATTGGATTTGTTTACCGATTGTGTTTAACAGGGTATA

CN 119300 CTAATTAAT-----------TATATTGGATTTGTTTACTGATTGTGTTTAACAGGGTATA

Yellowstone CTAATTAAT-----------TATATTGGATTTGTTTACTGATTGTGTTTAACAGGGTATA

Hoga CTAATTAAT-----------TATATTGGATTTGTTTACTGATTGTGTTTAACAGGGTATA

CN 120027 CTAATTAAT-----------TATATTGGATTTGTTTACTGATTGTGTTTAACAGGGTATA

CN 120030 CTAATTAAT-----------TATATTGGATTTGTTTACTGATTGTGTTTAACAGGGTATA

CN 119294 CTAATTAAT-----------TATATTGGATTTGTTTACTGATTGTGTTTAACAGGGTATA

CN 120013 CTAATTAAT-----------TATATTGGATTTGTTTACTGATTGTGTTTAACAGGGTATA

CN 120017 CTAATTAAT-----------TATATTGGATTTGTTTACTGATTGTGTTTAACAGGGTATA

CN 119205 ------------------------------------------------------------

DH55 ref genome NNNNNNNNNNNNNNNNNNNNNNNNNNNNNNNNNNNNNNNNNNNNNNNNNNNNNNNNNNNN

09-CS0040 CTTGGCAATGTCTGAAGCAGTTCATTGAAGCTCTTATCCGACCAGCCATTATGTGTCTTA

CN 113754 CTTGGCAATGTCCGAAGCAGTTCATTGAAGCTCTTATCCGACCAGCCATTATGTGTCTTA

CO46 NCBI CTTGGCAATGTCTGAAGCAGTTCATTGAAGCTCTTATCCGACCAGCCATTATGTGTCTTA

Jasper CTTGGCAATGTCTGAAGCAGTTCATTGAAGCTCTTATCCGACCAGCCATTATGTGTCTTA

Joelle phyto CTTGGCAATGTCTGAAGCAGTTCATTGAAGCTCTTATCCGACCAGCCATTATGTGTCTTA

Joelle NCBI CTTGGCAATGTCTGAAGCAGTTCATTGAAGCTCTTATCCGACCAGCCATTATGTGTCTTA

CN 119243 ------------------------------------------------------------

CN 120025 ------------------------------------------------------------

Joelle AAFC ------------------------------------------------------------

CAM 241 CAAGGAAACAAATGTAAACTATTGGATTTGTCTAAGGGTGACGTCATTGTTGCTTG----

17CS1133 CAAGGAAACAAATGTAAACTATTGGATTTGTCTAAGGGTGACGTCATTGTTGCTGA----

CAM 236 CAAGGAAACAAATGTAAACTATTGGATTTGTCTAAGGGTGACGTCATTGTTGCTGA----

Blaine Creek CAAGGAAACAAATGTAAACTATTGGATTTGTCTAAGGGTGACGTCATTGTTGCTGA----

CN 119300 CAAGGAAACAAATGTAAACTATTGGATTTGTCTAAGGGTGACGTCATTGTTGCTGA----

Yellowstone CAAGGAAACAAATGTAAACTATTGGATTTGTCTAAGGGTGACGTCATTGTTGCTGA----

Hoga CAAGGAAACAAATGTAAACTATTGGATTTGTCTAAGGGTGACGTCATTGTTGCTGA----

CN 120027 CAAGGAAACAAATGTAAACTATTGGATTTGTCTAAGGGTGACGTCATTGTTGCTGA----

CN 120030 CAAGGAAACAAATGTAAACTATTGGATTTGTCTAAGGGTGACGTCATTGTTGCTGA----

CN 119294 CAAGGAAACAAATGTAAACTATTGGATTTGTCTAAGGGTGACGTCATTGTTGCTGA----

CN 120013 CAAGGAAACAAATGTAAACTATTGGATTTGTCTAAGGGTGACGTCATTGTTGCTTG----

CN 120017 CAAGGAAACAAATGTAAACTATTGGATTTGTCTAAGGGTGACGTCATTGTTGCTGA----

CN 119205 ------------------------------------------------------------

DH55 ref genome NNNNNNNNNNNNNNNNNNNNNNNNNNNNNNNNNNNNNNNNNNNNNNNNNNNNNNNNNNNN 09-CS0040 ATCCTAAACAAAGTCACAATAGCCGAT-------------AACTTGCTGTGGTTTGAACA

CN 113754 ATCCTAAACAAAGTCACAATAGCCGAT-------------AACTTGCTGTGGTTTGAACA

CO46 NCBI ATCCTAAACAAAGTCACAATAGCCGAT-------------AACTTGCTGTGGTTTGAACA

Jasper ATCCTAAACAAAGTCACAATAGCCGAT-------------AACTTGCTGTGGTTTGAACA

Joelle phyto ATCCTAAACAAAGTCACAATAGCCGAT-------------AACTTGCTGTGGTTTGAACA

Joelle NCBI ATCCTAAACAAAGTCACAATAGCCGAT-------------AACTTGCTGTGGTTTGAACA

CN 119243 ------------------------------------------------------------

CN 120025 ------------------------------------------------------------

Joelle AAFC ------------------------------------------------------------

CAM 241 --AGGGGCGTTGGGAGACACAAGAACCAAGTGCGTTGGTTAACGGAATTCCTC------T

17CS1133 ---GGGGCGTTGGGAGACACAAGAACCAAGTGCGTTGGTTAACGGAATTCCTC------T

CAM 236 ---GGGGCGTTGGGAGACACAAGAACCAAGTGCGTTGGTTAACGGAATTCCTC------T

Blaine Creek ----GGGCGTTGGGAGACACAAGAACCAAGTGCGTTGGTTAACGGAATTCCTC------T

CN 119300 ---GGGGCGTTGGGAGACACAAGAACCAAGTGCGTTGGTTAACGGAATTCCTC------T

Yellowstone ----GGGCGTTGGGAGACACAAGAACCAAGTGCGTTGGTTAACGGAATTCCTC------T

Hoga ---GGGGCGTTGGGAGACACAAGAACCAAGTGCGTTGGTTAACGGAATTCCTC------T

CN 120027 ---GGGGCGTTGGGAGACACAAGAACCAAGTGCGTTGGTTAACGGAATTCCTC------T

CN 120030 ---GGGGCGTTGGGAGACACAAGAACCAAGTGCGTTGGTTAACGGAATTCCTC------T

CN 119294 ---GGGGCGTTGGGAGACACAAGAACCAAGTGCGTTGGTTAACGGAATTCCTC------T

CN 120013 ---AGGGCGTTGGGAGACACAAGAACCAAGTGCGTTGGTTAACGGAATTCCTC------T

CN 120017 ----GGGCGTTGGGAGACACAAGAACCAAGTGCGTTGGTTAACGGAATTCCTC------T

CN 119205 ------------------------------------------------------------

DH55 ref genome NNNNNNNNNNNNNNNNNNNNNNNNNNNNNNNNNNNNNNNNNNNNNNNNNNNNNNNNNNNN 09-CS0040 GCTAGGGTATAGTGGGGTTTCAGCATCCCGGATCTTTGC--AAGGAACTCATCCTCTTGT

CN 113754 GCTAGGGTATAGTGGGGTTTCAGCATCCCGGATCTTTGC--AAGGAACTCATCCTCTTGT

CO46 NCBI GCTAGGGTATAGTGGGGTTTCAGCATCCCGGATCTTTGC--AAGGAACTCATCCTCTTGT

Jasper GCTAGGGTATAGTGGGGTTTCAGCATCCCGGATCTTTGC--AAGGAACTCATCCTCTTGT

Joelle phyto GCTAGGGTATAGTGGGGTTTCAGCATCCCGGATCTTTGC--AAGGAACTCATCCTCTTGT

Joelle NCBI GCTAGGGTATAGTGGGGTTTCAGCATCCCGGATCTTTGC--AAGGAACTCATCCTCTTGT

CN 119243 ------------------------------------------------------------

CN 120025 ------------------------------------------------------------

Joelle AAFC ------------------------------------------------------------

CAM 241 TGGACCAAAAGCAGTTAAAGTATTTGTGGATTCAGTAAAGCAGCCTCGATACATCACTAT

17CS1133 TGGACCAAAAGCAGTTAAAGTATTTGTGGATTCAGTAAAGCAGCCT-GATACATCACTAT

CAM 236 TGGACCAAAAGCAGTTAAAGTATTTGTGGATTCAGTAAAGCAGCCT-GATACATCACTAT

Blaine Creek TGGACCAAAAGCAGTTAAAGTATTTGTGGATTCAGTAAAGCAGCCTCGATACATCACTAT

CN 119300 TGGACCAAAAGCAGTTAAAGTATTTGTGGATTCAGTAAAGCAGCCT-GATACATCACTAT

Yellowstone TGGACCAAAAGCAGTTAAAGTATTTGTGGATTCAGTAAAGCAGCCT-GATACATCACTAT

Hoga TGGACCAAAAGCAGTTAAAGTATTTGTGGATTCAGTAAAGCAGCTC-GATACATCACTAT

CN 120027 TGGACCAAAAGCAGTTAAAGTATTTGTGGATTCAGTAAAGCAGCCT-GATACATCACTAT

CN 120030 TGGACCAAAAGCAGTTAAAGTATTTGTGGATTCAGTAAAGCAGCCT-GATACATCACTAT

CN 119294 TGGACCAAAAGCAGTTAAAGTATTTGTGGATTCAGTAAAGCAGCCT-GATACATCACTAT

CN 120013 TGGACCAAAAGCAGTTAAAGTATTTGTGGATTCAGTAAAGCAGCCT-GATACATCACTAT

CN 120017 TGGACCAAAAGCAGTTAAAGTATTTGTGGATTCAGTAAAGCAGCCT-GATACATCACTAT

CN 119205 ------------------------------------------------------------

DH55 ref genome NNNNNNNNNNNNNNNNNNNNNNNNNNNNNNNNNNNNNNNNNNNNNNNNNNNNNNNNNNNN

09-CS0040 TGGTCCTCACCC----------TCTGCAATCTCACTTAAGTCGCCC--ACAGGTTGGTCA

CN 113754 TGGTCCTCACCC----------TCTGCAATCTCACTTAAGTCGCCC--ACAGGTTGGTCA

CO46 NCBI TGGTCCTCACCC----------TCTGCAATCTCACTTAAGTCGCCC--ACAGGTTGGTCA

Jasper TGGTCCTCACCC----------TCTGCAATCTCACTTAAGTCGCCC--ACAGGTTGGTCA

Joelle phyto TGGTCCTCACCC----------TCTGCAATCTCACTTAAGTCGCCC--ACAGGTTGGTCA

Joelle NCBI TGGTCCTCACCC----------TCTGCAATCTCACTTAAGTCGCCC--ACAGGTTGGTCA

CN 119243 ------------------------------------------------------------

CN 120025 ------------------------------------------------------------

Joelle AAFC ------------------------------------------------------------

CAM 241 GGAGACATACAGCTGAAATGTCATTTCTTGA--GGACTGTTTGATGGC-TTACGTATCTT

17CS1133 GGAGACATACAGCTGAAATGTCATTTCTTGA--GGACTGTTTGATGGC-TTACGTATCTT

CAM 236 GGAGACATACAGCTGAAATGTCATTTCTTGA--GGACTGTTTGATGGC-TTACGTATCTT

Blaine Creek GGAGACATACAGCTGAAATGTCATTTCTTGA--GGACTGTTTGATGGC-TTACGTATCTT

CN 119300 GGAGACATACAGCTGAAATGTCATTTCTTGA--GGACTGTTTGATGGC-TTACGTATCTT

Yellowstone GGAGACATACAGCTGAAATGTCATTTCTTGA--GGACTGTTTGATGGC-TTACGTATCTT

Hoga GGAGACATACAGCTGAAATGTCATTTCTTGA--GGACTGTTTGATGGC-TTACGTATCTT

CN 120027 GGAGACATACAGCTGAAATGTCATTTCTTGA--GGACTGTTTGATGGC-TTACGTATCTT

CN 120030 GGAGACATACAGCTGAAATGTCATTTCTTGA--GGACTGTTTGATGGC-TTACGTATCTT

CN 119294 GGAGACCTACAGCTGAAATGTCATTTCTTGA--GGACTGTTTGATGGC-TTACGTATCTT

CN 120013 GGAGACATACAGCTGAAATGTCATTTCTTGA--GGACTGTTTGATGGC-TTACGTATCTT

CN 120017 GGAGACCTACAGCTGAAATGTCATTTCTTGA--GGACTGTTTGATGGC-TTACGTATCTT

CN 119205 ------------------------------------------------------------

DH55 ref genome NNNNNNNNNNNNNNNNNNNNNNNNNNNNNNNNNNNNNNNNNNNNNNNNNNNNNNNNNNNN

09-CS0040 GCTAAGTCACCTCTAAAA-GCCAACTCTTGATCAAAAAATTCAGCAGCTTTATATAACTC

CN 113754 GCTAAGTCACCTCTAAAA-GCCAACTCTTGATCAAAAAATTCAGCAGCTTTATATAACTC

CO46 NCBI GCTAAGTCACCTCTAAAA-GCCAACTCTTGATCAAAAAATTCAGCAGCTTTATATAACTC

Jasper GCTAAGTCACCTCTAAAA-GCCAACTCTTGATCAAAAAATTCAGCAGCTTTATATAACTC

Joelle phyto GCTAAGTCACCTCTAAAA-GCCAACTCTTGATCAAAAAATTCAGCAGCTTTATATAACTC

Joelle NCBI GCTAAGTCACCTCTAAAA-GCCAACTCTTGATCAAAAAATTCAGCAGCTTTATATAACTC

CN 119243 ------------------------------------------------------------

CN 120025 ------------------------------------------------------------

Joelle AAFC ------------------------------------------------------------

CAM 241 GG-------------CCCCTGAGTAAGGTTGATTTTGAAA---ATCCCTCAACTCCAACT

17CS1133 GG-------------CCCCTGAGTAAGGTTGATTTTGAAA---ATCCCTCAACTCCAACT

CAM 236 GG-------------CCCCTGAGTAAGGTTGATTTTGAAA---ATCCCTCAACTCCAACT

Blaine Creek GG-------------CCCC-GAGTAAGGTTGATTTTGAAA---ATCCCTCAACTCCAACT

CN 119300 GG-------------CCCCCGAGTAAGGTTGATTTTGAAA---ATCCCTCAACTCCAACT

Yellowstone GG-------------CCCCTGAGTAAGGTTGATTTTGAAA---ATCCCTCAACTCCAACT

Hoga GG-------------CCCCTGAGTAAGGTTGATTTTGAAA---ATCCCTCAACTCCAACT

CN 120027 GG-------------CCCCTGAGTAAGGTTGATTTTGAAA---ATCCCTCAACTCCAACT

CN 120030 GG-------------CCCTCGAGTAAGGTTGATTTTGAAA---ATCCCTCAACTCCAACT

CN 119294 GG-------------CCCCTGAGTAAGGTTGATTTTGAAA---ATCCCTCAACTCCAACT

CN 120013 GG-------------CCCTCGAGTAAGGTTGATTTTGAAA---ATCCCTCAACTCCAACT

CN 120017 GG-------------CCCCTGAGTAAGGTTGATTTTGAAA---ATCCCTCAACTCCAACT

CN 119205 ------------------------------------------------------------

DH55 ref genome NNNNNNNNNNNNNNNNNNNNNNNNNNNNNNNNNNNNNNNNNNNNNNNNNNNNNNNNNNNN

09-CS0040 AAAAACTTCCTCATTCCACTGAGT-TGGTTTACTTTGAAATTCATCTACTGACTTCACAT

CN 113754 AAAAACTTCCTCATTCCACTGAGT-TGGTTTACTTTGAAATTCATCTACTGACTTCACAT

CO46 NCBI AAAAACTTCCTCATTCCACTGAGT-TGGTTTACTTTGAAATTCATCTACTGACTTCACAT

Jasper AAAAACTTCCTCATTCCACTGAGT-TGGTTTACTTTGAAATTCATCTACTGACTTCACAT

Joelle phyto AAAAACTTCCTCATTCCACTGAGT-TGGTTTACTTTGAAATTCATCTACTGACTTCACAT

Joelle NCBI AAAAACTTCCTCATTCCACTGAGT-TGGTTTACTTTGAAATTCATCTACTGACTTCACAT

CN 119243 ------------------------------------------------------------

CN 120025 ------------------------------------------------------------

Joelle AAFC ------------------------------------------------------------

CAM 241 GGTCAGAATGCTAC--ATCACATGCTTCTTTGTCTGCATCAAAGGCCAAGTCTGCAGCCA

17CS1133 GGTCAGAATGCTAC--ATCACATGCTTCTTTGTCTGCATCAAAGGCCAAGTCTGCAGCCA

CAM 236 GGTCAGAATGCTAC--ATCACATGCTTCTTTGTCTGCATCAAAGGCCAAGTCTGCAGCCA

Blaine Creek GGTCAGAATGCTAC--ATCACATGCTTCTTTGTCTGCATCAAAGGCCAAGTCCGCAGCCA

CN 119300 GGTCAGAATGCTAC--ATCACATGCTTCTTTGTCTGCATCAAAGGCCAAGTCTGCAGCCA

Yellowstone GGTCAGAATGCTAC--ATCACATGCTTCTTTGTCTGCATCAAAGGCCAAGTCTGCAGCCA

Hoga GGTCAGAATGCTAC--ATCACATGCTTCTTTGTCTGCATCAAAGGCCAAGTCTGCAGCCA

CN 120027 GGTCAGAATGCTAC--ATCACATGCTTCTTTGTCTGCATCAAAGGCCAAGTCTGCAGCCA

CN 120030 GGTCAGAATGCTAC--ATCACATGCTTCTTTGTCTGCATCAAAGGCCAAGTCTGCAGCCA

CN 119294 GGTCAGAATGCTAC--ATCACATGCTTCTTTGTCTGCATCAAAGGCCAAGTCTGCAGCCA

CN 120013 GGTCAGAATGCTAC--ATCACATGCTTCTTTGTCCGCATCAAAGGCCAAGTCTGCAGCCA

CN 120017 GGTCAGAATGCTAC--ATCACATGCTTCTTTGTCTGCATCAAAGGCCAAGTCTGCAGCCA

CN 119205 ------------------------------------------------------------

DH55 ref genome NNNNNNNNNNNNNNNNNNNNNNNNNNNNNNNNNNNNNNNNNNNNNNNNNNNNNNNNNNNN

09-CS0040 CTCCATGATGATACCAATCACTATGCACCTTGTATGCCTCA-------------------

CN 113754 CTCCATGATGATACCAATCACTATGCACCTTGTATGCCTCA-------------------

CO46 NCBI CTCCATGATGATACCAATCACTATGCACCTTGTATGCCTCA-------------------

Jasper CTCCATGATGATACCAATCACTATGCACCTTGTATGCCTCA-------------------

Joelle phyto CTCCATGATGATACCAATCACTATGCACCTTGTATGCCTCA-------------------

Joelle NCBI CTCCATGATGATACCAATCACTATGCACCTTGTATGCCTCA-------------------

CN 119243 ------------------------------------------------------------

CN 120025 ------------------------------------------------------------

Joelle AAFC ------------------------------------------------------------

CAM 241 CGG-CTTCTAAGTCTCGCTACAGAGTCCAAAACTTCAGCAACAGCTTCAAAG----GCCG

17CS1133 CAG-CTTCTAAGTCT-GCTACAGAGTCCAAAACTTCAGCAACAGCTTCAAAG----GCTG

CAM 236 CGAGCTTCTAAGTCC-GCTACAGAGTCCAAAACTTCAGCAACAGCTTCAAAG----GCTG

Blaine Creek CGG-CTTCTAAGTCTCGCTAC-GAGTCCAAAACTTCAGCAACAGCTTCAAAG----GCTG

CN 119300 CAG-CTTCTAAGTCT-GCTACAGAGTCCAAAACTTCAGCAACAGCTTCAAAG----GCTG

Yellowstone CAG-CTTCTAAGTCT-GCTAGAGAGTCCAAAACTTCAGCAACAGCTTCAAAG----GCTG

Hoga CAG-CTTCTAAGTCT-GCTACAGAGTCCAAAACTTCAGCAACAGCTTCAAAG----GCTG

CN 120027 CAG-CTTCTAAGTCT-GCTACAGAGTCCAAAACTTCAGCAACAGCTTCAAAG----GCTG

CN 120030 CAGCCTTCTAAGTCT-GCTACTGAGTCCAAAACTTCAACAACAGCTTCAAAG----GCCG

CN 119294 CAG-CTTCTAAGTCT-GCTACAGAGTCCAAAACTTCAGCAACAGCTTCAAAG----GCTG

CN 120013 CAG-CTTCTAAGTCT-GCTACAGAGTCCAAAACTTCAGCAACAGCTTCAAAG----GCTG

CN 120017 CAG-CTTCTAAGTCT-GCTAC-GAGTCCAAAACTTCAGCAACAGCTTCAAAG----GCTG

CN 119205 ------------------------------------------------------------

DH55 ref genome NNNNNNNNNNNNNNNNNNNNNNNNNNNNNNNNNNNNNNNNNNNNNNNNNNNNNNNNNNNN

09-CS0040 ----TCCATCCCTCTTATTACAAGATGCTCAACCACAACA-CTGTTTAACTGTCGTACTA

CN 113754 ----TCCATCCCTCTTATTACAAGATGCTCAACCACAACATCTGTTTAACCGTCGTACTA

CO46 NCBI ----TCCATCCCTCTTATTACAAGATGCTCAACCACAACA-CTGTTTAACTGTCGTACTA

Jasper ----TCCATCCCTCTTATTACAAGATGCTCAACCACAACA-CTGTTTAACTGTCGTACTA

Joelle phyto ----TCCATCCCTCTTATTACAAGATGCTCAACCACAACA-CTGTTTAACTGTCGTACTA

Joelle NCBI ----TCCATCCCTCTTATTACAAGATGCTCAACCACAACA-CTGTTTAACTGTCGTACTA

CN 119243 ------------------------------------------------------------

CN 120025 ------------------------------------------------------------

Joelle AAFC ------------------------------------------------------------

CAM 241 CATCGC-------------AGGCCAAATCTGCA--TCTATGTCTTCTAAGTCCGC-----

17CS1133 CATCGC-------------AGGCCAAATCTGCA--TCTATGTCTTCTAAGTCTGC-----

CAM 236 CATCGC-------------AGGCCAAATCTGCA--TCTATGTCTTCTAAGTCTGC-----

Blaine Creek CATCGC-------------AGGCCAAATCTGCA--TCTATGTCTTCTAAGTCCGC-----

CN 119300 CATCGC-------------AGGCCAAATCTGCA--TCTATGTCTTCTAAGTCTGC-----

Yellowstone CATCGC-------------AGGCCAAATCTGCA--TCTATGTCTTCTAAGTCTGC-----

Hoga CATCGC-------------AGGCCAAATCTGCA--TCTATGTCTTCTAAGTCTGC-----

CN 120027 CATCGC-------------AGGCCAAATCTGCA--TCTATGTCTTCTAAGTCTGC-----

CN 120030 CATCGC-------------GAGCCAAATCTGCA--TCTATGTCTTCTAAGTCTGC-----

CN 119294 CATCGC-------------AGGCCAAATCTGCA--TCTATGTCTTCTAAGTCTGC-----

CN 120013 CATCGC-------------GAGCCAAATCTGCA--TCTATGTCTTCTAAGTCCGC-----

CN 120017 CATCGC-------------AGGCCAAATCTGCA--TCTATGTCTTCTAAGTCTGC-----

CN 119205 ------------------------------------------------------------

DH55 ref genome NNNNNNNNNNNNNNNNNNNNNNNNNNNNNNNNNNNNNNNNNNNNNNNNNNNNNNNNNNNN 09-CS0040 CATTACGACAGTCTTTACAAGGACATATTATCATTTCTATGTCACCTAAAGCCGC-----

CN 113754 CATTACGACAGTCTTTACAAGGACATATTATCATTTCTATGTCACCTAAAGCCGC-----

CO46 NCBI CATTACGACAGTCTTTACAAGGACATATTATCATTTCTATGTCACCTAAAGCCGC-----

Jasper CATTACGACAGTCTTTACAAGGACATATTATCATTTCTATGTCACCTAAAGCCGC-----

Joelle phyto CATTACGACAGTCTTTACAAGGACATATTATCATTTCTATGTCACCTAAAGCCGC-----

Joelle NCBI CATTACGACAGTCTTTACAAGGACATATTATCATTTCTATGTCACCTAAAGCCGC-----

CN 119243 ------------------------------------------------------------

CN 120025 ------------------------------------------------------------

Joelle AAFC ------------------------------------------------------------

CAM 241 -----------------------------------------------------AT-----

17CS1133 -----------------------------------------------------AT-----

CAM 236 -----------------------------------------------------AT-----

Blaine Creek -----------------------------------------------------AT-----

CN 119300 -----------------------------------------------------AT-----

Yellowstone -----------------------------------------------------AT-----

Hoga -----------------------------------------------------AT-----

CN 120027 -----------------------------------------------------AT-----

CN 120030 -----------------------------------------------------AT-----

CN 119294 -----------------------------------------------------AT-----

CN 120013 -----------------------------------------------------AT-----

CN 120017 -----------------------------------------------------AT-----

CN 119205 ------------------------------------------------------------

DH55 ref genome NNNNNNNNNNNNNNNNNNNNNNNNNNNNNNNNNNNNNNNNNNNNNNNNNNNNNNNNNNNN

09-CS0040 -----------------------------------------------------AGACACA

CN 113754 -----------------------------------------------------AGACACA

CO46 NCBI -----------------------------------------------------AGACACA

Jasper -----------------------------------------------------AGACACA

Joelle phyto -----------------------------------------------------AGACACA

Joelle NCBI -----------------------------------------------------AGACACA

CN 119243 ------------------------------------------------------------

CN 120025 ------------------------------------------------------------

Joelle AAFC ------------------------------------------------------------

CAM 241 -------CTGGTTC-CGAGTCTCCTGTTGATGA-------------TGCAACGGGT----

17CS1133 -------CTGGTTC-CGAGTCTCCTGTTGATGA-------------TGCAACGGGT----

CAM 236 -------CTGGTTC-CGAGTCTCCTGTTGATGA-------------TGCAACGGGT----

Blaine Creek -------CTGGTTC-TGAGTCTCCTGTTGATGA-------------TGCAACGGGT----

CN 119300 -------CTGGTTC-CGAGTCTCCTGTTGATGA-------------TGCAACGGGT----

Yellowstone -------CTGGTTCTCGAGTCTCCTGTTGATGA-------------TGCAACGGGT----

Hoga -------CTGGTTC-CGAGTCTCCTGTTGATGA-------------TGCAACGGGT----

CN 120027 -------CTGGTTC-CGAGTCTCCTGTTGATGA-------------TGCAACGGGT----

CN 120030 -------CTGGTTC-TGAGTCTCCTGTTGATGA-------------TGCAACGGGT----

CN 119294 -------CTGGTTC-CGAGTCTCCTGTTGATGA-------------TGCAACGGGT----

CN 120013 -------CTGGTTC-CGAGTCTCCTGTTGATGA-------------TGCAACGGGT----

CN 120017 -------CTGGTTC-TGAGTCTCCTGTTGATGA-------------TGCAACGGGT----

CN 119205 ------------------------------------------------------------

DH55 ref genome NNNNNNNNNNNNNNNNNNNNNNNNNNNNNNNNNNNNNNNNNNNNNNNNNNNNNNNNNNNN

09-CS0040 TCCCTTACAAATTTCCAAGCTCCACTTTTATAACCAGGATCAGCTCTTCAATGGATAATT

CN 113754 TCCCTTACAAATTTCCAAGCTCCACTTTTATAACCAGGATCAGCTCTTCAATGGATAATT

CO46 NCBI TCCCTTACAAATTTCCAAGCTCCACTTTTATAACCAGGATCAGCTCTTCAATGGATAATT

Jasper TCCCTTACAAATTTCCAAGCTCCACTTTTATAACCAGGATCAGCTCTTCAATGGATAATT

Joelle phyto TCCCTTACAAATTTCCAAGCTCCACTTTTATAACCAGGATCAGCTCTTCAATGGATAATT

Joelle NCBI TCCCTTACAAATTTCCAAGCTCCACTTTTATAACCAGGATCAGCTCTTCAATGGATAATT

CN 119243 ------------------------------------------------------------

CN 120025 ------------------------------------------------------------

Joelle AAFC ------------------------------------------------------------

CAM 241 -----CCAATATCACCAAT-AAAGAATACTTTA------------CCATCAC-AGTCTCC

17CS1133 -----CCAATATCACCAAT-AAAGAATACTTTA------------CCATCAC-GGTCTCC

CAM 236 -----CCAATATCACCAAT-AAAGAATACTTTA------------CCATCAC-AGTCTCC

Blaine Creek -----CCAATATCACCAAT-AAAGAATACTTTA------------CCATCACGAGTCTCC

CN 119300 -----CCAATATCACCAAT-AAAGAATACTTTA------------CCATCAC-AGTCTCC

Yellowstone -----CCAATATCACCAAT-AAAGAATACTTTA------------CCATCAC-AGTCTCC

Hoga -----CCAATATCACCAAT-AAAGAATACTTTA------------CCATCACGAGTCTCC

CN 120027 -----CCAATATCACCAAT-AAAGAATACTTTA------------CCATCAC-AGTCTCC

CN 120030 -----CCAATATCACCAAT-AAAGAATACTTTA------------CCATCAC-AGTCTCC

CN 119294 -----CCAATATCACCAAT-AAAGAATACTTTA------------CCATCAC-AGTCTCC

CN 120013 -----CCAATATCACCAAT-AAAGAATACTTTA------------CCATCAC-AGTCTCC

CN 120017 -----CCAATATCACCAAT-AAAGAATACTTTA------------CCATCAC-AGTCTCC

CN 119205 ------------------------------------------------------------

DH55 ref genome NNNNNNNNNNNNNNNNNNNNNNNNNNNNNNNNNNNNNNNNNNNNNNNNNNNNNNNNNNNN

09-CS0040 AAAGAAAAAAATCATAACTAAAAGAACATCTTAAAGTATCCAAACCCAAAAT-AAAACCT

CN 113754 AAAGAAAAAAATCATAACTAAAAGAACATCTTAAAGTATCCAAACCCAAAAT-AAAACCT

CO46 NCBI AAAGAAAAAAATCATAACTAAAAGAACATCTTAAAGTATCCAAACCCAAAAT-AAAACCT

Jasper AAAGAAAAAAATCATAACTAAAAGAACATCTTAAAGTATCCAAACCCAAAAT-AAAACCT

Joelle phyto AAAGAAAAAAATCATAACTAAAAGAACATCTTAAAGTATCCAAACCCAAAAT-AAAACCT

Joelle NCBI AAAGAAAAAAATCATAACTAAAAGAACATCTTAAAGTATCCAAACCCAAAAT-AAAACCT

CN 119243 ------------------------------------------------------------

CN 120025 ------------------------------------------------------------

Joelle AAFC ------------------------------------------------------------

CAM 241 TCTCG-GAAGTCT----------CCGAGTAAGTGTTTTC-----------------ATAT

17CS1133 TCTCAGGAAGTCT----------CCGAGTAAGTGTTTTC-----------------ATAT

CAM 236 TCTCGGGAAGTCT----------CC-AGTAAGTGTTTTC-----------------ATAT

Blaine Creek TCTCGAGAAGTCT----------CCGAGTAAGTGTTTTC-----------------ATAT

CN 119300 TCTCGTGAAGTCT----------CC-AGTAAGTGTTTTC-----------------ATAT

Yellowstone TCTCAGGAAGTCT----------CC-AGTAAGTGTTTTC-----------------ATAT

Hoga TCTCGTGAAGTCT----------CCGAGTAAGTGTTTTC-----------------ATAT

CN 120027 TCTCAGGAAGTCT----------CC-AGTAAGTGTTTTC-----------------ATAT

CN 120030 TCTC-GGAAGTCT----------CC-AGTAAGTGTTTTC-----------------ATAT

CN 119294 TCTC-GGAAGTCT----------CC-AGTAAGTGTTTTC-----------------ATAT

CN 120013 TCTC-GGAAGTCT----------CC-AGTAAGTGTTTTC-----------------ATAT

CN 120017 TCTCGGGAAGTCT----------CCGAGTAAGTGTTTTC-----------------ATAT

CN 119205 ------------------------------------------------------------

DH55 ref genome NNNNNNNNNNNNNNNNNNNNNNNNNNNNNNNNNNNNNNNNNNNNNNNNNNNNNNNNNNNN

09-CS0040 AATTATGAAATATAATTCCTTACCTGCATAGATGCACCCATGCCTTGTCCAACATTATAT

CN 113754 AATTATGAAATATAATTCCTTACCTGCATAGATGCACCCATGCCTTGTCCAACATTATAT

CO46 NCBI AATTATGAAATATAATTCCTTACCTGCATAGATGCACCCATGCCTTGTCCAACATTATAT

Jasper AATTATGAAATATAATTCCTTACCTGCATAGATGCACCCATGCCTTGTCCAACATTATAT

Joelle phyto AATTATGAAATATAATTCCTTACCTGCATAGATGCACCCATGCCTTGTCCAACATTATAT

Joelle NCBI AATTATGAAATATAATTCCTTACCTGCATAGATGCACCCATGCCTTGTCCAACATTATAT

CN 119243 ------------------------------------------------------------

CN 120025 ------------------------------------------------------------

Joelle AAFC ------------------------------------------------------------

CAM 241 GTTATGAAGTTGTTTTTGTTAATTATATGATGACTGATGGTTTATATTGTA---------

17CS1133 GTTATGAAGTTGTTTTTGTTAATTATATGATGACTGATGGTTTATATTGTA---------

CAM 236 GTTATGAAGTTGTTTTTGTTAATTATATGATGACTGATGGTTTATATTGTA---------

Blaine Creek GTTATGAAGTTGTTTTTGTTAATTATATGATGACTGATGGTTTATATTGTA---------

CN 119300 GTTATGAAGTTGTTTTTGTTAATTATATGATGACTGATGGTTTATATTGTA---------

Yellowstone GTTATGAAGTTGTTTTTGTTAATTATATGATGACTGATGGTTTATATTGTA---------

Hoga GTTATGAAGTTGTTTTTGTTAATTATATGATGACTGATGGTTTATATTGTA---------

CN 120027 GTTATGAAGTTGTTTTTGTTAATTATATGATGACTGATGGTTTATATTGTA---------

CN 120030 GTTATGAAGTTGTTTTTGTTAATTATATGATGACTGATGGTTTATATTGTA---------

CN 119294 GTTATGAAGTTGTTTTTGTTAATTATATGATGACTGATGGTTTATATTGTA---------

CN 120013 GTTATGAAGTTGTTTTTGTTAATTATATGATGACTGATGGTTTATATTGTA---------

CN 120017 GTTATGAAGTTGTTTTTGTTAATTATATGATGACTGATGGTTTATATTGTA---------

CN 119205 ------------------------------------------------------------

DH55 ref genome NNNNNNNNNNNNNNNNNNNNNNNNNNNNNNNNNNNNNNNNNNNNNNNNNNNNNNNNNNNN

09-CS0040 GTTATAATATAAATTTTAACCTTTATAGATAAAATAGAGATACATATTATACAAAGCTCA

CN 113754 GTTATAATATAAATTTTAACCTTTATAGATAAAATAGAGATACATATTATACAAAGCTCA

CO46 NCBI GTTATAATATAAATTTTAACCTTTATAGATAAAATAGAGATACATATTATACAAAGCTCA

Jasper GTTATAATATAAATTTTAACCTTTATAGATAAAATAGAGATACATATTATACAAAGCTCA

Joelle phyto GTTATAATATAAATTTTAACCTTTATAGATAAAATAGAGATACATATTATACAAAGCTCA

Joelle NCBI GTTATAATATAAATTTTAACCTTTATAGATAAAATAGAGATACATATTATACAAAGCTCA

CN 119243 ------------------------------------------------------------

CN 120025 ------------------------------------------------------------

Joelle AAFC ------------------------------------------------------------

CAM 241 --------------------AACATGGCAGCGCATTA-----ATAAAGTTATTTCCAAGG

17CS1133 --------------------AACATGGCAGCGCATTA-----ATAAAGTTATTTCCAAGG

CAM 236 --------------------AACATGGCAGCGCATTA-----ATAAAGTTATTTCCAAGG

Blaine Creek --------------------AACATGGCAGCGCATTA-----ATAAAGTTATTTCCAAGG

CN 119300 --------------------AACATGGCAGCGCATTA-----ATAAAGTTATTTCCAAGG

Yellowstone --------------------AACATGGCAGCGCATTA-----ATAAAGTTATTTCCAAGG

Hoga --------------------AACATGGCAGCGCATTA-----ATAAAGTTATTTCCAAGG

CN 120027 --------------------AACATGGCAGCGCATTA-----ATAAAGTTATTTCCAAGG

CN 120030 --------------------AACATGGCAGCGCATTA-----ATAAAGTTATTTCCAAGG

CN 119294 --------------------AACATGGCAGCGCATTA-----ATAAAGTTATTTCCAAGG

CN 120013 --------------------AACATGGCAGCGCATTA-----ATAAAGTTATTTCCAAGG

CN 120017 --------------------AACATGGCAGCGCATTA-----ATAAAGTTATTTCCAAGG

CN 119205 ------------------------------------------------------------

DH55 ref genome NNNNNNNNNNNNNNNNNNNNNNNNNNNNNNNNNNNNNNNNNNNNNNNNNNNNNNNNNNNN

09-CS0040 CATTAAGCAACAATTCGGACAATACTGCACTACTTCAGACACATACGGATGCTCTTAAAC

CN 113754 CATTAAGCAACAATTCGGACAATACTGCACTACTTCGAACACATACGGATGCTCTTAAAC

CO46 NCBI CATTAAGCAACAATTCAGACAATACTGCACTACTTCAGACACATACAGATGCTCTTAAAC

Jasper CATTAAGCAACAATTCGGACAATACTGCACTACTTCG-ACACATACAGATGCTCTTAAAC

Joelle phyto CATTAAGCAACAATTCAGACAATACTGCACTACTTCAGACACATACAGATGCTCTTAAAC

Joelle NCBI CATTAAGCAACAATTCAGACAATACTGCACTACTTCAGACACATACAGATGCTCTTAAAC

CN 119243 ------------------------------------------------------------

CN 120025 ------------------------------------------------------------

Joelle AAFC ------------------------------------------------------------

CAM 241 AGAATAAGAA----GTGCAAGTTGATGGATTTAACTGAAAAGAATAGGGTTGTGG-CTGA

17CS1133 AGAATAAGAA----GTGCAAGTTGATGGATTTAACTGAAAAGAATAGGGTTGTGG-CTGA

CAM 236 AGAATAAGAA----GTGCAAGTTGATGGATTTAACTGAAAAGAATAGGGTTGTGGTCTGA

Blaine Creek AGAATAAGAA----GTGCAAGTTGATGGATTTAACTGAAAAGAATAGGGTTGTGG-CTGA

CN 119300 AGAATAAGAA----GTGCAAGTTGATGGATTTAACTGAAAAGAATAGGGTTGTGG-CTGA

Yellowstone AGAATAAGAA----GTGCAAGTTGATGGATTTAACTGAAAAGAATAGGGTTGTGG-CTGA

Hoga AGAATAAGAA----GTGCAAGTTGATGGATTTAACTGAAAAGAATAGGGTTGTGG-CTGA

CN 120027 AGAATAAGAA----GTGCAAGTTGATGGATTTAACTGAAAAGAATAGGGTTGTGG-CTGA

CN 120030 AGAATAAGAA----GTGCAAGTTGATGGATTTAACTGAAAAGAATAGGGTTGTGG-CTGA

CN 119294 AGAATAAGAA----GTGCAAGTTGATGGATTTAACTGAAAAGAATAGGGTTGTGG-CTGA

CN 120013 AGAATAAGAA----GTGCAAGTTGATGGATTTAACTGAAAAGAATAGGGTTGTGG-CTGA

CN 120017 AGAATAAGAA----GTGCAAGTTGATGGATTTAACTGAAAAGAATAGGGTTGTGG-CTGA

CN 119205 ------------------------------------------------------------

DH55 ref genome NNNNNNNNNNNNNNNNNNNNNNNNNNNNNNNNNNNNNNNNNNNNNNNNNNNNNNNNNNNN

09-CS0040 CAATATCTAACTGTGTAAAATCTCACTAATCTAATCACTATTTATACGAATCAGACTCAA

CN 113754 CAATATCTAACTGTGTAAAATCTCACTAATCTAATCACTATTTATACGAATCAGACTCAA

CO46 NCBI CAATATCTAACTGTGTAAAATCTCACTAATCTAATCACTATTTATACGAATCAGACTCAA

Jasper CAATATCTAACTGTGTAAAATCTCACTAATCTAATCACTATTTATACGAATCAGACTCAA

Joelle phyto CAATATCTAACTGTGTAAAATCTCACTAATCTAATCACTATTTATACGAATCAGACTCAA

Joelle NCBI CAATATCTAACTGTGTAAAATCTCACTAATCTAATCACTATTTATACGAATCAGACTCAA

CN 119243 ------------------------------------------------------------

CN 120025 ------------------------------------------------------------

Joelle AAFC ------------------------------------------------------------

CAM 241 AGGACGATGGGGAACAAATGATCCAGAACATAAGGTTCACT-TTACTCGCTTGGGTTCTA

17CS1133 AGGACGATGGGGAACAAATGATCCAGAACATAAGGTTCACT-TTACTCGCTTGGGTTCTA

CAM 236 AGGACGATGGGGAACAAATGATCCAGAACATAAGGTTCACT-TTACTCGCTTGGGTTCTA

Blaine Creek AGGACGATGGGGAACAAATGATCCAGAACATAAGGTTCACT-TTACTCGCTTGGGTTCTA

CN 119300 AGGACGATGGGGAACAAATGATCC-GAACATAAGGTTCACT-TTACTCGCTTGGGTTCTA

Yellowstone AGGACGATGGGGAACAAATGATCCAGAACATAAGGTTCACT-TTACTCGCTTGGGTTCTA

Hoga AGGACGATGGGGAACAAATGATCCAGAACATAAGGTTCACT-TTACTCGCTTGGGTTCTA

CN 120027 AGGACGATGGGGAACAAATGATCCAGAACATAAGGTTCACT-TTACTCGCTTGGGTTCTA

CN 120030 AGGACGATGGGGAACAAATGATCCGGAACATAAGGTTCACT-TTACTCGC-TGGGTTCTA

CN 119294 AGGACGATGGGGAACAAATGATCCAGAACATAAGGTTCACT-TTACTCGCTTGGGTTCTA

CN 120013 AGGACGATGGGGAACAAATGATCCAGAACATAAGGTTCACT-TTACTCGCTTGGGTTCTA

CN 120017 AGGACGATGGGGAACAAATGATCCAGAACATAAGGTTCACT-TTACTCGCTTGGGTTCTA

CN 119205 ------------------------------------------------------------

DH55 ref genome NNNNNNNNNNNNNNNNNNNNNNNNNNNNNNNNNNNNNNNNNNNNNNNNNNNNNNNNNNNN

09-CS0040 AGGACAA-------------------AACATAGCAATCACCAATATTCG--TGTGTAATA

CN 113754 AGGACAA-------------------AACATAGCAATCACCAATATTCG--TGTGTAATA

CO46 NCBI AGGACAA-------------------AACATAGCAATCACCAATATTCG--TGTGTAATA

Jasper AGGACAA-------------------AACATAGCAATCACCAATATTCG--TGTGTAATA

Joelle phyto AGGACAA-------------------AACATAGCAATCACCAATATTCG--TGTGTAATA

Joelle NCBI AGGACAA-------------------AACATAGCAATCACCAATATTCG--TGTGTAATA

CN 119243 ------------------------------------------------------------

CN 120025 ------------------------------------------------------------

Joelle AAFC ------------------------------------------------------------

CAM 241 ATGCAGTTAAAGTGTGGATAGA------------TGTTGTGAAGG-----TGAAAAATGT

17CS1133 ATGCAGTTAAAGTGTGGATAGA------------TGTTGTGAAGG-----TGAAAAATGT

CAM 236 ATGCAGTTAAAGTGTGGATAGA------------TGTTGTGAAGG-----TGAAAAATGT

Blaine Creek ATGCAGTTAAAGTGTGGATAGA------------TGTTGTGAAGG-----TGAAAAATGT

CN 119300 ATGCAGTTAAAGTGTGGATAGA------------TGTTGTGAAGG-----TGAAAAATGT

Yellowstone ATGCAGTTAAAGTGTGGATAGA------------TGTTGTGAAGG-----TGAAAAATGT

Hoga ATGCAGTTAAAGTGTGGATAGA------------TGTTGTGAAGG-----TGAAAAATGT

CN 120027 ATGCAGTTAAAGTGTGGATAGA------------TGTTGTGAAGG-----TGAAAAATGT

CN 120030 ATGCAGTTAAAGTGTGGATAGA------------TGTTGTGAAGG-----TGAAAAATGT

CN 119294 ATGCAGTTAAAGTGTGGATAGA------------TGTTGTGAAGG-----TGAAAAATGT

CN 120013 ATGCGGTTAAAGTGTGGATAGA------------TGTTGTGAAGG-----TGAAAAATGT

CN 120017 ATGCGGTTAAAGTGTGGATAGA------------TGTTGTGAAGG-----TGAAAAATGT

CN 119205 ------------------------------------------------------------

DH55 ref genome NNNNNNNNNNNNNNNNNNNNNNNNNNNNNNNNNNNNNNNNNNNNNNNNNNNNNNNNNNNN

09-CS0040 ATAATGTTTAACTATTGGTTGAGTGATACTAACCTGTTATGTGAAGTACGAGAGAAATGA

CN 113754 ATAATGTTTAACTATTGGTTGAGTGATACTAACCTGTTGTGTGAGTAC--AGAGAAATGA

CO46 NCBI ATAATGTTTAACTATTGGTTGAGTGATACTAACCTGTTGTGTGAGTAC--AGAGAAATGA

Jasper ATAATGTTTAACTATTGGTTGAGTGATACTAACCTGTTGTGTGAGTAC--AGAGAAATGA

Joelle phyto ATAATGTTTAACTATTGGTTGAGTGATACTAACCTGTTGTGTGAGTAC--AGAGAAATGA

Joelle NCBI ATAATGTTTAACTATTGGTTGAGTGATACTAACCTGTTGTGTGAGTAC--AGAGAAATGA

CN 119243 ------------------------------------------------------------

CN 120025 ------------------------------------------------------------

Joelle AAFC ------------------------------------------------------------

CAM 241 AAAAGTTTGGAGGCCATCCGATGAAATAGAGATTATTGAAGATGCACTTAGCTCCTGCAT

17CS1133 AAAAGTTTGGAGGCCATCCGATGAAATAGAGATTATTGAAGATGCACTTAGCTCCTGCAT

CAM 236 AAAAGTTTGGAGGCCATCCGATGAAATAGAGATTATTGAAGATGCACTTAGCTCCTGCAT

Blaine Creek AAAAGTTTGGAGGCCATCCGATGAAATAGAGATTATTGAAGATGCACTTAGCTCCTGCAT

CN 119300 AAAAGTTTGGAGGCCATCCGATGAAATAGAGATTATTGAAGATGCACTTAGCTCCTGCAT

Yellowstone AAAAGTTTGGAGGCCATCCGATGAAATAGAGATTATTGAAGATGCACTTAGCTCCTGCAT

Hoga AAAAGTTTGGAGGCCATCCGATGAAATAGAGATTATTGAAGATGCACTTAGCTCCTGCAT

CN 120027 AAAAGTTTGGAGGCCATCCGATGAAATAGAGATTATTGAAGATGCACTTAGCTCCTGCAT

CN 120030 AAAAGTTTGGAGGCCATCCGATGAAATAGAGATTATTGAAGATGCACTTAGCTCCTGCAT

CN 119294 AAAAGTTTGGAGGCCATCCGATGAAATAGAGATTATTGAAGATGCACTTAGCTCCTGCAT

CN 120013 AAAAGTTTGGAGGCCATCCGATGAAATAGAGATTATTGAAGATGCACTTAGCTCCTGCAT

CN 120017 AAAAGTTTGGAGGCCATCCGATGAAATAGAGATTATTGAAGATGCACTTAGCTCCTGCAT

CN 119205 ------------------------------------------------------------

DH55 ref genome NNNNNNNNNNNNNNNNNNNNNNNNNNNNNNNNNNNNNNNNNNNNNNNNNNNNNNNNNNNN

09-CS0040 GAAGCTTCAGTTCCCAACATTTCAA----------------------------TCACCAA

CN 113754 GAAGCTTCAGTTCCCCAACATTTCAAT---------------------------CACCAA

CO46 NCBI GAAGCTTCAGTTCCCCAACATTTCAAT---------------------------CACCAA

Jasper GAAGCTTCAGTTCCCCAACATTTCAAT---------------------------CACCAA

Joelle phyto GAAGCTTCAGTTCCCCAACATTTCAAT---------------------------CACCAA

Joelle NCBI GAAGCTTCAGTTCCCCAACATTTCAAT---------------------------CACCAA

CN 119243 ------------------------------------------------------------

CN 120025 ------------------------------------------------------------

Joelle AAFC ------------------------------------------------------------

CAM 241 TGCTTGGCCGGAGAACAAGGTCATTATGTCTTAAACT-GATGGTTTTATTGTGTAATGTT

17CS1133 TGCTTGGCCGGAGAACAAGGTCATTATGTCTTAAACT-GATGGTTTTATTGTGTAATGTT

CAM 236 TGCTTGGCCGGAGAACAAGGTCATTATGTCTTAAACT-GATGGTTTTATTGTGTAATGTT

Blaine Creek TGCTTGGC--GAGAACAAGGTCATTATGTCTTAAACTCGATGGTTTTATTGTGTAATGTT

CN 119300 TGCTTGGCCGTAGAACAAGGTCATTATGTCTTAAACT-GATGGTTTTATTGTGTAATGTT

Yellowstone TGCTTGGCCGGAGAACAAGGTCATTATGTCTTAAACT-GATGGTTTTATTGTGTAATGTT

Hoga TGCTTGGCCGGAGAACAAGGTCATTATGTCTTAAACTCGATGGTTTTATTGTGTAATGTT

CN 120027 TGCTTGGCCGGAGAACAAGGTCATTATGTCTTAAACT-GATGGTTTTATTGTGTAATGTT

CN 120030 TGCTTGGCCAGAGAACAAGGTCATTATGTCTTAAACT-GATGGTTTTATTGTGTAATGTT

CN 119294 TGCTTGGCCAGAGAACAAGGTCATTATGTCTTAAACTCGATGGTTTTATTGTGTAATGTT

CN 120013 TGCTTGGCCAGAGAACAAGGTCATTATGTCTTAAACT-GATGGTTTTATTGTGTAATGTT

CN 120017 TGCTTGGCCGGAGAACAAGGTCATTATGTCTTAAACT-GATGGTTTTATTGTGTAATGTT

CN 119205 ------------------------------------------------------------

DH55 ref genome NNNNNNNNNNNNNNNNNNNNNNNNNNNNNNNNNNNNNNNNNNNNNNNNNNNNNNNNNNNN

09-CS0040 CACTTGG---GAAAAAAAAATCGTTCAGT-------------------TTATGAAGGCGC

CN 113754 CACTTGGG--AAAAAAAAAATCGTTCAGT-------------------TTATGAAGGCGC

CO46 NCBI CACTTGGG-AAAAAAAAAAATCGTTCAGT-------------------TTATGAAGGCGC

Jasper CACTTGG---GAAAAAAAAATCGTTCAGT-------------------TTATGAAGGCGC

Joelle phyto CACTTGGGAAAAAAAAAAAATCGTTCAGT-------------------TTATGAAGGCGC

Joelle NCBI CACTTGGGAAAAAAAAAAAATCGTTCAGT-------------------TTATGAAGGCGC

CN 119243 ------------------------------------------------------------

CN 120025 ------------------------------------------------------------

Joelle AAFC ------------------------------------------------------------

CAM 241 AAGTACTTGAGATTGCTTGTTTATTTTGGAT----------GTTCTTGAGACTTTGATGG

17CS1133 AAGTACTTGAGATTGCTTGTTTATTTTGGAT----------GTTCTTGAGACTTTGATGG

CAM 236 AAGTACTTGAGATTGCTTGTTTATTTTGGAT----------GTTCTTGAGACTTTGATGG

Blaine Creek AAGTACTTGAGATTGCTTGTTTATTTTGGAT----------GTTCTTGAGACTTTGATGG

CN 119300 AAGTACTTGAGATTGCTTGTTTATTTTGGAT----------GTTCTTGAGACTTTGATGG

Yellowstone AAGTACTTGAGATTGCTTGTTTATTTTGGAT----------GTTCTTGAGACTTTGATGG

Hoga AAGTACTTGAGATTGCTTGTTTATTTTGGAT----------GTTCTTGAGACTTTGATGG

CN 120027 AAGTACTTGAGATTGCTTGTTTATTTTGGAT----------GTTCTTGAGACTTTGATGG

CN 120030 AAGTACTTGAGATTGCTTGTTTATTTTGGAT----------GTTCTTGAGACTTTGATGG

CN 119294 AAGTACTTGAGATTGCTTGTTTATTTTGGAT----------GTTCTTGAGACTTTGATGG

CN 120013 AAGTACTTGAGATTGCTTGTTTATTTTGGAT----------GTTCTTGAGACTTTGATGG

CN 120017 AAGTACTTGAGATTGCTTGTTTATTTTGGAT----------GTTCTTGAGACTTTGATGG

CN 119205 ------------------------------------------------------------

DH55 ref genome NNNNNNNNNNNNNNNNNNNNNNNNNNNNNNNNNNNNNNNNNNNNNNNNNNNNNNNNNNNN

09-CS0040 AAATACTCAAATCCAAAAGCTATTTTTGAGTCAAATCTGTAAATAATCGAACCCTAACA-

CN 113754 AAATACTCAAATCCAAAAGCTATTTTTGAGTCAAATCTGTAAATAATCGAACCCTAACA-

CO46 NCBI AAATACTCAAATCCAAAAGCTATTTTTGAGTCAAATCTGTAAATAATCGAACCCTAACA-

Jasper AAATACTCAAATCCAAAAGCTATTTTTGAGTCAAATCTGTAAATAATCGAACCCTAACA-

Joelle phyto AAATACTCAAATCCAAAAGCTATTTTTGAGTCAAATCTGTAAATAATCGAACCCTAACA-

Joelle NCBI AAATACTCAAATCCAAAAGCTATTTTTGAGTCAAATCTGTAAATAATCGAACCCTAACA-

CN 119243 ------------------------------------------------------------

CN 120025 ------------------------------------------------------------

Joelle AAFC ------------------------------------------------------------

CAM 241 TAAACTGATGTTCTTGAGACTTTTGTTATGTATTTTGGATCT-GAGACGTTTATTAATCA

17CS1133 TAAACTGATGTTCTTGAGACTTTTGTTATGTATTTTGGATCT-GAGACGTTTATTAATCA

CAM 236 TAAACTGATGTTCTTGAGACTTTTGTTATGTATTTTGGATCTCGAGACGTTTATTAATCA

Blaine Creek TAAACTGATGTTCTTGAGACTTTTGTTATGTATTTTGGATCC-GAGACGTTTATTAATCA

CN 119300 TAAACTGATGTTCTTGAGACTTTTGTTATGTATTTTGGATCT-GAGACGTTTATTAATCA

Yellowstone TAAACTGATGTTCTTGAGACTTTTGTTATGTATTTTGGATCT-GAGACGTTTATTAATCA

Hoga TAAACTGATGTTCTTGAGACTTTTGTTATGTATTTTGGATCT-GAGACGTTTATTAATCA

CN 120027 TAAACTGATGTTCTTGAGACTTTTGTTATGTATTTTGGATCT-GAGACGTTTATTAATCA

CN 120030 TAAACTGATGTTCTTGAGACTTTTGTTATGTATTTTGGATCTCGAGACGTTTATTAATCA

CN 119294 TAAACTGATGTTCTTGAGACTTTTGTTATGTATTTTGGATCT-GAGACGTTTATTAATCA

CN 120013 TAAACTGATGTTCTTGAGACTTTTGTTATGTATTTTGGATCT-GAGACGTTTATTAATCA

CN 120017 TAAACTGATGTTCTTGAGACTTTTGTTATGTATTTTGGATCT-GAGACGTTTATTAATCA

CN 119205 ------------------------------------------------------------

DH55 ref genome NNNNNNNNNNNNNNNNNNNNNNNNNNNNNNNNNNNNNNNNNNNNNNNNNNNNNNNNNNNN

09-CS0040 -AAACAGATGGATTCGAAACT----CCACATTCTCAAAATCA-------GTAAATAATCG

CN 113754 -AAACAGATGGATTCGAAACT----CCACATTCTCAAAATCT-------GTAAATAATCG

CO46 NCBI -AAACAGATGGATTCGAAACT----CCACATTCTCAAAATCT-------GTAAATAATCG

Jasper -AAACAGATGGATTCGAAACT----CCACATTCTCAAAATCT-------GTAAATAATCG

Joelle phyto -AAACAGATGGATTCGAAACT----CCACATTCTCAAAATCT-------GTAAATAATCG

Joelle NCBI -AAACAGATGGATTCGAAACT----CCACATTCTCAAAATCT-------GTAAATAATCG

CN 119243 ------------------------------------------------------------

CN 120025 ------------------------------------------------------------

Joelle AAFC ------------------------------------------------------------

CAM 241 GATTTTATGA-------GATTTTATAAAACAAGGTTATGTGTGCTATAATCACAT-----

17CS1133 GATTTTATGA-------GATTTTATAAAACAG-GTTATGTGTGCTATAATCACAT-----

CAM 236 GATTTTATGA-------GATTTTATAAAACAAGGTTATGTGTGCTATAATCACAT-----

Blaine Creek GATTTTATGA-------GATTTTATAAAACAAGGTTATGTGTGCTATAATCACAT-----

CN 119300 GATTTTATGA-------GATTTTATAAAACAG-GTTATGTGTGCTATAATCACAT-----

Yellowstone GATTTTATGA-------GATTTTATAAAACAG-GTTATGTGTGCTATAATCACAT-----

Hoga GATTTTATGA-------GATTTTATAAAACAAGGTTATGTGTGCTATAATCACAT-----

CN 120027 GATTTTATGA-------GATTTTATAAAACAG-GTTATGTGTGCTATAATCACAT-----

CN 120030 GATTTTATGA-------GATTTTATAAAACAGAGTTATGTGTGCTATAATCACAT-----

CN 119294 GATTTTATGA-------GATTTTATAAAACAG-GTTATGTGTGCTATAATCACAT-----

CN 120013 GATTTTATGA-------GATTTTATAAAACAG-GTTATGTGTGCTATAATCACAT-----

CN 120017 GATTTTATGA-------GATTTTATAAAACAG-GTTATGTGTGCTATAATCACAT-----

CN 119205 ------------------------------------------------------------

DH55 ref genome NNNNNNNNNNNNNNNNNNNNNNNNNNNNNNNNNNNNNNNNNNNNNNNNNNNNNNNNNNNN

09-CS0040 AACCCTAAAAAACAGATAACGAAATAAATCTAGATAAATCGAACCCTAACAAAACGAGAT

CN 113754 AACCCTAAAAAACAGATAACGAAATAAATCTAGATAAATCGAACCCTAACAAAAC-AGAT

CO46 NCBI AACCCTAAAAAACAGATAACGAAATAAATCTAGATAAATCGAACCCTAACAAAAC-AGAT

Jasper AACCCTAAAAAACAGATAACGAAATAAATCTAGATAAATCGAACCCTAACAAAAC-AGAT

Joelle phyto AACCCTAAAAAACAGATAACGAAATAAATCTAGATAAATCGAACCCTAACAAAAC-AGAT

Joelle NCBI AACCCTAAAAAACAGATAACGAAATAAATCTAGATAAATCGAACCCTAACAAAAC-AGAT

CN 119243 ------------------------------------------------------------

CN 120025 ------------------------------------------------------------

Joelle AAFC ------------------------------------------------------------

CAM 241 -----------------------ATC-GGAAAATGCTATAAAAGTACAGGTAAACGTTAT

17CS1133 -----------------------ATCGAGAAAATGCTATAAAAGTACAGGTAAACGTTAT

CAM 236 -----------------------ATC-AGAAAATGCTATAAAAGTACAGTTAAACGTTAT

Blaine Creek -----------------------ATC-GGAAAATGCTATAAAAGTACAGTTAAACGTTAT

CN 119300 -----------------------ATC-AGAAAATGCTATAAAAGTACAGGTAAACGTTAT

Yellowstone -----------------------ATC-AGAAAATGCTATAAAAGTACAGGTAAACGTTAT

Hoga -----------------------ATC-GGAAAATGCTATAAAAGTACAGGTAAACGTTAT

CN 120027 -----------------------ATC-AGAAAATGCTATAAAAGTACAGGTAAACGTTAT

CN 120030 -----------------------ATC-AGAAAATGCTATAAAAGTACA-GTAAACGTTAT

CN 119294 -----------------------ATC-AGAAAATGCTATAAAAGTACAGGTAAACGTTAT

CN 120013 -----------------------ATC-AGAAAATGCTATAAAAGTACAGGTAAACGTTAT

CN 120017 -----------------------ATC-AGAAAATGCTATAAAAGTACAGGTAAACGTTAT

CN 119205 ------------------------------------------------------------

DH55 ref genome NNNNNNNNNNNNNNNNNNNNNNNNNNNNNNNNNNNNNNNNNNNNNNNNNNNNNNNNNNNN

09-CS0040 AACTAAATCTGACCCTTATCGAAATC-AAAAGCTGTT-TGAAATCACAAATAATCGAAAC

CN 113754 AACTAAATCTGACCCTTATCGAAATC-AAAAGCTGTT-TGAAATCACAAATAATCGAAAC

CO46 NCBI AACTAAATCTGACCCTTATCGAAATC-AAAAGCTGTT-TGAAATCACAAATAATCGAAAC

Jasper AACTAAATCTGACCCTTATCGAAATC-AAAAGCTGTT-TGAAATCACAAATAATCGAAAC

Joelle phyto AACTAAATCTGACCCTTATCGAAATC-AAAAGCTGTT-TGAAATCACAAATAATCGAAAC

Joelle NCBI AACTAAATCTGACCCTTATCGAAATC-AAAAGCTGTT-TGAAATCACAAATAATCGAAAC

CN 119243 ------------------------------------------------------------

CN 120025 ------------------------------------------------------------

Joelle AAFC ------------------------------------------------------------

CAM 241 GATAAAAGAATATACCATAAGCAC-AAGAAACAAGA-------TAAAACAGCATTT-CGC

17CS1133 GATAAAAGAATATACCAT-AGCAC-AGTAAACAAGA-------TAAAACAGCATTT-CGC

CAM 236 GATAAAAGAATATACCAT-AGCAC-AATAAACAAGA-------TAAAACAGCATTT-CGT

Blaine Creek GATAAAAGAATATACCAT-AGCAC-AATAAACAAGA-------TAAAACAGCATTT-CGC

CN 119300 GATAAAAGAATATACCAT-AGCACAAGTAAACAAGA-------TAAAACAGCATTT-CGC

Yellowstone GATAAAAGAATATACCAT-AGCAC-AGTAAACAAGA-------TAAAACAGCATTT-CGC

Hoga GATAAAAGAATATACCAT-AGCAC-AATAAACAAGA-------TAAAACAGCATTT-CGC

CN 120027 GATAAAAGAATATACCAT-AGCAC-AATAAACAAGA-------TAAAACAGCATTT-CGC

CN 120030 GATAAAAGAATATACCAT-AGCACAAGTAAACAAGA-------TAAAACAGCATTT-CGC

CN 119294 GATAAAAGAATATACCAT-AGCAC-AGTAAACAAGA-------TAAAACAGCATTT-CGC

CN 120013 GATAAAAGAATATACCAT-AGCAC-AATAAACAAGA-------TAAAACAGCATTTACGC

CN 120017 GATAAAAGAATATACCAT-AGCACGAGTAAACAAGA-------TAAAACAGCATTT-CGC

CN 119205 ------------------------------------------------------------

DH55 ref genome NNNNNNNNNNNNNNNNNNNNNNNNNNNNNNNNNNNNNNNNNNNNNNNNNNNNNNNNNNNN

09-CS0040 AAATAACGAA------ATTAGGAGGAAGAAGAAAGACATGCCTCAAAAATGGATTCGAGC

CN 113754 AAATAACGAA------ATTAGGAGGAAGAAGAAAGACATGCCTCAAAAATGGATTCGAGC

CO46 NCBI AAATAACGAA------ATTAGGAGGAAGAAGAAAGACATGCCTCAAAAATGGATTCGAGC

Jasper AAATAACGAA------ATTAGGAGGAAGAAGAAAGACATGCCTCAAAAATGGATTCGAGC

Joelle phyto AAATAACGAA------ATTAGGAGGAAGAAGAAAGACATGCCTCAAAAATGGATTCGAGC

Joelle NCBI AAATAACGAA------ATTAGGAGGAAGAAGAAAGACATGCCTCAAAAATGGATTCGAGC

CN 119243 ------------------------------------------------------------

CN 120025 ------------------------------------------------------------

Joelle AAFC ------------------------------------------------------------

CAM 241 -AACAGCTATATTAGATATGTATAACATAG-------------------CAC-GAAAAAT

17CS1133 -AACAGCTATATTAGATATGTATAACATAG-------------------CAC-GAAAAAT

CAM 236 -AACAGCTATATTAGATATGTATAACATAG-------------------CAC-GAAAAAT

Blaine Creek -AACAGCTATATTAGATATGTATAACATAG-------------------CAC-TAAAAAT

CN 119300 -AACAGCTATATTAGATATGTATAACATAG-------------------CACTGAAAAAT

Yellowstone -AACAGCTATATTAGATATGTATAACATAG-------------------CAC-GAAAAAT

Hoga -AACAGCTATATTAGATATGTATAACATAG-------------------CAC-GAAAAAT

CN 120027 -AACAGCTATATTAGATATGTATAACATAG-------------------CAC-GAAAAAT

CN 120030 -AACAGCTATATTAGATATGTATAACATAA-------------------CAC-GAAAAAT

CN 119294 -AACAGCTATATTAGATATGTATAACATAG-------------------CAC-GAAAAAT

CN 120013 -AACAGCTATATTAGATATGTATAACATAG-------------------CAC-GAAAAAT

CN 120017 AACATGCTATATTAGATATGTATAACATAG-------------------CAC-GAAAAAT

CN 119205 ------------------------------------------------------------

DH55 ref genome NNNNNNNNNNNNNNNNNNNNNNNNNNNNNNNNNNNNNNNNNNNNNNNNNNNNNNNNNNNN

09-CS0040 TATCAATTGCGATGGATATGGA-GATTTAG-GGCGAAATTGGGGCTTTTCAC-AAACGGC

CN 113754 TATCAATTGCGATGGATATGGA-GATTTAG-GGCGAAATTGGGGCTTTTCAC-AAACGGC

CO46 NCBI TATCAATTGCGATGGATATGGA-GATTTAG-GGCGAAATTGGGGCTTTTCAC-AAACGGC

Jasper TATCAATTGCGATGGATATGGA-GATTTAGAGGCGAAATTGGGGCTTTTCAC-AAACGGC

Joelle phyto TATCAATTGCGATGGATATGGA-GATTTAG-GGCGAAATTGGGGCTTTTCAC-AAACGGC

Joelle NCBI TATCAATTGCGATGGATATGGA-GATTTAG-GGCGAAATTGGGGCTTTTCAC-AAACGGC

CN 119243 ------------------------------------------------------------

CN 120025 ------------------------------------------------------------

Joelle AAFC ------------------------------------------------------------

CAM 241 TAAAT------ATTGTATAAATTTTTGCAAAATTATAATCTTGATA--------------

17CS1133 TAAAT------ATTGTATAAATTTTTGCAAAATTATAATCTTGATA--------------

CAM 236 TAAAT------ATTGTATAAATTTTTGCAAAATTATAATCTTGATA--------------

Blaine Creek TAAAT------ATTGTATAAATTTTTGCAAAATTATAATCTTGATA--------------

CN 119300 TAAAT------ATTGTATAAATTTTTGCAAAATTATAATCTTGATA--------------

Yellowstone TAAAT------ATTGTATAAATTTTTGCAAAATTATAATCTTGATA--------------

Hoga TAAAT------ATTGTATAAATTTTTGCAAAATTATAATCTTGATA--------------

CN 120027 TAAAT------ATTGTATAAATTTTTGCAAAATTATAATCTTGATA--------------

CN 120030 TAAAT------ATTGTATAAATTTTTGCAAAATTATAATCTTGATA--------------

CN 119294 TAAAT------ATTGTATAAATTTTTGCAAAATTATAATCTTGATA--------------

CN 120013 TAAAT------ATTGTATAAATTTTTGCAAAATTATAATCTTGATA--------------

CN 120017 TAAAT------ATTGTATAAATTTTTGCAAAATTATAATCTTGATA--------------

CN 119205 ------------------------------------------------------------

DH55 ref genome NNNNNNNNNNNNNNNNNNNNNNNNNNNNNNNNNNNNNNNNNNNNNNNNNNNNNNNNNNNN

09-CS0040 GAAATTGGGGCTTTTCACAAACGGCGATAAAACTGAAGAATTGATAAGAAATTTAGAAGA

CN 113754 GAAATTGGGGCTTTTCACAAACGGCGATAAAACTGAAGAATTGATAAGAAATTTAGAAGA

CO46 NCBI GAAATTGGGGCTTTTCACAAACGGCGATAAAACTGAAGAATTGATAAGAAATTTAGAAGA

Jasper GAAATTGGGGCTTTTCACAAACGGCGATAAAACTGAAGAATTGATAAGAAATTTAGAAGA

Joelle phyto GAAATTGGGGCTTTTCACAAACGGCGATAAAACTGAAGAATTGATAAGAAATTTAGAAGA

Joelle NCBI GAAATTGGGGCTTTTCACAAACGGCGATAAAACTGAAGAATTGATAAGAAATTTAGAAGA

CN 119243 ------------------------------------------------------------

CN 120025 ------------------------------------------------------------

Joelle AAFC ------------------------------------------------------------

CAM 241 ----------ATACAGATAGTTTATTAGAAGCTATGCT------------ATAAAACTAT

17CS1133 ----------ATACAGACAGTTTATTAG-AGCTATGCT------------ATAAAACTAT

CAM 236 ----------ATACAAGCAGTTTATTAG-AGCTATGCT------------ATAAAACTAT

Blaine Creek ----------ATACAAAGAGTTTATTAG-AGCTATGCT------------ATAAAACTAT

CN 119300 ----------ATAC-AGCAGTTTATTAG-AGCTATGCT------------ATAAAACTAT

Yellowstone ----------ATAC-AGTAGTTTATTAG-AGCTATGCT------------ATAAAACTAT

Hoga ----------ATAC-AGCAGTTTATTAG-AGCTATGCT------------ATAAAACTAT

CN 120027 ----------ATACAAATAGTTTATTAG-AGCTATGCT------------ATAAAACTAT

CN 120030 ----------ATACAATGAGTTTATTAG-AGCTATGCT------------ATAAAACTAT

CN 119294 ----------ATACAAGCAGTTTATTAG-AGCTATGCT------------ATAAAACTAT

CN 120013 ----------ATAC-AGCAGTTTATTAG-AGCTATGCT------------ATAAAACTAT

CN 120017 ----------ATAC-AGCAGTTTATTAG-AGCTATGCT------------ATAAAACTAT

CN 119205 ------------------------------------------------------------

DH55 ref genome NNNNNNNNNNNNNNNNNNNNNNNNNNNNNNNNNNNNNNNNNNNNNNNNNNNNNNNNNNNN

09-CS0040 ATTTGAGCACACACAGAGAATTTAGAAG-AAGAAATCTTCTTAGGTTGAGCTTAGGTTGT

CN 113754 ATTTGAGCACACACAGAGAATTTAGAAG-AAGAAATCTTCTTAGGTTGAGCTTAGGTTGT

CO46 NCBI ATTTGAGCACACACAGAGAATTTAGAAG-AAGAAATCTTCTTAGGTTGAGCTTAGGTTGT

Jasper ATTTGAGCACACACAGAGAATTTAGAAG-AAGAAATCTTCTTAGGTTGAGCTTAGGTTGT

Joelle phyto ATTTGAGCACACACAGAGAATTTAGAAG-AAGAAATCTTCTTAGGTTGAGCTTAGGTTGT

Joelle NCBI ATTTGAGCACACACAGAGAATTTAGAAG-AAGAAATCTTCTTAGGTTGAGCTTAGGTTGT

CN 119243 ------------------------------------------------------------

CN 120025 ------------------------------------------------------------

Joelle AAFC ------------------------------------------------------------

CAM 241 AACAT-AGCA--TCGAAAAAACAAATGT------TGTATAAATTTTTTCAAAATTATGAT

17CS1133 AACAT-AACGATCTGAAAAAACAAATGT------TGTATAAATTTTTTCAAAATTATGAT

CAM 236 AACATAAGCA--C-GAAAAAACAAATGT------TGTATAAATTTTTTCAAAATTATGAT

Blaine Creek AACAT-AGCA--C-GAAAAAACAAATGT------TGTATAAATTTTTTCAAAATTATGAT

CN 119300 AACAT-AGCA--C-GAAAAAACAAATGT------TGTATAAATTTTTTCAAAATTATGAT

Yellowstone AACAT-AGCA--CTGAAAAAACAAATGT------TGTATAAATTTTTTCAAAATTATGAT

Hoga AACAT-AGCA--C-GAAAAAACAAATGT------TGTATAAATTTTTTCAAAATTATGAT

CN 120027 AACAT-AGCA--C-GAAAAAACAAATGT------TGTATAAATTTTTTCAAAATTATGAT

CN 120030 AACAT-AACA--C-GAAAAAACAAATGT------TGTATAAATTTTTTCAAAATTATGAT

CN 119294 AACAT-AGCA--C-GAAAAAACAAATGT------TGTATAAATTTTTTCAAAATTATGAT

CN 120013 AACAT-AGCA--C-GAAAAAACAAATGT------TGTATAAATTTTTTCAAAATTATGAT

CN 120017 AACAT-AGCA--C-GAAAAAACAAATGT------TGTATAAATTTTTTCAAAATTATGAT

CN 119205 ------------------------------------------------------------

DH55 ref genome NNNNNNNNNNNNNNNNNNNNNNNNNNNNNNNNNNNNNNNGGGTTTTTTTTTTATTTGG--

09-CS0040 AACGAGAGA-----GAGAGAGAGAGTGTGTCGAGTTTTTGGGTTTTTTTTTTATTTGG--

CN 113754 AACGAGAGA-----GAGAGAGAGAGTGTGTCGAGTTTTTGGGTTTTTTTTTTATTTGG--

CO46 NCBI AACGAGA-------GAGAGAGAGAGTGTGTCGAGTTTTTGGGTTTTTTTTTTATTTGG--

Jasper AACGAGA-------GAGAGAGAGAGTGTGTCGAGTTTTTGGGTTTTTTTTTTATTTGG--

Joelle phyto AACGA-GAGA----GAGAGAGAGAGTGTGTCGAGTTTTTGGGTTTTTTTTTTATTTGG--

Joelle NCBI AACGA-GAGA----GAGAGAGAGAGTGTGTCGAGTTTTTGGGTTTTTTTTTTATTTGG--

CN 119243 ------------------------------------------------------------

CN 120025 ------------------------------------------------------------

Joelle AAFC ------------------------------------------------------------

CAM 241 CTTGATAATATAAGCAATTTCTTAAAG--------------------CTATGTTAT----

17CS1133 CTTGATAATAT-AGCAATTTCTTAAAG--------------------CTATGTTAT----

CAM 236 CTTGATAATAT-AGCAATTTCTTAAAG--------------------TTATGTTAT----

Blaine Creek CTTGATAATAT-AGCAATTTCTTAAAG--------------------CTATGTTAT----

CN 119300 CTTGATAATAT-AGCAATTTCTTAAAG--------------------CTATGTTAT----

Yellowstone CTTGATAATAT-AGCAATTTCTTAAAG--------------------CTATGTTAT----

Hoga CTTGATAATAT-AGCAATTTCTTAAAG--------------------CTATGTTAT----

CN 120027 CTTGATAATAT-AGCAATTTCTTAAAG--------------------CTATGTTAT----

CN 120030 CTTGATAATAT-AGCAATTTCTTAAAG--------------------CTATGTTAT----

CN 119294 CTTGATAATAT-AGCAATTTCTTAAAG--------------------CTATGTTAT----

CN 120013 CTTGATAATAT-AGCAATTTCTTAAAG--------------------CTATGTTAT----

CN 120017 CTTGATAATAT-AGCAATTTCTTAAAG--------------------CTATGTTAT----

CN 119205 ------------------------------------------------------------

DH55 ref genome --CGATTAAGTGAATAATTTCACTAAGTATTGGCGGACAAAAGTCACTTTTGTTTCCCGC

09-CS0040 --CGATTAAGTGAATAATTTCACTAAGTATTGGCGGACAAAAGTCACTTTTGTTTCCCGC

CN 113754 --CGATTAAGTGAATAATTTCACTAAGTATTGGCGGACAAAAGTCACTTTTGTTTCCCGC

CO46 NCBI --CGATTAAGTGAATAATTTCACTAAGTATTGGCGGACAAAAGTCACTTTTGTTTCCCGC

Jasper --CGATTAAGTGAATAATTTCACTAAGTATTGGCGGACAAAAGTCACTTTTGTTTCCCGC

Joelle phyto --CGATTAAGTGAATAATTTCACTAAGTATTGGCGGACAAAAGTCACTTTTGTTTCCCGC

Joelle NCBI --CGATTAAGTGAATAATTTCACTAAGTATTGGCGGACAAAAGTCACTTTTGTTTCCCGC

CN 119243 ------------------------------------------------------------

CN 120025 ------------------------------------------------------------

Joelle AAFC ------------------------------------------------------------

CAM 241 ------ATTTTTATAACAT--AAGATACGTAATAAGTCGCTATT-GTATATGGGGTACAT

17CS1133 ------ATTTTTATAACAT--AGCATACGTAATAAGTGC-TATT-GTATATGGGGTACAT

CAM 236 ------ATTTTTATAACAT-AAGCATACGTAATAAGTCGCTATT-GTATATGAGGTACAT

Blaine Creek ------ATTTTTATAACAT--AGCATACGTAATAAGTGC-TATT-GTATATGGGGTACAT

CN 119300 ------ATTTTTATAACAT--AGCATACGTAATAAGTGC-TATT-GTATATGGGGTACAT

Yellowstone ------ATTTTTATAACAT--AGCATACGTAATAAGTGC-TATT-GTATATGGGGTACAT

Hoga ------ATTTTTATAACAT-AAGCATACGTAATAAGTCGTTATT-GTATATGGGGTACAT

CN 120027 ------ATTTTTATAACAT-AAGCATACGTAATAAGTGC-TATT-GTATATGGGGTACAT

CN 120030 ------ATTTTTATAACATAAAGCATACGTAATAAGTGCTTATTAGTATAT-GGGTACAT

CN 119294 ------ATTTTTATAACAT-AAGCATACGTAATAAGTGCTTATT-GTATATGGGGTACAT

CN 120013 ------ATTTTTATAACAT--AGCATACGTAATAAGTGC-TATT-GTATATGGGGTACAT

CN 120017 ------ATTTTTATAACAT--AGCATACGTAATAAGTGC-TATT-GTATATAGGGTACAT

CN 119205 ------------------------------------------------------------

DH55 ref genome TACATAATTTTCCTATCAT--AGCGT---TAATAAGATGCTATT-----AAAAAGTAAGC

09-CS0040 TACATAATTTTCCTATCAT--AGCGT---TAATAAGATGCTATT-----AAAAAGTAAGC

CN 113754 TACATAATTTTCCTATCAT--AGCGT---TAATAAGATGCTATT-----AAAAAGTAAGC

CO46 NCBI TACATAATTTTCCTATCAT--AGCGT---TAATAAGATGCTATT-----AAAAAGTAAGC

Jasper TACATAATTTTCCTATCAT--AGCGT---TAATAAGATGCTATT-----AAAAAGTAAGC

Joelle phyto TACATAATTTTCCTATCAT--AGCGT---TAATAAGATGCTATT-----AAAAAGTAAGC

Joelle NCBI TACATAATTTTCCTATCAT--AGCGT---TAATAAGATGCTATT-----AAAAAGTAAGC

CN 119243 ------------------------------------------------------------

CN 120025 ------------------------------------------------------------

Joelle AAFC ------------------------------------------------------------

CAM 241 ATAATAAC-AGTGCC-CTAAAACAGCGCTCATG-AAAACGCTATCAAAGACCTATGATAG

17CS1133 ATAATAAC-AGTGCC-CTAAAACAGCGCTCATG-AAAACGCTATCAAAGACCTATGATAG

CAM 236 ATAATAACGAGTGCCTCTAAAACAGTATTATCGTAAAACGCTATCAAAGACCTATGATAG

Blaine Creek ATAATAAC-AGTGCC-CTAAAACAGCGCTCATG-AAAACGCTATCAAAGACCTATGATAG

CN 119300 ATAATAAC-AGTGCC-CTAAAACAGCGCTCATG-AAAACGCTATCAAAGACCTATGATAG

Yellowstone ATAATAAC-AGTGCC-CTAAAACAGCGCTCATG-AAAACGCTATCAAAGACCTATGATAG

Hoga ATAATAAC-AGTGCC-CTAAAACAGCGCTCATG-AAAACGCTATCAAAGACCTATGATAG

CN 120027 ATAATAAC-AGTGCC-CTAAAACAGCGCTCATG-AAAACGCTATCAAAGACCTATGATAG

CN 120030 ATAATAACGATTACTCCTAAAACAGCGCTCATG-AAAACGCTATCAAAGACCTATGATAG

CN 119294 ATAATAACAAGTGCCTCTAAAACAATACTCATG-AAAACGCTATCAAAGACCTATGATAG

CN 120013 ATAATAAC-AGTGCC-CTAAAACAGCGCTCATG-AAAACGCTATCAAAGACCTATGATAG

CN 120017 ATAATAAC-AGTGCC-CTAAAACAGCGCTCATG-AAAACGCTATCAAAGACCTATGATAG

CN 119205 ------------------------------------------------------------

DH55 ref genome GAAAAAAA-ATCTCATCTTTCATAGCAGTGACGGAAAATG------------------AG

09-CS0040 GAAAAAAA-ATCTCATCTTTCATAGCAGTGACGGAAAATG------------------AG

CN 113754 GAAAAAAA-ATCTCATCTTTCATAGCAGTGACGGAAAATG------------------AG

CO46 NCBI GAAAAAAA-ATCTCATCTTTCATAGCAGTGACGGAAAATG------------------AG

Jasper GAAAAAAA-ATCTCATCTTTCATAGCAGTGACGGAAAATG------------------AG

Joelle phyto GAAAAAAA-ATCTCATCTTTCATAGCAGTGACGGAAAATG------------------AG

Joelle NCBI GAAAAAAA-ATCTCATCTTTCATAGCAGTGACGGAAAATG------------------AG

CN 119243 ------------------------------------------------------------

CN 120025 ------------------------------------------------------------

Joelle AAFC ------------------------------------------------------------

CAM 241 CGTTTTTCTCTGGTTAACAATGTGCATATTTGTTGTAGTGATTGCTAAATGATTCACTAG

17CS1133 CGTTTTTCTCTGGTTAACAATGTGCATATTTGTTGTAGTGATTGCTAAATGATTCACTAG

CAM 236 CGTTTTTCTCTGGTTAACAATGTGCATATTTGTTGTAGTGATTGCTAAATGATTCACTAG

Blaine Creek CGTTTTTCTCTGGTTAACAATGTGCATATTTGTTGTAGTGATTGCTAAATGATTCACTAG

CN 119300 CGTTTTTCTCTGGTTAACAATGTGCATATTTGTTGTAGTGATTGCTAAATGATTCACTAG

Yellowstone CGTTTTTCTCTGGTTAACAATGTGCATATTTGTTGTAGTGATTGCTAAATGATTCACTAG

Hoga CGTTTTTCTCTGGTTAACAATGTGCATATTTGTTGTAGTGATTGCTAAATGATTCACTAG

CN 120027 CGTTTTTCTCTGGTTAACAATGTGCATATTTGTTGTAGTGATTGCTAAATGATTCACTAG

CN 120030 CGTTTTTCTCTGGTTAACAATGTGCATATTTGTTGTAGTGATTGCTAAATGATTCACTAG

CN 119294 CGTTTTTCTCTGGTTAACAATGTGCATATTTGTTGTAGTGATTGCTAAATGATTCACTAG

CN 120013 CGTTTTTCTCTGGTTAACAATGTGCATATTTGTTGTAGTGATTGCTAAATGATTCACTAG

CN 120017 CGTTTTTCTCTGGTTAACAATGTGCATATTTGTTGTAGTGATTGCTAAATGATTCACTAG

CN 119205 -------------------------------------------GCTAAATGATTCACTAG

DH55 ref genome CTATATCCGTCTGCTATCAATGTACATATTTGTTGTAGTGACGGAAAA------------

09-CS0040 CTATATCCGTCTGCTATCAATGTACATATTTGTTGTAGTGACGGAAAA------------

CN 113754 CTATATCCGTCTGCTATCAATGTACATATTTGTTGTAGTGACGGAAAA------------

CO46 NCBI CTATATCCGTCTGCTATCAATGTACATATTTGTTGTAGTGACGGAAAA------------

Jasper CTATATCCGTCTGCTATCAATGTACATATTTGTTGTAGTGACGGAAAA------------

Joelle phyto CTATATCCGTCTGCTATCAATGTACATATTTGTTGTAGTG--------------------

Joelle NCBI CTATATCCGTCTGCTATCAATGTACATATTTGTTGTAGTG--------------------

CN 119243 -------------------------------------------GCTAAATGATTCACTAG

CN 120025 -------------------------------------------GCTAAATGATTCACTAG

Joelle AAFC -------------------------------------------GCTAAATGATTCACTAG

CAM 241 ATCTCTCCTTTTTTATAGTGATTAAAACTCATTAGATCTCTTTGGATTTGTATTCAGTGC

17CS1133 ATCTCTCCTTTTTTATAGTGATTAAAACTCATTAGATCTCTTTGGATTTGTATTCAGTGC

CAM 236 ATCTCTCCTTTTTTATAGTGATTAAAACTCATTAGATCTCTTTGGATTTGTATTCAGTGC

Blaine Creek ATCTCTCCTTTTTTATAGTGATTAAAACTCATTAGATCTCTTTGGATTTGTATTCAGTGC

CN 119300 ATCTCTCCTTTTTTATAGTGATTAAAACTCATTAGATCTCTTTGGATTTGTATTCAGTGC

Yellowstone ATCTCTCCTTTTTTATAGTGATTAAAACTCATTAGATCTCTTTGGATTTGTATTCAGTGC

Hoga ATCTCTCCTTTTTTATAGTGATTAAAACTCATTAGATCTCTTTGGATTTGTATTCAGTGC

CN 120027 ATCTCTCCTTTTTTATAGTGATTAAAACTCATTAGATCTCTTTGGATTTGTATTCAGTGC

CN 120030 ATCTCTCCTTTTTTATAGTGATTAAAACTCATTAGATCTCTTTGGATTTGTATTCAGTGC

CN 119294 ATCTCTCCTTTTTTATAGTGATTAAAACTCATTAGATCTCTTTGGATTTGTATTCAGTGC

CN 120013 ATCTCTCCTTTTTTATAGTGATTAAAACTCATTAGATCTCTTTGGATTTGTATTCAGTGC

CN 120017 ATCTCTCCTTTTTTATAGTGATTAAAACTCATTAGATCTCTTTGGATTTGTATTCAGTGC

CN 119205 ATCTCTCCTTTTTTATAGTGATTAAAACTCATTAGATCTCTTTGGATTTGTATTCAGTGC

DH55 ref genome ----TCCCATCTGCACAATG--TAGTATACATTA------TTTAGCATTAACCCCACTAC

09-CS0040 ----TCCCATCTGCACAATG--TAGTATACATTA------TTTAGCATTAACCCCACTAC

CN 113754 ----TCCCATCTGCACAATG--TAGTATACATTA------TTTAGCATTAACCCCACTAC

CO46 NCBI ----TCCCATCTGCACAATG--TAGTATACATTA------TTTAGCATTAACCCCACTAC

Jasper ----TCCCATCTGCACAATG--TAGTATACATTA------TTTAGCATTAACCCCACTAC

Joelle phyto ------------------------------------------------------------

Joelle NCBI ------------------------------------------------------------

CN 119243 ATCTCTCCTTTTTTATAGTGATTAAAACTCATTAGATCTCTTTGGATTTGTATCCAGTGC

CN 120025 ATCTCTCCTTTTTTATAGTGATTAAAACTCATTAGATCTCTTTGGATTTGTATCCAGTGC

Joelle AAFC ATCTCTCCTTTTTTATAGTGATTAAAACTCATTAGATCTCTTTGGATTTGTATCCAGTGC

CAM 241 AATGAACCTTCG---GGAGATCCATAGAATTTCAATGGGGTTAATGCTAAATAATGTATA

17CS1133 AATGAACCTTCG---GGAGATCCATAGAATTTCAATGGGGTTAATGCTAAATAATGTATA

CAM 236 AATGAACCTTCG---GGAGATCCATAGAATTTCAATGGGGTTAATGCTAAATAATGTATA

Blaine Creek AATGAACCTTCG---GGAGATCCATAGAATTTCAATGGGGTTAATGCTAAATAATGTATA

CN 119300 AATGAACCTTCG---GGAGATCCATAGAATTTCAATGGGGTTAATGCTAAATAATGTATA

Yellowstone AATGAACCTTCG---GGAGATCCATAGAATTTCAATGGGGTTAATGCTAAATAATGTATA

Hoga AATGAACCTTCG---GGAGATCCATAGAATTTCAATGGGGTTAATGCTAAATAATGTATA

CN 120027 AATGAACCTTCG---GGAGATCCATAGAATTTCAATGGGGTTAATGCTAAATAATGTATA

CN 120030 AATGAACCTTCG---GGAGATCCATAGAATTTCAATGGGGTTAATGCTAAATAATGTATA

CN 119294 AATGAACCTTCG---GGAGATCCATAGAATTTCAATGGGGTTAATGCTAAATAATGTATA

CN 120013 AATGAACCTTCG---GGAGATCCATAGAATTTCAATGGGGTTAATGCTAAATAATGTATA

CN 120017 AATGAACCTTCG---GGAGATCCATAGAATTTCAATGGGGTTAATGCTAAATAATGTATA

CN 119205 AATGAACCTTCG---GGAGATCCATAGAATTTCAATGGGGTTAATGCTAAATAATGTATA

DH55 ref genome AACAAATATGTACATTGATAGCTAAATAATTTCAATGGGGTTAATGCTAAATAATGTATA

09-CS0040 AACAAATATGTACATTGATAGCTAAATAATTTCAATGGGGTTAATGCTAAATAATGTATA

CN 113754 AACAAATATGTACATTGATAGCTAAATAATTTCAATGGGGTTAATGCTAAATAATGTATA

CO46 NCBI AACAAATATGTACATTGATAGCTAAATAATTTCAATGGGGTTAATGCTAAATAATGTATA

Jasper AACAAATATGTACATTGATAGCTAAATAATTTCAATGGGGTTAATGCTAAATAATGTATA

Joelle phyto ------------------------------------GGGGTTAATGCTAAATAATGTATA

Joelle NCBI ------------------------------------GGGGTTAATGCTAAATAATGTATA

CN 119243 AATGAACCTTCG---GGAGATCCATAGAATTTCAATGGGGTTAATGCTAAATAATGTATA

CN 120025 AATGAACCTTCG---GGAGATCCATAGAATTTCAATGGGGTTAATGCTAAATAATGTATA

Joelle AAFC AATGAACCTTCG---GGAGATCCATAGAATTTCAATGGGGTTAATGCTAAATAATGTATA

************************

CAM 241 CTACATTGTGCAGATATTGACTCTAAGTTTATTGTTGATCTTCTATGAATTTCTCTCTTT

17CS1133 CTACATTGTGCAGATATTGACTCTAAGTTTATTGTTGATCTTCTATGAATTTCTCTCTTT

CAM 236 CTACATTGTGCAGATATTGACTCTAAGTTTATTGTTGATCTTCTATGAATTTCTCTCTTT

Blaine Creek CTACATTGTGCAGATATTGACTCTAAGTTTATTGTTGATCTTCTATGAATTTCTCTCTTT

CN 119300 CTACATTGTGCAGATATTGACTCTAAGTTTATTGTTGATCTTCTATGAATTTCTCTCTTT

Yellowstone CTACATTGTGCAGATATTGACTCTAAGTTTATTGTTGATCTTCTATGAATTTCTCTCTTT

Hoga CTACATTGTGCAGATATTGACTCTAAGTTTATTGTTGATCTTCTATGAATTTCTCTCTTT

CN 120027 CTACATTGTGCAGATATTGACTCTAAGTTTATTGTTGATCTTCTATGAATTTCTCTCTTT

CN 120030 CTACATTGTGCAGATATTGACTCTAAGTTTATTGTTGATCTTCTATGAATTTCTCTCTTT

CN 119294 CTACATTGTGCAGATATTGACTCTAAGTTTATTGTTGATCTTCTATGAATTTCTCTCTTT

CN 120013 CTACATTGTGCAGATATTGACTCTAAGTTTATTGTTGATCTTCTATGAATTTCTCTCTTT

CN 120017 CTACATTGTGCAGATATTGACTCTAAGTTTATTGTTGATCTTCTATGAATTTCTCTCTTT

CN 119205 CCACATTGTGCAGATATTGACTCTAAGTTTATTGTTGATCTTCTATGAATTTCTCTCTTT

DH55 ref genome CTACATTGTGCAGATATTGACTCTAAGTTTATTGTTGATCTTCTATGAATTTCTCTCTTT

09-CS0040 CTACATTGTGCAGATATTGACTCTAAGTTTATTGTTGATCTTCTATGAATTTCTCTCTTT

CN 113754 CTACATTGTGCAGATATTGACTCTAAGTTTATTGTTGATCTTCTATGAATTTCTCTCTTT

CO46 NCBI CTACATTGTGCAGATATTGACTCTAAGTTTATTGTTGATCTTCTATGAATTTCTCTCTTT

Jasper CTACATTGTGCAGATATTGACTCTAAGTTTATTGTTGATCTTCTATGAATTTCTCTCTTT

Joelle phyto CCACATTGTGCAGATATTGACTCTAAGTTTATTGTTGATCTTCTATGAATTTCTCTCTTT

Joelle NCBI CCACATTGTGCAGATATTGACTCTAAGTTTATTGTTGATCTTCTATGAATTTCTCTCTTT

CN 119243 CCACATTGTGCAGATATTGACTCTAAGTTTATTGTTGATCTTCTATGAATTTCTCTCTTT

CN 120025 CCACATTGTGCAGATATTGACTCTAAGTTTATTGTTGATCTTCTATGAATTTCTCTCTTT

Joelle AAFC CCACATTGTGCAGATATTGACTCTAAGTTTATTGTTGATCTTCTATGAATTTCTCTCTTT

* **********************************************************

CAM 241 GTCATGGACCTATATTACTTGATGATTATCCAAATTAGTGTTTCTAATTGAAAAACTCAG

17CS1133 GTCATGGACCTATATTACTTGATGATTATCCAAATTAGTGTTTCTAATTGAAAAACTCAG

CAM 236 GTCATGGACCTATATTACTTGATGATTATCCAAATTAGTGTTTCTAATTGAAAAACTCAG

Blaine Creek GTCATGGACCTATATTACTTGATGATTATCCAAATTAGTGTTTCTAATTGAAAAACTCAG

CN 119300 GTCATGGACCTATATTACTTGATGATTATCCAAATTAGTGTTTCTAATTGAAAAACTCAG

Yellowstone GTCATGGACCTATATTACTTGATGATTATCCAAATTAGTGTTTCTAATTGAAAAACTCAG

Hoga GTCATGGACCTATATTACTTGATGATTATCCAAATTAGTGTTTCTAATTGAAAAACTCAG

CN 120027 GTCATGGACCTATATTACTTGATGATTATCCAAATTAGTGTTTCTAATTGAAAAACTCAG

CN 120030 GTCATGGACCTATATTACTTGATGATTATCCAAATTAGTGTTTCTAATTGAAAAACTCAG

CN 119294 GTCATGGACCTATATTACTTGATGATTATCCAAATTAGTGTTTCTAATTGAAAAACTCAG

CN 120013 GTCATGGACCTATATTACTTGATGATTATCCAAATTAGTGTTTCTAATTGAAAAACTCAG

CN 120017 GTCATGGACCTATATTACTTGATGATTATCCAAATTAGTGTTTCTAATTGAAAAACTCAG

CN 119205 GTCATGGACCTATATTACTTGATGATTATCCAAATTAGTGTTTCTAATTGAAAAACTCAG

DH55 ref genome GTCATGGACCTATATTACTTGATGATTATCCAAATTAGTGTTTCTAATTGAAAAACTCAG

09-CS0040 GTCATGGACCTATATTACTTGATGATTATCCAAATTAGTGTTTCTAATTGAAAAACTCAG

CN 113754 GTCATGGACCTATATTACTTGATGATTATCCAAATTAGTGTTTCTAATTGAAAAACTCAG

CO46 NCBI GTCATGGACCTATATTACTTGATGATTATCCAAATTAGTGTTTCTAATTGAAAAACTCAG

Jasper GTCATGGACCTATATTACTTGATGATTATCCAAATTAGTGTTTCTAATTGAAAAACTCAG

Joelle phyto GTCATGGACCTATATTACTTGATGATTATCCAAATTAGTGTTTCTAATTGAAAAACTCAG

Joelle NCBI GTCATGGACCTATATTACTTGATGATTATCCAAATTAGTGTTTCTAATTGAAAAACTCAG

CN 119243 GTCATGGACCTATATTACTTGATGATTATCCAAATTAGTGTTTCTAATTGAAAAAATCAG

CN 120025 GTCATGGACCTATATTACTTGATGATTATCCAAATTAGTGTTTCTAATTGAAAAAATCAG

Joelle AAFC GTCATGGACCTATATTACTTGATGATTATCCAAATTAGTGTTTCTAATTGAAAAAATCAG

******************************************************* ****

CAM 241 TCTCACAATTAGTCCTTAACGCACATATGCTACTTAAGCTATGTGATCTGGTATCGATTG

17CS1133 TCTCACAATTAGTCCTTAACGCACATATGCTACTTAAGCTATGTGATCTGGTATCGATTG

CAM 236 TCTCACAATTAGTCCTTAACGCACATATGCTACTTAAGCTATGTGATCTGGTATCGATTG

Blaine Creek TCTCACAATTAGTCCTTAACGCACATATGCTACTTAAGCTATGTGATCTGGTATCGATTG

CN 119300 TCTCACAATTAGTCCTTAACGCACATATGCTACTTAAGCTATGTGATCTGGTATCGATTG

Yellowstone TCTCACAATTAGTCCTTAACGCACATATGCTACTTAAGCTATGTGATCTGGTATCGATTG

Hoga TCTCACAATTAGTCCTTAACGCACATATGCTACTTAAGCTATGTGATCTGGTATCGATTG

CN 120027 TCTCACAATTAGTCCTTAACGCACATATGCTACTTAAGCTATGTGATCTGGTATCGATTG

CN 120030 TCTCACAATTAGTCCTTAACGCACATATGCTACTTAAGCTATGTGATCTGGTATCGATTG

CN 119294 TCTCACAATTAGTCCTTAACGCACATATGCTACTTAAGCTATGTGATCTGGTATCGATTG

CN 120013 TCTCACAATTAGTCCTTAACGCACATATGCTACTTAAGCTATGTGATCTGGTATCGATTG

CN 120017 TCTCACAATTAGTCCTTAACGCACATATGCTACTTAAGCTATGTGATCTGGTATCGATTG

CN 119205 TCTCACAATTAGTCCTTAACGCACATATGCTACTTAAGCTATGTGATCTGGTATCGATTG

DH55 ref genome TCTCACAATTAGTCCTTAACGCACATATGCTACTTAAGCTATGTGATCTGGTATCGATTG

09-CS0040 TCTCACAATTAGTCCTTAACGCACATATGCTACTTAAGCTATGTGATCTGGTATCGATTG

CN 113754 TCTCACAATTAGTCCTTAACGCACATATGCTACTTAAGCTATGTGATCTGGTATCGATTG

CO46 NCBI TCTCACAATTAGTCCTTAACGCACATATGCTACTTAAGCTATGTGATCTGGTATCGATTG

Jasper TCTCACAATTAGTCCTTAACGCACATATGCTACTTAAGCTATGTGATCTGGTATCGATTG

Joelle phyto TCTCACAATTAGTCCTTAACGCACATATGCTACTTAAACTATGTGATCTGGTATCGATTG

Joelle NCBI TCTCACAATTAGTCCTTAACGCACATATGCTACTTAAACTATGTGATCTGGTATCGATTG

CN 119243 CCTCACAATTAGTCCTTAACGCACATATGCTACTTAAGCTATGTGATCTGGTATCGATTG

CN 120025 CCTCACAATTAGTCCTTAACGCACATATGCTACTTAAGCTATGTGATCTGGTATCGATTG

Joelle AAFC CCTCACAATTAGTCCTTAACGCACATATGCTACTTAAGCTATGTGATCTGGTATCGATTG

************************************ **********************

CAM 241 CGATTAATTGCAATTGTTGTGTGCATCTTTAACACTTTGTACCACACATAATGAACATTA

17CS1133 CGATTAATTGCAATTGTTGTGTGCATCTTTAACACTTTGTACCACACATAATGAACATTA

CAM 236 CGATTAATTGCAATTGTTGTGTGCATCTTTAACACTTTGTACCACACATAATGAACATTA

Blaine Creek CGATTAATTGCAATTGTTGTGTGCATCTTTAACACTTTGTACCACACATAATGAACATTA

CN 119300 CGATTAATTGCAATTGTTGTGTGCATCTTTAACACTTTGTACCACACATAATGAACATTA

Yellowstone CGATTAATTGCAATTGTTGTGTGCATCTTTAACACTTTGTACCACACATAATGAACATTA

Hoga CGATTAATTGCAATTGTTGTGTGCATCTTTAACACTTTGTACCACACATAATGAACATTA

CN 120027 CGATTAATTGCAATTGTTGTGTGCATCTTTAACACTTTGTACCACACATAATGAACATTA

CN 120030 CGATTAATTGCAATTGTTGTGTGCATCTTTAACACTTTGTACCACACATAATGAACATTA

CN 119294 CGATTAATTGCAATTGTTGTGTGCATCTTTAACACTTTGTACCACACATAATGAACATTA

CN 120013 CGATTAATTGCAATTGTTGTGTGCATCTTTAACACTTTGTACCACACATAATGAACATTA

CN 120017 CGATTAATTGCAATTGTTGTGTGCATCTTTAACACTTTGTACCACACATAATGAACATTA

CN 119205 CGATTAATTGCAATTGTTGTGTGCATCTTTAACACTTTGTACCACACATAATGAACATTA

DH55 ref genome CGATTAATTGCAATTGTTGTGTGCATCTTTAACACTTTGTACCACACATAATGAACATTA

09-CS0040 CGATTAATTGCAATTGTTGTGTGCATCTTTAACACTTTGTACCACACATAATGAACATTA

CN 113754 CGATTAATTGCAATTGTTGTGTGCATCTTTAACACTTTGTACCACACATAATGAACATTA

CO46 NCBI CGATTAATTGCAATTGTTGTGTGCATCTTTAACACTTTGTACCACACATAATGAACATTA

Jasper CGATTAATTGCAATTGTTGTGTGCATCTTTAACACTTTGTACCACACATAATGAACATTA

Joelle phyto CGATTAATTGCAATTGTTGTGTGCATCTTTAACACTTTGTACCACACATAATGAACATTA

Joelle NCBI CGATTAATTGCAATTGTTGTGTGCATCTTTAACACTTTGTACCACACATAATGAACATTA

CN 119243 CGATTAATTGCAATTGTTGTGTGCATCTTTAACACTTTGTACCACACATAATGAACATTA

CN 120025 CGATTAATTGCAATTGTTGTGTGCATCTTTAACACTTTGTACCACACATAATGAACATTA

Joelle AAFC CGATTAATTGCAATTGTTGTGTGCATCTTTAACACTTTGTACCACACATAATGAACATTA

************************************************************

CAM 241 ACTGGACTATTTTTAGACTAAAATTCATTGCTCTCATGGATTTGCATACAAATACACTCC

17CS1133 ACTGGACTATTTTTAGACTAAAATTCATTGCTCTCATGGATTTGCATACAAATACACTCC

CAM 236 ACTGGACTATTTTTAGACTAAAATTCATTGCTCTCATGGATTTGCATACAAATACACTCC

Blaine Creek ACTGGACTATTTTTAGACTAAAATTCATTGCTCTCATGGATTTGCATACAAATACACTCC

CN 119300 ACTGGACTATTTTTAGACTAAAATTCATTGCTCTCATGGATTTGCATACAAATACACTCC

Yellowstone ACTGGACTATTTTTAGACTAAAATTCATTGCTCTCATGGATTTGCATACAAATACACTCC

Hoga ACTGGACTATTTTTAGACTAAAATTCATTGCTCTCATGGATTTGCATACAAATACACTCC

CN 120027 ACTGGACTATTTTTAGACTAAAATTCATTGCTCTCATGGATTTGCATACAAATACACTCC

CN 120030 ACTGGACTATTTTTAGACTAAAATTCATTGCTCTCATGGATTTGCATACAAATACACTCC

CN 119294 ACTGGACTATTTTTAGACTAAAATTCATTGCTCTCATGGATTTGCATACAAATACACTCC

CN 120013 ACTGGACTATTTTTAGACTAAAATTCATTGCTCTCATGGATTTGCATACAAATACACTCC

CN 120017 ACTGGACTATTTTTAGACTAAAATTCATTGCTCTCATGGATTTGCATACAAATACACTCC

CN 119205 ACTGGACTATTTTTAGACTAAAATTCATTGCTCTCATGGATTTGCATACAAATACACTCC

DH55 ref genome ACTGGACTATTTTTAGACTAAAATTCATTGCTCTCATGGATTTGCATACAAATACACTCC

09-CS0040 ACTGGACTATTTTTAGACTAAAATTCATTGCTCTCATGGATTTGCATACAAATACACTCC

CN 113754 ACTGGACTATTTTTAGACTAAAATTCATTGCTCTCATGGATTTGCATACAAATACACTCC

CO46 NCBI ACTGGACTATTTTTAGACTAAAATTCATTGCTCTCATGGATTTGCATACAAATACACTCC

Jasper ACTGGACTATTTTTAGACTAAAATTCATTGCTCTCATGGATTTGCATACAAATACACTCC

Joelle phyto ACTGGACTATTTTTAGACTAAAATTCATTGCTCTCATGGATTTGCATACAAATACACTCC

Joelle NCBI ACTGGACTATTTTTAGACTAAAATTCATTGCTCTCATGGATTTGCATACAAATACACTCC

CN 119243 ACTGGACTATTTTTAGACTAAAATTCATTGCTCTCATGGATTTGCATACAAATACACTCC

CN 120025 ACTGGACTATTTTTAGACTAAAATTCATTGCTCTCATGGATTTGCATACAAATACACTCC

Joelle AAFC ACTGGACTATTTTTAGACTAAAATTCATTGCTCTCATGGATTTGCATACAAATACACTCC

************************************************************

CAM 241 CGGGAGATTTGTAAATAAAATTAGTACCGTAGATCAATACTAATTTTGGTTCAAATGTAT

17CS1133 CGGGAGATTTGTAAATAAAATTAGTACCGTAGATCAATACTAATTTTGGTTCAAATGTAT

CAM 236 CGGGAGATTTGTAAATAAAATTAGTACCGTAGATCAATACTAATTTTGGTTCAAATGTAT

Blaine Creek CGGGAGATTTGTAAATAAAATTAGTACCGTAGATCAATACTAATTTTGGTTCAAATGTAT

CN 119300 CGGGAGATTTGTAAATAAAATTAGTACCGTAGATCAATACTAATTTTGGTTCAAATGTAT

Yellowstone CGGGAGATTTGTAAATAAAATTAGTACCGTAGATCAATACTAATTTTGGTTCAAATGTAT

Hoga CGGGAGATTTGTAAATAAAATTAGTACCGTAGATCAATACTAATTTTGGTTCAAATGTAT

CN 120027 CGGGAGATTTGTAAATAAAATTAGTACCGTAGATCAATACTAATTTTGGTTCAAATGTAT

CN 120030 CGGGAGATTTGTAAATAAAATTAGTACCGTAGATCAATACTAATTTTGGTTCAAATGTAT

CN 119294 CGGGAGATTTGTAAATAAAATTAGTACCGTAGATCAATACTAATTTTGGTTCAAATGTAT

CN 120013 CGGGAGATTTGTAAATAAAATTAGTACCGTAGATCAATACTAATTTTGGTTCAAATGTAT

CN 120017 CGGGAGATTTGTAAATAAAATTAGTACCGTAGATCAATACTAATTTTGGTTCAAATGTAT

CN 119205 CGGGAGATTTGTAAATAAAATTAGTACCGTAGATCAATACTAATTTTGGTTCAAATGTAT

DH55 ref genome CGGGAGATTTGTAAATAAAATTAGTACCGTAGATCAATACTAATTTTGGTTCAAATGTAT

09-CS0040 CGGGAGATTTGTAAATAAAATTAGTACCGTAGATCAATACTAATTTTGGTTCAAATGTAT

CN 113754 CGGGAGATTTGTAAATAAAATTAGTACCGTAGATCAATACTAATTTTGGTTCAAATGTAT

CO46 NCBI CGGGAGATTTGTAAATAAAATTAGTACCGTAGATCAATACTAATTTTGGTTCAAATGTAT

Jasper CGGGAGATTTGTAAATAAAATTAGTACCGTAGATCAATACTAATTTTGGTTCAAATGTAT

Joelle phyto CGGGAGATTTGTAAATAAAATTAGTACCGTAGATCAATACTAATTTTGGTTCAAATGTAT

Joelle NCBI CGGGAGATTTGTAAATAAAATTAGTACCGTAGATCAATACTAATTTTGGTTCAAATGTAT

CN 119243 CGGGAGATTTGTAAATAAAATTAGTACCGTAGATCAATACTAATTTTGGTTCAAATGTAT

CN 120025 CGGGAGATTTGTAAATAAAATTAGTACCGTAGATCAATACTAATTTTGGTTCAAATGTAT

Joelle AAFC CGGGAGATTTGTAAATAAAATTAGTACCGTAGATCAATACTAATTTTGGTTCAAATGTAT

************************************************************

CAM 241 GCCACATTACTTGTAAAACTATTGACTAAAAGATTAATTGGGATATACATGTTTTTTATA

17CS1133 GCCACATTACTTGTAAAACTATTGACTAAAAGATTAATTGGGATATACATGTTTTTTATA

CAM 236 GCCACATTACTTGTAAAACTATTGACTAAAAGATTAATTGGGATATACATGTTTTTTATA

Blaine Creek GCCACATTACTTGTAAAACTATTGACTAAAAGATTAATTGGGATATACATGTTTTTTATA

CN 119300 GCCACATTACTTGTAAAACTATTGACTAAAAGATTAATTGGGATATACATGTTTTTTATA

Yellowstone GCCACATTACTTGTAAAACTATTGACTAAAAGATTAATTGGGATATACATGTTTTTTATA

Hoga GCCACATTACTTGTAAAACTATTGACTAAAAGATTAATTGGGATATACATGTTTTTTATA

CN 120027 GCCACATTACTTGTAAAACTATTGACTAAAAGATTAATTGGGATATACATGTTTTTTATA

CN 120030 GCCACATTACTTGTAAAACTATTGACTAAAAGATTAATTGGGATATACATGTTTTTTATA

CN 119294 GCCACATTACTTGTAAAACTATTGACTAAAAGATTAATTGGGATATACATGTTTTTTATA

CN 120013 GCCACATTACTTGTAAAACTATTGACTAAAAGATTAATTGGGATATACATGTTTTTTATA

CN 120017 GCCACATTACTTGTAAAACTATTGACTAAAAGATTAATTGGGATATACATGTTTTTTATA

CN 119205 GCCACATTACTTGTAAAACTATTGACTAAAAGATTAATTGGGATATACATGTTTTTTATA

DH55 ref genome GCCACATTACTTGTAAAACTATTGACTAAAAGATTAATTGGGATATACATGTTTTTTATA

09-CS0040 GCCACATTACTTGTAAAACTATTGACTAAAAGATTAATTGGGATATACATGTTTTTTATA

CN 113754 GCCACATTACTTGTAAAACTATTGACTAAAAGATTAATTGGGATATACATGTTTTTTATA

CO46 NCBI GCCACATTACTTGTAAAACTATTGACTAAAAGATTAATTGGGATATACATGTTTTTTATA

Jasper GCCACATTACTTGTAAAACTATTGACTAAAAGATTAATTGGGATATACATGTTTTTTATA

Joelle phyto GCCACATTACTTGTAAAACTATTGACTAAAAGATTAATTGGGATATACATGTTTTTTATA

Joelle NCBI GCCACATTACTTGTAAAACTATTGACTAAAAGATTAATTGGGATATACATGTTTTTTATA

CN 119243 GCCACATTACTTGTAAAACTATTGACTAAAAGATTAATTGGGATATACATGTTTTTTATA

CN 120025 GCCACATTACTTGTAAAACTATTGACTAAAAGATTAATTGGGATATACATGTTTTTTATA

Joelle AAFC GCCACATTACTTGTAAAACTATTGACTAAAAGATTAATTGGGATATACATGTTTTTTATA

************************************************************

CAM 241 ATGATTTCTCTCCTTTTTATGGATTTGCTTACTTGAAGATTAATTATCCAAAGGTGAATA

17CS1133 ATGATTTCTCTCCTTTTTATGGATTTGCTTACTTGAAGATTAATTATCCAAAGGTGAATA

CAM 236 ATGATTTCTCTCCTTTTTATGGATTTGCTTACTTGAAGATTAATTATCCAAAGGTGAATA

Blaine Creek ATGATTTCTCTCCTTTTTATGGATTTGCTTACTTGAAGATTAATTATCCAAAGGTGAATA

CN 119300 ATGATTTCTCTCCTTTTTATGGATTTGCTTACTTGAAGATTAATTATCCAAAGGTGAATA

Yellowstone ATGATTTCTCTCCTTTTTATGGATTTGCTTACTTGAAGATTAATTATCCAAAGGTGAATA

Hoga ATGATTTCTCTCCTTTTTATGGATTTGCTTACTTGAAGATTAATTATCCAAAGGTGAATA

CN 120027 ATGATTTCTCTCCTTTTTATGGATTTGCTTACTTGAAGATTAATTATCCAAAGGTGAATA

CN 120030 ATGATTTCTCTCCTTTTTATGGATTTGCTTACTTGAAGATTAATTATCCAAAGGTGAATA

CN 119294 ATGATTTCTCTCCTTTTTATGGATTTGCTTACTTGAAGATTAATTATCCAAAGGTGAATA

CN 120013 ATGATTTCTCTCCTTTTTATGGATTTGCTTACTTGAAGATTAATTATCCAAAGGTGAATA

CN 120017 ATGATTTCTCTCCTTTTTATGGATTTGCTTACTTGAAGATTAATTATCCAAAGGTGAATA

CN 119205 ATGATTTCTCTCCTTTTTATGGATTTGCTTACTTGAAGATTAATTATCCAAAGGTGAATA

DH55 ref genome ATGATTTCTCTCCTTTTTATGGATTTGCTTACTTGAAGATTAATTATCCAAAGGTGAATA

09-CS0040 ATGATTTCTCTCCTTTTTATGGATTTGCTTACTTGAAGATTAATTATCCAAAGGTGAATA

CN 113754 ATGATTTCTCTCCTTTTTATGGATTTGCTTACTTGAAGATTAATTATCCAAAGGTGAATA

CO46 NCBI ATGATTTCTCTCCTTTTTATGGATTTGCTTACTTGAAGATTAATTATCCAAAGGTGAATA

Jasper ATGATTTCTCTCCTTTTTATGGATTTGCTTACTTGAAGATTAATTATCCAAAGGTGAATA

Joelle phyto ATGATTTCTCTCCTTTTTATGGATTTGCTTACTTGAAGATTAATTATCCAAAGGTGAATA

Joelle NCBI ATGATTTCTCTCCTTTTTATGGATTTGCTTACTTGAAGATTAATTATCCAAAGGTGAATA

CN 119243 ATGATTTCTCTCCTTTTTATGGATTTGCTTACTTGAAGATTAATTATCCAAAGGTGAATA

CN 120025 ATGATTTCTCTCCTTTTTATGGATTTGCTTACTTGAAGATTAATTATCCAAAGGTGAATA

Joelle AAFC ATGATTTCTCTCCTTTTTATGGATTTGCTTACTTGAAGATTAATTATCCAAAGGTGAATA

************************************************************

CAM 241 GTTTCCTACCCTAGTAGTTAATTACCTCACATAGATATGCTACATATTTATGTTATTGTT

17CS1133 GTTTCCTACCCTAGTAGTTAATTACCTCACATAGATATGCTACATATTTATGTTATTGTT

CAM 236 GTTTCCTACCCTAGTAGTTAATTACCTCACATAGATATGCTACATATTTATGTTATTGTT

Blaine Creek GTTTCCTACCCTAGTAGTTAATTACCTCACATAGATATGCTACATATTTATGTTATTGTT

CN 119300 GTTTCCTACCCTAGTAGTTAATTACCTCACATAGATATGCTACATATTTATGTTATTGTT

Yellowstone GTTTCCTACCCTAGTAGTTAATTACCTCACATAGATATGCTACATATTTATGTTATTGTT

Hoga GTTTCCTACCCTAGTAGTTAATTACCTCACATAGATATGCTACATATTTATGTTATTGTT

CN 120027 GTTTCCTACCCTAGTAGTTAATTACCTCACATAGATATGCTACATATTTATGTTATTGTT

CN 120030 GTTTCCTACCCTAGTAGTTAATTACCTCACATAGATATGCTACATATTTATGTTATTGTT

CN 119294 GTTTCCTACCCTAGTAGTTAATTACCTCACATAGATATGCTACATATTTATGTTATTGTT

CN 120013 GTTTCCTACCCTAGTAGTTAATTACCTCACATAGATATGCTACATATTTATGTTATTGTT

CN 120017 GTTTCCTACCCTAGTAGTTAATTACCTCACATAGATATGCTACATATTTATGTTATTGTT

CN 119205 GTTTCCTACCCTAGTAGTTAATTACCTCACATAGATATGCTACATATTTATGTTATTGTT

DH55 ref genome GTTTCCTACCCTAGTAGTTAATTACCTCACATAGATATGCTACATATTTATGTTATTGTT

09-CS0040 GTTTCCTACCCTAGTAGTTAATTACCTCACATAGATATGCTACATATTTATGTTATTGTT

CN 113754 GTTTCCTACCCTAGTAGTTAATTACCTCACATAGATATGCTACATATTTATGTTATTGTT

CO46 NCBI GTTTCCTACCCTAGTAGTTAATTACCTCACATAGATATGCTACATATTTATGTTATTGTT

Jasper GTTTCCTACCCTAGTAGTTAATTACCTCACATAGATATGCTACATATTTATGTTATTGTT

Joelle phyto GTTTCCTACCCTAGTAGTTAATTACCTCACATAGATATGCTACATATTTATGTTATTGTT

Joelle NCBI GTTTCCTACCCTAGTAGTTAATTACCTCACATAGATATGCTACATATTTATGTTATTGTT

CN 119243 GTTTCCTACCCTAGTAGTTAATTACCTCACATAGATATGCTACATATTTATGTTATTGTT

CN 120025 GTTTCCTACCCTAGTAGTTAATTACCTCACATAGATATGCTACATATTTATGTTATTGTT

Joelle AAFC GTTTCCTACCCTAGTAGTTAATTACCTCACATAGATATGCTACATATTTATGTTATTGTT

************************************************************

CAM 241 TATCTCTTAATCTTTTCATGGAGGTGTTCATATATATAAGATTGTGTCCTCAACATGAAT

17CS1133 TATCTCTTAATCTTTTCATGGAGGTGTTCATATATATAAGATTGTGTCCTCAACATGAAT

CAM 236 TATCTCTTAATCTTTTCATGGAGGTGTTCATATATATAAGATTGTGTCCTCAACATGAAT

Blaine Creek TATCTCTTAATCTTTTCATGGAGGTGTTCATATATATAAGATTGTGTCCTCAACATGAAT

CN 119300 TATCTCTTAATCTTTTCATGGAGGTGTTCATATATATAAGATTGTGTCCTCAACATGAAT

Yellowstone TATCTCTTAATCTTTTCATGGAGGTGTTCATATATATAAGATTGTGTCCTCAACATGAAT

Hoga TATCTCTTAATCTTTTCATGGAGGTGTTCATATATATAAGATTGTGTCCTCAACATGAAT

CN 120027 TATCTCTTAATCTTTTCATGGAGGTGTTCATATATATAAGATTGTGTCCTCAACATGAAT

CN 120030 TATCTCTTAATCTTTTCATGGAGGTGTTCATATATATAAGATTGTGTCCTCAACATGAAT

CN 119294 TATCTCTTAATCTTTTCATGGAGGTGTTCATATATATAAGATTGTGTCCTCAACATGAAT

CN 120013 TATCTCTTAATCTTTTCATGGAGGTGTTCATATATATAAGATTGTGTCCTCAACATGAAT

CN 120017 TATCTCTTAATCTTTTCATGGAGGTGTTCATATATATAAGATTGTGTCCTCAACATGAAT

CN 119205 TATCTCTTAATCTTTTCATGGAGGTGTTCATATATATAAGATTGTGTCCTCAACATGAAT

DH55 ref genome TATCTCTTAATCTTTTCATGGAGGTGTTCATATATATAAGATTGTGTCCTCAACATGAAT

09-CS0040 TATCTCTTAATCTTTTCATGGAGGTGTTCATATATATAAGATTGTGTCCTCAACATGAAT

CN 113754 TATCTCTTAATCTTTTCATGGAGGTGTTCATATATATAAGATTGTGTCCTCAACATGAAT

CO46 NCBI TATCTCTTAATCTTTTCATGGAGGTGTTCATATATATAAGATTGTGTCCTCAACATGAAT

Jasper TATCTCTTAATCTTTTCATGGAGGTGTTCATATATATAAGATTGTGTCCTCAACATGAAT

Joelle phyto TATCTCTTAATCTTTTCATGGAGGTGTTCATATATATAAGATTGTGTCCTCAACATGAAT

Joelle NCBI TATCTCTTAATCTTTTCATGGAGGTGTTCATATATATAAGATTGTGTCCTCAACATGAAT

CN 119243 TATCTCTTAATCTTTTCATGGAGGTGTTCATATATATAAGATTGTGTCCTCAACATGAAT

CN 120025 TATCTCTTAATCTTTTCATGGAGGTGTTCATATATATAAGATTGTGTCCTCAACATGAAT

Joelle AAFC TATCTCTTAATCTTTTCATGGAGGTGTTCATATATATAAGATTGTGTCCTCAACATGAAT

************************************************************

CAM 241 CCACAACCTTGTAACTTTTGTTGTGCAAATTGACAAATCACACAACCTTTGTATCATGGG

17CS1133 CCACAACCTTGTAACTTTTGTTGTGCAAATTGACAAATCACACAACCTTTGTATCATGGG

CAM 236 CCACAACCTTGTAACTTTTGTTGTGCAAATTGACAAATCACACAACCTTTGTATCATGGG

Blaine Creek CCACAACCTTGTAACTTTTGTTGTGCAAATTGACAAATCACACAACCTTTGTATCATGGG

CN 119300 CCACAACCTTGTAACTTTTGTTGTGCAAATTGACAAATCACACAACCTTTGTATCATGGG

Yellowstone CCACAACCTTGTAACTTTTGTTGTGCAAATTGACAAATCACACAACCTTTGTATCATGGG

Hoga CCACAACCTTGTAACTTTTGTTGTGCAAATTGACAAATCACACAACCTTTGTATCATGGG

CN 120027 CCACAACCTTGTAACTTTTGTTGTGCAAATTGACAAATCACACAACCTTTGTATCATGGG

CN 120030 CCACAACCTTGTAACTTTTGTTGTGCAAATTGACAAATCACACAACCTTTGTATCATGGG

CN 119294 CCACAACCTTGTAACTTTTGTTGTGCAAATTGACAAATCACACAACCTTTGTATCATGGG

CN 120013 CCACAACCTTGTAACTTTTGTTGTGCAAATTGACAAATCACACAACCTTTGTATCATGGG

CN 120017 CCACAACCTTGTAACTTTTGTTGTGCAAATTGACAAATCACACAACCTTTGTATCATGGG

CN 119205 CCACAACCTTGTAACTTTTGTTGTGCAAATTGACAAATCACACAACCTTTGTATCATGGG

DH55 ref genome CCACAACCTTGTAACTTTTGTTGTGCAAATTGACAAATCACACAACCTTTGTATCATGGG

09-CS0040 CCACAACCTTGTAACTTTTGTTGTGCAAATTGACAAATCACACAACCTTTGTATCATGGG

CN 113754 CCACAACCTTGTAACTTTTGTTGTGCAAATTGACAAATCACACAACCTTTGTATCATGGG

CO46 NCBI CCACAACCTTGTAACTTTTGTTGTGCAAATTGACAAATCACACAACCTTTGTATCATGGG

Jasper CCACAACCTTGTAACTTTTGTTGTGCAAATTGACAAATCACACAACCTTTGTATCATGGG

Joelle phyto CCACAACCTTGTAACTTTTGTTGTGCAAATTGACAAATCACACAACCTTTGTATCATGGG

Joelle NCBI CCACAACCTTGTAACTTTTGTTGTGCAAATTGACAAATCACACAACCTTTGTATCATGGG

CN 119243 CCACAACCTTGTAACTTTTGTTGTGCAAATTGACAAATCACACAACCTTTGTATCATGGG

CN 120025 CCACAACCTTGTAACTTTTGTTGTGCAAATTGACAAATCACACAACCTTTGTATCATGGG

Joelle AAFC CCACAACCTTGTAACTTTTGTTGTGCAAATTGACAAATCACACAACCTTTGTATCATGGG

************************************************************

CAM 241 TCTTTTGTCATGAATATTGTCAGTAACAGAACCTTGTTTCTCTCTTGCCTCTAGGAAATG

17CS1133 TCTTTTGTCATGAATATTGTCAGTAACACAACCTTGTTTCTCTCTTGCCTCTAGGAAATG

CAM 236 TCTTTTGTCATGAATATTGTCAGTAACACAACCTTGTTTCTCTCTTGCCTCTAGGAAATG

Blaine Creek TCTTTTGTCATGAATATTGTCAGTAACACAACCTTGTTTCTCTCTTGCCTCTAGGAAATG

CN 119300 TCTTTTGTCATGAATATTGTCAGTAACACAACCTTGTTTCTCTCTTGCCTCTAGGAAATG

Yellowstone TCTTTTGTCATGAATATTGTCAGTAACACAACCTTGTTTCTCTCTTGCCTCTAGGAAATG

Hoga TCTTTTGTCATGAATATTGTCAGTAACACAACCTTGTTTCTCTCTTGCCTCTAGGAAATG

CN 120027 TCTTTTGTCATGAATATTGTCAGTAACACAACCTTGTTTCTCTCTTGCCTCTAGGAAATG

CN 120030 TCTTTTGTCATGAATATTGTCAGTAACACAACCTTGTTTCTCTCTTGCCTCTAGGAAATG

CN 119294 TCTTTTGTCATGAATATTGTCAGTAACACAACCTTGTTTCTCTCTTGCCTCTAGGAAATG

CN 120013 TCTTTTGTCATGAATATTGTCAGTAACACAACCTTGTTTCTCTCTTGCCTCTAGGAAATG

CN 120017 TCTTTTGTCATGAATATTGTCAGTAACACAACCTTGTTTCTCTCTTGCCTCTAGGAAATG

CN 119205 TCTTTTGTCATGAATATTGTCAGTAACACAACCTTGTTTCTCTCTTGCCTCTAGGAAATG

DH55 ref genome TCTTTTGTCATGAATATTGTCAGTAACACAACCTTGTTTCTCTCTTGCCTCTAGGAAATG

09-CS0040 TCTTTTGTCATGAATATTGTCAGTAACACAACCTTGTTTCTCTCTTGCCTCTAGGAAATG

CN 113754 TCTTTTGTCATGAATATTGTCAGTAACACAACCTTGTTTCTCTCTTGCCTCTAGGAAATG

CO46 NCBI TCTTTTGTCATGAATATTGTCAGTAACACAACCTTGTTTCTCTCTTGCCTCTAGGAAATG

Jasper TCTTTTGTCATGAATATTGTCAGTAACACAACCTTGTTTCTCTCTTGCCTCTAGGAAATG

Joelle phyto TCTTTTGTCATGAATATTGTCAGTAACACAACCTTGTTTCTCTCTTGCCTCTAGGAAATG

Joelle NCBI TCTTTTGTCATGAATATTGTCAGTAACACAACCTTGTTTCTCTCTTGCCTCTAGGAAATG

CN 119243 TCTTTTGTCATGAATATTGTCAGTAACACAACCTTGTTTCTCTCTTGCCTCTAGGAAATG

CN 120025 TCTTTTGTCATGAATATTGTCAGTAACACAACCTTGTTTCTCTCTTGCCTCTAGGAAATG

Joelle AAFC TCTTTTGTCATGAATATTGTCAGTAACACAACCTTGTTTCTCTCTTGCCTCTAGGAAATG

**************************** *******************************

CAM 241 TAAAACTCCAGAAAACTTGTCTTCATATAAGAAATATCAATATGATTCGACATTGTCAAA

17CS1133 TAAAACTCCAGAAAACTTGTCTTCATATAAGAAATATCAATATGATTCGACATTGTCAAA

CAM 236 TAAAACTCCAGAAAACTTGTCTTCATATAAGAAATATCAATATGATTCGACATTGTCAAA

Blaine Creek TAAAACTCCAGAAAACTTGTCTTCATATAAGAAATATCAATATGATTCGACATTGTCAAA

CN 119300 TAAAACTCCAGAAAACTTGTCTTCATATAAGAAATATCAATATGATTCGACATTGTCAAA

Yellowstone TAAAACTCCAGAAAACTTGTCTTCATATAAGAAATATCAATATGATTCGACATTGTCAAA

Hoga TAAAACTCCAGAAAACTTGTCTTCATATAAGAAATATCAATATGATTCGACATTGTCAAA

CN 120027 TAAAACTCCAGAAAACTTGTCTTCATATAAGAAATATCAATATGATTCGACATTGTCAAA

CN 120030 TAAAACTCCAGAAAACTTGTCTTCATATAAGAAATATCAATATGATTCGACATTGTCAAA

CN 119294 TAAAACTCCAGAAAACTTGTCTTCATATAAGAAATATCAATATGATTCGACATTGTCAAA

CN 120013 TAAAACTCCAGAAAACTTGTCTTCATATAAGAAATATCAATATGATTCGACATTGTCAAA

CN 120017 TAAAACTCCAGAAAACTTGTCTTCATATAAGAAATATCAATATGATTCGACATTGTCAAA

CN 119205 TAAAACTCCAGAAAACTTGTCTTCATATAAGAAATATCAATATGATTCGACATTGTCAAA

DH55 ref genome TAAAACTCCAGAAAACTTGTCTTCATATAAGAAATATCAATATGATTCGACATTGTCAAA

09-CS0040 TAAAACTCCAGAAAACTTGTCTTCATATAAGAAATATCAATATGATTCGACATTGTCAAA

CN 113754 TAAAACTCCAGAAAACTTGTCTTCATATAAGAAATATCAATATGATTCGACATTGTCAAA

CO46 NCBI TAAAACTCCAGAAAACTTGTCTTCATATAAGAAATATCAATATGATTCGACATTGTCAAA

Jasper TAAAACTCCAGAAAACTTGTCTTCATATAAGAAATATCAATATGATTCGACATTGTCAAA

Joelle phyto TAAAACTCCAGAAAACTTGTCTTCATATAAGAAATATCAATATGATTCGACATTGTCAAA

Joelle NCBI TAAAACTCCAGAAAACTTGTCTTCATATAAGAAATATCAATATGATTCGACATTGTCAAA

CN 119243 TAAAACTCCAGAAAACGTGTCTTCATATAAGAAATATCAATATGATTCGACATTGTCAAA

CN 120025 TAAAACTCCAGAAAACGTGTCTTCATATAAGAAATATCAATATGATTCGACATTGTCAAA

Joelle AAFC TAAAACTCCAGAAAACGTGTCTTCATATAAGAAATATCAATATGATTCGACATTGTCAAA

**************** *******************************************

CAM 241 AACAAACTCGGTGTAGTGTTTAGTACAACCCTCCAACATATTAACCAAGTGGTTGTAGTG

17CS1133 AACAAACTCGGTGTAGTGTTTAGTACAACCCTCCAACATATTAACCAAGTGGTTGTAGTG

CAM 236 AACAAACTCGGTGTAGTGTTTAGTACAACCCTCCAACATATTAACCAAGTGGTTGTAGTG

Blaine Creek AACAAACTCGGTGTAGTGTTTAGTACAACCCTCCAACATATTAACCAAGTGGTTGTAGTG

CN 119300 AACAAACTCGGTGTAGTGTTTAGTACAACCCTCCAACATATTAACCAAGTGGTTGTAGTG

Yellowstone AACAAACTCGGTGTAGTGTTTAGTACAACCCTCCAACATATTAACCAAGTGGTTGTAGTG

Hoga AACAAACTCGGTGTAGTGTTTAGTACAACCCTCCAACATATTAACCAAGTGGTTGTAGTG

CN 120027 AACAAACTCGGTGTAGTGTTTAGTACAACCCTCCAACATATTAACCAAGTGGTTGTAGTG

CN 120030 AACAAACTCGGTGTAGTGTTTAGTACAACCCTCCAACATATTAACCAAGTGGTTGTAGTG

CN 119294 AACAAACTCGGTGTAGTGTTTAGTACAACCCTCCAACATATTAACCAAGTGGTTGTAGTG

CN 120013 AACAAACTCGGTGTAGTGTTTAGTACAACCCTCCAACATATTAACCAAGTGGTTGTAGTG

CN 120017 AACAAACTCGGTGTAGTGTTTAGTACAACCCTCCAACATATTAACCAAGTGGTTGTAGTG

CN 119205 AACAAACTCGGTGTAGTGTTTAGTACAACCCTCCAACATATTAACCAAGTGGTTGTAGTG

DH55 ref genome AACAAACTCGGTGTAGTGTTTAGTACAACCCTCCAACATATTAACCAAGTGGTTGTAGTG

09-CS0040 AACAAACTCGGTGTAGTGTTTAGTACAACCCTCCAACATATTAACCAAGTGGTTGTAGTG

CN 113754 AACAAACTCGGTGTAGTGTTTAGTACAACCCTCCAACATATTAACCAAGTGGTTGTAGTG

CO46 NCBI AACAAACTCGGTGTAGTGTTTAGTACAACCCTCCAACATATTAACCAAGTGGTTGTAGTG

Jasper AACAAACTCGGTGTAGTGTTTAGTACAACCCTCCAACATATTAACCAAGTGGTTGTAGTG

Joelle phyto AACAAACTCGGTGTAGTGTTTAGTACAACCCTCCAACATATTAACCAAGTGGTTGTAGTG

Joelle NCBI AACAAACTCGGTGTAGTGTTTAGTACAACCCTCCAACATATTAACCAAGTGGTTGTAGTG

CN 119243 ATCAAACTCGGTGTAGTGTTTAGTACAACCCTCCAACATATTAACCAAGTGGTTGTAGTG

CN 120025 ATCAAACTCGGTGTAGTGTTTAGTACAACCCTCCAACATATTAACCAAGTCGTTGTAGTG

Joelle AAFC ATCAAACTCGGTGTAGTGTTTAGTACAACCCTCCAACATATTAACCAAGTGGTTGTAGTG

* ************************************************ *********

CAM 241 GTTTAGCCATGTTGGTCAAGATCGTAGGCCGATTCTCTCACTTAATGCATACTTTGTTAG

17CS1133 GTTTAGCCATGTTGGTCAAGATCGTAGGCCGATTCTCTCACTTAATGCATACTTTGTTAG

CAM 236 GTTTAGCCATGTTGGTCAAGATCGTAGGCCGATTCTCTCACTTAATGCATACTTTGTTAG

Blaine Creek GTTTAGCCATGTTGGTCAAGATCGTAGGCCGATTCTCTCACTTAATGCATACTTTGTTAG

CN 119300 GTTTAGCCATGTTGGTCAAGATCGTAGGCCGATTCTCTCACTTAATGCATACTTTGTTAG

Yellowstone GTTTAGCCATGTTGGTCAAGATCGTAGGCCGATTCTCTCACTTAATGCATACTTTGTTAG

Hoga GTTTAGCCATGTTGGTCAAGATCGTAGGCCGATTCTCTCACTTAATGCATACTTTGTTAG

CN 120027 GTTTAGCCATGTTGGTCAAGATCGTAGGCCGATTCTCTCACTTAATGCATACTTTGTTAG

CN 120030 GTTTAGCCATGTTGGTCAAGATCGTAGGCCGATTCTCTCACTTAATGCATACTTTGTTAG

CN 119294 GTTTAGCCATGTTGGTCAAGATCGTAGGCCGATTCTCTCACTTAATGCATACTTTGTTAG

CN 120013 GTTTAGCCATGTTGGTCAAGATCGTAGGCCGATTCTCTCACTTAATGCATACTTTGTTAG

CN 120017 GTTTAGCCATGTTGGTCAAGATCGTAGGCCGATTCTCTCACTTAATGCATACTTTGTTAG

CN 119205 GTTTAGCCATGTTGGTCAAGATCGTAGGCCGATTCTCTCACTTAATGCATACTTTGTTAG

DH55 ref genome GTTTAGCCATGTTGGTCAAGATCGTAGGCCGATTCTCTCACTTAATGCATACTTTGTTAG

09-CS0040 GTTTAGCCATGTTGGTCAAGATCGTAGGCCGATTCTCTCACTTAATGCATACTTTGTTAG

CN 113754 GTTTAGCCATGTTGGTCAAGATCGTAGGCCGATTCTCTCACTTAATGCATACTTTGTTAG

CO46 NCBI GTTTAGCCATGTTGGTCAAGATCGTAGGCCGATTCTCTCACTTAATGCATACTTTGTTAG

Jasper GTTTAGCCATGTTGGTCAAGATCGTAGGCCGATTCTCTCACTTAATGCATACTTTGTTAG

Joelle phyto GTTTAGCCATGTTGGTCAAGATCGTAGGCCGATTCTCTCACTTAATGCATACTTTGTTAG

Joelle NCBI GTTTAGCCATGTTGGTCAAGATCGTAGGCCGATTCTCTCACTTAATGCATACTTTGTTAG

CN 119243 GTTTAGCCATGTTGGTCAAGATCGTAGGCCGATTCTCTCACTTGCTGCATACTTTGTTAG

CN 120025 GTTTAGCCATGTTGGTCAAGATCGTAGGCCGATTCTCTCACTTGCTGCATACTTTGTTAG

Joelle AAFC GTTTAGCCATGTTGGTCAAGATCGTAGGCCGATTCTCTCACTTGCTGCATACTTTGTTAG

******************************************* ***************

CAM 241 GGTTTGTTCACGCCTAATACTGAATGTTAGGTCCAGCCTTGAAATAGCCGAGACACCTGA

17CS1133 GGTTTGTTCACGCCTAATACTGAATGTTAGGTCCAGCCTTGAAATAGCCGAGACACCTGA

CAM 236 GGTTTGTTCACGCCTAATACTGAATGTTAGGTCCAGCCTTGAAATAGCCGAGACACCTGA

Blaine Creek GGTTTGTTCACGCCTAATACTGAATGTTAGGTCCAGCCTTGAAATAGCCGAGACACCTGA

CN 119300 GGTTTGTTCACGCCTAATACTGAATGTTAGGTCCAGCCTTGAAATAGCCGAGACACCTGA

Yellowstone GGTTTGTTCACGCCTAATACTGAATGTTAGGTCCAGCCTTGAAATAGCCGAGACACCTGA

Hoga GGTTTGTTCACGCCTAATACTGAATGTTAGGTCCAGCCTTGAAATAGCCGAGACACCTGA

CN 120027 GGTTTGTTCACGCCTAATACTGAATGTTAGGTCCAGCCTTGAAATAGCCGAGACACCTGA

CN 120030 GGTTTGTTCACGCCTAATACTGAATGTTAGGTCCAGCCTTGAAATAGCCGAGACACCTGA

CN 119294 GGTTTGTTCACGCCTAATACTGAATGTTAGGTCCAGCCTTGAAATAGCCGAGACACCTGA

CN 120013 GGTTTGTTCACGCCTAATACTGAATGTTAGGTCCAGCCTTGAAATAGCCGAGACACCTGA

CN 120017 GGTTTGTTCACGCCTAATACTGAATGTTAGGTCCAGCCTTGAAATAGCCGAGACACCTGA

CN 119205 GGTTTGTTCACGCCTAATACTGAATGTTAGGTCCAGCCTTGAAATAGCCGAGACACCTGA

DH55 ref genome GGTTTGTTCACGCCTAATACTGAATGTTAGGTCCAGCCTTGAAATAGCCGAGACACCTGA

09-CS0040 GGTTTGTTCACGCCTAATACTGAATGTTAGGTCCAGCCTTGAAATAGCCGAGACACCTGA

CN 113754 GGTTTGTTCACGCCTAATACTGAATGTTAGGTCCAGCCTTGAAATAGCCGAGACACCTGA

CO46 NCBI GGTTTGTTCACGCCTAATACTGAATGTTAGGTCCAGCCTTGAAATAGCCGAGACACCTGA

Jasper GGTTTGTTCACGCCTAATACTGAATGTTAGGTCCAGCCTTGAAATAGCCGAGACACCTGA

Joelle phyto GGTTTGTTCACGCCTAATACTGAATGTTAGGTCCAGCCTTGAAATAGCCGAGACACCTGA

Joelle NCBI GGTTTGTTCACGCCTAATACTGAATGTTAGGTCCAGCCTTGAAATAGCCGAGACACCTGA

CN 119243 GGTTTGTTCACGCCTAATACTGAATGTTAGGTCCAGCCTTGAAATAGCCGAGACACCTGA

CN 120025 GGTTTGTTCACGCCTAATACTGAATGTTAGGTCCAGCCTTGAAATAGCCGAGACACCTGA

Joelle AAFC GGTTTGTTCACGCCTAATACTGAATGTTAGGTCCAGCCTTGAAATAGCCGAGACACCTGA

************************************************************

CAM 241 CTAATTATGAGTTAAAAAGAAACGTTATCTACTCAATAACTCATTTTTGCATCCTTAATT

17CS1133 CTAATTATGAGTTAAAAAGAAACGTTATCTACTCAATAACTCATTTTTGCATCCTTAATT

CAM 236 CTAATTATGAGTTAAAAAGAAACGTTATCTACTCAATAACTCATTTTTGCATCCTTAATT

Blaine Creek CTAATTATGAGTTAAAAAGAAACGTTATCTACTCAATAACTCATTTTTGCATCCTTAATT

CN 119300 CTAATTATGAGTTAAAAAGAAACGTTATCTACTCAATAACTCATTTTTGCATCCTTAATT

Yellowstone CTAATTATGAGTTAAAAAGAAACGTTATCTACTCAATAACTCATTTTTGCATCCTTAATT

Hoga CTAATTATGAGTTAAAAAGAAACGTTATCTACTCAATAACTCATTTTTGCATCCTTAATT

CN 120027 CTAATTATGAGTTAAAAAGAAACGTTATCTACTCAATAACTCATTTTTGCATCCTTAATT

CN 120030 CTAATTATGAGTTAAAAAGAAACGTTATCTACTCAATAACTCATTTTTGCATCCTTAATT

CN 119294 CTAATTATGAGTTAAAAAGAAACGTTATCTACTCAATAACTCATTTTTGCATCCTTAATT

CN 120013 CTAATTATGAGTTAAAAAGAAACGTTATCTACTCAATAACTCATTTTTGCATCCTTAATT

CN 120017 CTAATTATGAGTTAAAAAGAAACGTTATCTACTCAATAACTCATTTTTGCATCCTTAATT

CN 119205 CTAATTATGAGTTAAAAAGAAACGTTATCTACTCAATAACTCATTTTTGCATCCTTAATT

DH55 ref genome CTAATTATGAGTTAAAAAGAAACGTTATCTACTCAATAACTCATTTTTGCATCCTTAATT

09-CS0040 CTAATTATGAGTTAAAAAGAAACGTTATCTACTCAATAACTCATTTTTGCATCCTTAATT

CN 113754 CTAATTATGAGTTAAAAAGAAACGTTATCTACTCAATAACTCATTTTTGCATCCTTAATT

CO46 NCBI CTAATTATGAGTTAAAAAGAAACGTTATCTACTCAATAACTCATTTTTGCATCCTTAATT

Jasper CTAATTATGAGTTAAAAAGAAACGTTATCTACTCAATAACTCATTTTTGCATCCTTAATT

Joelle phyto CTAATTATGAGTTAAAAAGAAACGTTATCTACTCAATAACTCATTTTTGCATCCTTAATT

Joelle NCBI CTAATTATGAGTTAAAAAGAAACGTTATCTACTCAATAACTCATTTTTGCATCCTTAATT

CN 119243 CTAATTATGAGTTAAAAAGAAACGTTATCTACTCAATAACTCATTTTTGCATCCTTAATT

CN 120025 CTAATTATGAGTTAAAAAGAAACGTTATCTACTCAATAACTCATTTTTGCATCCTTAATT

Joelle AAFC CTAATTATGAGTTAAAAAGAAACGTTATCTACTCAATAACTCATTTTTGCATCCTTAATT

************************************************************

CAM 241 TTGGTGCAAAGAGCTTAACTTCACAATGGAACTGAAACCCATTCACACAAATTATTAAGT

17CS1133 TTGGTGCAAAGAGCTTAACTTCACAATGGAACTGAAACCCATTCACACAAATTATTAAGT

CAM 236 TTGGTGCAAAGAGCTTAACTTCACAATGGAACTGAAACCCATTCACACAAATTATTAAGT

Blaine Creek TTGGTGCAAAGAGCTTAACTTCACAATGGAACTGAAACCCATTCACACAAATTATTAAGT

CN 119300 TTGGTGCAAAGAGCTTAACTTCACAATGGAACTGAAACCCATTCACACAAATTATTAAGT

Yellowstone TTGGTGCAAAGAGCTTAACTTCACAATGGAACTGAAACCCATTCACACAAATTATTAAGT

Hoga TTGGTGCAAAGAGCTTAACTTCACAATGGAACTGAAACCCATTCACACAAATTATTAAGT

CN 120027 TTGGTGCAAAGAGCTTAACTTCACAATGGAACTGAAACCCATTCACACAAATTATTAAGT

CN 120030 TTGGTGCAAAGAGCTTAACTTCACAATGGAACTGAAACCCATTCACACAAATTATTAAGT

CN 119294 TTGGTGCAAAGAGCTTAACTTCACAATGGAACTGAAACCCATTCACACAAATTATTAAGT

CN 120013 TTGGTGCAAAGAGCTTAACTTCACAATGGAACTGAAACCCATTCACACAAATTATTAAGT

CN 120017 TTGGTGCAAAGAGCTTAACTTCACAATGGAACTGAAACCCATTCACACAAATTATTAAGT

CN 119205 TTGGTGCAAAGAGCTTAACTTCACAATGGAACTGAAACCCATTCACACAAATTATTAAGT

DH55 ref genome TTGGTGCAAAGAGCTTAACTTCACAATGGAACTGAAACCCATTCACACAAATTATTAAGT

09-CS0040 TTGGTGCAAAGAGCTTAACTTCACAATGGAACTGAAACCCATTCACACAAATTATTAAGT

CN 113754 TTGGTGCAAAGAGCTTAACTTCACAATGGAACTGAAACCCATTCACACAAATTATTAAGT

CO46 NCBI TTGGTGCAAAGAGCTTAACTTCACAATGGAACTGAAACCCATTCACACAAATTATTAAGT

Jasper TTGGTGCAAAGAGCTTAACTTCACAATGGAACTGAAACCCATTCACACAAATTATTAAGT

Joelle phyto TTGGTGCAAAGAGCTTAACTTCACAATGGAACTGAAACCCATTCACACAAATTATTAAGT

Joelle NCBI TTGGTGCAAAGAGCTTAACTTCACAATGGAACTGAAACCCATTCACACAAATTATTAAGT

CN 119243 TTGGTGCAAAGAGCTTAACTTCACAATGGAACTGAAACCCATTCACACAAATTATTAAGT

CN 120025 TTGGTGCAAAGAGCTTAACTTCACAATGGAACTGAAACCCATTCACACAAATTATTAAGT

Joelle AAFC TTGGTGCAAAGAGCTTAACTTCACAATGGAACTGAAACCCATTCACACAAATTATTAAGT

************************************************************

CAM 241 GAATTAGTGGTAGTTTTGTCAAATTTGCTTGACCTTTATTAGGTAATATGTATAGTTTTA

17CS1133 GAATTAGTGGTAGTTTTGTCAAATTTGCTTGACCTTTATTAGGTAATATGTATAGTTTTA

CAM 236 GAATTAGTGGTAGTTTTGTCAAATTTGCTTGACCTTTATTAGGTAATATGTATAGTTTTA

Blaine Creek GAATTAGTGGTAGTTTTGTCAAATTTGCTTGACCTTTATTAGGTAATATGTATAGTTTTA

CN 119300 GAATTAGTGGTAGTTTTGTCAAATTTGCTTGACCTTTATTAGGTAATATGTATAGTTTTA

Yellowstone GAATTAGTGGTAGTTTTGTCAAATTTGCTTGACCTTTATTAGGTAATATGTATAGTTTTA

Hoga GAATTAGTGGTAGTTTTGTCAAATTTGCTTGACCTTTATTAGGTAATATGTATAGTTTTA

CN 120027 GAATTAGTGGTAGTTTTGTCAAATTTGCTTGACCTTTATTAGGTAATATGTATAGTTTTA

CN 120030 GAATTAGTGGTAGTTTTGTCAAATTTGCTTGACCTTTATTAGGTAATATGTATAGTTTTA

CN 119294 GAATTAGTGGTAGTTTTGTCAAATTTGCTTGACCTTTATTAGGTAATATGTATAGTTTTA

CN 120013 GAATTAGTGGTAGTTTTGTCAAATTTGCTTGACCTTTATTAGGTAATATGTATAGTTTTA

CN 120017 GAATTAGTGGTAGTTTTGTCAAATTTGCTTGACCTTTATTAGGTAATATGTATAGTTTTA

CN 119205 GAATTAGTGGTAGTTTTGTCAAATTTGCTTGACCTTTATTAGGTAATATGTATAGTTTTA

DH55 ref genome GAATTAGTGGTAGTTTTGTCAAATTTGCTTGACCTTTATTAGGTAATATGTATAGTTTTA

09-CS0040 GAATTAGTGGTAGTTTTGTCAAATTTGCTTGACCTTTATTAGGTAATATGTATAGTTTTA

CN 113754 GAATTAGTGGTAGTTTTGTCAAATTTGCTTGACCTTTATTAGGTAATATGTATAGTTTTA

CO46 NCBI GAATTAGTGGTAGTTTTGTCAAATTTGCTTGACCTTTATTAGGTAATATGTATAGTTTTA

Jasper GAATTAGTGGTAGTTTTGTCAAATTTGCTTGACCTTTATTAGGTAATATGTATAGTTTTA

Joelle phyto GAATTAGTGGTAGTTTTGTCAAATTTGCTTGACCTTTATTAGGTAATATGTATAGTTTTA

Joelle NCBI GAATTAGTGGTAGTTTTGTCAAATTTGCTTGACCTTTATTAGGTAATATGTATAGTTTTA

CN 119243 GAATTAGTGGTAGTTTTGTCAAATTTGCTTGACCTTTATTAGGTAATATGTATAGTTTTA

CN 120025 GAATTAGTGGTAGTTTTGTCAAATTTGCTTGACCTTTATTAGGTAATATGTATAGTTTTA

Joelle AAFC GAATTAGTGGTAGTTTTGTCAAATTTGCTTGACCTTTATTAGGTAATATGTATAGTTTTA

************************************************************

CAM 241 GTTTTATCTGTTTTAGGTTTTTCATTCTTTGGAACAATTTATCTATATATTGGTGATACT

17CS1133 GTTTTATCTGTTTTAGGTTTTTCATTCTTTGGAACAATTTATCTATATATTGGTGATACT

CAM 236 GTTTTATCTGTTTTAGGTTTTTCATTCTTTGGAACAATTTATCTATATATTGGTGATACT

Blaine Creek GTTTTATCTGTTTTAGGTTTTTCATTCTTTGGAACAATTTATCTATATATTGGTGATACT

CN 119300 GTTTTATCTGTTTTAGGTTTTTCATTCTTTGGAACAATTTATCTATATATTGGTGATACT

Yellowstone GTTTTATCTGTTTTAGGTTTTTCATTCTTTGGAACAATTTATCTATATATTGGTGATACT

Hoga GTTTTATCTGTTTTAGGTTTTTCATTCTTTGGAACAATTTATCTATATATTGGTGATACT

CN 120027 GTTTTATCTGTTTTAGGTTTTTCATTCTTTGGAACAATTTATCTATATATTGGTGATACT

CN 120030 GTTTTATCTGTTTTAGGTTTTTCATTCTTTGGAACAATTTATCTATATATTGGTGATACT

CN 119294 GTTTTATCTGTTTTAGGTTTTTCATTCTTTGGAACAATTTATCTATATATTGGTGATACT

CN 120013 GTTTTATCTGTTTTAGGTTTTTCATTCTTTGGAACAATTTATCTATATATTGGTGATACT

CN 120017 GTTTTATCTGTTTTAGGTTTTTCATTCTTTGGAACAATTTATCTATATATTGGTGATACT

CN 119205 GTTTTATCTGTTTTAGGTTTTTCATTCTTTGGAACAATTTATCTATATATTGGTGATACT

DH55 ref genome GTTTTATCTGTTTTAGGTTTTTCATTCTTTGGAACAATTTATCTATATATTGGTGATACT

09-CS0040 GTTTTATCTGTTTTAGGTTTTTCATTCTTTGGAACAATTTATCTATATATTGGTGATACT

CN 113754 GTTTTATCTGTTTTAGGTTTTTCATTCTTTGGAACAATTTATCTATATATTGGTGATACT

CO46 NCBI GTTTTATCTGTTTTAGGTTTTTCATTCTTTGGAACAATTTATCTATATATTGGTGATACT

Jasper GTTTTATCTGTTTTAGGTTTTTCATTCTTTGGAACAATTTATCTATATATTGGTGATACT

Joelle phyto GTTTTATCTGTTTTAGGTTTTTCATTCTTTGGAACAATTTATCTATATATTGGTGATACT

Joelle NCBI GTTTTATCTGTTTTAGGTTTTTCATTCTTTGGAACAATTTATCTATATATTGGTGATACT

CN 119243 GTTTTATCTGTTTTAGGTTTTTCATTCTTTGGAACAATTTATCTATATATTGGTGATACT

CN 120025 GTTTTATCTGTTTTAGGTTTTTCATTCTTTGGAACAATTTATCTATATATTGGTGATACA

Joelle AAFC GTTTTATCTGTTTTAGGTTTTTCATTCTTTGGAACAATTTATCTATATATTGGTGATACA

***********************************************************

CAM 241 TGATAGATAGATGCCTCAGTAAGTTGATGTAATTTCAATGGTGTAAACTCTTCACCTTTA

17CS1133 TGATAGATAGATGCCTCAGTAAGTTGATGTAATTTCAATGGTGTAAACTCTTCACCTTTA

CAM 236 TGATAGATAGATGCCTCAGTAAGTTGATGTAATTTCAATGGTGTAAACTCTTCACCTTTA

Blaine Creek TGATAGATAGATGCCTCAGTAAGTTGATGTAATTTCAATGGTGTAAACTCTTCACCTTTA

CN 119300 TGATAGATAGATGCCTCAGTAAGTTGATGTAATTTCAATGGTGTAAACTCTTCACCTTTA

Yellowstone TGATAGATAGATGCCTCAGTAAGTTGATGTAATTTCAATGGTGTAAACTCTTCACCTTTA

Hoga TGATAGATAGATGCCTCAGTAAGTTGATGTAATTTCAATGGTGTAAACTCTTCACCTTTA

CN 120027 TGATAGATAGATGCCTCAGTAAGTTGATGTAATTTCAATGGTGTAAACTCTTCACCTTTA

CN 120030 TGATAGATAGATGCCTCAGTAAGTTGATGTAATTTCAATGGTGTAAACTCTTCACCTTTA

CN 119294 TGATAGATAGATGCCTCAGTAAGTTGATGTAATTTCAATGGTGTAAACTCTTCACCTTTA

CN 120013 TGATAGATAGATGCCTCAGTAAGTTGATGTAATTTCAATGGTGTAAACTCTTCACCTTTA

CN 120017 TGATAGATAGATGCCTCAGTAAGTTGATGTAATTTCAATGGTGTAAACTCTTCACCTTTA

CN 119205 TGATAGATAGATGCCTCAGTAAGTTGATGTAATTTCAATGGTGTAAACTCTTCACCTTTA

DH55 ref genome TGATAGATAGATGCCTCAGTAAGTTGATGTAATTTCAATGGTGTAAACTCTTCACCTTTA

09-CS0040 TGATAGATAGATGCCTCAGTAAGTTGATGTAATTTCAATGGTGTAAACTCTTCACCTTTA

CN 113754 TGATAGATAGATGCCTCAGTAAGTTGATGTAATTTCAATGGTGTAAACTCTTCACCTTTA

CO46 NCBI TGATAGATAGATGCCTCAGTAAGTTGATGTAATTTCAATGGTGTAAACTCTTCACCTTTA

Jasper TGATAGATAGATGCCTCAGTAAGTTGATGTAATTTCAATGGTGTAAACTCTTCACCTTTA

Joelle phyto TGATAGATAGATGCCTCAGTAAGTTGATGTAATTTCAATGGTGTAAACTCTTCACCTTTA

Joelle NCBI TGATAGATAGATGCCTCAGTAAGTTGATGTAATTTCAATGGTGTAAACTCTTCACCTTTA

CN 119243 TGATAGATAGATGCCTCAGTAAGTTGATGTAATTTCAATGGTGTAAACTCTTCACCTTTA

CN 120025 TGATAGATAGATGCCTCAGTAAGTTGATGTAATTTCAGTGGTGTAAACTCTTCACCTTTA

Joelle AAFC TGATAGATAGATGCCTCAGTAAGTTGATGTAATTTCAGTGGTGTAAACTCTTCACCTTTA

************************************* **********************

CAM 241 CTGCTTCATTTTTGGCTTTCAATATAGTTAATTGGAACCTCAACATTTTCTATATATACA

17CS1133 CTGCTTCATTTTTGGCTTTCAATATAGTTAATTGGAACCTCAACATTTTCTATATATACA

CAM 236 CTGCTTCATTTTTGGCTTTCAATATAGTTAATTGGAACCTCAACATTTTCTATATATACA

Blaine Creek CTGCTTCATTTTTGGCTTTCAATATAGTTAATTGGAACCTCAACATTTTCTATATATACA

CN 119300 CTGCTTCATTTTTGGCTTTCAATATAGTTAATTGGAACCTCAACATTTTCTATATATACA

Yellowstone CTGCTTCATTTTTGGCTTTCAATATAGTTAATTGGAACCTCAACATTTTCTATATATACA

Hoga CTGCTTCATTTTTGGCTTTCAATATAGTTAATTGGAACCTCAACATTTTCTATATATACA

CN 120027 CTGCTTCATTTTTGGCTTTCAATATAGTTAATTGGAACCTCAACATTTTCTATATATACA

CN 120030 CTGCTTCATTTTTGGCTTTCAATATAGTTAATTGGAACCTCAACATTTTCTATATATACA

CN 119294 CTGCTTCATTTTTGGCTTTCAATATAGTTAATTGGAACCTCAACATTTTCTATATATACA

CN 120013 CTGCTTCATTTTTGGCTTTCAATATAGTTAATTGGAACCTCAACATTTTCTATATATACA

CN 120017 CTGCTTCATTTTTGGCTTTCAATATAGTTAATTGGAACCTCAACATTTTCTATATATACA

CN 119205 CTGCTTCATTTTTGGCTTTCAATATAGTTAATTGGAACCTCAACATTTTCTATATATACA

DH55 ref genome CTGCTTCATTTTTGGCTTTCAATATAGTTAATTGGAACCTCAACATTTTCTATATATACA

09-CS0040 CTGCTTCATTTTTGGCTTTCAATATAGTTAATTGGAACCTCAACATTTTCTATATATACA

CN 113754 CTGCTTCATTTTTGGCTTTCAATATAGTTAATTGGAACCTCAACATTTTCTATATATACA

CO46 NCBI CTGCTTCATTTTTGGCTTTCAATATAGTTAATTGGAACCTCAACATTTTCTATATATACA

Jasper CTGCTTCATTTTTGGCTTTCAATATAGTTAATTGGAACCTCAACATTTTCTATATATACA

Joelle phyto CTGCTTCATTTTTGGCTTTCAATATAGTTAATTGGAACCTCAACATTTTCTATATATACA

Joelle NCBI CTGCTTCATTTTTGGCTTTCAATATAGTTAATTGGAACCTCAACATTTTCTATATATACA

CN 119243 CTGCTTCATTTTTGGCTTTCAATATAGTTAATTGGAACCTCAACATTTTCTATATATACA

CN 120025 CTGCTTCATTTTTGGCTTTCAATATAGTTAATTGGAACCTCAACATTTTCTATATATACA

Joelle AAFC CTGCTTCATTTTTGGCTTTCAATATAGTTAATTGGAACCTCAACATTTTCTATATATACA

************************************************************

CAM 241 AACGAGGAAGAAAATGGAAGACCGGTTTCCTATTCTTAGGAAAGTGTTTCCATTTTCGTT

17CS1133 AACGAGGAAGAAAATGGAAGACCGGTTTCCTATTCTTAGGAAAGTGTTTCCATTTTCGTT

CAM 236 AACGAGGAAGAAAATGGAAGACCGGTTTCCTATTCTTAGGAAAGTGTTTCCATTTTCGTT

Blaine Creek AACGAGGAAGAAAATGGAAGACCGGTTTCCTATTCTTAGGAAAGTGTTTCCATTTTCGTT

CN 119300 AACGAGGAAGAAAATGGAAGACCGGTTTCCTATTCTTAGGAAAGTGTTTCCATTTTCGTT

Yellowstone AACGAGGAAGAAAATGGAAGACCGGTTTCCTATTCTTAGGAAAGTGTTTCCATTTTCGTT

Hoga AACGAGGAAGAAAATGGAAGACCGGTTTCCTATTCTTAGGAAAGTGTTTCCATTTTCGTT

CN 120027 AACGAGGAAGAAAATGGAAGACCGGTTTCCTATTCTTAGGAAAGTGTTTCCATTTTCGTT

CN 120030 AACGAGGAAGAAAATGGAAGACCGGTTTCCTATTCTTAGGAAAGTGTTTCCATTTTCGTT

CN 119294 AACGAGGAAGAAAATGGAAGACCGGTTTCCTATTCTTAGGAAAGTGTTTCCATTTTCGTT

CN 120013 AACGAGGAAGAAAATGGAAGACCGGTTTCCTATTCTTAGGAAAGTGTTTCCATTTTCGTT

CN 120017 AACGAGGAAGAAAATGGAAGACCGGTTTCCTATTCTTAGGAAAGTGTTTCCATTTTCGTT

CN 119205 AACGAGGAAGAAAATGGAAGACCGGTTTCCTATTCTTAGGAAAGTGTTTCCATTTTCGTT

DH55 ref genome AACGAGGAAGAAAATGGAAGACCGGTTTCCTATTCTTAGGAAAGTGTTTCCATTTTCGTT

09-CS0040 AACGAGGAAGAAAATGGAAGACCGGTTTCCTATTCTTAGGAAAGTGTTTCCATTTTCGTT

CN 113754 AACGAGGAAGAAAATGGAAGACCGGTTTCCTATTCTTAGGAAAGTGTTTCCATTTTCGTT

CO46 NCBI AACGAGGAAGAAAATGGAAGACCGGTTTCCTATTCTTAGGAAAGTGTTTCCATTTTCGTT

Jasper AACGAGGAAGAAAATGGAAGACCGGTTTCCTATTCTTAGGAAAGTGTTTCCATTTTCGTT

Joelle phyto AACGAGGAAGAAAATGGAAGACCGGTTTCCTATTCTTAGGAAAGTGTTTCCATTTTCGTT

Joelle NCBI AACGAGGAAGAAAATGGAAGACCGGTTTCCTATTCTTAGGAAAGTGTTTCCATTTTCGTT

CN 119243 AACGAGGAAGAAAATGGAAGACCGGTTTCCTATTCTTAGGAAAGTGTTTCCATTTTCGTT

CN 120025 AACGAGGAAGAAAATGGAAGACCGGTTTCCTATTCTTAGGAAAGTGTTTCCATTTTCGTT

Joelle AAFC AACGAGGAAGAAAATGGAAGACCGGTTTCCTATTCTTAGGAAAGTGTTTCCATTTTCGTT

************************************************************

CAM 241 GCAAAGAAACATAAAATAAAATTATGAGATTGTTGTATATATAAAGACTCGGTCAATGTA

17CS1133 GCAAAGAAACATAAAATAAAATTATGAGATTGTTGTATATATAAAGACTCGGTCAATGTA

CAM 236 GCAAAGAAACATAAAATAAAATTATGAGATTGTTGTATATATAAAGACTCGGTCAATGTA

Blaine Creek GCAAAGAAACATAAAATAAAATTATGAGATTGTTGTATATATAAAGACTCGGTCAATGTA

CN 119300 GCAAAGAAACATAAAATAAAATTATGAGATTGTTGTATATATAAAGACTCGGTCAATGTA

Yellowstone GCAAAGAAACATAAAATAAAATTATGAGATTGTTGTATATATAAAGACTCGGTCAATGTA

Hoga GCAAAGAAACATAAAATAAAATTATGAGATTGTTGTATATATAAAGACTCGGTCAATGTA

CN 120027 GCAAAGAAACATAAAATAAAATTATGAGATTGTTGTATATATAAAGACTCGGTCAATGTA

CN 120030 GCAAAGAAACATAAAATAAAATTATGAGATTGTTGTATATATAAAGACTCGGTCAATGTA

CN 119294 GCAAAGAAACATAAAATAAAATTATGAGATTGTTGTATATATAAAGACTCGGTCAATGTA

CN 120013 GCAAAGAAACATAAAATAAAATTATGAGATTGTTGTATATATAAAGACTCGGTCAATGTA

CN 120017 GCAAAGAAACATAAAATAAAATTATGAGATTGTTGTATATATAAAGACTCGGTCAATGTA

CN 119205 GCAAAGAAACATAAAATAAAATTATGAGATTGTTGTATATATAAAGACTCGGTCAATGTA

DH55 ref genome GCAAAGAAACATAAAATAAAATTATGAGATTGTTGTATATATAAAGACTCGGTCAATGTA

09-CS0040 GCAAAGAAACATAAAATAAAATTATGAGATTGTTGTATATATAAAGACTCGGTCAATGTA

CN 113754 GCAAAGAAACATAAAATAAAATTATGAGATTGTTGTATATATAAAGACTCGGTCAATGTA

CO46 NCBI GCAAAGAAACATAAAATAAAATTATGAGATTGTTGTATATATAAAGACTCGGTCAATGTA

Jasper GCAAAGAAACATAAAATAAAATTATGAGATTGTTGTATATATAAAGACTCGGTCAATGTA

Joelle phyto GCAAAGAAACATAAAATAAAATTATGAGATTGTTGTATATATAAAGACTCGGTCAATGTA

Joelle NCBI GCAAAGAAACATAAAATAAAATTATGAGATTGTTGTATATATAAAGACTCGGTCAATGTA

CN 119243 GCAAAGAAACATAAAATAAAATTATGAGATTGTTGTATATATAAAGACTCGGTCAATGTA

CN 120025 GCAAAGAAACATAAAATAAAATTATGAGATTGTTGTATATATAAAGACTCGGTCAATGTA

Joelle AAFC GCAAAGAAACATAAAATAAAATTATGAGATTGTTGTATATATAAAGACTCGGTCAATGTA

************************************************************

CAM 241 TTTGGAGTCTGGATTTACGTATTGGTCATCTGCTTACTGGCCACATAATCATCATCATGT

17CS1133 TTTGGAGTCTGGATTTACGTATTGGTCATCTGCTTACTGGCCACATAATCATCATCATGT

CAM 236 TTTGGAGTCTGGATTTACGTATTGGTCATCTGCTTACTGGCCACATAATCATCATCATGT

Blaine Creek TTTGGAGTCTGGATTTACGTATTGGTCATCTGCTTACTGGCCACATAATCATCATCATGT

CN 119300 TTTGGAGTCTGGATTTACGTATTGGTCATCTGCTTACTGGCCACATAATCATCATCATGT

Yellowstone TTTGGAGTCTGGATTTACGTATTGGTCATCTGCTTACTGGCCACATAATCATCATCATGT

Hoga TTTGGAGTCTGGATTTACGTATTGGTCATCTGCTTACTGGCCACATAATCATCATCATGT

CN 120027 TTTGGAGTCTGGATTTACGTATTGGTCATCTGCTTACTGGCCACATAATCATCATCATGT

CN 120030 TTTGGAGTCTGGATTTACGTATTGGTCATCTGCTTACTGGCCACATAATCATCATCATGT

CN 119294 TTTGGAGTCTGGATTTACGTATTGGTCATCTGCTTACTGGCCACATAATCATCATCATGT

CN 120013 TTTGGAGTCTGGATTTACGTATTGGTCATCTGCTTACTGGCCACATAATCATCATCATGT

CN 120017 TTTGGAGTCTGGATTTACGTATTGGTCATCTGCTTACTGGCCACATAATCATCATCATGT

CN 119205 TTTGGAGTCTGGATTTACGTATTGGTCATCTGCTTACTGGCCACATAATCATCATCATGT

DH55 ref genome TTTGGAGTCTGGATTTACGTATTGGTCATCTGCTTACTGGCCACATAATCATCATCATGT

09-CS0040 TTTGGAGTCTGGATTTACGTATTGGTCATCTGCTTACTGGCCACATAATCATCATCATGT

CN 113754 TTTGGAGTCTGGATTTACGTATTGGTCATCTGCTTACTGGCCACATAATCATCATCATGT

CO46 NCBI TTTGGAGTCTGGATTTACGTATTGGTCATCTGCTTACTGGCCACATAATCATCATCATGT

Jasper TTTGGAGTCTGGATTTACGTATTGGTCATCTGCTTACTGGCCACATAATCATCATCATGT

Joelle phyto TTTGGAGTCTGGATTTACGTATTGGTCATCTGCTTACTGGCCACATAATCATCATCATGT

Joelle NCBI TTTGGAGTCTGGATTTACGTATTGGTCATCTGCTTACTGGCCACATAATCATCATCATGT

CN 119243 TTTGGAGTCTGGATTTACGTATTGGTCATCTGCTTACTGGCCACATAATCATCATCATGT

CN 120025 TTTGGAGTCTGGATTTACGTATTGGTCATCTGCTTACTGGCCACATAATCATCATCATGT

Joelle AAFC TTTGGAGTCTGGATTTACGTATTGGTCATCTGCTTACTGGCCACATAATCATCATCATGT

************************************************************

CAM 241 TATGGCTTATCAATACTCCATTACCAGAAAAAAACTTGAGGTCAAGGTTCATATGAGAAC

17CS1133 TATGGCTTATCAATACTCCATTACCAGAAAAAAACTTGAGGTCAAGGTTCATATGAGAAC

CAM 236 TATGGCTTATCAATACTCCATTACCAGAAAAAAACTTGAGGTCAAGGTTCATATGAGAAC

Blaine Creek TATGGCTTATCAATACTCCATTACCAGAAAAAAACTTGAGGTCAAGGTTCATATGAGAAC

CN 119300 TATGGCTTATCAATACTCCATTACCAGAAAAAAACTTGAGGTCAAGGTTCATATGAGAAC

Yellowstone TATGGCTTATCAATACTCCATTACCAGAAAAAAACTTGAGGTCAAGGTTCATATGAGAAC

Hoga TATGGCTTATCAATACTCCATTACCAGAAAAAAACTTGAGGTCAAGGTTCATATGAGAAC

CN 120027 TATGGCTTATCAATACTCCATTACCAGAAAAAAACTTGAGGTCAAGGTTCATATGAGAAC

CN 120030 TATGGCTTATCAATACTCCATTACCAGAAAAAAACTTGAGGTCAAGGTTCATATGAGAAC

CN 119294 TATGGCTTATCAATACTCCATTACCAGAAAAAAACTTGAGGTCAAGGTTCATATGAGAAC

CN 120013 TATGGCTTATCAATACTCCATTACCAGAAAAAAACTTGAGGTCAAGGTTCATATGAGAAC

CN 120017 TATGGCTTATCAATACTCCATTACCAGAAAAAAACTTGAGGTCAAGGTTCATATGAGAAC

CN 119205 TATGGCTTATCAATACTCCATTACCAGAAAAAAACTTGAGGTCAAGGTTCATATGAGAAC

DH55 ref genome TATGGCTTATCAATACTCCATTACCAGAAAAAAACTTGAGGTCAAGGTTCATATGAGAAC

09-CS0040 TATGGCTTATCAATACTCCATTACCAGAAAAAAACTTGAGGTCAAGGTTCATATGAGAAC

CN 113754 TATGGCTTATCAATACTCCATTACCAGAAAAAAACTTGAGGTCAAGGTTCATATGAGAAC

CO46 NCBI TATGGCTTATCAATACTCCATTACCAGAAAAAAACTTGAGGTCAAGGTTCATATGAGAAC

Jasper TATGGCTTATCAATACTCCATTACCAGAAAAAAACTTGAGGTCAAGGTTCATATGAGAAC

Joelle phyto TATGGCTTATCAATACTCCATTACCAGAAAAAAACTTGAGGTCAAGGTTCATATGAGAAC

Joelle NCBI TATGGCTTATCAATACTCCATTACCAGAAAAAAACTTGAGGTCAAGGTTCATATGAGAAC

CN 119243 TATGGCTTATCAATACTCCATTACCAGAAAAAAACTTGAGGTCAAGGTTCATATGAGAAC

CN 120025 TATGGCTTATCAATACTCCATTACCAGAGAAAAACTTGAGGTCAAGGTTCATATGAGAAC

Joelle AAFC TATGGCTTATCAATACTCCATTACCAGAGAAAAACTTGAGGTCAAGGTTCATATGAGAAC

**************************** *******************************

CAM 241 TCAGAAAGTTGTCTTGTATATATGTTTAGATGGTATTACTTAGAAAACCAATAGTTGATG

17CS1133 TCAGAAAGTTGTCTTGTATATATGTTTAGATGGTATTACTTAGAAAACCAATAGTTGATG

CAM 236 TCAGAAAGTTGTCTTGTATATATGTTTAGATGGTATTACTTAGAAAACCAATAGTTGATG

Blaine Creek TCAGAAAGTTGTCTTGTATATATGTTTAGATGGTATTACTTAGAAAACCAATAGTTGATG

CN 119300 TCAGAAAGTTGTCTTGTATATATGTTTAGATGGTATTACTTAGAAAACCAATAGTTGATG

Yellowstone TCAGAAAGTTGTCTTGTATATATGTTTAGATGGTATTACTTAGAAAACCAATAGTTGATG

Hoga TCAGAAAGTTGTCTTGTATATATGTTTAGATGGTATTACTTAGAAAACCAATAGTTGATG

CN 120027 TCAGAAAGTTGTCTTGTATATATGTTTAGATGGTATTACTTAGAAAACCAATAGTTGATG

CN 120030 TCAGAAAGTTGTCTTGTATATATGTTTAGATGGTATTACTTAGAAAACCAATAGTTGATG

CN 119294 TCAGAAAGTTGTCTTGTATATATGTTTAGATGGTATTACTTAGAAAACCAATAGTTGATG

CN 120013 TCAGAAAGTTGTCTTGTATATATGTTTAGATGGTATTACTTAGAAAACCAATAGTTGATG

CN 120017 TCAGAAAGTTGTCTTGTATATATGTTTAGATGGTATTACTTAGAAAACCAATAGTTGATG

CN 119205 TCAGAAAGTTGTCTTGTATATATGTTTAGATGGTATTACTTAGAAAACCAATAGTTGATG

DH55 ref genome TCAGAAAGTTGTCTTGTATATATGTTTAGATGGTATTACTTAGAAAACCAATAGTTGATG

09-CS0040 TCAGAAAGTTGTCTTGTATATATGTTTAGATGGTATTACTTAGAAAACCAATAGTTGATG

CN 113754 TCAGAAAGTTGTCTTGTATATATGTTTAGATGGTATTACTTAGAAAACCAATAGTTGATG

CO46 NCBI TCAGAAAGTTGTCTTGTATATATGTTTAGATGGTATTACTTAGAAAACCAATAGTTGATG

Jasper TCAGAAAGTTGTCTTGTATATATGTTTAGATGGTATTACTTAGAAAACCAATAGTTGATG

Joelle phyto TCAGAAAGTTGTCTTGTATATATGTTTAGATGGTATTACTTAGAAAACCAATAGTTGATG

Joelle NCBI TCAGAAAGTTGTCTTGTATATATGTTTAGATGGTATTACTTAGAAAACCAATAGTTGATG

CN 119243 TCAGAAAGTTGTCTTGTATATATGTTTAGATGGTATTACTTAGAAAACCAATAGTTGATG

CN 120025 TCAGAAAGTTGTCTTGTATATATGTTTAGATGGTATTACTTAGAAAACCAATAGTTGATG

Joelle AAFC TCAGAAAGTTGTCTTGTATATATGTTTAGATGGTATTACTTAGAAAACCAATAGTTGATG

************************************************************

CAM 241 TCACCCTTTTTGACACACGATTAGGCGTTTTCTTTTTAGTTATGCATGAAAACACTAAGA

17CS1133 TCACCCTTTTTGACACACGATTAGGCGTTTTCTTTTTAGTTATGCATGAAAACACTAAGA

CAM 236 TCACCCTTTTTGACACACGATTAGGCGTTTTCTTTTTAGTTATGCATGAAAACACTAAGA

Blaine Creek TCACCCTTTTTGACACACGATTAGGCGTTTTCTTTTTAGTTATGCATGAAAACACTAAGA

CN 119300 TCACCCTTTTTGACACACGATTAGGCGTTTTCTTTTTAGTTATGCATGAAAACACTAAGA

Yellowstone TCACCCTTTTTGACACACGATTAGGCGTTTTCTTTTTAGTTATGCATGAAAACACTAAGA

Hoga TCACCCTTTTTGACACACGATTAGGCGTTTTCTTTTTAGTTATGCATGAAAACACTAAGA

CN 120027 TCACCCTTTTTGACACACGATTAGGCGTTTTCTTTTTAGTTATGCATGAAAACACTAAGA

CN 120030 TCACCCTTTTTGACACACGATTAGGCGTTTTCTTTTTAGTTATGCATGAAAACACTAAGA

CN 119294 TCACCCTTTTTGACACACGATTAGGCGTTTTCTTTTTAGTTATGCATGAAAACACTAAGA

CN 120013 TCACCCTTTTTGACACACGATTAGGCGTTTTCTTTTTAGTTATGCATGAAAACACTAAGA

CN 120017 TCACCCTTTTTGACACACGATTAGGCGTTTTCTTTTTAGTTATGCATGAAAACACTAAGA

CN 119205 TCACCCTTTTTGACACACGATTAGGCGTTTTCTTTTTAGTTATGCATGAAAACACTAAGA

DH55 ref genome TCACCCTTTTTGACACACGATTAGGCGTTTTCTTTTTAGTTATGCATGAAAACACTAAGA

09-CS0040 TCACCCTTTTTGACACACGATTAGGCGTTTTCTTTTTAGTTATGCATGAAAACACTAAGA

CN 113754 TCACCCTTTTTGACACACGATTAGGCGTTTTCTTTTTAGTTATGCATGAAAACACTAAGA

CO46 NCBI TCACCCTTTTTGACACACGATTAGGCGTTTTCTTTTTAGTTATGCATGAAAACACTAAGA

Jasper TCACCCTTTTTGACACACGATTAGGCGTTTTCTTTTTAGTTATGCATGAAAACACTAAGA

Joelle phyto TCACCCTTTTTGACACACGATTAGGCGTTTTCTTTTTAGTTATGCATGAAAACACTAAGA

Joelle NCBI TCACCCTTTTTGACACACGATTAGGCGTTTTCTTTTTAGTTATGCATGAAAACACTAAGA

CN 119243 TCACCCTTTTTGACACACGATTAGGCGTTTTCTTTTTAGTTATGCATGAAAACACTAAGA

CN 120025 TCACCCTTTTTGACACACGATTAGGCGTTTTCTTTTTAGTTGTGCATGAAAACACTAAGA

Joelle AAFC TCACCCTTTTTGACACACGATTAGGCGTTTTCTTTTTAGTTGTGCATGAAAACACTAAGA

***************************************** ******************

CAM 241 ATATTGATACCACTATTCGTAGTCTCAATTAATCAAATTTCATAGGAAGATGTTAAAGAT

17CS1133 ATATTGATACCACTATTCGTAGTCTCAATTAATCAAATTTCATAGGAAGATGTTAAAGAT

CAM 236 ATATTGATACCACTATTCGTAGTCTCAATTAATCAAATTTCATAGGAAGATGTTAAAGAT

Blaine Creek ATATTGATACCACTATTCGTAGTCTCAATTAATCAAATTTCATAGGAAGATGTTAAAGAT

CN 119300 ATATTGATACCACTATTCGTAGTCTCAATTAATCAAATTTCATAGGAAGATGTTAAAGAT

Yellowstone ATATTGATACCACTATTCGTAGTCTCAATTAATCAAATTTCATAGGAAGATGTTAAAGAT

Hoga ATATTGATACCACTATTCGTAGTCTCAATTAATCAAATTTCATAGGAAGATGTTAAAGAT

CN 120027 ATATTGATACCACTATTCGTAGTCTCAATTAATCAAATTTCATAGGAAGATGTTAAAGAT

CN 120030 ATATTGATACCACTATTCGTAGTCTCAATTAATCAAATTTCATAGGAAGATGTTAAAGAT

CN 119294 ATATTGATACCACTATTCGTAGTCTCAATTAATCAAATTTCATAGGAAGATGTTAAAGAT

CN 120013 ATATTGATACCACTATTCGTAGTCTCAATTAATCAAATTTCATAGGAAGATGTTAAAGAT

CN 120017 ATATTGATACCACTATTCGTAGTCTCAATTAATCAAATTTCATAGGAAGATGTTAAAGAT

CN 119205 ATATTGATACCACTATTCGTAGTCTCAATTAATCAAATTTCATAGGAAGATGTTAAAGAT

DH55 ref genome ATATTGATACCACTATTCGTAGTCTCAATTAATCAAATTTCATAGGAAGATGTTAAAGAT

09-CS0040 ATATTGATACCACTATTCGTAGTCTCAATTAATCAAATTTCATAGGAAGATGTTAAAGAT

CN 113754 ATATTGATACCACTATTCGTAGTCTCAATTAATCAAATTTCATAGGAAGATGTTAAAGAT

CO46 NCBI ATATTGATACCACTATTCGTAGTCTCAATTAATCAAATTTCATAGGAAGATGTTAAAGAT

Jasper ATATTGATACCACTATTCGTAGTCTCAATTAATCAAATTTCATAGGAAGATGTTAAAGAT

Joelle phyto ATATTGATACCACTATTCGTAGTCTCAATTAATCAAATTTCATAGGAAGATGTTAAAGAT

Joelle NCBI ATATTGATACCACTATTCGTAGTCTCAATTAATCAAATTTCATAGGAAGATGTTAAAGAT

CN 119243 ATATTGATACCACTATTCGTAGTCTCAATTAATCAAATTTCATAGGAAGATGTTAAAGAT

CN 120025 ATATTGATACCACTATTCGTAGTCTCAATTAATCAAATTTCATAGGAAGATGTTAAAGAT

Joelle AAFC ATATTGATACCACTATTCGTAGTCTCAATTAATCAAATTTCATAGGAAGATGTTAAAGAT

************************************************************

CAM 241 AGAATTTAGAGGGTAAATAGGTTTTGTTTTTATAATGGTATTGATGCCACAATTTGTGGT

17CS1133 AGAATTTAGAGGGTAAATAGGTTTTGTTTTTATAATGGTATTGATGCCACAATTTGTGGT

CAM 236 AGAATTTAGAGGGTAAATAGGTTTTGTTTTTATAATGGTATTGATGCCACAATTTGTGGT

Blaine Creek AGAATTTAGAGGGTAAATAGGTTTTGTTTTTATAATGGTATTGATGCCACAATTTGTGGT

CN 119300 AGAATTTAGAGGGTAAATAGGTTTTGTTTTTATAATGGTATTGATGCCACAATTTGTGGT

Yellowstone AGAATTTAGAGGGTAAATAGGTTTTGTTTTTATAATGGTATTGATGCCACAATTTGTGGT

Hoga AGAATTTAGAGGGTAAATAGGTTTTGTTTTTATAATGGTATTGATGCCACAATTTGTGGT

CN 120027 AGAATTTAGAGGGTAAATAGGTTTTGTTTTTATAATGGTATTGATGCCACAATTTGTGGT

CN 120030 AGAATTTAGAGGGTAAATAGGTTTTGTTTTTATAATGGTATTGATGCCACAATTTGTGGT

CN 119294 AGAATTTAGAGGGTAAATAGGTTTTGTTTTTATAATGGTATTGATGCCACAATTTGTGGT

CN 120013 AGAATTTAGAGGGTAAATAGGTTTTGTTTTTATAATGGTATTGATGCCACAATTTGTGGT

CN 120017 AGAATTTAGAGGGTAAATAGGTTTTGTTTTTATAATGGTATTGATGCCACAATTTGTGGT

CN 119205 AGAATTTAGAGGGTAAATAGGTTTTGTTTTTATAATGGTATTGATGCCACAATTTGTGGT

DH55 ref genome AGAATTTAGAGGGTAAATAGGTTTTGTTTTTATAATGGTATTGATGCCACAATTTGTGGT

09-CS0040 AGAATTTAGAGGGTAAATAGGTTTTGTTTTTATAATGGTATTGATGCCACAATTTGTGGT

CN 113754 AGAATTTAGAGGGTAAATAGGTTTTGTTTTTATAATGGTATTGATGCCACAATTTGTGGT

CO46 NCBI AGAATTTAGAGGGTAAATAGGTTTTGTTTTTATAATGGTATTGATGCCACAATTTGTGGT

Jasper AGAATTTAGAGGGTAAATAGGTTTTGTTTTTATAATGGTATTGATGCCACAATTTGTGGT

Joelle phyto AGAATTTAGAGGGTAAATAGGTTTTGTTTTTATAATGGTATTGATGCCACAATTTGTGGT

Joelle NCBI AGAATTTAGAGGGTAAATAGGTTTTGTTTTTATAATGGTATTGATGCCACAATTTGTGGT

CN 119243 AGAATTTAGAGGGTAAATAGGTTTTGTTTTTATAATGGTATTGATGCCACAATTTGTGGT

CN 120025 AGAATTTAGAGGGTAAATAGGTTTTGTTTTTATAATGGTATTGATGCCACAATTTGTGGT

Joelle AAFC AGAATTTAGAGGGTAAATAGGTTTTGTTTTTATAATGGTATTGATGCCACAATTTGTGGT

************************************************************

CAM 241 TCAAAACACAGCCGAGACAAAAGAAAGAAGATATATAGGATGTTGTTTTGACTTTTGATG

17CS1133 TCAAAACACAGCCGAGACAAAAGAAAGAAGATATATAGGATGTTGTTTTGACTTTTGATG

CAM 236 TCAAAACACAGCCGAGACAAAAGAAAGAAGATATATAGGATGTTGTTTTGACTTTTGATG

Blaine Creek TCAAAACACAGCCGAGACAAAAGAAAGAAGATATATAGGATGTTGTTTTGACTTTTGATG

CN 119300 TCAAAACACAGCCGAGACAAAAGAAAGAAGATATATAGGATGTTGTTTTGACTTTTGATG

Yellowstone TCAAAACACAGCCGAGACAAAAGAAAGAAGATATATAGGATGTTGTTTTGACTTTTGATG

Hoga TCAAAACACAGCCGAGACAAAAGAAAGAAGATATATAGGATGTTGTTTTGACTTTTGATG

CN 120027 TCAAAACACAGCCGAGACAAAAGAAAGAAGATATATAGGATGTTGTTTTGACTTTTGATG

CN 120030 TCAAAACACAGCCGAGACAAAAGAAAGAAGATATATAGGATGTTGTTTTGACTTTTGATG

CN 119294 TCAAAACACAGCCGAGACAAAAGAAAGAAGATATATAGGATGTTGTTTTGACTTTTGATG

CN 120013 TCAAAACACAGCCGAGACAAAAGAAAGAAGATATATAGGATGTTGTTTTGACTTTTGATG

CN 120017 TCAAAACACAGCCGAGACAAAAGAAAGAAGATATATAGGATGTTGTTTTGACTTTTGATG

CN 119205 TCAAAACACAGCCGAGACAAAAGAAAGAAGATATATAGGATGTTGTTTTGACTTTTGATG

DH55 ref genome TCAAAACACAGCCGAGACAAAAGAAAGAAGATATATAGGATGTTGTTTTGACTTTTGATG

09-CS0040 TCAAAACACAGCCGAGACAAAAGAAAGAAGATATATAGGATGTTGTTTTGACTTTTGATG

CN 113754 TCAAAACACAGCCGAGACAAAAGAAAGAAGATATATAGGATGTTGTTTTGACTTTTGATG

CO46 NCBI TCAAAACACAGCCGAGACAAAAGAAAGAAGATATATAGGATGTTGTTTTGACTTTTGATG

Jasper TCAAAACACAGCCGAGACAAAAGAAAGAAGATATATAGGATGTTGTTTTGACTTTTGATG

Joelle phyto TCAAAACACAGCCGAGACAAAAGAAAGAAGATATATAGGATGTTGTTTTGACTTTTGATG

Joelle NCBI TCAAAACACAGCCGAGACAAAAGAAAGAAGATATATAGGATGTTGTTTTGACTTTTGATG

CN 119243 TCAAAACACAGCCGAGACAAAAGAAAGAAGATATATAGGATGTTGTTTTGACTTTTGATG

CN 120025 TCAAAACACAGCCGAGACAAAAGAAAGAAGATATATAGGATGTTGTTTTGACTTTTGATG

Joelle AAFC TCAAAACACAGCCGAGACAAAAGAAAGAAGATATATAGGATGTTGTTTTGACTTTTGATG

************************************************************

CAM 241 CTAATTGCGGTATGAATCGAATCCAAAAATGGAAAATCAGATAGAGGTTACATACAATCA

17CS1133 CTAATTGCGGTATGAATCGAATCCAAAAATGGAAAATCAGATAGAGGTTACATACAATCA

CAM 236 CTAATTGCGGTATGAATCGAATCCAAAAATGGAAAATCAGATAGAGGTTACATACAATCA

Blaine Creek CTAATTGCGGTATGAATCGAATCCAAAAATGGAAAATCAGATAGAGGTTACATACAATCA

CN 119300 CTAATTGCGGTATGAATCGAATCCAAAAATGGAAAATCAGATAGAGGTTACATACAATCA

Yellowstone CTAATTGCGGTATGAATCGAATCCAAAAATGGAAAATCAGATAGAGGTTACATACAATCA

Hoga CTAATTGCGGTATGAATCGAATCCAAAAATGGAAAATCAGATAGAGGTTACATACAATCA

CN 120027 CTAATTGCGGTATGAATCGAATCCAAAAATGGAAAATCAGATAGAGGTTACATACAATCA

CN 120030 CTAATTGCGGTATGAATCGAATCCAAAAATGGAAAATCAGATAGAGGTTACATACAATCA

CN 119294 CTAATTGCGGTATGAATCGAATCCAAAAATGGAAAATCAGATAGAGGTTACATACAATCA

CN 120013 CTAATTGCGGTATGAATCGAATCCAAAAATGGAAAATCAGATAGAGGTTACATACAATCA

CN 120017 CTAATTGCGGTATGAATCGAATCCAAAAATGGAAAATCAGATAGAGGTTACATACAATCA

CN 119205 CTAATTGCGGTATGAATCGAATCCAAAAATGGAAAATCAGATAGAGGTTACATACAATCA

DH55 ref genome CTAATTGCGGTATGAATCGAATCCAAAAATGGAAAATCAGATAGAGGTTACATACAATCA

09-CS0040 CTAATTGCGGTATGAATCGAATCCAAAAATGGAAAATCAGATAGAGGTTACATACAATCA

CN 113754 CTAATTGCGGTATGAATCGAATCCAAAAATGGAAAATCAGATAGAGGTTACATACAATCA

CO46 NCBI CTAATTGCGGTATGAATCGAATCCAAAAATGGAAAATCAGATAGAGGTTACATACAATCA

Jasper CTAATTGCGGTATGAATCGAATCCAAAAATGGAAAATCAGATAGAGGTTACATACAATCA

Joelle phyto CTAATTGCGGTATGAATCGAATCCAAAAATGGAAAATCAGATAGAGGTTACATACAATCA

Joelle NCBI CTAATTGCGGTATGAATCGAATCCAAAAATGGAAAATCAGATAGAGGTTACATACAATCA

CN 119243 CTAATTGCGGTATGAATCGAATCCAAAAATGGAAAATCAGATAGAGGTTACATACAATCA

CN 120025 CTAATTGCGGTATGAATCGAATCCAAAAATGGAAAATCAGATAGAGGTTACATACAATCA

Joelle AAFC CTAATTGCGGTATGAATCGAATCCAAAAATGGAAAATCAGATAGAGGTTACATACAATCA

************************************************************

CAM 241 GAATAATGTAAGTCAATGAATTGGAAGCAGTTTTCAACTGTTTCTTATGTTTAGGGTTGT

17CS1133 GAATAATGTAAGTCAATGAATTGGAAGCAGTTTTCAACTGTTTCTTATGTTTAGGGTTGT

CAM 236 GAATAATGTAAGTCAATGAATTGGAAGCAGTTTTCAACTGTTTCTTATGTTTAGGGTTGT

Blaine Creek GAATAATGTAAGTCAATGAATTGGAAGCAGTTTTCAACTGTTTCTTATGTTTAGGGTTGT

CN 119300 GAATAATGTAAGTCAATGAATTGGAAGCAGTTTTCAACTGTTTCTTATGTTTAGGGTTGT

Yellowstone GAATAATGTAAGTCAATGAATTGGAAGCAGTTTTCAACTGTTTCTTATGTTTAGGGTTGT

Hoga GAATAATGTAAGTCAATGAATTGGAAGCAGTTTTCAACTGTTTCTTATGTTTAGGGTTGT

CN 120027 GAATAATGTAAGTCAATGAATTGGAAGCAGTTTTCAACTGTTTCTTATGTTTAGGGTTGT

CN 120030 GAATAATGTAAGTCAATGAATTGGAAGCAGTTTTCAACTGTTTCTTATGTTTAGGGTTGT

CN 119294 GAATAATGTAAGTCAATGAATTGGAAGCAGTTTTCAACTGTTTCTTATGTTTAGGGTTGT

CN 120013 GAATAATGTAAGTCAATGAATTGGAAGCAGTTTTCAACTGTTTCTTATGTTTAGGGTTGT

CN 120017 GAATAATGTAAGTCAATGAATTGGAAGCAGTTTTCAACTGTTTCTTATGTTTAGGGTTGT

CN 119205 GAATAATGTAAGTCAATGAATTGGAAGCAGTTTTCAACTGTTTCTTATGTTTAGGGTTGT

DH55 ref genome GAATAATGTAAGTCAATGAATTGGAAGCAGTTTTCAACTGTTTCTTATGTTTAGGGTTGT

09-CS0040 GAATAATGTAAGTCAATGAATTGGAAGCAGTTTTCAACTGTTTCTTATGTTTAGGGTTGT

CN 113754 GAATAATGTAAGTCAATGAATTGGAAGCAGTTTTCAACTGTTTCTTATGTTTAGGGTTGT

CO46 NCBI GAATAATGTAAGTCAATGAATTGGAAGCAGTTTTCAACTGTTTCTTATGTTTAGGGTTGT

Jasper GAATAATGTAAGTCAATGAATTGGAAGCAGTTTTCAACTGTTTCTTATGTTTAGGGTTGT

Joelle phyto GAATAATGTAAGTCAATGAATTGGAAGCAGTTTTCAACTGTTTCTTATGTTTAGGGTTGT

Joelle NCBI GAATAATGTAAGTCAATGAATTGGAAGCAGTTTTCAACTGTTTCTTATGTTTAGGGTTGT

CN 119243 GAATAATGTAAGTCAATGAATTGGAAGCAGTTTTCAACTGTTTCTTATGTTTAGGGTTGT

CN 120025 GAATAATGTAAGTCAATGAATTGGAAGCAGTTTTCAACTGTTTCTTATGTTTAGGGTTGT

Joelle AAFC GAATAATGTAAGTCAATGAATTGGAAGCAGTTTTCAACTGTTTCTTATGTTTAGGGTTGT

************************************************************

CAM 241 CTTTTAATAATGTGCCAAAATTATAAATCAAAATTACTTTCTGTAAGACTC**G**GTCCATGT

17CS1133 CTTTTAATAATGTGCCAAAATTATAAATCAAAATTACTTTCTGTAAGACTC**G**GTCCATGT

CAM 236 CTTTTAATAATGTGCCAAAATTATAAATCAAAATTACTTTCTGTAAGACTC**G**GTCCATGT

Blaine Creek CTTTTAATAATGTGCCAAAATTATAAATCAAAATTACTTTCTGTAAGACTC**G**GTCCATGT

CN 119300 CTTTTAATAATGTGCCAAAATTATAAATCAAAATTACTTTCTGTAAGACTC**G**GTCCATGT

Yellowstone CTTTTAATAATGTGCCAAAATTATAAATCAAAATTACTTTCTGTAAGACTC**G**GTCCATGT

Hoga CTTTTAATAATGTGCCAAAATTATAAATCAAAATTACTTTCTGTAAGACTC**G**GTCCATGT

CN 120027 CTTTTAATAATGTGCCAAAATTATAAATCAAAATTACTTTCTGTAAGACTC**G**GTCCATGT

CN 120030 CTTTTAATAATGTGCCAAAATTATAAATCAAAATTACTTTCTGTAAGACTC**G**GTCCATGT

CN 119294 CTTTTAATAATGTGCCAAAATTATAAATCAAAATTACTTTCTGTAAGACTC**G**GTCCATGT

CN 120013 CTTTTAATAATGTGCCAAAATTATAAATCAAAATTACTTTCTGTAAGACTC**G**GTCCATGT

CN 120017 CTTTTAATAATGTGCCAAAATTATAAATCAAAATTACTTTCTGTAAGACTC**G**GTCCATGT

CN 119205 CTTTTAATAATGTGCCAAAATTATAAATCAAAATTACTTTCTGTAAGACTC**G**GTCCATGT

DH55 ref genome CTTTTAATAATGTGCCAAAATTATAAATCAAAATTACTTTCTGTAAGACTC**G**GTCCATGT

09-CS0040 CTTTTAATAATGTGCCAAAATTATAAATCAAAATTACTTTCTGTAAGACTC**G**GTCCATGT

CN 113754 CTTTTAATAATGTGCCAAAATTATAAATCAAAATTACTTTCTGTAAGACTC**G**GTCCATGT

CO46 NCBI CTTTTAATAATGTGCCAAAATTATAAATCAAAATTACTTTCTGTAAGACTC**G**GTCCATGT

Jasper CTTTTAATAATGTGCCAAAATTATAAATCAAAATTACTTTCTGTAAGACTC**G**GTCCATGT

Joelle phyto CTTTTAATAATGTGCCAAAATTATAAATCAAAATTACTTTCTGTAAGACTC**G**GTCCATGT

Joelle NCBI CTTTTAATAATGTGCCAAAATTATAAATCAAAATTACTTTCTGTAAGACTC**G**GTCCATGT

CN 119243 CTTTTAATAATGTGCCGAAATTATAAATCAAAATTACTTTCTGTAAGACTC**T**GTCCATGT

CN 120025 CTTTTAATAATGTGCCGAAATTATAAATCAAAATTACTTTCTGTAAGACTC**T**GTCCATGT

Joelle AAFC CTTTTAATAATGTGCCGAAATTATAAATCAAAATTACTTTCTGTAAGACTC**T**GTCCATGT

**************** ********************************** ********

CAM 241 CCTTTGAATTTGGCTTAAACATACGTATTGAACGTCTGCTTACTGCCATGTCATTACGTT

17CS1133 CCTTTGAATTTGGCTTAAACATACGTATTGAACGTCTGCTTACTGCCATGTCATTACGTT

CAM 236 CCTTTGAATTTGGCTTAAACATACGTATTGAACGTCTGCTTACTGCCATGTCATTACGTT

Blaine Creek CCTTTGAATTTGGCTTAAACATACGTATTGAACGTCTGCTTACTGCCATGTCATTACGTT

CN 119300 CCTTTGAATTTGGCTTAAACATACGTATTGAACGTCTGCTTACTGCCATGTCATTACGTT

Yellowstone CCTTTGAATTTGGCTTAAACATACGTATTGAACGTCTGCTTACTGCCATGTCATTACGTT

Hoga CCTTTGAATTTGGCTTAAACATACGTATTGAACGTCTGCTTACTGCCATGTCATTACGTT

CN 120027 CCTTTGAATTTGGCTTAAACATACGTATTGAACGTCTGCTTACTGCCATGTCATTACGTT

CN 120030 CCTTTGAATTTGGCTTAAACATACGTATTGAACGTCTGCTTACTGCCATGTCATTACGTT

CN 119294 CCTTTGAATTTGGCTTAAACATACGTATTGAACGTCTGCTTACTGCCATGTCATTACGTT

CN 120013 CCTTTGAATTTGGCTTAAACATACGTATTGAACGTCTGCTTACTGCCATGTCATTACGTT

CN 120017 CCTTTGAATTTGGCTTAAACATACGTATTGAACGTCTGCTTACTGCCATGTCATTACGTT

CN 119205 CCTTTGAATTTGGCTTAAACATACGTATTGAACGTCTGCTTACTGCCATGTCATTACGTT

DH55 ref genome CCTTTGAATTTGGCTTAAACATACGTATTGAACGTCTGCTTACTGCCATGTCATTACGTT

09-CS0040 CCTTTGAATTTGGCTTAAACATACGTATTGAACGTCTGCTTACTGCCATGTCATTACGTT

CN 113754 CCTTTGAATTTGGCTTAAACATACGTATTGAACGTCTGCTTACTGCCATGTCATTACGTT

CO46 NCBI CCTTTGAATTTGGCTTAAACATACGTATTGAACGTCTGCTTACTGCCATGTCATTACGTT

Jasper CCTTTGAATTTGGCTTAAACATACGTATTGAACGTCTGCTTACTGCCATGTCATTACGTT

Joelle phyto CCTTTGAATTTGGCTTAAACATACGTATTGAACGTCTGCTTACTGCCATGTCATTACGTT

Joelle NCBI CCTTTGAATTTGGCTTAAACATACGTATTGAACGTCTGCTTACTGCCATGTCATTACGTT

CN 119243 CCTTTGAATTTGGCTTAAACATACGTATTGAACGTCTGCTTACTGCCATGTCATTACGTT

CN 120025 CCTTTGAATTTGGCTTAAACATACGTATTGAACGTCTGCTTACTGCCATGTCATTACGTT

Joelle AAFC CCTTTGAATTTGGCTTAAACATACGTATTGAACGTCTGCTTACTGCCATGTCATTACGTT

************************************************************

CAM 241 GTGGTTCATCAATATTTGTGTGTACGTTTTCGTGAGTATATGTTTTCTTTAGCAGTAAAA

17CS1133 GTGGTTCATCAATATTTGTGTGTACGTTTTCGTGAGTATATGTTTTCTTTAGCAGTAAAA

CAM 236 GTGGTTCATCAATATTTGTGTGTACGTTTTCGTGAGTATATGTTTTCTTTAGCAGTAAAA

Blaine Creek GTGGTTCATCAATATTTGTGTGTACGTTTTCGTGAGTATATGTTTTCTTTAGCAGTAAAA

CN 119300 GTGGTTCATCAATATTTGTGTGTACGTTTTCGTGAGTATATGTTTTCTTTAGCAGTAAAA

Yellowstone GTGGTTCATCAATATTTGTGTGTACGTTTTCGTGAGTATATGTTTTCTTTAGCAGTAAAA

Hoga GTGGTTCATCAATATTTGTGTGTACGTTTTCGTGAGTATATGTTTTCTTTAGCAGTAAAA

CN 120027 GTGGTTCATCAATATTTGTGTGTACGTTTTCGTGAGTATATGTTTTCTTTAGCAGTAAAA

CN 120030 GTGGTTCATCAATATTTGTGTGTACGTTTTCGTGAGTATATGTTTTCTTTAGCAGTAAAA

CN 119294 GTGGTTCATCAATATTTGTGTGTACGTTTTCGTGAGTATATGTTTTCTTTAGCAGTAAAA

CN 120013 GTGGTTCATCAATATTTGTGTGTACGTTTTCGTGAGTATATGTTTTCTTTAGCAGTAAAA

CN 120017 GTGGTTCATCAATATTTGTGTGTACGTTTTCGTGAGTATATGTTTTCTTTAGCAGTAAAA

CN 119205 GTGGTTCATCAATATTTGTGTGTACGTTTTCGTGAGTATATGTTTTCTTTAGCAGTAAAA

DH55 ref genome GTGGTTCATCAATATTTGTGTGTACGTTTTCGTGAGTATATGTTTTCTTTAGCAGTAAAA

09-CS0040 GTGGTTCATCAATATTTGTGTGTACGTTTTCGTGAGTATATGTTTTCTTTAGCAGTAAAA

CN 113754 GTGGTTCATCAATATTTGTGTGTACGTTTTCGTGAGTATATGTTTTCTTTAGCAGTAAAA

CO46 NCBI GTGGTTCATCAATATTTGTGTGTACGTTTTCGTGAGTATATGTTTTCTTTAGCAGTAAAA

Jasper GTGGTTCATCAATATTTGTGTGTACGTTTTCGTGAGTATATGTTTTCTTTAGCAGTAAAA

Joelle phyto GTGGTTCATCAATATTTGTGTGTACGTTTTCGTGAGTATATGTTTTCTTTAGCAGTAAAA

Joelle NCBI GTGGTTCATCAATATTTGTGTGTACGTTTTCGTGAGTATATGTTTTCTTTAGCAGTAAAA

CN 119243 GTGGTTCATCAATATTTGTGTGTACGTTTTCGTGAGTATATGTTTTCTTTAGCAGTAAAA

CN 120025 GTGGTTCATCAATATTTGTGTGTACGTTTTCGTGAGTATATGTTTTCTTTAGCAGTAAAA

Joelle AAFC GTGGTTCATCAATATTTGTGTGTACGTTTTCGTGAGTATATGTTTTCTTTAGCAGTAAAA

************************************************************

CAM 241 AGTCTTGAGTGTATTGTACACACTCTATTAAACCACAATTGCTTGTTTTCATTTAAGGTT

17CS1133 AGTCTTGAGTGTATTGTACACACTCTATTAAACCACAATTGCTTGTTTTCATTTAAGGTT

CAM 236 AGTCTTGAGTGTATTGTACACACTCTATTAAACCACAATTGCTTGTTTTCATTTAAGGTT

Blaine Creek AGTCTTGAGTGTATTGTACACACTCTATTAAACCACAATTGCTTGTTTTCATTTAAGGTT

CN 119300 AGTCTTGAGTGTATTGTACACACTCTATTAAACCACAATTGCTTGTTTTCATTTAAGGTT

Yellowstone AGTCTTGAGTGTATTGTACACACTCTATTAAACCACAATTGCTTGTTTTCATTTAAGGTT

Hoga AGTCTTGAGTGTATTGTACACACTCTATTAAACCACAATTGCTTGTTTTCATTTAAGGTT

CN 120027 AGTCTTGAGTGTATTGTACACACTCTATTAAACCACAATTGCTTGTTTTCATTTAAGGTT

CN 120030 AGTCTTGAGTGTATTGTACACACTCTATTAAACCACAATTGCTTGTTTTCATTTAAGGTT

CN 119294 AGTCTTGAGTGTATTGTACACACTCTATTAAACCACAATTGCTTGTTTTCATTTAAGGTT

CN 120013 AGTCTTGAGTGTATTGTACACACTCTATTAAACCACAATTGCTTGTTTTCATTTAAGGTT

CN 120017 AGTCTTGAGTGTATTGTACACACTCTATTAAACCACAATTGCTTGTTTTCATTTAAGGTT

CN 119205 AGTCTTGAGTGTATTGTACACACTCTATTAAACCACAATTGCTTGTTTTCATTTAAGGTT

DH55 ref genome AGTCTTGAGTGTATTGTACACACTCTATTAAACCACAATTGCTTGTTTTCATTTAAGGTT

09-CS0040 AGTCTTGAGTGTATTGTACACACTCTATTAAACCACAATTGCTTGTTTTCATTTAAGGTT

CN 113754 AGTCTTGAGTGTATTGTACACACTCTATTAAACCACAATTGCTTGTTTTCATTTAAGGTT

CO46 NCBI AGTCTTGAGTGTATTGTACACACTCTATTAAACCACAATTGCTTGTTTTCATTTAAGGTT

Jasper AGTCTTGAGTGTATTGTACACACTCTATTAAACCACAATTGCTTGTTTTCATTTAAGGTT

Joelle phyto AGTCTTGAGTGTATTGTACACACTCTATTAAACCACAATTGCTTGTTTTCATTTAAGGTT

Joelle NCBI AGTCTTGAGTGTATTGTACACACTCTATTAAACCACAATTGCTTGTTTTCATTTAAGGTT

CN 119243 AGTCTTGAGTGTATTGTACACACTCTATTAAACCACAATTGCTTGTTTTCATTTAAGGTT

CN 120025 AGTCTTGAGTGTATTGTACACACTCTATTAAACCACAATTGCTTGTTTTCATTTAAGGTT

Joelle AAFC AGTCTTGAGTGTATTGTACACACTCTATTAAACCACAATTGCTTGTTTTCATTTAAGGTT

************************************************************

CAM 241 GCTGGTGTGTTTCCATGTTATATTATATCAATTAATGTACCATATATAGAAGCACAAACA

17CS1133 GCTGGTGTGTTTCCATGTTATATTATATCAATTAATGTACCATATATAGAAGCACAAACA

CAM 236 GCTGGTGTGTTTCCATGTTATATTATATCAATTAATGTACCATATATAGAAGCACAAACA

Blaine Creek GCTGGTGTGTTTCCATGTTATATTATATCAATTAATGTACCATATATAGAAGCACAAACA

CN 119300 GCTGGTGTGTTTCCATGTTATATTATATCAATTAATGTACCATATATAGAAGCACAAACA

Yellowstone GCTGGTGTGTTTCCATGTTATATTATATCAATTAATGTACCATATATAGAAGCACAAACA

Hoga GCTGGTGTGTTTCCATGTTATATTATATCAATTAATGTACCATATATAGAAGCACAAACA

CN 120027 GCTGGTGTGTTTCCATGTTATATTATATCAATTAATGTACCATATATAGAAGCACAAACA

CN 120030 GCTGGTGTGTTTCCATGTTATATTATATCAATTAATGTACCATATATAGAAGCACAAACA

CN 119294 GCTGGTGTGTTTCCATGTTATATTATATCAATTAATGTACCATATATAGAAGCACAAACA

CN 120013 GCTGGTGTGTTTCCATGTTATATTATATCAATTAATGTACCATATATAGAAGCACAAACA

CN 120017 GCTGGTGTGTTTCCATGTTATATTATATCAATTAATGTACCATATATAGAAGCACAAACA

CN 119205 GCTGGTGTGTTTCCATGTTATATTATATCAATTAATGTACCATATATAGAAGCACAAACA

DH55 ref genome GCTGGTGTGTTTCCATGTTATATTATATCAATTAATGTACCATATATAGAAGCACAAACA

09-CS0040 GCTGGTGTGTTTCCATGTTATATTATATCAATTAATGTACCATATATAGAAGCACAAACA

CN 113754 GCTGGTGTGTTTCCATGTTATATTATATCAATTAATGTACCATATATAGAAGCACAAACA

CO46 NCBI GCTGGTGTGTTTCCATGTTATATTATATCAATTAATGTACCATATATAGAAGCACAAACA

Jasper GCTGGTGTGTTTCCATGTTATATTATATCAATTAATGTACCATATATAGAAGCACAAACA

Joelle phyto GCTGGTGTGTTTCCATGTTATATTATATCAATTAATGTACCATATATAGAAGCACAAACA

Joelle NCBI GCTGGTGTGTTTCCATGTTATATTATATCAATTAATGTACCATATATAGAAGCACAAACA

CN 119243 GCTGGTGTGTTTCCATGTTATATTATATCAATTAATGTACCATATATAGAAGCACAAACA

CN 120025 GCTGGTGTGTTTCCATGTTATATTATATCAATTAATGTACCATATATAGAAGCACAAACA

Joelle AAFC GCTGGTGTGTTTCCATGTTATATTATATCAATTAATGTACCATATATAGAAGCACAAACA

************************************************************

CAM 241 AATTGCATAGAAACAATCTGGACAGTGGATGCTTGAGATTAGGGTTTTCTGTAAACGAAA

17CS1133 AATTGCATAGAAACAATCTGGACAGTGGATGCTTGAGATTAGGGTTTTCTGTAAACGAAA

CAM 236 AATTGCATAGAAACAATCTGGACAGTGGATGCTTGAGATTAGGGTTTTCTGTAAACGAAA

Blaine Creek AATTGCATAGAAACAATCTGGACAGTGGATGCTTGAGATTAGGGTTTTCTGTAAACGAAA

CN 119300 AATTGCATAGAAACAATCTGGACAGTGGATGCTTGAGATTAGGGTTTTCTGTAAACGAAA

Yellowstone AATTGCATAGAAACAATCTGGACAGTGGATGCTTGAGATTAGGGTTTTCTGTAAACGAAA

Hoga AATTGCATAGAAACAATCTGGACAGTGGATGCTTGAGATTAGGGTTTTCTGTAAACGAAA

CN 120027 AATTGCATAGAAACAATCTGGACAGTGGATGCTTGAGATTAGGGTTTTCTGTAAACGAAA

CN 120030 AATTGCATAGAAACAATCTGGACAGTGGATGCTTGAGATTAGGGTTTTCTGTAAACGAAA

CN 119294 AATTGCATAGAAACAATCTGGACAGTGGATGCTTGAGATTAGGGTTTTCTGTAAACGAAA

CN 120013 AATTGCATAGAAACAATCTGGACAGTGGATGCTTGAGATTAGGGTTTTCTGTAAACGAAA

CN 120017 AATTGCATAGAAACAATCTGGACAGTGGATGCTTGAGATTAGGGTTTTCTGTAAACGAAA

CN 119205 AATTGCATAGAAACAATCTGGACAGTGGATGCTTGAGATTAGGGTTTTCTGTAAACGAAA

DH55 ref genome AATTGCATAGAAACAATCTGGACAGTGGATGCTTGAGATTAGGGTTTTCTGTAAACGAAA

09-CS0040 AATTGCATAGAAACAATCTGGACAGTGGATGCTTGAGATTAGGGTTTTCTGTAAACGAAA

CN 113754 AATTGCATAGAAACAATCTGGACAGTGGATGCTTGAGATTAGGGTTTTCTGTAAACGAAA

CO46 NCBI AATTGCATAGAAACAATCTGGACAGTGGATGCTTGAGATTAGGGTTTTCTGTAAACGAAA

Jasper AATTGCATAGAAACAATCTGGACAGTGGATGCTTGAGATTAGGGTTTTCTGTAAACGAAA

Joelle phyto AATTGCATAGAAACAATCTGGACAGTGGATGCTTGAGATTAGGGTTTTCTGTAAACGAAA

Joelle NCBI AATTGCATAGAAACAATCTGGACAGTGGATGCTTGAGATTAGGGTTTTCTGTAAACGAAA

CN 119243 AATTGCATAGAAACAATCTGGACAGTGGATGCTTGAGATTAGGGTTTTCTGTAAACGAAA

CN 120025 AATTGCATAGAAACAATCTGGACAGTGGATGCTTGAGATTAGGGTTTTCTGTAAACGAAA

Joelle AAFC AATTGCATAGAAACAATCTGGACAGTGGATGCTTGAGATTAGGGTTTTCTGTAAACGAAA

************************************************************

CAM 241 TGTTAGATCACAGGGATAATCTATATACATGCCTTAATTTTAGTAAGCCTTTTCTTTGTG

17CS1133 TGTTAGATCACAGGGATAATCTATATACATGCCTTAATTTTAGTAAGCCTTTTCTTTGTG

CAM 236 TGTTAGATCACAGGGATAATCTATATACATGCCTTAATTTTAGTAAGCCTTTTCTTTGTG

Blaine Creek TGTTAGATCACAGGGATAATCTATATACATGCCTTAATTTTAGTAAGCCTTTTCTTTGTG

CN 119300 TGTTAGATCACAGGGATAATCTATATACATGCCTTAATTTTAGTAAGCCTTTTCTTTGTG

Yellowstone TGTTAGATCACAGGGATAATCTATATACATGCCTTAATTTTAGTAAGCCTTTTCTTTGTG

Hoga TGTTAGATCACAGGGATAATCTATATACATGCCTTAATTTTAGTAAGCCTTTTCTTTGTG

CN 120027 TGTTAGATCACAGGGATAATCTATATACATGCCTTAATTTTAGTAAGCCTTTTCTTTGTG

CN 120030 TGTTAGATCACAGGGATAATCTATATACATGCCTTAATTTTAGTAAGCCTTTTCTTTGTG

CN 119294 TGTTAGATCACAGGGATAATCTATATACATGCCTTAATTTTAGTAAGCCTTTTCTTTGTG

CN 120013 TGTTAGATCACAGGGATAATCTATATACATGCCTTAATTTTAGTAAGCCTTTTCTTTGTG

CN 120017 TGTTAGATCACAGGGATAATCTATATACATGCCTTAATTTTAGTAAGCCTTTTCTTTGTG

CN 119205 TGTTAGATCACAGGGATAATCTATATACATGCCTTAATTTTAGTAAGCCTTTTCTTTGTG

DH55 ref genome TGTTAGATCACAGGGATAATCTATATACATGCCTTAATTTTAGTAAGCCTTTTCTTTGTG

09-CS0040 TGTTAGATCACAGGGATAATCTATATACATGCCTTAATTTTAGTAAGCCTTTTCTTTGTG

CN 113754 TGTTAGATCACAGGGATAATCTATATACATGCCTTAATTTTAGTAAGCCTTTTCTTTGTG

CO46 NCBI TGTTAGATCACAGGGATAATCTATATACATGCCTTAATTTTAGTAAGCCTTTTCTTTGTG

Jasper TGTTAGATCACAGGGATAATCTATATACATGCCTTAATTTTAGTAAGCCTTTTCTTTGTG

Joelle phyto TGTTAGATCACAGGGATAATCTATATACATGCCTTAATTTTAGTAAGCCTTTTCTTTGTG

Joelle NCBI TGTTAGATCACAGGGATAATCTATATACATGCCTTAATTTTAGTAAGCCTTTTCTTTGTG

CN 119243 TGTTAGATCACAGGGATAATCTATATACATGCCTTAATTTTAGTAAGCCTTTTCTTTGTG

CN 120025 TGTTAGATCACAGGGATAATCTATATACATGCCTTAATTTTAGTAAGCCTTTTCTTTGTG

Joelle AAFC TGTTAGATCACAGGGATAATCTATATACATGCCTTAATTTTAGTAAGCCTTTTCTTTGTG

************************************************************

CAM 241 GAAAAATGTTCAATTTGGACCTACCGAAATATATAATAATAATTCATTGGATTATCAAAC

17CS1133 GAAAAATGTTCAATTTGGACCTACCGAAATATATAATAATAATTCATTGGATTATCAAAC

CAM 236 GAAAAATGTTCAATTTGGACCTACCGAAATATATAATAATAATTCATTGGATTATCAAAC

Blaine Creek GAAAAATGTTCAATTTGGACCTACCGAAATATATAATAATAATTCATTGGATTATCAAAC

CN 119300 GAAAAATGTTCAATTTGGACCTACCGAAATATATAATAATAATTCATTGGATTATCAAAC

Yellowstone GAAAAATGTTCAATTTGGACCTACCGAAATATATAATAATAATTCATTGGATTATCAAAC

Hoga GAAAAATGTTCAATTTGGACCTACCGAAATATATAATAATAATTCATTGGATTATCAAAC

CN 120027 GAAAAATGTTCAATTTGGACCTACCGAAATATATAATAATAATTCATTGGATTATCAAAC

CN 120030 GAAAAATGTTCAATTTGGACCTACCGAAATATATAATAATAATTCATTGGATTATCAAAC

CN 119294 GAAAAATGTTCAATTTGGACCTACCGAAATATATAATAATAATTCATTGGATTATCAAAC

CN 120013 GAAAAATGTTCAATTTGGACCTACCGAAATATATAATAATAATTCATTGGATTATCAAAC

CN 120017 GAAAAATGTTCAATTTGGACCTACCGAAATATATAATAATAATTCATTGGATTATCAAAC

CN 119205 GAAAAATGTTCAATTTGGACCTACCGAAATATATAATAATAATTCATTGGATTATCAAAC

DH55 ref genome GAAAAATGTTCAATTTGGACCTACCGAAATATATAATAATAATTCATTGGATTATCAAAC

09-CS0040 GAAAAATGTTCAATTTGGACCTACCGAAATATATAATAATAATTCATTGGATTATCAAAC

CN 113754 GAAAAATGTTCAATTTGGACCTACCGAAATATATAATAATAATTCATTGGATTATCAAAC

CO46 NCBI GAAAAATGTTCAATTTGGACCTACCGAAATATATAATAATAATTCATTGGATTATCAAAC

Jasper GAAAAATGTTCAATTTGGACCTACCGAAATATATAATAATAATTCATTGGATTATCAAAC

Joelle phyto GAAAAATGTTCAATTTGGACCTACCGAAATATATAATAATAATTCATTGGATTATCAAAC

Joelle NCBI GAAAAATGTTCAATTTGGACCTACCGAAATATATAATAATAATTCATTGGATTATCAAAC

CN 119243 GAAAAATGTTCAATTTGGACCTACCGAAATATATAATAATAATTCATTGGATTATCAAAC

CN 120025 GAAAAATGTTCAATTTGGACCTACCGAAATATATAATAATAATTCATTGGATTATCAAAC

Joelle AAFC GAAAAATGTTCAATTTGGACCTACCGAAATATATAATAATAATTCATTGGATTATCAAAC

************************************************************

CAM 241 TTTGAACATTTCTAGGTAAATGTTTATAGTTCTTTTTTTAGAGATGAAAAAAATCTATAT

17CS1133 TTTGAACATTTCTAGGTAAATGTTTATAGTTCTTTTTTTAGAGATGAAAAAAATCTATAT

CAM 236 TTTGAACATTTCTAGGTAAATGTTTATAGTTCTTTTTTTAGAGATGAAAAAAATCTATAT

Blaine Creek TTTGAACATTTCTAGGTAAATGTTTATAGTTCTTTTTTTAGAGATGAAAAAAATCTATAT

CN 119300 TTTGAACATTTCTAGGTAAATGTTTATAGTTCTTTTTTTAGAGATGAAAAAAATCTATAT

Yellowstone TTTGAACATTTCTAGGTAAATGTTTATAGTTCTTTTTTTAGAGATGAAAAAAATCTATAT

Hoga TTTGAACATTTCTAGGTAAATGTTTATAGTTCTTTTTTTAGAGATGAAAAAAATCTATAT

CN 120027 TTTGAACATTTCTAGGTAAATGTTTATAGTTCTTTTTTTAGAGATGAAAAAAATCTATAT

CN 120030 TTTGAACATTTCTAGGTAAATGTTTATAGTTCTTTTTTTAGAGATGAAAAAAATCTATAT

CN 119294 TTTGAACATTTCTAGGTAAATGTTTATAGTTCTTTTTTTAGAGATGAAAAAAATCTATAT

CN 120013 TTTGAACATTTCTAGGTAAATGTTTATAGTTCTTTTTTTAGAGATGAAAAAAATCTATAT

CN 120017 TTTGAACATTTCTAGGTAAATGTTTATAGTTCTTTTTTTAGAGATGAAAAAAATCTATAT

CN 119205 TTTGAACATTTCTAGGTAAATGTTTATAGTTCTTTTTTTAGAGATGAAAAAAATCTATAT

DH55 ref genome TTTGAACATTTCTAGGTAAATGTTTATAGTTCTTTTTTTAGAGATGAAAAAAATCTATAT

09-CS0040 TTTGAACATTTCTAGGTAAATGTTTATAGTTCTTTTTTTAGAGATGAAAAAAATCTATAT

CN 113754 TTTGAACATTTCTAGGTAAATGTTTATAGTTCTTTTTTTAGAGATGAAAAAAATCTATAT

CO46 NCBI TTTGAACATTTCTAGGTAAATGTTTATAGTTCTTTTTTTAGAGATGAAAAAAATCTATAT

Jasper TTTGAACATTTCTAGGTAAATGTTTATAGTTCTTTTTTTAGAGATGAAAAAAATCTATAT

Joelle phyto TTTGAACATTTCTAGGTAAATGTTTATAGTTCTTTTTTTAGAGATGAAAAAAATCTATAT

Joelle NCBI TTTGAACATTTCTAGGTAAATGTTTATAGTTCTTTTTTTAGAGATGAAAAAAATCTATAT

CN 119243 TTTGAACATTTCTTGGTAAATGTTTATAGTTCTTTTTTTAGAGATGAAAAAAATCTATAT

CN 120025 TTTGAACATTTCTTGGTAAATGTTTATAGTTCTTTTTTTAGAGATGAAAAAAATCTATAT

Joelle AAFC TTTGAACATTTCTTGGTAAATGTTTATAGTTCTTTTTTTAGAGATGAAAAAAATCTATAT

************* **********************************************

CAM 241 GTGTGTAATAATAAGCAATTAAGGTTTTGTCTTTAATTGTCTCTTCTGATGCCATCATAT

17CS1133 GTGTGTAATAATAAGCAATTAAGGTTTTGTCTTTAATTGTCTCTTCTGATGCCATCATAT

CAM 236 GTGTGTAATAATAAGCAATTAAGGTTTTGTCTTTAATTGTCTCTTCTGATGCCATCATAT

Blaine Creek GTGTGTAATAATAAGCAATTAAGGTTTTGTCTTTAATTGTCTCTTCTGATGCCATCATAT

CN 119300 GTGTGTAATAATAAGCAATTAAGGTTTTGTCTTTAATTGTCTCTTCTGATGCCATCATAT

Yellowstone GTGTGTAATAATAAGCAATTAAGGTTTTGTCTTTAATTGTCTCTTCTGATGCCATCATAT

Hoga GTGTGTAATAATAAGCAATTAAGGTTTTGTCTTTAATTGTCTCTTCTGATGCCATCATAT

CN 120027 GTGTGTAATAATAAGCAATTAAGGTTTTGTCTTTAATTGTCTCTTCTGATGCCATCATAT

CN 120030 GTGTGTAATAATAAGCAATTAAGGTTTTGTCTTTAATTGTCTCTTCTGATGCCATCATAT

CN 119294 GTGTGTAATAATAAGCAATTAAGGTTTTGTCTTTAATTGTCTCTTCTGATGCCATCATAT

CN 120013 GTGTGTAATAATAAGCAATTAAGGTTTTGTCTTTAATTGTCTCTTCTGATGCCATCATAT

CN 120017 GTGTGTAATAATAAGCAATTAAGGTTTTGTCTTTAATTGTCTCTTCTGATGCCATCATAT

CN 119205 GTGTGTAATAATAAGCAATTAAGGTTTTGTCTTTAATTGTCTCTTCTGATGCCATCATAT

DH55 ref genome GTGTGTAATAATAAGCAATTAAGGTTTTGTCTTTAATTGTCTCTTCTGATGCCATCATAT

09-CS0040 GTGTGTAATAATAAGCAATTAAGGTTTTGTCTTTAATTGTCTCTTCTGATGCCATCATAT

CN 113754 GTGTGTAATAATAAGCAATTAAGGTTTTGTCTTTAATTGTCTCTTCTGATGCCATCATAT

CO46 NCBI GTGTGTAATAATAAGCAATTAAGGTTTTGTCTTTAATTGTCTCTTCTGATGCCATCATAT

Jasper GTGTGTAATAATAAGCAATTAAGGTTTTGTCTTTAATTGTCTCTTCTGATGCCATCATAT

Joelle phyto GTGTGTAATAATAAGCAATTAAGGTTTTGTCTTTAATTGTCTCTTCTGATGCCATCATAT

Joelle NCBI GTGTGTAATAATAAGCAATTAAGGTTTTGTCTTTAATTGTCTCTTCTGATGCCATCATAT

CN 119243 GTGTGTAATAATAAGCAATTAAGGTTTTGTCTTTAATTGTCTCTTCTGATGCCATCATAT

CN 120025 GTGTGTAATAATAAGCAATTAAGGTTTTGTCTTTAATTGTCTCTTCTGATGCCATCATAT

Joelle AAFC GTGTGTAATAATAAGCAATTAAGGTTTTGTCTTTAATTGTCTCTTCTGATGCCATCATAT

************************************************************

CAM 241 ACATGTTTTATAGTTTCCAGTGGTCTTTTCAAGGGTTAGCTTGTATTTTATTATCCTAAC

17CS1133 ACATGTTTTATAGTTTCCAGTGGTCTTTTCAAGGGTTAGCTTGTATTTTATTATCCTAAC

CAM 236 ACATGTTTTATAGTTTCCAGTGGTCTTTTCAAGGGTTAGCTTGTATTTTATTATCCTAAC

Blaine Creek ACATGTTTTATAGTTTCCAGTGGTCTTTTCAAGGGTTAGCTTGTATTTTATTATCCTAAC

CN 119300 ACATGTTTTATAGTTTCCAGTGGTCTTTTCAAGGGTTAGCTTGTATTTTATTATCCTAAC

Yellowstone ACATGTTTTATAGTTTCCAGTGGTCTTTTCAAGGGTTAGCTTGTATTTTATTATCCTAAC

Hoga ACATGTTTTATAGTTTCCAGTGGTCTTTTCAAGGGTTAGCTTGTATTTTATTATCCTAAC

CN 120027 ACATGTTTTATAGTTTCCAGTGGTCTTTTCAAGGGTTAGCTTGTATTTTATTATCCTAAC

CN 120030 ACATGTTTTATAGTTTCCAGTGGTCTTTTCAAGGGTTAGCTTGTATTTTATTATCCTAAC

CN 119294 ACATGTTTTATAGTTTCCAGTGGTCTTTTCAAGGGTTAGCTTGTATTTTATTATCCTAAC

CN 120013 ACATGTTTTATAGTTTCCAGTGGTCTTTTCAAGGGTTAGCTTGTATTTTATTATCCTAAC

CN 120017 ACATGTTTTATAGTTTCCAGTGGTCTTTTCAAGGGTTAGCTTGTATTTTATTATCCTAAC

CN 119205 ACATGTTTTATAGTTTCCAGTGGTCTTTTCAAGGGTTAGCTTGTATTTTATTATCCTAAC

DH55 ref genome ACATGTTTTATAGTTTCCAGTGGTCTTTTCAAGGGTTAGCTTGTATTTTATTATCCTAAC

09-CS0040 ACATGTTTTATAGTTTCCAGTGGTCTTTTCAAGGGTTAGCTTGTATTTTATTATCCTAAC

CN 113754 ACATGTTTTATAGTTTCCAGTGGTCTTTTCAAGGGTTAGCTTGTATTTTATTATCCTAAC

CO46 NCBI ACATGTTTTATAGTTTCCAGTGGTCTTTTCAAGGGTTAGCTTGTATTTTATTATCCTAAC

Jasper ACATGTTTTATAGTTTCCAGTGGTCTTTTCAAGGGTTAGCTTGTATTTTATTATCCTAAC

Joelle phyto ACATGTTTTATAGTTTCCAGTGGTCTTTTCAAGGGTTAGCTTGTATTTTATTATCCTAAC

Joelle NCBI ACATGTTTTATAGTTTCCAGTGGTCTTTTCAAGGGTTAGCTTGTATTTTATTATCCTAAC

CN 119243 ACATGTTTTATAGTTTCCAGTGGTCTTTTCAAGGGTTAGCTTGTATTTTATTAGCCTAAC

CN 120025 ACATGTTTTATAGTTTCCAGTGGTCTTTTCAAGGGTTAGCTTGTATTTTATTAGCCTAAC

Joelle AAFC ACATGTTTTATAGTTTCCAGTGGTCTTTTCAAGGGTTAGCTTGTATTTTATTAGCCTAAC

***************************************************** ******

CAM 241 ATACTTTTCTTTTTTGTCATCTCTCCAGCCTGGTCAAGATCCTTGATCGTTATGGGAAAC

17CS1133 ATACTTTTCTTTTTTGTCATCTCTCCAGCCTGGTCAAGATCCTTGATCGTTATGGGAAAC

CAM 236 ATACTTTTCTTTTTTGTCATCTCTCCAGCCTGGTCAAGATCCTTGATCGTTATGGGAAAC

Blaine Creek ATACTTTTCTTTTTTGTCATCTCTCCAGCCTGGTCAAGATCCTTGATCGTTATGGGAAAC

CN 119300 ATACTTTTCTTTTTTGTCATCTCTCCAGCCTGGTCAAGATCCTTGATCGTTATGGGAAAC

Yellowstone ATACTTTTCTTTTTTGTCATCTCTCCAGCCTGGTCAAGATCCTTGATCGTTATGGGAAAC

Hoga ATACTTTTCTTTTTTGTCATCTCTCCAGCCTGGTCAAGATCCTTGATCGTTATGGGAAAC

CN 120027 ATACTTTTCTTTTTTGTCATCTCTCCAGCCTGGTCAAGATCCTTGATCGTTATGGGAAAC

CN 120030 ATACTTTTCTTTTTTGTCATCTCTCCAGCCTGGTCAAGATCCTTGATCGTTATGGGAAAC

CN 119294 ATACTTTTCTTTTTTGTCATCTCTCCAGCCTGGTCAAGATCCTTGATCGTTATGGGAAAC

CN 120013 ATACTTTTCTTTTTTGTCATCTCTCCAGCCTGGTCAAGATCCTTGATCGTTATGGGAAAC

CN 120017 ATACTTTTCTTTTTTGTCATCTCTCCAGCCTGGTCAAGATCCTTGATCGTTATGGGAAAC

CN 119205 ATACTTTTCTTTTTTGTCATCTCTCCAGCCTGGTCAAGATCCTTGATCGTTATGGGAAAC

DH55 ref genome ATACTTTTCTTTTTTGTCATCTCTCCAGCCTGGTCAAGATCCTTGATCGTTATGGGAAAC

09-CS0040 ATACTTTTCTTTTTTGTCATCTCTCCAGCCTGGTCAAGATCCTTGATCGTTATGGGAAAC

CN 113754 ATACTTTTCTTTTTTGTCATCTCTCCAGCCTGGTCAAGATCCTTGATCGTTATGGGAAAC

CO46 NCBI ATACTTTTCTTTTTTGTCATCTCTCCAGCCTGGTCAAGATCCTTGATCGTTATGGGAAAC

Jasper ATACTTTTCTTTTTTGTCATCTCTCCAGCCTGGTCAAGATCCTTGATCGTTATGGGAAAC

Joelle phyto ATACTTTTCTTTTTTGTCATCTCTCCAGCCTGGTCAAGATCCTTGATCGTTATGGGAAAC

Joelle NCBI ATACTTTTCTTTTTTGTCATCTCTCCAGCCTGGTCAAGATCCTTGATCGTTATGGGAAAC

CN 119243 ATACTTTTCTTTTTTGTCATCTCTCCAGCCTGGTCAAGATCCTTGATCGTTATGGGAAAC

CN 120025 ATACTTTTCTTTTTTGTCATCTCTCCAGCCTGGTCAAGATCCTTGATCGTTATGGGAAAC

Joelle AAFC ATACTTTTCTTTTTTGTCATCTCTCCAGCCTGGTCAAGATCCTTGATCGTTATGGGAAAC

************************************************************

CAM 241 AACATGCTGATGATCTCAAAGCCTTGGTAATACAATCATTTCGAATATTTTCCCAGATGG

17CS1133 AACATGCTGATGATCTCAAAGCCTTGGTAATACAATCATTTCGAATATTTTCCCAGATGG

CAM 236 AACATGCTGATGATCTCAAAGCCTTGGTAATACAATCATTTCGAATATTTTCCCAGATGG

Blaine Creek AACATGCTGATGATCTCAAAGCCTTGGTAATACAATCATTTCGAATATTTTCCCAGATGG

CN 119300 AACATGCTGATGATCTCAAAGCCTTGGTAATACAATCATTTCGAATATTTTCCCAGATGG

Yellowstone AACATGCTGATGATCTCAAAGCCTTGGTAATACAATCATTTCGAATATTTTCCCAGATGG

Hoga AACATGCTGATGATCTCAAAGCCTTGGTAATACAATCATTTCGAATATTTTCCCAGATGG

CN 120027 AACATGCTGATGATCTCAAAGCCTTGGTAATACAATCATTTCGAATATTTTCCCAGATGG

CN 120030 AACATGCTGATGATCTCAAAGCCTTGGTAATACAATCATTTCGAATATTTTCCCAGATGG

CN 119294 AACATGCTGATGATCTCAAAGCCTTGGTAATACAATCATTTCGAATATTTTCCCAGATGG

CN 120013 AACATGCTGATGATCTCAAAGCCTTGGTAATACAATCATTTCGAATATTTTCCCAGATGG

CN 120017 AACATGCTGATGATCTCAAAGCCTTGGTAATACAATCATTTCGAATATTTTCCCAGATGG

CN 119205 AACATGCTGATGATCTCAAAGCCTTGGTAATACAATCATTTCGAATATTTTCCCAGATGG

DH55 ref genome AACATGCTGATGATCTCAAAGCCTTGGTAATACAATCATTTCGAATATTTTCCCAGATGG

09-CS0040 AACATGCTGATGATCTCAAAGCCTTGGTAATACAATCATTTCGAATATTTTCCCAGATGG

CN 113754 AACATGCTGATGATCTCAAAGCCTTGGTAATACAATCATTTCGAATATTTTCCCAGATGG

CO46 NCBI AACATGCTGATGATCTCAAAGCCTTGGTAATACAATCATTTCGAATATTTTCCCAGATGG

Jasper AACATGCTGATGATCTCAAAGCCTTGGTAATACAATCATTTCGAATATTTTCCCAGATGG

Joelle phyto AACATGCTGATGATCTCAAAGCCTTGGTAATACAATCATTTCGAATATTTTCCCAGATGG

Joelle NCBI AACATGCTGATGATCTCAAAGCCTTGGTAATACAATCATTTCGAATATTTTCCCAGATGG

CN 119243 AACATGCTGATGATCTCAAAGCCTTGGTAATACAATCATTTCGAATATTTTCCCAGATGG

CN 120025 AACATGCTGATGATCTCAAAGCCTTGGTAATACAATCATTTCGAATATTTTCCCAGATGG

Joelle AAFC AACATGCTGATGATCTCAAAGCCTTGGTAATACAATCATTTCGAATATTTTCCCAGATGG

************************************************************

CAM 241 AGTTTTAGAAGGCGTAAATTTACTAAAGACGTAGAGAGTTCAATAATCAATACTGTCAGA

17CS1133 AGTTTTAGAAGGCGTAAATTTACTAAAGACGTAGAGAGTTCAATAATCAATACTGTCAGA

CAM 236 AGTTTTAGAAGGCGTAAATTTACTAAAGACGTAGAGAGTTCAATAATCAATACTGTCAGA

Blaine Creek AGTTTTAGAAGGCGTAAATTTACTAAAGACGTAGAGAGTTCAATAATCAATACTGTCAGA

CN 119300 AGTTTTAGAAGGCGTAAATTTACTAAAGACGTAGAGAGTTCAATAATCAATACTGTCAGA

Yellowstone AGTTTTAGAAGGCGTAAATTTACTAAAGACGTAGAGAGTTCAATAATCAATACTGTCAGA

Hoga AGTTTTAGAAGGCGTAAATTTACTAAAGACGTAGAGAGTTCAATAATCAATACTGTCAGA

CN 120027 AGTTTTAGAAGGCGTAAATTTACTAAAGACGTAGAGAGTTCAATAATCAATACTGTCAGA

CN 120030 AGTTTTAGAAGGCGTAAATTTACTAAAGACGTAGAGAGTTCAATAATCAATACTGTCAGA

CN 119294 AGTTTTAGAAGGCGTAAATTTACTAAAGACGTAGAGAGTTCAATAATCAATACTGTCAGA

CN 120013 AGTTTTAGAAGGCGTAAATTTACTAAAGACGTAGAGAGTTCAATAATCAATACTGTCAGA

CN 120017 AGTTTTAGAAGGCGTAAATTTACTAAAGACGTAGAGAGTTCAATAATCAATACTGTCAGA

CN 119205 AGTTTTAGAAGGCGTAAATTTACTAAAGACGTAGAGAGTTCAATAATCAATACTGTCAGA

DH55 ref genome AGTTTTAGAAGGCGTAAATTTACTAAAGACGTAGAGAGTTCAATAATCAATACTGTCAGA

09-CS0040 AGTTTTAGAAGGCGTAAATTTACTAAAGACGTAGAGAGTTCAATAATCAATACTGTCAGA

CN 113754 AGTTTTAGAAGGCGTAAATTTACTAAAGACGTAGAGAGTTCAATAATCAATACTGTCAGA

CO46 NCBI AGTTTTAGAAGGCGTAAATTTACTAAAGACGTAGAGAGTTCAATAATCAATACTGTCAGA

Jasper AGTTTTAGAAGGCGTAAATTTACTAAAGACGTAGAGAGTTCAATAATCAATACTGTCAGA

Joelle phyto AGTTTTAGAAGGCGTAAATTTACTAAAGACGTAGAGAGTTCAATAATCAATACTGTCAGA

Joelle NCBI AGTTTTAGAAGGCGTAAATTTACTAAAGACGTAGAGAGTTCAATAATCAATACTGTCAGA

CN 119243 AGTTTTAGAAGGCGTAAATTTACTAAAGACGTAGAGAGTTCAATAATCAATACTGTCAGA

CN 120025 AGTTTTAGAAGGCGTAAATTTACTAAAGACGTAGAGAGTTCAATAATCAATACTGTCAGA

Joelle AAFC AGTTTTAGAAGGCGTAAATTTACTAAAGACGTAGAGAGTTCAATAATCAATACTGTCAGA

************************************************************

CAM 241 GCCTATTCATAGCCTCCTGTACAGCTTGCA-TTTTTAGGCATGGGCTCACAGGCATATAA

17CS1133 GCCTATTCATAGCCTCCTGTACAGCTTGCA-TTTTTAGGCATGGGCTCACAGGCATATAA

CAM 236 GCCTATTCATAGCCTCCTGTACAGCTTGCA-TTTTTAGGCATGGGCTCACAGGCATATAA

Blaine Creek GCCTATTCATAGCCTCCTGTACAGCTTGCA-TTTTTAGGCATGGGCTCACAGGCATATAA

CN 119300 GCCTATTCATAGCCTCCTGTACAGCTTGCA-TTTTTAGGCATGGGCTCACAGGCATATAA

Yellowstone GCCTATTCATAGCCTCCTGTACAGCTTGCA-TTTTTAGGCATGGGCTCACAGGCATATAA

Hoga GCCTATTCATAGCCTCCTGTACAGCTTGCA-TTTTTAGGCATGGGCTCACAGGCATATAA

CN 120027 GCCTATTCATAGCCTCCTGTACAGCTTGCA-TTTTTAGGCATGGGCTCACAGGCATATAA

CN 120030 GCCTATTCATAGCCTCCTGTACAGCTTGCA-TTTTTAGGCATGGGCTCACAGGCATATAA

CN 119294 GCCTATTCATAGCCTCCTGTACAGCTTGCA-TTTTTAGGCATGGGCTCACAGGCATATAA

CN 120013 GCCTATTCATAGCCTCCTGTACAGCTTGCA-TTTTTAGGCATGGGCTCACAGGCATATAA

CN 120017 GCCTATTCATAGCCTCCTGTACAGCTTGCA-TTTTTAGGCATGGGCTCACAGGCATATAA

CN 119205 GCCTATTCATAGCCTCCTGTACAGCTTGCA-TTTTTAGGCATGGGCTCACAGGCATATAA

DH55 ref genome GCCTATTCATAGCCTCCTGTACAGCTTGCA-TTTTTAGGCATGGGCTCACAGGCATATAA

09-CS0040 GCCTATTCATAGCCTCCTGTACAGCTTGCA-TTTTTAGGCATGGGCTCACAGGCATATAA

CN 113754 GCCTATTCATAGCCTCCTGTACAGCTTGCA-TTTTTAGGCATGGGCTCACAGGCATATAA

CO46 NCBI GCCTATTCATAGCCTCCTGTACAGCTTGCA-TTTTTAGGCATGGGCTCACAGGCATATAA

Jasper GCCTATTCATAGCCTCCTGTACAGCTTGCA-TTTTTAGGCATGGGCTCACAGGCATATAA

Joelle phyto GCCTATTCATAGCCTCCTGTACAGCTTGCA-TTTTTAGGCATGGGCTCACAGGCATATAA

Joelle NCBI GCCTATTCATAGCCTCCTGTACAGCTTGCA-TTTTTAGGCATGGGCTCACAGGCATATAA

CN 119243 GCCTATTCATAGCCTCCTGTACAGCTTGCATTTTTTAGGCATGGGCTCACAGGCATATAA

CN 120025 GCCTATTCATAGCCTCCTGTACAGCTTGCATTTTTTAGGCATGGGCTCACAGGCATATAA

Joelle AAFC GCCTATTCATAGCCTCCTGTACAGCTTGCATTTTTTAGGCATGGGCTCACAGGCATATAA

****************************** *****************************

CAM 241 CGTTTTCTCATTATAGTCACTATTTGGAGTTGTGTAGTTGTAATTGTTTAATAGATATGA

17CS1133 CGTTTTCTCATTATAGTCACTATTTGGAGTTGTGTAGTTGTAATTGTTTAATAGATATGA

CAM 236 CGTTTTCTCATTATAGTCACTATTTGGAGTTGTGTAGTTGTAATTGTTTAATAGATATGA

Blaine Creek CGTTTTCTCATTATAGTCACTATTTGGAGTTGTGTAGTTGTAATTGTTTAATAGATATGA

CN 119300 CGTTTTCTCATTATAGTCACTATTTGGAGTTGTGTAGTTGTAATTGTTTAATAGATATGA

Yellowstone CGTTTTCTCATTATAGTCACTATTTGGAGTTGTGTAGTTGTAATTGTTTAATAGATATGA

Hoga CGTTTTCTCATTATAGTCACTATTTGGAGTTGTGTAGTTGTAATTGTTTAATAGATATGA

CN 120027 CGTTTTCTCATTATAGTCACTATTTGGAGTTGTGTAGTTGTAATTGTTTAATAGATATGA

CN 120030 CGTTTTCTCATTATAGTCACTATTTGGAGTTGTGTAGTTGTAATTGTTTAATAGATATGA

CN 119294 CGTTTTCTCATTATAGTCACTATTTGGAGTTGTGTAGTTGTAATTGTTTAATAGATATGA

CN 120013 CGTTTTCTCATTATAGTCACTATTTGGAGTTGTGTAGTTGTAATTGTTTAATAGATATGA

CN 120017 CGTTTTCTCATTATAGTCACTATTTGGAGTTGTGTAGTTGTAATTGTTTAATAGATATGA

CN 119205 CGTTTTCTCATTATAGTCACTATTTGGAGTTGTGTAGTTGTAATTGTTTAATAGATATGA

DH55 ref genome CGTTTTCTCATTATAGTCACTATTTGGAGTTGTGTAGTTGTAATTGTTTAATAGATATGA

09-CS0040 CGTTTTCTCATTATAGTCACTATTTGGAGTTGTGTAGTTGTAATTGTTTAATAGATATGA

CN 113754 CGTTTTCTCATTATAGTCACTATTTGGAGTTGTGTAGTTGTAATTGTTTAATAGATATGA

CO46 NCBI CGTTTTCTCATTATAGTCACTATTTGGAGTTGTGTAGTTGTAATTGTTTAATAGATATGA

Jasper CGTTTTCTCATTATAGTCACTATTTGGAGTTGTGTAGTTGTAATTGTTTAATAGATATGA

Joelle phyto CGTTTTCTCATTATAGTCACTATTTGGAGTTGTGTAGTTGTAATTGTTTAATAGATATGA

Joelle NCBI CGTTTTCTCATTATAGTCACTATTTGGAGTTGTGTAGTTGTAATTGTTTAATAGATATGA

CN 119243 CGTTTTCTCATTATAGTCACTATTTGGAGTTGTGTAGTTGTAATTGTTTAATAGATATGA

CN 120025 CGTTTTCTCATTATAGTCACTATTTGGAGTTGTGTAGTTGTAATTGTTTAATAGATATGA

Joelle AAFC CGTTTTCTCATTATAGTCACTATTTGGAGTTGTGTAGTTGTAATTGTTTAATAGATATGA

************************************************************

CAM 241 GTAAACGCATGTTTAGCACCATAACATTGTGGAGATATTCTAGATAAAGGTATTACATCA

17CS1133 GTAAACGCATGTTTAGCACCATAACATTGTGGAGATATTCTAGATAAAGGTATTACATCA

CAM 236 GTAAACGCATGTTTAGCACCATAACATTGTGGAGATATTCTAGATAAAGGTATTACATCA

Blaine Creek GTAAACGCATGTTTAGCACCATAACATTGTGGAGATATTCTAGATAAAGGTATTACATCA

CN 119300 GTAAACGCATGTTTAGCACCATAACATTGTGGAGATATTCTAGATAAAGGTATTACATCA

Yellowstone GTAAACGCATGTTTAGCACCATAACATTGTGGAGATATTCTAGATAAAGGTATTACATCA

Hoga GTAAACGCATGTTTAGCACCATAACATTGTGGAGATATTCTAGATAAAGGTATTACATCA

CN 120027 GTAAACGCATGTTTAGCACCATAACATTGTGGAGATATTCTAGATAAAGGTATTACATCA

CN 120030 GTAAACGCATGTTTAGCACCATAACATTGTGGAGATATTCTAGATAAAGGTATTACATCA

CN 119294 GTAAACGCATGTTTAGCACCATAACATTGTGGAGATATTCTAGATAAAGGTATTACATCA

CN 120013 GTAAACGCATGTTTAGCACCATAACATTGTGGAGATATTCTAGATAAAGGTATTACATCA

CN 120017 GTAAACGCATGTTTAGCACCATAACATTGTGGAGATATTCTAGATAAAGGTATTACATCA

CN 119205 GTAAACGCATGTTTAGCACCATAACATTGTGGAGATATTCTAGATAAAGGTATTACATCA

DH55 ref genome GTAAACGCATGTTTAGCACCATAACATTGTGGAGATATTCTAGATAAAGGTATTACATCA

09-CS0040 GTAAACGCATGTTTAGCACCATAACATTGTGGAGATATTCTAGATAAAGGTATTACATCA

CN 113754 GTAAACGCATGTTTAGCACCATAACATTGTGGAGATATTCTAGATAAAGGTATTACATCA

CO46 NCBI GTAAACGCATGTTTAGCACCATAACATTGTGGAGATATTCTAGATAAAGGTATTACATCA

Jasper GTAAACGCATGTTTAGCACCATAACATTGTGGAGATATTCTAGATAAAGGTATTACATCA

Joelle phyto GTAAACGCATGTTTAGCACCATAACATTGTGGAGATATTCTAGATAAAGGTATTACATCA

Joelle NCBI GTAAACGCATGTTTAGCACCATAACATTGTGGAGATATTCTAGATAAAGGTATTACATCA

CN 119243 GTAAACGCATGTTTAGCACCATAACATTGTGGAGATATTCTAGATAAAGGTATTACATCA

CN 120025 GTAAACGCATGTTTAGCACCATAACATTGTGGAGATATTCTAGATAAAGGTATTACATCA

Joelle AAFC GTAAACGCATGTTTAGCACCATAACATTGTGGAGATATTCTAGATAAAGGTATTACATCA

************************************************************

CAM 241 CTGATCCACCATGAGCTATTAGCTTGCTAAGTGATCCTAAATATATAATGAGGTGGTGTA

17CS1133 CTGATCCACCATGAGCTATTAGCTTGCTAAGTGATCCTAAATATATAATGAGGTGGTGTA

CAM 236 CTGATCCACCATGAGCTATTAGCTTGCTAAGTGATCCTAAATATATAATGAGGTGGTGTA

Blaine Creek CTGATCCACCATGAGCTATTAGCTTGCTAAGTGATCCTAAATATATAATGAGGTGGTGTA

CN 119300 CTGATCCACCATGAGCTATTAGCTTGCTAAGTGATCCTAAATATATAATGAGGTGGTGTA

Yellowstone CTGATCCACCATGAGCTATTAGCTTGCTAAGTGATCCTAAATATATAATGAGGTGGTGTA

Hoga CTGATCCACCATGAGCTATTAGCTTGCTAAGTGATCCTAAATATATAATGAGGTGGTGTA

CN 120027 CTGATCCACCATGAGCTATTAGCTTGCTAAGTGATCCTAAATATATAATGAGGTGGTGTA

CN 120030 CTGATCCACCATGAGCTATTAGCTTGCTAAGTGATCCTAAATATATAATGAGGTGGTGTA

CN 119294 CTGATCCACCATGAGCTATTAGCTTGCTAAGTGATCCTAAATATATAATGAGGTGGTGTA

CN 120013 CTGATCCACCATGAGCTATTAGCTTGCTAAGTGATCCTAAATATATAATGAGGTGGTGTA

CN 120017 CTGATCCACCATGAGCTATTAGCTTGCTAAGTGATCCTAAATATATAATGAGGTGGTGTA

CN 119205 CTGATCCACCATGAGCTATTAGCTTGCTAAGTGATCCTAAATATATAATGAGGTGGTGTA

DH55 ref genome CTGATCCACCATGAGCTATTAGCTTGCTAAGTGATCCTAAATATATAATGAGGTGGTGTA

09-CS0040 CTGATCCACCATGAGCTATTAGCTTGCTAAGTGATCCTAAATATATAATGAGGTGGTGTA

CN 113754 CTGATCCACCATGAGCTATTAGCTTGCTAAGTGATCCTAAATATATAATGAGGTGGTGTA

CO46 NCBI CTGATCCACCATGAGCTATTAGCTTGCTAAGTGATCCTAAATATATAATGAGGTGGTGTA

Jasper CTGATCCACCATGAGCTATTAGCTTGCTAAGTGATCCTAAATATATAATGAGGTGGTGTA

Joelle phyto CTGATCCACCATGAGCTATTAGCTTGCTAAGTGATCCTAAATATATAATGAGGTGGTGTA

Joelle NCBI CTGATCCACCATGAGCTATTAGCTTGCTAAGTGATCCTAAATATATAATGAGGTGGTGTA

CN 119243 CTGATCCACCATGAGCTATTAGCTTGCTAAGTGATCCTAAATATATAATGAGGTGGTGTA

CN 120025 CTGATCCACCATGAGCTACTAGCTTGCTAAGTGATCCTAAATATATAATGAGGTGGTGTA

Joelle AAFC CTGATCCACCATGAGCTACTAGCTTGCTAAGTGATCCTAAATATATAATGAGGTGGTGTA

****************** *****************************************

CAM 241 CCAACATGCATACTGAAATCCTCGTAGGTTTTT-TTTTACCGCATCTTATATGTCCCTTT

17CS1133 CCAACATGCATACTGAAATCCTCGTAGGTTTTT-TTTTACCGCATCTTATATGTCCCTTT

CAM 236 CCAACATGCATACTGAAATCCTCGTAGGTTTTT-TTTTACCGCATCTTATATGTCCCTTT

Blaine Creek CCAACATGCATACTGAAATCCTCGTAGGTTTTT-TTTTACCGCATCTTATATGTCCCTTT

CN 119300 CCAACATGCATACTGAAATCCTCGTAGGTTTTT-TTTTACCGCATCTTATATGTCCCTTT

Yellowstone CCAACATGCATACTGAAATCCTCGTAGGTTTTT-TTTTACCGCATCTTATATGTCCCTTT

Hoga CCAACATGCATACTGAAATCCTCGTAGGTTTTT-TTTTACCGCATCTTATATGTCCCTTT

CN 120027 CCAACATGCATACTGAAATCCTCGTAGGTTTTT-TTTTACCGCATCTTATATGTCCCTTT

CN 120030 CCAACATGCATACTGAAATCCTCGTAGGTTTTT-TTTTACCGCATCTTATATGTCCCTTT

CN 119294 CCAACATGCATACTGAAATCCTCGTAGGTTTTT-TTTTACCGCATCTTATATGTCCCTTT

CN 120013 CCAACATGCATACTGAAATCCTCGTAGGTTTTT-TTTTACCGCATCTTATATGTCCCTTT

CN 120017 CCAACATGCATACTGAAATCCTCGTAGGTTTTT-TTTTACCGCATCTTATATGTCCCTTT

CN 119205 CCAACATGCATACTGAAATCCTCGTAGGTTTTT-TTTTACCGCATCTTATATGTCCCTTT

DH55 ref genome CCAACATGCATACTGAAATCCTCGTAGGTTTTT-TTTTACCGCATCTTATATGTCCCTTT

09-CS0040 CCAACATGCATACTGAAATCCTCGTAGGTTTTT-TTTTACCGCATCTTATATGTCCCTTT

CN 113754 CCAACATGCATACTGAAATCCTCGTAGGTTTTT-TTTTACCGCATCTTATATGTCCCTTT

CO46 NCBI CCAACATGCATACTGAAATCCTCGTAGGTTTTT-TTTTACCGCATCTTATATGTCCCTTT

Jasper CCAACATGCATACTGAAATCCTCGTAGGTTTTT-TTTTACCGCATCTTATATGTCCCTTT

Joelle phyto CCAACATGCATACTGAAATCCTCGTAGGTTTTT-TTTTACCGCATCTTATATGTCCCTTT

Joelle NCBI CCAACATGCATACTGAAATCCTCGTAGGTTTTT-TTTTACCGCATCTTATATGTCCCTTT

CN 119243 CCAACATGCATACTGAAATCCTCGTAGCTTTTTCTTTTACCGCATCTTATATGTCCCTTT

CN 120025 CCAACATGCATACTGAAATCCTCGTAGCTTTTTCTTTTACCGCATCTTATATGTCCCTTT

Joelle AAFC CCAACATGCATACTGAAATCCTCGTAGCTTTTTCTTTTACCGCATCTTATATGTCCCTTT

*************************** ***** **************************

CAM 241 TCTTTGGCCAGGATCTTCAGTCAAAAGCTCTGAACTATGGTTCGCACCATGAGCTACTAG

17CS1133 TCTTTGGCCAGGATCTTCAGTCAAAAGCTCTGAACTATGGTTCGCACCATGAGCTACTAG

CAM 236 TCTTTGGCCAGGATCTTCAGTCAAAAGCTCTGAACTATGGTTCGCACCATGAGCTACTAG

Blaine Creek TCTTTGGCCAGGATCTTCAGTCAAAAGCTCTGAACTATGGTTCGCACCATGAGCTACTAG

CN 119300 TCTTTGGCCAGGATCTTCAGTCAAAAGCTCTGAACTATGGTTCGCACCATGAGCTACTAG

Yellowstone TCTTTGGCCAGGATCTTCAGTCAAAAGCTCTGAACTATGGTTCGCACCATGAGCTACTAG

Hoga TCTTTGGCCAGGATCTTCAGTCAAAAGCTCTGAACTATGGTTCGCACCATGAGCTACTAG

CN 120027 TCTTTGGCCAGGATCTTCAGTCAAAAGCTCTGAACTATGGTTCGCACCATGAGCTACTAG

CN 120030 TCTTTGGCCAGGATCTTCAGTCAAAAGCTCTGAACTATGGTTCGCACCATGAGCTACTAG

CN 119294 TCTTTGGCCAGGATCTTCAGTCAAAAGCTCTGAACTATGGTTCGCACCATGAGCTACTAG

CN 120013 TCTTTGGCCAGGATCTTCAGTCAAAAGCTCTGAACTATGGTTCGCACCATGAGCTACTAG

CN 120017 TCTTTGGCCAGGATCTTCAGTCAAAAGCTCTGAACTATGGTTCGCACCATGAGCTACTAG

CN 119205 TCTTTGGCCAGGATCTTCAGTCAAAAGCTCTGAACTATGGTTCGCACCATGAGCTACTAG

DH55 ref genome TCTTTGGCCAGGATCTTCAGTCAAAAGCTCTGAACTATGGTTCGCACCATGAGCTACTAG

09-CS0040 TCTTTGGCCAGGATCTTCAGTCAAAAGCTCTGAACTATGGTTCGCACCATGAGCTACTAG

CN 113754 TCTTTGGCCAGGATCTTCAGTCAAAAGCTCTGAACTATGGTTCGCACCATGAGCTACTAG

CO46 NCBI TCTTTGGCCAGGATCTTCAGTCAAAAGCTCTGAACTATGGTTCGCACCATGAGCTACTAG

Jasper TCTTTGGCCAGGATCTTCAGTCAAAAGCTCTGAACTATGGTTCGCACCATGAGCTACTAG

Joelle phyto TCTTTGGCCAGGATCTTCAGTCAAAAGCTCTGAACTATGGTTCGCACCATGAGCTACTAG

Joelle NCBI TCTTTGGCCAGGATCTTCAGTCAAAAGCTCTGAACTATGGTTCGCACCATGAGCTACTAG

CN 119243 TCTTTGGCCAGGATCTTCAGTCAAAAGCTCTGAACTATGGTTCGCACCATGAGCTACTAG

CN 120025 TCTTTGGCCAGGATCTTCAGTCAAAAGCTCTGAACTATGGTTCGCACCATGAGCTACTAG

Joelle AAFC TCTTTGGCCAGGATCTTCAGTCAAAAGCTCTGAACTATGGTTCGCACCATGAGCTACTAG

************************************************************

CAM 241 AACTTGTGGAAAGGTTAGTACTAACTAAAACTATATTTGCTCTCCTCCTTTGAGTATAAA

17CS1133 AACTTGTGGAAAGGTTAGTACTAACTAAAACTATATTTGCTCTCCTCCTTTGAGTATAAA

CAM 236 AACTTGTGGAAAGGTTAGTACTAACTAAAACTATATTTGCTCTCCTCCTTTGAGTATAAA

Blaine Creek AACTTGTGGAAAGGTTAGTACTAACTAAAACTATATTTGCTCTCCTCCTTTGAGTATAAA

CN 119300 AACTTGTGGAAAGGTTAGTACTAACTAAAACTATATTTGCTCTCCTCCTTTGAGTATAAA

Yellowstone AACTTGTGGAAAGGTTAGTACTAACTAAAACTATATTTGCTCTCCTCCTTTGAGTATAAA

Hoga AACTTGTGGAAAGGTTAGTACTAACTAAAACTATATTTGCTCTCCTCCTTTGAGTATAAA

CN 120027 AACTTGTGGAAAGGTTAGTACTAACTAAAACTATATTTGCTCTCCTCCTTTGAGTATAAA

CN 120030 AACTTGTGGAAAGGTTAGTACTAACTAAAACTATATTTGCTCTCCTCCTTTGAGTATAAA

CN 119294 AACTTGTGGAAAGGTTAGTACTAACTAAAACTATATTTGCTCTCCTCCTTTGAGTATAAA

CN 120013 AACTTGTGGAAAGGTTAGTACTAACTAAAACTATATTTGCTCTCCTCCTTTGAGTATAAA

CN 120017 AACTTGTGGAAAGGTTAGTACTAACTAAAACTATATTTGCTCTCCTCCTTTGAGTATAAA

CN 119205 AACTTGTGGAAAGGTTAGTACTAACTAAAACTATATTTGCTCTCCTCCTTTGAGTATAAA

DH55 ref genome AACTTGTGGAAAGGTTAGTACTAACTAAAACTATATTTGCTCTCCTCCTTTGAGTATAAA

09-CS0040 AACTTGTGGAAAGGTTAGTACTAACTAAAACTATATTTGCTCTCCTCCTTTGAGTATAAA

CN 113754 AACTTGTGGAAAGGTTAGTACTAACTAAAACTATATTTGCTCTCCTCCTTTGAGTATAAA

CO46 NCBI AACTTGTGGAAAGGTTAGTACTAACTAAAACTATATTTGCTCTCCTCCTTTGAGTATAAA

Jasper AACTTGTGGAAAGGTTAGTACTAACTAAAACTATATTTGCTCTCCTCCTTTGAGTATAAA

Joelle phyto AACTTGTGGAAAGGTTAGTACTAACTAAAACTATATTTGCTCTCCTCCTTTGAGTATAAA

Joelle NCBI AACTTGTGGAAAGGTTAGTACTAACTAAAACTATATTTGCTCTCCTCCTTTGAGTATAAA

CN 119243 AACTTGTGGAAAGGTTAGTACTAACTAAAACTATATTTGCTCTCCTCCTTTGAGTATAAA

CN 120025 AACTTGTGGAAAGGTTAGTACTAACTAAAACTATATTTGCTCTCCTCCTTTGAGTATAAA

Joelle AAFC AACTTGTGGAAAGGTTAGTACTAACTAAAACTATATTTGCTCTCCTCCTTTGAGTATAAA

************************************************************

CAM 241 GGAATTAGGGTTTTCTTGTAAAACTATGAATATATGCAGCAATCTTGTGGAATCAAATGT

17CS1133 GGAATTAGGGTTTTCTTGTAAAACTATGAATATATGCAGCAATCTTGTGGAATCAAATGT

CAM 236 GGAATTAGGGTTTTCTTGTAAAACTATGAATATATGCAGCAATCTTGTGGAATCAAATGT

Blaine Creek GGAATTAGGGTTTTCTTGTAAAACTATGAATATATGCAGCAATCTTGTGGAATCAAATGT

CN 119300 GGAATTAGGGTTTTCTTGTAAAACTATGAATATATGCAGCAATCTTGTGGAATCAAATGT

Yellowstone GGAATTAGGGTTTTCTTGTAAAACTATGAATATATGCAGCAATCTTGTGGAATCAAATGT

Hoga GGAATTAGGGTTTTCTTGTAAAACTATGAATATATGCAGCAATCTTGTGGAATCAAATGT

CN 120027 GGAATTAGGGTTTTCTTGTAAAACTATGAATATATGCAGCAATCTTGTGGAATCAAATGT

CN 120030 GGAATTAGGGTTTTCTTGTAAAACTATGAATATATGCAGCAATCTTGTGGAATCAAATGT

CN 119294 GGAATTAGGGTTTTCTTGTAAAACTATGAATATATGCAGCAATCTTGTGGAATCAAATGT

CN 120013 GGAATTAGGGTTTTCTTGTAAAACTATGAATATATGCAGCAATCTTGTGGAATCAAATGT

CN 120017 GGAATTAGGGTTTTCTTGTAAAACTATGAATATATGCAGCAATCTTGTGGAATCAAATGT

CN 119205 GGAATTAGGGTTTTCTTGTAAAACTATGAATATATGCAGCAATCTTGTGGAATCAAATGT

DH55 ref genome GGAATTAGGGTTTTCTTGTAAAACTATGAATATATGCAGCAATCTTGTGGAATCAAATGT

09-CS0040 GGAATTAGGGTTTTCTTGTAAAACTATGAATATATGCAGCAATCTTGTGGAATCAAATGT

CN 113754 GGAATTAGGGTTTTCTTGTAAAACTATGAATATATGCAGCAATCTTGTGGAATCAAATGT

CO46 NCBI GGAATTAGGGTTTTCTTGTAAAACTATGAATATATGCAGCAATCTTGTGGAATCAAATGT

Jasper GGAATTAGGGTTTTCTTGTAAAACTATGAATATATGCAGCAATCTTGTGGAATCAAATGT

Joelle phyto GGAATTAGGGTTTTCTTGTAAAACTATGAATATATGCAGCAATCTTGTGGAATCAAATGT

Joelle NCBI GGAATTAGGGTTTTCTTGTAAAACTATGAATATATGCAGCAATCTTGTGGAATCAAATGT

CN 119243 GGAATTAGGGTTTTCTTGTAAAACTATGAATATATGCAGCAATCTTGTGGAATCAAATGT

CN 120025 GGAATTAGGGTTTTCTTGTAAAACTATGAATATATGCAGCAATCTTGTGGAATCAAATGT

Joelle AAFC GGAATTAGGGTTTTCTTGTAAAACTATGAATATATGCAGCAATCTTGTGGAATCAAATGT

************************************************************

CAM 241 CAACAATGTAAGTGTCGATGACCTCGTTCAACTGGAGGAACACCTTGAGACCGCCCTCTC

17CS1133 CAACAATGTAAGTGTCGATGACCTCGTTCAACTGGAGGAACACCTTGAGACCGCCCTCTC

CAM 236 CAACAATGTAAGTGTCGATGACCTCGTTCAACTGGAGGAACACCTTGAGACCGCCCTCTC

Blaine Creek CAACAATGTAAGTGTCGATGACCTCGTTCAACTGGAGGAACACCTTGAGACCGCCCTCTC

CN 119300 CAACAATGTAAGTGTCGATGACCTCGTTCAACTGGAGGAACACCTTGAGACCGCCCTCTC

Yellowstone CAACAATGTAAGTGTCGATGACCTCGTTCAACTGGAGGAACACCTTGAGACCGCCCTCTC

Hoga CAACAATGTAAGTGTCGATGACCTCGTTCAACTGGAGGAACACCTTGAGACCGCCCTCTC

CN 120027 CAACAATGTAAGTGTCGATGACCTCGTTCAACTGGAGGAACACCTTGAGACCGCCCTCTC

CN 120030 CAACAATGTAAGTGTCGATGACCTCGTTCAACTGGAGGAACACCTTGAGACCGCCCTCTC

CN 119294 CAACAATGTAAGTGTCGATGACCTCGTTCAACTGGAGGAACACCTTGAGACCGCCCTCTC

CN 120013 CAACAATGTAAGTGTCGATGACCTCGTTCAACTGGAGGAACACCTTGAGACCGCCCTCTC

CN 120017 CAACAATGTAAGTGTCGATGACCTCGTTCAACTGGAGGAACACCTTGAGACCGCCCTCTC

CN 119205 CAACAATGTAAGTGTCGATGACCTCGTTCAACTGGAGGAACACCTTGAGACCGCCCTCTC

DH55 ref genome CAACAATGTAAGTGTCGATGACCTCGTTCAACTGGAGGAACACCTTGAGACCGCCCTCTC

09-CS0040 CAACAATGTAAGTGTCGATGACCTCGTTCAACTGGAGGAACACCTTGAGACCGCCCTCTC

CN 113754 CAACAATGTAAGTGTCGATGACCTCGTTCAACTGGAGGAACACCTTGAGACCGCCCTCTC

CO46 NCBI CAACAATGTAAGTGTCGATGACCTCGTTCAACTGGAGGAACACCTTGAGACCGCCCTCTC

Jasper CAACAATGTAAGTGTCGATGACCTCGTTCAACTGGAGGAACACCTTGAGACCGCCCTCTC

Joelle phyto CAACAATGTAAGTGTCGATGACCTCGTTCAACTGGAGGAACACCTTGAGACCGCCCTCTC

Joelle NCBI CAACAATGTAAGTGTCGATGACCTCGTTCAACTGGAGGAACACCTTGAGACCGCCCTCTC

CN 119243 CAACAATGTAAGTGTCGATGACCTCGTTCAACTGGAGGAACACCTTGAGACCGCCCTCTC

CN 120025 CAACAATGTAAGTGTCGATGACCTCGTTCAACTGGAGGAACACCTTGAGACCGCCCTCTC

Joelle AAFC CAACAATGTAAGTGTCGATGACCTCGTTCAACTGGAGGAACACCTTGAGACCGCCCTCTC

************************************************************

CAM 241 CGTAACTAGAGCCAAGAAGGTAAGTGGATTCTCTAATGTCTACTCTTTTCAGAATTTGTT

17CS1133 CGTAACTAGAGCCAAGAAGGTAAGTGGATTCTCTAATGTCTACTCTTTTCAGAATTTGTT

CAM 236 CGTAACTAGAGCCAAGAAGGTAAGTGGATTCTCTAATGTCTACTCTTTTCAGAATTTGTT

Blaine Creek CGTAACTAGAGCCAAGAAGGTAAGTGGATTCTCTAATGTCTACTCTTTTCAGAATTTGTT

CN 119300 CGTAACTAGAGCCAAGAAGGTAAGTGGATTCTCTAATGTCTACTCTTTTCAGAATTTGTT

Yellowstone CGTAACTAGAGCCAAGAAGGTAAGTGGATTCTCTAATGTCTACTCTTTTCAGAATTTGTT

Hoga CGTAACTAGAGCCAAGAAGGTAAGTGGATTCTCTAATGTCTACTCTTTTCAGAATTTGTT

CN 120027 CGTAACTAGAGCCAAGAAGGTAAGTGGATTCTCTAATGTCTACTCTTTTCAGAATTTGTT

CN 120030 CGTAACTAGAGCCAAGAAGGTAAGTGGATTCTCTAATGTCTACTCTTTTCAGAATTTGTT

CN 119294 CGTAACTAGAGCCAAGAAGGTAAGTGGATTCTCTAATGTCTACTCTTTTCAGAATTTGTT

CN 120013 CGTAACTAGAGCCAAGAAGGTAAGTGGATTCTCTAATGTCTACTCTTTTCAGAATTTGTT

CN 120017 CGTAACTAGAGCCAAGAAGGTAAGTGGATTCTCTAATGTCTACTCTTTTCAGAATTTGTT

CN 119205 CGTAACTAGAGCCAAGAAGGTAAGTGGATTCTCTAATGTCTACCCTTTTCAGAATTTGTT

DH55 ref genome CGTAACTAGAGCCAAGAAGGTAAGTGGATTCTCTAATGTCTACTCTTTTCAGAATTTGTT

09-CS0040 CGTAACTAGAGCCAAGAAGGTAAGTGGATTCTCTAATGTCTACTCTTTTCAGAATTTGTT

CN 113754 CGTAACTAGAGCCAAGAAGGTAAGTGGATTCTCTAATGTCTACTCTTTTCAGAATTTGTT

CO46 NCBI CGTAACTAGAGCCAAGAAGGTAAGTGGATTCTCTAATGTCTACTCTTTTCAGAATTTGTT

Jasper CGTAACTAGAGCCAAGAAGGTAAGTGGATTCTCTAATGTCTACTCTTTTCAGAATTTGTT

Joelle phyto CGTAACTAGAGCCAAGAAGGTAAGTGGATTCTCTAATGTCTACTCTTTTCAGAATTTGTT

Joelle NCBI CGTAACTAGAGCCAAGAAGGTAAGTGGATTCTCTAATGTCTACTCTTTTCAGAATTTGTT

CN 119243 CGTAACTAGAGCCAAGAAGGTAAGTGGATTCTCTAATGTCTACTCTTTTCAGAATTTGTT

CN 120025 CGTAACTAGAGCCAAGAAGGTAAGTGGATTCTCTAATGTCTACTCTTTTCAGAATTTGTT

Joelle AAFC CGTAACTAGAGCCAAGAAGGTAAGTGGATTCTCTAATGTCTACTCTTTTCAGAATTTGTT

******************************************* ****************

CAM 241 TGCCGAGAATAACCTTATTGCTTTTGTTTGTTACAGACAGAACTAATTTTAAAGCTTGTT

17CS1133 TGCCGAGAATAACCTTATTGCTTTTGTTTGTTACAGACAGAACTAATTTTAAAGCTTGTT

CAM 236 TGCCGAGAATAACCTTATTGCTTTTGTTTGTTACAGACAGAACTAATTTTAAAGCTTGTT

Blaine Creek TGCCGAGAATAACCTTATTGCTTTTGTTTGTTACAGACAGAACTAATTTTAAAGCTTGTT

CN 119300 TGCCGAGAATAACCTTATTGCTTTTGTTTGTTACAGACAGAACTAATTTTAAAGCTTGTT

Yellowstone TGCCGAGAATAACCTTATTGCTTTTGTTTGTTACAGACAGAACTAATTTTAAAGCTTGTT

Hoga TGCCGAGAATAACCTTATTGCTTTTGTTTGTTACAGACAGAACTAATTTTAAAGCTTGTT

CN 120027 TGCCGAGAATAACCTTATTGCTTTTGTTTGTTACAGACAGAACTAATTTTAAAGCTTGTT

CN 120030 TGCCGAGAATAACCTTATTGCTTTTGTTTGTTACAGACAGAACTAATTTTAAAGCTTGTT

CN 119294 TGCCGAGAATAACCTTATTGCTTTTGTTTGTTACAGACAGAACTAATTTTAAAGCTTGTT

CN 120013 TGCCGAGAATAACCTTATTGCTTTTGTTTGTTACAGACAGAACTAATTTTAAAGCTTGTT

CN 120017 TGCCGAGAATAACCTTATTGCTTTTGTTTGTTACAGACAGAACTAATTTTAAAGCTTGTT

CN 119205 TGCCGAGAATAACCTTATTGCTTTTGTTTGTTACAGACAGAACTAATTTTAAAGCTTGTT

DH55 ref genome TGCCGAGAATAACCTTATTGCTTTTGTTTGTTACAGACAGAACTAATTTTAAAGCTTGTT

09-CS0040 TGCCGAGAATAACCTTATTGCTTTTGTTTGTTACAGACAGAACTAATTTTAAAGCTTGTT

CN 113754 TGCCGAGAATAACCTTATTGCTTTTGTTTGTTACAGACAGAACTAATTTTAAAGCTTGTT

CO46 NCBI TGCCGAGAATAACCTTATTGCTTTTGTTTGTTACAGACAGAACTAATTTTAAAGCTTGTT

Jasper TGCCGAGAATAACCTTATTGCTTTTGTTTGTTACAGACAGAACTAATTTTAAAGCTTGTT

Joelle phyto TGCCGAGAATAACCTTATTGCTTTTGTTTGTTACAGACAGAACTAATTTTAAAGCTTGTT

Joelle NCBI TGCCGAGAATAACCTTATTGCTTTTGTTTGTTACAGACAGAACTAATTTTAAAGCTTGTT

CN 119243 TGCCGAGAATAACCTTATTGCTTTTGTTTGTTACAGACAGAACTAATTTTAAAGCTTGTT

CN 120025 TGCCGAGAATAACCTTATTGCTTTTGTTTGTTACAGACAGAACTAATTTTAAAGCTTGTT

Joelle AAFC TGCCGAGAATAACCTTATTGCTTTTGTTTGTTACAGACAGAACTAATTTTAAAGCTTGTT

************************************************************

CAM 241 GAGAACCTCAAAGAAAAGGTTAGATATCTGATTCCAAGTTTAGAACATATATCAGATATA

17CS1133 GAGAACCTCAAAGAAAAGGTTAGATATCTGATTCCAAGTTTAGAACATATATCAGATATA

CAM 236 GAGAACCTCAAAGAAAAGGTTAGATATCTGATTCCAAGTTTAGAACATATATCAGATATA

Blaine Creek GAGAACCTCAAAGAAAAGGTTAGATATCTGATTCCAAGTTTAGAACATATATCAGATATA

CN 119300 GAGAACCTCAAAGAAAAGGTTAGATATCTGATTCCAAGTTTAGAACATATATCAGATATA

Yellowstone GAGAACCTCAAAGAAAAGGTTAGATATCTGATTCCAAGTTTAGAACATATATCAGATATA

Hoga GAGAACCTCAAAGAAAAGGTTAGATATCTGATTCCAAGTTTAGAACATATATCAGATATA

CN 120027 GAGAACCTCAAAGAAAAGGTTAGATATCTGATTCCAAGTTTAGAACATATATCAGATATA

CN 120030 GAGAACCTCAAAGAAAAGGTTAGATATCTGATTCCAAGTTTAGAACATATATCAGATATA

CN 119294 GAGAACCTCAAAGAAAAGGTTAGATATCTGATTCCAAGTTTAGAACATATATCAGATATA

CN 120013 GAGAACCTCAAAGAAAAGGTTAGATATCTGATTCCAAGTTTAGAACATATATCAGATATA

CN 120017 GAGAACCTCAAAGAAAAGGTTAGATATCTGATTCCAAGTTTAGAACATATATCAGATATA

CN 119205 GAGAACCTCAAAGAAAAGGTTAGATATCTGATTCCAAGTTTAGAACATATATCAGATATA

DH55 ref genome GAGAACCTCAAAGAAAAGGTTAGATATCTGATTCCAAGTTTAGAACATATATCAGATATA

09-CS0040 GAGAACCTCAAAGAAAAGGTTAGATATCTGATTCCAAGTTTAGAACATATATCAGATATA

CN 113754 GAGAACCTCAAAGAAAAGGTTAGATATCTGATTCCAAGTTTAGAACATATATCAGATATA

CO46 NCBI GAGAACCTCAAAGAAAAGGTTAGATATCTGATTCCAAGTTTAGAACATATATCAGATATA

Jasper GAGAACCTCAAAGAAAAGGTTAGATATCTGATTCCAAGTTTAGAACATATATCAGATATA

Joelle phyto GAGAACCTCAAAGAAAAGGTTAGATATCTGATTCCAAGTTTAGAACATATATCAGATATA

Joelle NCBI GAGAACCTCAAAGAAAAGGTTAGATATCTGATTCCAAGTTTAGAACATATATCAGATATA

CN 119243 GAGAACCTCAAAGAAAAGGTTAGATATCTGATTCCAAGTTTAGAACATATATCAGATATA

CN 120025 GAGAACCTCAAAGAAAAGGTTAGATATCTGATTCCAAGTTTAGAACATATATCAGATATA

Joelle AAFC GAGAACCTCAAAGAAAAGGTTAGATATCTGATTCCAAGTTTAGAACATATATCAGATATA

************************************************************

CAM 241 TACTCTAGGGTGTTTTCATTGTTTCTGTAAGTATGTTTAATGAGCTTTGCACACGTCTTT

17CS1133 TACTCTAGGGTGTTTTCATTGTTTCTGTAAGTATGTTTAATGAGCTTTGCACACGTCTTT

CAM 236 TACTCTAGGGTGTTTTCATTGTTTCTGTAAGTATGTTTAATGAGCTTTGCACACGTCTTT

Blaine Creek TACTCTAGGGTGTTTTCATTGTTTCTGTAAGTATGTTTAATGAGCTTTGCACACGTCTTT

CN 119300 TACTCTAGGGTGTTTTCATTGTTTCTGTAAGTATGTTTAATGAGCTTTGCACACGTCTTT

Yellowstone TACTCTAGGGTGTTTTCATTGTTTCTGTAAGTATGTTTAATGAGCTTTGCACACGTCTTT

Hoga TACTCTAGGGTGTTTTCATTGTTTCTGTAAGTATGTTTAATGAGCTTTGCACACGTCTTT

CN 120027 TACTCTAGGGTGTTTTCATTGTTTCTGTAAGTATGTTTAATGAGCTTTGCACACGTCTTT

CN 120030 TACTCTAGGGTGTTTTCATTGTTTCTGTAAGTATGTTTAATGAGCTTTGCACACGTCTTT

CN 119294 TACTCTAGGGTGTTTTCATTGTTTCTGTAAGTATGTTTAATGAGCTTTGCACACGTCTTT

CN 120013 TACTCTAGGGTGTTTTCATTGTTTCTGTAAGTATGTTTAATGAGCTTTGCACACGTCTTT

CN 120017 TACTCTAGGGTGTTTTCATTGTTTCTGTAAGTATGTTTAATGAGCTTTGCACACGTCTTT

CN 119205 TATTCTAGGGTGTTTTCATTGTTTCTGTAAGTATGTTTAATGAGCTTTGCACACGTCTTT

DH55 ref genome TACTCTAGGGTGTTTTCATTGTTTCTGTAAGTATGTTTAATGAGCTTTGCACACGTCTTT

09-CS0040 TACTCTAGGGTGTTTTCATTGTTTCTGTAAGTATGTTTAATGAGCTTTGCACACGTCTTT

CN 113754 TACTCTAGGGTGTTTTCATTGTTTCTGTAAGTATGTTTAATGAGCTTTGCACACGTCTTT

CO46 NCBI TACTCTAGGGTGTTTTCATTGTTTCTGTAAGTATGTTTAATGAGCTTTGCACACGTCTTT

Jasper TACTCTAGGGTGTTTTCATTGTTTCTGTAAGTATGTTTAATGAGCTTTGCACACGTCTTT

Joelle phyto TACTCTAGGGTGTTTTCATTGTTTCTGTAAGTATGTTTAATGAGCTTTGCACACGTCTTT

Joelle NCBI TACTCTAGGGTGTTTTCATTGTTTCTGTAAGTATGTTTAATGAGCTTTGCACACGTCTTT

CN 119243 TACTCTAGGGTGTTTTCATTGTTTCTGTAAGTATGTTTAATGAGCTTTGCACACGTCTTT

CN 120025 TACTCTAGGGTGTTTTCATTGTTTCTGTAAGTATGTTTAATGAGCTTTGCACACGTCTTT

Joelle AAFC TACTCTAGGGTGTTTTCATTGTTTCTGTAAGTATGTTTAATGAGCTTTGCACACGTCTTT

** *********************************************************

CAM 241 GCAACTTCTTCCCAATGCATATGTTGTGGATTTCAAAATCTGAATTTGTTAAGTTGTGAT

17CS1133 GCAACTTCTTCCCAATGCATATGTTGTGGATTTCAAAATCTGAATTTGTTAAGTTGTGAT

CAM 236 GCAACTTCTTCCCAATGCATATGTTGTGGATTTCAAAATCTGAATTTGTTAAGTTGTGAT

Blaine Creek GCAACTTCTTCCCAATGCATATGTTGTGGATTTCAAAATCTGAATTTGTTAAGTTGTGAT

CN 119300 GCAACTTCTTCCCAATGCATATGTTGTGGATTTCAAAATCTGAATTTGTTAAGTTGTGAT

Yellowstone GCAACTTCTTCCCAATGCATATGTTGTGGATTTCAAAATCTGAATTTGTTAAGTTGTGAT

Hoga GCAACTTCTTCCCAATGCATATGTTGTGGATTTCAAAATCTGAATTTGTTAAGTTGTGAT

CN 120027 GCAACTTCTTCCCAATGCATATGTTGTGGATTTCAAAATCTGAATTTGTTAAGTTGTGAT

CN 120030 GCAACTTCTTCCCAATGCATATGTTGTGGATTTCAAAATCTGAATTTGTTAAGTTGTGAT

CN 119294 GCAACTTCTTCCCAATGCATATGTTGTGGATTTCAAAATCTGAATTTGTTAAGTTGTGAT

CN 120013 GCAACTTCTTCCCAATGCATATGTTGTGGATTTCAAAATCTGAATTTGTTAAGTTGTGAT

CN 120017 GCAACTTCTTCCCAATGCATATGTTGTGGATTTCAAAATCTGAATTTGTTAAGTTGTGAT

CN 119205 GCAACTTCTTCCCAATGCATATGTTGTGGATTTCAAAATCTGAATTTGTTAAGTTGTGAT

DH55 ref genome GCAACTTCTTCCCAATGCATATGTTGTGGATTTCAAAATCTGAATTTGTTAAGTTGTGAT

09-CS0040 GCAACTTCTTCCCAATGCATATGTTGTGGATTTCAAAATCTGAATTTGTTAAGTTGTGAT

CN 113754 GCAACTTCTTCCCAATGCATATGTTGTGGATTTCAAAATCTGAATTTGTTAAGTTGTGAT

CO46 NCBI GCAACTTCTTCCCAATGCATATGTTGTGGATTTCAAAATCTGAATTTGTTAAGTTGTGAT

Jasper GCAACTTCTTCCCAATGCATATGTTGTGGATTTCAAAATCTGAATTTGTTAAGTTGTGAT

Joelle phyto GCAACTTCTTCCCAATGCATATGTTGTGGATTTCAAAATCTGAATTTGTTAAGTTGTGAT

Joelle NCBI GCAACTTCTTCCCAATGCATATGTTGTGGATTTCAAAATCTGAATTTGTTAAGTTGTGAT

CN 119243 GCAACTTCTTCCCAATGCATATGTTGTGGATTTCAAAATCTGAATTTGTTAAGTTGTGAT

CN 120025 GCAACTTCTTCCCAATGCATATGTTGTGGATTTCAAAATCTGAATTTGTTAAGTTGTGAT

Joelle AAFC GCAACTTCTTCCCAATGCATATGTTGTGGATTTCAAAATCTGAATTTGTTAAGTTGTGAT

************************************************************

CAM 241 TTGTGATTGCTGAACATGATGATCTTTAAAACAGGAGAAATTGCTGAAAGAAGAGAACCA

17CS1133 TTGTGATTGCTGAACATGATGATCTTTAAAACAGGAGAAATTGCTGAAAGAAGAGAACCA

CAM 236 TTGTGATTGCTGAACATGATGATCTTTAAAACAGGAGAAATTGCTGAAAGAAGAGAACCA

Blaine Creek TTGTGATTGCTGAACATGATGATCTTTAAAACAGGAGAAATTGCTGAAAGAAGAGAACCA

CN 119300 TTGTGATTGCTGAACATGATGATCTTTAAAACAGGAGAAATTGCTGAAAGAAGAGAACCA

Yellowstone TTGTGATTGCTGAACATGATGATCTTTAAAACAGGAGAAATTGCTGAAAGAAGAGAACCA

Hoga TTGTGATTGCTGAACATGATGATCTTTAAAACAGGAGAAATTGCTGAAAGAAGAGAACCA

CN 120027 TTGTGATTGCTGAACATGATGATCTTTAAAACAGGAGAAATTGCTGAAAGAAGAGAACCA

CN 120030 TTGTGATTGCTGAACATGATGATCTTTAAAACAGGAGAAATTGCTGAAAGAAGAGAACCA

CN 119294 TTGTGATTGCTGAACATGATGATCTTTAAAACAGGAGAAATTGCTGAAAGAAGAGAACCA

CN 120013 TTGTGATTGCTGAACATGATGATCTTTAAAACAGGAGAAATTGCTGAAAGAAGAGAACCA

CN 120017 TTGTGATTGCTGAACATGATGATCTTTAAAACAGGAGAAATTGCTGAAAGAAGAGAACCA

CN 119205 TTGTGATTGCTGAACATGATGATCTTTAAAACAGGAGAAATTGCTGAAAGAAGAGAACCA

DH55 ref genome TTGTGATTGCTGAACATGATGATCTTTAAAACAGGAGAAATTGCTGAAAGAAGAGAACCA

09-CS0040 TTGTGATTGCTGAACATGATGATCTTTAAAACAGGAGAAATTGCTGAAAGAAGAGAACCA

CN 113754 TTGTGATTGCTGAACATGATGATCTTTAAAACAGGAGAAATTGCTGAAAGAAGAGAACCA

CO46 NCBI TTGTGATTGCTGAACATGATGATCTTTAAAACAGGAGAAATTGCTGAAAGAAGAGAACCA

Jasper TTGTGATTGCTGAACATGATGATCTTTAAAACAGGAGAAATTGCTGAAAGAAGAGAACCA

Joelle phyto TTGTGATTGCTGAACATGATGATCTTTAAAACAGGAGAAATTGCTGAAAGAAGAGAACCA

Joelle NCBI TTGTGATTGCTGAACATGATGATCTTTAAAACAGGAGAAATTGCTGAAAGAAGAGAACCA

CN 119243 TTGTGATTGCTGAACATGATGATCTTTAAAACAGGAGAAATTGCTGAAAGAAGAGAACCA

CN 120025 TTGTGATTGCTGAACATGATGATCTTTAAAACAGGAGAAATTGCTGAAAGAAGAGAACCA

Joelle AAFC TTGTGATTGCTGAACATGATGATCTTTAAAACAGGAGAAATTGCTGAAAGAAGAGAACCA

************************************************************

CAM 241 GGTTTTGGCTAGGCAGGTAACGAAAGCTACTTTTTCTATATATATGCATACCTAATAAGC

17CS1133 GGTTTTGGCTAGGCAGGTAACGAAAGCTACTTTTTCTATATATATGCATACCTAATAAGC

CAM 236 GGTTTTGGCTAGGCAGGTAACGAAAGCTACTTTTTCTATATATATGCATACCTAATAAGC

Blaine Creek GGTTTTGGCTAGGCAGGTAACGAAAGCTACTTTTTCTATATATATGCATACCTAATAAGC

CN 119300 GGTTTTGGCTAGGCAGGTAACGAAAGCTACTTTTTCTATATATATGCATACCTAATAAGC

Yellowstone GGTTTTGGCTAGGCAGGTAACGAAAGCTACTTTTTCTATATATATGCATACCTAATAAGC

Hoga GGTTTTGGCTAGGCAGGTAACGAAAGCTACTTTTTCTATATATATGCATACCTAATAAGC

CN 120027 GGTTTTGGCTAGGCAGGTAACGAAAGCTACTTTTTCTATATATATGCATACCTAATAAGC

CN 120030 GGTTTTGGCTAGGCAGGTAACGAAAGCTACTTTTTCTATATATATGCATACCTAATAAGC

CN 119294 GGTTTTGGCTAGGCAGGTAACGAAAGCTACTTTTTCTATATATATGCATACCTAATAAGC

CN 120013 GGTTTTGGCTAGGCAGGTAACGAAAGCTACTTTTTCTATATATATGCATACCTAATAAGC

CN 120017 GGTTTTGGCTAGGCAGGTAACGAAAGCTACTTTTTCTATATATATGCATACCTAATAAGC

CN 119205 GGTTTTGGCTAGGCAGGTAACGAAAGCTACTTTTTCTATATATATGCATACCTAATAAGC

DH55 ref genome GGTTTTGGCTAGGCAGGTAACGAAAGCTACTTTTTCTATATATATGCATACCTAATAAGC

09-CS0040 GGTTTTGGCTAGGCAGGTAACGAAAGCTACTTTTTCTATATATATGCATACCTAATAAGC

CN 113754 GGTTTTGGCTAGGCAGGTAACGAAAGCTACTTTTTCTATATATATGCATACCTAATAAGC

CO46 NCBI GGTTTTGGCTAGGCAGGTAACGAAAGCTACTTTTTCTATATATATGCATACCTAATAAGC

Jasper GGTTTTGGCTAGGCAGGTAACGAAAGCTACTTTTTCTATATATATGCATACCTAATAAGC

Joelle phyto GGTTTTGGCTAGGCAGGTAACGAAAGCTACTTTTTCTATATATATGCATACCTAATAAGC

Joelle NCBI GGTTTTGGCTAGGCAGGTAACGAAAGCTACTTTTTCTATATATATGCATACCTAATAAGC

CN 119243 GGTTTTGGCTAGGCAGGTAACGAAAGCTACTTTTTCTATATATATGCATACCTAATAAGC

CN 120025 GGTTTTGGCTAGGCAGGTAACGAAAGCTACTTTTTCTATATATATGCATACCTAATAAGC

Joelle AAFC GGTTTTGGCTAGGCAGGTAACGAAAGCTACTTTTTCTATATATATGCATACCTAATAAGC

************************************************************

CAM 241 CATTCTTTTCACTTTAAGCATTTACTATATAGTTAACACTTCTCTGTCTTGTATTTTAGA

17CS1133 CATTCTTTTCACTTTAAGCATTTACTATATAGTTAACACTTCTCTGTCTTGTATTTTAGA

CAM 236 CATTCTTTTCACTTTAAGCATTTACTATATAGTTAACACTTCTCTGTCTTGTATTTTAGA

Blaine Creek CATTCTTTTCACTTTAAGCATTTACTATATAGTTAACACTTCTCTGTCTTGTATTTTAGA

CN 119300 CATTCTTTTCACTTTAAGCATTTACTATATAGTTAACACTTCTCTGTCTTGTATTTTAGA

Yellowstone CATTCTTTTCACTTTAAGCATTTACTATATAGTTAACACTTCTCTGTCTTGTATTTTAGA

Hoga CATTCTTTTCACTTTAAGCATTTACTATATAGTTAACACTTCTCTGTCTTGTATTTTAGA

CN 120027 CATTCTTTTCACTTTAAGCATTTACTATATAGTTAACACTTCTCTGTCTTGTATTTTAGA

CN 120030 CATTCTTTTCACTTTAAGCATTTACTATATAGTTAACACTTCTCTGTCTTGTATTTTAGA

CN 119294 CATTCTTTTCACTTTAAGCATTTACTATATAGTTAACACTTCTCTGTCTTGTATTTTAGA

CN 120013 CATTCTTTTCACTTTAAGCATTTACTATATAGTTAACACTTCTCTGTCTTGTATTTTAGA

CN 120017 CATTCTTTTCACTTTAAGCATTTACTATATAGTTAACACTTCTCTGTCTTGTATTTTAGA

CN 119205 CATTCTTTTCACTTTAAGCATTTACTATATAGTTAACACTTCTCTGTCTTGTATTTTAGA

DH55 ref genome CATTCTTTTCACTTTAAGCATTTACTATATAGTTAACACTTCTCTGTCTTGTATTTTAGA

09-CS0040 CATTCTTTTCACTTTAAGCATTTACTATATAGTTAACACTTCTCTGTCTTGTATTTTAGA

CN 113754 CATTCTTTTCACTTTAAGCATTTACTATATAGTTAACACTTCTCTGTCTTGTATTTTAGA

CO46 NCBI CATTCTTTTCACTTTAAGCATTTACTATATAGTTAACACTTCTCTGTCTTGTATTTTAGA

Jasper CATTCTTTTCACTTTAAGCATTTACTATATAGTTAACACTTCTCTGTCTTGTATTTTAGA

Joelle phyto CATTCTTTTCACTTTAAGCATTTACTATATAGTTAACACTTCTCTGTCTTGTATTTTAGA

Joelle NCBI CATTCTTTTCACTTTAAGCATTTACTATATAGTTAACACTTCTCTGTCTTGTATTTTAGA

CN 119243 CATTCTTTTCACTTTAAGCATTTACTATATAGTTAACACTTCTCTGTCTTGTATTTTAGA

CN 120025 CATTCTTTTCACTTTAAGCATTTACTATATAGTTAACACTTCTCTGTCTTGTATTTTAGA

Joelle AAFC CATTCTTTTCACTTTAAGCATTTACTATATAGTTAACACTTCTCTGTCTTGTATTTTAGA

************************************************************

CAM 241 AAATTTATTTCTCAAGATTTTGGTTGTTGGTAGATTAGGTATTAGGGTTTGTGAGATTAT

17CS1133 AAATTTATTTCTCAAGATTTTGGTTGTTGGTAGATTAGGTATTAGGGTTTGTGAGATTAT

CAM 236 AAATTTATTTCTCAAGATTTTGGTTGTTGGTAGATTAGGTATTAGGGTTTGTGAGATTAT

Blaine Creek AAATTTATTTCTCAAGATTTTGGTTGTTGGTAGATTAGGTATTAGGGTTTGTGAGATTAT

CN 119300 AAATTTATTTCTCAAGATTTTGGTTGTTGGTAGATTAGGTATTAGGGTTTGTGAGATTAT

Yellowstone AAATTTATTTCTCAAGATTTTGGTTGTTGGTAGATTAGGTATTAGGGTTTGTGAGATTAT

Hoga AAATTTATTTCTCAAGATTTTGGTTGTTGGTAGATTAGGTATTAGGGTTTGTGAGATTAT

CN 120027 AAATTTATTTCTCAAGATTTTGGTTGTTGGTAGATTAGGTATTAGGGTTTGTGAGATTAT

CN 120030 AAATTTATTTCTCAAGATTTTGGTTGTTGGTAGATTAGGTATTAGGGTTTGTGAGATTAT

CN 119294 AAATTTATTTCTCAAGATTTTGGTTGTTGGTAGATTAGGTATTAGGGTTTGTGAGATTAT

CN 120013 AAATTTATTTCTCAAGATTTTGGTTGTTGGTAGATTAGGTATTAGGGTTTGTGAGATTAT

CN 120017 AAATTTATTTCTCAAGATTTTGGTTGTTGGTAGATTAGGTATTAGGGTTTGTGAGATTAT

CN 119205 AAATTTATTTCTCAAGATTTTGGTTGTTGGTAGATTAGGTATTAGGGTTTGTGAGATTAT

DH55 ref genome AAATTTATTTCTCAAGATTTTGGTTGTTGGTAGATTAGGTATTAGGGTTTGTGAGATTAT

09-CS0040 AAATTTATTTCTCAAGATTTTGGTTGTTGGTAGATTAGGTATTAGGGTTTGTGAGATTAT

CN 113754 AAATTTATTTCTCAAGATTTTGGTTGTTGGTAGATTAGGTATTAGGGTTTGTGAGATTAT

CO46 NCBI AAATTTATTTCTCAAGATTTTGGTTGTTGGTAGATTAGGTATTAGGGTTTGTGAGATTAT

Jasper AAATTTATTTCTCAAGATTTTGGTTGTTGGTAGATTAGGTATTAGGGTTTGTGAGATTAT

Joelle phyto AAATTTATTTCTCAAGATTTTGGTTGTTGGTAGATTAGGTATTAGGGTTTGTGAGATTAT

Joelle NCBI AAATTTATTTCTCAAGATTTTGGTTGTTGGTAGATTAGGTATTAGGGTTTGTGAGATTAT

CN 119243 AAATTTATTTCTCAAGATTTTGGTTGTTGGTAGATTAGGTATTAGGGTTTGTGAGATTAT

CN 120025 AAATTTATTTCTCAAGATTTTGGTTGTTGGTAGATTAGGTATTAGGGTTTGTGAGATTAT

Joelle AAFC AAATTTATTTCTCAAGATTTTGGTTGTTGGTAGATTAGGTATTAGGGTTTGTGAGATTAT

************************************************************

CAM 241 TACTGCTGAATATATAAGGAGAGAAGAAAATTGATTCGGTCTGGTTTGAGTTAAGGGAAA

17CS1133 TACTGCTGAATATATAAGGAGAGAAGAAAATTGATTCGGTCTGGTTTGAGTTAAGGGAAA

CAM 236 TACTGCTGAATATATAAGGAGAGAAGAAAATTGATTCGGTCTGGTTTGAGTTAAGGGAAA

Blaine Creek TACTGCTGAATATATAAGGAGAGAAGAAAATTGATTCGGTCTGGTTTGAGTTAAGGGAAA

CN 119300 TACTGCTGAATATATAAGGAGAGAAGAAAATTGATTCGGTCTGGTTTGAGTTAAGGGAAA

Yellowstone TACTGCTGAATATATAAGGAGAGAAGAAAATTGATTCGGTCTGGTTTGAGTTAAGGGAAA

Hoga TACTGCTGAATATATAAGGAGAGAAGAAAATTGATTCGGTCTGGTTTGAGTTAAGGGAAA

CN 120027 TACTGCTGAATATATAAGGAGAGAAGAAAATTGATTCGGTCTGGTTTGAGTTAAGGGAAA

CN 120030 TACTGCTGAATATATAAGGAGAGAAGAAAATTGATTCGGTCTGGTTTGAGTTAAGGGAAA

CN 119294 TACTGCTGAATATATAAGGAGAGAAGAAAATTGATTCGGTCTGGTTTGAGTTAAGGGAAA

CN 120013 TACTGCTGAATATATAAGGAGAGAAGAAAATTGATTCGGTCTGGTTTGAGTTAAGGGAAA

CN 120017 TACTGCTGAATATATAAGGAGAGAAGAAAATTGATTCGGTCTGGTTTGAGTTAAGGGAAA

CN 119205 TACTGCTGAATATATAAGGAGAGAAGAAAATTGATTCGGTCTGGTTTGAGTTAAGGGAAA

DH55 ref genome TACTGCTGAATATATAAGGAGAGAAGAAAATTGATTCGGTCTGGTTTGAGTTAAGGGAAA

09-CS0040 TACTGCTGAATATATAAGGAGAGAAGAAAATTGATTCGGTCTGGTTTGAGTTAAGGGAAA

CN 113754 TACTGCTGAATATATAAGGAGAGAAGAAAATTGATTCGGTCTGGTTTGAGTTAAGGGAAA

CO46 NCBI TACTGCTGAATATATAAGGAGAGAAGAAAATTGATTCGGTCTGGTTTGAGTTAAGGGAAA

Jasper TACTGCTGAATATATAAGGAGAGAAGAAAATTGATTCGGTCTGGTTTGAGTTAAGGGAAA

Joelle phyto TACTGCTGAATATATAAGGAGAGAAGAAAATTGATTCGGTCTGGTTTGAGTTAAGGGAAA

Joelle NCBI TACTGCTGAATATATAAGGAGAGAAGAAAATTGATTCGGTCTGGTTTGAGTTAAGGGAAA

CN 119243 TACTGCTGAATATATAAGGAG---AGAAAATTGATTCGGTCTGGTTTGAGTTAAGGGAAA

CN 120025 TACTGCTGAATATATAAGGAG---AGAAAATTGATTCGGTCTGGTTTGAGTTAAGGGAAA

Joelle AAFC TACTGCTGAATATATAAGGAG---AGAAAATTGATTCGGTCTGGTTTGAGTTAAGGGAAA

********************* ************************************

CAM 241 ACTTTGATTCGGATTTTAGAATGACAGATGAAAAGTTTAATTTTAAGATAGTTATTTTAT

17CS1133 ACTTTGATTCGGATTTTAGAATGACAGATGAAAAGTTTAATTTTAAGATAGTTATTTTAT

CAM 236 ACTTTGATTCGGATTTTAGAATGACAGATGAAAAGTTTAATTTTAAGATAGTTATTTTAT

Blaine Creek ACTTTGATTCGGATTTTAGAATGACAGATGAAAAGTTTAATTTTAAGATAGTTATTTTAT

CN 119300 ACTTTGATTCGGATTTTAGAATGACAGATGAAAAGTTTAATTTTAAGATAGTTATTTTAT

Yellowstone ACTTTGATTCGGATTTTAGAATGACAGATGAAAAGTTTAATTTTAAGATAGTTATTTTAT

Hoga ACTTTGATTCGGATTTTAGAATGACAGATGAAAAGTTTAATTTTAAGATAGTTATTTTAT

CN 120027 ACTTTGATTCGGATTTTAGAATGACAGATGAAAAGTTTAATTTTAAGATAGTTATTTTAT

CN 120030 ACTTTGATTCGGATTTTAGAATGACAGATGAAAAGTTTAATTTTAAGATAGTTATTTTAT

CN 119294 ACTTTGATTCGGATTTTAGAATGACAGATGAAAAGTTTAATTTTAAGATAGTTATTTTAT

CN 120013 ACTTTGATTCGGATTTTAGAATGACAGATGAAAAGTTTAATTTTAAGATAGTTATTTTAT

CN 120017 ACTTTGATTCGGATTTTAGAATGACAGATGAAAAGTTTAATTTTAAGATAGTTATTTTAT

CN 119205 ACTTTGATTCGGATTTTAGAATGACAGATGAAAAGTTTAATTTTAAGATAGTTATTTTAT

DH55 ref genome ACTTTGATTCGGATTTTAGAATGACAGATGAAAAGTTTAATTTTAAGATAGTTATTTTAT

09-CS0040 ACTTTGATTCGGATTTTAGAATGACAGATGAAAAGTTTAATTTTAAGATAGTTATTTTAT

CN 113754 ACTTTGATTCGGATTTTAGAATGACAGATGAAAAGTTTAATTTTAAGATAGTTATTTTAT

CO46 NCBI ACTTTGATTCGGATTTTAGAATGACAGATGAAAAGTTTAATTTTAAGATAGTTATTTTAT

Jasper ACTTTGATTCGGATTTTAGAATGACAGATGAAAAGTTTAATTTTAAGATAGTTATTTTAT

Joelle phyto ACTTTGATTCGGATTTTAGAATGACAGATGAAAAGTTTAATTTTAAGATAGTTATTTTAT

Joelle NCBI ACTTTGATTCGGATTTTAGAATGACAGATGAAAAGTTTAATTTTAAGATAGTTATTTTAT

CN 119243 ACTTTGATTCGGATTTTAGAATGACAGATGAAAAGTTTAATTTTAAGATAGTTATTTTAT

CN 120025 ACTTTGATTCGGATTTTAGAATGACAGATGAAAAGTTTAATTTTAAGATAGTTATTTTAT

Joelle AAFC ACTTTGATTCGGATTTTAGAATGACAGATGAAAAGTTTAATTTTAAGATAGTTATTTTAT

************************************************************

CAM 241 TGATAAAAGACACACAAACAAACAAAATTCATGAACAGTATATAAATTCATTATTACATG

17CS1133 TGATAAAAGACACACAAACAAACAAAATTCATGAACAGTATATAAATTCATTATTACATG

CAM 236 TGATAAAAGACACACAAACAAACAAAATTCATGAACAGTATATAAATTCATTATTACATG

Blaine Creek TGATAAAAGACACACAAACAAACAAAATTCATGAACAGTATATAAATTCATTATTACATG

CN 119300 TGATAAAAGACACACAAACAAACAAAATTCATGAACAGTATATAAATTCATTATTACATG

Yellowstone TGATAAAAGACACACAAACAAACAAAATTCATGAACAGTATATAAATTCATTATTACATG

Hoga TGATAAAAGACACACAAACAAACAAAATTCATGAACAGTATATAAATTCATTATTACATG

CN 120027 TGATAAAAGACACACAAACAAACAAAATTCATGAACAGTATATAAATTCATTATTACATG

CN 120030 TGATAAAAGACACACAAACAAACAAAATTCATGAACAGTATATAAATTCATTATTACATG

CN 119294 TGATAAAAGACACACAAACAAACAAAATTCATGAACAGTATATAAATTCATTATTACATG

CN 120013 TGATAAAAGACACACAAACAAACAAAATTCATGAACAGTATATAAATTCATTATTACATG

CN 120017 TGATAAAAGACACACAAACAAACAAAATTCATGAACAGTATATAAATTCATTATTACATG

CN 119205 TGATAAAAGACACACAAACAAACAAAATTCATGAACAGTATATAAATTCATTATTACATG

DH55 ref genome TGATAAAAGACACACAAACAAACAAAATTCATGAACAGTATATAAATTCATTATTACATG

09-CS0040 TGATAAAAGACACACAAACAAACAAAATTCATGAACAGTATATAAATTCATTATTACATG

CN 113754 TGATAAAAGACACACAAACAAACAAAATTCATGAACAGTATATAAATTCATTATTACATG

CO46 NCBI TGATAAAAGACACACAAACAAACAAAATTCATGAACAGTATATAAATTCATTATTACATG

Jasper TGATAAAAGACACACAAACAAACAAAATTCATGAACAGTATATAAATTCATTATTACATG

Joelle phyto TGATAAAAGACACACAAACAAACAAAATTCATGAACAGTATATAAATTCATTATTACATG

Joelle NCBI TGATAAAAGACACACAAACAAACAAAATTCATGAACAGTATATAAATTCATTATTACATG

CN 119243 TGATAAAAGACACACAAACAAACAAAATTCATGAACAGTATATAAATTCATTATTACATG

CN 120025 TGATAAAAGACACACAAACAAACAAAATTCATGAACAGTATATAAATTCATTATTACATG

Joelle AAFC TGATAAAAGACACACAAACAAACAAAATTCATGAACAGTATATAAATTCATTATTACATG

************************************************************

CAM 241 ATAAATTCATTATTACATGATAATTACATGAACAATATATTTTTGTAACGATTATTGCTA

17CS1133 ATAAATTCATTATTACATGATAATTACATGAACAATATATTTTTGTAACGATTATTGCTA

CAM 236 ATAAATTCATTATTACATGATAATTACATGAACAATATATTTTTGTAACGATTATTGCTA

Blaine Creek ATAAATTCATTATTACATGATAATTACATGAACAATATATTTTTGTAACGATTATTGCTA

CN 119300 ATAAATTCATTATTACATGATAATTACATGAACAATATATTTTTGTAACGATTATTGCTA

Yellowstone ATAAATTCATTATTACATGATAATTACATGAACAATATATTTTTGTAACGATTATTGCTA

Hoga ATAAATTCATTATTACATGATAATTACATGAACAATATATTTTTGTAACGATTATTGCTA

CN 120027 ATAAATTCATTATTACATGATAATTACATGAACAATATATTTTTGTAACGATTATTGCTA

CN 120030 ATAAATTCATTATTACATGATAATTACATGAACAATATATTTTTGTAACGATTATTGCTA

CN 119294 ATAAATTCATTATTACATGATAATTACATGAACAATATATTTTTGTAACGATTATTGCTA

CN 120013 ATAAATTCATTATTACATGATAATTACATGAACAATATATTTTTGTAACGATTATTGCTA

CN 120017 ATAAATTCATTATTACATGATAATTACATGAACAATATATTTTTGTAACGATTATTGCTA

CN 119205 ATAAATTCATTATTACATGATAATTACATGAACAATATATTTTTGTAACGATTATTGCTA

DH55 ref genome ATAAATTCATTATTACATGATAATTACATGAACAATATATTTTTGTAACGATTATTGCTA

09-CS0040 ATAAATTCATTATTACATGATAATTACATGAACAATATATTTTTGTAACGATTATTGCTA

CN 113754 ATAAATTCATTATTACATGATAATTACATGAACAATATATTTTTGTAACGATTATTGCTA

CO46 NCBI ATAAATTCATTATTACATGATAATTACATGAACAATATATTTTTGTAACGATTATTGCTA

Jasper ATAAATTCATTATTACATGATAATTACATGAACAATATATTTTTGTAACGATTATTGCTA

Joelle phyto ATAAATTCATTATTACATGATAATTACATGAACAATATATTTTTGTAACGATTATTGCTA

Joelle NCBI ATAAATTCATTATTACATGATAATTACATGAACAATATATTTTTGTAACGATTATTGCTA

CN 119243 ATAAATTCATTATTACATGATAATTACATGAACAATATATTTTTGTAACGATTATTGCTA

CN 120025 ATAAATTCATTATTACATGATAATTACATGAACAATATATTTTTGTAACGATTATTGCTA

Joelle AAFC ATAAATTCATTATTACATGATAATTACATGAACAATATATTTTTGTAACGATTATTGCTA

************************************************************

CAM 241 TATCATTGTACTAAAATAATATATACTTTTCTGTTGGTGGAAATAATATTAGGTTTTGGT

17CS1133 TATCATTGTACTAAAATAATATATACTTTTCTGTTGGTGGAAATAATATTAGGTTTTGGT

CAM 236 TATCATTGTACTAAAATAATATATACTTTTCTGTTGGTGGAAATAATATTAGGTTTTGGT

Blaine Creek TATCATTGTACTAAAATAATATATACTTTTCTGTTGGTGGAAATAATATTAGGTTTTGGT

CN 119300 TATCATTGTACTAAAATAATATATACTTTTCTGTTGGTGGAAATAATATTAGGTTTTGGT

Yellowstone TATCATTGTACTAAAATAATATATACTTTTCTGTTGGTGGAAATAATATTAGGTTTTGGT

Hoga TATCATTGTACTAAAATAATATATACTTTTCTGTTGGTGGAAATAATATTAGGTTTTGGT

CN 120027 TATCATTGTACTAAAATAATATATACTTTTCTGTTGGTGGAAATAATATTAGGTTTTGGT

CN 120030 TATCATTGTACTAAAATAATATATACTTTTCTGTTGGTGGAAATAATATTAGGTTTTGGT

CN 119294 TATCATTGTACTAAAATAATATATACTTTTCTGTTGGTGGAAATAATATTAGGTTTTGGT

CN 120013 TATCATTGTACTAAAATAATATATACTTTTCTGTTGGTGGAAATAATATTAGGTTTTGGT

CN 120017 TATCATTGTACTAAAATAATATATACTTTTCTGTTGGTGGAAATAATATTAGGTTTTGGT

CN 119205 TATCATTTTACTAAAATAATATATACTTTTCTGTTGGTGGAAATAATATTAGGTTTTGGT

DH55 ref genome TATCATTGTACTAAAATAATATATACTTTTCTGTTGGTGGAAATAATATTAGGTTTTGGT

09-CS0040 TATCATTGTACTAAAATAATATATACTTTTCTGTTGGTGGAAATAATATTAGGTTTTGGT

CN 113754 TATCATTGTACTAAAATAATATATACTTTTCTGTTGGTGGAAATAATATTAGGTTTTGGT

CO46 NCBI TATCATTGTACTAAAATAATATATACTTTTCTGTTGGTGGAAATAATATTAGGTTTTGGT

Jasper TATCATTGTACTAAAATAATATATACTTTTCTGTTGGTGGAAATAATATTAGGTTTTGGT

Joelle phyto TATCATTGTACTAAAATAATATATACTTTTCTGTTGGTGGAAATAATATTAGGTTTTGGT

Joelle NCBI TATCATTGTACTAAAATAATATATACTTTTCTGTTGGTGGAAATAATATTAGGTTTTGGT

CN 119243 TATCATTGTACTAAAATAATATATACTTTTCTGTTGGTGGAAATAATATTAGGTTTTGGT

CN 120025 TATCATTGTACTAAAATAATATATACTTTTCTGTTGGTGGAAATAATATTAGGTTTTGGT

Joelle AAFC TATCATTGTACTAAAATAATATATACTTTTCTGTTGGTGGAAATAATATTAGGTTTTGGT

******* ****************************************************

CAM 241 TGTTC-TTTTTCTGGATTTGGGATAAAGGTTTTAGTTAGGTTTTGGTTCAGTTTGATATT

17CS1133 TGTTC-TTTTTCTGGATTTGGGATAAAGGTTTTAGTTAGGTTTTGGTTCAGTTTGATATT

CAM 236 TGTTC-TTTTTCTGGATTTGGGATAAAGGTTTTAGTTAGGTTTTGGTTCAGTTTGATATT

Blaine Creek TGTTC-TTTTTCTGGATTTGGGATAAAGGTTTTAGTTAGGTTTTGGTTCAGTTTGATATT

CN 119300 TGTTC-TTTTTCTGGATTTGGGATAAAGGTTTTAGTTAGGTTTTGGTTCAGTTTGATATT

Yellowstone TGTTC-TTTTTCTGGATTTGGGATAAAGGTTTTAGTTAGGTTTTGGTTCAGTTTGATATT

Hoga TGTTC-TTTTTCTGGATTTGGGATAAAGGTTTTAGTTAGGTTTTGGTTCAGTTTGATATT

CN 120027 TGTTC-TTTTTCTGGATTTGGGATAAAGGTTTTAGTTAGGTTTTGGTTCAGTTTGATATT

CN 120030 TGTTC-TTTTTCTGGATTTGGGATAAAGGTTTTAGTTAGGTTTTGGTTCAGTTTGATATT

CN 119294 TGTTC-TTTTTCTGGATTTGGGATAAAGGTTTTAGTTAGGTTTTGGTTCAGTTTGATATT

CN 120013 TGTTC-TTTTTCTGGATTTGGGATAAAGGTTTTAGTTAGGTTTTGGTTCAGTTTGATATT

CN 120017 TGTTC-TTTTTCTGGATTTGGGATAAAGGTTTTAGTTAGGTTTTGGTTCAGTTTGATATT

CN 119205 TGTTC-TTTTTCTGGATTTGGGATAAAGGTTTTAGTTAGGTTTTGGTTCAGTTTGATATT

DH55 ref genome TGTTC-TTTTTCTGGATTTGGGATAAAGGTTTTAGTTAGGTTTTGGTTCAGTTTGATATT

09-CS0040 TGTTC-TTTTTCTGGATTTGGGATAAAGGTTTTAGTTAGGTTTTGGTTCAGTTTGATATT

CN 113754 TGTTC-TTTTTCTGGATTTGGGATAAAGGTTTTAGTTAGGTTTTGGTTCAGTTTGATATT

CO46 NCBI TGTTC-TTTTTCTGGATTTGGGATAAAGGTTTTAGTTAGGTTTTGGTTCAGTTTGATATT

Jasper TGTTC-TTTTTCTGGATTTGGGATAAAGGTTTTAGTTAGGTTTTGGTTCAGTTTGATATT

Joelle phyto TGTTC-TTTTTCTGGATTTGGGATAAAGGTTTTAGTTAGGTTTTGGTTCAGTTTGATATT

Joelle NCBI TGTTC-TTTTTCTGGATTTGGGATAAAGGTTTTAGTTAGGTTTTGGTTCAGTTTGATATT

CN 119243 TGTTCTTTTTTCTGGATTTGGGATAAAGGTTTTAGTTAGGTTTTGGTTCAGTTTGATATT

CN 120025 TGTTCTTTTTTCTGGATTTGGGATAAAGGTTTTAGTTAGGTTTTGGTTCAGTTTGATATT

Joelle AAFC TGTTCTTTTTTCTGGATTTGGGATAAAGGTTTTAGTTAGGTTTTGGTTCAGTTTGATATT

***** ******************************************************

CAM 241 TACGAACGGATTGGTTTTTTGTGCGTCATGGTTAAGGTTTGGATCTATGTGTCCAGTAGT

17CS1133 TACGAACGGATTGGTTTTTTGTGCGTCATGGTTAAGGTTTGGATCTATGTGTCCAGTAGT

CAM 236 TACGAACGGATTGGTTTTTTGTGCGTCATGGTTAAGGTTTGGATCTATGTGTCCAGTAGT

Blaine Creek TACGAACGGATTGGTTTTTTGTGCGTCATGGTTAAGGTTTGGATCTATGTGTCCAGTAGT

CN 119300 TACGAACGGATTGGTTTTTTGTGCGTCATGGTTAAGGTTTGGATCTATGTGTCCAGTAGT

Yellowstone TACGAACGGATTGGTTTTTTGTGCGTCATGGTTAAGGTTTGGATCTATGTGTCCAGTAGT

Hoga TACGAACGGATTGGTTTTTTGTGCGTCATGGTTAAGGTTTGGATCTATGTGTCCAGTAGT

CN 120027 TACGAACGGATTGGTTTTTTGTGCGTCATGGTTAAGGTTTGGATCTATGTGTCCAGTAGT

CN 120030 TACGAACGGATTGGTTTTTTGTGCGTCATGGTTAAGGTTTGGATCTATGTGTCCAGTAGT

CN 119294 TACGAACGGATTGGTTTTTTGTGCGTCATGGTTAAGGTTTGGATCTATGTGTCCAGTAGT

CN 120013 TACGAACGGATTGGTTTTTTGTGCGTCATGGTTAAGGTTTGGATCTATGTGTCCAGTAGT

CN 120017 TACGAACGGATTGGTTTTTTGTGCGTCATGGTTAAGGTTTGGATCTATGTGTCCAGTAGT

CN 119205 TACGAACGGATTGGTTTTTTGTGCGTCATGGTTAAGGTTTGGATCTATGTGTCCAGTAGT

DH55 ref genome TACGAACGGATTGGTTTTTTGTGCGTCATGGTTAAGGTTTGGATCTATGTGTCCAGTAGT

09-CS0040 TACGAACGGATTGGTTTTTTGTGCGTCATGGTTAAGGTTTGGATCTATGTGTCCAGTAGT

CN 113754 TACGAACGGATTGGTTTTTTGTGCGTCATGGTTAAGGTTTGGATCTATGTGTCCAGTAGT

CO46 NCBI TACGAACGGATTGGTTTTTTGTGCGTCATGGTTAAGGTTTGGATCTATGTGTCCAGTAGT

Jasper TACGAACGGATTGGTTTTTTGTGCGTCATGGTTAAGGTTTGGATCTATGTGTCCAGTAGT

Joelle phyto TACGAACGGATTGGTTTTTTGTGCGTCATGGTTAAGGTTTGGATCTATGTGTCCAGTAGT

Joelle NCBI TACGAACGGATTGGTTTTTTGTGCGTCATGGTTAAGGTTTGGATCTATGTGTCCAGTAGT

CN 119243 TACGAACGGATTAGTTTTTTGTGCGTCATGGTTAAGGTTTGGATCTATGTGTCCAGTAGT

CN 120025 TACGAACGGATTAGTTTTTTGTGCGTCATGGTTAAGGTTTGGATCTATGTGTCCAGTAGT

Joelle AAFC TACGAACGGATTAGTTTTTTGTGCGTCATGGTTAAGGTTTGGATCTATGTGTCCAGTAGT

************ ***********************************************

CAM 241 CTAGCTACTTTTACATCTTCAAGGTTAAATTATAAACAGGGAAGTAGTCTAAAACAGACA

17CS1133 CTAGCTACTTTTACATCTTCAAGGTTAAATTATAAACAGGGAAGTAGTCTAAAACAGACA

CAM 236 CTAGCTACTTTTACATCTTCAAGGTTAAATTATAAACAGGGAAGTAGTCTAAAACAGACA

Blaine Creek CTAGCTACTTTTACATCTTCAAGGTTAAATTATAAACAGGGAAGTAGTCTAAAACAGACA

CN 119300 CTAGCTACTTTTACATCTTCAAGGTTAAATTATAAACAGGGAAGTAGTCTAAAACAGACA

Yellowstone CTAGCTACTTTTACATCTTCAAGGTTAAATTATAAACAGGGAAGTAGTCTAAAACAGACA

Hoga CTAGCTACTTTTACATCTTCAAGGTTAAATTATAAACAGGGAAGTAGTCTAAAACAGACA

CN 120027 CTAGCTACTTTTACATCTTCAAGGTTAAATTATAAACAGGGAAGTAGTCTAAAACAGACA

CN 120030 CTAGCTACTTTTACATCTTCAAGGTTAAATTATAAACAGGGAAGTAGTCTAAAACAGACA

CN 119294 CTAGCTACTTTTACATCTTCAAGGTTAAATTATAAACAGGGAAGTAGTCTAAAACAGACA

CN 120013 CTAGCTACTTTTACATCTTCAAGGTTAAATTATAAACAGGGAAGTAGTCTAAAACAGACA

CN 120017 CTAGCTACTTTTACATCTTCAAGGTTAAATTATAAACAGGGAAGTAGTCTAAAACAGACA

CN 119205 CTAGCTACTTTTACATCTTCAAGGTTAAATTATAAACAGGGAAGTAGTCTAAAACAGACA

DH55 ref genome CTAGCTACTTTTACATCTTCAAGGTTAAATTATAAACAGGGAAGTAGTCTAAAACAGACA

09-CS0040 CTAGCTACTTTTACATCTTCAAGGTTAAATTATAAACAGGGAAGTAGTCTAAAACAGACA

CN 113754 CTAGCTACTTTTACATCTTCAAGGTTAAATTATAAACAGGGAAGTAGTCTAAAACAGACA

CO46 NCBI CTAGCTACTTTTACATCTTCAAGGTTAAATTATAAACAGGGAAGTAGTCTAAAACAGACA

Jasper CTAGCTACTTTTACATCTTCAAGGTTAAATTATAAACAGGGAAGTAGTCTAAAACAGACA

Joelle phyto CTAGCTACTTTTACATCTTCAAGGTTAAATTATAAACAGGGAAGTAGTCTAAAACAGACA

Joelle NCBI CTAGCTACTTTTACATCTTCAAGGTTAAATTATAAACAGGGAAGTAGTCTAAAACAGACA

CN 119243 CTAGCTACTTTTACATCTTCAAGGTT---------------------------------A

CN 120025 CTAGCTACTTTTACATCTTCAAGGTT---------------------------------A

Joelle AAFC CTAGCTACTTTTACATCTTCAAGGTT---------------------------------A

************************** *

CAM 241 AATTATGGAAGCAATATGGTGGAACGAGTGTTAGTCTTAAATCGGTGGAATGAAAAGAAT

17CS1133 AATTATGGAAGCAATATGGTGGAACGAGTGTTAGTCTTAAATCGGTGGAATGAAAAGAAT

CAM 236 AATTATGGAAGCAATATGGTGGAACGAGTGTTAGTCTTAAATCGGTGGAATGAAAAGAAT

Blaine Creek AATTATGGAAGCAATATGGTGGAACGAGTGTTAGTCTTAAATCGGTGGAATGAAAAGAAT

CN 119300 AATTATGGAAGCAATATGGTGGAACGAGTGTTAGTCTTAAATCGGTGGAATGAAAAGAAT

Yellowstone AATTATGGAAGCAATATGGTGGAACGAGTGTTAGTCTTAAATCGGTGGAATGAAAAGAAT

Hoga AATTATGGAAGCAATATGGTGGAACGAGTGTTAGTCTTAAATCGGTGGAATGAAAAGAAT

CN 120027 AATTATGGAAGCAATATGGTGGAACGAGTGTTAGTCTTAAATCGGTGGAATGAAAAGAAT

CN 120030 AATTATGGAAGCAATATGGTGGAACGAGTGTTAGTCTTAAATCGGTGGAATGAAAAGAAT

CN 119294 AATTATGGAAGCAATATGGTGGAACGAGTGTTAGTCTTAAATCGGTGGAATGAAAAGAAT

CN 120013 AATTATGGAAGCAATATGGTGGAACGAGTGTTAGTCTTAAATCGGTGGAATGAAAAGAAT

CN 120017 AATTATGGAAGCAATATGGTGGAACGAGTGTTAGTCTTAAATCGGTGGAATGAAAAGAAT

CN 119205 AATTATGGAAGCAATATGGTGGAACGAGTGTTAGTCTTAAATCGGTGGAATGAAAAGAAT

DH55 ref genome AATTATGGAAGCAATATGGTGGAACGAGTGTTAGTCTTAAATCGGTGGAATGAAAAGAAT

09-CS0040 AATTATGGAAGCAATATGGTGGAACGAGTGTTAGTCTTAAATCGGTGGAATGAAAAGAAT

CN 113754 AATTATGGAAGCAATATGGTGGAACGAGTGTTAGTCTTAAATCGGTGGAATGAAAAGAAT

CO46 NCBI AATTATGGAAGCAATATGGTGGAACGAGTGTTAGTCTTAAATCGGTGGAATGAAAAGAAT

Jasper AATTATGGAAGCAATATGGTGGAACGAGTGTTAGTCTTAAATCGGTGGAATGAAAAGAAT

Joelle phyto AATTATGGAAGCAATATGGTGGAACGAGTGTTAGTCTTAAATCGGTGGAATGAAAAGAAT

Joelle NCBI AATTATGGAAGCAATATGGTGGAACGAGTGTTAGTCTTAAATCGGTGGAATGAAAAGAAT

CN 119243 AATTATGGAAGCAATATGGTGGAACGAGTGTTAGTCTTAAATCGGTGGAATGAAAAGAAT

CN 120025 AATTATGGAAGCAATATGGTGGAACGAGTGTTAGTCTTAAATCGGTGGAATGAAAAGAAT

Joelle AAFC AATTATGGAAGCAATATGGTGGAACGAGTGTTAGTCTTAAATCGGTGGAATGAAAAGAAT

************************************************************

CAM 241 TTTCATTTTCATATATATGAGTAGAAGACAAAAAGAAAAAAGTGAATAATGATTTTGACC

17CS1133 TTTCATTTTCATATATATGAGTAGAAGACAAAAAGAAAAAAGTGAATAATGATTTTGACC

CAM 236 TTTCATTTTCATATATATGAGTAGAAGACAAAAAGAAAAAAGTGAATAATGATTTTGACC

Blaine Creek TTTCATTTTCATATATATGAGTAGAAGACAAAAAGAAAAAAGTGAATAATGATTTTGACC

CN 119300 TTTCATTTTCATATATATGAGTAGAAGACAAAAAGAAAAAAGTGAATAATGATTTTGACC

Yellowstone TTTCATTTTCATATATATGAGTAGAAGACAAAAAGAAAAAAGTGAATAATGATTTTGACC

Hoga TTTCATTTTCATATATATGAGTAGAAGACAAAAAGAAAAAAGTGAATAATGATTTTGACC

CN 120027 TTTCATTTTCATATATATGAGTAGAAGACAAAAAGAAAAAAGTGAATAATGATTTTGACC

CN 120030 TTTCATTTTCATATATATGAGTAGAAGACAAAAAGAAAAAAGTGAATAATGATTTTGACC

CN 119294 TTTCATTTTCATATATATGAGTAGAAGACAAAAAGAAAAAAGTGAATAATGATTTTGACC

CN 120013 TTTCATTTTCATATATATGAGTAGAAGACAAAAAGAAAAAAGTGAATAATGATTTTGACC

CN 120017 TTTCATTTTCATATATATGAGTAGAAGACAAAAAGAAAAAAGTGAATAATGATTTTGACC

CN 119205 TTTCATTTTCATATATATGAGTAGAAGACAAAAAGAAAAAAGTGAATAATGATTTTGACC

DH55 ref genome TTTCATTTTCATATATATGAGTAGAAGACAAAAAGAAAAAAGTGAATAATGATTTTGACC

09-CS0040 TTTCATTTTCATATATATGAGTAGAAGACAAAAAGAAAAAAGTGAATAATGATTTTGACC

CN 113754 TTTCATTTTCATATATATGAGTAGAAGACAAAAAGAAAAAAGTGAATAATGATTTTGACC

CO46 NCBI TTTCATTTTCATATATATGAGTAGAAGACAAAAAGAAAAAAGTGAATAATGATTTTGACC

Jasper TTTCATTTTCATATATATGAGTAGAAGACAAAAAGAAAAAAGTGAATAATGATTTTGACC

Joelle phyto TTTCATTTTCATATATATGAGTAGAAGACAAAAAGAAAAAAGTGAATAATGATTTTGACC

Joelle NCBI TTTCATTTTCATATATATGAGTAGAAGACAAAAAGAAAAAAGTGAATAATGATTTTGACC

CN 119243 TTTCATTTTCATATATATGAGTAGAAGACAAAAAGAAAAAAGTGAATAATGATTTTGACC

CN 120025 TTTCATTTTCATATATATGAGTAGAAGACCAAAAGAAAAAAGTGAATAATGATTTTGACC

Joelle AAFC TTTCATTTTCATATATATGAGTAGAAGACCAAAAGAAAAAAGTGAATAATGATTTTGACC

***************************** ******************************

CAM 241 TATGATTATCGTACAGATGGAGACGAATCATGTTGTTGGGGCAGAAGCTGATATGGAGAT

17CS1133 TATGATTATCGTACAGATGGAGACGAATCATGTTGTTGGGGCAGAAGCTGATATGGAGAT

CAM 236 TATGATTATCGTACAGATGGAGACGAATCATGTTGTTGGGGCAGAAGCTGATATGGAGAT

Blaine Creek TATGATTATCGTACAGATGGAGACGAATCATGTTGTTGGGGCAGAAGCTGATATGGAGAT

CN 119300 TATGATTATCGTACAGATGGAGACGAATCATGTTGTTGGGGCAGAAGCTGATATGGAGAT

Yellowstone TATGATTATCGTACAGATGGAGACGAATCATGTTGTTGGGGCAGAAGCTGATATGGAGAT

Hoga TATGATTATCGTACAGATGGAGACGAATCATGTTGTTGGGGCAGAAGCTGATATGGAGAT

CN 120027 TATGATTATCGTACAGATGGAGACGAATCATGTTGTTGGGGCAGAAGCTGATATGGAGAT

CN 120030 TATGATTATCGTACAGATGGAGACGAATCATGTTGTTGGGGCAGAAGCTGATATGGAGAT

CN 119294 TATGATTATCGTACAGATGGAGACGAATCATGTTGTTGGGGCAGAAGCTGATATGGAGAT

CN 120013 TATGATTATCGTACAGATGGAGACGAATCATGTTGTTGGGGCAGAAGCTGATATGGAGAT

CN 120017 TATGATTATCGTACAGATGGAGACGAATCATGTTGTTGGGGCAGAAGCTGATATGGAGAT

CN 119205 TATGATTATCGTACAGATGGAGACGAATCATGTTGTTGGGGCAGAAGCTGATATGGAGAT

DH55 ref genome TATGATTATCGTACAGATGGAGACGAATCATGTTGTTGGGGCAGAAGCTGATATGGAGAT

09-CS0040 TATGATTATCGTACAGATGGAGACGAATCATGTTGTTGGGGCAGAAGCTGATATGGAGAT

CN 113754 TATGATTATCGTACAGATGGAGACGAATCATGTTGTTGGGGCAGAAGCTGATATGGAGAT

CO46 NCBI TATGATTATCGTACAGATGGAGACGAATCATGTTGTTGGGGCAGAAGCTGATATGGAGAT

Jasper TATGATTATCGTACAGATGGAGACGAATCATGTTGTTGGGGCAGAAGCTGATATGGAGAT

Joelle phyto TATGATTATCGTACAGATGGAGACGAATCATGTTGTTGGGGCAGAAGCTGATATGGAGAT

Joelle NCBI TATGATTATCGTACAGATGGAGACGAATCATGTTGTTGGGGCAGAAGCTGATATGGAGAT

CN 119243 TATGATTATCGTACAGATGGAGACGAATCATGTTGTTGGGGCAGAAGCTGATATGGAGAT

CN 120025 TATGATTATCGTACAGATGGAGACGAATCATGTTGTTGGGGCAGAAGCTGATATGGAGAT

Joelle AAFC TATGATTATCGTACAGATGGAGACGAATCATGTTGTTGGGGCAGAAGCTGATATGGAGAT

************************************************************

CAM 241 GGAGATGTCACCTGCTGGACAAATCTCCGACAATCTACCGGTGACTCTCCCGCTGCTCAA

17CS1133 GGAGATGTCACCTGCTGGACAAATCTCCGACAATCTACCGGTGACTCTCCCGCTGCTCAA

CAM 236 GGAGATGTCACCTGCTGGACAAATCTCCGACAATCTACCGGTGACTCTCCCGCTGCTCAA

Blaine Creek GGAGATGTCACCTGCTGGACAAATCTCCGACAATCTACCGGTGACTCTCCCGCTGCTCAA

CN 119300 GGAGATGTCACCTGCTGGACAAATCTCCGACAATCTACCGGTGACTCTCCCGCTGCTCAA

Yellowstone GGAGATGTCACCTGCTGGACAAATCTCCGACAATCTACCGGTGACTCTCCCGCTGCTCAA

Hoga GGAGATGTCACCTGCTGGACAAATCTCCGACAATCTACCGGTGACTCTCCCGCTGCTCAA

CN 120027 GGAGATGTCACCTGCTGGACAAATCTCCGACAATCTACCGGTGACTCTCCCGCTGCTCAA

CN 120030 GGAGATGTCACCTGCTGGACAAATCTCCGACAATCTACCGGTGACTCTCCCGCTGCTCAA

CN 119294 GGAGATGTCACCTGCTGGACAAATCTCCGACAATCTACCGGTGACTCTCCCGCTGCTCAA

CN 120013 GGAGATGTCACCTGCTGGACAAATCTCCGACAATCTACCGGTGACTCTCCCGCTGCTCAA

CN 120017 GGAGATGTCACCTGCTGGACAAATCTCCGACAATCTACCGGTGACTCTCCCGCTGCTCAA

CN 119205 GGAGATGTCACCTGCTGGACAAATCTCCGACAATCTACCGGTGACTCTCCCGCTGCTCAA

DH55 ref genome GGAGATGTCACCTGCTGGACAAATCTCCGACAATCTTCCGGTGACTCTCCCGCTGCTCAA

09-CS0040 GGAGATGTCACCTGCTGGACAAATCTCCGACAATCTACCGGTGACTCTCCCGCTGCTCAA

CN 113754 GGAGATGTCACCTGCTGGACAAATCTCCGACAATCTACCGGTGACTCTCCCGCTGCTCAA

CO46 NCBI GGAGATGTCACCTGCTGGACAAATCTCCGACAATCTACCGGTGACTCTCCCGCTGCTCAA

Jasper GGAGATGTCACCTGCTGGACAAATCTCCGACAATCTACCGGTGACTCTCCCGCTGCTCAA

Joelle phyto GGAGATGTCACCTGCTGGACAAATCTCCGACAATCTACCGGTGACTCTCCCGCTGCTCAA

Joelle NCBI GGAGATGTCACCTGCTGGACAAATCTCCGACAATCTACCGGTGACTCTCCCGCTGCTCAA

CN 119243 GGAGATGTCACCTGCTGGACAAATCTCCGACAATCTACCGGTGACTCTCCCGCTGCTCAA

CN 120025 GGAGATGTCACCTGCTGGACAAATCTCCGACAATCTACCGGTGACTCTCCCGCTGCTCAA

Joelle AAFC GGAGATGTCACCTGCTGGACAAATCTCCGACAATCTACCGGTGACTCTCCCGCTGCTCAA

************************************ ***********************

CAM 241 TTAGGGCGGTTGATATCAAAAATCCAAAACATATATATAATTAAGGGAAGAAAAAAAAAA

17CS1133 TTAGGGCGGTTGATATCAAAAATCCAAAACATATATATAATTAAGGGAAGAAAAAAAAAA

CAM 236 TTAGGGCGGTTGATATCAAAAATCCAAAACATATATATAATTAAGGGAAGAAAAAAAAAA

Blaine Creek TTAGGGCGGTTGATATCAAAAATCCAAAACATATATATAATTAAGGGAAGAAAAAAAAAA

CN 119300 TTAGGGCGGTTGATATCAAAAATCCAAAACATATATATAATTAAGGGAAGAAAAAAAAAA

Yellowstone TTAGGGCGGTTGATATCAAAAATCCAAAACATATATATAATTAAGGGAAGAAAAAAAAAA

Hoga TTAGGGCGGTTGATATCAAAAATCCAAAACATATATATAATTAAGGGAAGAAAAAAAAAA

CN 120027 TTAGGGCGGTTGATATCAAAAATCCAAAACATATATATAATTAAGGGAAGAAAAAAAAAA

CN 120030 TTAGGGCGGTTGATATCAAAAATCCAAAACATATATATAATTAAGGGAAGAAAAAAAAAA

CN 119294 TTAGGGCGGTTGATATCAAAAATCCAAAACATATATATAATTAAGGGAAGAAAAAAAAAA

CN 120013 TTAGGGCGGTTGATATCAAAAATCCAAAACATATATATAATTAAGGGAAGAAAAAAAAAA

CN 120017 TTAGGGCGGTTGATATCAAAAATCCAAAACATATATATAATTAAGGGAAGAAAAAAAAAA

CN 119205 TTAGGGCGGTTGATATCAAAAATCCAAAACATATATATAATTAAGGGAAGAAAAAAAAAA

DH55 ref genome TTAGGGCGGTTGATATCAAAAATCCAAAACATATATATAATTAAGGGAAGAAAAAAAAAA

09-CS0040 TTAGGGCGGTTGATATCAAAAATCCAAAACATATATATAATTAAGGGAAGAAAAAAAAAA

CN 113754 TTAGGGCGGTTGATATCAAAAATCCAAAACATATATATAATTAAGGGAAGAAAAAAAAAA

CO46 NCBI TTAGGGCGGTTGATATCAAAAATCCAAAACATATATATAATTAAGGGAAGAAAAAAAAAA

Jasper TTAGGGCGGTTGATATCAAAAATCCAAAACATATATATAATTAAGGGAAGAAAAAAAAAA

Joelle phyto TTAGGGCGGTTGATATCAAAAATCCAAAACATATATATAATTAAGGGAAGAAAAAAAAAA

Joelle NCBI TTAGGGCGGTTGATATCAAAAATCCAAAACATATATATAATTAAGGGAAGAAAAAAAAAA

CN 119243 TTAGGGCAGTTGATATCAAAAATCCAAAACATATATATAATTAAGGGAAGAAAAAAAAAT

CN 120025 TTAGGGCAGTTGATATCAAAAATCCAAAACATATATATAATTAAGGGAAGAAAAAAAAAT

Joelle AAFC TTAGGGCAGTTGATATCAAAAATCCAAAACATATATATAATTAAGGGAAGAAAAAAAAAT

******* ***************************************************

CAM 241 AATATGTAATTATTCCGCTGATAACGGCGCGTGGTATACTGTTATGTATGTATATCTTAA

17CS1133 AATATGTAATTATTCCGCTGATAACGGCGCGTGGTATACTGTTATGTATGTATATCTTAA

CAM 236 AATATGTAATTATTCCGCTGATAACGGCGCGTGGTATACTGTTATGTATGTATATCTTAA

Blaine Creek AATATGTAATTATTCCGCTGATAACGGCGCGTGGTATACTGTTATGTATGTATATCTTAA

CN 119300 AATATGTAATTATTCCGCTGATAACGGCGCGTGGTATACTGTTATGTATGTATATCTTAA

Yellowstone AATATGTAATTATTCCGCTGATAACGGCGCGTGGTATACTGTTATGTATGTATATCTTAA

Hoga AATATGTAATTATTCCGCTGATAACGGCGCGTGGTATACTGTTATGTATGTATATCTTAA

CN 120027 AATATGTAATTATTCCGCTGATAACGGCGCGTGGTATACTGTTATGTATGTATATCTTAA

CN 120030 AATATGTAATTATTCCGCTGATAACGGCGCGTGGTATACTGTTATGTATGTATATCTTAA

CN 119294 AATATGTAATTATTCCGCTGATAACGGCGCGTGGTATACTGTTATGTATGTATATCTTAA

CN 120013 AATATGTAATTATTCCGCTGATAACGGCGCGTGGTATACTGTTATGTATGTATATCTTAA

CN 120017 TATATGTAATTATTCCGCTGATAACGGCGCGTGGTATACTGTTATGTATGTATATCTTAA

CN 119205 TATATGTAATTATTCCGCTGATAACGGCGCGTGGTATACTGTTATGTATGTATATCTTAA

DH55 ref genome AATATGTAATTATTCCGCTGATAACGGCGCGTGGTATACTGTTATGTATGTATATCTTAA

09-CS0040 AATATGTAATTATTCCGCTGATAACGGCGCGTGGTATACTGTTATGTATGTATATCTTAA

CN 113754 AATATGTAATTATTCCGCTGATAACGGCGCGTGGTATACTGTTATGTATGTATATCTTAA

CO46 NCBI AATATGTAATTATTCCGCTGATAACGGCGCGTGGTATACTGTTATGTATGTATATCTTAA

Jasper AATATGTAATTATTCCGCTGATAACGGCGCGTGGTATACTGTTATGTATGTATATCTTAA

Joelle phyto TATATGTAATTATTCCGCTGATAACGGCGCGTGGTATACTGTTATGTATGTATATCTTAA

Joelle NCBI TATATGTAATTATTCCGCTGATAACGGCGCGTGGTATACTGTTATGTATGTATATCTTAA

CN 119243 TATATGTAATTATTCCGCTGATAACGGCGCGTGGTATACTGTTATGTATGTATATCTTAA

CN 120025 TATATGTAATTATTCCGCTGATAACGGCGCGTGGTATACTGTTATGTATGTATATCTTAA

Joelle AAFC TATATGTAATTATTCCGCTGATAACGGCGCGTGGTATACTGTTATGTATGTATATCTTAA

***********************************************************

CAM 241 CACTCCCTC--------CTCTCTCTCTCT---TCTCTCTCTCTTTGGCTTGTGTGTGATA

17CS1133 CACTCCCTC--------CTCTCTCTCTCT---TCTCTCTCTCTTTGGCTTGTGTGTGATA

CAM 236 CACTCCCTC--------CTCTCTCTCTCT---TCTCTCTCTCTTTGGCTTGTGTGTGATA

Blaine Creek CACTCCCTC--------CTCTCTCTCTCT---TCTCTCTCTCTTTGGCTTGTGTGTGATA

CN 119300 CACTCCCTC--------CTCTCTCTCTCT---TCTCTCTCTCTTTGGCTTGTGTGTGATA

Yellowstone CACTCCCTC--------CTCTCTCTCTCT---TCTCTCTCTCTTTGGCTTGTGTGTGATA

Hoga CACTCCCTC--------CTCTCTCTCTCT---TCTCTCTCTCTTTGGCTTGTGTGTGATA

CN 120027 CACTCCCTC--------CTCTCTCTCTCT---TCTCTCTCTCTTTGGCTTGTGTGTGATA

CN 120030 CACTCCCTC--------CTCTCTCTCTCT---TCTCTCTCTCTTTGGCTTGTGTGTGATA

CN 119294 CACTCCCTC--------CTCTCTCTCTCT---TCTCTCTCTCTTTGGCTTGTGTGTGATA

CN 120013 CACTCCCTC--------CTCTCTCTCTCT---TCTCTCTCTCTTTGGCTTGTGTGTGATA

CN 120017 CACTCCCTC--------CTCTCTCTCTCT---TCTCTCTCTCTTTGGCTTGTGTGTGATA

CN 119205 CACTCCCTC--------CTCTCTCTCTCT---TCTCTCTCTCTTTGGCTTGTGTGTGATA

DH55 ref genome CACTCCCTC--------CTCTCTCTCTCT---TCTCTCTCTCTTTGGCTTGTGTGTGATA

09-CS0040 CACTCCCTC--------CTCTCTCTCTCT---TCTCTCTCTCTTTGGCTTGTGTGTGATA

CN 113754 CACTCCCTC--------CTCTCTCTCTCT---TCTCTCTCTCTTTGGCTTGTGTGTGATA

CO46 NCBI CACTCCCTC--------CTCTCTCTCTCT---TCTCTCTCTCTTTGGCTTGTGTGTGATA

Jasper CACTCCCTC--------CTCTCTCTCTCT---TCTCTCTCTCTTTGGCTTGTGTGTGATA

Joelle phyto CACTCCCTC--------CTCTCTCTCTCT---TCTCTCTCTCTTTGGCTTGTGTGTGATA

Joelle NCBI CACTCCCTC--------CTCTCTCTCTCT---TCTCTCTCTCTTTGGCTTGTGTGTGATA

CN 119243 CACTCCCTCCTCA----CTCTCTCTCTCTCTCTCTCTCTCTCTCTGGCTTGTGTGTGATA

CN 120025 CACTCCCTCCTCACTCTCTCTCTCTCTCTCTCTCTCTCTCTCTCTGGCTTGTGTGTGATA

Joelle AAFC CACTCCCTCCTCACT--CTCTCTCTCTCTCTCTCTCTCTCTCTCTGGCTTGTGTGTGATA

********* ************ *********** ****************

CAM 241 CTTAAAAGTAGATTGAAAGTCAAATATTATCTGTTTAAGACAGAACTATGATGAAACTTT

17CS1133 CTTAAAAGTAGATTGAAAGTCAAATATTATCTGTTTAAGACAGAACTATGATGAAACTTT

CAM 236 CTTAAAAGTAGATTGAAAGTCAAATATTATCTGTTTAAGACAGAACTATGATGAAACTTT

Blaine Creek CTTAAAAGTAGATTGAAAGTCAAATATTATCTGTTTAAGACAGAACTATGATGAAACTTT

CN 119300 CTTAAAAGTAGATTGAAAGTCAAATATTATCTGTTTAAGACAGAACTATGATGAAACTTT

Yellowstone CTTAAAAGTAGATTGAAAGTCAAATATTATCTGTTTAAGACAGAACTATGATGAAACTTT

Hoga CTTAAAAGTAGATTGAAAGTCAAATATTATCTGTTTAAGACAGAACTATGATGAAACTTT

CN 120027 CTTAAAAGTAGATTGAAAGTCAAATATTATCTGTTTAAGACAGAACTATGATGAAACTTT

CN 120030 CTTAAAAGTAGATTGAAAGTCAAATATTATCTGTTTAAGACAGAACTATGATGAAACTTT

CN 119294 CTTAAAAGTAGATTGAAAGTCAAATATTATCTGTTTAAGACAGAACTATGATGAAACTTT

CN 120013 CTTAAAAGTAGATTGAAAGTCAAATATTATCTGTTTAAGACAGAACTATGATGAAACTTT

CN 120017 CTTAAAAGTAGATTGAAAGTCAAATATTATCTGTTTAAGACAGAACTATGATGAAACTTT

CN 119205 CTTAAAAGTAGATTGAAAGTCAAATATTATCTGTTTAAGACAGAACTATGATGAAACTTT

DH55 ref genome CTTAAAAGTAGATTGAAAGTCAAATATTATCTGTTTAAGACAGAACTATGATGAAACTTT

09-CS0040 CTTAAAAGTAGATTGAAAGTCAAATATTATCTGTTTAAGACAGAACTATGATGAAACTTT

CN 113754 CTTAAAAGTAGATTGAAAGTCAAATATTATCTGTTTAAGACAGAACTATGATGAAACTTT

CO46 NCBI CTTAAAAGTAGATTGAAAGTCAAATATTATCTGTTTAAGACAGAACTATGATGAAACTTT

Jasper CTTAAAAGTAGATTGAAAGTCAAATATTATCTGTTTAAGACAGAACTATGATGAAACTTT

Joelle phyto CTTAAAAGTAGATTGAAAGTCAAATATTATCTGTTTAAGACAGAACTATGATGAAACTTT

Joelle NCBI CTTAAAAGTAGATTGAAAGTCAAATATTATCTGTTTAAGACAGAACTATGATGAAACTTT

CN 119243 CTTAAAAGTAGATTGAAAGTCAAATATTATCTGTTTAAGACAGAACTATGATGAAACTTT

CN 120025 CTTAAAAGTAGATTGAAAGTCAAATATTATCTGTTTAAGACAGAACTATGATGAAACTTT

Joelle AAFC CTTAAAAGTAGATTGAAAGTCAAATATTATCTGTTTAAGACAGAACTATGATGAAACTTT

************************************************************

CAM 241 GTACCTTATTCGAGAGAGCTTTGCATCGAGATGTTGCTGTGTGTGTTCTCCTCTTCTGTC

17CS1133 GTACCTTATTCGAGAGAGCTTTGCATCGAGATGTTGCTGTGTGTGTTCTCCTCTTCTGTC

CAM 236 GTACCTTATTCGAGAGAGCTTTGCATCGAGATGTTGCTGTGTGTGTTCTCCTCTTCTGTC

Blaine Creek GTACCTTATTCGAGAGAGCTTTGCATCGAGATGTTGCTGTGTGTGTTCTCCTCTTCTGTC

CN 119300 GTACCTTATTCGAGAGAGCTTTGCATCGAGATGTTGCTGTGTGTGTTCTCCTCTTCTGTC

Yellowstone GTACCTTATTCGAGAGAGCTTTGCATCGAGATGTTGCTGTGTGTGTTCTCCTCTTCTGTC

Hoga GTACCTTATTCGAGAGAGCTTTGCATCGAGATGTTGCTGTGTGTGTTCTCCTCTTCTGTC

CN 120027 GTACCTTATTCGAGAGAGCTTTGCATCGAGATGTTGCTGTGTGTGTTCTCCTCTTCTGTC

CN 120030 GTACCTTATTCGAGAGAGCTTTGCATCGAGATGTTGCTGTGTGTGTTCTCCTCTTCTGTC

CN 119294 GTACCTTATTCGAGAGAGCTTTGCATCGAGATGTTGCTGTGTGTGTTCTCCTCTTCTGTC

CN 120013 GTACCTTATTCGAGAGAGCTTTGCATCGAGATGTTGCTGTGTGTGTTCTCCTCTTCTGTC

CN 120017 GTACCTTATTCGAGAGAGCTTTGCATCGAGATGTTGCTGTGTGTGTTCTCCTCTTCTGTC

CN 119205 GTACCTTATTCGAGAGAGCTTTGCATCGAGATGTTGCTGTGTGTGTTCTCCTCTTCTGTC

DH55 ref genome GTACCTTATTCGAGAGAGCTTTGCATCGAGATGTTGCTGTGTGTGTTCTCCTCTTCTGTC

09-CS0040 GTACCTTATTCGAGAGAGCTTTGCATCGAGATGTTGCTGTGTGTGTTCTCCTCTTCTGTC

CN 113754 GTACCTTATTCGAGAGAGCTTTGCATCGAGATGTTGCTGTGTGTGTTCTCCTCTTCTGTC

CO46 NCBI GTACCTTATTCGAGAGAGCTTTGCATCGAGATGTTGCTGTGTGTGTTCTCCTCTTCTGTC

Jasper GTACCTTATTCGAGAGAGCTTTGCATCGAGATGTTGCTGTGTGTGTTCTCCTCTTCTGTC

Joelle phyto GTACCTTATTCGAGAGAGCTTTGCATCGAGATGTTGCTGTGTGTGTTCTCCTCTTCTGTC

Joelle NCBI GTACCTTATTCGAGAGAGCTTTGCATCGAGATGTTGCTGTGTGTGTTCTCCTCTTCTGTC

CN 119243 GTACCTTATTCGAGAGAGCTTTGCATCGAGATGTTGCTGTGTGTGTTCTCCTCTTCTGTC

CN 120025 GTACCTTATTCGAGAGAGCTTTGCATCGAGATGTTGCTGTGTGTGTTCTCCTCTTCTGTC

Joelle AAFC GTACCTTATTCGAGAGAGCTTTGCATCGAGATGTTGCTGTGTGTGTTCTCCTCTTCTGTC

************************************************************

CAM 241 GAAAGCTTGTGTTTGCTTCACAGTGAAGAAGCCTTCTGCTTATTTTGCAATAGAGGCGTG

17CS1133 GAAAGCTTGTGTTTGCTTCACAGTGAAGAAGCCTTCTGCTTATTTTGCAATAGAGGCGTG

CAM 236 GAAAGCTTGTGTTTGCTTCACAGTGAAGAAGCCTTCTGCTTATTTTGCAATAGAGGCGTG

Blaine Creek GAAAGCTTGTGTTTGCTTCACAGTGAAGAAGCCTTCTGCTTATTTTGCAATAGAGGCGTG

CN 119300 GAAAGCTTGTGTTTGCTTCACAGTGAAGAAGCCTTCTGCTTATTTTGCAATAGAGGCGTG

Yellowstone GAAAGCTTGTGTTTGCTTCACAGTGAAGAAGCCTTCTGCTTATTTTGCAATAGAGGCGTG

Hoga GAAAGCTTGTGTTTGCTTCACAGTGAAGAAGCCTTCTGCTTATTTTGCAATAGAGGCGTG

CN 120027 GAAAGCTTGTGTTTGCTTCACAGTGAAGAAGCCTTCTGCTTATTTTGCAATAGAGGCGTG

CN 120030 GAAAGCTTGTGTTTGCTTCACAGTGAAGAAGCCTTCTGCTTATTTTGCAATAGAGGCGTG

CN 119294 GAAAGCTTGTGTTTGCTTCACAGTGAAGAAGCCTTCTGCTTATTTTGCAATAGAGGCGTG

CN 120013 GAAAGCTTGTGTTTGCTTCACAGTGAAGAAGCCTTCTGCTTATTTTGCAATAGAGGCGTG

CN 120017 GAAAGCTTGTGTTTGCTTCACAGTGAAGAAGCCTTCTGCTTATTTTGCAATAGAGGCGTG

CN 119205 GAAAGCTTGTGTTTGCTTCACAGTGAAGAAGCCTTCTGCTTATTTTGCAATAGAGGCGTG

DH55 ref genome GAAAGCTTGTGTTTGCTTCACAGTGAAGAAGCCTTCTGCTTATTTTGCAATAGAGGCGTG

09-CS0040 GAAAGCTTGTGTTTGCTTCACAGTGAAGAAGCCTTCTGCTTATTTTGCAATAGAGGCGTG

CN 113754 GAAAGCTTGTGTTTGCTTCACAGTGAAGAAGCCTTCTGCTTATTTTGCAATAGAGGCGTG
[truncated: 40,197 more chars]
