# Supplementary material for: All roads lead to Rome: QTL analysis for vernalization requirement and dissection of allelic variation uncovered unexpected diversity of FLC loci in Camelina sativa
Source: Front Plant Sci. 2025 Jul 25;16:1639872. doi: 10.3389/fpls.2025.1639872 (PMC12331742; doi:10.3389/fpls.2025.1639872)
Supplement: Supplementary Table 6 — Sequence alignment of the FLC.C20 gene. Two C. microcarpa (CN 120025 and CN 119205; CN 119243 is a tetraploid C. microcarpa line that does not have a third subgenome and thus is not included); ‘CO46’ (GCA_036971115.1) and ‘Joelle’ (GCA_036769185.1) reference sequences from NCBI; ‘Joelle’ sequences from the AAFC and the DOE-JGI phytozome reference genomes; the DH55 reference genome sequence and 15 spring type C. sativa lines were aligned using the EMBL-EBI online tool MUSCLE. All winter Camelina lines are in blue font. Exons are shown in purple font and the SNP utilized for marker development is highlighted in blue (Anderson et al., 2018). A large insert only present within the ‘CO46’ reference sequence is represented by an N base highlighted in red. The complete sequence for the insert is presented at the end of the alignment and is in red font. [file Table6.docx]

**Supplementary File 6.** Sequence alignment of the FLC.C20 gene. Two *C. microcarpa* (CN 120025 and CN 119205; CN 119243 is a tetraploid *C. microcarpa* line that does not have a third subgenome and thus is not included); ‘CO46’ (GCA_036971115.1) and ‘Joelle’ (GCA_036769185.1) reference sequences from NCBI; ‘Joelle’ sequences from the AAFC and the DOE-JGI phytozome reference genomes; the DH55 reference genome sequence and 15 spring type *C. sativa* lines were aligned using the EMBL-EBI online tool MUSCLE. All winter Camelina lines are in blue font. Exons are shown in purple font and the SNP utilized for marker development is highlighted in blue (Anderson et al. 2018). A large insert only present within the ‘CO46’ reference sequence is represented by an N base highlighted in red. The complete sequence for the insert is presented at the end of the alignment and is in red font.

CN 120025 GAATTAAAAATTGATGAAGAGGTAATTTTTTAAATAAAAAATTAAAGAGTCATTAGAGAA

CN 120013 GAATTAAAAATTGATGAAGAGGTAATTTTTTTAATGAAAAATTAAAGAGTCATTAGAGAT

CS17CS1133 GAATTAAAAATTGATGAAGAGGTAATTTTTTTAATGAAAAATTAAAGAGTCATTAGAGAT

CN 120030 GAATTAAAAATTGATGAAGAGGTAATTTTTTTAATGAAAAATTAAAGAGTCATTAGAGAT

CO46 NCBI GAATTAAAAATTGATGAAGAGGTAATTTTTTTAATGAAAAATTAAAGAGTCATTAGAGAT

DH55 ref genome GAATTAAAAATTGATGAAGAGGTAATTTTTTTAATGAAAAATTAAAGAGTCATTAGAGAT

Hoga GAATTAAAAATTGATGAAGAGGTAATTTTTTTAATGAAAAATTAAAGAGTCATTAGAGAT

CAM 236 GAATTAAAAATTGATGAAGAGGTAATTTTTTTAATGAAAAATTAAAGAGTCATTAGAGAT

09-CS0040 GAATTAAAAATTGATGAAGAGGTAATTTTTTTAATGAAAAATTAAAGAGTCATTAGAGAT

CN 119205 GAATTAAAAATTGATGAAGAGGTAATTTTTTTAATGAAAAATTAAAGAGTCATTAGAGAT

Yellowstone GAATTAAAAATTGATGAAGAGGTAATTTTTTTAATGAAAAATTAAAGAGTCATTAGAGAT

CN 120017 GAATTAAAAATTGATGAAGAGGTAATTTTTTTAATGAAAAATTAAAGAGTCATTAGAGAT

CN 119300 GAATTAAAAATTGATGAAGAGGTAATTTTTTTAATGAAAAATTAAAGAGTCATTAGAGAT

Jasper GAATTAAAAATTGATGAAGAGGTAATTTTTTTAATGAAAAATTAAAGAGTCATTAGAGAT

CN 120027 GAATTAAAAATTGATGAAGAGGTAATTTTTTTAATGAAAAATTAAAGAGTCATTAGAGAT

CN 113754 GAATTAAAAATTGATGAAGAGGTAATTTTTTTAATGAAAAATTAAAGAGTCATTAGAGAT

Joelle AAFC GAATTAAAAATTGATGAAGAGGTAATTTTTTTAATGAAAAATTAAAGAGTCATTAGAGAT

Joelle NCBI GAATTAAAAATTGATGAAGAGGTAATTTTTTTAATGAAAAATTAAAGAGTCATTAGAGAT

Joelle phyto GAATTAAAAATTGATGAAGAGGTAATTTTTTTAATGAAAAATTAAAGAGTCATTAGAGAT

Blaine Creek GAATTAAAAATTGATGAAGAGGTAATTTTTTTAATGAAAAATTAAAGAGTCATTAGAGAT

CAM 241 GAATTAAAAATTGATGAAGAGGTAATTTTTTTAATGAAAAATTAAAGAGTCATTAGAGAT

CN 119294 GAATTAAAAATTGATGAAGAGGTAATTTTTTTAATGAAAAATTAAAGAGTCATTAGAGAT

******************************* *** ***********************

CN 120025 AGCCAATGGAAGAAGAGCAAACGCGGTCGCATGATATTCGTCATGCGGTACACGTGGCAA

CN 120013 TGCCAATGGAAGAAGAGCAAACGCGGTCGCATGATACTCGTCATGCGGTACACGTGGCAA

CS17CS1133 TGCCAATGGAAGAAGAGCAAACGCGGTCGCATGATACTCGTCATGCGGTACACGTGGCAA

CN 120030 AGCCAATGGAAGAAGAGCAAACGCGGTCGCATGATACTCGTCATGCGGTACACGTGGCAA

CO46 NCBI AGCCAATGGAAGAAGAGCAAACGCGGTCGCATGATACTCGTCATGCGGTACACGTGGCAA

DH55 ref genome TGCCAATGGAAGAAGAGCAAACGCGGTCGCATGATACTCGTCATGCGGTACACGTGGCAA

Hoga TGCCAATGGAAGAAGAGCAAACGCGGTCGCATGATACTCGTCATGCGGTACACGTGGCAA

CAM 236 TGCCAATGGAAGAAGAGCAAACGCGGTCGCATGATACTCGTCATGCGGTACACGTGGCAA

09-CS0040 TGCCAATGGAAGAAGAGCAAACGCGGTCGCATGATACTCGTCATGCGGTACACGTGGCAA

CN 119205 TGCCAATGGAAGAAGAGCAAACGCGGTCGCATGATACTCGTCATGCGGTACACGTGGCAA

Yellowstone TGCCAATGGAAGAAGAGCAAACGCGGTCGCATGATACTCGTCATGCGGTACACGTGGCAA

CN 120017 TGCCAATGGAAGAAGAGCAAACGCGGTCGCATGATACTCGTCATGCGGTACACGTGGCAA

CN 119300 TGCCAATGGAAGAAGAGCAAACGCGGTCGCATGATACTCGTCATGCGGTACACGTGGCAA

Jasper TGCCAATGGAAGAAGAGCAAACGCGGTCGCATGATACTCGTCATGCGGTACACGTGGCAA

CN 120027 TGCCAATGGAAGAAGAGCAAACGCGGTCGCATGATACTCGTCATGCGGTACACGTGGCAA

CN 113754 TGCCAATGGAAGAAGAGCAAACGCGGTCGCATGATACTCGTCATGCGGTACACGTGGCAA

Joelle AAFC TGCCAATGGAAGAAGAGCAAACGCGGTCGCATGATACTCGTCATGCGGTACACGTGGCAA

Joelle NCBI TGCCAATGGAAGAAGAGCAAACGCGGTCGCATGATACTCGTCATGCGGTACACGTGGCAA

Joelle phyto TGCCAATGGAAGAAGAGCAAACGCGGTCGCATGATACTCGTCATGCGGTACACGTGGCAA

Blaine Creek AGCCAATGGAAGAAGAGCAAACGCGGTCGCATGATACTCGTCATGCGGTACACGTGGCAA

CAM 241 AGCCAATGGAAGAAGAGCAAACGCGGTCGCATGATACTCGTCATGCGGTACACGTGGCAA

CN 119294 AGCCAATGGAAGAAGAGCAAACGCGGTCGCATGATACTCGTCATGCGGTACACGTGGCAA

*********************************** ***********************

CN 120025 TGTTGCGTTCAAAACGCAACGTTTTTATTCATAAATTTTATTTTCATAACTCTCGTTTAC

CN 120013 TCTTGCGTTCAAAACGCATCGTTTTTGTTCATAAATTTTATTTTCATCACTCTCGTTTAC

CS17CS1133 TCTTGCGTTCAAAACGCATCGTTTTTGTTCATAAATTTTATTTTCATCACTCTCGTTTAC

CN 120030 TCTTGCGTTCAAAACGCATCGTTTTTGTTCATAAATTTTATTTTCATCACTCTCGTTTAC

CO46 NCBI TCTTGCGTTCAAAACGCATCGTTTTTGTTCATAAATTTTATTTTCATCACTCTCGTTTAC

DH55 ref genome TCTTGCGTTCAAAACGCATCGTTTTTGTTCATAAATTTTATTTTCATCACTCTCGTTTAC

Hoga TCTTGCGTTCAAAACGCATCGTTTTTGTTCATAAATTTTATTTTCATCACTCTCGTTTAC

CAM 236 TCTTGCGTTCAAAACGCATCGTTTTTGTTCATAAATTTTATTTTCATCACTCTCGTTTAC

09-CS0040 TCTTGCGTTCAAAACGCATCGTTTTTGTTCATAAATTTTATTTTCATCACTCTCGTTTAC

CN 119205 TCTTGCGTTCAAAACGCATCGTTTTTGTTCATAAATTTTATTTTCATCACTCTCGTTTAC

Yellowstone TCTTGCGTTCAAAACGCATCGTTTTTGTTCATAAATTTTATTTTCATCACTCTCGTTTAC

CN 120017 TCTTGCGTTCAAAACGCATCGTTTTTGTTCATAAATTTTATTTTCATCACTCTCGTTTAC

CN 119300 TCTTGCGTTCAAAACGCATCGTTTTTGTTCATAAATTTTATTTTCATCACTCTCGTTTAC

Jasper TCTTGCGTTCAAAACGCATCGTTTTTGTTCATAAATTTTATTTTCATCACTCTCGTTTAC

CN 120027 TCTTGCGTTCAAAACGCATCGTTTTTGTTCATAAATTTTATTTTCATCACTCTCGTTTAC

CN 113754 TCTTGCGTTCAAAACGCATCGTTTTTGTTCATAAATTTTATTTTCATCACTCTCGTTTAC

Joelle AAFC TCTTGCGTTCAAAACGCATCGTTTTTGTTCATAAATTTTATTTTCATCACTCTCGTTTAC

Joelle NCBI TCTTGCGTTCAAAACGCATCGTTTTTGTTCATAAATTTTATTTTCATCACTCTCGTTTAC

Joelle phyto TCTTGCGTTCAAAACGCATCGTTTTTGTTCATAAATTTTATTTTCATCACTCTCGTTTAC

Blaine Creek TCTTGCGTTCAAAACGCATCGTTTTTGTTCATAAATTTTATTTTCATCACTCTCGTTTAC

CAM 241 TCTTGCGTTCAAAACGCATCGTTTTTGTTCATAAATTTTATTTTCATCACTCTCGTTTAC

CN 119294 TCTTGCGTTCAAAACGCATCGTTTTTGTTCATAAATTTTATTTTCATCACTCTCGTTTAC

* **************** ******* ******************** ************

CN 120025 CCC---AAAAAAAAAAAAAAAAAATCTAGCCCGAGGAAGG--AAAAAAAAAAGTAGATTA

CN 120013 CCT------AAAAAAAAAAAAAAATCTAGCCCAAGGAAGGAAAAAAAAAAAAGTAGATTA

CS17CS1133 CCT----AAAAAAAAAAAAAAAAATCTAGCCCAAGGAAGGAAAAAAAAAAAAGTAGATTA

CN 120030 CCT----AAAAAAAAAAAAAAAAATCTAGCCCAAGGAAGG---AAAAAAAAAGTAGATTA

CO46 NCBI CCT-AAAAAAAAAAAAAAAAAAAATCTAGCCCAAGGAAGGAAAAAAAAAAAAGTAGATTA

DH55 ref genome CCT-AAAAAAAAAAAAAAAAAAAATCTAGCCCAAGGAAGGAAAAAAAAAAAAGTAGATTA

Hoga CCT-AAAAAAAAAAAAAAAAAAAATCTAGCCCAAGGAAGGAAAAAAAAAAAAGTAGATTA

CAM 236 CCT--AAAAAAAAAAAAAAAAAAATCTAGCCCAAGGAAGGAAAAAAAAAAAAGTAGATTA

09-CS0040 CCT--AAAAAAAAAAAAAAAAAAATCTAGCCCAAGGAAGGAAAAAAAAAAAAGTAGATTA

CN 119205 CCT-----------------AAAATCTAGCCCAAGGAAGGAAAAAAAAAAAAGTAGATTA

Yellowstone CCT-AAAAAAAAAAAAAAAAAAAATCTAGCCCAAGGAAGGAAAAAAAAAAAAGTAGATTA

CN 120017 CCT------AAAAAAAAAAAAAAATCTAGCCCAAGGAAGGAAAAAAAAAAAAGTAGATTA

CN 119300 CCT-AAAAAAAAAAAAAAAAAAAATCTAGCCCAAGGAAGGAAAAAAAAAAAAGTAGATTA

Jasper CCT-AAAAAAAAAAAAAAAAAAAATCTAGCCCAAGGAAGGAAAAAAAAAAAAGTAGATTA

CN 120027 CCT-AAAAAAAAAAAAAAAAAAAATCTAGCCCAAGGAAGGAAAAAAAAAAAAGTAGATTA

CN 113754 CCT-AAAAAAAAAAAAAAAAAAAATCTAGCCCAAGGAAGGAAAAAAAAAAAAGTAGATTA

Joelle AAFC CCT-AAAAAAAAAAAAAAAAAAAATCTAGCCCAAGGAAGGAAAAAAAAAAAAGTAGATTA

Joelle NCBI CCTAAAAAAAAAAAAAAAAAAAAATCTAGCCCAAGGAAGGAAAAAAAAAAAAGTAGATTA

Joelle phyto CCT-AAAAAAAAAAAAAAAAAAAATCTAGCCCAAGGAAGGAAAAAAAAAAAAGTAGATTA

Blaine Creek CCT-------AAAAAAAAAAAAAATCTAGCCCAAGGAAGGAAAAAAAAAAAAGTAGATTA

CAM 241 CCT----AAAAAAAAAAAAAAAAATCTAGCCCAAGGAAGGAAAAAAAAAAAAGTAGATTA

CN 119294 CCT------AAAAAAAAAAAAAAATCTAGCCCAAGGAAGGAAAAAAAAAAAAGTAGATTA

** ************ ******* *****************

CN 120025 GACAAAAAAAATAGAAAGAAATAAATAAAAGGAAAAAAGGAAATAAAAAAATAGGAAAGG

CN 120013 GAC-AAAAAAATAGAAAG----AAATAAAAGGAAAAAAGGAAATAAAAAAATAGGAAAGG

CS17CS1133 GAC-AAAAAAATAGAAAG----AAATAAAAGGAAAAAAGGAAATAAAAAAATAGGAAAGG

CN 120030 GAC-AAAAAAATAGAAAG----AAATAAAAGGAAAAAAGGAAATAAAAAAATAGGAAAGG

CO46 NCBI GAC-AAAAAAATAGAAAG----AAATAAAAGGAAAAAAGGAAATAAAAAAATAGGAAAGG

DH55 ref genome GAC-AAAAAAATAGAAAG----AAATAAAAGGAAAAAAGGAAATAAAAAAATAGGAAAGG

Hoga GAC-AAAAAAATAGAAAG----AAATAAAAGGAAAAAAGGAAATAAAAAAATAGGAAAGG

CAM 236 GAC-AAAAAAATAGAAAG----AAATAAAAGGAAAAAAGGAAATAAAAAAATAGGAAAGG

09-CS0040 GAC-AAAAAAATAGAAAG----AAATAAAAGGAAAAAAGGAAATAAAAAAATAGGAAAGG

CN 119205 GAC-AAAAAAATAGAAAG----AAATAAAAGGAAAAAAGGAAATAAAAAAATAGGAAAGG

Yellowstone GAC-AAAAAAATAGAAAG----AAATAAAAGGAAAAAAGGAAATAAAAAAATAGGAAAGG

CN 120017 GAC-AAAAAAATAGAAAG----AAATAAAAGGAAAAAAGGAAATAAAAAAATAGGAAAGG

CN 119300 GAC-AAAAAAATAGAAAG----AAATAAAAGGAAAAAAGGAAATAAAAAAATAGGAAAGG

Jasper GAC-AAAAAAATAGAAAG----AAATAAAAGGAAAAAAGGAAATAAAAAAATAGGAAAGG

CN 120027 GAC-AAAAAAATAGAAAG----AAATAAAAGGAAAAAAGGAAATAAAAAAATAGGAAAGG

CN 113754 GAC-AAAAAAATAGAAAG----AAATAAAAGGAAAAAAGGAAATAAAAAAATAGGAAAGG

Joelle AAFC GAC-AAAAAAATAGAAAG----AAATAAAAGGAAAAAAGGAAATAAAAAAATAGGAAAGG

Joelle NCBI GAC-AAAAAAATAGAAAG----AAATAAAAGGAAAAAAGGAAATAAAAAAATAGGAAAGG

Joelle phyto GAC-AAAAAAATAGAAAG----AAATAAAAGGAAAAAAGGAAATAAAAAAATAGGAAAGG

Blaine Creek GAC-AAAAAAATAGAAAG----AAATAAAAGGAAAAAAGGAAATAAAAAAATAGGAAAGG

CAM 241 GAC-AAAAAAATAGAAAG----AAATAAAAGGAAAAAAGGAAATAAAAAAATAGGAAAGG

CN 119294 GAC-AAAAAAATAGAAAG----AAATAAAAGGAAAAAAGGAAATAAAAAAATAGGAAAGG

*** ************** **************************************

CN 120025 CAAAAAAAAAAAAAAGAGAGAAACGCTTAGTATCTCTCCGGCGACTTG-----AACCCAA

CN 120013 CAAAAAAAGA------AGAGAAACGCTTAGTATCTCTCCGGCGACTTGAACCCAACCCAA

CS17CS1133 CAAAAAAAGA------AGAGAAACGCTTAGTATCTCTCCGGCGACTTGAACCCAACCCAA

CN 120030 CAAAAAAAGA------AGAGAAACGCTTAGTATCTCTCCGGCGACTTGAACCCAACCCAA

CO46 NCBI CAAAAAAAGA------AGAGAAACGCTTAGTATCTCTCCGGCGACTTGAACCCAACCCAA

DH55 ref genome CAAAAAAAGA------AGAGAAACGCTTAGTATCTCTCCGGCGACTTGAACCCAACCCAA

Hoga CAAAAAAAGA------AGAGAAACGCTTAGTATCTCTCCGGCGACTTGAACCCAACCCAA

CAM 236 CAAAAAAAGA------AGAGAAACGCTTAGTATCTCTCCGGCGACTTGAACCCAACCCAA

09-CS0040 CAAAAAAAGA------AGAGAAACGCTTAGTATCTCTCCGGCGACTTGAACCCAACCCAA

CN 119205 CAAAAAAAGA------AGAGAAACGCTTAGTATCTCTCCGGCGACTTGAACCCAACCCAA

Yellowstone CAAAAAAAGA------AGAGAAACGCTTAGTATCTCTCCGGCGACTTGAACCCAACCCAA

CN 120017 CAAAAAAAGA------AGAGAAACGCTTAGTATCTCTCCGGCGACTTGAACCCAACCCAA

CN 119300 CAAAAAAAGA------AGAGAAACGCTTAGTATCTCTCCGGCGACTTGAACCCAACCCAA

Jasper CAAAAAAAGA------AGAGAAACGCTTAGTATCTCTCCGGCGACTTGAACCCAACCCAA

CN 120027 CAAAAAAAGA------AGAGAAACGCTTAGTATCTCTCCGGCGACTTGAACCCAACCCAA

CN 113754 CAAAAAAAGA------AGAGAAACGCTTAGTATCTCTCCGGCGACTTGAACCCAACCCAA

Joelle AAFC CAAAAAAAGA------AGAGAAACGCTTAGTATCTCTCCGGCGACTTGAACCCAACCCAA

Joelle NCBI CAAAAAAAGA------AGAGAAACGCTTAGTATCTCTCCGGCGACTTGAACCCAACCCAA

Joelle phyto CAAAAAAAGA------AGAGAAACGCTTAGTATCTCTCCGGCGACTTGAACCCAACCCAA

Blaine Creek CAAAAAAAGA------AGAGAAACGCTTAGTATCTCTCCGGCGACTTGAACCCAACCCAA

CAM 241 CAAAAAAAGA------AGAGAAACGCTTAGTATCTCTCCGGCGACTTGAACCCAACCCAA

CN 119294 CAAAAAAAGA------AGAGAAACGCTTAGTATCTCTCCGGCGACTTGAACCCAACCCAA

******** * ******************************** *******

CN 120025 ACCTGAGGATCAAATTAGGGCACACAAGGGCCTCTCGGAGACTGAAGCCATGGGAAGGAA

CN 120013 ACCTGAGGATCAAATTAGGG--CACAAGGGCCTCTCGGAGACTGAAGCCATGGGAAGGAA

CS17CS1133 ACCTGAGGATCAAATTAGGG--CACAAGGGCCTCTCGGAGACTGAAGCCATGGGAAGGAA

CN 120030 ACCTGAGGATCAAATTAGGG--CACAAGGGCCTCTCGGAGACTGAAGCCATGGGAAGGAA

CO46 NCBI ACCTGAGGATCAAATTAGGG--CACAAGGGCCTCTCGGAGACTGAAGCCATGGGAAGGAA

DH55 ref genome ACCTGAGGATCAAATTAGGG--CACAAGGGCCTCTCGGAGACTGAAGCCATGGGAAGGAA

Hoga ACCTGAGGATCAAATTAGGG--CACAAGGGCCTCTCGGAGACTGAAGCCATGGGAAGGAA

CAM 236 ACCTGAGGATCAAATTAGGG--CACAAGGGCCTCTCGGAGACTGAAGCCATGGGAAGGAA

09-CS0040 ACCTGAGGATCAAATTAGGG--CACAAGGGCCTCTCGGAGACTGAAGCCATGGGAAGGAA

CN 119205 ACCTGAGGATCAAATTAGGG--CACAAGGGCCTCTCGGAGACTGAAGCCATGGGAAGGAA

Yellowstone ACCTGAGGATCAAATTAGGG--CACAAGGGCCTCTCGGAGACTGAAGCCATGGGAAGGAA

CN 120017 ACCTGAGGATCAAATTAGGG--CACAAGGGCCTCTCGGAGACTGAAGCCATGGGAAGGAA

CN 119300 ACCTGAGGATCAAATTAGGG--CACAAGGGCCTCTCGGAGACTGAAGCCATGGGAAGGAA

Jasper ACCTGAGGATCAAATTAGGG--CACAAGGGCCTCTCGGAGACTGAAGCCATGGGAAGGAA

CN 120027 ACCTGAGGATCAAATTAGGG--CACAAGGGCCTCTCGGAGACTGAAGCCATGGGAAGGAA

CN 113754 ACCTGAGGATCAAATTAGGG--CACAAGGGCCTCTCGGAGACTGAAGCCATGGGAAGGAA

Joelle AAFC ACCTGAGGATCAAATTAGGG--CACAAGGGCCTCTCGGAGACTGAAGCCATGGGAAGGAA

Joelle NCBI ACCTGAGGATCAAATTAGGG--CACAAGGGCCTCTCGGAGACTGAAGCCATGGGAAGGAA

Joelle phyto ACCTGAGGATCAAATTAGGG--CACAAGGGCCTCTCGGAGACTGAAGCCATGGGAAGGAA

Blaine Creek ACCTGAGGATCAAATTAGGG--CACAAGGGCCTCTCGGAGACTGAAGCCATGGGAAGGAA

CAM 241 ACCTGAGGATCAAATTAGGG--CACAAGGGCCTCTCGGAGACTGAAGCCATGGGAAGGAA

CN 119294 ACCTGAGGATCAAATTAGGG--CACAAGGGCCTCTCGGAGACTGAAGCCATGGGAAGGAA

******************** **************************************

CN 120025 AAAACTAGAAATCAAGCGAATTGAGAACAAAAGTAGCCGACAAGTCACCTTCTCCAAACG

CN 120013 AAAACTAGAAATCAAGCGAATTGAGAACAAAAGTAGCCGACAAGTCACCTTCTCCAAACG

CS17CS1133 AAAACTAGAAATCAAGCGAATTGAGAACAAAAGTAGCCGACAAGTCACCTTCTCCAAACG

CN 120030 AAAACTAGAAATCAAGCGAATTGAGAACAAAAGTAGCCGACAAGTCACCTTCTCCAAACG

CO46 NCBI AAAACTAGAAATCAAGCGAATTGAGAACAAAAGTAGCCGACAAGTCACCTTCTCCAAACG

DH55 ref genome AAAACTAGAAATCAAGCGAATTGAGAACAAAAGTAGCCGACAAGTCACCTTCTCCAAACG

Hoga AAAACTAGAAATCAAGCGAATTGAGAACAAAAGTAGCCGACAAGTCACCTTCTCCAAACG

CAM 236 AAAACTAGAAATCAAGCGAATTGAGAACAAAAGTAGCCGACAAGTCACCTTCTCCAAACG

09-CS0040 AAAACTAGAAATCAAGCGAATTGAGAACAAAAGTAGCCGACAAGTCACCTTCTCCAAACG

CN 119205 AAAACTAGAAATCAAGCGAATTGAGAACAAAAGTAGCCGACAAGTCACCTTCTCCAAACG

Yellowstone AAAACTAGAAATCAAGCGAATTGAGAACAAAAGTAGCCGACAAGTCACCTTCTCCAAACG

CN 120017 AAAACTAGAAATCAAGCGAATTGAGAACAAAAGTAGCCGACAAGTCACCTTCTCCAAACG

CN 119300 AAAACTAGAAATCAAGCGAATTGAGAACAAAAGTAGCCGACAAGTCACCTTCTCCAAACG

Jasper AAAACTAGAAATCAAGCGAATTGAGAACAAAAGTAGCCGACAAGTCACCTTCTCCAAACG

CN 120027 AAAACTAGAAATCAAGCGAATTGAGAACAAAAGTAGCCGACAAGTCACCTTCTCCAAACG

CN 113754 AAAACTAGAAATCAAGCGAATTGAGAACAAAAGTAGCCGACAAGTCACCTTCTCCAAACG

Joelle AAFC AAAACTAGAAATCAAGCGAATTGAGAACAAAAGTAGCCGACAAGTCACCTTCTCCAAACG

Joelle NCBI AAAACTAGAAATCAAGCGAATTGAGAACAAAAGTAGCCGACAAGTCACCTTCTCCAAACG

Joelle phyto AAAACTAGAAATCAAGCGAATTGAGAACAAAAGTAGCCGACAAGTCACCTTCTCCAAACG

Blaine Creek AAAACTAGAAATCAAGCGAATTGAGAACAAAAGTAGCCGACAAGTCACCTTCTCCAAACG

CAM 241 AAAACTAGAAATCAAGCGAATTGAGAACAAAAGTAGCCGACAAGTCACCTTCTCCAAACG

CN 119294 AAAACTAGAAATCAAGCGAATTGAGAACAAAAGTAGCCGACAAGTCACCTTCTCCAAACG

************************************************************

CN 120025 TCGCAATGGTCTCATCGAGAAAGCTCGTCAGCTTTCTGTTCTCTGTGACGCATCCGTCGC

CN 120013 TCGTAATGGTCTCATCGAGAAAGCTCGTCAGCTTTCTGTTCTCTGTGACGCATCCGTCGC

CS17CS1133 TCGTAATGGTCTCATCGAGAAAGCTCGTCAGCTTTCTGTTCTCTGTGACGCATCCGTCGC

CN 120030 TCGTAATGGTCTCATCGAGAAAGCTCGTCAGCTTTCTGTTCTCTGTGACGCATCCGTCGC

CO46 NCBI TCGTAATGGTCTCATCGAGAAAGCTCGTCAGCTTTCTGTTCTCTGTGACGCATCCGTCGC

DH55 ref genome TCGTAATGGTCTCATCGAGAAAGCTCGTCAGCTTTCTGTTCTCTGTGACGCATCCGTCGC

Hoga TCGTAATGGTCTCATCGAGAAAGCTCGTCAGCTTTCTGTTCTCTGTGACGCATCCGTCGC

CAM 236 TCGTAATGGTCTCATCGAGAAAGCTCGTCAGCTTTCTGTTCTCTGTGACGCATCCGTCGC

09-CS0040 TCGTAATGGTCTCATCGAGAAAGCTCGTCAGCTTTCTGTTCTCTGTGACGCATCCGTCGC

CN 119205 TCGTAATGGTCTCATCGAGAAAGCTCGTCAGCTTTCTGTTCTCTGTGACGCATCCGTCGC

Yellowstone TCGTAATGGTCTCATCGAGAAAGCTCGTCAGCTTTCTGTTCTCTGTGACGCATCCGTCGC

CN 120017 TCGTAATGGTCTCATCGAGAAAGCTCGTCAGCTTTCTGTTCTCTGTGACGCATCCGTCGC

CN 119300 TCGTAATGGTCTCATCGAGAAAGCTCGTCAGCTTTCTGTTCTCTGTGACGCATCCGTCGC

Jasper TCGTAATGGTCTCATCGAGAAAGCTCGTCAGCTTTCTGTTCTCTGTGACGCATCCGTCGC

CN 120027 TCGTAATGGTCTCATCGAGAAAGCTCGTCAGCTTTCTGTTCTCTGTGACGCATCCGTCGC

CN 113754 TCGTAATGGTCTCATCGAGAAAGCTCGTCAGCTTTCTGTTCTCTGTGACGCATCCGTCGC

Joelle AAFC TCGTAATGGTCTCATCGAGAAAGCTCGTCAGCTTTCTGTTCTCTGTGACGCATCCGTCGC

Joelle NCBI TCGTAATGGTCTCATCGAGAAAGCTCGTCAGCTTTCTGTTCTCTGTGACGCATCCGTCGC

Joelle phyto TCGTAATGGTCTCATCGAGAAAGCTCGTCAGCTTTCTGTTCTCTGTGACGCATCCGTCGC

Blaine Creek TCGTAATGGTCTCATCGAGAAAGCTCGTCAGCTTTCTGTTCTCTGTGACGCATCCGTCGC

CAM 241 TCGTAATGGTCTCATCGAGAAAGCTCGTCAGCTTTCTGTTCTCTGTGACGCATCCGTCGC

CN 119294 TCGTAATGGTCTCATCGAGAAAGCTCGTCAGCTTTCTGTTCTCTGTGACGCATCCGTCGC

*** ********************************************************

CN 120025 TCTTCTCGTCGTCTCCGCCTCCGGCAAGCTCTACAGCTTCTCCTCCGGTGATAAGTACGT

CN 120013 TCTTCTCGTCGTCTCCGCCTCCGGCAAGCTCTACAGCTTCTCCTCCGGTGATAAGTACGT

CS17CS1133 TCTTCTCGTCGTCTCCGCCTCCGGCAAGCTCTACAGCTTCTCCTCCGGTGATAAGTACGT

CN 120030 TCTTCTCGTCGTCTCCGCCTCCGGCAAGCTCTACAGCTTCTCCTCCGGTGATAAGTACGT

CO46 NCBI TCTTCTCGTCGTCTCCGCCTCCGGCAAGCTCTACAGCTTCTCCTCCGGTGATAAGTACGT

DH55 ref genome TCTTCTCGTCGTCTCCGCCTCCGGCAAGCTCTACAGCTTCTCCTCCGGTGATAAGTACGT

Hoga TCTTCTCGTCGTCTCCGCCTCCGGCAAGCTCTACAGCTTCTCCTCCGGTGATAAGTACGT

CAM 236 TCTTCTCGTCGTCTCCGCCTCCGGCAAGCTCTACAGCTTCTCCTCCGGTGATAAGTACGT

09-CS0040 TCTTCTCGTCGTCTCCGCCTCCGGCAAGCTCTACAGCTTCTCCTCCGGTGATAAGTACGT

CN 119205 TCTTCTCGTCGTCTCCGCCTCCGGCAAGCTCTACAGCTTCTCCTCCGGTGATAAGTACGT

Yellowstone TCTTCTCGTCGTCTCCGCCTCCGGCAAGCTCTACAGCTTCTCCTCCGGTGATAAGTACGT

CN 120017 TCTTCTCGTCGTCTCCGCCTCCGGCAAGCTCTACAGCTTCTCCTCCGGTGATAAGTACGT

CN 119300 TCTTCTCGTCGTCTCCGCCTCCGGCAAGCTCTACAGCTTCTCCTCCGGTGATAAGTACGT

Jasper TCTTCTCGTCGTCTCCGCCTCCGGCAAGCTCTACAGCTTCTCCTCCGGTGATAAGTACGT

CN 120027 TCTTCTCGTCGTCTCCGCCTCCGGCAAGCTCTACAGCTTCTCCTCCGGTGATAAGTACGT

CN 113754 TCTTCTCGTCGTCTCCGCCTCCGGCAAGCTCTACAGCTTCTCCTCCGGTGATAAGTACGT

Joelle AAFC TCTTCTCGTCGTCTCCGCCTCCGGCAAGCTCTACAGCTTCTCCTCCGGTGATAAGTACGT

Joelle NCBI TCTTCTCGTCGTCTCCGCCTCCGGCAAGCTCTACAGCTTCTCCTCCGGTGATAAGTACGT

Joelle phyto TCTTCTCGTCGTCTCCGCCTCCGGCAAGCTCTACAGCTTCTCCTCCGGTGATAAGTACGT

Blaine Creek TCTTCTCGTCGTCTCCGCCTCCGGCAAGCTCTACAGCTTCTCCTCCGGTGATAAGTACGT

CAM 241 TCTTCTCGTCGTCTCCGCCTCCGGCAAGCTCTACAGCTTCTCCTCCGGTGATAAGTACGT

CN 119294 TCTTCTCGTCGTCTCCGCCTCCGGCAAGCTCTACAGCTTCTCCTCCGGTGATAAGTACGT

************************************************************

CN 120025 CTTTTCCTTATCTGGGTTCTCG-TTTTTTTCCCCCTTTAAGCTTCGGTTTTGTGCTTTCT

CN 120013 CTTTTCCTTATCTGGGTTCTCGTTTTTTTTTCCCCTTTAAGCTTCGGTTTTGTGCTTTCT

CS17CS1133 CTTTTCCTTATCTGGGTTCTCGTTTTTTTTTCCCCTTTAAGCTTCGGTTTTGTGCTTTCT

CN 120030 CTTTTCCTTATCTGGGTTCTCGTTTTTTTTTCCCCTTTAAGCTTCGGTTTTGTGCTTTCT

CO46 NCBI CTTTTCCTTATCTGGGTTCTCGTTTTTTTTTCCCCTTTAAGCTTCGGTTTTGTGCTTTCT

DH55 ref genome CTTTTCCTTATCTGGGTTCTCGTTTTTTTTTCCCCTTTAAGCTTCGGTTTTGTGCTTTCT

Hoga CTTTTCCTTATCTGGGTTCTCGTTTTTTTTTCCCCTTTAAGCTTCGGTTTTGTGCTTTCT

CAM 236 CTTTTCCTTATCTGGGTTCTCGTTTTTTTTTCCCCTTTAAGCTTCGGTTTTGTGCTTTCT

09-CS0040 CTTTTCCTTATCTGGGTTCTCGTTTTTTTTTCCCCTTTAAGCTTCGGTTTTGTGCTTTCT

CN 119205 CTTTTCCTTATCTGGGTTCTCGTTTTTTTTTCCCCTTTAAGCTTCGGTTTTGTGCTTTCT

Yellowstone CTTTTCCTTATCTGGGTTCTCGTTTTTTTTTCCCCTTTAAGCTTCGGTTTTGTGCTTTCT

CN 120017 CTTTTCCTTATCTGGGTTCTCGTTTTTTTTTCCCCTTTAAGCTTCGGTTTTGTGCTTTCT

CN 119300 CTTTTCCTTATCTGGGTTCTCGTTTTTTTTTCCCCTTTAAGCTTCGGTTTTGTGCTTTCT

Jasper CTTTTCCTTATCTGGGTTCTCGTTTTTTTTTCCCCTTTAAGCTTCGGTTTTGTGCTTTCT

CN 120027 CTTTTCCTTATCTGGGTTCTCGTTTTTTTTTCCCCTTTAAGCTTCGGTTTTGTGCTTTCT

CN 113754 CTTTTCCTTATCTGGGTTCTCGTTTTTTTTTCCCCTTTAAGCTTCGGTTTTGTGCTTTCT

Joelle AAFC CTTTTCCTTATCTGGGTTCTCGTTTTTTTTTCCCCTTTAAGCTTCGGTTTTGTGCTTTCT

Joelle NCBI CTTTTCCTTATCTGGGTTCTCGTTTTTTTTTCCCCTTTAAGCTTCGGTTTTGTGCTTTCT

Joelle phyto CTTTTCCTTATCTGGGTTCTCGTTTTTTTTTCCCCTTTAAGCTTCGGTTTTGTGCTTTCT

Blaine Creek CTTTTCCTTATCTGGGTTCTCGTTTTTTTTTCCCCTTTAAGCTTCGGTTTTGTGCTTTCT

CAM 241 CTTTTCCTTATCTGGGTTCTCGTTTTTTTTTCCCCTTTAAGCTTCGGTTTTGTGCTTTCT

CN 119294 CTTTTCCTTATCTGGGTTCTCGTTTTTTTTTCCCCTTTAAGCTTCGGTTTTGTGCTTTCT

********************** ******* *****************************

CN 120025 CTTTACTTTTTCTCTGAAG-AAAATAATTA-TATAAAAAGACACAA--AAAATAAAAAAT

CN 120013 CTTTACTTTGTTTTTGAAGAAAAATAAATATTTTAAAAAGACACAAACAAAATAAAAAAT

CS17CS1133 CTTTACTTTGTTTTTGAAGAAAAATAAATATTTTAAAAAGACACAAACAAAATAAAAAAT

CN 120030 CTTTACTTTGTTTTTGAAGAAAAATAAATATTTTAAAAAGACACAAACAAAATAAAAAAT

CO46 NCBI CTTTACTTTGTTTTTGAAGAAAAATAAATATTTTAAAAAGACACAAACAAAATAAAAAAT

DH55 ref genome CTTTACTTTGTTTTTGAAGAAAAATAAATATTTTAAAAAGACACAAACAAAATAAAAAAT

Hoga CTTTACTTTGTTTTTGAAGAAAAATAAATATTTTAAAAAGACACAAACAAAATAAAAAAT

CAM 236 CTTTACTTTGTTTTTGAAGAAAAATAAATATTTTAAAAAGACACAAACAAAATAAAAAAT

09-CS0040 CTTTACTTTGTTTTTGAAGAAAAATAAATATTTTAAAAAGACACAAACAAAATAAAAAAT

CN 119205 CTTTACTTTGTTTTTGAAGAAAAATAAATATTTTAAAAAGACACAAACAAAATAAAAAAT

Yellowstone CTTTACTTTGTTTTTGAAGAAAAATAAATATTTTAAAAAGACACAAACAAAATAAAAAAT

CN 120017 CTTTACTTTGTTTTTGAAGAAAAATAAATATTTTAAAAAGACACAAACAAAATAAAAAAT

CN 119300 CTTTACTTTGTTTTTGAAGAAAAATAAATATTTTAAAAAGACACAAACAAAATAAAAAAT

Jasper CTTTACTTTGTTTTTGAAGAAAAATAAATATTTTAAAAAGACACAAACAAAATAAAAAAT

CN 120027 CTTTACTTTGTTTTTGAAGAAAAATAAATATTTTAAAAAGACACAAACAAAATAAAAAAT

CN 113754 CTTTACTTTGTTTTTGAAGAAAAATAAATATTTTAAAAAGACACAAACAAAATAAAAAAT

Joelle AAFC CTTTACTTTGTTTTTGAAGAAAAATAAATATTTTAAAAAGACACAAACAAAATAAAAAAT

Joelle NCBI CTTTACTTTGTTTTTGAAGAAAAATAAATATTTTAAAAAGACACAAACAAAATAAAAAAT

Joelle phyto CTTTACTTTGTTTTTGAAGAAAAATAAATATTTTAAAAAGACACAAACAAAATAAAAAAT

Blaine Creek CTTTACTTTGTTTTTGAAGAAAAATAAATATTTTAAAAAGACACAAACAAAATAAAAAAT

CAM 241 CTTTACTTTGTTTTTGAAGAAAAATAAATATTTTAAAAAGACACAAACAAAATAAAAAAT

CN 119294 CTTTACTTTGTTTTTGAAGAAAAATAAATATTTTAAAAAGACACAAACAAAATAAAAAAT

********* * * ***** ******* ** * ************* ************

CN 120025 AAAATAAAAACAATTAATGTATAGTTTGATTTTTCCGGCGACTCTCTTGTTGTTTTACTC

CN 120013 A----AAAAACAATTAATGTATAGTTTGATTTTTCCGGCGAATCTCTTGTTGTTTTACTC

CS17CS1133 A----AAAAACAATTAATGTATAGTTTGATTTTTCCGGCGAATCTCTTGTTGTTTTACTC

CN 120030 A----AAAAACAATTAATGTATAGTTTGATTTTTCCGGCGAATCTCTTGTTGTTTTACTC

CO46 NCBI A----AAAAACAATTAATGTATAGTTTGATTTTTCCGGCGAATCTCTTGTTGTTTTACTC

DH55 ref genome A----AAAAACAATTAATGTATAGTTTGATTTTTCCGGCGAATCTCTTGTTGTTTTACTC

Hoga A----AAAAACAATTAATGTATAGTTTGATTTTTCCGGCGAATCTCTTGTTGTTTTACTC

CAM 236 A----AAAAACAATTAATGTATAGTTTGATTTTTCCGGCGAATCTCTTGTTGTTTTACTC

09-CS0040 A----AAAAACAATTAATGTATAGTTTGATTTTTCCGGCGAATCTCTTGTTGTTTTACTC

CN 119205 A----AAAAACAATTAATGTATAGTTTGATTTTTCCGGCGAATCTCTTGTTGTTTTACTC

Yellowstone A----AAAAACAATTAATGTATAGTTTGATTTTTCCGGCGAATCTCTTGTTGTTTTACTC

CN 120017 A----AAAAACAATTAATGTATAGTTTGATTTTTCCGGCGAATCTCTTGTTGTTTTACTC

CN 119300 A----AAAAACAATTAATGTATAGTTTGATTTTTCCGGCGAATCTCTTGTTGTTTTACTC

Jasper A----AAAAACAATTAATGTATAGTTTGATTTTTCCGGCGAATCTCTTGTTGTTTTACTC

CN 120027 A----AAAAACAATTAATGTATAGTTTGATTTTTCCGGCGAATCTCTTGTTGTTTTACTC

CN 113754 A----AAAAACAATTAATGTATAGTTTGATTTTTCCGGCGAATCTCTTGTTGTTTTACTC

Joelle AAFC A----AAAAACAATTAATGTATAGTTTGATTTTTCCGGCGAATCTCTTGTTGTTTTACTC

Joelle NCBI A----AAAAACAATTAATGTATAGTTTGATTTTTCCGGCGAATCTCTTGTTGTTTTACTC

Joelle phyto A----AAAAACAATTAATGTATAGTTTGATTTTTCCGGCGAATCTCTTGTTGTTTTACTC

Blaine Creek A----AAAAACAATTAATGTATAGTTTGATTTTTCCGGCGAATCTCTTGTTGTTTTACTC

CAM 241 A----AAAAACAATTAATGTATAGTTTGATTTTTCCGGCGAATCTCTTGTTGTTTTACTC

CN 119294 A----AAAAACAATTAATGTATAGTTTGATTTTTCCGGCGAATCTCTTGTTGTTTTACTC

* ************************************ ******************

CN 120025 GGTTCGGTCTATGTTAGTGTTTTTTCTATGACCATGTGAGATACATGAGATAACCAAATC

CN 120013 GGTTCGGTCTTTGTTAGTGTTTTTTCTATGACCATGTGAGATACATGAGATAACTAAATC

CS17CS1133 GGTTCGGTCTTTGTTAGTGTTTTTTCTATGACCATGTGAGATACATGAGATAACTAAATC

CN 120030 GGTTCGGTCTTTGTTAGTGTTTTTTCTATGACCATGTGAGATACATGAGATAACTAAATC

CO46 NCBI GGTTCGGTCTTTGTTAGTGTTTTTTCTATGACCATGTGAGATACATGAGATAACTAAATC

DH55 ref genome GGTTCGGTCTTTGTTAGTGTTTTTTCTATGACCATGTGAGATACATGAGATAACTAAATC

Hoga GGTTCGGTCTTTGTTAGTGTTTTTTCTATGACCATGTGAGATACATGAGATAACTAAATC

CAM 236 GGTTCGGTCTTTGTTAGTGTTTTTTCTATGACCATGTGAGATACATGAGATAACTAAATC

09-CS0040 GGTTCGGTCTTTGTTAGTGTTTTTTCTATGACCATGTGAGATACATGAGATAACTAAATC

CN 119205 GGTTCGGTCTTTGTTAGTGTTTTTTCTATGACCATGTGAGATACATGAGATAACTAAATC

Yellowstone GGTTCGGTCTTTGTTAGTGTTTTTTCTATGACCATGTGAGATACATGAGATAACTAAATC

CN 120017 GGTTCGGTCTTTGTTAGTGTTTTTTCTATGACCATGTGAGATACATGAGATAACTAAATC

CN 119300 GGTTCGGTCTTTGTTAGTGTTTTTTCTATGACCATGTGAGATACATGAGATAACTAAATC

Jasper GGTTCGGTCTTTGTTAGTGTTTTTTCTATGACCATGTGAGATACATGAGATAACTAAATC

CN 120027 GGTTCGGTCTTTGTTAGTGTTTTTTCTATGACCATGTGAGATACATGAGATAACTAAATC

CN 113754 GGTTCGGTCTTTGTTAGTGTTTTTTCTATGACCATGTGAGATACATGAGATAACTAAATC

Joelle AAFC GGTTCGGTCTTTGTTAGTGTTTTTTCTATGACCATGTGAGATACATGAGATAACTAAATC

Joelle NCBI GGTTCGGTCTTTGTTAGTGTTTTTTCTATGACCATGTGAGATACATGAGATAACTAAATC

Joelle phyto GGTTCGGTCTTTGTTAGTGTTTTTTCTATGACCATGTGAGATACATGAGATAACTAAATC

Blaine Creek GGTTCGGTCTTTGTTAGTGTTTTTTCTATGACCATGTGAGATACATGAGATAACTAAATC

CAM 241 GGTTCGGTCTTTGTTAGTGTTTTTTCTATGACCATGTGAGATACATGAGATAACTAAATC

CN 119294 GGTTCGGTCTTTGTTAGTGTTTTTTCTATGACCATGTGAGATACATGAGATAACTAAATC

********** ******************************************* *****

CN 120025 TGTGGAAGAACAATGTCGTGTTGAGCTTAAGCTTCTTACTTTTTTTTTCTTCTTTTCTCT

CN 120013 TATGGAAGAACAATGTCGTGTTGAGCTTAAGCTTCTTAC-TTTTTTCTCTTCTTTTCTCT

CS17CS1133 TATGGAAGAACAATGTCGTGTTGAGCTTAAGCTTCTTAC-TTTTTTCTCTTCTTTTCTCT

CN 120030 TATGGAAGAACAATGTCGTGTTGAGCTTAAGCTTCTTAC-TTTTTTCTCTTCTTTTCTCT

CO46 NCBI TATGGAAGAACAATGTCGTGTTGAGCTTAAGCTTCTTAC-TTTTTTCTCTTCTTTTCTCT

DH55 ref genome TATGGAAGAACAATGTCGTGTTGAGCTTAAGCTTCTTAC-TTTTTTCTCTTCTTTTCTCT

Hoga TATGGAAGAACAATGTCGTGTTGAGCTTAAGCTTCTTAC-TTTTTTCTCTTCTTTTCTCT

CAM 236 TATGGAAGAACAATGTCGTGTTGAGCTTAAGCTTCTTAC-TTTTTTCTCTTCTTTTCTCT

09-CS0040 TATGGAAGAACAATGTCGTGTTGAGCTTAAGCTTCTTAC-TTTTTTCTCTTCTTTTCTCT

CN 119205 TATGGAAGAACAATGTCGTGTTGAGCTTAAGCTTCTTAC-TTTTTTCTCTTCTTTTCTCT

Yellowstone TATGGAAGAACAATGTCGTGTTGAGCTTAAGCTTCTTAC-TTTTTTCTCTTCTTTTCTCT

CN 120017 TATGGAAGAACAATGTCGTGTTGAGCTTAAGCTTCTTAC-TTTTTTCTCTTCTTTTCTCT

CN 119300 TATGGAAGAACAATGTCGTGTTGAGCTTAAGCTTCTTAC-TTTTTTCTCTTCTTTTCTCT

Jasper TATGGAAGAACAATGTCGTGTTGAGCTTAAGCTTCTTAC-TTTTTTCTCTTCTTTTCTCT

CN 120027 TATGGAAGAACAATGTCGTGTTGAGCTTAAGCTTCTTAC-TTTTTTCTCTTCTTTTCTCT

CN 113754 TATGGAAGAACAATGTCGTGTTGAGCTTAAGCTTCTTAC-TTTTTTCTCTTCTTTTCTCT

Joelle AAFC TATGGAAGAACAATGTCGTGTTGAGCTTAAGCTTCTTAC-TTTTTTCTCTTCTTTTCTCT

Joelle NCBI TATGGAAGAACAATGTCGTGTTGAGCTTAAGCTTCTTAC-TTTTTTCTCTTCTTTTCTCT

Joelle phyto TATGGAAGAACAATGTCGTGTTGAGCTTAAGCTTCTTAC-TTTTTTCTCTTCTTTTCTCT

Blaine Creek TATGGAAGAACAATGTCGTGTTGAGCTTAAGCTTCTTAC-TTTTTTCTCTTCTTTTCTCT

CAM 241 TATGGAAGAACAATGTCGTGTTGAGCTTAAGCTTCTTAC-TTTTTTCTCTTCTTTTCTCT

CN 119294 TATGGAAGAACAATGTCGTGTTGAGCTTAAGCTTCTTAC-TTTTTTCTCTTCTTTTCTCT

* ************************************* ****** *************

CN 120025 CTCTATCTCTCTCTCTCTATTTCCTTAAAAAAATTTCTGCATGGATTTTTATTTTATTTG

CN 120013 CTCTATCTCTCTCTCTT--TTTCCTTAAATTTTTTTCTGCATGGA-TTTTATTTTATTTG

CS17CS1133 CTCTATCTCTCTCTCTT--TTTCCTTAAATTTTTTTCTGCATGGA-TTTTATTTTATTTG

CN 120030 CTCTATCTCTCTCTCTT--TTTCCTTAAATTTTTTTCTGCATGGA-TTTTATTTTATTTG

CO46 NCBI CTCTATCTCTCTCTCTT--TTTCCTTAAATTTTTTTCTGCATGGA-TTTTATTTTATTTG

DH55 ref genome CTCTATCTCTCTCTCTT--TTTCCTTAAATTTTTTTCTGCATGGA-TTTTATTTTATTTG

Hoga CTCTATCTCTCTCTCTT--TTTCCTTAAATTTTTTTCTGCATGGA-TTTTATTTTATTTG

CAM 236 CTCTATCTCTCTCTCTT--TTTCCTTAAATTTTTTTCTGCATGGA-TTTTATTTTATTTG

09-CS0040 CTCTATCTCTCTCTCTT--TTTCCTTAAATTTTTTTCTGCATGGA-TTTTATTTTATTTG

CN 119205 CTCTATCTCTCTCTCTT--TTTCCTTAAATTTTTTTCTGCATGGA-TTTTATTTTATTTG

Yellowstone CTCTATCTCTCTCTCTT--TTTCCTTAAATTTTTTTCTGCATGGA-TTTTATTTTATTTG

CN 120017 CTCTATCTCTCTCTCTT--TTTCCTTAAATTTTTTTCTGCATGGA-TTTTATTTTATTTG

CN 119300 CTCTATCTCTCTCTCTT--TTTCCTTAAATTTTTTTCTGCATGGA-TTTTATTTTATTTG

Jasper CTCTATCTCTCTCTCTT--TTTCCTTAAATTTTTTTCTGCATGGA-TTTTATTTTATTTG

CN 120027 CTCTATCTCTCTCTCTT--TTTCCTTAAATTTTTTTCTGCATGGA-TTTTATTTTATTTG

CN 113754 CTCTATCTCTCTCTCTT--TTTCCTTAAATTTTTTTCTGCATGGA-TTTTATTTTATTTG

Joelle AAFC CTCTATCTCTCTCTCTT--TTTCCTTAAATTTTTTTCTGCATGGA-TTTTATTTTATTTG

Joelle NCBI CTCTATCTCTCTCTCTT--TTTCCTTAAATTTTTTTCTGCATGGA-TTTTATTTTATTTG

Joelle phyto CTCTATCTCTCTCTCTT--TTTCCTTAAATTTTTTTCTGCATGGA-TTTTATTTTATTTG

Blaine Creek CTCTATCTCTCTCTCTT--TTTCCTTAAATTTTTTTCTGCATGGA-TTTTATTTTATTTG

CAM 241 CTCTATCTCTCTCTCTT--TTTCCTTAAATTTTTTTCTGCATGGA-TTTTATTTTATTTG

CN 119294 CTCTATCTCTCTCTCTT--TTTCCTTAAATTTTTTTCTGCATGGA-TTTTATTTTATTTG

**************** ********** ************ **************

CN 120025 GAAATTTTTTTGCATGTCCTTCGAGATTTGCTTGACACGTTCTGCTGCGTACTCGATGTT

CN 120013 GAAA-TTTTTTGCATGTCATTTGAGATTTGCTTGACACGTTCTGCTGCGTACTCGATGTT

CS17CS1133 GAAA-TTTTTTGCATGTCATTTGAGATTTGCTTGACACGTTCTGCTGCGTACTCGATGTT

CN 120030 GAAA-TTTTTTGCATGTCATTTGAGATTTGCTTGACACGTTCTGCTGCGTACTCGATGTT

CO46 NCBI GAAA-TTTTTTGCATGTCATTTGAGATTTGCTTGACACGTTCTGCTGCGTACTCGATGTT

DH55 ref genome GAAA-TTTTTTGCATGTCATTTGAGATTTGCTTGACACGTTCTGCTGCGTACTCGATGTT

Hoga GAAA-TTTTTTGCATGTCATTTGAGATTTGCTTGACACGTTCTGCTGCGTACTCGATGTT

CAM 236 GAAA-TTTTTTGCATGTCATTTGAGATTTGCTTGACACGTTCTGCTGCGTACTCGATGTT

09-CS0040 GAAA-TTTTTTGCATGTCATTTGAGATTTGCTTGACACGTTCTGCTGCGTACTCGATGTT

CN 119205 GAAA-TTTTTTGCATGTCATTTGAGATTTGCTTGACACGTTCTGCTGCGTACTCGATGTT

Yellowstone GAAA-TTTTTTGCATGTCATTTGAGATTTGCTTGACACGTTCTGCTGCGTACTCGATGTT

CN 120017 GAAA-TTTTTTGCATGTCATTTGAGATTTGCTTGACACGTTCTGCTGCGTACTCGATGTT

CN 119300 GAAA-TTTTTTGCATGTCATTTGAGATTTGCTTGACACGTTCTGCTGCGTACTCGATGTT

Jasper GAAA-TTTTTTGCATGTCATTTGAGATTTGCTTGACACGTTCTGCTGCGTACTCGATGTT

CN 120027 GAAA-TTTTTTGCATGTCATTTGAGATTTGCTTGACACGTTCTGCTGCGTACTCGATGTT

CN 113754 GAAA-TTTTTTGCATGTCATTTGAGATTTGCTTGACACGTTCTGCTGCGTACTCGATGTT

Joelle AAFC GAAA-TTTTTTGCATGTCATTTGAGATTTGCTTGACACGTTCTGCTGCGTACTCGATGTT

Joelle NCBI GAAA-TTTTTTGCATGTCATTTGAGATTTGCTTGACACGTTCTGCTGCGTACTCGATGTT

Joelle phyto GAAA-TTTTTTGCATGTCATTTGAGATTTGCTTGACACGTTCTGCTGCGTACTCGATGTT

Blaine Creek GAAA-TTTTTTGCATGTCATTTGAGATTTGCTTGACACGTTCTGCTGCGTACTCGATGTT

CAM 241 GAAA-TTTTTTGCATGTCATTTGAGATTTGCTTGACACGTTCTGCTGCGTACTCGATGTT

CN 119294 GAAA-TTTTTTGCATGTCATTTGAGATTTGCTTGACACGTTCTGCTGCGTACTCGATGTT

**** ************* ** **************************************

CN 120025 GTCCACTGAAGTTTCAAAGCCGTCTTTGATTGCTACTTAGCTTTAGGGATT-AATTCCAT

CN 120013 GTCTAGTGAAGTTTC-AAGCCGTCTTTGATTGCTACTTGGCTTTAGGGATT-AATTCCCT

CS17CS1133 GTCTAGTGAAGTTTC-AAGCCGTCTTTGATTGCTACTTGGCTTTAGGGATT-AATTCCCT

CN 120030 GTCTAGTGAAGTTTC-AAGCCGTCTTTGATTGCTACTTGGCTTTAGGGATT-AATTCCCT

CO46 NCBI GTCTAGTGAAGTTTC-AAGCCGTCTTTGATTGCTACTTGGCTTTAGGGATTNAATTCCCT

DH55 ref genome GTCTAGTGAAGTTTC-AAGCCGTCTTTGATTGCTACTTGGCTTTAGGGATT-AATTCCCT

Hoga GTCTAGTGAAGTTTC-AAGCCGTCTTTGATTGCTACTTGGCTTTAGGGATT-AATTCCCT

CAM 236 GTCTAGTGAAGTTTC-AAGCCGTCTTTGATTGCTACTTGGCTTTAGGGATT-AATTCCCT

09-CS0040 GTCTAGTGAAGTTTC-AAGCCGTCTTTGATTGCTACTTGGCTTTAGGGATT-AATTCCCT

CN 119205 GTCTAGTGAAGTTTC-AAGCCGTCTTTGATTGCTACTTGGCTTTAGGGATT-AATTCCCT

Yellowstone GTCTAGTGAAGTTTC-AAGCCGTCTTTGATTGCTACTTGGCTTTAGGGATT-AATTCCCT

CN 120017 GTCTAGTGAAGTTTC-AAGCCGTCTTTGATTGCTACTTGGCTTTAGGGATT-AATTCCCT

CN 119300 GTCTAGTGAAGTTTC-AAGCCGTCTTTGATTGCTACTTGGCTTTAGGGATT-AATTCCCT

Jasper GTCTAGTGAAGTTTC-AAGCCGTCTTTGATTGCTACTTGGCTTTAGGGATT-AATTCCCT

CN 120027 GTCTAGTGAAGTTTC-AAGCCGTCTTTGATTGCTACTTGGCTTTAGGGATT-AATTCCCT

CN 113754 GTCTAGTGAAGTTTC-AAGCCGTCTTTGATTGCTACTTGGCTTTAGGGATT-AATTCCCT

Joelle AAFC GTCTAGTGAAGTTTC-AAGCCGTCTTTGATTGCTACTTGGCTTTAGGGATT-AATTCCCT

Joelle NCBI GTCTAGTGAAGTTTC-AAGCCGTCTTTGATTGCTACTTGGCTTTAGGGATT-AATTCCCT

Joelle phyto GTCTAGTGAAGTTTC-AAGCCGTCTTTGATTGCTACTTGGCTTTAGGGATT-AATTCCCT

Blaine Creek GTCTAGTGAAGTTTC-AAGCCGTCTTTGATTGCTACTTGGCTTTAGGGATT-AATTCCCT

CAM 241 GTCTAGTGAAGTTTC-AAGCCGTCTTTGATTGCTACTTGGCTTTAGGGATT-AATTCCCT

CN 119294 GTCTAGTGAAGTTTC-AAGCCGTCTTTGATTGCTACTTGGCTTTAGGGATT-AATTCCCT

*** * ********* ********************** ************ ****** *

CN 120025 ATGTTTGTGATTAGTTTTTATATCAGAATTGCTAAATGATTCACTGGATTTTTTTTTTCT

CN 120013 ATGTTTCTGATTAGTTTTTATGTCAGAATTGCTGGA-----------------TCTCTCT

CS17CS1133 ATGTTTCTGATTAGTTTTTATGTCAGAATTGCTGGA-----------------TCTCTCT

CN 120030 ATGTTTCTGATTAGTTTTTATGTCAGAATTGCTGGA-----------------TCTCTCT

CO46 NCBI ATGTTTCTGATTAGTTTTTATGTCAGAATTGCTGGA-----------------TCTCTCT

DH55 ref genome ATGTTTCTGATTAGTTTTTATGTCAGAATTGCTGGA-----------------TCTCTCT

Hoga ATGTTTCTGATTAGTTTTTATGTCAGAATTGCTGGA-----------------TCTCTCT

CAM 236 ATGTTTCTGATTAGTTTTTATGTCAGAATTGCTGGA-----------------TCTCTCT

09-CS0040 ATGTTTCTGATTAGTTTTTATGTCAGAATTGCTGGA-----------------TCTCTCT

CN 119205 ATGTTTCTGATTAGTTTTTATGTCAGAATTGCTGGA-----------------TCTCTCT

Yellowstone ATGTTTCTGATTAGTTTTTATGTCAGAATTGCTGGA-----------------TCTCTCT

CN 120017 ATGTTTCTGATTAGTTTTTATGTCAGAATTGCTGGA-----------------TCTCTCT

CN 119300 ATGTTTCTGATTAGTTTTTATGTCAGAATTGCTGGA-----------------TCTCTCT

Jasper ATGTTTCTGATTAGTTTTTATGTCAGAATTGCTGGA-----------------TCTCTCT

CN 120027 ATGTTTCTGATTAGTTTTTATGTCAGAATTGCTGGA-----------------TCTCTCT

CN 113754 ATGTTTCTGATTAGTTTTTATGTCAGAATTGCTGGA-----------------TCTCTCT

Joelle AAFC ATGTTTCTGATTAGTTTTTATGTCAGAATTGCTGGA-----------------TCTCTCT

Joelle NCBI ATGTTTCTGATTAGTTTTTATGTCAGAATTGCTGGA-----------------TCTCTCT

Joelle phyto ATGTTTCTGATTAGTTTTTATGTCAGAATTGCTGGA-----------------TCTCTCT

Blaine Creek ATGTTTCTGATTAGTTTTTATGTCAGAATTGCTGGA-----------------TCTCTCT

CAM 241 ATGTTTCTGATTAGTTTTTATGTCAGAATTGCTGGA-----------------TCTCTCT

CN 119294 ATGTTTCTGATTAGTTTTTATGTCAGAATTGCTGGA-----------------TCTCTCT

****** ************** *********** * * * ***

CN 120025 TTTTCTGAAGTGATTAAAACTCATTCGATCTCTTTGGATTTGTATCCAGTGCAATGTACC

CN 120013 TTTTTCTAAGTGATTAAAATTCATTAGATCTCTTTGGATTTGTATCCAGTGCAATGTACC

CS17CS1133 TTTTTCTAAGTGATTAAAATTCATTAGATCTCTTTGGATTTGTATCCAGTGCAATGTACC

CN 120030 TTTTTCTAAGTGATTAAAATTCATTAGATCTCTTTGGATTTGTATCCAGTGCAATGTACC

CO46 NCBI TTTTTCTAAGTGATTAAAATTCATTAGATCTCTTTGGATTTGTATCCAGTGCAATGTACC

DH55 ref genome TTTTTCTAAGTGATTAAAATTCATTAGATCTCTTTGGATTTGTATCCAGTGCAATGTACC

Hoga TTTTTCTAAGTGATTAAAATTCATTAGATCTCTTTGGATTTGTATCCAGTGCAATGTACC

CAM 236 TTTTTCTAAGTGATTAAAATTCATTAGATCTCTTTGGATTTGTATCCAGTGCAATGTACC

09-CS0040 TTTTTCTAAGTGATTAAAATTCATTAGATCTCTTTGGATTTGTATCCAGTGCAATGTACC

CN 119205 TTTTTCTAAGTGATTAAAATTCATTAGATCTCTTTGGATTTGTATCCAGTGCAATGTACC

Yellowstone TTTTTCTAAGTGATTAAAATTCATTAGATCTCTTTGGATTTGTATCCAGTGCAATGTACC

CN 120017 TTTTTCTAAGTGATTAAAATTCATTAGATCTCTTTGGATTTGTATCCAGTGCAATGTACC

CN 119300 TTTTTCTAAGTGATTAAAATTCATTAGATCTCTTTGGATTTGTATCCAGTGCAATGTACC

Jasper TTTTTCTAAGTGATTAAAATTCATTAGATCTCTTTGGATTTGTATCCAGTGCAATGTACC

CN 120027 TTTTTCTAAGTGATTAAAATTCATTAGATCTCTTTGGATTTGTATCCAGTGCAATGTACC

CN 113754 TTTTTCTAAGTGATTAAAATTCATTAGATCTCTTTGGATTTGTATCCAGTGCAATGTACC

Joelle AAFC TTTTTCTAAGTGATTAAAATTCATTAGATCTCTTTGGATTTGTATCCAGTGCAATGTACC

Joelle NCBI TTTTTCTAAGTGATTAAAATTCATTAGATCTCTTTGGATTTGTATCCAGTGCAATGTACC

Joelle phyto TTTTTCTAAGTGATTAAAATTCATTAGATCTCTTTGGATTTGTATCCAGTGCAATGTACC

Blaine Creek TTTTTCTAAGTGATTAAAATTCATTAGATCTCTTTGGATTTGTATCCAGTGCAATGTACC

CAM 241 TTTTTCTAAGTGATTAAAATTCATTAGATCTCTTTGGATTTGTATCCAGTGCAATGTACC

CN 119294 TTTTTCTAAGTGATTAAAATTCATTAGATCTCTTTGGATTTGTATCCAGTGCAATGTACC

**** ************ ***** **********************************

CN 120025 TTCTGGAGATCTATGCAAATCCGAGAGATCCATAGAATTTCAATGGGGTT-AATGCTGAA

CN 120013 TTCGGGAGATCTATGCAAATCCGAGAGATCCATAGAATTTCAATGGGGTTAAATGCTGAA

CS17CS1133 TTCGGGAGATCTATGCAAATCCGAGAGATCCATAGAATTTCAATGGGGTTAAATGCTGAA

CN 120030 TTCGGGAGATCTATGCAAATCCGAGAGATCCATAGAATTTCAATGGGGTTAAATGCTGAA

CO46 NCBI TTCGGGAGATCTATGCAAATCCGAGAGATCCATAGAATTTCAATGGGGTTAAATGCTGAA

DH55 ref genome TTCGGGAGATCTATGCAAATCCGAGAGATCCATAGAATTTCAATGGGGTTAAATGCTGAA

Hoga TTCGGGAGATCTATGCAAATCCGAGAGATCCATAGAATTTCAATGGGGTTAAATGCTGAA

CAM 236 TTCGGGAGATCTATGCAAATCCGAGAGATCCATAGAATTTCAATGGGGTTAAATGCTGAA

09-CS0040 TTCGGGAGATCTATGCAAATCCGAGAGATCCATAGAATTTCAATGGGGTTAAATGCTGAA

CN 119205 TTCGGGAGATCTATGCAAATCCGAGAGATCCATAGAATTTCAATGGGGTTAAATGCTGAA

Yellowstone TTCGGGAGATCTATGCAAATCCGAGAGATCCATAGAATTTCAATGGGGTTAAATGCTGAA

CN 120017 TTCGGGAGATCTATGCAAATCCGAGAGATCCATAGAATTTCAATGGGGTTAAATGCTGAA

CN 119300 TTCGGGAGATCTATGCAAATCCGAGAGATCCATAGAATTTCAATGGGGTTAAATGCTGAA

Jasper TTCGGGAGATCTATGCAAATCCGAGAGATCCATAGAATTTCAATGGGGTTAAATGCTGAA

CN 120027 TTCGGGAGATCTATGCAAATCCGAGAGATCCATAGAATTTCAATGGGGTTAAATGCTGAA

CN 113754 TTCGGGAGATCTATGCAAATCCGAGAGATCCATAGAATTTCAATGGGGTTAAATGCTGAA

Joelle AAFC TTCGGGAGATCTATGCAAATCCGAGAGATCCATAGAATTTCAATGGGGTTAAATGCTGAA

Joelle NCBI TTCGGGAGATCTATGCAAATCCGAGAGATCCATAGAATTTCAATGGGGTTAAATGCTGAA

Joelle phyto TTCGGGAGATCTATGCAAATCCGAGAGATCCATAGAATTTCAATGGGGTTAAATGCTGAA

Blaine Creek TTCGGGAGATCTATGCAAATCCGAGAGATCCATAGAATTTCAATGGGGTTAAATGCTGAA

CAM 241 TTCGGGAGATCTATGCAAATCCGAGAGATCCATAGAATTTCAATGGGGTTAAATGCTGAA

CN 119294 TTCGGGAGATCTATGCAAATCCGAGAGATCCATAGAATTTCAATGGGGTTAAATGCTGAA

*** ********************************************** *********

CN 120025 TAATGTATACCACATTGTGCAGCTATTGACTATAAGTTTATTGTTGATCTTCTATGAATT

CN 120013 TAATGCATACCACATTGTGCAGTTACTG-----------ATTGTTGATCTTCTATGGGTT

CS17CS1133 TAATGCATACCACATTGTGCAGTTACTG-----------ATTGTTGATCTTCTATGGGTT

CN 120030 TAATGCATACCACATTGTGCAGTTACTG-----------ATTGTTGATCTTCTATGGGTT

CO46 NCBI TAATGCATACCACATTGTGCAGTTACTG-----------ATTGTTGATCTTCTATGGGTT

DH55 ref genome TAATGCATACCACATTGTGCAGTTACTG-----------ATTGTTGATCTTCTATGGGTT

Hoga TAATGCATACCACATTGTGCAGTTACTG-----------ATTGTTGATCTTCTATGGGTT

CAM 236 TAATGCATACCACATTGTGCAGTTACTG-----------ATTGTTGATCTTCTATGGGTT

09-CS0040 TAATGCATACCACATTGTGCAGTTACTG-----------ATTGTTGATCTTCTATGGGTT

CN 119205 TAATGCATACCACATTGTGCAGTTACTG-----------ATTGTTGATCTTCTATGGGTT

Yellowstone TAATGCATACCACATTGTGCAGTTACTG-----------ATTGTTGATCTTCTATGGGTT

CN 120017 TAATGCATACCACATTGTGCAGTTACTG-----------ATTGTTGATCTTCTATGGGTT

CN 119300 TAATGCATACCACATTGTGCAGTTACTG-----------ATTGTTGATCTTCTATGGGTT

Jasper TAATGCATACCACATTGTGCAGTTACTG-----------ATTGTTGATCTTCTATGGGTT

CN 120027 TAATGCATACCACATTGTGCAGTTACTG-----------ATTGTTGATCTTCTATGGGTT

CN 113754 TAATGCATACCACATTGTGCAGTTACTG-----------ATTGTTGATCTTCTATGGGTT

Joelle AAFC TAATGCATACCACATTGTGCAGTTACTG-----------ATTGTTGATCTTCTATGGGTT

Joelle NCBI TAATGCATACCACATTGTGCAGTTACTG-----------ATTGTTGATCTTCTATGGGTT

Joelle phyto TAATGCATACCACATTGTGCAGTTACTG-----------ATTGTTGATCTTCTATGGGTT

Blaine Creek TAATGCATACCACATTGTGCAGTTACTG-----------ATTGTTGATCTTCTATGGGTT

CAM 241 TAATGCATACCACATTGTGCAGTTACTG-----------ATTGTTGATCTTCTATGGGTT

CN 119294 TAATGCATACCACATTGTGCAGTTACTG-----------ATTGTTGATCTTCTATGGGTT

***** **************** ** ** ***************** **

CN 120025 TATATCTTTGTCATGGACCTATATTACTTGATGATTATCCAAATTAGTGTTTCTAATTGA

CN 120013 TCTCTCTTTGTCATGGACC--TATCACTTGATGATTATCCAAATTAGTGTTTCTAATTGA

CS17CS1133 TCTCTCTTTGTCATGGACC--TATCACTTGATGATTATCCAAATTAGTGTTTCTAATTGA

CN 120030 TCTCTCTTTGTCATGGACC--TATCACTTGATGATTATCCAAATTAGTGTTTCTAATTGA

CO46 NCBI TCTCTCTTTGTCATGGACC--TATCACTTGATGATTATCCAAATTAGTGTTTCTAATTGA

DH55 ref genome TCTCTCTTTGTCATGGACC--TATCACTTGATGATTATCCAAATTAGTGTTTCTAATTGA

Hoga TCTCTCTTTGTCATGGACC--TATCACTTGATGATTATCCAAATTAGTGTTTCTAATTGA

CAM 236 TCTCTCTTTGTCATGGACC--TATCACTTGATGATTATCCAAATTAGTGTTTCTAATTGA

09-CS0040 TCTCTCTTTGTCATGGACC--TATCACTTGATGATTATCCAAATTAGTGTTTCTAATTGA

CN 119205 TCTCTCTTTGTCATGGACC--TATCACTTGATGATTATCCAAATTAGTGTTTCTAATTGA

Yellowstone TCTCTCTTTGTCATGGACC--TATCACTTGATGATTATCCAAATTAGTGTTTCTAATTGA

CN 120017 TCTCTCTTTGTCATGGACC--TATCACTTGATGATTATCCAAATTAGTGTTTCTAATTGA

CN 119300 TCTCTCTTTGTCATGGACC--TATCACTTGATGATTATCCAAATTAGTGTTTCTAATTGA

Jasper TCTCTCTTTGTCATGGACC--TATCACTTGATGATTATCCAAATTAGTGTTTCTAATTGA

CN 120027 TCTCTCTTTGTCATGGACC--TATCACTTGATGATTATCCAAATTAGTGTTTCTAATTGA

CN 113754 TCTCTCTTTGTCATGGACC--TATCACTTGATGATTATCCAAATTAGTGTTTCTAATTGA

Joelle AAFC TCTCTCTTTGTCATGGACC--TATCACTTGATGATTATCCAAATTAGTGTTTCTAATTGA

Joelle NCBI TCTCTCTTTGTCATGGACC--TATCACTTGATGATTATCCAAATTAGTGTTTCTAATTGA

Joelle phyto TCTCTCTTTGTCATGGACC--TATCACTTGATGATTATCCAAATTAGTGTTTCTAATTGA

Blaine Creek TCTCTCTTTGTCATGGACC--TATCACTTGATGATTATCCAAATTAGTGTTTCTAATTGA

CAM 241 TCTCTCTTTGTCATGGACC--TATCACTTGATGATTATCCAAATTAGTGTTTCTAATTGA

CN 119294 TCTCTCTTTGTCATGGACC--TATCACTTGATGATTATCCAAATTAGTGTTTCTAATTGA

* * *************** *** ***********************************

CN 120025 TTATTAACTTTTTTTATATATATAGTACTTGTGAATATTGGTAGCTTTAAAAAAACTCAG

CN 120013 T---------------TATATATAGTACTTGTGAATATTGGTAGCTTT-AAAAAACTTAG

CS17CS1133 T---------------TATATATAGTACTTGTGAATATTGGTAGCTTT-AAAAAACTTAG

CN 120030 T---------------TATATATAGTACTTGTGAATATTGGTAGCTTT-AAAAAACTTAG

CO46 NCBI T---------------TATATATAGTACTTGTGAATATTGGTAGCTTT-AAAAAACTTAG

DH55 ref genome T---------------TATATATAGTACTTGTGAATATTGGTAGCTTT-AAAAAACTTAG

Hoga T---------------TATATATAGTACTTGTGAATATTGGTAGCTTT-AAAAAACTTAG

CAM 236 T---------------TATATATAGTACTTGTGAATATTGGTAGCTTT-AAAAAACTTAG

09-CS0040 T---------------TATATATAGTACTTGTGAATATTGGTAGCTTT-AAAAAACTTAG

CN 119205 T---------------TATATATAGTACTTGTGAATATTGGTAGCTTT-AAAAAACTTAG

Yellowstone T---------------TATATATAGTACTTGTGAATATTGGTAGCTTT-AAAAAACTTAG

CN 120017 T---------------TATATATAGTACTTGTGAATATTGGTAGCTTT-AAAAAACTTAG

CN 119300 T---------------TATATATAGTACTTGTGAATATTGGTAGCTTT-AAAAAACTTAG

Jasper T---------------TATATATAGTACTTGTGAATATTGGTAGCTTT-AAAAAACTTAG

CN 120027 T---------------TATATATAGTACTTGTGAATATTGGTAGCTTT-AAAAAACTTAG

CN 113754 T---------------TATATATAGTACTTGTGAATATTGGTAGCTTT-AAAAAACTTAG

Joelle AAFC T---------------TATATATAGTACTTGTGAATATTGGTAGCTTT-AAAAAACTTAG

Joelle NCBI T---------------TATATATAGTACTTGTGAATATTGGTAGCTTT-AAAAAACTTAG

Joelle phyto T---------------TATATATAGTACTTGTGAATATTGGTAGCTTT-AAAAAACTTAG

Blaine Creek T---------------TATATATAGTACTTGTGAATATTGGTAGCTTT-AAAAAACTTAG

CAM 241 T---------------TATATATAGTACTTGTGAATATTGGTAGCTTT-AAAAAACTTAG

CN 119294 T---------------TATATATAGTACTTGTGAATATTGGTAGCTTT-AAAAAACTTAG

* ******************************** ******** **

CN 120025 CCTCACAATTAGTCCTTACCGCACATATGCTACTTAAGCTATGTGATCTGGGTTGGATTG

CN 120013 CCTCGCAATTAGTCCTTACCGCACATATGCTACTTAAGCTATGTGATCTGGTATCGATTG

CS17CS1133 CCTCGCAATTAGTCCTTACCGCACATATGCTACTTAAGCTATGTGATCTGGTATCGATTG

CN 120030 CCTCGCAATTAGTCCTTACCGCACATATGCTACTTAAGCTATGTGATCTGGTATCGATTG

CO46 NCBI CCTCGCAATTAGTCCTTACCGCACATATGCTACTTAAGCTATGTGATCTGGTATCGATTG

DH55 ref genome CCTCGCAATTAGTCCTTACCGCACATATGCTACTTAAGCTATGTGATCTGGTATCGATTG

Hoga CCTCGCAATTAGTCCTTACCGCACATATGCTACTTAAGCTATGTGATCTGGTATCGATTG

CAM 236 CCTCGCAATTAGTCCTTACCGCACATATGCTACTTAAGCTATGTGATCTGGTATCGATTG

09-CS0040 CCTCGCAATTAGTCCTTACCGCACATATGCTACTTAAGCTATGTGATCTGGTATCGATTG

CN 119205 CCTCGCAATTAGTCCTTACCGCACATATGCTACTTAAGCTATGTGATCTGGTATCGATTG

Yellowstone CCTCGCAATTAGTCCTTACCGCACATATGCTACTTAAGCTATGTGATCTGGTATCGATTG

CN 120017 CCTCGCAATTAGTCCTTACCGCACATATGCTACTTAAGCTATGTGATCTGGTATCGATTG

CN 119300 CCTCGCAATTAGTCCTTACCGCACATATGCTACTTAAGCTATGTGATCTGGTATCGATTG

Jasper CCTCGCAATTAGTCCTTACCGCACATATGCTACTTAAGCTATGTGATCTGGTATCGATTG

CN 120027 CCTCGCAATTAGTCCTTACCGCACATATGCTACTTAAGCTATGTGATCTGGTATCGATTG

CN 113754 CCTCGCAATTAGTCCTTACCGCACATATGCTACTTAAGCTATGTGATCTGGTATCGATTG

Joelle AAFC CCTCGCAATTAGTCCTTACCGCACATATGCTACTTAAGCTATGTGATCTGGTATCGATTG

Joelle NCBI CCTCGCAATTAGTCCTTACCGCACATATGCTACTTAAGCTATGTGATCTGGTATCGATTG

Joelle phyto CCTCGCAATTAGTCCTTACCGCACATATGCTACTTAAGCTATGTGATCTGGTATCGATTG

Blaine Creek CCTCGCAATTAGTCCTTACCGCACATATGCTACTTAAGCTATGTGATCTGGTATCGATTG

CAM 241 CCTCGCAATTAGTCCTTACCGCACATATGCTACTTAAGCTATGTGATCTGGTATCGATTG

CN 119294 CCTCGCAATTAGTCCTTACCGCACATATGCTACTTAAGCTATGTGATCTGGTATCGATTG

**** ********************************************** * *****

CN 120025 CGATTAATTGCAATT-TTGTATGCATCTTTAACACTTTGTGCCGCACATAATGTACATTA

CN 120013 CGATTAATTGCAATTGTTGTGTGCATCTTTAACACTTTGTGCCGCACATAATGTACATTA

CS17CS1133 CGATTAATTGCAATTGTTGTGTGCATCTTTAACACTTTGTGCCGCACATAATGTACATTA

CN 120030 CGATTAATTGCAATTGTTGTGTGCATCTTTAACACTTTGTGCCGCACATAATGTACATTA

CO46 NCBI CGATTAATTGCAATTGTTGTGTGCATCTTTAACACTTTGTGCCGCACATAATGTACATTA

DH55 ref genome CGATTAATTGCAATTGTTGTGTGCATCTTTAACACTTTGTGCCGCACATAATGTACATTA

Hoga CGATTAATTGCAATTGTTGTGTGCATCTTTAACACTTTGTGCCGCACATAATGTACATTA

CAM 236 CGATTAATTGCAATTGTTGTGTGCATCTTTAACACTTTGTGCCGCACATAATGTACATTA

09-CS0040 CGATTAATTGCAATTGTTGTGTGCATCTTTAACACTTTGTGCCGCACATAATGTACATTA

CN 119205 CGATTAATTGCAATTGTTGTGTGCATCTTTAACACTTTGTGCCGCACATAATGTACATTA

Yellowstone CGATTAATTGCAATTGTTGTGTGCATCTTTAACACTTTGTGCCGCACATAATGTACATTA

CN 120017 CGATTAATTGCAATTGTTGTGTGCATCTTTAACACTTTGTGCCGCACATAATGTACATTA

CN 119300 CGATTAATTGCAATTGTTGTGTGCATCTTTAACACTTTGTGCCGCACATAATGTACATTA

Jasper CGATTAATTGCAATTGTTGTGTGCATCTTTAACACTTTGTGCCGCACATAATGTACATTA

CN 120027 CGATTAATTGCAATTGTTGTGTGCATCTTTAACACTTTGTGCCGCACATAATGTACATTA

CN 113754 CGATTAATTGCAATTGTTGTGTGCATCTTTAACACTTTGTGCCGCACATAATGTACATTA

Joelle AAFC CGATTAATTGCAATTGTTGTGTGCATCTTTAACACTTTGTGCCGCACATAATGTACATTA

Joelle NCBI CGATTAATTGCAATTGTTGTGTGCATCTTTAACACTTTGTGCCGCACATAATGTACATTA

Joelle phyto CGATTAATTGCAATTGTTGTGTGCATCTTTAACACTTTGTGCCGCACATAATGTACATTA

Blaine Creek CGATTAATTGCAATTGTTGTGTGCATCTTTAACACTTTGTGCCGCACATAATGTACATTA

CAM 241 CGATTAATTGCAATTGTTGTGTGCATCTTTAACACTTTGTGCCGCACATAATGTACATTA

CN 119294 CGATTAATTGCAATTGTTGTGTGCATCTTTAACACTTTGTGCCGCACATAATGTACATTA

*************** **** ***************************************

CN 120025 ACTGGACTATTTTTAGACTAAAATTCATTGCTCTCTTGGATTTGCATACAAATACACTCC

CN 120013 ACTGGACTATTTTTAGACTAAAACTCATTGCTCTCTTGGATTTGCATACATATACATTCC

CS17CS1133 ACTGGACTATTTTTAGACTAAAACTCATTGCTCTCTTGGATTTGCATACATATACATTCC

CN 120030 ACTGGACTATTTTTAGACTAAAACTCATTGCTCTCTTGGATTTGCATACATATACATTCC

CO46 NCBI ACTGGACTATTTTTAGACTAAAACTCATTGCTCTCTTGGATTTGCATACATATACATTCC

DH55 ref genome ACTGGACTATTTTTAGACTAAAACTCATTGCTCTCTTGGATTTGCATACATATACATTCC

Hoga ACTGGACTATTTTTAGACTAAAACTCATTGCTCTCTTGGATTTGCATACATATACATTCC

CAM 236 ACTGGACTATTTTTAGACTAAAACTCATTGCTCTCTTGGATTTGCATACATATACATTCC

09-CS0040 ACTGGACTATTTTTAGACTAAAACTCATTGCTCTCTTGGATTTGCATACATATACATTCC

CN 119205 ACTGGACTATTTTTAGACTAAAACTCATTGCTCTCTTGGATTTGCATACATATACATTCC

Yellowstone ACTGGACTATTTTTAGACTAAAACTCATTGCTCTCTTGGATTTGCATACATATACATTCC

CN 120017 ACTGGACTATTTTTAGACTAAAACTCATTGCTCTCTTGGATTTGCATACATATACATTCC

CN 119300 ACTGGACTATTTTTAGACTAAAACTCATTGCTCTCTTGGATTTGCATACATATACATTCC

Jasper ACTGGACTATTTTTAGACTAAAACTCATTGCTCTCTTGGATTTGCATACATATACATTCC

CN 120027 ACTGGACTATTTTTAGACTAAAACTCATTGCTCTCTTGGATTTGCATACATATACATTCC

CN 113754 ACTGGACTATTTTTAGACTAAAACTCATTGCTCTCTTGGATTTGCATACATATACATTCC

Joelle AAFC ACTGGACTATTTTTAGACTAAAACTCATTGCTCTCTTGGATTTGCATACATATACATTCC

Joelle NCBI ACTGGACTATTTTTAGACTAAAACTCATTGCTCTCTTGGATTTGCATACATATACATTCC

Joelle phyto ACTGGACTATTTTTAGACTAAAACTCATTGCTCTCTTGGATTTGCATACATATACATTCC

Blaine Creek ACTGGACTATTTTTAGACTAAAACTCATTGCTCTCTTGGATTTGCATACATATACATTCC

CAM 241 ACTGGACTATTTTTAGACTAAAACTCATTGCTCTCTTGGATTTGCATACATATACATTCC

CN 119294 ACTGGACTATTTTTAGACTAAAACTCATTGCTCTCTTGGATTTGCATACATATACATTCC

*********************** ************************** ***** ***

CN 120025 GGGGAGATTTGTAAATAAAATTAGTACCATAGATCAATAATACTTTTGGTTCAAATGTAT

CN 120013 CGGGAGATTTATAAATAAAATTAGTACCATAGATCAATAATAATTTTGGTTCAAATGTAT

CS17CS1133 CGGGAGATTTATAAATAAAATTAGTACCATAGATCAATAATAATTTTGGTTCAAATGTAT

CN 120030 CGGGAGATTTATAAATAAAATTAGTACCATAGATCAATAATAATTTTGGTTCAAATGTAT

CO46 NCBI CGGGAGATTTATAAATAAAATTAGTACCATAGATCAATAATAATTTTGGTTCAAATGTAT

DH55 ref genome CGGGAGATTTATAAATAAAATTAGTACCATAGATCAATAATAATTTTGGTTCAAATGTAT

Hoga CGGGAGATTTATAAATAAAATTAGTACCATAGATCAATAATAATTTTGGTTCAAATGTAT

CAM 236 CGGGAGATTTATAAATAAAATTAGTACCATAGATCAATAATAATTTTGGTTCAAATGTAT

09-CS0040 CGGGAGATTTATAAATAAAATTAGTACCATAGATCAATAATAATTTTGGTTCAAATGTAT

CN 119205 CGGGAGATTTATAAATAAAATTAGTACCATAGATCAATAATAATTTTGGTTCAAATGTAT

Yellowstone CGGGAGATTTATAAATAAAATTAGTACCATAGATCAATAATAATTTTGGTTCAAATGTAT

CN 120017 CGGGAGATTTATAAATAAAATTAGTACCATAGATCAATAATAATTTTGGTTCAAATGTAT

CN 119300 CGGGAGATTTATAAATAAAATTAGTACCATAGATCAATAATAATTTTGGTTCAAATGTAT

Jasper CGGGAGATTTATAAATAAAATTAGTACCATAGATCAATAATAATTTTGGTTCAAATGTAT

CN 120027 CGGGAGATTTATAAATAAAATTAGTACCATAGATCAATAATAATTTTGGTTCAAATGTAT

CN 113754 CGGGAGATTTATAAATAAAATTAGTACCATAGATCAATAATAATTTTGGTTCAAATGTAT

Joelle AAFC CGGGAGATTTATAAATAAAATTAGTACCATAGATCAATAATAATTTTGGTTCAAATGTAT

Joelle NCBI CGGGAGATTTATAAATAAAATTAGTACCATAGATCAATAATAATTTTGGTTCAAATGTAT

Joelle phyto CGGGAGATTTATAAATAAAATTAGTACCATAGATCAATAATAATTTTGGTTCAAATGTAT

Blaine Creek CGGGAGATTTATAAATAAAATTAGTACCATAGATCAATAATAATTTTGGTTCAAATGTAT

CAM 241 CGGGAGATTTATAAATAAAATTAGTACCATAGATCAATAATAATTTTGGTTCAAATGTAT

CN 119294 CGGGAGATTTATAAATAAAATTAGTACCATAGATCAATAATAATTTTGGTTCAAATGTAT

********* ******************************* *****************

CN 120025 GCCACATTACTTGTAAAACTATTGACTAAAAGATTAAT-------TGG---GATATACAT

CN 120013 GTCACATTACTTGTAAAACTA-TGACTAAAATATTAATCTTTTTATGGATTAATATACAT

CS17CS1133 GTCACATTACTTGTAAAACTA-TGACTAAAATATTAATCTTTTTATGGATTAATATACAT

CN 120030 GTCACATTACTTGTAAAACTA-TGACTAAAATATTAATCTTTTTATGGATTAATATACAT

CO46 NCBI GTCACATTACTTGTAAAACTA-TGACTAAAATATTAATCTTTTTATGGATTAATATACAT

DH55 ref genome GTCACATTACTTGTAAAACTA-TGACTAAAATATTAATCTTTTTATGGATTAATATACAT

Hoga GTCACATTACTTGTAAAACTA-TGACTAAAATATTAATCTTTTTATGGATTAATATACAT

CAM 236 GTCACATTACTTGTAAAACTA-TGACTAAAATATTAATCTTTTTATGGATTAATATACAT

09-CS0040 GTCACATTACTTGTAAAACTA-TGACTAAAATATTAATCTTTTTATGGATTAATATACAT

CN 119205 GTCACATTACTTGTAAAACTA-TGACTAAAATATTAATCTTTTTATGGATTAATATACAT

Yellowstone GTCACATTACTTGTAAAACTA-TGACTAAAATATTAATCTTTTTATGGATTAATATACAT

CN 120017 GTCACATTACTTGTAAAACTA-TGACTAAAATATTAATCTTTTTATGGATTAATATACAT

CN 119300 GTCACATTACTTGTAAAACTA-TGACTAAAATATTAATCTTTTTATGGATTAATATACAT

Jasper GTCACATTACTTGTAAAACTA-TGACTAAAATATTAATCTTTTTATGGATTAATATACAT

CN 120027 GTCACATTACTTGTAAAACTA-TGACTAAAATATTAATCTTTTTATGGATTAATATACAT

CN 113754 GTCACATTACTTGTAAAACTA-TGACTAAAATATTAATCTTTTTATGGATTAATATACAT

Joelle AAFC GTCACATTACTTGTAAAACTA-TGACTAAAATATTAATCTTTTTATGGATTAATATACAT

Joelle NCBI GTCACATTACTTGTAAAACTA-TGACTAAAATATTAATCTTTTTATGGATTAATATACAT

Joelle phyto GTCACATTACTTGTAAAACTA-TGACTAAAATATTAATCTTTTTATGGATTAATATACAT

Blaine Creek GTCACATTACTTGTAAAACTA-TGACTAAAATATTAATCTTTTTATGGATTAATATACAT

CAM 241 GTCACATTACTTGTAAAACTA-TGACTAAAATATTAATCTTTTTATGGATTAATATACAT

CN 119294 GTCACATTACTTGTAAAACTA-TGACTAAAATATTAATCTTTTTATGGATTAATATACAT

* ******************* ********* ****** *** ********

CN 120025 GTTTTTAATAATGATTTCTCTCCTTTTTATGGATTTGCTTACTTGAAGATTAATTATCCA

CN 120013 GTTTTTTATAATGATTTCTCTCCTTTTTATGGATTTTCTTACTTGAAGATTAATTATCCA

CS17CS1133 GTTTTTTATAATGATTTCTCTCCTTTTTATGGATTTTCTTACTTGAAGATTAATTATCCA

CN 120030 GTTTTTTATAATGATTTCTCTCCTTTTTATGGATTTTCTTACTTGAAGATTAATTATCCA

CO46 NCBI GTTTTTTATAATGATTTCTCTCCTTTTTATGGATTTTCTTACTTGAAGATTAATTATCCA

DH55 ref genome GTTTTTTATAATGATTTCTCTCCTTTTTATGGATTTTCTTACTTGAAGATTAATTATCCA

Hoga GTTTTTTATAATGATTTCTCTCCTTTTTATGGATTTTCTTACTTGAAGATTAATTATCCA

CAM 236 GTTTTTTATAATGATTTCTCTCCTTTTTATGGATTTTCTTACTTGAAGATTAATTATCCA

09-CS0040 GTTTTTTATAATGATTTCTCTCCTTTTTATGGATTTTCTTACTTGAAGATTAATTATCCA

CN 119205 GTTTTTTATAATGATTTCTCTCCTTTTTATGGATTTTCTTACTTGAAGATTAATTATCCA

Yellowstone GTTTTTTATAATGATTTCTCTCCTTTTTATGGATTTTCTTACTTGAAGATTAATTATCCA

CN 120017 GTTTTTTATAATGATTTCTCTCCTTTTTATGGATTTTCTTACTTGAAGATTAATTATCCA

CN 119300 GTTTTTTATAATGATTTCTCTCCTTTTTATGGATTTTCTTACTTGAAGATTAATTATCCA

Jasper GTTTTTTATAATGATTTCTCTCCTTTTTATGGATTTTCTTACTTGAAGATTAATTATCCA

CN 120027 GTTTTTTATAATGATTTCTCTCCTTTTTATGGATTTTCTTACTTGAAGATTAATTATCCA

CN 113754 GTTTTTTATAATGATTTCTCTCCTTTTTATGGATTTTCTTACTTGAAGATTAATTATCCA

Joelle AAFC GTTTTTTATAATGATTTCTCTCCTTTTTATGGATTTTCTTACTTGAAGATTAATTATCCA

Joelle NCBI GTTTTTTATAATGATTTCTCTCCTTTTTATGGATTTTCTTACTTGAAGATTAATTATCCA

Joelle phyto GTTTTTTATAATGATTTCTCTCCTTTTTATGGATTTTCTTACTTGAAGATTAATTATCCA

Blaine Creek GTTTTTTATAATGATTTCTCTCCTTTTTATGGATTTTCTTACTTGAAGATTAATTATCCA

CAM 241 GTTTTTTATAATGATTTCTCTCCTTTTTATGGATTTTCTTACTTGAAGATTAATTATCCA

CN 119294 GTTTTTTATAATGATTTCTCTCCTTTTTATGGATTTTCTTACTTGAAGATTAATTATCCA

****** ***************************** ***********************

CN 120025 AAGGTTAATAGTTTCCTACCCTAGCTAGTTACCTCACATAGTTATGCTGC----ATATTT

CN 120013 AAGGTTAATAGTTTCCTACCCTAG-TAGTTACCTCACATAGCTGTGCTACATATATATTT

CS17CS1133 AAGGTTAATAGTTTCCTACCCTAG-TAGTTACCTCACATAGCTGTGCTACATATATATTT

CN 120030 AAGGTTAATAGTTTCCTACCCTAG-TAGTTACCTCACATAGCTGTGCTACATATATATTT

CO46 NCBI AAGGTTAATAGTTTCCTACCCTAG-TAGTTACCTCACATAGCTGTGCTACATATATATTT

DH55 ref genome AAGGTTAATAGTTTCCTACCCTAG-TAGTTACCTCACATAGCTGTGCTACATATATATTT

Hoga AAGGTTAATAGTTTCCTACCCTAG-TAGTTACCTCACATAGCTGTGCTACATATATATTT

CAM 236 AAGGTTAATAGTTTCCTACCCTAG-TAGTTACCTCACATAGCTGTGCTACATATATATTT

09-CS0040 AAGGTTAATAGTTTCCTACCCTAG-TAGTTACCTCACATAGCTGTGCTACATATATATTT

CN 119205 AAGGTTAATAGTTTCCTACCCTAG-TAGTTACCTCACATAGCTGTGCTACATATATATTT

Yellowstone AAGGTTAATAGTTTCCTACCCTAG-TAGTTACCTCACATAGCTGTGCTACATATATATTT

CN 120017 AAGGTTAATAGTTTCCTACCCTAG-TAGTTACCTCACATAGCTGTGCTACATATATATTT

CN 119300 AAGGTTAATAGTTTCCTACCCTAG-TAGTTACCTCACATAGCTGTGCTACATATATATTT

Jasper AAGGTTAATAGTTTCCTACCCTAG-TAGTTACCTCACATAGCTGTGCTACATATATATTT

CN 120027 AAGGTTAATAGTTTCCTACCCTAG-TAGTTACCTCACATAGCTGTGCTACATATATATTT

CN 113754 AAGGTTAATAGTTTCCTACCCTAG-TAGTTACCTCACATAGCTGTGCTACATATATATTT

Joelle AAFC AAGGTTAATAGTTTCCTACCCTAG-TAGTTACCTCACATAGCTGTGCTACATATATATTT

Joelle NCBI AAGGTTAATAGTTTCCTACCCTAG-TAGTTACCTCACATAGCTGTGCTACATATATATTT

Joelle phyto AAGGTTAATAGTTTCCTACCCTAG-TAGTTACCTCACATAGCTGTGCTACATATATATTT

Blaine Creek AAGGTTAATAGTTTCCTACCCTAG-TAGTTACCTCACATAGCTGTGCTACATATATATTT

CAM 241 AAGGTTAATAGTTTCCTACCCTAG-TAGTTACCTCACATAGCTGTGCTACATATATATTT

CN 119294 AAGGTTAATAGTTTCCTACCCTAG-TAGTTACCTCACATAGCTGTGCTACATATATATTT

************************ **************** * **** * ******

CN 120025 ATGTTATTTGTTTATCAGTTATCGCTCTTAATCTTTTAATGGATGTGTGCATAGATATAA

CN 120013 ATGTTA-TTGTTTATCAACTATCGCTCTTAATCTTTTAATGGATGTGTGCCTATATACAA

CS17CS1133 ATGTTA-TTGTTTATCAACTATCGCTCTTAATCTTTTAATGGATGTGTGCCTATATACAA

CN 120030 ATGTTA-TTGTTTATCAACTATCGCTCTTAATCTTTTAATGGATGTGTGCCTATATACAA

CO46 NCBI ATGTTA-TTGTTTATCAACTATCGCTCTTAATCTTTTAATGGATGTGTGCCTATATACAA

DH55 ref genome ATGTTA-TTGTTTATCAACTATCGCTCTTAATCTTTTAATGGATGTGTGCCTATATACAA

Hoga ATGTTA-TTGTTTATCAACTATCGCTCTTAATCTTTTAATGGATGTGTGCCTATATACAA

CAM 236 ATGTTA-TTGTTTATCAACTATCGCTCTTAATCTTTTAATGGATGTGTGCCTATATACAA

09-CS0040 ATGTTA-TTGTTTATCAACTATCGCTCTTAATCTTTTAATGGATGTGTGCCTATATACAA

CN 119205 ATGTTA-TTGTTTATCAACTATCGCTCTTAATCTTTTAATGGATGTGTGCCTATATACAA

Yellowstone ATGTTA-TTGTTTATCAACTATCGCTCTTAATCTTTTAATGGATGTGTGCCTATATACAA

CN 120017 ATGTTA-TTGTTTATCAACTATCGCTCTTAATCTTTTAATGGATGTGTGCCTATATACAA

CN 119300 ATGTTA-TTGTTTATCAACTATCGCTCTTAATCTTTTAATGGATGTGTGCCTATATACAA

Jasper ATGTTA-TTGTTTATCAACTATCGCTCTTAATCTTTTAATGGATGTGTGCCTATATACAA

CN 120027 ATGTTA-TTGTTTATCAACTATCGCTCTTAATCTTTTAATGGATGTGTGCCTATATACAA

CN 113754 ATGTTA-TTGTTTATCAACTATCGCTCTTAATCTTTTAATGGATGTGTGCCTATATACAA

Joelle AAFC ATGTTA-TTGTTTATCAACTATCGCTCTTAATCTTTTAATGGATGTGTGCCTATATACAA

Joelle NCBI ATGTTA-TTGTTTATCAACTATCGCTCTTAATCTTTTAATGGATGTGTGCCTATATACAA

Joelle phyto ATGTTA-TTGTTTATCAACTATCGCTCTTAATCTTTTAATGGATGTGTGCCTATATACAA

Blaine Creek ATGTTA-TTGTTTATCAACTATCGCTCTTAATCTTTTAATGGATGTGTGCCTATATACAA

CAM 241 ATGTTA-TTGTTTATCAACTATCGCTCTTAATCTTTTAATGGATGTGTGCCTATATACAA

CN 119294 ATGTTA-TTGTTTATCAACTATCGCTCTTAATCTTTTAATGGATGTGTGCCTATATACAA

****** ********** ******************************* ** *** **

CN 120025 GACTGTGTCCTCAATATGAATCCACAACCTTGTAAGTTTTGTTGTGGAAATTGACAAATC

CN 120013 GACTGTGTCCTCAACATGAATCCACAACCTTGTAATTTTTGTTGTGCAAATTGACAAATC

CS17CS1133 GACTGTGTCCTCAACATGAATCCACAACCTTGTAATTTTTGTTGTGCAAATTGACAAATC

CN 120030 GACTGTGTCCTCAACATGAATCCACAACCTTGTAATTTTTGTTGTGCAAATTGACAAATC

CO46 NCBI GACTGTGTCCTCAACATGAATCCACAACCTTGTAATTTTTGTTGTGCAAATTGACAAATC

DH55 ref genome GACTGTGTCCTCAACATGAATCCACAACCTTGTAATTTTTGTTGTGCAAATTGACAAATC

Hoga GACTGTGTCCTCAACATGAATCCACAACCTTGTAATTTTTGTTGTGCAAATTGACAAATC

CAM 236 GACTGTGTCCTCAACATGAATCCACAACCTTGTAATTTTTGTTGTGCAAATTGACAAATC

09-CS0040 GACTGTGTCCTCAACATGAATCCACAACCTTGTAATTTTTGTTGTGCAAATTGACAAATC

CN 119205 GACTGTGTCCTCAACATGAATCCACAACCTTGTAATTTTTGTTGTGCAAATTGACAAATC

Yellowstone GACTGTGTCCTCAACATGAATCCACAACCTTGTAATTTTTGTTGTGCAAATTGACAAATC

CN 120017 GACTGTGTCCTCAACATGAATCCACAACCTTGTAATTTTTGTTGTGCAAATTGACAAATC

CN 119300 GACTGTGTCCTCAACATGAATCCACAACCTTGTAATTTTTGTTGTGCAAATTGACAAATC

Jasper GACTGTGTCCTCAACATGAATCCACAACCTTGTAATTTTTGTTGTGCAAATTGACAAATC

CN 120027 GACTGTGTCCTCAACATGAATCCACAACCTTGTAATTTTTGTTGTGCAAATTGACAAATC

CN 113754 GACTGTGTCCTCAACATGAATCCACAACCTTGTAATTTTTGTTGTGCAAATTGACAAATC

Joelle AAFC GACTGTGTCCTCAACATGAATCCACAACCTTGTAATTTTTGTTGTGCAAATTGACAAATC

Joelle NCBI GACTGTGTCCTCAACATGAATCCACAACCTTGTAATTTTTGTTGTGCAAATTGACAAATC

Joelle phyto GACTGTGTCCTCAACATGAATCCACAACCTTGTAATTTTTGTTGTGCAAATTGACAAATC

Blaine Creek GACTGTGTCCTCAACATGAATCCACAACCTTGTAATTTTTGTTGTGCAAATTGACAAATC

CAM 241 GACTGTGTCCTCAACATGAATCCACAACCTTGTAATTTTTGTTGTGCAAATTGACAAATC

CN 119294 GACTGTGTCCTCAACATGAATCCACAACCTTGTAATTTTTGTTGTGCAAATTGACAAATC

************** ******************** ********** *************

CN 120025 ACACAACCTTTGTATCTTGGGTC-TTTTGTCATGAATATTGTCAATAACACAACCTTGTT

CN 120013 ACACAACCTTTGTATCTTGGGTCTTTTTGTCATGAATATTGCCAATAACTCAACCTTGTT

CS17CS1133 ACACAACCTTTGTATCTTGGGTCTTTTTGTCATGAATATTGCCAATAACTCAACCTTGTT

CN 120030 ACACAACCTTTGTATCTTGGGTCTTTTTGTCATGAATATTGCCAATAACTCAACCTTGTT

CO46 NCBI ACACAACCTTTGTATCTTGGGTCTTTTTGTCATGAATATTGCCAATAACTCAACCTTGTT

DH55 ref genome ACACAACCTTTGTATCTTGGGTCTTTTTGTCATGAATATTGCCAATAACTCAACCTTGTT

Hoga ACACAACCTTTGTATCTTGGGTCTTTTTGTCATGAATATTGCCAATAACTCAACCTTGTT

CAM 236 ACACAACCTTTGTATCTTGGGTCTTTTTGTCATGAATATTGCCAATAACTCAACCTTGTT

09-CS0040 ACACAACCTTTGTATCTTGGGTCTTTTTGTCATGAATATTGCCAATAACTCAACCTTGTT

CN 119205 ACACAACCTTTGTATCTTGGGTCTTTTTGTCATGAATATTGCCAATAACTCAACCTTGTT

Yellowstone ACACAACCTTTGTATCTTGGGTCTTTTTGTCATGAATATTGCCAATAACTCAACCTTGTT

CN 120017 ACACAACCTTTGTATCTTGGGTCTTTTTGTCATGAATATTGCCAATAACTCAACCTTGTT

CN 119300 ACACAACCTTTGTATCTTGGGTCTTTTTGTCATGAATATTGCCAATAACTCAACCTTGTT

Jasper ACACAACCTTTGTATCTTGGGTCTTTTTGTCATGAATATTGCCAATAACTCAACCTTGTT

CN 120027 ACACAACCTTTGTATCTTGGGTCTTTTTGTCATGAATATTGCCAATAACTCAACCTTGTT

CN 113754 ACACAACCTTTGTATCTTGGGTCTTTTTGTCATGAATATTGCCAATAACTCAACCTTGTT

Joelle AAFC ACACAACCTTTGTATCTTGGGTCTTTTTGTCATGAATATTGCCAATAACTCAACCTTGTT

Joelle NCBI ACACAACCTTTGTATCTTGGGTCTTTTTGTCATGAATATTGCCAATAACTCAACCTTGTT

Joelle phyto ACACAACCTTTGTATCTTGGGTCTTTTTGTCATGAATATTGCCAATAACTCAACCTTGTT

Blaine Creek ACACAACCTTTGTATCTTGGGTCTTTTTGTCATGAATATTGCCAATAACTCAACCTTGTT

CAM 241 ACACAACCTTTGTATCTTGGGTCTTTTTGTCATGAATATTGCCAATAACTCAACCTTGTT

CN 119294 ACACAACCTTTGTATCTTGGGTCTTTTTGTCATGAATATTGCCAATAACTCAACCTTGTT

*********************** ***************** ******* **********

CN 120025 TCTTTGTTGCCTCTAGGAAATGTAAAACTCCAGAAAGCTTGTCTTCATATAAGAAATATT

CN 120013 TCTTTGTTGCCTCTAGGAAATGTAAAACTCCAGAAAACTTGTCTTCATATAAGAAATATC

CS17CS1133 TCTTTGTTGCCTCTAGGAAATGTAAAACTCCAGAAAACTTGTCTTCATATAAGAAATATC

CN 120030 TCTTTGTTGCCTCTAGGAAATGTAAAACTCCAGAAAACTTGTCTTCATATAAGAAATATC

CO46 NCBI TCTTTGTTGCCTCTAGGAAATGTAAAACTCCAGAAAACTTGTCTTCATATAAGAAATATC

DH55 ref genome TCTTTGTTGCCTCTAGGAAATGTAAAACTCCAGAAAACTTGTCTTCATATAAGAAATATC

Hoga TCTTTGTTGCCTCTAGGAAATGTAAAACTCCAGAAAACTTGTCTTCATATAAGAAATATC

CAM 236 TCTTTGTTGCCTCTAGGAAATGTAAAACTCCAGAAAACTTGTCTTCATATAAGAAATATC

09-CS0040 TCTTTGTTGCCTCTAGGAAATGTAAAACTCCAGAAAACTTGTCTTCATATAAGAAATATC

CN 119205 TCTTTGTTGCCTCTAGGAAATGTAAAACTCCAGAAAACTTGTCTTCATATAAGAAATATC

Yellowstone TCTTTGTTGCCTCTAGGAAATGTAAAACTCCAGAAAACTTGTCTTCATATAAGAAATATC

CN 120017 TCTTTGTTGCCTCTAGGAAATGTAAAACTCCAGAAAACTTGTCTTCATATAAGAAATATC

CN 119300 TCTTTGTTGCCTCTAGGAAATGTAAAACTCCAGAAAACTTGTCTTCATATAAGAAATATC

Jasper TCTTTGTTGCCTCTAGGAAATGTAAAACTCCAGAAAACTTGTCTTCATATAAGAAATATC

CN 120027 TCTTTGTTGCCTCTAGGAAATGTAAAACTCCAGAAAACTTGTCTTCATATAAGAAATATC

CN 113754 TCTTTGTTGCCTCTAGGAAATGTAAAACTCCAGAAAACTTGTCTTCATATAAGAAATATC

Joelle AAFC TCTTTGTTGCCTCTAGGAAATGTAAAACTCCAGAAAACTTGTCTTCATATAAGAAATATC

Joelle NCBI TCTTTGTTGCCTCTAGGAAATGTAAAACTCCAGAAAACTTGTCTTCATATAAGAAATATC

Joelle phyto TCTTTGTTGCCTCTAGGAAATGTAAAACTCCAGAAAACTTGTCTTCATATAAGAAATATC

Blaine Creek TCTTTGTTGCCTCTAGGAAATGTAAAACTCCAGAAAACTTGTCTTCATATAAGAAATATC

CAM 241 TCTTTGTTGCCTCTAGGAAATGTAAAACTCCAGAAAACTTGTCTTCATATAAGAAATATC

CN 119294 TCTTTGTTGCCTCTAGGAAATGTAAAACTCCAGAAAACTTGTCTTCATATAAGAAATATC

************************************ **********************

CN 120025 AATATGATTTGTACATTGTCAAAACTAAACCCGGTGTAGTGTTTACTACAACCCTCCAAT

CN 120013 AATATGATTC-TACATTGTCAAAATTAAACCCGGTGTAGTGTTTACTACAACCCTCCTAT

CS17CS1133 AATATGATTC-TACATTGTCAAAATTAAACCCGGTGTAGTGTTTACTACAACCCTCCTAT

CN 120030 AATATGATTC-TACATTGTCAAAATTAAACCCGGTGTAGTGTTTACTACAACCCTCCTAT

CO46 NCBI AATATGATTC-TACATTGTCAAAATTAAACCCGGTGTAGTGTTTACTACAACCCTCCTAT

DH55 ref genome AATATGATTC-TACATTGTCAAAATTAAACCCGGTGTAGTGTTTACTACAACCCTCCTAT

Hoga AATATGATTC-TACATTGTCAAAATTAAACCCGGTGTAGTGTTTACTACAACCCTCCTAT

CAM 236 AATATGATTC-TACATTGTCAAAATTAAACCCGGTGTAGTGTTTACTACAACCCTCCTAT

09-CS0040 AATATGATTC-TACATTGTCAAAATTAAACCCGGTGTAGTGTTTACTACAACCCTCCTAT

CN 119205 AATATGATTC-TACATTGTCAAAATTAAACCCGGTGTAGTGTTTACTACAACCCTCCTAT

Yellowstone AATATGATTC-TACATTGTCAAAATTAAACCCGGTGTAGTGTTTACTACAACCCTCCTAT

CN 120017 AATATGATTC-TACATTGTCAAAATTAAACCCGGTGTAGTGTTTACTACAACCCTCCTAT

CN 119300 AATATGATTC-TACATTGTCAAAATTAAACCCGGTGTAGTGTTTACTACAACCCTCCTAT

Jasper AATATGATTC-TACATTGTCAAAATTAAACCCGGTGTAGTGTTTACTACAACCCTCCTAT

CN 120027 AATATGATTC-TACATTGTCAAAATTAAACCCGGTGTAGTGTTTACTACAACCCTCCTAT

CN 113754 AATATGATTC-TACATTGTCAAAATTAAACCCGGTGTAGTGTTTACTACAACCCTCCTAT

Joelle AAFC AATATGATTC-TACATTGTCAAAATTAAACCCGGTGTAGTGTTTACTACAACCCTCCTAT

Joelle NCBI AATATGATTC-TACATTGTCAAAATTAAACCCGGTGTAGTGTTTACTACAACCCTCCTAT

Joelle phyto AATATGATTC-TACATTGTCAAAATTAAACCCGGTGTAGTGTTTACTACAACCCTCCTAT

Blaine Creek AATATGATTC-TACATTGTCAAAATTAAACCCGGTGTAGTGTTTACTACAACCCTCCTAT

CAM 241 AATATGATTC-TACATTGTCAAAATTAAACCCGGTGTAGTGTTTACTACAACCCTCCTAT

CN 119294 AATATGATTC-TACATTGTCAAAATTAAACCCGGTGTAGTGTTTACTACAACCCTCCTAT

********* ************* ******************************** **

CN 120025 ATATTAACTTAGTGGTTGTAGTGGTTTGGCCATGTTGGTAAAGATTGTAGGCCGATTCTC

CN 120013 ATACTAACCAAGTGGTTGTAGTGGTTTGGCCATGTTGGTCAAGATGATATGCGGATTCTC

CS17CS1133 ATACTAACCAAGTGGTTGTAGTGGTTTGGCCATGTTGGTCAAGATGATATGCGGATTCTC

CN 120030 ATACTAACCAAGTGGTTGTAGTGGTTTGGCCATGTTGGTCAAGATGATATGCGGATTCTC

CO46 NCBI ATACTAACCAAGTGGTTGTAGTGGTTTGGCCATGTTGGTCAAGATGATATGCGGATTCTC

DH55 ref genome ATACTAACCAAGTGGTTGTAGTGGTTTGGCCATGTTGGTCAAGATGATATGCGGATTCTC

Hoga ATACTAACCAAGTGGTTGTAGTGGTTTGGCCATGTTGGTCAAGATGATATGCGGATTCTC

CAM 236 ATACTAACCAAGTGGTTGTAGTGGTTTGGCCATGTTGGTCAAGATGATATGCGGATTCTC

09-CS0040 ATACTAACCAAGTGGTTGTAGTGGTTTGGCCATGTTGGTCAAGATGATATGCGGATTCTC

CN 119205 ATACTAACCAAGTGGTTGTAGTGGTTTGGCCATGTTGGTCAAGATGATATGCGGATTCTC

Yellowstone ATACTAACCAAGTGGTTGTAGTGGTTTGGCCATGTTGGTCAAGATGATATGCGGATTCTC

CN 120017 ATACTAACCAAGTGGTTGTAGTGGTTTGGCCATGTTGGTCAAGATGATATGCGGATTCTC

CN 119300 ATACTAACCAAGTGGTTGTAGTGGTTTGGCCATGTTGGTCAAGATGATATGCGGATTCTC

Jasper ATACTAACCAAGTGGTTGTAGTGGTTTGGCCATGTTGGTCAAGATGATATGCGGATTCTC

CN 120027 ATACTAACCAAGTGGTTGTAGTGGTTTGGCCATGTTGGTCAAGATGATATGCGGATTCTC

CN 113754 ATACTAACCAAGTGGTTGTAGTGGTTTGGCCATGTTGGTCAAGATGATATGCGGATTCTC

Joelle AAFC ATACTAACCAAGTGGTTGTAGTGGTTTGGCCATGTTGGTCAAGATGATATGCGGATTCTC

Joelle NCBI ATACTAACCAAGTGGTTGTAGTGGTTTGGCCATGTTGGTCAAGATGATATGCGGATTCTC

Joelle phyto ATACTAACCAAGTGGTTGTAGTGGTTTGGCCATGTTGGTCAAGATGATATGCGGATTCTC

Blaine Creek ATACTAACCAAGTGGTTGTAGTGGTTTGGCCATGTTGGTCAAGATGATATGCGGATTCTC

CAM 241 ATACTAACCAAGTGGTTGTAGTGGTTTGGCCATGTTGGTCAAGATGATATGCGGATTCTC

CN 119294 ATACTAACCAAGTGGTTGTAGTGGTTTGGCCATGTTGGTCAAGATGATATGCGGATTCTC

*** **** ***************************** ***** ** ** *******

CN 120025 TCACTTGATGCATACTTTGTTAGGGTTTGTTCACGCCTTATACTGAATGTTAGGTCCAGC

CN 120013 TCACTTGCTGCATACTTTGTTAGGGTTTGTTCACGCCTTATACTGAATGTTAGGTCCAGC

CS17CS1133 TCACTTGCTGCATACTTTGTTAGGGTTTGTTCACGCCTTATACTGAATGTTAGGTCCAGC

CN 120030 TCACTTGCTGCATACTTTGTTAGGGTTTGTTCACGCCTTATACTGAATGTTAGGTCCAGC

CO46 NCBI TCACTTGCTGCATACTTTGTTAGGGTTTGTTCACGCCTTATACTGAATGTTAGGTCCAGC

DH55 ref genome TCACTTGCTGCATACTTTGTTAGGGTTTGTTCACGCCTTATACTGAATGTTAGGTCCAGC

Hoga TCACTTGCTGCATACTTTGTTAGGGTTTGTTCACGCCTTATACTGAATGTTAGGTCCAGC

CAM 236 TCACTTGCTGCATACTTTGTTAGGGTTTGTTCACGCCTTATACTGAATGTTAGGTCCAGC

09-CS0040 TCACTTGCTGCATACTTTGTTAGGGTTTGTTCACGCCTTATACTGAATGTTAGGTCCAGC

CN 119205 TCACTTGCTGCATACTTTGTTAGGGTTTGTTCACGCCTTATACTGAATGTTAGGTCCAGC

Yellowstone TCACTTGCTGCATACTTTGTTAGGGTTTGTTCACGCCTTATACTGAATGTTAGGTCCAGC

CN 120017 TCACTTGCTGCATACTTTGTTAGGGTTTGTTCACGCCTTATACTGAATGTTAGGTCCAGC

CN 119300 TCACTTGCTGCATACTTTGTTAGGGTTTGTTCACGCCTTATACTGAATGTTAGGTCCAGC

Jasper TCACTTGCTGCATACTTTGTTAGGGTTTGTTCACGCCTTATACTGAATGTTAGGTCCAGC

CN 120027 TCACTTGCTGCATACTTTGTTAGGGTTTGTTCACGCCTTATACTGAATGTTAGGTCCAGC

CN 113754 TCACTTGCTGCATACTTTGTTAGGGTTTGTTCACGCCTTATACTGAATGTTAGGTCCAGC

Joelle AAFC TCACTTGCTGCATACTTTGTTAGGGTTTGTTCACGCCTTATACTGAATGTTAGGTCCAGC

Joelle NCBI TCACTTGCTGCATACTTTGTTAGGGTTTGTTCACGCCTTATACTGAATGTTAGGTCCAGC

Joelle phyto TCACTTGCTGCATACTTTGTTAGGGTTTGTTCACGCCTTATACTGAATGTTAGGTCCAGC

Blaine Creek TCACTTGCTGCATACTTTGTTAGGGTTTGTTCACGCCTTATACTGAATGTTAGGTCCAGC

CAM 241 TCACTTGCTGCATACTTTGTTAGGGTTTGTTCACGCCTTATACTGAATGTTAGGTCCAGC

CN 119294 TCACTTGCTGCATACTTTGTTAGGGTTTGTTCACGCCTTATACTGAATGTTAGGTCCAGC

******* ****************************************************

CN 120025 CTTGGAATAGCCGAGACACCTGACTCAGTAATTATGAGTTAAAAAGAAACGTTATTCACT

CN 120013 CTTGGAATAGCCGAGACACCTGGCTCACTAATTATGAGTTAAAAAGAAACATTATTCACT

CS17CS1133 CTTGGAATAGCCGAGACACCTGGCTCACTAATTATGAGTTAAAAAGAAACATTATTCACT

CN 120030 CTTGGAATAGCCGAGACACCTGGCTCACTAATTATGAGTTAAAAAGAAACATTATTCACT

CO46 NCBI CTTGGAATAGCCGAGACACCTGGCTCACTAATTATGAGTTAAAAAGAAACATTATTCACT

DH55 ref genome CTTGGAATAGCCGAGACACCTGGCTCACTAATTATGAGTTAAAAAGAAACATTATTCACT

Hoga CTTGGAATAGCCGAGACACCTGGCTCACTAATTATGAGTTAAAAAGAAACATTATTCACT

CAM 236 CTTGGAATAGCCGAGACACCTGGCTCACTAATTATGAGTTAAAAAGAAACATTATTCACT

09-CS0040 CTTGGAATAGCCGAGACACCTGGCTCACTAATTATGAGTTAAAAAGAAACATTATTCACT

CN 119205 CTTGGAATAGCCGAGACACCTGGCTCACTAATTATGAGTTAAAAAGAAACATTATTCACT

Yellowstone CTTGGAATAGCCGAGACACCTGGCTCACTAATTATGAGTTAAAAAGAAACATTATTCACT

CN 120017 CTTGGAATAGCCGAGACACCTGGCTCACTAATTATGAGTTAAAAAGAAACATTATTCACT

CN 119300 CTTGGAATAGCCGAGACACCTGGCTCACTAATTATGAGTTAAAAAGAAACATTATTCACT

Jasper CTTGGAATAGCCGAGACACCTGGCTCACTAATTATGAGTTAAAAAGAAACATTATTCACT

CN 120027 CTTGGAATAGCCGAGACACCTGGCTCACTAATTATGAGTTAAAAAGAAACATTATTCACT

CN 113754 CTTGGAATAGCCGAGACACCTGGCTCACTAATTATGAGTTAAAAAGAAACATTATTCACT

Joelle AAFC CTTGGAATAGCCGAGACACCTGGCTCACTAATTATGAGTTAAAAAGAAACATTATTCACT

Joelle NCBI CTTGGAATAGCCGAGACACCTGGCTCACTAATTATGAGTTAAAAAGAAACATTATTCACT

Joelle phyto CTTGGAATAGCCGAGACACCTGGCTCACTAATTATGAGTTAAAAAGAAACATTATTCACT

Blaine Creek CTTGGAATAGCCGAGACACCTGGCTCACTAATTATGAGTTAAAAAGAAACATTATTCACT

CAM 241 CTTGGAATAGCCGAGACACCTGGCTCACTAATTATGAGTTAAAAAGAAACATTATTCACT

CN 119294 CTTGGAATAGCCGAGACACCTGGCTCACTAATTATGAGTTAAAAAGAAACATTATTCACT

********************** **** ********************** *********

CN 120025 CAATAACTCATTTTTGCATCCTTAATTTTGGTGCAAAGAGCTTAACTTCACAATGGAACT

CN 120013 CAATAACTCATTTTTGCATCCTTAATTTTGGTGCAAAGAGCTTAACTTCACAATGGAACT

CS17CS1133 CAATAACTCATTTTTGCATCCTTAATTTTGGTGCAAAGAGCTTAACTTCACAATGGAACT

CN 120030 CAATAACTCATTTTTGCATCCTTAATTTTGGTGCAAAGAGCTTAACTTCACAATGGAACT

CO46 NCBI CAATAACTCATTTTTGCATCCTTAATTTTGGTGCAAAGAGCTTAACTTCACAATGGAACT

DH55 ref genome CAATAACTCATTTTTGCATCCTTAATTTTGGTGCAAAGAGCTTAACTTCACAATGGAACT

Hoga CAATAACTCATTTTTGCATCCTTAATTTTGGTGCAAAGAGCTTAACTTCACAATGGAACT

CAM 236 CAATAACTCATTTTTGCATCCTTAATTTTGGTGCAAAGAGCTTAACTTCACAATGGAACT

09-CS0040 CAATAACTCATTTTTGCATCCTTAATTTTGGTGCAAAGAGCTTAACTTCACAATGGAACT

CN 119205 CAATAACTCATTTTTGCATCCTTAATTTTGGTGCAAAGAGCTTAACTTCACAATGGAACT

Yellowstone CAATAACTCATTTTTGCATCCTTAATTTTGGTGCAAAGAGCTTAACTTCACAATGGAACT

CN 120017 CAATAACTCATTTTTGCATCCTTAATTTTGGTGCAAAGAGCTTAACTTCACAATGGAACT

CN 119300 CAATAACTCATTTTTGCATCCTTAATTTTGGTGCAAAGAGCTTAACTTCACAATGGAACT

Jasper CAATAACTCATTTTTGCATCCTTAATTTTGGTGCAAAGAGCTTAACTTCACAATGGAACT

CN 120027 CAATAACTCATTTTTGCATCCTTAATTTTGGTGCAAAGAGCTTAACTTCACAATGGAACT

CN 113754 CAATAACTCATTTTTGCATCCTTAATTTTGGTGCAAAGAGCTTAACTTCACAATGGAACT

Joelle AAFC CAATAACTCATTTTTGCATCCTTAATTTTGGTGCAAAGAGCTTAACTTCACAATGGAACT

Joelle NCBI CAATAACTCATTTTTGCATCCTTAATTTTGGTGCAAAGAGCTTAACTTCACAATGGAACT

Joelle phyto CAATAACTCATTTTTGCATCCTTAATTTTGGTGCAAAGAGCTTAACTTCACAATGGAACT

Blaine Creek CAATAACTCATTTTTGCATCCTTAATTTTGGTGCAAAGAGCTTAACTTCACAATGGAACT

CAM 241 CAATAACTCATTTTTGCATCCTTAATTTTGGTGCAAAGAGCTTAACTTCACAATGGAACT

CN 119294 CAATAACTCATTTTTGCATCCTTAATTTTGGTGCAAAGAGCTTAACTTCACAATGGAACT

************************************************************

CN 120025 GAAACCTATTCGCACAAATTATTAAGTGACTTAGCGGTAGTTTTGTCAAATTTGCTTTGA

CN 120013 GAAACCTATTCGCACAAATTATTAAGTGACTTAGCGGTAGTTTTATCAAATTTGCTTTGA

CS17CS1133 GAAACCTATTCGCACAAATTATTAAGTGACTTAGCGGTAGTTTTATCAAATTTGCTTTGA

CN 120030 GAAACCTATTCGCACAAATTATTAAGTGACTTAGCGGTAGTTTTATCAAATTTGCTTTGA

CO46 NCBI GAAACCTATTCGCACAAATTATTAAGTGACTTAGCGGTAGTTTTATCAAATTTGCTTTGA

DH55 ref genome GAAACCTATTCGCACAAATTATTAAGTGACTTAGCGGTAGTTTTATCAAATTTGCTTTGA

Hoga GAAACCTATTCGCACAAATTATTAAGTGACTTAGCGGTAGTTTTATCAAATTTGCTTTGA

CAM 236 GAAACCTATTCGCACAAATTATTAAGTGACTTAGCGGTAGTTTTATCAAATTTGCTTTGA

09-CS0040 GAAACCTATTCGCACAAATTATTAAGTGACTTAGCGGTAGTTTTATCAAATTTGCTTTGA

CN 119205 GAAACCTATTCGCACAAATTATTAAGTGACTTAGCGGTAGTTTTATCAAATTTGCTTTGA

Yellowstone GAAACCTATTCGCACAAATTATTAAGTGACTTAGCGGTAGTTTTATCAAATTTGCTTTGA

CN 120017 GAAACCTATTCGCACAAATTATTAAGTGACTTAGCGGTAGTTTTATCAAATTTGCTTTGA

CN 119300 GAAACCTATTCGCACAAATTATTAAGTGACTTAGCGGTAGTTTTATCAAATTTGCTTTGA

Jasper GAAACCTATTCGCACAAATTATTAAGTGACTTAGCGGTAGTTTTATCAAATTTGCTTTGA

CN 120027 GAAACCTATTCGCACAAATTATTAAGTGACTTAGCGGTAGTTTTATCAAATTTGCTTTGA

CN 113754 GAAACCTATTCGCACAAATTATTAAGTGACTTAGCGGTAGTTTTATCAAATTTGCTTTGA

Joelle AAFC GAAACCTATTCGCACAAATTATTAAGTGACTTAGCGGTAGTTTTATCAAATTTGCTTTGA

Joelle NCBI GAAACCTATTCGCACAAATTATTAAGTGACTTAGCGGTAGTTTTATCAAATTTGCTTTGA

Joelle phyto GAAACCTATTCGCACAAATTATTAAGTGACTTAGCGGTAGTTTTATCAAATTTGCTTTGA

Blaine Creek GAAACCTATTCGCACAAATTATTAAGTGACTTAGCGGTAGTTTTATCAAATTTGCTTTGA

CAM 241 GAAACCTATTCGCACAAATTATTAAGTGACTTAGCGGTAGTTTTATCAAATTTGCTTTGA

CN 119294 GAAACCTATTCGCACAAATTATTAAGTGACTTAGCGGTAGTTTTATCAAATTTGCTTTGA

******************************************** ***************

CN 120025 CCTCTATTAGGTAAATTATGTAGTTTTAGGTTTTTCCTTCTTAA-----------TTTGG

CN 120013 CCTCTATTAGGT-AATTATGTAGTTTTAGTTTTATCTGTCTTAGGTTTTTCCTTCTTTGG

CS17CS1133 CCTCTATTAGGT-AATTATGTAGTTTTAGTTTTATCTGTCTTAGGTTTTTCCTTCTTTGG

CN 120030 CCTCTATTAGGT-AATTATGTAGTTTTAGTTTTATCTGTCTTAGGTTTTTCCTTCTTTGG

CO46 NCBI CCTCTATTAGGT-AATTATGTAGTTTTAGTTTTATCTGTCTTAGGTTTTTCCTTCTTTGG

DH55 ref genome CCTCTATTAGGT-AATTATGTAGTTTTAGTTTTATCTGTCTTAGGTTTTTCCTTCTTTGG

Hoga CCTCTATTAGGT-AATTATGTAGTTTTAGTTTTATCTGTCTTAGGTTTTTCCTTCTTTGG

CAM 236 CCTCTATTAGGT-AATTATGTAGTTTTAGTTTTATCTGTCTTAGGTTTTTCCTTCTTTGG

09-CS0040 CCTCTATTAGGT-AATTATGTAGTTTTAGTTTTATCTGTCTTAGGTTTTTCCTTCTTTGG

CN 119205 CCTCTATTAGGT-AATTATGTAGTTTTAGTTTTATCTGTCTTAGGTTTTTCCTTCTTTGG

Yellowstone CCTCTATTAGGT-AATTATGTAGTTTTAGTTTTATCTGTCTTAGGTTTTTCCTTCTTTGG

CN 120017 CCTCTATTAGGT-AATTATGTAGTTTTAGTTTTATCTGTCTTAGGTTTTTCCTTCTTTGG

CN 119300 CCTCTATTAGGT-AATTATGTAGTTTTAGTTTTATCTGTCTTAGGTTTTTCCTTCTTTGG

Jasper CCTCTATTAGGT-AATTATGTAGTTTTAGTTTTATCTGTCTTAGGTTTTTCCTTCTTTGG

CN 120027 CCTCTATTAGGT-AATTATGTAGTTTTAGTTTTATCTGTCTTAGGTTTTTCCTTCTTTGG

CN 113754 CCTCTATTAGGT-AATTATGTAGTTTTAGTTTTATCTGTCTTAGGTTTTTCCTTCTTTGG

Joelle AAFC CCTCTATTAGGT-AATTATGTAGTTTTAGTTTTATCTGTCTTAGGTTTTTCCTTCTTTGG

Joelle NCBI CCTCTATTAGGT-AATTATGTAGTTTTAGTTTTATCTGTCTTAGGTTTTTCCTTCTTTGG

Joelle phyto CCTCTATTAGGT-AATTATGTAGTTTTAGTTTTATCTGTCTTAGGTTTTTCCTTCTTTGG

Blaine Creek CCTCTATTAGGT-AATTATGTAGTTTTAGTTTTATCTGTCTTAGGTTTTTCCTTCTTTGG

CAM 241 CCTCTATTAGGT-AATTATGTAGTTTTAGTTTTATCTGTCTTAGGTTTTTCCTTCTTTGG

CN 119294 CCTCTATTAGGT-AATTATGTAGTTTTAGTTTTATCTGTCTTAGGTTTTTCCTTCTTTGG

************ **************** *** ** ***** *****

CN 120025 AACAATTTATCTATATTGGTAATACTTGATAGAGGCCTCAGTCAGTTGATGTAATTTCAT

CN 120013 AACAATTTCTATATATTGGTGATACTTGATAGATGCCTCAGTAAGTTGATG---TCTCAG

CS17CS1133 AACAATTTCTATATATTGGTGATACTTGATAGATGCCTCAGTAAGTTGATG---TCTCAG

CN 120030 AACAATTTCTATATATTGGTGATACTTGATAGATGCCTCAGTAAGTTGATG---TCTCAG

CO46 NCBI AACAATTTCTATATATTGGTGATACTTGATAGATGCCTCAGTAAGTTGATG---TCTCAG

DH55 ref genome AACAATTTCTATATATTGGTGATACTTGATAGATGCCTCAGTAAGTTGATG---TCTCAG

Hoga AACAATTTCTATATATTGGTGATACTTGATAGATGCCTCAGTAAGTTGATG---TCTCAG

CAM 236 AACAATTTCTATATATTGGTGATACTTGATAGATGCCTCAGTAAGTTGATG---TCTCAG

09-CS0040 AACAATTTCTATATATTGGTGATACTTGATAGATGCCTCAGTAAGTTGATG---TCTCAG

CN 119205 AACAATTTCTATATATTGGTGATACTTGATAGATGCCTCAGTAAGTTGATG---TCTCAG

Yellowstone AACAATTTCTATATATTGGTGATACTTGATAGATGCCTCAGTAAGTTGATG---TCTCAG

CN 120017 AACAATTTCTATATATTGGTGATACTTGATAGATGCCTCAGTAAGTTGATG---TCTCAG

CN 119300 AACAATTTCTATATATTGGTGATACTTGATAGATGCCTCAGTAAGTTGATG---TCTCAG

Jasper AACAATTTCTATATATTGGTGATACTTGATAGATGCCTCAGTAAGTTGATG---TCTCAG

CN 120027 AACAATTTCTATATATTGGTGATACTTGATAGATGCCTCAGTAAGTTGATG---TCTCAG

CN 113754 AACAATTTCTATATATTGGTGATACTTGATAGATGCCTCAGTAAGTTGATG---TCTCAG

Joelle AAFC AACAATTTCTATATATTGGTGATACTTGATAGATGCCTCAGTAAGTTGATG---TCTCAG

Joelle NCBI AACAATTTCTATATATTGGTGATACTTGATAGATGCCTCAGTAAGTTGATG---TCTCAG

Joelle phyto AACAATTTCTATATATTGGTGATACTTGATAGATGCCTCAGTAAGTTGATG---TCTCAG

Blaine Creek AACAATTTCTATATATTGGTGATACTTGATAGATGCCTCAGTAAGTTGATG---TCTCAG

CAM 241 AACAATTTCTATATATTGGTGATACTTGATAGATGCCTCAGTAAGTTGATG---TCTCAG

CN 119294 AACAATTTCTATATATTGGTGATACTTGATAGATGCCTCAGTAAGTTGATG---TCTCAG

******** * ********* ************ ******** ******** * ***

CN 120025 TGGCAGAAAACTCTTGACCTTTACTGCTTGATTTTTGGCTTTCAATATAGTTAATTGGAA

CN 120013 T-------AACTCTTCACCTTTACTGCTTGATTTTTTGCTTTCAATATAGTTAATTGGAA

CS17CS1133 T-------AACTCTTCACCTTTACTGCTTGATTTTTTGCTTTCAATATAGTTAATTGGAA

CN 120030 T-------AACTCTTCACCTTTACTGCTTGATTTTTTGCTTTCAATATAGTTAATTGGAA

CO46 NCBI T-------AACTCTTCACCTTTACTGCTTGATTTTTTGCTTTCAATATAGTTAATTGGAA

DH55 ref genome T-------AACTCTTCACCTTTACTGCTTGATTTTTTGCTTTCAATATAGTTAATTGGAA

Hoga T-------AACTCTTCACCTTTACTGCTTGATTTTTTGCTTTCAATATAGTTAATTGGAA

CAM 236 T-------AACTCTTCACCTTTACTGCTTGATTTTTTGCTTTCAATATAGTTAATTGGAA

09-CS0040 T-------AACTCTTCACCTTTACTGCTTGATTTTTTGCTTTCAATATAGTTAATTGGAA

CN 119205 T-------AACTCTTCACCTTTACTGCTTGATTTTTTGCTTTCAATATAGTTAATTGGAA

Yellowstone T-------AACTCTTCACCTTTACTGCTTGATTTTTTGCTTTCAATATAGTTAATTGGAA

CN 120017 T-------AACTCTTCACCTTTACTGCTTGATTTTTTGCTTTCAATATAGTTAATTGGAA

CN 119300 T-------AACTCTTCACCTTTACTGCTTGATTTTTTGCTTTCAATATAGTTAATTGGAA

Jasper T-------AACTCTTCACCTTTACTGCTTGATTTTTTGCTTTCAATATAGTTAATTGGAA

CN 120027 T-------AACTCTTCACCTTTACTGCTTGATTTTTTGCTTTCAATATAGTTAATTGGAA

CN 113754 T-------AACTCTTCACCTTTACTGCTTGATTTTTTGCTTTCAATATAGTTAATTGGAA

Joelle AAFC T-------AACTCTTCACCTTTACTGCTTGATTTTTTGCTTTCAATATAGTTAATTGGAA

Joelle NCBI T-------AACTCTTCACCTTTACTGCTTGATTTTTTGCTTTCAATATAGTTAATTGGAA

Joelle phyto T-------AACTCTTCACCTTTACTGCTTGATTTTTTGCTTTCAATATAGTTAATTGGAA

Blaine Creek T-------AACTCTTCACCTTTACTGCTTGATTTTTTGCTTTCAATATAGTTAATTGGAA

CAM 241 T-------AACTCTTCACCTTTACTGCTTGATTTTTTGCTTTCAATATAGTTAATTGGAA

CN 119294 T-------AACTCTTCACCTTTACTGCTTGATTTTTTGCTTTCAATATAGTTAATTGGAA

* ******* ******************** ***********************

CN 120025 CCTC-ACAGTTTCTATACAAACGAGGAAGAAAATGGAAG--------------------A

CN 120013 CCTCAACAGTTTCTATACAAACGAGGAAGAAAATGGAAGATGGAAATACAAAATGGAAAA

CS17CS1133 CCTCAACAGTTTCTATACAAACGAGGAAGAAAATGGAAGATGGAAATACAAAATGGAAAA

CN 120030 CCTCAACAGTTTCTATACAAACGAGGAAGAAAATGGAAGATGGAAATACAAAATGGAAAA

CO46 NCBI CCTCAACAGTTTCTATACAAACGAGGAAGAAAATGGAAGATGGAAATACAAAATGGAAAA

DH55 ref genome CCTCAACAGTTTCTATACAAACGAGGAAGAAAATGGAAGATGGAAATACAAAATGGAAAA

Hoga CCTCAACAGTTTCTATACAAACGAGGAAGAAAATGGAAGATGGAAATACAAAATGGAAAA

CAM 236 CCTCAACAGTTTCTATACAAACGAGGAAGAAAATGGAAGATGGAAATACAAAATGGAAAA

09-CS0040 CCTCAACAGTTTCTATACAAACGAGGAAGAAAATGGAAGATGGAAATACAAAATGGAAAA

CN 119205 CCTCAACAGTTTCTATACAAACGAGGAAGAAAATGGAAGATGGAAATACAAAATGGAAAA

Yellowstone CCTCAACAGTTTCTATACAAACGAGGAAGAAAATGGAAGATGGAAATACAAAATGGAAAA

CN 120017 CCTCAACAGTTTCTATACAAACGAGGAAGAAAATGGAAGATGGAAATACAAAATGGAAAA

CN 119300 CCTCAACAGTTTCTATACAAACGAGGAAGAAAATGGAAGATGGAAATACAAAATGGAAAA

Jasper CCTCAACAGTTTCTATACAAACGAGGAAGAAAATGGAAGATGGAAATACAAAATGGAAAA

CN 120027 CCTCAACAGTTTCTATACAAACGAGGAAGAAAATGGAAGATGGAAATACAAAATGGAAAA

CN 113754 CCTCAACAGTTTCTATACAAACGAGGAAGAAAATGGAAGATGGAAATACAAAATGGAAAA

Joelle AAFC CCTCAACAGTTTCTATACAAACGAGGAAGAAAATGGAAGATGGAAATACAAAATGGAAAA

Joelle NCBI CCTCAACAGTTTCTATACAAACGAGGAAGAAAATGGAAGATGGAAATACAAAATGGAAAA

Joelle phyto CCTCAACAGTTTCTATACAAACGAGGAAGAAAATGGAAGATGGAAATACAAAATGGAAAA

Blaine Creek CCTCAACAGTTTCTATACAAACGAGGAAGAAAATGGAAGATGGAAATACAAAATGGAAAA

CAM 241 CCTCAACAGTTTCTATACAAACGAGGAAGAAAATGGAAGATGGAAATACAAAATGGAAAA

CN 119294 CCTCAACAGTTTCTATACAAACGAGGAAGAAAATGGAAGATGGAAATACAAAATGGAAAA

**** ********************************** *

CN 120025 CCGGTTTCCTAGTCTTAGGAAAGTGTTTAGATTTTCGTTTCAAAGAAACATAAAATAAAA

CN 120013 CCGGTTTCCTATTCTTAGGAAACTGTTTAGATTTTCGTTGCAAAGAAACATAAAATAAAA

CS17CS1133 CCGGTTTCCTATTCTTAGGAAACTGTTTAGATTTTCGTTGCAAAGAAACATAAAATAAAA

CN 120030 CCGGTTTCCTATTCTTAGGAAACTGTTTAGATTTTCGTTGCAAAGAAACATAAAATAAAA

CO46 NCBI CCGGTTTCCTATTCTTAGGAAACTGTTTAGATTTTCGTTGCAAAGAAACATAAAATAAAA

DH55 ref genome CCGGTTTCCTATTCTTAGGAAACTGTTTAGATTTTCGTTGCAAAGAAACATAAAATAAAA

Hoga CCGGTTTCCTATTCTTAGGAAACTGTTTAGATTTTCGTTGCAAAGAAACATAAAATAAAA

CAM 236 CCGGTTTCCTATTCTTAGGAAACTGTTTAGATTTTCGTTGCAAAGAAACATAAAATAAAA

09-CS0040 CCGGTTTCCTATTCTTAGGAAACTGTTTAGATTTTCGTTGCAAAGAAACATAAAATAAAA

CN 119205 CCGGTTTCCTATTCTTAGGAAACTGTTTAGATTTTCGTTGCAAAGAAACATAAAATAAAA

Yellowstone CCGGTTTCCTATTCTTAGGAAACTGTTTAGATTTTCGTTGCAAAGAAACATAAAATAAAA

CN 120017 CCGGTTTCCTATTCTTAGGAAACTGTTTAGATTTTCGTTGCAAAGAAACATAAAATAAAA

CN 119300 CCGGTTTCCTATTCTTAGGAAACTGTTTAGATTTTCGTTGCAAAGAAACATAAAATAAAA

Jasper CCGGTTTCCTATTCTTAGGAAACTGTTTAGATTTTCGTTGCAAAGAAACATAAAATAAAA

CN 120027 CCGGTTTCCTATTCTTAGGAAACTGTTTAGATTTTCGTTGCAAAGAAACATAAAATAAAA

CN 113754 CCGGTTTCCTATTCTTAGGAAACTGTTTAGATTTTCGTTGCAAAGAAACATAAAATAAAA

Joelle AAFC CCGGTTTCCTATTCTTAGGAAACTGTTTAGATTTTCGTTGCAAAGAAACATAAAATAAAA

Joelle NCBI CCGGTTTCCTATTCTTAGGAAACTGTTTAGATTTTCGTTGCAAAGAAACATAAAATAAAA

Joelle phyto CCGGTTTCCTATTCTTAGGAAACTGTTTAGATTTTCGTTGCAAAGAAACATAAAATAAAA

Blaine Creek CCGGTTTCCTATTCTTAGGAAACTGTTTAGATTTTCGTTGCAAAGAAACATAAAATAAAA

CAM 241 CCGGTTTCCTATTCTTAGGAAACTGTTTAGATTTTCGTTGCAAAGAAACATAAAATAAAA

CN 119294 CCGGTTTCCTATTCTTAGGAAACTGTTTAGATTTTCGTTGCAAAGAAACATAAAATAAAA

*********** ********** **************** ********************

CN 120025 TTATGAGATTGTTGTCTAAAGACTCGGTCAATGTATTTGGAGTTTGGATTTACGTATTGG

CN 120013 TTATGAGATTGTTGTCTAAAAACTCGGTCAATGTATTTGGAGTTTGGATTTACGTATTGG

CS17CS1133 TTATGAGATTGTTGTCTAAAAACTCGGTCAATGTATTTGGAGTTTGGATTTACGTATTGG

CN 120030 TTATGAGATTGTTGTCTAAAAACTCGGTCAATGTATTTGGAGTTTGGATTTACGTATTGG

CO46 NCBI TTATGAGATTGTTGTCTAAAAACTCGGTCAATGTATTTGGAGTTTGGATTTACGTATTGG

DH55 ref genome TTATGAGATTGTTGTCTAAAAACTCGGTCAATGTATTTGGAGTTTGGATTTACGTATTGG

Hoga TTATGAGATTGTTGTCTAAAAACTCGGTCAATGTATTTGGAGTTTGGATTTACGTATTGG

CAM 236 TTATGAGATTGTTGTCTAAAAACTCGGTCAATGTATTTGGAGTTTGGATTTACGTATTGG

09-CS0040 TTATGAGATTGTTGTCTAAAAACTCGGTCAATGTATTTGGAGTTTGGATTTACGTATTGG

CN 119205 TTATGAGATTGTTGTCTAAAAACTCGGTCAATGTATTTGGAGTTTGGATTTACGTATTGG

Yellowstone TTATGAGATTGTTGTCTAAAAACTCGGTCAATGTATTTGGAGTTTGGATTTACGTATTGG

CN 120017 TTATGAGATTGTTGTCTAAAAACTCGGTCAATGTATTTGGAGTTTGGATTTACGTATTGG

CN 119300 TTATGAGATTGTTGTCTAAAAACTCGGTCAATGTATTTGGAGTTTGGATTTACGTATTGG

Jasper TTATGAGATTGTTGTCTAAAAACTCGGTCAATGTATTTGGAGTTTGGATTTACGTATTGG

CN 120027 TTATGAGATTGTTGTCTAAAAACTCGGTCAATGTATTTGGAGTTTGGATTTACGTATTGG

CN 113754 TTATGAGATTGTTGTCTAAAAACTCGGTCAATGTATTTGGAGTTTGGATTTACGTATTGG

Joelle AAFC TTATGAGATTGTTGTCTAAAAACTCGGTCAATGTATTTGGAGTTTGGATTTACGTATTGG

Joelle NCBI TTATGAGATTGTTGTCTAAAAACTCGGTCAATGTATTTGGAGTTTGGATTTACGTATTGG

Joelle phyto TTATGAGATTGTTGTCTAAAAACTCGGTCAATGTATTTGGAGTTTGGATTTACGTATTGG

Blaine Creek TTATGAGATTGTTGTCTAAAAACTCGGTCAATGTATTTGGAGTTTGGATTTACGTATTGG

CAM 241 TTATGAGATTGTTGTCTAAAAACTCGGTCAATGTATTTGGAGTTTGGATTTACGTATTGG

CN 119294 TTATGAGATTGTTGTCTAAAAACTCGGTCAATGTATTTGGAGTTTGGATTTACGTATTGG

******************** ***************************************

CN 120025 TCATCTGCTTACCGGCCACA-CATCATCATCATCATGTTATGGCTTATCAATACTCCATT

CN 120013 TCATCTGCTTACCGGCCACATCATCATCATCATCATGTTATGGCTTATCAATACTCCATT

CS17CS1133 TCATCTGCTTACCGGCCACATCATCATCATCATCATGTTATGGCTTATCAATACTCCATT

CN 120030 TCATCTGCTTACCGGCCACATCATCATCATCATCATGTTATGGCTTATCAATACTCCATT

CO46 NCBI TCATCTGCTTACCGGCCACATCATCATCATCATCATGTTATGGCTTATCAATACTCCATT

DH55 ref genome TCATCTGCTTACCGGCCACATCATCATCATCATCATGTTATGGCTTATCAATACTCCATT

Hoga TCATCTGCTTACCGGCCACATCATCATCATCATCATGTTATGGCTTATCAATACTCCATT

CAM 236 TCATCTGCTTACCGGCCACATCATCATCATCATCATGTTATGGCTTATCAATACTCCATT

09-CS0040 TCATCTGCTTACCGGCCACATCATCATCATCATCATGTTATGGCTTATCAATACTCCATT

CN 119205 TCATCTGCTTACCGGCCACATCATCATCATCATCATGTTATGGCTTATCAATACTCCATT

Yellowstone TCATCTGCTTACCGGCCACATCATCATCATCATCATGTTATGGCTTATCAATACTCCATT

CN 120017 TCATCTGCTTACCGGCCACATCATCATCATCATCATGTTATGGCTTATCAATACTCCATT

CN 119300 TCATCTGCTTACCGGCCACATCATCATCATCATCATGTTATGGCTTATCAATACTCCATT

Jasper TCATCTGCTTACCGGCCACATCATCATCATCATCATGTTATGGCTTATCAATACTCCATT

CN 120027 TCATCTGCTTACCGGCCACATCATCATCATCATCATGTTATGGCTTATCAATACTCCATT

CN 113754 TCATCTGCTTACCGGCCACATCATCATCATCATCATGTTATGGCTTATCAATACTCCATT

Joelle AAFC TCATCTGCTTACCGGCCACATCATCATCATCATCATGTTATGGCTTATCAATACTCCATT

Joelle NCBI TCATCTGCTTACCGGCCACATCATCATCATCATCATGTTATGGCTTATCAATACTCCATT

Joelle phyto TCATCTGCTTACCGGCCACATCATCATCATCATCATGTTATGGCTTATCAATACTCCATT

Blaine Creek TCATCTGCTTACCGGCCACATCATCATCATCATCATGTTATGGCTTATCAATACTCCATT

CAM 241 TCATCTGCTTACCGGCCACATCATCATCATCATCATGTTATGGCTTATCAATACTCCATT

CN 119294 TCATCTGCTTACCGGCCACATCATCATCATCATCATGTTATGGCTTATCAATACTCCATT

******************** ***************************************

CN 120025 ACCAGAAAGAACCTTGAGGTCAAGGTTCATATATATGAAAGCTCAGAAAAGTTGTCTTGT

CN 120013 ACCAGAAAGAACCTTGAGGTCAAGGTTC----ATATGAAAACTCAAAAAAGTTGTCTTGT

CS17CS1133 ACCAGAAAGAACCTTGAGGTCAAGGTTC----ATATGAAAACTCAAAAAAGTTGTCTTGT

CN 120030 ACCAGAAAGAACCTTGAGGTCAAGGTTC----ATATGAAAACTCAAAAAAGTTGTCTTGT

CO46 NCBI ACCAGAAAGAACCTTGAGGTCAAGGTTC----ATATGAAAACTCAAAAAAGTTGTCTTGT

DH55 ref genome ACCAGAAAGAACCTTGAGGTCAAGGTTC----ATATGAAAACTCAAAAAAGTTGTCTTGT

Hoga ACCAGAAAGAACCTTGAGGTCAAGGTTC----ATATGAAAACTCAAAAAAGTTGTCTTGT

CAM 236 ACCAGAAAGAACCTTGAGGTCAAGGTTC----ATATGAAAACTCAAAAAAGTTGTCTTGT

09-CS0040 ACCAGAAAGAACCTTGAGGTCAAGGTTC----ATATGAAAACTCAAAAAAGTTGTCTTGT

CN 119205 ACCAGAAAGAACCTTGAGGTCAAGGTTC----ATATGAAAACTCAAAAAAGTTGTCTTGT

Yellowstone ACCAGAAAGAACCTTGAGGTCAAGGTTC----ATATGAAAACTCAAAAAAGTTGTCTTGT

CN 120017 ACCAGAAAGAACCTTGAGGTCAAGGTTC----ATATGAAAACTCAAAAAAGTTGTCTTGT

CN 119300 ACCAGAAAGAACCTTGAGGTCAAGGTTC----ATATGAAAACTCAAAAAAGTTGTCTTGT

Jasper ACCAGAAAGAACCTTGAGGTCAAGGTTC----ATATGAAAACTCAAAAAAGTTGTCTTGT

CN 120027 ACCAGAAAGAACCTTGAGGTCAAGGTTC----ATATGAAAACTCAAAAAAGTTGTCTTGT

CN 113754 ACCAGAAAGAACCTTGAGGTCAAGGTTC----ATATGAAAACTCAAAAAAGTTGTCTTGT

Joelle AAFC ACCAGAAAGAACCTTGAGGTCAAGGTTC----ATATGAAAACTCAAAAAAGTTGTCTTGT

Joelle NCBI ACCAGAAAGAACCTTGAGGTCAAGGTTC----ATATGAAAACTCAAAAAAGTTGTCTTGT

Joelle phyto ACCAGAAAGAACCTTGAGGTCAAGGTTC----ATATGAAAACTCAAAAAAGTTGTCTTGT

Blaine Creek ACCAGAAAGAACCTTGAGGTCAAGGTTC----ATATGAAAACTCAAAAAAGTTGTCTTGT

CAM 241 ACCAGAAAGAACCTTGAGGTCAAGGTTC----ATATGAAAACTCAAAAAAGTTGTCTTGT

CN 119294 ACCAGAAAGAACCTTGAGGTCAAGGTTC----ATATGAAAACTCAAAAAAGTTGTCTTGT

**************************** ******** **** **************

CN 120025 ATATATGTTCAGATGGTGTTACTTAGAAAACCAATAGTTGGTGTCACCCTTTTTGACACA

CN 120013 ATATATGTTCAGATGGTAATACTTAGAAAACCAATAGTTGATGTCACCGTTTTTGACACA

CS17CS1133 ATATATGTTCAGATGGTAATACTTAGAAAACCAATAGTTGATGTCACCGTTTTTGACACA

CN 120030 ATATATGTTCAGATGGTAATACTTAGAAAACCAATAGTTGATGTCACCGTTTTTGACACA

CO46 NCBI ATATATGTTCAGATGGTAATACTTAGAAAACCAATAGTTGATGTCACCGTTTTTGACACA

DH55 ref genome ATATATGTTCAGATGGTAATACTTAGAAAACCAATAGTTGATGTCACCGTTTTTGACACA

Hoga ATATATGTTCAGATGGTAATACTTAGAAAACCAATAGTTGATGTCACCGTTTTTGACACA

CAM 236 ATATATGTTCAGATGGTAATACTTAGAAAACCAATAGTTGATGTCACCGTTTTTGACACA

09-CS0040 ATATATGTTCAGATGGTAATACTTAGAAAACCAATAGTTGATGTCACCGTTTTTGACACA

CN 119205 ATATATGTTCAGATGGTAATACTTAGAAAACCAATAGTTGATGTCACCGTTTTTGACACA

Yellowstone ATATATGTTCAGATGGTAATACTTAGAAAACCAATAGTTGATGTCACCGTTTTTGACACA

CN 120017 ATATATGTTCAGATGGTAATACTTAGAAAACCAATAGTTGATGTCACCGTTTTTGACACA

CN 119300 ATATATGTTCAGATGGTAATACTTAGAAAACCAATAGTTGATGTCACCGTTTTTGACACA

Jasper ATATATGTTCAGATGGTAATACTTAGAAAACCAATAGTTGATGTCACCGTTTTTGACACA

CN 120027 ATATATGTTCAGATGGTAATACTTAGAAAACCAATAGTTGATGTCACCGTTTTTGACACA

CN 113754 ATATATGTTCAGATGGTAATACTTAGAAAACCAATAGTTGATGTCACCGTTTTTGACACA

Joelle AAFC ATATATGTTCAGATGGTAATACTTAGAAAACCAATAGTTGATGTCACCGTTTTTGACACA

Joelle NCBI ATATATGTTCAGATGGTAATACTTAGAAAACCAATAGTTGATGTCACCGTTTTTGACACA

Joelle phyto ATATATGTTCAGATGGTAATACTTAGAAAACCAATAGTTGATGTCACCGTTTTTGACACA

Blaine Creek ATATATGTTCAGATGGTAATACTTAGAAAACCAATAGTTGATGTCACCGTTTTTGACACA

CAM 241 ATATATGTTCAGATGGTAATACTTAGAAAACCAATAGTTGATGTCACCGTTTTTGACACA

CN 119294 ATATATGTTCAGATGGTAATACTTAGAAAACCAATAGTTGATGTCACCGTTTTTGACACA

***************** ********************* ******* ***********

CN 120025 CGATTAGGCGTTTTCTTTTTAGTTATGCATAAGAACACTAAGAATTTTGATACCACTATT

CN 120013 CGATTAGGCGTTTTCTTTTTAGTTATGCATAAGAACACTAAAATTTTTG-----------

CS17CS1133 CGATTAGGCGTTTTCTTTTTAGTTATGCATAAGAACACTAAAATTTTTG-----------

CN 120030 CGATTAGGCGTTTTCTTTTTAGTTATGCATAAGAACACTAAAATTTTTG-----------

CO46 NCBI CGATTAGGCGTTTTCTTTTTAGTTATGCATAAGAACACTAAAATTTTTG-----------

DH55 ref genome CGATTAGGCGTTTTCTTTTTAGTTATGCATAAGAACACTAAAATTTTTG-----------

Hoga CGATTAGGCGTTTTCTTTTTAGTTATGCATAAGAACACTAAAATTTTTG-----------

CAM 236 CGATTAGGCGTTTTCTTTTTAGTTATGCATAAGAACACTAAAATTTTTG-----------

09-CS0040 CGATTAGGCGTTTTCTTTTTAGTTATGCATAAGAACACTAAAATTTTTG-----------

CN 119205 CGATTAGGCGTTTTCTTTTTAGTTATGCATAAGAACACTAAAATTTTTG-----------

Yellowstone CGATTAGGCGTTTTCTTTTTAGTTATGCATAAGAACACTAAAATTTTTG-----------

CN 120017 CGATTAGGCGTTTTCTTTTTAGTTATGCATAAGAACACTAAAATTTTTG-----------

CN 119300 CGATTAGGCGTTTTCTTTTTAGTTATGCATAAGAACACTAAAATTTTTG-----------

Jasper CGATTAGGCGTTTTCTTTTTAGTTATGCATAAGAACACTAAAATTTTTG-----------

CN 120027 CGATTAGGCGTTTTCTTTTTAGTTATGCATAAGAACACTAAAATTTTTG-----------

CN 113754 CGATTAGGCGTTTTCTTTTTAGTTATGCATAAGAACACTAAAATTTTTG-----------

Joelle AAFC CGATTAGGCGTTTTCTTTTTAGTTATGCATAAGAACACTAAAATTTTTG-----------

Joelle NCBI CGATTAGGCGTTTTCTTTTTAGTTATGCATAAGAACACTAAAATTTTTG-----------

Joelle phyto CGATTAGGCGTTTTCTTTTTAGTTATGCATAAGAACACTAAAATTTTTG-----------

Blaine Creek CGATTAGGCGTTTTCTTTTTAGTTATGCATAAGAACACTAAAATTTTTG-----------

CAM 241 CGATTAGGCGTTTTCTTTTTAGTTATGCATAAGAACACTAAAATTTTTG-----------

CN 119294 CGATTAGGCGTTTTCTTTTTAGTTATGCATAAGAACACTAAAATTTTTG-----------

***************************************** * *****

CN 120025 CGTAGTCTCAATCAAATTTCATAGGATGATGTTAAAGATAGAATTTAGAGGGTAAATGGG

CN 120013 --------------------ATAGGATGATGATAAAGATAGAATTTAGAGGATAAATAGG

CS17CS1133 --------------------ATAGGATGATGATAAAGATAGAATTTAGAGGATAAATAGG

CN 120030 --------------------ATAGGATGATGATAAAGATAGAATTTAGAGGATAAATAGG

CO46 NCBI --------------------ATAGGATGATGATAAAGATAGAATTTAGAGGATAAATAGG

DH55 ref genome --------------------ATAGGATGATGATAAAGATAGAATTTAGAGGATAAATAGG

Hoga --------------------ATAGGATGATGATAAAGATAGAATTTAGAGGATAAATAGG

CAM 236 --------------------ATAGGATGATGATAAAGATAGAATTTAGAGGATAAATAGG

09-CS0040 --------------------ATAGGATGATGATAAAGATAGAATTTAGAGGATAAATAGG

CN 119205 --------------------ATAGGATGATGATAAAGATAGAATTTAGAGGATAAATAGG

Yellowstone --------------------ATAGGATGATGATAAAGATAGAATTTAGAGGATAAATAGG

CN 120017 --------------------ATAGGATGATGATAAAGATAGAATTTAGAGGATAAATAGG

CN 119300 --------------------ATAGGATGATGATAAAGATAGAATTTAGAGGATAAATAGG

Jasper --------------------ATAGGATGATGATAAAGATAGAATTTAGAGGATAAATAGG

CN 120027 --------------------ATAGGATGATGATAAAGATAGAATTTAGAGGATAAATAGG

CN 113754 --------------------ATAGGATGATGATAAAGATAGAATTTAGAGGATAAATAGG

Joelle AAFC --------------------ATAGGATGATGATAAAGATAGAATTTAGAGGATAAATAGG

Joelle NCBI --------------------ATAGGATGATGATAAAGATAGAATTTAGAGGATAAATAGG

Joelle phyto --------------------ATAGGATGATGATAAAGATAGAATTTAGAGGATAAATAGG

Blaine Creek --------------------ATAGGATGATGATAAAGATAGAATTTAGAGGATAAATAGG

CAM 241 --------------------ATAGGATGATGATAAAGATAGAATTTAGAGGATAAATAGG

CN 119294 --------------------ATAGGATGATGATAAAGATAGAATTTAGAGGATAAATAGG

*********** ******************* ***** **

CN 120025 TTTTGTTTTTATAATGGTATGGATGCCACCATTTGTGGTTCAAATCACAGCCGAGACAAA

CN 120013 TTTTGTTTTTATAATGGTATTGATGCCACAATTTGTGGTTCAAATCACTTCCGAGACAAA

CS17CS1133 TTTTGTTTTTATAATGGTATTGATGCCACAATTTGTGGTTCAAATCACTTCCGAGACAAA

CN 120030 TTTTGTTTTTATAATGGTATTGATGCCACAATTTGTGGTTCAAATCACTTCCGAGACAAA

CO46 NCBI TTTTGTTTTTATAATGGTATTGATGCCACAATTTGTGGTTCAAATCACTTCCGAGACAAA

DH55 ref genome TTTTGTTTTTATAATGGTATTGATGCCACAATTTGTGGTTCAAATCACTTCCGAGACAAA

Hoga TTTTGTTTTTATAATGGTATTGATGCCACAATTTGTGGTTCAAATCACTTCCGAGACAAA

CAM 236 TTTTGTTTTTATAATGGTATTGATGCCACAATTTGTGGTTCAAATCACTTCCGAGACAAA

09-CS0040 TTTTGTTTTTATAATGGTATTGATGCCACAATTTGTGGTTCAAATCACTTCCGAGACAAA

CN 119205 TTTTGTTTTTATAATGGTATTGATGCCACAATTTGTGGTTCAAATCACTTCCGAGACAAA

Yellowstone TTTTGTTTTTATAATGGTATTGATGCCACAATTTGTGGTTCAAATCACTTCCGAGACAAA

CN 120017 TTTTGTTTTTATAATGGTATTGATGCCACAATTTGTGGTTCAAATCACTTCCGAGACAAA

CN 119300 TTTTGTTTTTATAATGGTATTGATGCCACAATTTGTGGTTCAAATCACTTCCGAGACAAA

Jasper TTTTGTTTTTATAATGGTATTGATGCCACAATTTGTGGTTCAAATCACTTCCGAGACAAA

CN 120027 TTTTGTTTTTATAATGGTATTGATGCCACAATTTGTGGTTCAAATCACTTCCGAGACAAA

CN 113754 TTTTGTTTTTATAATGGTATTGATGCCACAATTTGTGGTTCAAATCACTTCCGAGACAAA

Joelle AAFC TTTTGTTTTTATAATGGTATTGATGCCACAATTTGTGGTTCAAATCACTTCCGAGACAAA

Joelle NCBI TTTTGTTTTTATAATGGTATTGATGCCACAATTTGTGGTTCAAATCACTTCCGAGACAAA

Joelle phyto TTTTGTTTTTATAATGGTATTGATGCCACAATTTGTGGTTCAAATCACTTCCGAGACAAA

Blaine Creek TTTTGTTTTTATAATGGTATTGATGCCACAATTTGTGGTTCAAATCACTTCCGAGACAAA

CAM 241 TTTTGTTTTTATAATGGTATTGATGCCACAATTTGTGGTTCAAATCACTTCCGAGACAAA

CN 119294 TTTTGTTTTTATAATGGTATTGATGCCACAATTTGTGGTTCAAATCACTTCCGAGACAAA

******************** ******** ****************** **********

CN 120025 AGAAAGAAGATATATAGGGTGTTGTTTTGACTTTTGATGCTAATTGCGGTATGGATCGAA

CN 120013 AGAAAGAAGATATATAGGGTGTTGTTTTGACTTTTGATGCTAATTGCGGTATGGATCGAA

CS17CS1133 AGAAAGAAGATATATAGGGTGTTGTTTTGACTTTTGATGCTAATTGCGGTATGGATCGAA

CN 120030 AGAAAGAAGATATATAGGGTGTTGTTTTGACTTTTGATGCTAATTGCGGTATGGATCGAA

CO46 NCBI AGAAAGAAGATATATAGGGTGTTGTTTTGACTTTTGATGCTAATTGCGGTATGGATCGAA

DH55 ref genome AGAAAGAAGATATATAGGGTGTTGTTTTGACTTTTGATGCTAATTGCGGTATGGATCGAA

Hoga AGAAAGAAGATATATAGGGTGTTGTTTTGACTTTTGATGCTAATTGCGGTATGGATCGAA

CAM 236 AGAAAGAAGATATATAGGGTGTTGTTTTGACTTTTGATGCTAATTGCGGTATGGATCGAA

09-CS0040 AGAAAGAAGATATATAGGGTGTTGTTTTGACTTTTGATGCTAATTGCGGTATGGATCGAA

CN 119205 AGAAAGAAGATATATAGGGTGTTGTTTTGACTTTTGATGCTAATTGCGGTATGGATCGAA

Yellowstone AGAAAGAAGATATATAGGGTGTTGTTTTGACTTTTGATGCTAATTGCGGTATGGATCGAA

CN 120017 AGAAAGAAGATATATAGGGTGTTGTTTTGACTTTTGATGCTAATTGCGGTATGGATCGAA

CN 119300 AGAAAGAAGATATATAGGGTGTTGTTTTGACTTTTGATGCTAATTGCGGTATGGATCGAA

Jasper AGAAAGAAGATATATAGGGTGTTGTTTTGACTTTTGATGCTAATTGCGGTATGGATCGAA

CN 120027 AGAAAGAAGATATATAGGGTGTTGTTTTGACTTTTGATGCTAATTGCGGTATGGATCGAA

CN 113754 AGAAAGAAGATATATAGGGTGTTGTTTTGACTTTTGATGCTAATTGCGGTATGGATCGAA

Joelle AAFC AGAAAGAAGATATATAGGGTGTTGTTTTGACTTTTGATGCTAATTGCGGTATGGATCGAA

Joelle NCBI AGAAAGAAGATATATAGGGTGTTGTTTTGACTTTTGATGCTAATTGCGGTATGGATCGAA

Joelle phyto AGAAAGAAGATATATAGGGTGTTGTTTTGACTTTTGATGCTAATTGCGGTATGGATCGAA

Blaine Creek AGAAAGAAGATATATAGGGTGTTGTTTTGACTTTTGATGCTAATTGCGGTATGGATCGAA

CAM 241 AGAAAGAAGATATATAGGGTGTTGTTTTGACTTTTGATGCTAATTGCGGTATGGATCGAA

CN 119294 AGAAAGAAGATATATAGGGTGTTGTTTTGACTTTTGATGCTAATTGCGGTATGGATCGAA

************************************************************

CN 120025 TCCAAAAATGGAAGATCAGATAGAGGTTACATACACAATCAGAATAATGTAA-TCTATGA

CN 120013 TCCAAAAATGGAAGATCAGATAGAGGTTACAT--ACAATCAGAATAATGTAAGTCAATGA

CS17CS1133 TCCAAAAATGGAAGATCAGATAGAGGTTACAT--ACAATCAGAATAATGTAAGTCAATGA

CN 120030 TCCAAAAATGGAAGATCAGATAGAGGTTACAT--ACAATCAGAATAATGTAAGTCAATGA

CO46 NCBI TCCAAAAATGGAAGATCAGATAGAGGTTACAT--ACAATCAGAATAATGTAAGTCAATGA

DH55 ref genome TCCAAAAATGGAAGATCAGATAGAGGTTACAT--ACAATCAGAATAATGTAAGTCAATGA

Hoga TCCAAAAATGGAAGATCAGATAGAGGTTACAT--ACAATCAGAATAATGTAAGTCAATGA

CAM 236 TCCAAAAATGGAAGATCAGATAGAGGTTACAT--ACAATCAGAATAATGTAAGTCAATGA

09-CS0040 TCCAAAAATGGAAGATCAGATAGAGGTTACAT--ACAATCAGAATAATGTAAGTCAATGA

CN 119205 TCCAAAAATGGAAGATCAGATAGAGGTTACAT--ACAATCAGAATAATGTAAGTCAATGA

Yellowstone TCCAAAAATGGAAGATCAGATAGAGGTTACAT--ACAATCAGAATAATGTAAGTCAATGA

CN 120017 TCCAAAAATGGAAGATCAGATAGAGGTTACAT--ACAATCAGAATAATGTAAGTCAATGA

CN 119300 TCCAAAAATGGAAGATCAGATAGAGGTTACAT--ACAATCAGAATAATGTAAGTCAATGA

Jasper TCCAAAAATGGAAGATCAGATAGAGGTTACAT--ACAATCAGAATAATGTAAGTCAATGA

CN 120027 TCCAAAAATGGAAGATCAGATAGAGGTTACAT--ACAATCAGAATAATGTAAGTCAATGA

CN 113754 TCCAAAAATGGAAGATCAGATAGAGGTTACAT--ACAATCAGAATAATGTAAGTCAATGA

Joelle AAFC TCCAAAAATGGAAGATCAGATAGAGGTTACAT--ACAATCAGAATAATGTAAGTCAATGA

Joelle NCBI TCCAAAAATGGAAGATCAGATAGAGGTTACAT--ACAATCAGAATAATGTAAGTCAATGA

Joelle phyto TCCAAAAATGGAAGATCAGATAGAGGTTACAT--ACAATCAGAATAATGTAAGTCAATGA

Blaine Creek TCCAAAAATGGAAGATCAGATAGAGGTTACAT--ACAATCAGAATAATGTAAGTCAATGA

CAM 241 TCCAAAAATGGAAGATCAGATAGAGGTTACAT--ACAATCAGAATAATGTAAGTCAATGA

CN 119294 TCCAAAAATGGAAGATCAGATAGAGGTTACAT--ACAATCAGAATAATGTAAGTCAATGA

******************************** ****************** ** ****

CN 120025 ATTGGAAGCAGTCTTCCACTGTTTCGTATGTTTAGGGTTGTCTTTTAACTATGTGCCGAA

CN 120013 ATTGGAAGCAGTCTTCCACTGTTTCTTATGTTTAGGGTTGTCTTTTAATTATGTGCCGAA

CS17CS1133 ATTGGAAGCAGTCTTCCACTGTTTCTTATGTTTAGGGTTGTCTTTTAATTATGTGCCGAA

CN 120030 ATTGGAAGCAGTCTTCCACTGTTTCTTATGTTTAGGGTTGTCTTTTAATTATGTGCCGAA

CO46 NCBI ATTGGAAGCAGTCTTCCACTGTTTCTTATGTTTAGGGTTGTCTTTTAATTATGTGCCGAA

DH55 ref genome ATTGGAAGCAGTCTTCCACTGTTTCTTATGTTTAGGGTTGTCTTTTAATTATGTGCCGAA

Hoga ATTGGAAGCAGTCTTCCACTGTTTCTTATGTTTAGGGTTGTCTTTTAATTATGTGCCGAA

CAM 236 ATTGGAAGCAGTCTTCCACTGTTTCTTATGTTTAGGGTTGTCTTTTAATTATGTGCCGAA

09-CS0040 ATTGGAAGCAGTCTTCCACTGTTTCTTATGTTTAGGGTTGTCTTTTAATTATGTGCCGAA

CN 119205 ATTGGAAGCAGTCTTCCACTGTTTCTTATGTTTAGGGTTGTCTTTTAATTATGTGCCGAA

Yellowstone ATTGGAAGCAGTCTTCCACTGTTTCTTATGTTTAGGGTTGTCTTTTAATTATGTGCCGAA

CN 120017 ATTGGAAGCAGTCTTCCACTGTTTCTTATGTTTAGGGTTGTCTTTTAATTATGTGCCGAA

CN 119300 ATTGGAAGCAGTCTTCCACTGTTTCTTATGTTTAGGGTTGTCTTTTAATTATGTGCCGAA

Jasper ATTGGAAGCAGTCTTCCACTGTTTCTTATGTTTAGGGTTGTCTTTTAATTATGTGCCGAA

CN 120027 ATTGGAAGCAGTCTTCCACTGTTTCTTATGTTTAGGGTTGTCTTTTAATTATGTGCCGAA

CN 113754 ATTGGAAGCAGTCTTCCACTGTTTCTTATGTTTAGGGTTGTCTTTTAATTATGTGCCGAA

Joelle AAFC ATTGGAAGCAGTCTTCCACTGTTTCTTATGTTTAGGGTTGTCTTTTAATTATGTGCCGAA

Joelle NCBI ATTGGAAGCAGTCTTCCACTGTTTCTTATGTTTAGGGTTGTCTTTTAATTATGTGCCGAA

Joelle phyto ATTGGAAGCAGTCTTCCACTGTTTCTTATGTTTAGGGTTGTCTTTTAATTATGTGCCGAA

Blaine Creek ATTGGAAGCAGTCTTCCACTGTTTCTTATGTTTAGGGTTGTCTTTTAATTATGTGCCGAA

CAM 241 ATTGGAAGCAGTCTTCCACTGTTTCTTATGTTTAGGGTTGTCTTTTAATTATGTGCCGAA

CN 119294 ATTGGAAGCAGTCTTCCACTGTTTCTTATGTTTAGGGTTGTCTTTTAATTATGTGCCGAA

************************* ********************** ***********

CN 120025 ATTATAAATAAAAATGACTTTCTGTAAGACTCAGTCCATGTCCTTGGAATTTGGCTTAAA

CN 120013 ATTATAAATAAAAATGACTTTCTGTAAGACTCAGTCCATGTCCTTGGAATTTGGCTTAAA

CS17CS1133 ATTATAAATAAAAATGACTTTCTGTAAGACTCAGTCCATGTCCTTGGAATTTGGCTTAAA

CN 120030 ATTATAAATAAAAATGACTTTCTGTAAGACTCAGTCCATGTCCTTGGAATTTGGCTTAAA

CO46 NCBI ATTATAAATAAAAATGACTTTCTGTAAGACTCAGTCCATGTCCTTGGAATTTGGCTTAAA

DH55 ref genome ATTATAAATAAAAATGACTTTCTGTAAGACTCAGTCCATGTCCTTGGAATTTGGCTTAAA

Hoga ATTATAAATAAAAATGACTTTCTGTAAGACTCAGTCCATGTCCTTGGAATTTGGCTTAAA

CAM 236 ATTATAAATAAAAATGACTTTCTGTAAGACTCAGTCCATGTCCTTGGAATTTGGCTTAAA

09-CS0040 ATTATAAATAAAAATGACTTTCTGTAAGACTCAGTCCATGTCCTTGGAATTTGGCTTAAA

CN 119205 ATTATAAATAAAAATGACTTTCTGTAAGACTCAGTCCATGTCCTTGGAATTTGGCTTAAA

Yellowstone ATTATAAATAAAAATGACTTTCTGTAAGACTCAGTCCATGTCCTTGGAATTTGGCTTAAA

CN 120017 ATTATAAATAAAAATGACTTTCTGTAAGACTCAGTCCATGTCCTTGGAATTTGGCTTAAA

CN 119300 ATTATAAATAAAAATGACTTTCTGTAAGACTCAGTCCATGTCCTTGGAATTTGGCTTAAA

Jasper ATTATAAATAAAAATGACTTTCTGTAAGACTCAGTCCATGTCCTTGGAATTTGGCTTAAA

CN 120027 ATTATAAATAAAAATGACTTTCTGTAAGACTCAGTCCATGTCCTTGGAATTTGGCTTAAA

CN 113754 ATTATAAATAAAAATGACTTTCTGTAAGACTCAGTCCATGTCCTTGGAATTTGGCTTAAA

Joelle AAFC ATTATAAATAAAAATGACTTTCTGTAAGACTCAGTCCATGTCCTTGGAATTTGGCTTAAA

Joelle NCBI ATTATAAATAAAAATGACTTTCTGTAAGACTCAGTCCATGTCCTTGGAATTTGGCTTAAA

Joelle phyto ATTATAAATAAAAATGACTTTCTGTAAGACTCAGTCCATGTCCTTGGAATTTGGCTTAAA

Blaine Creek ATTATAAATAAAAATGACTTTCTGTAAGACTCAGTCCATGTCCTTGGAATTTGGCTTAAA

CAM 241 ATTATAAATAAAAATGACTTTCTGTAAGACTCAGTCCATGTCCTTGGAATTTGGCTTAAA

CN 119294 ATTATAAATAAAAATGACTTTCTGTAAGACTCAGTCCATGTCCTTGGAATTTGGCTTAAA

************************************************************

CN 120025 CATAGGTATATTGGACGTCT-------GCTTACTGCCATGTCATCACGTTGTGGTTCATC

CN 120013 CGTAGGTATATTGGACGTCTGCTGAGCGCTTACTGCCATGTCATCATGTAGTGATTCATC

CS17CS1133 CGTAGGTATATTGGACGTCTGCTGAGCGCTTACTGCCATGTCATCATGTAGTGATTCATC

CN 120030 CGTAGGTATATTGGACGTCTGCTGAGCGCTTACTGCCATGTCATCATGTAGTGATTCATC

CO46 NCBI CGTAGGTATATTGGACGTCTGCTGAGCGCTTACTGCCATGTCATCATGTAGTGATTCATC

DH55 ref genome CGTAGGTATATTGGACGTCTGCTGAGCGCTTACTGCCATGTCATCATGTAGTGATTCATC

Hoga CGTAGGTATATTGGACGTCTGCTGAGCGCTTACTGCCATGTCATCATGTAGTGATTCATC

CAM 236 CGTAGGTATATTGGACGTCTGCTGAGCGCTTACTGCCATGTCATCATGTAGTGATTCATC

09-CS0040 CGTAGGTATATTGGACGTCTGCTGAGCGCTTACTGCCATGTCATCATGTAGTGATTCATC

CN 119205 CGTAGGTATATTGGACGTCTGCTGAGCGCTTACTGCCATGTCATCATGTAGTGATTCATC

Yellowstone CGTAGGTATATTGGACGTCTGCTGAGCGCTTACTGCCATGTCATCATGTAGTGATTCATC

CN 120017 CGTAGGTATATTGGACGTCTGCTGAGCGCTTACTGCCATGTCATCATGTAGTGATTCATC

CN 119300 CGTAGGTATATTGGACGTCTGCTGAGCGCTTACTGCCATGTCATCATGTAGTGATTCATC

Jasper CGTAGGTATATTGGACGTCTGCTGAGCGCTTACTGCCATGTCATCATGTAGTGATTCATC

CN 120027 CGTAGGTATATTGGACGTCTGCTGAGCGCTTACTGCCATGTCATCATGTAGTGATTCATC

CN 113754 CGTAGGTATATTGGACGTCTGCTGAGCGCTTACTGCCATGTCATCATGTAGTGATTCATC

Joelle AAFC CGTAGGTATATTGGACGTCTGCTGAGCGCTTACTGCCATGTCATCATGTAGTGATTCATC

Joelle NCBI CGTAGGTATATTGGACGTCTGCTGAGCGCTTACTGCCATGTCATCATGTAGTGATTCATC

Joelle phyto CGTAGGTATATTGGACGTCTGCTGAGCGCTTACTGCCATGTCATCATGTAGTGATTCATC

Blaine Creek CGTAGGTATATTGGACGTCTGCTGAGCGCTTACTGCCATGTCATCATGTAGTGATTCATC

CAM 241 CGTAGGTATATTGGACGTCTGCTGAGCGCTTACTGCCATGTCATCATGTAGTGATTCATC

CN 119294 CGTAGGTATATTGGACGTCTGCTGAGCGCTTACTGCCATGTCATCATGTAGTGATTCATC

* ****************** ******************* ** *** ******

CN 120025 AATATATGTGTCTACGTTTTCGTGAGTATATTTTTTCTTTTTAACAG--AAAAGTCTGTG

CN 120013 AATATCTGTGTGTACGTTTTCGTGAGTATATGTTTTC--TTTAACAGTAAAAAGTCTGTT

CS17CS1133 AATATCTGTGTGTACGTTTTCGTGAGTATATGTTTTC--TTTAACAGTAAAAAGTCTGTT

CN 120030 AATATCTGTGTGTACGTTTTCGTGAGTATATGTTTTC--TTTAACAGTAAAAAGTCTGTT

CO46 NCBI AATATCTGTGTGTACGTTTTCGTGAGTATATGTTTTC--TTTAACAGTAAAAAGTCTGTT

DH55 ref genome AATATCTGTGTGTACGTTTTCGTGAGTATATGTTTTC--TTTAACAGTAAAAAGTCTGTT

Hoga AATATCTGTGTGTACGTTTTCGTGAGTATATGTTTTC--TTTAACAGTAAAAAGTCTGTT

CAM 236 AATATCTGTGTGTACGTTTTCGTGAGTATATGTTTTC--TTTAACAGTAAAAAGTCTGTT

09-CS0040 AATATCTGTGTGTACGTTTTCGTGAGTATATGTTTTC--TTTAACAGTAAAAAGTCTGTT

CN 119205 AATATCTGTGTGTACGTTTTCGTGAGTATATGTTTTC--TTTAACAGTAAAAAGTCTGTT

Yellowstone AATATCTGTGTGTACGTTTTCGTGAGTATATGTTTTC--TTTAACAGTAAAAAGTCTGTT

CN 120017 AATATCTGTGTGTACGTTTTCGTGAGTATATGTTTTC--TTTAACAGTAAAAAGTCTGTT

CN 119300 AATATCTGTGTGTACGTTTTCGTGAGTATATGTTTTC--TTTAACAGTAAAAAGTCTGTT

Jasper AATATCTGTGTGTACGTTTTCGTGAGTATATGTTTTC--TTTAACAGTAAAAAGTCTGTT

CN 120027 AATATCTGTGTGTACGTTTTCGTGAGTATATGTTTTC--TTTAACAGTAAAAAGTCTGTT

CN 113754 AATATCTGTGTGTACGTTTTCGTGAGTATATGTTTTC--TTTAACAGTAAAAAGTCTGTT

Joelle AAFC AATATCTGTGTGTACGTTTTCGTGAGTATATGTTTTC--TTTAACAGTAAAAAGTCTGTT

Joelle NCBI AATATCTGTGTGTACGTTTTCGTGAGTATATGTTTTC--TTTAACAGTAAAAAGTCTGTT

Joelle phyto AATATCTGTGTGTACGTTTTCGTGAGTATATGTTTTC--TTTAACAGTAAAAAGTCTGTT

Blaine Creek AATATCTGTGTGTACGTTTTCGTGAGTATATGTTTTC--TTTAACAGTAAAAAGTCTGTT

CAM 241 AATATCTGTGTGTACGTTTTCGTGAGTATATGTTTTC--TTTAACAGTAAAAAGTCTGTT

CN 119294 AATATCTGTGTGTACGTTTTCGTGAGTATATGTTTTC--TTTAACAGTAAAAAGTCTGTT

***** ***** ******************* ***** ******** **********

CN 120025 TGTATTGTACACTCTCTCTTGAGCCTCAATTGCTTGTTTGCATTTAAGTTTGCTGCTGTG

CN 120013 TGTATTGTACACTCTCTCTTAAGCCTCAGTTGCTTGTTTGCATTTAAGTTTCCTTCTGTG

CS17CS1133 TGTATTGTACACTCTCTCTTAAGCCTCAGTTGCTTGTTTGCATTTAAGTTTCCTTCTGTG

CN 120030 TGTATTGTACACTCTCTCTTAAGCCTCAGTTGCTTGTTTGCATTTAAGTTTCCTTCTGTG

CO46 NCBI TGTATTGTACACTCTCTCTTAAGCCTCAGTTGCTTGTTTGCATTTAAGTTTCCTTCTGTG

DH55 ref genome TGTATTGTACACTCTCTCTTAAGCCTCAGTTGCTTGTTTGCATTTAAGTTTCCTTCTGTG

Hoga TGTATTGTACACTCTCTCTTAAGCCTCAGTTGCTTGTTTGCATTTAAGTTTCCTTCTGTG

CAM 236 TGTATTGTACACTCTCTCTTAAGCCTCAGTTGCTTGTTTGCATTTAAGTTTCCTTCTGTG

09-CS0040 TGTATTGTACACTCTCTCTTAAGCCTCAGTTGCTTGTTTGCATTTAAGTTTCCTTCTGTG

CN 119205 TGTATTGTACACTCTCTCTTAAGCCTCAGTTGCTTGTTTGCATTTAAGTTTCCTTCTGTG

Yellowstone TGTATTGTACACTCTCTCTTAAGCCTCAGTTGCTTGTTTGCATTTAAGTTTCCTTCTGTG

CN 120017 TGTATTGTACACTCTCTCTTAAGCCTCAGTTGCTTGTTTGCATTTAAGTTTCCTTCTGTG

CN 119300 TGTATTGTACACTCTCTCTTAAGCCTCAGTTGCTTGTTTGCATTTAAGTTTCCTTCTGTG

Jasper TGTATTGTACACTCTCTCTTAAGCCTCAGTTGCTTGTTTGCATTTAAGTTTCCTTCTGTG

CN 120027 TGTATTGTACACTCTCTCTTAAGCCTCAGTTGCTTGTTTGCATTTAAGTTTCCTTCTGTG

CN 113754 TGTATTGTACACTCTCTCTTAAGCCTCAGTTGCTTGTTTGCATTTAAGTTTCCTTCTGTG

Joelle AAFC TGTATTGTACACTCTCTCTTAAGCCTCAGTTGCTTGTTTGCATTTAAGTTTCCTTCTGTG

Joelle NCBI TGTATTGTACACTCTCTCTTAAGCCTCAGTTGCTTGTTTGCATTTAAGTTTCCTTCTGTG

Joelle phyto TGTATTGTACACTCTCTCTTAAGCCTCAGTTGCTTGTTTGCATTTAAGTTTCCTTCTGTG

Blaine Creek TGTATTGTACACTCTCTCTTAAGCCTCAGTTGCTTGTTTGCATTTAAGTTTCCTTCTGTG

CAM 241 TGTATTGTACACTCTCTCTTAAGCCTCAGTTGCTTGTTTGCATTTAAGTTTCCTTCTGTG

CN 119294 TGTATTGTACACTCTCTCTTAAGCCTCAGTTGCTTGTTTGCATTTAAGTTTCCTTCTGTG

******************** ******* ********************** ** *****

CN 120025 TTTCCATGTTATATTATATCAATCAATGTACCATATTTAGAAAGCACAAACACAATTGCA

CN 120013 TTTCCATGTTATATTATATCAATCAATGTACCATATATAG-AAGCACAAACAAAATTGCA

CS17CS1133 TTTCCATGTTATATTATATCAATCAATGTACCATATATAG-AAGCACAAACAAAATTGCA

CN 120030 TTTCCATGTTATATTATATCAATCAATGTACCATATATAG-AAGCACAAACAAAATTGCA

CO46 NCBI TTTCCATGTTATATTATATCAATCAATGTACCATATATAG-AAGCACAAACAAAATTGCA

DH55 ref genome TTTCCATGTTATATTATATCAATCAATGTACCATATATAG-AAGCACAAACAAAATTGCA

Hoga TTTCCATGTTATATTATATCAATCAATGTACCATATATAG-AAGCACAAACAAAATTGCA

CAM 236 TTTCCATGTTATATTATATCAATCAATGTACCATATATAG-AAGCACAAACAAAATTGCA

09-CS0040 TTTCCATGTTATATTATATCAATCAATGTACCATATATAG-AAGCACAAACAAAATTGCA

CN 119205 TTTCCATGTTATATTATATCAATCAATGTACCATATATAG-AAGCACAAACAAAATTGCA

Yellowstone TTTCCATGTTATATTATATCAATCAATGTACCATATATAG-AAGCACAAACAAAATTGCA

CN 120017 TTTCCATGTTATATTATATCAATCAATGTACCATATATAG-AAGCACAAACAAAATTGCA

CN 119300 TTTCCATGTTATATTATATCAATCAATGTACCATATATAG-AAGCACAAACAAAATTGCA

Jasper TTTCCATGTTATATTATATCAATCAATGTACCATATATAG-AAGCACAAACAAAATTGCA

CN 120027 TTTCCATGTTATATTATATCAATCAATGTACCATATATAG-AAGCACAAACAAAATTGCA

CN 113754 TTTCCATGTTATATTATATCAATCAATGTACCATATATAG-AAGCACAAACAAAATTGCA

Joelle AAFC TTTCCATGTTATATTATATCAATCAATGTACCATATATAG-AAGCACAAACAAAATTGCA

Joelle NCBI TTTCCATGTTATATTATATCAATCAATGTACCATATATAG-AAGCACAAACAAAATTGCA

Joelle phyto TTTCCATGTTATATTATATCAATCAATGTACCATATATAG-AAGCACAAACAAAATTGCA

Blaine Creek TTTCCATGTTATATTATATCAATCAATGTACCATATATAG-AAGCACAAACAAAATTGCA

CAM 241 TTTCCATGTTATATTATATCAATCAATGTACCATATATAG-AAGCACAAACAAAATTGCA

CN 119294 TTTCCATGTTATATTATATCAATCAATGTACCATATATAG-AAGCACAAACAAAATTGCA

************************************ *** *********** *******

CN 120025 TAGAAACAATCTGGACAGTGGATGCTTGAGATTAGGGTTTTCTGTAAATGAATTGTTAGA

CN 120013 TAGAAACAATCTGGACAGTGGACGCTTGAGATTAGGGTTTTCTGTAAACGAATT-TTAGA

CS17CS1133 TAGAAACAATCTGGACAGTGGACGCTTGAGATTAGGGTTTTCTGTAAACGAATT-TTAGA

CN 120030 TAGAAACAATCTGGACAGTGGACGCTTGAGATTAGGGTTTTCTGTAAACGAATT-TTAGA

CO46 NCBI TAGAAACAATCTGGACAGTGGACGCTTGAGATTAGGGTTTTCTGTAAACGAATT-TTAGA

DH55 ref genome TAGAAACAATCTGGACAGTGGACGCTTGAGATTAGGGTTTTCTGTAAACGAATT-TTAGA

Hoga TAGAAACAATCTGGACAGTGGACGCTTGAGATTAGGGTTTTCTGTAAACGAATT-TTAGA

CAM 236 TAGAAACAATCTGGACAGTGGACGCTTGAGATTAGGGTTTTCTGTAAACGAATT-TTAGA

09-CS0040 TAGAAACAATCTGGACAGTGGACGCTTGAGATTAGGGTTTTCTGTAAACGAATT-TTAGA

CN 119205 TAGAAACAATCTGGACAGTGGACGCTTGAGATTAGGGTTTTCTGTAAACGAATT-TTAGA

Yellowstone TAGAAACAATCTGGACAGTGGACGCTTGAGATTAGGGTTTTCTGTAAACGAATT-TTAGA

CN 120017 TAGAAACAATCTGGACAGTGGACGCTTGAGATTAGGGTTTTCTGTAAACGAATT-TTAGA

CN 119300 TAGAAACAATCTGGACAGTGGACGCTTGAGATTAGGGTTTTCTGTAAACGAATT-TTAGA

Jasper TAGAAACAATCTGGACAGTGGACGCTTGAGATTAGGGTTTTCTGTAAACGAATT-TTAGA

CN 120027 TAGAAACAATCTGGACAGTGGACGCTTGAGATTAGGGTTTTCTGTAAACGAATT-TTAGA

CN 113754 TAGAAACAATCTGGACAGTGGACGCTTGAGATTAGGGTTTTCTGTAAACGAATT-TTAGA

Joelle AAFC TAGAAACAATCTGGACAGTGGACGCTTGAGATTAGGGTTTTCTGTAAACGAATT-TTAGA

Joelle NCBI TAGAAACAATCTGGACAGTGGACGCTTGAGATTAGGGTTTTCTGTAAACGAATT-TTAGA

Joelle phyto TAGAAACAATCTGGACAGTGGACGCTTGAGATTAGGGTTTTCTGTAAACGAATT-TTAGA

Blaine Creek TAGAAACAATCTGGACAGTGGACGCTTGAGATTAGGGTTTTCTGTAAACGAATT-TTAGA

CAM 241 TAGAAACAATCTGGACAGTGGACGCTTGAGATTAGGGTTTTCTGTAAACGAATT-TTAGA

CN 119294 TAGAAACAATCTGGACAGTGGACGCTTGAGATTAGGGTTTTCTGTAAACGAATT-TTAGA

********************** ************************* ***** *****

CN 120025 TCACAGGGATAACCTATCTACATGCCTTAA--------TTTTAGTAAGCCTTTTCTTCGT

CN 120013 TCACAGGGATAATCTATATACATGCCTTAGTTTTAGACTTTTAGTAAACCTTTTCTTTGT

CS17CS1133 TCACAGGGATAATCTATATACATGCCTTAGTTTTAGACTTTTAGTAAACCTTTTCTTTGT

CN 120030 TCACAGGGATAATCTATATACATGCCTTAGTTTTAGACTTTTAGTAAACCTTTTCTTTGT

CO46 NCBI TCACAGGGATAATCTATATACATGCCTTAGTTTTAGACTTTTAGTAAACCTTTTCTTTGT

DH55 ref genome TCACAGGGATAATCTATATACATGCCTTAGTTTTAGACTTTTAGTAAACCTTTTCTTTGT

Hoga TCACAGGGATAATCTATATACATGCCTTAGTTTTAGACTTTTAGTAAACCTTTTCTTTGT

CAM 236 TCACAGGGATAATCTATATACATGCCTTAGTTTTAGACTTTTAGTAAACCTTTTCTTTGT

09-CS0040 TCACAGGGATAATCTATATACATGCCTTAGTTTTAGACTTTTAGTAAACCTTTTCTTTGT

CN 119205 TCACAGGGATAATCTATATACATGCCTTAGTTTTAGACTTTTAGTAAACCTTTTCTTTGT

Yellowstone TCACAGGGATAATCTATATACATGCCTTAGTTTTAGACTTTTAGTAAACCTTTTCTTTGT

CN 120017 TCACAGGGATAATCTATATACATGCCTTAGTTTTAGACTTTTAGTAAACCTTTTCTTTGT

CN 119300 TCACAGGGATAATCTATATACATGCCTTAGTTTTAGACTTTTAGTAAACCTTTTCTTTGT

Jasper TCACAGGGATAATCTATATACATGCCTTAGTTTTAGACTTTTAGTAAACCTTTTCTTTGT

CN 120027 TCACAGGGATAATCTATATACATGCCTTAGTTTTAGACTTTTAGTAAACCTTTTCTTTGT

CN 113754 TCACAGGGATAATCTATATACATGCCTTAGTTTTAGACTTTTAGTAAACCTTTTCTTTGT

Joelle AAFC TCACAGGGATAATCTATATACATGCCTTAGTTTTAGACTTTTAGTAAACCTTTTCTTTGT

Joelle NCBI TCACAGGGATAATCTATATACATGCCTTAGTTTTAGACTTTTAGTAAACCTTTTCTTTGT

Joelle phyto TCACAGGGATAATCTATATACATGCCTTAGTTTTAGACTTTTAGTAAACCTTTTCTTTGT

Blaine Creek TCACAGGGATAATCTATATACATGCCTTAGTTTTAGACTTTTAGTAAACCTTTTCTTTGT

CAM 241 TCACAGGGATAATCTATATACATGCCTTAGTTTTAGACTTTTAGTAAACCTTTTCTTTGT

CN 119294 TCACAGGGATAATCTATATACATGCCTTAGTTTTAGACTTTTAGTAAACCTTTTCTTTGT

************ **** *********** ********* ********* **

CN 120025 GGAAAAATGTTCAATTTCAACATACCGAAATATATAATAGTAATTCATTTGATAATCAAA

CN 120013 GGAAAAATGTAAAATCTCAACCTACCGATATATATTAAAATAATTCATTGGATAATCAAA

CS17CS1133 GGAAAAATGTAAAATCTCAACCTACCGATATATATTAAAATAATTCATTGGATAATCAAA

CN 120030 GGAAAAATGTAAAATCTCAACCTACCGATATATATTAAAATAATTCATTGGATAATCAAA

CO46 NCBI GGAAAAATGTAAAATCTCAACCTACCGATATATATTAAAATAATTCATTGGATAATCAAA

DH55 ref genome GGAAAAATGTAAAATCTCAACCTACCGATATATATTAAAATAATTCATTGGATAATCAAA

Hoga GGAAAAATGTAAAATCTCAACCTACCGATATATATTAAAATAATTCATTGGATAATCAAA

CAM 236 GGAAAAATGTAAAATCTCAACCTACCGATATATATTAAAATAATTCATTGGATAATCAAA

09-CS0040 GGAAAAATGTAAAATCTCAACCTACCGATATATATTAAAATAATTCATTGGATAATCAAA

CN 119205 GGAAAAATGTAAAATCTCAACCTACCGATATATATTAAAATAATTCATTGGATAATCAAA

Yellowstone GGAAAAATGTAAAATCTCAACCTACCGATATATATTAAAATAATTCATTGGATAATCAAA

CN 120017 GGAAAAATGTAAAATCTCAACCTACCGATATATATTAAAATAATTCATTGGATAATCAAA

CN 119300 GGAAAAATGTAAAATCTCAACCTACCGATATATATTAAAATAATTCATTGGATAATCAAA

Jasper GGAAAAATGTAAAATCTCAACCTACCGATATATATTAAAATAATTCATTGGATAATCAAA

CN 120027 GGAAAAATGTAAAATCTCAACCTACCGATATATATTAAAATAATTCATTGGATAATCAAA

CN 113754 GGAAAAATGTAAAATCTCAACCTACCGATATATATTAAAATAATTCATTGGATAATCAAA

Joelle AAFC GGAAAAATGTAAAATCTCAACCTACCGATATATATTAAAATAATTCATTGGATAATCAAA

Joelle NCBI GGAAAAATGTAAAATCTCAACCTACCGATATATATTAAAATAATTCATTGGATAATCAAA

Joelle phyto GGAAAAATGTAAAATCTCAACCTACCGATATATATTAAAATAATTCATTGGATAATCAAA

Blaine Creek GGAAAAATGTAAAATCTCAACCTACCGATATATATTAAAATAATTCATTGGATAATCAAA

CAM 241 GGAAAAATGTAAAATCTCAACCTACCGATATATATTAAAATAATTCATTGGATAATCAAA

CN 119294 GGAAAAATGTAAAATCTCAACCTACCGATATATATTAAAATAATTCATTGGATAATCAAA

********** *** ***** ****** ****** * * ********* **********

CN 120025 CTTTGAATATTTCTTGGAAAATTTTTAGAGTTCTTTTTTTTAGAGATGAAAAGATCTTTA

CN 120013 CTTTGAACATTTCTTGGTAAATTTTTAGAGTTA-TTTATTTAGAGATGAAAAGATCTATA

CS17CS1133 CTTTGAACATTTCTTGGTAAATTTTTAGAGTTA-TTTATTTAGAGATGAAAAGATCTATA

CN 120030 CTTTGAACATTTCTTGGTAAATTTTTAGAGTTA-TTTATTTAGAGATGAAAAGATCTATA

CO46 NCBI CTTTGAACATTTCTTGGTAAATTTTTAGAGTTA-TTTATTTAGAGATGAAAAGATCTATA

DH55 ref genome CTTTGAACATTTCTTGGTAAATTTTTAGAGTTA-TTTATTTAGAGATGAAAAGATCTATA

Hoga CTTTGAACATTTCTTGGTAAATTTTTAGAGTTA-TTTATTTAGAGATGAAAAGATCTATA

CAM 236 CTTTGAACATTTCTTGGTAAATTTTTAGAGTTA-TTTATTTAGAGATGAAAAGATCTATA

09-CS0040 CTTTGAACATTTCTTGGTAAATTTTTAGAGTTA-TTTATTTAGAGATGAAAAGATCTATA

CN 119205 CTTTGAACATTTCTTGGTAAATTTTTAGAGTTA-TTTATTTAGAGATGAAAAGATCTATA

Yellowstone CTTTGAACATTTCTTGGTAAATTTTTAGAGTTA-TTTATTTAGAGATGAAAAGATCTATA

CN 120017 CTTTGAACATTTCTTGGTAAATTTTTAGAGTTA-TTTATTTAGAGATGAAAAGATCTATA

CN 119300 CTTTGAACATTTCTTGGTAAATTTTTAGAGTTA-TTTATTTAGAGATGAAAAGATCTATA

Jasper CTTTGAACATTTCTTGGTAAATTTTTAGAGTTA-TTTATTTAGAGATGAAAAGATCTATA

CN 120027 CTTTGAACATTTCTTGGTAAATTTTTAGAGTTA-TTTATTTAGAGATGAAAAGATCTATA

CN 113754 CTTTGAACATTTCTTGGTAAATTTTTAGAGTTA-TTTATTTAGAGATGAAAAGATCTATA

Joelle AAFC CTTTGAACATTTCTTGGTAAATTTTTAGAGTTA-TTTATTTAGAGATGAAAAGATCTATA

Joelle NCBI CTTTGAACATTTCTTGGTAAATTTTTAGAGTTA-TTTATTTAGAGATGAAAAGATCTATA

Joelle phyto CTTTGAACATTTCTTGGTAAATTTTTAGAGTTA-TTTATTTAGAGATGAAAAGATCTATA

Blaine Creek CTTTGAACATTTCTTGGTAAATTTTTAGAGTTA-TTTATTTAGAGATGAAAAGATCTATA

CAM 241 CTTTGAACATTTCTTGGTAAATTTTTAGAGTTA-TTTATTTAGAGATGAAAAGATCTATA

CN 119294 CTTTGAACATTTCTTGGTAAATTTTTAGAGTTA-TTTATTTAGAGATGAAAAGATCTATA

******* ********* ************** *** ******************* **

CN 120025 TATGTGTAATTAGTAAGGTTTTGTCTCTAAATGTCTCTTCTGATGCCACCATATACATGT

CN 120013 TATGTGTAATTATTTAGGTTTTGTCTCTAAATGTCTCCTTTGATGACACCATATACATGT

CS17CS1133 TATGTGTAATTATTTAGGTTTTGTCTCTAAATGTCTCCTTTGATGACACCATATACATGT

CN 120030 TATGTGTAATTATTTAGGTTTTGTCTCTAAATGTCTCCTTTGATGACACCATATACATGT

CO46 NCBI TATGTGTAATTATTTAGGTTTTGTCTCTAAATGTCTCCTTTGATGACACCATATACATGT

DH55 ref genome TATGTGTAATTATTTAGGTTTTGTCTCTAAATGTCTCCTTTGATGACACCATATACATGT

Hoga TATGTGTAATTATTTAGGTTTTGTCTCTAAATGTCTCCTTTGATGACACCATATACATGT

CAM 236 TATGTGTAATTATTTAGGTTTTGTCTCTAAATGTCTCCTTTGATGACACCATATACATGT

09-CS0040 TATGTGTAATTATTTAGGTTTTGTCTCTAAATGTCTCCTTTGATGACACCATATACATGT

CN 119205 TATGTGTAATTATTTAGGTTTTGTCTCTAAATGTCTCCTTTGATGACACCATATACATGT

Yellowstone TATGTGTAATTATTTAGGTTTTGTCTCTAAATGTCTCCTTTGATGACACCATATACATGT

CN 120017 TATGTGTAATTATTTAGGTTTTGTCTCTAAATGTCTCCTTTGATGACACCATATACATGT

CN 119300 TATGTGTAATTATTTAGGTTTTGTCTCTAAATGTCTCCTTTGATGACACCATATACATGT

Jasper TATGTGTAATTATTTAGGTTTTGTCTCTAAATGTCTCCTTTGATGACACCATATACATGT

CN 120027 TATGTGTAATTATTTAGGTTTTGTCTCTAAATGTCTCCTTTGATGACACCATATACATGT

CN 113754 TATGTGTAATTATTTAGGTTTTGTCTCTAAATGTCTCCTTTGATGACACCATATACATGT

Joelle AAFC TATGTGTAATTATTTAGGTTTTGTCTCTAAATGTCTCCTTTGATGACACCATATACATGT

Joelle NCBI TATGTGTAATTATTTAGGTTTTGTCTCTAAATGTCTCCTTTGATGACACCATATACATGT

Joelle phyto TATGTGTAATTATTTAGGTTTTGTCTCTAAATGTCTCCTTTGATGACACCATATACATGT

Blaine Creek TATGTGTAATTATTTAGGTTTTGTCTCTAAATGTCTCCTTTGATGACACCATATACATGT

CAM 241 TATGTGTAATTATTTAGGTTTTGTCTCTAAATGTCTCCTTTGATGACACCATATACATGT

CN 119294 TATGTGTAATTATTTAGGTTTTGTCTCTAAATGTCTCCTTTGATGACACCATATACATGT

************ * ********************** * ***** **************

CN 120025 CTCATAGTTTCCAGTGGTTTTCTGAAGGGTTAGCTTGTATT-------------------

CN 120013 TTTATAGTTTCCAGTGGTCTTTTCAAGGGTTACCTTGTAT--------------------

CS17CS1133 TTTATAGTTTCCAGTGGTCTTTTCAAGGGTTACCTTGTAT--------------------

CN 120030 TTTATAGTTTCCAGTGGTCTTTTCAAGGGTTACCTTGTATTTTCACTACACGAAAACACG

CO46 NCBI TTTATAGTTTCCAGTGGTCTTTTCAAGGGTTACCTTGTAT--------------------

DH55 ref genome TTTATAGTTTCCAGTGGTCTTTTCAAGGGTTACCTTGTAT--------------------

Hoga TTTATAGTTTCCAGTGGTCTTTTCAAGGGTTACCTTGTAT--------------------

CAM 236 TTTATAGTTTCCAGTGGTCTTTTCAAGGGTTACCTTGTAT--------------------

09-CS0040 TTTATAGTTTCCAGTGGTCTTTTCAAGGGTTACCTTGTAT--------------------

CN 119205 TTTATAGTTTCCAGTGGTCTTTTCAAGGGTTACCTTGTAT--------------------

Yellowstone TTTATAGTTTCCAGTGGTCTTTTCAAGGGTTACCTTGTAT--------------------

CN 120017 TTTATAGTTTCCAGTGGTCTTTTCAAGGGTTACCTTGTAT--------------------

CN 119300 TTTATAGTTTCCAGTGGTCTTTTCAAGGGTTACCTTGTAT--------------------

Jasper TTTATAGTTTCCAGTGGTCTTTTCAAGGGTTACCTTGTAT--------------------

CN 120027 TTTATAGTTTCCAGTGGTCTTTTCAAGGGTTACCTTGTAT--------------------

CN 113754 TTTATAGTTTCCAGTGGTCTTTTCAAGGGTTACCTTGTAT--------------------

Joelle AAFC TTTATAGTTTCCAGTGGTCTTTTCAAGGGTTACCTTGTAT--------------------

Joelle NCBI TTTATAGTTTCCAGTGGTCTTTTCAAGGGTTACCTTGTAT--------------------

Joelle phyto TTTATAGTTTCCAGTGGTCTTTTCAAGGGTTACCTTGTAT--------------------

Blaine Creek TTTATAGTTTCCAGTGGTCTTTTCAAGGGTTACCTTGTATTTTCACTACACGAAAACACG

CAM 241 TTTATAGTTTCCAGTGGTCTTTTCAAGGGTTACCTTGTATTTTCACTACACGAAAACACG

CN 119294 TTTATAGTTTCCAGTGGTCTTTTCAAGGGTTACCTTGTAT--------------------

* *************** ** * ******** *******

CN 120025 ------------------------------------------------------------

CN 120013 ------------------------------------------------------------

CS17CS1133 ------------------------------------------------------------

CN 120030 GCGTTTGGGACTACTTGTTGTGACTGAATTTATAGTCGCAAACTTTTGTGACTACTGTGT

CO46 NCBI ------------------------------------------------------------

DH55 ref genome ------------------------------------------------------------

Hoga ------------------------------------------------------------

CAM 236 ------------------------------------------------------------

09-CS0040 ------------------------------------------------------------

CN 119205 ------------------------------------------------------------

Yellowstone ------------------------------------------------------------

CN 120017 ------------------------------------------------------------

CN 119300 ------------------------------------------------------------

Jasper ------------------------------------------------------------

CN 120027 ------------------------------------------------------------

CN 113754 ------------------------------------------------------------

Joelle AAFC ------------------------------------------------------------

Joelle NCBI ------------------------------------------------------------

Joelle phyto ------------------------------------------------------------

Blaine Creek GCGTTTGGGACTACTTGTTGTGACTGAATTTATAGTCGCAAACTTTTGTGACTACTGTGT

CAM 241 GCGTTTGGGACTACTTGTTGTGACTGAATTTATAGTCGCAAACTTTTGTGACTACTGTGT

CN 119294 ------------------------------------------------------------

CN 120025 ------------------------------------------------------------

CN 120013 ------------------------------------------------------------

CS17CS1133 ------------------------------------------------------------

CN 120030 GACACTTTTGCGACGGACTAACGATGACGAATACAGTTGACGTAAAAGCGTCGCAAATTT

CO46 NCBI ------------------------------------------------------------

DH55 ref genome ------------------------------------------------------------

Hoga ------------------------------------------------------------

CAM 236 ------------------------------------------------------------

09-CS0040 ------------------------------------------------------------

CN 119205 ------------------------------------------------------------

Yellowstone ------------------------------------------------------------

CN 120017 ------------------------------------------------------------

CN 119300 ------------------------------------------------------------

Jasper ------------------------------------------------------------

CN 120027 ------------------------------------------------------------

CN 113754 ------------------------------------------------------------

Joelle AAFC ------------------------------------------------------------

Joelle NCBI ------------------------------------------------------------

Joelle phyto ------------------------------------------------------------

Blaine Creek GACACTTTTGCGACGGACTAACGATGACGAATACAGTTGACGTAAAAGCGTCGCAAATTT

CAM 241 GACACTTTTGCGACGGACTAACGATGACGAATACAGTTGACGTAAAAGCGTCGCAAATTT

CN 119294 ------------------------------------------------------------

CN 120025 ------------------------------------------------------------

CN 120013 ------------------------------------------------------------

CS17CS1133 ------------------------------------------------------------

CN 120030 ACGACTACTAATTGTAGTCGCTAGCTCGTTGCTATTCTGCGACTACTTTGGGACATTTTA

CO46 NCBI ------------------------------------------------------------

DH55 ref genome ------------------------------------------------------------

Hoga ------------------------------------------------------------

CAM 236 ------------------------------------------------------------

09-CS0040 ------------------------------------------------------------

CN 119205 ------------------------------------------------------------

Yellowstone ------------------------------------------------------------

CN 120017 ------------------------------------------------------------

CN 119300 ------------------------------------------------------------

Jasper ------------------------------------------------------------

CN 120027 ------------------------------------------------------------

CN 113754 ------------------------------------------------------------

Joelle AAFC ------------------------------------------------------------

Joelle NCBI ------------------------------------------------------------

Joelle phyto ------------------------------------------------------------

Blaine Creek ACGACTACTAATTGTAGTCGCTAGCTCGTTGCTATTCTGCGACTACTTTGGGACATTTTA

CAM 241 ACGACTACTAATTGTAGTCGCTAGCTCGTTGCTATTCTGCGACTACTTTGGGACATTTTA

CN 119294 ------------------------------------------------------------

CN 120025 ------------------------------------------------------------

CN 120013 ------------------------------------------------------------

CS17CS1133 ------------------------------------------------------------

CN 120030 TCGTGAAAAAATAGCTCGTCGCAAAATAATCGCAATTTATGACAAACCATAAACTGAAGA

CO46 NCBI ------------------------------------------------------------

DH55 ref genome ------------------------------------------------------------

Hoga ------------------------------------------------------------

CAM 236 ------------------------------------------------------------

09-CS0040 ------------------------------------------------------------

CN 119205 ------------------------------------------------------------

Yellowstone ------------------------------------------------------------

CN 120017 ------------------------------------------------------------

CN 119300 ------------------------------------------------------------

Jasper ------------------------------------------------------------

CN 120027 ------------------------------------------------------------

CN 113754 ------------------------------------------------------------

Joelle AAFC ------------------------------------------------------------

Joelle NCBI ------------------------------------------------------------

Joelle phyto ------------------------------------------------------------

Blaine Creek TCGTGAAAAAATAGCTCGTCGCAAAATAATCGCAATTTATGACAAACCATAAACTGAAGA

CAM 241 TCGTGAAAAAATAGCTCGTCGCAAAATAATCGCAATTTATGACAAACCATAAACTGAAGA

CN 119294 ------------------------------------------------------------

CN 120025 ------------------------------------------------------------

CN 120013 ------------------------------------------------------------

CS17CS1133 ------------------------------------------------------------

CN 120030 TTAAGTCAGTAAAGTTAGTCGCAAAACTGTCTTCAAATCACGACGCTTTTGCCACTATTG

CO46 NCBI ------------------------------------------------------------

DH55 ref genome ------------------------------------------------------------

Hoga ------------------------------------------------------------

CAM 236 ------------------------------------------------------------

09-CS0040 ------------------------------------------------------------

CN 119205 ------------------------------------------------------------

Yellowstone ------------------------------------------------------------

CN 120017 ------------------------------------------------------------

CN 119300 ------------------------------------------------------------

Jasper ------------------------------------------------------------

CN 120027 ------------------------------------------------------------

CN 113754 ------------------------------------------------------------

Joelle AAFC ------------------------------------------------------------

Joelle NCBI ------------------------------------------------------------

Joelle phyto ------------------------------------------------------------

Blaine Creek TTAAGTCAGTAAAGTTAGTCGCAAAACTGTCTTCAAATCACGACGTTTTTGCCACTATTG

CAM 241 TTAAGTCAGTAAAGTTAGTCGCAAAACTGTCTTCAAATCACGACGTTTTTGCCACTATTG

CN 119294 ------------------------------------------------------------

CN 120025 ------------------------------------------------------------

CN 120013 ------------------------------------------------------------

CS17CS1133 ------------------------------------------------------------

CN 120030 TTTTGCTTTTGAATTTTATAGTCACAAATCAGTCGGTGACTATGTCGGTAAAAAACGTGC

CO46 NCBI ------------------------------------------------------------

DH55 ref genome ------------------------------------------------------------

Hoga ------------------------------------------------------------

CAM 236 ------------------------------------------------------------

09-CS0040 ------------------------------------------------------------

CN 119205 ------------------------------------------------------------

Yellowstone ------------------------------------------------------------

CN 120017 ------------------------------------------------------------

CN 119300 ------------------------------------------------------------

Jasper ------------------------------------------------------------

CN 120027 ------------------------------------------------------------

CN 113754 ------------------------------------------------------------

Joelle AAFC ------------------------------------------------------------

Joelle NCBI ------------------------------------------------------------

Joelle phyto ------------------------------------------------------------

Blaine Creek TTTTGCTTTTGAATTTTATAGTCACAAATCAGTCGGTGACTATGTCGGTAAAAAACGTGC

CAM 241 TTTTGCTTTTGAATTTTATAGTCACAAATCAGTCGGTGACTATGTCGGTAAAAAACGTGC

CN 119294 ------------------------------------------------------------

CN 120025 ------------------------------------------------------------

CN 120013 ------------------------------------------------------------

CS17CS1133 ------------------------------------------------------------

CN 120030 TAAATGTGGTAGTGTGAGTTTGTATTGAACTTTTGTGACCCTTTTATGAGTCTCAATTAC

CO46 NCBI ------------------------------------------------------------

DH55 ref genome ------------------------------------------------------------

Hoga ------------------------------------------------------------

CAM 236 ------------------------------------------------------------

09-CS0040 ------------------------------------------------------------

CN 119205 ------------------------------------------------------------

Yellowstone ------------------------------------------------------------

CN 120017 ------------------------------------------------------------

CN 119300 ------------------------------------------------------------

Jasper ------------------------------------------------------------

CN 120027 ------------------------------------------------------------

CN 113754 ------------------------------------------------------------

Joelle AAFC ------------------------------------------------------------

Joelle NCBI ------------------------------------------------------------

Joelle phyto ------------------------------------------------------------

Blaine Creek TAAATGTGGTAGTGTGAGTTTGTATTGAACTTTTGTGACCCTTTTATGAGTCTCAATTAC

CAM 241 TAAATGTGGTAGTGTGAGTTTGTATTGAACTTTTGTGACCCTTTTATGAGTCTCAATTAC

CN 119294 ------------------------------------------------------------

CN 120025 ------------------------------------------------------------

CN 120013 ------------------------------------------------------------

CS17CS1133 ------------------------------------------------------------

CN 120030 GTGGTAAAACAGTGACAAATGCGTCGTTATTTGCGACACACTCTTTCTTTGTTGCAAAAG

CO46 NCBI ------------------------------------------------------------

DH55 ref genome ------------------------------------------------------------

Hoga ------------------------------------------------------------

CAM 236 ------------------------------------------------------------

09-CS0040 ------------------------------------------------------------

CN 119205 ------------------------------------------------------------

Yellowstone ------------------------------------------------------------

CN 120017 ------------------------------------------------------------

CN 119300 ------------------------------------------------------------

Jasper ------------------------------------------------------------

CN 120027 ------------------------------------------------------------

CN 113754 ------------------------------------------------------------

Joelle AAFC ------------------------------------------------------------

Joelle NCBI ------------------------------------------------------------

Joelle phyto ------------------------------------------------------------

Blaine Creek GTGGTAAAACAGTGACAAATGCGTCGTTATTTGCGACACACTCTTTCTTTGTTGCAAAAG

CAM 241 GTGGTAAAACAGTGACAAATGCGTCGTTATTTGCGACACACTCTTTCTTTGTTGCAAAAG

CN 119294 ------------------------------------------------------------

CN 120025 ------------------------------------------------------------

CN 120013 ------------------------------------------------------------

CS17CS1133 ------------------------------------------------------------

CN 120030 AATCGCAAAGCAGTCACTTATTGATGTCGGCAAAAACGTGATAAATGTTTCAGTTTGCGT

CO46 NCBI ------------------------------------------------------------

DH55 ref genome ------------------------------------------------------------

Hoga ------------------------------------------------------------

CAM 236 ------------------------------------------------------------

09-CS0040 ------------------------------------------------------------

CN 119205 ------------------------------------------------------------

Yellowstone ------------------------------------------------------------

CN 120017 ------------------------------------------------------------

CN 119300 ------------------------------------------------------------

Jasper ------------------------------------------------------------

CN 120027 ------------------------------------------------------------

CN 113754 ------------------------------------------------------------

Joelle AAFC ------------------------------------------------------------

Joelle NCBI ------------------------------------------------------------

Joelle phyto ------------------------------------------------------------

Blaine Creek AATCGCAAAGCAGTCACTTATTGATGTCGGCAAAAAACGTGATAAATGTTTCAGTTTGCG

CAM 241 AATCGC-AAGCAGTCACTTATTGATGTCGGC-AAAAACGTGATAAATGTTTCAGTTTGCG

CN 119294 ------------------------------------------------------------

CN 120025 ------------------------------------------------------------

CN 120013 ------------------------------------------------------------

CS17CS1133 ------------------------------------------------------------

CN 120030 TTGTACTTGAGCTGTTTGGAGCTTTAATTTGTCTCAATTATGTGGTTTTTAGTGACAAAG

CO46 NCBI ------------------------------------------------------------

DH55 ref genome ------------------------------------------------------------

Hoga ------------------------------------------------------------

CAM 236 ------------------------------------------------------------

09-CS0040 ------------------------------------------------------------

CN 119205 ------------------------------------------------------------

Yellowstone ------------------------------------------------------------

CN 120017 ------------------------------------------------------------

CN 119300 ------------------------------------------------------------

Jasper ------------------------------------------------------------

CN 120027 ------------------------------------------------------------

CN 113754 ------------------------------------------------------------

Joelle AAFC ------------------------------------------------------------

Joelle NCBI ------------------------------------------------------------

Joelle phyto ------------------------------------------------------------

Blaine Creek TTTGTACTGAGCTGTTTGGAGCTTTAATTTGTCTCAATTATGTGGTTTTTAGTGACAAAG

CAM 241 TTTGTACTGAGCTGTTTGGAGCTTTAATTTGTCTCAATTATGTGGTTTTTAGTGACAAAG

CN 119294 ------------------------------------------------------------

CN 120025 ------------------------------------------------------------

CN 120013 ------------------------------------------------------------

CS17CS1133 ------------------------------------------------------------

CN 120030 GAGTCCTTATTTGCGATCTGTTTTGCCTAGAGTCGCAAAGTAGTCGCAAAATTTATACTA

CO46 NCBI ------------------------------------------------------------

DH55 ref genome ------------------------------------------------------------

Hoga ------------------------------------------------------------

CAM 236 ------------------------------------------------------------

09-CS0040 ------------------------------------------------------------

CN 119205 ------------------------------------------------------------

Yellowstone ------------------------------------------------------------

CN 120017 ------------------------------------------------------------

CN 119300 ------------------------------------------------------------

Jasper ------------------------------------------------------------

CN 120027 ------------------------------------------------------------

CN 113754 ------------------------------------------------------------

Joelle AAFC ------------------------------------------------------------

Joelle NCBI ------------------------------------------------------------

Joelle phyto ------------------------------------------------------------

Blaine Creek GAGTCCTTATTTGCGA-CTGTTTTGCCTAGAGTCGCAAAGTAGTCGCAAAATTTATACTA

CAM 241 GAGTCCTTATTTGCGATCTGTTTTGCCTAGAGTCGCAAAGTAGTCGCAAAATTTATACTA

CN 119294 ------------------------------------------------------------

CN 120025 ------------------------------------------------------------

CN 120013 ------------------------------------------------------------

CS17CS1133 ------------------------------------------------------------

CN 120030 TATATAACACATCATTCTCACCATTCCATTCATATCAATCTCATTCATTTCTAACTAGAG

CO46 NCBI ------------------------------------------------------------

DH55 ref genome ------------------------------------------------------------

Hoga ------------------------------------------------------------

CAM 236 ------------------------------------------------------------

09-CS0040 ------------------------------------------------------------

CN 119205 ------------------------------------------------------------

Yellowstone ------------------------------------------------------------

CN 120017 ------------------------------------------------------------

CN 119300 ------------------------------------------------------------

Jasper ------------------------------------------------------------

CN 120027 ------------------------------------------------------------

CN 113754 ------------------------------------------------------------

Joelle AAFC ------------------------------------------------------------

Joelle NCBI ------------------------------------------------------------

Joelle phyto ------------------------------------------------------------

Blaine Creek TATATAACACATCATTCTCACCATTCCATTCATATCAATCTCATTCATTTCTAACTAGAG

CAM 241 TATATAACACATCATTCTCACCATTCCATTCATATCAATCTCATTCATTTCTAACTAGAG

CN 119294 ------------------------------------------------------------

CN 120025 ------------------------------------------------------------

CN 120013 ------------------------------------------------------------

CS17CS1133 ------------------------------------------------------------

CN 120030 AGACGGAATAAAAAAAAATTAGTGAAAAAAAGATTTTTTTTTTTTTAGAAGAGAAGAAAA

CO46 NCBI ------------------------------------------------------------

DH55 ref genome ------------------------------------------------------------

Hoga ------------------------------------------------------------

CAM 236 ------------------------------------------------------------

09-CS0040 ------------------------------------------------------------

CN 119205 ------------------------------------------------------------

Yellowstone ------------------------------------------------------------

CN 120017 ------------------------------------------------------------

CN 119300 ------------------------------------------------------------

Jasper ------------------------------------------------------------

CN 120027 ------------------------------------------------------------

CN 113754 ------------------------------------------------------------

Joelle AAFC ------------------------------------------------------------

Joelle NCBI ------------------------------------------------------------

Joelle phyto ------------------------------------------------------------

Blaine Creek AGACGGAATAAAAAAAAATTAGTGAAAAAAAGATTTTTTTTTTTTT-TAGAAGAAAAAAA

CAM 241 AGACGGAAT--AAAAAAATTAGTGAAAAAAGA--TTTTTTTTTTTA-GAAGAGAAGAAAA

CN 119294 ------------------------------------------------------------

CN 120025 ------------------------------------------------------------

CN 120013 ------------------------------------------------------------

CS17CS1133 ------------------------------------------------------------

CN 120030 AAAAAGAGAGAAAGAAGATTTGTTGTTTTAGAAGAAAAGAAAAAAAAAAGCTATGTGGAA

CO46 NCBI ------------------------------------------------------------

DH55 ref genome ------------------------------------------------------------

Hoga ------------------------------------------------------------

CAM 236 ------------------------------------------------------------

09-CS0040 ------------------------------------------------------------

CN 119205 ------------------------------------------------------------

Yellowstone ------------------------------------------------------------

CN 120017 ------------------------------------------------------------

CN 119300 ------------------------------------------------------------

Jasper ------------------------------------------------------------

CN 120027 ------------------------------------------------------------

CN 113754 ------------------------------------------------------------

Joelle AAFC ------------------------------------------------------------

Joelle NCBI ------------------------------------------------------------

Joelle phyto ------------------------------------------------------------

Blaine Creek AAAAAGAGAGAAAGAAGATTTGTTGTTTTAGAAGAAAAGAAAAAAAAAAGCTATGTGGAA

CAM 241 AAAAAGAGAGAAAGAAGATTTGTTGTTTTAGAAGAAAAG--AAAAAAAAGCTATGTGGAA

CN 119294 ------------------------------------------------------------

CN 120025 ------------------------------------------------------------

CN 120013 ------------------------------------------------------------

CS17CS1133 ------------------------------------------------------------

CN 120030 TTCATCTCGAGAGTGGATGTATAATCGGATCGATGGAAGAACAAATAATATCTCTAAAGA

CO46 NCBI ------------------------------------------------------------

DH55 ref genome ------------------------------------------------------------

Hoga ------------------------------------------------------------

CAM 236 ------------------------------------------------------------

09-CS0040 ------------------------------------------------------------

CN 119205 ------------------------------------------------------------

Yellowstone ------------------------------------------------------------

CN 120017 ------------------------------------------------------------

CN 119300 ------------------------------------------------------------

Jasper ------------------------------------------------------------

CN 120027 ------------------------------------------------------------

CN 113754 ------------------------------------------------------------

Joelle AAFC ------------------------------------------------------------

Joelle NCBI ------------------------------------------------------------

Joelle phyto ------------------------------------------------------------

Blaine Creek TTCATCTCGAGAGTGGATGTATAATCGGATCGATGGAAGAACAAATAATATCTCTAAAGA

CAM 241 TTCATCTCGAGAGTGGATGTATAATCGGATCGATGGAAGAACAAATAATATCTCTAAAGA

CN 119294 ------------------------------------------------------------

CN 120025 ------------------------------------------------------------

CN 120013 ------------------------------------------------------------

CS17CS1133 ------------------------------------------------------------

CN 120030 ATTTTTGGCGGGAGTTGAGCAGTTCATGAATTTCGCAAACAACCAGCCTATGGCACAAAA

CO46 NCBI ------------------------------------------------------------

DH55 ref genome ------------------------------------------------------------

Hoga ------------------------------------------------------------

CAM 236 ------------------------------------------------------------

09-CS0040 ------------------------------------------------------------

CN 119205 ------------------------------------------------------------

Yellowstone ------------------------------------------------------------

CN 120017 ------------------------------------------------------------

CN 119300 ------------------------------------------------------------

Jasper ------------------------------------------------------------

CN 120027 ------------------------------------------------------------

CN 113754 ------------------------------------------------------------

Joelle AAFC ------------------------------------------------------------

Joelle NCBI ------------------------------------------------------------

Joelle phyto ------------------------------------------------------------

Blaine Creek ATTTTTGGCGGGAGTTGAGCAGTTCATGAATTTCGCAAACAACCAGCCTATGGCACAAAA

CAM 241 ATTTTTGGCGGGAGTTGAGCAGTTCATGAATTTCGCAAACAACCAGCCTATGGCACAAAA

CN 119294 ------------------------------------------------------------

CN 120025 ------------------------------------------------------------

CN 120013 ------------------------------------------------------------

CS17CS1133 ------------------------------------------------------------

CN 120030 CAGTGGAGGTAGGTTTTACTGTCCTTGCGTAAAATGTC-GAACGATGTTATTTTGCATGG

CO46 NCBI ------------------------------------------------------------

DH55 ref genome ------------------------------------------------------------

Hoga ------------------------------------------------------------

CAM 236 ------------------------------------------------------------

09-CS0040 ------------------------------------------------------------

CN 119205 ------------------------------------------------------------

Yellowstone ------------------------------------------------------------

CN 120017 ------------------------------------------------------------

CN 119300 ------------------------------------------------------------

Jasper ------------------------------------------------------------

CN 120027 ------------------------------------------------------------

CN 113754 ------------------------------------------------------------

Joelle AAFC ------------------------------------------------------------

Joelle NCBI ------------------------------------------------------------

Joelle phyto ------------------------------------------------------------

Blaine Creek CAGTGGAGGTAGGTTTTACTGTCCTTGCGTAAAATGTCAGAACGATGTTATTTTGCATGG

CAM 241 CAGTGGAGGTAGGTTTTACTGTCCTTGCGTAAAATGTC-GAACGATGTTATTTTGCATGG

CN 119294 ------------------------------------------------------------

CN 120025 ------------------------------------------------------------

CN 120013 ------------------------------------------------------------

CS17CS1133 ------------------------------------------------------------

CN 120030 TACGACAATATCTAATCATTTGCATAGTAAAGGATTTATGCCAAATTATTATGTATGGTC

CO46 NCBI ------------------------------------------------------------

DH55 ref genome ------------------------------------------------------------

Hoga ------------------------------------------------------------

CAM 236 ------------------------------------------------------------

09-CS0040 ------------------------------------------------------------

CN 119205 ------------------------------------------------------------

Yellowstone ------------------------------------------------------------

CN 120017 ------------------------------------------------------------

CN 119300 ------------------------------------------------------------

Jasper ------------------------------------------------------------

CN 120027 ------------------------------------------------------------

CN 113754 ------------------------------------------------------------

Joelle AAFC ------------------------------------------------------------

Joelle NCBI ------------------------------------------------------------

Joelle phyto ------------------------------------------------------------

Blaine Creek TACGACAATATCTAATCATTTGCATAGTAAAGGATTTATGCCAAATTATTATGTATGGTC

CAM 241 TACGACAATATCTAATCATTTGCATAGTAAAGGATTTATGCCAAATTATTATGTATGGTC

CN 119294 ------------------------------------------------------------

CN 120025 ------------------------------------------------------------

CN 120013 ------------------------------------------------------------

CS17CS1133 ------------------------------------------------------------

CN 120030 TGAACATGGTGAAGATTATGACGTGTTAGGGGTAGGAACTAGTAGTCATTATCCTAATAC

CO46 NCBI ------------------------------------------------------------

DH55 ref genome ------------------------------------------------------------

Hoga ------------------------------------------------------------

CAM 236 ------------------------------------------------------------

09-CS0040 ------------------------------------------------------------

CN 119205 ------------------------------------------------------------

Yellowstone ------------------------------------------------------------

CN 120017 ------------------------------------------------------------

CN 119300 ------------------------------------------------------------

Jasper ------------------------------------------------------------

CN 120027 ------------------------------------------------------------

CN 113754 ------------------------------------------------------------

Joelle AAFC ------------------------------------------------------------

Joelle NCBI ------------------------------------------------------------

Joelle phyto ------------------------------------------------------------

Blaine Creek TGAACATGGTGAAGATTATGACGTGTTAGGGGTAGGAACTAGTAGTCATTATCCTAATAC

CAM 241 TGAACATGGTGAAGATTATGACGTGTTAGGGGTAGGAACTAGTAGTCATTATCCTAATAC

CN 119294 ------------------------------------------------------------

CN 120025 ------------------------------------------------------------

CN 120013 ------------------------------------------------------------

CS17CS1133 ------------------------------------------------------------

CN 120030 AAATTATACTAGTACTTAGTAGTCAGCATGGTAGTCAACCCGGTAGTCGACCAATTGGAT

CO46 NCBI ------------------------------------------------------------

DH55 ref genome ------------------------------------------------------------

Hoga ------------------------------------------------------------

CAM 236 ------------------------------------------------------------

09-CS0040 ------------------------------------------------------------

CN 119205 ------------------------------------------------------------

Yellowstone ------------------------------------------------------------

CN 120017 ------------------------------------------------------------

CN 119300 ------------------------------------------------------------

Jasper ------------------------------------------------------------

CN 120027 ------------------------------------------------------------

CN 113754 ------------------------------------------------------------

Joelle AAFC ------------------------------------------------------------

Joelle NCBI ------------------------------------------------------------

Joelle phyto ------------------------------------------------------------

Blaine Creek AAATTATACTAGTAC-TAGTAGTCAGCATGGTAGTCAACCCGGTAGTCAGCCAATTGGAT

CAM 241 AAATTATACTAGTAC-TAGTAGTCAGCATGGTAGTCAACCTGGTAGTCAGCCAATTGGAT

CN 119294 ------------------------------------------------------------

CN 120025 ------------------------------------------------------------

CN 120013 ------------------------------------------------------------

CS17CS1133 ------------------------------------------------------------

CN 120030 TTGAGGGAAATGTATATGCTGAGATGGTGAATGATGCATTTCATGGTACCACGCCTTTTA

CO46 NCBI ------------------------------------------------------------

DH55 ref genome ------------------------------------------------------------

Hoga ------------------------------------------------------------

CAM 236 ------------------------------------------------------------

09-CS0040 ------------------------------------------------------------

CN 119205 ------------------------------------------------------------

Yellowstone ------------------------------------------------------------

CN 120017 ------------------------------------------------------------

CN 119300 ------------------------------------------------------------

Jasper ------------------------------------------------------------

CN 120027 ------------------------------------------------------------

CN 113754 ------------------------------------------------------------

Joelle AAFC ------------------------------------------------------------

Joelle NCBI ------------------------------------------------------------

Joelle phyto ------------------------------------------------------------

Blaine Creek TTGAGGGAAATGTATATGCTGAGATGGTGAATGATGCATTTCATGGTACCACGCCTTTTA

CAM 241 TTGAGGGAAATGTATATGCTGAGATGGTGAATGATGCATTTCATGGTACCACGCCTTTTA

CN 119294 ------------------------------------------------------------

CN 120025 ------------------------------------------------------------

CN 120013 ------------------------------------------------------------

CS17CS1133 ------------------------------------------------------------

CN 120030 ATGAGTATCATGAGTATGAAAGTGGATATGATCATGGATATGATCATATCCATGAAGAAC

CO46 NCBI ------------------------------------------------------------

DH55 ref genome ------------------------------------------------------------

Hoga ------------------------------------------------------------

CAM 236 ------------------------------------------------------------

09-CS0040 ------------------------------------------------------------

CN 119205 ------------------------------------------------------------

Yellowstone ------------------------------------------------------------

CN 120017 ------------------------------------------------------------

CN 119300 ------------------------------------------------------------

Jasper ------------------------------------------------------------

CN 120027 ------------------------------------------------------------

CN 113754 ------------------------------------------------------------

Joelle AAFC ------------------------------------------------------------

Joelle NCBI ------------------------------------------------------------

Joelle phyto ------------------------------------------------------------

Blaine Creek ATGAGTATCATGAGTATGAAAGTGGATATGATCATGGATATGATCATATCCATGAAGAAC

CAM 241 ATGAGTATCATGAGTATGAAAGTGGATATGATCATGGATATGATCATATCCATGAAGAAC

CN 119294 ------------------------------------------------------------

CN 120025 ------------------------------------------------------------

CN 120013 ------------------------------------------------------------

CS17CS1133 ------------------------------------------------------------

CN 120030 CCACTGAAGAGGCGAAACGGTTCTACGACATGTTAGATGCTGCAAATACTCCACTTTATG

CO46 NCBI ------------------------------------------------------------

DH55 ref genome ------------------------------------------------------------

Hoga ------------------------------------------------------------

CAM 236 ------------------------------------------------------------

09-CS0040 ------------------------------------------------------------

CN 119205 ------------------------------------------------------------

Yellowstone ------------------------------------------------------------

CN 120017 ------------------------------------------------------------

CN 119300 ------------------------------------------------------------

Jasper ------------------------------------------------------------

CN 120027 ------------------------------------------------------------

CN 113754 ------------------------------------------------------------

Joelle AAFC ------------------------------------------------------------

Joelle NCBI ------------------------------------------------------------

Joelle phyto ------------------------------------------------------------

Blaine Creek CCACTGAAGAGGCGAAACGGTTCTACGACATGTTAGATGCTGCAAATACTCCACTTTATG

CAM 241 CCATCGAAGAGGCGAAACGGTTCTACGACATGTTAGATGCTGCAAATACTCCACTTTATG

CN 119294 ------------------------------------------------------------

CN 120025 ------------------------------------------------------------

CN 120013 ------------------------------------------------------------

CS17CS1133 ------------------------------------------------------------

CN 120030 ATGGATGTCATGAAGGTCATTCGCAACTATCATTGGCGTCTAGGTTCATGAACATCAAGG

CO46 NCBI ------------------------------------------------------------

DH55 ref genome ------------------------------------------------------------

Hoga ------------------------------------------------------------

CAM 236 ------------------------------------------------------------

09-CS0040 ------------------------------------------------------------

CN 119205 ------------------------------------------------------------

Yellowstone ------------------------------------------------------------

CN 120017 ------------------------------------------------------------

CN 119300 ------------------------------------------------------------

Jasper ------------------------------------------------------------

CN 120027 ------------------------------------------------------------

CN 113754 ------------------------------------------------------------

Joelle AAFC ------------------------------------------------------------

Joelle NCBI ------------------------------------------------------------

Joelle phyto ------------------------------------------------------------

Blaine Creek ATGGATGTCATGAAGGTCATTCGCAACTATCATTGGCGTCTAGGTTCATGAACATCAAGG

CAM 241 ATGGATGTCATGAAGGTCATTCGCAACTATCATTGGCGTCTAGGTTCATGAACATCAAGG

CN 119294 ------------------------------------------------------------

CN 120025 ------------------------------------------------------------

CN 120013 ------------------------------------------------------------

CS17CS1133 ------------------------------------------------------------

CN 120030 TTGATAATAATTTGTC-CGAGGCATGCATGGACGATTGGGCTGAATTGTTTAC-GGAGGT

CO46 NCBI ------------------------------------------------------------

DH55 ref genome ------------------------------------------------------------

Hoga ------------------------------------------------------------

CAM 236 ------------------------------------------------------------

09-CS0040 ------------------------------------------------------------

CN 119205 ------------------------------------------------------------

Yellowstone ------------------------------------------------------------

CN 120017 ------------------------------------------------------------

CN 119300 ------------------------------------------------------------

Jasper ------------------------------------------------------------

CN 120027 ------------------------------------------------------------

CN 113754 ------------------------------------------------------------

Joelle AAFC ------------------------------------------------------------

Joelle NCBI ------------------------------------------------------------

Joelle phyto ------------------------------------------------------------

Blaine Creek TTGATAATAATTTGTCTCGAGGCATGCATGGACGATTGGGCTGAATTGTTTACTGGAGGT

CAM 241 TTGATAATAATTTGTC-CGAGGCATGCATGGACGATTGGGCTGAATTGTTTACTGGAGGT

CN 119294 ------------------------------------------------------------

CN 120025 ------------------------------------------------------------

CN 120013 ------------------------------------------------------------

CS17CS1133 ------------------------------------------------------------

CN 120030 TTTACCGGAGGGTAATCAAGCTACTGGTTCATACTACGAGACAGAGACTTTAGTTCGAAA

CO46 NCBI ------------------------------------------------------------

DH55 ref genome ------------------------------------------------------------

Hoga ------------------------------------------------------------

CAM 236 ------------------------------------------------------------

09-CS0040 ------------------------------------------------------------

CN 119205 ------------------------------------------------------------

Yellowstone ------------------------------------------------------------

CN 120017 ------------------------------------------------------------

CN 119300 ------------------------------------------------------------

Jasper ------------------------------------------------------------

CN 120027 ------------------------------------------------------------

CN 113754 ------------------------------------------------------------

Joelle AAFC ------------------------------------------------------------

Joelle NCBI ------------------------------------------------------------

Joelle phyto ------------------------------------------------------------

Blaine Creek TTTACCGGAGGGTAATCAAGCTACTGGTTCATACTACGAGACAGAGACTTTAGTTCGAAA

CAM 241 TTTACCGGAGGGTAATCAAGCTATCGGTTCATACTACGAGAC-GAGACTTTAGTTCGAAA

CN 119294 ------------------------------------------------------------

CN 120025 ------------------------------------------------------------

CN 120013 ------------------------------------------------------------

CS17CS1133 ------------------------------------------------------------

CN 120030 GATAGGATTGCCATACCATACAATTGATGTATGTATAGAGAATTGTATGATATTTTGGAA

CO46 NCBI ------------------------------------------------------------

DH55 ref genome ------------------------------------------------------------

Hoga ------------------------------------------------------------

CAM 236 ------------------------------------------------------------

09-CS0040 ------------------------------------------------------------

CN 119205 ------------------------------------------------------------

Yellowstone ------------------------------------------------------------

CN 120017 ------------------------------------------------------------

CN 119300 ------------------------------------------------------------

Jasper ------------------------------------------------------------

CN 120027 ------------------------------------------------------------

CN 113754 ------------------------------------------------------------

Joelle AAFC ------------------------------------------------------------

Joelle NCBI ------------------------------------------------------------

Joelle phyto ------------------------------------------------------------

Blaine Creek GATAGGATTGCCATACCATACAATTGATGTATGTATAGAGAATTGTATGATATTTTGGAA

CAM 241 GATAGGATTGCCATACCATACAATTGATGTATGTATAGAGAATTGTATGATATTTTGGAA

CN 119294 ------------------------------------------------------------

CN 120025 ------------------------------------------------------------

CN 120013 ------------------------------------------------------------

CS17CS1133 ------------------------------------------------------------

CN 120030 AGAAGATGGGAATTTGGAGCATTGCAAGTTCTGTGGGAAGCCAAGGTACAAAAGTAGTGG

CO46 NCBI ------------------------------------------------------------

DH55 ref genome ------------------------------------------------------------

Hoga ------------------------------------------------------------

CAM 236 ------------------------------------------------------------

09-CS0040 ------------------------------------------------------------

CN 119205 ------------------------------------------------------------

Yellowstone ------------------------------------------------------------

CN 120017 ------------------------------------------------------------

CN 119300 ------------------------------------------------------------

Jasper ------------------------------------------------------------

CN 120027 ------------------------------------------------------------

CN 113754 ------------------------------------------------------------

Joelle AAFC ------------------------------------------------------------

Joelle NCBI ------------------------------------------------------------

Joelle phyto ------------------------------------------------------------

Blaine Creek AGAAGATGGGAATTTGGAGCATTGCAAGTTCTGTGGGAAGCCAAGGTACAAAAGTAGTGG

CAM 241 AGAAGATGGGAATTTGGAGCATTGCAAGTTCTGTGGGAAGCCAAGGTACAAAAGTAGT-G

CN 119294 ------------------------------------------------------------

CN 120025 ------------------------------------------------------------

CN 120013 ------------------------------------------------------------

CS17CS1133 ------------------------------------------------------------

CN 120030 GGGTAGAACTAGAATACCCTTCGATCGTATGTGGTATCTACCTATTGCAGATAGATTGAA

CO46 NCBI ------------------------------------------------------------

DH55 ref genome ------------------------------------------------------------

Hoga ------------------------------------------------------------

CAM 236 ------------------------------------------------------------

09-CS0040 ------------------------------------------------------------

CN 119205 ------------------------------------------------------------

Yellowstone ------------------------------------------------------------

CN 120017 ------------------------------------------------------------

CN 119300 ------------------------------------------------------------

Jasper ------------------------------------------------------------

CN 120027 ------------------------------------------------------------

CN 113754 ------------------------------------------------------------

Joelle AAFC ------------------------------------------------------------

Joelle NCBI ------------------------------------------------------------

Joelle phyto ------------------------------------------------------------

Blaine Creek GGGTAGAACTAGAATACCCTTCAGTCGTATGTGGTATCTACCTATTGCAGATAGATTGAA

CAM 241 GGGTAGAACTAGAATACCCTTCGATCGTATGTGGTATCTACCTATTGCAGATAGATTGAA

CN 119294 ------------------------------------------------------------

CN 120025 ------------------------------------------------------------

CN 120013 ------------------------------------------------------------

CS17CS1133 ------------------------------------------------------------

CN 120030 GAGGATGTACCAATCAGAGAAGACCGCATCATCAATGAGATGGCATGCTGAGCATGATTC

CO46 NCBI ------------------------------------------------------------

DH55 ref genome ------------------------------------------------------------

Hoga ------------------------------------------------------------

CAM 236 ------------------------------------------------------------

09-CS0040 ------------------------------------------------------------

CN 119205 ------------------------------------------------------------

Yellowstone ------------------------------------------------------------

CN 120017 ------------------------------------------------------------

CN 119300 ------------------------------------------------------------

Jasper ------------------------------------------------------------

CN 120027 ------------------------------------------------------------

CN 113754 ------------------------------------------------------------

Joelle AAFC ------------------------------------------------------------

Joelle NCBI ------------------------------------------------------------

Joelle phyto ------------------------------------------------------------

Blaine Creek GAGGATGTACCAATCAGAGAAGACCGCATCATCAATGAGATGGCATGCTGAGCATGATTC

CAM 241 GAGGATGTACCAATCGGAGAAGACCGCATCATCAATGAGATGGCATGCTGAGCATGATTC

CN 119294 ------------------------------------------------------------

CN 120025 ------------------------------------------------------------

CN 120013 ------------------------------------------------------------

CS17CS1133 ------------------------------------------------------------

CN 120030 AGAAGATGGAGTAATGTGTCATCCATCCGATGCGCTCGAATGGAAGAATTTCCAACATTT

CO46 NCBI ------------------------------------------------------------

DH55 ref genome ------------------------------------------------------------

Hoga ------------------------------------------------------------

CAM 236 ------------------------------------------------------------

09-CS0040 ------------------------------------------------------------

CN 119205 ------------------------------------------------------------

Yellowstone ------------------------------------------------------------

CN 120017 ------------------------------------------------------------

CN 119300 ------------------------------------------------------------

Jasper ------------------------------------------------------------

CN 120027 ------------------------------------------------------------

CN 113754 ------------------------------------------------------------

Joelle AAFC ------------------------------------------------------------

Joelle NCBI ------------------------------------------------------------

Joelle phyto ------------------------------------------------------------

Blaine Creek AGAAGATGGAGTAATGTGTCATCCATCTGATGCGCCTGAATGGAAGAATTTCCAACATTT

CAM 241 AGAAGATGGAGTAATGTGTCATCCATCCGATGCGCTCGAATGGAAGAATTTCCAACATTT

CN 119294 ------------------------------------------------------------

CN 120025 ------------------------------------------------------------

CN 120013 ------------------------------------------------------------

CS17CS1133 ------------------------------------------------------------

CN 120030 ACATCCCACATTTGCGC-AAGAGCCACGAAACGTTTACCTTGGGTTATGTACAGATGGTT

CO46 NCBI ------------------------------------------------------------

DH55 ref genome ------------------------------------------------------------

Hoga ------------------------------------------------------------

CAM 236 ------------------------------------------------------------

09-CS0040 ------------------------------------------------------------

CN 119205 ------------------------------------------------------------

Yellowstone ------------------------------------------------------------

CN 120017 ------------------------------------------------------------

CN 119300 ------------------------------------------------------------

Jasper ------------------------------------------------------------

CN 120027 ------------------------------------------------------------

CN 113754 ------------------------------------------------------------

Joelle AAFC ------------------------------------------------------------

Joelle NCBI ------------------------------------------------------------

Joelle phyto ------------------------------------------------------------

Blaine Creek ACATCCCACATTTGCGC-AAGAGCCACGAAACGTTTACCTTGGGTTATGTACAGATGGTT

CAM 241 ACATCCCACATTTGCGCAAAGAGCCACGAAACGTTTACCTTGGGTTATGTACAGATGGTT

CN 119294 ------------------------------------------------------------

CN 120025 ------------------------------------------------------------

CN 120013 ------------------------------------------------------------

CS17CS1133 ------------------------------------------------------------

CN 120030 TTAATCCATTTGGGGTCTCCAAAAATCATTCTTTGTGGC-CTGTGATCTTAACTCCATAC

CO46 NCBI ------------------------------------------------------------

DH55 ref genome ------------------------------------------------------------

Hoga ------------------------------------------------------------

CAM 236 ------------------------------------------------------------

09-CS0040 ------------------------------------------------------------

CN 119205 ------------------------------------------------------------

Yellowstone ------------------------------------------------------------

CN 120017 ------------------------------------------------------------

CN 119300 ------------------------------------------------------------

Jasper ------------------------------------------------------------

CN 120027 ------------------------------------------------------------

CN 113754 ------------------------------------------------------------

Joelle AAFC ------------------------------------------------------------

Joelle NCBI ------------------------------------------------------------

Joelle phyto ------------------------------------------------------------

Blaine Creek TTAATCCATTTGGGGTCTCC-AAAATCATTCTTTGTGGC-CTGTGATCTTAACTCCATAC

CAM 241 TTAATCCATTTGGGGTCTCCAAAAATCATTCTTTGTGGCTCTGTGATCTTAACTCCATAC

CN 119294 ------------------------------------------------------------

CN 120025 ------------------------------------------------------------

CN 120013 ------------------------------------------------------------

CS17CS1133 ------------------------------------------------------------

CN 120030 AACCTACCTCCG-GATATGTGCATGAACAGCGAGTATTTATTTCTTACGATTCTGAACTC

CO46 NCBI ------------------------------------------------------------

DH55 ref genome ------------------------------------------------------------

Hoga ------------------------------------------------------------

CAM 236 ------------------------------------------------------------

09-CS0040 ------------------------------------------------------------

CN 119205 ------------------------------------------------------------

Yellowstone ------------------------------------------------------------

CN 120017 ------------------------------------------------------------

CN 119300 ------------------------------------------------------------

Jasper ------------------------------------------------------------

CN 120027 ------------------------------------------------------------

CN 113754 ------------------------------------------------------------

Joelle AAFC ------------------------------------------------------------

Joelle NCBI ------------------------------------------------------------

Joelle phyto ------------------------------------------------------------

Blaine Creek AACCTACCTCCG-GATATGTGCATGAACAGCGAGTATTTATTTCTTACGATTCTGAACTC

CAM 241 AACCTACCTCCGAGATATGTGCATGAACAGCGAGTATTTATTTCTTACGATTC-GAACTC

CN 119294 ------------------------------------------------------------

CN 120025 ------------------------------------------------------------

CN 120013 ------------------------------------------------------------

CS17CS1133 ------------------------------------------------------------

CN 120030 CGGACCAAACCACCCACGAGCCAGCCTTGATGTTTTCCTCCAACCATTAATCGATGAGTT

CO46 NCBI ------------------------------------------------------------

DH55 ref genome ------------------------------------------------------------

Hoga ------------------------------------------------------------

CAM 236 ------------------------------------------------------------

09-CS0040 ------------------------------------------------------------

CN 119205 ------------------------------------------------------------

Yellowstone ------------------------------------------------------------

CN 120017 ------------------------------------------------------------

CN 119300 ------------------------------------------------------------

Jasper ------------------------------------------------------------

CN 120027 ------------------------------------------------------------

CN 113754 ------------------------------------------------------------

Joelle AAFC ------------------------------------------------------------

Joelle NCBI ------------------------------------------------------------

Joelle phyto ------------------------------------------------------------

Blaine Creek CGGACCAAACCACCCACGAGCCAGCCTTGATGTTTTCCTCCAACCATTAATCGATGAGTT

CAM 241 CGGACCAAACCACCCACGAGCCAGCCTTGATGTTTTCCTCCAACCATTAATCGATGAGTT

CN 119294 ------------------------------------------------------------

CN 120025 ------------------------------------------------------------

CN 120013 ------------------------------------------------------------

CS17CS1133 ------------------------------------------------------------

CN 120030 AAAGGAGTTATGGTATAATGGGGTTGAGGCTTATGATATCTCACTAAATCAAAATTTCAA

CO46 NCBI ------------------------------------------------------------

DH55 ref genome ------------------------------------------------------------

Hoga ------------------------------------------------------------

CAM 236 ------------------------------------------------------------

09-CS0040 ------------------------------------------------------------

CN 119205 ------------------------------------------------------------

Yellowstone ------------------------------------------------------------

CN 120017 ------------------------------------------------------------

CN 119300 ------------------------------------------------------------

Jasper ------------------------------------------------------------

CN 120027 ------------------------------------------------------------

CN 113754 ------------------------------------------------------------

Joelle AAFC ------------------------------------------------------------

Joelle NCBI ------------------------------------------------------------

Joelle phyto ------------------------------------------------------------

Blaine Creek AAAGGAGTTATGGTATAATGGGGTTGAGGCTTATGATATCTCACTAAATCAAAATTTCAA

CAM 241 AAAGGAGTTATGGTATAATGGGGTTGAGGCTTATGATATCTCACTAAATCAAAATTTCAA

CN 119294 ------------------------------------------------------------

CN 120025 ------------------------------------------------------------

CN 120013 ------------------------------------------------------------

CS17CS1133 ------------------------------------------------------------

CN 120030 CATGAAAGTTGTTCTTATGTGGACAATAAGCGATTTTCCAGC-GTACGGTATGTTGTCGG

CO46 NCBI ------------------------------------------------------------

DH55 ref genome ------------------------------------------------------------

Hoga ------------------------------------------------------------

CAM 236 ------------------------------------------------------------

09-CS0040 ------------------------------------------------------------

CN 119205 ------------------------------------------------------------

Yellowstone ------------------------------------------------------------

CN 120017 ------------------------------------------------------------

CN 119300 ------------------------------------------------------------

Jasper ------------------------------------------------------------

CN 120027 ------------------------------------------------------------

CN 113754 ------------------------------------------------------------

Joelle AAFC ------------------------------------------------------------

Joelle NCBI ------------------------------------------------------------

Joelle phyto ------------------------------------------------------------

Blaine Creek CATGAAAGTTGTTCTTATGTGGACAATAAGCGATTTTCCAGC-GTACGGTATGTTGTCGG

CAM 241 CATGAAAGTTGTTCTTATGTGGACAATAAGCGATTTTCCAGCAGTACGGTATGTTGTCGG

CN 119294 ------------------------------------------------------------

CN 120025 ------------------------------------------------------------

CN 120013 ------------------------------------------------------------

CS17CS1133 ------------------------------------------------------------

CN 120030 GATGGACGACGCATGGAAGATTAGCATGTCCAATTTGTATGGATGACACT-GGTGCTTTT

CO46 NCBI ------------------------------------------------------------

DH55 ref genome ------------------------------------------------------------

Hoga ------------------------------------------------------------

CAM 236 ------------------------------------------------------------

09-CS0040 ------------------------------------------------------------

CN 119205 ------------------------------------------------------------

Yellowstone ------------------------------------------------------------

CN 120017 ------------------------------------------------------------

CN 119300 ------------------------------------------------------------

Jasper ------------------------------------------------------------

CN 120027 ------------------------------------------------------------

CN 113754 ------------------------------------------------------------

Joelle AAFC ------------------------------------------------------------

Joelle NCBI ------------------------------------------------------------

Joelle phyto ------------------------------------------------------------

Blaine Creek GATGGACGACGCATGGAAGATTAGCATGTCCAATTTGTATGGATGACACT-GGTGCTTTT

CAM 241 GATGGACGACGCATGGAAGATTAGCATGTCCAATTTGTATGGATGACACTCGGTGCTTTT

CN 119294 ------------------------------------------------------------

CN 120025 ------------------------------------------------------------

CN 120013 ------------------------------------------------------------

CS17CS1133 ------------------------------------------------------------

CN 120030 CAATTACCAGCTGGGAGGAAAACATGTTGGTTTGA-CTGTCATAGGAGATTTCTTCCTAC

CO46 NCBI ------------------------------------------------------------

DH55 ref genome ------------------------------------------------------------

Hoga ------------------------------------------------------------

CAM 236 ------------------------------------------------------------

09-CS0040 ------------------------------------------------------------

CN 119205 ------------------------------------------------------------

Yellowstone ------------------------------------------------------------

CN 120017 ------------------------------------------------------------

CN 119300 ------------------------------------------------------------

Jasper ------------------------------------------------------------

CN 120027 ------------------------------------------------------------

CN 113754 ------------------------------------------------------------

Joelle AAFC ------------------------------------------------------------

Joelle NCBI ------------------------------------------------------------

Joelle phyto ------------------------------------------------------------

Blaine Creek CAATTACCAGCTGGGAGGAAAACATGTTGGTTTGA-CTGTCATAGGAGATTTCTTCCTAC

CAM 241 CAATTACCAGCTGGGAGGAAAACATGTTGGTTTGATCTGTCATAGGAGATTTCTTCCTAC

CN 119294 ------------------------------------------------------------

CN 120025 ------------------------------------------------------------

CN 120013 ------------------------------------------------------------

CS17CS1133 ------------------------------------------------------------

CN 120030 AAGTCATCCGATGCGGAAGAATAAAAATGACTTTCTCGAAGGGAAAAGATTCATTGAATG

CO46 NCBI ------------------------------------------------------------

DH55 ref genome ------------------------------------------------------------

Hoga ------------------------------------------------------------

CAM 236 ------------------------------------------------------------

09-CS0040 ------------------------------------------------------------

CN 119205 ------------------------------------------------------------

Yellowstone ------------------------------------------------------------

CN 120017 ------------------------------------------------------------

CN 119300 ------------------------------------------------------------

Jasper ------------------------------------------------------------

CN 120027 ------------------------------------------------------------

CN 113754 ------------------------------------------------------------

Joelle AAFC ------------------------------------------------------------

Joelle NCBI ------------------------------------------------------------

Joelle phyto ------------------------------------------------------------

Blaine Creek AAGTCATCCGATGCGGAAGAATAAAAATGACTTTCT-GAAGGGAAAAGATTCATTGAATG

CAM 241 AAGTCATCCGATGCGGAAGAATAAAAATGACTTTCC-GAAGGGAAAAGATTCATTGAATG

CN 119294 ------------------------------------------------------------

CN 120025 ------------------------------------------------------------

CN 120013 ------------------------------------------------------------

CS17CS1133 ------------------------------------------------------------

CN 120030 ACGAGCCACCAGCATCTCCGAGTAGTCAAGCTATCTATGAGCGTATAAGGAAAGCCAAAG

CO46 NCBI ------------------------------------------------------------

DH55 ref genome ------------------------------------------------------------

Hoga ------------------------------------------------------------

CAM 236 ------------------------------------------------------------

09-CS0040 ------------------------------------------------------------

CN 119205 ------------------------------------------------------------

Yellowstone ------------------------------------------------------------

CN 120017 ------------------------------------------------------------

CN 119300 ------------------------------------------------------------

Jasper ------------------------------------------------------------

CN 120027 ------------------------------------------------------------

CN 113754 ------------------------------------------------------------

Joelle AAFC ------------------------------------------------------------

Joelle NCBI ------------------------------------------------------------

Joelle phyto ------------------------------------------------------------

Blaine Creek ACGAGCCACCAGCATCTCTGAGTAGTCAAGCTATCTATGAGCGTATAAGGAAAGCCAAAG

CAM 241 ACGAGCCACCAGCATCTCCGAGTAGTCAAGCTATCTATGAGCGTATAAGGAAAGCCAAAG

CN 119294 ------------------------------------------------------------

CN 120025 ------------------------------------------------------------

CN 120013 ------------------------------------------------------------

CS17CS1133 ------------------------------------------------------------

CN 120030 CACCTAAAACATCTATTTGCGGTGGGAATGGTCACGAAAAGAAAGTTAAAGGCTACGGAA

CO46 NCBI ------------------------------------------------------------

DH55 ref genome ------------------------------------------------------------

Hoga ------------------------------------------------------------

CAM 236 ------------------------------------------------------------

09-CS0040 ------------------------------------------------------------

CN 119205 ------------------------------------------------------------

Yellowstone ------------------------------------------------------------

CN 120017 ------------------------------------------------------------

CN 119300 ------------------------------------------------------------

Jasper ------------------------------------------------------------

CN 120027 ------------------------------------------------------------

CN 113754 ------------------------------------------------------------

Joelle AAFC ------------------------------------------------------------

Joelle NCBI ------------------------------------------------------------

Joelle phyto ------------------------------------------------------------

Blaine Creek CACCTAAAACATCTATTTGCGGTGGGAATGGTCACGAAAAGAAAGTTAAAGGCTACGGAA

CAM 241 CACCTAAAACATCTATTTGCGGTGGGAATGGTCACGAAAAGAAAGTTAAAGGCTACGGAA

CN 119294 ------------------------------------------------------------

CN 120025 ------------------------------------------------------------

CN 120013 ------------------------------------------------------------

CS17CS1133 ------------------------------------------------------------

CN 120030 GATGGCATAATTGGCATAAAGAAAGCATATTATGGCACTAGTGTTATTATAAATTTATTG

CO46 NCBI ------------------------------------------------------------

DH55 ref genome ------------------------------------------------------------

Hoga ------------------------------------------------------------

CAM 236 ------------------------------------------------------------

09-CS0040 ------------------------------------------------------------

CN 119205 ------------------------------------------------------------

Yellowstone ------------------------------------------------------------

CN 120017 ------------------------------------------------------------

CN 119300 ------------------------------------------------------------

Jasper ------------------------------------------------------------

CN 120027 ------------------------------------------------------------

CN 113754 ------------------------------------------------------------

Joelle AAFC ------------------------------------------------------------

Joelle NCBI ------------------------------------------------------------

Joelle phyto ------------------------------------------------------------

Blaine Creek GATGGCATAATTGGCATAAAGAAAGCATATTATGGCACTAGTGTTATTATAAATTTATTG

CAM 241 GATGGCATAATTGGCATAAAGAAAGCATATTATGGCACTAGTGTTATTATAAATTTATTG

CN 119294 ------------------------------------------------------------

CN 120025 ------------------------------------------------------------

CN 120013 ------------------------------------------------------------

CS17CS1133 ------------------------------------------------------------

CN 120030 AAATTTCGATGATATTTTCTCTCTAATTTTATCAATATTTTATAGTCGGAAAAACGTCAC

CO46 NCBI ------------------------------------------------------------

DH55 ref genome ------------------------------------------------------------

Hoga ------------------------------------------------------------

CAM 236 ------------------------------------------------------------

09-CS0040 ------------------------------------------------------------

CN 119205 ------------------------------------------------------------

Yellowstone ------------------------------------------------------------

CN 120017 ------------------------------------------------------------

CN 119300 ------------------------------------------------------------

Jasper ------------------------------------------------------------

CN 120027 ------------------------------------------------------------

CN 113754 ------------------------------------------------------------

Joelle AAFC ------------------------------------------------------------

Joelle NCBI ------------------------------------------------------------

Joelle phyto ------------------------------------------------------------

Blaine Creek AAATTTCGATGATATTTTCTCTCTAATTTTATCAATATTTTATAGTCAGAAAAACGTCAC

CAM 241 AAATTTCGATGATATTTTCTCTCTAATTTTATCAATATTTTATAGTC-GAAAAACGTCAC

CN 119294 ------------------------------------------------------------

CN 120025 ------------------------------------------------------------

CN 120013 ------------------------------------------------------------

CS17CS1133 ------------------------------------------------------------

CN 120030 AAATTGAGACTATAGTGCGACTCACGTTTTGCGACTGTTTTGTCACTAAAGTCGTCACAA

CO46 NCBI ------------------------------------------------------------

DH55 ref genome ------------------------------------------------------------

Hoga ------------------------------------------------------------

CAM 236 ------------------------------------------------------------

09-CS0040 ------------------------------------------------------------

CN 119205 ------------------------------------------------------------

Yellowstone ------------------------------------------------------------

CN 120017 ------------------------------------------------------------

CN 119300 ------------------------------------------------------------

Jasper ------------------------------------------------------------

CN 120027 ------------------------------------------------------------

CN 113754 ------------------------------------------------------------

Joelle AAFC ------------------------------------------------------------

Joelle NCBI ------------------------------------------------------------

Joelle phyto ------------------------------------------------------------

Blaine Creek AAATTGAGACTATAGTGCGACTCACG-TTTACGACTGTTTTGTCACTAAAGTCGTCACAA

CAM 241 AAATTGAGACTATAGTGCGACTCACGCTTTATGACTGTTTTGTCACTAAAGTCGTCACAA

CN 119294 ------------------------------------------------------------

CN 120025 ------------------------------------------------------------

CN 120013 ------------------------------------------------------------

CS17CS1133 ------------------------------------------------------------

CN 120030 ATTGAGACTATACTGTTTTGTCACAAAGTCGTCACAAATTGAGACTATATTGTGACTGAA

CO46 NCBI ------------------------------------------------------------

DH55 ref genome ------------------------------------------------------------

Hoga ------------------------------------------------------------

CAM 236 ------------------------------------------------------------

09-CS0040 ------------------------------------------------------------

CN 119205 ------------------------------------------------------------

Yellowstone ------------------------------------------------------------

CN 120017 ------------------------------------------------------------

CN 119300 ------------------------------------------------------------

Jasper ------------------------------------------------------------

CN 120027 ------------------------------------------------------------

CN 113754 ------------------------------------------------------------

Joelle AAFC ------------------------------------------------------------

Joelle NCBI ------------------------------------------------------------

Joelle phyto ------------------------------------------------------------

Blaine Creek ATTGAGACTATACTGTTTTGTCACAAAGTCGTCACAAATTGAGACTATATTGTGACTGAA

CAM 241 ATTGAGACTATACTGTTTTGTCACAAAGTCGTCACAAATTGAGACTATATTGTGACTGAA

CN 119294 ------------------------------------------------------------

CN 120025 ------------------------------------------------------------

CN 120013 ------------------------------------------------------------

CS17CS1133 ------------------------------------------------------------

CN 120030 CTCATGTTTT--GCGACTGTTTTGTCACTAAAGTGATTGTGCATCTGTTTATTTCAGTCG

CO46 NCBI ------------------------------------------------------------

DH55 ref genome ------------------------------------------------------------

Hoga ------------------------------------------------------------

CAM 236 ------------------------------------------------------------

09-CS0040 ------------------------------------------------------------

CN 119205 ------------------------------------------------------------

Yellowstone ------------------------------------------------------------

CN 120017 ------------------------------------------------------------

CN 119300 ------------------------------------------------------------

Jasper ------------------------------------------------------------

CN 120027 ------------------------------------------------------------

CN 113754 ------------------------------------------------------------

Joelle AAFC ------------------------------------------------------------

Joelle NCBI ------------------------------------------------------------

Joelle phyto ------------------------------------------------------------

Blaine Creek CTCATGTTTT--GCGACTGTTTTGTCACTAAAGTGATTGTGCATCTGTTTATTTCAGTCG

CAM 241 CTCATGTTTTCATGACATGTTTTGTCACTAAAGTGATTGTGCATCTGTTTATTTCAGTCG

CN 119294 ------------------------------------------------------------

CN 120025 ------------------------------------------------------------

CN 120013 ------------------------------------------------------------

CS17CS1133 ------------------------------------------------------------

CN 120030 CAATAGAGTCTTAAAGTACAACGGTTTAGCGAGTATCTCACATTAATTTTAGAATTTTAC

CO46 NCBI ------------------------------------------------------------

DH55 ref genome ------------------------------------------------------------

Hoga ------------------------------------------------------------

CAM 236 ------------------------------------------------------------

09-CS0040 ------------------------------------------------------------

CN 119205 ------------------------------------------------------------

Yellowstone ------------------------------------------------------------

CN 120017 ------------------------------------------------------------

CN 119300 ------------------------------------------------------------

Jasper ------------------------------------------------------------

CN 120027 ------------------------------------------------------------

CN 113754 ------------------------------------------------------------

Joelle AAFC ------------------------------------------------------------

Joelle NCBI ------------------------------------------------------------

Joelle phyto ------------------------------------------------------------

Blaine Creek CAATAGAGTCTTAAAGTACAACGGTTTAGC------------------------------

CAM 241 CAATAGAGTCTTAAAGTACAACGGTTTAGC------------------------------

CN 119294 ------------------------------------------------------------

CN 120025 ------------------------------------------------------------

CN 120013 ------------------------------------------------------------

CS17CS1133 ------------------------------------------------------------

CN 120030 AAATTTGAGTACAAATAATGTCAACTTATACATTTTTACTTATATGTGACATCTAATTTT

CO46 NCBI ------------------------------------------------------------

DH55 ref genome ------------------------------------------------------------

Hoga ------------------------------------------------------------

CAM 236 ------------------------------------------------------------

09-CS0040 ------------------------------------------------------------

CN 119205 ------------------------------------------------------------

Yellowstone ------------------------------------------------------------

CN 120017 ------------------------------------------------------------

CN 119300 ------------------------------------------------------------

Jasper ------------------------------------------------------------

CN 120027 ------------------------------------------------------------

CN 113754 ------------------------------------------------------------

Joelle AAFC ------------------------------------------------------------

Joelle NCBI ------------------------------------------------------------

Joelle phyto ------------------------------------------------------------

Blaine Creek ------------------------------------------------------------

CAM 241 ------------------------------------------------------------

CN 119294 ------------------------------------------------------------

CN 120025 ------------------------------------------------------------

CN 120013 ------------------------------------------------------------

CS17CS1133 ------------------------------------------------------------

CN 120030 AACTAAATTTCTATAATCACATAAATAAAACACCAAATTAGACAATTATGTTTATATCCT

CO46 NCBI ------------------------------------------------------------

DH55 ref genome ------------------------------------------------------------

Hoga ------------------------------------------------------------

CAM 236 ------------------------------------------------------------

09-CS0040 ------------------------------------------------------------

CN 119205 ------------------------------------------------------------

Yellowstone ------------------------------------------------------------

CN 120017 ------------------------------------------------------------

CN 119300 ------------------------------------------------------------

Jasper ------------------------------------------------------------

CN 120027 ------------------------------------------------------------

CN 113754 ------------------------------------------------------------

Joelle AAFC ------------------------------------------------------------

Joelle NCBI ------------------------------------------------------------

Joelle phyto ------------------------------------------------------------

Blaine Creek ------------------------------------------------------------

CAM 241 ------------------------------------------------------------

CN 119294 ------------------------------------------------------------

CN 120025 ------------------------------------------------------------

CN 120013 ------------------------------------------------------------

CS17CS1133 ------------------------------------------------------------

CN 120030 CCAACACATGTATTAGGGTTACATGTGTTTTAATTAAGTTAGAGTAGTGATGGTTGTATA

CO46 NCBI ------------------------------------------------------------

DH55 ref genome ------------------------------------------------------------

Hoga ------------------------------------------------------------

CAM 236 ------------------------------------------------------------

09-CS0040 ------------------------------------------------------------

CN 119205 ------------------------------------------------------------

Yellowstone ------------------------------------------------------------

CN 120017 ------------------------------------------------------------

CN 119300 ------------------------------------------------------------

Jasper ------------------------------------------------------------

CN 120027 ------------------------------------------------------------

CN 113754 ------------------------------------------------------------

Joelle AAFC ------------------------------------------------------------

Joelle NCBI ------------------------------------------------------------

Joelle phyto ------------------------------------------------------------

Blaine Creek ------------------------------------------------------------

CAM 241 ------------------------------------------------------------

CN 119294 ------------------------------------------------------------

CN 120025 ------------------------------------------------------------

CN 120013 ------------------------------------------------------------

CS17CS1133 ------------------------------------------------------------

CN 120030 TATTTACAAAAACATATAAAAATTGTTATGTTTGATATTTTATCAAAACTAAACTCTTAA

CO46 NCBI ------------------------------------------------------------

DH55 ref genome ------------------------------------------------------------

Hoga ------------------------------------------------------------

CAM 236 ------------------------------------------------------------

09-CS0040 ------------------------------------------------------------

CN 119205 ------------------------------------------------------------

Yellowstone ------------------------------------------------------------

CN 120017 ------------------------------------------------------------

CN 119300 ------------------------------------------------------------

Jasper ------------------------------------------------------------

CN 120027 ------------------------------------------------------------

CN 113754 ------------------------------------------------------------

Joelle AAFC ------------------------------------------------------------

Joelle NCBI ------------------------------------------------------------

Joelle phyto ------------------------------------------------------------

Blaine Creek ------------------------------------------------------------

CAM 241 ------------------------------------------------------------

CN 119294 ------------------------------------------------------------

CN 120025 ------------------------------------------------------------

CN 120013 ------------------------------------------------------------

CS17CS1133 ------------------------------------------------------------

CN 120030 AAGGAGAGTATACATGATCAAAGGGTCATGTCTAATATGTTTTTGGTTAAAAAATCTTAA

CO46 NCBI ------------------------------------------------------------

DH55 ref genome ------------------------------------------------------------

Hoga ------------------------------------------------------------

CAM 236 ------------------------------------------------------------

09-CS0040 ------------------------------------------------------------

CN 119205 ------------------------------------------------------------

Yellowstone ------------------------------------------------------------

CN 120017 ------------------------------------------------------------

CN 119300 ------------------------------------------------------------

Jasper ------------------------------------------------------------

CN 120027 ------------------------------------------------------------

CN 113754 ------------------------------------------------------------

Joelle AAFC ------------------------------------------------------------

Joelle NCBI ------------------------------------------------------------

Joelle phyto ------------------------------------------------------------

Blaine Creek ------------------------------------------------------------

CAM 241 ------------------------------------------------------------

CN 119294 ------------------------------------------------------------

CN 120025 ------------------------------------------------------------

CN 120013 ------------------------------------------------------------

CS17CS1133 ------------------------------------------------------------

CN 120030 CACAAAATTAGACTAATATTGTTTTGTCACAAAGTCGTCACAAATTGAGACTATATTGTG

CO46 NCBI ------------------------------------------------------------

DH55 ref genome ------------------------------------------------------------

Hoga ------------------------------------------------------------

CAM 236 ------------------------------------------------------------

09-CS0040 ------------------------------------------------------------

CN 119205 ------------------------------------------------------------

Yellowstone ------------------------------------------------------------

CN 120017 ------------------------------------------------------------

CN 119300 ------------------------------------------------------------

Jasper ------------------------------------------------------------

CN 120027 ------------------------------------------------------------

CN 113754 ------------------------------------------------------------

Joelle AAFC ------------------------------------------------------------

Joelle NCBI ------------------------------------------------------------

Joelle phyto ------------------------------------------------------------

Blaine Creek ------------------------------------------------------------

CAM 241 ------------------------------------------------------------

CN 119294 ------------------------------------------------------------

CN 120025 ------------------------------------------------------------

CN 120013 ------------------------------------------------------------

CS17CS1133 ------------------------------------------------------------

CN 120030 ACTGAACTCATGTTTTGCGACTGTTTTTTCACTAAAGTGATTGTGCATCTGTTTATTTCA

CO46 NCBI ------------------------------------------------------------

DH55 ref genome ------------------------------------------------------------

Hoga ------------------------------------------------------------

CAM 236 ------------------------------------------------------------

09-CS0040 ------------------------------------------------------------

CN 119205 ------------------------------------------------------------

Yellowstone ------------------------------------------------------------

CN 120017 ------------------------------------------------------------

CN 119300 ------------------------------------------------------------

Jasper ------------------------------------------------------------

CN 120027 ------------------------------------------------------------

CN 113754 ------------------------------------------------------------

Joelle AAFC ------------------------------------------------------------

Joelle NCBI ------------------------------------------------------------

Joelle phyto ------------------------------------------------------------

Blaine Creek ------------------------------------------------------------

CAM 241 ------------------------------------------------------------

CN 119294 ------------------------------------------------------------

CN 120025 ------------------------------------------------------------

CN 120013 ------------------------------------------------------------

CS17CS1133 ------------------------------------------------------------

CN 120030 GTCGCAATAGAGTCTTAAAGTACAACGGTTTAGTGAGTATCTCACATTAATTTTAGAATT

CO46 NCBI ------------------------------------------------------------

DH55 ref genome ------------------------------------------------------------

Hoga ------------------------------------------------------------

CAM 236 ------------------------------------------------------------

09-CS0040 ------------------------------------------------------------

CN 119205 ------------------------------------------------------------

Yellowstone ------------------------------------------------------------

CN 120017 ------------------------------------------------------------

CN 119300 ------------------------------------------------------------

Jasper ------------------------------------------------------------

CN 120027 ------------------------------------------------------------

CN 113754 ------------------------------------------------------------

Joelle AAFC ------------------------------------------------------------

Joelle NCBI ------------------------------------------------------------

Joelle phyto ------------------------------------------------------------

Blaine Creek ---------------------------------TGAGTATCTCACATTAATTTTAGAATT

CAM 241 ----------------------------------GAGTATCTCACATTAATTTTAGAATT

CN 119294 ------------------------------------------------------------

CN 120025 ------------------------------------------------------------

CN 120013 ------------------------------------------------------------

CS17CS1133 ------------------------------------------------------------

CN 120030 TTACAAATTTGAGTACAAATAATGTCAACTTATACATTTTTACTTATATGTGACATCTAA

CO46 NCBI ------------------------------------------------------------

DH55 ref genome ------------------------------------------------------------

Hoga ------------------------------------------------------------

CAM 236 ------------------------------------------------------------

09-CS0040 ------------------------------------------------------------

CN 119205 ------------------------------------------------------------

Yellowstone ------------------------------------------------------------

CN 120017 ------------------------------------------------------------

CN 119300 ------------------------------------------------------------

Jasper ------------------------------------------------------------

CN 120027 ------------------------------------------------------------

CN 113754 ------------------------------------------------------------

Joelle AAFC ------------------------------------------------------------

Joelle NCBI ------------------------------------------------------------

Joelle phyto ------------------------------------------------------------

Blaine Creek TTACAAATTTGAGTACAAATAATGTCAACTTATACATTTTTACTTATATGTGACATCTAA

CAM 241 TTACAAATTTGAGTACAAATAATGTCAACTTATACATTTTTACTTATATGTGACATCTAA

CN 119294 ------------------------------------------------------------

CN 120025 ------------------------------------------------------------

CN 120013 ------------------------------------------------------------

CS17CS1133 ------------------------------------------------------------

CN 120030 TTTTAACTAAATTTCTATAATCACATAAATAAAACACCAAATTAGACAATTATGTTTATA

CO46 NCBI ------------------------------------------------------------

DH55 ref genome ------------------------------------------------------------

Hoga ------------------------------------------------------------

CAM 236 ------------------------------------------------------------

09-CS0040 ------------------------------------------------------------

CN 119205 ------------------------------------------------------------

Yellowstone ------------------------------------------------------------

CN 120017 ------------------------------------------------------------

CN 119300 ------------------------------------------------------------

Jasper ------------------------------------------------------------

CN 120027 ------------------------------------------------------------

CN 113754 ------------------------------------------------------------

Joelle AAFC ------------------------------------------------------------

Joelle NCBI ------------------------------------------------------------

Joelle phyto ------------------------------------------------------------

Blaine Creek TTTTAACTAAATTTCTATAATCACATAAATAAAACACCAAATTAGACAATTATGTTTATA

CAM 241 TTTTAACTAAATTTCTATAATCACATAAATAAAACACCAAATTAGACAATTATGTTTATA

CN 119294 ------------------------------------------------------------

CN 120025 ------------------------------------------------------------

CN 120013 ------------------------------------------------------------

CS17CS1133 ------------------------------------------------------------

CN 120030 TCCTCCAACACATGTATTAGGGTTACATGTGTTTTAATTAAGTTAGAGTAGTGATGGTTG

CO46 NCBI ------------------------------------------------------------

DH55 ref genome ------------------------------------------------------------

Hoga ------------------------------------------------------------

CAM 236 ------------------------------------------------------------

09-CS0040 ------------------------------------------------------------

CN 119205 ------------------------------------------------------------

Yellowstone ------------------------------------------------------------

CN 120017 ------------------------------------------------------------

CN 119300 ------------------------------------------------------------

Jasper ------------------------------------------------------------

CN 120027 ------------------------------------------------------------

CN 113754 ------------------------------------------------------------

Joelle AAFC ------------------------------------------------------------

Joelle NCBI ------------------------------------------------------------

Joelle phyto ------------------------------------------------------------

Blaine Creek TCCTCCAACACATGTATTAGGGTTACATGTGTTTTAATTAAGTTAGAGTAGTGATGGTTG

CAM 241 TCCTCCAACACATGTATTAGGGTTACATGTGTTTTAATTAAGTTAGAGTAGTGATGGTTG

CN 119294 ------------------------------------------------------------

CN 120025 ------------------------------------------------------------

CN 120013 ------------------------------------------------------------

CS17CS1133 ------------------------------------------------------------

CN 120030 TATATATTTACAAAAACATATAAAAATTGTTATGTTTGATATTTTATCAAAACTAAACTC

CO46 NCBI ------------------------------------------------------------

DH55 ref genome ------------------------------------------------------------

Hoga ------------------------------------------------------------

CAM 236 ------------------------------------------------------------

09-CS0040 ------------------------------------------------------------

CN 119205 ------------------------------------------------------------

Yellowstone ------------------------------------------------------------

CN 120017 ------------------------------------------------------------

CN 119300 ------------------------------------------------------------

Jasper ------------------------------------------------------------

CN 120027 ------------------------------------------------------------

CN 113754 ------------------------------------------------------------

Joelle AAFC ------------------------------------------------------------

Joelle NCBI ------------------------------------------------------------

Joelle phyto ------------------------------------------------------------

Blaine Creek TATATATTTACAAAAACATATAAAAATTGTTATGTTTGATATTTTATCAAAACTAAACTC

CAM 241 TATATATTTACAAAAACATATAAAAATTGTTATGTTTGATATTTTATCAAAACTAAACTC

CN 119294 ------------------------------------------------------------

CN 120025 ------------------------------------------------------------

CN 120013 ------------------------------------------------------------

CS17CS1133 ------------------------------------------------------------

CN 120030 TTAAAAGGAGAGTATACATGATCAAAGGGTCATGTCTAATATGTTTTTGGTTAAAAAATC

CO46 NCBI ------------------------------------------------------------

DH55 ref genome ------------------------------------------------------------

Hoga ------------------------------------------------------------

CAM 236 ------------------------------------------------------------

09-CS0040 ------------------------------------------------------------

CN 119205 ------------------------------------------------------------

Yellowstone ------------------------------------------------------------

CN 120017 ------------------------------------------------------------

CN 119300 ------------------------------------------------------------

Jasper ------------------------------------------------------------

CN 120027 ------------------------------------------------------------

CN 113754 ------------------------------------------------------------

Joelle AAFC ------------------------------------------------------------

Joelle NCBI ------------------------------------------------------------

Joelle phyto ------------------------------------------------------------

Blaine Creek TTAAAAGGAGAGTATACATGATCAAAGGGTCATGTCTAATATGTTTTTGGTTAAAAAATC

CAM 241 TTAAAAGGAGAGTATACATGATCAAAGGGTCATGTCTAATATGTTTTTGGTTAAAAAATC

CN 119294 ------------------------------------------------------------

CN 120025 ------------------------------------------------------------

CN 120013 ------------------------------------------------------------

CS17CS1133 ------------------------------------------------------------

CN 120030 TTAACACAAAATTAGACTAATATTGTTTTGTCACAAAGTCGTCACAAATTGAGACTATAT

CO46 NCBI ------------------------------------------------------------

DH55 ref genome ------------------------------------------------------------

Hoga ------------------------------------------------------------

CAM 236 ------------------------------------------------------------

09-CS0040 ------------------------------------------------------------

CN 119205 ------------------------------------------------------------

Yellowstone ------------------------------------------------------------

CN 120017 ------------------------------------------------------------

CN 119300 ------------------------------------------------------------

Jasper ------------------------------------------------------------

CN 120027 ------------------------------------------------------------

CN 113754 ------------------------------------------------------------

Joelle AAFC ------------------------------------------------------------

Joelle NCBI ------------------------------------------------------------

Joelle phyto ------------------------------------------------------------

Blaine Creek TTAACACAAAATTAGACTAATATTGTTTTGTCACAAAGTCGTCACAAATTGAGACTATAT

CAM 241 TTAACACAAAATTAGACTAATATTGTTTTGTCACAAAGTCGTCACAAATTGAGACTATAT

CN 119294 ------------------------------------------------------------

CN 120025 ------------------------------------------------------------

CN 120013 ------------------------------------------------------------

CS17CS1133 ------------------------------------------------------------

CN 120030 TGTGACTGAACTCATGTTTTGCGACTGTTTTGTCACTAAGTGTAATTTTTGTTTAATGTT

CO46 NCBI ------------------------------------------------------------

DH55 ref genome ------------------------------------------------------------

Hoga ------------------------------------------------------------

CAM 236 ------------------------------------------------------------

09-CS0040 ------------------------------------------------------------

CN 119205 ------------------------------------------------------------

Yellowstone ------------------------------------------------------------

CN 120017 ------------------------------------------------------------

CN 119300 ------------------------------------------------------------

Jasper ------------------------------------------------------------

CN 120027 ------------------------------------------------------------

CN 113754 ------------------------------------------------------------

Joelle AAFC ------------------------------------------------------------

Joelle NCBI ------------------------------------------------------------

Joelle phyto ------------------------------------------------------------

Blaine Creek TGTGGCTGAACTCATGTTTTGCGACTGTTTTGTCACTAAGTGTAATTTTTGTTTAATGTT

CAM 241 TGTGACTGAACTCATGTTTTGCGACTGTTTTGTCACTAAGTGTAATTTTTGTTTAATGTT

CN 119294 ------------------------------------------------------------

CN 120025 ------------------------------------------------------------

CN 120013 ------------------------------------------------------------

CS17CS1133 ------------------------------------------------------------

CN 120030 TACGACAG-TCACAAAGTAGTCTTAAATTACTACTACTTTCTTACGGATTTAAATTTCCC

CO46 NCBI ------------------------------------------------------------

DH55 ref genome ------------------------------------------------------------

Hoga ------------------------------------------------------------

CAM 236 ------------------------------------------------------------

09-CS0040 ------------------------------------------------------------

CN 119205 ------------------------------------------------------------

Yellowstone ------------------------------------------------------------

CN 120017 ------------------------------------------------------------

CN 119300 ------------------------------------------------------------

Jasper ------------------------------------------------------------

CN 120027 ------------------------------------------------------------

CN 113754 ------------------------------------------------------------

Joelle AAFC ------------------------------------------------------------

Joelle NCBI ------------------------------------------------------------

Joelle phyto ------------------------------------------------------------

Blaine Creek TACGACAG-TCACAAAGTAGTCTTAAATTACTACTACTTTCTTACGGATTTAAATTTCCC

CAM 241 TACGACAGATCACAAAGTAGTCTTAAATTACTACTACTTTCTTACGGATTTAAATTTCCC

CN 119294 ------------------------------------------------------------

CN 120025 ------------------------------------------------------------

CN 120013 ------------------------------------------------------------

CS17CS1133 ------------------------------------------------------------

CN 120030 CAAATTTTAGTGAAAAATTTTAGCGGTAATTATT-CCCCCAGATTAATTATTCCCCCCAT

CO46 NCBI ------------------------------------------------------------

DH55 ref genome ------------------------------------------------------------

Hoga ------------------------------------------------------------

CAM 236 ------------------------------------------------------------

09-CS0040 ------------------------------------------------------------

CN 119205 ------------------------------------------------------------

Yellowstone ------------------------------------------------------------

CN 120017 ------------------------------------------------------------

CN 119300 ------------------------------------------------------------

Jasper ------------------------------------------------------------

CN 120027 ------------------------------------------------------------

CN 113754 ------------------------------------------------------------

Joelle AAFC ------------------------------------------------------------

Joelle NCBI ------------------------------------------------------------

Joelle phyto ------------------------------------------------------------

Blaine Creek CAAATTTTAGTGAAAAATTTTAGCGGTAATTATTCCCCCCAGATTAATTATTCCCCCCAT

CAM 241 CAAATTTTAGTGAAAAATTTTAGCGGTAATTATT-CCCCCAGATTAATTATTCCCCCCAT

CN 119294 ------------------------------------------------------------

CN 120025 ------------------------------------------------------------

CN 120013 ------------------------------------------------------------

CS17CS1133 ------------------------------------------------------------

CN 120030 CATCAATTGAACACTCTTAGTTCTCTTAGGAAATAACGAAACCCTCATAATTTCCCGAAA

CO46 NCBI ------------------------------------------------------------

DH55 ref genome ------------------------------------------------------------

Hoga ------------------------------------------------------------

CAM 236 ------------------------------------------------------------

09-CS0040 ------------------------------------------------------------

CN 119205 ------------------------------------------------------------

Yellowstone ------------------------------------------------------------

CN 120017 ------------------------------------------------------------

CN 119300 ------------------------------------------------------------

Jasper ------------------------------------------------------------

CN 120027 ------------------------------------------------------------

CN 113754 ------------------------------------------------------------

Joelle AAFC ------------------------------------------------------------

Joelle NCBI ------------------------------------------------------------

Joelle phyto ------------------------------------------------------------

Blaine Creek CATCAATTGAACACTCTTAGTTCTCTTAGGAAATAACGAAACCCTCATAATTTCCCGAAA

CAM 241 CATCAATTGAACACTCTTAGTTCTCTTAGGAAATAACGAAACCCTCATAATTTCCCGAAA

CN 119294 ------------------------------------------------------------

CN 120025 ------------------------------------------------------------

CN 120013 ------------------------------------------------------------

CS17CS1133 ------------------------------------------------------------

CN 120030 CCCTC-TTTTTCTTTTCTCTCTGTCTTCTCGTTTTCTTCTGAAATTCCGACTTCTTCTCT

CO46 NCBI ------------------------------------------------------------

DH55 ref genome ------------------------------------------------------------

Hoga ------------------------------------------------------------

CAM 236 ------------------------------------------------------------

09-CS0040 ------------------------------------------------------------

CN 119205 ------------------------------------------------------------

Yellowstone ------------------------------------------------------------

CN 120017 ------------------------------------------------------------

CN 119300 ------------------------------------------------------------

Jasper ------------------------------------------------------------

CN 120027 ------------------------------------------------------------

CN 113754 ------------------------------------------------------------

Joelle AAFC ------------------------------------------------------------

Joelle NCBI ------------------------------------------------------------

Joelle phyto ------------------------------------------------------------

Blaine Creek CCCTCTTTTTTCTTTTCTCTCTGTCTTCTCGTTTTCTTCTGAAATTCCGACTTCTTCTCT

CAM 241 CCCTC--TTTTCTTTTCTCTCTGTCTTCTCGTTTTCTTC-GAAATTCCGACTTCTTCTCC

CN 119294 ------------------------------------------------------------

CN 120025 ------------------------------------------------------------

CN 120013 ------------------------------------------------------------

CS17CS1133 ------------------------------------------------------------

CN 120030 GACTTCTTCTCCGACTTCTTCTCCGACTTCTGTTCCTATGAAATCCGGTGGTGGTCGTGG

CO46 NCBI ------------------------------------------------------------

DH55 ref genome ------------------------------------------------------------

Hoga ------------------------------------------------------------

CAM 236 ------------------------------------------------------------

09-CS0040 ------------------------------------------------------------

CN 119205 ------------------------------------------------------------

Yellowstone ------------------------------------------------------------

CN 120017 ------------------------------------------------------------

CN 119300 ------------------------------------------------------------

Jasper ------------------------------------------------------------

CN 120027 ------------------------------------------------------------

CN 113754 ------------------------------------------------------------

Joelle AAFC ------------------------------------------------------------

Joelle NCBI ------------------------------------------------------------

Joelle phyto ------------------------------------------------------------

Blaine Creek GACTTCTTCTCCGACTTCTTCTCCGACTTCTGTTCCTATGAAATCCGGTGGTGGTCGTGG

CAM 241 GACTTCTTCTCCGACTTCTTCTCCGACTTC-GTTCCTATGAAATCCGGTGGTGGTCGTGG

CN 119294 ------------------------------------------------------------

CN 120025 ------------------------------------------------------------

CN 120013 ------------------------------------------------------------

CS17CS1133 ------------------------------------------------------------

CN 120030 TTCTAAATCTGGTGGTGGTCGTGGTGCTAGATCTGGCGGTGGTGGTCGTGGATCCGGCGG

CO46 NCBI ------------------------------------------------------------

DH55 ref genome ------------------------------------------------------------

Hoga ------------------------------------------------------------

CAM 236 ------------------------------------------------------------

09-CS0040 ------------------------------------------------------------

CN 119205 ------------------------------------------------------------

Yellowstone ------------------------------------------------------------

CN 120017 ------------------------------------------------------------

CN 119300 ------------------------------------------------------------

Jasper ------------------------------------------------------------

CN 120027 ------------------------------------------------------------

CN 113754 ------------------------------------------------------------

Joelle AAFC ------------------------------------------------------------

Joelle NCBI ------------------------------------------------------------

Joelle phyto ------------------------------------------------------------

Blaine Creek TTCTAAATCTGGTGGTGGTCGTGGTGCTAGATCTGGCGGTGGTGGTCGTGGATCTGGCGG

CAM 241 TTCTAAATCTGGTGGTGGTCGTGGTGCTAGATCTGGCGGTGGTGGTCGTGGATCCGGCGG

CN 119294 ------------------------------------------------------------

CN 120025 ------------------------------------------------------------

CN 120013 ------------------------------------------------------------

CS17CS1133 ------------------------------------------------------------

CN 120030 CGTTGCGAGACGAAGTAGAGATGTCGC-TCGTCGTCTCACTCCTCTAACCCTTCCTCTCA

CO46 NCBI ------------------------------------------------------------

DH55 ref genome ------------------------------------------------------------

Hoga ------------------------------------------------------------

CAM 236 ------------------------------------------------------------

09-CS0040 ------------------------------------------------------------

CN 119205 ------------------------------------------------------------

Yellowstone ------------------------------------------------------------

CN 120017 ------------------------------------------------------------

CN 119300 ------------------------------------------------------------

Jasper ------------------------------------------------------------

CN 120027 ------------------------------------------------------------

CN 113754 ------------------------------------------------------------

Joelle AAFC ------------------------------------------------------------

Joelle NCBI ------------------------------------------------------------

Joelle phyto ------------------------------------------------------------

Blaine Creek CGTTGCGAGACGAAGTAGAGATGTCGCTTCGTCGTCTCACTCCTCTAACCCTTCCTCTCA

CAM 241 CGTTGCGAGACGAAGTAGAGATGTCGC-TCGTCGTCTCACTCCTCTAACCCTTCCTCTCA

CN 119294 ------------------------------------------------------------

CN 120025 ------------------------------------------------------------

CN 120013 ------------------------------------------------------------

CS17CS1133 ------------------------------------------------------------

CN 120030 CAACGAAAATTCTTCACACTCCCAACAATCTCAACCGTCGCTTCCATCTCGGTATACGCA

CO46 NCBI ------------------------------------------------------------

DH55 ref genome ------------------------------------------------------------

Hoga ------------------------------------------------------------

CAM 236 ------------------------------------------------------------

09-CS0040 ------------------------------------------------------------

CN 119205 ------------------------------------------------------------

Yellowstone ------------------------------------------------------------

CN 120017 ------------------------------------------------------------

CN 119300 ------------------------------------------------------------

Jasper ------------------------------------------------------------

CN 120027 ------------------------------------------------------------

CN 113754 ------------------------------------------------------------

Joelle AAFC ------------------------------------------------------------

Joelle NCBI ------------------------------------------------------------

Joelle phyto ------------------------------------------------------------

Blaine Creek CAACGAAAATTCTTCACACTCCCAACAATCTCAACCGTCGCTTCCATCTCGGTATACGCA

CAM 241 CAACGAAAATTCTTCACACTCCCAACAATCTCAACCGTCGCTTCCATCTCGGTATACGCA

CN 119294 ------------------------------------------------------------

CN 120025 ------------------------------------------------------------

CN 120013 ------------------------------------------------------------

CS17CS1133 ------------------------------------------------------------

CN 120030 AGCTTCATTTCCCCAGTATCCTCGAGAAGCTTCTCCTCAGCCACAACAACCAGGCTTTCA

CO46 NCBI ------------------------------------------------------------

DH55 ref genome ------------------------------------------------------------

Hoga ------------------------------------------------------------

CAM 236 ------------------------------------------------------------

09-CS0040 ------------------------------------------------------------

CN 119205 ------------------------------------------------------------

Yellowstone ------------------------------------------------------------

CN 120017 ------------------------------------------------------------

CN 119300 ------------------------------------------------------------

Jasper ------------------------------------------------------------

CN 120027 ------------------------------------------------------------

CN 113754 ------------------------------------------------------------

Joelle AAFC ------------------------------------------------------------

Joelle NCBI ------------------------------------------------------------

Joelle phyto ------------------------------------------------------------

Blaine Creek AGCTTCATTTCCCCAGTATCCTCGAGAAGCTTCTCCTCAGCCACAACAACCAGGCTTTCA

CAM 241 AGCTTCATTTCCCC-GTATCCTCGAGAAGCTTCTCCTCAGCCACAACAACCAGGCTTTCA

CN 119294 ------------------------------------------------------------

CN 120025 ------------------------------------------------------------

CN 120013 ------------------------------------------------------------

CS17CS1133 ------------------------------------------------------------

CN 120030 ACAACAGCTCCACTCCCGGATGCAGCAGCCACCACCACCGCATGCTCATGTTCCGGTGTA

CO46 NCBI ------------------------------------------------------------

DH55 ref genome ------------------------------------------------------------

Hoga ------------------------------------------------------------

CAM 236 ------------------------------------------------------------

09-CS0040 ------------------------------------------------------------

CN 119205 ------------------------------------------------------------

Yellowstone ------------------------------------------------------------

CN 120017 ------------------------------------------------------------

CN 119300 ------------------------------------------------------------

Jasper ------------------------------------------------------------

CN 120027 ------------------------------------------------------------

CN 113754 ------------------------------------------------------------

Joelle AAFC ------------------------------------------------------------

Joelle NCBI ------------------------------------------------------------

Joelle phyto ------------------------------------------------------------

Blaine Creek ACAACAGCTCCACTCCCAGATGCAGCAGCCACCACCACCGCATGCTCATGTTCCGGTGTA

CAM 241 ACAACAGCTCCACTCCCGGATGCAGCAGCCACCACCACCGCATGCTCATGTTCCGGTGTA

CN 119294 ------------------------------------------------------------

CN 120025 ------------------------------------------------------------

CN 120013 ------------------------------------------------------------

CS17CS1133 ------------------------------------------------------------

CN 120030 TCAACAGGTGGCTCCTCGGAATCCGGGATACCAACAGCCGCTTCACAACGGTGGATATCA

CO46 NCBI ------------------------------------------------------------

DH55 ref genome ------------------------------------------------------------

Hoga ------------------------------------------------------------

CAM 236 ------------------------------------------------------------

09-CS0040 ------------------------------------------------------------

CN 119205 ------------------------------------------------------------

Yellowstone ------------------------------------------------------------

CN 120017 ------------------------------------------------------------

CN 119300 ------------------------------------------------------------

Jasper ------------------------------------------------------------

CN 120027 ------------------------------------------------------------

CN 113754 ------------------------------------------------------------

Joelle AAFC ------------------------------------------------------------

Joelle NCBI ------------------------------------------------------------

Joelle phyto ------------------------------------------------------------

Blaine Creek TCAACAGGTGGCTCCTCAGAATCCGGGATACCAACAGCCGCTTCACAACGGTGGATATCA

CAM 241 TCAACAGGTGGCTCCTCGGAATCCGGGATACCAACAGCCGCTTCACAACGGTGGATATCA

CN 119294 ------------------------------------------------------------

CN 120025 ------------------------------------------------------------

CN 120013 ------------------------------------------------------------

CS17CS1133 ------------------------------------------------------------

CN 120030 ACCACCGCTTCACCATCCGGCTTATCCACCGCCGCTTCGGGATCTGGGGTTTCTGCCACC

CO46 NCBI ------------------------------------------------------------

DH55 ref genome ------------------------------------------------------------

Hoga ------------------------------------------------------------

CAM 236 ------------------------------------------------------------

09-CS0040 ------------------------------------------------------------

CN 119205 ------------------------------------------------------------

Yellowstone ------------------------------------------------------------

CN 120017 ------------------------------------------------------------

CN 119300 ------------------------------------------------------------

Jasper ------------------------------------------------------------

CN 120027 ------------------------------------------------------------

CN 113754 ------------------------------------------------------------

Joelle AAFC ------------------------------------------------------------

Joelle NCBI ------------------------------------------------------------

Joelle phyto ------------------------------------------------------------

Blaine Creek ACCACCGCTTCACCATCCGGCTTATCCACCGCCGCTTCAGGATCTGGGGTTTCTGCCACC

CAM 241 ACCACCGCTTCACCATCCGGCTTATCCACCGCCGCTTCAGGATCTGGGGTTTC-GCCACC

CN 119294 ------------------------------------------------------------

CN 120025 ------------------------------------------------------------

CN 120013 ------------------------------------------------------------

CS17CS1133 ------------------------------------------------------------

CN 120030 GAATCCTCGGAATCCGGTGTATCAACCGCCAGCTCCTCCGAATCTAGGATTCCAACCGCC

CO46 NCBI ------------------------------------------------------------

DH55 ref genome ------------------------------------------------------------

Hoga ------------------------------------------------------------

CAM 236 ------------------------------------------------------------

09-CS0040 ------------------------------------------------------------

CN 119205 ------------------------------------------------------------

Yellowstone ------------------------------------------------------------

CN 120017 ------------------------------------------------------------

CN 119300 ------------------------------------------------------------

Jasper ------------------------------------------------------------

CN 120027 ------------------------------------------------------------

CN 113754 ------------------------------------------------------------

Joelle AAFC ------------------------------------------------------------

Joelle NCBI ------------------------------------------------------------

Joelle phyto ------------------------------------------------------------

Blaine Creek GAATCCTCAGAATCCGGTGTATCAACCGCCAGCTCCTCCGAATCTAGGATTCCAACCGCC

CAM 241 GAATCCTCGGAATCCGGTGTATCAACCGCCAGCTCCTCCGAATCTAGGATTCCAACCGCC

CN 119294 ------------------------------------------------------------

CN 120025 ------------------------------------------------------------

CN 120013 ------------------------------------------------------------

CS17CS1133 ------------------------------------------------------------

CN 120030 GCCGCCTCCGAATCAGGACGGTCAAGAGCATCCACCACCGGCTCAGAATCAAGACTTCCA

CO46 NCBI ------------------------------------------------------------

DH55 ref genome ------------------------------------------------------------

Hoga ------------------------------------------------------------

CAM 236 ------------------------------------------------------------

09-CS0040 ------------------------------------------------------------

CN 119205 ------------------------------------------------------------

Yellowstone ------------------------------------------------------------

CN 120017 ------------------------------------------------------------

CN 119300 ------------------------------------------------------------

Jasper ------------------------------------------------------------

CN 120027 ------------------------------------------------------------

CN 113754 ------------------------------------------------------------

Joelle AAFC ------------------------------------------------------------

Joelle NCBI ------------------------------------------------------------

Joelle phyto ------------------------------------------------------------

Blaine Creek GCCGCCTCCGAATCAGGACGGTCAAGAGCATCCACCACCGGCTCAGAATCAAGACTTCCA

CAM 241 GCCGCCTCCGAATCGGGACGGTCAAGAGCATCCACCACCGGCTCGGAATCAAGACTTCCA

CN 119294 ------------------------------------------------------------

CN 120025 ------------------------------------------------------------

CN 120013 ------------------------------------------------------------

CS17CS1133 ------------------------------------------------------------

CN 120030 ACAGCAACT-GGATGAGTTGCTTGCACTTCCTGGTCGACAACATCTCCCGATATTGAGTC

CO46 NCBI ------------------------------------------------------------

DH55 ref genome ------------------------------------------------------------

Hoga ------------------------------------------------------------

CAM 236 ------------------------------------------------------------

09-CS0040 ------------------------------------------------------------

CN 119205 ------------------------------------------------------------

Yellowstone ------------------------------------------------------------

CN 120017 ------------------------------------------------------------

CN 119300 ------------------------------------------------------------

Jasper ------------------------------------------------------------

CN 120027 ------------------------------------------------------------

CN 113754 ------------------------------------------------------------

Joelle AAFC ------------------------------------------------------------

Joelle NCBI ------------------------------------------------------------

Joelle phyto ------------------------------------------------------------

Blaine Creek ACAGCAACT-GGATGAGTTGCTTGCACTTCCTGGTCGACAACATCTCCCGATATTGAGTC

CAM 241 ACAGCAACTCGGATGAGTTGCTTGCACTTCCTGGTCGACAACATCTCCCGATATTGAGTC

CN 119294 ------------------------------------------------------------

CN 120025 ------------------------------------------------------------

CN 120013 ------------------------------------------------------------

CS17CS1133 ------------------------------------------------------------

CN 120030 CGCATTCCATCCCCGACACTCAAAGCATATGGTAAAATATTTTTTTCTTTTATTTTATCA

CO46 NCBI ------------------------------------------------------------

DH55 ref genome ------------------------------------------------------------

Hoga ------------------------------------------------------------

CAM 236 ------------------------------------------------------------

09-CS0040 ------------------------------------------------------------

CN 119205 ------------------------------------------------------------

Yellowstone ------------------------------------------------------------

CN 120017 ------------------------------------------------------------

CN 119300 ------------------------------------------------------------

Jasper ------------------------------------------------------------

CN 120027 ------------------------------------------------------------

CN 113754 ------------------------------------------------------------

Joelle AAFC ------------------------------------------------------------

Joelle NCBI ------------------------------------------------------------

Joelle phyto ------------------------------------------------------------

Blaine Creek CGCATTCCATCCCCGACACTCAAAGCATATGGTAAAATATTTTTTTCTTTTATTTTATCA

CAM 241 CGCATTCCATCCCCGACACTCAAAGCATATGGTAAAATA-TTTTTTCTTTTATTTTATCA

CN 119294 ------------------------------------------------------------

CN 120025 ------------------------------------------------------------

CN 120013 ------------------------------------------------------------

CS17CS1133 ------------------------------------------------------------

CN 120030 TTTGATTATTTCTTACGATAAATAAGTAAAATATTTATCATTTGATTATCATTTTATCAT

CO46 NCBI ------------------------------------------------------------

DH55 ref genome ------------------------------------------------------------

Hoga ------------------------------------------------------------

CAM 236 ------------------------------------------------------------

09-CS0040 ------------------------------------------------------------

CN 119205 ------------------------------------------------------------

Yellowstone ------------------------------------------------------------

CN 120017 ------------------------------------------------------------

CN 119300 ------------------------------------------------------------

Jasper ------------------------------------------------------------

CN 120027 ------------------------------------------------------------

CN 113754 ------------------------------------------------------------

Joelle AAFC ------------------------------------------------------------

Joelle NCBI ------------------------------------------------------------

Joelle phyto ------------------------------------------------------------

Blaine Creek TTTGATTATTTCTTACGATAAATAAGTAAAATATTTATCATTTGATTATCATTTTATCAT

CAM 241 TTTGATTATTTCTTACGATAAATAAGTAAAATATTTATCATTTGATTATCATTTTATCAT

CN 119294 ------------------------------------------------------------

CN 120025 ------------------------------------------------------------

CN 120013 ------------------------------------------------------------

CS17CS1133 ------------------------------------------------------------

CN 120030 TTGGTAAAATATTTTTTTCTTTTATTTTATAATTTGATTATCGCATTGCTCCCATTTGAA

CO46 NCBI ------------------------------------------------------------

DH55 ref genome ------------------------------------------------------------

Hoga ------------------------------------------------------------

CAM 236 ------------------------------------------------------------

09-CS0040 ------------------------------------------------------------

CN 119205 ------------------------------------------------------------

Yellowstone ------------------------------------------------------------

CN 120017 ------------------------------------------------------------

CN 119300 ------------------------------------------------------------

Jasper ------------------------------------------------------------

CN 120027 ------------------------------------------------------------

CN 113754 ------------------------------------------------------------

Joelle AAFC ------------------------------------------------------------

Joelle NCBI ------------------------------------------------------------

Joelle phyto ------------------------------------------------------------

Blaine Creek TTGGTAAAATATTTTTTTCTTTTATTTTATAATTTGATTATCGCATTGCTCCCATTTGAA

CAM 241 TTGGTAAAATATTTTTTTCTTTTATTTTATAATTTGATTATCGCATTGCTCCCATTTGAA

CN 119294 ------------------------------------------------------------

CN 120025 ------------------------------------------------------------

CN 120013 ------------------------------------------------------------

CS17CS1133 ------------------------------------------------------------

CN 120030 AAACGCTTTGTGTGTTGTGTAGTTTAGATAAAATAAGTAGATTATTGTTTGGTTCAGTTT

CO46 NCBI ------------------------------------------------------------

DH55 ref genome ------------------------------------------------------------

Hoga ------------------------------------------------------------

CAM 236 ------------------------------------------------------------

09-CS0040 ------------------------------------------------------------

CN 119205 ------------------------------------------------------------

Yellowstone ------------------------------------------------------------

CN 120017 ------------------------------------------------------------

CN 119300 ------------------------------------------------------------

Jasper ------------------------------------------------------------

CN 120027 ------------------------------------------------------------

CN 113754 ------------------------------------------------------------

Joelle AAFC ------------------------------------------------------------

Joelle NCBI ------------------------------------------------------------

Joelle phyto ------------------------------------------------------------

Blaine Creek AAACGCTTTGTGTGTTGTGTAGTTTAGATAAAATAAGTAGATTATTGTTTGGTTCAGTTT

CAM 241 AAACGCTTTGTGTGTTGTGTAGTTTAGATAAAATAAGTAGATTATTGTTTGGTTCAGTTT

CN 119294 ------------------------------------------------------------

CN 120025 ------------------------------------------------------------

CN 120013 ------------------------------------------------------------

CS17CS1133 ------------------------------------------------------------

CN 120030 CTTATTGTTTTCAAAGTTTTGTAACTTTTCTTAGTTGTTTTCAAAGTTTCTAAATTGTTT

CO46 NCBI ------------------------------------------------------------

DH55 ref genome ------------------------------------------------------------

Hoga ------------------------------------------------------------

CAM 236 ------------------------------------------------------------

09-CS0040 ------------------------------------------------------------

CN 119205 ------------------------------------------------------------

Yellowstone ------------------------------------------------------------

CN 120017 ------------------------------------------------------------

CN 119300 ------------------------------------------------------------

Jasper ------------------------------------------------------------

CN 120027 ------------------------------------------------------------

CN 113754 ------------------------------------------------------------

Joelle AAFC ------------------------------------------------------------

Joelle NCBI ------------------------------------------------------------

Joelle phyto ------------------------------------------------------------

Blaine Creek CTTATTGTTTTCAAAGTTTTGTAACTTTTCTTAGTTGTTTTCAAAGTTTCTAAATTGTTT

CAM 241 CTTATTGTTTTCAAAGTTTTGTAACTTTTCTTAGTTGTTTTCAAAGTTTCTAAATTGTTT

CN 119294 ------------------------------------------------------------

CN 120025 ------------------------------------------------------------

CN 120013 ------------------------------------------------------------

CS17CS1133 ------------------------------------------------------------

CN 120030 TCAAAGTTTGTTGTGTTGTGTTGTATTGTGTTGTGAAGTTTAGTTACATGTCCAATTTGA

CO46 NCBI ------------------------------------------------------------

DH55 ref genome ------------------------------------------------------------

Hoga ------------------------------------------------------------

CAM 236 ------------------------------------------------------------

09-CS0040 ------------------------------------------------------------

CN 119205 ------------------------------------------------------------

Yellowstone ------------------------------------------------------------

CN 120017 ------------------------------------------------------------

CN 119300 ------------------------------------------------------------

Jasper ------------------------------------------------------------

CN 120027 ------------------------------------------------------------

CN 113754 ------------------------------------------------------------

Joelle AAFC ------------------------------------------------------------

Joelle NCBI ------------------------------------------------------------

Joelle phyto ------------------------------------------------------------

Blaine Creek TCAAAGTTTGTTGTGTTGTGTTGTATTGTGTTGTGAAGTTTAGTTACATGTCCAATTTGA

CAM 241 TCAAAGTTTGTTGTGTTGTGTTGTATTGTGTTGTGAAGTTTAGTTACATGTCCAATTTGA

CN 119294 ------------------------------------------------------------

CN 120025 ------------------------------------------------------------

CN 120013 ------------------------------------------------------------

CS17CS1133 ------------------------------------------------------------

CN 120030 TTATTGTGTTGTGATCCATTCGCTTATATTTTATAACTTAACACTCTCATATTTATGTAA

CO46 NCBI ------------------------------------------------------------

DH55 ref genome ------------------------------------------------------------

Hoga ------------------------------------------------------------

CAM 236 ------------------------------------------------------------

09-CS0040 ------------------------------------------------------------

CN 119205 ------------------------------------------------------------

Yellowstone ------------------------------------------------------------

CN 120017 ------------------------------------------------------------

CN 119300 ------------------------------------------------------------

Jasper ------------------------------------------------------------

CN 120027 ------------------------------------------------------------

CN 113754 ------------------------------------------------------------

Joelle AAFC ------------------------------------------------------------

Joelle NCBI ------------------------------------------------------------

Joelle phyto ------------------------------------------------------------

Blaine Creek TTATTGTGTTGTGATCCATTCGCTTATATTTTATAACTTAACACTCTCATATTTATGTAA

CAM 241 TTATTGTGTTGTGATCCATTCGCTTATATTTTATAACTTAACACTCTCATATTTATGTAA

CN 119294 ------------------------------------------------------------

CN 120025 ------------------------------------------------------------

CN 120013 ------------------------------------------------------------

CS17CS1133 ------------------------------------------------------------

CN 120030 CAGGTTTGGACGAGACAAAGGAAAGCTATCTCGTGTTATTTCTGGGATTTTAAGGAGAAA

CO46 NCBI ------------------------------------------------------------

DH55 ref genome ------------------------------------------------------------

Hoga ------------------------------------------------------------

CAM 236 ------------------------------------------------------------

09-CS0040 ------------------------------------------------------------

CN 119205 ------------------------------------------------------------

Yellowstone ------------------------------------------------------------

CN 120017 ------------------------------------------------------------

CN 119300 ------------------------------------------------------------

Jasper ------------------------------------------------------------

CN 120027 ------------------------------------------------------------

CN 113754 ------------------------------------------------------------

Joelle AAFC ------------------------------------------------------------

Joelle NCBI ------------------------------------------------------------

Joelle phyto ------------------------------------------------------------

Blaine Creek CAGGTTTGGACGAGACAAAGGAAAGCTATCTCGTGTTATTTCTGGGATTTTAAGGAGAAA

CAM 241 CAGGTTTGGACGAGACAAAGGAAAGCTATCTCGTGTTATTTC-GGGATTTTAAGGAGAAA

CN 119294 ------------------------------------------------------------

CN 120025 ------------------------------------------------------------

CN 120013 ------------------------------------------------------------

CS17CS1133 ------------------------------------------------------------

CN 120030 GTTCGATGGTCCATACTTCAGCTGGAAGGTTACGCCATTACACATACGGGAGAGATATTT

CO46 NCBI ------------------------------------------------------------

DH55 ref genome ------------------------------------------------------------

Hoga ------------------------------------------------------------

CAM 236 ------------------------------------------------------------

09-CS0040 ------------------------------------------------------------

CN 119205 ------------------------------------------------------------

Yellowstone ------------------------------------------------------------

CN 120017 ------------------------------------------------------------

CN 119300 ------------------------------------------------------------

Jasper ------------------------------------------------------------

CN 120027 ------------------------------------------------------------

CN 113754 ------------------------------------------------------------

Joelle AAFC ------------------------------------------------------------

Joelle NCBI ------------------------------------------------------------

Joelle phyto ------------------------------------------------------------

Blaine Creek GTTCGATGGTCCATACTTCAGCTGGAAGGTTACGCCATTACACATACGGGAGAGATATTT

CAM 241 GTTCGATGGTCCATACTTCAGCTGGAAGGTTACGCCATTACACATACGGGAGAGATATTT

CN 119294 ------------------------------------------------------------

CN 120025 ------------------------------------------------------------

CN 120013 ------------------------------------------------------------

CS17CS1133 ------------------------------------------------------------

CN 120030 TAGATCCTTTGCGGTAATGAGTTTCTTTTTAGAAACAGTTTC-TTTTTTACTAAACTAAG

CO46 NCBI ------------------------------------------------------------

DH55 ref genome ------------------------------------------------------------

Hoga ------------------------------------------------------------

CAM 236 ------------------------------------------------------------

09-CS0040 ------------------------------------------------------------

CN 119205 ------------------------------------------------------------

Yellowstone ------------------------------------------------------------

CN 120017 ------------------------------------------------------------

CN 119300 ------------------------------------------------------------

Jasper ------------------------------------------------------------

CN 120027 ------------------------------------------------------------

CN 113754 ------------------------------------------------------------

Joelle AAFC ------------------------------------------------------------

Joelle NCBI ------------------------------------------------------------

Joelle phyto ------------------------------------------------------------

Blaine Creek TAGATCCTTTGCGGTAATGAGTTTCTTTTTAGAAACAGTTTCTTTTTTTACTAAACTAAG

CAM 241 TAGATCCTTTGCGGTAATGAGTTTCTTTTTAGAAACAGTTTCTTTTTTTACTAAACTAAG

CN 119294 ------------------------------------------------------------

CN 120025 ------------------------------------------------------------

CN 120013 ------------------------------------------------------------

CS17CS1133 ------------------------------------------------------------

CN 120030 TTAATGAGTTTGTCTTTTGTTTACTTACTTGACACTTACTTTTACATTTTTTTACTGTGT

CO46 NCBI ------------------------------------------------------------

DH55 ref genome ------------------------------------------------------------

Hoga ------------------------------------------------------------

CAM 236 ------------------------------------------------------------

09-CS0040 ------------------------------------------------------------

CN 119205 ------------------------------------------------------------

Yellowstone ------------------------------------------------------------

CN 120017 ------------------------------------------------------------

CN 119300 ------------------------------------------------------------

Jasper ------------------------------------------------------------

CN 120027 ------------------------------------------------------------

CN 113754 ------------------------------------------------------------

Joelle AAFC ------------------------------------------------------------

Joelle NCBI ------------------------------------------------------------

Joelle phyto ------------------------------------------------------------

Blaine Creek TTAATGAGTTTGTCTTTTGTTTACTTACTTGACACTTACTTTTACATTTTTTTACTGTGT

CAM 241 TTAATGAGTTTGTCTTTTGTTTACTTACTTGACACTTACTTTTACATTTTTTTACTGTGT

CN 119294 ------------------------------------------------------------

CN 120025 ------------------------------------------------------------

CN 120013 ------------------------------------------------------------

CS17CS1133 ------------------------------------------------------------

CN 120030 AGAGGAAATACAATTGGGATGTTGCGATTACGGAGCTTGTGCGAGAAGGATTTGTGAAGA

CO46 NCBI ------------------------------------------------------------

DH55 ref genome ------------------------------------------------------------

Hoga ------------------------------------------------------------

CAM 236 ------------------------------------------------------------

09-CS0040 ------------------------------------------------------------

CN 119205 ------------------------------------------------------------

Yellowstone ------------------------------------------------------------

CN 120017 ------------------------------------------------------------

CN 119300 ------------------------------------------------------------

Jasper ------------------------------------------------------------

CN 120027 ------------------------------------------------------------

CN 113754 ------------------------------------------------------------

Joelle AAFC ------------------------------------------------------------

Joelle NCBI ------------------------------------------------------------

Joelle phyto ------------------------------------------------------------

Blaine Creek AGAGGAAATACAATTGGGATGTTGCGATTACGGAGCTTGTGCGAGAAGGATTTGTGAAGA

CAM 241 AGAGGAAATACAATTGGGATGTTGCGATTACGGAGCTTGTGCGAGAAGGATTTGTGAAGA

CN 119294 ------------------------------------------------------------

CN 120025 ------------------------------------------------------------

CN 120013 ------------------------------------------------------------

CS17CS1133 ------------------------------------------------------------

CN 120030 TAGCGAAAAACAGAATGAAAGGAATTGTAAGTCAAGCAAAGACATATGATGAGCCACCGA

CO46 NCBI ------------------------------------------------------------

DH55 ref genome ------------------------------------------------------------

Hoga ------------------------------------------------------------

CAM 236 ------------------------------------------------------------

09-CS0040 ------------------------------------------------------------

CN 119205 ------------------------------------------------------------

Yellowstone ------------------------------------------------------------

CN 120017 ------------------------------------------------------------

CN 119300 ------------------------------------------------------------

Jasper ------------------------------------------------------------

CN 120027 ------------------------------------------------------------

CN 113754 ------------------------------------------------------------

Joelle AAFC ------------------------------------------------------------

Joelle NCBI ------------------------------------------------------------

Joelle phyto ------------------------------------------------------------

Blaine Creek TAGCGAAAAACAGAATGAAAGGAATTGTAAGTCAAGCAAAGACATATGATGAGCCACCGA

CAM 241 TAGCGAAAAACGGAATGAAAGGAATTGTAAGTCAAGCAAAGACATATGATGAGCCACCGA

CN 119294 ------------------------------------------------------------

CN 120025 ------------------------------------------------------------

CN 120013 ------------------------------------------------------------

CS17CS1133 ------------------------------------------------------------

CN 120030 TTTGGATCAATCCTACACTTTGGTTACAAATGTGGGAGCATTGGGACACACCCGAAGCTA

CO46 NCBI ------------------------------------------------------------

DH55 ref genome ------------------------------------------------------------

Hoga ------------------------------------------------------------

CAM 236 ------------------------------------------------------------

09-CS0040 ------------------------------------------------------------

CN 119205 ------------------------------------------------------------

Yellowstone ------------------------------------------------------------

CN 120017 ------------------------------------------------------------

CN 119300 ------------------------------------------------------------

Jasper ------------------------------------------------------------

CN 120027 ------------------------------------------------------------

CN 113754 ------------------------------------------------------------

Joelle AAFC ------------------------------------------------------------

Joelle NCBI ------------------------------------------------------------

Joelle phyto ------------------------------------------------------------

Blaine Creek TTTGGATCAATCCTACACTTTGGTTACAAATGTGGGAGCATTGGGACACACCCGAAGCTA

CAM 241 TTTGGATCAATCCTACACTTTGGTTACAAATGTGGGAGCATTGGGACACACCCGAAGCTA

CN 119294 ------------------------------------------------------------

CN 120025 ------------------------------------------------------------

CN 120013 ------------------------------------------------------------

CS17CS1133 ------------------------------------------------------------

CN 120030 AGGAGAAGAGCTCTAATGCATCCCAAGCCCGTAATTC-GATCGTGACGGTCTTGGAATCC

CO46 NCBI ------------------------------------------------------------

DH55 ref genome ------------------------------------------------------------

Hoga ------------------------------------------------------------

CAM 236 ------------------------------------------------------------

09-CS0040 ------------------------------------------------------------

CN 119205 ------------------------------------------------------------

Yellowstone ------------------------------------------------------------

CN 120017 ------------------------------------------------------------

CN 119300 ------------------------------------------------------------

Jasper ------------------------------------------------------------

CN 120027 ------------------------------------------------------------

CN 113754 ------------------------------------------------------------

Joelle AAFC ------------------------------------------------------------

Joelle NCBI ------------------------------------------------------------

Joelle phyto ------------------------------------------------------------

Blaine Creek AGGAGAAGAGCTCTAATGCATCCCAAGCCCGTAATTCTGATCGTGACGGTCTTGGAATCC

CAM 241 AGGAGAAGAGCTCTAATGCATCCCAAGCCCGTAATTCTGATCGTGACGGTCTTGGAATCC

CN 119294 ------------------------------------------------------------

CN 120025 ------------------------------------------------------------

CN 120013 ------------------------------------------------------------

CS17CS1133 ------------------------------------------------------------

CN 120030 ACAAACATCTATCCGGCCAGAAATCTTACTTGCGAATTCAACAAGAGTTGGTAAATGCTC

CO46 NCBI ------------------------------------------------------------

DH55 ref genome ------------------------------------------------------------

Hoga ------------------------------------------------------------

CAM 236 ------------------------------------------------------------

09-CS0040 ------------------------------------------------------------

CN 119205 ------------------------------------------------------------

Yellowstone ------------------------------------------------------------

CN 120017 ------------------------------------------------------------

CN 119300 ------------------------------------------------------------

Jasper ------------------------------------------------------------

CN 120027 ------------------------------------------------------------

CN 113754 ------------------------------------------------------------

Joelle AAFC ------------------------------------------------------------

Joelle NCBI ------------------------------------------------------------

Joelle phyto ------------------------------------------------------------

Blaine Creek ACAAACATCTATCCGGCCAGAAATCTTACTTGCGAATTCAACAAGAGTTGGTAAATGCTC

CAM 241 ACAAACATCTATCCGGCCAGAAATCTTACTTGCGAATTCAACAAGAGTTGGTAAATGCTC

CN 119294 ------------------------------------------------------------

CN 120025 ------------------------------------------------------------

CN 120013 ------------------------------------------------------------

CS17CS1133 ------------------------------------------------------------

CN 120030 TAAACTAACTCTAATTATTTCATTTAAATAGTTTAGTATCCACAATTAACATTCTTTCTA

CO46 NCBI ------------------------------------------------------------

DH55 ref genome ------------------------------------------------------------

Hoga ------------------------------------------------------------

CAM 236 ------------------------------------------------------------

09-CS0040 ------------------------------------------------------------

CN 119205 ------------------------------------------------------------

Yellowstone ------------------------------------------------------------

CN 120017 ------------------------------------------------------------

CN 119300 ------------------------------------------------------------

Jasper ------------------------------------------------------------

CN 120027 ------------------------------------------------------------

CN 113754 ------------------------------------------------------------

Joelle AAFC ------------------------------------------------------------

Joelle NCBI ------------------------------------------------------------

Joelle phyto ------------------------------------------------------------

Blaine Creek TAAACTAACTCTAATTATTTCATTTAAATAGTTTAGTATCCACAATTAACATTCTTTCTA

CAM 241 TAAACTAACTCTAATTATTTCATTTAAATAGTTTAGTATCCACAATTAACATTCTTTCTA

CN 119294 ------------------------------------------------------------

CN 120025 ------------------------------------------------------------

CN 120013 ------------------------------------------------------------

CS17CS1133 ------------------------------------------------------------

CN 120030 TCCATTGTAGGAAGAAAAGTTGGGACGTCCTGTCTCGCTTGGAGAGGTGTTTGTCGCAAC

CO46 NCBI ------------------------------------------------------------

DH55 ref genome ------------------------------------------------------------

Hoga ------------------------------------------------------------

CAM 236 ------------------------------------------------------------

09-CS0040 ------------------------------------------------------------

CN 119205 ------------------------------------------------------------

Yellowstone ------------------------------------------------------------

CN 120017 ------------------------------------------------------------

CN 119300 ------------------------------------------------------------

Jasper ------------------------------------------------------------

CN 120027 ------------------------------------------------------------

CN 113754 ------------------------------------------------------------

Joelle AAFC ------------------------------------------------------------

Joelle NCBI ------------------------------------------------------------

Joelle phyto ------------------------------------------------------------

Blaine Creek TCCATTGTAGGAAGAAAAGTTGGGACGTCCTGTCTCGCTTGGAGAGGTGTTTGTCGCAAC

CAM 241 TCCATTGTAGGAAGAAAAGTTGGGACGTCCTGTCTCGCTTGGAGAGGTGTTTGTCGCAAC

CN 119294 ------------------------------------------------------------

CN 120025 ------------------------------------------------------------

CN 120013 ------------------------------------------------------------

CS17CS1133 ------------------------------------------------------------

CN 120030 ACATACAAAGGC-AGATGGAAGTTTTGTTGATCAGAAGGCCAAACAAGTTGTGGAGACAT

CO46 NCBI ------------------------------------------------------------

DH55 ref genome ------------------------------------------------------------

Hoga ------------------------------------------------------------

CAM 236 ------------------------------------------------------------

09-CS0040 ------------------------------------------------------------

CN 119205 ------------------------------------------------------------

Yellowstone ------------------------------------------------------------

CN 120017 ------------------------------------------------------------

CN 119300 ------------------------------------------------------------

Jasper ------------------------------------------------------------

CN 120027 ------------------------------------------------------------

CN 113754 ------------------------------------------------------------

Joelle AAFC ------------------------------------------------------------

Joelle NCBI ------------------------------------------------------------

Joelle phyto ------------------------------------------------------------

Blaine Creek ACATACAAAGGC-AGATGGAAGTTTTGTTGATCAGAAGGCCAAACAAGTTGTGGAGACAT

CAM 241 ACATACAAAGGCGAGATGGAAGTTTTGTTGATCAGAAGGCCAAACAAGTTGTGGAGACAT

CN 119294 ------------------------------------------------------------

CN 120025 ------------------------------------------------------------

CN 120013 ------------------------------------------------------------

CS17CS1133 ------------------------------------------------------------

CN 120030 ATGAGAAGAACATAGAGGAGGTAATGTCTCAAATGGAGTCTGATGTTAC-AGACCACTCC

CO46 NCBI ------------------------------------------------------------

DH55 ref genome ------------------------------------------------------------

Hoga ------------------------------------------------------------

CAM 236 ------------------------------------------------------------

09-CS0040 ------------------------------------------------------------

CN 119205 ------------------------------------------------------------

Yellowstone ------------------------------------------------------------

CN 120017 ------------------------------------------------------------

CN 119300 ------------------------------------------------------------

Jasper ------------------------------------------------------------

CN 120027 ------------------------------------------------------------

CN 113754 ------------------------------------------------------------

Joelle AAFC ------------------------------------------------------------

Joelle NCBI ------------------------------------------------------------

Joelle phyto ------------------------------------------------------------

Blaine Creek ATGAGAAGAACATAGAGGAGGTAATGTCTCAAATGGAGTCTGATGTTAC-AGACCACTCC

CAM 241 ATGAGAAGAACATAGAGGAGGTAATGTCTCAAATGGAGTCTGATGTTACGAGACCACTCC

CN 119294 ------------------------------------------------------------

CN 120025 ------------------------------------------------------------

CN 120013 ------------------------------------------------------------

CS17CS1133 ------------------------------------------------------------

CN 120030 TCGCCAGATTCTCCTCATGCAACCGTC-CTCCCCATTCAAGAAAAAGATGACATTTTCTT

CO46 NCBI ------------------------------------------------------------

DH55 ref genome ------------------------------------------------------------

Hoga ------------------------------------------------------------

CAM 236 ------------------------------------------------------------

09-CS0040 ------------------------------------------------------------

CN 119205 ------------------------------------------------------------

Yellowstone ------------------------------------------------------------

CN 120017 ------------------------------------------------------------

CN 119300 ------------------------------------------------------------

Jasper ------------------------------------------------------------

CN 120027 ------------------------------------------------------------

CN 113754 ------------------------------------------------------------

Joelle AAFC ------------------------------------------------------------

Joelle NCBI ------------------------------------------------------------

Joelle phyto ------------------------------------------------------------

Blaine Creek TCGCCAGATTCTCCTCATGCAACCGTCTCTCCCCATTCAAGAAAAAGATGACATTTTCTT

CAM 241 TCGCCGGATTCTCCTCATGCAACCGTC-CTCCCCATTCAAGAAAAAGATGACATTTTCTT

CN 119294 ------------------------------------------------------------

CN 120025 ------------------------------------------------------------

CN 120013 ------------------------------------------------------------

CS17CS1133 ------------------------------------------------------------

CN 120030 AAAGGTAATTTCCAGTACAATTGTATTTGTTGTGCCTATGATAATTGTCTTATTTTGCTA

CO46 NCBI ------------------------------------------------------------

DH55 ref genome ------------------------------------------------------------

Hoga ------------------------------------------------------------

CAM 236 ------------------------------------------------------------

09-CS0040 ------------------------------------------------------------

CN 119205 ------------------------------------------------------------

Yellowstone ------------------------------------------------------------

CN 120017 ------------------------------------------------------------

CN 119300 ------------------------------------------------------------

Jasper ------------------------------------------------------------

CN 120027 ------------------------------------------------------------

CN 113754 ------------------------------------------------------------

Joelle AAFC ------------------------------------------------------------

Joelle NCBI ------------------------------------------------------------

Joelle phyto ------------------------------------------------------------

Blaine Creek AAAGGTAATTTCCAGTACAATTGTATTTGTTGTGCCTATGATAATTGTCTTATTTTGCTA

CAM 241 AAAGGTAATTTCCAGTACAATTGTATTTGTTGTGCCTATGATAATTGTCTTATTTTGCTA

CN 119294 ------------------------------------------------------------

CN 120025 ------------------------------------------------------------

CN 120013 ------------------------------------------------------------

CS17CS1133 ------------------------------------------------------------

CN 120030 AGCTAAGTATCTGATTTTGATTGATGCTTAAGAATCCTTGATATTGATATACTTTTGATT

CO46 NCBI ------------------------------------------------------------

DH55 ref genome ------------------------------------------------------------

Hoga ------------------------------------------------------------

CAM 236 ------------------------------------------------------------

09-CS0040 ------------------------------------------------------------

CN 119205 ------------------------------------------------------------

Yellowstone ------------------------------------------------------------

CN 120017 ------------------------------------------------------------

CN 119300 ------------------------------------------------------------

Jasper ------------------------------------------------------------

CN 120027 ------------------------------------------------------------

CN 113754 ------------------------------------------------------------

Joelle AAFC ------------------------------------------------------------

Joelle NCBI ------------------------------------------------------------

Joelle phyto ------------------------------------------------------------

Blaine Creek AGCTAAGTATCTGATTTTGATTGATGCTTAAGAATCCTTGATATTGATATACTTTTGATT

CAM 241 AGCTAAGTATCTGATTTTGATTGATGCTTAAGAATCCTTGATATTGATATACTTTTGATT

CN 119294 ------------------------------------------------------------

CN 120025 ------------------------------------------------------------

CN 120013 ------------------------------------------------------------

CS17CS1133 ------------------------------------------------------------

CN 120030 GATGCTTGAGAATACTCGATGTTGATTATCTGGGTTGATATTATATTTCTATCTGGGTTT

CO46 NCBI ------------------------------------------------------------

DH55 ref genome ------------------------------------------------------------

Hoga ------------------------------------------------------------

CAM 236 ------------------------------------------------------------

09-CS0040 ------------------------------------------------------------

CN 119205 ------------------------------------------------------------

Yellowstone ------------------------------------------------------------

CN 120017 ------------------------------------------------------------

CN 119300 ------------------------------------------------------------

Jasper ------------------------------------------------------------

CN 120027 ------------------------------------------------------------

CN 113754 ------------------------------------------------------------

Joelle AAFC ------------------------------------------------------------

Joelle NCBI ------------------------------------------------------------

Joelle phyto ------------------------------------------------------------

Blaine Creek GATGCTTGAGAATACTCGATGTTGATTATCTGGGTTGATATTATATTTCTATCTGGGTTT

CAM 241 GATGCTTGAGAATACTCGATGTTGATTATCCGGGTTGATATTATATTTCTATCCGGGTTT

CN 119294 ------------------------------------------------------------

CN 120025 ------------------------------------------------------------

CN 120013 ------------------------------------------------------------

CS17CS1133 ------------------------------------------------------------

CN 120030 GTGTGATATTTCTGTTTGTTATGTTTGTTTTGTCAATGATCATCTCTTCTTTTGTCTTAG

CO46 NCBI ------------------------------------------------------------

DH55 ref genome ------------------------------------------------------------

Hoga ------------------------------------------------------------

CAM 236 ------------------------------------------------------------

09-CS0040 ------------------------------------------------------------

CN 119205 ------------------------------------------------------------

Yellowstone ------------------------------------------------------------

CN 120017 ------------------------------------------------------------

CN 119300 ------------------------------------------------------------

Jasper ------------------------------------------------------------

CN 120027 ------------------------------------------------------------

CN 113754 ------------------------------------------------------------

Joelle AAFC ------------------------------------------------------------

Joelle NCBI ------------------------------------------------------------

Joelle phyto ------------------------------------------------------------

Blaine Creek GTGTGATATTTCTGTTTGTTATGTTTGTTTTGTCAATGATCATCTCTTCTTTTGTCTTAG

CAM 241 GTGTGATATTTCTGTTTGTTATGTTTGTTTTGTCAATGATCATCTCTTCTTTTGTCTTAG

CN 119294 ------------------------------------------------------------

CN 120025 ------------------------------------------------------------

CN 120013 ------------------------------------------------------------

CS17CS1133 ------------------------------------------------------------

CN 120030 TGTACTGTAACGAATGCCAAGGGAACCCCCTTTGGACTTGGAAGTCTCTCGGAGATAATC

CO46 NCBI ------------------------------------------------------------

DH55 ref genome ------------------------------------------------------------

Hoga ------------------------------------------------------------

CAM 236 ------------------------------------------------------------

09-CS0040 ------------------------------------------------------------

CN 119205 ------------------------------------------------------------

Yellowstone ------------------------------------------------------------

CN 120017 ------------------------------------------------------------

CN 119300 ------------------------------------------------------------

Jasper ------------------------------------------------------------

CN 120027 ------------------------------------------------------------

CN 113754 ------------------------------------------------------------

Joelle AAFC ------------------------------------------------------------

Joelle NCBI ------------------------------------------------------------

Joelle phyto ------------------------------------------------------------

Blaine Creek TGTACTGTAACGAATGCCAAGGGAACCCCCTTTGGACTTGGAAGTCTCTCGGAGATAATC

CAM 241 TGTACTGTAACGAATGCCAAGGGAACCCCCTTTGGACTTGGAAGTCTCTCGGAGATAATC

CN 119294 ------------------------------------------------------------

CN 120025 ------------------------------------------------------------

CN 120013 ------------------------------------------------------------

CS17CS1133 ------------------------------------------------------------

CN 120030 ACCAAAGGGAAAAGAAAGGCTACTTATCCAAGCTCTAGTCCAGATTCGCTGTTAGAGATT

CO46 NCBI ------------------------------------------------------------

DH55 ref genome ------------------------------------------------------------

Hoga ------------------------------------------------------------

CAM 236 ------------------------------------------------------------

09-CS0040 ------------------------------------------------------------

CN 119205 ------------------------------------------------------------

Yellowstone ------------------------------------------------------------

CN 120017 ------------------------------------------------------------

CN 119300 ------------------------------------------------------------

Jasper ------------------------------------------------------------

CN 120027 ------------------------------------------------------------

CN 113754 ------------------------------------------------------------

Joelle AAFC ------------------------------------------------------------

Joelle NCBI ------------------------------------------------------------

Joelle phyto ------------------------------------------------------------

Blaine Creek ACCAAAGGGAAAAGAAAGGCTACTTATCCAAGCTCTAGTCCAGATTCGCTGTTAGAGATT

CAM 241 ACCAAAGGGAAAAGAAAGGCTACTTATCCAAGCTCTAGTCCAGATTCGCTGTTAGAGATT

CN 119294 ------------------------------------------------------------

CN 120025 ------------------------------------------------------------

CN 120013 ------------------------------------------------------------

CS17CS1133 ------------------------------------------------------------

CN 120030 CAAGAACAACTCCAGGTTGCTCGTCGTAAGCTCGCTGAGCAAGATGAAGAAAATGCTCGA

CO46 NCBI ------------------------------------------------------------

DH55 ref genome ------------------------------------------------------------

Hoga ------------------------------------------------------------

CAM 236 ------------------------------------------------------------

09-CS0040 ------------------------------------------------------------

CN 119205 ------------------------------------------------------------

Yellowstone ------------------------------------------------------------

CN 120017 ------------------------------------------------------------

CN 119300 ------------------------------------------------------------

Jasper ------------------------------------------------------------

CN 120027 ------------------------------------------------------------

CN 113754 ------------------------------------------------------------

Joelle AAFC ------------------------------------------------------------

Joelle NCBI ------------------------------------------------------------

Joelle phyto ------------------------------------------------------------

Blaine Creek CAAGAACAACTCCAGGTTGCTCGTCGTAAGCTCGCTGAGCAAGATGAAGAAAATGCTCGA

CAM 241 CAAGAACAACTCCAGGTTGCTCGTCGTAAGCTCGCTGAGCAAGATGAAGAAAATGCTCGA

CN 119294 ------------------------------------------------------------

CN 120025 ------------------------------------------------------------

CN 120013 ------------------------------------------------------------

CS17CS1133 ------------------------------------------------------------

CN 120030 CGTGCCAGAGAAGATGCTAGACGTGATCAGGAGCATCGTGACGCTCAAAACCGCATTGCA

CO46 NCBI ------------------------------------------------------------

DH55 ref genome ------------------------------------------------------------

Hoga ------------------------------------------------------------

CAM 236 ------------------------------------------------------------

09-CS0040 ------------------------------------------------------------

CN 119205 ------------------------------------------------------------

Yellowstone ------------------------------------------------------------

CN 120017 ------------------------------------------------------------

CN 119300 ------------------------------------------------------------

Jasper ------------------------------------------------------------

CN 120027 ------------------------------------------------------------

CN 113754 ------------------------------------------------------------

Joelle AAFC ------------------------------------------------------------

Joelle NCBI ------------------------------------------------------------

Joelle phyto ------------------------------------------------------------

Blaine Creek CGTGCCAGAGAAGATGCTAGACGTGATCAGGAGCATCGTGACGCTCAAAACCGCATTGCA

CAM 241 CGTGCCGGAGAAGATGCTAGACGTGATCAGGAGCATCGTGACGCTCAAAACCGCATTGCA

CN 119294 ------------------------------------------------------------

CN 120025 ------------------------------------------------------------

CN 120013 ------------------------------------------------------------

CS17CS1133 ------------------------------------------------------------

CN 120030 ACCTTGGAGATGCTCGTATCCTACTTGAAGACTAGTGATCCGGGGTTTGCAGAATTCTTG

CO46 NCBI ------------------------------------------------------------

DH55 ref genome ------------------------------------------------------------

Hoga ------------------------------------------------------------

CAM 236 ------------------------------------------------------------

09-CS0040 ------------------------------------------------------------

CN 119205 ------------------------------------------------------------

Yellowstone ------------------------------------------------------------

CN 120017 ------------------------------------------------------------

CN 119300 ------------------------------------------------------------

Jasper ------------------------------------------------------------

CN 120027 ------------------------------------------------------------

CN 113754 ------------------------------------------------------------

Joelle AAFC ------------------------------------------------------------

Joelle NCBI ------------------------------------------------------------

Joelle phyto ------------------------------------------------------------

Blaine Creek ACCTTGGAGATGCTCGTATCCTACTTGAAGACTAGTGATCCGGGGTTTGCAGAATTCTTG

CAM 241 ACCTTGGAGATGCTCGTATCCTACTTGAAGACTAGTGATCCGGGGTTTGCAGAATTCTTG

CN 119294 ------------------------------------------------------------

CN 120025 ------------------------------------------------------------

CN 120013 ------------------------------------------------------------

CS17CS1133 ------------------------------------------------------------

CN 120030 TCTACTCAACCACCAGCCAATGCGCCCTTAACCACAGCAGCAAGCAGCAACCAACATCAC

CO46 NCBI ------------------------------------------------------------

DH55 ref genome ------------------------------------------------------------

Hoga ------------------------------------------------------------

CAM 236 ------------------------------------------------------------

09-CS0040 ------------------------------------------------------------

CN 119205 ------------------------------------------------------------

Yellowstone ------------------------------------------------------------

CN 120017 ------------------------------------------------------------

CN 119300 ------------------------------------------------------------

Jasper ------------------------------------------------------------

CN 120027 ------------------------------------------------------------

CN 113754 ------------------------------------------------------------

Joelle AAFC ------------------------------------------------------------

Joelle NCBI ------------------------------------------------------------

Joelle phyto ------------------------------------------------------------

Blaine Creek TCTACTCAACCACCAGCCAATGCGCCATTAACCACAGCAGC-AGCAGCAACCAACATCAC

CAM 241 TCTACTCAACCACCAGCCAATGCGCCATTAACCACAGCAGC-AGCAGCAACCAACATCAC

CN 119294 ------------------------------------------------------------

CN 120025 ------------------------------------------------------------

CN 120013 ------------------------------------------------------------

CS17CS1133 ------------------------------------------------------------

CN 120030 TCCAGCCACTGCGACCAATGTCAACCAACCTGCAACAACCACTTCAGGAACTAGTTCACC

CO46 NCBI ------------------------------------------------------------

DH55 ref genome ------------------------------------------------------------

Hoga ------------------------------------------------------------

CAM 236 ------------------------------------------------------------

09-CS0040 ------------------------------------------------------------

CN 119205 ------------------------------------------------------------

Yellowstone ------------------------------------------------------------

CN 120017 ------------------------------------------------------------

CN 119300 ------------------------------------------------------------

Jasper ------------------------------------------------------------

CN 120027 ------------------------------------------------------------

CN 113754 ------------------------------------------------------------

Joelle AAFC ------------------------------------------------------------

Joelle NCBI ------------------------------------------------------------

Joelle phyto ------------------------------------------------------------

Blaine Creek TCCAGCCACTGCGACCAATGTCAACCAACCTGCAACAACCACTTCAGGAACTAGTTCACC

CAM 241 TCCAGCCACTGCGACCAATGTCAACCAACCTGCAACAACCACTTCAGGAACTAGTTCACC

CN 119294 ------------------------------------------------------------

CN 120025 ------------------------------------------------------------

CN 120013 ------------------------------------------------------------

CS17CS1133 ------------------------------------------------------------

CN 120030 ATCGATCGCGATCGCACCAACCACTTCACCTGCGACTGCACCAACCA-----CTTCACCT

CO46 NCBI ------------------------------------------------------------

DH55 ref genome ------------------------------------------------------------

Hoga ------------------------------------------------------------

CAM 236 ------------------------------------------------------------

09-CS0040 ------------------------------------------------------------

CN 119205 ------------------------------------------------------------

Yellowstone ------------------------------------------------------------

CN 120017 ------------------------------------------------------------

CN 119300 ------------------------------------------------------------

Jasper ------------------------------------------------------------

CN 120027 ------------------------------------------------------------

CN 113754 ------------------------------------------------------------

Joelle AAFC ------------------------------------------------------------

Joelle NCBI ------------------------------------------------------------

Joelle phyto ------------------------------------------------------------

Blaine Creek ATCGATCGCGACTGCACCAACCACTTCACCTGCGCGACTCGCTACCAACCACTTCACCTT

CAM 241 ATCGATCGCGACTGCACCAACCACTTCACCTGCGA----TCGCACCAACCACTTCACCTT

CN 119294 ------------------------------------------------------------

CN 120025 ------------------------------------------------------------

CN 120013 ------------------------------------------------------------

CS17CS1133 ------------------------------------------------------------

CN 120030 TTGTCAGTATCATCCTCTCATGCTTAACAAACTAATTCTATCTCTCTTAAGCCTCTATAA

CO46 NCBI ------------------------------------------------------------

DH55 ref genome ------------------------------------------------------------

Hoga ------------------------------------------------------------

CAM 236 ------------------------------------------------------------

09-CS0040 ------------------------------------------------------------

CN 119205 ------------------------------------------------------------

Yellowstone ------------------------------------------------------------

CN 120017 ------------------------------------------------------------

CN 119300 ------------------------------------------------------------

Jasper ------------------------------------------------------------

CN 120027 ------------------------------------------------------------

CN 113754 ------------------------------------------------------------

Joelle AAFC ------------------------------------------------------------

Joelle NCBI ------------------------------------------------------------

Joelle phyto ------------------------------------------------------------

Blaine Creek TGTC-AGTATCATCCTCTCATGCTTAACAAACTAATTCTATCTCTCTTAAGCCTCTATAA

CAM 241 TGTCGAGTATCATCCTCTCATGCTTAACAAACTAATTCTATCTCTCTTAAGCCTCTATAA

CN 119294 ------------------------------------------------------------

CN 120025 ------------------------------------------------------------

CN 120013 ------------------------------------------------------------

CS17CS1133 ------------------------------------------------------------

CN 120030 CTATTTTTTGGATTGTGTAGTAGTGTTTCTAGATCCTTAGCCTACTAAAACTATGACTAA

CO46 NCBI ------------------------------------------------------------

DH55 ref genome ------------------------------------------------------------

Hoga ------------------------------------------------------------

CAM 236 ------------------------------------------------------------

09-CS0040 ------------------------------------------------------------

CN 119205 ------------------------------------------------------------

Yellowstone ------------------------------------------------------------

CN 120017 ------------------------------------------------------------

CN 119300 ------------------------------------------------------------

Jasper ------------------------------------------------------------

CN 120027 ------------------------------------------------------------

CN 113754 ------------------------------------------------------------

Joelle AAFC ------------------------------------------------------------

Joelle NCBI ------------------------------------------------------------

Joelle phyto ------------------------------------------------------------

Blaine Creek CTATTTTTTGGATTGTGTAGTAGTGTTTCTAGATCCTTAGCCTACTAAAACTATGACTAA

CAM 241 CTATTTTTTGGATTGTGTAGTAGTGTTTCTAGATCCTTAGCCTACTAAAACTATGACTAA

CN 119294 ------------------------------------------------------------

CN 120025 ------------------------------------------------------------

CN 120013 ------------------------------------------------------------

CS17CS1133 ------------------------------------------------------------

CN 120030 TATATGATCTAGAATGATTTGTATGTTTTTTGTTAAGATCATTCAAATCAGGATTTTAAT

CO46 NCBI ------------------------------------------------------------

DH55 ref genome ------------------------------------------------------------

Hoga ------------------------------------------------------------

CAM 236 ------------------------------------------------------------

09-CS0040 ------------------------------------------------------------

CN 119205 ------------------------------------------------------------

Yellowstone ------------------------------------------------------------

CN 120017 ------------------------------------------------------------

CN 119300 ------------------------------------------------------------

Jasper ------------------------------------------------------------

CN 120027 ------------------------------------------------------------

CN 113754 ------------------------------------------------------------

Joelle AAFC ------------------------------------------------------------

Joelle NCBI ------------------------------------------------------------

Joelle phyto ------------------------------------------------------------

Blaine Creek TATATGATCTAGAATGATTTGTATGTTTTTTGTTAAGATCATTCAAATCAGGATTTTAAT

CAM 241 TATATGATCTAGAATGATTTGTATGTTTTTTGTTAAGATCATTCAAATCAGGATTTTAAT

CN 119294 ------------------------------------------------------------

CN 120025 ------------------------------------------------------------

CN 120013 ------------------------------------------------------------

CS17CS1133 ------------------------------------------------------------

CN 120030 TTAAATTCAGGTTTTTTTTTCAAAATTTATTGTATACTGTCCCAAATTACTAGGTATATT

CO46 NCBI ------------------------------------------------------------

DH55 ref genome ------------------------------------------------------------

Hoga ------------------------------------------------------------

CAM 236 ------------------------------------------------------------

09-CS0040 ------------------------------------------------------------

CN 119205 ------------------------------------------------------------

Yellowstone ------------------------------------------------------------

CN 120017 ------------------------------------------------------------

CN 119300 ------------------------------------------------------------

Jasper ------------------------------------------------------------

CN 120027 ------------------------------------------------------------

CN 113754 ------------------------------------------------------------

Joelle AAFC ------------------------------------------------------------

Joelle NCBI ------------------------------------------------------------

Joelle phyto ------------------------------------------------------------

Blaine Creek TTAAATTCAGG-TTTTTTTTCAAAATTTATTGTATACTGTCCCAAATTACTAGGTATATT

CAM 241 TTAAATTCAGGTTTTTTTTTCAAAATTTATTGTATACTGTCCCAAATTACTAGGTATATT

CN 119294 ------------------------------------------------------------

CN 120025 ------------------------------------------------------------

CN 120013 ------------------------------------------------------------

CS17CS1133 ------------------------------------------------------------

CN 120030 TTCACTGATTTGCGACCATTTATAATTTAAATAAACACAAACAAAGTACCAAATTCGTTG

CO46 NCBI ------------------------------------------------------------

DH55 ref genome ------------------------------------------------------------

Hoga ------------------------------------------------------------

CAM 236 ------------------------------------------------------------

09-CS0040 ------------------------------------------------------------

CN 119205 ------------------------------------------------------------

Yellowstone ------------------------------------------------------------

CN 120017 ------------------------------------------------------------

CN 119300 ------------------------------------------------------------

Jasper ------------------------------------------------------------

CN 120027 ------------------------------------------------------------

CN 113754 ------------------------------------------------------------

Joelle AAFC ------------------------------------------------------------

Joelle NCBI ------------------------------------------------------------

Joelle phyto ------------------------------------------------------------

Blaine Creek TTCACTGATTTGCGACCATTTATAATTTAAATAAACACAAACAAAGTACCAAATTCGTTG

CAM 241 TTCACTGATTTGCGACCATTTATAATTTAAATAAACACAAACAAAGTACCAAATTCGTTG

CN 119294 ------------------------------------------------------------

CN 120025 ------------------------------------------------------------

CN 120013 ------------------------------------------------------------

CS17CS1133 ------------------------------------------------------------

CN 120030 TTTATATGTCACGATTTTGGGACTAAACAAAATTAGTCTTCAG-TCCGTCACAAACAATT

CO46 NCBI ------------------------------------------------------------

DH55 ref genome ------------------------------------------------------------

Hoga ------------------------------------------------------------

CAM 236 ------------------------------------------------------------

09-CS0040 ------------------------------------------------------------

CN 119205 ------------------------------------------------------------

Yellowstone ------------------------------------------------------------

CN 120017 ------------------------------------------------------------

CN 119300 ------------------------------------------------------------

Jasper ------------------------------------------------------------

CN 120027 ------------------------------------------------------------

CN 113754 ------------------------------------------------------------

Joelle AAFC ------------------------------------------------------------

Joelle NCBI ------------------------------------------------------------

Joelle phyto ------------------------------------------------------------

Blaine Creek TTTATATGTCACGATTTTGGGACTAAACAAAATTAGTCTTCAG-TCCGTCACAAACAATT

CAM 241 TTTATATGTCACGATTTTGGGACTAAACAAAATTAGTCTTCAGATCCGTCACAAACAATT

CN 119294 ------------------------------------------------------------

CN 120025 ------------------------------------------------------------

CN 120013 ------------------------------------------------------------

CS17CS1133 ------------------------------------------------------------

CN 120030 CTGACTCCTTTGCGACTACCTTGTGACGAAACAGCGACTACTTTAGGACCAAATTTCAGA

CO46 NCBI ------------------------------------------------------------

DH55 ref genome ------------------------------------------------------------

Hoga ------------------------------------------------------------

CAM 236 ------------------------------------------------------------

09-CS0040 ------------------------------------------------------------

CN 119205 ------------------------------------------------------------

Yellowstone ------------------------------------------------------------

CN 120017 ------------------------------------------------------------

CN 119300 ------------------------------------------------------------

Jasper ------------------------------------------------------------

CN 120027 ------------------------------------------------------------

CN 113754 ------------------------------------------------------------

Joelle AAFC ------------------------------------------------------------

Joelle NCBI ------------------------------------------------------------

Joelle phyto ------------------------------------------------------------

Blaine Creek CTGACTCCTTTGCGACTACCTTGTGACGAAACAGCAACTACTTTAGGACCAAATTTCAGA

CAM 241 CTGACTCCTTTGCGACTACCTTGTGACGAAACAGCGACTACTTTAGGACCAAATTTCAGA

CN 119294 ------------------------------------------------------------

CN 120025 ------------------------------------------------------------

CN 120013 ------------------------------------------------------------

CS17CS1133 ------------------------------------------------------------

CN 120030 TTTTTAAAGTTTACTCGTGGTATTAAGTCAGTCTTCCAAACGTAGCTAATTTGTGACTAC

CO46 NCBI ------------------------------------------------------------

DH55 ref genome ------------------------------------------------------------

Hoga ------------------------------------------------------------

CAM 236 ------------------------------------------------------------

09-CS0040 ------------------------------------------------------------

CN 119205 ------------------------------------------------------------

Yellowstone ------------------------------------------------------------

CN 120017 ------------------------------------------------------------

CN 119300 ------------------------------------------------------------

Jasper ------------------------------------------------------------

CN 120027 ------------------------------------------------------------

CN 113754 ------------------------------------------------------------

Joelle AAFC ------------------------------------------------------------

Joelle NCBI ------------------------------------------------------------

Joelle phyto ------------------------------------------------------------

Blaine Creek TTTTTAAAGTTTACT-GTGGTATTAAGTCAGTCTTCCAAACGTAGCTAATTTGTGACTAC

CAM 241 TTTTTAAAGTTTACT-GTGGTATTAAGTCAGTCTTCCAAACGTAGCTAATTTGTGACTAC

CN 119294 ------------------------------------------------------------

CN 120025 ------------------------------------------------------------

CN 120013 ------------------------------------------------------------

CS17CS1133 ------------------------------------------------------------

CN 120030 CATGTCGTCCGAAAGAAGTCGTCTATTACGACTGCCAAGGCGAGTCACTATACCGTCGTT

CO46 NCBI ------------------------------------------------------------

DH55 ref genome ------------------------------------------------------------

Hoga ------------------------------------------------------------

CAM 236 ------------------------------------------------------------

09-CS0040 ------------------------------------------------------------

CN 119205 ------------------------------------------------------------

Yellowstone ------------------------------------------------------------

CN 120017 ------------------------------------------------------------

CN 119300 ------------------------------------------------------------

Jasper ------------------------------------------------------------

CN 120027 ------------------------------------------------------------

CN 113754 ------------------------------------------------------------

Joelle AAFC ------------------------------------------------------------

Joelle NCBI ------------------------------------------------------------

Joelle phyto ------------------------------------------------------------

Blaine Creek CATGTCGTCTGAAAGAAGTCGTCTATTACGACTGCCAAGGCGAGTCACTATACCGTCGTT

CAM 241 CATGTCGTCCGAAAGAAGTCGTCTATTACGACTGCCAAGGCGAGTCACTATACCGTCGTT

CN 119294 ------------------------------------------------------------

CN 120025 ------------------------------------------------------------

CN 120013 ------------------------------------------------------------

CS17CS1133 ------------------------------------------------------------

CN 120030 AATTACGACGATATATTCCGGTCTGTCCGTAGTCGCCAATTACGATCTACGCCAGTGAGT

CO46 NCBI ------------------------------------------------------------

DH55 ref genome ------------------------------------------------------------

Hoga ------------------------------------------------------------

CAM 236 ------------------------------------------------------------

09-CS0040 ------------------------------------------------------------

CN 119205 ------------------------------------------------------------

Yellowstone ------------------------------------------------------------

CN 120017 ------------------------------------------------------------

CN 119300 ------------------------------------------------------------

Jasper ------------------------------------------------------------

CN 120027 ------------------------------------------------------------

CN 113754 ------------------------------------------------------------

Joelle AAFC ------------------------------------------------------------

Joelle NCBI ------------------------------------------------------------

Joelle phyto ------------------------------------------------------------

Blaine Creek AATTACGACGATATATTCCGGTCTGTCCGTAGTCGCCAATTACGA-CTGCGCCAGTGAGT

CAM 241 AATTACGACGATATATTCCGGTCTGTCCGTAGTCGCCAATTACGA-CTGCGCCAGTGAGT

CN 119294 ------------------------------------------------------------

CN 120025 ------------------------------------------------------------

CN 120013 ------------------------------------------------------------

CS17CS1133 ------------------------------------------------------------

CN 120030 GACTATAGAGTCGTTCATTACGACGGCAGGTTTC-GTCTGTGTGTAGTCGCTAATTAACG

CO46 NCBI ------------------------------------------------------------

DH55 ref genome ------------------------------------------------------------

Hoga ------------------------------------------------------------

CAM 236 ------------------------------------------------------------

09-CS0040 ------------------------------------------------------------

CN 119205 ------------------------------------------------------------

Yellowstone ------------------------------------------------------------

CN 120017 ------------------------------------------------------------

CN 119300 ------------------------------------------------------------

Jasper ------------------------------------------------------------

CN 120027 ------------------------------------------------------------

CN 113754 ------------------------------------------------------------

Joelle AAFC ------------------------------------------------------------

Joelle NCBI ------------------------------------------------------------

Joelle phyto ------------------------------------------------------------

Blaine Creek GACTATAGAGTCGTTCATTACGACGGCAGGTTTCGGTCTGTGTGTAGTCGCTAATTAACG

CAM 241 GACTATAGAGTCGTTCATTACGACGGCAGGTTTCGGTCTGTGTGTAGTCGCTAATTAACG

CN 119294 ------------------------------------------------------------

CN 120025 ------------------------------------------------------------

CN 120013 ------------------------------------------------------------

CS17CS1133 ------------------------------------------------------------

CN 120030 ACCACAATCCTTGTCTCGAATGCGTCGCAAATAAGGGACTGGTTTGCGACCAAAAAAATT

CO46 NCBI ------------------------------------------------------------

DH55 ref genome ------------------------------------------------------------

Hoga ------------------------------------------------------------

CAM 236 ------------------------------------------------------------

09-CS0040 ------------------------------------------------------------

CN 119205 ------------------------------------------------------------

Yellowstone ------------------------------------------------------------

CN 120017 ------------------------------------------------------------

CN 119300 ------------------------------------------------------------

Jasper ------------------------------------------------------------

CN 120027 ------------------------------------------------------------

CN 113754 ------------------------------------------------------------

Joelle AAFC ------------------------------------------------------------

Joelle NCBI ------------------------------------------------------------

Joelle phyto ------------------------------------------------------------

Blaine Creek ACCACAATCCTTGTCTCGAATGCGTCGCAAATAAGGGACTGGTTTGCGACCAAAAAAATT

CAM 241 ACCACAATCCTTGTCTCGAATGCGTCGCAAATAAGGGACTGGTTTGCGACCAAAAAAATT

CN 119294 ------------------------------------------------------------

CN 120025 ------------------------------------------------------------

CN 120013 ------------------------------------------------------------

CS17CS1133 ------------------------------------------------------------

CN 120030 TGCGACCAAATATTTGCGACCAAATATAACAGTCCTTCAATTGTCGCACATACCGACTTA

CO46 NCBI ------------------------------------------------------------

DH55 ref genome ------------------------------------------------------------

Hoga ------------------------------------------------------------

CAM 236 ------------------------------------------------------------

09-CS0040 ------------------------------------------------------------

CN 119205 ------------------------------------------------------------

Yellowstone ------------------------------------------------------------

CN 120017 ------------------------------------------------------------

CN 119300 ------------------------------------------------------------

Jasper ------------------------------------------------------------

CN 120027 ------------------------------------------------------------

CN 113754 ------------------------------------------------------------

Joelle AAFC ------------------------------------------------------------

Joelle NCBI ------------------------------------------------------------

Joelle phyto ------------------------------------------------------------

Blaine Creek TGCGACCAAATATTTGCGACCAAATATAACAGTCCTTCAATTGTCGCACATACCGACTTA

CAM 241 TGCGACCAAATATTTGCGACCAAATATAACAGTCCTTCAATTGTCGCACATACCGACTTA

CN 119294 ------------------------------------------------------------

CN 120025 ------------------------------------------------------TTTATT

CN 120013 ------------------------------------------------------TTTATT

CS17CS1133 ------------------------------------------------------TTTATT

CN 120030 GCGACTGATTTGAGACGATTCCGGTAGTCCCAAACGCCGTGTTTTCGTGTAGTGTTTATT

CO46 NCBI ------------------------------------------------------TTTATT

DH55 ref genome ------------------------------------------------------TTTATT

Hoga ------------------------------------------------------TTTATT

CAM 236 ------------------------------------------------------TTTATT

09-CS0040 ------------------------------------------------------TTTATT

CN 119205 ------------------------------------------------------TTTATT

Yellowstone ------------------------------------------------------TTTATT

CN 120017 ------------------------------------------------------TTTATT

CN 119300 ------------------------------------------------------TTTATT

Jasper ------------------------------------------------------TTTATT

CN 120027 ------------------------------------------------------TTTATT

CN 113754 ------------------------------------------------------TTTATT

Joelle AAFC ------------------------------------------------------TTTATT

Joelle NCBI ------------------------------------------------------TTTATT

Joelle phyto ------------------------------------------------------TTTATT

Blaine Creek GCGACTGATTTGAGACGATTCCGGTAGTCCCAAACGCCGTGTTTTCGTGTAGTGTTTATT

CAM 241 GCGACTGATTTGAGACGATTCCGGTAGTCCCAAACGCCGTGTTTTCGTGTAGTGTTTATT

CN 119294 ------------------------------------------------------TTTATT

******

CN 120025 ATCCTAACATACTTTTCTTTTTTTTCATCTCTCCAGCCTGGTCAAGATCCTTGATCGATA

CN 120013 ATCCTAACATACTTTTCTTTTTTGTCATCTCTCCAGCCTGGTGAAGATCCTTGATCGATA

CS17CS1133 ATCCTAACATACTTTTCTTTTTTGTCATCTCTCCAGCCTGGTGAAGATCCTTGATCGATA

CN 120030 ATCCTAACATACTTTTCTTTTTTGTCATCTCTCCAGCCTGGTGAAGATCCTTGATCGATA

CO46 NCBI ATCCTAACATACTTTTCTTTTTTGTCATCTCTCCAGCCTGGTGAAGATCCTTGATCGATA

DH55 ref genome ATCCTAACATACTTTTCTTTTTTGTCATCTCTCCAGCCTGGTGAAGATCCTTGATCGATA

Hoga ATCCTAACATACTTTTCTTTTTTGTCATCTCTCCAGCCTGGTGAAGATCCTTGATCGATA

CAM 236 ATCCTAACATACTTTTCTTTTTTGTCATCTCTCCAGCCTGGTGAAGATCCTTGATCGATA

09-CS0040 ATCCTAACATACTTTTCTTTTTTGTCATCTCTCCAGCCTGGTGAAGATCCTTGATCGATA

CN 119205 ATCCTAACATACTTTTCTTTTTTGTCATCTCTCCAGCCTGGTGAAGATCCTTGATCGATA

Yellowstone ATCCTAACATACTTTTCTTTTTTGTCATCTCTCCAGCCTGGTGAAGATCCTTGATCGATA

CN 120017 ATCCTAACATACTTTTCTTTTTTGTCATCTCTCCAGCCTGGTGAAGATCCTTGATCGATA

CN 119300 ATCCTAACATACTTTTCTTTTTTGTCATCTCTCCAGCCTGGTGAAGATCCTTGATCGATA

Jasper ATCCTAACATACTTTTCTTTTTTGTCATCTCTCCAGCCTGGTGAAGATCCTTGATCGATA

CN 120027 ATCCTAACATACTTTTCTTTTTTGTCATCTCTCCAGCCTGGTGAAGATCCTTGATCGATA

CN 113754 ATCCTAACATACTTTTCTTTTTTGTCATCTCTCCAGCCTGGTGAAGATCCTTGATCGATA

Joelle AAFC ATCCTAACATACTTTTCTTTTTTGTCATCTCTCCAGCCTGGTGAAGATCCTTGATCGATA

Joelle NCBI ATCCTAACATACTTTTCTTTTTTGTCATCTCTCCAGCCTGGTGAAGATCCTTGATCGATA

Joelle phyto ATCCTAACATACTTTTCTTTTTTGTCATCTCTCCAGCCTGGTGAAGATCCTTGATCGATA

Blaine Creek ATCCTAACATACTTTTCTTTTTTGTCATCTCTCCAGCCTGGTGAAGATCCTTGATCGATA

CAM 241 ATCCTAACATACTTTTCTTTTTTGTCATCTCTCCAGCCTGGTGAAGATCCTTGATCGATA

CN 119294 ATCCTAACATACTTTTCTTTTTTGTCATCTCTCCAGCCTGGTGAAGATCCTTGATCGATA

*********************** ****************** *****************

CN 120025 TGGGAAACAACATGCTGATGATCTCAAAGCCTTGGTAATACAAATATTTCGGATATATTC

CN 120013 TGGGAAACAACATGCTGATGATCTCAAAGCCTTGGTAATACAAATGTTTCGGATATTTTC

CS17CS1133 TGGGAAACAACATGCTGATGATCTCAAAGCCTTGGTAATACAAATGTTTCGGATATTTTC

CN 120030 TGGGAAACAACATGCTGATGATCTCAAAGCCTTGGTAATACAAATGTTTCGGATATTTTC

CO46 NCBI TGGGAAACAACATGCTGATGATCTCAAAGCCTTGGTAATACAAATGTTTCGGATATTTTC

DH55 ref genome TGGGAAACAACATGCTGATGATCTCAAAGCCTTGGTAATACAAATGTTTCGGATATTTTC

Hoga TGGGAAACAACATGCTGATGATCTCAAAGCCTTGGTAATACAAATGTTTCGGATATTTTC

CAM 236 TGGGAAACAACATGCTGATGATCTCAAAGCCTTGGTAATACAAATGTTTCGGATATTTTC

09-CS0040 TGGGAAACAACATGCTGATGATCTCAAAGCCTTGGTAATACAAATGTTTCGGATATTTTC

CN 119205 TGGGAAACAACATGCTGATGATCTCAAAGCCTTGGTAATACAAATGTTTCGGATATTTTC

Yellowstone TGGGAAACAACATGCTGATGATCTCAAAGCCTTGGTAATACAAATGTTTCGGATATTTTC

CN 120017 TGGGAAACAACATGCTGATGATCTCAAAGCCTTGGTAATACAAATGTTTCGGATATTTTC

CN 119300 TGGGAAACAACATGCTGATGATCTCAAAGCCTTGGTAATACAAATGTTTCGGATATTTTC

Jasper TGGGAAACAACATGCTGATGATCTCAAAGCCTTGGTAATACAAATGTTTCGGATATTTTC

CN 120027 TGGGAAACAACATGCTGATGATCTCAAAGCCTTGGTAATACAAATGTTTCGGATATTTTC

CN 113754 TGGGAAACAACATGCTGATGATCTCAAAGCCTTGGTAATACAAATGTTTCGGATATTTTC

Joelle AAFC TGGGAAACAACATGCTGATGATCTCAAAGCCTTGGTAATACAAATGTTTCGGATATTTTC

Joelle NCBI TGGGAAACAACATGCTGATGATCTCAAAGCCTTGGTAATACAAATGTTTCGGATATTTTC

Joelle phyto TGGGAAACAACATGCTGATGATCTCAAAGCCTTGGTAATACAAATGTTTCGGATATTTTC

Blaine Creek TGGGAAACAACATGCTGATGATCTCAAAGCCTTGGTAATACAAATGTTTCGGATATTTTC

CAM 241 TGGGAAACAACATGCTGATGATCTCAAAGCCTTGGTAATACAAATGTTTCGGATATTTTC

CN 119294 TGGGAAACAACATGCTGATGATCTCAAAGCCTTGGTAATACAAATGTTTCGGATATTTTC

********************************************* ********** ***

CN 120025 CCAGATGGATTTTCATAAGGCGTAGATTTACTAAAGACGTAGAGAGTTCAATAATCAATA

CN 120013 CCAGATGGATTTTCATGAGGCGTAGATTTACTAAAGACGTAGAGAGTTCAGTAATCAATA

CS17CS1133 CCAGATGGATTTTCATGAGGCGTAGATTTACTAAAGACGTAGAGAGTTCAGTAATCAATA

CN 120030 CCAGATGGATTTTCATGAGGCGTAGATTTACTAAAGACGTAGAGAGTTCAGTAATCAATA

CO46 NCBI CCAGATGGATTTTCATGAGGCGTAGATTTACTAAAGACGTAGAGAGTTCAGTAATCAATA

DH55 ref genome CCAGATGGATTTTCATGAGGCGTAGATTTACTAAAGACGTAGAGAGTTCAGTAATCAATA

Hoga CCAGATGGATTTTCATGAGGCGTAGATTTACTAAAGACGTAGAGAGTTCAGTAATCAATA

CAM 236 CCAGATGGATTTTCATGAGGCGTAGATTTACTAAAGACGTAGAGAGTTCAGTAATCAATA

09-CS0040 CCAGATGGATTTTCATGAGGCGTAGATTTACTAAAGACGTAGAGAGTTCAGTAATCAATA

CN 119205 CCAGATGGATTTTCATGAGGCGTAGATTTACTAAAGACGTAGAGAGTTCAGTAATCAATA

Yellowstone CCAGATGGATTTTCATGAGGCGTAGATTTACTAAAGACGTAGAGAGTTCAGTAATCAATA

CN 120017 CCAGATGGATTTTCATGAGGCGTAGATTTACTAAAGACGTAGAGAGTTCAGTAATCAATA

CN 119300 CCAGATGGATTTTCATGAGGCGTAGATTTACTAAAGACGTAGAGAGTTCAGTAATCAATA

Jasper CCAGATGGATTTTCATGAGGCGTAGATTTACTAAAGACGTAGAGAGTTCAGTAATCAATA

CN 120027 CCAGATGGATTTTCATGAGGCGTAGATTTACTAAAGACGTAGAGAGTTCAGTAATCAATA

CN 113754 CCAGATGGATTTTCATGAGGCGTAGATTTACTAAAGACGTAGAGAGTTCAGTAATCAATA

Joelle AAFC CCAGATGGATTTTCATGAGGCGTAGATTTACTAAAGACGTAGAGAGTTCAGTAATCAATA

Joelle NCBI CCAGATGGATTTTCATGAGGCGTAGATTTACTAAAGACGTAGAGAGTTCAGTAATCAATA

Joelle phyto CCAGATGGATTTTCATGAGGCGTAGATTTACTAAAGACGTAGAGAGTTCAGTAATCAATA

Blaine Creek CCAGATGGATTTTCATGAGGCGTAGATTTACTAAAGACGTAGAGAGTTCAGTAATCAATA

CAM 241 CCAGATGGATTTTCATGAGGCGTAGATTTACTAAAGACGTAGAGAGTTCAGTAATCAATA

CN 119294 CCAGATGGATTTTCATGAGGCGTAGATTTACTAAAGACGTAGAGAGTTCAGTAATCAATA

**************** ********************************* *********

CN 120025 CTGTCAGAGCCTATTCATAGCCTCCTGTCCAGCTTGCA-TTTTATAGGCCTGGGCTAAGA

CN 120013 CTGTCAGAGCCTATTCATAGCCTCCTGTCTAGCTTGCATTTTTATAGGCCTGGGCTAAGA

CS17CS1133 CTGTCAGAGCCTATTCATAGCCTCCTGTCTAGCTTGCATTTTTATAGGCCTGGGCTAAGA

CN 120030 CTGTCAGAGCCTATTCATAGCCTCCTGTCTAGCTTGCATTTTTATAGGCCTGGGCTAAGA

CO46 NCBI CTGTCAGAGCCTATTCATAGCCTCCTGTCTAGCTTGCATTTTTATAGGCCTGGGCTAAGA

DH55 ref genome CTGTCAGAGCCTATTCATAGCCTCCTGTCTAGCTTGCATTTTTATAGGCCTGGGCTAAGA

Hoga CTGTCAGAGCCTATTCATAGCCTCCTGTCTAGCTTGCATTTTTATAGGCCTGGGCTAAGA

CAM 236 CTGTCAGAGCCTATTCATAGCCTCCTGTCTAGCTTGCATTTTTATAGGCCTGGGCTAAGA

09-CS0040 CTGTCAGAGCCTATTCATAGCCTCCTGTCTAGCTTGCATTTTTATAGGCCTGGGCTAAGA

CN 119205 CTGTCAGAGCCTATTCATAGCCTCCTGTCTAGCTTGCATTTTTATAGGCCTGGGCTAAGA

Yellowstone CTGTCAGAGCCTATTCATAGCCTCCTGTCTAGCTTGCATTTTTATAGGCCTGGGCTAAGA

CN 120017 CTGTCAGAGCCTATTCATAGCCTCCTGTCTAGCTTGCATTTTTATAGGCCTGGGCTAAGA

CN 119300 CTGTCAGAGCCTATTCATAGCCTCCTGTCTAGCTTGCATTTTTATAGGCCTGGGCTAAGA

Jasper CTGTCAGAGCCTATTCATAGCCTCCTGTCTAGCTTGCATTTTTATAGGCCTGGGCTAAGA

CN 120027 CTGTCAGAGCCTATTCATAGCCTCCTGTCTAGCTTGCATTTTTATAGGCCTGGGCTAAGA

CN 113754 CTGTCAGAGCCTATTCATAGCCTCCTGTCTAGCTTGCATTTTTATAGGCCTGGGCTAAGA

Joelle AAFC CTGTCAGAGCCTATTCATAGCCTCCTGTCTAGCTTGCATTTTTATAGGCCTGGGCTAAGA

Joelle NCBI CTGTCAGAGCCTATTCATAGCCTCCTGTCTAGCTTGCATTTTTATAGGCCTGGGCTAAGA

Joelle phyto CTGTCAGAGCCTATTCATAGCCTCCTGTCTAGCTTGCATTTTTATAGGCCTGGGCTAAGA

Blaine Creek CTGTCAGAGCCTATTCATAGCCTCCTGTCTAGCTTGCATTTTTATAGGCCTGGGCTAAGA

CAM 241 CTGTCAGAGCCTATTCATAGCCTCCTGTCTAGCTTGCATTTTTATAGGCCTGGGCTAAGA

CN 119294 CTGTCAGAGCCTATTCATAGCCTCCTGTCTAGCTTGCATTTTTATAGGCCTGGGCTAAGA

***************************** ******** *********************

CN 120025 GGCATATAACGTTTTCACATATAGTCACTATTTGGAGTTGTGTAGTTGTAATTGTTTAAT

CN 120013 GGCATATAACGTTTTCACATATAGTCACTATTTGGAGTTGTGTAGTTGTAATTGTTTAAT

CS17CS1133 GGCATATAACGTTTTCACATATAGTCACTATTTGGAGTTGTGTAGTTGTAATTGTTTAAT

CN 120030 GGCATATAACGTTTTCACATATAGTCACTATTTGGAGTTGTGTAGTTGTAATTGTTTAAT

CO46 NCBI GGCATATAACGTTTTCACATATAGTCACTATTTGGAGTTGTGTAGTTGTAATTGTTTAAT

DH55 ref genome GGCATATAACGTTTTCACATATAGTCACTATTTGGAGTTGTGTAGTTGTAATTGTTTAAT

Hoga GGCATATAACGTTTTCACATATAGTCACTATTTGGAGTTGTGTAGTTGTAATTGTTTAAT

CAM 236 GGCATATAACGTTTTCACATATAGTCACTATTTGGAGTTGTGTAGTTGTAATTGTTTAAT

09-CS0040 GGCATATAACGTTTTCACATATAGTCACTATTTGGAGTTGTGTAGTTGTAATTGTTTAAT

CN 119205 GGCATATAACGTTTTCACATATAGTCACTATTTGGAGTTGTGTAGTTGTAATTGTTTAAT

Yellowstone GGCATATAACGTTTTCACATATAGTCACTATTTGGAGTTGTGTAGTTGTAATTGTTTAAT

CN 120017 GGCATATAACGTTTTCACATATAGTCACTATTTGGAGTTGTGTAGTTGTAATTGTTTAAT

CN 119300 GGCATATAACGTTTTCACATATAGTCACTATTTGGAGTTGTGTAGTTGTAATTGTTTAAT

Jasper GGCATATAACGTTTTCACATATAGTCACTATTTGGAGTTGTGTAGTTGTAATTGTTTAAT

CN 120027 GGCATATAACGTTTTCACATATAGTCACTATTTGGAGTTGTGTAGTTGTAATTGTTTAAT

CN 113754 GGCATATAACGTTTTCACATATAGTCACTATTTGGAGTTGTGTAGTTGTAATTGTTTAAT

Joelle AAFC GGCATATAACGTTTTCACATATAGTCACTATTTGGAGTTGTGTAGTTGTAATTGTTTAAT

Joelle NCBI GGCATATAACGTTTTCACATATAGTCACTATTTGGAGTTGTGTAGTTGTAATTGTTTAAT

Joelle phyto GGCATATAACGTTTTCACATATAGTCACTATTTGGAGTTGTGTAGTTGTAATTGTTTAAT

Blaine Creek GGCATATAACGTTTTCACATATAGTCACTATTTGGAGTTGTGTAGTTGTAATTGTTTAAT

CAM 241 GGCATATAACGTTTTCACATATAGTCACTATTTGGAGTTGTGTAGTTGTAATTGTTTAAT

CN 119294 GGCATATAACGTTTTCACATATAGTCACTATTTGGAGTTGTGTAGTTGTAATTGTTTAAT

************************************************************

CN 120025 AGATATATATGAGTAAACGAATGTTTAGCACCATACCATTGTGGAGATATATCTAGTTAA

CN 120013 AG----ATATGAGTAAACGAATGTTTAGCACCATAACATTGTGGAGATATATCTAGATAA

CS17CS1133 AG----ATATGAGTAAACGAATGTTTAGCACCATAACATTGTGGAGATATATCTAGATAA

CN 120030 AG----ATATGAGTAAACGAATGTTTAGCACCATAACATTGTGGAGATATATCTAGATAA

CO46 NCBI AG----ATATGAGTAAACGAATGTTTAGCACCATAACATTGTGGAGATATATCTAGATAA

DH55 ref genome AG----ATATGAGTAAACGAATGTTTAGCACCATAACATTGTGGAGATATATCTAGATAA

Hoga AG----ATATGAGTAAACGAATGTTTAGCACCATAACATTGTGGAGATATATCTAGATAA

CAM 236 AG----ATATGAGTAAACGAATGTTTAGCACCATAACATTGTGGAGATATATCTAGATAA

09-CS0040 AG----ATATGAGTAAACGAATGTTTAGCACCATAACATTGTGGAGATATATCTAGATAA

CN 119205 AG----ATATGAGTAAACGAATGTTTAGCACCATAACATTGTGGAGATATATCTAGATAA

Yellowstone AG----ATATGAGTAAACGAATGTTTAGCACCATAACATTGTGGAGATATATCTAGATAA

CN 120017 AG----ATATGAGTAAACGAATGTTTAGCACCATAACATTGTGGAGATATATCTAGATAA

CN 119300 AG----ATATGAGTAAACGAATGTTTAGCACCATAACATTGTGGAGATATATCTAGATAA

Jasper AG----ATATGAGTAAACGAATGTTTAGCACCATAACATTGTGGAGATATATCTAGATAA

CN 120027 AG----ATATGAGTAAACGAATGTTTAGCACCATAACATTGTGGAGATATATCTAGATAA

CN 113754 AG----ATATGAGTAAACGAATGTTTAGCACCATAACATTGTGGAGATATATCTAGATAA

Joelle AAFC AG----ATATGAGTAAACGAATGTTTAGCACCATAACATTGTGGAGATATATCTAGATAA

Joelle NCBI AG----ATATGAGTAAACGAATGTTTAGCACCATAACATTGTGGAGATATATCTAGATAA

Joelle phyto AG----ATATGAGTAAACGAATGTTTAGCACCATAACATTGTGGAGATATATCTAGATAA

Blaine Creek AG----ATATGAGTAAACGAATGTTTAGCACCATAACATTGTGGAGATATATCTAGATAA

CAM 241 AG----ATATGAGTAAACGAATGTTTAGCACCATAACATTGTGGAGATATATCTAGATAA

CN 119294 AG----ATATGAGTAAACGAATGTTTAGCACCATAACATTGTGGAGATATATCTAGATAA

** ***************************** ******************** ***

CN 120025 AGGAATTGCATCATTGATCCACCATGCGCTATTAGCTTGCCAAGTGATCCTAAATATATA

CN 120013 AGGAATTGCATCATTGATCCACCATGCGCTATTAGCTTGCTAAGTGATTCTAAATATATA

CS17CS1133 AGGAATTGCATCATTGATCCACCATGCGCTATTAGCTTGCTAAGTGATTCTAAATATATA

CN 120030 AGGAATTGCATCATTGATCCACCATGCGCTATTAGCTTGCTAAGTGATTCTAAATATATA

CO46 NCBI AGGAATTGCATCATTGATCCACCATGCGCTATTAGCTTGCTAAGTGATTCTAAATATATA

DH55 ref genome AGGAATTGCATCATTGATCCACCATGCGCTATTAGCTTGCTAAGTGATTCTAAATATATA

Hoga AGGAATTGCATCATTGATCCACCATGCGCTATTAGCTTGCTAAGTGATTCTAAATATATA

CAM 236 AGGAATTGCATCATTGATCCACCATGCGCTATTAGCTTGCTAAGTGATTCTAAATATATA

09-CS0040 AGGAATTGCATCATTGATCCACCATGCGCTATTAGCTTGCTAAGTGATTCTAAATATATA

CN 119205 AGGAATTGCATCATTGATCCACCATGCGCTATTAGCTTGCTAAGTGATTCTAAATATATA

Yellowstone AGGAATTGCATCATTGATCCACCATGCGCTATTAGCTTGCTAAGTGATTCTAAATATATA

CN 120017 AGGAATTGCATCATTGATCCACCATGCGCTATTAGCTTGCTAAGTGATTCTAAATATATA

CN 119300 AGGAATTGCATCATTGATCCACCATGCGCTATTAGCTTGCTAAGTGATTCTAAATATATA

Jasper AGGAATTGCATCATTGATCCACCATGCGCTATTAGCTTGCTAAGTGATTCTAAATATATA

CN 120027 AGGAATTGCATCATTGATCCACCATGCGCTATTAGCTTGCTAAGTGATTCTAAATATATA

CN 113754 AGGAATTGCATCATTGATCCACCATGCGCTATTAGCTTGCTAAGTGATTCTAAATATATA

Joelle AAFC AGGAATTGCATCATTGATCCACCATGCGCTATTAGCTTGCTAAGTGATTCTAAATATATA

Joelle NCBI AGGAATTGCATCATTGATCCACCATGCGCTATTAGCTTGCTAAGTGATTCTAAATATATA

Joelle phyto AGGAATTGCATCATTGATCCACCATGCGCTATTAGCTTGCTAAGTGATTCTAAATATATA

Blaine Creek AGGAATTGCATCATTGATCCACCATGCGCTATTAGCTTGCTAAGTGATTCTAAATATATA

CAM 241 AGGAATTGCATCATTGATCCACCATGCGCTATTAGCTTGCTAAGTGATTCTAAATATATA

CN 119294 AGGAATTGCATCATTGATCCACCATGCGCTATTAGCTTGCTAAGTGATTCTAAATATATA

**************************************** ******* ***********

CN 120025 ATGAGGTGATGTACCAACATGCATACTGCAATCCTCGTAGCTTTTTCTTTTACCGCATCT

CN 120013 ATGAGATGGTGTACCAACATGCATACTGAAATCCTGCTAGCCTTGTCTTTTACCGCGTCT

CS17CS1133 ATGAGATGGTGTACCAACATGCATACTGAAATCCTGCTAGCCTTGTCTTTTACCGCGTCT

CN 120030 ATGAGATGGTGTACCAACATGCATACTGAAATCCTGCTAGCCTTGTCTTTTACCGCGTCT

CO46 NCBI ATGAGATGGTGTACCAACATGCATACTGAAATCCTGCTAGCCTTGTCTTTTACCGCGTCT

DH55 ref genome ATGAGATGGTGTACCAACATGCATACTGAAATCCTGCTAGCCTTGTCTTTTACCGCGTCT

Hoga ATGAGATGGTGTACCAACATGCATACTGAAATCCTGCTAGCCTTGTCTTTTACCGCGTCT

CAM 236 ATGAGATGGTGTACCAACATGCATACTGAAATCCTGCTAGCCTTGTCTTTTACCGCGTCT

09-CS0040 ATGAGATGGTGTACCAACATGCATACTGAAATCCTGCTAGCCTTGTCTTTTACCGCGTCT

CN 119205 ATGAGATGGTGTACCAACATGCATACTGAAATCCTGCTAGCCTTGTCTTTTACCGCGTCT

Yellowstone ATGAGATGGTGTACCAACATGCATACTGAAATCCTGCTAGCCTTGTCTTTTACCGCGTCT

CN 120017 ATGAGATGGTGTACCAACATGCATACTGAAATCCTGCTAGCCTTGTCTTTTACCGCGTCT

CN 119300 ATGAGATGGTGTACCAACATGCATACTGAAATCCTGCTAGCCTTGTCTTTTACCGCGTCT

Jasper ATGAGATGGTGTACCAACATGCATACTGAAATCCTGCTAGCCTTGTCTTTTACCGCGTCT

CN 120027 ATGAGATGGTGTACCAACATGCATACTGAAATCCTGCTAGCCTTGTCTTTTACCGCGTCT

CN 113754 ATGAGATGGTGTACCAACATGCATACTGAAATCCTGCTAGCCTTGTCTTTTACCGCGTCT

Joelle AAFC ATGAGATGGTGTACCAACATGCATACTGAAATCCTGCTAGCCTTGTCTTTTACCGCGTCT

Joelle NCBI ATGAGATGGTGTACCAACATGCATACTGAAATCCTGCTAGCCTTGTCTTTTACCGCGTCT

Joelle phyto ATGAGATGGTGTACCAACATGCATACTGAAATCCTGCTAGCCTTGTCTTTTACCGCGTCT

Blaine Creek ATGAGATGGTGTACCAACATGCATACTGAAATCCTGCTAGCCTTGTCTTTTACCGCGTCT

CAM 241 ATGAGATGGTGTACCAACATGCATACTGAAATCCTGCTAGCCTTGTCTTTTACCGCGTCT

CN 119294 ATGAGATGGTGTACCAACATGCATACTGAAATCCTGCTAGCCTTGTCTTTTACCGCGTCT

***** ** ******************* ****** **** ** *********** ***

CN 120025 --TATATGTCCCTTTTCTTTGGCCAGGATCTTCAGTCAAAAGCTCTGAACTATGGTTCGC

CN 120013 TATATATGTCCCTTTTCTTTGGCCAGGATCTTCAGTCAAAAGAACTGAACTATGGTTCAC

CS17CS1133 TATATATGTCCCTTTTCTTTGGCCAGGATCTTCAGTCAAAAGAACTGAACTATGGTTCAC

CN 120030 TATATATGTCCCTTTTCTTTGGCCAGGATCTTCAGTCAAAAGAACTGAACTATGGTTCAC

CO46 NCBI TATATATGTCCCTTTTCTTTGGCCAGGATCTTCAGTCAAAAGAACTGAACTATGGTTCAC

DH55 ref genome TATATATGTCCCTTTTCTTTGGCCAGGATCTTCAGTCAAAAGAACTGAACTATGGTTCAC

Hoga TATATATGTCCCTTTTCTTTGGCCAGGATCTTCAGTCAAAAGAACTGAACTATGGTTCAC

CAM 236 TATATATGTCCCTTTTCTTTGGCCAGGATCTTCAGTCAAAAGAACTGAACTATGGTTCAC

09-CS0040 TATATATGTCCCTTTTCTTTGGCCAGGATCTTCAGTCAAAAGAACTGAACTATGGTTCAC

CN 119205 TATATATGTCCCTTTTCTTTGGCCAGGATCTTCAGTCAAAAGAACTGAACTATGGTTCAC

Yellowstone TATATATGTCCCTTTTCTTTGGCCAGGATCTTCAGTCAAAAGAACTGAACTATGGTTCAC

CN 120017 TATATATGTCCCTTTTCTTTGGCCAGGATCTTCAGTCAAAAGAACTGAACTATGGTTCAC

CN 119300 TATATATGTCCCTTTTCTTTGGCCAGGATCTTCAGTCAAAAGAACTGAACTATGGTTCAC

Jasper TATATATGTCCCTTTTCTTTGGCCAGGATCTTCAGTCAAAAGAACTGAACTATGGTTCAC

CN 120027 TATATATGTCCCTTTTCTTTGGCCAGGATCTTCAGTCAAAAGAACTGAACTATGGTTCAC

CN 113754 TATATATGTCCCTTTTCTTTGGCCAGGATCTTCAGTCAAAAGAACTGAACTATGGTTCAC

Joelle AAFC TATATATGTCCCTTTTCTTTGGCCAGGATCTTCAGTCAAAAGAACTGAACTATGGTTCAC

Joelle NCBI TATATATGTCCCTTTTCTTTGGCCAGGATCTTCAGTCAAAAGAACTGAACTATGGTTCAC

Joelle phyto TATATATGTCCCTTTTCTTTGGCCAGGATCTTCAGTCAAAAGAACTGAACTATGGTTCAC

Blaine Creek TATATATGTCCCTTTTCTTTGGCCAGGATCTTCAGTCAAAAGAACTGAACTATGGTTCAC

CAM 241 TATATATGTCCCTTTTCTTTGGCCAGGATCTTCAGTCAAAAGAACTGAACTATGGTTCAC

CN 119294 TATATATGTCCCTTTTCTTTGGCCAGGATCTTCAGTCAAAAGAACTGAACTATGGTTCAC

**************************************** ************** *

CN 120025 ACCATGAGCTACTAGAACTTGTGGAAAGGTTAGTACTAACTAAGACTATATTTGCTCTCC

CN 120013 ACCATGAGCTACTAGAACTTGTGGAAAGGTTAGTACTAGCTAAGACTATATTTGCTCTCC

CS17CS1133 ACCATGAGCTACTAGAACTTGTGGAAAGGTTAGTACTAGCTAAGACTATATTTGCTCTCC

CN 120030 ACCATGAGCTACTAGAACTTGTGGAAAGGTTAGTACTAGCTAAGACTATATTTGCTCTCC

CO46 NCBI ACCATGAGCTACTAGAACTTGTGGAAAGGTTAGTACTAGCTAAGACTATATTTGCTCTCC

DH55 ref genome ACCATGAGCTACTAGAACTTGTGGAAAGGTTAGTACTAGCTAAGACTATATTTGCTCTCC

Hoga ACCATGAGCTACTAGAACTTGTGGAAAGGTTAGTACTAGCTAAGACTATATTTGCTCTCC

CAM 236 ACCATGAGCTACTAGAACTTGTGGAAAGGTTAGTACTAGCTAAGACTATATTTGCTCTCC

09-CS0040 ACCATGAGCTACTAGAACTTGTGGAAAGGTTAGTACTAGCTAAGACTATATTTGCTCTCC

CN 119205 ACCATGAGCTACTAGAACTTGTGGAAAGGTTAGTACTAGCTAAGACTATATTTGCTCTCC

Yellowstone ACCATGAGCTACTAGAACTTGTGGAAAGGTTAGTACTAGCTAAGACTATATTTGCTCTCC

CN 120017 ACCATGAGCTACTAGAACTTGTGGAAAGGTTAGTACTAGCTAAGACTATATTTGCTCTCC

CN 119300 ACCATGAGCTACTAGAACTTGTGGAAAGGTTAGTACTAGCTAAGACTATATTTGCTCTCC

Jasper ACCATGAGCTACTAGAACTTGTGGAAAGGTTAGTACTAGCTAAGACTATATTTGCTCTCC

CN 120027 ACCATGAGCTACTAGAACTTGTGGAAAGGTTAGTACTAGCTAAGACTATATTTGCTCTCC

CN 113754 ACCATGAGCTACTAGAACTTGTGGAAAGGTTAGTACTAGCTAAGACTATATTTGCTCTCC

Joelle AAFC ACCATGAGCTACTAGAACTTGTGGAAAGGTTAGTACTAGCTAAGACTATATTTGCTCTCC

Joelle NCBI ACCATGAGCTACTAGAACTTGTGGAAAGGTTAGTACTAGCTAAGACTATATTTGCTCTCC

Joelle phyto ACCATGAGCTACTAGAACTTGTGGAAAGGTTAGTACTAGCTAAGACTATATTTGCTCTCC

Blaine Creek ACCATGAGCTACTAGAACTTGTGGAAAGGTTAGTACTAGCTAAGACTATATTTGCTCTCC

CAM 241 ACCATGAGCTACTAGAACTTGTGGAAAGGTTAGTACTAGCTAAGACTATATTTGCTCTCC

CN 119294 ACCATGAGCTACTAGAACTTGTGGAAAGGTTAGTACTAGCTAAGACTATATTTGCTCTCC

************************************** *********************

CN 120025 TCCTTTGAGTATAAAGGAATTAGGGTTTTCTTGTCAAACTATGAATATATGCAGCAATCT

CN 120013 TCCTTTGATTATAAAGGAATTAGGGTTTCCTTGTAAAACTATGAATATATGCAGCAATCT

CS17CS1133 TCCTTTGATTATAAAGGAATTAGGGTTTCCTTGTAAAACTATGAATATATGCAGCAATCT

CN 120030 TCCTTTGATTATAAAGGAATTAGGGTTTCCTTGTAAAACTATGAATATATGCAGCAATCT

CO46 NCBI TCCTTTGATTATAAAGGAATTAGGGTTTCCTTGTAAAACTATGAATATATGCAGCAATCT

DH55 ref genome TCCTTTGATTATAAAGGAATTAGGGTTTCCTTGTAAAACTATGAATATATGCAGCAATCT

Hoga TCCTTTGATTATAAAGGAATTAGGGTTTCCTTGTAAAACTATGAATATATGCAGCAATCT

CAM 236 TCCTTTGATTATAAAGGAATTAGGGTTTCCTTGTAAAACTATGAATATATGCAGCAATCT

09-CS0040 TCCTTTGATTATAAAGGAATTAGGGTTTCCTTGTAAAACTATGAATATATGCAGCAATCT

CN 119205 TCCTTTGATTATAAAGGAATTAGGGTTTCCTTGTAAAACTATGAATATATGCAGCAATCT

Yellowstone TCCTTTGATTATAAAGGAATTAGGGTTTCCTTGTAAAACTATGAATATATGCAGCAATCT

CN 120017 TCCTTTGATTATAAAGGAATTAGGGTTTCCTTGTAAAACTATGAATATATGCAGCAATCT

CN 119300 TCCTTTGATTATAAAGGAATTAGGGTTTCCTTGTAAAACTATGAATATATGCAGCAATCT

Jasper TCCTTTGATTATAAAGGAATTAGGGTTTCCTTGTAAAACTATGAATATATGCAGCAATCT

CN 120027 TCCTTTGATTATAAAGGAATTAGGGTTTCCTTGTAAAACTATGAATATATGCAGCAATCT

CN 113754 TCCTTTGATTATAAAGGAATTAGGGTTTCCTTGTAAAACTATGAATATATGCAGCAATCT

Joelle AAFC TCCTTTGATTATAAAGGAATTAGGGTTTCCTTGTAAAACTATGAATATATGCAGCAATCT

Joelle NCBI TCCTTTGATTATAAAGGAATTAGGGTTTCCTTGTAAAACTATGAATATATGCAGCAATCT

Joelle phyto TCCTTTGATTATAAAGGAATTAGGGTTTCCTTGTAAAACTATGAATATATGCAGCAATCT

Blaine Creek TCCTTTGATTATAAAGGAATTAGGGTTTCCTTGTAAAACTATGAATATATGCAGCAATCT

CAM 241 TCCTTTGATTATAAAGGAATTAGGGTTTCCTTGTAAAACTATGAATATATGCAGCAATCT

CN 119294 TCCTTTGATTATAAAGGAATTAGGGTTTCCTTGTAAAACTATGAATATATGCAGCAATCT

******** ******************* ***** *************************

CN 120025 TGTGGAATCAAATGTCAATAATGTAAGTGTGGATGCCCTCGTTCAACTGGAGGAACACCT

CN 120013 TGTGGAATCAAATGTCAACAATGTGAGTGTCGATTCCCTCGTTCAACTGGAGGAACACCT

CS17CS1133 TGTGGAATCAAATGTCAACAATGTGAGTGTCGATTCCCTCGTTCAACTGGAGGAACACCT

CN 120030 TGTGGAATCAAATGTCAACAATGTGAGTGTCGATTCCCTCGTTCAACTGGAGGAACACCT

CO46 NCBI TGTGGAATCAAATGTCAACAATGTGAGTGTCGATTCCCTCGTTCAACTGGAGGAACACCT

DH55 ref genome TGTGGAATCAAATGTCAACAATGTGAGTGTCGATTCCCTCGTTCAACTGGAGGAACACCT

Hoga TGTGGAATCAAATGTCAACAATGTGAGTGTCGATTCCCTCGTTCAACTGGAGGAACACCT

CAM 236 TGTGGAATCAAATGTCAACAATGTGAGTGTCGATTCCCTCGTTCAACTGGAGGAACACCT

09-CS0040 TGTGGAATCAAATGTCAACAATGTGAGTGTCGATTCCCTCGTTCAACTGGAGGAACACCT

CN 119205 TGTGGAATCAAATGTCAACAATGTGAGTGTCGATTCCCTCGTTCAACTGGAGGAACACCT

Yellowstone TGTGGAATCAAATGTCAACAATGTGAGTGTCGATTCCCTCGTTCAACTGGAGGAACACCT

CN 120017 TGTGGAATCAAATGTCAACAATGTGAGTGTCGATTCCCTCGTTCAACTGGAGGAACACCT

CN 119300 TGTGGAATCAAATGTCAACAATGTGAGTGTCGATTCCCTCGTTCAACTGGAGGAACACCT

Jasper TGTGGAATCAAATGTCAACAATGTGAGTGTCGATTCCCTCGTTCAACTGGAGGAACACCT

CN 120027 TGTGGAATCAAATGTCAACAATGTGAGTGTCGATTCCCTCGTTCAACTGGAGGAACACCT

CN 113754 TGTGGAATCAAATGTCAACAATGTGAGTGTCGATTCCCTCGTTCAACTGGAGGAACACCT

Joelle AAFC TGTGGAATCAAATGTCAACAATGTGAGTGTCGATTCCCTCGTTCAACTGGAGGAACACCT

Joelle NCBI TGTGGAATCAAATGTCAACAATGTGAGTGTCGATTCCCTCGTTCAACTGGAGGAACACCT

Joelle phyto TGTGGAATCAAATGTCAACAATGTGAGTGTCGATTCCCTCGTTCAACTGGAGGAACACCT

Blaine Creek TGTGGAATCAAATGTCAACAATGTGAGTGTCGATTCCCTCGTTCAACTGGAGGAACACCT

CAM 241 TGTGGAATCAAATGTCAACAATGTGAGTGTCGATTCCCTCGTTCAACTGGAGGAACACCT

CN 119294 TGTGGAATCAAATGTCAACAATGTGAGTGTCGATTCCCTCGTTCAACTGGAGGAACACCT

****************** ***** ***** *** *************************

CN 120025 TGAGACTGCCCTCTCCGTAACCAGAGCCAAGAAGGTAAGTGGATTCTCTAATGT----CT

CN 120013 TGAGACCGCCCTCTCCATAACTAGAGCGAAGAAGGTAAGTGGATTCTATAATGTCTAGCT

CS17CS1133 TGAGACCGCCCTCTCCATAACTAGAGCGAAGAAGGTAAGTGGATTCTATAATGTCTAGCT

CN 120030 TGAGACCGCCCTCTCCATAACTAGAGCGAAGAAGGTAAGTGGATTCTATAATGTCTAGCT

CO46 NCBI TGAGACCGCCCTCTCCATAACTAGAGCGAAGAAGGTAAGTGGATTCTATAATGTCTAGCT

DH55 ref genome TGAGACCGCCCTCTCCATAACTAGAGCGAAGAAGGTAAGTGGATTCTATAATGTCTAGCT

Hoga TGAGACCGCCCTCTCCATAACTAGAGCGAAGAAGGTAAGTGGATTCTATAATGTCTAGCT

CAM 236 TGAGACCGCCCTCTCCATAACTAGAGCGAAGAAGGTAAGTGGATTCTATAATGTCTAGCT

09-CS0040 TGAGACCGCCCTCTCCATAACTAGAGCGAAGAAGGTAAGTGGATTCTATAATGTCTAGCT

CN 119205 TGAGACCGCCCTCTCCATAACTAGAGCGAAGAAGGTAAGTGGATTCTATAATGTCTAGCT

Yellowstone TGAGACCGCCCTCTCCATAACTAGAGCGAAGAAGGTAAGTGGATTCTATAATGTCTAGCT

CN 120017 TGAGACCGCCCTCTCCATAACTAGAGCGAAGAAGGTAAGTGGATTCTATAATGTCTAGCT

CN 119300 TGAGACCGCCCTCTCCATAACTAGAGCGAAGAAGGTAAGTGGATTCTATAATGTCTAGCT

Jasper TGAGACCGCCCTCTCCATAACTAGAGCGAAGAAGGTAAGTGGATTCTATAATGTCTAGCT

CN 120027 TGAGACCGCCCTCTCCATAACTAGAGCGAAGAAGGTAAGTGGATTCTATAATGTCTAGCT

CN 113754 TGAGACCGCCCTCTCCATAACTAGAGCGAAGAAGGTAAGTGGATTCTATAATGTCTAGCT

Joelle AAFC TGAGACCGCCCTCTCCATAACTAGAGCGAAGAAGGTAAGTGGATTCTATAATGTCTAGCT

Joelle NCBI TGAGACCGCCCTCTCCATAACTAGAGCGAAGAAGGTAAGTGGATTCTATAATGTCTAGCT

Joelle phyto TGAGACCGCCCTCTCCATAACTAGAGCGAAGAAGGTAAGTGGATTCTATAATGTCTAGCT

Blaine Creek TGAGACCGCCCTCTCCATAACTAGAGCGAAGAAGGTAAGTGGATTCTATAATGTCTAGCT

CAM 241 TGAGACCGCCCTCTCCATAACTAGAGCGAAGAAGGTAAGTGGATTCTATAATGTCTAGCT

CN 119294 TGAGACCGCCCTCTCCATAACTAGAGCGAAGAAGGTAAGTGGATTCTATAATGTCTAGCT

****** ********* **** ***** ******************* ****** **

CN 120025 ACTCTTTTCAGAA-TTTGTTTGCCGAGAATAACCTTATTGCTTTTGTTTGTTACAGACAG

CN 120013 ACTCTTTCCTGAATTTTATTTTCCGAGAATAACCTTATTGCTTTTATTTGTTACAGACAG

CS17CS1133 ACTCTTTCCTGAATTTTATTTTCCGAGAATAACCTTATTGCTTTTATTTGTTACAGACAG

CN 120030 ACTCTTTCCTGAATTTTATTTTCCGAGAATAACCTTATTGCTTTTATTTGTTACAGACAG

CO46 NCBI ACTCTTTCCTGAATTTTATTTTCCGAGAATAACCTTATTGCTTTTATTTGTTACAGACAG

DH55 ref genome ACTCTTTCCTGAATTTTATTTTCCGAGAATAACCTTATTGCTTTTATTTGTTACAGACAG

Hoga ACTCTTTCCTGAATTTTATTTTCCGAGAATAACCTTATTGCTTTTATTTGTTACAGACAG

CAM 236 ACTCTTTCCTGAATTTTATTTTCCGAGAATAACCTTATTGCTTTTATTTGTTACAGACAG

09-CS0040 ACTCTTTCCTGAATTTTATTTTCCGAGAATAACCTTATTGCTTTTATTTGTTACAGACAG

CN 119205 ACTCTTTCCTGAATTTTATTTTCCGAGAATAACCTTATTGCTTTTATTTGTTACAGACAG

Yellowstone ACTCTTTCCTGAATTTTATTTTCCGAGAATAACCTTATTGCTTTTATTTGTTACAGACAG

CN 120017 ACTCTTTCCTGAATTTTATTTTCCGAGAATAACCTTATTGCTTTTATTTGTTACAGACAG

CN 119300 ACTCTTTCCTGAATTTTATTTTCCGAGAATAACCTTATTGCTTTTATTTGTTACAGACAG

Jasper ACTCTTTCCTGAATTTTATTTTCCGAGAATAACCTTATTGCTTTTATTTGTTACAGACAG

CN 120027 ACTCTTTCCTGAATTTTATTTTCCGAGAATAACCTTATTGCTTTTATTTGTTACAGACAG

CN 113754 ACTCTTTCCTGAATTTTATTTTCCGAGAATAACCTTATTGCTTTTATTTGTTACAGACAG

Joelle AAFC ACTCTTTCCTGAATTTTATTTTCCGAGAATAACCTTATTGCTTTTATTTGTTACAGACAG

Joelle NCBI ACTCTTTCCTGAATTTTATTTTCCGAGAATAACCTTATTGCTTTTATTTGTTACAGACAG

Joelle phyto ACTCTTTCCTGAATTTTATTTTCCGAGAATAACCTTATTGCTTTTATTTGTTACAGACAG

Blaine Creek ACTCTTTCCTGAATTTTATTTTCCGAGAATAACCTTATTGCTTTTATTTGTTACAGACAG

CAM 241 ACTCTTTCCTGAATTTTATTTTCCGAGAATAACCTTATTGCTTTTATTTGTTACAGACAG

CN 119294 ACTCTTTCCTGAATTTTATTTTCCGAGAATAACCTTATTGCTTTTATTTGTTACAGACAG

******* * *** *** *** *********************** **************

CN 120025 AACTAATG**T**TGAAGCTTGTTGAGAACCTCAAAGAAAAGGTTAGATATCTGATTCCAAGTT

CN 120013 AACTAATG**-**TGAAGCTTGTTGAGAACCTCAAAGAAAAGGTTAGATATTTGATTCCAAGTT

CS17CS1133 AACTAATG**-**TGAAGCTTGTTGAGAACCTCAAAGAAAAGGTTAGATATTTGATTCCAAGTT

CN 120030 AACTAATG**T**TGAAGCTTGTTGAGAACCTCAAAGAAAAGGTTAGATATTTGATTCCAAGTT

CO46 NCBI AACTAATG**T**TGAAGCTTGTTGAGAACCTCAAAGAAAAGGTTAGATATTTGATTCCAAGTT

DH55 ref genome AACTAATG**-**TGAAGCTTGTTGAGAACCTCAAAGAAAAGGTTAGATATTTGATTCCAAGTT

Hoga AACTAATG**-**TGAAGCTTGTTGAGAACCTCAAAGAAAAGGTTAGATATTTGATTCCAAGTT

CAM 236 AACTAATG**-**TGAAGCTTGTTGAGAACCTCAAAGAAAAGGTTAGATATTTGATTCCAAGTT

09-CS0040 AACTAATG**-**TGAAGCTTGTTGAGAACCTCAAAGAAAAGGTTAGATATTTGATTCCAAGTT

CN 119205 AACTAATG**T**TGAAGCTTGTTGAGAACCTCAAAGAAAAGGTTAGATATTTGATTCCAAGTT

Yellowstone AACTAATG**-**TGAAGCTTGTTGAGAACCTCAAAGAAAAGGTTAGATATTTGATTCCAAGTT

CN 120017 AACTAATG**-**TGAAGCTTGTTGAGAACCTCAAAGAAAAGGTTAGATATTTGATTCCAAGTT

CN 119300 AACTAATG**-**TGAAGCTTGTTGAGAACCTCAAAGAAAAGGTTAGATATTTGATTCCAAGTT

Jasper AACTAATG**-**TGAAGCTTGTTGAGAACCTCAAAGAAAAGGTTAGATATTTGATTCCAAGTT

CN 120027 AACTAATG**-**TGAAGCTTGTTGAGAACCTCAAAGAAAAGGTTAGATATTTGATTCCAAGTT

CN 113754 AACTAATG**-**TGAAGCTTGTTGAGAACCTCAAAGAAAAGGTTAGATATTTGATTCCAAGTT

Joelle AAFC AACTAATG**-**TGAAGCTTGTTGAGAACCTCAAAGAAAAGGTTAGATATTTGATTCCAAGTT

Joelle NCBI AACTAATG**T**TGAAGCTTGTTGAGAACCTCAAAGAAAAGGTTAGATATTTGATTCCAAGTT

Joelle phyto AACTAATG**T**TGAAGCTTGTTGAGAACCTCAAAGAAAAGGTTAGATATTTGATTCCAAGTT

Blaine Creek AACTAATG**T**TGAAGCTTGTTGAGAACCTCAAAGAAAAGGTTAGATATTTGATTCCAAGTT

CAM 241 AACTAATG**T**TGAAGCTTGTTGAGAACCTCAAAGAAAAGGTTAGATATTTGATTCCAAGTT

CN 119294 AACTAATG**T**TGAAGCTTGTTGAGAACCTCAAAGAAAAGGTTAGATATTTGATTCCAAGTT

******** ************************************** ************

CN 120025 TAGAACATATATCAGATATATACTCTAGGG---TGTTTTCATTGTTTCTGTAAGTATGTT

CN 120013 TAGAACATATATCAGATTTATACTCTAGGGTACTGTTTTCATTGTTTCTGTAAGTCTGTT

CS17CS1133 TAGAACATATATCAGATTTATACTCTAGGGTACTGTTTTCATTGTTTCTGTAAGTCTGTT

CN 120030 TAGAACATATATCAGATTTATACTCTAGGGTACTGTTTTCATTGTTTCTGTAAGTCTGTT

CO46 NCBI TAGAACATATATCAGATTTATACTCTAGGGTACTGTTTTCATTGTTTCTGTAAGTCTGTT

DH55 ref genome TAGAACATATATCAGATTTATACTCTAGGGTACTGTTTTCATTGTTTCTGTAAGTCTGTT

Hoga TAGAACATATATCAGATTTATACTCTAGGGTACTGTTTTCATTGTTTCTGTAAGTCTGTT

CAM 236 TAGAACATATATCAGATTTATACTCTAGGGTACTGTTTTCATTGTTTCTGTAAGTCTGTT

09-CS0040 TAGAACATATATCAGATTTATACTCTAGGGTACTGTTTTCATTGTTTCTGTAAGTCTGTT

CN 119205 TAGAACATATATCAGATTTATACTCTAGGGTACTGTTTTCATTGTTTCTGTAAGTCTGTT

Yellowstone TAGAACATATATCAGATTTATACTCTAGGGTACTGTTTTCATTGTTTCTGTAAGTCTGTT

CN 120017 TAGAACATATATCAGATTTATACTCTAGGGTACTGTTTTCATTGTTTCTGTAAGTCTGTT

CN 119300 TAGAACATATATCAGATTTATACTCTAGGGTACTGTTTTCATTGTTTCTGTAAGTCTGTT

Jasper TAGAACATATATCAGATTTATACTCTAGGGTACTGTTTTCATTGTTTCTGTAAGTCTGTT

CN 120027 TAGAACATATATCAGATTTATACTCTAGGGTACTGTTTTCATTGTTTCTGTAAGTCTGTT

CN 113754 TAGAACATATATCAGATTTATACTCTAGGGTACTGTTTTCATTGTTTCTGTAAGTCTGTT

Joelle AAFC TAGAACATATATCAGATTTATACTCTAGGGTACTGTTTTCATTGTTTCTGTAAGTCTGTT

Joelle NCBI TAGAACATATATCAGATTTATACTCTAGGGTACTGTTTTCATTGTTTCTGTAAGTCTGTT

Joelle phyto TAGAACATATATCAGATTTATACTCTAGGGTACTGTTTTCATTGTTTCTGTAAGTCTGTT

Blaine Creek TAGAACATATATCAGATTTATACTCTAGGGTACTGTTTTCATTGTTTCTGTAAGTCTGTT

CAM 241 TAGAACATATATCAGATTTATACTCTAGGGTACTGTTTTCATTGTTTCTGTAAGTCTGTT

CN 119294 TAGAACATATATCAGATTTATACTCTAGGGTACTGTTTTCATTGTTTCTGTAAGTCTGTT

***************** ************ ********************** ****

CN 120025 TAATGAGTTTTGCACACGTCTTTGCAACTTCTCCCCAATGCATATGTTGTGGATGTCAAA

CN 120013 TAATGAGCTTTTCACACGTCTTTGCAACTTCTTCCCAATACATATGTTGTGGATTCCAAA

CS17CS1133 TAATGAGCTTTTCACACGTCTTTGCAACTTCTTCCCAATACATATGTTGTGGATTCCAAA

CN 120030 TAATGAGCTTTTCACACGTCTTTGCAACTTCTTCCCAATACATATGTTGTGGATTCCAAA

CO46 NCBI TAATGAGCTTTTCACACGTCTTTGCAACTTCTTCCCAATACATATGTTGTGGATTCCAAA

DH55 ref genome TAATGAGCTTTTCACACGTCTTTGCAACTTCTTCCCAATACATATGTTGTGGATTCCAAA

Hoga TAATGAGCTTTTCACACGTCTTTGCAACTTCTTCCCAATACATATGTTGTGGATTCCAAA

CAM 236 TAATGAGCTTTTCACACGTCTTTGCAACTTCTTCCCAATACATATGTTGTGGATTCCAAA

09-CS0040 TAATGAGCTTTTCACACGTCTTTGCAACTTCTTCCCAATACATATGTTGTGGATTCCAAA

CN 119205 TAATGAGCTTTTCACACGTCTTTGCAACTTCTTCCCAATACATATGTTGTGGATTCCAAA

Yellowstone TAATGAGCTTTTCACACGTCTTTGCAACTTCTTCCCAATACATATGTTGTGGATTCCAAA

CN 120017 TAATGAGCTTTTCACACGTCTTTGCAACTTCTTCCCAATACATATGTTGTGGATTCCAAA

CN 119300 TAATGAGCTTTTCACACGTCTTTGCAACTTCTTCCCAATACATATGTTGTGGATTCCAAA

Jasper TAATGAGCTTTTCACACGTCTTTGCAACTTCTTCCCAATACATATGTTGTGGATTCCAAA

CN 120027 TAATGAGCTTTTCACACGTCTTTGCAACTTCTTCCCAATACATATGTTGTGGATTCCAAA

CN 113754 TAATGAGCTTTTCACACGTCTTTGCAACTTCTTCCCAATACATATGTTGTGGATTCCAAA

Joelle AAFC TAATGAGCTTTTCACACGTCTTTGCAACTTCTTCCCAATACATATGTTGTGGATTCCAAA

Joelle NCBI TAATGAGCTTTTCACACGTCTTTGCAACTTCTTCCCAATACATATGTTGTGGATTCCAAA

Joelle phyto TAATGAGCTTTTCACACGTCTTTGCAACTTCTTCCCAATACATATGTTGTGGATTCCAAA

Blaine Creek TAATGAGCTTTTCACACGTCTTTGCAACTTCTTCCCAATACATATGTTGTGGATTCCAAA

CAM 241 TAATGAGCTTTTCACACGTCTTTGCAACTTCTTCCCAATACATATGTTGTGGATTCCAAA

CN 119294 TAATGAGCTTTTCACACGTCTTTGCAACTTCTTCCCAATACATATGTTGTGGATTCCAAA

******* *** ******************** ****** ************** ****

CN 120025 ATCTGAATTTGTTAAGTTGTGATTTGTGATTGCTGAACATGATGATCTTTAAAACAGGAG

CN 120013 ATCTGAATTTGTTAGGTTGTGATTTGTGATTGCTGATCATGATGATCTTTAAAACAGGAG

CS17CS1133 ATCTGAATTTGTTAGGTTGTGATTTGTGATTGCTGATCATGATGATCTTTAAAACAGGAG

CN 120030 ATCTGAATTTGTTAGGTTGTGATTTGTGATTGCTGATCATGATGATCTTTAAAACAGGAG

CO46 NCBI ATCTGAATTTGTTAGGTTGTGATTTGTGATTGCTGATCATGATGATCTTTAAAACAGGAG

DH55 ref genome ATCTGAATTTGTTAGGTTGTGATTTGTGATTGCTGATCATGATGATCTTTAAAACAGGAG

Hoga ATCTGAATTTGTTAGGTTGTGATTTGTGATTGCTGATCATGATGATCTTTAAAACAGGAG

CAM 236 ATCTGAATTTGTTAGGTTGTGATTTGTGATTGCTGATCATGATGATCTTTAAAACAGGAG

09-CS0040 ATCTGAATTTGTTAGGTTGTGATTTGTGATTGCTGATCATGATGATCTTTAAAACAGGAG

CN 119205 ATCTGAATTTGTTAGGTTGTGATTTGTGATTGCTGATCATGATGATCTTTAAAACAGGAG

Yellowstone ATCTGAATTTGTTAGGTTGTGATTTGTGATTGCTGATCATGATGATCTTTAAAACAGGAG

CN 120017 ATCTGAATTTGTTAGGTTGTGATTTGTGATTGCTGATCATGATGATCTTTAAAACAGGAG

CN 119300 ATCTGAATTTGTTAGGTTGTGATTTGTGATTGCTGATCATGATGATCTTTAAAACAGGAG

Jasper ATCTGAATTTGTTAGGTTGTGATTTGTGATTGCTGATCATGATGATCTTTAAAACAGGAG

CN 120027 ATCTGAATTTGTTAGGTTGTGATTTGTGATTGCTGATCATGATGATCTTTAAAACAGGAG

CN 113754 ATCTGAATTTGTTAGGTTGTGATTTGTGATTGCTGATCATGATGATCTTTAAAACAGGAG

Joelle AAFC ATCTGAATTTGTTAGGTTGTGATTTGTGATTGCTGATCATGATGATCTTTAAAACAGGAG

Joelle NCBI ATCTGAATTTGTTAGGTTGTGATTTGTGATTGCTGATCATGATGATCTTTAAAACAGGAG

Joelle phyto ATCTGAATTTGTTAGGTTGTGATTTGTGATTGCTGATCATGATGATCTTTAAAACAGGAG

Blaine Creek ATCTGAATTTGTTAGGTTGTGATTTGTGATTGCTGATCATGATGATCTTTAAAACAGGAG

CAM 241 ATCTGAATTTGTTAGGTTGTGATTTGTGATTGCTGATCATGATGATCTTTAAAACAGGAG

CN 119294 ATCTGAATTTGTTAGGTTGTGATTTGTGATTGCTGATCATGATGATCTTTAAAACAGGAG

************** ********************* ***********************

CN 120025 AAATTGCTGCAAGAAGAGAACAAGGTTTTGGCTAGCCAGGTAACGAAAGCTACTTTTTCT

CN 120013 AAATTGCTGAAAGAAGAGAACCAGGTTTTGGCTAGCCAGGTAACGAAAGCTACTTTTTCT

CS17CS1133 AAATTGCTGAAAGAAGAGAACCAGGTTTTGGCTAGCCAGGTAACGAAAGCTACTTTTTCT

CN 120030 AAATTGCTGAAAGAAGAGAACCAGGTTTTGGCTAGCCAGGTAACGAAAGCTACTTTTTCT

CO46 NCBI AAATTGCTGAAAGAAGAGAACCAGGTTTTGGCTAGCCAGGTAACGAAAGCTACTTTTTCT

DH55 ref genome AAATTGCTGAAAGAAGAGAACCAGGTTTTGGCTAGCCAGGTAACGAAAGCTACTTTTTCT

Hoga AAATTGCTGAAAGAAGAGAACCAGGTTTTGGCTAGCCAGGTAACGAAAGCTACTTTTTCT

CAM 236 AAATTGCTGAAAGAAGAGAACCAGGTTTTGGCTAGCCAGGTAACGAAAGCTACTTTTTCT

09-CS0040 AAATTGCTGAAAGAAGAGAACCAGGTTTTGGCTAGCCAGGTAACGAAAGCTACTTTTTCT

CN 119205 AAATTGCTGAAAGAAGAGAACCAGGTTTTGGCTAGCCAGGTAACGAAAGCTACTTTTTCT

Yellowstone AAATTGCTGAAAGAAGAGAACCAGGTTTTGGCTAGCCAGGTAACGAAAGCTACTTTTTCT

CN 120017 AAATTGCTGAAAGAAGAGAACCAGGTTTTGGCTAGCCAGGTAACGAAAGCTACTTTTTCT

CN 119300 AAATTGCTGAAAGAAGAGAACCAGGTTTTGGCTAGCCAGGTAACGAAAGCTACTTTTTCT

Jasper AAATTGCTGAAAGAAGAGAACCAGGTTTTGGCTAGCCAGGTAACGAAAGCTACTTTTTCT

CN 120027 AAATTGCTGAAAGAAGAGAACCAGGTTTTGGCTAGCCAGGTAACGAAAGCTACTTTTTCT

CN 113754 AAATTGCTGAAAGAAGAGAACCAGGTTTTGGCTAGCCAGGTAACGAAAGCTACTTTTTCT

Joelle AAFC AAATTGCTGAAAGAAGAGAACCAGGTTTTGGCTAGCCAGGTAACGAAAGCTACTTTTTCT

Joelle NCBI AAATTGCTGAAAGAAGAGAACCAGGTTTTGGCTAGCCAGGTAACGAAAGCTACTTTTTCT

Joelle phyto AAATTGCTGAAAGAAGAGAACCAGGTTTTGGCTAGCCAGGTAACGAAAGCTACTTTTTCT

Blaine Creek AAATTGCTGAAAGAAGAGAACCAGGTTTTGGCTAGCCAGGTAACGAAAGCTACTTTTTCT

CAM 241 AAATTGCTGAAAGAAGAGAACCAGGTTTTGGCTAGCCAGGTAACGAAAGCTACTTTTTCT

CN 119294 AAATTGCTGAAAGAAGAGAACCAGGTTTTGGCTAGCCAGGTAACGAAAGCTACTTTTTCT

********* *********** **************************************

CN 120025 ATATATACGCATACCTAATAAGCCATTCTTTCCACTTTAAGCATTTACTAGTTAAAACGT

CN 120013 ATATATATGCATACCTAATAAGCCATTCTTTCCACTTTAAGCATTTACTAGGTAGCACTT

CS17CS1133 ATATATATGCATACCTAATAAGCCATTCTTTCCACTTTAAGCATTTACTAGGTAGCACTT

CN 120030 ATATATATGCATACCTAATAAGCCATTCTTTCCACTTTAAGCATTTACTAGGTAGCACTT

CO46 NCBI ATATATATGCATACCTAATAAGCCATTCTTTCCACTTTAAGCATTTACTAGGTAGCACTT

DH55 ref genome ATATATATGCATACCTAATAAGCCATTCTTTCCACTTTAAGCATTTACTAGGTAGCACTT

Hoga ATATATATGCATACCTAATAAGCCATTCTTTCCACTTTAAGCATTTACTAGGTAGCACTT

CAM 236 ATATATATGCATACCTAATAAGCCATTCTTTCCACTTTAAGCATTTACTAGGTAGCACTT

09-CS0040 ATATATATGCATACCTAATAAGCCATTCTTTCCACTTTAAGCATTTACTAGGTAGCACTT

CN 119205 ATATATATGCATACCTAATAAGCCATTCTTTCCACTTTAAGCATTTACTAGGTAGCACTT

Yellowstone ATATATATGCATACCTAATAAGCCATTCTTTCCACTTTAAGCATTTACTAGGTAGCACTT

CN 120017 ATATATATGCATACCTAATAAGCCATTCTTTCCACTTTAAGCATTTACTAGGTAGCACTT

CN 119300 ATATATATGCATACCTAATAAGCCATTCTTTCCACTTTAAGCATTTACTAGGTAGCACTT

Jasper ATATATATGCATACCTAATAAGCCATTCTTTCCACTTTAAGCATTTACTAGGTAGCACTT

CN 120027 ATATATATGCATACCTAATAAGCCATTCTTTCCACTTTAAGCATTTACTAGGTAGCACTT

CN 113754 ATATATATGCATACCTAATAAGCCATTCTTTCCACTTTAAGCATTTACTAGGTAGCACTT

Joelle AAFC ATATATATGCATACCTAATAAGCCATTCTTTCCACTTTAAGCATTTACTAGGTAGCACTT

Joelle NCBI ATATATATGCATACCTAATAAGCCATTCTTTCCACTTTAAGCATTTACTAGGTAGCACTT

Joelle phyto ATATATATGCATACCTAATAAGCCATTCTTTCCACTTTAAGCATTTACTAGGTAGCACTT

Blaine Creek ATATATATGCATACCTAATAAGCCATTCTTTCCACTTTAAGCATTTACTAGGTAGCACTT

CAM 241 ATATATATGCATACCTAATAAGCCATTCTTTCCACTTTAAGCATTTACTAGGTAGCACTT

CN 119294 ATATATATGCATACCTAATAAGCCATTCTTTCCACTTTAAGCATTTACTAGGTAGCACTT

******* ******************************************* ** ** *

CN 120025 CTCTGTCTTGTGTTCTAGAAACATTACTTCTCAAGGTTAG-------GGTTTTTGGTAGA

CN 120013 CTGTGTCTTGTATTCTAGAAATTTTATTTCTCAAGATTAGGCATTTTGGTTGTTGGTAGA

CS17CS1133 CTGTGTCTTGTATTCTAGAAATTTTATTTCTCAAGATTAGGCATTTTGGTTGTTGGTAGA

CN 120030 CTGTGTCTTGTATTCTAGAAATTTTATTTCTCAAGATTAGGCATTTTGGTTGTTGGTAGA

CO46 NCBI CTGTGTCTTGTATTCTAGAAATTTTATTTCTCAAGATTAGGCATTTTGGTTGTTGGTAGA

DH55 ref genome CTGTGTCTTGTATTCTAGAAATTTTATTTCTCAAGATTAGGCATTTTGGTTGTTGGTAGA

Hoga CTGTGTCTTGTATTCTAGAAATTTTATTTCTCAAGATTAGGCATTTTGGTTGTTGGTAGA

CAM 236 CTGTGTCTTGTATTCTAGAAATTTTATTTCTCAAGATTAGGCATTTTGGTTGTTGGTAGA

09-CS0040 CTGTGTCTTGTATTCTAGAAATTTTATTTCTCAAGATTAGGCATTTTGGTTGTTGGTAGA

CN 119205 CTGTGTCTTGTATTCTAGAAATTTTATTTCTCAAGATTAGGCATTTTGGTTGTTGGTAGA

Yellowstone CTGTGTCTTGTATTCTAGAAATTTTATTTCTCAAGATTAGGCATTTTGGTTGTTGGTAGA

CN 120017 CTGTGTCTTGTATTCTAGAAATTTTATTTCTCAAGATTAGGCATTTTGGTTGTTGGTAGA

CN 119300 CTGTGTCTTGTATTCTAGAAATTTTATTTCTCAAGATTAGGCATTTTGGTTGTTGGTAGA

Jasper CTGTGTCTTGTATTCTAGAAATTTTATTTCTCAAGATTAGGCATTTTGGTTGTTGGTAGA

CN 120027 CTGTGTCTTGTATTCTAGAAATTTTATTTCTCAAGATTAGGCATTTTGGTTGTTGGTAGA

CN 113754 CTGTGTCTTGTATTCTAGAAATTTTATTTCTCAAGATTAGGCATTTTGGTTGTTGGTAGA

Joelle AAFC CTGTGTCTTGTATTCTAGAAATTTTATTTCTCAAGATTAGGCATTTTGGTTGTTGGTAGA

Joelle NCBI CTGTGTCTTGTATTCTAGAAATTTTATTTCTCAAGATTAGGCATTTTGGTTGTTGGTAGA

Joelle phyto CTGTGTCTTGTATTCTAGAAATTTTATTTCTCAAGATTAGGCATTTTGGTTGTTGGTAGA

Blaine Creek CTGTGTCTTGTATTCTAGAAATTTTATTTCTCAAGATTAGGCATTTTGGTTGTTGGTAGA

CAM 241 CTGTGTCTTGTATTCTAGAAATTTTATTTCTCAAGATTAGGCATTTTGGTTGTTGGTAGA

CN 119294 CTGTGTCTTGTATTCTAGAAATTTTATTTCTCAAGATTAGGCATTTTGGTTGTTGGTAGA

** ******** ********* *** ******** **** **** ********

CN 120025 TTAGG---TATTAGGGTTTGTGAGATTATTACTGCTGAATAAGGACAGAAATTTGATTCG

CN 120013 TTAGGTATTATTAGGGTTTGTGAGATTGTTACTGCTGAATAAGGACAGAGATTTGATTCG

CS17CS1133 TTAGGTATTATTAGGGTTTGTGAGATTGTTACTGCTGAATAAGGACAGAGATTTGATTCG

CN 120030 TTAGGTATTATTAGGGTTTGTGAGATTGTTACTGCTGAATAAGGACAGAGATTTGATTCG

CO46 NCBI TTAGGTATTATTAGGGTTTGTGAGATTGTTACTGCTGAATAAGGACAGAGATTTGATTCG

DH55 ref genome TTAGGTATTATTAGGGTTTGTGAGATTGTTACTGCTGAATAAGGACAGAGATTTGATTCG

Hoga TTAGGTATTATTAGGGTTTGTGAGATTGTTACTGCTGAATAAGGACAGAGATTTGATTCG

CAM 236 TTAGGTATTATTAGGGTTTGTGAGATTGTTACTGCTGAATAAGGACAGAGATTTGATTCG

09-CS0040 TTAGGTATTATTAGGGTTTGTGAGATTGTTACTGCTGAATAAGGACAGAGATTTGATTCG

CN 119205 TTAGGTATTATTAGGGTTTGTGAGATTGTTACTGCTGAATAAGGACAGAGATTTGATTCG

Yellowstone TTAGGTATTATTAGGGTTTGTGAGATTGTTACTGCTGAATAAGGACAGAGATTTGATTCG

CN 120017 TTAGGTATTATTAGGGTTTGTGAGATTGTTACTGCTGAATAAGGACAGAGATTTGATTCG

CN 119300 TTAGGTATTATTAGGGTTTGTGAGATTGTTACTGCTGAATAAGGACAGAGATTTGATTCG

Jasper TTAGGTATTATTAGGGTTTGTGAGATTGTTACTGCTGAATAAGGACAGAGATTTGATTCG

CN 120027 TTAGGTATTATTAGGGTTTGTGAGATTGTTACTGCTGAATAAGGACAGAGATTTGATTCG

CN 113754 TTAGGTATTATTAGGGTTTGTGAGATTGTTACTGCTGAATAAGGACAGAGATTTGATTCG

Joelle AAFC TTAGGTATTATTAGGGTTTGTGAGATTGTTACTGCTGAATAAGGACAGAGATTTGATTCG

Joelle NCBI TTAGGTATTATTAGGGTTTGTGAGATTGTTACTGCTGAATAAGGACAGAGATTTGATTCG

Joelle phyto TTAGGTATTATTAGGGTTTGTGAGATTGTTACTGCTGAATAAGGACAGAGATTTGATTCG

Blaine Creek TTAGGTATTATTAGGGTTTGTGAGATTGTTACTGCTGAATAAGGACAGAGATTTGATTCG

CAM 241 TTAGGTATTATTAGGGTTTGTGAGATTGTTACTGCTGAATAAGGACAGAGATTTGATTCG

CN 119294 TTAGGTATTATTAGGGTTTGTGAGATTGTTACTGCTGAATAAGGACAGAGATTTGATTCG

***** ******************* ********************* **********

CN 120025 GTCT-----------GGAAAATTTTGATTCGGATTTTAGAATGACAAATAAAAAGTTTAA

CN 120013 GTCTGGTTTGAGTTAGGGAAACTTTGATTCGGATTTTAGAATGACAGATGAAAAGTTTAA

CS17CS1133 GTCTGGTTTGAGTTAGGGAAACTTTGATTCGGATTTTAGAATGACAGATGAAAAGTTTAA

CN 120030 GTCTGGTTTGAGTTAGGGAAACTTTGATTCGGATTTTAGAATGACAGATGAAAAGTTTAA

CO46 NCBI GTCTGGTTTGAGTTAGGGAAACTTTGATTCGGATTTTAGAATGACAGATGAAAAGTTTAA

DH55 ref genome GTCTGGTTTGAGTTAGGGAAACTTTGATTCGGATTTTAGAATGACAGATGAAAAGTTTAA

Hoga GTCTGGTTTGAGTTAGGGAAACTTTGATTCGGATTTTAGAATGACAGATGAAAAGTTTAA

CAM 236 GTCTGGTTTGAGTTAGGGAAACTTTGATTCGGATTTTAGAATGACAGATGAAAAGTTTAA

09-CS0040 GTCTGGTTTGAGTTAGGGAAACTTTGATTCGGATTTTAGAATGACAGATGAAAAGTTTAA

CN 119205 GTCTGGTTTGAGTTAGGGAAACTTTGATTCGGATTTTAGAATGACAGATGAAAAGTTTAA

Yellowstone GTCTGGTTTGAGTTAGGGAAACTTTGATTCGGATTTTAGAATGACAGATGAAAAGTTTAA

CN 120017 GTCTGGTTTGAGTTAGGGAAACTTTGATTCGGATTTTAGAATGACAGATGAAAAGTTTAA

CN 119300 GTCTGGTTTGAGTTAGGGAAACTTTGATTCGGATTTTAGAATGACAGATGAAAAGTTTAA

Jasper GTCTGGTTTGAGTTAGGGAAACTTTGATTCGGATTTTAGAATGACAGATGAAAAGTTTAA

CN 120027 GTCTGGTTTGAGTTAGGGAAACTTTGATTCGGATTTTAGAATGACAGATGAAAAGTTTAA

CN 113754 GTCTGGTTTGAGTTAGGGAAACTTTGATTCGGATTTTAGAATGACAGATGAAAAGTTTAA

Joelle AAFC GTCTGGTTTGAGTTAGGGAAACTTTGATTCGGATTTTAGAATGACAGATGAAAAGTTTAA

Joelle NCBI GTCTGGTTTGAGTTAGGGAAACTTTGATTCGGATTTTAGAATGACAGATGAAAAGTTTAA

Joelle phyto GTCTGGTTTGAGTTAGGGAAACTTTGATTCGGATTTTAGAATGACAGATGAAAAGTTTAA

Blaine Creek GTCTGGTTTGAGTTAGGGAAACTTTGATTCGGATTTTAGAATGACAGATGAAAAGTTTAA

CAM 241 GTCTGGTTTGAGTTAGGGAAACTTTGATTCGGATTTTAGAATGACAGATGAAAAGTTTAA

CN 119294 GTCTGGTTTGAGTTAGGGAAACTTTGATTCGGATTTTAGAATGACAGATGAAAAGTTTAA

**** ** *** ************************ ** **********

CN 120025 TATAGTTTTAAGATAGTAATTTATTTATAAAAGACACACAAACAAACAAAAATCATGAAC

CN 120013 TATAATTTTAAG--------------ATAAAAGACACACAAACAAACAAAATTCATGAAC

CS17CS1133 TATAATTTTAAG--------------ATAAAAGACACACAAACAAACAAAATTCATGAAC

CN 120030 TATAATTTTAAG--------------ATAAAAGACACACAAACAAACAAAATTCATGAAC

CO46 NCBI TATAATTTTAAG--------------ATAAAAGACACACAAACAAACAAAATTCATGAAC

DH55 ref genome TATAATTTTAAG--------------ATAAAAGACACACAAACAAACAAAATTCATGAAC

Hoga TATAATTTTAAG--------------ATAAAAGACACACAAACAAACAAAATTCATGAAC

CAM 236 TATAATTTTAAG--------------ATAAAAGACACACAAACAAACAAAATTCATGAAC

09-CS0040 TATAATTTTAAG--------------ATAAAAGACACACAAACAAACAAAATTCATGAAC

CN 119205 TATAATTTTAAG--------------ATAAAAGACACACAAACAAACAAAATTCATGAAC

Yellowstone TATAATTTTAAG--------------ATAAAAGACACACAAACAAACAAAATTCATGAAC

CN 120017 TATAATTTTAAG--------------ATAAAAGACACACAAACAAACAAAATTCATGAAC

CN 119300 TATAATTTTAAG--------------ATAAAAGACACACAAACAAACAAAATTCATGAAC

Jasper TATAATTTTAAG--------------ATAAAAGACACACAAACAAACAAAATTCATGAAC

CN 120027 TATAATTTTAAG--------------ATAAAAGACACACAAACAAACAAAATTCATGAAC

CN 113754 TATAATTTTAAG--------------ATAAAAGACACACAAACAAACAAAATTCATGAAC

Joelle AAFC TATAATTTTAAG--------------ATAAAAGACACACAAACAAACAAAATTCATGAAC

Joelle NCBI TATAATTTTAAG--------------ATAAAAGACACACAAACAAACAAAATTCATGAAC

Joelle phyto TATAATTTTAAG--------------ATAAAAGACACACAAACAAACAAAATTCATGAAC

Blaine Creek TATAATTTTAAG--------------ATAAAAGACACACAAACAAACAAAATTCATGAAC

CAM 241 TATAATTTTAAG--------------ATAAAAGACACACAAACAAACAAAATTCATGAAC

CN 119294 TATAATTTTAAG--------------ATAAAAGACACACAAACAAACAAAATTCATGAAC

**** ******* ************************* ********

CN 120025 AGTATAAATTCATTATTACATAATAATTTGTTCAGTGAATTATATATTTTTTGTAACGAT

CN 120013 AGTATAATTTCATTATTACATAATAATTTGTTCGGTAAAATATATA-TTTTTATAACGAT

CS17CS1133 AGTATAATTTCATTATTACATAATAATTTGTTCGGTAAAATATATA-TTTTTATAACGAT

CN 120030 AGTATAATTTCATTATTACATAATAATTTGTTCGGTAAAATATATA-TTTTTATAACGAT

CO46 NCBI AGTATAATTTCATTATTACATAATAATTTGTTCGGTAAAATATATA-TTTTTATAACGAT

DH55 ref genome AGTATAATTTCATTATTACATAATAATTTGTTCGGTAAAATATATA-TTTTTATAACGAT

Hoga AGTATAATTTCATTATTACATAATAATTTGTTCGGTAAAATATATA-TTTTTATAACGAT

CAM 236 AGTATAATTTCATTATTACATAATAATTTGTTCGGTAAAATATATA-TTTTTATAACGAT

09-CS0040 AGTATAATTTCATTATTACATAATAATTTGTTCGGTAAAATATATA-TTTTTATAACGAT

CN 119205 AGTATAATTTCATTATTACATAATAATTTGTTCGGTAAAATATATA-TTTTTATAACGAT

Yellowstone AGTATAATTTCATTATTACATAATAATTTGTTCGGTAAAATATATA-TTTTTATAACGAT

CN 120017 AGTATAATTTCATTATTACATAATAATTTGTTCGGTAAAATATATA-TTTTTATAACGAT

CN 119300 AGTATAATTTCATTATTACATAATAATTTGTTCGGTAAAATATATA-TTTTTATAACGAT

Jasper AGTATAATTTCATTATTACATAATAATTTGTTCGGTAAAATATATA-TTTTTATAACGAT

CN 120027 AGTATAATTTCATTATTACATAATAATTTGTTCGGTAAAATATATA-TTTTTATAACGAT

CN 113754 AGTATAATTTCATTATTACATAATAATTTGTTCGGTAAAATATATA-TTTTTATAACGAT

Joelle AAFC AGTATAATTTCATTATTACATAATAATTTGTTCGGTAAAATATATA-TTTTTATAACGAT

Joelle NCBI AGTATAATTTCATTATTACATAATAATTTGTTCGGTAAAATATATA-TTTTTATAACGAT

Joelle phyto AGTATAATTTCATTATTACATAATAATTTGTTCGGTAAAATATATA-TTTTTATAACGAT

Blaine Creek AGTATAATTTCATTATTACATAATAATTTGTTCGGTAAAATATATA-TTTTTATAACGAT

CAM 241 AGTATAATTTCATTATTACATAATAATTTGTTCGGTAAAATATATA-TTTTTATAACGAT

CN 119294 AGTATAATTTCATTATTACATAATAATTTGTTCGGTAAAATATATA-TTTTTATAACGAT

******* ************************* ** ** ****** ***** *******

CN 120025 ----------TCATTGTACTAAAATAATATATACTTTTCTGTTGGTGGAAATAATATTAG

CN 120013 TATTGCTATATCATTTCACTAAAATAATATATACTTTTCTGTTGGTGCGAGTAATATTAG

CS17CS1133 TATTGCTATATCATTTCACTAAAATAATATATACTTTTCTGTTGGTGCGAGTAATATTAG

CN 120030 TATTGCTATATCATTTCACTAAAATAATATATACTTTTCTGTTGGTGCGAGTAATATTAG

CO46 NCBI TATTGCTATATCATTTCACTAAAATAATATATACTTTTCTGTTGGTGCGAGTAATATTAG

DH55 ref genome TATTGCTATATCATTTCACTAAAATAATATATACTTTTCTGTTGGTGCGAGTAATATTAG

Hoga TATTGCTATATCATTTCACTAAAATAATATATACTTTTCTGTTGGTGCGAGTAATATTAG

CAM 236 TATTGCTATATCATTTCACTAAAATAATATATACTTTTCTGTTGGTGCGAGTAATATTAG

09-CS0040 TATTGCTATATCATTTCACTAAAATAATATATACTTTTCTGTTGGTGCGAGTAATATTAG

CN 119205 TATTGCTATATCATTTCACTAAAATAATATATACTTTTCTGTTGGTGCGAGTAATATTAG

Yellowstone TATTGCTATATCATTTCACTAAAATAATATATACTTTTCTGTTGGTGCGAGTAATATTAG

CN 120017 TATTGCTATATCATTTCACTAAAATAATATATACTTTTCTGTTGGTGCGAGTAATATTAG

CN 119300 TATTGCTATATCATTTCACTAAAATAATATATACTTTTCTGTTGGTGCGAGTAATATTAG

Jasper TATTGCTATATCATTTCACTAAAATAATATATACTTTTCTGTTGGTGCGAGTAATATTAG

CN 120027 TATTGCTATATCATTTCACTAAAATAATATATACTTTTCTGTTGGTGCGAGTAATATTAG

CN 113754 TATTGCTATATCATTTCACTAAAATAATATATACTTTTCTGTTGGTGCGAGTAATATTAG

Joelle AAFC TATTGCTATATCATTTCACTAAAATAATATATACTTTTCTGTTGGTGCGAGTAATATTAG

Joelle NCBI TATTGCTATATCATTTCACTAAAATAATATATACTTTTCTGTTGGTGCGAGTAATATTAG

Joelle phyto TATTGCTATATCATTTCACTAAAATAATATATACTTTTCTGTTGGTGCGAGTAATATTAG

Blaine Creek TATTGCTATATCATTTCACTAAAATAATATATACTTTTCTGTTGGTGCGAGTAATATTAG

CAM 241 TATTGCTATATCATTTCACTAAAATAATATATACTTTTCTGTTGGTGCGAGTAATATTAG

CN 119294 TATTGCTATATCATTTCACTAAAATAATATATACTTTTCTGTTGGTGCGAGTAATATTAG

***** ****************************** * *********

CN 120025 GTTTTGGTTGTTCTTTTTCTGGATTTGGG---ATAAAGGTTTTAGTTAGGTTTTGGTTCA

CN 120013 GTTTTGGTTGTTCTTTTTCTGGATTTGGGATAATAAAGGTTTTAATTAGGTTTTGGTTCA

CS17CS1133 GTTTTGGTTGTTCTTTTTCTGGATTTGGGATAATAAAGGTTTTAATTAGGTTTTGGTTCA

CN 120030 GTTTTGGTTGTTCTTTTTCTGGATTTGGGATAATAAAGGTTTTAATTAGGTTTTGGTTCA

CO46 NCBI GTTTTGGTTGTTCTTTTTCTGGATTTGGGATAATAAAGGTTTTAATTAGGTTTTGGTTCA

DH55 ref genome GTTTTGGTTGTTCTTTTTCTGGATTTGGGATAATAAAGGTTTTAATTAGGTTTTGGTTCA

Hoga GTTTTGGTTGTTCTTTTTCTGGATTTGGGATAATAAAGGTTTTAATTAGGTTTTGGTTCA

CAM 236 GTTTTGGTTGTTCTTTTTCTGGATTTGGGATAATAAAGGTTTTAATTAGGTTTTGGTTCA

09-CS0040 GTTTTGGTTGTTCTTTTTCTGGATTTGGGATAATAAAGGTTTTAATTAGGTTTTGGTTCA

CN 119205 GTTTTGGTTGTTCTTTTTCTGGATTTGGGATAATAAAGGTTTTAATTAGGTTTTGGTTCA

Yellowstone GTTTTGGTTGTTCTTTTTCTGGATTTGGGATAATAAAGGTTTTAATTAGGTTTTGGTTCA

CN 120017 GTTTTGGTTGTTCTTTTTCTGGATTTGGGATAATAAAGGTTTTAATTAGGTTTTGGTTCA

CN 119300 GTTTTGGTTGTTCTTTTTCTGGATTTGGGATAATAAAGGTTTTAATTAGGTTTTGGTTCA

Jasper GTTTTGGTTGTTCTTTTTCTGGATTTGGGATAATAAAGGTTTTAATTAGGTTTTGGTTCA

CN 120027 GTTTTGGTTGTTCTTTTTCTGGATTTGGGATAATAAAGGTTTTAATTAGGTTTTGGTTCA

CN 113754 GTTTTGGTTGTTCTTTTTCTGGATTTGGGATAATAAAGGTTTTAATTAGGTTTTGGTTCA

Joelle AAFC GTTTTGGTTGTTCTTTTTCTGGATTTGGGATAATAAAGGTTTTAATTAGGTTTTGGTTCA

Joelle NCBI GTTTTGGTTGTTCTTTTTCTGGATTTGGGATAATAAAGGTTTTAATTAGGTTTTGGTTCA

Joelle phyto GTTTTGGTTGTTCTTTTTCTGGATTTGGGATAATAAAGGTTTTAATTAGGTTTTGGTTCA

Blaine Creek GTTTTGGTTGTTCTTTTTCTGGATTTGGGATAATAAAGGTTTTAATTAGGTTTTGGTTCA

CAM 241 GTTTTGGTTGTTCTTTTTCTGGATTTGGGATAATAAAGGTTTTAATTAGGTTTTGGTTCA

CN 119294 GTTTTGGTTGTTCTTTTTCTGGATTTGGGATAATAAAGGTTTTAATTAGGTTTTGGTTCA

***************************** ************ ***************

CN 120025 GTTTGAGATTTACGAACGGATTAGTTTTTTATGCGTCATGGTTAAGGTTTGGATCCAAGT

CN 120013 GTTTGAGATTTCTGAACGGATTAGTTTTTTGTGCGTCATGGTTAAGGTTTGGATCCATGT

CS17CS1133 GTTTGAGATTTCTGAACGGATTAGTTTTTTGTGCGTCATGGTTAAGGTTTGGATCCATGT

CN 120030 GTTTGAGATTTCTGAACGGATTAGTTTTTTGTGCGTCATGGTTAAGGTTTGGATCCATGT

CO46 NCBI GTTTGAGATTTCTGAACGGATTAGTTTTTTGTGCGTCATGGTTAAGGTTTGGATCCATGT

DH55 ref genome GTTTGAGATTTCTGAACGGATTAGTTTTTTGTGCGTCATGGTTAAGGTTTGGATCCATGT

Hoga GTTTGAGATTTCTGAACGGATTAGTTTTTTGTGCGTCATGGTTAAGGTTTGGATCCATGT

CAM 236 GTTTGAGATTTCTGAACGGATTAGTTTTTTGTGCGTCATGGTTAAGGTTTGGATCCATGT

09-CS0040 GTTTGAGATTTCTGAACGGATTAGTTTTTTGTGCGTCATGGTTAAGGTTTGGATCCATGT

CN 119205 GTTTGAGATTTCTGAACGGATTAGTTTTTTGTGCGTCATGGTTAAGGTTTGGATCCATGT

Yellowstone GTTTGAGATTTCTGAACGGATTAGTTTTTTGTGCGTCATGGTTAAGGTTTGGATCCATGT

CN 120017 GTTTGAGATTTCTGAACGGATTAGTTTTTTGTGCGTCATGGTTAAGGTTTGGATCCATGT

CN 119300 GTTTGAGATTTCTGAACGGATTAGTTTTTTGTGCGTCATGGTTAAGGTTTGGATCCATGT

Jasper GTTTGAGATTTCTGAACGGATTAGTTTTTTGTGCGTCATGGTTAAGGTTTGGATCCATGT

CN 120027 GTTTGAGATTTCTGAACGGATTAGTTTTTTGTGCGTCATGGTTAAGGTTTGGATCCATGT

CN 113754 GTTTGAGATTTCTGAACGGATTAGTTTTTTGTGCGTCATGGTTAAGGTTTGGATCCATGT

Joelle AAFC GTTTGAGATTTCTGAACGGATTAGTTTTTTGTGCGTCATGGTTAAGGTTTGGATCCATGT

Joelle NCBI GTTTGAGATTTCTGAACGGATTAGTTTTTTGTGCGTCATGGTTAAGGTTTGGATCCATGT

Joelle phyto GTTTGAGATTTCTGAACGGATTAGTTTTTTGTGCGTCATGGTTAAGGTTTGGATCCATGT

Blaine Creek GTTTGAGATTTCTGAACGGATTAGTTTTTTGTGCGTCATGGTTAAGGTTTGGATCCATGT

CAM 241 GTTTGAGATTTCTGAACGGATTAGTTTTTTGTGCGTCATGGTTAAGGTTTGGATCCATGT

CN 119294 GTTTGAGATTTCTGAACGGATTAGTTTTTTGTGCGTCATGGTTAAGGTTTGGATCCATGT

*********** ***************** ************************** **

CN 120025 GTCAAGTAGTCTAGCTACTTTTACATCCTCAAGGTTAAATTATAAACAGGGAAGTAGTCT

CN 120013 GTCCAGTAGTCTAGCTACTTTTACATCCTCAAGGTTAAATTATAAACAGGGAAGTAGTCT

CS17CS1133 GTCCAGTAGTCTAGCTACTTTTACATCCTCAAGGTTAAATTATAAACAGGGAAGTAGTCT

CN 120030 GTCCAGTAGTCTAGCTACTTTTACATCCTCAAGGTTAAATTATAAACAGGGAAGTAGTCT

CO46 NCBI GTCCAGTAGTCTAGCTACTTTTACATCCTCAAGGTTAAATTATAAACAGGGAAGTAGTCT

DH55 ref genome GTCCAGTAGTCTAGCTACTTTTACATCCTCAAGGTTAAATTATAAACAGGGAAGTAGTCT

Hoga GTCCAGTAGTCTAGCTACTTTTACATCCTCAAGGTTAAATTATAAACAGGGAAGTAGTCT

CAM 236 GTCCAGTAGTCTAGCTACTTTTACATCCTCAAGGTTAAATTATAAACAGGGAAGTAGTCT

09-CS0040 GTCCAGTAGTCTAGCTACTTTTACATCCTCAAGGTTAAATTATAAACAGGGAAGTAGTCT

CN 119205 GTCCAGTAGTCTAGCTACTTTTACATCCTCAAGGTTAAATTATAAACAGGGAAGTAGTCT

Yellowstone GTCCAGTAGTCTAGCTACTTTTACATCCTCAAGGTTAAATTATAAACAGGGAAGTAGTCT

CN 120017 GTCCAGTAGTCTAGCTACTTTTACATCCTCAAGGTTAAATTATAAACAGGGAAGTAGTCT

CN 119300 GTCCAGTAGTCTAGCTACTTTTACATCCTCAAGGTTAAATTATAAACAGGGAAGTAGTCT

Jasper GTCCAGTAGTCTAGCTACTTTTACATCCTCAAGGTTAAATTATAAACAGGGAAGTAGTCT

CN 120027 GTCCAGTAGTCTAGCTACTTTTACATCCTCAAGGTTAAATTATAAACAGGGAAGTAGTCT

CN 113754 GTCCAGTAGTCTAGCTACTTTTACATCCTCAAGGTTAAATTATAAACAGGGAAGTAGTCT

Joelle AAFC GTCCAGTAGTCTAGCTACTTTTACATCCTCAAGGTTAAATTATAAACAGGGAAGTAGTCT

Joelle NCBI GTCCAGTAGTCTAGCTACTTTTACATCCTCAAGGTTAAATTATAAACAGGGAAGTAGTCT

Joelle phyto GTCCAGTAGTCTAGCTACTTTTACATCCTCAAGGTTAAATTATAAACAGGGAAGTAGTCT

Blaine Creek GTCCAGTAGTCTAGCTACTTTTACATCCTCAAGGTTAAATTATAAACAGGGAAGTAGTCT

CAM 241 GTCCAGTAGTCTAGCTACTTTTACATCCTCAAGGTTAAATTATAAACAGGGAAGTAGTCT

CN 119294 GTCCAGTAGTCTAGCTACTTTTACATCCTCAAGGTTAAATTATAAACAGGGAAGTAGTCT

*** ********************************************************

CN 120025 AAAACAGACAAATTATGGAAGCAATATGGTGGACCGTGGACGAGTGTTAGTCTTAAATCG

CN 120013 AGAACAGACAAATTATGGAAGCAAAATGGTGGAAC-----TGAGTGTTAGTTTTAGATTG

CS17CS1133 AGAACAGACAAATTATGGAAGCAAAATGGTGGAAC-----TGAGTGTTAGTTTTAGATTG

CN 120030 AGAACAGACAAATTATGGAAGCAAAATGGTGGAAC-----TGAGTGTTAGTTTTAGATTG

CO46 NCBI AGAACAGACAAATTATGGAAGCAAAATGGTGGAAC-----TGAGTGTTAGTTTTAGATTG

DH55 ref genome AGAACAGACAAATTATGGAAGCAAAATGGTGGAAC-----TGAGTGTTAGTTTTAGATTG

Hoga AGAACAGACAAATTATGGAAGCAAAATGGTGGAAC-----TGAGTGTTAGTTTTAGATTG

CAM 236 AGAACAGACAAATTATGGAAGCAAAATGGTGGAAC-----TGAGTGTTAGTTTTAGATTG

09-CS0040 AGAACAGACAAATTATGGAAGCAAAATGGTGGAAC-----TGAGTGTTAGTTTTAGATTG

CN 119205 AGAACAGACAAATTATGGAAGCAAAATGGTGGAAC-----TGAGTGTTAGTTTTAGATTG

Yellowstone AGAACAGACAAATTATGGAAGCAAAATGGTGGAAC-----TGAGTGTTAGTTTTAGATTG

CN 120017 AGAACAGACAAATTATGGAAGCAAAATGGTGGAAC-----TGAGTGTTAGTTTTAGATTG

CN 119300 AGAACAGACAAATTATGGAAGCAAAATGGTGGAAC-----TGAGTGTTAGTTTTAGATTG

Jasper AGAACAGACAAATTATGGAAGCAAAATGGTGGAAC-----TGAGTGTTAGTTTTAGATTG

CN 120027 AGAACAGACAAATTATGGAAGCAAAATGGTGGAAC-----TGAGTGTTAGTTTTAGATTG

CN 113754 AGAACAGACAAATTATGGAAGCAAAATGGTGGAAC-----TGAGTGTTAGTTTTAGATTG

Joelle AAFC AGAACAGACAAATTATGGAAGCAAAATGGTGGAAC-----TGAGTGTTAGTTTTAGATTG

Joelle NCBI AGAACAGACAAATTATGGAAGCAAAATGGTGGAAC-----TGAGTGTTAGTTTTAGATTG

Joelle phyto AGAACAGACAAATTATGGAAGCAAAATGGTGGAAC-----TGAGTGTTAGTTTTAGATTG

Blaine Creek AGAACAGACAAATTATGGAAGCAAAATGGTGGAAC-----TGAGTGTTAGTTTTAGATTG

CAM 241 AGAACAGACAAATTATGGAAGCAAAATGGTGGAAC-----TGAGTGTTAGTTTTAGATTG

CN 119294 AGAACAGACAAATTATGGAAGCAAAATGGTGGAAC-----TGAGTGTTAGTTTTAGATTG

* ********************** ******** * ********** *** ** *

CN 120025 GTGGAATG--AAAAGAATTTTCATATATATGAGTAGAAGACAAAAAGA-AAAAAGTGAAT

CN 120013 GTGGAATGAAAAAAGAATATTCATATATG-----AGTAGACAAAAAGGTAAAAAGTGAAT

CS17CS1133 GTGGAATGAAAAAAGAATATTCATATATG-----AGTAGACAAAAAGGTAAAAAGTGAAT

CN 120030 GTGGAATGAAAAAAGAATATTCATATATG-----AGTAGACAAAAAGGTAAAAAGTGAAT

CO46 NCBI GTGGAATGAAAAAAGAATATTCATATATG-----AGTAGACAAACAGGTAAAAAGTGAAT

DH55 ref genome GTGGAATGAAAAAAGAATATTCATATATG-----AGTAGACAAAAAGGTAAAAAGTGAAT

Hoga GTGGAATGAAAAAAGAATATTCATATATG-----AGTAGACAAAAAGGTAAAAAGTGAAT

CAM 236 GTGGAATGAAAAAAGAATATTCATATATG-----AGTAGACAAAAAGGTAAAAAGTGAAT

09-CS0040 GTGGAATGAAAAAAGAATATTCATATATG-----AGTAGACAAAAAGGTAAAAAGTGAAT

CN 119205 GTGGAATGAAAAAAGAATATTCATATATG-----AGTAGACAAAAAGGTAAAAAGTGAAT

Yellowstone GTGGAATGAAAAAAGAATATTCATATATG-----AGTAGACAAAAAGGTAAAAAGTGAAT

CN 120017 GTGGAATGAAAAAAGAATATTCATATATG-----AGTAGACAAAAAGGTAAAAAGTGAAT

CN 119300 GTGGAATGAAAAAAGAATATTCATATATG-----AGTAGACAAAAAGGTAAAAAGTGAAT

Jasper GTGGAATGAAAAAAGAATATTCATATATG-----AGTAGACAAAAAGGTAAAAAGTGAAT

CN 120027 GTGGAATGAAAAAAGAATATTCATATATG-----AGTAGACAAAAAGGTAAAAAGTGAAT

CN 113754 GTGGAATGAAAAAAGAATATTCATATATG-----AGTAGACAAAAAGGTAAAAAGTGAAT

Joelle AAFC GTGGAATGAAAAAAGAATATTCATATATG-----AGTAGACAAAAAGGTAAAAAGTGAAT

Joelle NCBI GTGGAATGAAAAAAGAATATTCATATATG-----AGTAGACAAAAAGGTAAAAAGTGAAT

Joelle phyto GTGGAATGAAAAAAGAATATTCATATATG-----AGTAGACAAAAAGGTAAAAAGTGAAT

Blaine Creek GTGGAATGAAAAAAGAATATTCATATATG-----AGTAGACAAAAAGGTAAAAAGTGAAT

CAM 241 GTGGAATGAAAAAAGAATATTCATATATG-----AGTAGACAAAAAGGTAAAAAGTGAAT

CN 119294 GTGGAATGAAAAAAGAATATTCATATATG-----AGTAGACAAAAAGGTAAAAAGTGAAT

******** ******** ********* ** ******* ** ***********

CN 120025 AGTGATTTTGACCTATGATTATCGTACAGATGGAGACGAATCATGTTGTTGGAGCAGAAG

CN 120013 AGTGATTTTGACCTATGATTATCGTACAGATGGAGACGAATCATGTTGTTGGAGCAGAAG

CS17CS1133 AGTGATTTTGACCTATGATTATCGTACAGATGGAGACGAATCATGTTGTTGGAGCAGAAG

CN 120030 AGTGATTTTGACCTATGATTATCGTACAGATGGAGACGAATCATGTTGTTGGAGCAGAAG

CO46 NCBI AGTGATTTTGACCTATGATTATCGTACAGATGGAGACGAATCATGTTGTTGGAGCAGAAG

DH55 ref genome AGTGATTTTGACCTATGATTATCGTACAGATGGAGACGAATCATGTTGTTGGAGCAGAAG

Hoga AGTGATTTTGACCTATGATTATCGTACAGATGGAGACGAATCATGTTGTTGGAGCAGAAG

CAM 236 AGTGATTTTGACCTATGATTATCGTACAGATGGAGACGAATCATGTTGTTGGAGCAGAAG

09-CS0040 AGTGATTTTGACCTATGATTATCGTACAGATGGAGACGAATCATGTTGTTGGAGCAGAAG

CN 119205 AGTGATTTTGACCTATGATTATCGTACAGATGGAGACGAATCATGTTGTTGGAGCAGAAG

Yellowstone AGTGATTTTGACCTATGATTATCGTACAGATGGAGACGAATCATGTTGTTGGAGCAGAAG

CN 120017 AGTGATTTTGACCTATGATTATCGTACAGATGGAGACGAATCATGTTGTTGGAGCAGAAG

CN 119300 AGTGATTTTGACCTATGATTATCGTACAGATGGAGACGAATCATGTTGTTGGAGCAGAAG

Jasper AGTGATTTTGACCTATGATTATCGTACAGATGGAGACGAATCATGTTGTTGGAGCAGAAG

CN 120027 AGTGATTTTGACCTATGATTATCGTACAGATGGAGACGAATCATGTTGTTGGAGCAGAAG

CN 113754 AGTGATTTTGACCTATGATTATCGTACAGATGGAGACGAATCATGTTGTTGGAGCAGAAG

Joelle AAFC AGTGATTTTGACCTATGATTATCGTACAGATGGAGACGAATCATGTTGTTGGAGCAGAAG

Joelle NCBI AGTGATTTTGACCTATGATTATCGTACAGATGGAGACGAATCATGTTGTTGGAGCAGAAG

Joelle phyto AGTGATTTTGACCTATGATTATCGTACAGATGGAGACGAATCATGTTGTTGGAGCAGAAG

Blaine Creek AGTGATTTTGACCTATGATTATCGTACAGATGGAGACGAATCATGTTGTTGGAGCAGAAG

CAM 241 AGTGATTTTGACCTATGATTATCGTACAGATGGAGACGAATCATGTTGTTGGAGCAGAAG

CN 119294 AGTGATTTTGACCTATGATTATCGTACAGATGGAGACGAATCATGTTGTTGGAGCAGAAG

************************************************************

CN 120025 CTGATATGGAGATGGAGATGTCACCTGCTGGACAAATATCCGACAATCTTCCGGTGACTC

CN 120013 CTGATATGGAGATGGAGATGTCACCTGCTGGACAAATCTCCGACAATCTTCCGGTGACTC

CS17CS1133 CTGATATGGAGATGGAGATGTCACCTGCTGGACAAATCTCCGACAATCTTCCGGTGACTC

CN 120030 CTGATATGGAGATGGAGATGTCACCTGCTGGACAAATCTCCGACAATCTTCCGGTGACTC

CO46 NCBI CTGATATGGAGATGGAGATGTCACCTGCTGGACAAATCTCCGACAATCTTCCGGTGACTC

DH55 ref genome CTGATATGGAGATGGAGATGTCACCTGCTGGACAAATCTCCGACAATCTTCCGGTGACTC

Hoga CTGATATGGAGATGGAGATGTCACCTGCTGGACAAATCTCCGACAATCTTCCGGTGACTC

CAM 236 CTGATATGGAGATGGAGATGTCACCTGCTGGACAAATCTCCGACAATCTTCCGGTGACTC

09-CS0040 CTGATATGGAGATGGAGATGTCACCTGCTGGACAAATCTCCGACAATCTTCCGGTGACTC

CN 119205 CTGATATGGAGATGGAGATGTCACCTGCTGGACAAATCTCCGACAATCTTCCGGTGACTC

Yellowstone CTGATATGGAGATGGAGATGTCACCTGCTGGACAAATCTCCGACAATCTTCCGGTGACTC

CN 120017 CTGATATGGAGATGGAGATGTCACCTGCTGGACAAATCTCCGACAATCTTCCGGTGACTC

CN 119300 CTGATATGGAGATGGAGATGTCACCTGCTGGACAAATCTCCGACAATCTTCCGGTGACTC

Jasper CTGATATGGAGATGGAGATGTCACCTGCTGGACAAATCTCCGACAATCTTCCGGTGACTC

CN 120027 CTGATATGGAGATGGAGATGTCACCTGCTGGACAAATCTCCGACAATCTTCCGGTGACTC

CN 113754 CTGATATGGAGATGGAGATGTCACCTGCTGGACAAATCTCCGACAATCTTCCGGTGACTC

Joelle AAFC CTGATATGGAGATGGAGATGTCACCTGCTGGACAAATCTCCGACAATCTTCCGGTGACTC

Joelle NCBI CTGATATGGAGATGGAGATGTCACCTGCTGGACAAATCTCCGACAATCTTCCGGTGACTC

Joelle phyto CTGATATGGAGATGGAGATGTCACCTGCTGGACAAATCTCCGACAATCTTCCGGTGACTC

Blaine Creek CTGATATGGAGATGGAGATGTCACCTGCTGGACAAATCTCCGACAATCTTCCGGTGACTC

CAM 241 CTGATATGGAGATGGAGATGTCACCTGCTGGACAAATCTCCGACAATCTTCCGGTGACTC

CN 119294 CTGATATGGAGATGGAGATGTCACCTGCTGGACAAATCTCCGACAATCTTCCGGTGACTC

************************************* **********************

CN 120025 TCCCGCTGCTTAATTAGGGCGGTTGATATCAAAAATCCAAAACATATATATAATTAAGGG

CN 120013 TCCCGCTGCTCAATTAGGGCGGTTGATATCAAAAATCCAAAACATATATATAATTAAGGG

CS17CS1133 TCCCGCTGCTCAATTAGGGCGGTTGATATCAAAAATCCAAAACATATATATAATTAAGGG

CN 120030 TCCCGCTGCTCAATTAGGGCGGTTGATATCAAAAATCCAAAACATATATATAATTAAGGG

CO46 NCBI TCCCGCTGCTCAATTAGGGCGGTTGATATCAAAAATCCAAAACATATATATAATTAAGGG

DH55 ref genome TCCCGCTGCTCAATTAGGGCGGTTGATATCAAAAATCCAAAACATATATATAATTAAGGG

Hoga TCCCGCTGCTCAATTAGGGCGGTTGATATCAAAAATCCAAAACATATATATAATTAAGGG

CAM 236 TCCCGCTGCTCAATTAGGGCGGTTGATATCAAAAATCCAAAACATATATATAATTAAGGG

09-CS0040 TCCCGCTGCTCAATTAGGGCGGTTGATATCAAAAATCCAAAACATATATATAATTAAGGG

CN 119205 TCCCGCTGCTCAATTAGGGCGGTTGATATCAAAAATCCAAAACATATATATAATTAAGGG

Yellowstone TCCCGCTGCTCAATTAGGGCGGTTGATATCAAAAATCCAAAACATATATATAATTAAGGG

CN 120017 TCCCGCTGCTCAATTAGGGCGGTTGATATCAAAAATCCAAAACATATATATAATTAAGGG

CN 119300 TCCCGCTGCTCAATTAGGGCGGTTGATATCAAAAATCCAAAACATATATATAATTAAGGG

Jasper TCCCGCTGCTCAATTAGGGCGGTTGATATCAAAAATCCAAAACATATATATAATTAAGGG

CN 120027 TCCCGCTGCTCAATTAGGGCGGTTGATATCAAAAATCCAAAACATATATATAATTAAGGG

CN 113754 TCCCGCTGCTCAATTAGGGCGGTTGATATCAAAAATCCAAAACATATATATAATTAAGGG

Joelle AAFC TCCCGCTGCTCAATTAGGGCGGTTGATATCAAAAATCCAAAACATATATATAATTAAGGG

Joelle NCBI TCCCGCTGCTCAATTAGGGCGGTTGATATCAAAAATCCAAAACATATATATAATTAAGGG

Joelle phyto TCCCGCTGCTCAATTAGGGCGGTTGATATCAAAAATCCAAAACATATATATAATTAAGGG

Blaine Creek TCCCGCTGCTCAATTAGGGCGGTTGATATCAAAAATCCAAAACATATATATAATTAAGGG

CAM 241 TCCCGCTGCTCAATTAGGGCGGTTGATATCAAAAATCCAAAACATATATATAATTAAGGG

CN 119294 TCCCGCTGCTCAATTAGGGCGGTTGATATCAAAAATCCAAAACATATATATAATTAAGGG

********** *************************************************

CN 120025 AAG--AAAAAAGTAATAAGATATGTAATTATTCCGCTGATAAGGGCGCGTCGTATGTATG

CN 120013 AAGAAAAAAAAAAAAAAAGATATGTAATTATTCCGCTGATAAGGGCGCGTCGTATGTATG

CS17CS1133 AAGAAAAAAAAAAAAAAAGATATGTAATTATTCCGCTGATAAGGGCGCGTCGTATGTATG

CN 120030 AAGAAAAAAAAAAAAAAAGATATGTAATTATTCCGCTGATAAGGGCGCGTCGTATGTATG

CO46 NCBI AAGAAAAAAAAAAAAAAAGATATGTAATTATTCCGCTGATAAGGGCGCGTCGTATGTATG

DH55 ref genome AAG-------AAAAAAAAGATATGTAATTATTCCGCTGATAAGGGCGCGTCGTATGTATG

Hoga AAGAAAAAAAAAAAAAAAGATATGTAATTATTCCGCTGATAAGGGCGCGTCGTATGTATG

CAM 236 AAGAAAAAAAAAAAAAAAGATATGTAATTATTCCGCTGATAAGGGCGCGTCGTATGTATG

09-CS0040 AAGAAAAAAAAAAAAAAAGATATGTAATTATTCCGCTGATAAGGGCGCGTCGTATGTATG

CN 119205 AAGAAAAAAAAAAAAAAAGATATGTAATTATTCCGCTGATAAGGGCGCGTCGTATGTATG

Yellowstone AAGAAAAAAAAAAAAAAAGATATGTAATTATTCCGCTGATAAGGGCGCGTCGTATGTATG

CN 120017 AAGAAAAAAAAAAAAAAAGATATGTAATTATTCCGCTGATAAGGGCGCGTCGTATGTATG

CN 119300 AAGAAAAAAAAAAAAAAAGATATGTAATTATTCCGCTGATAAGGGCGCGTCGTATGTATG

Jasper AAGAAAAAAAAAAAAAAAGATATGTAATTATTCCGCTGATAAGGGCGCGTCGTATGTATG

CN 120027 AAGAAAAAAAAAAAAAAAGATATGTAATTATTCCGCTGATAAGGGCGCGTCGTATGTATG

CN 113754 AAGAAAAAAAAAAAAAAAGATATGTAATTATTCCGCTGATAAGGGCGCGTCGTATGTATG

Joelle AAFC AAGAAAAAAAAAAAAAAAGATATGTAATTATTCCGCTGATAAGGGCGCGTCGTATGTATG

Joelle NCBI AAGAAAAAAAAAAAAAAAGATATGTAATTATTCCGCTGATAAGGGCGCGTCGTATGTATG

Joelle phyto AAGAAAAAAAAAAAAAAAGATATGTAATTATTCCGCTGATAAGGGCGCGTCGTATGTATG

Blaine Creek AAGAAAAAAAAAAAAAAAGATATGTAATTATTCCGCTGATAAGGGCGCGTCGTATGTATG

CAM 241 AAGAAAAAAAAAAAAAAAGATATGTAATTATTCCGCTGATAAGGGCGCGTCGTATGTATG

CN 119294 AAGAAAAAAAAAAAAAAAGATATGTAATTATTCCGCTGATAAGGGCGCGTCGTATGTATG

*** * ** ********************************************

CN 120025 TATATCTTAATACTCCCTC----CTC-------CTCTCTCTCTCTTTGGCTTGTGTGTGA

CN 120013 TATATCTTAATACTCCC------CTCTCTCTCTCTCTCTCTCTCTTTGGCTTGTGTGTGA

CS17CS1133 TATATCTTAATACTCCCTC----CTCTCTCTCTCTCTCTCTCTCTTTGGCTTGTGTGTGA

CN 120030 TATATCTTAATACTCCCTC----CTCTCTCTCTCTCTCTCTCTCTTTGGCTTGTGTGTGA

CO46 NCBI TATATCTTAATACTCCCTCCTCTCTCTCTCTCTCTCTCTCTCTCTTTGGCTTGTGTGTGA

DH55 ref genome TATATCTTAATACTCCCTC----CTCTCTCTCTCTCTCTCTCTCTTTGGCTTGTGTGTGA

Hoga TATATCTTAATACTCCCTC----CTCTCTCTCTCTCTCTCTCTCTTTGGCTTGTGTGTGA

CAM 236 TATATCTTAATACTCCCTC----CTCTCTCTCTCTCTCTCTCTCTTTGGCTTGTGTGTGA

09-CS0040 TATATCTTAATACTCCCTC----CTCTCTCTCTCTCTCTCTCTCTTTGGCTTGTGTGTGA

CN 119205 TATATCTTAATACTCCCTC----CTCTCTCTCTCTCTCTCTCTCTTTGGCTTGTGTGTGA

Yellowstone TATATCTTAATACTCC-------CTCTCTCTCTCTCTCTCTCTCTTTGGCTTGTGTGTGA

CN 120017 TATATCTTAATACTCCCTC----CTCTCTCTCTCTCTCTCTCTCTTTGGCTTGTGTGTGA

CN 119300 TATATCTTAATACTCCCTC----CTCTCTCTCTCTCTCTCTCTCTTTGGCTTGTGTGTGA

Jasper TATATCTTAATACTCCCTC----CTCTCTCTCTCTCTCTCTCTCTTTGGCTTGTGTGTGA

CN 120027 TATATCTTAATACTCCCTC----CTCTCTCTCTCTCTCTCTCTCTTTGGCTTGTGTGTGA

CN 113754 TATATCTTAATACTCCCTC----CTCTCTCTCTCTCTCTCTCTCTTTGGCTTGTGTGTGA

Joelle AAFC TATATCTTAATACTCCCTC----CTCTCTCTCTCTCTCTCTCTCTTTGGCTTGTGTGTGA

Joelle NCBI TATATCTTAATACTCCCTC----CTCTCTCTCTCTCTCTCTCTCTTTGGCTTGTGTGTGA

Joelle phyto TATATCTTAATACTCCCTC----CTCTCTCTCTCTCTCTCTCTCTTTGGCTTGTGTGTGA

Blaine Creek TATATCTTAATACTCCCTC----CTCTCTCTCTCTCTCTCTCTCTTTGGCTTGTGTGTGA

CAM 241 TATATCTTAATACTCCCTC----CTCTCTCTCTCTCTCTCTCTCTTTGGCTTGTGTGTGA

CN 119294 TATATCTTAATACTCCCTC----CTCTCTCTCTCTCTCTCTCTCTTTGGCTTGTGTGTGA

**************** *** ***************************

CN 120025 TACTT-AAAAGTAGATTGAAAATCAAATATTATCTGTTTAAGACAAAGC----TATGATG

CN 120013 TACTTAAAAAGTAGATTGAAAGTCAAATATTATCTGTTTAAGACAAAACTATATATGATG

CS17CS1133 TACTTAAAAAGTAGATTGAAAGTCAAATATTATCTGTTTAAGACAAAACTATATATGATG

CN 120030 TACTTAAAAAGTAGATTGAAAGTCAAATATTATCTGTTTAAGACAAAACTATATATGATG

CO46 NCBI TACTTAAAAAGTAGATTGAAAGTCAAATATTATCTGTTTAAGACAAAACTATATATGATG

DH55 ref genome TACTTAAAAAGTAGATTGAAAGTCAAATATTATCTGTTTAAGACAAAACTATATATGATG

Hoga TACTTAAAAAGTAGATTGAAAGTCAAATATTATCTGTTTAAGACAAAACTATATATGATG

CAM 236 TACTTAAAAAGTAGATTGAAAGTCAAATATTATCTGTTTAAGACAAAACTATATATGATG

09-CS0040 TACTTAAAAAGTAGATTGAAAGTCAAATATTATCTGTTTAAGACAAAACTATATATGATG

CN 119205 TACTTAAAAAGTAGATTGAAAGTCAAATATTATCTGTTTAAGACAAAACTATATATGATG

Yellowstone TACTTAAAAAGTAGATTGAAAGTCAAATATTATCTGTTTAAGACAAAACTATATATGATG

CN 120017 TACTTAAAAAGTAGATTGAAAGTCAAATATTATCTGTTTAAGACAAAACTATATATGATG

CN 119300 TACTTAAAAAGTAGATTGAAAGTCAAATATTATCTGTTTAAGACAAAACTATATATGATG

Jasper TACTTAAAAAGTAGATTGAAAGTCAAATATTATCTGTTTAAGACAAAACTATATATGATG

CN 120027 TACTTAAAAAGTAGATTGAAAGTCAAATATTATCTGTTTAAGACAAAACTATATATGATG

CN 113754 TACTTAAAAAGTAGATTGAAAGTCAAATATTATCTGTTTAAGACAAAACTATATATGATG

Joelle AAFC TACTTAAAAAGTAGATTGAAAGTCAAATATTATCTGTTTAAGACAAAACTATATATGATG

Joelle NCBI TACTTAAAAAGTAGATTGAAAGTCAAATATTATCTGTTTAAGACAAAACTATATATGATG

Joelle phyto TACTTAAAAAGTAGATTGAAAGTCAAATATTATCTGTTTAAGACAAAACTATATATGATG

Blaine Creek TACTTAAAAAGTAGATTGAAAGTCAAATATTATCTGTTTAAGACAAAACTATATATGATG

CAM 241 TACTTAAAAAGTAGATTGAAAGTCAAATATTATCTGTTTAAGACAAAACTATATATGATG

CN 119294 TACTTAAAAAGTAGATTGAAAGTCAAATATTATCTGTTTAAGACAAAACTATATATGATG

***** *************** ************************* * *******

CN 120025 AAAC-TTTGTACCTTATTAGAGAGAGCTTTGCATCGAGATGTTATTGTGTGTGTGTTCTC

CN 120013 AAACTTTTGTACCTTATTCGAGAGAGCTTTGCATCGAGATGTTGT--TTTGTGTGTTCTC

CS17CS1133 AAACTTTTGTACCTTATTCGAGAGAGCTTTGCATCGAGATGTTGT--TTTGTGTGTTCTC

CN 120030 AAACTTTTGTACCTTATTCGAGAGAGCTTTGCATCGAGATGTTGT--TTTGTGTGTTCTC

CO46 NCBI AAACTTTTGTACCTTATTTGAGAGAGCTTTGCATCGAGATGTTGT--TTTGTGTGTTCTC

DH55 ref genome AAACTTTTGTACCTTATTCGAGAGAGCTTTGCATCGAGATGTTGT--TTTGTGTGTTCTC

Hoga AAACTTTTGTACCTTATTCGAGAGAGCTTTGCATCGAGATGTTGT--TTTGTGTGTTCTC

CAM 236 AAACTTTTGTACCTTATTCGAGAGAGCTTTGCATCGAGATGTTGT--TTTGTGTGTTCTC

09-CS0040 AAACTTTTGTACCTTATTCGAGAGAGCTTTGCATCGAGATGTTGT--TTTGTGTGTTCTC

CN 119205 AAACTTTTGTACCTTATTCGAGAGAGCTTTGCATCGAGATGTTGT--TTTGTGTGTTCTC

Yellowstone AAACTTTTGTACCTTATTCGAGAGAGCTTTGCATCGAGATGTTGT--TTTGTGTGTTCTC

CN 120017 AAACTTTTGTACCTTATTCGAGAGAGCTTTGCATCGAGATGTTGT--TTTGTGTGTTCTC

CN 119300 AAACTTTTGTACCTTATTCGAGAGAGCTTTGCATCGAGATGTTGT--TTTGTGTGTTCTC

Jasper AAACTTTTGTACCTTATTCGAGAGAGCTTTGCATCGAGATGTTGT--TTTGTGTGTTCTC

CN 120027 AAACTTTTGTACCTTATTCGAGAGAGCTTTGCATCGAGATGTTGT--TTTGTGTGTTCTC

CN 113754 AAACTTTTGTACCTTATTCGAGAGAGCTTTGCATCGAGATGTTGT--TTTGTGTGTTCTC

Joelle AAFC AAACTTTTGTACCTTATTCGAGAGAGCTTTGCATCGAGATGTTGT--TTTGTGTGTTCTC

Joelle NCBI AAACTTTTGTACCTTATTCGAGAGAGCTTTGCATCGAGATGTTGT--TTTGTGTGTTCTC

Joelle phyto AAACTTTTGTACCTTATTCGAGAGAGCTTTGCATCGAGATGTTGT--TTTGTGTGTTCTC

Blaine Creek AAACTTTTGTACCTTATTCGAGAGAGCTTTGCATCGAGATGTTGT--TTTGTGTGTTCTC

CAM 241 AAACTTTTGTACCTTATTCGAGAGAGCTTTGCATCGAGATGTTGT--TTTGTGTGTTCTC

CN 119294 AAACTTTTGTACCTTATTCGAGAGAGCTTTGCATCGAGATGTTGT--TTTGTGTGTTCTC

**** ************* ************************ * * ***********

CN 120025 CTCTTCTGTCGAAAGCTTGTGTTTGCTTCACAGTGAAGAAGTCTTCTGCTTATTTTGCAA

CN 120013 CTCTTCTGTCCAAAGCTTGTGTTTGCTTCACAGTGAAGAAGTCTTCTGCTTATTTTGCAG

CS17CS1133 CTCTTCTGTCCAAAGCTTGTGTTTGCTTCACAGTGAAGAAGTCTTCTGCTTATTTTGCAG

CN 120030 CTCTTCTGTCCAAAGCTTGTGTTTGCTTCACAGTGAAGAAGTCTTCTGCTTATTTTGCAG

CO46 NCBI CTCTTCTGTCCAAAGCTTGTGTTTGCTTCACAGTGAAGAAGTCTTCTGCTTATTTTGCAG

DH55 ref genome CTCTTCTGTCCAAAGCTTGTGTTTGCTTCACAGTGAAGAAGTCTTCTGCTTATTTTGCAG

Hoga CTCTTCTGTCCAAAGCTTGTGTTTGCTTCACAGTGAAGAAGTCTTCTGCTTATTTTGCAG

CAM 236 CTCTTCTGTCCAAAGCTTGTGTTTGCTTCACAGTGAAGAAGTCTTCTGCTTATTTTGCAG

09-CS0040 CTCTTCTGTCCAAAGCTTGTGTTTGCTTCACAGTGAAGAAGTCTTCTGCTTATTTTGCAG

CN 119205 CTCTTCTGTCCAAAGCTTGTGTTTGCTTCACAGTGAAGAAGTCTTCTGCTTATTTTGCAG

Yellowstone CTCTTCTGTCCAAAGCTTGTGTTTGCTTCACAGTGAAGAAGTCTTCTGCTTATTTTGCAG

CN 120017 CTCTTCTGTCCAAAGCTTGTGTTTGCTTCACAGTGAAGAAGTCTTCTGCTTATTTTGCAG

CN 119300 CTCTTCTGTCCAAAGCTTGTGTTTGCTTCACAGTGAAGAAGTCTTCTGCTTATTTTGCAG

Jasper CTCTTCTGTCCAAAGCTTGTGTTTGCTTCACAGTGAAGAAGTCTTCTGCTTATTTTGCAG

CN 120027 CTCTTCTGTCCAAAGCTTGTGTTTGCTTCACAGTGAAGAAGTCTTCTGCTTATTTTGCAG

CN 113754 CTCTTCTGTCCAAAGCTTGTGTTTGCTTCACAGTGAAGAAGTCTTCTGCTTATTTTGCAG

Joelle AAFC CTCTTCTGTCCAAAGCTTGTGTTTGCTTCACAGTGAAGAAGTCTTCTGCTTATTTTGCAG

Joelle NCBI CTCTTCTGTCCAAAGCTTGTGTTTGCTTCACAGTGAAGAAGTCTTCTGCTTATTTTGCAG

Joelle phyto CTCTTCTGTCCAAAGCTTGTGTTTGCTTCACAGTGAAGAAGTCTTCTGCTTATTTTGCAG

Blaine Creek CTCTTCTGTCCAAAGCTTGTGTTTGCTTCACAGTGAAGAAGTCTTCTGCTTATTTTGCAG

CAM 241 CTCTTCTGTCCAAAGCTTGTGTTTGCTTCACAGTGAAGAAGTCTTCTGCTTATTTTGCAG

CN 119294 CTCTTCTGTCCAAAGCTTGTGTTTGCTTCACAGTGAAGAAGTCTTCTGCTTATTTTGCAG

********** ************************************************

CN 120025 TAGAGGCGTGGCACAACTCTGCTCTCTTTGTCCTCGTAATTTAATTTGTTTTTATCTAAA

CN 120013 TAGAGGCGTGGCACAACTCTGCTCTATATGTCCTCGTAATTTAATTTGTTTTTATCTAAA

CS17CS1133 TAGAGGCGTGGCACAACTCTGCTCTATATGTCCTCGTAATTTAATTTGTTTTTATCTAAA

CN 120030 TAGAGGCGTGGCACAACTCTGCTCTATATGTCCTCGTAATTTAATTTGTTTTTATCTAAA

CO46 NCBI TAGAGGCGTGGCACAACTCTGCTCTATATGTCCTCGTAATTTAATTTGTTTTTATCTAAA

DH55 ref genome TAGAGGCGTGGCACAACTCTGCTCTATATGTCCTCGTAATTTAATTTGTTTTTATCTAAA

Hoga TAGAGGCGTGGCACAACTCTGCTCTATATGTCCTCGTAATTTAATTTGTTTTTATCTAAA

CAM 236 TAGAGGCGTGGCACAACTCTGCTCTATATGTCCTCGTAATTTAATTTGTTTTTATCTAAA

09-CS0040 TAGAGGCGTGGCACAACTCTGCTCTATATGTCCTCGTAATTTAATTTGTTTTTATCTAAA

CN 119205 TAGAGGCGTGGCACAACTCTGCTCTATATGTCCTCGTAATTTAATTTGTTTTTATCTAAA

Yellowstone TAGAGGCGTGGCACAACTCTGCTCTATATGTCCTCGTAATTTAATTTGTTTTTATCTAAA

CN 120017 TAGAGGCGTGGCACAACTCTGCTCTATATGTCCTCGTAATTTAATTTGTTTTTATCTAAA

CN 119300 TAGAGGCGTGGCACAACTCTGCTCTATATGTCCTCGTAATTTAATTTGTTTTTATCTAAA

Jasper TAGAGGCGTGGCACAACTCTGCTCTATATGTCCTCGTAATTTAATTTGTTTTTATCTAAA

CN 120027 TAGAGGCGTGGCACAACTCTGCTCTATATGTCCTCGTAATTTAATTTGTTTTTATCTAAA

CN 113754 TAGAGGCGTGGCACAACTCTGCTCTATATGTCCTCGTAATTTAATTTGTTTTTATCTAAA

Joelle AAFC TAGAGGCGTGGCACAACTCTGCTCTATATGTCCTCGTAATTTAATTTGTTTTTATCTAAA

Joelle NCBI TAGAGGCGTGGCACAACTCTGCTCTATATGTCCTCGTAATTTAATTTGTTTTTATCTAAA

Joelle phyto TAGAGGCGTGGCACAACTCTGCTCTATATGTCCTCGTAATTTAATTTGTTTTTATCTAAA

Blaine Creek TAGAGGCGTGGCACAACTCTGCTCTATATGTCCTCGTAATTTAATTTGTTTTTATCTAAA

CAM 241 TAGAGGCGTGGCACAACTCTGCTCTATATGTCCTCGTAATTTAATTTGTTTTTATCTAAA

CN 119294 TAGAGGCGTGGCACAACTCTGCTCTATATGTCCTCGTAATTTAATTTGTTTTTATCTAAA

************************* * ********************************

CN 120025 ACGCGTTGTGGTTGTCATGGGTTTCTATTGGGCCCATGTCGGTCA--TTTTTTGGCCCAA

CN 120013 ACGCGTTGCGGTTGTCATGGGTTTCTATTGGGCCCATGTCGGTCATTTTTTTTGGCCCAA

CS17CS1133 ACGCGTTGCGGTTGTCATGGGTTTCTATTGGGCCCATGTCGGTCATTTTTTTTGGCCCAA

CN 120030 ACGCGTTGCGGTTGTCATGGGTTTCTATTGGGCCCATGTCGGTCATTTTTTTTGGCCCAA

CO46 NCBI ACGCGTTGCGGTTGTCATGGGTTTCTATTGGGCCCATGTCGGTCATTTTTTTTGGCCCAA

DH55 ref genome ACGCGTTGCGGTTGTCATGGGTTTCTATTGGGCCCATGTCGGTCATTTTTTTTGGCCCAA

Hoga ACGCGTTGCGGTTGTCATGGGTTTCTATTGGGCCCATGTCGGTCATTTTTTTTGGCCCAA

CAM 236 ACGCGTTGCGGTTGTCATGGGTTTCTATTGGGCCCATGTCGGTCATTTTTTTTGGCCCAA

09-CS0040 ACGCGTTGCGGTTGTCATGGGTTTCTATTGGGCCCATGTCGGTCATTTTTTTTGGCCCAA

CN 119205 ACGCGTTGCGGTTGTCATGGGTTTCTATTGGGCCCATGTCGGTCATTTTTTTTGGCCCAA

Yellowstone ACGCGTTGCGGTTGTCATGGGTTTCTATTGGGCCCATGTCGGTCATTTTTTTTGGCCCAA

CN 120017 ACGCGTTGCGGTTGTCATGGGTTTCTATTGGGCCCATGTCGGTCATTTTTTTTGGCCCAA

CN 119300 ACGCGTTGCGGTTGTCATGGGTTTCTATTGGGCCCATGTCGGTCATTTTTTTTGGCCCAA

Jasper ACGCGTTGCGGTTGTCATGGGTTTCTATTGGGCCCATGTCGGTCATTTTTTTTGGCCCAA

CN 120027 ACGCGTTGCGGTTGTCATGGGTTTCTATTGGGCCCATGTCGGTCATTTTTTTTGGCCCAA

CN 113754 ACGCGTTGCGGTTGTCATGGGTTTCTATTGGGCCCATGTCGGTCATTTTTTTTGGCCCAA

Joelle AAFC ACGCGTTGCGGTTGTCATGGGTTTCTATTGGGCCCATGTCGGTCATTTTTTTTGGCCCAA

Joelle NCBI ACGCGTTGCGGTTGTCATGGGTTTCTATTGGGCCCATGTCGGTCATTTTTTTTGGCCCAA

Joelle phyto ACGCGTTGCGGTTGTCATGGGTTTCTATTGGGCCCATGTCGGTCATTTTTTTTGGCCCAA

Blaine Creek ACGCGTTGCGGTTGTCATGGGTTTCTATTGGGCCCATGTCGGTCATTTTTTTTGGCCCAA

CAM 241 ACGCGTTGCGGTTGTCATGGGTTTCTATTGGGCCCATGTCGGTCATTTTTTTTGGCCCAA

CN 119294 ACGCGTTGCGGTTGTCATGGGTTTCTATTGGGCCCATGTCGGTCATTTTTTTTGGCCCAA

******** ************************************ *************

CN 120025 TAGGCTTAGTTCCTGAGATTCATTGCTCTTAAGTGTTGTTAACGTGACCGACCTCTTTCC

CN 120013 TAGGCGTAGTTCTTGAGATTCATTGCTGTGAAGTGTTGTTAACGTGACCGACCTCTT---

CS17CS1133 TAGGCGTAGTTCTTGAGATTCATTGCTGTGAAGTGTTGTTAACGTGACCGACCTCTT---

CN 120030 TAGGCGTAGTTCTTGAGATTCATTGCTGTGAAGTGTTGTTAACGTGACCGACCTCTT---

CO46 NCBI TAGGCGTAGTTCTTGAGATTCATTGCTGTGAAGTGTTGTTAACGTGACCGACCTCTT---

DH55 ref genome TAGGCGTAGTTCTTGAGATTCATTGCTGTGAAGTGTTGTTAACGTGACCGACCTCTT---

Hoga TAGGCGTAGTTCTTGAGATTCATTGCTGTGAAGTGTTGTTAACGTGACCGACCTCTT---

CAM 236 TAGGCGTAGTTCTTGAGATTCATTGCTGTGAAGTGTTGTTAACGTGACCGACCTCTT---

09-CS0040 TAGGCGTAGTTCTTGAGATTCATTGCTGTGAAGTGTTGTTAACGTGACCGACCTCTT---

CN 119205 TAGGCGTAGTTCTTGAGATTCATTGCTGTGAAGTGTTGTTAACGTGACCGACCTCTT---

Yellowstone TAGGCGTAGTTCTTGAGATTCATTGCTGTGAAGTGTTGTTAACGTGACCGACCTCTT---

CN 120017 TAGGCGTAGTTCTTGAGATTCATTGCTGTGAAGTGTTGTTAACGTGACCGACCTCTT---

CN 119300 TAGGCGTAGTTCTTGAGATTCATTGCTGTGAAGTGTTGTTAACGTGACCGACCTCTT---

Jasper TAGGCGTAGTTCTTGAGATTCATTGCTGTGAAGTGTTGTTAACGTGACCGACCTCTT---

CN 120027 TAGGCGTAGTTCTTGAGATTCATTGCTGTGAAGTGTTGTTAACGTGACCGACCTCTT---

CN 113754 TAGGCGTAGTTCTTGAGATTCATTGCTGTGAAGTGTTGTTAACGTGACCGACCTCTT---

Joelle AAFC TAGGCGTAGTTCTTGAGATTCATTGCTGTGAAGTGTTGTTAACGTGACCGACCTCTT---

Joelle NCBI TAGGCGTAGTTCTTGAGATTCATTGCTGTGAAGTGTTGTTAACGTGACCGACCTCTT---

Joelle phyto TAGGCGTAGTTCTTGAGATTCATTGCTGTGAAGTGTTGTTAACGTGACCGACCTCTT---

Blaine Creek TAGGCGTAGTTCTTGAGATTCATTGCTGTGAAGTGTTGTTAACGTGACCGACCTCTT---

CAM 241 TAGGCGTAGTTCTTGAGATTCATTGCTGTGAAGTGTTGTTAACGTGACCGACCTCTT---

CN 119294 TAGGCGTAGTTCTTGAGATTCATTGCTGTGAAGTGTTGTTAACGTGACCGACCTCTT---

***** ****** ************** * ***************************

CN 120025 AAAATTATGCTTGATATCCAAAATTATGCTTGATATCTAAAGCGGCTTAACTAGCCGTAT

CN 120013 ----------------TCCAAAATTATGCTTGATATCTAAGCCGGCTTAACAAGCCGTAT

CS17CS1133 ----------------TCCAAAATTATGCTTGATATCTAAGCCGGCTTAACAAGCCGTAT

CN 120030 ----------------TCCAAAATTATGCTTGATATCTAAGCCGGCTTAACAAGCCGTAT

CO46 NCBI ----------------TCCAAAATTATGCTTGATATCTAAGCCGGCTTAACAAGCCGTAT

DH55 ref genome ----------------TCCAAAATTATGCTTGATATCTAAGCCGGCTTAACAAGCCGTAT

Hoga ----------------TCCAAAATTATGCTTGATATCTAAGCCGGCTTAACAAGCCGTAT

CAM 236 ----------------TCCAAAATTATGCTTGATATCTAAGCCGGCTTAACAAGCCGTAT

09-CS0040 ----------------TCCAAAATTATGCTTGATATCTAAGCCGGCTTAACAAGCCGTAT

CN 119205 ----------------TCCAAAATTATGCTTGATATCTAAGCCGGCTTAACAAGCCGTAT

Yellowstone ----------------TCCAAAATTATGCTTGATATCTAAGCCGGCTTAACAAGCCGTAT

CN 120017 ----------------TCCAAAATTATGCTTGATATCTAAGCCGGCTTAACAAGCCGTAT

CN 119300 ----------------TCCAAAATTATGCTTGATATCTAAGCCGGCTTAACAAGCCGTAT

Jasper ----------------TCCAAAATTATGCTTGATATCTAAGCCGGCTTAACAAGCCGTAT

CN 120027 ----------------TCCAAAATTATGCTTGATATCTAAGCCGGCTTAACAAGCCGTAT

CN 113754 ----------------TCCAAAATTATGCTTGATATCTAAGCCGGCTTAACAAGCCGTAT

Joelle AAFC ----------------TCCAAAATTATGCTTGATATCTAAGCCGGCTTAACAAGCCGTAT

Joelle NCBI ----------------TCCAAAATTATGCTTGATATCTAAGCCGGCTTAACAAGCCGTAT

Joelle phyto ----------------TCCAAAATTATGCTTGATATCTAAGCCGGCTTAACAAGCCGTAT

Blaine Creek ----------------TCCAAAATTATGCTTGATATCTAAGCCGGCTTAACAAGCCGTAT

CAM 241 ----------------TCCAAAATTATGCTTGATATCTAAGCCGGCTTAACAAGCCGTAT

CN 119294 ----------------TCCAAAATTATGCTTGATATCTAAGCCGGCTTAACAAGCCGTAT

************************ ********* ********

Portion of *Camelina sativa* cultivar ‘CO46’ chromosome 20

GenBank: GCA_036971115.1

GAATTAAAAATTGATGAAGAGGTAATTTTTTTAATGAAAAATTAAAGAGTCATTAGAGATAGCCAATGGAAGAAGAGCAAACGCGGTCGCATGATACTCGTCATGCGGTACACGTGGCAATCTTGCGTTCAAAACGCATCGTTTTTGTTCATAAATTTTATTTTCATCACTCTCGTTTACCCTAAAAAAAAAAAAAAAAAAAATCTAGCCCAAGGAAGGAAAAAAAAAAAAGTAGATTAGACAAAAAAATAGAAAGAAATAAAAGGAAAAAAGGAAATAAAAAAATAGGAAAGGCAAAAAAAGAAGAGAAACGCTTAGTATCTCTCCGGCGACTTGAACCCAACCCAAACCTGAGGATCAAATTAGGGCACAAGGGCCTCTCGGAGACTGAAGCCATGGGAAGGAAAAAACTAGAAATCAAGCGAATTGAGAACAAAAGTAGCCGACAAGTCACCTTCTCCAAACGTCGTAATGGTCTCATCGAGAAAGCTCGTCAGCTTTCTGTTCTCTGTGACGCATCCGTCGCTCTTCTCGTCGTCTCCGCCTCCGGCAAGCTCTACAGCTTCTCCTCCGGTGATAAGTACGTCTTTTCCTTATCTGGGTTCTCGTTTTTTTTTCCCCTTTAAGCTTCGGTTTTGTGCTTTCTCTTTACTTTGTTTTTGAAGAAAAATAAATATTTTAAAAAGACACAAACAAAATAAAAAATAAAAAACAATTAATGTATAGTTTGATTTTTCCGGCGAATCTCTTGTTGTTTTACTCGGTTCGGTCTTTGTTAGTGTTTTTTCTATGACCATGTGAGATACATGAGATAACTAAATCTATGGAAGAACAATGTCGTGTTGAGCTTAAGCTTCTTACTTTTTTCTCTTCTTTTCTCTCTCTATCTCTCTCTCTTTTTCCTTAAATTTTTTTCTGCATGGATTTTATTTTATTTGGAAATTTTTTGCATGTCATTTGAGATTTGCTTGACACGTTCTGCTGCGTACTCGATGTTGTCTAGTGAAGTTTCAAGCCGTCTTTGATTGCTACTTGGCTTTAGGGATTAATCACTACACGAAAACACGGCGTTTGGGACTACTGGAATCGTCTCAAATCAGTCGCTAAGTCGGTATGTGCAACTATTGAAGGACTGTTATATTTGGTCGCAAATATTTGGTCGCAAATTGTTTTGGTCGCAAACCAGTCCCTTATTTGCGACGCATTGGAGACAAGGGTTATGGTCGTTAATTAGCGACTACGGACAGACCGAAACGTACCGTCGTAATGAACGACTCTATAGTGACTCACTGGCGCAGTCGTAATTGGCGACTACGGACAGACCGGAATATACCGTCGTAATTAACGACAGTATAGTGACTCGCTTTGGCAGTCGTAATAGACGACTTCTTTCAGACGACATGGTAGTCACAAATTAGCTACGTTTGGAAGACTGACTTAATACCACAGTAAACTTTAAAAATCTGAATTTTGGTCCTAAAATAGTCGCTGTTTAGTCACAAGATAGTCGCAAAGGAGTCAGAATTGTTTGTGACGGACTGAAGACTAAGTTTTTTTAGTCCCAAAATCTTGACATATACACAACAAATTTGGTACTTTGTTTGTGTTTATTTAAATTATAAATGGTCGCAAATCAGTGAAAATATACCTAGTAATTTGGGACAGTATACAATAAATTTTGAAAAAAAAAACCTGAATTTAAATTAAAATCCTGATTTGAATGATCTTAACAAAAAACATACAAATCATTCAACGATCATATACAGAAAATAGTTTTAGGCTAAGGATCTAGAAACACTACTACACAATCCAAAAAATAGTTATAGAGGCTTAAAAGAGATAGAATTAGTTTGTTAAGCATGAGAGGATGATACTGACAAAGGTGAAGTGGTTGGTGCAGTCGCAGGTGAAGTAGATGGTGAACTAGCTCCTGAAGTGGTTGTTGCAGGTTGGTTGACATTGGTCGCAGTGGCTGGAGTGATGTTGGTTGCTGGTGCTGCTGTGGTTAATGGCGCATTGGCTGGTGGTTGAGTAGACAAGAATTCTGCAACCCCCGGATCACTAGTCTTCAAGTAGGATACGAGCATCTCCAAGGTTGCAATGCGGTTTTGAGCGTCACGTTGCTCCTCATCACGTCTAGCATCTTCTCTGGCACGTCGAGCATTTTCTTCATCTTGATCAGCGAGCTTACGACGAGCAACCTGGAGTTGTTCTTGAATCTCTAACAGCGAATCTGGACTAGAGCTTGGATAAGTAGCCTTTCTTTTCCCTTTGGTGATTATCTCCGAGAGACTTCCAAGTCCAAAGGGGGTTCCCTTGGCATTCGTTACAGTACACTAAGACAAAAGAAGAGATGATCATTCACAAAACAAACATAACAAACAGAAATATCACACAAACCCAGATAGAAATATCACACAAACCCAGATAATCAACATCGAGTATTCTCAAGCATCAATCAAAAGTATATCAATATCAAGGATTCTTAAGCATCAATCAAAATCAGATACTTAGCTTAGCAAAATAAGACAATTATGATAGGCACAACAAAGACAATTGTACTGGAAATTACCTTTAAGAAAATGTCATCTTTTTCTTGAATGGGGAGGAGGGTTGCATGAGGAGAATCTGGCGAGGAGTGGTCTGTAACATCAGACTCCATTTGAGACATTACCTCCTCTATGTTCTTCTCATATGTCTCCACAACTTGTTTGGCCTTCTGAATAAAAACTTCCATCTGCCTTTGTATGTGTTGCGACAAACACCTCTCCAAGCGAGACAGGACGTCCCAACTTTTCTTCCTACAATGGATAGAAAAATGTTAATTGTGGATACTGAAATATTTAAATGAAATAATTAGAGTTAGTTTAGAGCATTTACCAACTCTTGTTGAATTCGCAAGTAAGATTTCTGGCTGGATAGATGTTTGTGGATTCCAAGACCGTCACGATCAGAATTACGGGCTTGGGATGCATTAGAGCTCTTCTCCTTAGCTTCAGGTGTGTCCCAATGCTCCCACATTTGTTTCCAAAGTGTAGGATTGATCCAAATCGGTGGCTCATCATACGTCTTTGCTTGACTTACAATTCCTTTCATTCTGTTTTTCGCTATCTTCACAAATCCTTCTCTCACAAGCTCAGTAATCGCAACATCCCAATTGTATTTCCTCTACACAGTACAACAATGTAAAGTAAGTGTCAAGTAAGTAAACAAAAGACAAACTCATTGACTTAGTTTAGTAAAAAAAGAAACTGTGTCTAAAAAGAAACTCATTACCGTAAAGGATCTAAAATATCTCTCCCGTATGTGTAATGGCGTAACCTTCCAGCTGAAGTATGGACCATCGAACTTTCTCCTTAAAATCCCAGAAATAACACGAGATAGCTTTCCTTTGTCTCGTCCAAACCTGTTACATAAATATGAGAGTGTTAAGTTATAAAATATAAGCCATCAAACATAAGCGAATGGATCACAACACAATAATCTAATGGAAATTGAACTAAACTTCACAACACAATACAACACAACACAACAAACTTTGAAAACAATTTAGAAACTTTGAAAACAACTAAGAAAACGAACCAAACTTTGAAAACAATAAGAAACTGAACCAAACAATAATCTACTTATTTTATCTAAACGATAACACAACACACAAAGCGTTATTCAAATGGGAGCAATGCGATAATCAAATTATAAAATAAAAGAAAAAAATATTTTACCAAATGATAAAATGATAATCAAATGATAAATATTTTACTTATTTATCAAAATAATAATCAAATGATAAAATAAAAGAAAAAAATATTTTACCATATGCTTTGAGTGTCGGGGATGGGATGCGGACTCAATATCGGGAGATGGTGTCGACCAGGAAGTGCAAGCAACTCATCCAGTTGCTGTTGGAAGTCTTGATTCTGAGCCGGTGGTGGATGCTCTTGACCGTCCTGATTCGGAGGCGGCGGCGGTTGGAATCCTTGATTCGGAGGAGCCAGCGGTTGATACACCGGATTCTGAGGATTCGGCGGCAGAAACCCCAGATCCTGAAGCGGCGGTGGATAAGCCGGATGGTGAAGCGGTGGTTGATATCCACCATTGTGAAGCGGCTGGTTGTATCCCGGATTCTGAGGAGCCACCTGTTGATACACCGGAACATGAGCATGCGGTGGTGGTGGCTGCTGCATCTGGGAGTGGAGCTGTTGTTGAAAGCCTGGTTGTTGTGGCTGAGGAGAAGCTTCTCGAGGATACTGGGGAAATGATGCTTGCGTATACCGAGATGGAAGCGACGGTTGAGATTGTTGGGAGTGTGAAGAATTTTCGTTGTGAGAGGAAGGGTTAGAGGAGTGAGACGACGAAGCGACATCTCTACTTCGTCTCGCAACGCCGCCAGATCCATGACCACCACCGCCAGATCTACGACCACCACCGCCAGATCCACGACCACCACCGCCGGATTTCATCGGAACAGAAGTCGGAGAAGAAGTCGGAGAAGAAGTCGGAATTTCAGTAGATTAGAAAACGAAAAGACAGAGAGAAAAGAAAAAAGAGGGTTTCGGGAAATTATGAGGGTTTCGATATTTACTAAGAGTGTTACTAAGAGTGTTCAATTTGATGGGGGGGGGAATAATTAAACTCGGGGGAATAATTACCGCTAATAATTTTCACTAAAATTTGGGGAAATTTTAATCCGTAAGAAAGTAGTGGTACTTTAAGACTACTTTGTGACTGTCGTAAACATTAAACAAAAACTATACTTAGTGGCAGAACAGTCGCAAATTATAAACCCAGACACCTACACGTCTCAATTTGTTGACATGCACAATCTAAATAGTGACAAAACAGTCGCAAAATATAAGTTCAGTCACAATATAGTCTCAATTTGTGACGTCTTTGTGACAATAAAATATTAGTATAATTTTGTGTTAAGATTTTATAACCAAAAACATATTAGACATGACCCTTTGATCATGTATACTCTCATTTAAGAGTTTAGTTTTGATAAAATATCAAACATAATAATTTTTATATGTTTTTGTAAATATATACAACCTTCACTACTTTAACTTAATTAAACACATGTAATTCTAATACATGTGTTGGAGGATATAAACATAATTGTCTAATTTGGTGTTTTATTTATGTGATTATAGAAATTTAGTAAATATTAGATGTCACATATAAGTAGAAATGTATAAGTTGATATTTTTTGTACTCAAATTTGTAAAATTCTAAAATTAATGTAAGATACTCGCTAAACCGTTGTACTTTAAGACTCTATTGCGACTGACAGAAACAGATGCACAATCACTTTAGTGACAAAACAGTCGCAAAATATAAGTTCAGTCACAATATAGTCTCAATTTGTGACGTCTTTGTGACAATAAAATATTAGTATAATTTTGTGTTAAGATTTTATAACCAAAAACATATTAGACATGACCCTTTGATCATGTATACTCTCATTTAAGAGTTTAGTTTTGATAAAATATCAAACATAATAATTTTTATATGTTTTTGTAAATATATACAACCTTCACTACTCTAACTTAATTAAACACATGTAACCCTAATACATGTGTTGGAGGATATAAACATAATTGTCTAATTTGGTGTTTTATTTATGTGATTATAGAAATTTAGTAAATATTAGATGTCACATATAAGTAGAAATGTATAAGTTGACATTTTTTGTACTCAAATTTGTAAAATTCTAAAATTAATGTAAGATACTCGCTAAACCGTTGTACTTTAAGACTCTATTGCGACTGACAGAAACAGATGCACAATCACTTTAGTGACAAAACAGTCGCAAAATATAAGTTCAGTCACAATATAGTCTCAATTTGTGACGTCTTTGTGACAATAAAATATTAGTCTAATTTTGTGTTAAGATTTTATAACCAAAAACATATTAGACATGACCCTTTGATCATGTATACTCTCATTTAAGAGTTTAGTTTTGATAAAATATCAAACATAATAATTTTTATATGTTTTTGTAAATATATACAACCTTCACTACTCTAACTTAATTAAACACATGTAACCCTAATACATGTGTTGGAGGATATAAACATAATTGTCTAATTTGGTGTTTTATTTATGTGATTATAGAAATTTAGTAAATATTAGATGTCACATATAAGTAGAAATGTATAAGTTGACATTTTTTGTACTCAAATTTGTAAAATTCTAAAATTAATGTAAGATACTCGCTAAACCGTTGTACTTTAAGACTCTATTGCGACTGACAGAAACAGATGCACAATCACTTTAGTGACAAAACAGTCGCAAAATATAAGTTCAGTCACAATATAGTCTCAATTTGTGACGTCTTTGTGACAATAAAATATTAGTCTAATTTTGTGTTAAGATTTTTTAACCAAAAAAATATTAGACAAAACATAACAGTCGCACTATAGTCTCAATTTATGACGTTTTTCTGACTATAAAATATTGATAAAATTAGAGAGAAAACATCATCGAAATTTCAATAAAATTATAATAACACTAGTGTTTGCGATTTCAATAAAATTACAAATACACAAATAATAAACATGAAAATTATTCATATTCCTCATTTTCATCTTCTTGATCTTCATCCTCATCTGAAGACGAAGACGACGACGAGTAGTTGCCACGAAATTCGTCTTCTTGTTCAGCATCGTTGATCTCAAAATCAAGTTCTTCATTAGGCTGATTACCATCTACGAAACTGTCAACTATGATCTCCTCTATTGAATCTGTCATTCCATCATCAACTGTTTGCTGCAATATAACAACCTCCTTTTGTTTTAAGTTCCCTTGTACAGCATGCCTTGGATTAACTTTCAGAACGTTGAGCCAAAGTTTTCGAGGTCGTTTAGTGTGAGGATATGAAAGATAACAAACTTGATCAGCTTGAGAACCTACACATAAAAAATGGTTCTCAATTAGATGTATATATATATCTATAAATATATATTTAAAATAGAACATTACCTAATATAAACGGTTCATAATGTCGGTATCTCCTTGATGACAACACGTCAACAATGCCTCCATCGCGTATTCGAGTGCCTTTGTCAATTGTAGAGTCATACCATTCACAATAGAAAAGTGTGACCCGTAAATTCAAAAATCCTGGATAGGTAAGCTCTATGATATTTTCAATGGTCCCGTAAAAATCGTCCTCGCCAGATGAATTGGAATAAGTTTGTCCTCGGACACATACCTCATAATTCATTGTTTTTTTAGTGAGACCGTGAGCTTTTGTGTGAAACAGATAACCTCTTGTAAAATATATAGGCCAAGACTTAACTTTATTTTCAGGACCGTCCACGATTTCATGGACCCATGGAGGAAAACTTTCAAAACCACCACGCACCTGTGCCAAATATATATACAAAATTTTAGATCACAATATTTATCATGTTGATATAATTAATTGGTGGATAGTAAAACATACATATTGCTTAATCCATTCATGGAAATCTTCTTCTCTTTTCGACCTAACATCTTCATCAGATAGTTTTGGATATTCATATCGAATTTGCGCCTCCAACAATCTGTATTTTATCAACAAAAAGTAAATATTTTAAAGTTTACCTAAGAACATATGGTTATAAATATACTTGGTAAATCAAAAATACCTCTCATGTTGCTGGAAATAATCACAATTGCGCAGAACATATGCGTGTGCACATTTATAATCTTTTTCAGTTAACCAAAATTCACTACTTTTTCCGCCTGGACGACCAACTTGAGTGAATATATCTGGTACTTGTATAGGATATACAGGTACTTCACCTTCTTCATGCCGAATTAACCTGTTAAAGTTTATTTTGTAAAACCTTTTCACAATTTTAGAAGTTGATAGAAAAATTCAATAATCATATTTGAAGTTTTACCTTCTTCTCCTTGTTGTAGAAATGTGTGAAGCGAAATAGTGTTCTGAGAAATAAGATATCTCATCATTCAAGTAGGATTGAACTATTGAGCCAACTGGATATCTTTTGTTCTTCGCTTTCCCTTTTAAACGTTAAAAAAACCTTTCGAAAGGATACATCCACCTATATTGCACAGGACCACCAAGTTGTGCTTCATAAGGTAGGTGAATAGGGAGGTGCTCCATTACATCAAAGAATGAAGGAGGGAAGATTTTCTCCAAATTACACAAGATCATGACAATATTATCCTTCAGAATTTCGAGGCGGGATTGCTCCAATGTTCTACTACATAGGTCGCGGAAAAATGCTCCAATCCCTACAAAGTTATAACATGTTATGTTGTTACATATAAATATAATATCAACAACTAAACCAAATTAAATGTTTAATTTATATACCTGAAATCGCCAGGTGGACATTACGCGGTAAAAGCTCTGCAAATATGAACGGAAGCAATCGTTCCATAAACACATGGCAGTCATGACTCTTCATTCCCGAAAACTTTCCATTTTTCATGTCAGCACAGCTAGCTAAATCTGCGACATATCCGTCTGGGAATCTAACATCTTCTTTTACCCATTTTAATAAAGCCTCTTTCGCTTTGGTTTTCAATCTCCAAATAGGGAATGGGGCTCTCCCTTGAGCATCTAGATGTAAATCTTGCCGGGAACAAAATCTTGCAATATCCAATCTTGACTGCACGTTGTCTTTCGATCTATCTTTTACATTCATAGTGGTGTACATCAAGTTATCAAAGACATTTTTCTCGACGTGCATCAAATCCAGGTTGTGCCTTAAATTCAGATCGACCCAGTACGGTAACTGCCATAATATGCTTTCTTTATGCCAATTATGCCATCTTCCGTAGCCTTTAACTTTCTTTTCGTGACCATTCCCACCGCAAATAGATGTTTTAGGTGCTTTGGCTTTCCTTATACGCTCATAGATAGCTTGACTACTCAGAGATGCTGGTGGCTCGTCATTCAATGAATCTTTTCCCTTCAGAAAGTCCTTTTTATTCTTCCGCATCGGATGACTTGTAGGAAGAAATCTCCTATGACAGTCAAACCAACATGTTTTCCTCCCAGCTGGTAATTGAAAAGCACCAGTGTCATCCATACAAATTGGACATGCTAATCTTCCATGTGTCGTCCATCCCGACAACATACCGTATGCTGGAAAATCGCTTATTGTCCACATAAGAACAACTTTCATGTTGAAATTTTGATTTAGTGAGATATTATAAGCCTCAACCCCATTATACCATAACTCCTTTAACTCATCGATTAATGGTTGGAGGAAAACATCAAGGCTGGCTCGTGGGTGGTTTGGTCCGGAGTTCAGAATCGTAAGAAATAAATACTCGCTGTTCATGCACATATCCGGAGGTAGGTTGTATGGAGTTAAGATCACAGGCCACAAAGTATGATTTTTGGAGACCCCAAATGGATTACAACCATCTGTACATAACCCAAGGTAAACGTTTCGTGGCTTTTGCGCAAATGTGGGATGTAAATGTTGGAAATTCTTCCATTCAGGCGCATCAGATGGATGACACATTACTCCATCTTCTGAATCATGCTCAGCATGCCATCTCATTGATGATGCGGTCTTCTCTGATTGGTACATCCTCTTCAATCTATCTGCAATAGGTAGATACCACATACGACTGAAGGGTATTCTAGTTCTACCCCCACTACTTTTGTACCTTGGCTTCCCACAGAACTTGCAATGCTCCAAATTCCCATCTTCTTTCCAAAATATCATACAATTCTCTATACATACATCAATTGTATGGTATGGCAATCCTATCTTTCGAACTAAATTCTCTGTCTCGTAGTATGAACCAGTAGCTTGATTACCCTCTGGTAAAACCTCCGTAAACAATTCAGCCCAATCGTCCATGCATGCCTCAGACAAATTATTATCAACCTTGATGTTTATGAACCTAGACGCCAATGATAGTTGCGAATGACCTTCATGACATCCATCATAAAGTGGACTATTTGCAGCATCTAACATGTCATAGAACCGTTTCGCCTCTTCAATGGGTTCTTCATGGATATGATCATATCCATGATCATATCCACTTTCATACTCATGATACTCATTAAAAGGCGTGGTACCATGAAATGCATCATTCACCATCTCAGCATATACATTTCCCTCAAATCCTATTGGCTGACTACCAGGTTGACTACCATGCTGACTACTAGTACTAGTATAATTTGTATTAGGATAATGACTACTAGTTCCTACCCCTAGCACGTCATAATCTTCACCATGTTCAGACCATACATAATAATTTGGCATAAATCCTTTACTATGCAAATGATTAGATATTGTCGTACCATGCAAAATAACATCGTTCTGACATTTTACGCAAGGACAGTAAAACCTACCTCCACTGTTTTGTGCCATAGGCTGGCTGTTTGCGAAATTCATGAACTGCTCAACTCCCGCCAAAAATTCTTTAGAGATATTATTTGTTCTTCCATCGATCCGATTATACATCCACTCTCGAGATGAATTCCACATAGCTTTTTTTTTTCTTTTCTTCTAAAAAAACAAATCTTCTTTCTCTCTTTTTTTTTCTTCTCTTCTAAAAAAAAAAAAAAAATCTTTTTTTCACTACTTTTTTTTTATTCCGTCTCTCTACTTAGAAATGAATGAGAGTGATATGAATGGAATGGTGAGAGTGATGCGTTATATATAGTATAAATTTTGCGACTACTTTGCGACTCCAGTAAAAACAGTCGCAAATAAGGACTCCTTTGTAACTAAAAACTACATAATTGAGACAAATTAAAGCTCGAAACAGCTCAATACAAACGCAAACTGAAACATTTATCACGTTTTTTGCCGACATCAATAAGTGACTGCTTTGCGACTCTTTTGCAACAACGAACGGATGTGTCGCAAATAACGACGCATTTGTCACTGTTTTACCACGTAATAGAGACTCAAAAAAGGGTCACAAAAGTTCAGTACAAATCCACACTACCACATTTACCACGTTTTTTACCGACATAGTCACCGACTGATTTGTGACAATAAAATTAAAAGCAAAACAAAAGTGGCAAATACGTCGTGATTTAAAGACAGCTTTGCGACTAACTTTACTGACTTAATCTTCAGTTTATAGTTTGTCATAAATTGCGATTATTTTGCGACGCTCTATTTTTTCTCGATAAAATGTCCCAAAGTAGTCGCATAATAGCAACGAGTTAGCGACTACAGTTAATCGCCGTAAATTTGCGACTGTTTTACGTCCATTGTATTCGTCATCGTTAGTCCGTGGCAAAAGTGTCACAAAGTAGTCACAAAAGTTTGCGACTATAAATTCAGTCACAAAAAGTAGTCCCAAACGCCGTGTTTTCGTGTAGTGAATTCCCTATGTTTCTGATTAGTTTTTATGTCAGAATTGCTGGATCTCTCTTTTTTCTAAGTGATTAAAATTCATTAGATCTCTTTGGATTTGTATCCAGTGCAATGTACCTTCGGGAGATCTATGCAAATCCGAGAGATCCATAGAATTTCAATGGGGTTAAATGCTGAATAATGCATACCACATTGTGCAGTTACTGATTGTTGATCTTCTATGGGTTTCTCTCTTTGTCATGGACCTATCACTTGATGATTATCCAAATTAGTGTTTCTAATTGATTATATATAGTACTTGTGAATATTGGTAGCTTTAAAAAACTTAGCCTCGCAATTAGTCCTTACCGCACATATGCTACTTAAGCTATGTGATCTGGTATCGATTGCGATTAATTGCAATTGTTGTGTGCATCTTTAACACTTTGTGCCGCACATAATGTACATTAACTGGACTATTTTTAGACTAAAACTCATTGCTCTCTTGGATTTGCATACATATACATTCCCGGGAGATTTATAAATAAAATTAGTACCATAGATCAATAATAATTTTGGTTCAAATGTATGTCACATTACTTGTAAAACTATGACTAAAATATTAATCTTTTTATGGATTAATATACATGTTTTTTATAATGATTTCTCTCCTTTTTATGGATTTTCTTACTTGAAGATTAATTATCCAAAGGTTAATAGTTTCCTACCCTAGTAGTTACCTCACATAGCTGTGCTACATATATATTTATGTTATTGTTTATCAACTATCGCTCTTAATCTTTTAATGGATGTGTGCCTATATACAAGACTGTGTCCTCAACATGAATCCACAACCTTGTAATTTTTGTTGTGCAAATTGACAAATCACACAACCTTTGTATCTTGGGTCTTTTTGTCATGAATATTGCCAATAACTCAACCTTGTTTCTTTGTTGCCTCTAGGAAATGTAAAACTCCAGAAAACTTGTCTTCATATAAGAAATATCAATATGATTCTACATTGTCAAAATTAAACCCGGTGTAGTGTTTACTACAACCCTCCTATATACTAACCAAGTGGTTGTAGTGGTTTGGCCATGTTGGTCAAGATGATATGCGGATTCTCTCACTTGCTGCATACTTTGTTAGGGTTTGTTCACGCCTTATACTGAATGTTAGGTCCAGCCTTGGAATAGCCGAGACACCTGGCTCACTAATTATGAGTTAAAAAGAAACATTATTCACTCAATAACTCATTTTTGCATCCTTAATTTTGGTGCAAAGAGCTTAACTTCACAATGGAACTGAAACCTATTCGCACAAATTATTAAGTGACTTAGCGGTAGTTTTATCAAATTTGCTTTGACCTCTATTAGGTAATTATGTAGTTTTAGTTTTATCTGTCTTAGGTTTTTCCTTCTTTGGAACAATTTCTATATATTGGTGATACTTGATAGATGCCTCAGTAAGTTGATGTCTCAGTAACTCTTCACCTTTACTGCTTGATTTTTTGCTTTCAATATAGTTAATTGGAACCTCAACAGTTTCTATACAAACGAGGAAGAAAATGGAAGATGGAAATACAAAATGGAAAACCGGTTTCCTATTCTTAGGAAACTGTTTAGATTTTCGTTGCAAAGAAACATAAAATAAAATTATGAGATTGTTGTCTAAAAACTCGGTCAATGTATTTGGAGTTTGGATTTACGTATTGGTCATCTGCTTACCGGCCACATCATCATCATCATCATGTTATGGCTTATCAATACTCCATTACCAGAAAGAACCTTGAGGTCAAGGTTCATATGAAAACTCAAAAAAGTTGTCTTGTATATATGTTCAGATGGTAATACTTAGAAAACCAATAGTTGATGTCACCGTTTTTGACACACGATTAGGCGTTTTCTTTTTAGTTATGCATAAGAACACTAAAATTTTTGATAGGATGATGATAAAGATAGAATTTAGAGGATAAATAGGTTTTGTTTTTATAATGGTATTGATGCCACAATTTGTGGTTCAAATCACTTCCGAGACAAAAGAAAGAAGATATATAGGGTGTTGTTTTGACTTTTGATGCTAATTGCGGTATGGATCGAATCCAAAAATGGAAGATCAGATAGAGGTTACATACAATCAGAATAATGTAAGTCAATGAATTGGAAGCAGTCTTCCACTGTTTCTTATGTTTAGGGTTGTCTTTTAATTATGTGCCGAAATTATAAATAAAAATGACTTTCTGTAAGACTCAGTCCATGTCCTTGGAATTTGGCTTAAACGTAGGTATATTGGACGTCTGCTGAGCGCTTACTGCCATGTCATCATGTAGTGATTCATCAATATCTGTGTGTACGTTTTCGTGAGTATATGTTTTCTTTAACAGTAAAAAGTCTGTTTGTATTGTACACTCTCTCTTAAGCCTCAGTTGCTTGTTTGCATTTAAGTTTCCTTCTGTGTTTCCATGTTATATTATATCAATCAATGTACCATATATAGAAGCACAAACAAAATTGCATAGAAACAATCTGGACAGTGGACGCTTGAGATTAGGGTTTTCTGTAAACGAATTTTAGATCACAGGGATAATCTATATACATGCCTTAGTTTTAGACTTTTAGTAAACCTTTTCTTTGTGGAAAAATGTAAAATCTCAACCTACCGATATATATTAAAATAATTCATTGGATAATCAAACTTTGAACATTTCTTGGTAAATTTTTAGAGTTATTTATTTAGAGATGAAAAGATCTATATATGTGTAATTATTTAGGTTTTGTCTCTAAATGTCTCCTTTGATGACACCATATACATGTTTTATAGTTTCCAGTGGTCTTTTCAAGGGTTACCTTGTATTTTATTATCCTAACATACTTTTCTTTTTTGTCATCTCTCCAGCCTGGTGAAGATCCTTGATCGATATGGGAAACAACATGCTGATGATCTCAAAGCCTTGGTAATACAAATGTTTCGGATATTTTCCCAGATGGATTTTCATGAGGCGTAGATTTACTAAAGACGTAGAGAGTTCAGTAATCAATACTGTCAGAGCCTATTCATAGCCTCCTGTCTAGCTTGCATTTTTATAGGCCTGGGCTAAGAGGCATATAACGTTTTCACATATAGTCACTATTTGGAGTTGTGTAGTTGTAATTGTTTAATAGATATGAGTAAACGAATGTTTAGCACCATAACATTGTGGAGATATATCTAGATAAAGGAATTGCATCATTGATCCACCATGCGCTATTAGCTTGCTAAGTGATTCTAAATATATAATGAGATGGTGTACCAACATGCATACTGAAATCCTGCTAGCCTTGTCTTTTACCGCGTCTTATATATGTCCCTTTTCTTTGGCCAGGATCTTCAGTCAAAAGAACTGAACTATGGTTCACACCATGAGCTACTAGAACTTGTGGAAAGGTTAGTACTAGCTAAGACTATATTTGCTCTCCTCCTTTGATTATAAAGGAATTAGGGTTTCCTTGTAAAACTATGAATATATGCAGCAATCTTGTGGAATCAAATGTCAACAATGTGAGTGTCGATTCCCTCGTTCAACTGGAGGAACACCTTGAGACCGCCCTCTCCATAACTAGAGCGAAGAAGGTAAGTGGATTCTATAATGTCTAGCTACTCTTTCCTGAATTTTATTTTCCGAGAATAACCTTATTGCTTTTATTTGTTACAGACAGAACTAATGTTGAAGCTTGTTGAGAACCTCAAAGAAAAGGTTAGATATTTGATTCCAAGTTTAGAACATATATCAGATTTATACTCTAGGGTACTGTTTTCATTGTTTCTGTAAGTCTGTTTAATGAGCTTTTCACACGTCTTTGCAACTTCTTCCCAATACATATGTTGTGGATTCCAAAATCTGAATTTGTTAGGTTGTGATTTGTGATTGCTGATCATGATGATCTTTAAAACAGGAGAAATTGCTGAAAGAAGAGAACCAGGTTTTGGCTAGCCAGGTAACGAAAGCTACTTTTTCTATATATATGCATACCTAATAAGCCATTCTTTCCACTTTAAGCATTTACTAGGTAGCACTTCTGTGTCTTGTATTCTAGAAATTTTATTTCTCAAGATTAGGCATTTTGGTTGTTGGTAGATTAGGTATTATTAGGGTTTGTGAGATTGTTACTGCTGAATAAGGACAGAGATTTGATTCGGTCTGGTTTGAGTTAGGGAAACTTTGATTCGGATTTTAGAATGACAGATGAAAAGTTTAATATAATTTTAAGATAAAAGACACACAAACAAACAAAATTCATGAACAGTATAATTTCATTATTACATAATAATTTGTTCGGTAAAATATATATTTTTATAACGATTATTGCTATATCATTTCACTAAAATAATATATACTTTTCTGTTGGTGCGAGTAATATTAGGTTTTGGTTGTTCTTTTTCTGGATTTGGGATAATAAAGGTTTTAATTAGGTTTTGGTTCAGTTTGAGATTTCTGAACGGATTAGTTTTTTGTGCGTCATGGTTAAGGTTTGGATCCATGTGTCCAGTAGTCTAGCTACTTTTACATCCTCAAGGTTAAATTATAAACAGGGAAGTAGTCTAGAACAGACAAATTATGGAAGCAAAATGGTGGAACTGAGTGTTAGTTTTAGATTGGTGGAATGAAAAAAGAATATTCATATATGAGTAGACAAACAGGTAAAAAGTGAATAGTGATTTTGACCTATGATTATCGTACAGATGGAGACGAATCATGTTGTTGGAGCAGAAGCTGATATGGAGATGGAGATGTCACCTGCTGGACAAATCTCCGACAATCTTCCGGTGACTCTCCCGCTGCTCAATTAGGGCGGTTGATATCAAAAATCCAAAACATATATATAATTAAGGGAAGAAAAAAAAAAAAAAAGATATGTAATTATTCCGCTGATAAGGGCGCGTCGTATGTATGTATATCTTAATACTCCCTCCTCTCTCTCTCTCTCTCTCTCTCTCTTTGGCTTGTGTGTGATACTTAAAAAGTAGATTGAAAGTCAAATATTATCTGTTTAAGACAAAACTATATATGATGAAACTTTTGTACCTTATTTGAGAGAGCTTTGCATCGAGATGTTGTTTTGTGTGTTCTCCTCTTCTGTCCAAAGCTTGTGTTTGCTTCACAGTGAAGAAGTCTTCTGCTTATTTTGCAGTAGAGGCGTGGCACAACTCTGCTCTATATGTCCTCGTAATTTAATTTGTTTTTATCTAAAACGCGTTGCGGTTGTCATGGGTTTCTATTGGGCCCATGTCGGTCATTTTTTTTGGCCCAATAGGCGTAGTTCTTGAGATTCATTGCTGTGAAGTGTTGTTAACGTGACCGACCTCTTTCCAAAATTATGCTTGATATCTAAGCCGGCTTAACAAGCCGTAT
